# A single nucleotide substitution in the *SlMCT* gene contributes to great morphological alternations in tomato

**DOI:** 10.1186/s43897-025-00159-x

**Published:** 2025-08-01

**Authors:** Mengyi Yu, Yinge Xie, Zilin Qian, Yu Zhong, Huolin Shen, Wencai Yang

**Affiliations:** 1 Department of Vegetable Science, Beijing Key Laboratory of Growth and Developmental Regulation for Protected Vegetable Crops, China Agricultural University, Beijing, 100193 China; 2 Joint Laboratory for International Cooperation in Crop Molecular Breeding, Ministry of Education of the People’s Republic of China, Beijing, 100193 China

**Keywords:** 4-diphosphocytidyl-2C-methyl-D-erythritol cytidyltransferase, Terpenoid biosynthesis, Phytohormone, Carotenoids, Chlorophyll

## Abstract

Terpenoids, a group of metabolites, are important to plant development and color formation, and serve as valuable nutrients for humans. The enzyme 4-diphosphocytidyl- 2 C-methyl-D-erythritol cytidyltransferase (MCT) plays a pivotal role in the methylerythritol phosphate (MEP) pathway for terpenoid biosynthesis. However, the potential lethality of *MCT* mutants has hindered further exploration into its functional role in terpenoid metabolite families in plants. Here, we characterized a rare *MCT* mutant *yfm* with dwarfism, chlorosis, small leaves, and yellow fruits in tomato. Map-based cloning and sequence analysis revealed that a single nucleotide substitution in the *SlMCT* gene, which resulted in a point mutation (Leu297Pro) in amino acid in the mutant. Over-expression and complementation of the wild-type *SlMCT*^*T*^ in the *yfm* mutant restored the fruit color and the other defective phenotypes. This mutation altered the gene expressions and metabolic components in the MEP and other pathways. Consequently, the total contents of carotenoids, chlorophyll, IAA, GAs, and SA were decreased, while the contents of CK, JA, and ABA were increased. Eventually, these alterations led to changes in plant phenotypes and fruit color in *yfm*. These findings provide novel insights into understanding the roles of MCT on plant development and pigment biosynthesis.

**Supplementary Information:**

The online version contains supplementary material available at 10.1186/s43897-025-00159-x.

## Core

The single nucleotide substitution in the *SlMCT* gene disturbs the flux of MEP and other pathways, leading to low contents of carotenoids, chlorophyll, GAs, SA, IAA, and high contents of CK, ABA, and JA, and eventually alters plant morphological traits and fruit color in *yfm*. The data obtained here provide novel insights for understanding the roles of MCT on plant development and pigments biosynthesis.

## Genes and accession numbers

Gene information utilized in this study is accessible in the Sol Genomics Network (https://solgenomics.net/). The accession number of the *SlMCT* gene is *Solyc01g102820* and the accessions of other genes are provided in Supplementary Information (Table S6).

## Introduction

Terpenoids, one of the most diverse secondary metabolites, including carotenoids, sterols, phytol, plastoquinone, phytohormones, antioxidants and vitamins, are essential in physiological and biochemical processes, such as pollination, photosynthesis, fruit ripening and stress resistance (Kessler and Baldwin 2001; Pichersky et al. 2006; Li et al. 2024; Zhu et al. 2024). The ubiquity, physiological relevance, and biological activity of terpenoids as well as their contributions to the organoleptic, nutritional, and medicinal quality of plant organs make these molecules as targets for crop improvement (Contreras-Avilés et al. 2024).

Biosynthesis of terpenoids has been intensively investigated for decades. All terpenoids are derived from the five-carbon isopentenyl diphosphate (IPP) and its isomer dimethylallyl diphosphate (DMAPP) by mevalonate (MVA) and methylerythritol phosphate (MEP) pathways (McGarvey and Croteau 1995). The MVA pathway, initially identified in animal systems and fungi, synthesizes IPP from acetyl-CoA by six steps of enzymatic reactions that ultimately generate triterpenes, sesquiterpenes and steroids in the cytosol (Lichtenthaler 1999). The MEP pathway that occurs in the plastid of photosynthetic eukaryotes and most eubacteria (Eisenreich et al. 2004) uses pyruvate and glyceraldehyde- 3-phosphate as substrate, and produces IPP by seven enzymatic reactions under the catalyzation of 1-deoxy-D-xylulose 5-phosphate synthase (DXS), 1-deoxy-D-xylulose 5-phosphate reductoisomerase (DXR), 4-diphosphocytidyl- 2 C-methyl-D-erythritol cytidyltransferase (MCT), 4-(cytidine 5′-diphospho)− 2-C-methyl-D-erythritol kinase (CMK), 2-C-methyl-D-erythritol 2,4-cyclodiphosphate synthase (MDS), 4-hydroxy- 3-methylbut- 2-enyl diphosphate synthase (HDS), and 4-hydroxy- 3-methylbut- 2-enyl diphosphate reductase (HDR). Subsequently, monoterpene, diterpene, and tetraterpene are synthesized using MEP pathway products as precursors (Lichtenthaler et al. 1997; Andrade et al. 2017).

The MCT encoded by the *MCT* gene, also named as IspD (isoprenoid synthase domain-containing protein), is a key enzyme in the MEP pathway. MCT catalyzes the cytidylation of MEP to produce cytidine diphosphate methylerythritol (CDP-ME) in the third step of the MEP pathway (Rohdich et al. 1999). Previous studies on the IspD protein are mainly focused on the development of new antibacterial and antiparasitic drugs as well as herbicides (Rohdich et al. 2005; Corniani et al. 2014). The enzyme activity of IspD is reduced with the inhibition of DXR, suggesting that IspD potentially acts as a point of particularly metabolic control of the MEP pathway flux (Zhang et al. 2011). However, study on the *IspD* gene in plants is limited. The first plant *IspD* gene is identified in *Arabidopsis* (Rohdich et al. 1999), and two T-DNA insertion mutants of *IspD* gene exhibit albino with defect in chloroplast development (Hsieh et al. 2008). It has been proposed that IspD may be one of the rate-limiting enzymes in the MEP pathway for saponin biosynthesis due to its extremely low transcription in *Panax ginseng* (Xue et al. 2019). Although understanding the mechanisms of these biosynthetic routes are important for the potential modulation of the production of key isoprenoids (Cordoba et al. 2011), the lethal phenotype due to mutation of the *MCT* gene in *Arabidopsis* (Hsieh et al. 2008) suggests that it might not be possible to create artificial mutants of *MCT*, which prevents fully elucidation of the role of the *MCT* gene in terpenoids metabolism.

In this study, we described a mutant *yfm* with yellow fruit and defective growth phenotypes in tomato (*Solanum lycopersicum*). Map-based cloning found that the *yfm* locus contained a single nucleotide substitution (T to C) mutation in the *SlMCT* (*Solyc01 g102820*) gene. Genetic functional complementation confirmed that this mutation was responsible for the mutant phenotypes. The *SlMCT*^*C*^ allele in the *yfm* mutant influenced the biosynthesis and metabolism of carotenoids, hormones, and chlorophyll, which resulted in great morphological alternations. These findings provided new insights to understanding the *MCT* function in fruit coloration and plant development.

## Results

### Characterization of the *yfm* mutant

The *yfm* mutant was discovered in T1 generation of a transgenic line derived from genetic transformation of the *Rx4* gene from PI 128216 conferring hypersensitive response to *Xanthomonas euvesicatoria* pv. *perforans* race T3 into the susceptible line OH 88119 (Zhang et al. 2021). Six of 20 individuals showed dwarfism with yellow fruits, but the morphological alternations were not associated with resistance to race T3, suggesting that these changes were possibly not a transgenic event. To validate this, genome of T2 individuals showing morphological alternations were re-sequenced in 40 × depth and no T-DNA insertion was detected. Therefore, the *yfm* is a transgene-free plant and the mutation was most likely caused by tissue culture.

**Fig. 1:**
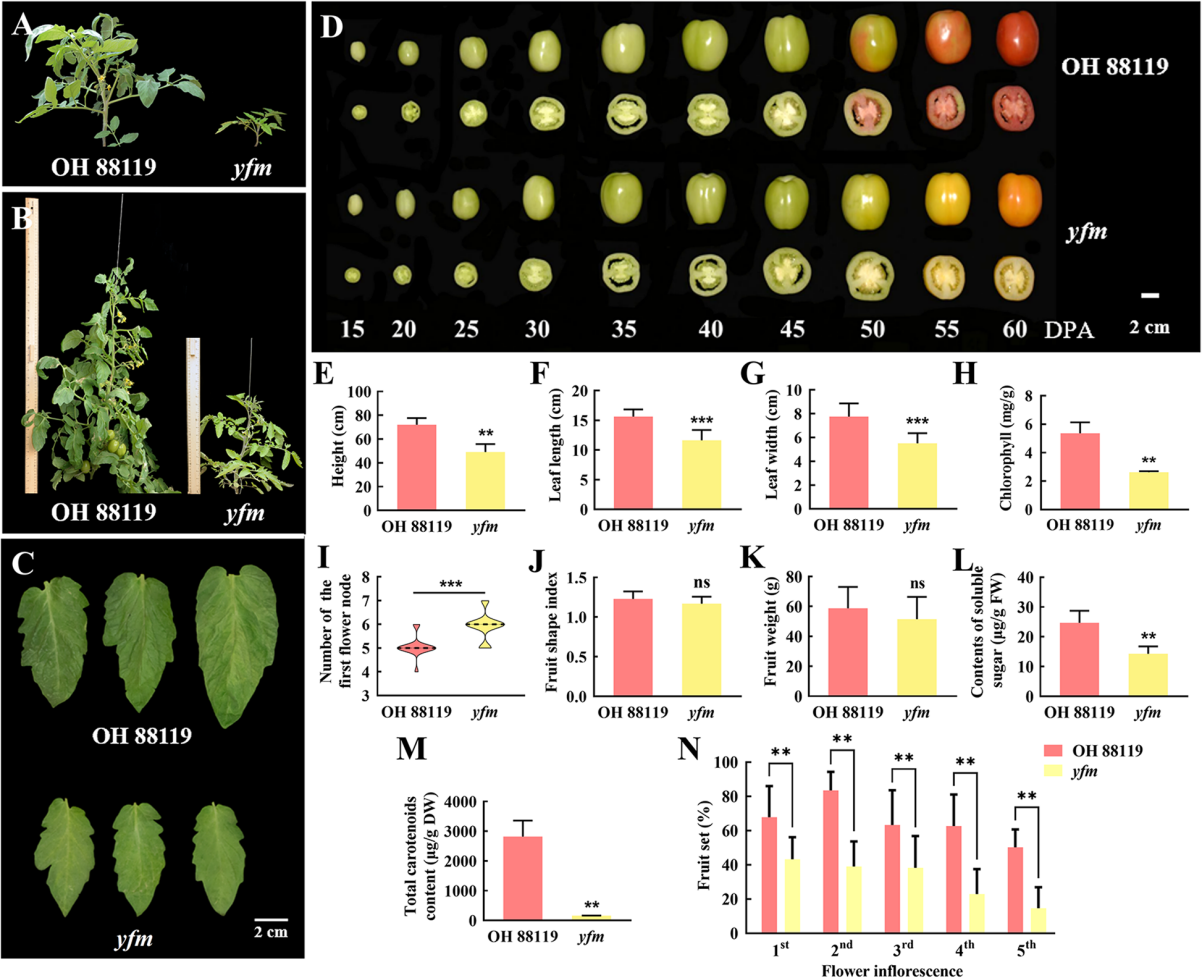
Phenotypes of OH 88119 and *yfm* mutant. **A** and **B** The plants of OH 88119 and *yfm* at 15 and 20 weeks, respectively. **C** Terminal leaflets of OH 88119 and *yfm*. Bar = 2 cm. **D** Fruits of OH 88119 and *yfm* at different developmental stages. DPA, day post anthesis. Bar = 2 cm. **E** Plant height. Mean values ± SD are shown (*n* = 30). **F** Leaf length. **G** Leaf width. Error bars mean ± SD (*n* = 15). **H** Content of chlorophyll in leaves. Mean values ± SD of three biological replicates. **I** Number of the first flower node. 30 individual plants are counted. **J** Fruit shape index. **K** Fruit weight. **L** The content of soluble sugar in fruit. **M** Total carotenoids content. Error bars mean ± SD (*n* = 15). **N** Percentage of fruit set at each flower inflorescence. Fruit set is calculated as the ratio of fruits:flowers for each flower inflorescence of 15 individual plants of OH 88119 and *yfm*. A total of 45 fully ripe fruits from 15 individual plants of each genotype are collected for measurements in E-M. Asterisks represent statistically significant differences (**, *P* < 0.01, ***, *P* < 0.001, ns, no significance) analyzed by student’s* t*-test

The mutant showed great morphological alternations. Comparing to wild type, plant height of the *yfm* mutant was decreased 28.6% (Fig. 1A, B, E), leaf size in terms of length and width was also significantly decreased (Fig. 1C, F, G), and the chlorophyll content in leaves of the *yfm* mutant was significantly lower than that in the wild type (Fig. 1H). The number of chloroplasts was decreased in *yfm* (Fig. S1) though the chloroplast development in leaf was not significantly different from OH 88119. The number of the first flower node changed from an average of five in OH 88119 to six in *yfm* (Fig. 1I). The fruit color changed from red to yellow though the developmental stages of fruit had no obvious change (Fig. 1D). The fruit shape index and fruit weight had no significant differences (Fig. 1J, K), but the total soluble sugar content and total carotenoids content in fruits were markedly lower in the *yfm* mutant comparing with OH 88119 fruits at the fully ripe stage (Fig. 1L, M). Meanwhile, the fruit set was markedly decreased in *yfm* than in OH 88119 (Fig. 1N).

### The phenotypic change of *yfm* is conditioned by a single recessive gene

Two F_2_ populations, OH 88119 × *yfm* and *yfm* × PI 128216, were used to determine the genetics of the altered phenotypes in *yfm*. All F_1_ plants in both crosses showed normal phenotypes with red fruit. In both F_2_ populations, fruit color segregated with a ratio fitting 1 (yellow fruit): 3 (red fruit) (Table 1). Besides, in the F_2_ population of OH 88119 × *yfm*, all yellow-fruited individuals also exhibited dwarf phenotype, and the red-fruited plants showed normal plant height (Table 1). Since PI 128216 was an accession of *S. pimpinellifolium* with indeterminate growth habit, plant height in the F_2_ population of *yfm* × PI 128216 was not evaluated. These results indicated that the yellow fruit color co-segregated with dwarf plant height and was probably controlled by a single recessive gene.

**Table 1:** The segregation of fruit color and plant height in two genetic populations

Population	Generation	Total plants	Red fruit plants	Yellow fruit plants	Normal height plants	High dwarf plants	χ^2^ value
OH 88119 × *yfm*	F_1_	6	6	0	6	0	
	F_2_	234	185	49	185	49	2.06
*yfm* x PI 128216	F_1_	6	6	0			
	F_2_	436	338	98			1.48

### Mapping and identification of the candidate gene for altered phenotypes in *yfm*

**Fig. 2:**
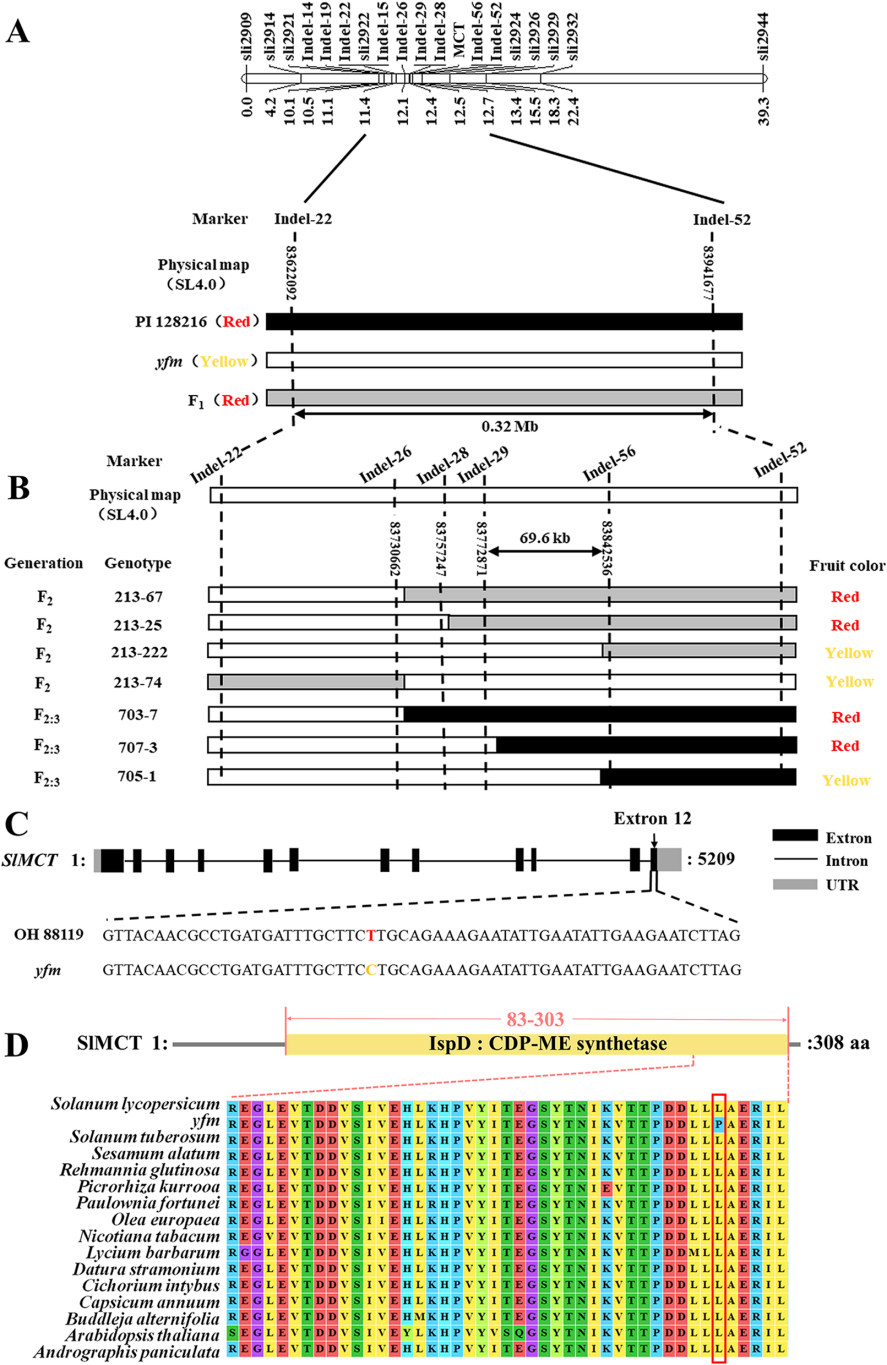
Map-based cloning of *yfm*. **A** Preliminary mapping of the *yfm* locus. **B** Fine mapping of the *yfm* locus. **C** The gene structure of the candidate gene *SlMCT* (*Solyc01 g102820*). The SNP (T to C) mutation is indicated by different color. **D** Alignment of *MCT* homologs from different species. The amino acid polymorphism caused by *yfm* mutation is marked by red box. The protein accession numbers of MCT proteins in different species are as follows. *Solanum lycopersicum*, XP_010314187; *Solanum tuberosum*, XP_006358470; *Sesamum alatum*, KAK4438429; *Rehmannia glutinosa*, KAK6159732; *Picrorhiza kurrooa*, AFM93780; *Paulownia fortune*, KAI3445531; *Olea europaea*, XP_022895625; *Nicotiana tabacum*, XP_016443221; *Lycium barbarum*, XP_060167511; *Datura stramonium*, MCD9639315; *Cichorium intybus*, KAI3707769; *Capsicum annuum*, XP_016538918; *Buddleja alternifolia*, KAG8368946; *Arabidopsis thaliana*, AAF61714; *Andrographis paniculata*, XP_051135393

Since the potential co-segregation between fruit color and dwarf phenotype, the fruit color was used as the trait for genetic mapping. For preliminary mapping of the gene conferring the yellow fruit, 87 InDel markers (Table S1) evenly spanning across all the 12 tomato chromosomes were used to genotype 92 individual plants randomly selected from the F_2_ population of *yfm* × PI 128216. Single marker-trait association analysis revealed that the marker Sli2914 on chromosome 1 was highly associated with the fruit color (*P* < 0.0001). Therefore, additional markers (Table S1) near the region of Sli2914 were developed and used to determine the precise position of the gene using all individuals in the F_2_ population. The gene was found to be located at a 0.32 Mb region between two markers Indel- 22 and Indel- 52 (Fig. 2A). With 648 F_2:3_ individuals derived from three F_2_ heterozygous recombinants (213–25, 213–222, and 213–74), the *yfm* locus was ultimately narrowed down to a 69.6 kb region between two markers Indel- 29 and Indel- 56 (Fig. 2B). In this region, ten genes were annotated in the reference genome SL4.0 of Heinz 1706 (Table S2).

Comparison of re-sequenced genomes between OH 88119 and *yfm* revealed that only one nucleotide substitution (T in OH 88119 and C in *yfm*) was found at the last exon of the gene *Solyc01 g102820* in this region (Fig. 2C). Based on the annotation, *Solyc01 g102820* encodes MCT, a key enzyme in the MEP pathway (Table S2).

**Fig. 3:**
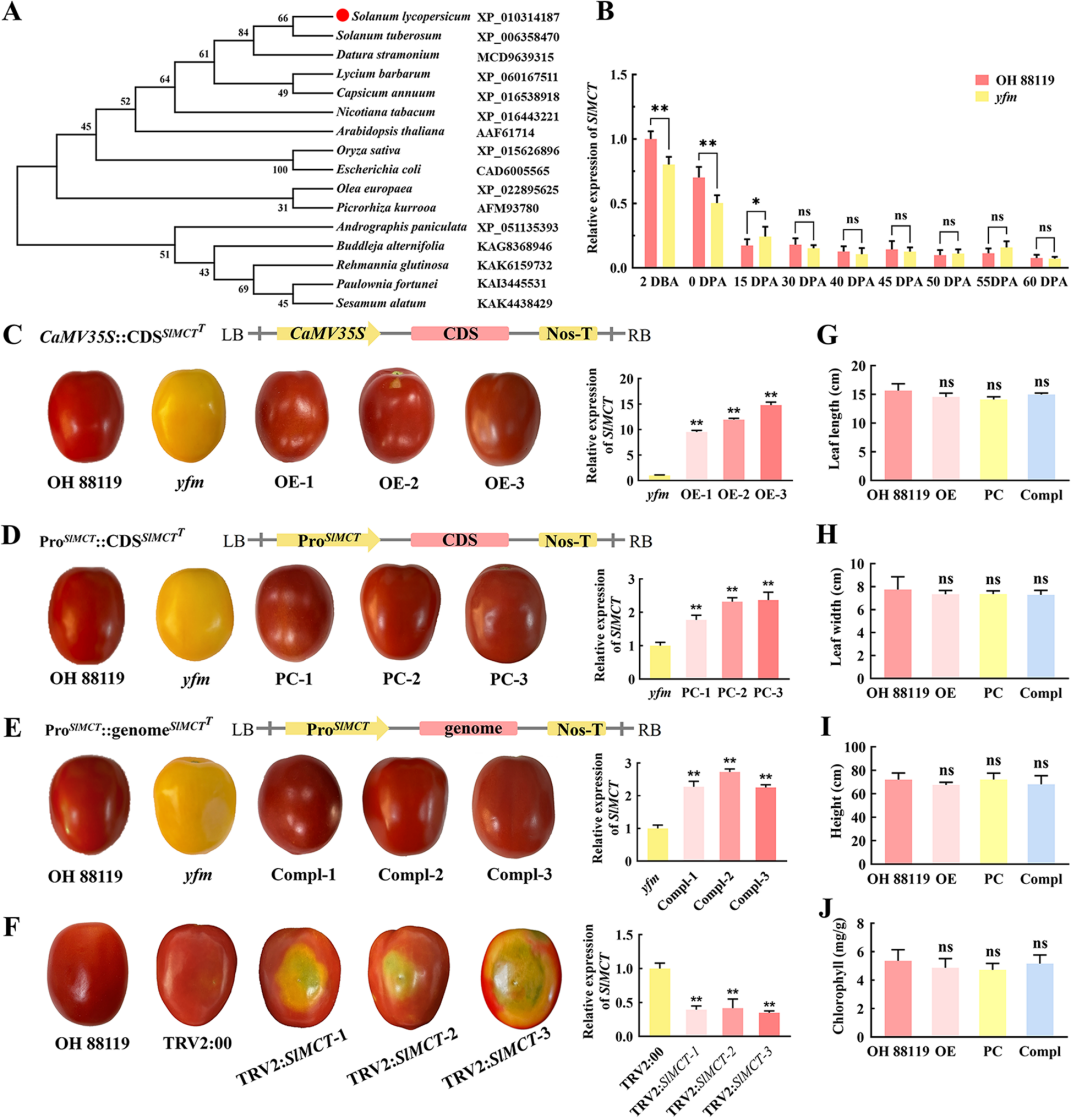
Functional validation of the *SlMCT* gene. **A** Phylogenetic tree of *MCT* homologs in different species. The phylogenetic tree is constructed by MEGA 7.0 using Neighbor-Joining method with 1000 bootstrap replicates. The accession numbers of protein are listed in the right.** B** The relative expression levels of *SlMCT* at different fruit developmental stages in OH 88119 and *yfm* mutant quantified by RT-qPCR. **C** Fruit color and gene expression in lines with over-expression of *SlMCT*^*T*^ from OH 88119 driven by CaMV35S promoter in *yfm* mutant. **D** Fruit color and gene expression in lines with over-expression of *SlMCT*^*T*^ from OH 88119 driven by the promotor of *SlMCT*^*T*^ in *yfm* mutant. **E** Fruit color and gene expression in lines with functional complementation of the *SlMCT*^*T*^ gene in *yfm*. A 7281 bp fragment, containing promotor (2147 bp) and genomic DNA (5134 bp) of *SlMCT* is transformed in *yfm* mutant to restore the phenotype. **F** Fruit color and gene expression in lines with *SlMCT*^*T*^ silenced by VIGS in OH 88119 fruits. TRV2:00 is empty vector. For B, C, D, E, and F, the relative expression of *SlMCT* is quantified by RT-qPCR with three biological replications and three technical replications. Error bars mean ± SD (*n* = 3). **G-J**. Leaf length, leaf width, plant height, and content of Chlorophyll in transgenic lines and OH 88119. For, G, H, I, and J, at least three individual plants are pooled together for each line as one biological replicate, and three lines are detected in different types of transgenic plants. Error bars represent mean values ± SD. Significance analysis is analyzed by student’s *t*-test (**, *P* < 0.01, ns, no significance)

Sequence alignment showed that the IspD domain in the MCT protein was highly conserved in diverse plant species (Fig. 2D). In plant species, *MCT* was a single-copy gene, and the *SlMCT* from *S. lycopersicum* showed the highest homology to that of *S. tuberosum* (Fig. 3A). The single nucleotide substitution of T to C resulted in an amino acid substitution (Leu297Pro) in *yfm* (Fig. 2D). The SNP could be detected as a dCAPS marker (Fig. S2) and exhibited complete co-segregation with fruit color in both F_2_ populations derived from crosses OH 88119 × *yfm* and *yfm* × PI 128216. These results indicated that *SlMCT* (*Solyc01 g102820*) was the candidate gene for the yellow fruit and other abnormal phenotypes in *yfm*.

### Functional validation of the *SlMCT* gene

The relative expression of the *SlMCT* gene could be detected in all organs and tissues with the highest in leaves (Fig. S3). Expression of the *SlMCT* gene showed a high level at the early stage of fruit development, 2 days before anthesis (DBA) and 0 days post anthesis (DPA), and then gradually decreased accompanied by the fruit development, but there was no significant difference between OH 88119 and *yfm* in each developmental stage (Fig. 3B, Fig. S3). To further confirm whether *SlMCT* was the candidate gene for the *yfm* mutant, *SlMCT*^*T*^ was functionally complemented and over-expressed in the *yfm* mutant. In the over-expression transgenic lines, the expression of *SlMCT*^*T*^ driven by CaMV35S promoter was up-regulated by 9.5 ~ 14.8-fold compared to that in *yfm*, and colors of fully ripe fruits of OE- 1, OE- 2, and OE- 3 lines were red (Fig. 3C). The transgenic lines PC- 1, PC- 2, and PC- 3 expressing *SlMCT*^*T*^ CDS driven by its native promotor also borne red fruits (Fig. 3D). Similarly, the fruit color was changed from yellow to red in Compl- 1, Compl- 2, and Compl- 3 lines, which were functionally complemented by the full-length genomic DNA of *SlMCT*^*T*^ in the *yfm* mutant (Fig. 3E). Furthermore, all other altered morphological phenotypes in *yfm* were also recovered in transgenic plants. In all transgenic lines, leaf length, leaf width, plant height, and the chlorophyll content were not significantly different from OH 88119 (Fig. 3G to J). These results suggested that the yellow fruit and dwarf plant height were controlled by a single recessive gene and the single nucleotide substitution in the *SlMCT* gene was the cause of morphological alternations in the *yfm* mutant.

Surprisingly, in vitro assay showed that the enzymatic activity of SlMCT^Leu297Pro^ was significantly higher than that of wild type. But enzymatic activities in different mixture of SlMCT^Leu297Pro^ and wild type were not significantly different from that of wild type (Fig. S4B). This data suggested that there was a potential competition between the mutant and wild type. Presence of wild type might have priority to use substrates and inhibit the activity of the mutant type.

### The mutation of SlMCT alters transcription and metabolism in tomato

**Fig. 4:**
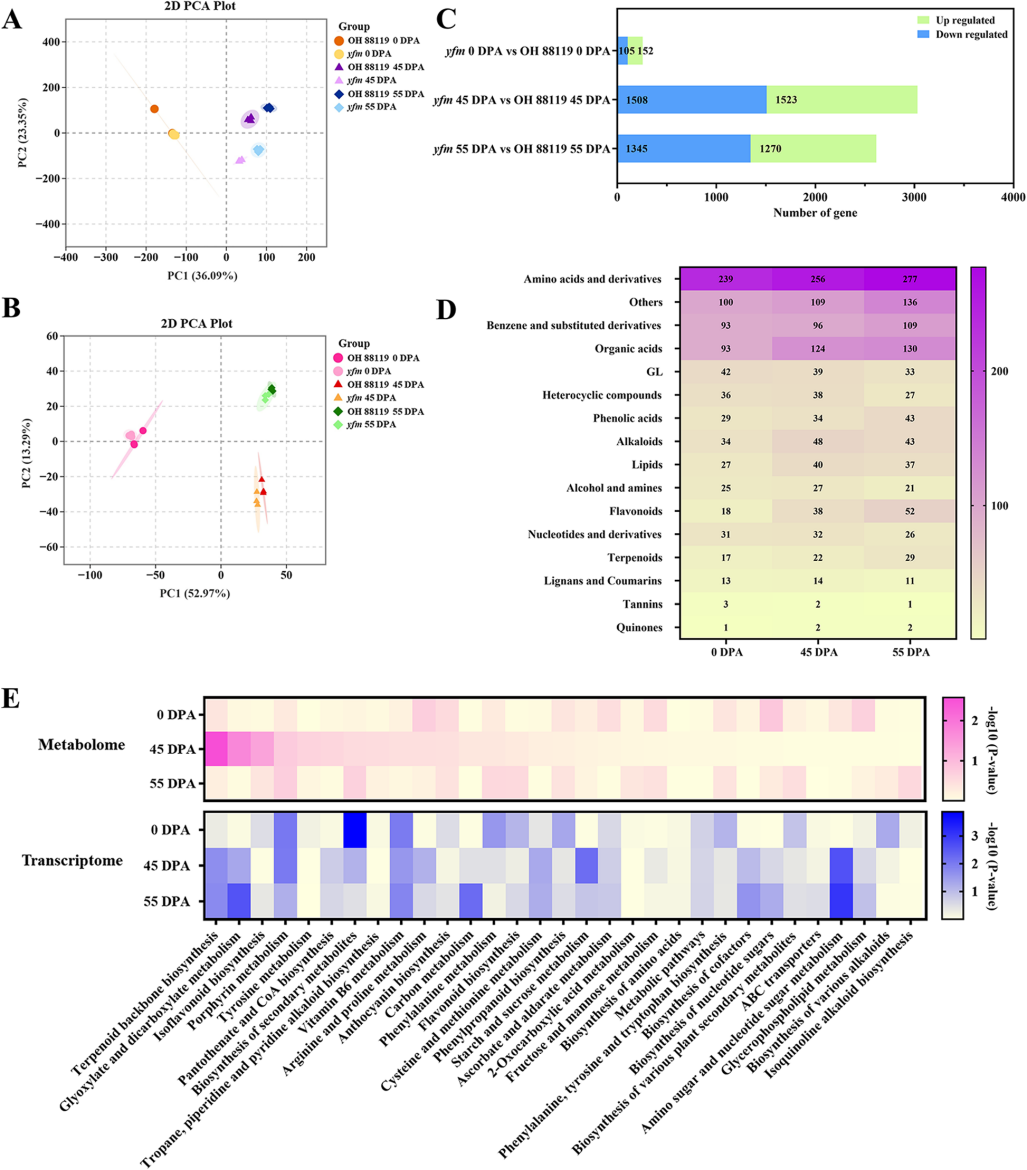
Transcriptome and metabolome analysis of the *yfm* mutant and OH 88119 fruits. **A** and **B**. Principal component analysis (PCA) plot. The transcriptome (A) and metabolome (B) PCA are analyzed from the OH 88119 and *yfm* fruit at 0, 45, and 55 DPA developmental stages. **C** Distribution of the downregulated and upregulated DEGs (differentially expressed gene). The expressed genes in *yfm* fruits are compared with the same developmental stage in OH 88119. **D** Heatmap shows the DAMs (differential accumulation metabolite) categories. The metabolites in *yfm* fruits are compared with the same developmental stage in OH 88119. **E** KEGG enrichment analysis of DEGs and DAMs from the OH 88119 and *yfm* fruit at 0, 45, and 55 DPA developmental stages

Comparative transcriptomic and metabolic analyses were performed to unravel the potential network for SlMCT-mediated fruit development and color formation using fruits of OH 88119 and *yfm* collected at 0, 45, and 55 DPA. Principal component analysis (PCA) of transcriptome and metabolome, and correlation analysis showed a significant similarity among the three biological replications within each genotype at the same period, suggesting the high reproducibility of the data (Fig. 4A, B). In transcriptome, the samples of OH 88119 and *yfm* at 0 DPA were clustered together, the samples of OH 88119 at 45 DPA and 55 DPA were clustered together, and the samples of *yfm* at 45 DPA and 55 DPA were in the same cluster (Fig. 4A). However, the samples of OH 88119 at 45 DPA were clustered together with the samples of *yfm* at 45 DPA and the samples of OH 88119 at 55 DPA were clustered together with the samples of *yfm* at 55 DPA in metabolome (Fig. 4B). One possible interpretation of this phenomenon was that the broad-target metabolomics approach used here mainly detected the primary metabolites, which might not be significantly different between wild type and the mutant at the same fruit developmental stages.

Differences at transcriptional and metabolic levels between OH 88119 and *yfm* were gradually increased during the fruit development. Transcriptomic changes were detected after 0 DPA (Fig. 4C). A total of 257 (152 upregulated, 105 downregulated), 3031 (1523 upregulated, 1508 downregulated), and 2615 (1270 upregulated, 1345 downregulated) differentially expressed genes (DEGs) were identified between *yfm* and OH 88119 at 0, 45, and 55 DPA, respectively (Table S3). Meanwhile, the total differential accumulation metabolites (DAMs) were increased, but the upregulated DAMs were decreased during the fruit development. A total of 801 (439 upregulated, 362 downregulated), 921 (386 upregulated, 535 downregulated), and 977 (350 upregulated, 627 downregulated) DAMs were identified between *yfm*, and OH 88119 at 0, 45, and 55 DPA, respectively. These DAMs could be divided into 16 classes (Fig. 4D, Table S4).

KEGG enrichment conjoint analysis using DEGs and DAMs at three stages revealed that these DEGs and DAMs were primarily involved in thirty-one biosynthesis and metabolism pathways (Fig. 4E, Table S5). A significant correlation in the terpenoid backbone biosynthesis between both metabolome and transcriptome was detected at 45 DPA, indicating that *SlMCT* gene might regulate plant development and fruit color formation through the terpenoid backbone biosynthesis. Some changes were also found in glyoxylate and dicarboxylate, porphyrin, vitamin B6 metabolism etc., which might be secondary effects due to the metabolic and transcriptional changes (Fig. 4E).

### The mutation of SlMCT influences MEP pathway and carotenoid biosynthesis

The biosynthesis of the terpenoid backbone serves as precursors for the synthesis of carotenoids and phytohormones. To clarify the mechanism underlying the difference in fruit color between OH 88119 and the *yfm* mutant, we investigated the expression levels of genes involved in terpenoid backbone and carotenoids biosynthesis, and the carotenoid content in fruits at 60 DPA.

**Fig. 5:**
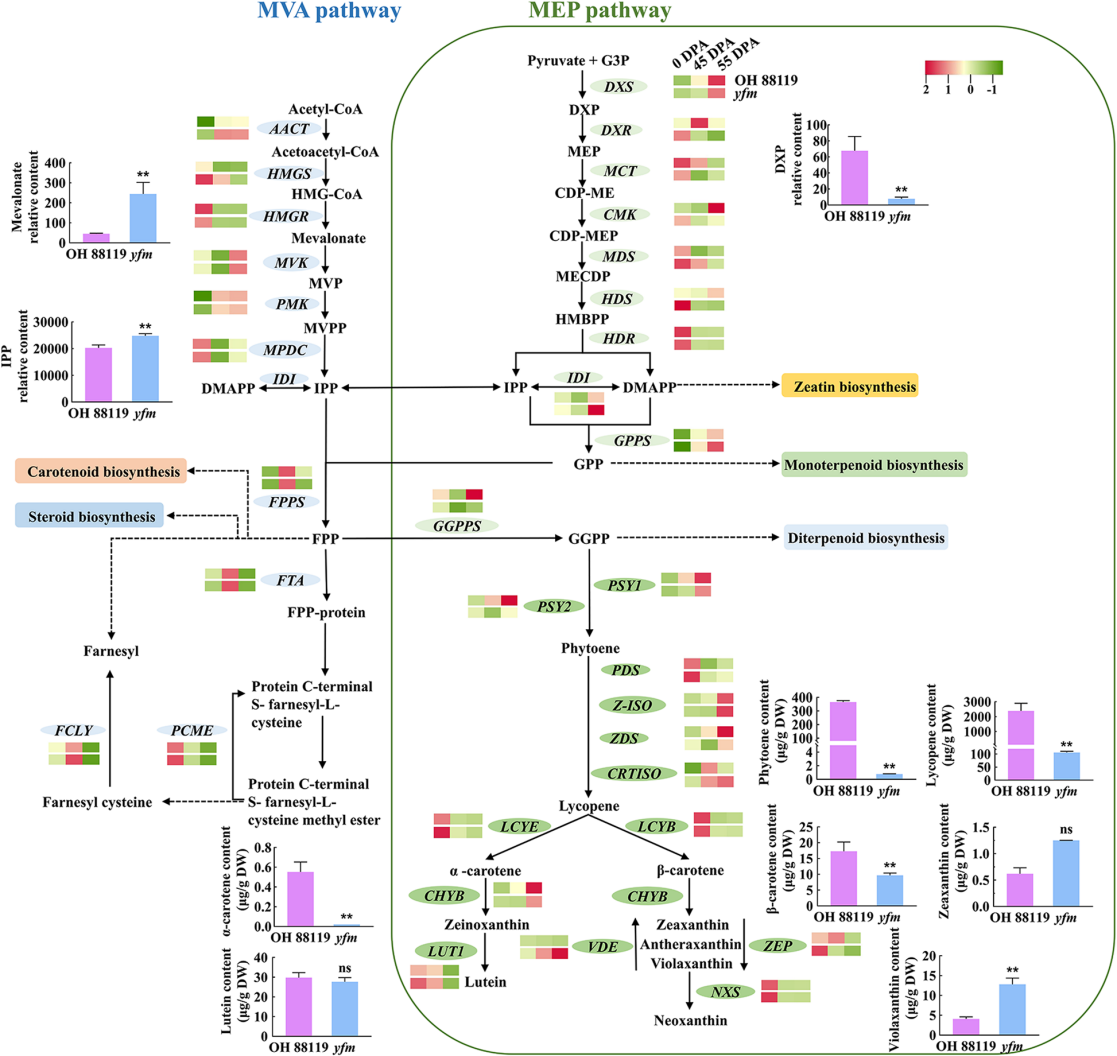
Schematic representation of isoprenoid biosynthesis in tomato. Solid lines with arrow present the direction of signal transduction pathways, including MEP, MVA, and carotenoid biosynthesis pathways. MEP pathway occurring in plastid is indicated by green box, while the blank space outside the green box presents cytosol with MVA pathway occurring. Heatmap shows the expression pattern of genes related to isoprenoid biosynthesis from RNA-seq data at 0, 45, and 55 DPA fruit developmental stage in OH 88119 and *yfm*. Histogram shows the content of metabolites involved in isoprenoid biosynthesis detected by metabolome at 55 DPA fruit developmental stage and carotenoid-targeted metabolome at 60 DPA in OH 88119 and *yfm*, respectively. Error bars represent mean values ± SD (*n* = 3). Significance analysis is analyzed by student’s *t*-test (**, *P* < 0.01, ns, no significance)

In the MVA pathway, most genes such as *AACT*, *HMGS*, *MVK*, *PMK*, and *MPDC* were up-regulated in the *yfm*, while the gene *HMGR* was down-regulated. However, the relative content of mevalonate in *yfm* was increased over 5.4-fold (Fig. 5, Table S6). In the MEP pathway, except for *SlMCT* and *MECPS*, the expressions of other genes were up-regulated at 0 DPA but subsequently down-regulated at 45 and 55 DPA. Specifically, the expression of *SlMCT* was down-regulated at 0 and 45 DPA but slightly upregulated (1.2-fold) at 55 DPA, which was consistent with the RT-qPCR data (Fig. 3B). The transcript level of *MECPS* showed an up-regulation trend during all stages. In metabolome, the content of 1-deoxy-D-xylulose 5-phosphate (DXP) was significantly decreased in the *yfm* mutant, exhibiting a more than nine-fold reduction compared to OH 88119. Additionally, IDI, a key enzyme involved in both the MVA and MEP pathways, was up-regulated at 0, 45, and 55 DPA, and the relative content of isopentenyl pyrophosphate (IPP) was also increased (1.25-fold) in the *yfm* (Fig. 5, Table S6).

IPP and DMAPP were precursor substances for the synthesis of terpenoid derivatives. As shown in Fig. 5 and Table S6, the genes in the farnesyl pyrophosphate (FPP) metabolic pathway exhibited significant increases in transcription level in *yfm* than in OH 88119, but the expression of *geranylgeranyl pyrophosphate synthase* (*GGPPS*) was down-regulated. Both *PSY1* and *PSY2*, which encode the crucial enzyme PSY (Phytoene synthase) for carotenoid biosynthesis, were also significantly down-regulated (Fig. 5, Table S6). The carotenoid content measurement showed that phytoene content in OH 88119 was 365.86 μg/g, but hardly detected in *yfm* (0.78 μg/g). Moreover, the contents of lycopene, α-carotene and β-carotene were reduced 22-fold, 2.5-fold, and two-fold, respectively, in *yfm* compared to OH 88119. No significant difference in lutein and zeaxanthin content was detected between *yfm* and OH 88119. However, the content of violaxanthin was 12.81 μg/g in *yfm*, which was three-fold higher than in OH 88119. These results suggested that the *SlMCT* gene in the MEP pathway acted upstream of carotenoid synthesis to regulate the fruit color in tomato.

### SlMCT affects plant development by regulating phytohormone biosynthesis

**Fig. 6:**
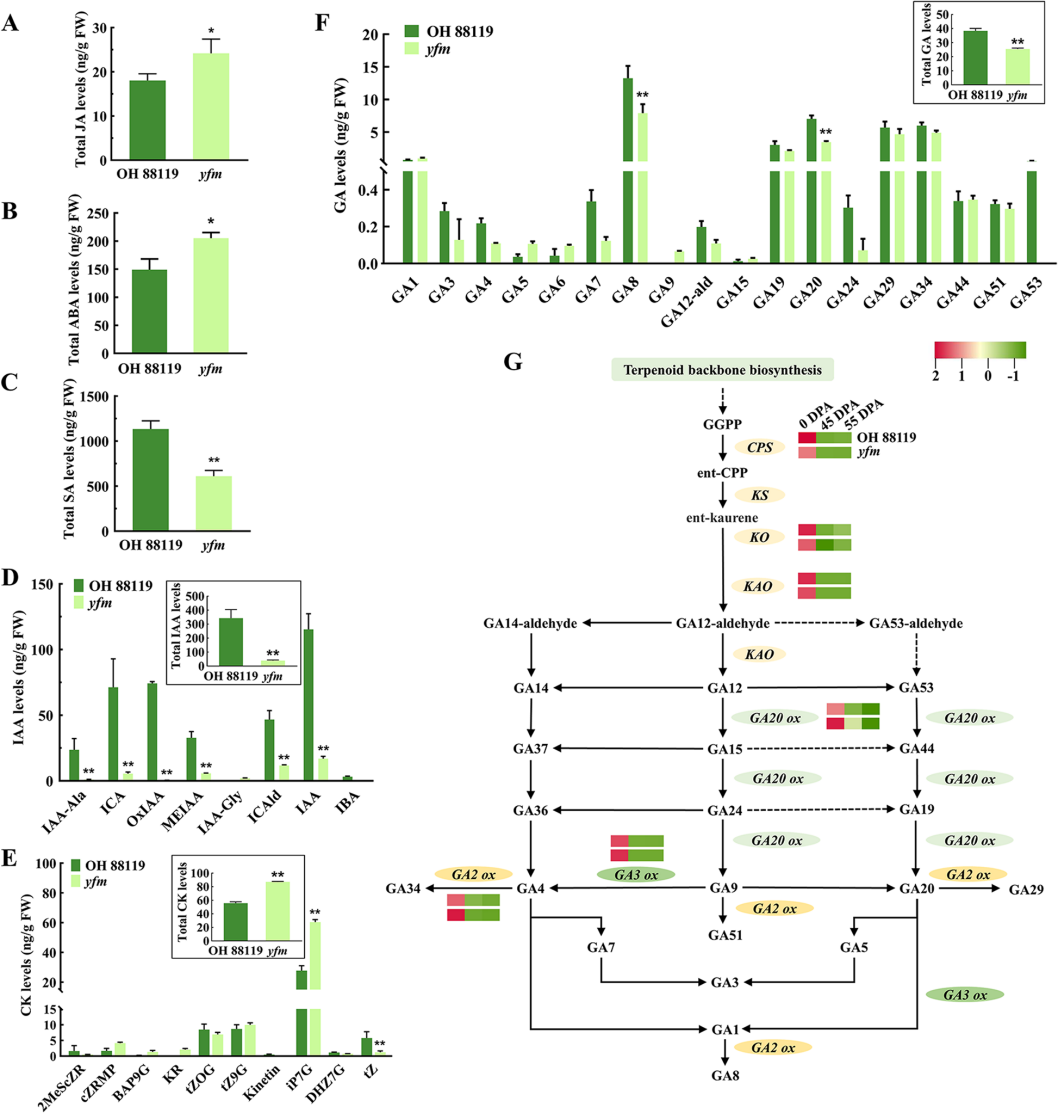
Phytohormone concentrations in OH 88119 and *yfm*. The jasmonic acid (JA, **A**), abscisic acid (ABA, **B**), salicylic acid (SA, **C**), indoleacetic acid (IAA, **D**), cytokinin (CK, **E**), and gibberellin acid (GA, **F**) levels are measured using LC-MS/MS. FW, fresh weight. Error bars represent mean values ± SD (*n* = 3). Significance analysis is analyzed by student’s *t*-test (**, *P* < 0.01, *, *P* < 0.05). G. Schematic diagram of GA biosynthesis pathway

To determine the causes of the plant dwarfism and morphological changes in *yfm*, the contents of six phytohormones were measured. Compared to OH 88119, significant increases in the total JA, ABA, and CK contents were detected in the *yfm* mutant. Total JA contents were 24.19 ng/g in *yfm* and 18.03 ng/g in OH 88119 (Fig. 6A), and the total ABA contents were 205 ng/g in *yfm* and 149.21 ng/g in OH 88119 (Fig. 6B). On the contrary, the total SA and IAA contents were decreased in *yfm*. The total SA content in *yfm* was decreased by 46.25% compared with OH 88119 (Fig. 6C). Meanwhile, the total IAA content was significantly decreased to 39.07 ng/g in *yfm* than that of OH 88119 (342.49 ng/g), and two common bioactive IAA forms IAA and IAA-Ala decreased by 85.35% and 95.65%, respectively (Fig. 6D). The total CK content was 87.13 ng/g in *yfm*, which was 1.56-fold than in OH 88119 (Fig. 6E). Intriguingly, the content of iP7G was increased 2.17-fold in *yfm*, while the content of tZ was significantly decreased by 79.17% compared to OH 88119 (Fig. 6E).

Overall, the GA contents were decreased in *yfm* compared to OH 88119 (Fig. 6F). The content of GA8 was significantly lower in *yfm* (7.90 ng/g) than in OH 88119 (13.26 ng/g), and the content of GA20 in *yfm* (3.45 ng/g) was decreased compared with OH 88119 (7.02 ng/g) (Fig. 6F). Expressions of genes in GA biosynthesis pathway, including *ent-Copalyl Diphosphate Synthase* (*CPS*), *ent-Kaurene Oxidase* (*KO*), *ent-Kaurenoic Acid Oxidase* (*KAO*), *GA 2-oxidase* (*GA2ox*), *GA 3-oxidase* (*GA3ox*), and *GA 20-oxidase* (*GA20ox*) were thoroughly examined in the RNA-seq data. The results showed that expressions of *CPS*, *KO*, and *KAO* were decreased in *yfm*, but *GA2ox*, *GA3ox*, *GA20ox* were up-regulated at 0 DPA and down-regulated at 45, 55 DPA (Fig. 6G, Table S6). The altered GAs content in *yfm* was possibly caused by changes in the expression of GA biosynthesis pathway genes.

## Discussion

### *yfm* reveals crucial roles of MCT on plant development and pigments biosynthesis

MCT is a key enzyme in the MEP pathway that produces terpenoids as precursors for biosynthesis of a wide variety of monoterpenes, diterpenes, and carotenoids. It is not surprising that loss of function of MCT can severely affect development and even lead to a lethal phenotype. Therefore, few works on characterization of the *MCT* gene have been conducted in plants due to the lack of mutant. In the current study, the tomato mutant *yfm* showed great morphological alternations including dwarfism, chlorosis, small leaves, and yellow fruits (Fig. 1A to D) but could survive under greenhouse conditions. However, the fruit weight and size of *yfm* were not significantly different from that of OH 88119 (Fig. 1J, K), suggesting that *yfm* might diminish the fruit count to facilitate normal fruit development (Fig. 1N). Contents of total carotenoids and chlorophyll were significantly decreased in the mutant comparing with the wild type (Fig. 1H, M). Genetic mapping, sequence analysis, and functional validation indicated that mutation of the *SlMCT* gene was responsible for the abnormal phenotypes in *yfm*. In addition, the chloroplast of *yfm* showed abnormal development (Fig. S1) and the chloroplast number was reduced, which were consistent with the phenomena observed in T-DNA insertion mutants of the *MCT* gene in *Arabidopsis* (Hsieh et al. 2008). Abnormal development and reduced number of chloroplasts might affect normal photosynthesis, reduce photosynthetic products, and subsequently result in dwarf and chlorosis of plants. All data obtained here indicated that the *MCT* gene not only plays a crucial role in development but also severely affects pigments biosynthesis in plants.

### The single nucleotide substitution in the *SlMCT* gene contributes to multiple phenotypic changes of *yfm*

Single nucleotide polymorphisms (SNPs) are the most commonly occurring variants during plant evolution and domestication (Akbari et al. 2021). In many crops, phenotypic changes due to SNPs have been reported, but most SNPs lead to alternations of a single trait. A single nucleotide substitution (C/T) in the *SP* (*self-pruning*) gene results in the change of plant growth habit from indeterminate to determinate in tomato (Pnueli et al. 1998). Similarly, the introduction of a premature stop codon SNP in *Rht-B1 d* and *Rht-B1e* (*Reduced height*) loci conferring insensitivity to the growth-promoting hormone gibberellic acid inhibits cell growth and reduces plant height in the Green Revolution wheat (*Triticum aestivum*) varieties (Pearce et al. 2011).A naturally occurring rare SNP in *Brachytic2* coding sequence increases yield and reduces plant height, which is considered as the causative mutation of the major plant height QTL, *qph1* in maize (*Zea mays*) (Xing et al. 2015). However, the single nucleotide substitution in the *SlMCT* gene reported here led to pleiotropic effects on tomato plant and fruit, which was different from most previous findings. This might be due to the crucial role of the *SlMCT* gene on plant development and pigment biosynthesis.

### *SlMCT*^*C*^ alters the terpenoid biosynthesis flux in *yfm*

The MEP pathway is one of the main pathways to synthesize terpenoids, which are essential to the plant growth and development (Dudareva et al. 2013). It has been demonstrated that the first enzyme in the MEP pathway1-deoxy-D-xylulose 5-phosphate synthase (DXS) is subject to feedback regulation, resulting in the inhibition of the MEP pathway flow (Cordoba et al. 2011). The increase of IPP/DMAPP content resulting in lower DXS activity has been reported in both bacteria and plants (Di et al. 2023). In the present study, the IPP content was increased in *yfm* (Fig. 5), while the *DXS* gene was down-regulated comparing with OH 88119 (Fig. 5), which was consistent with previous findings (Di et al. 2023). This data suggested that the MEP pathway flux might be inhibited by the feedback regulation in *yfm*.

In addition, over-expression of the fusion gene *DXS-FPPS* encoding a fusion protein of DXS linked with farnesyl diphosphate synthase (FPPS) in plastids of tomato results in substantial reduction in lycopene content and the variation of yellow fruit (Chen et al. 2023), which suggests a metabolic competition between the FPP metabolic pathway and carotenoid biosynthesis. In *yfm*, the transcriptional levels of genes involved in the FPP metabolic pathway, including *FTA*, *FCLY*, and *PCME*, were significantly increased (Fig. 5). However, *GGPPS* was down-regulated, causing significantly decrease of chlorophyll and GAs in *yfm*, which were derived from GGPP (Fig. 5). The genes in the carotenoid biosynthesis, such as *PSY1* and *PSY2*, also exhibited significant reductions at transcriptional level leading to substantial decrease in lycopene content in *yfm* (Fig. 5). Therefore, the feedback inhibition of MEP pathway and the upregulation of FPP metabolic pathway might potentially break metabolic balance and was responsible for the plant dwarfing and yellow fruit in *yfm* mutant.

## Materials and methods

### Mapping populations

The *yfm* mutant with yellow fruit and other abnormal phenotypes is derived from OH 88119 during the tissue culture process of *Agrobacterium*-mediated transforming the *Rx4* gene for resistance to bacterial spot from resistance accession PI 128216 into susceptible line OH 88119 (Zhang et al. 2021; see results for details). Two F_2_ populations and derived F_2:3_ families were used to finely locate the gene conferring mutant phenotypes in *yfm*. The first F_2_ population was derived from a cross between a red-fruited processing tomato inbred line OH 88119 (Xiao et al. 2020) and the *yfm* mutant. The second F_2_ population was developed by crossing the *yfm* mutant to *S. pimpinellifolium* accession PI 128216 with red fruit. All seeds were sown in 72 Square Plug Tray Deep (Taizhou Longji Gardening Materials Co., Zhejiang, China) filled with a mixture of peat, perlite, and vermiculite (3:1:1). The seedlings were transplanted to a greenhouse and grown at 24–28℃ (day) and 16–20℃ (night) with water and fertilizers supplied as recommended for commercial production (Gao et al. 2010).

### Phenotypic data collection

Plant height was measured and number of nodes of the first flower was counted from 15 25-week-old plants of each genotype. Leaf length and width were measured using the 4 th fully developed terminal leaflet from 15 individual plants of each genotype. The chlorophyll was extracted from 0.25 g fresh leaves with 95% ethanol in darkness (the same sample used for leaf length and width measurement), and the OD value at 649 and 665 nm were determined using Multimode microplate reader (Tecan Group (Männedorf), Männedorf, Switzerland), and then concentrations were calculated using the method described previously (Lichtenthaler and Wellburn 1983). A total of 45 fully ripe fruits from 15 individual plants of each genotype were subjected to fruit shape index (fruit longitudinal diameter/transverse diameter), fruit weight, and soluble sugar content measurement. The soluble sugar content was detected by the plant soluble sugar content assay kit according to the manufacturer’s protocol (Solarbio Science and Technology (Beijing) Co., Ltd., Beijing, China). For the fruit set, all the flowers from the first to the fifth inflorescences were marked and fruit set was recorded and calculated as the ratio of fruits:flowers for each flower inflorescence of 15 individual plants of OH 88119 and *yfm*.

### Marker analysis and genetic mapping

Genomic DNA was isolated from freshly collected young leaves using the modified CTAB methods. Molecular markers (Table S1) used for mapping of the gene were either adopted from a previous publication (Yang et al. 2014) or developed by comparing genomic DNA sequences between PI 128216 and OH 88119 (this study). Insertion/deletion (InDel) markers were analyzed following our previous protocol (Yang et al. 2014), while single nucleotide polymorphic markers were detected by dCAPs (Fig. S2) or Sanger sequencing. Linkage map was created using JOINMAP4.0 (https://joinmap.software.informer.com/).

### Genetic transformation and virus-induced gene silence

Gene function was validated through complementary experiments in the *yfm* mutant and virus-induced gene silence (VIGS) of the gene in fruits of OH 88119.

For the complementation experiment, a 7281 bp fragment consisting of promoter (2147 bp) and genomic DNA (5134 bp) region of the *SlMCT*^*T*^ allele was cloned from OH 88119 and inserted into plant expression vector pCAMBIA- 1305 using *Kpn* I and *Nco* I restriction sites (Table S1). In addition, pCAMBIA- 1300 vector containing the full-length CDS (924 bp) of *SlMCT*^*T*^ allele from OH 88119 was constructed using *Kpn* I and *Hind* III restriction sites (Table S1), and this recombinant vector was used to over express the *SlMCT*^*T*^ allele from OH 88119 in the *yfm* mutant driven by CaMV35S promoter and its native promoter region, respectively. *Agrobacterium tumefaciens* strain GV3101 was used to transform the recombinant vectors into tomato according to the previous method (Fillatti et al. 1987) with some modifications.

VIGS was performed using the previously described method (Zhang et al. 2021). A specific 300 bp fragment (from 311 to 610 bp) of the *SlMCT*^*T*^ gene amplified from the cDNA of OH 88119 using gene-specific primers (Table S1) was inserted into pTRV2 vector using *EcoR* I and *Bam*H I restriction sites. The empty vector pTRV1 and pTRV2 and the recombinant vector pTRV2-*MCT* were transformed into GV3101 cells, respectively. Transformed *Agrobacterium* was mixed by 1:1 ratio following the combination of pTRV1:pTRV2 and pTRV1:pTRV2-*MCT*. At least 30 fruits at mature green (MG) stage from 10 OH 88119 individual plants were infiltrated for each combination according to the protocol previously described (Fu et al. 2005).

### Carotenoid content measurement

The fully ripe fruits of OH 88119 and *yfm* at 60 DPA were used to measure the carotenoid content. Fifteen fruits from six individual plants were pooled as one sample, and three samples were harvested for each genotype. The fruit sample was grounded into powder in liquid nitrogen, and at least 5 g freeze-dried sample was subjected to measurement of carotenoids contents using the AB Sciex QTRAP 6500 LC-MS/MS platform at Metware Biotechnology Company (Wuhan, China).

### Transcriptome and metabolome analysis

Fruits of OH 88119 and the *yfm* mutant were harvested at 0, 45, and 55 DPA. Three fruits from each of five plants were collected and mixed as one sample with three biological replications for each genotype. Transcriptome sequencing and metabolome detection were performed on the Illumina sequencing platform and UPLC-MS/MS system, respectively, at Metware Biotechnology Company. The threshold of log2 (fold change) ≥ 1 and false discovery rate (FDR) ≤ 0.05 were used for defining the differentially expressed genes (DEGs), and differential accumulation metabolites (DAMs) were screened by VIP (Variable Importance in Projection) > 1 and absolute log2 FC ≥ 1. The transcriptomic and metabolomic graphs were analyzed using the online platform at Metware Cloud (https://cloud.metware.cn/).

### *In vitro *enzyme activity assay

The enzyme activity was assayed following the diagram of the catalytic reaction of SlMCT (Fig. S4 A), the *SlMCT* CDS amplified from cDNA of OH 88119 and the *yfm* mutant were separately ligated into the pET- 28a plasmid at *Bam*H I and *Xho* I restriction sites to create recombinant vectors of the pET- 28a-SlMCT, pET- 28a-SlMCT^Leu297Pro^. All resulting constructs were transformed into *Escherichia coli* strain BL21 (DE3) cells according to the manufacturer’s instructions (Sangon Biotechnology (Shanghai) Co., Ltd., Shanghai, China), and the BL21 cells harboring pET- 28a were used as the negative control. The transformed cells were cultured in lysogeny broth (LB) liquid medium with 100 mg/L kanamycin at 37℃ until the optical density at 600 nm (OD600) reached 1.0. Then isopropyl-beta-D-thiogalactopyranoside (IPTG) was added to a final concentration of 1.0 mM, and the cells were cultured at 160 rpm and 16℃ for 12 h to induce recombinant protein expression. Cells were collected *via* centrifugation, and then broken up by ultrasound. The SlMCT and SlMCT^Leu297Pro^ recombinant proteins were isolated and purified using the HisSep Ni–NTA Agarose Resin 6 FF (Yeasen Biotechnology (Shanghai) Co., Ltd., Shanghai, China). The enzyme activity of the SlMCT, SlMCT^Leu297Pro^ and proteins mixed at different proportions were performed in vitro. A concentration of 20 mM recombinant protein was added to 60 μL reaction system containing 5 mM MgCl_2_, 1 mM DTT, 1 mM CTP, 0.5 mM MEP, and 0.1 U inorganic pyrophosphatase in 100 mM Tris–HCl buffer solution (pH = 8.0). The mixture was incubated at 37℃ for 2 h and the reaction was quenched by incubating at 80℃ for 20 min. The pyrophosphatase in the reaction system was used to hydrolyze PPi to produce Pi. Then, the mix solution of malachite green and molybate (Beyotime Biotechnology (Shanghai) Co., Ltd., Shanghai, China) was added in the system to chelate Pi for 30 min color formation and the absorbance at 630 nm of the mixture measured by the microplate reader was used to calculate the Pi content, which represented the enzyme activity (Wang et al. 2024). All experiments were conducted with three replications.

### RNA extraction and RT-qPCR analysis

To explore the expression pattern of the *SlMCT* gene during fruit development, flower and fruits of OH 88119 and the *yfm* mutant were collected at 2 DBA, 0 DPA, 15 DPA, 30 DPA, 40 DPA, 45 DPA, 50 DPA, 55 DPA, and 60 DPA. Total RNA was extracted using Maxwell® RSC Plant RNA Kit (Promega (Beijing) Biotech Co., Ltd., Beijing, China) and reverse transcribed into cDNA using HiScript II RT SuperMix for qPCR (Vazyme Biotechnology (Nanjing) Co., Ltd., Nanjing, China). The One Step PrimeScript III RT-qPCR Mix (Takara Biotechnology (Dalian) Inc., Dalian, China) was used for RT-qPCR with gene-specific primers (Table S1). Expression of the tomato gene *UBI* (*Solyc01 g056940*) was used as the internal control. Reactions for the reference genes were included in each 96-well plate and each sample was analyzed independently with three biological replications and three technical replications. The relative expression was calculated using the 2^− ΔΔCt^ method (Livak and Schmittgen 2001).

### Transmission electron microscopy

Three leaves of OH 88119 and the *yfm* mutant plants at 45-d-old were used to visualize the chloroplast ultrastructure and the middle region of the leaf away from vein. Leaves were fixed in 2.5% glutaraldehyde and dehydrated in ethanol. Thirty fields of view per sample were screened and the electron microscopy imaging experiment was conducted at the electron microscopy Laboratory in College of Biological Science at China Agriculture University.

### Phytohormone content measurement

Leaves were collected from OH 88119 and the *yfm* mutant 15 weeks after planting for phytohormone measurement. Thirty leaves from 10 individuals of each genotype were separately pooled as one sample, and three biological replications were performed for each genotype. Leaves were immediately frozen in liquid nitrogen and stored at − 80℃. JA, ABA, SA, IAAs, and CK were extracted from 50 mg for each sample with 1 mL MeOH/H_2_O/formic acid (15:4:1, v/v/v). GAs was extracted from 100 mg for each sample with 1.5 mL 70% (v/v) acetonitrile by ultrasound-assisted extraction for 30 min and centrifugation at 12000 rpm for 10 min. Then 1 mL supernatant was collected for measurement of phytohormone content using LC-MS/MS (Niu et al. 2014) at Metware Biotechnology Company.

## Supplementary Information

Supplementary Material 1: Supplementary Figure S1. Transmission electron microscopy of plastid in OH 88119 and *yfm* leaf. Supplementary Figure S2. Images of agarose gel electrophoresis for partial individuals in the F_2_ populations of OH 88119 × *yfm* (A) and *yfm* × PI 128216 (B) using the dCAPs marker. Supplementary Figure S3. Spatial and temporal expression analysis of the *SlMCT* gene. Supplementary Figure S4. Catalytic activity assay of wild type SlMCT and mutant SlMCTLeu297Pro. Supplementary Table S1. Information for primers used in this study. Supplementary Table S2. Candidate genes in the fine mapping region between Indel- 29 and Indel- 56 markers on chromosome 1. Supplementary Table S3. Differentially expressed genes (DEGs) between OH 88119 and *yfm* in transcriptome. Supplementary Table S4. Differential accumulation metabolites (DAMs) between OH 88119 and *yfm* in metabolome. Supplementary Table S5. The conjoint analysis of transcriptome and metabolome between OH 88119 and *yfm*. Supplementary Table S6. Heatmap of the transcriptional level of genes involved in the terpenoid and carotenoid biosynthesis pathway.

## Data Availability

The data underlying this article are available in the article and in its online supplementary material.

## Abbreviations

IPP: Isopentenyl diphosphate
DMAPP: Dimethylallyl diphosphate
MVA: Mevalonate
MEP: Methylerythritol phosphate
DXS: 1-Deoxy-D-xylulose 5-phosphate synthase
DXR: 1-Deoxy-D-xylulose 5-phosphate reductoisomerase
MCT: 4-Diphosphocytidyl- 2 C-methyl-D-erythritol cytidyltransferase
CMK: 4-(Cytidine 5′-diphospho)- 2-C-methyl-D-erythritol kinase
MDS: 2-C-methyl-D-erythritol 2,4-cyclodiphosphate synthase
HDS: 4-Hydroxy- 3-methylbut- 2-enyl diphosphate synthase
HDR: 4-Hydroxy- 3-methylbut- 2-enyl diphosphate reductase
CDP-ME: Cytidine diphosphate methylerythritol
PCA: Principal Component Analysis
DEGs: Differentially Expressed Genes
DAMs: Differential Accumulation Metabolites
KEGG: Kyoto Encyclopedia of Genes and Genomes
VIGS: Virus-Induced Gene Silence
LC-MS/MS: Liquid Chromatography-Tandem Mass Spectrometry
