# Supplementary material for: A single nucleotide substitution in the SlMCT gene contributes to great morphological alternations in tomato
Source: Mol Hortic. 2025 Aug 1;5:49. doi: 10.1186/s43897-025-00159-x (PMC12315435; doi:10.1186/s43897-025-00159-x)
Supplement: Supplementary file 1 — Supplementary Material 1: Supplementary Figure S1. Transmission electron microscopy of plastid in OH 88119 and yfm leaf. Supplementary Figure S2. Images of agarose gel electrophoresis for partial individuals in the F2 populations of OH 88119 × yfm (A) and yfm × PI 128216 (B) using the dCAPs marker. Supplementary Figure S3. Spatial and temporal expression analysis of the SlMCT gene. Supplementary Figure S4. Catalytic activity assay of wild type SlMCT and mutant SlMCTLeu297Pro. Supplementary Table S1. Information for primers used in this study. Supplementary Table S2. Candidate genes in the fine mapping region between Indel- 29 and Indel- 56 markers on chromosome 1. Supplementary Table S3. Differentially expressed genes (DEGs) between OH 88119 and yfm in transcriptome. Supplementary Table S4. Differential accumulation metabolites (DAMs) between OH 88119 and yfm in metabolome. Supplementary Table S5. The conjoint analysis of transcriptome and metabolome between OH 88119 and yfm. Supplementary Table S6. Heatmap of the transcriptional level of genes involved in the terpenoid and carotenoid biosynthesis pathway. [file 43897_2025_159_MOESM1_ESM.pdf]

Supplementary information for:

A single nucleotide substitution in the *SlMCT* gene contributes to great morphological alternations in tomato

Mengyi Yu, Yinge Xie, Zilin Qian, Yu Zhong, Huolin Shen, Wencai Yang*

* Correspondence should be addressed: yangwencai@cau.edu.cn

This PDF file includes

Figs. S1 to S4

Tables S1 to S6

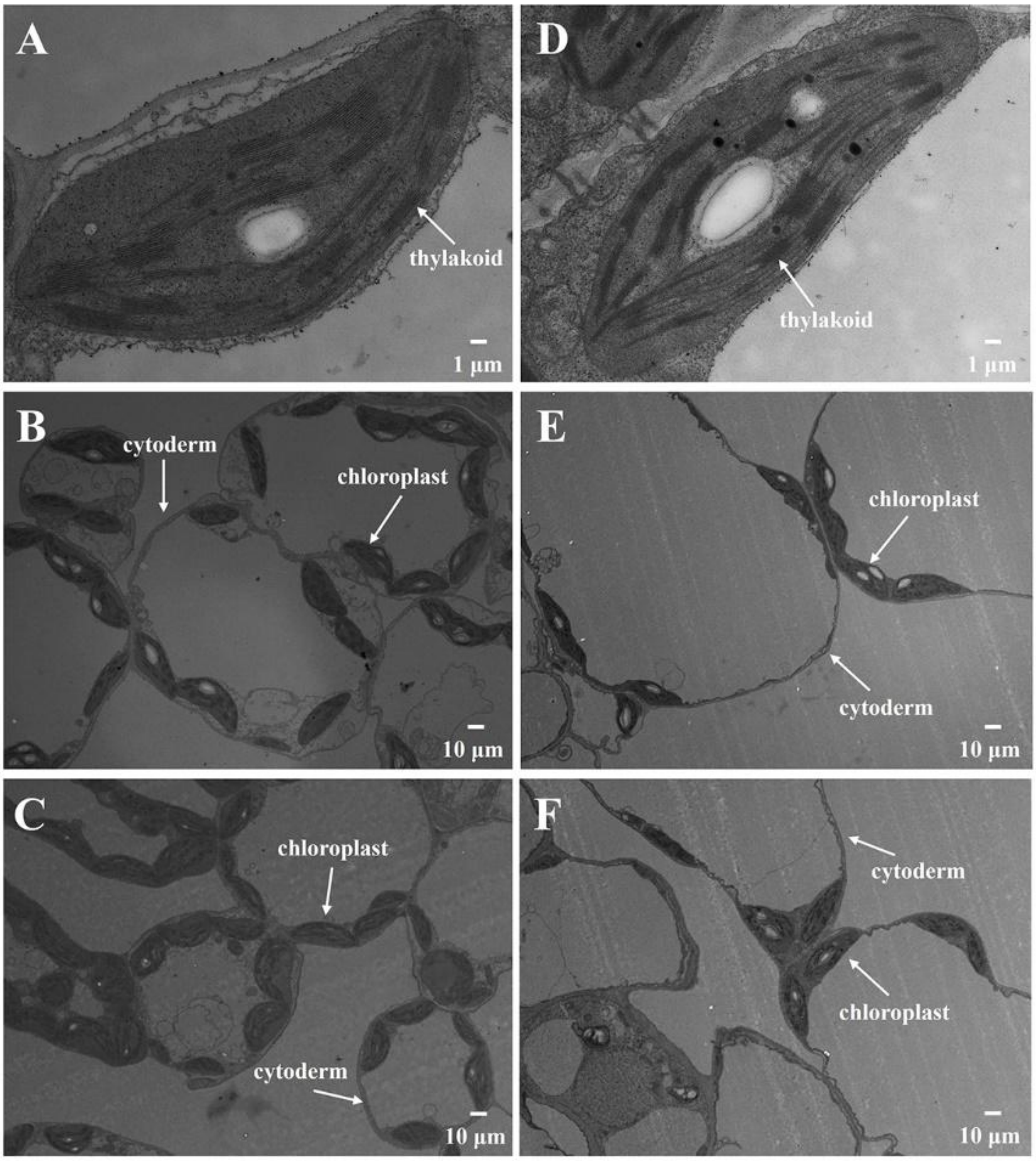

Supplementary Figure S1. Transmission electron microscopy of plastid in OH 88119 and *yfm* leaf. A, B and C are the plastid in OH 88119, D, E and F are the plastid in *yfm*.

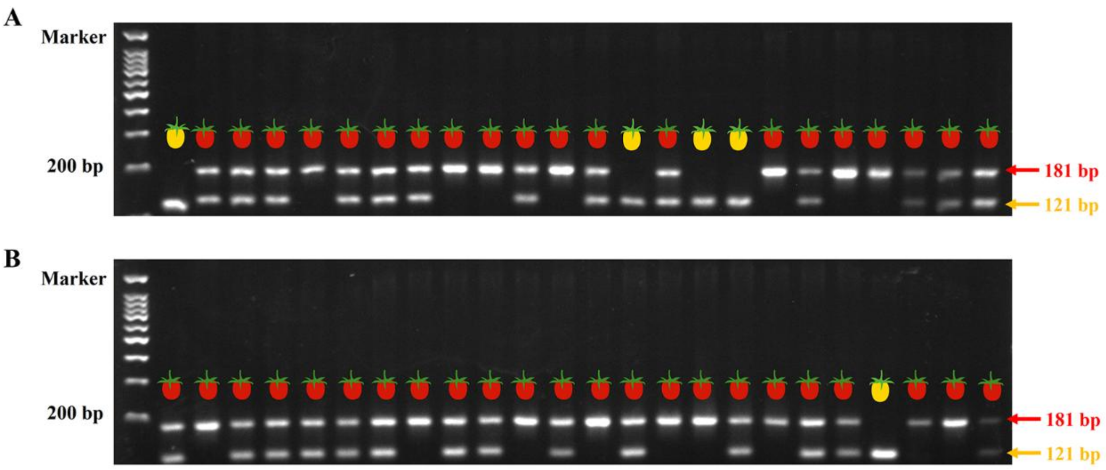

dCAPs-*Bam*H I-F:
5'-CGTCAATATATTCAAATGTTGTTATGATATGCAGGTTACAACGCCTGATGATTTGGATC-3'
dCAPs-*Bam*H I-R:
5'-GGAAGGTTAACTTCACCTATTCAGA-3'

Supplementary Figure S2. Images of agarose gel electrophoresis for partial individuals in the F_2 populations of OH 88119 x *yfm* (A) and *yfm* x PI 128216 (B) using the dCAPs marker. The bands in electrophoretogram show the DNA fragment digested with the restriction enzyme *Bam*H I. The fruit color of each individual plants is indicated in the corresponding lane.

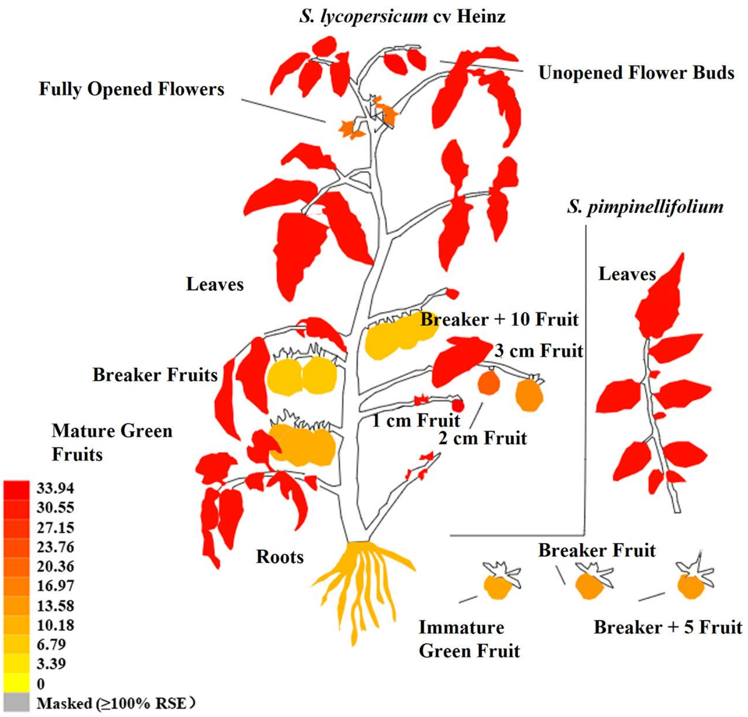

Supplementary Figure S3. Spatial and temporal expression analysis of *SlMCT* gene. Images and data analysis using ePlant (https://bar.utoronto.ca/eplant) database.

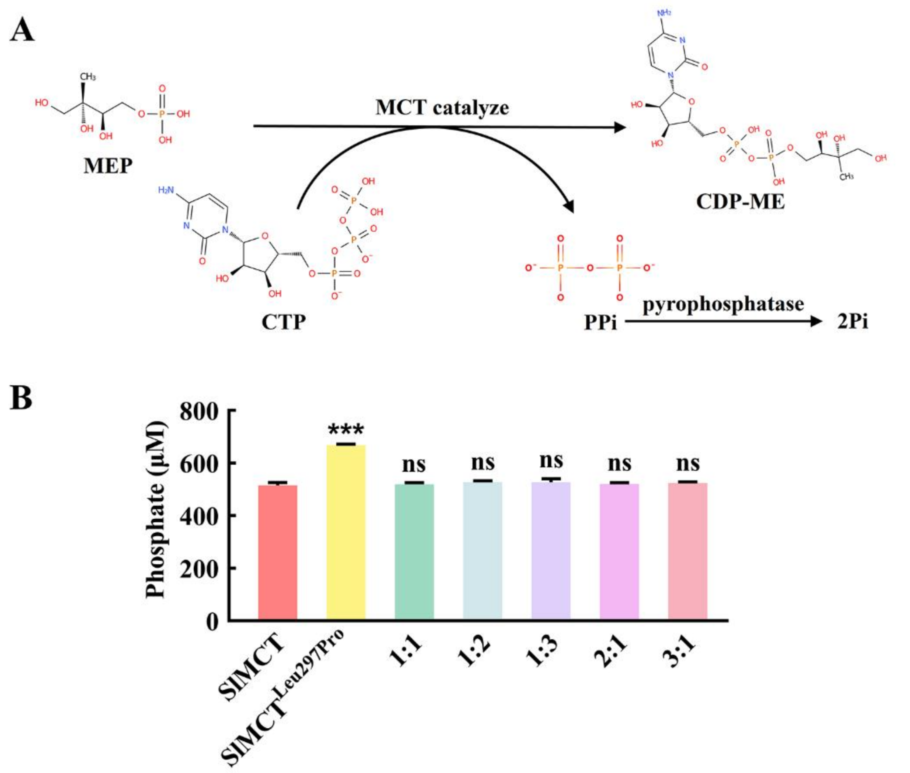

Supplementary Figure S4. Catalytic activity assay of wild type SlMCT and mutant SlMCT$^{\text{Leu297Pro}}$. A. Schematic diagram of the catalytic reaction of SlMCT. MEP, 2-C-methyl-D-erythritol-4-phosphate; CTP, cytidine-3-phosphate; CDP-ME, cytidine diphosphate methylerythritol; PPi, cytidine-3-phosphate. B. The concentration of phosphate is calculated by measuring the absorption value at 630 nm. 1:1, 1:2, 1:3, 2:1 and 3:1 indicate proportions of proteins between wild type and mutant mixed for enzymatic activity assay. Significance analysis is analyzed by student's t-test (***, P<0.001, ns, no significance).

Table S1 Information for primers used in this study.

Marker name	Chromosome	Forward sequence (5'-3')	Reverse sequence (5'-3')	Purpose	Reference
sli41	1	CATCGCAAATACACGCACAT	AGGAAACCACATTTTGTGAGG	Preliminary mapping	Yang et al. 2014
sli123	1	CCAAGATGCCAATGATAACG	CAACACAGTCCGAACTTCCA	Preliminary mapping	Yang et al. 2014
sli166	1	TGCAACTTCCTTTGTTGTCG	TGAGTTGGACCTACCCCAAG	Preliminary mapping	Yang et al. 2014
sli205	1	CATGTGAATCCAAAGCATGTG	TCACCCTACACACTCCTAGAAACA	Preliminary mapping	Yang et al. 2014
sli256	1	AAACCCCCACTAACCCAAAG	GGGGTTTATAGGGTTCAAAGC	Preliminary mapping	Yang et al. 2014
sli2817	1	CATGGTGCTTTTACTTGCCC	TATGAAATGCCTGAAGCGTG	Preliminary mapping	Yang et al. 2014
sli2843	1	ATGTCCTCTCACCAAATGGC	TTGAGGAAATTCGGAACGAC	Preliminary mapping	Yang et al. 2014
sli2871	1	ATGACAATGAGCACCTGCAT	CCCAGGCAAACCAAAATAGA	Preliminary mapping	Yang et al. 2014
sli2914	1	CAGCTATGGCGAAGTGACAA	CCACGAGCAAAGAACAAAAA	Preliminary mapping	Yang et al. 2014
sli2953	1	GTGTTTGCATCTCGTCTGGA	GTGTCACAATCAGCACAGCC	Preliminary mapping	Yang et al. 2014
sli42	2	TGAGTCTGAGGCCCCTTATG	TCCTCCTCTCGACCCCTACT	Preliminary mapping	Yang et al. 2014
sli297	2	CCGATTCCAATCCTATCCTTT	TGTAGCTCACATGGTCTACATCG	Preliminary mapping	Yang et al. 2014
sli334	2	TTGGTTGTATCACCAACGATGT	GGTTCTAAGGCCAATTTGGAG	Preliminary mapping	Yang et al. 2014
sli2703	2	GATGTTGAGTTAGGCCCACG	AATCATCCCCTCACCCATTT	Preliminary mapping	Yang et al. 2014
sli2753	2	CCCATCAACTGGAGTCACCT	AACCAAACCAATTCCCCTTC	Preliminary mapping	Yang et al. 2014
sli2794	2	TAAGCTGTGCGCCCTTATTT	ATGCCTAGTAGATGCAGGGG	Preliminary mapping	Yang et al. 2014
sli43	3	ACCCAACTTTGACCATCCAC	ATGCAATGATGGAGGCTAGG	Preliminary mapping	Yang et al. 2014
sli2471	3	TGAGTTTGCCCCTACATGAAA	ACAATGCCTTCATCTGACCC	Preliminary mapping	Yang et al. 2014
sli2507	3	TGAAATGAGCCCGAAAGACT	AGTTCGATCACCTGGTCGTT	Preliminary mapping	Yang et al. 2014
sli2545	3	ATGTGCGCAACCACTATCAA	GCAACTGCAAGTCAACATGG	Preliminary mapping	Yang et al. 2014
sli2574	3	GGCTTTAGGCCCATTTGAGT	AAAGGCGTATAGGTGTCGGA	Preliminary mapping	Yang et al. 2014
sli2617	3	GAGGTATTGGGAAGGTGCAT	CATCCACTGCCAAAAGATGA	Preliminary mapping	Yang et al. 2014
sli2666	3	CATGCAAAGCGGTGTTTAGA	CGGTGTTGAGATTGTTCCAA	Preliminary mapping	Yang et al. 2014
sli2181	4	CGCGATCACTCAAAGTTGTT	CCAAAAGGGACCATTCCAA	Preliminary mapping	Yang et al. 2014
sli2227	4	AGCCCCATGGCATTTATGTA	ATAAACGCCAATTTCCTTGG	Preliminary mapping	Yang et al. 2014
sli2256	4	TGGTGGCCTTCTTAATCACA	CCCCACAACGTAAAAAGGTG	Preliminary mapping	Yang et al. 2014
sli2297	4	CCCATTGGGAAGCAGTAGAA	TGGTATGCCTCCGGATAGAG	Preliminary mapping	Yang et al. 2014
sli2331	4	GGTGCATACAAGCCATCTGA	TGAGATTGACTGCCACTTTGTT	Preliminary mapping	Yang et al. 2014
sli2370	4	TTTCCTGTCTTGTCTCTTGCC	AAAGCCCTTTAGCTTTTGCC	Preliminary mapping	Yang et al. 2014
sli2417	4	AAGGAGGATGTCAACTGGCT	TCCCTCTCCGTGTCCAAATA	Preliminary mapping	Yang et al. 2014
sli346	5	GCCAAAGAACAATCATTCCC	TCGGTTTAGAGCTGTTGCCT	Preliminary mapping	Yang et al. 2014
sli389	5	CATTTGGGGATGAATGTTCC	GCATTCTTGGACCTTTTGGT	Preliminary mapping	Yang et al. 2014
sli441	5	TGGTGGGCTGGTCTAGGATA	AATATCGGCATAGAGTGCGG	Preliminary mapping	Yang et al. 2014
sli485	5	GGGCAGACACTGGATTTCAT	ATCGGCTATGCCCATTACAT	Preliminary mapping	Yang et al. 2014
sli518	5	CCCAAGTTTGAAAACCCAGA	TTGTGACCGGATGGTTTGTA	Preliminary mapping	Yang et al. 2014
sli551	5	AGTTGGGGTCATGGTTTGAA	TTGATCCTTCCCTTTCTGGA	Preliminary mapping	Yang et al. 2014
sli593	5	TTGGCCTCGAACAAAGAAAG	AAGGTTACCTCCAACCAGCA	Preliminary mapping	Yang et al. 2014
sli601	6	GTCTGGCCTTGTTGATGGTT	CAAGCTCCCAAAGGCAGTAG	Preliminary mapping	Yang et al. 2014
sli647	6	AGAACTCATGCTTTGACGCC	TGGTGGTGGTGATTATGTGC	Preliminary mapping	Yang et al. 2014
sli685	6	GAACCCCCTGCTGATTCATA	TCCTCCTTCGTTGTTGCTCT	Preliminary mapping	Yang et al. 2014
sli728	6	CTAATTCTCGCTCATTCGGC	TAAAACATGGTGAGCCCAGG	Preliminary mapping	Yang et al. 2014
sli775	6	TGACACATGTCATTGGCCTT	TCGTCGATGACGTGTCAAAT	Preliminary mapping	Yang et al. 2014
sli778	7	TCGCAAGCCAAGACTATTCA	GGAGAAAGGAATGAGATGCC	Preliminary mapping	Yang et al. 2014
sli818	7	ATGATCACCCCTCTTTCTCG	TGACACATATCAAAGGGCGA	Preliminary mapping	Yang et al. 2014
sli850	7	TAGCTTTTGAACAAGGGGGA	TGCAATGGCCTTGTTTATGA	Preliminary mapping	Yang et al. 2014
sli887	7	CGAGCGCATCCTGAAACTA	CTTGCATAACTTCCATGCCC	Preliminary mapping	Yang et al. 2014
sli924	7	TGCCCAAAGATTATGACATGC	TTCAGTCCCATTCGTCACAA	Preliminary mapping	Yang et al. 2014
sli966	7	TTGTTCGATTTTTCCCCTCA	TCTGGTTTCACATCAGCCAA	Preliminary mapping	Yang et al. 2014
sli1016	7	TCACAAGCTAGACAACCCCC	AACCCCTCACGCCTAAGACT	Preliminary mapping	Yang et al. 2014
sli1031	8	GGCTGAATGCAGGAAAAGAG	TCATCTTCTTCGTTGGTCCC	Preliminary mapping	Yang et al. 2014
sli1072	8	TTCCAAAGGAAGGTGTTTGG	GACGTTAGACAAATCTTAGGCGA	Preliminary mapping	Yang et al. 2014
sli1108	8	AAGGTACACGACCCTAACACA	TTGGGCTAGTCCCATTCTTC	Preliminary mapping	Yang et al. 2014
sli1143	8	AAGTGGTAAACCCAAATGACC	TGCATTATCGTCTCCAATCTCA	Preliminary mapping	Yang et al. 2014
sli1184	8	CCTAAGGCCCCAATTCATTC	GAACCTCTTGGGGAAAGGAG	Preliminary mapping	Yang et al. 2014
sli1223	8	TCCCCGATAAGAAAGCAAGA	CTTCATCTTCCTCGTCGTCA	Preliminary mapping	Yang et al. 2014
sli1273	8	TGGAGGAAAACAAAAGTGTGAG	GGAATTCATGGATAGTGGTTGA	Preliminary mapping	Yang et al. 2014
sli1274	9	ATGTTTTGCCCGTTTGAGTC	CACGTGCATGACGTAGATGTT	Preliminary mapping	Yang et al. 2014
sli1481	9	TCAAATCCACCCTTGAACCT	AAAACTACCAATTCCGTCGAGA	Preliminary mapping	Yang et al. 2014
sli1341	9	AATACCGGGTTGGATTAGGG	TAACCGGTGATGAAACCTCA	Preliminary mapping	Yang et al. 2014
sli1365	9	GCACCAGCAATGTTAAACGA	GGTCACCTTGTTTAATGGCG	Preliminary mapping	Yang et al. 2014
sli1517	9	TGTTCATGCATGTGTGTTCG	AGCATTTTTGCATCGCCTAT	Preliminary mapping	Yang et al. 2014
sli1526	9	GTCGCCCTTCTATTGTTCCA	CTCCACTGCGACGAGATGTA	Preliminary mapping	Yang et al. 2014
sli1444	9	AGGGTCCTCTCCAAACCCTA	CCCTTGTTAAACCAACTCCC	Preliminary mapping	Yang et al. 2014
sli1475	9	CGACTTTCTTTCCCACAACA	ATCCCAACCCATCATTTTGA	Preliminary mapping	Yang et al. 2014
sli1537	10	CATGACCAAGGCGAGAAGTT	GCTGGAATCAGAAGCCAGAT	Preliminary mapping	Yang et al. 2014
sli1575	10	CCTTTCACTTTAGACACCACTTCA	TTCTTCACCCTTAACCCTAGACT	Preliminary mapping	Yang et al. 2014
sli1605	10	TGTCACATTAAGGTCAGTCTCCA	GACGTGCAAAGATTTGCTGA	Preliminary mapping	Yang et al. 2014
sli1640	10	ATGAGCGTCAACCACATGAC	GGCAGTCAAACGATTTCCAT	Preliminary mapping	Yang et al. 2014
sli1681	10	CCACCCCTCAAGAAAAAGAA	TTTTTGGGTGTTCTGGGGTA	Preliminary mapping	Yang et al. 2014
sli1721	10	GCAGGGGATAGCATTTTGAA	TCAACCACATTGTCAAACCG	Preliminary mapping	Yang et al. 2014
sli1762	10	GGGTGGTGCATAAATTTGGA	CAGCAAGTGTGAGTTCATCG	Preliminary mapping	Yang et al. 2014
sli1782	10	TCCATTGTTCCATTTAGGCTG	CTTTTGGTGCAGGCTTTCTT	Preliminary mapping	Yang et al. 2014
sli1786	11	GCAATACCAAATGGACACCA	AGTGCTTCTTGCGTCTCAAA	Preliminary mapping	Yang et al. 2014
sli1822	11	ACGATGAAATAACTTGGCGG	GCATGTTTCCAGTTCAGCAG	Preliminary mapping	Yang et al. 2014
sli1842	11	TTTGGGTGGGTAGAAGGTCA	TGAAGAGAGTAGGGTTGGCTC	Preliminary mapping	Yang et al. 2014
sli2985	11	GGTTATTTTTCCTTAGTTTTACAC	ACCCTCTTCTTATTCTTATTCTTC	Preliminary mapping	Yang et al. 2014
sli1896	11	GCGAAGAAAGAACTTGGGAA	ATAGTGGATCAAGATGCGGC	Preliminary mapping	Yang et al. 2014
sli1920	11	AAGAAGGTCCTTGAGGAGGG	CCTAGTGGCCTTCTGCTTGA	Preliminary mapping	Yang et al. 2014
sli3000	11	GTTTGCCACTATTAGGATTTTGAG	AGTGGGTTTCTTGCTTGTTTTC	Preliminary mapping	Yang et al. 2014
sli1927	12	CGAAATGAGTATTGACCGATGA	TTGCTCCCTCCATAAGGAAA	Preliminary mapping	Yang et al. 2014
sli1970	12	CTTAGCTGGGGCTTGTCCTA	AAGTTAGCCCTGATGGCGT	Preliminary mapping	Yang et al. 2014
sli2008	12	AGGCCCCACCAAAAGAGTAT	GGAAGGACAAGGTTCAAGGG	Preliminary mapping	Yang et al. 2014
sli2161	12	TGGGTTCTCCTATTTTGGACAT	TAACCCGAAACCTTAAGCCC	Preliminary mapping	Yang et al. 2014
sli2176	12	TTCGATAGAGGGTCGGATTG	AGATCTTGGCCCTCTCCATT	Preliminary mapping	Yang et al. 2014
sli2177	12	AACACCACCACCACCACC	TGTTGTTGTTGTTGCGGC	Preliminary mapping	Yang et al. 2014
sli2125	12	TTCATAGCCACATAAGGCCC	CCTTGGGTCATGAAAATTGC	Preliminary mapping	Yang et al. 2014
sli2148	12	AAAGGCATCAACTGCTCAGAA	TGTGGAATCACCAGGAAATG	Preliminary mapping	Yang et al. 2014
sli2909	1	CGTCGATTGGTTGAAGGATT	ATTTTTCAGGGCTGCTCTTG	Fine mapping	Yang et al. 2014
sli2914	1	AGAGTTCCACATTGGTTGGG	ACCAACTTGGGTGGATTTGA	Fine mapping	Yang et al. 2014
sli2921	1	GGGCCGTATCACACTGTCTT	AAGGCGACTAGTGTGCCATATT	Fine mapping	Yang et al. 2014
Indel-14	1	CAAATGCTAATTAAACAATCAGTAG	TAGCTGTCTAAATCCTATTCGTACT	Fine mapping	This study
Indel-19	1	ATGCTCTGAAAAATCTATTAATAAA	AAGAGGAGAATAGACACAGAGTCTA	Fine mapping	This study
Indel-22	1	GGACAATTCTCCATCTGTCTCTATC	AATTTTTTTGCATATAGAGGAAAGA	Fine mapping	This study
sli2922	1	AGAGTTCCACATTGGTTGGG	ACCAACTTGGGTGGATTTGA	Fine mapping	Yang et al. 2014
Indel-15	1	GAGCTGAATCATACTATTTTTTACC	GCATTAATTTGAATAATTTTGTGAT	Fine mapping	This study
Indel-26	1	GAACCTGTATTCAATTTTATCCACT	AGGTAGGAAAGTCTACGTACACAAG	Fine mapping	This study
Indel-29	1	CAAGTGTGACATTAGAATTTCAGTG	ATTAAAAATTGATATATCACGAAGG	Fine mapping	This study
Indel-28	1	AAAAAGACTTTTACATGAGATTTGA	AAAGGGTTTTTGGTAAATAGATTAC	Fine mapping	This study
Indel-30	1	ATTATCCAAATTAAAAAGGTGTCTC	CAAGACTTGTACATCTTTATTAGGG	Fine mapping	This study
Indel-49	1	GTACGTTTTTGAGGATTAGTAATAT	AGTCCATCTTCAACTCAAACACTAA	Fine mapping	This study
Indel-50	1	AAGAGAATATTTTTTTTCAAACAAA	AGTAAAAACGAATTTTATTTATTCA	Fine mapping	This study
Indel-56	1	TCTTGATTGTAAACTTCCATATGAT	ATTCGAAATGTGATATATAAAATGG	Fine mapping	This study
Indel-52	1	AACCTTTCAGAAAGTTCACAATCTT	CAAAACTAGCCTTTCATGAACTTTA	Fine mapping	This study
sli2924	1	ATCGAACCCAAGAATTTCCC	TTTTCTCCGGCTTTAGGGTT	Fine mapping	Yang et al. 2014
sli2926	1	CGGCAAAAGCTCCTAGAACA	GAAAGATAATGTGACAACCCCA	Fine mapping	Yang et al. 2014
sli2929	1	AGAGTTCCACATTGGTTGGG	ACCAACTTGGGTGGATTTGA	Fine mapping	Yang et al. 2014
sli2932	1	AAAAAGGATTATGTGGGGCG	AATAGGGATGGCAATTGGAC	Fine mapping	Yang et al. 2014
sli2944	-	CCATGCCATTTGCAGTAAAA	TCACGGGTGTTATCACGAAA	Fine mapping	Yang et al. 2014
SlMCT	-	TGAGGGAGTAAAGTACAACAACATG	TTCCAGGGAATTATAATGTTTGAAC	*SlMCT* gene cloning.	This study
SlMCT-pro	-	AAATTTTTAGGATATGGGTTGTTGA	GAGATAGAAGGCAATACATTCTTGG	The upstream of *SlMCT* gene cloning.	This study
SlMCT-qPCR	-	TTGGAGCAGCAGTTCTTGGT	TGACAAGCTCGAAACCCTTCT	Using for RT-qPCR.	This study

UBI-3-qPCR	-	TGGTCGGAATGGGACAGAAG	CTCAGTCAGGAGAACAGGGT	RT-qPCR of reference gene.	This study
1305-pro	-	TACGAATTCGAGCTCGGTACCAAATTTCGATTGTAATATGCAAAAT	CTTGCATGCCTGCAGGTCGACGAAAACTGAAAATGAATGAAAATAG	Using for the functional complementation of *SlMCT* . Kpn I and Sal I restriction are underlined.	This study
1305-CDS	-	GACCTGCAGGCATGCAAGCTTATGTCTACTCTTCAATTGGGTATCA	TTACCCTCAGATCTACCATGGCTAAGATTCTTCAATATTCAATATT	Using for the functional complementation of *SlMCT* . Hind III and Nco I restriction are underlined.	This study
1305-genome	-	TACGAATTCGAGCTCGGTACCAAATTTCGATTGTAATATGCAAAAT	TTACCCTCAGATCTACCATGGAACTTCATAACAAGCCAATTTTCAT	Using for the functional complementation of SlMCT. Kpn I and Nco I restriction are underlined.	This study
1300-CDS	-	CAAATCGACTCTAGAAAGCTTATGTCTACTCTTCAATTGGGTATCA	GAGCTTTTGCTCCATGGTACCCTAAGATTCTTCAATATTCAATATT	Using for over-expression of *SlMCT* . Hind III and Kpn I restriction are underlined.	This study
VIGS-SlMCT	-	GTGAGTAAGGTTACCGAATTCATCTTCCACTTCTAGGCCAGCCAAT	CGTGAGCTCGGTACCGGATCCCCAGCCAACCATCCTTCAGGACCTT	Using for VIGS *SlMCT* gene. Eco RI and Bam HI restriction are underlined.	This study

Table S2 Candidate genes in the fine mapping region between Indel-29 and Indel-56 markers on chromosome 1.

Number	Gene ID	Physcial location	Description
1	Solyc01g102800	83773538--83780518(+strand)	Histidine--tRNA ligase
2	Solyc01g102810	83785370--83791556 (+ strand)	DNA polymerase III subunit gamma/tau
3	Solyc01g102820	83791747--83796964 (- strand)	4-diphosphocytidyl-2C-methyl-D-erythritol cytidyltransferase
4	Solyc01g102830	83800745--83801582 (+ strand)	NADH dehydrogenase [ubiquinone] 1 beta subcomplex subunit 3-A
5	Solyc01g102840	83804275--83809437 (+ strand)	Disease resistance protein (TIR-NBS-LRR class) family
6	Solyc01g102850	83811356--83815538 (+ strand)	Disease resistance protein (TIR-NBS-LRR class) family
7	Solyc01g102860	83816023--83817132 (+ strand)	golgin family A protein
8	Solyc01g102870	83822525--83824924 (+ strand)	receptor-like protein kinase BRI1-like 3
9	Solyc01g102880	83825339--83837695 (- strand)	Disease resistance protein (TIR-NBS-LRR class) family
10	Solyc01g102890	83838985--83840113 (- strand)	Germin-like protein subfamily 1 member

Table S3 Differentially expressed genes (DEGs) between OH 88119 and *yfm* in transcriptome.

Index	*yfm* -0 DPA-1	*yfm* -0 DPA-2	*yfm* -0 DPA-3	OH 88119-0 DPA-1	OH 88119-0 DPA-2	OH 88119-0 DPA-3	baseMean	*yfm* -0 DPA	0 DPA OH 88119-0 DPA	log2FoldChange	pvalue	padj	regulated	Annotation
Solyc06g035940.3	9	10	24	1079	2275	2284	957.5819	13.2698	1901.89411	7.146310467	3.86E-92	8.36E-88	up	--
Solyc09g011080.3	2265	1609	1589	68	97	93	911.4542	1732.4	90.5077036	-4.264028763	1.37E-63	1.49E-59	down	26S proteasome regulatory complex, ATPase RPT4
Solyc06g071830.2	1559	1285	1139	51	57	89	666.6522	1264.36	68.9423163	-4.201728173	9.20E-52	6.65E-48	down	CREB binding protein/P300
Solyc04g079910.4	651	587	555	14	51	34	300.2035	567.937	32.4700153	-4.095508772	2.85E-40	1.24E-36	down	Uncharacterized conserved protein
Solyc04g080620.3	482	394	554	22	54	59	246.9655	449.106	44.8249964	-3.297708304	6.77E-40	2.45E-36	down	--
novel.90	387	397	499	38	75	81	234.3791	403.151	65.6067338	-2.604081239	5.23E-39	1.62E-35	down	Tam3-transposase (Ac family)
Solyc07g006630.4	1069	1019	1216	98	204	237	610.5734	1040.28	180.866992	-2.514028166	6.65E-39	1.80E-35	down	GATA-4/5/6 transcription factors
novel.1323	5	8	12	126	229	248	106.591	7.78804	205.394002	4.71108628	4.16E-38	1.00E-34	up	--
Solyc02g077590.1	131	153	166	497	648	729	399.8816	141.762	658.001578	2.212259667	8.93E-36	1.76E-32	up	Transcription factor HEX
Solyc01g107460.2	416	499	454	27	26	18	229.7419	433.031	26.4526795	-4.082710092	2.86E-35	5.17E-32	down	--
Solyc10g005030.4	937	948	875	120	239	219	535.306	873.885	196.727212	-2.145126934	8.24E-35	1.28E-31	down	GATA-4/5/6 transcription factors
Solyc09g097960.3	186	195	165	3	6	9	89.57979	173.17	5.98950154	-4.806591836	4.25E-30	5.42E-27	down	Voltage-gated shaker-like K+ channel
Solyc05g024230.3	201	191	222	21	20	19	107.7341	193.452	22.0162331	-3.192364486	1.28E-29	1.54E-26	down	--
novel.1175	304	282	305	14	49	38	157.176	281.278	33.0742541	-3.049182674	9.69E-28	1.00E-24	down	--
Solyc04g079230.4	308	298	320	40	71	87	179.7269	292.266	67.1881619	-2.107212223	9.64E-27	9.50E-24	down	Ca2+-independent phospholipase A2
Solyc09g082690.3	4928	4344	4559	563	574	522	2488.428	4371.4	605.450493	-2.855080623	1.05E-26	9.87E-24	down	--
novel.889	2196	1298	1829	147	269	322	963.9101	1677.78	250.037233	-2.743518905	2.13E-25	1.78E-22	down	--
novel.1291	53	66	66	193	260	299	160.6052	58.3674	262.843085	2.168550739	3.19E-25	2.56E-22	up	--
Solyc08g014430.3	396	315	401	47	79	52	206.3251	350.195	62.4551377	-2.495811338	4.36E-25	3.38E-22	down	Rho GTPase effector BNI1 and related formins
Solyc08g079900.3	38	42	27	176	355	258	152.4646	34.1222	270.80702	2.995541394	2.54E-24	1.90E-21	up	--
Solyc06g049050.3	995	928	988	92	250	180	546.9341	919.266	174.602356	-2.388382084	5.07E-24	3.66E-21	down	--
Solyc12g041930.1	131	108	134	4	11	13	63.34436	117.497	9.1921249	-3.617151724	1.39E-23	9.72E-21	down	--
Solyc09g010530.3	158	153	164	14	26	33	87.27883	149.926	24.6320669	-2.581659585	3.03E-23	1.99E-20	down	Predicted K+/H+-antiporter
Solyc07g005370.4	720	524	522	81	127	126	337.8268	559.796	115.857299	-2.273431064	3.95E-22	2.45E-19	down	--
Solyc02g070940.1	12049	8176	9702	1727	2542	2305	5880.551	9455.99	2305.10916	-2.036736358	4.56E-22	2.75E-19	down	--
Solyc04g015750.3	6956	5953	5543	598	1602	1256	3501.615	5849.29	1153.93538	-2.340509374	9.36E-20	4.72E-17	down	--
Solyc01g090790.4	431	494	465	59	143	101	270.9331	439.472	102.394187	-2.092255731	1.52E-19	7.47E-17	down	--
Solyc04g080040.3	225	217	185	13	27	41	112.9063	198.953	26.8595193	-2.85939704	3.43E-19	1.55E-16	down	Auxilin-like protein
Solyc09g092490.3	1466	1379	1450	145	400	365	829.0715	1356.62	301.526892	-2.163709414	4.76E-19	2.11E-16	down	UDP-glucuronosyl and UDP-glucosyl transferase
Solyc02g077140.3	259	299	309	32	51	81	164.4628	273.483	55.4424654	-2.289970274	9.40E-19	3.92E-16	down	Predicted lipase
Solyc05g012230.4	219	209	179	13	37	39	110.9368	192.603	29.2704194	-2.682604121	1.47E-18	6.01E-16	down	--
Solyc05g005280.4	1520	1489	1660	295	276	304	895.5576	1472.14	318.97749	-2.212560927	3.82E-18	1.54E-15	down	--
Solyc10g084370.3	111	112	113	16	17	17	62.11736	106.168	18.0670359	-2.598454859	1.08E-17	4.20E-15	down	Zuotin and related molecular chaperones
Solyc04g077755.1	110	99	139	15	17	19	63.6838	109.185	18.1823436	-2.617686493	1.41E-17	5.35E-15	down	NADH:ubiquinone oxidoreductase
Solyc08g082590.3	480	421	314	43	88	81	229.4272	386.888	71.9668312	-2.418961575	1.84E-17	6.76E-15	down	Glutaredoxin-related protein
Solyc06g011350.3	909	916	594	79	165	165	454.9054	771.706	138.105291	-2.47718939	2.40E-17	8.55E-15	down	Aquaporin (major intrinsic protein family)
Solyc03g033590.1	171	219	201	32	55	45	116.2973	186.81	45.7840638	-2.030531745	5.25E-17	1.81E-14	down	--
Solyc06g075650.3	98	110	95	11	15	15	55.21293	96.0066	14.4192853	-2.751285607	7.17E-17	2.43E-14	down	Aquaporin (major intrinsic protein family)
Solyc12g006050.2	2233	2117	2256	113	423	396	1193.974	2085.76	302.191172	-2.782294862	2.05E-16	6.55E-14	down	H+/oligopeptide symporter
Solyc02g077430.4	60	96	100	216	474	380	221.7802	80.5333	363.027024	2.173845698	4.63E-16	1.41E-13	up	Predicted lipase
Solyc01g044365.1	17	7	13	60	150	178	69.93537	11.6409	128.229863	3.466641864	1.02E-15	2.95E-13	up	--
Solyc10g085140.2	1270	1418	1182	101	312	299	730.4283	1227.26	233.598548	-2.387079762	2.43E-15	6.42E-13	down	Cis-prenyltransferase
Solyc08g079870.3	249	363	228	745	1858	1576	833.5225	267.595	1399.45024	2.388168157	3.27E-15	8.34E-13	up	--
Solyc11g012980.1	16	10	7	65	85	96	48.43825	10.5456	86.3309184	3.040580804	5.08E-15	1.24E-12	up	--
Solyc11g005290.1	130	76	78	1	5	5	46.82876	90.1531	3.50440637	-4.592853357	5.13E-15	1.24E-12	down	FOG: Predicted E3 ubiquitin ligase
Solyc08g008280.3	183	162	123	1151	775	1211	655.0128	148.96	1161.06562	2.962226944	2.21E-14	5.04E-12	up	--
Solyc06g062920.3	25	23	22	247	156	201	125.2235	22.1631	228.283849	3.360634791	3.92E-14	8.25E-12	up	Serine/threonine protein kinase
Solyc04g081700.3	105	99	119	17	14	11	58.76535	101.69	15.8402563	-2.757286272	3.98E-14	8.29E-12	down	--
Solyc03g083470.3	25	36	21	140	139	227	102.463	26.1641	178.76198	2.775242478	4.89E-14	9.99E-12	up	Serine/threonine protein kinase
Solyc02g080120.3	210	178	177	18	38	55	107.8495	178.857	36.8416855	-2.254175899	5.77E-14	1.16E-11	down	Iron/ascorbate family oxidoreductases

Solyc10g085870.1	352	196	211	22	13	30	132.2648	240.816	23.7139781	-3.367336872	1.15E-13	2.21E-11	down	UDP-glucuronosyl and UDP-glucosyl transferase
Solyc01g110600.2	89	90	104	9	21	26	53.84831	89.1256	18.5710615	-2.223354332	3.70E-13	6.70E-11	down	--
Solyc01g008620.4	76	117	80	274	441	323	225.4897	86.7828	364.196635	2.069944662	6.64E-13	1.16E-10	up	--
Solyc05g017790.1	0	0	0	72	69	80	40.04853	0	80.0970524	8.835392672	9.16E-13	1.58E-10	up	--
novel.884	55	58	83	3	0	3	31.86796	61.3658	2.37012383	-4.864239159	1.10E-12	1.87E-10	down	--
Solyc01g087270.3	84	77	72	4	16	12	42.10454	73.8115	10.397575	-2.76462642	1.27E-12	2.08E-10	down	Beta, beta-carotene 15,15'-dioxygenase and related enzymes
Solyc01g087800.2	11	17	24	50	104	120	54.10395	16.2306	91.9773221	2.503667506	6.30E-12	9.18E-10	up	--
Solyc08g041930.1	48	67	86	0	0	0	31.47566	62.9513	0	-8.332680249	1.63E-11	2.22E-09	down	--
Solyc02g037550.3	471	419	378	37	124	115	246.1285	402.107	90.1499818	-2.142416234	2.38E-11	3.13E-09	down	Predicted membrane protein
Solyc10g008660.2	1357	1239	1159	34	234	151	661.1374	1189.57	132.708523	-3.156788389	6.07E-11	7.44E-09	down	--
Solyc09g014990.4	69	58	50	313	228	301	184.3012	56.2111	312.391352	2.47186482	7.94E-11	9.57E-09	up	--
Solyc04g074450.1	1357	784	704	3416	3680	4890	2579.273	905.613	4252.93269	2.231520072	1.08E-10	1.25E-08	up	--
Solyc03g083480.4	42	39	27	284	152	304	155.3274	34.4276	276.227268	3.003400215	2.16E-10	2.33E-08	up	--
Solyc08g005610.3	152	72	83	629	444	738	380.5518	97.4309	663.672608	2.76746037	2.34E-10	2.50E-08	up	Cytochrome P450 CYP4/CYP19/CYP26
Solyc12g062200.1	11	4	8	82	63	66	43.09124	7.23649	78.9459935	3.434561469	2.44E-10	2.60E-08	up	--
Solyc07g016020.1	90	70	97	12	17	27	49.95913	80.7864	19.1318328	-2.068787739	2.57E-10	2.70E-08	down	--
Solyc12g038800.1	40	52	86	0	1	5	28.60662	55.4029	1.81036163	-4.778018732	4.68E-10	4.60E-08	down	--
Solyc05g055080.2	29	17	28	153	103	151	87.2807	23.2396	151.321751	2.694363231	5.76E-10	5.53E-08	up	--
Solyc05g053060.1	521	289	406	1314	1197	1810	966.11	383.59	1548.63021	2.012825296	7.78E-10	7.36E-08	up	--
Solyc07g055560.4	877	768	745	35	168	179	439.2705	756.79	121.751228	-2.626670845	8.49E-10	8.00E-08	down	Cytochrome P450 CYP4/CYP19/CYP26
Solyc08g008480.4	49	55	41	3	3	8	25.43878	46.0942	4.78337869	-3.253532366	1.11E-09	1.03E-07	down	Transcription factor, Myb superfamily
novel.953	64	63	99	271	227	290	179.4488	70.6341	288.263445	2.022474132	1.66E-09	1.49E-07	up	--
novel.979	36	63	53	5	2	4	26.14965	48.048	4.25131661	-3.614813018	1.80E-09	1.58E-07	down	--
Solyc04g015360.3	304	200	188	844	708	1029	577.9364	219.824	936.048897	2.089674278	2.27E-09	1.98E-07	up	GATA-4/5/6 transcription factors
novel.128	69	59	137	10	14	10	47.10742	82.0942	12.1206654	-2.784361021	2.41E-09	2.07E-07	down	--
Solyc11g006300.2	411	343	373	89	77	63	220.881	356.083	85.678705	-2.072718677	2.64E-09	2.25E-07	down	Steroid reductase
Solyc05g054250.1	17	12	12	62	54	86	42.75123	12.999	72.5034685	2.473874179	2.94E-09	2.47E-07	up	Copper chaperone
Solyc10g009150.3	240	136	124	578	689	625	418.8318	159.139	678.524377	2.091840397	3.15E-09	2.60E-07	up	--
Solyc11g071740.2	425	390	287	2650	1323	2141	1344.852	350.889	2338.81571	2.736436518	3.86E-09	3.10E-07	up	Calmodulin and related proteins (EF-Hand superfamily)
Solyc11g073055.1	42	17	17	232	134	224	122.7458	24.2225	221.269159	3.190614523	5.18E-09	4.06E-07	up	--
Solyc11g071760.3	25	17	14	346	91	227	141.3525	17.8312	264.873747	3.891823831	5.57E-09	4.32E-07	up	Calmodulin and related proteins (EF-Hand superfamily)
novel.794	369	392	439	20	90	109	223.9162	378.043	69.7897082	-2.421051337	5.90E-09	4.54E-07	down	--
novel.1701	57	62	36	5	5	7	27.79609	49.5312	6.06100547	-3.05777344	7.30E-09	5.48E-07	down	--
Solyc11g018805.1	7	8	6	60	71	36	34.11822	6.67404	61.5623943	3.199567145	7.65E-09	5.70E-07	up	--
novel.1803	0	0	0	37	21	36	17.63006	0	35.2601232	7.646724888	7.86E-09	5.82E-07	up	--
novel.2066	1	4	6	21	41	66	22.96912	3.40783	42.5304132	3.635333987	1.23E-08	8.81E-07	up	--
Solyc11g042710.3	59	46	62	6	2	11	29.69104	52.53	6.85205591	-2.977535115	1.34E-08	9.41E-07	down	Dehydrogenases with different specificities
Solyc03g097050.3	299	221	253	1352	782	1113	737.9864	244.232	1231.74092	2.33338415	1.87E-08	1.29E-06	up	--
Solyc03g093120.5.1	79	42	34	289	204	324	174.9233	49.4672	300.379444	2.601748336	2.49E-08	1.67E-06	up	--
novel.1956	23	30	25	0	0	0	12.35425	24.7085	0	-6.980669843	3.39E-08	2.23E-06	down	--
novel.1894	839	664	869	16	133	142	418.6464	746.535	90.7577186	-3.030566311	4.24E-08	2.71E-06	down	FOG: Transposon-encoded proteins with TYA
novel.477	6	2	5	33	37	31	20.34767	4.07402	36.6213209	3.145614236	4.48E-08	2.85E-06	up	Tam3-transposase (Ac family)
Solyc07g056640.1	94	87	140	451	306	409	268.1025	100.329	435.876394	2.115127417	4.71E-08	2.98E-06	up	--
Solyc01g100010.3	150	91	106	588	378	484	328.4603	109.815	547.105659	2.314846408	4.75E-08	3.00E-06	up	--
Solyc01g007010.2	25	18	15	130	69	136	71.88559	18.4553	125.315881	2.759889411	5.13E-08	3.21E-06	up	--
Solyc08g082110.4	44	52	36	189	131	197	116.6177	42.0139	191.221572	2.182953183	5.96E-08	3.66E-06	up	--
Solyc03g083680.1	12	6	14	109	47	95	53.02056	9.9836	96.0575281	3.253219749	6.76E-08	4.10E-06	up	--
Solyc09g018610.1	34	52	62	8	6	7	27.11817	46.4091	7.82722691	-2.638790882	7.63E-08	4.56E-06	down	--
Solyc10g086250.2	104	69	70	12	13	25	47.19307	77.0637	17.3224804	-2.153866336	9.01E-08	5.31E-06	down	Transcription factor, Myb superfamily
Solyc12g088170.2	75	102	55	12	13	18	44.68522	74.1604	15.2099993	-2.299014847	1.10E-07	6.33E-06	down	--
Solyc04g025650.3	98	89	130	535	293	445	291.5921	99.3487	483.835471	2.280740921	1.29E-07	7.31E-06	up	Kynurenine 3-monooxygenase
novel.1729	239	173	219	13	65	59	121.3438	198.941	43.7465847	-2.160029644	1.34E-07	7.59E-06	down	FOG: Transposon-encoded proteins with TYA

Solyc02g063000.4	85	74	47	9	7	2	36.45492	65.8018	7.10801679	-3.301067551	1.40E-07	7.89E-06	down	UDP-glucuronosyl and UDP-glucosyl transferase
Solyc01g007000.4	17	8	6	256	62	108	93.09583	9.91528	176.276373	4.151639602	1.42E-07	7.95E-06	up	--
Solyc03g093080.3	63	42	42	352	168	251	172.4442	46.6309	298.257478	2.674405406	1.50E-07	8.32E-06	up	--
Solyc01g007040.4	13	5	3	47	42	78	32.94727	6.74656	59.147971	3.14003141	1.52E-07	8.37E-06	up	--
Solyc03g093130.3	92	37	43	670	232	457	294.8279	54.6724	534.983491	3.289745888	1.53E-07	8.37E-06	up	--
novel.849	13	16	20	288	105	84	106.4968	15.3736	197.62005	3.678399222	1.56E-07	8.51E-06	up	--
Solyc11g071750.2	2	1	1	65	24	32	24.95044	1.27232	48.6285627	5.250212952	1.57E-07	8.54E-06	up	Calmodulin and related proteins (EF-Hand superfamily)
Solyc03g026230.1	18	13	21	71	54	82	45.99376	16.2969	75.6906609	2.199810532	1.66E-07	8.96E-06	up	Uncharacterized membrane protein
Solyc11g073075.1	176	89	74	1246	449	812	548.4749	108.183	988.767139	3.191823156	1.67E-07	9.03E-06	up	FOG: Zn-finger
novel.1934	27	55	59	2	8	7	24.87539	44.2502	5.50057049	-2.947678248	1.92E-07	1.02E-05	down	--
Solyc09g089540.4	76	41	90	412	320	231	215.9747	64.6122	367.3372	2.503122422	2.08E-07	1.09E-05	up	--
Solyc06g066530.1	35	31	26	1	1	1	15.15678	29.2221	1.09148789	-4.808125645	2.24E-07	1.16E-05	down	--
Solyc04g005040.1	309	207	189	1494	751	1073	751.8541	224.051	1279.65743	2.513243368	2.26E-07	1.17E-05	up	Gelatinase A and related matrix metalloproteases
novel.2071	31	34	37	0	1	0	16.22445	32.1475	0.30144662	-6.400185559	2.28E-07	1.18E-05	down	FOG: Transposon-encoded proteins with TYA
Solyc03g083730.1	135	84	99	761	325	530	365.0321	100.585	629.479688	2.644220577	3.18E-07	1.60E-05	up	--
novel.1564	0	0	0	41	25	11	15.43718	0	30.8743677	7.455008777	3.62E-07	1.79E-05	up	--
Solyc03g026270.3	57	16	13	364	176	267	169.4679	27.5791	311.356678	3.497260946	4.13E-07	2.00E-05	up	--
Solyc10g009365.1	19	18	21	82	62	72	49.36402	18.2728	80.4552449	2.122969772	4.14E-07	2.00E-05	up	--
Solyc03g116890.3	301	169	176	1940	748	1112	856.6858	205.086	1508.28582	2.878114722	4.31E-07	2.07E-05	up	--
Solyc08g077020.1	65	48	45	371	161	268	180.3485	50.1425	310.55457	2.628127917	4.33E-07	2.08E-05	up	--
novel.821	0	1	0	16	41	27	14.32497	0.33036	28.3195847	6.392104612	4.59E-07	2.20E-05	up	--
Solyc03g026280.3	553	346	269	1357	1086	1754	945.9096	372.554	1519.26489	2.027725842	4.81E-07	2.29E-05	up	--
Solyc11g018774.1	32	49	31	284	125	193	135.1275	35.6648	234.590296	2.714876111	6.03E-07	2.80E-05	up	--
novel.842	36	61	63	9	12	13	31.1296	50.3243	11.9348629	-2.089951658	6.09E-07	2.82E-05	down	--
Solyc08g068770.3	56	39	41	310	150	195	149.251	43.0772	255.424743	2.564127022	6.58E-07	3.03E-05	up	Diamine acetyltransferase
Solyc12g005450.1	23	8	18	258	69	130	100.6934	15.3845	186.002242	3.59055285	6.67E-07	3.06E-05	up	Serine/threonine protein kinase
Solyc06g048700.1	11	9	10	49	42	41	29.21712	9.47573	48.9585164	2.349973063	6.86E-07	3.12E-05	up	--
Solyc04g056713.1	137	162	201	32	20	10	90.81526	156.959	24.6710271	-2.711640461	7.57E-07	3.41E-05	down	Aldehyde dehydrogenase
Solyc10g050960.3	11	5	4	80	38	52	36.30019	6.39202	66.2083495	3.368094604	7.89E-07	3.52E-05	up	--
Solyc12g098590.3	54	60	38	9	12	10	29.7575	48.4855	11.0295139	-2.157600309	8.76E-07	3.85E-05	down	UDP-glucuronosyl and UDP-glucosyl transferase
Solyc07g056000.2	905	547	595	4015	1961	2850	2030.187	648.798	3411.57534	2.394321097	8.88E-07	3.89E-05	up	--
Solyc08g007830.1	39	29	16	531	102	237	194.228	26.9207	361.53527	3.747057728	9.23E-07	4.00E-05	up	--
novel.439	556	276	241	2035	1162	1824	1118.258	342.177	1894.33875	2.468662819	9.28E-07	4.02E-05	up	AAA+-type ATPase
Solyc06g060690.3	240	130	158	728	516	605	430.3602	167.143	693.577194	2.051536637	9.39E-07	4.06E-05	up	Serine/threonine protein kinase
Solyc09g089500.3	4	3	7	22	38	24	16.8915	4.34355	29.4394455	2.739383837	1.13E-06	4.75E-05	up	--
Solyc01g057770.3	821	513	516	2968	1728	2284	1623.23	587.137	2659.32269	2.178944961	1.15E-06	4.86E-05	up	Na+-independent Cl/HCO3 exchanger AE1
Solyc02g091380.1	11	16	23	71	59	62	43.38437	15.6065	71.162234	2.169812245	1.32E-06	5.53E-05	up	--
novel.219	40	18	28	2	1	1	14.35756	27.1354	1.57974617	-4.261672753	1.37E-06	5.67E-05	down	--
Solyc06g076080.3	65	31	26	498	157	260	203.9446	38.9458	368.94332	3.242833783	1.45E-06	5.94E-05	up	--
novel.803	42	67	43	4	15	8	28.63305	48.3771	8.88899635	-2.402718394	1.49E-06	6.05E-05	down	--
novel.248	751	215	194	2575	2003	2727	1527.725	371.425	2684.02488	2.853300781	1.51E-06	6.12E-05	up	FOG: Transposon-encoded proteins with TYA
Solyc03g093110.3	113	36	56	411	237	407	229.9548	64.9668	394.942681	2.602597653	1.66E-06	6.68E-05	up	--
Solyc08g068600.3	151	63	66	374	341	375	243.8555	89.1405	398.570517	2.159854144	1.75E-06	7.02E-05	up	Glutamate decarboxylase and related proteins
Solyc08g016150.1	142	121	132	631	341	443	334.6717	124.769	544.574137	2.123546601	1.84E-06	7.35E-05	up	--
Solyc05g007210.2	58	57	34	2	8	13	27.46367	47.6161	7.31126851	-2.634189515	1.93E-06	7.69E-05	down	--
Solyc08g007820.1	181	113	101	905	371	642	436.5587	125.662	747.45512	2.571534752	2.03E-06	8.01E-05	up	--
Solyc10g084020.1	117	50	66	349	240	324	207.1763	73.8255	340.527018	2.20348529	2.06E-06	8.10E-05	up	--
Solyc02g077370.1	25	31	42	403	117	157	155.0487	30.6802	279.41727	3.182706895	2.07E-06	8.14E-05	up	--
Solyc10g086280.2	1077	759	761	4805	2380	3112	2413.007	823.341	4002.67266	2.281147043	2.11E-06	8.26E-05	up	Copper chaperone
Solyc10g005400.3	47	40	35	3	10	12	23.41443	38.7282	8.10063701	-2.193379829	2.23E-06	8.66E-05	down	Aldehyde reductase
novel.437	1	1	2	11	22	32	11.45081	1.24191	21.6597226	4.121397967	2.24E-06	8.68E-05	up	--
Solyc03g124110.2	456	211	212	1316	857	1356	794.9396	279.774	1310.10539	2.227013153	2.43E-06	9.31E-05	up	--
Solyc02g086210.4	39	44	55	246	147	143	125.455	43.3309	207.579157	2.253442745	2.61E-06	9.91E-05	up	Serine/threonine protein kinase
novel.1047	37	38	54	2	12	12	24.31099	40.4067	8.21527197	-2.217891227	2.71E-06	1.02E-04	down	FOG: Transposon-encoded proteins with TYA

Solyc01g090890.3	43	26	30	3	7	6	18.36184	31.3381	5.38559915	-2.512139571	2.76E-06	1.04E-04	down	Protein involved in vacuolar polyphosphate accumulation
novel.9	26	18	35	0	2	4	13.23181	24.6536	1.81002524	-3.609872695	2.80E-06	1.05E-04	down	FOG: Transposon-encoded proteins with TYA
Solyc01g096510.2	122	83	106	488	265	359	262.295	98.0965	426.493489	2.117341988	3.03E-06	1.12E-04	up	--
Solyc06g075690.3	241	174	179	1697	557	854	721.187	188.171	1254.20274	2.735932718	3.36E-06	1.22E-04	up	--
novel.865	33	43	34	8	5	5	20.90504	34.8879	6.92221429	-2.431089586	3.48E-06	1.25E-04	down	FOG: Transposon-encoded proteins with TYA
novel.1299	112	108	111	541	282	358	280.8885	104.583	457.193987	2.125451394	3.86E-06	1.38E-04	up	--
Solyc09g007900.4	810	655	667	3307	1841	2116	1741.52	674.833	2808.20617	2.056615946	4.30E-06	1.51E-04	up	Phenylalanine and histidine ammonia-lyase
Solyc10g011910.4	128	55	70	515	231	434	266.1393	80.2175	452.061003	2.493049482	5.19E-06	1.78E-04	up	--
Solyc01g079660.2	243	116	110	861	434	774	467.0953	149.392	784.79825	2.392598667	5.44E-06	1.87E-04	up	--
Solyc02g090770.1	55	13	28	430	119	226	172.1858	30.3454	314.026164	3.369324712	5.66E-06	1.93E-04	up	--
Solyc05g026595.1	39	42	50	6	5	16	25.23345	41.2016	9.26531077	-2.147152442	6.26E-06	2.12E-04	down	4-hydroxyphenylpyruvate dioxygenase
Solyc07g042230.1	10	10	12	215	31	77	73.81353	10.0694	137.557665	3.766075177	6.81E-06	2.26E-04	up	--
Solyc05g015890.4	36	41	23	0	4	7	17.64349	31.9687	3.31826748	-3.135246127	7.17E-06	2.37E-04	down	--
Solyc10g050970.1	447	261	263	1872	792	1331	931.3962	308.354	1554.43839	2.333220783	9.91E-06	3.13E-04	up	--
Solyc04g063370.3	25	21	22	2	4	0	11.84233	21.5023	2.18230301	-3.358523161	1.06E-05	3.33E-04	down	Serine/threonine protein kinase
Solyc01g108240.3	202	58	50	1030	381	777	475.7811	99.3197	852.242576	3.100995914	1.11E-05	3.46E-04	up	--
Solyc07g054720.2	41	29	54	2	13	9	23.17065	38.7299	7.61136958	-2.276053405	1.12E-05	3.48E-04	down	--
Solyc03g082530.1	49	57	50	381	143	190	167.9352	49.3983	286.472039	2.532515146	1.15E-05	3.56E-04	up	--
Solyc03g111993.1	26	32	40	7	4	2	17.9872	30.7472	5.22716039	-2.694512	1.25E-05	3.83E-04	down	Cytochrome P450 CYP2 subfamily
Solyc12g041940.1	496	534	517	31	174	177	305.0154	489.028	121.003309	-2.006049075	1.26E-05	3.85E-04	down	Hydroxyindole-O-methyltransferase
Solyc06g035700.1	164	83	74	694	298	473	336.8684	102.311	571.425694	2.480595119	1.40E-05	4.23E-04	up	--
Solyc07g054790.1	26	17	28	286	75	99	107.1971	22.2673	192.12688	3.103538316	1.47E-05	4.44E-04	up	--
Solyc02g094000.1	287	112	141	1397	505	853	631.5928	171.437	1091.74825	2.670280666	1.54E-05	4.62E-04	up	--
novel.1379	11	16	5	68	41	56	36.39023	10.3197	62.4607221	2.596018605	1.66E-05	4.92E-04	up	--
novel.1535	427	360	462	22	125	148	243.056	393.026	93.0863934	-2.067302191	1.84E-05	5.37E-04	down	FOG: Transposon-encoded proteins with TYA
Solyc03g025710.3	78	34	77	11	6	2	33.45637	59.1297	7.78308672	-3.015004117	2.14E-05	6.13E-04	down	--
Solyc03g097580.4	30	45	22	5	6	4	18.25441	31.0517	5.45710308	-2.55046242	2.15E-05	6.14E-04	down	Multitransmembrane protein
Solyc03g082370.1	400	308	307	2403	804	1184	1047.265	321.57	1772.95879	2.462416379	2.35E-05	6.61E-04	up	--
Solyc04g007580.1	399	216	175	1480	665	1113	755.526	252.083	1258.96873	2.319837695	2.43E-05	6.82E-04	up	--
Solyc09g015700.4	117	104	71	465	246	310	243.9412	93.1337	394.748696	2.081611869	2.61E-05	7.25E-04	up	--
Solyc08g068680.4	44	28	20	216	101	111	99.39682	29.3858	169.407809	2.523884783	2.80E-05	7.71E-04	up	Glutamate decarboxylase and related proteins
Solyc06g076020.3	2430	1239	842	10262	4340	6584	4874.985	1444.25	8305.72401	2.523738693	2.81E-05	7.74E-04	up	Molecular chaperones HSP70/HSC70
Solyc02g083880.3	47	29	35	279	103	132	121.1013	35.0942	207.108416	2.556542461	2.83E-05	7.77E-04	up	--
novel.1306	7	2	2	124	13	38	39.7238	3.51702	75.9305861	4.430583071	3.01E-05	8.17E-04	up	--
Solyc12g042090.1	34	19	39	2	9	4	16.82423	28.7518	4.8966681	-2.50267041	3.19E-05	8.58E-04	down	--
novel.1716	46	22	51	7	9	10	23.15277	37.1569	9.14865749	-2.038980032	4.68E-05	1.22E-03	down	--
novel.1325	9	6	4	25	35	21	17.58433	6.07414	29.0945315	2.256874255	5.56E-05	1.39E-03	up	--
novel.720	36	11	59	4	3	2	18.04612	32.6313	3.46093895	-3.322900222	5.89E-05	1.45E-03	down	FOG: Transposon-encoded proteins with TYA
Solyc10g008160.3	74	33	39	5	12	15	28.46363	46.3419	10.5853958	-2.09950211	6.27E-05	1.52E-03	down	--
Solyc01g111250.3	12	15	23	20	111	89	42.84234	15.6003	70.0844271	2.175240611	6.42E-05	1.55E-03	up	Glycosylphosphatidylinositol-specific phospholipase C
Solyc09g082870.3	110	71	38	287	185	311	180.0114	70.2705	289.752262	2.043255328	7.16E-05	1.69E-03	up	Calcium transporting ATPase
Solyc01g091590.3	375	222	143	1494	631	921	717.2503	236.888	1197.61282	2.337472678	7.82E-05	1.81E-03	up	--
Solyc10g008350.4	584	350	413	2164	1021	1247	1083.455	426.218	1740.6913	2.029385481	8.02E-05	1.86E-03	up	--
Solyc12g150114.1	5	5	4	9	28	34	13.77136	4.44728	23.0954518	2.408343844	8.20E-05	1.89E-03	up	K+-channel ERG and related proteins
Solyc01g007020.4	24	9	11	67	40	71	40.09041	13.9831	66.1977622	2.236548615	8.32E-05	1.91E-03	up	--
Solyc10g055560.2	10	13	7	42	57	24	27.26202	9.59194	44.9320967	2.223961004	8.51E-05	1.95E-03	up	Predicted E3 ubiquitin ligase
Solyc02g068670.3	12	3	11	99	30	43	39.23451	8.11138	70.3576368	3.1031561	9.48E-05	2.12E-03	up	FOG: Ankyrin repeat
novel.254	8	10	8	24	54	22	21.44092	8.24631	34.6355419	2.073849036	1.02E-04	2.27E-03	up	--
Solyc07g054780.1	220	155	232	1634	432	576	646.2601	190.654	1101.86597	2.529937181	1.51E-04	3.14E-03	up	--
Solyc08g036620.4	13	3	6	164	25	30	51.81549	6.96696	96.6640125	3.789131105	1.60E-04	3.30E-03	up	--
Solyc08g007805.1	28	42	32	209	71	118	95.70427	32.3495	159.059083	2.29250657	1.61E-04	3.31E-03	up	NADH:ubiquinone oxidoreductase
novel.2059	55	57	35	231	103	154	118.6243	46.9374	190.311245	2.0158268	2.06E-04	4.05E-03	up	--
Solyc06g054630.3	8	1	6	53	18	32	22.8232	4.68561	40.9607837	3.109377689	2.13E-04	4.18E-03	up	--

Solyc09g010980.1	45	16	19	201	63	117	88.94577	25.4519	152.439661	2.578740595	2.18E-04	4.27E-03	up	--
novel.669	132	73	133	2	22	38	62.52018	105.964	19.0760962	-2.440828722	2.20E-04	4.30E-03	down	FOG: Transposon-encoded proteins with TYA
Solyc09g072630.3	23	13	25	2	5	4	11.3916	19.0923	3.69088164	-2.342513498	2.29E-04	4.45E-03	down	FOG: Leucine rich repeat
Solyc07g055630.4	23	4	7	51	52	52	33.55069	10.8323	56.2691121	2.374907542	2.39E-04	4.63E-03	up	--
Solyc07g064410.1	27	23	10	0	4	1	10.39719	19.2868	1.50756947	-3.532574769	2.41E-04	4.66E-03	down	Ubiquitin-conjugating enzyme
Solyc01g095140.4	644	407	500	2767	1009	1391	1282.5	490.049	2074.95044	2.081571478	2.58E-04	4.91E-03	up	--
Solyc08g075480.4	2861	2778	2749	122	893	1070	1652.072	2652.48	651.66715	-2.023799851	2.89E-04	5.41E-03	down	Beta, beta-carotene 15,15'-dioxygenase
Solyc04g079450.4	26	14	26	173	41	85	71.58415	20.6888	122.479548	2.557858487	3.27E-04	5.99E-03	up	Serpin
Solyc04g009440.3	174	162	142	913	287	451	410.0112	151.623	668.399117	2.138835902	3.33E-04	6.08E-03	up	--
novel.1693	243	247	328	1526	541	643	679.4549	256.699	1102.21122	2.101143066	3.49E-04	6.34E-03	up	--
Solyc08g080190.4	1808	429	499	90	282	218	534.5217	874.304	194.739885	-2.164855097	3.68E-04	6.62E-03	down	Glucose dehydrogenase
Solyc07g053230.3	95	57	58	483	149	190	202.3704	66.6577	338.083063	2.340125137	3.92E-04	6.96E-03	up	Transcription factor, Myb superfamily
novel.2031	19	24	36	3	9	1	14.5701	24.6606	4.47957737	-2.460335711	4.31E-04	7.55E-03	down	--
Solyc03g098735.1	8	6	3	46	14	32	20.89675	5.45631	36.3371893	2.726615484	4.54E-04	7.92E-03	up	--
Solyc10g005320.3	7	4	17	55	38	27	27.52035	8.58338	46.4573176	2.410692829	4.63E-04	8.04E-03	up	Tryptophan synthase beta chain
Solyc10g005480.3	149	86	116	604	226	301	282.3238	110.776	453.871617	2.032348003	4.82E-04	8.32E-03	up	--
novel.1965	66	21	30	2	12	2	21.16928	37.1411	5.19744194	-2.799514018	4.83E-04	8.33E-03	down	--
Solyc08g036505.2	15	22	30	2	2	8	12.46741	20.9411	3.9936738	-2.357883324	4.97E-04	8.52E-03	down	--
Solyc08g068610.3	10	2	10	48	25	29	23.28166	6.83906	39.7242696	2.515438653	4.99E-04	8.55E-03	up	Glutamate decarboxylase and related proteins
Solyc12g007045.1	55	45	76	316	145	115	143.8598	55.0151	232.704419	2.075317833	5.54E-04	9.33E-03	up	--
novel.1171	33	31	28	186	63	80	81.55553	29.1612	133.949816	2.192922583	5.63E-04	9.43E-03	up	--
Solyc07g048060.3	21	10	7	90	38	41	39.96877	12.1662	67.7713192	2.470851497	5.72E-04	9.55E-03	up	Predicted membrane protein
Solyc10g047767.1	23	14	23	1	4	7	11.32089	18.8353	3.80652575	-2.222234075	5.74E-04	9.56E-03	down	FOG: Transposon-encoded proteins with TYA
Solyc12g010710.1	4	7	11	40	33	17	20.72411	6.83984	34.6083803	2.312478199	6.12E-04	1.01E-02	up	--
Solyc08g080540.3	175	129	124	1209	281	317	453.2173	135.759	770.675965	2.504055155	6.61E-04	1.08E-02	up	Heat shock transcription factor
Solyc08g036660.4	10	10	4	94	46	13	35.70286	7.71972	63.6860012	3.040427272	6.72E-04	1.09E-02	up	--
Solyc10g085380.2	12	13	17	91	42	29	39.51062	13.1773	65.8439679	2.305972193	7.12E-04	1.14E-02	up	Reductases with broad range of substrate
novel.1563	87	23	31	10	6	5	26.55117	44.9022	8.20017745	-2.50900254	7.43E-04	1.18E-02	down	Tam3-transposase (Ac family)
Solyc01g007030.3	25	7	1	144	42	79	58.76008	10.7094	106.810807	3.319122239	7.55E-04	1.20E-02	up	--
novel.754	40	67	76	19	6	9	35.61144	57.4212	13.8016339	-2.120405162	7.80E-04	1.23E-02	down	--
Solyc09g010570.1	32	41	27	0	8	12	18.94001	31.847	6.03296896	-2.306801897	7.90E-04	1.24E-02	down	--
Solyc01g099870.3	75	44	46	3	16	0	29.32193	52.3559	6.28792067	-3.039404885	8.83E-04	1.36E-02	down	Multitransmembrane protein
Solyc02g091990.3	48	49	17	175	72	152	94.87961	36.7389	153.020371	2.056701334	1.02E-03	1.53E-02	up	1-aminocyclopropane-1-carboxylate synthase
Solyc03g034410.1	2	2	5	4	24	31	10.66027	2.77752	18.543025	2.748720245	1.09E-03	1.60E-02	up	--
Solyc02g090800.1	3	7	3	49	8	26	19.17432	4.16605	34.1825864	3.027412637	1.18E-03	1.72E-02	up	--
novel.1229	5	9	26	100	35	43	42.29173	12.2303	72.3531281	2.548768834	1.36E-03	1.92E-02	up	--
Solyc01g087590.3	144	104	89	735	190	258	300.5883	107.172	494.004703	2.203015076	1.43E-03	2.00E-02	up	Amine oxidase
Solyc03g111290.2	33	22	21	223	65	42	82.64125	24.132	141.150511	2.543367184	1.45E-03	2.01E-02	up	Cytochrome P450 CYP4/CYP19/CYP26
novel.1836	32	25	39	172	54	78	76.94167	30.0857	123.797615	2.032142913	1.61E-03	2.19E-02	up	FOG: RRM domain
Solyc02g063527.1	4	0	1	220	2	50	62.34953	1.59021	123.108864	6.27532867	1.64E-03	2.22E-02	up	Squalene monooxygenase
Solyc09g007650.3	132	61	129	4	49	25	62.54677	100.825	24.2684923	-2.029781291	1.66E-03	2.24E-02	down	--
Solyc03g025570.2	22	31	24	0	9	9	14.92505	24.421	5.42906656	-2.06387727	1.92E-03	2.52E-02	down	RNA polymerase, 25-kDa subunit
Solyc07g056190.3	16	10	15	74	23	41	34.16637	12.8953	55.4374876	2.087987752	2.05E-03	2.65E-02	up	--
Solyc03g104790.3	13	19	29	103	35	54	48.0728	19.0081	77.137516	2.007063029	2.34E-03	2.96E-02	up	Cdc2-related protein kinase
novel.1845	13	6	15	57	21	34	27.51158	10.6014	44.4217227	2.045649515	2.77E-03	3.38E-02	up	--
Solyc05g023841.1	23	23	45	196	52	60	78.87548	28.2701	129.480826	2.187147369	3.29E-03	3.92E-02	up	--
Solyc10g084430.2	27	29	20	7	3	0	14.26412	24.2061	4.32214777	-2.614378501	3.66E-03	4.26E-02	down	FOG: Predicted E3 ubiquitin ligase
Solyc01g073670.3	21	21	11	11	118	89	42.38764	16.9751	67.8002289	2.010097673	4.13E-03	4.65E-02	up	Predicted sugar transporter

**Index**	***yfm* -45 DPA-1**	***yfm* -45 DPA-2**	***yfm* -45 DPA-3**	**OH 88119-45 DPA-1**	**OH 88119-45 DPA-2**	**OH 88119-45 DPA-3**	**baseMean**	***yfm* -45 DPA**	**45 DPA OH 88119-45 DPA**	**log2FoldChange**	**pvalue**	**padj**	**regulated**	**Annotation**
Solyc02g089610.2	1283	855	1125	47176	39842	54726	30446.65	836.513	60056.7824	6.16566854	0	0	up	S-adenosylmethionine decarboxylase
Solyc02g092800.3	3047	2894	3020	15804	13822	18274	11315.89	2302.11	20329.6807	3.142594461	0	0	up	Mn2+ and Fe2+ transporters of the NRAMP family
Solyc06g074710.1	350	362	381	12609	11546	13907	8254.386	280.703	16228.0688	5.853308662	0	0	up	--

Solyc07g043130.3	1681	1894	2112	34053	31025	48627	24621.2	1458.99	47783.4088	5.033447477	0	0	up	--
Solyc07g053140.3	269	273	241	6313	5576	9137	4509.333	201.587	8817.07872	5.451866694	0	0	up	--
Solyc10g005080.3	90	84	85	9914	10025	17878	7909.589	66.569	15752.6081	7.887113794	0	0	up	Zuotin and related molecular chaperones (DnaJ superfamily)
Solyc03g118420.4	1265	1097	1164	16064	12189	16257	9907.905	905.84	18909.9697	4.383791249	5.59E-281	1.57E-277	up	Transcription factor NF-X1
Solyc02g150147.1	396	273	339	16274	13223	14288	9524.247	258.585	18789.908	6.182949436	5.93E-269	1.33E-265	up	--
Solyc11g010380.2	1554	1532	1243	26373	27023	35369	19376.08	1115.8	37636.3643	5.076218914	6.09E-269	1.33E-265	up	Uncharacterized membrane protein
Solyc11g011170.2	894	917	1165	9660	8961	12090	6887.185	762.202	13012.1689	4.093204639	7.84E-266	1.54E-262	up	--
Solyc07g019460.3	3526	2995	3414	20565	19879	26679	15493.17	2549.96	28436.3785	3.479171627	7.43E-261	1.33E-257	up	NADP/FAD dependent oxidoreductase
Solyc11g008010.2	3199	3545	3897	25704	22328	28475	17633.32	2730.62	32536.0153	3.574690634	8.57E-257	1.40E-253	up	--
Solyc11g012360.2	124	201	135	8525	7196	9655	5442.123	118.806	10765.4404	6.503513702	7.23E-253	1.09E-249	up	Na+/dicarboxylate, Na+/tricarboxylate
Solyc03g116530.3	434	360	420	5424	5083	5585	3602.189	311.512	6892.86642	4.467344986	1.78E-249	2.50E-246	up	--
Solyc05g007880.4	87	86	112	3468	2353	3769	2056.868	72.9745	4040.76241	5.789702211	4.67E-249	6.12E-246	up	--
Solyc07g150145.1	1720	1731	1922	8100	7023	9279	5868.436	1378.92	10357.9497	2.909024666	7.78E-249	9.57E-246	up	--
Solyc03g119300.4	194	173	212	1780	1722	2302	1303.896	148.435	2459.35665	4.049524564	2.84E-237	3.29E-234	up	Zn-finger protein
Solyc03g025720.3	4263	4682	4454	53634	52164	53865	36019.07	3445.59	68592.546	4.315239257	4.70E-226	5.13E-223	up	Acyl-CoA synthetase
Solyc06g075530.1	113	81	88	1757	1688	2340	1259.49	72.4612	2446.51927	5.077960379	1.16E-225	1.20E-222	up	--
Solyc02g036370.3	1035	954	1306	14699	10923	15602	9149.251	843.257	17455.2445	4.371258411	1.40E-219	1.38E-216	up	Zuotin and related molecular chaperones (DnaJ superfamily)
Solyc11g013810.3	1151	906	1070	15549	16104	16292	10707.04	802.379	20611.7039	4.682898475	8.09E-218	7.58E-215	up	Sulfite oxidase, molybdopterin-binding component
Solyc02g063000.4	28	25	18	3788	2818	5832	2589.079	18.3291	5159.82928	8.14202255	1.28E-216	1.15E-213	up	UDP-glucuronosyl and UDP-glucosyl transferase
Solyc03g093140.3	572	460	757	27204	30665	27425	18671.34	456.822	36885.8612	6.334817609	5.89E-215	5.04E-212	up	Permease of the major facilitator superfamily
Solyc04g082480.3	226	205	216	1824	1492	2041	1218.625	166.213	2271.03724	3.772558338	2.26E-214	1.85E-211	up	--
Solyc08g007130.4	62	57	51	1140	1126	1466	813.5741	43.7672	1583.38107	5.17930015	6.97E-213	5.49E-210	up	--
Solyc03g119530.3	3431	3956	4080	71	76	88	1522.669	2945.06	100.281389	-4.881224944	6.42E-211	4.85E-208	down	--
Solyc04g054740.3	182	118	195	8263	7504	7028	4985.347	126.511	9844.18313	6.280638295	1.20E-197	8.73E-195	up	Myo-inositol-1-phosphate synthase
Solyc11g073120.2	1045	1130	931	9579	9972	12482	7207.108	800.307	13613.9096	4.088700241	1.18E-195	8.26E-193	up	Transcription factor, Myb superfamily
Solyc02g091930.3	1350	1362	1455	4622	4125	5691	3591.146	1070	6112.29091	2.514200738	6.53E-191	4.43E-188	up	Transcription factor HEX, contains HOX and HALZ domains
Solyc02g067230.3	86	98	101	1492	1409	2270	1120.943	73.1989	2168.68637	4.889624724	3.81E-190	2.50E-187	up	--
Solyc07g063590.4	61	63	71	999	1061	1309	741.3741	50.0304	1432.71772	4.83883122	2.97E-186	1.89E-183	up	Myosin class V heavy chain
Solyc06g069420.3	249	298	301	3708	2849	5131	2551.198	217.84	4884.55677	4.487354528	2.15E-177	1.32E-174	up	--
Solyc01g104740.3	384	330	240	7915	7907	8919	5412.172	246.261	10578.0826	5.425526801	3.12E-176	1.86E-173	up	Transcription factor MBF1
Solyc12g038910.1	27	23	27	1341	1034	1915	904.84	19.7549	1789.92523	6.501578168	3.84E-171	2.22E-168	up	--
Solyc05g010516.1	42	50	77	1328	1353	1679	948.6755	43.1334	1854.21753	5.42145406	1.52E-169	8.57E-167	up	--
Solyc02g088910.3	438	474	509	3329	2695	4466	2382.98	364.793	4401.16685	3.593085315	2.13E-169	1.16E-166	up	--
Solyc07g005760.3	4117	4632	4905	23657	21576	25333	16816.83	3505.59	30128.067	3.103333762	1.23E-167	6.52E-165	up	--
Solyc04g071780.3	121	139	189	3225	3727	5643	2702	114.836	5289.16311	5.524348128	1.49E-167	7.69E-165	up	Cytochrome P450 CYP2 subfamily
Solyc03g044300.3	3576	4140	3652	25959	26820	38195	20655.96	2926.11	38385.799	3.71360067	2.38E-167	1.20E-164	up	--
Solyc06g073180.3	1144	1297	1300	5276	4933	6844	4087.395	961.231	7213.55944	2.907923815	4.11E-166	2.02E-163	up	--
Solyc07g006370.1	218	265	152	7101	7913	8600	5136.541	164.342	10108.7391	5.944158841	1.86E-164	8.92E-162	up	K+-dependent Na+:Ca2+ antiporter
Solyc03g122300.3	2867	2812	3373	16895	19328	24143	13979.15	2320.66	25637.645	3.465584444	1.49E-156	6.96E-154	up	--
Solyc03g098320.4	319	337	425	4261	4010	7026	3330.747	276.867	6384.62667	4.527108805	1.70E-156	7.76E-154	up	Zuotin and related molecular chaperones (DnaJ superfamily)
Solyc11g012850.2	276	325	363	2514	2546	3743	1977.832	247.279	3708.38449	3.90653507	8.44E-156	3.78E-153	up	--
Solyc08g081190.3	17316	16999	16482	58398	48948	70694	44149.16	13062.2	75236.1651	2.526064079	1.21E-155	5.30E-153	up	Aquaporin (major intrinsic protein family)
Solyc01g109780.3	1216	1436	1547	7140	6023	8590	5137.856	1077.67	9198.03908	3.093426311	1.65E-151	7.05E-149	up	--
Solyc02g094030.3	1265	1048	1267	5947	5684	8500	4697.927	918.172	8477.68259	3.206836171	2.50E-151	1.05E-148	up	--

Solyc07g045190.3	773	600	671	4580	4191	6497	3472.393	524.895	6419.89095	3.612628313	3.14E-150	1.29E-147	up	FOG: Predicted E3 ubiquitin ligase
Solyc04g074180.4	1257	1239	1488	5763	6056	7248	4572.108	1021.34	8122.87327	2.99122903	3.79E-147	1.52E-144	up	Deoxyribodipyrimidine photolyase/cryptochrome
Solyc01g086870.3	2318	2246	2427	9597	8003	12987	7319.049	1795.04	12843.0592	2.839061109	1.28E-146	5.05E-144	up	--
Solyc03g115720.3	1061	1249	1477	7570	7177	9648	5653.234	970.578	10335.8913	3.412525564	1.41E-146	5.45E-144	up	Predicted E3 ubiquitin ligase
Solyc01g096620.4	103	115	118	1123	889	1490	777.5585	86.3072	1468.80982	4.090217171	6.76E-146	2.56E-143	up	--
Solyc05g012230.4	65	60	88	1452	1865	2565	1268.051	54.4542	2481.64723	5.508011127	1.88E-145	6.97E-143	up	--
Solyc01g150156.1	2359	1921	2574	49	53	66	913.3948	1755.39	71.3963327	-4.622018272	9.66E-145	3.52E-142	down	--
Solyc02g076700.1	80	65	74	974	778	963	606.7742	56.2177	1157.33071	4.362939035	6.89E-144	2.46E-141	up	FOG: PPR repeat
Solyc02g088560.4	1290	1305	1227	5582	5313	6173	4135.927	983.158	7288.69493	2.89018141	7.81E-143	2.74E-140	up	K+-channel ERG and related proteins
Solyc07g049550.3	1762	1486	1581	10148	7790	13267	7161.287	1240.63	13081.9451	3.398592876	6.73E-140	2.32E-137	up	Iron/ascorbate family oxidoreductases
Solyc04g071770.3	468	551	591	3863	2932	4896	2660.27	413.236	4907.30309	3.570114835	1.29E-139	4.36E-137	up	--
Solyc03g097980.4	305	290	317	1287	1295	1696	1024.131	234.142	1814.1201	2.953694315	1.70E-138	5.66E-136	up	G-protein alpha subunit (small G protein superfamily)
Solyc07g007560.3	465	574	559	2711	2651	3499	2083.838	410.697	3756.97859	3.193624153	3.25E-138	1.07E-135	up	Permease of the major facilitator superfamily
Solyc07g008250.3	1884	1819	2111	10571	7941	13631	7482.374	1491.32	13473.4314	3.175533472	7.61E-138	2.45E-135	up	Leucine rich repeat proteins, some proteins contain F-box
Solyc08g081990.3	682	673	806	2649	2330	3379	2041.599	554.017	3529.18058	2.671252159	2.66E-137	8.44E-135	up	--
Solyc06g053840.3	2005	1495	1554	14083	11727	19158	10086.98	1299.25	18874.7043	3.860810705	2.29E-135	7.16E-133	up	--
Solyc09g090570.2	653	760	478	11244	9612	16315	8025.866	488.875	15562.8576	4.993052237	1.24E-133	3.81E-131	up	--
Solyc09g092490.3	185	299	343	4745	4471	5753	3284.874	211.889	6357.85877	4.906796533	3.18E-132	9.51E-130	up	UDP-glucuronosyl and UDP-glucosyl transferase
Solyc06g008870.2	1191	1331	1335	4235	3685	4915	3218.086	991.057	5445.11485	2.458011145	3.19E-132	9.51E-130	up	Arylacetamide deacetylase
Solyc09g098130.3	227	198	247	1468	1119	1531	959.6763	172.245	1747.10812	3.341715368	6.46E-130	1.90E-127	up	Apoptotic ATPase
Solyc07g064130.2	31633	30207	37779	164622	161565	173492	119873	25525.7	214220.342	3.06905796	5.26E-129	1.52E-126	up	Ubiquitin and ubiquitin-like proteins
Solyc04g051800.3	4629	4615	5539	15467	15443	19853	12669.97	3789.7	21550.2416	2.507474075	6.57E-128	1.87E-125	up	Predicted transporter (ABC superfamily)
Solyc02g078400.3	1441	1720	1901	8283	8727	10809	6563.656	1298.63	11828.6815	3.187133553	2.73E-125	7.67E-123	up	Dihydroorotase and related enzymes
Solyc08g075210.2	2345	2570	2302	9440	9376	13120	7675.806	1857.43	13494.1804	2.861125073	3.57E-124	9.88E-122	up	--
Solyc03g096670.3	3454	2812	3869	16986	14349	22525	12624.04	2594.71	22653.3827	3.126036138	8.73E-123	2.39E-120	up	Serine/threonine protein phosphatase
Solyc03g119140.4	941	883	1041	2678	2499	3465	2195.307	734.779	3655.83506	2.314733075	9.09E-121	2.45E-118	up	Tyrosine kinase specific for activated (GTP-bound) p21cdc42Hs
Solyc04g009910.3	362	417	353	2417	2774	3632	2014.267	291.543	3736.99054	3.680703157	1.56E-120	4.15E-118	up	Ca2+/calmodulin-dependent protein kinase
Solyc05g056170.3	46	53	91	1554	1190	1497	927.7322	48.4145	1807.04982	5.217950188	1.77E-120	4.65E-118	up	Phenylalanine and histidine ammonia-lyase
Solyc05g008200.4	4392	4126	5008	11918	10313	14229	9453.897	3467.5	15440.2913	2.154674778	4.32E-120	1.12E-117	up	Phosphatidylinositol-4-phosphate 5-kinase
Solyc01g099960.4	35	22	41	764	772	1275	600.621	25.0069	1176.23516	5.551499667	2.19E-119	5.58E-117	up	--
Solyc02g089400.4	704	753	895	2982	2989	3921	2398.731	602.912	4194.54977	2.798203036	6.30E-119	1.59E-116	up	Amino acid transporter protein
Solyc06g069760.3	138	102	124	846	804	1180	643.2567	93.383	1193.13037	3.675508692	3.33E-118	8.30E-116	up	--
Solyc12g056600.3	116	223	150	3565	4228	5174	2819.112	126.283	5511.9399	5.448973525	1.19E-116	2.93E-114	up	Reductases with broad range of substrate specificities
Solyc06g073320.3	16029	13927	16856	42465	36088	48832	33008.51	12004	54012.9924	2.16977077	1.78E-116	4.32E-114	up	Predicted hydrolase (HIT family)
Solyc10g078240.2	542	702	835	11789	7891	9349	6477.024	532.648	12421.4004	4.543318058	1.03E-111	2.47E-109	up	Cytochrome P450 CYP2 subfamily
Solyc06g053910.4	1267	1202	1429	3694	3233	4817	2975.664	999.556	4951.77083	2.308671283	3.89E-111	9.23E-109	up	--
Solyc05g007770.3	685	1043	627	12551	11946	19002	9431.997	609.138	18254.8562	4.905773017	2.03E-110	4.75E-108	up	--
Solyc06g060340.3	3288	3992	3876	13095	11385	15671	9936.048	2867.39	17004.7071	2.568180475	4.47E-110	1.04E-107	up	--
Solyc01g097340.3	1046	1140	1140	2913	2728	3625	2391.797	854.7	3928.89403	2.200764573	3.60E-109	8.24E-107	up	dTDP-glucose 4-6-dehydratase/UDP-glucuronic acid decarboxylase
Solyc07g064620.2	6128	5968	4237	49014	46981	70706	37195.57	4216.84	70174.2977	4.056774363	4.11E-109	9.29E-107	up	Translation initiation factor 1 (eIF-1/SUI1)
Solyc05g053210.4	2780	3548	4536	28363	30835	38809	22202.26	2780.37	41624.1551	3.904009206	8.84E-108	1.98E-105	up	Serine/threonine protein kinase
Solyc06g053260.1	4	8	2	1923	2986	4076	1895.011	3.65582	3786.36664	10.03657451	1.66E-106	3.66E-104	up	--
Solyc09g074050.3	92	93	112	958	1145	1100	727.9145	76.1305	1379.69855	4.177953287	2.07E-106	4.52E-104	up	Uncharacterized conserved protein

Solyc01g088810.4	7	12	14	671	494	833	423.5102	8.45167	838.568694	6.630640201	2.93E-106	6.33E-104	up	FOG: Ankyrin repeat
Solyc02g085600.1	687	772	1125	8333	8134	8682	5722.878	660.214	10785.5427	4.029617955	1.24E-105	2.64E-103	up	--
Solyc07g063880.4	26	14	27	491	476	720	363.4814	17.1019	709.86096	5.370242128	1.45E-105	3.06E-103	up	Beta-glucosidase, lactase phlorizinhydrolase
Solyc02g087740.3	2601	2944	3133	54	91	98	1165.829	2227.83	103.823902	-4.427915981	3.48E-105	7.28E-103	down	Uncharacterized conserved protein
Solyc01g086975.1	250	266	226	1853	1716	1745	1238.25	191.114	2285.38626	3.580164109	9.73E-103	2.01E-100	up	--
Solyc04g007220.4	865	843	851	3462	3459	5512	2935.864	657.677	5214.051	2.987324256	1.55E-102	3.17E-100	up	--
Solyc02g014430.3	73	69	77	539	555	623	395.173	56.2094	734.13672	3.705499213	1.65E-102	3.34E-100	up	Nucleoside transporter
Solyc10g005250.3	15	9	23	596	436	692	368.9832	11.9402	726.026064	5.91547404	6.70E-102	1.34E-99	up	Uncharacterized membrane protein
Solyc06g035960.3	668	455	726	6403	6418	6092	4315.632	472.698	8158.56621	4.108814955	2.31E-99	4.59E-97	up	Acyl-CoA synthetase
Solyc02g071510.3	1648	1360	1726	4543	4254	5514	3644.819	1213.32	6076.31415	2.323991425	5.69E-99	1.12E-96	up	Transcription initiation factor TFIID
Solyc09g008830.3	1197	1282	1512	12	14	32	523.4611	1023.17	23.7545205	-5.393622661	2.42E-98	4.71E-96	down	--
Solyc12g006260.1	2879	3227	2163	39	43	71	1100.054	2136.15	63.9548885	-5.054295706	4.35E-98	8.39E-96	down	--
Solyc06g082410.1	35	87	62	1514	1616	2368	1181.351	47.4708	2315.23189	5.610012354	6.99E-98	1.33E-95	up	--
Solyc09g063010.4	366	396	508	1980	2009	2340	1513.088	325.167	2701.0102	3.053344485	9.30E-98	1.76E-95	up	--
Solyc05g008105.1	3712	3962	3824	9137	8927	11228	7704.499	2956.31	12452.6834	2.074596036	1.11E-97	2.07E-95	up	--
Solyc10g083610.2	1564	1576	1816	4018	4013	5159	3435.352	1271.22	5599.48801	2.138898739	1.41E-97	2.61E-95	up	Tyrosine kinase specific for activated (GTP-bound) p21cdc42Hs
Solyc12g010540.1	1883	1289	1674	32	28	50	644.1713	1242.44	45.9066833	-4.746178669	2.78E-97	5.12E-95	down	UDP-glucose 4-epimerase/UDP-sulfoquinovose synthase
Solyc02g090890.4	1508	1472	1720	4037	3288	4598	3127.15	1205.41	5048.88633	2.06637526	3.28E-97	5.97E-95	up	Kynurenine 3-monooxygenase
Solyc06g035940.3	30	29	32	3857	3371	1709	1990.685	23.3587	3958.01082	7.404288848	8.91E-97	1.61E-94	up	--
Solyc10g083690.3	177	236	246	2033	2621	3900	1880.525	169.169	3591.88016	4.408304041	1.02E-96	1.83E-94	up	Cytochrome P450 CYP2 subfamily
Solyc04g005020.3	242	341	260	1949	2157	2745	1562.382	217.37	2907.39427	3.742333337	1.61E-96	2.86E-94	up	FOG: WD40 repeat
Solyc06g081980.1	3624	4534	3910	16249	14955	18298	12096.97	3106.89	21087.0447	2.762856445	1.78E-96	3.13E-94	up	Stationary phase-induced protein, SOR/SNZ family
Solyc02g081570.4	27	30	27	737	613	587	428.7991	21.6171	835.981086	5.273829636	3.98E-96	6.93E-94	up	--
Solyc05g012770.3	291	343	434	2210	2562	2882	1771.467	273.435	3269.49948	3.579071362	8.14E-96	1.40E-93	up	--
Solyc01g096630.3	130	164	141	2108	1455	3400	1492.184	111.994	2872.37361	4.681950885	1.36E-95	2.33E-93	up	--
Solyc01g103590.4	1192	1422	1480	61	48	79	565.0869	1051.24	78.9373707	-3.72795065	2.39E-95	4.06E-93	down	--
Solyc10g005960.1	665	549	604	2139	1827	2844	1666.05	466.881	2865.22005	2.617987334	3.82E-95	6.42E-93	up	--
Solyc04g049670.4	4987	4963	6048	380	269	317	2256.758	4100.27	413.248145	-3.312374147	7.75E-95	1.29E-92	down	GATA-4/5/6 transcription factors
Solyc01g110370.4	181	172	231	1619	1724	3072	1409.99	149.484	2670.49631	4.158750665	1.22E-94	2.01E-92	up	--
Solyc10g086420.1	997	950	1025	6837	6119	5709	4412.894	763.153	8062.63596	3.401003581	1.26E-94	2.06E-92	up	--
Solyc01g095470.3	1088	1349	1162	5848	6688	9196	5052.744	926.586	9178.90276	3.308526654	1.80E-94	2.93E-92	up	Uncharacterized enzymes related to aldose 1-epimerase
Solyc02g062790.3	15	9	13	471	397	483	292.8013	9.47735	576.125281	5.922037038	1.92E-94	3.09E-92	up	Serine/threonine protein kinase
Solyc03g093550.1	1781	1645	2366	79	68	108	794.1684	1481.18	107.156758	-3.784126605	2.16E-94	3.45E-92	down	--
Solyc03g095610.4	350	306	409	2123	1453	2292	1373.456	272.701	2474.21045	3.181257968	2.74E-94	4.34E-92	up	--
Solyc07g005390.3	473	469	507	3094	2775	5436	2531.492	372.027	4690.95598	3.65676517	3.34E-94	5.26E-92	up	Aldehyde dehydrogenase
Solyc02g079170.3	972	959	987	5342	5472	5095	3809.184	749.719	6868.64968	3.195379059	4.59E-94	7.16E-92	up	NADH-dehydrogenase (ubiquinone)
Solyc01g007100.3	329	265	340	1297	1338	1926	1081.348	239.366	1923.32895	3.00609893	1.06E-93	1.65E-91	up	--
Solyc11g066320.2	2435	2488	2632	313	258	382	1171.216	1940.18	402.249976	-2.267974852	1.18E-93	1.82E-91	down	Acetylglucosaminyltransferase EXT1/exostosin 1
Solyc02g076800.1	457	318	306	2601	2729	3438	2001.494	278.213	3724.77574	3.743188035	4.16E-93	6.35E-91	up	--
Solyc02g084420.3	87	125	102	1139	828	1580	779.8321	80.8867	1478.77743	4.194461304	1.62E-92	2.45E-90	up	--
Solyc09g091580.3	1413	1581	1601	5247	5398	8145	4541.416	1180.52	7902.31031	2.743024075	5.98E-92	8.97E-90	up	Predicted unusual protein kinase
Solyc03g096900.4	283	315	359	1982	1610	3060	1507.3	245.444	2769.15629	3.496355121	6.02E-92	8.97E-90	up	Predicted guanosine polyphosphate pyrophosphohydrolase/synthase
Solyc02g092150.3	194	282	371	3380	4392	5568	2935.686	216.592	5654.78065	4.705908875	1.12E-91	1.65E-89	up	--
Solyc11g005190.3	396	562	490	2637	2219	3289	1904.76	372.657	3436.86325	3.205697855	2.61E-91	3.81E-89	up	--

Solyc01g057770.3	5352	2979	5539	52	76	68	1813.119	3541.56	84.6831247	-5.393497187	2.82E-91	4.08E-89	down	Na+-independent Cl/HCO3 exchanger AE1
Solyc02g093710.1	341	317	310	1415	1621	1757	1150.324	248.916	2051.73153	3.042879985	3.99E-91	5.73E-89	up	--
Solyc06g068970.3	357	426	327	2009	2159	3052	1666.362	286.24	3046.48391	3.412738981	4.53E-91	6.46E-89	up	Predicted steroid reductase
Solyc03g115930.3	132	111	118	825	1079	1202	709.5849	92.747	1326.42286	3.837807609	1.31E-90	1.86E-88	up	--
Solyc10g078590.2	3091	3292	2995	8207	8167	10771	6960.498	2413.21	11507.7812	2.253685848	3.06E-90	4.30E-88	up	--
Solyc10g084370.3	20	23	30	545	537	1041	449.4791	18.6824	880.275755	5.556887421	1.02E-89	1.42E-87	up	Zuotin and related molecular chaperones (DnaJ superfamily)
Solyc07g005520.1	150	144	211	891	798	1135	661.285	129.088	1193.48164	3.207218124	1.22E-89	1.69E-87	up	SETA binding protein SB1 and related proteins
novel.883	869	804	855	26	26	37	343.445	649.323	37.5673453	-4.106880523	1.69E-89	2.33E-87	down	--
Solyc06g072330.3	146	118	103	764	724	904	556.0569	94.5375	1017.57623	3.429271421	2.15E-89	2.94E-87	up	Molecular chaperone (DnaJ superfamily)
novel.1218	1620	1234	1276	22	14	11	541.1023	1061.73	20.4762019	-5.722713597	7.59E-89	1.03E-86	down	--
Solyc08g083050.1	1081	971	1312	15	7	22	439.5798	861.16	18.0001296	-5.540515537	1.29E-88	1.74E-86	down	--
Solyc11g007110.2	1625	1442	1811	4030	3605	4643	3233.67	1250.12	5217.21918	2.060901851	1.47E-88	1.97E-86	up	mRNA-binding protein Encore
Solyc05g051240.1	2651	1827	2898	36	50	77	976.9965	1885.74	68.2517122	-4.782310207	4.65E-88	6.19E-86	down	Aspartyl protease
novel.1030	1308	1276	1486	109	102	113	591.3073	1043.89	138.724724	-2.918220307	1.81E-87	2.39E-85	down	FOG: Transposon-encoded proteins with TYA
Solyc10g085680.2	703	950	990	3905	3714	4787	2973.386	678.458	5268.31321	2.956954282	2.29E-87	3.00E-85	up	Protein kinase PCTAIRE and related kinases
Solyc06g059860.3	160	181	141	1164	1558	1813	1027.914	124.279	1931.54854	3.959066836	4.55E-87	5.93E-85	up	--
Solyc03g098010.3	13	14	14	1198	2776	1657	1247.826	10.5363	2485.11504	7.882013051	4.88E-87	6.32E-85	up	Purple (tartrate-resistant) acid phosphatase
novel.1231	1947	1631	1507	29	25	60	677.8095	1308.85	46.7715804	-4.783838027	4.93E-87	6.33E-85	down	--
Solyc03g097030.3	474	608	786	4677	3565	4885	3023.407	477.978	5568.83596	3.542034459	8.97E-87	1.15E-84	up	Acyl-CoA synthetase
Solyc11g011260.1	1051	1183	1124	2892	2479	3508	2309.409	863.492	3755.32658	2.12105668	3.44E-86	4.37E-84	up	--
Solyc11g006010.3	21	14	22	415	302	439	251.8623	14.5766	489.147958	5.064772679	1.39E-85	1.76E-83	up	Predicted hydrolase involved in interstrand cross-link repair
Solyc04g080790.3	258	395	439	2945	2744	3178	2033.896	279.961	3787.83059	3.757736349	2.57E-85	3.22E-83	up	Transcription factor MEIS1 and related HOX domain proteins
Solyc09g060110.4	58	62	73	469	382	488	309.6094	49.4807	569.738218	3.523665139	4.11E-85	5.12E-83	up	--
Solyc05g054860.1	1241	1079	1505	42	50	46	519.2026	978.825	59.5799117	-4.052025709	5.56E-85	6.87E-83	down	Serine/threonine protein kinase
novel.1302	1417	1491	1267	69	69	71	582.5712	1075.35	89.7893006	-3.590562519	6.77E-85	8.32E-83	down	--
Solyc05g008920.3	2142	2569	2491	7787	8651	10937	6736.202	1851.19	11621.2176	2.650263174	2.69E-84	3.27E-82	up	--
Solyc08g062220.3	408	238	235	3247	3671	4539	2547.941	226.765	4869.11598	4.424641174	4.36E-84	5.26E-82	up	UDP-glucuronosyl and UDP-glucosyl transferase
novel.1323	11	13	8	465	363	689	320.1082	8.27499	631.94137	6.264533797	5.81E-84	6.97E-82	up	--
Solyc04g074580.1	4623	4532	4134	383	416	522	1990.582	3420.03	561.136197	-2.608096024	8.18E-84	9.76E-82	down	Histones H3 and H4
Solyc04g078900.3	4481	3520	3268	24800	19490	22868	15805.3	2900.78	28709.8307	3.307054564	8.47E-84	1.00E-81	up	Cytochrome P450 CYP4/CYP19/CYP26 subfamilies
Solyc06g050170.4	1042	1152	1140	5825	5014	9824	4718.018	856.857	8579.17957	3.323977395	2.03E-83	2.39E-81	up	--
Solyc03g114100.1	1447	1409	1305	95	76	125	597.4609	1070.66	124.261269	-3.100644539	3.17E-83	3.72E-81	down	--
Solyc07g053410.4	12	9	8	387	408	449	269.9216	7.46964	532.373563	6.157592802	5.85E-83	6.81E-81	up	--
Solyc04g005610.3	277	263	303	857	860	1208	725.8961	216.266	1235.52581	2.514347168	1.52E-82	1.76E-80	up	--
Solyc12g014530.3	386	293	384	1948	1661	3061	1525.993	272.401	2779.58581	3.351243616	2.63E-82	3.03E-80	up	Succinate dehydrogenase, flavoprotein subunit
Solyc04g009440.3	327	204	193	2862	3044	2972	2004.782	186.417	3823.14724	4.35834768	3.43E-82	3.92E-80	up	--
Solyc03g093080.3	2484	1581	2125	23	30	58	816.2841	1586.71	45.863198	-5.097476075	5.25E-82	5.97E-80	down	--
Solyc08g065190.3	425	562	582	2778	3111	4721	2429.653	402.819	4456.48729	3.46784621	7.19E-82	8.13E-80	up	Fe2+/Zn2+ regulated transporter
Solyc07g054780.1	5639	6664	2985	37	48	60	2014.313	3967.11	61.5165436	-6.011542309	9.77E-82	1.10E-79	down	--
Solyc01g099580.3	130	94	92	849	683	1220	615.5794	81.3035	1149.85534	3.823740246	9.88E-82	1.10E-79	up	--
Solyc08g066070.1	1671	1583	1633	169	139	219	738.6892	1255.69	221.684839	-2.49747171	1.61E-81	1.78E-79	down	Exocyst component protein and related proteins
novel.1001	1031	989	1081	79	68	75	445.6247	796.118	95.1318279	-3.073043238	1.64E-81	1.81E-79	down	--
Solyc08g007240.4	4182	3448	3320	68	23	82	1443.965	2817.06	70.8685484	-5.298549076	1.88E-81	2.07E-79	down	Predicted NUDIX hydrolase FGF-2 and related proteins
Solyc08g075550.4	23	13	6	632	882	855	515.0635	10.8875	1019.23942	6.556153684	2.07E-81	2.27E-79	up	--

Solyc07g061990.3	2602	2972	3365	8035	7467	9469	6454.264	2292.68	10615.8473	2.211011608	3.71E-81	4.03E-79	up	Geranylgeranyl pyrophosphate synthase/Polyprenyl synthetase
Solyc03g113270.3	10860	13254	11842	34759	33504	42273	28120.77	9252.58	46988.9667	2.344409068	8.23E-81	8.89E-79	up	Transcription factor HEX, contains HOX and HALZ domains
Solyc05g007480.1	230	152	231	1227	995	1603	882.0732	156.843	1607.30339	3.356719755	9.04E-81	9.72E-79	up	--
Solyc04g058000.2	1	6	4	943	1086	1769	795.4539	2.84008	1588.0678	9.134784026	5.83E-80	6.24E-78	up	Multicopper oxidases
Solyc12g056590.2	702	674	905	2381	2210	2772	1857.971	583.841	3132.10006	2.422832434	7.46E-80	7.93E-78	up	--
Solyc03g026110.4	296	343	364	1581	1393	2507	1271.82	257.489	2286.15183	3.150986588	8.03E-80	8.50E-78	up	--
Solyc10g076370.3	140	89	58	2269	1854	2127	1374.1	74.1872	2674.01358	5.173228398	1.00E-79	1.05E-77	up	--
Solyc04g009900.4	163	173	188	2211	3090	2115	1691.558	134.502	3248.61362	4.593607603	1.41E-79	1.48E-77	up	Ca2+/calmodulin-dependent protein kinase
Solyc03g117340.1	65	67	49	640	889	878	540.6267	46.7112	1034.54221	4.47078336	1.59E-79	1.65E-77	up	--
Solyc01g006680.4	1144	1389	1418	74	54	60	547.7005	1014.77	80.6337048	-3.660606263	1.21E-78	1.25E-76	down	Uncharacterized conserved protein, contains JmjC domain
novel.695	53	40	33	393	344	495	276.475	32.4827	520.467173	4.005735462	2.13E-78	2.18E-76	up	--
Solyc07g055060.3	3384	3300	3721	22974	32127	25915	18935.14	2669.96	35200.3099	3.720640663	2.32E-78	2.37E-76	up	--
novel.1539	3767	3080	2702	179	177	195	1347.734	2459.57	235.893421	-3.384993532	1.12E-77	1.14E-75	down	--
Solyc08g006500.4	102	105	85	489	453	646	373.018	75.2606	670.775349	3.158675579	1.73E-77	1.75E-75	up	Glutamate-gated kainate-type ion channel receptor
Solyc02g087960.3	238	182	250	898	855	1289	726.0516	171.573	1280.52983	2.899544538	3.00E-77	3.02E-75	up	Transcription factor, Myb superfamily
novel.887	2160	1966	2056	240	193	306	949.5696	1588.29	310.84495	-2.349667598	1.67E-76	1.67E-74	down	--
Solyc08g078340.3	583	655	632	1775	1432	1940	1332.111	480.758	2183.46454	2.183506988	2.25E-76	2.23E-74	up	--
Solyc03g097050.3	3686	2354	3401	118	142	174	1301.041	2417.63	184.447118	-3.713353353	3.76E-76	3.72E-74	down	--
Solyc07g045180.4	937	974	1113	2500	2603	3063	2129.015	775.685	3482.3447	2.166049328	1.05E-75	1.03E-73	up	--
Solyc01g100760.2	671	1109	936	5866	6278	8757	4762.757	699.176	8826.33854	3.658255577	3.13E-75	3.04E-73	up	--
Solyc02g088070.3	176	150	145	1000	732	1411	715.308	121.157	1309.45873	3.435685539	4.29E-75	4.15E-73	up	--
Solyc07g008840.3	1644	1532	2104	5387	5477	6561	4388.059	1351.14	7424.97541	2.457896451	4.86E-75	4.68E-73	up	Ypt/Rab-specific GTPase-activating protein GYP6
Solyc06g011560.3	767	858	927	2073	1976	2822	1778.108	655.028	2901.18908	2.147207483	2.96E-74	2.84E-72	up	--
Solyc06g065540.3	839	811	1032	2167	2036	2695	1806.189	687.015	2925.36303	2.0897885	3.45E-74	3.29E-72	up	Phosphatidylinositol-4-phosphate 5-kinase
Solyc04g078950.1	75	61	71	366	324	469	271.2627	53.1209	489.404442	3.204154152	5.15E-74	4.89E-72	up	--
Solyc11g042460.2	441	462	538	1212	1180	1619	1033.274	369.52	1697.02843	2.199135705	8.96E-74	8.47E-72	up	Predicted bile acid beta-glucosidase
Solyc06g061020.3	2682	3184	4186	17860	20294	20516	13891.19	2571.98	25210.4033	3.292963795	4.24E-73	4.00E-71	up	Predicted E3 ubiquitin ligase
Solyc11g011340.2	57	45	61	1943	1378	987	962.5108	41.744	1883.27756	5.494149413	5.06E-73	4.74E-71	up	Alcohol dehydrogenase, class V
Solyc06g074800.1	43	36	58	358	408	545	294.8296	34.9883	554.67084	3.983103009	6.63E-72	6.15E-70	up	FOG: Zn-finger
Solyc06g060310.3	176	175	204	642	556	884	508.561	142.339	874.7832	2.620451982	1.21E-71	1.12E-69	up	--
Solyc05g051425.2	12	26	22	469	664	573	377.0177	15.4401	738.595245	5.58061909	1.29E-71	1.18E-69	up	Phospholipase/carboxyhydrolase
Solyc01g080460.3	6976	9127	9640	30910	34403	44796	26635.86	6607.34	46664.375	2.820175423	1.82E-71	1.65E-69	up	--
Solyc04g054260.4	300	330	350	978	849	1346	792.4874	251.617	1333.35774	2.406742725	3.13E-71	2.82E-69	up	Cytochrome P450 CYP2 subfamily
novel.1657	1272	799	1078	9	9	11	409.7696	807.194	12.3453728	-6.035524653	5.23E-71	4.70E-69	down	--
Solyc02g090360.3	3250	1830	3786	39	58	70	1165.582	2260.24	70.9247373	-4.99531419	1.12E-70	1.00E-68	down	Multicopper oxidases
Solyc12g007070.2	1094	1347	1692	7658	6112	11488	5791.135	1058.14	10524.1316	3.314104168	3.41E-70	3.04E-68	up	Heat shock transcription factor
Solyc07g056000.2	3197	2098	2321	38	64	89	1018.676	1957.01	80.3444871	-4.603861634	3.46E-70	3.07E-68	down	--
Solyc05g013150.4	438	457	484	1112	990	1500	935.57	354.114	1517.02633	2.099813265	4.02E-70	3.54E-68	up	N-methyltransferase
Solyc01g080900.4	372	210	235	2842	2766	2678	1890.671	210.001	3571.3403	4.087894703	4.69E-70	4.12E-68	up	Cytochrome P450 CYP4/CYP19/CYP26 subfamilies
Solyc05g047460.3	556	415	584	2186	1714	2951	1633.809	398.103	2869.51607	2.849604657	6.31E-70	5.47E-68	up	--
Solyc04g076220.3	302	282	332	1259	1685	1775	1128.141	234.932	2021.35036	3.104296013	2.93E-69	2.52E-67	up	--
Solyc02g090120.1	683	668	613	17	14	20	263.4905	505.412	21.5687735	-4.547364883	3.21E-69	2.74E-67	down	--
Solyc02g092460.3	1682	1562	1645	103	62	142	691.5093	1255.91	127.109766	-3.29312009	9.76E-69	8.31E-67	down	CREB binding protein/P300 and related TAZ Zn-finger proteins

Solyc04g080300.4	1827	1610	1947	5936	5224	9430	4983.708	1380.58	8586.83896	2.637000415	1.82E-68	1.54E-66	up	--
Solyc02g037530.3	362	373	376	1055	826	1126	780.5638	285.503	1275.62481	2.159896275	5.12E-68	4.29E-66	up	--
Solyc09g091030.3	3094	3113	4502	13899	12487	19970	11099.62	2737.53	19461.712	2.829646729	7.03E-68	5.86E-66	up	--
Solyc06g061030.3	370	304	401	1081	1182	1511	938.364	275.374	1601.3538	2.539128311	1.20E-67	9.97E-66	up	GATA-4/5/6 transcription factors
Solyc02g065680.3	2075	1971	1895	6377	5513	9865	5303.761	1527.98	9079.54517	2.571265855	3.85E-67	3.18E-65	up	Amino acid transporter protein
Solyc05g055790.4	591	686	756	1901	1890	2182	1536.156	521.614	2550.69743	2.289294494	3.98E-67	3.28E-65	up	Serine/threonine protein phosphatase
Solyc09g075080.3	277	289	287	733	649	951	601.8574	219.242	984.472319	2.168076982	4.01E-67	3.29E-65	up	--
Solyc10g077040.2	951	898	1081	3019	2491	4428	2451.596	751.208	4151.98309	2.466816079	4.93E-67	4.03E-65	up	--
Solyc03g115940.4	144	140	185	620	520	836	475.0679	120.068	830.067615	2.789336234	4.98E-67	4.05E-65	up	--
Solyc05g012850.3	957	643	879	10	12	14	325.2583	635.174	15.3427873	-5.377528016	5.49E-67	4.44E-65	down	--
Solyc10g006330.3	1544	1883	2219	6312	5164	7362	4707.682	1446.96	7968.39873	2.461207965	8.21E-67	6.62E-65	up	--
Solyc02g092700.3	588	601	753	1783	1675	2557	1514.048	497.487	2530.60812	2.346796688	1.67E-66	1.34E-64	up	--
Solyc07g008180.4	1192	1360	1025	5372	3956	6527	3792.507	922.681	6662.33323	2.852527877	2.08E-66	1.66E-64	up	--
Solyc02g083310.3	1001	957	1060	96	102	124	455.8584	774.67	137.046951	-2.501789235	2.22E-66	1.76E-64	down	--
Solyc12g099250.2	638	554	624	1921	1491	1860	1356.585	466.15	2247.02029	2.268789839	3.10E-66	2.45E-64	up	Tyrosine kinase specific for activated (GTP-bound) p21cdc42Hs
Solyc08g081710.3	12	3	8	285	304	408	213.9212	5.87348	421.968888	6.160326805	3.47E-66	2.73E-64	up	--
Solyc01g100850.3	552	545	620	1718	1402	2452	1385.476	440.518	2330.43339	2.403974377	4.26E-66	3.34E-64	up	Predicted ABC-type transport, ATPase component
Solyc12g056650.2	6214	6234	6959	655	514	524	2854.503	4980.25	728.752356	-2.774500876	4.35E-66	3.40E-64	down	--
novel.2007	1571	1469	1339	145	113	155	651.1178	1127.08	175.153697	-2.685688015	5.61E-66	4.36E-64	down	FOG: Transposon-encoded proteins with TYA
Solyc04g007890.3	2454	2366	2623	165	161	296	1084.588	1910.43	258.750331	-2.878584069	6.66E-66	5.15E-64	down	--
Solyc04g071790.3	2	2	3	1317	1167	1212	795.0396	1.78843	1588.29067	9.787023211	7.71E-66	5.95E-64	up	Cytochrome P450 CYP2 subfamily
Solyc11g008250.2	342	452	486	2072	2740	3683	1958.243	328.436	3588.05036	3.449490777	9.79E-66	7.53E-64	up	--
Solyc07g042580.3	32	23	51	398	326	532	277.1476	26.9596	527.335536	4.284212572	1.04E-65	7.93E-64	up	--
Solyc11g072623.1	46	32	25	410	427	741	342.3495	26.5729	658.126002	4.634356407	1.12E-65	8.51E-64	up	--
Solyc08g081200.3	651	759	842	2156	1715	2605	1654.272	577.741	2730.80372	2.24103033	1.29E-65	9.80E-64	up	Dehydrogenases with different specificities
Solyc02g077950.3	53	41	61	378	294	514	268.0136	39.6448	496.382284	3.645574542	2.38E-65	1.80E-63	up	--
novel.1498	1154	750	943	8	18	18	374.6178	730.376	18.8592689	-5.28664549	2.49E-65	1.88E-63	down	--
Solyc03g123370.3	1543	1570	1796	3945	4436	5008	3489.31	1259.26	5719.36089	2.18298869	2.86E-65	2.15E-63	up	Predicted guanosine polyphosphate pyrophosphohydrolase/synthase
Solyc03g113380.4	1129	893	1184	4310	4310	4188	3169.952	821.305	5518.59968	2.747867227	7.32E-65	5.45E-63	up	FOG: PPR repeat
Solyc10g051020.2	1532	924	645	17857	30756	31600	17582.24	801.081	34363.3983	5.422879284	7.73E-65	5.74E-63	up	Cytochrome P450 CYP4/CYP19/CYP26 subfamilies
Solyc09g090910.3	82	76	100	574	395	758	393.2357	66.065	720.406398	3.447243313	8.75E-65	6.47E-63	up	--
Solyc03g098100.4	77	70	54	376	383	496	292.1554	51.8458	532.464944	3.363018342	1.06E-64	7.78E-63	up	Aldo/keto reductase family proteins
Solyc02g062390.3	380	724	541	5208	7615	7807	4635.131	424.174	8846.08839	4.382489295	1.66E-64	1.22E-62	up	--
Solyc11g008720.3	333	643	612	5228	6823	6631	4223.507	407.95	8039.06354	4.300522142	1.67E-64	1.22E-62	up	Beta-glucosidase, lactase phlorizinhydrolase, and related proteins
Solyc04g049680.2	560	580	622	28	20	34	243.4033	452.391	34.4152555	-3.705983948	1.80E-64	1.31E-62	down	GATA-4/5/6 transcription factors
Solyc01g098520.3	638	563	715	31	28	34	265.2979	490.957	39.6390233	-3.635844159	2.13E-64	1.55E-62	down	DNA mismatch repair protein - MLH3 family
Solyc01g105370.4	445	450	577	2872	4499	6415	3085.256	376.968	5793.5435	3.941796731	2.48E-64	1.79E-62	up	--
Solyc10g005640.4	161	149	155	490	453	678	401.173	119.473	682.873277	2.516454192	2.68E-64	1.93E-62	up	Serine/threonine protein phosphatase
Solyc01g106580.3	1456	1499	1495	190	143	183	681.7172	1143.73	219.707741	-2.381807914	2.95E-64	2.12E-62	down	--
Solyc06g073450.4	1854	2284	1860	5941	5629	8176	4937.771	1545.44	8330.10308	2.430552501	3.14E-64	2.24E-62	up	FOG: PPR repeat
Solyc03g007370.3	481	871	740	4670	4477	6728	3610.848	538.426	6683.27017	3.633941416	3.22E-64	2.29E-62	up	--
Solyc08g008490.4	848	1084	1266	4105	4434	4894	3283.213	819.601	5746.82488	2.809500774	3.62E-64	2.56E-62	up	WD40 repeat-containing protein
Solyc02g085630.3	105	47	57	1029	1526	2170	1020.327	53.7112	1986.94226	5.209462421	4.24E-64	2.99E-62	up	Transcription factor HEX, contains HOX and HALZ domains
Solyc02g084430.3	93	172	136	1300	1115	2216	1012.298	103.316	1921.27903	4.218196075	4.31E-64	3.03E-62	up	--

Solyc04g078500.1	28	14	30	289	256	407	209.1214	18.3582	399.88449	4.44031485	5.29E-64	3.71E-62	up	--
Solyc03g097370.4	1091	1263	1291	3021	3184	3633	2569.132	936.255	4202.0083	2.165857501	5.43E-64	3.79E-62	up	DNA helicase
novel.1742	1341	889	1043	12	20	35	434.076	840.371	27.7813922	-4.902365264	1.08E-63	7.50E-62	down	--
Solyc08g074270.4	463	660	536	2165	2190	3110	1789.201	427.393	3151.00906	2.882687562	1.21E-63	8.35E-62	up	Deoxyribodipyrimidine photolyase/cryptochrome
Solyc03g121090.4	2220	1865	2370	6745	5700	10212	5556.334	1654.29	9458.38253	2.515489995	1.49E-63	1.03E-61	up	Nuclear localization sequence binding protein
Solyc03g115920.3	1778	1629	1658	8606	8495	7245	5929.803	1301.78	10557.8301	3.019631683	1.99E-63	1.37E-61	up	Predicted E3 ubiquitin ligase
Solyc08g081400.4	409	327	385	1409	1545	1522	1106.979	287.644	1926.31496	2.742572126	2.33E-63	1.60E-61	up	Transcription factor MEIS1 and related HOX domain proteins
Solyc09g083380.4	1763	1582	1710	168	158	257	771.2909	1298.2	244.384375	-2.40450012	3.13E-63	2.13E-61	down	--
Solyc08g077230.3	467	956	1122	10075	10817	13297	7596.247	651.497	14540.9971	4.480120955	4.50E-63	3.06E-61	up	GATA-4/5/6 transcription factors
Solyc02g083250.3	1816	1865	2119	4721	5286	5767	4119.443	1487.93	6750.95662	2.181513397	4.69E-63	3.17E-61	up	Hismacro and SEC14 domain-containing proteins
Solyc10g052470.1	7	3	4	694	407	1159	464.2382	3.59454	924.881821	8.008412277	9.84E-63	6.63E-61	up	Zuotin and related molecular chaperones (DnaJ superfamily)
Solyc05g006810.4	448	399	468	1077	1096	1598	963.2939	337.331	1589.25668	2.23639005	1.07E-62	7.17E-61	up	Monooxygenase involved in coenzyme Q (ubiquinone) biosynthesis
Solyc08g082590.3	82	119	120	732	708	1341	618.7129	82.43	1154.99575	3.809564174	1.86E-62	1.24E-60	up	Glutaredoxin-related protein
Solyc07g007930.3	16241	21291	16091	1574	1423	1591	7896.733	13829.5	1963.97568	-2.816290488	2.18E-62	1.45E-60	down	--
Solyc02g069260.3	1485	920	1119	5123	4914	6462	3951.374	904.596	6998.15092	2.951599225	3.46E-62	2.29E-60	up	Translation initiation factor 2C (eIF-2C) and related proteins
Solyc09g091000.4	18	19	28	327	545	722	344.6017	16.6082	672.595136	5.336548995	6.77E-62	4.47E-60	up	--
Solyc02g092000.3	1360	1399	1368	166	119	155	624.1386	1061	187.272603	-2.503694392	6.93E-62	4.56E-60	down	--
Solyc02g064960.3	45	21	32	486	327	453	281.3199	25.1119	537.527935	4.418442947	8.25E-62	5.41E-60	up	--
novel.674	1101	877	1004	82	69	77	431.5665	765.471	97.6617337	-2.976806265	1.12E-61	7.26E-60	down	--
Solyc03g117850.4	599	726	780	1919	1677	2115	1485.123	540.236	2430.01	2.169025478	1.42E-61	9.17E-60	up	26S proteasome regulatory complex, ATPase RPT4
Solyc11g067080.3	234	264	308	804	687	899	610.898	206.637	1015.15878	2.295785539	1.57E-61	1.01E-59	up	Putative serine/threonine protein kinase
Solyc08g075540.5.1	3855	2485	2282	19925	21280	21958	14670.89	2220.62	27121.1726	3.610403349	1.61E-61	1.03E-59	up	--
Solyc12g087790.1	2325	1556	1981	31	59	80	787.5024	1503.45	71.5584773	-4.390802747	1.88E-61	1.20E-59	down	--
Solyc08g008280.3	1708	955	1655	25	30	50	573.7218	1103.62	43.8228454	-4.645882326	2.03E-61	1.29E-59	down	--
Solyc01g111700.2	9	4	3	354	262	430	221.8807	4.13179	439.629609	6.741961049	2.85E-61	1.82E-59	up	26S proteasome regulatory complex, subunit RPN3/PSMD3
Solyc02g081330.4	728	866	946	3283	2868	3007	2292.866	651.744	3933.98745	2.593230279	5.46E-61	3.47E-59	up	Squalene synthetase
Solyc01g102610.3	481	882	695	5035	4456	7853	3886.43	530.269	7242.59071	3.772004858	6.91E-61	4.37E-59	up	Ferric reductase, NADH/NADPH oxidase
Solyc02g087560.1	570	525	571	28	19	34	230.8565	427.787	33.9263061	-3.643926514	8.09E-61	5.10E-59	down	FOG: PPR repeat
Solyc03g005100.3	561	589	596	30	32	33	244.7169	448.641	40.7930381	-3.471335275	9.69E-61	6.07E-59	down	Arylacetamide deacetylase
Solyc11g069590.2	218	273	281	1150	825	1514	828.1568	198.242	1458.07202	2.879659224	1.30E-60	8.09E-59	up	Serine/threonine protein kinase
Solyc01g079610.3	902	904	944	71	86	87	405.5953	706.384	104.806491	-2.761705289	1.70E-60	1.06E-58	down	Molecular chaperone (DnaJ superfamily)
Solyc06g150137.1	2791	4475	4314	36431	29596	27176	21641.7	2975.15	40308.247	3.76002438	3.92E-60	2.42E-58	up	Cytochrome P450 CYP2 subfamily
Solyc03g117600.3	291	270	415	1357	1206	2004	1081.395	249.335	1913.45365	2.939602931	4.56E-60	2.81E-58	up	--
Solyc02g071500.2	147	124	130	610	425	669	410.7161	103.042	718.390082	2.802745012	4.57E-60	2.81E-58	up	Iron/ascorbate family oxidoreductases
Solyc11g068960.2	930	714	989	3490	2882	5465	2800.607	674.167	4927.04774	2.86964708	6.79E-60	4.14E-58	up	--
Solyc06g071680.4	2006	1916	1975	295	276	312	946.4328	1515.2	377.670601	-2.00762634	7.58E-60	4.60E-58	down	Histone deacetylase complex, catalytic component RPD3
Solyc04g078930.4	66	33	52	541	405	494	326.6632	38.6639	614.662552	3.988662014	7.89E-60	4.78E-58	up	Alpha-amylase
Solyc03g119440.3	929	867	1080	2255	2014	3219	1940.53	737.023	3144.03786	2.093070035	1.06E-59	6.42E-58	up	Ca2+-dependent lipid-binding protein CLB1
Solyc04g051500.3	503	449	514	1188	1353	1604	1070.923	376.193	1765.65341	2.23009497	1.23E-59	7.39E-58	up	Glucose-6-phosphate/phosphate and phosphoenolpyruvate
Solyc03g031420.1	1426	1068	1818	45	25	66	578.4068	1100.86	55.9561864	-4.282124646	1.42E-59	8.49E-58	down	Molybdenum cofactor sulfurase

Solyc08g061270.1	2114	3544	2406	17793	22284	32967	16386.77	2082.38	30691.1498	3.881599431	1.88E-59	1.12E-57	up	--
Solyc02g093520.3	3273	3072	3891	8829	10180	10974	7730.261	2622.46	12838.065	2.291268528	1.98E-59	1.18E-57	up	Copine
Solyc03g063730.4	500	382	571	1539	1550	1855	1239.297	371.632	2106.963	2.502282412	2.24E-59	1.33E-57	up	Uncharacterized membrane protein, predicted efflux pump
novel.1546	1181	912	1165	63	66	54	457.3193	835.135	79.503446	-3.407190186	2.64E-59	1.57E-57	down	--
Solyc10g084125.1	12	8	7	246	193	280	155.4762	6.95732	303.995109	5.45423368	3.33E-59	1.97E-57	up	FOG: Transposon-encoded proteins with TYA
Solyc02g062710.1	78	56	104	481	406	624	348.4868	60.6946	636.278981	3.387386004	4.51E-59	2.65E-57	up	--
Solyc05g015060.4	245	371	179	3128	2878	2991	2035.714	206.177	3865.25108	4.229531997	4.64E-59	2.72E-57	up	AAA+-type ATPase
Solyc12g010690.2	550	565	634	17	28	27	239.8668	448.769	30.9648074	-3.871844634	4.81E-59	2.81E-57	down	Predicted transporter (major facilitator superfamily)
Solyc08g008480.4	9	34	20	815	494	635	422.8515	16.2995	829.403411	5.673745876	9.44E-59	5.48E-57	up	Transcription factor, Myb superfamily
Solyc06g036260.3	563	640	599	1850	2133	2174	1553.877	463.465	2644.29006	2.512028778	9.78E-59	5.66E-57	up	--
Solyc12g010130.1	1977	2446	2290	5619	4675	7158	4539.061	1726.27	7351.85463	2.090668823	1.31E-58	7.55E-57	up	Serine/threonine protein kinase
Solyc08g079090.4	1690	1272	2144	40	27	78	681.4244	1303.73	59.1198322	-4.444633881	1.47E-58	8.44E-57	down	Multicopper oxidases
Solyc07g056240.3	166	143	118	914	811	1661	755.8128	110.058	1401.56806	3.672511936	4.01E-58	2.30E-56	up	--
novel.990	828	670	763	49	53	67	326.084	580.407	71.7607245	-3.017220842	4.10E-58	2.35E-56	down	--
Solyc06g076030.3	62	61	53	325	345	553	278.8361	45.3239	512.348263	3.501950111	6.46E-58	3.66E-56	up	--
Solyc07g054760.1	2661	3233	2198	152	147	228	1155.701	2089.96	221.440315	-3.236206462	1.05E-57	5.95E-56	down	--
Solyc06g072840.3	5262	8098	4538	268	233	292	2485.538	4633.53	337.548386	-3.779366645	1.21E-57	6.81E-56	down	--
Solyc01g096320.3	855	1346	1148	5300	5403	8893	4531.276	862.049	8200.50185	3.250071188	1.45E-57	8.17E-56	up	Transcription factor HEX, contains HOX and HALZ domains
Solyc08g007800.3	450	413	381	1174	1289	1446	995.4046	320.146	1670.66327	2.383473831	2.09E-57	1.17E-55	up	Predicted transporter/transmembrane protein
Solyc02g067690.3	997	1133	1223	96	53	89	480.4677	860.601	100.334719	-3.096835772	3.81E-57	2.13E-55	down	UDP-glucuronosyl and UDP-glucosyl transferase
Solyc12g005950.2	1474	1538	1764	4089	3824	6484	3622.981	1225.01	6020.95048	2.297402396	4.63E-57	2.58E-55	up	--
novel.1533	782	574	660	12	22	25	271.3568	517.598	25.1153729	-4.369724759	6.81E-57	3.79E-55	down	--
Solyc05g052950.4	6	1	1	515	609	785	405.5695	2.06486	809.074077	8.621042155	1.10E-56	6.04E-55	up	FOG: RCC1 domain
Solyc02g091180.1	849	781	958	2	4	6	334.2067	663.397	5.01693026	-7.028346752	1.85E-56	1.01E-54	down	--
Solyc10g044520.3	5575	6396	4798	16587	16506	21843	13805.39	4325.77	23285.0123	2.42844024	1.85E-56	1.01E-54	up	--
Solyc10g055630.2	1404	1106	1210	11419	10665	7496	6948.112	955.53	12940.6933	3.759374236	2.34E-56	1.27E-54	up	Aquaporin (major intrinsic protein family)
Solyc01g059965.1	4	6	22	372	311	531	258.1571	8.04954	508.264739	5.962549936	2.81E-56	1.52E-54	up	--
Solyc02g082440.2	1968	1420	1828	169	160	209	782.7586	1337.21	228.308858	-2.550640592	3.95E-56	2.13E-54	down	DNA mismatch repair protein - MLH3 family
Solyc02g093840.3	587	516	623	1248	1337	1790	1147.224	442.598	1851.85055	2.064719526	4.97E-56	2.68E-54	up	--
Solyc08g080750.4	151	159	178	948	725	1654	748.5245	125.21	1371.83897	3.454667227	5.30E-56	2.85E-54	up	--
Solyc06g059750.3	1499	1354	1865	3621	3401	4868	3113.987	1207.41	5020.56877	2.055809574	5.77E-56	3.08E-54	up	--
Solyc06g059870.1	398	507	329	1842	1957	2712	1534.834	318.89	2750.77864	3.109542479	6.00E-56	3.20E-54	up	--
Solyc09g008020.4	2512	3140	2703	10714	13670	21121	10608.75	2151.04	19066.464	3.148011692	6.74E-56	3.59E-54	up	OTU (ovarian tumor)-like cysteine protease
novel.885	3677	3947	4008	388	379	641	1788.589	2988.58	588.59465	-2.341651566	9.22E-56	4.88E-54	down	--
Solyc01g099990.3	122	193	142	1045	1421	2267	1047.916	117.885	1977.94683	4.069667535	1.14E-55	5.98E-54	up	--
Solyc12g005660.2	5	5	8	382	322	869	322.8879	4.59423	641.181524	7.120225184	1.30E-55	6.81E-54	up	--
Solyc07g043170.4	44	42	51	351	227	401	222.878	35.1192	410.636846	3.548285823	1.76E-55	9.14E-54	up	UDP-glucuronosyl and UDP-glucosyl transferase
Solyc05g009360.4	2576	3271	2896	7831	7409	11631	6768.03	2249.99	11286.0756	2.326717617	2.65E-55	1.38E-53	up	--
Solyc11g069570.2	77	32	58	541	435	688	371.3725	42.7219	700.023045	4.033111581	4.04E-55	2.09E-53	up	--
Solyc08g076410.4	412	523	539	1852	1309	1862	1253.535	378.49	2128.58031	2.491653264	4.40E-55	2.27E-53	up	--
Solyc08g081170.4	1340	1207	1162	72	67	48	517.8797	954.017	81.742563	-3.559623209	5.80E-55	2.99E-53	down	--
Solyc07g008280.4	45	49	56	400	669	957	444.629	38.4716	850.786473	4.466779931	5.86E-55	3.01E-53	up	--
Solyc08g006460.3	1361	1701	1951	4508	4050	5408	3603.912	1285.19	5922.63426	2.204166688	6.09E-55	3.12E-53	up	FOG: Predicted E3 ubiquitin ligase
Solyc06g048520.3	734	726	710	1729	1973	2156	1532.252	557.928	2506.5748	2.167224095	6.76E-55	3.46E-53	up	Uncharacterized conserved protein
Solyc07g056280.3	85	56	66	394	484	708	360.0599	53.1469	666.972932	3.650062332	9.78E-55	4.97E-53	up	--

Solyc02g150143.1	923	896	1055	77	38	79	409.0369	737.028	81.0461321	-3.175495481	1.20E-54	6.08E-53	down	DNA mismatch repair protein - MLH3 family
novel.1368	1034	873	906	55	75	74	405.3134	722.934	87.692699	-3.052018288	1.72E-54	8.70E-53	down	--
Solyc06g072710.3	503	635	709	2177	2061	3593	1871.443	473.701	3269.18453	2.787141945	1.79E-54	9.02E-53	up	--
Solyc09g007840.3	1	5	1	627	642	685	419.7966	1.83518	837.758011	8.862041057	2.48E-54	1.24E-52	up	K+-channel ERG and related proteins
Solyc01g010390.3	225	174	291	1201	894	1693	877.7608	176.179	1579.3426	3.16379318	2.65E-54	1.33E-52	up	Beta-glucosidase, lactase phlorizinhydrolase
Solyc08g083130.4	3711	4428	3995	406	278	535	1815.299	3122.14	508.458269	-2.615526405	2.92E-54	1.46E-52	down	Transcription factor HEX, contains HOX and HALZ domains
Solyc07g053180.3	734	627	913	2087	2003	3033	1789.495	581.588	2997.40086	2.365467328	3.09E-54	1.54E-52	up	Protein phosphatase 1, regulatory subunit, and related proteins
Solyc05g055860.4	4	0	4	480	503	844	382.728	2.02018	763.435878	8.54061936	3.27E-54	1.63E-52	up	Serine/threonine protein kinase
Solyc12g056980.1	147	131	177	519	405	605	380.9837	116.48	645.487443	2.469755419	4.31E-54	2.14E-52	up	--
Solyc10g076360.1	1047	815	982	4675	3373	3678	2881.924	729.586	5034.26199	2.786397929	8.29E-54	4.10E-52	up	Prenylated rab acceptor 1
Solyc03g093120.5.1	1430	994	1174	11	28	43	478.8837	923.597	34.1707308	-4.747099592	9.32E-54	4.59E-52	down	--
Solyc02g068040.3	577	509	597	42	38	48	243.0932	431.745	54.4413028	-2.990323941	1.28E-53	6.28E-52	down	--
Solyc02g086880.4	5078	5029	6264	16012	19067	18380	13609.2	4194.57	23023.823	2.456432047	1.75E-53	8.57E-52	up	Glyoxylate/hydroxypyruvate reductase
Solyc02g089160.3	290	270	346	990	1326	1658	958.8042	232.083	1685.52553	2.859976402	2.18E-53	1.06E-51	up	Cytochrome P450 CYP4/CYP19/CYP26 subfamilies
Solyc05g006980.3	802	635	650	2108	1725	2947	1687.929	536.538	2839.32045	2.404385227	2.80E-53	1.36E-51	up	Transcription factor HEX, contains HOX and HALZ domains
Solyc11g068710.3	1043	822	1384	3	6	2	417.1981	829.421	4.9746527	-7.463941961	3.67E-53	1.78E-51	down	--
Solyc04g054490.3	204	263	263	690	688	861	569.7319	187.526	951.938271	2.34356951	4.09E-53	1.98E-51	up	Aspartyl protease
Solyc06g066340.4	155	109	159	512	518	764	431.9397	108.264	755.615283	2.802529609	5.03E-53	2.43E-51	up	--
Solyc09g083090.4	1042	1108	891	3336	4420	5301	3167.88	783.826	5551.93342	2.824542171	6.31E-53	3.03E-51	up	Serine/threonine protein kinase
Solyc03g111550.4	1284	1661	2318	8084	9936	11650	6992.123	1345.01	12639.234	3.23207448	7.07E-53	3.39E-51	up	--
Solyc06g083310.3	5371	4575	5050	745	503	812	2359.15	3850.62	867.68394	-2.148808845	8.45E-53	4.04E-51	down	--
Solyc02g085000.3	1325	1659	1772	3737	3797	4957	3258.988	1220.62	5297.36094	2.117620583	1.04E-52	4.95E-51	up	--
Solyc11g066740.2	1277	1354	1214	132	113	113	571.8956	989.628	154.163159	-2.689065814	1.20E-52	5.74E-51	down	--
novel.352	1107	1163	1027	110	110	115	496.2874	848.772	143.802493	-2.567518412	1.33E-52	6.31E-51	down	--
Solyc07g062440.1	887	873	854	99	91	99	397.9804	672.09	123.870886	-2.4464799	1.93E-52	9.13E-51	down	--
Solyc01g106700.3	42	49	69	709	1904	1523	918.4678	40.897	1796.03852	5.455167289	2.47E-52	1.16E-50	up	MADS box transcription factor
Solyc02g092490.3	720	539	561	5232	2602	4415	2818.664	467.862	5169.46554	3.465994142	2.71E-52	1.28E-50	up	--
Solyc10g085240.1	33	26	43	366	427	339	259.2207	26.0461	492.395292	4.235093902	3.71E-52	1.73E-50	up	UDP-glucuronosyl and UDP-glucosyl transferase
novel.805	2	13	6	255	196	306	162.1631	5.4536	318.872662	5.883799789	6.16E-52	2.86E-50	up	--
Solyc01g095570.3	573	503	524	1586	1456	2641	1389.553	411.135	2367.9711	2.526724636	8.46E-52	3.93E-50	up	Mitochondrial solute carrier protein
Solyc09g150143.1	2929	3215	2786	7002	6720	10655	6265.142	2299.34	10230.9459	2.153857762	9.90E-52	4.58E-50	up	--
Solyc08g075020.3	688	1206	1404	6753	7834	10759	5774.632	844.643	10704.6219	3.663685335	1.06E-51	4.89E-50	up	Pectin acetylesterase and similar proteins
Solyc08g066100.3	1170	863	1043	74	41	54	430.6487	789.206	72.091012	-3.455108707	1.40E-51	6.46E-50	down	Pyrophosphate-dependent phosphofructo-1-kinase
novel.1654	567	447	511	23	23	23	210.5849	391.483	29.686839	-3.735093925	2.01E-51	9.18E-50	down	--
Solyc05g041430.3	477	508	491	1076	992	1558	951.4448	379.497	1523.39288	2.006280217	2.33E-51	1.06E-49	up	--
Solyc03g063600.4	6540	7620	7266	18448	19803	19978	15270.2	5508.93	25031.4722	2.183849603	2.34E-51	1.06E-49	up	Guanylate kinase
Solyc06g053710.3	6669	7323	7718	20229	17925	18503	14964.68	5574.62	24354.7406	2.127188905	2.51E-51	1.13E-49	up	Sensory transduction histidine kinase
Solyc05g014300.1	44	32	11	569	825	969	513.9809	22.6074	1005.35435	5.479806272	3.18E-51	1.44E-49	up	--
Solyc08g080640.2	1583	2385	3618	18834	21431	34544	16619.6	1935.16	31304.0458	4.015766586	3.87E-51	1.74E-49	up	--
Solyc02g087975.1	628	554	358	3481	3105	6179	2845.186	398.05	5292.32269	3.733448365	6.17E-51	2.77E-49	up	--
Solyc01g005560.3	1633	2086	2557	7312	7658	8145	5758.896	1607.24	9910.5482	2.624203173	6.74E-51	3.02E-49	up	NADP-dependent isocitrate dehydrogenase
Solyc11g008905.1	29	40	49	244	240	367	194.0081	30.2132	357.803035	3.565293234	7.74E-51	3.46E-49	up	--
Solyc06g062480.4	601	572	809	1869	1650	1985	1427.248	506.928	2347.56789	2.21050332	8.47E-51	3.78E-49	up	--
Solyc01g079660.2	735	478	674	4	9	8	246.2056	483.346	9.06524264	-5.75817573	1.14E-50	5.09E-49	down	--

Solyc09g075890.3	776	475	710	14	18	22	262.4823	502.023	22.9411818	-4.454151352	1.33E-50	5.92E-49	down	Haloacid dehalogenase-like hydrolase
Solyc10g008400.1	3555	3487	4844	186	180	380	1674.188	3040.54	307.834491	-3.29996977	1.37E-50	6.05E-49	down	Predicted E3 ubiquitin ligase
Solyc04g081530.1	282	300	331	1051	870	1706	870.5177	234.299	1506.73627	2.686001185	1.61E-50	7.12E-49	up	Molecular chaperone (DnaJ superfamily)
Solyc12g096070.2	162	194	230	683	876	1034	627.0066	150.174	1103.8388	2.876993068	2.37E-50	1.04E-48	up	--
Solyc02g089640.3	3713	2602	3601	242	305	324	1456.447	2539.85	373.041115	-2.769413727	2.50E-50	1.10E-48	down	--
Solyc01g096720.4	1	4	3	441	799	706	421.4412	2.06173	840.820575	8.675702837	2.78E-50	1.22E-48	up	Permease of the major facilitator superfamily
novel.1747	1314	896	1114	80	76	111	482.6658	852.733	112.598919	-2.91846461	3.53E-50	1.54E-48	down	--
Solyc03g093110.3	815	585	660	7	19	25	275.2625	529.063	21.46157	-4.618425137	4.24E-50	1.84E-48	down	--
Solyc09g091600.4	42	64	45	388	643	814	409.8468	38.9765	780.717069	4.326264776	5.67E-50	2.45E-48	up	--
Solyc06g075370.3	312	288	431	1906	2003	4290	1819.886	263.498	3376.27361	3.679489107	6.12E-50	2.64E-48	up	--
Solyc02g014860.3	1522	2073	2197	81	74	45	787.2038	1486.4	88.0085517	-4.090072756	7.69E-50	3.31E-48	down	Molecular chaperone (DnaJ superfamily)
Solyc08g014000.3	31698	32543	33311	5142	3480	4534	15333.14	25063.5	5602.76053	-2.161442558	1.01E-49	4.33E-48	down	--
Solyc01g087020.2	2780	2394	2068	48	74	141	987.0476	1865.54	108.556264	-4.096228566	1.62E-49	6.93E-48	down	--
Solyc04g009020.4	402	476	539	1348	1140	1961	1112.488	363.399	1861.57762	2.357436066	1.90E-49	8.09E-48	up	FOG: TPR repeat
novel.1111	296	396	338	1390	1965	1914	1265.695	265.184	2266.20486	3.095151416	1.92E-49	8.18E-48	up	--
Solyc12g007100.2	681	583	787	1518	1501	2203	1362.882	525.137	2200.62763	2.067071971	2.10E-49	8.93E-48	up	AAA+-type ATPase
Solyc11g072110.2	1810	2484	2075	6936	5569	9619	5450.999	1640.21	9261.78749	2.49762938	2.17E-49	9.18E-48	up	Iron/ascorbate family oxidoreductases
Solyc08g075870.3	7866	8538	10464	832	1043	1203	4098.068	6883.89	1312.24681	-2.391676777	2.23E-49	9.40E-48	down	--
Solyc04g014500.3	1859	2791	2906	8652	9188	11568	7215.642	1939.23	12492.0578	2.687434336	2.23E-49	9.40E-48	up	Diadenosine and diphosphoinositol polyphosphate phosphohydrolase
Solyc06g068800.3	311	367	378	884	748	1210	732.2522	271.203	1193.30182	2.138608441	2.93E-49	1.23E-47	up	Phosphatidylinositol transfer protein PDR16
novel.1675	753	565	639	46	31	50	278.0125	502.528	53.4970046	-3.226504054	2.99E-49	1.26E-47	down	--
Solyc02g092870.2	220	206	259	555	486	733	461.4987	175.517	747.480568	2.09072547	3.75E-49	1.57E-47	up	--
Solyc07g007410.4	4446	3732	4695	525	329	429	1923.196	3299.57	546.818677	-2.593687578	4.79E-49	1.99E-47	down	--
Solyc07g053270.3	11	25	22	206	251	298	168.1665	14.9154	321.417587	4.43024638	5.64E-49	2.34E-47	up	Predicted haloacid-halidohydrolase and related hydrolases
Solyc02g021220.1	534	641	778	4495	4005	3159	2787.87	500.312	5075.42827	3.342255771	8.56E-49	3.54E-47	up	--
Solyc07g065320.4	67	61	79	264	277	398	224.4797	53.0212	395.938127	2.900113504	8.80E-49	3.63E-47	up	Multidrug resistance-associated protein
Solyc05g006430.3	46	40	50	304	211	420	212.019	34.8583	389.179726	3.482542454	1.01E-48	4.15E-47	up	Rho GTPase effector BNI1 and related formins
Solyc04g014810.3	933	1135	1107	2797	2597	2801	2164.924	816.003	3513.84546	2.106107228	1.16E-48	4.76E-47	up	FOG: Armadillo/beta-catenin-like repeats
Solyc06g060690.3	991	677	1090	29	41	57	379.2425	704.983	53.501595	-3.717022246	1.32E-48	5.39E-47	down	Serine/threonine protein kinase
Solyc06g062780.4	686	508	542	1712	2026	2255	1503.633	446.138	2561.12826	2.52101155	1.72E-48	7.03E-47	up	P-type ATPase
Solyc12g099500.2	1891	1395	1843	75	86	163	724.2887	1314.33	134.249832	-3.284183528	2.75E-48	1.12E-46	down	--
Solyc11g006270.2	1063	1357	1177	4813	7367	6724	4541.681	925.94	8157.42319	3.139086876	2.87E-48	1.16E-46	up	Steroid reductase
Solyc08g068700.1	610	406	577	9	5	12	209.3557	407.957	10.7539671	-5.217739988	3.32E-48	1.35E-46	down	Diamine acetyltransferase
Solyc03g116710.3	167	193	233	598	551	649	459.7011	151.941	767.461175	2.334911695	3.34E-48	1.35E-46	up	--
Solyc03g118840.3	892	1219	1090	2698	2625	3538	2288.172	823.553	3752.7911	2.188220692	3.35E-48	1.35E-46	up	FOG: Predicted E3 ubiquitin ligase
novel.1842	469	426	474	33	26	31	194.9341	351.425	38.442731	-3.199258885	4.48E-48	1.80E-46	down	--
Solyc10g050990.2	585	746	493	5	7	11	240.4337	471.25	9.61791027	-5.599602923	5.26E-48	2.11E-46	down	--
Solyc09g082550.3	8	9	1	326	222	556	229.2243	4.71059	453.738056	6.611317684	5.61E-48	2.25E-46	up	Sulfate/bicarbonate/oxalate exchanger SAT-1
Solyc07g047850.3	356	258	269	1739	1465	3503	1490.201	227.004	2753.3982	3.60103384	5.98E-48	2.40E-46	up	--
Solyc03g083360.3	319	241	368	999	949	1570	855.176	237.29	1473.06165	2.633961474	7.65E-48	3.05E-46	up	--
Solyc08g016801.1	512	436	511	35	30	33	208.1635	374.325	42.0020941	-3.16543936	8.35E-48	3.32E-46	down	WD40 repeat protein
Solyc02g092670.1	2165	2610	2799	6386	4726	7355	4863.979	1943.9	7784.05509	2.001660673	1.19E-47	4.73E-46	up	--
Solyc04g040180.3	2629	3063	2570	6284	6996	8219	5646.133	2128.08	9164.19081	2.106485821	1.45E-47	5.75E-46	up	Methyltransferase
Solyc04g040190.1	405	312	317	1046	878	1477	845.4429	265.871	1425.01518	2.423219861	1.74E-47	6.87E-46	up	--
Solyc09g066100.3	2372	2374	2925	366	255	438	1205.043	1965.71	444.37079	-2.142688915	2.09E-47	8.24E-46	down	--
Solyc10g085140.2	639	863	840	48	40	72	334.2994	601.81	66.7889507	-3.161881848	2.10E-47	8.26E-46	down	Cis-prenyltransferase
Solyc07g054790.1	598	757	534	5	2	10	247.2231	487.638	6.80877159	-6.094152038	2.51E-47	9.84E-46	down	--
Solyc10g055680.1	125	78	110	506	653	640	427.0005	80.1865	773.814518	3.268684424	2.80E-47	1.10E-45	up	SAM-dependent methyltransferases

Solyc10g075118.1	1	11	4	348	384	293	225.4528	4.17021	446.735409	6.757679718	3.10E-47	1.21E-45	up	--
Solyc02g082450.3	241	184	173	1515	3007	2126	1530.766	153.917	2907.61504	4.23952489	3.46E-47	1.35E-45	up	Predicted membrane protein
Solyc02g087350.3	1667	1177	2273	45	57	99	693.9518	1304.28	83.6274962	-3.957083111	3.65E-47	1.42E-45	down	--
Solyc06g036240.2	18	26	15	284	345	266	202.5517	15.2687	389.834784	4.677564273	4.04E-47	1.57E-45	up	Pleiotropic drug resistance proteins (PDR1-15)
Solyc05g055870.3	989	801	750	2821	4000	4788	2794.05	653.715	4934.38534	2.91621105	4.65E-47	1.80E-45	up	--
Solyc07g008620.1	886	654	836	66	41	53	338.6827	609.138	68.2274927	-3.161354673	4.92E-47	1.90E-45	down	FOG: Leucine rich repeat
Solyc02g091670.1	1687	1471	1954	262	219	285	817.3126	1309.1	325.528007	-2.008630823	5.30E-47	2.05E-45	down	--
Solyc11g008540.3	3860	1853	1950	26593	22402	23122	16491.23	1972.01	31010.4487	3.975018446	7.42E-47	2.85E-45	up	dsRNA-specific nuclease Dicer and related ribonucleases
Solyc07g040960.1	7387	5651	10409	223	277	526	3201.512	5978.38	424.64725	-3.813606811	7.46E-47	2.86E-45	down	Predicted transposase
Solyc10g081560.3	49	49	37	765	378	1330	519.4476	34.8271	1004.06805	4.852222729	8.87E-47	3.39E-45	up	Reductases with broad range of substrate specificities
Solyc03g114160.1	143	104	163	474	436	539	360.8633	104.814	616.912421	2.554813375	1.07E-46	4.06E-45	up	FOG: Armadillo/beta-catenin-like repeats
Solyc03g093130.3	1747	1104	1430	22	52	60	577.4235	1097.94	56.9114769	-4.271693915	1.30E-46	4.94E-45	down	--
Solyc11g010930.2	210	213	162	1235	1015	2511	1051.175	150.902	1951.44927	3.694211546	1.57E-46	5.97E-45	up	Protein involved in membrane traffic
Solyc03g115590.4	499	475	590	1673	1291	1466	1148.99	400.793	1897.18709	2.242135757	1.81E-46	6.84E-45	up	--
novel.1142	457	441	451	32	28	42	194.8189	346.646	42.9915488	-3.005749458	2.61E-46	9.80E-45	down	--
Solyc07g064250.3	1863	1837	1738	254	226	232	852.4725	1398.81	306.138759	-2.19609665	4.78E-46	1.79E-44	down	COPII vesicle protein
Solyc01g060020.4	7	3	19	263	205	382	180.8775	7.28886	354.466112	5.585318281	6.29E-46	2.35E-44	up	--
novel.1175	680	663	772	88	79	92	326.5536	542.466	110.64145	-2.298909599	6.60E-46	2.45E-44	down	--
Solyc09g005570.3	466	359	504	7	12	14	177.1223	340.214	14.0306145	-4.603771835	7.13E-46	2.65E-44	down	--
Solyc12g036330.1	36	31	33	270	344	277	206.4601	25.6896	387.230671	3.911394008	7.67E-46	2.84E-44	up	Serine/threonine protein kinase
Solyc05g007790.3	51	65	48	317	242	459	233.2724	42.3102	424.234519	3.330736959	9.87E-46	3.65E-44	up	--
novel.1333	550	421	517	19	24	16	203.7603	381.645	25.8754819	-3.913545078	9.95E-46	3.67E-44	down	--
Solyc06g075690.3	1770	882	1603	21	20	55	563.2212	1087.44	39.005747	-4.781360709	9.98E-46	3.67E-44	down	--
Solyc08g079040.1	577	588	656	62	55	85	276.138	467.292	84.9837581	-2.453361209	1.05E-45	3.87E-44	down	Acetylglucosaminyltransferase EXT1/exostosin 1
Solyc08g063040.4	341	411	367	1270	1914	2383	1323.821	287.961	2359.68129	3.034859174	1.14E-45	4.16E-44	up	FOG: Zn-finger
Solyc06g072845.1	34598	44908	34594	3252	3450	5364	17241.6	29419.3	5063.86837	-2.538304165	1.22E-45	4.47E-44	down	--
novel.1750	714	671	806	79	85	104	337.8884	561.765	114.01133	-2.303470974	1.27E-45	4.65E-44	down	FOG: Reverse transcriptase
Solyc08g060920.4	1368	1212	1507	5094	6668	5329	4238.895	1047.56	7430.22786	2.826087424	1.33E-45	4.83E-44	up	Protein involved in vacuolar polyphosphate accumulation
Solyc05g052610.3	858	919	974	124	120	166	439.8886	706.378	173.399443	-2.025007206	1.59E-45	5.79E-44	down	Zuotin and related molecular chaperones (DnaJ superfamily)
Solyc12g057160.1	866	580	844	30	39	56	319.4219	586.247	52.5966954	-3.474431246	1.62E-45	5.89E-44	down	--
Solyc03g116740.3	733	911	860	47	54	90	361.792	643.828	79.7559036	-3.004680281	1.66E-45	6.03E-44	down	--
Solyc02g080640.4	378	403	487	1351	2034	2297	1373.704	324.962	2422.44618	2.897733916	2.32E-45	8.36E-44	up	Phosphoadenosine phosphosulfate reductase
Solyc02g083570.3	50	36	58	358	272	615	275.2404	36.7996	513.681152	3.803012582	2.53E-45	9.09E-44	up	Ubiquitin-protein ligase
Solyc06g073550.3	1780	1743	1846	248	170	322	843.9252	1378.92	308.928511	-2.153152868	2.82E-45	1.01E-43	down	RNA-binding protein LARP/SRO9
Solyc11g068380.1	63	76	78	337	378	669	315.866	55.7303	576.001734	3.371194989	2.85E-45	1.02E-43	up	--
Solyc10g085960.2	88	70	74	355	287	553	278.3644	59.6179	497.110927	3.062510473	2.98E-45	1.06E-43	up	Defense-related protein containing SCP domain
Solyc12g005750.1	37	63	20	771	600	1530	609.6885	31.2595	1188.11751	5.253388147	4.27E-45	1.52E-43	up	--
Solyc02g071200.4	32	23	39	297	207	473	213.7395	24.0041	403.474958	4.070340912	4.44E-45	1.57E-43	up	--
Solyc02g085910.4	344	381	430	11	8	11	154.5021	296.273	12.7312053	-4.541498432	4.84E-45	1.71E-43	down	--
Solyc07g063460.3	871	647	715	46	29	66	315.9714	573.593	58.3493749	-3.281802953	5.52E-45	1.95E-43	down	--
Solyc10g005030.4	55	72	72	364	239	424	240.8449	51.1184	430.571332	3.076368884	7.45E-45	2.61E-43	up	GATA-4/5/6 transcription factors
Solyc01g098110.4	1389	1940	2066	5758	5322	5914	4330.019	1384.34	7275.6988	2.393754048	9.09E-45	3.18E-43	up	--
novel.274	2254	1579	1408	6681	7608	8442	5534.201	1350.07	9718.33141	2.847702047	1.15E-44	4.00E-43	up	--
Solyc11g071740.2	441	387	581	8	6	17	186.3928	360.158	12.6274846	-4.791774536	1.33E-44	4.61E-43	down	Calmodulin and related proteins (EF-Hand superfamily)

Solyc12g009880.1	432	422	437	30	31	39	187.0826	331.675	42.4904396	-2.966645322	1.54E-44	5.32E-43	down	--
Solyc03g031980.4	9	4	13	168	150	256	123.3515	6.59467	240.108389	5.175230715	1.67E-44	5.78E-43	up	--
Solyc12g019710.1	863	739	870	92	100	128	384.9748	634.173	135.777056	-2.224464585	1.70E-44	5.87E-43	down	--
Solyc02g072260.4	241	367	314	984	1013	1427	841.5063	237.327	1445.68552	2.607444538	2.15E-44	7.38E-43	up	--
Solyc12g098910.2	28	22	30	168	182	213	130.2862	20.4865	240.085921	3.546937136	2.30E-44	7.87E-43	up	Serine/threonine protein kinase
novel.913	580	573	539	21	33	48	239.0368	435.262	42.8113461	-3.338685915	2.67E-44	9.11E-43	down	--
Solyc04g077220.3	124	79	162	603	676	1004	526.5633	93.0008	960.125895	3.366234169	3.32E-44	1.13E-42	up	Transcription factor HEX, contains HOX and HALZ domains
Solyc05g054620.4	2607	2416	2770	312	290	511	1231.993	1999.52	464.465509	-2.102640961	4.65E-44	1.58E-42	down	--
Solyc08g006470.4	67	28	20	628	770	766	480.004	29.7113	930.296659	4.970370829	4.74E-44	1.61E-42	up	C2H2-type Zn-finger protein
Solyc04g077650.3	2711	3822	3955	10630	11879	12474	8847.719	2692.32	15003.1188	2.4782757	5.07E-44	1.72E-42	up	Serine carboxypeptidases (lysosomal cathepsin A)
Solyc01g090790.4	198	186	232	1113	967	2446	1004.393	157.854	1850.93254	3.552082177	5.14E-44	1.74E-42	up	--
Solyc06g060760.3	13	22	26	169	162	225	125.3685	15.6199	235.117026	3.910028673	5.44E-44	1.83E-42	up	Aquaporin (major intrinsic protein family)
Solyc02g087000.4	971	987	996	2632	2100	4123	2219.76	759.126	3680.39409	2.278024937	5.51E-44	1.85E-42	up	--
Solyc12g094540.1	674	665	705	63	36	81	299.8087	524.944	74.6735437	-2.798857791	6.36E-44	2.13E-42	down	Predicted 3'-5' exonuclease
Solyc03g025710.3	10	16	8	255	232	624	230.5829	8.81432	452.351436	5.690922431	6.67E-44	2.23E-42	up	--
Solyc12g009480.2	270	212	351	2630	3922	2150	2032.075	212.709	3851.44002	4.177694606	7.34E-44	2.45E-42	up	Protein involved in vacuolar polyphosphate accumulation
Solyc06g076040.3	4295	2440	3057	99	138	224	1352.886	2513.37	192.400483	-3.705027395	7.36E-44	2.45E-42	down	FOG: Armadillo/beta-catenin-like repeats
Solyc09g008780.4	2674	4130	4087	12603	15993	17809	11309.42	2797.19	19821.6592	2.825005726	1.07E-43	3.54E-42	up	--
Solyc01g095510.3	160	151	168	566	606	552	433.9793	122.948	745.010875	2.597094507	1.12E-43	3.71E-42	up	Mitochondrial carrier protein CGI-69
Solyc09g011470.3	1381	1987	1946	6176	9039	11549	6347.259	1365.22	11329.3009	3.052869478	1.33E-43	4.40E-42	up	--
Solyc05g009500.3	133	70	118	526	470	771	411.4593	82.0987	740.819929	3.173176803	1.37E-43	4.51E-42	up	H+/oligopeptide symporter
Solyc09g074210.3	1184	1004	1212	134	79	106	503.9122	871.962	135.862919	-2.683517038	1.50E-43	4.95E-42	down	--
novel.1374	1299	947	1377	68	73	131	520.182	927.193	113.171216	-3.027250644	1.60E-43	5.25E-42	down	--
Solyc04g081930.3	1183	1062	1173	109	106	84	503.8202	877.527	130.113159	-2.764417895	1.80E-43	5.89E-42	down	Prolyl 4-hydroxylase alpha subunit
Solyc05g052240.3	869	2047	1612	11000	10512	17023	8660.307	1166.44	16154.178	3.791810973	1.84E-43	6.02E-42	up	--
Solyc06g076490.3	196	246	261	577	524	752	481.5236	180.44	782.606689	2.117211468	1.96E-43	6.42E-42	up	--
novel.562	439	403	445	18	29	26	180.9643	330.402	31.5267559	-3.407250604	2.41E-43	7.85E-42	down	--
Solyc09g083390.3	19	19	22	159	154	177	112.365	15.3893	209.340714	3.76273761	2.55E-43	8.29E-42	up	--
Solyc12g055750.3	953	780	901	94	105	110	404.2702	676.003	132.537532	-2.35715568	2.61E-43	8.49E-42	down	Ca2+/H+ antiporter VCX1 and related proteins
Solyc12g006380.2	72	36	16	873	746	1019	575.0309	32.1481	1117.91378	5.122847093	3.94E-43	1.27E-41	up	Iron/ascorbate family oxidoreductases
Solyc08g016720.1	259	247	263	754	512	782	531.295	197.501	865.089248	2.131669606	4.03E-43	1.30E-41	up	Beta, beta-carotene 15,15'-dioxygenase and related enzymes
Solyc04g063240.3	419	358	359	985	743	1260	772.6639	292.075	1253.25315	2.102549314	4.22E-43	1.36E-41	up	--
Solyc07g007420.3	2435	2110	2357	280	151	232	1026.367	1771.89	280.83972	-2.656874742	4.60E-43	1.48E-41	down	Leucine rich repeat proteins, some proteins contain F-box
Solyc10g081930.1	111	71	85	494	440	432	328.5853	68.5445	588.626114	3.10065629	6.72E-43	2.16E-41	up	--
Solyc12g042600.2	1	2	0	1631	1966	2241	1246.026	0.79081	2491.26115	11.65816502	6.86E-43	2.20E-41	up	UDP-glucuronosyl and UDP-glucosyl transferase
Solyc08g079700.3	1713	1842	2190	70	117	172	811.5725	1472.65	150.499834	-3.288157043	7.61E-43	2.42E-41	down	Predicted Zn-finger protein
Solyc03g026280.3	625	497	533	2	6	3	215.0561	425.211	4.90165357	-6.50443022	1.03E-42	3.26E-41	down	--
Solyc07g062730.1	511	518	680	17	33	35	236.914	437.503	36.3246888	-3.597417392	1.59E-42	5.03E-41	down	--
Solyc06g082160.4	502	537	587	54	45	46	239.854	417.324	62.3838562	-2.752790261	1.94E-42	6.12E-41	down	Predicted integral membrane protein
Solyc09g092480.1	87	95	159	521	594	871	461.3231	86.9443	835.701878	3.263224649	1.99E-42	6.25E-41	up	UDP-glucuronosyl and UDP-glucosyl transferase
Solyc02g085830.4	350	254	274	882	1134	1276	815.4152	225.619	1405.21135	2.638553738	2.36E-42	7.39E-41	up	Spindle pole body protein
Solyc01g005690.3	1335	1053	1162	155	135	149	549.9265	911.755	188.098142	-2.281390918	2.43E-42	7.59E-41	down	--
novel.378	983	1032	913	138	106	139	458.2995	753.76	162.839046	-2.211566342	2.64E-42	8.25E-41	down	Tam3-transposase (Ac family)

Solyc04g007660.1	432	467	454	27	29	46	195.292	347.833	42.7511107	-3.014548928	2.87E-42	8.92E-41	down	--
Solyc01g099035.1	33	55	52	348	382	322	246.1512	35.9775	456.324873	3.663259565	3.28E-42	1.02E-40	up	--
Solyc04g005040.1	644	368	542	7	5	13	204.1348	398.026	10.243577	-5.244557731	4.45E-42	1.38E-40	down	Gelatinase A and related matrix metalloproteases
Solyc10g075110.2	135	200	247	1059	1476	2592	1139.18	148.971	2129.38986	3.837250442	5.06E-42	1.56E-40	up	--
Solyc08g076790.3	222	462	571	3068	3001	4286	2346.008	320.979	4371.03579	3.767224782	5.28E-42	1.63E-40	up	Flavonol reductase/cinnamoyl-CoA reductase
Solyc01g104910.3	4454	6236	4724	15429	12212	18151	11654.27	3974.92	19333.6304	2.28219594	5.51E-42	1.70E-40	up	--
Solyc05g008060.4	664	535	559	1894	1811	3587	1736.397	451.815	3020.97919	2.74174956	5.52E-42	1.70E-40	up	--
Solyc09g007500.3	1574	1820	1331	141	124	207	708.4972	1219.26	197.730951	-2.620616373	1.17E-41	3.56E-40	down	--
Solyc01g111400.4	1779	1916	2123	5581	4892	10037	4991.664	1492.91	8490.41981	2.5078952	1.20E-41	3.67E-40	up	--
Solyc01g106290.3	668	608	580	42	43	75	272.0832	477.442	66.7246286	-2.82803884	1.25E-41	3.82E-40	down	--
Solyc01g095090.3	142	178	173	444	524	635	404.2521	126.704	681.799852	2.427710735	1.29E-41	3.94E-40	up	Predicted E3 ubiquitin ligase
Solyc02g090110.3	837	778	1112	76	88	107	406.3405	697.422	115.259181	-2.59928941	2.52E-41	7.64E-40	down	Serine/threonine protein kinase
Solyc04g082220.3	63	45	57	333	212	429	223.972	42.3114	405.632539	3.262740384	2.63E-41	7.96E-40	up	Amino acid transporters
Solyc12g056940.2	2718	3945	4844	14255	15986	16771	11554.18	2945.8	20162.5677	2.774876922	2.89E-41	8.71E-40	up	Acetyl-CoA carboxylase
Solyc03g026370.1	471	272	260	2219	2194	4833	2031.35	258.269	3804.43106	3.881153578	3.42E-41	1.03E-39	up	--
Solyc02g089540.3	256	336	249	1047	975	1866	915.791	216.953	1614.62908	2.897137441	3.57E-41	1.07E-39	up	GATA-4/5/6 transcription factors
Solyc07g039340.4	482	547	721	1516	1372	1662	1193.677	447.812	1939.5424	2.114023982	5.26E-41	1.58E-39	up	Serine/threonine protein kinase
Solyc05g014230.3	73	85	78	291	254	294	209.6586	60.7121	358.605098	2.561684369	5.99E-41	1.79E-39	up	Predicted E3 ubiquitin ligase
Solyc01g091700.4	56	62	69	331	279	279	215.4183	47.978	382.858593	2.993596077	6.04E-41	1.80E-39	up	--
Solyc01g086920.3	827	549	804	51	26	40	303.8262	558.057	49.5952944	-3.491355219	7.04E-41	2.09E-39	down	FOG: Leucine rich repeat
Solyc02g084890.3	764	509	758	14	32	31	276.4255	519.785	33.0659993	-3.984288815	9.13E-41	2.71E-39	down	Apoptotic ATPase
Solyc12g019410.3	90	99	116	315	379	532	297.5704	78.1943	516.946404	2.724830448	1.18E-40	3.50E-39	up	Tyrosine kinase specific for activated (GTP-bound) p21cdc42Hs
Solyc06g010030.4	631	453	836	2663	2519	2596	1916.04	489.683	3342.3967	2.770247853	1.28E-40	3.78E-39	up	--
Solyc08g007470.2	514	425	544	43	29	52	215.9897	380.044	51.9357167	-2.861764183	1.36E-40	4.00E-39	down	--
Solyc06g069370.4	173	163	241	524	468	668	424.4589	147.483	701.434892	2.248689853	1.46E-40	4.28E-39	up	--
Solyc12g049390.2	780	830	783	99	57	99	361.3619	615.477	107.246607	-2.515708754	1.47E-40	4.30E-39	down	Predicted membrane protein
Solyc05g009520.2	64	80	99	364	577	702	379.6812	62.2252	697.137144	3.484845113	1.48E-40	4.32E-39	up	--
Solyc02g086970.4	1278	1043	1249	2876	4045	4711	2934.079	915.772	4952.3864	2.434924232	1.61E-40	4.68E-39	up	Aldehyde dehydrogenase
Solyc06g054270.3	57	58	101	413	430	414	298.4014	55.0539	541.748901	3.294331642	1.63E-40	4.74E-39	up	Predicted transporter (major facilitator superfamily)
Solyc06g036310.3	937	765	732	29	20	63	335.8348	626.25	45.420009	-3.760426465	2.07E-40	6.01E-39	down	--
Solyc10g084880.3	1395	1368	3062	33	60	59	772.1486	1479.03	65.2699805	-4.506587376	2.31E-40	6.69E-39	down	--
Solyc11g069800.1	2535	2427	2103	5702	7273	7620	5323.17	1819.54	8826.79757	2.278283982	2.79E-40	8.05E-39	up	--
Solyc09g007520.3	127	100	241	1376	1248	3010	1213.849	118.82	2308.87811	4.27935654	3.07E-40	8.85E-39	up	--
Solyc03g117660.4	209	224	258	586	604	628	478.8437	177.213	780.474573	2.136821889	3.17E-40	9.13E-39	up	--
Solyc12g009240.1	785	733	1626	6	14	26	408.7657	798.588	18.943824	-5.380699043	3.21E-40	9.21E-39	down	--
Solyc10g055470.2	6	0	3	282	339	260	193.0657	2.29141	383.839955	7.375295272	3.28E-40	9.42E-39	up	Beta-galactosidase
Solyc11g069600.2	284	316	409	788	715	1026	663.2063	258.284	1068.12887	2.047728305	3.79E-40	1.08E-38	up	--
Solyc03g083400.3	138	143	130	357	371	559	323.5059	105.768	541.243808	2.357526424	3.98E-40	1.14E-38	up	--
Solyc11g007590.3	983	1065	904	3848	6681	5464	3850.555	760.322	6940.78798	3.190364898	4.04E-40	1.15E-38	up	OTU (ovarian tumor)-like cysteine protease
novel.574	364	342	359	10	15	24	147.02	273.587	20.4535535	-3.726167219	4.53E-40	1.29E-38	down	FOG: PPR repeat
Solyc09g092520.3	655	358	509	14	10	11	202.5529	390.085	15.0212769	-4.709559846	4.87E-40	1.38E-38	down	--
Solyc09g015040.1	2444	2193	3171	381	351	426	1245.14	1996.78	493.498092	-2.017746449	5.33E-40	1.51E-38	down	--
Solyc09g009080.4	779	706	843	119	86	117	366.8707	597.008	136.73301	-2.127126189	5.56E-40	1.57E-38	down	--
Solyc02g031710.3	342	354	511	1417	2267	2726	1515.043	308.522	2721.56328	3.140547894	7.06E-40	1.99E-38	up	Predicted E3 ubiquitin ligase
Solyc04g051490.3	3437	2529	2074	14910	9749	12676	8990.115	2072.93	15907.297	2.939991076	7.53E-40	2.12E-38	up	--
Solyc03g115220.4	1750	2768	3350	10773	8872	12925	7886.995	2014.26	13759.7357	2.772101589	1.63E-39	4.59E-38	up	Cytochrome P450 CYP2 subfamily
Solyc01g102950.3	255	272	360	728	710	1104	647.4348	227.006	1067.86322	2.233948685	1.73E-39	4.86E-38	up	--

Solyc06g075520.3	45	42	63	331	274	656	278.0615	38.3334	517.78956	3.755899393	1.83E-39	5.12E-38	up	Intracellular Cl- channel CLIC, contains GST domain
Solyc07g006220.2	9544	8763	11433	332	330	883	4122.459	7616.59	628.325057	-3.597440918	2.00E-39	5.60E-38	down	UDP-glucose 4-epimerase/UDP-sulfoquinovose synthase
Solyc06g007620.3	2887	2427	2440	335	304	513	1237.862	1993.62	482.099575	-2.045402968	2.99E-39	8.33E-38	down	--
Solyc09g092360.3	581	617	636	84	76	95	289.8172	471.116	108.518214	-2.120649001	3.06E-39	8.49E-38	down	--
Solyc10g083990.2	363	437	430	11	8	26	167.142	316.087	18.1970825	-4.073434342	3.22E-39	8.93E-38	down	--
Solyc01g112105.1	1500	1922	1592	104	147	195	739.9761	1291.53	188.420619	-2.776395704	3.28E-39	9.09E-38	down	--
Solyc04g080380.3	826	912	1167	2476	2057	3697	2089.837	743.769	3435.90537	2.207961656	5.21E-39	1.43E-37	up	--
Solyc02g063380.1	14	13	8	127	151	212	107.8412	9.05126	206.631069	4.52055294	6.03E-39	1.66E-37	up	--
Solyc09g064940.2	12929	13051	10033	34595	28794	33222	25302.29	9288.41	41316.1728	2.15321246	6.47E-39	1.78E-37	up	Predicted PhzC/PhzF-type epimerase
Solyc06g071620.3	169	149	190	428	376	449	332.4115	130.163	534.660212	2.036064653	6.61E-39	1.81E-37	up	RNA helicase BRR2, DEAD-box superfamily
Solyc08g005120.3	63	89	103	334	412	599	315.5761	65.3459	565.806413	3.114013286	8.17E-39	2.23E-37	up	Flavonol reductase/cinnamoyl-CoA reductase
Solyc11g012680.3	174	128	198	823	721	1725	734.4607	127.84	1341.08112	3.391206901	8.66E-39	2.36E-37	up	--
Solyc10g086220.2	537	523	600	1565	1357	2806	1398.181	425.858	2370.50455	2.47737734	9.36E-39	2.55E-37	up	NADH:flavin oxidoreductase/12-oxophytodienoate reductase
Solyc02g083670.4	146	130	174	497	439	858	429.9483	115.216	744.680249	2.693232757	1.04E-38	2.82E-37	up	Sensory transduction histidine kinase
Solyc12g011450.2	98	82	112	473	371	873	390.5785	74.7568	706.400186	3.241216673	1.07E-38	2.89E-37	up	--
novel.512	442	304	367	9	6	8	147.7081	285.631	9.78534924	-4.871054605	1.08E-38	2.93E-37	down	--
Solyc07g040680.3	721	883	1079	2927	2346	4761	2424.7	687.211	4162.18794	2.598718059	1.11E-38	3.01E-37	up	Heat shock transcription factor
Solyc04g074410.2	2196	1260	1891	45	88	106	735.2462	1369.16	101.335669	-3.756751265	1.12E-38	3.02E-37	down	--
Solyc09g007340.4	418	380	508	36	23	37	187.4199	334.365	40.4744066	-3.041647244	1.16E-38	3.11E-37	down	--
Solyc06g008150.3	594	579	579	76	73	91	276.2136	450.333	102.094671	-2.143339026	1.41E-38	3.79E-37	down	--
Solyc07g056670.3	3834	2549	3993	9144	9228	10637	7520.586	2653.61	12387.5633	2.222734204	1.50E-38	4.01E-37	up	Iron/ascorbate family oxidoreductases
Solyc01g010640.3	992	1261	1065	2376	2731	3379	2230.143	854.445	3605.84156	2.07739542	1.96E-38	5.23E-37	up	--
Solyc01g094810.3	1114	998	979	2188	2212	3697	2090.296	794.867	3385.72393	2.091163475	1.98E-38	5.26E-37	up	Ubiquitin-protein ligase
Solyc10g076930.2	534	580	593	38	54	42	248.4249	438.521	58.3285782	-2.928329252	2.00E-38	5.30E-37	down	--
Solyc09g007910.4	1302	2017	3029	12023	9719	13724	8315.625	1619.49	15011.7635	3.212393575	2.56E-38	6.78E-37	up	Phenylalanine and histidine ammonia-lyase
Solyc09g056340.3	872	996	1496	72	67	112	482.0554	859.047	105.063639	-3.026920012	2.66E-38	7.02E-37	down	GTPase Rab11/YPT3, small G protein superfamily
Solyc11g012260.3	705	1079	946	77	67	93	401.3917	702.456	100.327149	-2.806844055	2.91E-38	7.68E-37	down	--
Solyc10g084640.1	735	787	877	93	85	144	375.1278	615.545	134.710476	-2.184963722	3.57E-38	9.40E-37	down	Diamine acetyltransferase
Solyc03g044625.1	77	83	83	235	211	313	191.2282	62.4465	320.009827	2.359504058	4.62E-38	1.21E-36	up	--
Solyc06g036290.3	728	760	861	9283	11491	4787	6012.886	602.61	11423.161	4.244467268	4.89E-38	1.28E-36	up	Molecular chaperone (HSP90 family)
Solyc01g090160.4	297	376	330	9	7	9	134.3955	258.152	10.6386904	-4.60457804	5.08E-38	1.33E-36	down	Iron/ascorbate family oxidoreductases
Solyc03g113420.4	765	747	963	2786	4107	3299	2531.331	633.847	4428.81484	2.804272371	5.08E-38	1.33E-36	up	H+/oligopeptide symporter
Solyc03g118510.3	291	307	394	10	9	14	133.9412	254.006	13.8759392	-4.185610279	5.11E-38	1.33E-36	down	--
novel.124	645	534	559	62	56	58	261.1332	446.632	75.6341286	-2.570707316	5.23E-38	1.36E-36	down	--
Solyc03g123830.4	2615	2932	2245	370	338	442	1248.86	2009.56	488.160719	-2.041546513	6.65E-38	1.73E-36	down	D-3-phosphoglycerate dehydrogenase
Solyc03g120980.3	3380	5936	7182	29912	30753	29782	21597.4	4222.58	38972.215	3.206213687	7.57E-38	1.96E-36	up	Pleiotropic drug resistance proteins (PDR1-15), ABC superfamily
Solyc08g006330.3	208	438	460	2162	2403	2562	1668.895	283.634	3054.15639	3.428461427	8.84E-38	2.27E-36	up	UDP-glucuronosyl and UDP-glucosyl transferase
Solyc02g079750.4	1111	1608	1821	4372	3784	5183	3407.421	1163.74	5651.1004	2.279707105	9.25E-38	2.38E-36	up	1,4-benzoquinone reductase-like
Solyc05g051200.1	174	148	229	567	551	957	503.4653	140.796	866.134734	2.620915682	9.34E-38	2.39E-36	up	--
Solyc06g076580.1	10	8	4	297	237	168	156.3524	5.70094	307.003936	5.757208742	1.04E-37	2.65E-36	up	--
Solyc05g014240.4	318	238	327	793	647	1160	656.0157	226.136	1085.89577	2.264248269	1.11E-37	2.83E-36	up	--
Solyc04g079350.1	299	371	394	937	1321	1517	940.811	273.102	1608.51982	2.557916561	1.77E-37	4.50E-36	up	--
Solyc02g086452.1	224	156	208	728	478	893	514.1151	150.69	877.540296	2.542549356	2.02E-37	5.12E-36	up	--
Solyc07g007700.3	582	661	681	69	75	71	293.4431	494.163	92.7229968	-2.424488475	2.37E-37	5.98E-36	down	Farnesyl cysteine-carboxyl methyltransferase

Solyc01g044365.1	16	11	8	162	142	148	101.6274	9.03672	194.218132	4.428559475	2.59E-37	6.54E-36	up	--
Solyc12g006705.1	133	130	139	337	285	358	260.218	103.232	417.203588	2.01388755	3.47E-37	8.74E-36	up	--
Solyc03g121440.4	249	221	285	529	588	818	505.1847	193.415	816.954543	2.078325363	3.64E-37	9.13E-36	up	Serine/threonine protein kinase
Solyc05g024230.3	314	296	399	11	5	6	133.8527	258.263	9.44239813	-4.787340272	3.99E-37	1.00E-35	down	--
Solyc01g106690.3	1122	850	878	3572	5745	4563	3383.392	732.69	6034.09444	3.041709797	4.10E-37	1.03E-35	up	--
Solyc10g084170.1	56	50	50	513	582	334	335.3816	40.1062	630.656955	3.973574362	4.70E-37	1.17E-35	up	--
Solyc09g015457.1	295	271	317	9	12	13	120.5206	226.5	14.5410045	-3.971557526	4.81E-37	1.20E-35	down	--
Solyc06g082570.3	67	40	51	272	229	262	183.4745	40.5385	326.410398	3.007434897	6.49E-37	1.61E-35	up	--
Solyc03g032040.3	2804	3042	3509	456	294	566	1474.242	2399.04	549.447152	-2.123933531	7.54E-37	1.87E-35	down	Predicted transporter (major facilitator superfamily)
Solyc04g076010.3	5030	4285	3656	13232	10804	21720	11163.33	3341.91	18984.7562	2.506209506	8.61E-37	2.12E-35	up	--
Solyc02g081150.1	196	161	221	517	408	527	382.8168	147.977	617.656948	2.059918726	9.28E-37	2.29E-35	up	--
Solyc03g114340.3	1510	2035	1941	4096	3543	5812	3525.941	1410.13	5641.74615	2.000548721	1.01E-36	2.49E-35	up	--
Solyc10g076380.2	31	9	14	771	377	360	333.3036	13.8638	652.743384	5.556548147	1.07E-36	2.63E-35	up	--
Solyc08g082870.3	427	629	688	2434	3895	3202	2291.558	447.267	4135.84995	3.208683011	1.08E-36	2.64E-35	up	Dehydrogenases with different specificities
Solyc03g114360.4	294	200	296	822	1096	1076	744.8455	202.181	1287.50946	2.669620419	1.12E-36	2.74E-35	up	--
Solyc01g108570.3	62	89	58	310	425	601	308.1991	54.0041	562.394156	3.383085625	1.18E-36	2.89E-35	up	Arylacetamide deacetylase
Solyc04g011440.4	991	1084	1481	4950	5412	4171	3620.356	909.555	6331.15743	2.798889321	1.40E-36	3.42E-35	up	Molecular chaperones HSP70/HSC70, HSP70 superfamily
Solyc08g076780.2	41	71	105	447	455	537	334.5107	55.3573	613.66412	3.468126572	1.46E-36	3.55E-35	up	Flavonol reductase/cinnamoyl-CoA reductase
Solyc04g050960.3	998	912	1157	149	104	187	484.9878	785.812	184.163254	-2.087615126	1.49E-36	3.64E-35	down	Predicted helicase
Solyc02g085710.4	77	90	94	241	252	307	203.7563	67.0179	340.494746	2.343974038	2.00E-36	4.87E-35	up	Geranylgeranyl pyrophosphate synthase/Polyprenyl synthetase
Solyc02g070800.2	2254	2635	2834	375	222	310	1183.866	1982.2	385.529827	-2.362354758	2.43E-36	5.89E-35	down	--
Solyc11g032190.1	304	272	317	7	13	11	121.2607	229.095	13.4263884	-4.117315434	2.56E-36	6.21E-35	down	--
Solyc02g092140.1	195	257	325	767	868	977	657.3841	198.87	1115.89771	2.487223058	3.15E-36	7.61E-35	up	--
Solyc02g082770.3	1136	764	910	82	93	100	419.5465	721.316	117.77753	-2.619074379	3.65E-36	8.81E-35	down	Putative phosphoinositide phosphatase
novel.1687	69	76	69	255	189	319	187.6267	55.0662	320.18711	2.54337472	4.67E-36	1.12E-34	up	--
Solyc11g066040.2	626	602	735	81	80	117	310.1655	503.152	117.178458	-2.099082398	4.74E-36	1.14E-34	down	FOG: Armadillo/beta-catenin-like repeats
Solyc11g068720.2	21	26	33	151	174	279	136.6333	20.4781	252.788539	3.625557421	4.81E-36	1.15E-34	up	--
Solyc12g096710.2	902	875	1386	19	46	21	422.9916	807.529	38.4543272	-4.416958381	4.93E-36	1.18E-34	down	--
Solyc07g052990.1	933	739	885	89	83	145	394.1637	655.98	132.347405	-2.302206724	5.35E-36	1.28E-34	down	--
Solyc06g066770.1	276	296	386	6	8	11	127.8863	245.228	10.5442506	-4.53446274	5.37E-36	1.28E-34	down	FOG: Kelch repeat
Solyc02g068550.2	299	278	346	18	14	20	129.273	236.54	22.0061645	-3.424394853	5.53E-36	1.32E-34	down	--
Solyc06g075960.1	2348	2372	1489	151	134	225	909.4278	1605.3	213.553406	-2.907567365	5.57E-36	1.32E-34	down	Histone H4
Solyc07g049150.2	2000	1509	1386	4003	4566	5647	3650.722	1260.31	6041.13935	2.261103499	5.71E-36	1.35E-34	up	dsRNA-specific nuclease Dicer and related ribonucleases
Solyc03g119480.4	670	592	723	71	76	114	309.3388	508.922	109.755576	-2.208726321	5.80E-36	1.37E-34	down	--
Solyc01g079620.4	517	627	734	1983	1510	3196	1625.804	481.352	2770.25603	2.525256175	6.02E-36	1.43E-34	up	Transcription factor, Myb superfamily
novel.538	414	390	455	35	35	56	187.8826	322.937	52.8278526	-2.60317303	6.16E-36	1.46E-34	down	--
Solyc03g006640.3	460	448	444	1073	1113	2011	1046.937	347.561	1746.31307	2.329881492	6.64E-36	1.57E-34	up	--
novel.878	8	6	7	110	129	152	85.98263	5.39024	166.575028	4.947950163	8.90E-36	2.10E-34	up	FOG: Transposon-encoded proteins with TYA
Solyc01g081570.3	1840	1687	1548	266	203	229	802.6021	1306.16	299.048439	-2.129311158	9.42E-36	2.22E-34	down	--
Solyc05g050800.3	5127	4141	3560	547	455	718	2014.206	3305.05	723.358133	-2.190957221	1.21E-35	2.85E-34	down	Predicted phosphoglycerate mutase
Solyc12g096370.2	442	388	466	46	31	54	193.6571	332.36	54.9545719	-2.58790009	1.22E-35	2.86E-34	down	Exocyst component protein and related proteins
Solyc01g067300.3	1399	801	927	6415	3676	5342	3676.61	803.399	6549.82186	3.027288348	1.87E-35	4.38E-34	up	--
Solyc04g048900.3	3783	2815	2816	473	400	543	1510.812	2421.29	600.33042	-2.011870319	2.13E-35	4.95E-34	down	Calreticulin
Solyc01g109330.3	41	64	78	411	449	353	287.3908	46.8453	527.936256	3.491532877	2.28E-35	5.31E-34	up	--
Solyc02g086960.2	0	2	2	411	449	563	302.7416	1.02463	604.458537	9.200745151	2.32E-35	5.39E-34	up	--
Solyc01g104650.3	3928	5670	3923	257	363	483	1978.431	3490.96	465.89934	-2.905296901	2.56E-35	5.93E-34	down	--

Solyc03g025230.3	392	396	414	45	32	50	181.1458	308.743	53.548563	-2.522515536	2.69E-35	6.22E-34	down	Uncharacterized membrane protein, predicted efflux pump
Solyc12g038420.2	380	295	332	13	4	17	136.2051	258.574	13.8365406	-4.181289471	2.97E-35	6.86E-34	down	--
Solyc06g009110.3	484	338	393	20	22	16	168.641	311.947	25.3349741	-3.647082215	3.31E-35	7.62E-34	down	Aspartyl protease
Solyc06g005960.4	109	168	202	618	515	675	445.3642	122.647	768.081004	2.645932938	3.69E-35	8.48E-34	up	--
novel.2029	333	267	302	12	11	8	122.5584	231.575	13.5422689	-4.128188465	3.91E-35	8.95E-34	down	--
Solyc02g082633.1	572	474	648	49	49	39	246.6515	433.701	59.6019562	-2.87951041	4.23E-35	9.66E-34	down	--
novel.285	6	5	6	144	103	230	100.7583	4.36041	197.156198	5.501620081	4.33E-35	9.89E-34	up	--
Solyc03g026210.4	611	518	527	69	58	80	256.6936	425.697	87.6903838	-2.278530587	5.02E-35	1.14E-33	down	--
Solyc11g008520.3	35	15	9	329	217	347	195.8555	15.2635	376.447593	4.628958761	5.12E-35	1.17E-33	up	dsRNA-specific nuclease Dicer and related ribonucleases
Solyc01g102270.2	187	162	128	686	718	1555	670.377	123.009	1217.74511	3.308944101	5.61E-35	1.27E-33	up	Anaphase-promoting complex (APC), subunit 11
Solyc10g009380.3	775	573	506	20	9	37	252.1116	477.592	26.6308603	-4.131954214	5.61E-35	1.27E-33	down	--
Solyc05g052190.4	553	586	706	79	76	91	288.8691	472.864	104.873692	-2.176500574	5.81E-35	1.32E-33	down	Transcription elongation factor TFIIS/Cofactor
Solyc02g069800.1	1936	1799	2105	214	278	241	907.6612	1497.97	317.348015	-2.243716955	6.45E-35	1.46E-33	down	Arylacetamide deacetylase
Solyc06g060170.3	161	156	168	356	400	584	344.3162	124.537	564.095744	2.180345525	7.14E-35	1.61E-33	up	--
Solyc12g014490.3	571	694	819	42	28	74	296.5548	534.084	59.0259962	-3.159749379	8.65E-35	1.94E-33	down	Microtubule-associated protein
Solyc10g054440.3	6163	4160	7942	19400	18603	22209	15165.75	4657.42	25674.0873	2.462639374	8.67E-35	1.94E-33	up	--
Solyc02g085360.4	214	115	162	613	650	1065	549.9405	125.866	974.015021	2.952288147	9.95E-35	2.23E-33	up	Cytochrome P450 CYP4/CYP19/CYP26 subfamilies
Solyc04g082060.4	559	504	540	78	63	88	254.3528	411.719	96.9867836	-2.08484296	1.10E-34	2.45E-33	down	--
Solyc07g053100.4	539	556	620	80	76	82	271.0559	440.08	102.031557	-2.116171142	1.52E-34	3.38E-33	down	--
Solyc02g070890.3	286	409	421	902	869	1271	784.5314	286.497	1282.56563	2.162759619	1.85E-34	4.11E-33	up	--
Solyc05g053860.4	2095	2643	1711	148	126	89	912.6892	1666.61	158.772352	-3.397497448	2.17E-34	4.80E-33	down	Synaptic vesicle transporter SVOP
Solyc05g055420.4	459	418	451	43	50	61	203.2641	341.045	65.4831798	-2.383324851	2.33E-34	5.13E-33	down	Acetylglucosaminyltransferase EXT2/exostosin 2
Solyc11g012980.1	687	488	449	9	12	31	219.6355	418.171	21.1000572	-4.277534177	2.64E-34	5.82E-33	down	--
Solyc01g111340.4	287	549	776	3162	4850	5499	3084.829	411.432	5758.2252	3.806645316	3.43E-34	7.53E-33	up	--
Solyc06g066650.4	15	9	22	157	110	183	100.4162	11.694	189.138511	4.008668334	3.48E-34	7.65E-33	up	--
Solyc12g014120.3	746	838	790	88	62	131	363.5361	610.532	116.540592	-2.378415478	3.64E-34	7.98E-33	down	--
novel.1527	452	387	390	0	6	3	159.9951	315.963	4.02687168	-6.365671892	3.86E-34	8.44E-33	down	--
Solyc10g006510.3	347	320	302	13	16	8	132.8605	249.297	16.4244067	-3.969256699	4.00E-34	8.72E-33	down	--
Solyc12g011310.2	4182	3236	3757	18569	17097	12122	11883.47	2868.29	20898.6374	2.865075977	4.67E-34	1.02E-32	up	Glutathione S-transferase
Solyc04g079590.3	652	663	741	84	74	132	324.3042	527.586	121.022812	-2.115331232	4.99E-34	1.09E-32	down	--
Solyc11g071850.1	512	605	590	74	67	90	268.3311	438.74	97.921801	-2.16341187	6.18E-34	1.34E-32	down	--
Solyc09g007680.4	1748	1873	2269	4257	4121	7515	4062.372	1509.41	6615.33809	2.131990673	7.05E-34	1.52E-32	up	Protein involved in mRNA turnover and stability
Solyc01g097240.3	1215	712	1231	3084	3640	4358	2761.846	806.983	4716.70891	2.546904004	7.45E-34	1.60E-32	up	Predicted chitinase
Solyc07g055640.1	395	285	533	9	6	17	161.1818	309.299	13.0648755	-4.530554915	8.01E-34	1.72E-32	down	--
Solyc11g010710.2	249	551	540	2248	2517	2999	1825.379	344.007	3306.75146	3.264848011	8.10E-34	1.74E-32	up	--
Solyc02g062130.4	497	626	696	65	38	64	268.4419	466.552	70.3315635	-2.725492368	9.12E-34	1.95E-32	down	NADP/FAD dependent oxidoreductase
Solyc03g059160.3	569	511	642	1318	1215	2314	1227.524	441.29	2013.75741	2.190656198	9.48E-34	2.03E-32	up	--
Solyc07g021620.1	404	379	419	49	39	48	183.2746	308.557	57.9919888	-2.416093075	1.04E-33	2.22E-32	down	FOG: PPR repeat
Solyc11g006740.3	588	529	537	4481	3624	2235	2485.726	425.135	4546.31705	3.418514334	1.28E-33	2.72E-32	up	--
novel.289	749	1057	796	2644	2137	2506	1892.779	671.046	3114.51235	2.214643675	1.40E-33	2.97E-32	up	--
Solyc06g063280.1	101	166	151	434	454	646	377.3462	107.485	647.207796	2.590930731	1.41E-33	2.98E-32	up	--
novel.1568	526	437	433	50	42	51	209.9963	359.003	60.9894033	-2.56122949	1.45E-33	3.07E-32	down	--
Solyc11g071830.2	694	734	721	3098	2712	2098	1998.989	552.416	3445.56187	2.640580747	1.95E-33	4.10E-32	up	Molecular chaperone (DnaJ superfamily)
Solyc08g078150.1	596	486	597	66	60	94	261.5002	430.543	92.4575951	-2.213894012	2.02E-33	4.24E-32	down	FOG: PPR repeat

Solyc04g074450.1	4968	3029	4655	83	185	268	1731.096	3237.78	224.416088	-3.849776622	2.60E-33	5.43E-32	down	--
Solyc02g092360.3	554	599	631	76	85	91	283.036	458.11	107.962063	-2.091897353	2.69E-33	5.62E-32	down	--
Solyc03g111710.3	709	551	567	56	24	50	262.0669	469.685	54.4482684	-3.100902467	2.85E-33	5.95E-32	down	CREB binding protein/P300 and related TAZ Zn-finger proteins
Solyc01g091590.3	840	565	873	15	37	48	312.4071	582.671	42.142798	-3.787583178	2.99E-33	6.24E-32	down	--
novel.1298	4	4	2	121	140	203	98.97023	2.59171	195.348754	6.248923173	3.20E-33	6.65E-32	up	--
Solyc11g045225.1	966	754	733	1760	2065	2325	1628.886	631.073	2626.69948	2.057252817	3.20E-33	6.65E-32	up	FOG: Ankyrin repeat
Solyc01g089950.3	266	294	267	13	13	13	114.79	212.8	16.7795177	-3.679826617	3.29E-33	6.83E-32	down	--
Solyc04g009800.3	3279	2729	3420	262	355	253	1398.562	2416.76	380.364583	-2.671618996	3.67E-33	7.62E-32	down	Ca2+/calmodulin-dependent protein kinase
Solyc02g068000.2	503	465	462	64	53	72	223.8932	367.643	80.1435477	-2.197413816	3.91E-33	8.11E-32	down	--
Solyc06g048590.3	346	393	408	39	35	45	172.5668	294.564	50.5691064	-2.544071493	5.02E-33	1.03E-31	down	Predicted membrane protein
Solyc08g014130.3	2334	3236	4080	12430	23520	20311	13403.84	2469.66	24338.0208	3.300770184	5.18E-33	1.07E-31	up	Alpha-isopropylmalate synthase/homocitrate synthase
Solyc08g008670.3	343	353	375	38	30	33	159.1669	275.02	43.3142669	-2.676052516	5.42E-33	1.11E-31	down	Phosphomannomutase
Solyc12g011230.1	367	278	354	8	18	11	136.2071	256.106	16.3085262	-4.011124259	6.15E-33	1.26E-31	down	--
Solyc10g081430.2	61	56	56	212	158	227	148.5858	44.4739	252.697823	2.507789308	7.20E-33	1.47E-31	up	Acetylglucosaminyltransferase EXT1/exostosin 1
Solyc07g055050.3	863	912	1028	140	79	115	430.4379	719.109	141.766791	-2.34260998	8.85E-33	1.80E-31	down	--
Solyc02g062380.3	7	15	10	106	135	175	92.20238	8.2646	176.140173	4.419237005	9.18E-33	1.87E-31	up	GATA-4/5/6 transcription factors
Solyc10g051030.2	345	265	376	745	946	1287	754.8738	252.373	1257.37462	2.316442527	1.00E-32	2.03E-31	up	--
Solyc11g008590.2	495	483	435	55	47	68	217.7636	363.711	71.8157657	-2.337440696	1.12E-32	2.26E-31	down	Predicted UDP-galactose transporter
Solyc11g013110.2	6182	9823	10992	27618	23195	35230	21589.3	6920.03	36258.5674	2.389473961	1.22E-32	2.47E-31	up	Iron/ascorbate family oxidoreductases
Solyc01g111010.4	41	48	31	227	159	323	162.8713	31.0133	294.72925	3.254868394	1.36E-32	2.75E-31	up	--
Solyc08g082090.1	1839	1433	2051	92	129	222	773.2094	1362.21	184.209418	-2.882795779	1.67E-32	3.37E-31	down	--
Solyc03g031650.4	273	285	227	812	874	1739	809.2733	202.366	1416.18056	2.808268019	1.74E-32	3.50E-31	up	Microtubule-associated anchor protein
Solyc10g008040.4	22	40	39	264	267	702	263.8814	25.939	501.823744	4.275913594	1.94E-32	3.89E-31	up	--
Solyc05g014250.3	257	257	330	784	779	1564	754.9298	216.145	1293.71486	2.582077314	2.43E-32	4.86E-31	up	Predicted E3 ubiquitin ligase
Solyc08g067270.3	187	164	164	525	464	982	473.3714	132.407	814.335515	2.622613862	2.59E-32	5.18E-31	up	--
Solyc07g055290.3	630	605	610	1479	1410	2836	1421.967	474.2	2369.735	2.321890851	2.64E-32	5.28E-31	up	GTPase Rab11/YPT3, small G protein superfamily
Solyc08g081010.3	2852	3562	4119	8552	11472	10945	8019.045	2700.03	13338.063	2.304396145	2.65E-32	5.28E-31	up	--
Solyc12g088460.3	174	274	331	964	1134	1112	790.3769	199.437	1381.31715	2.791148834	2.66E-32	5.30E-31	up	Cytochrome P450 CYP2 subfamily
Solyc07g065540.1	413	314	349	28	22	35	156.0558	276.354	35.7575461	-2.943558917	2.84E-32	5.65E-31	down	--
Solyc08g014440.4	229	187	228	844	652	1692	734.8301	165.156	1304.50389	2.982535535	2.93E-32	5.82E-31	up	CREB binding protein/P300 and related TAZ Zn-finger proteins
Solyc01g009080.4	190	226	258	569	425	745	450.4897	172.828	728.150831	2.076071329	3.15E-32	6.25E-31	up	--
Solyc01g108100.3	471	418	531	51	48	42	212.4671	363.853	61.0809642	-2.58923646	3.32E-32	6.59E-31	down	--
Solyc09g090030.2	566	498	521	72	68	93	252.9418	407.254	98.6291439	-2.044735836	3.46E-32	6.85E-31	down	Predicted histone tail methylase containing SET domain
Solyc11g007840.3	289	333	399	11	16	27	142.0551	261.637	22.4730693	-3.525759235	3.53E-32	6.99E-31	down	--
Solyc05g052050.1	456	571	586	1253	1838	2045	1303.071	414.22	2191.92106	2.403511422	3.55E-32	7.01E-31	up	--
Solyc06g084430.4	703	565	577	50	41	87	273.9694	474.32	73.6185592	-2.674140614	3.80E-32	7.48E-31	down	Histone 2A
Solyc04g074850.3	1807	2780	2640	6227	6581	7127	5197.881	1857.33	8538.42968	2.200674198	4.04E-32	7.93E-31	up	Uncharacterized membrane protein, predicted efflux pump
Solyc08g067340.4	323	219	276	2295	1723	1170	1241.212	209.814	2272.61041	3.436669405	4.09E-32	8.03E-31	up	--
Solyc12g094550.2	313	291	330	25	25	22	135.4276	239.68	31.1751278	-2.961928681	4.21E-32	8.25E-31	down	--
novel.107	318	258	250	10	6	8	111.3573	212.492	10.2227402	-4.382030239	4.41E-32	8.64E-31	down	--
Solyc11g020960.2	1392	1130	1053	123	98	194	546.2754	920.143	172.408136	-2.409562315	4.53E-32	8.88E-31	down	--
Solyc01g150169.1	291	289	316	25	19	31	130.7642	230.007	31.5209579	-2.858921544	4.93E-32	9.65E-31	down	Sexual differentiation process protein ISP4
Solyc09g010980.1	876	429	436	7	15	16	232.2025	448.179	16.2262462	-4.793735185	4.94E-32	9.65E-31	down	--

Solyc08g005610.3	1485	1382	1367	160	97	232	645.265	1088.58	201.949541	-2.423116815	5.38E-32	1.05E-30	down	Cytochrome P450 CYP4/CYP19/CYP26 subfamilies
Solyc05g009820.4	409	280	541	7	1	4	159.2848	313.561	5.00825323	-5.949066001	6.47E-32	1.26E-30	down	--
Solyc09g082690.3	5116	10579	7826	34827	32962	36316	25324.29	6065.56	44583.0167	2.87779074	6.51E-32	1.26E-30	up	--
Solyc01g111050.4	39	35	36	205	126	215	128.9429	28.2689	229.617004	3.024491241	6.59E-32	1.28E-30	up	--
Solyc10g006130.1	2787	2631	3915	331	405	510	1456.967	2385.29	528.640723	-2.174136152	7.40E-32	1.43E-30	down	--
Solyc08g066790.4	550	521	656	68	68	60	263.6683	442.482	84.8546503	-2.394950832	7.55E-32	1.46E-30	down	Phospholipase D1
Solyc10g083210.2	188	243	205	664	438	682	458.4414	163.78	753.102628	2.202373489	7.75E-32	1.50E-30	up	--
Solyc12g044190.3	885	925	1126	81	101	161	447.9379	752.396	143.479635	-2.386151279	8.45E-32	1.63E-30	down	Apoptotic ATPase
Solyc02g089900.1	1166	673	1189	30	54	75	420.2193	773.584	66.8543804	-3.530291518	8.71E-32	1.68E-30	down	--
Solyc04g016230.4	11	8	3	128	238	196	124.7451	5.71341	243.776787	5.423026614	9.65E-32	1.85E-30	up	UDP-glucuronosyl and UDP-glucosyl transferase
Solyc01g106390.3	679	554	910	41	59	73	310.2897	547.198	73.381644	-2.89966912	1.09E-31	2.08E-30	down	--
Solyc12g099210.2	4	0	1	1042	645	2072	763.7174	1.28132	1526.15354	10.21607836	1.26E-31	2.40E-30	up	--
Solyc11g005630.1	732	514	1085	48	40	59	327.7116	593.371	62.0518572	-3.25573336	1.51E-31	2.89E-30	down	--
novel.1221	490	375	413	31	33	23	183.1719	328.268	38.0754603	-3.130458778	1.60E-31	3.05E-30	down	--
Solyc06g074750.1	344	393	546	20	7	27	175.0219	328.035	22.0090434	-3.867003214	1.70E-31	3.23E-30	down	Histone H2B
Solyc01g096230.4	481	706	841	1898	2235	2437	1665.199	519.406	2810.99271	2.435779097	1.93E-31	3.67E-30	up	AAA+-type ATPase
Solyc08g077090.3	396	385	411	30	44	47	178.9374	306.113	51.7619159	-2.571506746	2.05E-31	3.89E-30	down	Predicted haloacid dehalogenase-like hydrolase (eyes absent)
novel.1450	498	365	359	1251	1121	2328	1128.986	314.379	1943.59246	2.628957669	2.16E-31	4.10E-30	up	--
Solyc12g096810.2	354	345	401	40	37	37	165.6052	282.141	49.0692616	-2.535865345	2.52E-31	4.76E-30	down	Ca2+-binding transmembrane protein LETM1/MRS7
Solyc05g052520.3	770	617	1231	50	60	62	370.1809	666.563	73.798802	-3.180350104	2.55E-31	4.81E-30	down	Serine/threonine protein phosphatase
Solyc03g120570.3	467	1085	1038	5734	9348	6599	5074.223	665.127	9483.31999	3.833618419	2.58E-31	4.86E-30	up	H+/oligopeptide symporter
Solyc01g107780.4	44	33	46	163	248	310	168.5046	31.4935	305.51563	3.276480522	2.83E-31	5.33E-30	up	UDP-glucuronosyl and UDP-glucosyl transferase
Solyc08g079480.3	103	112	161	334	373	452	294.6356	96.0995	493.171791	2.357285853	3.60E-31	6.75E-30	up	--
Solyc02g067440.3	389	315	411	3	0	5	144.4069	285.68	3.13413189	-6.404326854	3.77E-31	7.06E-30	down	Uncharacterized conserved protein
Solyc06g011575.1	351	245	420	6	6	2	132.8642	259.442	6.28682553	-5.433414533	3.91E-31	7.33E-30	down	--
Solyc02g088955.1	503	451	580	55	33	73	229.8865	392.981	66.7924335	-2.542996407	3.94E-31	7.36E-30	down	--
Solyc08g068850.3	55	22	14	1438	1791	2615	1240.547	23.5323	2457.56109	6.70769614	4.45E-31	8.31E-30	up	--
Solyc12g009000.1	4591	3163	5925	127	230	368	1895.381	3488.66	302.103193	-3.528312933	4.54E-31	8.47E-30	down	Predicted transposase
Solyc03g119870.3	859	741	922	145	95	130	401.8597	646.477	157.242813	-2.040377693	4.54E-31	8.47E-30	down	Uncharacterized conserved protein
Solyc07g065970.1	254	278	283	2	10	12	109.7583	209.38	10.1369774	-4.365403353	4.81E-31	8.96E-30	down	Molecular chaperone (DnaJ superfamily)
Solyc02g150146.1	1096	857	1169	150	118	205	498.7498	799.495	198.004989	-2.008956559	6.14E-31	1.13E-29	down	--
Solyc05g005865.1	12	14	30	189	267	621	226.8609	14.2181	439.503687	4.945967765	6.25E-31	1.15E-29	up	--
Solyc05g046310.3	667	723	716	2291	2207	1767	1633.163	541.271	2725.05425	2.331359987	6.63E-31	1.22E-29	up	--
Solyc02g089050.4	341	328	320	32	30	31	147.1333	254.305	39.9611376	-2.681045902	7.68E-31	1.41E-29	down	--
Solyc06g060990.4	630	683	623	74	83	119	307.2319	498.151	116.312354	-2.095364566	7.71E-31	1.42E-29	down	Uncharacterized conserved protein
Solyc05g018310.1	3	4	2	116	120	197	91.76471	2.33296	181.196461	6.293744467	7.95E-31	1.46E-29	up	FOG: Predicted E3 ubiquitin ligase
Solyc08g077790.3	1500	1362	1236	230	165	197	653.9696	1054.88	253.061751	-2.061431266	8.23E-31	1.51E-29	down	--
Solyc02g087670.3	3	2	13	148	188	158	109.3702	4.51008	214.230248	5.54367539	9.65E-31	1.76E-29	up	--
Solyc12g006973.1	803	564	975	52	70	76	341.3086	597.953	84.664563	-2.824533989	9.80E-31	1.79E-29	down	--
novel.2017	408	426	425	41	21	53	185.5435	323.573	47.5137316	-2.748619286	9.83E-31	1.79E-29	down	--
Solyc10g086180.2	42	106	198	7897	7831	5828	4747.273	87.8318	9406.71431	6.742215463	9.84E-31	1.79E-29	up	Phenylalanine and histidine ammonia-lyase
Solyc08g067410.2	738	698	925	124	81	129	372.6573	604.467	140.84792	-2.098944401	1.02E-30	1.86E-29	down	--
Solyc06g065190.1	134	83	126	322	293	433	264.8349	87.7861	441.883705	2.331143337	1.40E-30	2.54E-29	up	--
Solyc08g068200.4	767	739	819	133	92	106	369.2765	596.771	141.78187	-2.077842242	1.53E-30	2.76E-29	down	Predicted GTPase
Solyc05g014000.4	7815	4200	4148	309	124	335	2239.481	4161.11	317.854781	-3.708581681	1.94E-30	3.50E-29	down	--

Solyc12g005940.2	30	13	26	207	122	224	124.72	17.6246	231.815515	3.715536576	2.03E-30	3.65E-29	up	Iron/ascorbate family oxidoreductases
Solyc07g065690.3	635	582	497	33	55	46	249.8161	441.544	58.0881401	-2.938749435	2.05E-30	3.69E-29	down	--
Solyc07g049440.3	473	888	952	4285	2793	7058	3202.412	593.091	5811.73321	3.292770127	2.09E-30	3.75E-29	up	--
Solyc02g083860.3	2247	3840	4306	11209	10086	13766	8756.983	2663.49	14850.4762	2.479100632	2.14E-30	3.84E-29	up	Iron/ascorbate family oxidoreductases
Solyc03g013304.2	12	4	10	111	88	140	74.61244	6.63208	142.592793	4.421331293	2.31E-30	4.15E-29	up	--
Solyc01g079750.3	300	287	339	22	25	36	136.2166	237.469	34.9644403	-2.758633849	2.40E-30	4.29E-29	down	MEKK and related serine/threonine protein kinases
novel.515	80	92	81	615	475	350	346.954	65.1245	628.783517	3.27050682	2.76E-30	4.93E-29	up	FOG: Transposon-encoded proteins with TYA
Solyc09g083030.1	2302	1722	1449	108	135	224	802.7501	1410.63	194.870153	-2.852876393	3.25E-30	5.78E-29	down	--
Solyc05g053930.4	811	674	728	87	82	144	349.5359	568.452	130.619282	-2.114244935	3.61E-30	6.40E-29	down	Serine/threonine protein kinase
Solyc08g061250.3	5	4	7	93	114	201	86.87113	4.08191	169.660341	5.373055289	3.89E-30	6.89E-29	up	Serine/threonine protein kinase
Solyc06g051010.1	378	368	572	22	18	41	184.9745	336.585	33.3637538	-3.315324277	4.07E-30	7.21E-29	down	--
Solyc12g094580.3	90	127	151	426	350	690	351.5772	94.2632	608.891173	2.692422151	4.25E-30	7.51E-29	up	--
Solyc04g007900.3	1034	812	908	96	123	105	423.7952	707.199	140.391444	-2.341025506	5.15E-30	9.06E-29	down	--
Solyc04g081300.4	1718	1391	1334	240	154	232	703.9743	1143.14	264.810931	-2.109028123	5.21E-30	9.15E-29	down	--
Solyc07g049495.1	33	16	24	161	116	185	106.6285	18.7063	194.550555	3.378503622	5.85E-30	1.03E-28	up	--
Solyc06g005430.1	553	511	367	25	24	45	204.2439	369.421	39.0671901	-3.228542158	5.90E-30	1.03E-28	down	Histone H4
Solyc03g115230.3	3377	3772	3876	13628	14397	9971	9732.705	2831.89	16633.5187	2.554165626	5.94E-30	1.04E-28	up	Chaperone HSP104 and related ATP-dependent Clp proteases
Solyc12g010750.2	19	8	11	191	97	226	111.538	9.75378	213.322309	4.453195319	6.05E-30	1.06E-28	up	Secretory carrier membrane protein
Solyc12g099950.3	4	0	0	276	230	359	182.515	1.03503	363.994917	8.470213236	7.02E-30	1.22E-28	up	FOG: Leucine rich repeat
Solyc11g006560.3	362	385	358	36	41	44	168.0441	284.262	51.826238	-2.463363073	7.82E-30	1.36E-28	down	--
Solyc03g117860.3	2792	1944	1361	10109	8633	8867	6724.276	1574.81	11873.7472	2.914570148	8.86E-30	1.54E-28	up	Predicted E3 ubiquitin ligase
Solyc12g011023.1	418	270	390	1	6	2	140.0701	276.04	4.09987081	-6.164394131	9.12E-30	1.58E-28	down	--
Solyc03g112010.4	65	74	83	196	177	264	162.7096	56.9472	268.472103	2.238164948	9.38E-30	1.62E-28	up	Cytochrome P450 CYP2 subfamily
Solyc06g072460.1	357	245	451	16	13	11	142.996	268.629	17.3629069	-3.971648334	1.02E-29	1.76E-28	down	--
Solyc01g079530.4	419	356	359	43	27	44	169.7926	291.543	48.0426834	-2.596111391	1.03E-29	1.78E-28	down	Protein involved in mRNA turnover and stability
Solyc10g074470.3	275	232	286	9	17	14	110.3326	203.315	17.3501432	-3.574289484	1.04E-29	1.80E-28	down	--
Solyc07g065340.1	864	1033	858	2737	5167	4321	3003.883	709.688	5298.07745	2.900143799	1.37E-29	2.36E-28	up	Serine O-acetyltransferase
Solyc08g005580.3	1	7	9	104	96	155	76.62304	4.33755	148.908529	5.096543838	1.44E-29	2.47E-28	up	Transporter, ABC superfamily (Breast cancer resistance protein)
novel.155	350	334	351	22	27	46	152.7258	265.865	39.5862572	-2.73423924	2.27E-29	3.86E-28	down	--
Solyc07g055020.3	498	465	551	34	57	64	227.1656	388.269	66.0624824	-2.559196029	2.28E-29	3.87E-28	down	--
novel.375	264	231	256	6	1	6	99.05685	192.814	5.29964591	-5.134962189	2.46E-29	4.18E-28	down	--
Solyc11g067190.3	29	50	50	229	249	551	227.9053	33.1198	422.690809	3.675790091	2.59E-29	4.39E-28	up	Acyl-CoA reductase
Solyc02g089720.3	1301	1198	1380	2833	4864	5498	3308.013	995.222	5620.80448	2.497598086	2.68E-29	4.52E-28	up	Predicted endo-1,3-beta-glucanase
Solyc07g042550.3	7210	5277	6733	18088	18126	15302	13638.94	4927.73	22350.1473	2.181210542	2.92E-29	4.92E-28	up	Glycosyltransferase
Solyc05g011980.4	2298	1940	1501	171	219	207	868.8494	1480.4	257.302869	-2.527466615	2.96E-29	4.98E-28	down	--
Solyc07g064820.1	326	435	724	11	20	17	199.5873	378.39	20.7849487	-4.201532127	3.35E-29	5.62E-28	down	MEKK and related serine/threonine protein kinases
Solyc10g086710.3	585	466	593	15	39	21	227.3364	421.391	33.2821178	-3.689422071	3.43E-29	5.75E-28	down	--
Solyc02g076680.3	275	514	667	2227	3273	3033	2025.886	372.171	3679.6013	3.305165056	3.69E-29	6.17E-28	up	--
Solyc01g150152.1	1285	1555	988	3491	3396	5954	3173.251	989.508	5356.99271	2.437010154	3.79E-29	6.33E-28	up	Stress responsive protein
Solyc09g074280.1	320	237	404	3	3	4	124.794	245.351	4.2365882	-5.855457851	4.10E-29	6.85E-28	down	FOG: Ankyrin repeat
Solyc10g079620.2	124	177	143	669	782	2035	765.4511	114.392	1416.51029	3.631427502	4.16E-29	6.94E-28	up	Predicted hydrolase (HAD superfamily)
Solyc09g010260.4	2	5	6	125	79	155	76.55349	3.32538	149.781599	5.492112286	4.33E-29	7.21E-28	up	--
novel.1033	264	211	236	4	5	7	94.6564	182.568	6.74505333	-4.751759805	4.69E-29	7.80E-28	down	--
Solyc11g013010.2	564	801	816	57	60	40	314.4211	559.998	68.8439187	-3.038236838	4.87E-29	8.07E-28	down	Junctional membrane complex protein Junctophilin

Solyc04g074350.3	482	547	761	34	43	70	259.5335	457.663	61.403542	-2.891091411	5.83E-29	9.63E-28	down	UDP-glucuronosyl and UDP-glucosyl transferase
Solyc12g088670.2	3520	3553	3917	7925	9852	18034	8837.813	2820.73	14854.8944	2.396865851	6.70E-29	1.10E-27	up	Cysteine proteinase Cathepsin L
Solyc12g094570.1	261	233	225	804	605	1519	692.9614	184.935	1200.98785	2.700583871	7.37E-29	1.21E-27	up	--
Solyc01g091630.3	931	1014	1513	3024	2279	3239	2250.27	883.289	3617.25092	2.033711107	7.73E-29	1.27E-27	up	--
Solyc02g084600.4	334	409	445	44	27	35	175.0145	304.828	45.2005481	-2.757061048	7.83E-29	1.29E-27	down	--
Solyc03g096760.1	3	4	2	124	353	224	155.3962	2.33296	308.459372	7.054934968	9.05E-29	1.48E-27	up	--
Solyc05g011890.1	130	152	62	728	598	1284	584.0179	89.3445	1078.69142	3.596127933	9.66E-29	1.58E-27	up	Sulfotransferase
Solyc12g011030.3	355	223	435	4	3	12	132.9538	258.318	7.58911364	-5.028571373	9.87E-29	1.61E-27	down	--
Solyc11g010160.1	2	2	6	135	85	158	80.35484	2.5273	158.182381	5.951050584	1.01E-28	1.65E-27	up	Apoptotic ATPase
Solyc11g010420.3	306	319	296	32	25	28	136.6835	236.944	36.4232153	-2.710028286	1.04E-28	1.69E-27	down	Phospholipase/carboxyhydrolase
Solyc01g049770.3	324	290	290	20	16	35	130.8668	232.409	29.3247223	-2.963749835	1.08E-28	1.76E-27	down	--
Solyc06g033850.3	4468	3649	5132	472	388	841	2046.714	3390.81	702.614397	-2.268712307	1.17E-28	1.90E-27	down	--
Solyc08g074250.3	23	12	26	144	111	231	108.4897	15.5472	201.432185	3.693839895	1.18E-28	1.91E-27	up	Apoptotic ATPase
Solyc07g063450.3	353	362	473	39	18	26	169.7356	304.138	35.3335227	-3.107667702	1.18E-28	1.92E-27	down	Amine oxidase
Solyc01g010490.4	1122	1734	1880	183	171	192	724.1277	1214.64	233.616114	-2.380305867	1.34E-28	2.18E-27	down	Cytochrome P450 CYP2 subfamily
Solyc01g005160.4	463	282	525	1	3	3	163.5616	324.126	2.9974145	-6.770292742	1.34E-28	2.18E-27	down	--
Solyc02g081830.4	716	910	1003	105	112	159	416.5045	674.382	158.626677	-2.086278143	1.49E-28	2.41E-27	down	Predicted hydrolase (HAD superfamily)
Solyc02g088953.1	297	311	368	35	19	28	142.5106	250.22	34.8016919	-2.84625345	1.54E-28	2.48E-27	down	DNA mismatch repair protein - MLH3 family
Solyc05g012910.4	2	3	7	108	88	181	79.63015	3.03962	156.220685	5.671652681	1.62E-28	2.61E-27	up	Apoptotic ATPase
Solyc01g067830.3	596	638	706	66	66	44	287.498	497.824	77.1717007	-2.704973773	1.68E-28	2.71E-27	down	U1 snRNP-specific protein C
Solyc11g068530.1	46	43	41	196	134	294	145.9094	33.4398	258.379034	2.954801171	1.80E-28	2.89E-27	up	--
Solyc06g074030.1	2557	1974	3448	65	107	229	1100.088	2035.98	164.193719	-3.627880177	1.87E-28	3.00E-27	down	mRNA deadenylase subunit
Solyc06g082230.4	30	36	43	149	116	189	109.3448	27.9301	190.759431	2.772949228	1.97E-28	3.16E-27	up	Predicted K+/H+-antiporter
Solyc10g086520.2	242	213	328	6	2	7	103.1093	200.066	6.1529871	-4.984051068	2.09E-28	3.35E-27	down	--
Solyc11g066510.3	294	429	360	26	26	29	156.7582	278.864	34.6522108	-3.014977258	2.23E-28	3.56E-27	down	FOG: Predicted E3 ubiquitin ligase
Solyc01g100230.3	313	440	380	26	29	22	162.6005	291.633	33.5683162	-3.138761382	2.30E-28	3.67E-27	down	Molecular chaperone (DnaJ superfamily)
Solyc06g011350.3	123	155	145	332	401	617	337.4427	108.773	566.11224	2.3811931	2.32E-28	3.70E-27	up	Aquaporin (major intrinsic protein family)
Solyc11g011020.2	308	320	369	40	27	34	149.3965	255.706	43.0865925	-2.573826738	2.64E-28	4.20E-27	down	--
Solyc05g015800.3	3	1	4	132	104	255	101.7669	2.02745	201.506252	6.622927505	2.85E-28	4.52E-27	up	--
Solyc04g051660.3	856	655	759	100	98	87	353.0176	582.677	123.358221	-2.248585371	2.90E-28	4.60E-27	down	--
Solyc01g097880.4	3045	4904	4578	11247	11860	11923	9141.471	3220.02	15062.9191	2.225814396	3.21E-28	5.08E-27	up	Cytosine deaminase FCY1 and related enzymes
Solyc01g100200.3	1033	774	953	1812	2475	2595	1828.107	707.914	2948.29884	2.057878632	3.43E-28	5.42E-27	up	--
Solyc05g005760.4	347	321	392	44	25	43	159.4332	271.729	47.1377838	-2.521234204	4.43E-28	6.97E-27	down	--
Solyc07g045140.4	154	217	289	678	854	1401	696.6903	168.754	1224.62675	2.85924978	4.62E-28	7.27E-27	up	--
Solyc12g010890.2	518	473	555	64	43	93	239.7318	396.557	82.906282	-2.244596199	4.84E-28	7.60E-27	down	Transcription factor GT-2 and related proteins
Solyc04g081290.3	346	323	432	812	1041	1710	884.5606	281.853	1487.26774	2.39982245	5.24E-28	8.22E-27	up	--
Solyc06g083200.3	332	318	317	32	36	40	147.3759	248.578	46.1743601	-2.435471253	5.36E-28	8.40E-27	down	FOG: PPR repeat
novel.219	279	248	330	2	6	1	111.8081	219.443	4.17286994	-5.83279471	5.42E-28	8.48E-27	down	--
Solyc04g071870.1	605	480	732	77	69	78	280.1818	464.524	95.8391708	-2.282027629	5.65E-28	8.83E-27	down	Serine/threonine protein kinase
Solyc05g055343.2	320	311	333	31	37	42	147.2528	247.551	46.9547022	-2.404327783	6.11E-28	9.53E-27	down	UDP-glucuronosyl and UDP-glucosyl transferase
Solyc04g015590.4	12	14	8	103	89	181	81.66123	8.79978	154.522679	4.145423279	7.09E-28	1.10E-26	up	Flavonol reductase/cinnamoyl-CoA reductase
Solyc11g067180.2	64	29	31	345	225	621	259.5555	31.9102	487.2008	3.934726608	9.44E-28	1.46E-26	up	Acyl-CoA reductase
Solyc11g017470.2	3681	6120	5444	12732	11831	14633	10303.57	3921.37	16685.7669	2.089192708	9.65E-28	1.49E-26	up	--
Solyc02g089130.4	163	171	161	459	292	542	334.1783	127.321	541.036023	2.089705786	9.76E-28	1.51E-26	up	--
Solyc02g089780.3	1474	2580	2723	7002	5754	9384	5516.941	1738.4	9295.47885	2.41880642	1.02E-27	1.57E-26	up	Flavonol reductase/cinnamoyl-CoA reductase
Solyc03g112340.1	1203	1532	2085	3823	3473	4356	3094.954	1232.35	4957.55749	2.008013955	1.07E-27	1.65E-26	up	FOG: Predicted E3 ubiquitin ligase
Solyc11g005150.3	1365	825	1854	64	82	126	571.6476	1029.29	114.000237	-3.172282351	1.13E-27	1.73E-26	down	--
Solyc08g081370.1	185	154	183	403	418	769	397.3879	133.909	660.866692	2.304585052	1.13E-27	1.74E-26	up	FOG: Predicted E3 ubiquitin ligase

Solyc07g009530.1	307	246	348	3676	11503	10276	5603.657	230.59	10976.724	5.572872161	1.15E-27	1.76E-26	up	Predicted chitinase
Solyc05g009170.3	40	35	52	172	223	422	185.2543	32.4682	338.040297	3.380283811	1.25E-27	1.91E-26	up	--
novel.320	306	246	251	16	13	25	114.4526	206.441	22.4643923	-3.182489019	1.29E-27	1.98E-26	down	--
Solyc01g095310.1	17	19	16	140	82	167	87.78801	13.394	162.182013	3.604135687	1.42E-27	2.17E-26	up	Chitinase
Solyc02g014110.1	232	159	147	470	490	712	421.5702	138.534	704.605907	2.347619774	1.54E-27	2.35E-26	up	--
Solyc09g013150.4	461	579	611	3851	4894	2172	2646.284	423.799	4868.76978	3.52185254	1.95E-27	2.95E-26	up	Permease of the major facilitator superfamily
Solyc12g006970.1	778	714	606	62	83	106	323.417	540.507	106.326569	-2.345673366	1.97E-27	2.99E-26	down	--
novel.1488	494	312	388	30	11	24	166.816	306.386	27.2455749	-3.482328578	2.60E-27	3.93E-26	down	--
Solyc01g056310.3	1	1	2	226	260	230	155.4023	1.01736	309.787307	8.234610859	2.74E-27	4.14E-26	up	Multicopper oxidases
novel.151	252	201	249	3	5	13	94.24907	180.004	8.49401326	-4.353763194	2.80E-27	4.22E-26	down	--
Solyc07g045380.3	547	497	574	71	41	93	250.0577	415.125	84.9901199	-2.27552441	2.81E-27	4.23E-26	down	FOG: PPR repeat
Solyc06g076050.4	1793	1206	1821	104	112	234	709.3953	1233.27	185.518672	-2.727565312	2.81E-27	4.23E-26	down	--
Solyc03g031860.3	40071	64661	63997	144482	145998	156543	117477.9	43332	191623.736	2.144768435	2.99E-27	4.49E-26	up	Squalene synthetase
Solyc01g097290.4	762	501	649	1568	1160	2152	1263.738	490.294	2037.18145	2.055285502	3.05E-27	4.57E-26	up	--
Solyc05g054880.3	300	272	327	32	31	35	136.2153	230.523	41.9076542	-2.467216643	3.39E-27	5.08E-26	down	Putative u4/u6 small nuclear ribonucleoprotein
Solyc09g008850.3	631	586	672	92	81	69	294.8308	484.674	104.987901	-2.217709822	3.50E-27	5.23E-26	down	--
Solyc10g080730.3	174	147	239	585	623	1325	593.1505	142.993	1043.30831	2.867329962	3.71E-27	5.54E-26	up	Thioredoxin
Solyc01g005370.3	620	689	695	79	106	106	319.9504	514.893	125.00805	-2.048867752	3.75E-27	5.59E-26	down	Calmodulin and related proteins (EF-Hand superfamily)
Solyc10g055760.2	2461	1657	2831	82	182	182	983.0128	1774.85	191.174153	-3.216718739	3.82E-27	5.70E-26	down	--
Solyc02g081300.3	96	71	97	249	185	285	185.4181	67.6186	303.217646	2.164976543	3.86E-27	5.75E-26	up	Glycosyltransferase
novel.678	257	247	238	21	15	23	107.8632	190.826	24.9004621	-2.93309377	3.96E-27	5.89E-26	down	--
Solyc02g072470.4	298	294	468	16	18	31	148.8401	270.585	27.09549	-3.308308674	4.01E-27	5.96E-26	down	--
Solyc03g120070.4	213	214	216	12	10	16	90.60595	165.243	15.9684541	-3.361675505	4.15E-27	6.16E-26	down	FOG: PPR repeat
Solyc05g026460.1	597	363	528	46	41	42	218.2788	381.086	55.4713639	-2.788227272	4.16E-27	6.18E-26	down	--
Solyc09g064380.3	569	556	524	76	69	60	256.521	424.199	88.8427273	-2.266754557	4.38E-27	6.50E-26	down	Hsp90 co-chaperone CNS1 (contains TPR repeats)
Solyc02g072440.4	255	221	271	5	7	19	102.0262	191.519	12.5330448	-3.886803417	5.08E-27	7.52E-26	down	--
Solyc02g067060.3	739	599	652	112	79	95	316.692	511.152	122.232008	-2.067499836	5.39E-27	7.94E-26	down	--
Solyc09g010660.3	341	335	309	38	29	46	150.5105	253.458	47.5624111	-2.407528834	5.48E-27	8.08E-26	down	Branching enzyme
Solyc02g081340.3	0	2	1	349	269	430	220.8218	0.77834	440.8653	9.161730622	5.98E-27	8.79E-26	up	Glutathione S-transferase
Solyc12g042830.3	1114	1178	1304	4676	5005	3205	3291.552	922.796	5660.30743	2.616522017	6.02E-27	8.85E-26	up	Molecular chaperone (small heat-shock protein Hsp26/Hsp42)
Solyc09g061280.4	179	101	166	527	1148	1260	682.5114	114.07	1250.95259	3.454363549	6.08E-27	8.92E-26	up	--
Solyc08g005790.3	55	33	42	180	183	367	167.6473	33.3547	301.9399	3.18056642	7.76E-27	1.13E-25	up	Pectin acetylesterase and similar proteins
Solyc12g096770.1	169	142	151	435	276	450	303.9434	118.695	489.191403	2.044431002	8.13E-27	1.19E-25	up	--
Solyc10g052490.2	267	599	595	2012	2203	2481	1618.111	374.98	2861.24214	2.931644808	1.21E-26	1.76E-25	up	--
Solyc09g090020.4	383	300	287	23	23	36	142.0105	249.597	34.4239325	-2.850479591	1.61E-26	2.32E-25	down	--
Solyc09g063150.3	864	846	710	60	80	129	367.928	623.49	112.36595	-2.466992394	1.61E-26	2.32E-25	down	Glutathione S-transferase
Solyc07g047950.1	333	215	406	950	1038	1978	943.5865	243.355	1643.81785	2.755810838	1.64E-26	2.36E-25	up	--
Solyc10g085420.3	2245	1305	1974	137	165	267	826.0704	1414.25	237.89182	-2.569540175	1.64E-26	2.36E-25	down	--
Solyc03g114860.4	135	116	145	407	537	412	346.1083	101.503	590.713354	2.538287133	1.67E-26	2.40E-25	up	--
Solyc09g014940.3	5	5	0	96	124	243	96.89519	2.62392	191.166463	6.213038965	1.74E-26	2.49E-25	up	--
Solyc06g083860.2	539	415	469	53	63	66	221.7081	365.381	78.0353902	-2.234224425	1.89E-26	2.70E-25	down	--
Solyc05g040050.3	332	323	402	36	28	55	160.1602	270.842	49.4782061	-2.438413105	1.93E-26	2.76E-25	down	--
Solyc04g008950.4	208	241	237	6	13	15	95.37551	176.304	14.4465647	-3.612223797	1.93E-26	2.76E-25	down	--
Solyc02g086530.4	104	28	52	575	715	556	425.4335	47.1665	803.700442	4.089817288	1.94E-26	2.78E-25	up	--
Solyc02g093150.3	1013	802	978	2092	1620	3462	1842.495	716.345	2968.64429	2.051568318	2.10E-26	3.01E-25	up	--
Solyc03g093610.1	248	207	205	10	7	15	91.4955	169.729	13.2624322	-3.653793673	2.32E-26	3.31E-25	down	--
Solyc01g100040.3	193	114	218	815	603	1695	701.4562	133.958	1268.95421	3.243896617	2.53E-26	3.61E-25	up	Serine/threonine protein phosphatase

Solyc10g008220.4	268	253	340	27	21	32	127.064	220.39	33.7380303	-2.703492531	2.56E-26	3.64E-25	down	Apoptotic ATPase
Solyc04g008840.3	445	434	487	62	34	39	204.2495	350.545	57.9537979	-2.603051052	3.67E-26	5.20E-25	down	--
Solyc02g065000.1	125	142	115	412	301	698	340.084	98.4437	581.724315	2.566077437	3.78E-26	5.35E-25	up	Calmodulin and related proteins (EF-Hand superfamily)
Solyc02g080040.4	405	294	615	15	22	30	181.3628	334.476	28.2495047	-3.563322327	4.04E-26	5.71E-25	down	--
Solyc03g058910.3	0	6	1	129	217	158	110.9709	1.84245	220.099352	6.927161835	4.18E-26	5.90E-25	up	--
Solyc03g123620.4	632	373	732	28	38	53	246.5928	443.046	50.1397887	-3.141318066	4.45E-26	6.28E-25	down	--
Solyc02g088200.4	86	70	66	214	351	420	237.6988	57.1301	418.267453	2.87249889	4.93E-26	6.95E-25	up	Transcription coactivator complex, P50 component
Solyc11g062030.1	328	333	454	39	39	42	168.3531	285.274	51.4317285	-2.479678027	5.42E-26	7.62E-25	down	--
Solyc09g083100.1	707	885	968	1795	2907	3230	2020.13	656.783	3383.47813	2.364863259	5.49E-26	7.72E-25	up	Serine/threonine protein kinase
Solyc10g082064.1	532	493	496	53	68	51	232.9918	390.969	75.0142599	-2.397210345	6.31E-26	8.84E-25	down	--
Solyc06g053730.1	9	1	9	110	84	109	66.85745	4.81144	128.903459	4.728685375	6.97E-26	9.75E-25	up	Ste20-like serine/threonine protein kinase
Solyc03g120040.3	222	202	240	14	16	13	94.4873	170.291	18.6837568	-3.212828591	7.34E-26	1.03E-24	down	--
Solyc12g038810.3	233	181	204	4	5	6	82.53244	158.684	6.38066152	-4.640515012	8.60E-26	1.20E-24	down	Tyrosine kinase specific for activated (GTP-bound) p21cdc42Hs
Solyc02g089520.2	475	514	658	53	68	54	248.9065	421.706	76.1074353	-2.483286957	8.96E-26	1.25E-24	down	GATA-4/5/6 transcription factors
Solyc12g042470.2	349	191	390	1066	919	1865	916.182	237.17	1595.19395	2.749635576	9.51E-26	1.32E-24	up	Aldo/keto reductase family proteins
Solyc09g010080.3	295	297	354	24	20	10	133.225	242.53	23.9202883	-3.374980667	1.13E-25	1.57E-24	down	Beta-fructofuranosidase (invertase)
Solyc06g005680.4	198	223	287	6	3	2	93.0314	181.243	4.81997741	-5.272985679	1.16E-25	1.61E-24	down	--
Solyc09g090380.1	327	272	347	29	15	39	138.3325	242.435	34.2298587	-2.802825668	1.18E-25	1.64E-24	down	--
novel.776	2255	1317	2225	95	174	193	839.4021	1481.85	196.95695	-2.912716915	1.43E-25	1.97E-24	down	--
Solyc07g044785.1	8	17	23	139	93	146	85.864	12.2572	159.470838	3.69851778	1.43E-25	1.98E-24	up	--
Solyc03g111860.1	4	3	5	74	84	122	60.47952	3.06455	117.894478	5.260686152	1.61E-25	2.22E-24	up	--
Solyc04g009260.3	398	395	480	61	45	45	195.6829	326.285	65.0812009	-2.335806305	1.63E-25	2.25E-24	down	Apoptotic ATPase
Solyc01g094170.3	427	373	518	46	57	55	202.6633	337.295	68.0316474	-2.319867905	1.70E-25	2.34E-24	down	--
novel.864	4	4	1	91	88	179	75.20084	2.34543	148.056255	6.001709279	1.74E-25	2.39E-24	up	--
Solyc11g006300.2	103	261	98	981	1120	1401	803.7191	120.221	1487.21674	3.630183486	1.78E-25	2.45E-24	up	Steroid reductase
Solyc03g120990.3	699	359	672	45	25	45	245.0924	441.881	48.3039584	-3.188778386	1.84E-25	2.52E-24	down	NADP+-dependent malic enzyme
Solyc04g014510.3	6	3	7	104	65	108	60.34966	4.07464	116.624683	4.83177772	2.00E-25	2.74E-24	up	Glutamine synthetase
Solyc07g047690.1	366	378	509	52	43	64	193.8573	320.624	67.0902281	-2.254025877	2.53E-25	3.45E-24	down	--
Solyc09g008030.1	206	190	198	3	3	8	79.15422	152.614	5.69415545	-4.69314626	2.55E-25	3.48E-24	down	--
Solyc01g065690.3	168	137	242	571	399	776	433.5639	139.519	727.609075	2.382602411	2.56E-25	3.49E-24	up	Copper chaperone
Solyc01g108490.3	459	222	399	1049	1303	1819	1017.425	276.097	1758.75284	2.671026497	2.77E-25	3.77E-24	up	Serine carboxypeptidases (lysosomal cathepsin A)
Solyc02g078240.4	122	133	169	377	363	755	363.0368	108.573	617.500827	2.509043283	2.91E-25	3.96E-24	up	Phosphoglycerate mutase
Solyc04g015360.3	1383	1044	1132	50	100	138	517.7214	914.392	121.050555	-2.915693983	3.03E-25	4.11E-24	down	GATA-4/5/6 transcription factors
Solyc09g065480.2	70	64	82	184	151	215	143.995	55.3344	232.655529	2.071358987	3.09E-25	4.20E-24	up	Inositol polyphosphate 5-phosphatase and related proteins
Solyc01g090740.3	449	501	401	26	39	60	200.264	348.223	52.3046988	-2.72793857	3.20E-25	4.34E-24	down	--
Solyc06g069360.3	301	281	350	32	17	41	138.0446	238.841	37.2487139	-2.662029904	3.38E-25	4.57E-24	down	Topoisomerase I-binding arginine-serine-rich protein
Solyc10g007280.4	175	77	107	484	952	1024	571.2169	92.1195	1050.31424	3.510843851	3.62E-25	4.88E-24	up	AAA+-type ATPase
Solyc02g079760.3	99	92	117	381	245	642	299.6427	78.9072	520.37809	2.723229124	4.17E-25	5.62E-24	up	--
Solyc09g007920.4	3305	3722	5964	15866	10466	13183	10087.49	3314.21	16860.7661	2.346861483	4.61E-25	6.19E-24	up	Phenylalanine and histidine ammonia-lyase
Solyc01g095080.3	564	1466	1169	23343	25165	14596	14328.47	823.846	27833.0907	5.078289167	4.62E-25	6.21E-24	up	1-aminocyclopropane-1-carboxylate synthase, and related proteins
Solyc12g056400.3	319	353	345	46	41	43	158.6282	261.421	55.8357557	-2.236436558	4.85E-25	6.50E-24	down	FOG: Predicted E3 ubiquitin ligase
Solyc03g124110.2	250	189	224	2	9	4	88.43498	170.137	6.7328935	-4.725667929	4.98E-25	6.67E-24	down	--
novel.236	320	278	222	19	15	22	117.5476	211.434	23.6612884	-3.156088002	5.28E-25	7.05E-24	down	--

Solyc04g082120.3	102	101	223	555	833	1267	609.9578	108.184	1111.73123	3.36006257	5.28E-25	7.05E-24	up	Predicted serine protease
Solyc02g071730.4	835	906	1033	1691	2611	3296	1964.405	711.499	3217.31031	2.176857044	5.61E-25	7.48E-24	up	MADS box transcription factor
Solyc01g097650.3	58	50	93	211	275	398	211.4963	51.2141	371.778508	2.857764188	5.70E-25	7.60E-24	up	Tumor differentially expressed (TDE) protein
Solyc01g107350.3	123	90	119	293	212	306	214.1973	85.0779	343.316708	2.012296326	6.04E-25	8.04E-24	up	--
Solyc11g020230.1	497	225	579	1	10	10	170.0152	331.06	8.9708028	-5.21405773	6.09E-25	8.09E-24	down	Serine/threonine protein kinase
Solyc03g114070.3	162	198	183	473	437	415	355.7211	139.663	571.779395	2.031927312	6.61E-25	8.79E-24	up	Ras-related small GTPase, Rho type
Solyc08g080190.4	174	203	268	539	385	676	417.6804	165.033	670.328093	2.022593408	7.18E-25	9.53E-24	up	Glucose dehydrogenase/choline dehydrogenase
Solyc06g063060.3	528	395	624	33	33	71	225.9148	395.389	56.4410491	-2.794617316	7.96E-25	1.05E-23	down	--
Solyc05g013530.4	748	877	983	1886	3162	2872	2043.234	668.958	3417.51052	2.352641434	8.71E-25	1.15E-23	up	--
Solyc10g005040.4	96	152	209	478	620	735	448.4003	116.751	780.049465	2.739024795	9.14E-25	1.21E-23	up	--
Solyc09g007430.1	502	459	429	71	60	62	220.3222	357.66	82.9840118	-2.115434228	9.37E-25	1.24E-23	down	--
Solyc04g072800.4	92	120	160	377	532	469	345.5262	95.1351	595.917213	2.644755335	9.38E-25	1.24E-23	up	Phosphoglycerate mutase
Solyc08g041930.1	252	218	270	0	1	7	96.36918	189.699	3.03969206	-5.820947892	9.76E-25	1.29E-23	down	--
Solyc04g150161.1	1340	1247	2616	5450	8653	12335	6216.097	1322.76	11109.4326	3.070048792	1.01E-24	1.33E-23	up	--
Solyc04g073950.2	744	606	1034	1953	2560	2305	1777.124	608.39	2945.85804	2.27496394	1.12E-24	1.47E-23	up	--
Solyc12g056300.3	339	462	463	36	40	62	191.2756	324.655	57.8963413	-2.48136519	1.15E-24	1.50E-23	down	--
Solyc11g008530.3	4962	2620	2579	15947	12998	12673	10282.25	2616.12	17948.3748	2.778340342	1.17E-24	1.53E-23	up	dsRNA-specific nuclease Dicer and related ribonucleases
Solyc03g117720.3	53	66	76	172	214	320	173.2308	49.9899	296.471788	2.568789447	1.20E-24	1.56E-23	up	--
Solyc02g093760.4	4	7	6	74	91	93	57.56236	4.37495	110.749761	4.661213047	1.21E-24	1.57E-23	up	Predicted E3 ubiquitin ligase
Solyc01g100750.2	3	4	7	69	114	183	78.08415	3.5644	152.603905	5.413168045	1.29E-24	1.68E-23	up	--
novel.1017	227	226	180	3	7	4	84.69218	163.192	6.1923857	-4.769547131	1.36E-24	1.76E-23	down	--
Solyc08g075230.1	71	41	59	181	273	305	183.8002	43.8099	323.790443	2.883955872	1.38E-24	1.80E-23	up	--
Solyc02g062100.4	396	352	404	31	48	58	176.8867	295.61	58.1634143	-2.347201982	1.45E-24	1.88E-23	down	--
Solyc12g100120.2	220	211	253	408	525	743	440.6332	175.369	705.897043	2.009243562	1.52E-24	1.97E-23	up	--
Solyc06g068520.3	615	583	502	58	67	104	266.9457	437.866	96.0250312	-2.183734552	1.60E-24	2.08E-23	down	--
Solyc10g006700.1	2544	2328	1860	210	248	419	1050.739	1735.69	365.791712	-2.244285251	1.79E-24	2.31E-23	down	Ca2+-binding protein (centrin/caltractin)
Solyc12g089110.1	3629	3874	3988	318	466	722	1790.925	2951.82	630.031617	-2.227176579	1.89E-24	2.45E-23	down	--
Solyc01g095700.3	1547	1211	1068	3285	2168	4046	2478.347	985.492	3971.20077	2.011022649	1.97E-24	2.54E-23	up	--
Solyc02g071520.3	12	6	5	88	113	263	97.75471	5.93269	189.576729	5.004520384	1.98E-24	2.55E-23	up	Multitransmembrane protein
Solyc07g009473.1	5461	4918	2926	13267	12212	15609	10451.87	3442.04	17461.7072	2.342915875	2.44E-24	3.13E-23	up	--
Solyc01g005570.4	418	373	482	66	38	64	198.4344	326.1	70.7689545	-2.199874379	2.50E-24	3.20E-23	down	--
Solyc06g048820.1	914	674	738	2270	1408	3390	1757.087	597.567	2916.6064	2.287619238	2.53E-24	3.24E-23	up	--
Solyc12g013910.2	406	345	447	57	56	60	190.5509	306.926	74.1759575	-2.056726091	2.60E-24	3.33E-23	down	--
Solyc05g053100.3	1124	1099	1296	217	141	154	561.1845	902.397	219.972036	-2.039544566	2.72E-24	3.47E-23	down	Dihydrolipoamide dehydrogenase
Solyc03g115640.3	245	259	262	27	20	35	115.5832	196.824	34.3422564	-2.507834671	2.80E-24	3.58E-23	down	--
novel.992	280	219	222	5	12	3	97.26778	185.388	9.14752264	-4.417799946	2.81E-24	3.58E-23	down	--
Solyc01g105720.2	1734	1182	1388	4399	2476	5126	3053.786	1104.98	5002.59384	2.17885279	2.97E-24	3.78E-23	up	--
Solyc10g006660.3	2634	2143	1652	156	212	320	973.5135	1658.53	288.495634	-2.521948872	3.22E-24	4.10E-23	down	Ca2+-binding protein (centrin/caltractin)
Solyc12g036650.2	233	159	259	6	9	7	87.97656	166.377	9.57563271	-4.147230552	4.11E-24	5.20E-23	down	--
Solyc01g100770.2	217	357	355	734	739	1170	673.6357	238.554	1108.71696	2.217008116	4.74E-24	5.97E-23	up	--
Solyc07g045520.4	259	198	260	22	12	18	102.8878	183.727	22.0490459	-3.058474781	4.86E-24	6.11E-23	down	--
Solyc02g069050.1	49	51	56	211	129	306	153.4533	40.0387	266.867853	2.74041595	5.58E-24	6.98E-23	up	--
Solyc04g005050.1	371	266	422	26	29	44	156.1404	270.696	41.5849361	-2.697197267	5.64E-24	7.05E-23	down	Gelatinase A and related matrix metalloproteases
Solyc06g005230.3	352	374	428	33	42	25	170.0339	295.988	44.0795701	-2.771440282	5.69E-24	7.11E-23	down	Serine/threonine protein kinase
Solyc11g066100.2	1827	2003	2346	7135	6598	4708	4822.912	1583.39	8062.429	2.347997692	5.83E-24	7.28E-23	up	Molecular chaperones HSP70/HSC70, HSP70 superfamily
Solyc05g010750.3	22	21	21	151	80	144	87.04285	16.4513	157.634403	3.263181796	6.31E-24	7.87E-23	up	--
Solyc03g078640.1	564	504	895	55	60	87	292.7703	500.445	85.095552	-2.554146021	6.39E-24	7.96E-23	down	--

Solyc11g010870.2	496	437	468	54	41	91	218.3431	359.861	76.8256902	-2.213100415	6.50E-24	8.10E-23	down	--
novel.1206	211	278	201	3	7	13	93.76463	178.057	9.471912	-4.203891014	6.77E-24	8.43E-23	down	--
Solyc02g088470.1	230	243	243	20	23	29	107.284	184.007	30.561017	-2.591138934	6.98E-24	8.68E-23	down	FOG: PPR repeat
Solyc01g108650.3	357	418	375	59	43	60	182.3142	295.934	68.6943975	-2.10644165	7.42E-24	9.20E-23	down	--
Solyc11g006900.1	247	294	225	13	17	25	110.324	197.54	23.1080169	-3.088030475	8.49E-24	1.05E-22	down	--
Solyc12g006050.2	9	18	21	172	113	434	150.459	12.2894	288.628568	4.55523101	9.13E-24	1.12E-22	up	H+/oligopeptide symporter
Solyc10g017980.1	883	1229	2399	21542	60679	82085	35074.31	1146.28	69002.3367	5.911574649	1.19E-23	1.45E-22	up	Predicted chitinase
Solyc10g081110.3	387	369	331	50	45	60	172.7802	279.825	65.7357777	-2.08953695	1.29E-23	1.56E-22	down	FOG: Predicted E3 ubiquitin ligase
Solyc12g009840.2	335	272	308	27	37	37	139.1416	234.9	43.3831794	-2.447101212	1.49E-23	1.81E-22	down	--
Solyc10g055390.2	574	766	694	19	48	18	280.7834	523.228	38.3390505	-3.795620946	1.51E-23	1.83E-22	down	--
Solyc11g005440.2	292	263	263	30	30	28	124.1447	210.296	37.9931803	-2.483045564	1.64E-23	1.99E-22	down	--
Solyc03g080170.3	294	271	245	29	25	33	122.7208	208.509	36.9330015	-2.497639208	1.76E-23	2.12E-22	down	--
Solyc07g064160.3	41073	42143	43841	7536	4420	4775	19916.98	32636.7	7197.30529	-2.181051362	1.76E-23	2.12E-22	down	Protein involved in thiamine biosynthesis
Solyc08g067970.3	258	207	222	948	915	609	630.2265	176.503	1083.94991	2.617475072	1.86E-23	2.24E-22	up	--
Solyc08g060815.1	11	11	18	113	73	192	82.64377	10.2058	155.081709	3.92582871	1.96E-23	2.36E-22	up	--
Solyc09g011850.4	1416	1461	1620	221	168	131	690.2978	1154.05	226.542221	-2.353638548	1.99E-23	2.40E-22	down	Ribokinase
Solyc06g054580.4	264	283	351	33	33	40	137.5949	230.045	45.144903	-2.353244784	2.00E-23	2.41E-22	down	Topoisomerase I-binding arginine-serine-rich protein
Solyc07g041555.1	1125	699	976	21	66	36	386.0032	717.432	54.5739737	-3.730824474	2.03E-23	2.44E-22	down	--
Solyc07g042160.3	225	222	275	19	16	31	106.2188	185.008	27.4297641	-2.736761678	2.04E-23	2.45E-22	down	--
novel.1283	495	461	250	10	8	26	165.0276	312.295	17.7596915	-4.104340257	2.09E-23	2.51E-22	down	--
Solyc05g049990.4	840	846	745	1715	1761	3699	1792.475	625.9	2959.05063	2.241710349	2.10E-23	2.52E-22	up	Copper chaperone
novel.1257	346	319	307	28	26	53	147.1378	250.003	44.2723962	-2.480161784	2.17E-23	2.61E-22	down	--
novel.1286	2	2	3	78	142	133	76.89993	1.78843	152.011416	6.400896156	2.25E-23	2.70E-22	up	--
Solyc03g119250.4	1629	982	1486	74	133	164	602.9482	1048.74	157.157454	-2.738707082	2.30E-23	2.75E-22	down	--
novel.1680	14	17	11	100	68	122	66.14885	10.8542	121.443453	3.492838754	2.39E-23	2.85E-22	up	--
Solyc09g009840.1	170	174	267	548	968	852	589.7458	156.037	1023.45505	2.712085004	2.56E-23	3.05E-22	up	--
Solyc07g054370.3	437	466	531	33	20	68	208.4082	367.825	48.9915319	-2.885046403	2.66E-23	3.16E-22	down	--
Solyc04g077460.3	222	199	260	12	20	18	98.00264	174.419	21.5867314	-3.031725063	2.68E-23	3.18E-22	down	Amino acid transporter protein
Solyc04g078120.3	324	341	288	32	14	38	140.0862	245.484	34.6886903	-2.803584431	2.88E-23	3.42E-22	down	Guanidinoacetate methyltransferase and related proteins
Solyc07g005100.4	134	93	227	499	639	874	482.2483	115.321	849.175175	2.878875941	2.89E-23	3.43E-22	up	Chitinase
novel.598	555	427	490	31	59	53	219.8026	377.885	61.7198983	-2.623759583	2.94E-23	3.48E-22	down	--
Solyc12g095810.2	49	40	57	137	141	238	126.4742	37.3586	215.589672	2.529834625	3.32E-23	3.93E-22	up	H+/oligopeptide symporter
Solyc06g073380.3	223	247	261	18	25	19	107.3566	187.693	27.0202158	-2.820057019	3.40E-23	4.02E-22	down	Predicted methyltransferase
Solyc07g063860.4	492	330	269	25	21	22	155.2842	281.349	29.2193303	-3.275250555	3.42E-23	4.04E-22	down	--
Solyc01g095140.4	1706	1594	1661	135	65	236	725.6992	1274.57	176.825954	-2.842177451	3.62E-23	4.27E-22	down	--
novel.167	714	515	704	71	24	61	280.0806	495.144	65.0174425	-2.921506715	3.86E-23	4.54E-22	down	--
novel.1514	185	214	176	8	2	4	77.04065	148.147	5.93459355	-4.63992122	4.25E-23	4.99E-22	down	--
Solyc02g092240.3	70	58	66	169	203	197	147.3793	49.7976	244.960979	2.294754014	4.32E-23	5.06E-22	up	--
Solyc03g059110.1	235	158	258	10	10	7	89.09842	166.383	11.8141459	-3.8468728	5.16E-23	6.03E-22	down	--
Solyc01g102470.2	247	280	313	36	25	31	127.3775	215.489	39.2659545	-2.461149508	5.18E-23	6.05E-22	down	Molecular chaperone (DnaJ superfamily)
Solyc01g096210.4	327	350	349	51	26	40	156.6366	263.678	49.5952944	-2.40939546	5.33E-23	6.22E-22	down	FOG: PPR repeat
novel.944	418	393	528	35	57	62	204.2603	342.75	65.7710897	-2.386612493	5.47E-23	6.38E-22	down	FOG: Transposon-encoded proteins with TYA
Solyc03g117610.2	7	5	11	91	64	166	68.71749	5.85061	131.584377	4.488094964	6.61E-23	7.69E-22	up	--
novel.1438	1475	1197	1313	149	223	201	635.464	1023.48	247.449715	-2.052550041	7.13E-23	8.28E-22	down	--
Solyc09g082570.3	352	358	508	41	49	57	187.0484	311.435	62.6618813	-2.317291989	7.25E-23	8.40E-22	down	--
Solyc02g086770.3	10	14	22	121	73	136	74.9526	11.7303	138.174895	3.555621359	7.78E-23	8.99E-22	up	Flavonol reductase/cinnamoyl-CoA reductase
Solyc10g018015.1	599	325	514	46	40	45	212.0611	368.047	56.0755899	-2.71925975	7.86E-23	9.06E-22	down	--
Solyc05g054405.1	220	226	236	21	19	31	102.4721	175.173	29.7713941	-2.546825294	8.20E-23	9.44E-22	down	--

Solyc02g022850.1	1284	911	964	144	64	128	476.4682	812.017	140.919208	-2.523412336	9.86E-23	1.13E-21	down	--
Solyc04g008790.1	237	267	244	21	18	35	111.5946	192.449	30.740012	-2.629007304	1.10E-22	1.25E-21	down	Uncharacterized conserved protein
Solyc02g072450.4	377	321	424	36	51	56	174.2305	287.373	61.0884335	-2.239661411	1.16E-22	1.32E-21	down	--
novel.1334	168	201	184	9	4	3	74.62264	142.26	6.98549143	-4.380057668	1.19E-22	1.35E-21	down	--
novel.1995	290	239	227	11	14	28	108.1936	194.528	21.8595624	-3.131400374	1.22E-22	1.39E-21	down	--
Solyc04g010250.3	2438	1518	2345	346	213	306	989.6067	1612.23	366.987378	-2.135216473	1.22E-22	1.39E-21	down	Lysophospholipase
Solyc06g067980.3	83	60	109	323	307	817	326.6884	64.2839	589.092843	3.196286449	1.25E-22	1.42E-21	up	--
Solyc04g010030.1	11	13	16	85	66	127	62.98597	10.2453	115.726649	3.500335518	1.31E-22	1.49E-21	up	Thioredoxin-like protein
Solyc02g077850.3	335	372	504	47	47	59	187.4062	309.775	65.0371118	-2.254005209	1.32E-22	1.50E-21	down	FOG: Leucine rich repeat
Solyc12g044250.2	105	110	111	327	190	370	227.2614	83.7705	370.75219	2.148568412	1.39E-22	1.57E-21	up	Glyoxylate/hydroxypyruvate reductase
Solyc07g054670.4	53	65	80	215	145	223	148.4525	50.709	246.196086	2.279302517	1.46E-22	1.65E-21	up	NADH-dehydrogenase (ubiquinone)
Solyc01g097170.3	414	461	422	54	69	71	208.4631	333.698	83.2284364	-2.010463196	1.46E-22	1.66E-21	down	--
Solyc01g058470.1	228	154	184	4	7	8	76.68463	145.282	8.08734389	-4.172008936	1.57E-22	1.78E-21	down	--
Solyc02g068820.3	334	338	457	40	49	46	173.5561	288.896	58.2161804	-2.322280826	1.63E-22	1.83E-21	down	--
Solyc06g150132.1	425	368	900	26	31	35	234.4064	429.53	39.2833086	-3.454562395	1.66E-22	1.87E-21	down	Calmodulin and related proteins (EF-Hand superfamily)
Solyc01g100400.3	475	451	467	77	39	79	219.7199	357.905	81.5350814	-2.125865927	1.68E-22	1.88E-21	down	RNA pseudouridylate synthases
Solyc09g075590.1	14	11	14	126	97	84	71.5726	9.99694	133.14826	3.730019465	1.73E-22	1.93E-21	up	--
novel.2035	286	253	379	33	21	21	133.5034	234.653	32.3540661	-2.870344641	1.77E-22	1.98E-21	down	--
novel.2071	247	321	325	27	31	24	132.532	229.352	35.7123896	-2.702330902	1.78E-22	1.99E-21	down	FOG: Transposon-encoded proteins with TYA
Solyc04g015470.3	212	271	225	18	6	19	100.0475	182.365	17.7301777	-3.337260069	1.88E-22	2.10E-21	down	Phosphatidylinositol-4-phosphate 5-kinase
Solyc11g017410.1	100	129	162	322	544	575	358.2228	100.092	616.353635	2.621194716	1.94E-22	2.16E-21	up	--
Solyc05g054040.4	224	321	292	21	25	29	123.6245	215.273	31.9763067	-2.755403402	1.95E-22	2.18E-21	down	Endosomal membrane proteins, EMP70
Solyc11g006410.2	383	436	404	724	1103	1174	799.1853	314.592	1283.77819	2.028549358	2.11E-22	2.34E-21	up	--
Solyc08g061930.4	13	8	10	84	64	143	64.04829	7.95495	120.141628	3.921587472	2.12E-22	2.35E-21	up	Proteins containing the FAD binding domain
Solyc02g091500.1	775	574	1032	29	41	92	336.8308	607.406	66.2553084	-3.185849927	2.15E-22	2.39E-21	down	Calmodulin and related proteins (EF-Hand superfamily)
Solyc10g006880.3	3543	7618	5797	16228	18120	22568	14276.22	4371.11	24181.3373	2.467839947	2.26E-22	2.50E-21	up	--
Solyc03g117350.1	290	269	229	24	26	26	117.8426	203.001	32.6842535	-2.645662598	2.28E-22	2.52E-21	down	Amino acid transporter protein
Solyc08g036507.1	365	260	322	19	17	42	137.4227	242.918	31.9270234	-2.903937943	2.32E-22	2.57E-21	down	--
Solyc07g007770.2	350	287	250	27	20	37	131.779	228.487	35.07104	-2.692185212	2.33E-22	2.57E-21	down	--
Solyc06g083330.2	20	19	15	90	73	119	66.17255	13.924	118.421115	3.094717179	2.42E-22	2.67E-21	up	--
Solyc12g006250.3	196	199	200	11	9	22	85.071	152.914	17.2284646	-3.118015713	2.65E-22	2.91E-21	down	Membrane protein involved in ER to Golgi transport
Solyc03g113130.3	197	172	222	2	9	2	78.70601	151.408	6.00410987	-4.755232641	2.66E-22	2.93E-21	down	Uncharacterized conserved protein
Solyc08g078960.4	140	203	266	519	653	1087	549.063	155.742	942.383741	2.597196991	2.84E-22	3.12E-21	up	Oxysterol-binding protein
Solyc01g104900.3	262	137	140	732	499	1092	550.3961	138.72	962.071767	2.795015999	3.03E-22	3.32E-21	up	--
Solyc02g091250.1	282	237	252	10	24	22	111.104	198.083	24.1253143	-3.051198283	3.03E-22	3.32E-21	down	--
Solyc08g079440.1	404	300	354	47	38	59	166.0844	271.532	60.6365675	-2.158295672	3.09E-22	3.38E-21	down	UDP-glucose 4-epimerase/UDP-sulfoquinovose synthase
novel.2027	262	198	190	14	14	15	92.84862	167.263	18.4346416	-3.191329994	3.35E-22	3.67E-21	down	--
Solyc05g015150.3	321	264	267	33	32	38	131.4892	219.051	43.92717	-2.322409045	3.46E-22	3.78E-21	down	--
Solyc10g086280.2	2075	1522	1475	75	156	201	743.7067	1305.09	182.323177	-2.839276282	3.91E-22	4.26E-21	down	Copper chaperone
Solyc01g005140.3	267	317	296	747	1893	1687	1046.681	226.32	1867.04119	3.044140601	4.01E-22	4.37E-21	up	Cytochrome b
Solyc09g057630.3	229	161	228	13	6	5	84.34065	158.24	10.4417376	-3.946195826	4.31E-22	4.68E-21	down	--
Solyc01g107820.2	2444	1917	2215	4560	7787	6401	4911.165	1687.91	8134.42347	2.268659625	4.38E-22	4.75E-21	up	UDP-glucuronosyl and UDP-glucosyl transferase
Solyc01g107170.2	1234	1512	2500	4436	4574	5367	3734.836	1337.26	6132.41152	2.196964864	4.53E-22	4.91E-21	up	FOG: Zn-finger
Solyc09g056180.3	63	32	52	164	160	281	144.9898	37.6216	252.358114	2.746556237	5.10E-22	5.51E-21	up	Intracellular Cl- channel CLIC, contains GST domain

Solyc04g077000.3	240	273	248	12	24	27	111.3144	195.807	26.8220552	-2.871946001	5.59E-22	6.02E-21	down	Predicted E3 ubiquitin ligase
novel.45	101	106	136	340	224	296	226.9631	87.8286	366.097557	2.058060185	5.88E-22	6.33E-21	up	--
Solyc02g088380.4	339	224	268	14	24	24	119.9589	213.314	26.6036617	-3.012968877	6.10E-22	6.56E-21	down	--
Solyc02g031780.1	379	220	202	0	4	4	104.8793	206.345	3.41336474	-5.926401446	6.23E-22	6.69E-21	down	--
Solyc06g071400.3	1	8	4	140	118	78	75.36272	3.37213	147.35332	5.456188827	6.58E-22	7.06E-21	up	Multitransmembrane protein
novel.1294	238	228	207	9	20	15	96.20077	173.22	19.1813831	-3.199505573	7.16E-22	7.67E-21	down	--
Solyc12g096350.2	205	191	366	6	5	15	102.2665	193.998	10.5349697	-4.163748701	7.42E-22	7.94E-21	down	--
Solyc04g051690.4	8	3	17	96	220	172	109.6444	7.05504	212.233784	4.896769171	8.38E-22	8.93E-21	up	--
Solyc05g009120.3	103	111	115	281	504	411	301.8033	84.5042	519.102374	2.617260442	8.65E-22	9.21E-21	up	--
Solyc01g099840.3	5589	5325	4254	11565	8220	18315	9830.963	3910.5	15751.4262	2.010184576	1.06E-21	1.12E-20	up	--
Solyc05g012210.4	144	95	91	299	357	654	314.2965	84.9459	543.647064	2.679820452	1.14E-21	1.20E-20	up	--
Solyc07g055470.3	954	1127	1319	179	105	230	542.4822	871.522	213.44278	-2.024502204	1.18E-21	1.25E-20	down	Cytochrome P450 CYP4/CYP19/CYP26 subfamilies
Solyc10g006150.3	45	50	73	180	131	203	129.8394	42.9245	216.754276	2.334969783	1.21E-21	1.28E-20	up	--
Solyc12g099780.2	32	35	52	120	118	169	101.0817	30.3982	171.765156	2.496290848	1.46E-21	1.54E-20	up	--
novel.558	496	513	519	88	42	69	238.4043	392.639	84.1693118	-2.219830471	1.49E-21	1.57E-20	down	--
Solyc01g007130.3	534	554	453	60	52	103	243.1627	397.124	89.2011807	-2.143957024	1.57E-21	1.64E-20	down	--
Solyc03g122340.3	2737	1453	2719	116	228	169	994.1072	1764.41	223.800023	-2.982319392	1.57E-21	1.64E-20	down	--
novel.1303	749	486	615	82	87	89	292.7007	474.566	110.835524	-2.103713955	1.72E-21	1.79E-20	down	--
Solyc10g074990.2	283	251	330	40	34	41	135.1682	221.276	49.0599808	-2.178248515	1.81E-21	1.88E-20	down	--
Solyc03g119920.1	400	340	439	61	54	50	186.6883	302.073	71.3037044	-2.094322525	1.88E-21	1.95E-20	down	--
novel.1090	621	851	670	74	58	131	330.276	552.091	108.461321	-2.338114446	1.96E-21	2.04E-20	down	Histones H3 and H4
Solyc05g009790.1	1473	1366	2399	86	121	239	759.6285	1335.39	183.868138	-2.856848744	2.10E-21	2.17E-20	down	--
Solyc05g039990.4	344	318	440	52	45	43	171.196	281.976	60.4158988	-2.233600991	2.27E-21	2.34E-20	down	--
Solyc10g080370.1	3837	3145	2331	137	215	410	1359.026	2403.61	314.447318	-2.932257786	2.29E-21	2.35E-20	down	--
Solyc01g098530.3	249	208	320	22	16	32	113.8414	198.577	29.1063287	-2.755832296	2.30E-21	2.36E-20	down	CTP synthase (UTP-ammonia lyase)
Solyc01g107070.4	317	288	409	37	32	58	156.1692	259.374	52.96457	-2.281763517	2.37E-21	2.44E-20	down	--
Solyc07g048090.2	199	296	365	23	13	22	122.2825	220.132	24.4329534	-3.166572118	2.51E-21	2.58E-20	down	--
Solyc02g077090.4	106	72	108	291	206	259	197.7816	73.1814	322.381815	2.137113191	2.66E-21	2.72E-20	up	--
Solyc01g111270.3	551	499	392	40	60	50	218.4601	371.868	65.0521908	-2.525795112	2.79E-21	2.85E-20	down	--
Solyc09g089740.3	163	168	225	485	418	1020	465.24	142.285	788.195094	2.470881978	3.34E-21	3.41E-20	up	Iron/ascorbate family oxidoreductases
Solyc03g120580.3	51	32	30	237	352	185	186.1412	29.0981	343.184318	3.558307881	3.37E-21	3.43E-20	up	H+/oligopeptide symporter
Solyc08g068690.1	3551	2105	2684	130	291	247	1214.511	2139.87	289.149868	-2.889743708	3.39E-21	3.45E-20	down	Diamine acetyltransferase
Solyc06g062800.4	37	54	48	124	134	177	110.0072	35.7613	184.253044	2.366507662	3.39E-21	3.46E-20	up	Translation initiation factor 4F, helicase subunit (eIF-4A)
Solyc06g053290.1	372	517	506	33	30	73	207.0594	358.416	55.7029846	-2.669547049	3.73E-21	3.80E-20	down	--
Solyc01g016460.4	240	275	321	27	21	44	126.2143	214.318	38.1107321	-2.474691112	4.47E-21	4.53E-20	down	--
novel.2008	178	151	181	9	9	4	70.30087	130.807	9.79463011	-3.791007872	4.73E-21	4.79E-20	down	--
Solyc09g074675.1	37	24	36	103	106	171	92.00798	24.8251	159.190901	2.681506807	5.76E-21	5.80E-20	up	--
Solyc09g098320.1	1	1	0	368	314	350	221.2759	0.52478	442.027105	9.747523075	5.80E-21	5.84E-20	up	NADH:flavin oxidoreductase/12-oxophytodienoate reductase
Solyc08g076120.3	95	80	61	201	187	314	177.3279	60.8877	293.768142	2.274343487	5.88E-21	5.91E-20	up	Uncharacterized conserved protein
Solyc03g044160.3	287	334	357	52	34	54	155.0436	251.041	59.0457657	-2.084054364	6.41E-21	6.43E-20	down	Serine/threonine protein kinase
Solyc11g005380.2	2757	2479	4049	235	398	274	1383.665	2370.1	397.232079	-2.579683696	6.53E-21	6.55E-20	down	Transcription factor GT-2 and related proteins
Solyc06g008360.3	139	84	72	293	368	598	301.0208	76.0463	525.99522	2.791668126	6.62E-21	6.63E-20	up	--
Solyc07g006630.4	18	13	11	168	63	197	93.44792	10.8252	176.070676	4.029285322	7.13E-21	7.13E-20	up	GATA-4/5/6 transcription factors
Solyc12g008580.2	157	139	172	8	3	6	63.55831	119.964	7.15232655	-4.05874388	7.22E-21	7.21E-20	down	--
Solyc03g114040.3	230	189	241	23	22	24	99.35556	169.149	29.5622814	-2.525847388	7.23E-21	7.21E-20	down	--
Solyc08g028780.1	288	137	254	6	6	11	91.54572	173.525	9.56635184	-4.164165071	7.92E-21	7.90E-20	down	--
Solyc03g119540.3	240	202	276	29	17	22	106.414	183.815	29.0130966	-2.668265699	8.24E-21	8.21E-20	down	GATA-4/5/6 transcription factors

Solyc02g062690.3	31	53	44	192	113	210	124.3551	32.9576	215.752621	2.714203834	8.25E-21	8.21E-20	up	--
Solyc05g052990.4	247	254	221	17	26	18	106.3105	185.914	26.7073824	-2.824592163	9.10E-21	9.03E-20	down	--
novel.1134	227	155	202	9	12	17	82.86048	149.722	15.9985718	-3.220592652	1.27E-20	1.25E-19	down	--
Solyc11g010480.2	2950	2604	3124	505	375	310	1371.337	2225.47	517.199904	-2.107441263	1.31E-20	1.30E-19	down	--
Solyc02g088240.4	395	180	365	19	15	17	130.9143	239.989	21.8393293	-3.464536739	1.35E-20	1.33E-19	down	Predicted small molecule transporter
Solyc11g011750.1	5	6	5	68	51	118	50.89931	4.12139	97.6772361	4.576126222	1.38E-20	1.36E-19	up	--
Solyc11g066090.1	189	197	250	20	18	25	94.77166	162.885	26.6587029	-2.609982117	1.43E-20	1.41E-19	down	--
Solyc10g009400.3	253	239	280	36	32	32	120.5299	198.007	43.0529919	-2.213504886	1.44E-20	1.42E-19	down	Predicted methyltransferase
Solyc07g018393.1	296	264	285	37	34	27	129.8309	217.016	42.6463226	-2.365648773	1.45E-20	1.43E-19	down	--
Solyc06g074460.4	286	313	313	38	36	61	145.4052	234.359	56.4509339	-2.043734936	1.61E-20	1.58E-19	down	Peroxisomal biogenesis protein (peroxin 16)
Solyc06g036250.2	33	48	41	146	529	508	269.5152	31.4061	507.624337	4.014815872	1.75E-20	1.71E-19	up	--
Solyc10g008310.1	197	208	247	13	22	24	96.16524	167.142	25.188372	-2.737124898	1.78E-20	1.74E-19	down	--
Solyc01g091770.4	58	68	58	151	123	188	121.0375	47.3825	194.692466	2.042416197	1.78E-20	1.74E-19	up	FOG: Predicted E3 ubiquitin ligase
Solyc05g051330.1	211	255	223	17	11	30	100.5515	177.357	23.7458435	-2.872173023	1.86E-20	1.81E-19	down	--
Solyc01g087580.3	350	262	169	0	5	2	102.5302	201.887	3.17353049	-6.070738174	1.87E-20	1.82E-19	down	--
Solyc02g071900.3	344	375	447	42	60	59	184.0351	298.864	69.206499	-2.119158583	1.96E-20	1.91E-19	down	Uncharacterized conserved protein
novel.967	263	282	337	41	33	53	139.7266	226.072	53.3811241	-2.076306585	2.11E-20	2.05E-19	down	--
Solyc06g069710.3	181	73	114	495	472	480	358.2663	94.3319	622.200691	2.720551447	2.16E-20	2.09E-19	up	--
novel.560	282	260	260	41	29	42	126.7943	206.172	47.4170167	-2.118672753	2.18E-20	2.11E-19	down	--
Solyc10g007460.4	299	258	312	46	26	38	134.7625	222.845	46.679556	-2.255317475	2.37E-20	2.29E-19	down	--
Solyc02g089210.4	25	41	42	125	117	228	111.3412	27.7202	194.962278	2.817531003	2.51E-20	2.42E-19	up	MADS box transcription factor
Solyc06g082440.1	830	636	1206	59	92	121	397.9333	680.986	114.880817	-2.567192062	2.52E-20	2.43E-19	down	Serine/threonine protein kinase
Solyc12g015770.3	137	89	154	626	285	517	349.3012	97.0546	601.547869	2.631302312	2.61E-20	2.52E-19	up	--
Solyc08g007230.3	148	149	146	6	7	6	61.0628	113.892	8.23334215	-3.814702335	2.74E-20	2.64E-19	down	--
Solyc10g050910.1	203	157	140	6	6	7	68.44158	128.774	8.10878459	-3.995903899	2.82E-20	2.71E-19	down	--
Solyc08g080560.3	5	0	3	121	86	72	61.62141	2.03265	121.210161	5.882915675	2.87E-20	2.76E-19	up	--
novel.566	184	136	153	7	6	8	65.19187	121.473	8.91056735	-3.769751461	2.90E-20	2.79E-19	down	--
Solyc05g005560.4	21429	17332	18320	3149	1542	3659	9066.156	14667.7	3464.61366	-2.08154552	2.90E-20	2.79E-19	down	--
Solyc02g037590.3	155	162	185	6	10	15	70.87344	128.767	12.9797166	-3.298647733	2.96E-20	2.84E-19	down	--
Solyc05g005290.4	870	650	440	1738	3074	3021	1935.223	506.403	3364.0435	2.731914874	2.97E-20	2.85E-19	up	--
Solyc03g096780.1	15	32	43	138	298	280	165.5406	22.9846	308.096571	3.741768418	3.00E-20	2.87E-19	up	--
Solyc03g007890.3	3963	2889	3019	11896	12192	7550	8226.592	2537.55	13915.6316	2.455115396	3.20E-20	3.07E-19	up	Molecular chaperone (HSP90 family)
novel.1652	240	371	313	34	22	36	138.3162	237.886	38.7462836	-2.613155376	3.24E-20	3.10E-19	down	--
Solyc02g082930.3	1026	1140	1479	2071	3248	3464	2344.607	933.017	3756.19745	2.009045768	3.87E-20	3.69E-19	up	Predicted chitinase
novel.1320	428	603	465	49	36	85	227.8472	385.687	70.0076377	-2.44889713	3.96E-20	3.77E-19	down	FOG: Transposon-encoded proteins with TYA
Solyc08g080650.3	3277	4048	4948	9458	8254	20539	9400.168	3143.46	15656.8751	2.316438783	4.05E-20	3.85E-19	up	--
Solyc07g063320.3	185	137	194	11	6	13	72.28894	132.096	12.4820902	-3.38504057	4.46E-20	4.23E-19	down	Lanthionine synthetase C-like protein 1
novel.192	265	278	346	43	39	45	141.0083	227.742	54.2744677	-2.075069616	4.49E-20	4.25E-19	down	--
Solyc07g042590.4	1024	844	1102	133	169	126	473.8116	760.904	186.718808	-2.034236356	5.13E-20	4.85E-19	down	Serine/threonine protein kinase
novel.775	1	1	0	199	458	461	239.7445	0.52478	478.964236	9.862950648	5.14E-20	4.85E-19	up	--
Solyc03g044060.4	499	539	719	100	71	75	277.6869	449.59	105.783886	-2.092939704	5.95E-20	5.60E-19	down	Rho GTPase effector BNI1 and related formins
Solyc11g069180.2	507	420	478	60	49	105	224.5552	360.647	88.4631162	-2.01501029	6.37E-20	5.99E-19	down	Isovaleryl-CoA dehydrogenase
Solyc08g077210.4	639	353	559	51	35	75	231.8391	396.929	66.7495521	-2.562848959	6.56E-20	6.16E-19	down	Inositol polyphosphate 5-phosphatase and related proteins
Solyc06g007190.4	713	439	785	31	51	84	281.8604	494.616	69.1044495	-2.834156024	6.77E-20	6.36E-19	down	Serine/threonine protein phosphatase
Solyc02g021410.4	4	5	0	81	53	127	54.99295	2.36516	107.620743	5.538362278	7.00E-20	6.56E-19	up	Predicted K+/H+-antiporter
Solyc06g064880.3	4	1	5	70	50	93	45.74289	2.5325	88.9532733	5.12199329	7.06E-20	6.62E-19	up	Carbon-nitrogen hydrolase
Solyc01g094960.3	291	303	384	37	48	48	153.8116	250.479	57.1438418	-2.141368191	7.69E-20	7.18E-19	down	Mitogen-activated protein kinase
Solyc06g067890.3	269	227	301	37	26	43	124.3459	204.127	44.5649966	-2.189343068	7.84E-20	7.32E-19	down	Predicted hydrolase/acyltransferase (alpha/beta hydrolase superfamily)

Solyc07g064410.1	51	144	89	373	504	568	344.988	73.4242	616.551856	3.070833798	8.24E-20	7.68E-19	up	Ubiquitin-conjugating enzyme
Solyc10g008460.3	174	199	199	21	11	18	84.04865	146.975	21.1227056	-2.794608095	8.69E-20	8.09E-19	down	Lanthionine synthetase C-like protein 1
Solyc10g006960.3	50	48	76	145	140	220	128.2329	44.4251	212.040798	2.2541286	8.83E-20	8.22E-19	up	Predicted E3 ubiquitin ligase
Solyc02g067450.4	0	0	0	255	266	181	153.7751	0	307.550142	11.0804909	9.09E-20	8.45E-19	up	--
novel.2012	278	313	270	36	39	47	136.8202	221.699	51.9415147	-2.09635466	9.13E-20	8.48E-19	down	--
Solyc03g044720.2	218	211	236	26	28	29	103.1475	170.665	35.6301096	-2.270766626	9.28E-20	8.62E-19	down	--
Solyc12g056730.1	18	8	8	89	63	133	63.47594	8.75615	118.195716	3.761015814	9.87E-20	9.15E-19	up	--
Solyc04g072700.4	48	16	24	164	151	342	146.3866	22.5877	270.18547	3.582174925	1.06E-19	9.80E-19	up	Copper chaperone
novel.1169	508	493	574	29	66	68	236.8517	403.97	69.7336393	-2.538922689	1.10E-19	1.02E-18	down	--
Solyc03g082900.3	8	11	12	66	57	85	47.83152	7.95182	87.7112206	3.464241202	1.13E-19	1.04E-18	up	--
Solyc03g083470.3	272	132	318	4	5	12	96.19216	183.817	8.56701239	-4.390847985	1.13E-19	1.04E-18	down	Serine/threonine protein kinase
Solyc04g082040.3	390	264	343	13	31	35	144.6103	255.623	33.5972263	-2.930668957	1.19E-19	1.10E-18	down	Predicted mechanosensitive ion channel
Solyc12g042010.1	193	173	174	18	14	17	79.86502	138.817	20.912989	-2.736403923	1.22E-19	1.12E-18	down	--
Solyc02g065170.3	373	243	345	42	26	33	144.6193	246.131	43.1080332	-2.517110823	1.24E-19	1.14E-18	down	Multicopper oxidases
Solyc02g014840.3	210	135	162	2	6	8	68.43752	130.151	6.72361263	-4.2663273	1.33E-19	1.23E-18	down	--
novel.2048	329	196	414	0	3	1	120.5336	239.236	1.83123993	-7.131263113	1.33E-19	1.23E-18	down	FOG: Transposon-encoded proteins with TYA
Solyc09g061410.1	74	70	67	142	189	242	148.4875	54.2713	242.703764	2.161628774	1.40E-19	1.29E-18	up	--
Solyc03g026270.3	279	172	171	1	1	5	81.40679	160.065	2.74829938	-5.769229996	1.47E-19	1.35E-18	down	--
Solyc07g039310.1	15	14	8	98	60	94	58.01508	9.57605	106.454105	3.482299203	1.48E-19	1.36E-18	up	Flavin-containing amine oxidase
Solyc07g042400.2	59	88	83	290	473	279	259.4504	59.119	459.781743	2.957304472	1.72E-19	1.58E-18	up	--
Solyc11g066460.1	331	388	318	54	44	46	164.5409	267.187	61.8949068	-2.117482057	1.72E-19	1.58E-18	down	--
Solyc04g079730.1	379	390	580	36	51	30	198.1406	344.667	51.6142464	-2.757652215	1.74E-19	1.59E-18	down	--
Solyc06g050520.3	152	121	180	543	460	356	353.9979	115.852	592.14348	2.351225306	1.79E-19	1.63E-18	up	--
Solyc12g043100.2	230	217	272	34	27	32	111.983	184.233	39.7334632	-2.219363629	1.80E-19	1.64E-18	down	FOG: PPR repeat
novel.677	227	192	168	14	16	12	84.75547	151.192	18.3193649	-3.06911705	1.95E-19	1.77E-18	down	--
Solyc05g150116.1	300	181	240	14	14	27	103.8473	184.887	22.8073434	-3.002933841	1.96E-19	1.79E-18	down	--
Solyc06g084620.1	0	0	0	222	245	176	140.5132	0	281.026345	10.95010521	1.99E-19	1.81E-18	up	--
Solyc03g065355.1	169	188	226	15	17	23	86.32916	149.404	23.2540152	-2.681902491	2.16E-19	1.96E-18	down	--
Solyc04g007580.1	622	420	669	8	31	47	236.6142	437.445	35.7829733	-3.605945066	2.16E-19	1.96E-18	down	--
Solyc08g005335.1	159	122	212	7	4	6	66.50739	125.811	7.20388498	-4.126090756	2.22E-19	2.01E-18	down	--
novel.1621	3	1	2	54	99	155	65.02035	1.53488	128.50583	6.384726694	2.26E-19	2.05E-18	up	--
Solyc02g088100.3	1838	1353	1959	262	221	165	800.3944	1318.01	282.778888	-2.224480699	2.51E-19	2.27E-18	down	--
Solyc12g010410.3	41	46	61	130	249	323	167.0888	37.8699	296.307772	2.966979711	2.75E-19	2.48E-18	up	Transcription factor MEIS1 and related HOX domain proteins
Solyc09g074300.1	279	176	153	3	0	6	80.09735	156.696	3.49852371	-5.384347157	2.88E-19	2.60E-18	down	Histone 2A
Solyc05g010540.2	356	411	378	49	62	51	182.4414	294.552	70.3309998	-2.079481302	3.03E-19	2.72E-18	down	--
Solyc10g085210.2	12	15	18	159	172	80	97.16224	11.5287	182.795797	3.981644525	3.20E-19	2.88E-18	up	--
Solyc11g066890.1	729	1362	1613	4072	2711	4518	2850.573	948.227	4752.91988	2.325513851	3.22E-19	2.89E-18	up	Prephenate dehydratase
Solyc09g065475.1	24	27	23	92	73	139	72.82064	19.0575	126.583733	2.738700501	3.50E-19	3.13E-18	up	--
novel.525	247	159	172	6	7	16	80.22512	148.573	11.8772603	-3.614148016	3.55E-19	3.17E-18	down	--
Solyc12g009300.3	5550	2746	5603	11838	9730	15209	9511.957	3546.57	15477.3465	2.125635346	3.73E-19	3.33E-18	up	Glycosyltransferase
novel.584	293	177	213	16	3	8	93.37118	175.362	11.3802377	-3.938887698	3.74E-19	3.33E-18	down	--
Solyc08g036600.3	166	128	164	3	1	5	60.50979	117.397	3.62308127	-4.953032818	3.92E-19	3.49E-18	down	WD40 repeat protein
novel.436	4	7	9	54	103	226	80.72363	5.11382	156.333446	4.933777418	4.05E-19	3.61E-18	up	--
Solyc01g010060.2	556	377	250	10	23	27	165.5959	305.733	25.458324	-3.587286747	4.09E-19	3.64E-18	down	--
novel.1849	214	160	186	16	10	21	81.64402	143.748	19.5399769	-2.861334109	4.27E-19	3.79E-18	down	--
Solyc01g102770.1	72	47	66	171	137	283	146.1458	47.3888	244.902798	2.372282089	4.43E-19	3.93E-18	up	--
Solyc07g053230.3	210	126	228	0	6	5	74.38394	144.012	4.7556553	-4.944096939	4.83E-19	4.27E-18	down	Transcription factor, Myb superfamily
Solyc10g085850.1	218	427	190	919	1018	1815	888.9407	216.798	1561.08388	2.849168257	4.92E-19	4.35E-18	up	--
Solyc05g007930.3	168	120	186	7	2	3	63.16848	121.204	5.13281079	-4.571025839	5.87E-19	5.17E-18	down	Galactosyltransferases

Solyc04g055090.1	186	121	250	3	1	6	72.93887	141.89	3.98747308	-5.08364477	5.90E-19	5.19E-18	down	--
novel.1577	465	410	435	31	55	76	202.3369	336.529	68.1451125	-2.301125206	6.09E-19	5.36E-18	down	--
Solyc01g066910.3	1	1	8	156	143	66	82.34885	2.49509	162.202607	5.993436909	6.11E-19	5.37E-18	up	--
Solyc01g108900.4	7	11	9	68	82	68	50.78464	6.9542	94.6150761	3.763316961	7.03E-19	6.16E-18	up	--
Solyc06g076080.3	275	165	223	4	8	19	91.27983	169.975	12.5846032	-3.721325774	7.65E-19	6.69E-18	down	--
Solyc06g083740.1	28	38	23	119	86	133	82.79108	23.0189	142.563279	2.6367554	7.81E-19	6.82E-18	up	--
Solyc04g074950.3	335	166	274	16	15	9	107.9696	198.327	17.612022	-3.519627804	7.98E-19	6.96E-18	down	Phospholipase/carboxyhydrolase
Solyc01g007267.1	202	206	192	6	12	1	81.60705	154.358	8.85612996	-4.209933885	8.06E-19	7.03E-18	down	FOG: PPR repeat
Solyc07g037950.1	207	182	239	19	18	32	94.80736	160.843	28.7720547	-2.469780081	8.19E-19	7.13E-18	down	Type I phosphodiesterase/nucleotide pyrophosphatase
Solyc04g071030.1	1430	1421	2758	157	202	283	848.9362	1427.31	270.561035	-2.398852865	8.54E-19	7.42E-18	down	--
Solyc01g079170.3	118	63	73	504	546	286	328.4498	65.2721	591.627452	3.178804255	8.56E-19	7.43E-18	up	Glycosyl transferase, family 8 - glycogenin
Solyc01g088310.3	414	453	476	85	52	58	214.3037	344.869	83.7383228	-2.047516499	8.98E-19	7.78E-18	down	--
novel.1420	139	134	150	8	8	5	58.89543	108.558	9.2326816	-3.594972633	9.01E-19	7.80E-18	down	--
Solyc01g095690.2	165	189	211	12	16	7	80.2818	144.941	15.622624	-3.258699817	9.02E-19	7.81E-18	down	FOG: PPR repeat
Solyc11g012710.2	234	251	295	36	35	45	124.617	199.977	49.2569336	-2.023000767	9.05E-19	7.82E-18	down	Protein involved in Snf1 protein kinase complex assembly
Solyc01g079150.4	26	20	51	145	116	236	115.3726	24.6089	206.136282	3.063505704	9.89E-19	8.54E-18	up	Na+-independent Cl/HCO3 exchanger AE1
novel.2009	269	287	285	24	9	39	122.6285	216.148	29.1092077	-2.861120261	1.01E-18	8.75E-18	down	--
Solyc06g082225.1	24	34	33	92	119	205	98.25394	23.3826	173.125264	2.891483162	1.04E-18	8.96E-18	up	--
novel.955	7	6	5	57	48	99	44.55727	4.63891	84.4756431	4.19609432	1.08E-18	9.29E-18	up	--
Solyc08g066670.3	293	302	337	35	41	66	149.2804	239.155	59.405467	-2.00102991	1.18E-18	1.02E-17	down	Acetylglucosaminyltransferase EXT1/exostosin 1
Solyc08g005690.1	63	77	43	204	144	245	148.1443	47.3762	248.912456	2.398267048	1.25E-18	1.07E-17	up	--
Solyc04g071650.4	2107	1429	1420	214	171	357	791.1919	1275.08	307.299882	-2.049470324	1.29E-18	1.10E-17	down	--
Solyc08g005333.1	158	132	145	5	0	5	57.85999	111.711	4.00891378	-4.730823657	1.35E-18	1.15E-17	down	--
Solyc08g080090.3	8278	15517	12955	28799	30102	30461	23937.56	9460.61	38414.5148	2.021637743	1.39E-18	1.18E-17	up	--
Solyc06g034230.2	245	247	227	29	33	30	112.3816	185.012	39.7514211	-2.232692717	1.48E-18	1.26E-17	down	--
Solyc11g008860.3	0	0	0	196	204	144	118.9734	0	237.946718	10.70979686	1.53E-18	1.30E-17	up	Multicopper oxidases
Solyc01g068110.2	1	0	2	122	172	121	91.15187	0.75133	181.552397	7.879395927	1.56E-18	1.33E-17	up	--
Solyc12g088240.2	327	690	699	53	46	52	252.475	440.328	64.6217655	-2.771626038	1.58E-18	1.34E-17	down	--
Solyc02g080150.3	362	313	257	33	27	52	143.4083	240.233	46.5839085	-2.354489332	1.60E-18	1.36E-17	down	--
novel.1361	175	131	137	4	1	7	59.33143	113.874	4.78925584	-4.500282237	1.60E-18	1.36E-17	down	--
Solyc03g111940.2	135	130	166	6	2	2	57.36535	110.4	4.33102804	-4.697244268	1.62E-18	1.38E-17	down	Cytochrome P450 CYP2 subfamily
Solyc02g075620.4	1	0	2	138	83	177	83.09572	0.75133	165.440099	7.752058625	1.80E-18	1.52E-17	up	--
Solyc01g058720.4	6	7	8	70	197	91	82.74255	5.38504	160.100048	4.888991414	1.83E-18	1.55E-17	up	Calmodulin and related proteins (EF-Hand superfamily)
Solyc09g011860.4	598	328	548	22	43	57	214.1888	376.96	51.4177571	-2.872719083	1.88E-18	1.59E-17	down	--
Solyc11g068910.3	271	240	329	44	35	40	132.9664	214.999	50.9341021	-2.084430166	2.21E-18	1.86E-17	down	--
Solyc08g077760.3	259	173	139	3	1	9	76.17779	147.275	5.08064852	-4.772627221	2.24E-18	1.88E-17	down	--
Solyc04g057950.2	3	7	2	67	49	70	40.95109	3.13104	78.7711394	4.671272105	2.49E-18	2.08E-17	up	--
Solyc08g075490.4	97	62	85	235	148	270	168.0324	62.5277	273.537168	2.130592601	2.77E-18	2.31E-17	up	Beta, beta-carotene 15,15'-dioxygenase and related enzymes
Solyc07g045470.3	427	346	478	49	56	35	190.9139	320.261	61.5670346	-2.39614654	2.81E-18	2.35E-17	down	N-acetylglucosaminyltransferase
Solyc01g113620.3	175	214	281	19	21	24	99.3716	171.419	27.3237683	-2.655291064	2.90E-18	2.42E-17	down	--
Solyc02g082920.4	389	243	331	661	912	795	635.7757	246.823	1024.72873	2.052521974	2.94E-18	2.45E-17	up	Predicted chitinase
Solyc11g072720.2	164	189	203	22	12	20	82.74487	142.712	22.7778295	-2.642354262	2.95E-18	2.46E-17	down	--
Solyc03g078260.3	2	0	1	129	160	109	87.56892	0.7638	174.374039	7.819322606	2.96E-18	2.47E-17	up	Pectin acetylesterase and similar proteins
Solyc03g083480.4	183	131	207	1	3	2	67.90844	133.184	2.63302269	-5.706058901	2.99E-18	2.49E-17	down	--
Solyc03g097380.4	35	29	28	90	93	111	74.47617	23.6674	125.284968	2.403782448	3.09E-18	2.57E-17	up	Copper chaperone

Solyc03g070430.1	157	113	160	4	7	4	58.36095	110.092	6.62977664	-4.10065482	3.14E-18	2.61E-17	down	--
novel.994	459	364	321	32	18	58	169.2972	294.662	43.932324	-2.725030977	3.14E-18	2.61E-17	down	--
novel.1810	51	50	39	145	102	149	101.8461	36.1032	167.588903	2.217895984	3.22E-18	2.67E-17	up	FOG: Transposon-encoded proteins with TYA
novel.2030	192	158	128	7	9	8	66.80802	123.239	10.3774155	-3.58888487	3.28E-18	2.72E-17	down	--
Solyc10g075070.3	3781	3866	3973	8214	6683	18279	8253.211	2985.33	13521.0958	2.179371553	3.33E-18	2.75E-17	up	--
Solyc01g150163.1	211	194	129	408	359	657	365.686	137.978	593.393751	2.10694853	3.44E-18	2.85E-17	up	--
Solyc04g076340.3	484	543	508	50	69	111	245.4302	394.806	96.0545451	-2.033338406	3.60E-18	2.97E-17	down	--
Solyc09g082240.4	138	79	45	341	421	641	328.1904	67.8076	588.57315	3.119301893	3.69E-18	3.04E-17	up	--
Solyc01g009245.1	233	213	151	14	14	17	86.65361	154.144	19.1634253	-3.010825599	4.03E-18	3.32E-17	down	Tousled-like protein kinase
Solyc12g098710.2	2608	5068	5172	11532	23364	17500	13070.77	3296.87	22844.6622	2.792653606	4.55E-18	3.74E-17	up	--
Solyc10g009160.3	408	379	501	53	25	76	196.4435	329.788	63.0992321	-2.370105507	4.93E-18	4.04E-17	down	--
Solyc08g014140.1	0	1	0	262	399	266	203.4407	0.26603	406.615449	10.52200827	5.66E-18	4.62E-17	up	--
Solyc10g085260.1	2	3	0	70	65	80	46.43301	1.3156	91.5504203	6.151621765	6.18E-18	5.04E-17	up	--
Solyc04g078160.4	196	204	224	30	18	31	96.68678	160.155	33.2189632	-2.262189745	6.30E-18	5.13E-17	down	--
Solyc05g007430.4	160	163	173	18	14	19	74.50664	127.372	21.6417727	-2.557736473	6.32E-18	5.15E-17	down	--
Solyc08g014290.1	456	484	526	44	81	68	229.9635	376.298	83.628744	-2.179453836	6.44E-18	5.24E-17	down	Serine/threonine specific protein phosphatase PP1, catalytic subunit
Solyc12g008600.2	191	119	178	8	7	13	68.28904	124.919	11.6588667	-3.404930934	6.55E-18	5.32E-17	down	--
Solyc12g005955.1	66	49	46	190	110	234	131.7995	41.4426	222.156395	2.426611045	6.83E-18	5.54E-17	up	--
novel.1151	149	144	174	1	1	4	61.05043	119.717	2.38390757	-5.564715567	6.90E-18	5.60E-17	down	--
Solyc12g011280.2	45	28	59	124	138	221	117.933	33.6239	202.242081	2.586744268	7.24E-18	5.86E-17	up	--
Solyc08g007780.3	3	1	0	86	64	106	54.28811	1.0423	107.533913	6.710900213	7.35E-18	5.95E-17	up	--
Solyc03g032210.3	195	196	185	18	10	28	85.56394	148.162	22.9655015	-2.661247688	7.36E-18	5.95E-17	down	Acyl-CoA synthetase
Solyc08g083190.3	6	9	19	66	85	150	66.85677	8.62627	125.087271	3.852330529	7.59E-18	6.14E-17	up	Predicted hydrolase/acyltransferase (alpha/beta hydrolase superfamily)
novel.808	62	101	101	264	172	362	199.6336	67.7868	331.480338	2.292304214	7.95E-18	6.42E-17	up	--
Solyc11g066080.2	535	361	505	79	43	76	221.0596	358.847	83.2724854	-2.10360115	8.11E-18	6.54E-17	down	--
Solyc12g005320.3	0	0	0	152	185	129	101.9728	0	203.945601	10.4868837	8.77E-18	7.06E-17	up	Pectin acetylesterase and similar proteins
novel.832	23	41	31	96	160	184	105.8815	24.4935	187.269524	2.935826689	8.85E-18	7.12E-17	up	--
novel.372	142	89	65	271	380	554	291.3177	76.4287	506.206772	2.72894274	9.09E-18	7.30E-17	up	--
Solyc05g050830.3	173	112	162	2	2	4	58.88452	114.459	3.31024788	-5.082133934	9.14E-18	7.34E-17	down	--
Solyc10g080880.2	60	76	110	215	224	223	173.8292	62.8352	284.823087	2.176419614	9.68E-18	7.75E-17	up	--
Solyc09g091070.3	808	896	1163	127	117	72	436.4309	733.87	138.991937	-2.408175836	1.02E-17	8.20E-17	down	NAD-dependent malate dehydrogenase
Solyc12g006140.2	26	17	16	81	101	226	91.17793	15.1908	167.165103	3.465062894	1.03E-17	8.23E-17	up	--
Solyc08g076230.1	317	297	276	31	33	61	140.4671	229.012	51.9223491	-2.128145745	1.17E-17	9.35E-17	down	--
Solyc10g050160.2	73	48	90	294	182	625	249.5756	53.8246	445.326606	3.049389487	1.19E-17	9.47E-17	up	O-methyltransferase
Solyc02g068600.3	15	7	9	71	63	171	66.06486	7.96014	124.169567	3.96854052	1.19E-17	9.47E-17	up	FOG: Ankyrin repeat
Solyc09g015700.4	205	178	214	28	14	22	90.10634	153.104	27.1088575	-2.496271936	1.25E-17	9.96E-17	down	--
Solyc02g089500.4	265	238	316	22	38	27	123.8768	209.712	38.0412559	-2.483240969	1.34E-17	1.06E-16	down	GATA-4/5/6 transcription factors
Solyc09g089680.4	5	9	13	69	49	106	49.8269	6.88978	92.7640265	3.750932622	1.36E-17	1.08E-16	up	Iron/ascorbate family oxidoreductases
novel.812	443	303	259	13	14	40	143.0657	259.024	27.107046	-3.231794407	1.42E-17	1.12E-16	down	--
Solyc02g091870.3	4	6	3	61	40	90	41.20207	3.37006	79.0340856	4.567452329	1.43E-17	1.13E-16	up	Alpha tubulin
Solyc10g008100.3	174	181	173	15	20	21	79.88734	135.783	23.9920797	-2.510019286	1.45E-17	1.14E-16	down	Protein kinase C substrate, 80 KD protein, heavy chain
Solyc01g106270.1	0	1	0	278	185	320	164.4609	0.26603	328.655696	10.21875457	1.46E-17	1.15E-16	up	Predicted regulator of rRNA gene transcription (MYB-binding protein)
Solyc01g105650.4	373	333	111	3	7	6	109.6813	212.442	6.92116932	-4.956472789	1.47E-17	1.16E-16	down	Iron/ascorbate family oxidoreductases
Solyc01g006140.4	203	182	172	23	16	18	83.87427	143.306	24.4422343	-2.560357564	1.47E-17	1.16E-16	down	DNA replication factor
Solyc09g007880.1	627	811	599	55	92	116	318.4125	525.516	111.309294	-2.238967713	1.49E-17	1.17E-16	down	Ubiquitin and ubiquitin-like proteins
Solyc05g007590.4	316	346	362	28	52	54	160.1593	262.969	57.3494717	-2.203021356	1.56E-17	1.22E-16	down	Cysteine desulfurase NFS1

Solyc03g117620.4	6	5	11	58	48	99	45.25244	5.59185	84.9130341	3.920598378	1.68E-17	1.32E-16	up	Molecular chaperones HSP70/HSC70, HSP70 superfamily
Solyc07g045100.1	390	339	643	12	38	35	193.0223	349.462	36.5824809	-3.263431399	1.71E-17	1.34E-16	down	Aspartyl protease
Solyc07g045200.1	10	14	5	70	55	84	47.83095	7.5434	88.1184939	3.558877827	1.82E-17	1.43E-16	up	--
Solyc11g065350.2	526	562	968	1486	1264	2414	1335.829	524.021	2147.63679	2.0350744	1.86E-17	1.46E-16	up	Transporter, ABC superfamily (Breast cancer resistance protein)
Solyc01g110110.3	35	16	33	91	124	169	91.72749	21.4405	162.014514	2.9150965	1.90E-17	1.48E-16	up	Cysteine proteinase Cathepsin F
Solyc05g056200.1	101	184	190	361	490	521	354.6049	121.878	587.331458	2.267959981	2.18E-17	1.70E-16	up	Serine/threonine protein kinase
novel.1892	369	341	315	51	54	43	164.0783	263.778	64.3790522	-2.048692475	2.27E-17	1.76E-16	down	--
Solyc03g097170.3	372	537	802	1645	1131	2612	1330.47	436.638	2224.30126	2.348972234	2.28E-17	1.77E-16	up	Flavonol reductase/cinnamoyl-CoA reductase
Solyc01g020495.1	187	203	232	28	11	22	92.58604	159.53	25.6420094	-2.629725891	2.34E-17	1.82E-16	down	FOG: Transposon-encoded proteins with TYA
Solyc01g090490.1	179	156	145	14	4	13	68.17299	123.53	12.8163643	-3.244049199	2.41E-17	1.87E-16	down	Mitochondrial transcription termination factor, mTERF
novel.545	170	239	218	12	3	21	87.81396	161.26	14.3677675	-3.440811349	2.62E-17	2.02E-16	down	--
novel.989	415	277	253	31	33	40	143.8274	243.385	44.2701211	-2.46096951	2.89E-17	2.23E-16	down	--
Solyc01g087590.3	222	138	134	4	1	2	65.0629	127.159	2.96729677	-5.419893583	2.91E-17	2.24E-16	down	Amine oxidase
Solyc02g021680.3	200	456	429	1177	957	1167	843.3483	278.718	1407.97894	2.336668194	2.91E-17	2.24E-16	up	--
novel.690	595	518	485	36	60	98	246.003	411.213	80.793434	-2.341707879	2.94E-17	2.26E-16	down	--
Solyc02g084390.3	512	456	728	74	52	116	266.576	433.09	100.061748	-2.105057942	3.10E-17	2.38E-16	down	Kinesin-like protein
Solyc06g064430.1	125	131	144	9	6	3	55.31158	102.66	7.96339017	-3.7331757	3.39E-17	2.59E-16	down	--
Solyc04g064580.1	458	284	630	50	28	53	202.0486	349.224	54.8728957	-2.665179103	3.62E-17	2.76E-16	down	--
Solyc03g114890.4	44	32	50	126	100	124	90.7017	32.2126	149.190781	2.207457593	3.64E-17	2.78E-16	up	--
Solyc01g099440.4	180	130	177	12	14	10	70.2454	124.753	15.7379007	-3.014783086	3.82E-17	2.91E-16	down	ATP-dependent DNA helicase
Solyc10g009190.1	243	389	322	1007	697	2351	941.8015	245.668	1637.93554	2.737987857	3.91E-17	2.98E-16	up	--
Solyc01g010020.3	136	106	154	2	6	4	53.29217	101.318	5.26604538	-4.307085244	3.99E-17	3.03E-16	down	Calmodulin and related proteins (EF-Hand superfamily)
Solyc10g085230.2	3397	7318	6861	18938	22195	16518	14835.07	4515.57	25154.565	2.477809033	4.01E-17	3.05E-16	up	UDP-glucuronosyl and UDP-glucosyl transferase
Solyc04g008100.3	478	330	793	29	39	64	230.9281	406.782	55.0744389	-2.880134124	4.12E-17	3.13E-16	down	--
Solyc05g044555.1	201	160	197	21	20	26	85.76589	143.093	28.4383844	-2.332348597	4.61E-17	3.49E-16	down	FOG: Transposon-encoded proteins with TYA
novel.1476	0	0	0	121	109	135	77.70634	0	155.412681	10.09740647	4.73E-17	3.58E-16	up	--
Solyc07g006360.1	5	8	17	72	56	94	50.36753	7.60891	93.1261432	3.605761338	4.83E-17	3.65E-16	up	FOG: Predicted E3 ubiquitin ligase
Solyc02g032523.1	419	375	256	0	0	0	135.6147	271.229	0	-10.16791181	4.90E-17	3.70E-16	down	--
Solyc01g109550.2	5	2	6	47	54	64	36.79264	3.30357	70.2817165	4.398205637	5.07E-17	3.83E-16	up	Mitochondrial transcription termination factor, mTERF
Solyc01g067440.4	34	39	39	108	91	201	96.87672	28.7781	164.975369	2.524932532	5.09E-17	3.84E-16	up	Glutaredoxin and related proteins
Solyc06g062540.3	14	15	20	135	377	107	147.4552	12.5388	282.371615	4.489220739	5.25E-17	3.95E-16	up	Predicted haloacid dehalogenase-like hydrolase
Solyc12g077520.1	4	3	4	49	52	57	35.22306	2.81826	67.627857	4.579256733	5.39E-17	4.06E-16	up	--
Solyc05g055080.2	167	99	221	2	4	6	64.49792	123.979	5.01693026	-4.613933537	5.39E-17	4.06E-16	down	--
Solyc02g088710.3	1	2	0	104	76	116	62.85454	0.79081	124.918261	7.340832316	5.42E-17	4.08E-16	up	Acyl-CoA synthetase
Solyc02g038805.1	325	580	479	882	1272	1443	944.952	356.364	1533.5398	2.105449079	5.58E-17	4.20E-16	up	FOG: Predicted E3 ubiquitin ligase
Solyc02g061770.4	3	5	3	40	134	102	61.51405	2.84527	120.182819	5.40454686	6.44E-17	4.83E-16	up	Predicted chitinase
Solyc01g105620.4	35	30	52	199	157	122	119.053	29.8443	208.261651	2.797055781	6.47E-17	4.85E-16	up	FOG: Predicted E3 ubiquitin ligase
Solyc10g008620.3	164	144	127	7	10	4	60.71577	112.023	9.40879759	-3.627752541	7.02E-17	5.25E-16	down	--
Solyc10g086660.2	353	557	744	1298	2655	2192	1543.699	422.757	2664.64088	2.655650676	7.79E-17	5.80E-16	up	--
Solyc08g080250.4	334	312	379	63	46	42	164.0603	262.769	65.3517568	-2.018418662	8.56E-17	6.37E-16	down	--
Solyc09g010850.4	1036	688	1243	110	153	172	471.4167	757.236	185.59765	-2.030409729	8.57E-17	6.37E-16	down	Serine/threonine protein kinase
Solyc05g009720.3	598	467	683	2807	2710	1267	1730.84	447.187	3014.49361	2.752509492	8.69E-17	6.46E-16	up	--
Solyc08g061210.3	199	168	211	27	19	28	89.72735	148.152	31.3025643	-2.240880211	8.86E-17	6.58E-16	down	--
Solyc08g007510.3	186	212	201	7	10	27	85.91021	154.031	17.7898093	-3.078349255	9.15E-17	6.79E-16	down	ER vesicle integral membrane protein

Solyc05g026215.1	355	280	285	20	24	54	138.3492	236.539	40.1597617	-2.539928869	9.23E-17	6.84E-16	down	--
Solyc10g079150.3	0	1	3	54	86	128	56.6579	1.00489	112.310909	6.777905147	9.24E-17	6.85E-16	up	CCAAT-binding factor, subunit B (HAP2)
Solyc02g069170.3	320	240	331	49	24	41	138.1387	228.17	48.1070055	-2.242536323	9.34E-17	6.91E-16	down	Predicted bromodomain transcription factor
Solyc05g026500.1	214	150	207	15	15	27	84.99677	146.26	23.7336837	-2.6097411	9.74E-17	7.20E-16	down	--
Solyc07g006380.3	0	0	1	224	233	198	142.1483	0.24629	284.050354	10.00382501	1.06E-16	7.84E-16	up	--
Solyc12g036160.3	42	40	48	93	129	177	100.79	33.3307	168.249178	2.335784097	1.08E-16	7.98E-16	up	Multidrug resistance-associated protein
Solyc02g092375.1	6	6	2	49	45	80	38.11375	3.64128	72.586223	4.334511377	1.13E-16	8.31E-16	up	--
novel.608	21	24	6	124	88	238	98.64278	13.2963	183.989273	3.800489834	1.19E-16	8.72E-16	up	--
Solyc03g121470.4	1	1	2	60	72	88	47.26583	1.01736	93.514291	6.508799855	1.22E-16	8.93E-16	up	Phospholipase D1
Solyc08g078190.2	397	426	782	26	35	73	231.8689	408.652	55.0859949	-2.881596719	1.38E-16	1.01E-15	down	--
Solyc02g093525.1	35	93	60	215	209	365	188.9034	48.5744	329.232483	2.763639218	1.52E-16	1.11E-15	up	--
Solyc01g087630.2	7	2	3	66	41	144	52.23468	3.08222	101.387148	5.045736178	1.55E-16	1.13E-15	up	Flavonol reductase/cinnamoyl-CoA reductase
Solyc05g053550.3	2566	5678	5990	12371	11655	16797	10440.05	3649.74	17230.3576	2.239086925	1.55E-16	1.13E-15	up	--
Solyc03g026300.4	212	234	272	40	31	37	115.1165	184.097	46.1355654	-2.001951617	1.68E-16	1.22E-15	down	Predicted membrane protein
Solyc05g054360.4	2	2	2	56	58	58	37.76491	1.54215	73.9876816	5.581559702	1.71E-16	1.24E-15	up	--
Solyc01g110510.3	11	7	8	57	45	71	39.74233	6.67883	72.8058243	3.449220465	1.78E-16	1.29E-15	up	Equilibrative nucleoside transporter protein
Solyc01g006890.3	370	290	383	28	48	26	156.2036	267.217	45.1907035	-2.585639572	1.86E-16	1.35E-15	down	--
Solyc02g070850.1	159	136	167	18	9	15	68.09587	118.452	17.7394585	-2.735333119	1.89E-16	1.37E-15	down	--
Solyc07g047710.3	33	12	26	101	86	119	73.86179	18.1348	129.588757	2.83415485	1.89E-16	1.37E-15	up	--
Solyc08g075290.2	3	0	2	71	43	96	44.16502	1.26885	87.0611941	6.090312559	1.99E-16	1.44E-15	up	--
Solyc03g025260.1	29	12	25	79	122	130	79.21507	16.8535	141.576644	3.065732797	2.01E-16	1.45E-15	up	Predicted E3 ubiquitin ligase
Solyc03g116580.4	5	2	12	74	50	150	58.12724	4.7813	111.47317	4.53199686	2.15E-16	1.55E-15	up	--
Solyc05g009610.1	336	262	358	34	37	68	151.277	244.813	57.7410621	-2.073197382	2.20E-16	1.59E-15	down	Arylacetamide deacetylase
Solyc09g009760.1	126	87	200	639	588	382	405.5991	105.005	706.192717	2.747273256	2.26E-16	1.63E-15	up	--
Solyc09g097960.3	5	9	1	160	464	446	231.6541	3.93432	459.373809	6.877167223	2.32E-16	1.67E-15	up	Voltage-gated shaker-like K+ channel, subunit beta/KCNAB
novel.1893	163	108	116	3	5	5	52.52835	99.4778	5.57887876	-4.170133737	2.48E-16	1.78E-15	down	--
Solyc11g068700.2	270	171	441	0	1	2	112.593	223.968	1.217733	-7.46671774	2.48E-16	1.78E-15	down	--
Solyc10g007270.4	0	0	2	171	190	139	109.4186	0.49258	218.344694	8.730967268	2.58E-16	1.85E-15	up	--
Solyc01g111250.3	282	566	672	1262	1718	1721	1204.084	389.047	2019.1207	2.375360933	2.60E-16	1.86E-15	up	Glycosylphosphatidylinositol-specific phospholipase C
Solyc09g082250.3	111	55	41	247	248	382	210.972	53.4514	368.49268	2.787117541	2.68E-16	1.92E-15	up	--
novel.871	208	167	171	18	19	12	80.94958	140.363	21.5357768	-2.73263846	2.69E-16	1.93E-15	down	--
novel.953	550	640	291	1325	1178	1948	1124.802	384.244	1865.36061	2.280081584	2.72E-16	1.94E-15	up	--
Solyc12g019080.3	29	12	25	82	80	119	67.59907	16.8535	118.344633	2.80970239	2.72E-16	1.95E-15	up	Inositol polyphosphate 5-phosphatase and related proteins
novel.268	297	285	762	1619	1246	2342	1255.557	340.341	2170.77248	2.672766211	2.83E-16	2.02E-15	up	Predicted chitinase
novel.1953	2	1	1	54	68	98	46.80395	1.02983	92.578066	6.495112655	2.93E-16	2.09E-15	up	--
Solyc02g086260.1	297	317	325	27	24	64	144.0454	241.225	46.8654164	-2.342596548	2.93E-16	2.09E-15	down	--
novel.1397	197	196	192	28	12	27	89.17841	150.404	27.9529178	-2.414158691	2.96E-16	2.11E-15	down	--
Solyc12g009650.2	3	3	4	51	68	50	38.1673	2.55951	73.7750862	4.839175068	3.03E-16	2.15E-15	up	--
Solyc04g079560.3	212	141	234	17	13	25	86.44983	149.998	22.9017832	-2.698303767	3.12E-16	2.22E-15	down	Amino acid transporters
Solyc06g076140.4	2579	3868	2427	5403	7099	12329	6310.466	2294.07	10326.8615	2.17054415	3.18E-16	2.25E-15	up	Predicted metallothionein
Solyc01g099880.4	35	39	54	100	131	143	96.31532	32.7311	159.899491	2.284110077	3.52E-16	2.48E-15	up	Multitransmembrane protein
Solyc05g007080.4	20	16	19	70	108	85	64.25413	14.1111	114.397202	3.013129051	3.71E-16	2.62E-15	up	--
novel.1879	8	15	4	63	75	182	68.79588	7.04562	130.546142	4.225710778	3.87E-16	2.73E-15	up	--
Solyc11g007930.1	207	215	160	11	12	27	85.34102	150.165	20.5172718	-2.846888466	4.02E-16	2.83E-15	down	Histone H2B
Solyc12g099860.2	62	46	47	119	120	136	100.0682	39.8558	160.280734	2.006082616	4.66E-16	3.27E-15	up	Conserved ATP/GTP binding protein
Solyc04g074750.3	553	526	587	98	41	52	254.727	427.594	81.8596111	-2.387534809	4.77E-16	3.35E-15	down	FOG: RRM domain
Solyc07g039343.1	165	154	191	16	21	22	77.99353	130.704	25.2828118	-2.380045437	4.87E-16	3.41E-15	down	FOG: Transposon-encoded proteins with TYA

Solyc03g123670.3	5	1	1	50	45	96	40.32999	1.8061	78.853883	5.458707159	4.95E-16	3.47E-15	up	--
novel.463	3	1	4	34	99	114	53.4227	2.02745	104.817947	5.678333117	4.96E-16	3.47E-15	up	--
novel.683	145	140	128	11	12	12	60.66997	106.289	15.0513946	-2.833299746	5.04E-16	3.53E-15	down	--
Solyc05g043405.1	296	311	241	40	24	52	133.4305	218.682	48.178797	-2.169229394	5.06E-16	3.54E-15	down	--
Solyc07g008530.1	917	943	976	183	111	98	449.274	728.522	170.026321	-2.10338103	5.10E-16	3.56E-15	down	Tyrosyl-tRNA synthetase
Solyc03g114740.4	194	214	281	32	22	20	104.1885	176.336	32.0412327	-2.474143079	5.11E-16	3.57E-15	down	--
Solyc09g011800.3	2	4	1	74	77	46	44.30298	1.82791	86.778055	5.5822548	5.22E-16	3.64E-15	up	--
novel.1370	0	1	0	120	202	306	131.5123	0.26603	262.758581	9.895166769	6.39E-16	4.45E-15	up	FOG: Reverse transcriptase
Solyc06g008130.4	753	743	947	2610	2661	1510	1809.327	625.738	2992.91628	2.257427826	6.52E-16	4.53E-15	up	Histone H3 (Lys9) methyltransferase SUV39H1/Clr4
novel.1997	127	184	155	6	4	16	65.19816	119.986	10.4104122	-3.475112922	6.72E-16	4.67E-15	down	--
Solyc12g015680.1	526	622	378	79	54	59	238.5643	394.672	82.4562677	-2.262558374	6.76E-16	4.69E-15	down	--
Solyc02g080010.2	204	181	287	29	15	17	98.91773	171.622	26.2132388	-2.719390212	7.06E-16	4.88E-15	down	--
Solyc05g005950.3	426	302	248	29	30	51	148.7936	251.65	45.936801	-2.446079185	7.11E-16	4.92E-15	down	H+/oligopeptide symporter
novel.2085	72	128	99	229	239	258	194.0495	77.0646	311.034514	2.012626087	7.39E-16	5.10E-15	up	DNA-binding proteins Bright/BRCAA1/RBP1
novel.100	151	116	118	5	7	11	54.30573	98.9935	9.61791027	-3.349946031	8.38E-16	5.77E-15	down	--
Solyc04g007620.1	303	241	287	10	31	32	122.1963	213.201	31.191878	-2.778725168	8.46E-16	5.82E-15	down	--
Solyc11g018710.1	23	22	31	79	96	90	66.86362	19.439	114.288288	2.548320005	8.47E-16	5.83E-15	up	--
Solyc02g067920.4	556	418	562	77	83	57	244.2574	393.483	95.032234	-2.061500104	8.82E-16	6.05E-15	down	Predicted alpha/beta hydrolase
Solyc10g024420.3	2036	3805	5262	11026	16299	11588	9924.832	2835.03	17014.6307	2.585263784	8.86E-16	6.08E-15	up	Multidrug resistance-associated protein
Solyc11g021360.3	151	172	148	8	16	17	69.39835	121.28	17.5169783	-2.798602314	9.75E-16	6.67E-15	down	--
Solyc05g054440.4	0	0	0	93	135	86	69.01161	0	138.023219	9.922733267	1.01E-15	6.92E-15	up	--
Solyc01g108420.2	168	178	213	12	24	18	83.41303	143.284	23.5425289	-2.627992046	1.03E-15	7.01E-15	down	--
Solyc02g067850.2	8	6	3	56	46	55	35.7161	4.40509	67.0271137	3.934809974	1.04E-15	7.08E-15	up	FOG: Armadillo/beta-catenin-like repeats
Solyc09g092110.4	276	118	190	5	3	14	79.17915	149.603	8.75528821	-4.043954666	1.04E-15	7.12E-15	down	--
Solyc03g059360.1	181	177	156	13	19	24	78.03221	132.343	23.7215239	-2.479407985	1.05E-15	7.16E-15	down	Calcium transporting ATPase
Solyc01g066420.3	0	1	1	138	173	129	96.23353	0.51232	191.954736	8.542474091	1.08E-15	7.38E-15	up	--
Solyc01g109060.4	183	187	203	25	20	37	90.64625	147.096	34.1962581	-2.091474835	1.24E-15	8.44E-15	down	FOG: PPR repeat
Solyc03g113320.1	28	21	8	132	99	104	79.42021	14.8021	144.038341	3.286809979	1.29E-15	8.75E-15	up	--
Solyc06g076700.1	136	157	173	3	9	17	65.73628	119.565	11.907378	-3.301178915	1.30E-15	8.81E-15	down	--
Solyc12g044730.2	216	211	195	34	23	25	97.63825	160.05	35.226923	-2.192513898	1.39E-15	9.38E-15	down	40S ribosomal protein S15/S22
Solyc03g083190.3	158	135	150	17	14	16	66.9259	113.741	20.1112063	-2.507883589	1.46E-15	9.88E-15	down	--
Solyc12g009050.3	47	59	57	109	136	223	118.6639	41.8956	195.432102	2.224797605	1.49E-15	1.01E-14	up	CCAAT-binding factor, subunit B (HAP2)
Solyc05g008330.3	201	228	278	36	29	26	110.2662	181.133	39.3997929	-2.214614382	1.52E-15	1.02E-14	down	FOG: PPR repeat
Solyc01g006090.3	240	204	223	40	24	35	106.6389	171.294	41.9841362	-2.02809716	1.62E-15	1.09E-14	down	Nucleolar protein-like/EBNA1-binding protein
Solyc01g094700.4	199	160	202	370	336	828	385.8227	143.807	627.838059	2.128011358	1.70E-15	1.14E-14	up	--
Solyc09g090730.2	84	73	119	221	174	411	200.9848	70.4639	331.505624	2.235684248	1.75E-15	1.18E-14	up	Ammonia permease
Solyc08g068680.4	292	146	348	1	14	8	105.1516	200.105	10.1978167	-4.326319302	1.77E-15	1.19E-14	down	Glutamate decarboxylase and related proteins
Solyc02g087410.4	26	16	29	138	61	138	79.29921	18.1265	140.471932	2.954044701	1.81E-15	1.21E-14	up	Multidrug/pheromone exporter, ABC superfamily
Solyc10g084660.2	234	206	172	27	18	30	94.62741	157.712	31.5423986	-2.315117734	1.83E-15	1.23E-14	down	Actin depolymerizing factor
Solyc04g007390.4	169	114	180	3	12	9	64.42405	118.389	10.4590916	-3.52690273	1.83E-15	1.23E-14	down	Serine/threonine protein kinase
novel.1620	42	20	38	119	94	230	103.6841	25.5473	181.82088	2.833122196	1.96E-15	1.31E-14	up	--
Solyc01g099410.3	3314	3125	1969	312	260	605	1328.924	2173.8	484.049858	-2.164873709	2.06E-15	1.38E-14	down	Histone 2A
Solyc07g054470.1	163	209	149	404	393	1064	445.525	134.474	756.575933	2.494162183	2.14E-15	1.43E-14	up	--
Solyc02g069250.4	10	3	8	47	64	65	40.44578	5.35596	75.5356021	3.808747688	2.24E-15	1.50E-14	up	Alcohol dehydrogenase, class V
Solyc12g010420.1	269	253	361	10	25	41	128.6792	225.821	31.5377081	-2.830322815	2.37E-15	1.57E-14	down	--
Solyc03g115710.1	44	34	77	156	156	166	122.1963	39.3945	204.99813	2.373802642	2.43E-15	1.61E-14	up	Serine/threonine protein kinase
Solyc07g065745.1	303	256	352	58	30	48	145.364	233.2	57.527963	-2.017568987	2.48E-15	1.65E-14	down	--
Solyc07g021020.4	0	0	0	83	108	83	59.67725	0	119.354501	9.713008197	2.51E-15	1.67E-14	up	--

Solyc07g005033.1	3	0	0	79	116	77	60.05323	0.77627	119.330181	7.269551968	2.55E-15	1.69E-14	up	Plasma membrane H+-transporting ATPase
Solyc12g094610.3	263	344	508	43	46	41	170.4603	284.681	56.2395461	-2.348946619	2.72E-15	1.80E-14	down	FOG: Armadillo/beta-catenin-like repeats
Solyc04g016460.3	15	38	18	93	107	140	81.21669	18.4236	144.009794	2.971959451	2.75E-15	1.82E-14	up	--
Solyc11g073055.1	157	128	200	1	7	14	66.4478	123.934	8.96152193	-3.756933899	2.80E-15	1.85E-14	down	--
Solyc09g007790.1	3814	4594	3554	361	384	937	1885.714	3084.34	687.089818	-2.164636852	2.83E-15	1.86E-14	down	--
Solyc04g056640.1	39	20	41	108	85	167	87.58113	25.5099	149.652351	2.552866165	2.86E-15	1.88E-14	up	FOG: Leucine rich repeat
Solyc04g078960.1	13	17	18	96	92	62	60.94233	12.3195	109.565165	3.146628801	3.52E-15	2.31E-14	up	--
Solyc09g010600.1	111	101	140	2	3	9	47.84618	90.0712	5.62115632	-3.943487593	3.56E-15	2.33E-14	down	FOG: Predicted E3 ubiquitin ligase
Solyc03g117590.3	334	319	269	879	647	2546	933.0488	237.539	1628.55844	2.778257941	3.83E-15	2.49E-14	up	Molecular chaperone (DnaJ superfamily)
Solyc12g006120.3	264	281	302	11	6	38	119.5183	217.445	21.5918855	-3.290630117	4.12E-15	2.67E-14	down	CCAAT-binding factor, subunit A (HAP3)
Solyc08g062330.3	157	122	193	17	11	11	68.71815	120.614	16.8223991	-2.856338277	4.17E-15	2.71E-14	down	FOG: Ankyrin repeat
Solyc06g072530.3	7	20	16	60	77	117	58.79943	11.0725	106.5264	3.269769772	4.23E-15	2.74E-14	up	--
Solyc04g054340.1	542	503	300	19	23	63	195.2288	347.945	42.5129477	-3.016828504	4.33E-15	2.80E-14	down	--
Solyc08g044280.1	205	163	326	22	20	25	102.6046	176.698	28.5113835	-2.634829997	4.33E-15	2.80E-14	down	--
Solyc01g103130.2	31	25	15	92	78	96	65.86306	18.3665	113.359632	2.629209517	4.33E-15	2.80E-14	up	Vacuolar sorting protein VPS1
Solyc04g082270.3	154	99	224	7	7	11	65.92331	121.354	10.4926922	-3.524322122	4.38E-15	2.83E-14	down	--
Solyc08g150101.1	119	175	159	11	14	12	66.26803	116.507	16.0292934	-2.880099597	4.41E-15	2.85E-14	down	--
Solyc02g070380.1	12	7	8	52	50	59	37.81425	6.93758	68.6909147	3.306174716	4.47E-15	2.89E-14	up	--
Solyc07g062560.3	0	0	0	92	86	75	54.8095	0	109.618999	9.590718215	4.56E-15	2.94E-14	up	--
Solyc09g008145.1	1	5	2	44	40	66	32.46725	2.08147	62.8530361	4.934722783	5.27E-15	3.38E-14	up	--
Solyc09g025230.4	1	0	3	62	46	74	38.78626	0.99762	76.5749038	6.22612866	5.77E-15	3.69E-14	up	Phosphatidylinositol transfer protein SEC14
novel.1780	26	23	16	67	85	159	72.79556	16.7869	128.804188	2.946547447	5.83E-15	3.73E-14	up	--
Solyc07g052980.3	651	314	207	11	18	30	163.7547	302.965	24.5441435	-3.618451261	5.95E-15	3.80E-14	down	--
Solyc12g088190.2	39	33	35	115	74	192	91.96798	27.4905	156.44544	2.514691695	6.15E-15	3.93E-14	up	Amino acid transporters
Solyc10g017970.1	1997	1165	1087	6200	15756	26271	10541.51	1094.38	19988.6475	4.191020332	6.22E-15	3.97E-14	up	Predicted chitinase
Solyc12g070200.3	1	3	3	45	62	49	34.82418	1.7957	67.8526524	5.232908724	6.29E-15	4.01E-14	up	--
Solyc09g090990.2	20	24	30	71	94	194	83.32823	18.9485	147.70801	2.965212253	6.46E-15	4.11E-14	up	--
Solyc05g053970.2	0	1	1	107	88	216	84.52466	0.51232	168.537007	8.362785285	6.78E-15	4.31E-14	up	Amino acid transporters
Solyc07g045160.3	177	162	216	410	627	405	387.7875	142.095	633.480228	2.154121836	6.83E-15	4.34E-14	up	Pyrophosphate-dependent phosphofructo-1-kinase
Solyc06g075510.4	8	9	14	43	67	105	48.87045	7.91234	89.8285589	3.503018882	7.03E-15	4.46E-14	up	--
novel.582	470	308	378	18	33	7	161.6042	296.649	26.559109	-3.512644983	7.09E-15	4.50E-14	down	--
Solyc02g082940.3	0	2	2	53	59	64	38.18772	1.02463	75.3508091	6.194712701	7.80E-15	4.94E-14	up	--
novel.1745	312	248	193	18	14	39	111.5851	194.241	28.9296089	-2.724185256	7.84E-15	4.96E-14	down	--
Solyc10g076760.3	144	101	176	6	6	14	59.06805	107.477	10.6595273	-3.304510983	7.88E-15	4.98E-14	down	Serine/threonine protein kinase
Solyc06g082275.1	47	40	40	93	85	138	82.58917	32.6542	132.524125	2.025182652	8.04E-15	5.08E-14	up	--
Solyc06g069040.4	92	81	65	143	193	274	159.0601	61.3626	256.757491	2.067157968	8.19E-15	5.17E-14	up	Glutathione S-transferase
Solyc09g015140.2	2	7	3	33	91	101	49.42522	3.11857	95.7318672	4.949857239	8.48E-15	5.35E-14	up	--
novel.564	0	1	3	47	184	84	71.06893	1.00489	141.132971	7.106476956	8.64E-15	5.44E-14	up	--
novel.557	199	145	146	13	12	23	72.97962	126.025	19.9344864	-2.643667993	8.83E-15	5.55E-14	down	FOG: Reverse transcriptase
Solyc12g056000.1	2	1	0	75	50	110	49.05922	0.78354	97.3348888	6.984345101	8.89E-15	5.59E-14	up	Cysteine proteinase Cathepsin L
Solyc08g075560.1	46	61	27	124	156	201	119.2678	34.7803	203.755334	2.554429822	9.06E-15	5.69E-14	up	--
Solyc01g086660.2	131	47	19	1641	2520	3630	1661.867	51.0799	3272.65324	6.002020213	9.10E-15	5.72E-14	up	--
Solyc04g015280.3	150	174	155	16	15	26	73.54187	123.277	23.8066828	-2.359526141	9.21E-15	5.78E-14	down	--
Solyc12g088220.2	579	1006	936	1726	2934	2202	1819.937	647.97	2991.905	2.206792827	9.46E-15	5.93E-14	up	Branched chain aminotransferase BCAT1
Solyc01g103640.4	204	195	199	34	27	26	95.61016	153.673	37.5471123	-2.045855931	9.54E-15	5.98E-14	down	--
Solyc01g087060.4	7	15	10	50	54	97	45.94171	8.2646	83.6188192	3.347449439	9.91E-15	6.21E-14	up	FOG: Predicted E3 ubiquitin ligase
Solyc04g078880.3	658	571	701	66	120	83	306.2992	494.812	117.786248	-2.079910887	1.01E-14	6.33E-14	down	--
Solyc01g150101.1	8	2	9	58	38	89	40.59917	4.81871	76.3796222	3.980966749	1.04E-14	6.50E-14	up	--
Solyc08g082470.1	131	143	121	5	12	12	57.08353	101.74	12.4270489	-3.043723186	1.06E-14	6.64E-14	down	--

Solyc01g090920.3	211	156	184	22	7	23	81.42068	141.415	21.4262581	-2.700614416	1.07E-14	6.66E-14	down	--
Solyc03g115330.2	175	187	245	27	27	23	94.38125	155.37	33.3922003	-2.234606198	1.10E-14	6.89E-14	down	--
Solyc01g057320.3	17	27	14	74	67	141	65.76772	15.0296	116.505783	2.964474831	1.11E-14	6.93E-14	up	--
Solyc10g050430.1	170	174	150	10	10	26	72.97917	127.221	18.7375903	-2.731536393	1.13E-14	7.06E-14	down	--
Solyc01g088670.3	120	131	157	13	11	17	60.91346	104.568	17.2591862	-2.592798007	1.15E-14	7.15E-14	down	FOG: PPR repeat
Solyc05g009950.3	190	165	148	10	8	25	73.45218	129.509	17.3952997	-2.859583124	1.20E-14	7.45E-14	down	Cytochrome b
Solyc01g066810.4	1	0	0	131	155	131	90.53973	0.25876	180.820694	9.351448961	1.22E-14	7.54E-14	up	--
Solyc08g077470.4	303	235	349	30	38	62	140.5843	226.875	54.2940969	-2.055842537	1.25E-14	7.75E-14	down	FOG: PPR repeat
Solyc06g052020.2	2	1	5	43	37	84	34.76142	2.01498	67.5078496	5.050790074	1.29E-14	8.01E-14	up	--
Solyc01g088440.2	134	105	165	11	8	14	58.53416	103.244	13.8243807	-2.890502906	1.31E-14	8.09E-14	down	FOG: Predicted E3 ubiquitin ligase
Solyc05g009650.4	611	362	551	11	49	36	215.9977	390.107	41.8879249	-3.229261944	1.31E-14	8.11E-14	down	--
Solyc07g051930.3	3	0	9	41	111	120	59.46315	2.99287	115.933427	5.251643083	1.35E-14	8.32E-14	up	MEKK and related serine/threonine protein kinases
Solyc12g087840.1	27	31	45	85	142	138	91.60569	26.3163	156.895111	2.570948189	1.36E-14	8.39E-14	up	FOG: Predicted E3 ubiquitin ligase
novel.1731	254	231	220	23	28	50	111.6651	181.36	41.9701648	-2.098688937	1.37E-14	8.47E-14	down	--
Solyc01g011050.3	0	1	0	127	160	129	90.52656	0.26603	180.787093	9.351108093	1.41E-14	8.70E-14	up	FOG: Leucine rich repeat
novel.735	219	135	122	9	10	13	68.0959	122.629	13.5631058	-3.176222955	1.42E-14	8.73E-14	down	--
novel.1424	0	2	0	87	99	190	78.11275	0.53205	155.693444	8.244725331	1.42E-14	8.78E-14	up	--
Solyc07g006140.4	863	504	620	1191	3124	2877	1803.425	510.084	3096.7657	2.601811658	1.57E-14	9.68E-14	up	Cytochrome P450 CYP4/CYP19/CYP26 subfamilies
Solyc02g067530.4	461	322	331	26	50	60	172.0762	286.469	57.683142	-2.3130418	1.59E-14	9.78E-14	down	Prolyl 4-hydroxylase alpha subunit
Solyc08g080630.4	482	1265	1382	5640	7579	15072	6233.181	801.616	11664.7456	3.863103521	1.68E-14	1.03E-13	up	--
Solyc12g009450.3	9	5	7	46	38	79	36.43499	5.38297	67.4870128	3.652765004	1.70E-14	1.04E-13	up	Apoptotic ATPase
Solyc04g074440.1	2680	1863	3138	123	307	375	1151.242	1961.93	340.553473	-2.52644133	1.72E-14	1.05E-13	down	--
Solyc02g067040.3	70	101	122	233	253	218	190.0413	75.029	305.053697	2.020077093	1.72E-14	1.06E-13	up	--
Solyc01g089880.3	129	104	146	3	7	13	53.23828	97.0046	9.471912	-3.330013393	1.74E-14	1.07E-13	down	--
novel.1226	171	312	198	10	20	8	96.54058	176.013	17.0680314	-3.40137032	1.93E-14	1.18E-13	down	--
Solyc11g071720.2	51	63	54	133	99	217	114.454	43.2559	185.652007	2.10779436	1.96E-14	1.20E-13	up	Arylacetamide deacetylase
Solyc01g103120.3	33	26	20	85	65	122	66.8986	20.3815	113.415741	2.483439231	1.97E-14	1.20E-13	up	Vacuolar sorting protein VPS1, dynamin
Solyc12g057060.2	52	81	64	157	262	201	160.392	50.7661	270.017868	2.409312542	1.98E-14	1.21E-13	up	UDP-glucuronosyl and UDP-glucosyl transferase
Solyc10g007300.2	109	100	98	4	6	6	42.90657	78.9435	6.86961089	-3.536534753	2.18E-14	1.32E-13	down	--
Solyc01g066360.3	0	0	0	71	102	63	51.94214	0	103.884277	9.51202636	2.28E-14	1.38E-13	up	--
Solyc08g069010.4	197	179	215	36	20	29	93.81925	151.546	36.092424	-2.070611644	2.50E-14	1.52E-13	down	FOG: PPR repeat
Solyc09g011910.3	177	136	129	13	4	16	63.61155	113.751	13.4721488	-3.044906687	2.64E-14	1.59E-13	down	--
Solyc10g005610.2	115	88	143	0	5	4	46.14453	88.3867	3.90231412	-4.529324785	2.83E-14	1.71E-13	down	--
Solyc11g006730.1	5	4	3	71	104	36	49.06018	3.09676	95.0235971	4.937623152	2.92E-14	1.76E-13	up	--
Solyc12g014240.3	1	1	0	122	110	94	70.96187	0.52478	141.398957	8.099979838	2.98E-14	1.79E-13	up	--
Solyc01g008240.4	0	0	2	115	133	93	74.85562	0.49258	149.218664	8.181472375	3.01E-14	1.81E-13	up	Predicted transporter (major facilitator superfamily)
Solyc12g099350.1	137	155	152	18	11	23	67.87616	114.12	21.6324918	-2.382357647	3.08E-14	1.85E-13	down	--
Solyc04g150162.1	290	245	207	30	38	34	117.6446	191.198	44.0911261	-2.127924658	3.15E-14	1.89E-13	down	Uncharacterized membrane protein
Solyc01g005040.3	16	6	6	63	46	107	48.12562	7.21401	89.0372246	3.632724966	3.21E-14	1.92E-13	up	--
Solyc04g014220.1	143	122	152	10	12	21	62.3935	106.893	17.89353	-2.562937788	3.31E-14	1.98E-13	down	FOG: Predicted E3 ubiquitin ligase
Solyc07g008820.3	123	99	104	6	4	10	46.00095	83.7778	8.22406128	-3.316517025	3.32E-14	1.99E-13	down	--
Solyc12g049380.3	9	7	5	37	68	69	39.99875	5.42245	74.5750574	3.783708947	3.47E-14	2.07E-13	up	--
Solyc11g073075.1	272	183	283	5	11	31	103.8131	188.765	18.861544	-3.293074635	3.56E-14	2.12E-13	down	FOG: Zn-finger
Solyc07g062670.1	23	83	57	195	361	317	209.6921	42.0701	377.314162	3.165099994	3.80E-14	2.27E-13	up	--
Solyc03g005670.3	5	3	2	43	32	72	31.63742	2.58444	60.690401	4.56690608	3.82E-14	2.28E-13	up	Apoptotic ATPase
Solyc01g104110.4	7	3	2	33	62	76	37.77225	3.10196	72.44254	4.551603414	3.82E-14	2.28E-13	up	--

Solyc12g014580.2	0	0	0	67	77	64	45.13769	0	90.275371	9.309920076	3.83E-14	2.28E-13	up	--
Solyc01g099150.5.1	56	125	148	351	468	352	297.4065	84.1945	510.618446	2.59874585	3.99E-14	2.37E-13	up	--
Solyc07g053840.1	0	1	0	123	106	126	75.90356	0.26603	151.541088	9.098688605	4.22E-14	2.51E-13	up	--
Solyc04g054528.1	37	47	51	196	142	111	115.1225	34.638	195.606927	2.494004358	4.30E-14	2.55E-13	up	--
Solyc02g091830.3	132	138	143	19	11	16	62.80306	106.087	19.51914	-2.442301505	4.51E-14	2.68E-13	down	Hexokinase
novel.629	1	1	0	67	164	141	80.69846	0.52478	160.872136	8.286885096	4.54E-14	2.69E-13	up	--
Solyc03g033840.4	144	64	94	247	531	419	298.8929	77.4379	520.34785	2.747107827	4.62E-14	2.73E-13	up	AAA+-type ATPase
Solyc11g006950.3	5	4	6	40	36	92	36.22874	3.83563	68.6218619	4.164787523	4.72E-14	2.79E-13	up	--
Solyc06g083650.3	14	7	2	79	52	207	70.69286	5.97737	135.408357	4.512406434	4.82E-14	2.85E-13	up	--
Solyc06g054620.4	105	127	132	6	11	12	52.92027	93.4651	12.3754905	-2.923878563	4.94E-14	2.91E-13	down	CCCH-type Zn-finger protein
Solyc02g087110.3	817	1695	2426	4248	4243	8300	4108.459	1259.82	6957.10096	2.465275541	5.04E-14	2.97E-13	up	Peroxidase/oxygenase
Solyc03g098730.1	1090	632	1473	63	136	106	472.8179	812.958	132.678276	-2.619559955	5.06E-14	2.98E-13	down	--
novel.98	102	98	108	2	2	2	40.82227	79.0631	2.58146426	-4.955696711	5.29E-14	3.11E-13	down	--
Solyc06g068510.1	110	130	133	7	13	11	54.6148	95.8032	13.4263884	-2.855449589	5.60E-14	3.30E-13	down	MEKK and related serine/threonine protein kinases
novel.1088	89	96	116	5	1	3	40.45328	77.1375	3.76907953	-4.339748956	5.61E-14	3.30E-13	down	--
novel.737	163	153	160	17	23	17	73.58095	122.286	24.8761424	-2.32102077	5.62E-14	3.30E-13	down	--
Solyc09g009710.4	174	186	196	30	25	21	87.88753	142.777	32.9976907	-2.130546646	5.87E-14	3.45E-13	down	3-methyladenine DNA glycosidase
Solyc03g098780.2	6	0	2	73	59	42	39.06356	2.04512	76.0820081	5.208478585	5.88E-14	3.45E-13	up	--
Solyc03g120890.3	147	138	190	7	13	23	69.67149	121.544	17.7990901	-2.754268817	5.96E-14	3.50E-13	down	GATA-4/5/6 transcription factors
novel.329	185	140	186	24	16	28	79.72355	130.924	28.5235434	-2.190231595	6.91E-14	4.04E-13	down	FOG: Transposon-encoded proteins with TYA
Solyc02g071310.3	33	16	18	126	59	135	75.19039	17.2286	133.152167	2.953802412	6.95E-14	4.06E-13	up	--
Solyc07g054860.1	117	63	108	281	159	310	193.6224	73.6335	313.611267	2.091088184	7.14E-14	4.16E-13	up	Aromatic-L-amino-acid/L-histidine decarboxylase
Solyc02g020880.2	150	183	148	19	13	8	70.76459	123.947	17.5819043	-2.843553026	7.30E-14	4.25E-13	down	--
Solyc01g107270.4	216	155	222	25	28	31	93.86167	151.802	35.9215022	-2.086161313	7.62E-14	4.42E-13	down	Phosphoribosylformylglycinamidine synthase
Solyc01g068120.4	0	0	2	98	112	91	65.63944	0.49258	130.786297	7.99086361	8.21E-14	4.76E-13	up	--
Solyc12g015850.1	108	91	105	8	3	6	42.58344	78.0146	7.15232655	-3.438225556	8.27E-14	4.79E-13	down	Glucose-6-phosphate/phosphate
Solyc11g066940.2	21	32	19	64	126	190	88.73067	18.6263	158.835086	3.096518691	8.29E-14	4.80E-13	up	--
Solyc02g064690.3	414	587	566	68	45	23	231.4046	402.683	60.1263177	-2.754449888	8.38E-14	4.84E-13	down	--
Solyc01g073910.4	27	21	28	62	112	198	86.74962	19.4691	154.030147	2.985429171	8.39E-14	4.85E-13	up	Transcription factor HEX
Solyc09g075530.3	275	263	284	35	22	64	130.228	211.069	49.3866452	-2.074610536	8.49E-14	4.91E-13	down	SNARE protein TLG2/Syntaxin 16
Solyc07g062310.4	171	142	166	24	13	25	74.43538	122.907	25.9635198	-2.232540699	8.67E-14	5.00E-13	down	--
novel.486	110	97	110	9	6	4	44.84373	81.3597	8.32778199	-3.321964863	8.73E-14	5.03E-13	down	--
Solyc01g066510.3	102	82	106	5	2	5	39.65043	74.3141	4.98681253	-3.874309468	8.81E-14	5.08E-13	down	--
Solyc03g019920.1	0	2	2	88	31	79	41.72971	1.02463	82.4347868	6.330295541	8.81E-14	5.08E-13	up	--
Solyc04g005060.3	345	264	385	29	23	69	151.6984	254.324	49.073208	-2.354523735	9.68E-14	5.56E-13	down	--
Solyc03g034280.3	0	0	0	64	74	56	42.29061	0	84.5812155	9.215399588	9.72E-14	5.58E-13	up	--
Solyc02g065480.2	45	46	99	177	147	254	145.0566	48.2639	241.849275	2.323366209	9.87E-14	5.66E-13	up	--
Solyc12g036850.2	116	108	110	1	4	0	44.11585	85.8385	2.39318844	-5.320744117	9.89E-14	5.67E-13	down	--
Solyc02g070940.1	45	41	43	147	72	176	98.38769	33.1416	163.633783	2.308400541	1.04E-13	5.94E-13	up	--
Solyc01g109320.4	13	34	81	791	1043	765	583.5342	32.3581	1134.71017	5.130424684	1.07E-13	6.11E-13	up	Uncharacterized membrane protein
Solyc02g087680.1	1	3	5	47	33	50	28.60029	2.28828	54.9122943	4.575059841	1.07E-13	6.11E-13	up	Nucleosome-binding factor SPN, POB3 subunit
Solyc06g008950.2	4	4	3	39	34	50	27.37006	2.838	51.9021161	4.199706046	1.09E-13	6.23E-13	up	Copper chaperone
Solyc03g063650.1	133	134	124	14	12	7	57.57187	100.602	14.5416084	-2.823392807	1.12E-13	6.42E-13	down	--
Solyc11g006040.2	141	94	139	544	456	253	324.4091	95.7254	553.092716	2.528581876	1.14E-13	6.52E-13	up	--
Solyc02g032100.4	11	43	65	177	227	366	176.0357	30.2943	321.777108	3.407588996	1.16E-13	6.62E-13	up	Fe2+/Zn2+ regulated transporter
Solyc05g054930.3	92	76	90	194	129	325	166.2727	66.1897	266.355651	2.012698124	1.20E-13	6.81E-13	up	--
Solyc08g062437.1	803	645	592	1721	2050	1201	1358.951	525.172	2192.7306	2.061383048	1.23E-13	7.02E-13	up	Molecular chaperone (small heat-shock protein Hsp26/Hsp42)

Solyc11g010760.1	158	269	276	24	18	37	106.6008	180.421	32.7809684	-2.448052574	1.24E-13	7.02E-13	down	UDP-glucuronosyl and UDP-glucosyl transferase
Solyc12g005920.2	19	6	15	54	65	111	53.02759	10.2069	95.8483114	3.229957019	1.38E-13	7.78E-13	up	Ca2+-binding actin-bundling protein
Solyc11g042560.1	130	82	122	4	0	2	43.98911	85.4999	2.4783474	-5.075357752	1.41E-13	7.98E-13	down	--
Solyc03g096770.1	6	3	5	31	61	62	34.7797	3.58207	65.9773234	4.19717114	1.42E-13	8.03E-13	up	--
Solyc04g150103.1	69	65	41	148	112	168	112.9789	45.2438	180.714014	2.001755465	1.54E-13	8.66E-13	up	--
Solyc11g068390.1	0	0	0	47	59	75	38.36739	0	76.7347733	9.080033836	1.59E-13	8.95E-13	up	--
novel.809	124	83	120	8	4	8	46.04542	83.7208	8.37005954	-3.309200391	1.61E-13	9.04E-13	down	tRNA delta(2)-isopentenylpyrophosphate transferase
novel.645	36	56	49	95	110	170	96.78205	36.2809	157.283179	2.119210441	1.67E-13	9.37E-13	up	--
Solyc02g032860.3	273	254	304	534	1091	668	611.7538	213.083	1010.42426	2.244272971	1.70E-13	9.56E-13	up	Phosphoadenosine phosphosulfate reductase
Solyc11g066160.1	233	157	113	6	7	17	71.06448	129.887	12.2416521	-3.379416654	1.72E-13	9.64E-13	down	Histone H4
Solyc12g096310.2	889	1343	1200	127	88	266	539.1802	882.856	195.504417	-2.168956484	1.80E-13	1.01E-12	down	--
Solyc04g074430.2	468	272	456	45	52	82	190.3767	305.765	74.9880885	-2.02370522	1.85E-13	1.04E-12	down	--
Solyc08g008240.3	0	0	5	47	53	52	33.32575	1.23144	65.4200654	5.677832387	1.85E-13	1.04E-12	up	Tyrosine kinase
Solyc11g017130.2	3	2	2	45	34	44	27.07051	1.8009	52.3401109	4.862547812	2.00E-13	1.12E-12	up	Anion exchanger adaptor protein Kanadaptin
Solyc03g019960.3	280	231	247	42	17	47	119.2733	194.738	43.8089742	-2.137119968	2.03E-13	1.13E-12	down	DNA replication factor
Solyc06g060820.2	22	21	28	57	123	131	75.49135	18.1753	132.807384	2.866391615	2.05E-13	1.14E-12	up	--
Solyc06g005820.3	5	12	7	41	64	63	39.1963	6.21013	72.1824728	3.542016301	2.05E-13	1.15E-12	up	Copper transporter
Solyc04g079740.3	254	312	287	30	50	33	134.5019	219.41	49.5941268	-2.162927719	2.06E-13	1.15E-12	down	--
Solyc01g102740.3	9	5	11	145	122	37	71.46207	6.36812	136.556008	4.414582475	2.08E-13	1.16E-12	up	--
Solyc01g009160.2	1042	633	955	5	27	11	346.3113	673.226	19.3968977	-5.133038519	2.11E-13	1.18E-12	down	--
Solyc06g069150.1	40	23	53	175	93	384	145.7322	29.5222	261.942163	3.150144397	2.12E-13	1.18E-12	up	--
novel.444	1	0	0	90	83	138	65.24641	0.25876	130.234053	8.88436785	2.15E-13	1.20E-12	up	--
Solyc02g069500.1	161	96	137	8	7	1	54.11311	100.94	7.28616498	-3.855992158	2.19E-13	1.22E-12	down	FOG: PPR repeat
Solyc03g033330.3	383	331	379	779	516	1635	734.6542	280.502	1188.80604	2.084563302	2.36E-13	1.31E-12	up	FOG: Predicted E3 ubiquitin ligase
novel.1705	253	227	172	32	27	33	103.7192	168.215	39.2230731	-2.103202186	2.38E-13	1.32E-12	down	--
Solyc12g099410.2	0	4	1	48	62	44	34.32663	1.3104	67.3428662	5.702123406	2.44E-13	1.35E-12	up	--
Solyc02g150145.1	177	152	208	27	18	14	81.58816	137.464	25.7121296	-2.437265523	2.50E-13	1.39E-12	down	--
Solyc07g053600.3	331	299	484	27	25	73	167.5142	284.394	50.6338921	-2.472916064	2.57E-13	1.42E-12	down	--
Solyc09g074990.4	174	135	148	23	13	17	69.99956	117.388	22.6109944	-2.38028778	2.76E-13	1.53E-12	down	DNA mismatch repair protein - MLH2/PMS1/Pms2 family
Solyc05g009230.3	2	2	2	39	40	44	27.0958	1.54215	52.6494615	5.091672884	2.82E-13	1.56E-12	up	Transcription factor, Myb superfamily
Solyc09g061794.1	117	83	117	7	2	1	42.78732	81.1706	4.40402717	-4.244265773	2.94E-13	1.62E-12	down	--
Solyc10g017620.3	0	0	0	60	86	45	42.34537	0	84.6907343	9.216643238	3.18E-13	1.75E-12	up	Ribokinase
Solyc07g017230.3	0	0	0	55	72	50	38.74022	0	77.4804474	9.088426288	3.20E-13	1.76E-12	up	--
Solyc11g061720.1	32	41	64	115	96	179	98.70752	34.9498	162.465233	2.217286987	3.26E-13	1.79E-12	up	Putative serine/threonine protein kinase
novel.1469	166	147	168	15	16	32	74.74038	123.436	26.0445921	-2.225465141	3.29E-13	1.81E-12	down	--
Solyc07g062390.4	135	115	182	15	15	12	64.30884	110.35	18.2678065	-2.616002618	3.40E-13	1.86E-12	down	Tub family proteins
Solyc03g120870.3	1	4	5	40	36	46	27.20707	2.55431	51.8598386	4.337222774	3.43E-13	1.88E-12	up	--
Solyc02g072540.3	0	4	2	34	40	74	31.47547	1.55669	61.3942611	5.317726629	3.71E-13	2.03E-12	up	Serine/threonine protein kinase
Solyc02g091520.3	643	783	793	62	107	171	355.8669	569.987	141.746821	-2.004424297	3.75E-13	2.05E-12	down	Predicted Ca2+-dependent phospholipid-binding protein
Solyc10g005190.1	0	0	0	89	30	81	41.55601	0	83.112012	9.198488499	3.81E-13	2.09E-12	up	--
Solyc02g079380.2	176	129	185	15	21	25	75.68038	125.422	25.9385963	-2.276997147	3.89E-13	2.12E-12	down	FOG: PPR repeat
novel.275	216	109	110	304	447	484	319.9366	111.98	527.892854	2.23682582	4.01E-13	2.19E-12	up	Karyopherin (importin) alpha
Solyc03g121825.1	9	8	6	45	34	63	32.59916	5.93476	59.2635554	3.329322762	4.05E-13	2.21E-12	up	--
Solyc04g072038.1	435	411	356	50	49	21	181.5278	309.575	53.4802946	-2.549572531	4.06E-13	2.21E-12	down	--
Solyc10g074800.3	9	1	4	40	44	64	32.95524	3.58	62.3304862	4.119175477	4.16E-13	2.26E-12	up	--
novel.620	175	182	140	4	14	2	68.75175	128.18	9.32363863	-3.852120558	4.22E-13	2.29E-12	down	--

Solyc01g058160.3	245	232	249	38	40	25	115.8642	186.44	45.2886261	-2.062054864	4.58E-13	2.49E-12	down	--
Solyc06g008650.4	0	0	0	44	77	55	38.46793	0	76.935853	9.078857081	4.82E-13	2.61E-12	up	--
Solyc08g006410.4	0	3	1	56	36	54	31.4088	1.04437	61.7732282	5.908452523	4.87E-13	2.63E-12	up	UDP-glucuronosyl and UDP-glucosyl transferase
Solyc03g121080.3	3	3	7	73	29	53	34.36011	3.29837	65.4218368	4.300317615	5.13E-13	2.77E-12	up	--
Solyc07g006300.4	202	361	473	915	575	1215	694.447	264.799	1124.09466	2.086057141	5.44E-13	2.93E-12	up	--
Solyc09g007780.1	0	0	0	42	67	58	36.13238	0	72.2647528	8.989454375	5.57E-13	3.00E-12	up	--
Solyc07g007270.3	5	7	2	41	45	44	29.80877	3.64855	55.9689903	3.949686942	5.61E-13	3.02E-12	up	--
Solyc04g064590.3	719	367	695	76	27	86	266.315	454.849	77.7810407	-2.540419253	5.65E-13	3.04E-12	down	MEKK and related serine/threonine protein kinases
Solyc11g042710.3	99	83	116	1	2	8	40.29856	76.2667	4.33042419	-4.057546574	5.73E-13	3.08E-12	down	Dehydrogenases with different specificities
Solyc02g083780.3	379	252	362	64	32	42	156.6041	254.264	58.9438565	-2.11149024	5.82E-13	3.12E-12	down	Histone acetyltransferase SAGA
Solyc05g053060.1	251	196	121	10	18	12	82.219	146.89	17.5476999	-3.086891278	5.92E-13	3.18E-12	down	--
Solyc05g006770.4	145	151	164	24	17	28	73.54686	118.081	29.0124927	-2.017940299	5.94E-13	3.18E-12	down	Serine/threonine protein kinase
novel.2019	0	0	2	74	106	81	57.10194	0.49258	113.7113	7.789420345	6.12E-13	3.28E-12	up	--
Solyc10g007780.3	123	121	141	17	14	16	59.42716	98.7431	20.1112063	-2.303956999	6.13E-13	3.28E-12	down	--
Solyc08g080860.4	36	28	25	72	72	134	69.22312	22.9212	115.525006	2.33974169	6.25E-13	3.34E-12	up	--
novel.1191	90	99	106	7	5	10	42.44092	75.7314	9.1504016	-3.02916595	6.26E-13	3.35E-12	down	--
Solyc03g118305.1	4	9	7	53	30	69	34.07327	5.15329	62.9932363	3.620514088	6.37E-13	3.40E-12	up	--
Solyc02g086230.2	96	97	104	0	5	1	39.53424	76.2593	2.80913868	-4.885837725	6.41E-13	3.42E-12	down	FOG: PPR repeat
Solyc03g119660.2	101	126	133	12	2	4	50.04722	92.4103	7.68415733	-3.592104199	6.78E-13	3.61E-12	down	--
Solyc08g081840.3	226	203	217	24	21	51	102.6383	165.927	39.3493019	-2.055058162	6.81E-13	3.63E-12	down	--
Solyc01g099290.3	283	186	206	36	29	33	107.6977	173.445	41.9505356	-2.053137106	7.01E-13	3.73E-12	down	Cullins
Solyc07g008020.3	59	40	44	117	82	169	94.79763	36.7445	152.850805	2.061473055	7.13E-13	3.79E-12	up	--
Solyc03g120250.3	3	2	2	39	33	43	25.33166	1.8009	48.8624241	4.763651937	7.20E-13	3.82E-12	up	Sulfate/bicarbonate/oxalate exchanger SAT-1
novel.993	127	161	134	5	12	19	61.83651	108.695	14.9777916	-2.845180335	7.23E-13	3.84E-12	down	--
Solyc02g084590.4	110	133	163	13	14	18	61.54019	103.99	19.0904261	-2.446739632	7.35E-13	3.90E-12	down	GATA-4/5/6 transcription factors
novel.1956	140	116	125	0	0	0	48.93562	97.8712	0	-8.697687998	7.79E-13	4.12E-12	down	--
Solyc11g021060.2	0	0	4	56	49	45	32.9176	0.98515	64.8500437	5.98463468	7.80E-13	4.13E-12	up	--
Solyc07g056690.2	0	0	0	97	20	124	48.69525	0	97.3904938	9.427914294	7.87E-13	4.16E-12	up	--
Solyc06g053210.4	133	113	120	10	12	18	55.41538	94.0304	16.8003545	-2.47646947	7.90E-13	4.18E-12	down	Ubiquitin/60s ribosomal protein L40 fusion
Solyc09g008300.2	137	139	138	18	4	8	59.57966	106.415	12.743969	-3.061215312	8.24E-13	4.35E-12	down	FOG: PPR repeat
Solyc04g009520.3	74	61	63	124	120	255	128.3611	50.8919	205.830314	2.020714441	8.54E-13	4.50E-12	up	CCAAT-binding factor, subunit A (HAP3)
Solyc09g083280.3	18	13	7	49	62	125	53.568	9.84	97.2959939	3.314687329	8.72E-13	4.59E-12	up	--
Solyc02g086410.3	105	82	126	3	7	9	44.01522	80.0161	8.01434476	-3.316320172	8.76E-13	4.62E-12	down	--
Solyc12g010360.2	1	1	0	86	95	68	54.68462	0.52478	108.844455	7.720751261	9.07E-13	4.78E-12	up	Plasma membrane H+-transporting ATPase
Solyc01g066440.3	80	96	94	6	2	3	37.04287	69.3903	4.69541985	-3.891826173	9.26E-13	4.87E-12	down	--
novel.1018	1452	963	1081	31	42	10	467.9382	898.138	37.7389111	-4.583224482	9.73E-13	5.11E-12	down	--
Solyc03g115660.3	22	23	28	60	63	83	52.99958	18.7074	87.2917876	2.220893652	1.00E-12	5.26E-12	up	Cdc2-related protein kinase
Solyc02g064980.1	93	76	95	4	1	3	35.50579	67.6799	3.33168859	-4.322291426	1.02E-12	5.37E-12	down	MEKK and related serine/threonine protein kinases
Solyc09g073030.4	93	98	124	10	2	4	43.74212	80.6749	6.80937544	-3.568771949	1.08E-12	5.66E-12	down	--
Solyc06g069850.3	145	140	195	25	18	21	75.08898	122.79	27.3880904	-2.171912001	1.15E-12	6.03E-12	down	Transcription factor, Myb superfamily
Solyc01g049880.3	197	227	157	11	1	21	81.49157	150.031	12.9524778	-3.48406842	1.16E-12	6.04E-12	down	--
Solyc06g060830.3	112	100	111	10	11	8	47.79452	82.9216	12.667487	-2.739031007	1.18E-12	6.17E-12	down	Transcription factor HEX, contains HOX and HALZ domains
Solyc11g061980.2	14	19	10	55	51	66	42.09139	11.14	73.0427796	2.719223803	1.39E-12	7.23E-12	up	UDP-glucuronosyl and UDP-glucosyl transferase
Solyc07g063600.3	32	15	33	121	111	80	78.37359	20.3982	136.34903	2.733499372	1.43E-12	7.43E-12	up	--
Solyc01g097490.1	1	2	5	37	29	72	29.31073	2.02225	56.5992072	4.796938123	1.44E-12	7.45E-12	up	--

Solyc09g092720.3	7	15	9	37	77	138	56.06847	8.01831	104.118637	3.705634755	1.44E-12	7.47E-12	up	--
Solyc01g066380.4	0	0	0	50	60	42	33.25548	0	66.5109657	8.867893359	1.46E-12	7.54E-12	up	--
Solyc10g083880.2	193	366	405	2078	3510	1667	1739.802	247.053	3232.55183	3.709598581	1.51E-12	7.82E-12	up	Aquaporin (major intrinsic protein family)
Solyc08g016220.1	15	7	18	47	140	103	68.35969	10.1767	126.542643	3.628862118	1.52E-12	7.88E-12	up	FOG: Leucine rich repeat
Solyc05g053600.3	95	82	170	226	369	348	247.1731	88.2652	406.081023	2.199087037	1.58E-12	8.16E-12	up	Pleiotropic drug resistance proteins (PDR1-15)
Solyc11g007490.1	147	135	200	20	22	20	75.00062	123.209	26.7925413	-2.216269483	1.67E-12	8.62E-12	down	UDP-glucuronosyl and UDP-glucosyl transferase
Solyc09g082870.3	130	73	133	5	6	1	45.64992	85.8148	5.48504277	-4.041527252	1.70E-12	8.74E-12	down	Calcium transporting ATPase
Solyc04g076680.2	239	281	203	33	10	36	109.5174	186.593	32.4415001	-2.506266839	1.73E-12	8.88E-12	down	MADS box transcription factor
Solyc09g008175.1	467	1075	463	44	71	77	301.4347	520.851	82.0187766	-2.668446416	1.73E-12	8.88E-12	down	--
Solyc03g098480.1	333	526	450	13	49	50	192.3953	336.926	47.8641922	-2.818740695	1.76E-12	9.04E-12	down	--
Solyc08g061950.1	208	144	135	19	10	23	73.47965	125.378	21.5809334	-2.522539991	1.78E-12	9.11E-12	down	--
Solyc08g023540.1	103	104	91	5	9	4	42.38807	76.7311	8.04506633	-3.305973964	1.79E-12	9.19E-12	down	--
Solyc03g123380.1	0	0	3	66	44	52	35.03441	0.73887	69.329949	6.495664674	1.82E-12	9.31E-12	up	--
Solyc02g078350.3	180	141	147	17	21	27	73.91636	120.291	27.5421618	-2.126598702	1.85E-12	9.49E-12	down	FOG: PPR repeat
Solyc10g081010.3	226	119	218	18	21	18	84.26364	143.827	24.7000265	-2.556109594	1.86E-12	9.53E-12	down	FOG: Predicted E3 ubiquitin ligase
Solyc01g079580.3	169	109	147	7	10	21	62.2674	108.931	15.6034584	-2.779710605	1.86E-12	9.55E-12	down	--
Solyc02g093720.3	37	22	61	98	145	194	107.4521	30.4502	184.453983	2.595017703	1.91E-12	9.79E-12	up	--
Solyc08g080150.1	172	128	160	24	12	23	71.35484	117.964	24.7457868	-2.244351839	1.95E-12	9.98E-12	down	--
Solyc05g015865.1	82	81	104	6	1	3	36.29339	68.3803	4.20647048	-4.012979933	1.98E-12	1.01E-11	down	--
Solyc10g084430.2	1	0	1	55	61	174	58.89582	0.50505	117.286589	7.842108455	1.99E-12	1.02E-11	up	FOG: Predicted E3 ubiquitin ligase
Solyc08g078280.1	106	90	101	1	1	9	40.22586	76.2459	4.20586663	-4.06915789	2.01E-12	1.03E-11	down	FOG: PPR repeat
Solyc06g072420.3	2	0	0	63	107	74	53.67786	0.51751	106.838207	7.695239633	2.14E-12	1.09E-11	up	Purple (tartrate-resistant) acid phosphatase
Solyc03g120920.1	0	1	1	107	100	52	57.57823	0.51232	114.644143	7.798304998	2.16E-12	1.10E-11	up	--
novel.1230	666	453	504	69	49	135	260.1517	416.972	103.331389	-2.002413223	2.17E-12	1.10E-11	down	--
Solyc06g076560.2	2667	2498	2883	8623	8695	4060	5783.58	2064.69	9502.46767	2.202247552	2.21E-12	1.12E-11	up	Molecular chaperone (small heat-shock protein Hsp26/Hsp42)
Solyc11g069230.3	176	186	228	31	15	40	93.32258	151.176	35.4690324	-2.073438979	2.25E-12	1.15E-11	down	--
Solyc12g017460.1	285	153	203	17	15	36	96.16626	164.445	27.8879919	-2.541539699	2.37E-12	1.21E-11	down	--
novel.834	122	124	110	6	12	16	52.98475	91.6475	14.3220071	-2.672684192	2.43E-12	1.24E-11	down	--
Solyc08g068050.3	249	239	251	50	31	27	118.3475	189.829	46.8655567	-2.02968433	2.44E-12	1.24E-11	down	--
novel.1185	92	84	112	1	4	0	38.06473	73.7363	2.39318844	-5.098352125	2.81E-12	1.42E-11	down	--
Solyc05g012790.4	130	135	157	334	196	645	292.5874	108.219	476.955371	2.142269437	2.82E-12	1.43E-11	up	DHHC-type Zn-finger proteins
Solyc01g080680.3	113	88	81	1	4	7	38.77163	72.5993	4.94393112	-3.846348446	2.86E-12	1.44E-11	down	Predicted transporter (major facilitator superfamily)
Solyc06g005650.2	2289	2034	5040	412	442	445	1466.582	2374.69	558.475048	-2.088962621	2.92E-12	1.48E-11	down	FOG: Predicted E3 ubiquitin ligase
Solyc06g073480.3	4	2	2	31	30	58	25.71099	2.05966	49.3623256	4.591505016	3.03E-12	1.53E-11	up	--
Solyc01g105110.1	0	1	0	90	57	91	50.33049	0.26603	100.394954	8.507630267	3.06E-12	1.54E-11	up	--
Solyc11g044740.2	0	0	0	46	57	39	31.10069	0	62.2013784	8.77101586	3.08E-12	1.55E-11	up	Regulatory protein MLP and related LIM proteins
novel.839	147	142	131	22	12	22	65.79179	108.077	23.5066131	-2.191731357	3.22E-12	1.62E-11	down	--
Solyc10g051050.3	31	17	56	127	96	147	91.19474	26.3361	156.053386	2.562300326	3.27E-12	1.64E-11	up	Apoptotic ATPase
Solyc06g075650.3	0	3	4	29	47	51	28.01609	1.78324	54.2489404	4.916484468	3.29E-12	1.66E-11	up	Aquaporin (major intrinsic protein family)
Solyc12g010800.2	20	22	49	77	144	178	96.02272	23.0959	168.949555	2.866679848	3.41E-12	1.71E-11	up	--
Solyc12g006230.3	371	424	295	40	66	48	174.3533	281.45	67.2571034	-2.076210088	3.51E-12	1.76E-11	down	FOG: Predicted E3 ubiquitin ligase
Solyc01g108210.4	137	90	147	10	5	16	54.12276	95.5966	12.6489253	-2.886241579	3.52E-12	1.77E-11	down	Cytochrome P450 CYP4/CYP19/CYP26 subfamilies
Solyc01g099820.3	3	2	0	43	46	36	27.86296	1.30833	54.417587	5.394822068	3.59E-12	1.80E-11	up	--
Solyc02g093430.3	535	351	495	58	82	48	218.3384	353.723	82.9533304	-2.104489607	3.61E-12	1.81E-11	down	--
novel.1495	28	26	19	55	87	126	65.67493	18.8414	112.508466	2.58219905	3.69E-12	1.84E-11	up	--

Solyc01g109860.3	11	7	3	37	45	84	37.12124	5.44738	68.795099	3.670541936	3.69E-12	1.84E-11	up	Phosphatidylinositol transfer protein SEC14
Solyc12g010020.3	163	128	69	7	3	4	49.60448	93.2228	5.98615198	-3.96671211	3.81E-12	1.90E-11	down	Predicted aminopeptidase of the M17 family
Solyc04g076260.2	119	135	114	16	12	10	55.64612	94.7827	16.5095657	-2.540839847	3.86E-12	1.93E-11	down	--
novel.1739	123	89	89	2	1	9	41.03325	77.4232	4.64325757	-3.964781673	3.97E-12	1.98E-11	down	--
Solyc04g005460.1	157	161	136	25	13	23	71.31132	116.951	25.6721271	-2.18066982	4.04E-12	2.01E-11	down	FOG: PPR repeat
Solyc08g006290.3	0	0	0	55	62	32	33.01595	0	66.0319011	8.85715741	4.06E-12	2.02E-11	up	--
novel.1339	234	139	181	0	2	0	71.54154	142.105	0.97789875	-7.379594395	4.07E-12	2.03E-11	down	--
Solyc09g091670.3	3	5	2	25	122	63	48.07113	2.59899	93.5432813	5.175232272	4.09E-12	2.04E-11	up	Pleiotropic drug resistance proteins (PDR1-15)
novel.502	0	1	1	40	89	130	54.44769	0.51232	108.383068	7.722924888	4.15E-12	2.06E-11	up	--
Solyc12g041895.1	11	5	7	43	42	46	31.0031	5.90048	56.1057076	3.245274013	4.23E-12	2.10E-11	up	FOG: Transposon-encoded proteins with TYA
Solyc02g086210.4	382	119	240	17	16	22	106.4436	189.612	23.2754559	-3.025548004	4.27E-12	2.12E-11	down	Serine/threonine protein kinase
Solyc08g007460.3	186	351	183	499	984	767	582.724	186.575	978.872785	2.391381077	4.33E-12	2.15E-11	up	--
Solyc03g098710.1	0	0	0	48	70	33	33.62308	0	67.2461513	8.883156647	4.50E-12	2.23E-11	up	--
Solyc11g005980.3	0	0	1	78	86	67	50.41334	0.24629	100.580391	8.503898922	4.60E-12	2.28E-11	up	1,3-beta-glucan synthase/callose synthase catalytic subunit
novel.1234	19	20	11	59	88	67	53.09698	12.9461	93.247862	2.847929527	4.70E-12	2.33E-11	up	FOG: Reverse transcriptase
Solyc08g005770.3	6313	15157	12023	26543	33082	25678	22884.39	8626.84	37141.944	2.106131594	4.71E-12	2.33E-11	up	--
novel.1287	152	94	121	2	1	0	47.75115	94.1386	1.36373126	-6.201517326	4.73E-12	2.34E-11	down	--
Solyc04g078970.3	10	7	19	71	62	51	44.54142	9.12925	79.9536006	3.117538731	4.76E-12	2.35E-11	up	--
Solyc01g079670.2	93	76	119	2	7	2	39.30852	73.5908	5.02621113	-3.951930375	4.77E-12	2.36E-11	down	--
Solyc03g119950.1	194	146	182	20	28	18	81.43042	133.863	28.997454	-2.231086573	4.88E-12	2.41E-11	down	FOG: PPR repeat
Solyc07g055990.3	344	184	285	5	18	34	115.7655	208.154	23.3773651	-3.141149307	4.91E-12	2.42E-11	down	--
Solyc07g017560.4	0	0	0	48	43	39	28.11543	0	56.2308691	8.627180343	4.96E-12	2.45E-11	up	--
Solyc03g119850.2	14	8	13	54	35	71	37.77836	8.95257	66.6041577	2.897752334	5.03E-12	2.48E-11	up	Uncharacterized mRNA-associated protein RAP55
Solyc03g120690.3	158	223	130	11	19	20	76.8072	132.225	21.3891747	-2.633787432	5.05E-12	2.49E-11	down	Dynein light chain type 1
Solyc01g068360.4	23	23	39	73	63	104	61.1527	21.6753	100.630098	2.21400255	5.15E-12	2.53E-11	up	--
Solyc07g056320.4	354	390	495	38	50	103	197.9321	317.264	78.6006812	-2.003599395	5.24E-12	2.57E-11	down	--
Solyc11g018774.1	300	94	175	14	5	5	78.06219	145.734	10.3901791	-3.821432965	5.50E-12	2.70E-11	down	--
novel.877	1	4	2	43	239	294	122.3067	1.81544	242.797904	7.069699449	5.56E-12	2.72E-11	up	--
Solyc09g072820.4	9	6	14	47	38	56	33.4582	7.37302	59.543392	3.006679594	5.90E-12	2.88E-11	up	--
Solyc10g009240.3	27	47	64	105	163	356	145.3002	35.2522	255.348282	2.857534573	5.97E-12	2.91E-11	up	--
Solyc11g011590.2	114	131	97	8	11	4	49.28651	88.2379	10.3351379	-3.143301108	6.06E-12	2.96E-11	down	--
Solyc03g113460.1	158	233	210	19	32	26	94.00979	154.589	33.430995	-2.223676876	6.17E-12	3.01E-11	down	Reductases with broad range of substrate specificities
Solyc06g008620.1	40	22	40	67	255	279	140.8535	26.0544	255.652599	3.292972487	6.36E-12	3.10E-11	up	--
Solyc09g065580.4	123	158	155	16	19	10	65.98319	112.034	19.9322113	-2.522619919	6.43E-12	3.13E-11	down	--
Solyc06g074370.3	84	76	105	2	2	8	36.29089	67.814	4.76781513	-3.760533056	6.50E-12	3.16E-11	down	Sialyltransferase
Solyc06g007970.3	0	1	6	28	97	70	43.46311	1.74376	85.1824625	5.580048202	6.53E-12	3.18E-11	up	--
Solyc06g008660.3	0	0	0	40	52	38	28.38395	0	56.767894	8.639261657	6.76E-12	3.29E-11	up	--
Solyc09g011780.3	111	139	100	11	11	13	52.62776	90.3287	14.9268371	-2.601464649	6.84E-12	3.33E-11	down	Transcription factor, Myb superfamily
Solyc08g074630.2	4	1	0	31	42	50	26.80782	1.30106	52.3145836	5.344165811	6.86E-12	3.34E-11	up	--
Solyc06g075600.3	17	13	12	52	40	66	38.58243	10.8127	66.3521636	2.622813777	7.04E-12	3.42E-11	up	FOG: Ankyrin repeat
Solyc06g076310.1	19	26	45	143	130	84	89.81766	22.9161	156.719236	2.767018293	7.19E-12	3.49E-11	up	--
novel.1073	119	81	81	1	6	3	38.37697	72.2897	4.46426262	-4.073369045	7.24E-12	3.51E-11	down	--
Solyc04g076720.3	132	136	142	19	4	16	60.70256	105.309	16.0964944	-2.687750279	7.28E-12	3.53E-11	down	Uncharacterized conserved protein
Solyc08g008495.1	66	41	53	194	179	112	127.113	41.0384	213.187664	2.373077816	7.29E-12	3.53E-11	up	--
Solyc06g053700.1	191	167	125	19	17	26	75.366	124.635	26.0967544	-2.249978154	7.29E-12	3.53E-11	down	--
Solyc08g078100.1	180	148	150	13	8	30	71.71052	122.892	20.5294316	-2.545801802	7.43E-12	3.59E-11	down	Amino acid transporters
Solyc06g016790.3	112	122	89	9	3	12	46.56594	83.3558	9.77606836	-3.054302731	7.51E-12	3.63E-11	down	--

Solyc06g066630.1	156	88	139	8	13	7	55.20842	98.0106	12.4062121	-3.017617002	7.51E-12	3.63E-11	down	--
Solyc02g088950.3	89	75	115	4	7	7	39.51378	71.3046	7.72295208	-3.220275467	7.53E-12	3.64E-11	down	--
Solyc01g107460.2	47	111	87	201	215	579	233.5701	63.1177	404.022554	2.68062723	7.66E-12	3.70E-11	up	--
Solyc12g008960.2	104	77	173	6	8	6	49.36253	90.0028	8.72229153	-3.392769011	7.76E-12	3.75E-11	down	--
Solyc03g098090.2	10	13	11	40	41	80	37.7245	8.7551	66.6939071	2.937716756	7.93E-12	3.83E-11	up	--
novel.1986	102	69	101	1	3	1	35.94644	69.6243	2.26863088	-5.025666994	7.94E-12	3.83E-11	down	--
Solyc11g027860.2	0	0	0	32	43	49	26.43827	0	52.8765321	8.541320047	8.48E-12	4.09E-11	up	--
Solyc03g025380.3	187	331	402	617	517	1174	592.9518	235.451	950.453026	2.01384636	8.52E-12	4.10E-11	up	--
Solyc12g010160.1	19	19	13	53	46	80	43.9987	13.1727	74.8247362	2.514535609	8.69E-12	4.18E-11	up	--
Solyc03g095350.3	8	2	14	46	51	62	36.84942	6.05015	67.6486938	3.468215143	8.86E-12	4.25E-11	up	Zinc-binding oxidoreductase
Solyc07g055220.3	82	86	82	6	5	5	35.59157	64.2921	6.89105159	-3.238092112	8.89E-12	4.27E-11	down	FOG: PPR repeat
Solyc05g055970.4	6	6	3	38	27	71	29.79094	3.88757	55.6943076	3.856877713	9.01E-12	4.32E-11	up	FOG: Ankyrin repeat
novel.136	41	26	32	76	81	183	82.46865	25.407	139.530313	2.461937752	9.02E-12	4.33E-11	up	--
novel.572	90	189	109	262	361	384	265.7232	100.413	431.033607	2.102408889	9.09E-12	4.36E-11	up	--
novel.863	10	12	15	53	37	58	35.9409	9.47423	62.407572	2.719556702	9.18E-12	4.40E-11	up	--
Solyc11g027840.2	94	121	115	14	8	9	49.07513	84.8357	13.3145945	-2.682068751	9.22E-12	4.41E-11	down	Predicted hydrolase related to dienelactone hydrolase
Solyc12g027890.1	0	0	0	44	35	41	25.64925	0	51.2984939	8.49730423	9.42E-12	4.50E-11	up	Amino acid transporters
Solyc01g102700.4	425	376	738	79	49	107	244.6307	391.759	97.5023278	-2.000785183	9.45E-12	4.52E-11	down	--
Solyc01g099660.4	466	313	433	7792	8283	1974	4243.959	310.49	8177.42733	4.718957201	1.00E-11	4.78E-11	up	Molecular chaperones GRP78/BiP/KAR2, HSP70 superfamily
Solyc04g082730.2	19	14	18	67	45	60	43.12269	13.074	73.1714238	2.482627916	1.01E-11	4.81E-11	up	--
Solyc06g060620.4	146	152	170	962	671	3384	1051.02	120.084	1981.95701	4.045043787	1.04E-11	4.96E-11	up	H+/oligopeptide symporter
Solyc01g087360.3	14	7	3	50	39	81	38.33898	6.22366	70.4543096	3.512123023	1.04E-11	4.97E-11	up	Putative transcription factor HALR/MLL3
Solyc08g080980.4	100	86	88	7	8	7	39.97576	70.4275	9.52407428	-2.907213885	1.05E-11	5.00E-11	down	--
Solyc03g097100.1	123	132	170	17	20	14	65.56395	108.812	22.3161189	-2.310184734	1.05E-11	5.00E-11	down	Calmodulin and related proteins (EF-Hand superfamily)
novel.828	6	9	8	36	46	45	30.27624	5.9171	54.6353767	3.203846449	1.05E-11	5.01E-11	up	--
Solyc01g095160.3	167	181	148	29	19	18	78.17376	127.814	28.5334281	-2.175356698	1.08E-11	5.16E-11	down	FOG: PPR repeat
Solyc02g072190.4	11	22	13	49	141	97	68.81035	11.9007	125.720024	3.401175729	1.09E-11	5.20E-11	up	--
Solyc08g061847.1	100	95	137	11	11	12	49.72614	84.8898	14.5624452	-2.553297528	1.10E-11	5.22E-11	down	FOG: Transposon-encoded proteins with TYA
Solyc02g089490.4	121	125	173	15	14	25	64.84345	107.171	22.5159507	-2.238801071	1.13E-11	5.37E-11	down	--
Solyc12g009920.1	77	74	89	4	5	4	33.59094	61.53	5.65187789	-3.472785356	1.14E-11	5.40E-11	down	UDP-glucuronosyl and UDP-glucosyl transferase
Solyc11g005790.3	152	134	149	19	24	19	69.3222	111.676	26.9686573	-2.070175744	1.14E-11	5.43E-11	down	--
Solyc01g095610.4	117	103	96	11	6	14	47.08279	81.3191	12.846482	-2.640466681	1.15E-11	5.47E-11	down	--
Solyc06g066065.1	6	4	3	29	32	59	26.59268	3.35552	49.8298342	3.90272358	1.19E-11	5.62E-11	up	--
novel.408	0	0	0	43	52	32	27.94686	0	55.893716	8.616236382	1.20E-11	5.68E-11	up	--
Solyc03g096050.3	116	94	49	308	248	216	200.8875	67.0905	334.684487	2.318838863	1.22E-11	5.76E-11	up	Iron/ascorbate family oxidoreductases
Solyc03g112390.3	30	18	35	74	72	181	77.34875	21.1713	133.526203	2.659246108	1.24E-11	5.86E-11	up	Transcription factor, Myb superfamily
Solyc04g051390.2	114	85	86	9	5	4	40.56514	73.2914	7.83883261	-3.249661552	1.25E-11	5.91E-11	down	--
Solyc09g150108.1	3	2	0	34	29	51	24.47157	1.30833	47.6348064	5.212834097	1.29E-11	6.11E-11	up	CCCH-type Zn-finger protein
novel.1000	122	107	125	16	13	18	55.3665	90.8194	19.9136496	-2.188732153	1.31E-11	6.19E-11	down	--
Solyc06g073010.3	2	0	0	58	76	59	42.27273	0.51751	84.0279441	7.348315471	1.47E-11	6.93E-11	up	--
Solyc02g077770.3	167	166	101	14	7	19	64.35884	112.248	16.4695633	-2.746762681	1.51E-11	7.11E-11	down	--
Solyc04g011990.3	81	85	71	2	4	2	32.30875	61.0581	3.55936301	-4.156916047	1.55E-11	7.26E-11	down	Apoptotic ATPase
Solyc02g069910.2	129	120	145	22	10	19	61.22516	101.015	21.4355389	-2.228896437	1.57E-11	7.38E-11	down	--
Solyc02g094000.1	992	820	866	30	110	130	401.1962	688.115	114.277095	-2.5904295	1.66E-11	7.76E-11	down	--
Solyc06g076090.3	40	19	31	82	89	87	67.06219	23.0397	111.084639	2.264250017	1.67E-11	7.83E-11	up	Actin and related proteins
Solyc03g059350.3	109	124	95	9	12	12	49.38297	84.5893	14.1766127	-2.58806257	1.69E-11	7.92E-11	down	Calcium transporting ATPase

Solyc02g092760.3	158	132	156	8	14	28	67.48382	114.42	20.5473895	-2.456201287	1.70E-11	7.93E-11	down	--
Solyc01g086820.4	491	414	278	36	24	80	181.1427	305.653	56.6322039	-2.417393578	1.75E-11	8.19E-11	down	Histones H3 and H4
Solyc02g079180.1	1	4	5	49	23	49	26.54375	2.55431	50.5331907	4.304935066	1.76E-11	8.21E-11	up	Heat shock transcription factor
Solyc02g070280.3	192	141	157	17	25	14	75.30969	125.859	24.7608658	-2.373521461	1.76E-11	8.23E-11	down	Amino acid transporters
Solyc10g079180.2	0	0	0	31	37	46	24.20613	0	48.4122694	8.415109786	1.77E-11	8.27E-11	up	Serine/threonine protein phosphatase
Solyc09g084450.3	0	0	0	37	44	35	25.22548	0	50.4509508	8.469389983	1.78E-11	8.31E-11	up	--
Solyc09g064460.1	1	0	1	47	59	78	39.1665	0.50505	77.8279488	7.245178393	1.84E-11	8.58E-11	up	--
Solyc06g008667.1	1	0	1	50	77	63	41.49019	0.50505	82.4753331	7.324044818	1.85E-11	8.61E-11	up	--
Solyc03g097400.1	119	137	101	11	12	17	54.49317	92.113	16.8733537	-2.44378224	1.86E-11	8.65E-11	down	--
Solyc09g082260.1	2	2	1	56	27	36	26.05474	1.29586	50.8136312	5.303147698	1.90E-11	8.82E-11	up	--
novel.842	89	68	97	0	5	3	34.27358	65.0092	3.5379223	-4.249749962	1.93E-11	8.98E-11	down	--
Solyc07g051840.4	322	254	643	3175	2768	1653	1826.861	309.254	3344.46778	3.434580645	1.97E-11	9.15E-11	up	--
Solyc02g085240.4	125	147	117	8	17	17	59.13617	100.266	18.0059277	-2.486311053	2.01E-11	9.36E-11	down	Clathrin adaptor complex, small subunit
Solyc03g120360.1	3	1	0	41	38	37	25.51895	1.0423	49.995602	5.598132575	2.04E-11	9.47E-11	up	--
Solyc02g094190.3	0	2	2	38	34	40	24.42272	1.02463	47.8208071	5.539556408	2.06E-11	9.56E-11	up	--
Solyc09g018550.1	99	76	67	2	2	3	32.64111	62.3364	2.94585607	-4.39402668	2.07E-11	9.58E-11	down	--
Solyc03g115850.3	16	17	9	55	85	57	48.63335	10.8792	86.387532	2.988165927	2.08E-11	9.64E-11	up	--
Solyc06g072780.2	23	15	15	56	46	106	49.62362	13.6362	85.6110961	2.658227143	2.09E-11	9.70E-11	up	--
novel.794	3	0	0	46	39	46	28.36365	0.77627	55.9510324	6.1796646	2.13E-11	9.86E-11	up	--
Solyc03g118900.3	0	1	1	49	52	78	37.8962	0.51232	75.280085	7.197780812	2.24E-11	1.04E-10	up	--
novel.722	82	80	79	3	4	0	32.61252	61.9571	3.26797033	-4.357744069	2.29E-11	1.06E-10	down	--
Solyc05g009320.4	90	62	82	2	3	3	31.70615	59.9775	3.43480545	-4.14162455	2.32E-11	1.07E-10	down	--
Solyc02g085400.3	30	22	27	63	61	75	52.48802	20.2651	84.7109272	2.061324018	2.36E-11	1.09E-10	up	--
Solyc02g083690.2	12	14	13	58	32	81	40.28101	10.0312	70.5307915	2.822324673	2.36E-11	1.09E-10	up	--
Solyc01g102790.3	177	132	141	21	19	12	69.24512	115.642	22.8479497	-2.363843087	2.47E-11	1.14E-10	down	FOG: PPR repeat
Solyc08g066760.4	99	73	111	1	6	0	37.87303	72.375	3.37108718	-4.564964353	2.51E-11	1.15E-10	down	FOG: Ankyrin repeat
Solyc02g087860.3	0	0	0	39	28	42	23.02664	0	46.0532854	8.344825711	2.70E-11	1.24E-10	up	--
Solyc09g066360.1	257	215	164	37	24	25	100.5579	164.088	37.0280452	-2.154938213	2.76E-11	1.27E-10	down	--
Solyc12g013680.3	9	0	2	46	49	48	32.19535	2.82139	61.5693097	4.4442281	2.80E-11	1.28E-10	up	FOG: Leucine rich repeat
Solyc10g055770.2	140	108	117	18	8	16	55.6938	93.7727	17.614901	-2.402548038	2.84E-11	1.30E-10	down	Predicted transporter ADD1
novel.1963	2	8	7	32	74	52	36.74845	4.36975	69.1271381	3.979627351	2.91E-11	1.33E-10	up	--
Solyc02g069300.1	0	0	1	58	77	57	42.0172	0.24629	83.7881098	8.239831449	2.98E-11	1.36E-10	up	--
Solyc11g072040.2	130	121	140	14	19	24	62.23352	100.308	24.1589148	-2.054304044	2.98E-11	1.36E-10	down	--
Solyc04g015610.3	0	0	2	132	103	30	59.76086	0.49258	119.029144	7.861986884	3.02E-11	1.38E-10	up	--
novel.218	68	80	133	3	5	1	37.87768	71.634	4.12131151	-4.200682525	3.05E-11	1.40E-10	down	--
Solyc04g054690.3	505	182	361	12	37	27	150.5889	267.999	33.1783971	-3.024523582	3.06E-11	1.40E-10	down	Multicopper oxidases
Solyc01g067460.3	190	169	122	22	9	23	73.28691	124.17	22.4041568	-2.454893286	3.07E-11	1.40E-10	down	Glutaredoxin and related proteins
Solyc01g091140.4	76	76	85	5	4	7	33.75582	60.8181	6.6934949	-3.169413796	3.07E-11	1.40E-10	down	--
Solyc12g019070.1	5	1	9	40	31	57	28.59991	3.77641	53.4234016	3.808400898	3.08E-11	1.40E-10	up	Inositol polyphosphate 5-phosphatase
Solyc03g114910.4	122	141	169	21	7	22	65.66273	110.701	20.6244753	-2.403811346	3.11E-11	1.42E-10	down	--
Solyc10g079910.2	10	10	11	47	63	41	37.12913	7.95702	66.3012492	3.050034257	3.44E-11	1.56E-10	up	Glucose-6-phosphate/phosphate
Solyc03g006365.1	0	1	1	53	45	72	35.96648	0.51232	71.4206523	7.122440145	3.51E-11	1.59E-10	up	--
Solyc07g018390.3	200	130	198	30	12	21	80.87073	135.1	26.6413489	-2.339977785	3.65E-11	1.65E-10	down	--
Solyc01g104290.3	15	12	9	42	40	63	35.08768	9.29028	60.8850788	2.719245808	3.67E-11	1.66E-10	up	--
Solyc11g066270.3	7	7	22	53	59	191	65.3602	9.09184	121.628569	3.738398994	3.69E-11	1.67E-10	up	--
Solyc07g053890.3	10	4	13	45	36	102	40.65308	6.85343	74.4527348	3.440485549	3.72E-11	1.68E-10	up	--
Solyc05g009050.3	151	175	189	14	24	36	81.576	132.176	30.9763634	-2.085825674	3.89E-11	1.76E-10	down	Serine/threonine protein kinase
Solyc11g065910.1	0	2	0	61	44	64	36.02388	0.53205	71.515696	7.119757926	3.90E-11	1.76E-10	up	--
novel.1443	7	3	5	28	61	45	31.15566	3.84082	58.4704898	3.920019898	3.98E-11	1.79E-10	up	Defense-related protein containing SCP domain
Solyc01g109120.3	5	8	11	42	65	41	35.61169	6.13118	65.0921932	3.397000328	3.98E-11	1.80E-10	up	FOG: WD40 repeat

novel.196	234	179	174	14	21	4	84.43562	151.022	17.8489773	-3.120907524	3.99E-11	1.80E-10	down	--
Solyc12g100260.1	4	0	2	21	54	65	30.40077	1.52761	59.2739438	5.26890691	4.10E-11	1.84E-10	up	--
Solyc03g123680.1	29	13	29	68	64	82	54.51008	18.1047	90.9154727	2.323476261	4.10E-11	1.84E-10	up	FOG: Predicted E3 ubiquitin ligase
Solyc03g114730.3	494	394	511	48	38	122	221.2624	358.494	84.0306426	-2.07986684	4.11E-11	1.85E-10	down	--
Solyc12g096210.2	11	17	10	61	34	69	39.13992	9.83168	68.4481613	2.809281503	4.38E-11	1.97E-10	up	--
Solyc08g078990.3	102	79	91	7	9	7	39.91733	69.8216	10.0130237	-2.827589294	4.42E-11	1.98E-10	down	Uncharacterized conserved protein
novel.1710	178	177	160	32	25	20	83.02993	132.552	33.5080808	-2.000198701	4.49E-11	2.02E-10	down	--
Solyc11g007570.2	66	73	84	2	4	4	30.73717	57.1862	4.28814663	-3.751064206	4.60E-11	2.06E-10	down	--
Solyc05g021180.3	98	64	120	0	3	1	36.88491	71.9386	1.83123993	-5.394009342	4.74E-11	2.12E-10	down	--
Solyc09g009220.4	22	33	34	70	157	107	84.60886	22.8454	146.372342	2.675519114	4.88E-11	2.19E-10	up	--
Solyc01g009190.3	1	0	2	32	43	49	26.81393	0.75133	52.8765321	6.102782682	5.06E-11	2.26E-10	up	--
Solyc05g043390.2	75	65	82	3	1	5	30.25865	56.8942	3.62308127	-3.912768276	5.21E-11	2.33E-10	down	--
Solyc03g006800.1	6	4	7	33	27	65	27.83084	4.34067	51.321002	3.566812083	5.22E-11	2.33E-10	up	--
Solyc01g104090.4	86	95	80	2	4	10	36.85164	67.2288	6.4744975	-3.330061043	5.26E-11	2.35E-10	down	FOG: PPR repeat
Solyc06g008680.3	1	0	1	52	84	48	40.90596	0.50505	81.3068835	7.30247094	5.35E-11	2.39E-10	up	--
Solyc03g058890.4	0	0	1	59	76	49	40.53385	0.24629	80.8214169	8.187305557	5.49E-11	2.45E-10	up	--
Solyc05g012360.3	78	80	85	4	7	7	35.06136	62.3998	7.72295208	-3.027224926	5.56E-11	2.48E-10	down	Predicted hydrolase/acyltransferase
Solyc01g097280.2	30	26	63	88	129	234	108.5141	30.1956	186.832556	2.627808654	5.64E-11	2.51E-10	up	Predicted chitinase
Solyc07g056685.1	33	13	6	363	150	543	221.7276	13.4751	429.980073	4.997971451	5.69E-11	2.53E-10	up	--
Solyc08g005460.3	25	25	39	76	59	84	57.71175	22.7249	92.698637	2.025543258	5.85E-11	2.60E-10	up	--
Solyc03g034000.3	108	83	88	10	3	9	40.40986	71.6994	9.12028387	-2.952501371	6.04E-11	2.68E-10	down	--
Solyc06g005300.4	74	71	105	0	0	0	31.94813	63.8963	0	-8.08334604	6.10E-11	2.71E-10	down	--
Solyc02g083880.3	931	665	615	122	32	110	339.1848	569.278	109.091174	-2.378774516	6.11E-11	2.71E-10	down	--
Solyc02g068060.3	112	68	124	5	2	11	42.3918	77.6104	7.1731634	-3.380143799	6.17E-11	2.74E-10	down	--
Solyc05g007940.3	8	11	12	53	121	45	53.34702	7.95182	98.7422258	3.627473407	6.19E-11	2.75E-10	up	Ribonuclease, T2 family
Solyc01g099040.4	664	1750	1613	3035	3856	3347	2733.558	1034.63	4432.48969	2.098900586	6.21E-11	2.75E-10	up	--
novel.604	2	0	3	24	79	44	33.207	1.25638	65.1576229	5.66873742	6.40E-11	2.83E-10	up	--
Solyc09g074820.4	0	0	1	53	73	52	39.03484	0.24629	77.8233985	8.133004807	6.47E-11	2.86E-10	up	--
Solyc01g005070.3	1	0	0	58	36	94	38.74122	0.25876	77.2236826	8.133618593	6.53E-11	2.89E-10	up	Clathrin assembly protein AP180
Solyc02g084980.3	809	529	971	7930	7840	2818	4458.969	589.209	8328.7294	3.82114772	6.56E-11	2.90E-10	up	Glycosyl transferase, family 8 - glycogenin
Solyc12g096640.3	1	1	2	35	33	35	22.60754	1.01736	44.1977258	5.425583598	6.75E-11	2.98E-10	up	Predicted RNA-binding protein
Solyc10g078940.2	3	2	5	52	26	33	25.01086	2.53977	47.4819426	4.212297028	6.95E-11	3.07E-10	up	Serine/threonine protein kinase
novel.2011	264	254	246	14	21	56	116.6336	196.47	36.7973515	-2.396604257	7.03E-11	3.10E-10	down	--
Solyc03g114650.3	1	6	2	58	23	45	27.67982	2.3475	53.0121419	4.514050577	7.26E-11	3.20E-10	up	--
novel.904	283	315	256	10	24	53	127.7491	220.077	35.4214604	-2.620512087	7.29E-11	3.21E-10	down	--
novel.1946	80	76	72	6	1	4	31.61114	58.6514	4.57086229	-3.65780287	7.29E-11	3.21E-10	down	--
Solyc10g054840.2	45	27	22	86	67	134	71.72444	24.2452	119.203732	2.303552804	7.69E-11	3.37E-10	up	Aquaporin (major intrinsic protein family)
Solyc11g069820.3	10	6	6	40	27	68	30.56869	5.66147	55.4759141	3.302283227	8.09E-11	3.54E-10	up	Transporter, ABC superfamily
novel.585	0	0	0	38	27	33	20.92371	0	41.8474188	8.203944265	8.33E-11	3.65E-10	up	Tam3-transposase (Ac family)
Solyc05g050090.4	15	5	13	55	66	46	40.75121	8.41324	73.0891839	3.108112196	8.43E-11	3.69E-10	up	Delta 6-fatty acid desaturase
Solyc06g063180.3	173	134	195	13	28	22	77.9161	128.439	27.3932846	-2.246385431	8.49E-11	3.71E-10	down	Glutamate-gated kainate-type ion channel receptor
Solyc01g108270.4	0	0	1	57	36	83	36.51214	0.24629	72.7779817	8.047517079	8.65E-11	3.78E-10	up	FOG: Leucine rich repeat
Solyc07g054340.1	100	105	88	3	1	12	40.8279	75.482	6.17382395	-3.524481627	8.70E-11	3.80E-10	down	--
Solyc09g042660.4	100	102	89	8	6	15	43.41444	74.9302	11.898701	-2.624351812	9.03E-11	3.94E-10	down	Serine/threonine protein kinase
Solyc01g067330.3	63	96	91	0	5	4	34.07745	64.2526	3.90231412	-4.067100251	9.08E-11	3.95E-10	down	--
Solyc06g008500.3	85	76	102	9	6	9	38.7418	67.3339	10.149741	-2.727740883	9.28E-11	4.04E-10	down	--
Solyc12g100180.2	1	0	5	41	44	30	25.93438	1.4902	50.3785555	5.037592125	9.94E-11	4.32E-10	up	Pleiotropic drug resistance proteins (PDR1-15)
Solyc01g006290.4	0	1	0	19	109	134	55.35022	0.26603	110.434412	8.643764039	1.00E-10	4.34E-10	up	--
Solyc08g078090.1	146	124	347	15	17	19	89.01225	156.228	21.7964479	-2.846585326	1.05E-10	4.57E-10	down	Predicted lipase

Solyc01g110100.4	110	89	82	11	4	8	41.0088	72.3354	9.68223238	-2.893196638	1.07E-10	4.62E-10	down	Predicted E3 ubiquitin ligase
Solyc03g058450.4	0	0	0	29	53	27	24.21862	0	48.4372331	8.408920861	1.07E-10	4.62E-10	up	--
Solyc01g090460.3	14	25	40	67	85	157	74.10012	20.1248	128.075405	2.669567832	1.08E-10	4.65E-10	up	Transcription factor HEX
Solyc01g104750.4	13	1	4	49	50	55	35.2681	4.61502	65.9211746	3.833589499	1.09E-10	4.73E-10	up	--
Solyc03g025430.1	126	153	142	23	13	28	67.44893	108.279	26.6193043	-2.00898576	1.12E-10	4.84E-10	down	--
Solyc07g005037.1	0	0	0	39	36	25	21.88511	0	43.7702196	8.263940419	1.14E-10	4.90E-10	up	Plasma membrane H+-transporting ATPase
Solyc07g065810.2	63	162	84	225	424	375	261.2304	80.0864	442.374426	2.466004384	1.17E-10	5.03E-10	up	FOG: Predicted E3 ubiquitin ligase
Solyc08g007820.1	117	101	104	12	13	17	50.27852	82.7574	17.799694	-2.216695747	1.17E-10	5.03E-10	down	--
novel.421	2	2	0	27	31	48	22.75368	1.04957	44.4577931	5.433878864	1.19E-10	5.10E-10	up	--
Solyc06g083160.1	4	0	2	53	58	23	30.7247	1.52761	59.9217954	5.2787763	1.20E-10	5.14E-10	up	--
Solyc03g116890.3	543	232	353	41	38	73	176.1385	289.163	63.1137072	-2.189619295	1.20E-10	5.15E-10	down	--
Solyc01g110060.3	174	200	331	28	33	43	111.9009	179.751	44.0511237	-2.029349302	1.21E-10	5.20E-10	down	Purple acid phosphatase
novel.311	101	64	73	3	0	5	32.13671	61.1393	3.13413189	-4.196293531	1.23E-10	5.27E-10	down	--
Solyc07g064200.4	5	4	3	30	39	32	23.47403	3.09676	43.8512919	3.821744829	1.24E-10	5.32E-10	up	--
Solyc05g012325.1	3	3	0	34	30	33	21.56953	1.57435	41.5647031	4.744473189	1.24E-10	5.34E-10	up	--
Solyc04g076150.3	3	0	0	37	51	37	27.68933	0.77627	54.60238	6.139882629	1.28E-10	5.50E-10	up	--
Solyc05g006050.3	104	78	106	151	173	397	184.5326	73.7675	295.297824	2.003637669	1.34E-10	5.73E-10	up	G1/S-specific cyclin D
Solyc12g042780.2	0	0	0	32	24	37	19.6069	0	39.2137922	8.113407877	1.34E-10	5.76E-10	up	--
Solyc07g021465.1	22	19	19	59	42	68	43.27362	15.4267	71.1205826	2.208713191	1.35E-10	5.76E-10	up	--
Solyc10g076810.1	103	124	86	6	12	10	46.47792	80.8202	12.1356563	-2.753853609	1.40E-10	5.97E-10	down	--
Solyc03g062900.3	252	174	184	22	13	44	94.41241	156.813	32.0121824	-2.268441496	1.41E-10	6.03E-10	down	--
Solyc04g051360.3	75	59	132	2	0	3	34.79022	67.6125	1.96795732	-5.014616681	1.48E-10	6.30E-10	down	--
Solyc06g068290.1	116	109	95	10	14	14	49.36545	82.4102	16.320686	-2.347013571	1.50E-10	6.39E-10	down	--
Solyc07g044800.2	7	9	6	48	27	44	27.95646	5.68328	50.2296381	3.15070161	1.51E-10	6.42E-10	up	Apoptotic ATPase
Solyc04g072490.4	122	115	173	16	19	23	64.71937	104.769	24.6693049	-2.090096199	1.52E-10	6.47E-10	down	Mitochondrial transcription termination factor, mTERF
Solyc02g086650.3	95	78	123	8	5	14	43.33545	75.6255	11.0453598	-2.744707618	1.57E-10	6.66E-10	down	Glucose-6-phosphate/phosphate
Solyc03g116730.4	75	99	118	10	9	7	43.06537	74.8055	11.3251965	-2.747451647	1.66E-10	7.03E-10	down	Fatty acid desaturase
Solyc02g087810.3	111	138	177	22	14	11	64.75154	109.027	20.4762019	-2.430495554	1.73E-10	7.32E-10	down	Predicted mutarotase
Solyc01g099270.2	1	4	6	39	21	61	26.17734	2.8006	49.5540842	4.14469209	1.76E-10	7.42E-10	up	Cyclin
Solyc05g055710.3	0	0	0	32	35	26	20.29196	0	40.5839254	8.15472912	1.79E-10	7.58E-10	up	Multicopper oxidases
Solyc05g023900.1	258	243	277	53	17	25	120.1148	199.626	40.6036547	-2.299592757	1.80E-10	7.62E-10	down	FOG: PPR repeat
Solyc01g057430.3	100	106	137	7	11	20	51.77208	87.8161	15.728016	-2.463225432	1.86E-10	7.86E-10	down	--
Solyc12g098880.2	10	6	5	37	56	37	31.23115	5.41518	57.0471269	3.392593692	1.92E-10	8.09E-10	up	--
Solyc07g150148.1	67	73	90	2	3	8	32.08973	58.9227	5.25676451	-3.43783034	1.92E-10	8.10E-10	down	--
novel.264	7	8	12	52	25	68	33.32084	6.89498	59.7467067	3.11831989	1.93E-10	8.12E-10	up	MADS box transcription factor
Solyc04g051590.3	0	0	0	24	39	31	20.43128	0	40.8625544	8.165413956	1.97E-10	8.29E-10	up	--
Solyc10g081740.3	6	1	1	28	33	42	22.8758	2.06486	43.6867319	4.408029914	2.00E-10	8.41E-10	up	Ca2+/calmodulin-dependent protein kinase
Solyc04g050720.4	125	91	121	12	15	16	52.38362	86.354	18.4132009	-2.238592608	2.05E-10	8.62E-10	down	--
Solyc11g066250.2	5	1	5	31	25	45	22.48587	2.79126	42.1804852	3.909998561	2.09E-10	8.77E-10	up	Serine carboxypeptidases (lysosomal cathepsin A)
Solyc06g009290.4	44	15	11	187	592	513	288.134	18.0849	558.183135	4.948475224	2.10E-10	8.82E-10	up	Multidrug/pheromone exporter, ABC superfamily
Solyc02g082310.2	62	71	88	2	4	6	30.8106	56.6043	5.01693026	-3.482232216	2.15E-10	9.00E-10	down	Serine/threonine protein kinase
novel.777	0	0	0	21	31	41	19.64135	0	39.2827047	8.114789347	2.18E-10	9.12E-10	up	--
Solyc02g087040.1	107	95	97	14	5	13	45.07745	76.8496	13.3053136	-2.511833126	2.20E-10	9.20E-10	down	FOG: Predicted E3 ubiquitin ligase
Solyc01g111280.3	470	388	229	45	37	76	173.351	281.235	65.467497	-2.095280959	2.24E-10	9.36E-10	down	Predicted RNA-binding protein containing PIN domain
Solyc02g072100.3	90	70	76	5	1	8	33.10951	60.628	5.59103859	-3.376695589	2.25E-10	9.38E-10	down	Uncharacterized conserved protein
Solyc05g012020.4	537	1196	1389	3835	7381	5328	4013.513	799.216	7227.80917	3.176833186	2.27E-10	9.49E-10	up	MADS box transcription factor

Solyc04g016160.4	184	116	139	12	15	28	67.74524	112.705	22.7859027	-2.291497874	2.29E-10	9.55E-10	down	--
Solyc10g080550.2	65	72	104	3	2	8	33.3962	61.5872	5.20520608	-3.507065969	2.30E-10	9.60E-10	down	--
Solyc06g009830.3	6	5	12	36	40	42	28.22332	5.83814	50.608505	3.102637613	2.30E-10	9.60E-10	up	Fumarase
Solyc08g081820.4	1	0	4	35	52	28	26.09047	1.24391	50.9370212	5.313963287	2.38E-10	9.92E-10	up	Predicted K+/H+-antiporter
Solyc06g009620.1	147	145	145	30	13	23	70.09109	112.323	27.8590819	-2.007086804	2.40E-10	1.00E-09	down	FOG: PPR repeat
Solyc12g097010.1	13	9	11	56	33	46	32.92925	8.46726	57.3912456	2.759928206	2.45E-10	1.02E-09	up	--
Solyc01g108800.4	136	199	249	27	14	37	90.7968	149.456	32.1373438	-2.201963012	2.56E-10	1.06E-09	down	--
Solyc12g005020.2	117	54	74	1	2	4	32.86913	62.8654	2.87285694	-4.416623857	2.80E-10	1.16E-09	down	FOG: Predicted E3 ubiquitin ligase
Solyc02g067490.3	20	57	42	111	117	123	90.63024	30.6828	150.577664	2.29486229	2.86E-10	1.19E-09	up	--
Solyc11g012370.1	0	1	1	42	36	65	30.08519	0.51232	59.6580649	6.864558796	2.90E-10	1.20E-09	up	--
Solyc07g063350.3	81	84	69	5	1	8	32.94528	60.2995	5.59103859	-3.368666737	3.00E-10	1.24E-09	down	--
Solyc03g113260.1	75	80	74	5	3	9	32.92383	58.9143	6.93332915	-3.046793293	3.02E-10	1.25E-09	down	--
Solyc10g055540.2	105	142	119	16	16	10	56.35954	94.2537	18.4653632	-2.377960042	3.21E-10	1.33E-09	down	Ras-related small GTPase, Rho type
Solyc09g014930.1	115	114	154	20	18	15	60.5137	98.0126	23.0147848	-2.108896159	3.28E-10	1.35E-09	down	FOG: PPR repeat
Solyc04g082530.3	111	117	107	11	17	17	52.75911	86.2001	19.3181005	-2.168187592	3.33E-10	1.37E-09	down	PHD finger protein BR140/LIN-49
novel.356	0	0	0	28	17	54	20.11812	0	40.2362436	8.157079543	3.53E-10	1.45E-09	up	--
Solyc04g018140.1	83	62	62	3	1	2	27.88516	53.2404	2.52990583	-4.387685835	3.79E-10	1.56E-09	down	--
Solyc02g077050.3	167	71	174	1	0	0	52.69599	104.955	0.43739094	-7.837402367	3.89E-10	1.60E-09	down	Cysteine proteinase Cathepsin L
Solyc04g075000.1	90	97	121	12	13	8	46.70694	78.8937	14.5201677	-2.473165189	3.95E-10	1.62E-09	down	Serine/threonine protein kinase
Solyc10g086240.2	24	19	30	75	42	99	54.03417	18.6533	89.4149839	2.264614226	4.11E-10	1.68E-09	up	UDP-glucuronosyl and UDP-glucosyl transferase
Solyc01g107520.3	0	0	0	41	21	27	19.01977	0	38.0395445	8.066655655	4.11E-10	1.68E-09	up	--
Solyc01g095500.3	108	75	34	385	191	222	199.4757	56.2716	342.679826	2.607414487	4.12E-10	1.68E-09	up	--
novel.1270	206	232	164	36	13	15	91.49096	155.414	27.568293	-2.500763242	4.17E-10	1.70E-09	down	--
Solyc11g010250.1	584	385	564	20	64	89	232.4564	392.441	72.4714501	-2.435276936	4.17E-10	1.70E-09	down	--
Solyc02g093890.1	7	7	16	36	48	52	32.88906	7.61411	58.1640181	2.922337781	4.37E-10	1.78E-09	up	--
Solyc07g005410.3	15	12	11	34	44	66	35.10889	9.78286	60.4349241	2.631606709	4.43E-10	1.80E-09	up	--
Solyc11g010670.1	1185	829	1011	70	124	255	480.1643	776.162	184.167001	-2.071712536	4.51E-10	1.83E-09	down	--
Solyc12g062910.2	0	0	0	22	34	29	18.40712	0	36.814242	8.015384004	4.65E-10	1.89E-09	up	--
Solyc01g104800.2	0	0	0	25	20	41	17.82691	0	35.6538254	7.980095047	4.67E-10	1.90E-09	up	Uncharacterized membrane protein
Solyc10g076180.1	5	2	3	24	25	58	23.21027	2.56471	43.8558421	4.102370814	4.72E-10	1.92E-09	up	--
Solyc10g055800.2	455	612	1623	4831	6909	8649	4661.541	680.27	8642.81166	3.667219478	4.77E-10	1.94E-09	up	Predicted chitinase
Solyc01g109950.4	0	0	0	31	28	24	17.99755	0	35.9951052	7.982791389	4.86E-10	1.97E-09	up	Serine/threonine protein kinase
Solyc12g088110.3	0	0	0	25	42	23	19.92583	0	39.851659	8.126968511	5.15E-10	2.08E-09	up	Sexual differentiation process protein ISP4
Solyc06g051680.1	65	81	69	5	5	4	30.7253	55.3613	6.08926884	-3.210475443	5.17E-10	2.09E-09	down	--
novel.860	17	40	22	85	58	143	69.05182	20.4583	117.645323	2.531592808	5.44E-10	2.19E-09	up	--
Solyc10g051330.3	0	0	0	21	23	41	17.68555	0	35.3711097	7.967351957	5.49E-10	2.21E-09	up	Serine/threonine protein kinase
Solyc08g078700.2	875	491	445	1502	1950	1070	1233.471	466.63	2000.31172	2.099504625	5.58E-10	2.25E-09	up	Molecular chaperone (small heat-shock protein Hsp26/Hsp42)
Solyc11g072670.1	25	27	33	77	64	63	54.85387	21.7792	87.9285468	2.007178749	5.60E-10	2.25E-09	up	--
Solyc03g080030.4	2	0	0	43	52	41	29.84538	0.51751	59.1732423	6.841687116	5.64E-10	2.27E-09	up	--
Solyc06g074900.3	109	172	180	22	18	28	73.45991	118.293	28.6266602	-2.042352957	6.23E-10	2.49E-09	down	--
Solyc12g027880.3	0	1	1	37	53	44	29.32167	0.51232	58.1310215	6.817556443	6.23E-10	2.49E-09	up	Amino acid transporters
Solyc02g080050.1	111	45	101	3	4	4	35.14697	65.5684	4.72553758	-3.808292104	6.27E-10	2.51E-09	down	--
Solyc06g051090.3	1	0	3	43	21	37	21.77793	0.99762	42.5582445	5.381810271	6.29E-10	2.51E-09	up	FOG: Armadillo/beta-catenin-like repeats
Solyc09g091770.1	0	0	0	40	24	22	18.62352	0	37.2470426	8.033607143	6.33E-10	2.53E-09	up	--
Solyc07g006700.1	2	6	17	78	86	42	48.88559	6.30058	91.4705959	3.846111789	6.55E-10	2.61E-09	up	Defense-related protein containing SCP domain
Solyc02g065116.1	120	159	123	14	15	28	63.65169	103.643	23.6606846	-2.115731558	6.59E-10	2.63E-09	down	--
Solyc10g086250.2	12	20	16	82	42	54	44.22267	12.3662	76.079089	2.621843487	6.78E-10	2.70E-09	up	Transcription factor, Myb superfamily
Solyc04g082030.1	81	51	125	199	350	206	199.2753	65.3128	333.237792	2.347273975	6.78E-10	2.70E-09	up	Ornithine decarboxylase

Solyc03g034090.3	163	127	131	25	19	15	66.95868	108.227	25.6906889	-2.092136748	6.82E-10	2.72E-09	down	--
Solyc12g087930.1	0	0	0	25	25	28	16.68074	0	33.3614787	7.876100981	6.93E-10	2.76E-09	up	--
novel.318	307	308	139	29	20	41	116.5062	195.609	37.4033891	-2.378079958	6.94E-10	2.76E-09	down	--
Solyc06g064610.1	9	6	2	37	26	83	31.77911	4.41755	59.1406691	3.758769538	7.05E-10	2.80E-09	up	--
Solyc12g007320.1	0	3	2	36	20	38	20.3313	1.29066	39.3719503	4.94066241	7.20E-10	2.86E-09	up	FOG: Predicted E3 ubiquitin ligase
Solyc08g007940.4	0	0	0	35	31	19	18.69478	0	37.3895581	8.036095333	7.56E-10	3.00E-09	up	Regulatory protein MLP and related LIM proteins
Solyc12g049320.3	4	7	2	32	27	37	22.03522	3.3898	40.6806403	3.599998519	7.65E-10	3.03E-09	up	--
Solyc07g055630.4	101	46	118	1	3	6	35.76218	67.4338	4.09058994	-4.010616875	7.71E-10	3.05E-09	down	--
Solyc03g078030.1	101	95	117	17	5	12	47.23796	80.2228	14.2530947	-2.4825173	7.71E-10	3.05E-09	down	--
Solyc05g041700.1	314	221	366	19	51	24	136.0878	230.183	41.9922495	-2.47282637	7.85E-10	3.11E-09	down	--
Solyc07g045530.2	143	79	120	0	1	0	44.031	87.5731	0.48894937	-7.57592183	8.19E-10	3.23E-09	down	--
Solyc02g091420.3	0	0	0	31	30	20	17.75772	0	35.5154367	7.961774637	8.99E-10	3.54E-09	up	Aquaporin (major intrinsic protein family)
Solyc09g075270.1	15	16	13	39	50	52	35.89682	11.3395	60.4540897	2.413071393	9.22E-10	3.62E-09	up	--
novel.1447	87	102	74	10	4	10	38.92282	67.872	9.97362506	-2.746998601	9.47E-10	3.72E-09	down	--
Solyc02g078890.1	120	90	57	5	2	8	37.55587	69.0318	6.07998797	-3.46358222	9.53E-10	3.74E-09	down	--
Solyc02g072010.3	64	83	60	4	3	5	29.2282	53.418	5.03837096	-3.394849451	9.66E-10	3.79E-09	down	FOG: PPR repeat
novel.1603	9	1	3	33	28	60	26.66085	3.33371	49.9879923	3.909959701	9.81E-10	3.85E-09	up	--
Solyc12g014020.2	128	95	144	19	12	22	58.02675	93.8591	22.1944403	-2.071330251	9.88E-10	3.87E-09	down	Predicted nucleic acid binding protein
Solyc04g057830.1	63	64	69	0	3	2	26.25849	50.3214	2.19563174	-4.56313387	1.00E-09	3.93E-09	down	--
novel.1002	101	87	127	10	11	19	48.61664	80.5575	16.675797	-2.259640676	1.01E-09	3.97E-09	down	--
Solyc02g081900.4	61	65	75	6	2	3	28.12151	51.5476	4.69541985	-3.462960052	1.02E-09	3.99E-09	down	FOG: PPR repeat
Solyc02g067970.4	6	4	9	38	27	37	24.06912	4.83325	43.304986	3.154993838	1.02E-09	4.00E-09	up	Predicted E3 ubiquitin ligase
Solyc02g005602.1	195	175	142	23	26	12	79.56538	131.985	27.1453772	-2.307289055	1.03E-09	4.03E-09	down	K+-channel ERG and related proteins
Solyc10g005060.4	16	17	64	116	162	149	104.3333	24.425	184.241528	2.908961404	1.04E-09	4.04E-09	up	Hydroxyindole-O-methyltransferase
Solyc02g067810.1	41	12	12	78	91	117	69.00078	16.7568	121.244729	2.856403296	1.04E-09	4.05E-09	up	--
Solyc05g014480.4	137	129	118	19	9	27	60.68942	98.8293	22.5495512	-2.106623692	1.06E-09	4.14E-09	down	Glyceraldehyde 3-phosphate dehydrogenase
novel.1873	62	64	73	5	0	3	27.16394	51.0478	3.28013016	-3.920515104	1.08E-09	4.20E-09	down	--
Solyc12g038800.1	303	260	264	20	12	62	124.8991	212.591	37.2075037	-2.489767959	1.10E-09	4.26E-09	down	--
novel.1016	75	59	70	1	4	6	28.46107	52.3426	4.57953931	-3.496494129	1.13E-09	4.40E-09	down	--
Solyc05g005030.4	0	0	0	33	34	17	18.62642	0	37.2528407	8.029932834	1.15E-09	4.45E-09	up	--
Solyc02g068680.1	272	122	336	20	28	35	110.3912	185.59	35.1921148	-2.399936177	1.17E-09	4.52E-09	down	--
Solyc03g007710.3	61	61	83	5	0	2	27.68477	52.4538	2.91573835	-4.147497054	1.18E-09	4.56E-09	down	--
Solyc02g088980.3	106	69	137	6	12	7	45.28408	79.5257	11.0424808	-2.881773775	1.23E-09	4.75E-09	down	--
Solyc06g084280.1	10	25	11	83	41	73	47.4492	11.9474	82.950975	2.803082016	1.24E-09	4.79E-09	up	--
Solyc02g085530.3	22	10	14	72	41	55	41.6908	11.801	71.580622	2.599345258	1.26E-09	4.88E-09	up	FOG: PPR repeat
Solyc05g053620.3	250	214	163	269	706	667	433.835	161.764	705.90576	2.125431845	1.28E-09	4.94E-09	up	--
Solyc05g050590.3	0	0	0	27	34	19	17.67864	0	35.3572786	7.954148367	1.28E-09	4.96E-09	up	Phosphatidylinositol 4-kinase
novel.1398	191	180	164	1	8	25	75.57881	137.699	13.4587812	-3.320885081	1.31E-09	5.04E-09	down	--
novel.391	131	115	134	9	14	28	59.23889	97.493	20.9847805	-2.195592246	1.31E-09	5.06E-09	down	--
Solyc07g007250.4	14	11	2	47	61	52	38.18657	7.04148	69.3316604	3.305438721	1.35E-09	5.19E-09	up	--
Solyc03g046580.1	4	0	0	31	24	42	20.81669	1.03503	40.5983603	5.305647748	1.36E-09	5.23E-09	up	--
Solyc02g093330.4	214	145	265	17	30	42	98.31152	159.214	37.4085834	-2.086793979	1.36E-09	5.26E-09	down	Nuclear pore complex, Nup98 component
Solyc10g076200.3	0	0	0	27	27	21	16.33171	0	32.6634166	7.841674715	1.40E-09	5.38E-09	up	--
Solyc01g006750.2	0	0	1	29	52	47	27.7412	0.24629	55.23612	7.640240444	1.45E-09	5.55E-09	up	--
Solyc12g096480.1	76	101	94	6	5	15	40.1102	69.6854	10.5349697	-2.686412338	1.47E-09	5.64E-09	down	--
Solyc12g098120.3	0	0	0	19	29	27	16.16427	0	32.3285387	7.828828069	1.49E-09	5.72E-09	up	Aldehyde reductase
Solyc08g077170.3	2	3	6	39	31	25	22.0594	2.79333	41.3254727	3.870461648	1.54E-09	5.89E-09	up	H+/oligopeptide symporter
novel.653	325	342	148	19	31	43	125.3324	211.528	39.1367064	-2.431712502	1.57E-09	5.99E-09	down	--
Solyc12g005380.3	23	11	17	36	76	155	61.22579	13.0646	109.386957	3.067910533	1.59E-09	6.08E-09	up	Diacylglycerol kinase

novel.366	121	126	101	18	11	22	55.48615	89.7042	21.2681	-2.062487424	1.60E-09	6.11E-09	down	--
Solyc06g072017.1	70	110	105	11	7	12	42.92148	73.2363	12.6066477	-2.530371935	1.61E-09	6.14E-09	down	AAA+-type ATPase
Solyc08g005337.1	56	76	122	0	1	2	32.98671	64.7557	1.217733	-5.678773368	1.62E-09	6.16E-09	down	--
Solyc11g007600.2	0	1	0	46	46	32	27.26911	0.26603	54.2721926	7.612383763	1.66E-09	6.31E-09	up	--
Solyc01g090180.4	13	24	22	67	51	55	44.725	15.1668	74.283161	2.289607205	1.66E-09	6.34E-09	up	--
novel.772	161	54	65	0	3	3	37.29707	72.0341	2.56002356	-4.822754041	1.74E-09	6.61E-09	down	FOG: Predicted E3 ubiquitin ligase
Solyc10g080920.2	20	21	20	58	36	85	44.81582	15.6875	73.9441562	2.245482548	1.74E-09	6.63E-09	up	Zuotin and related molecular chaperones (DnaJ superfamily)
Solyc02g072020.3	88	151	126	14	16	13	56.32843	93.9731	18.6837568	-2.347317755	1.75E-09	6.65E-09	down	FOG: PPR repeat
Solyc07g064450.4	15	11	8	39	36	49	30.64679	8.77797	52.5156231	2.585278353	1.76E-09	6.69E-09	up	Cytochrome P450 CYP4/CYP19/CYP26 subfamilies
Solyc02g086227.1	70	85	124	2	0	10	37.8919	71.2651	4.51870001	-3.872948162	1.76E-09	6.70E-09	down	--
Solyc01g009770.3	35	45	29	86	106	79	73.20065	28.1701	118.231208	2.066371929	1.76E-09	6.70E-09	up	--
Solyc03g123550.1	92	98	51	0	2	6	32.80065	62.437	3.16424962	-4.232030272	1.77E-09	6.71E-09	down	--
Solyc01g093960.3	15	14	40	68	101	85	63.77853	17.4573	110.099775	2.647853422	1.81E-09	6.88E-09	up	MADS box transcription factor
Solyc10g081670.2	0	0	0	28	22	22	15.51023	0	31.0204525	7.769711762	1.85E-09	6.99E-09	up	--
Solyc09g065450.4	0	0	0	26	25	21	15.62406	0	31.2481269	7.778393793	1.90E-09	7.19E-09	up	--
Solyc04g072070.3	17	7	2	30	130	113	62.30753	6.75364	117.861422	4.128788077	1.92E-09	7.25E-09	up	--
Solyc10g008230.3	338	249	301	41	45	16	136.7998	227.834	45.7660195	-2.333785304	1.94E-09	7.33E-09	down	Apoptotic ATPase
novel.99	77	41	61	0	0	0	22.92751	45.855	0	-7.604306197	1.97E-09	7.41E-09	down	--
Solyc12g089010.3	98	89	101	12	12	17	45.61026	73.9098	17.3107446	-2.090738904	2.01E-09	7.55E-09	down	mRNA splicing factor ATP-dependent RNA helicase
Solyc10g084000.3	186	174	286	6	29	10	92.65195	164.856	20.4477956	-3.041357369	2.04E-09	7.69E-09	down	Copper chaperone
Solyc01g094990.2	11	10	5	34	35	44	27.3779	6.73804	48.0177599	2.839706901	2.07E-09	7.80E-09	up	Galactosyltransferases
Solyc02g081510.3	2	2	1	25	31	26	18.43112	1.29586	35.5663913	4.782730711	2.08E-09	7.83E-09	up	--
Solyc12g100240.1	4	6	3	15	59	67	31.59659	3.37006	59.8231286	4.156257141	2.12E-09	7.96E-09	up	--
Solyc02g076770.1	0	0	0	23	45	17	19.12869	0	38.2573743	8.067377177	2.13E-09	8.01E-09	up	--
Solyc09g066410.3	3	1	2	25	71	23	27.78303	1.53488	54.0311908	5.125993049	2.17E-09	8.16E-09	up	Inorganic phosphate transporter
novel.134	74	48	67	3	1	2	25.47429	48.4187	2.52990583	-4.251530505	2.22E-09	8.34E-09	down	--
Solyc08g082310.3	173	152	170	7	22	4	71.17319	127.07	15.2761901	-3.102234225	2.25E-09	8.44E-09	down	--
Solyc10g054010.1	47	26	26	72	66	106	63.93507	25.4818	102.388339	2.010056019	2.29E-09	8.56E-09	up	--
Solyc11g013270.2	27	13	23	64	46	65	45.1398	16.1094	74.1701594	2.200692673	2.32E-09	8.70E-09	up	--
Solyc11g006310.2	7	2	4	19	40	56	25.80143	3.32851	48.2743444	3.85723506	2.35E-09	8.79E-09	up	--
novel.1709	32	25	35	53	103	95	65.85587	23.551	108.160728	2.194452791	2.37E-09	8.87E-09	up	--
Solyc02g063390.4	109	89	89	7	14	7	43.1292	73.8006	12.4577705	-2.605963133	2.39E-09	8.92E-09	down	Predicted steroid reductase
Solyc02g078250.4	0	0	0	21	18	32	14.82342	0	29.6468365	7.712359414	2.40E-09	8.95E-09	up	--
Solyc09g010050.1	58	63	76	3	2	7	27.66319	50.4856	4.84081426	-3.330111023	2.43E-09	9.06E-09	down	Cullins
Solyc05g054050.4	0	1	0	40	45	32	25.71246	0.26603	51.1588976	7.526895267	2.52E-09	9.38E-09	up	Glutamate decarboxylase/sphingosine phosphate lyase
Solyc04g008900.4	13	17	25	43	49	69	40.97644	14.0435	67.9093649	2.271496423	2.64E-09	9.84E-09	up	--
Solyc07g042390.3	124	95	133	19	6	6	51.77267	90.1149	13.4304751	-2.759360655	2.66E-09	9.90E-09	down	--
Solyc01g097000.3	126	86	112	17	8	17	50.30398	83.0661	17.5419018	-2.231377622	2.66E-09	9.91E-09	down	--
Solyc08g007350.2	72	72	74	6	5	10	32.36142	56.0098	8.71301065	-2.663062235	2.85E-09	1.06E-08	down	Predicted 3'-5' exonuclease
novel.1701	63	21	15	151	144	124	103.611	25.5826	181.639327	2.827919603	3.02E-09	1.12E-08	up	--
Solyc02g069680.3	109	90	104	12	16	15	48.14938	77.761	18.5377585	-2.083014006	3.05E-09	1.13E-08	down	Vacuolar assembly/sorting protein DID4
Solyc01g008220.3	117	91	133	10	11	24	52.8686	87.2394	18.497756	-2.215688417	3.16E-09	1.16E-08	down	WW domain binding protein WBP-2
Solyc08g076800.4	4	5	13	57	25	49	30.28857	5.56692	55.010217	3.296827246	3.16E-09	1.16E-08	up	FOG: Predicted E3 ubiquitin ligase
Solyc12g049616.1	96	126	128	17	10	24	55.4778	89.8851	21.0705433	-2.073090146	3.21E-09	1.19E-08	down	--
Solyc08g015820.1	115	63	116	0	0	1	37.72533	75.0863	0.36439181	-7.354308312	3.23E-09	1.19E-08	down	--
Solyc06g082400.1	0	0	0	21	23	23	14.40603	0	28.8120571	7.663126374	3.24E-09	1.19E-08	up	--

Solyc01g111800.4	80	57	71	6	4	1	29.14757	53.3506	4.94453497	-3.489758365	3.28E-09	1.21E-08	down	Amino acid transporters
Solyc08g066310.3	81	70	107	4	10	10	38.10855	65.9341	10.2829756	-2.691872566	3.33E-09	1.23E-08	down	--
novel.1886	2	1	3	48	14	37	21.42248	1.52241	41.3225536	4.753844994	3.43E-09	1.26E-08	up	--
Solyc05g010060.4	0	2	0	36	31	43	23.5522	0.53205	46.5723525	6.499765097	3.45E-09	1.27E-08	up	Predicted small molecule transporter
Solyc11g073170.1	1	0	2	47	24	27	21.44104	0.75133	42.1307383	5.776188769	3.52E-09	1.29E-08	up	FOG: Predicted E3 ubiquitin ligase
Solyc11g070040.3	98	97	110	18	5	11	46.29034	78.2546	14.3260938	-2.44325306	3.54E-09	1.30E-08	down	FOG: PPR repeat
Solyc02g068630.1	101	109	98	12	17	12	48.60063	79.2677	17.9335324	-2.168864452	3.56E-09	1.31E-08	down	FOG: PPR repeat
novel.208	117	91	94	9	13	18	47.24305	77.6342	16.851913	-2.199074728	3.73E-09	1.37E-08	down	--
Solyc01g060460.2	36	63	70	0	0	0	21.65759	43.3152	0	-7.521977402	3.74E-09	1.37E-08	down	DNA repair protein, SNF2 family
Solyc10g049580.2	10	8	11	30	32	50	27.20633	7.42496	46.9876989	2.663001898	3.75E-09	1.37E-08	up	--
Solyc06g064530.1	63	56	58	1	3	4	24.42289	45.484	3.36180631	-3.748584509	3.87E-09	1.42E-08	down	--
Solyc07g052730.3	88	45	69	3	3	4	27.98618	51.7358	4.2365882	-3.609969133	4.00E-09	1.46E-08	down	Rho GTPase effector BNI1 and related formins
Solyc02g067800.4	18	13	6	57	31	100	43.0608	9.59371	76.5278956	3.007154484	4.01E-09	1.47E-08	up	--
Solyc01g109420.3	198	193	253	25	39	15	100.1789	164.888	35.4696764	-2.24153549	4.14E-09	1.51E-08	down	1,4-benzoquinone reductase-like
Solyc08g007225.1	117	71	77	6	10	5	38.73127	68.1267	9.33579846	-2.906845816	4.18E-09	1.53E-08	down	Predicted lipase
Solyc04g056570.3	1	4	0	31	20	35	18.70734	1.32287	36.0918202	4.80632918	4.27E-09	1.56E-08	up	--
Solyc01g065510.3	49	62	77	1	2	5	25.68713	48.137	3.23724875	-3.843538909	4.32E-09	1.57E-08	down	--
Solyc06g076130.3	100	77	132	9	10	18	47.12748	78.8699	15.3850649	-2.344340341	4.43E-09	1.61E-08	down	--
Solyc04g080120.1	107	102	130	22	10	18	53.95523	86.8393	21.0711471	-2.038029956	4.47E-09	1.63E-08	down	FOG: PPR repeat
novel.2059	84	87	94	4	7	16	39.51679	68.0311	11.0024784	-2.595751219	4.49E-09	1.63E-08	down	--
novel.1456	0	1	2	32	40	23	21.34705	0.7586	41.9354968	5.76178336	4.50E-09	1.64E-08	up	--
Solyc07g055100.4	59	61	59	4	0	2	24.25185	46.0254	2.4783474	-4.182153694	4.58E-09	1.67E-08	down	Transcription factor GT-2 and related proteins
Solyc06g005390.1	126	168	128	25	21	16	67.92695	108.821	27.0329794	-2.025819602	4.66E-09	1.70E-08	down	Histone H2B
Solyc06g048430.4	64	55	58	1	4	2	24.29933	45.4767	3.12197206	-3.923784924	4.72E-09	1.72E-08	down	Mitochondrial F1F0-ATP synthase
Solyc05g026595.1	135	87	117	12	10	23	52.70577	86.8923	18.5191967	-2.208782144	4.80E-09	1.74E-08	down	4-hydroxyphenylpyruvate dioxygenase
Solyc05g021165.1	134	64	91	1	0	1	37.45662	74.1115	0.80178276	-6.455144855	4.91E-09	1.78E-08	down	--
Solyc09g089750.1	25	39	21	71	63	213	80.74504	22.0161	139.474024	2.671045587	4.94E-09	1.79E-08	up	Iron/ascorbate family oxidoreductases
Solyc03g116910.3	42	39	44	161	232	70	120.7216	32.0796	209.363624	2.702426663	4.99E-09	1.81E-08	up	Flavonol reductase/cinnamoyl-CoA reductase
Solyc07g056230.3	1	0	0	23	47	44	24.6663	0.25876	49.073852	7.470148535	5.00E-09	1.81E-08	up	Predicted membrane protein
Solyc09g018610.1	46	86	78	1	3	0	27.94796	53.9917	1.90423906	-4.963797662	5.02E-09	1.82E-08	down	--
Solyc10g050770.1	66	63	53	4	3	1	25.23589	46.891	3.58080371	-3.769732538	5.11E-09	1.85E-08	down	--
Solyc07g064600.3	595	322	203	32	24	68	170.0639	289.618	50.5099384	-2.50943331	5.35E-09	1.93E-08	down	Putative translation initiation inhibitor UK114/IBM1
Solyc02g067710.3	69	72	57	6	4	5	28.72438	51.0467	6.40210222	-3.000581572	5.40E-09	1.95E-08	down	Uncharacterized conserved protein
novel.325	17	9	13	46	29	73	35.44749	9.99487	60.9001176	2.613640121	5.43E-09	1.96E-08	up	--
novel.1212	128	49	84	2	1	8	35.56168	66.8445	4.27886576	-3.888684413	5.45E-09	1.97E-08	down	--
Solyc07g007705.1	109	108	82	10	8	19	46.17004	77.1311	15.2089489	-2.317273275	5.49E-09	1.98E-08	down	--
Solyc03g063680.3	119	137	199	10	9	32	68.34213	116.249	20.4349918	-2.477398444	5.52E-09	1.99E-08	down	CCCH-type Zn-finger protein
Solyc03g117310.1	103	212	176	23	21	17	76.45958	126.397	26.5225894	-2.265425503	5.56E-09	2.01E-08	down	--
Solyc01g101150.3	119	89	93	14	14	11	47.17522	77.3734	16.9770744	-2.208805573	5.64E-09	2.03E-08	down	FOG: PPR repeat
Solyc12g099980.3	15	11	12	48	26	72	34.85339	9.76312	59.9436595	2.627000159	5.73E-09	2.06E-08	up	FOG: Leucine rich repeat
Solyc05g009310.3	75	47	71	4	0	1	25.75525	49.3966	2.11395559	-4.541494545	5.86E-09	2.11E-08	down	GATA-4/5/6 transcription factors
Solyc05g052590.1	55	52	74	3	1	5	24.95675	46.2904	3.62308127	-3.618504794	6.28E-09	2.25E-08	down	Serine/threonine protein kinase
Solyc04g063210.3	136	94	124	8	14	24	54.91357	90.7373	19.0898223	-2.235179592	6.33E-09	2.27E-08	down	O-methyltransferase
Solyc04g071900.4	116	46	162	294	255	271	217.0885	82.1518	352.025209	2.096886461	6.41E-09	2.30E-08	up	--
Solyc06g050370.1	80	74	79	7	1	11	33.70119	59.8434	7.55899592	-2.926333123	6.52E-09	2.33E-08	down	--
novel.129	7	11	3	53	53	28	32.38774	5.47647	59.2990076	3.440211543	6.61E-09	2.36E-08	up	--
Solyc01g058350.1	75	55	75	1	6	6	29.03368	52.5099	5.55743806	-3.250290951	6.62E-09	2.37E-08	down	--
Solyc02g089590.3	0	0	0	24	29	15	15.0714	0	30.1427917	7.723435269	6.76E-09	2.42E-08	up	--
novel.1486	76	89	58	5	6	8	32.83125	57.6267	8.03578546	-2.839116939	6.83E-09	2.44E-08	down	--

Solyc12g006200.1	90	64	83	4	2	12	33.928	60.7558	7.10016427	-3.03279274	6.91E-09	2.46E-08	down	--
Solyc10g083550.1	73	29	67	0	0	0	21.5527	43.1054	0	-7.515526484	6.93E-09	2.47E-08	down	Glutamine synthetase
Solyc04g010330.3	58	53	69	5	2	4	25.36185	46.1013	4.62242072	-3.308252021	6.96E-09	2.48E-08	down	--
Solyc05g042170.1	0	0	0	23	24	17	13.99472	0	27.9894375	7.618002997	7.10E-09	2.53E-08	up	--
Solyc02g090500.3	58	59	63	3	5	4	25.7171	46.2197	5.21448695	-3.174689882	7.31E-09	2.60E-08	down	Predicted haloacid-halidohydrolase
Solyc09g091700.4	8	10	10	33	26	51	26.46189	7.19322	45.7305673	2.675651977	7.44E-09	2.65E-08	up	Predicted NAD-dependent oxidoreductase
Solyc01g008540.4	383	776	623	34	6	9	240.0317	458.979	21.0845147	-4.445687202	7.55E-09	2.68E-08	down	Flavonol reductase/cinnamoyl-CoA reductase
Solyc10g005480.3	401	253	286	8	31	55	140.1016	241.505	38.6981078	-2.634346039	7.62E-09	2.71E-08	down	--
Solyc05g009280.4	2	5	2	45	65	15	29.6352	2.34023	56.9301789	4.607020227	7.63E-09	2.71E-08	up	--
Solyc11g011240.1	7	6	7	33	31	29	22.64509	5.13148	40.1586943	2.961397448	7.70E-09	2.73E-08	up	Geranylgeranyl pyrophosphate synthase
novel.879	1	3	11	35	27	72	29.25627	3.76601	54.7465266	3.851054704	8.14E-09	2.88E-08	up	--
Solyc03g098790.3	0	0	0	23	25	16	14.057	0	28.113995	7.623759242	8.21E-09	2.91E-08	up	--
Solyc07g063465.1	108	102	61	0	0	2	35.41647	70.1042	0.72878362	-6.38420283	8.31E-09	2.94E-08	down	--
Solyc04g077810.1	16	16	32	39	96	113	60.72572	16.2778	105.173661	2.687702028	8.47E-09	2.99E-08	up	--
Solyc09g057900.3	83	53	65	1	2	0	26.50017	51.585	1.41528969	-5.325031034	8.58E-09	3.03E-08	down	--
Solyc06g062520.1	50	57	69	1	1	2	23.37522	45.0953	1.65512394	-4.737688516	8.59E-09	3.03E-08	down	--
Solyc02g038690.1	86	90	49	2	2	8	31.51576	58.2637	4.76781513	-3.549949092	8.63E-09	3.04E-08	down	Histone H2B
Solyc10g050970.1	100	87	72	0	8	1	35.51442	66.7529	4.2759868	-4.065986733	9.06E-09	3.19E-08	down	--
novel.1945	5	4	8	18	50	49	27.25195	4.3282	50.1757045	3.526246228	9.21E-09	3.24E-08	up	--
novel.147	195	173	100	9	21	22	71.66515	121.109	22.2210752	-2.450822437	9.26E-09	3.26E-08	down	--
Solyc03g082540.4	466	313	445	40	81	33	191.2856	313.446	69.1254668	-2.194412923	9.46E-09	3.32E-08	down	--
novel.1020	2	3	2	10	79	46	30.78555	1.80817	59.7629333	5.047437373	9.47E-09	3.32E-08	up	FOG: Transposon-encoded proteins with TYA
Solyc09g065780.3	74	71	64	2	3	10	29.89199	53.7984	5.98554813	-3.110119232	9.89E-09	3.47E-08	down	--
Solyc08g081040.3	55	56	60	3	2	0	23.09828	43.9065	2.29007158	-4.353103696	9.96E-09	3.49E-08	down	--
Solyc06g074550.3	92	79	120	11	11	18	45.56262	74.3764	16.7487961	-2.141944934	1.06E-08	3.70E-08	down	--
Solyc07g051870.1	6	23	28	92	59	72	54.94572	14.5673	95.3241904	2.707370282	1.09E-08	3.81E-08	up	MEKK and related serine/threonine protein kinases
Solyc01g105320.4	14	19	21	64	32	64	40.40483	13.8492	66.9604764	2.27692671	1.11E-08	3.86E-08	up	Metacaspase involved in regulation of
Solyc12g098220.2	94	120	115	14	10	25	52.3462	84.5696	20.1227623	-2.047202822	1.15E-08	4.00E-08	down	--
Solyc01g108220.3	73	67	69	9	5	7	31.31951	53.707	8.93200805	-2.590249987	1.15E-08	4.01E-08	down	--
Solyc10g007420.4	93	98	124	17	16	12	50.1532	80.6749	19.6315378	-2.061081371	1.15E-08	4.01E-08	down	FOG: TPR repeat
Solyc07g056530.3	115	105	111	13	20	10	52.06848	85.028	19.1089879	-2.187444494	1.17E-08	4.07E-08	down	Glucose-6-phosphate/phosphate
Solyc02g078870.1	79	91	40	0	1	3	28.04198	54.5018	1.58212481	-5.024536946	1.20E-08	4.18E-08	down	--
Solyc09g005890.4	8	11	12	36	26	52	27.67948	7.95182	47.4071319	2.581640775	1.21E-08	4.20E-08	up	--
Solyc02g067400.3	0	0	0	20	11	38	13.98658	0	27.9731509	7.633707519	1.22E-08	4.22E-08	up	Glutaredoxin-related protein
novel.1774	99	50	90	4	6	9	34.52354	61.0843	7.96278633	-2.930910505	1.22E-08	4.23E-08	down	FOG: Transposon-encoded proteins with TYA
novel.373	59	58	53	3	1	5	23.68631	43.7495	3.62308127	-3.535481032	1.23E-08	4.28E-08	down	--
Solyc09g061790.1	113	92	104	20	9	16	49.15335	79.3281	18.9786322	-2.058451646	1.26E-08	4.38E-08	down	--
Solyc03g120600.4	0	1	0	40	21	37	20.75605	0.26603	41.2460717	7.225076499	1.27E-08	4.39E-08	up	--
novel.1806	52	63	54	3	0	2	22.77781	43.5147	2.04095646	-4.366359499	1.27E-08	4.41E-08	down	--
novel.410	2	0	1	19	31	34	18.31049	0.7638	35.8571801	5.540210259	1.28E-08	4.44E-08	up	--
Solyc06g054610.2	77	60	88	8	8	8	33.94259	57.5593	10.325857	-2.493067802	1.34E-08	4.65E-08	down	--
Solyc04g071590.3	5	6	3	18	146	35	47.82109	3.62882	92.0133589	4.664661899	1.34E-08	4.65E-08	up	--
Solyc02g031960.3	148	280	115	20	20	24	84.18955	141.107	27.2722098	-2.372773649	1.37E-08	4.72E-08	down	--
novel.1108	90	91	43	4	5	2	31.50506	58.087	4.92309427	-3.608534665	1.38E-08	4.75E-08	down	--
Solyc03g007940.4	47	57	58	3	2	2	22.31437	41.6099	3.0188552	-3.804082075	1.45E-08	4.99E-08	down	--
Solyc07g053930.1	100	123	108	12	12	26	52.89325	85.1962	20.5902709	-2.027172717	1.45E-08	5.00E-08	down	--
Solyc10g008910.1	1145	1115	625	124	71	256	464.5319	746.828	182.236187	-2.029775876	1.47E-08	5.04E-08	down	Histones H3 and H4
Solyc04g025550.1	57	42	71	3	1	2	22.96935	43.4088	2.52990583	-4.094635021	1.50E-08	5.15E-08	down	--
Solyc05g015160.3	0	0	0	23	24	14	13.44813	0	26.896262	7.559502241	1.53E-08	5.26E-08	up	--

Solyc06g062560.2	1	1	0	100	451	42	140.0423	0.52478	279.559718	9.074286128	1.53E-08	5.27E-08	up	Predicted haloacid dehalogenase-like hydrolase
Solyc06g074770.4	56	61	54	0	4	3	23.53331	44.0176	3.04897293	-3.88320251	1.56E-08	5.35E-08	down	Ubiquitin fusion degradation protein-2
Solyc10g006720.4	59	77	143	147	197	385	185.9402	70.97	300.910343	2.084212862	1.59E-08	5.46E-08	up	--
Solyc03g112880.1	71	66	74	3	9	7	31.20919	54.1549	8.26345988	-2.737268803	1.64E-08	5.60E-08	down	--
novel.214	136	97	200	13	23	13	65.96117	110.253	21.6690114	-2.371622313	1.68E-08	5.73E-08	down	--
Solyc03g117880.1	69	28	52	0	0	0	19.055	38.11	0	-7.337551326	1.72E-08	5.88E-08	down	--
Solyc09g011750.3	3	3	3	24	16	39	17.42254	2.31322	32.5318533	3.823736615	1.76E-08	6.00E-08	up	--
Solyc10g084680.1	50	79	71	5	5	1	28.2183	51.4405	4.9960934	-3.42946743	1.78E-08	6.08E-08	down	--
Solyc08g066960.3	1	0	0	30	37	26	20.4729	0.25876	40.6870423	7.195898035	1.79E-08	6.12E-08	up	Na+-independent Cl/HCO3 exchanger AE1
Solyc03g116850.3	3	2	2	31	20	22	16.57781	1.8009	31.3547266	4.12053308	1.84E-08	6.26E-08	up	K+-channel ERG and related proteins
novel.1815	6	17	9	34	36	67	32.58966	8.2916	56.887721	2.788652728	1.87E-08	6.38E-08	up	FOG: Reverse transcriptase
Solyc12g006220.3	112	131	123	14	6	28	56.692	94.1239	19.2601402	-2.252876561	1.91E-08	6.49E-08	down	--
Solyc03g031955.1	63	42	68	2	0	4	23.27741	44.2225	2.33234914	-4.144922344	1.92E-08	6.53E-08	down	--
Solyc07g053380.2	102	79	100	16	9	16	44.63365	72.0382	17.2290685	-2.056418441	1.92E-08	6.54E-08	down	FOG: PPR repeat
Solyc05g007600.1	69	68	119	11	6	8	37.95628	65.2524	10.6601311	-2.618818621	1.95E-08	6.64E-08	down	Cysteine desulfurase NFS1
Solyc08g015640.1	70	57	77	1	0	8	27.79665	52.2408	3.35252544	-3.832135773	2.00E-08	6.79E-08	down	--
Solyc10g083330.1	116	94	160	16	21	15	58.58032	94.4286	22.7320691	-2.075757193	2.01E-08	6.81E-08	down	--
Solyc04g076190.1	2	2	9	39	33	26	22.96696	3.26617	42.6677633	3.684668528	2.02E-08	6.84E-08	up	Aspartyl protease
novel.1812	63	38	36	95	137	96	89.39847	35.2771	143.519818	2.020634879	2.04E-08	6.93E-08	up	--
Solyc06g066070.1	12	4	8	40	23	49	26.36809	6.1395	46.5966722	2.926437441	2.10E-08	7.12E-08	up	Glucose-6-phosphate/phosphate
Solyc06g060540.3	73	67	62	9	4	5	29.84863	51.983	7.71427505	-2.761050848	2.13E-08	7.19E-08	down	--
Solyc06g064560.4	72	82	88	9	5	15	36.98264	62.1181	11.8471425	-2.358081822	2.13E-08	7.19E-08	down	--
novel.1096	0	2	1	22	33	25	17.82303	0.77834	34.8677254	5.49117026	2.16E-08	7.29E-08	up	--
Solyc07g043110.2	6	4	1	36	25	25	19.97127	2.86294	37.0796036	3.703345567	2.17E-08	7.34E-08	up	UDP-glucuronosyl and UDP-glucosyl transferase
novel.1236	5	5	3	53	15	36	23.49843	3.36279	43.6340659	3.706298044	2.18E-08	7.35E-08	up	--
Solyc04g056380.3	71	49	132	0	1	0	32.20307	63.9172	0.48894937	-7.122488089	2.26E-08	7.61E-08	down	Uncharacterized conserved protein
novel.1028	1	2	2	17	22	33	15.75043	1.28339	30.2174621	4.559656651	2.30E-08	7.75E-08	up	--
Solyc08g007480.2	69	67	76	8	1	9	30.8318	54.396	7.26760324	-2.857984186	2.34E-08	7.86E-08	down	--
novel.491	9	4	6	22	33	41	22.78432	4.87065	40.6979944	3.060962597	2.37E-08	7.97E-08	up	--
Solyc07g054810.1	178	124	81	17	10	18	58.93986	98.9955	18.8841924	-2.382893229	2.40E-08	8.06E-08	down	--
novel.685	83	80	107	12	13	7	41.63384	69.1119	14.1557759	-2.321491167	2.43E-08	8.15E-08	down	FOG: Transposon-encoded proteins with TYA
Solyc03g119960.3	55	69	65	6	4	7	27.86358	48.5963	7.13088584	-2.757487691	2.44E-08	8.17E-08	down	--
Solyc08g006920.3	4	0	1	29	17	32	16.96917	1.28132	32.6570147	4.672724921	2.50E-08	8.37E-08	up	Leucine rich repeat proteins
Solyc03g116160.1	1	2	0	22	19	39	16.95737	0.79081	33.1239195	5.427025016	2.52E-08	8.43E-08	up	--
novel.548	79	86	64	11	3	7	33.95576	59.0826	8.82889119	-2.732857207	2.54E-08	8.48E-08	down	--
Solyc01g006660.3	119	85	133	8	19	9	51.11474	86.1608	16.068692	-2.457630496	2.54E-08	8.50E-08	down	--
Solyc01g094320.3	0	2	0	29	27	34	19.40367	0.53205	38.2752921	6.214728111	2.55E-08	8.53E-08	up	Regulatory protein MLP and related LIM proteins
Solyc02g090490.3	180	99	167	9	24	21	68.68335	114.043	23.3235315	-2.30114295	2.56E-08	8.56E-08	down	Ca2+-independent phospholipase A2
Solyc07g051860.1	10	28	30	58	85	73	55.47748	17.425	93.5299738	2.419956317	2.61E-08	8.72E-08	up	MEKK and related serine/threonine protein kinases
Solyc02g071910.1	1	1	2	26	13	42	17.02516	1.01736	33.0329625	5.019559038	2.70E-08	9.01E-08	up	Aquaporin (major intrinsic protein family)
Solyc01g057260.4	22	8	23	48	44	70	40.75074	13.4855	68.0159646	2.331656715	2.80E-08	9.32E-08	up	--
Solyc01g014840.3	146	72	135	14	14	18	54.85464	90.1815	19.5278171	-2.209011046	2.86E-08	9.49E-08	down	--
Solyc09g098350.4	55	60	84	8	3	5	28.83473	50.8815	6.78793473	-2.906317526	2.99E-08	9.92E-08	down	--
Solyc08g075190.1	61	53	63	0	0	3	23.24649	45.3998	1.09317544	-5.179242829	3.01E-08	9.97E-08	down	--
Solyc07g047870.1	0	1	1	34	22	33	19.08271	0.51232	37.6531081	6.197415413	3.04E-08	1.01E-07	up	--
Solyc04g014610.4	94	71	52	259	131	136	141.456	56.0181	226.893909	2.017870873	3.06E-08	1.01E-07	up	Uncharacterized conserved protein
Solyc05g052015.1	6	4	5	29	32	23	20.27991	3.84809	36.711729	3.244211189	3.12E-08	1.03E-07	up	--

Solyc10g086320.2	81	70	68	8	4	12	33.07824	56.3289	9.82762679	-2.486867008	3.13E-08	1.03E-07	down	Phosphatidylinositol-4-phosphate 5-kinase
Solyc08g006750.4	0	1	4	20	18	57	19.78521	1.25118	38.3192409	4.918695185	3.13E-08	1.03E-07	up	Glutamate decarboxylase and related proteins
Solyc03g026000.4	46	60	78	4	5	2	25.99903	47.075	4.92309427	-3.311719305	3.16E-08	1.04E-07	down	--
Solyc04g079780.4	5	13	9	34	32	39	25.84885	6.96874	44.7289527	2.684736268	3.21E-08	1.06E-07	up	Predicted E3 ubiquitin ligase
Solyc02g086820.3	1	0	3	31	21	22	16.42065	0.99762	31.843676	4.959148075	3.21E-08	1.06E-07	up	Predicted carbonic anhydrase
Solyc01g006300.3	1	0	1	23	37	31	19.97616	0.50505	39.4472647	6.259841165	3.22E-08	1.06E-07	up	--
Solyc05g054480.3	13	15	29	41	55	70	42.41465	14.4966	70.3326711	2.273141871	3.27E-08	1.08E-07	up	Actin and related proteins
Solyc04g080690.3	0	0	4	21	25	32	17.02732	0.98515	33.0694822	5.021091409	3.28E-08	1.08E-07	up	Phosphatidylinositol transfer protein SEC14
Solyc05g054650.1	1	0	0	14	37	50	21.34647	0.25876	42.4341906	7.264761431	3.41E-08	1.12E-07	up	FOG: Zn-finger
Solyc01g066730.4	15	21	19	74	32	59	41.82992	14.1474	69.5124267	2.299848931	3.45E-08	1.13E-07	up	MADS box transcription factor
Solyc04g077620.1	146	72	174	11	17	7	57.73045	99.7867	15.6741824	-2.701787843	3.45E-08	1.14E-07	down	--
Solyc08g006765.1	104	65	93	7	8	16	39.95548	67.1074	12.8036006	-2.370022081	3.52E-08	1.16E-07	down	Diamine acetyltransferase
Solyc03g078420.4	0	2	0	22	38	33	20.37983	0.53205	40.2276068	6.281477733	3.53E-08	1.16E-07	up	--
novel.142	49	30	46	0	0	0	15.9946	31.9892	0	-7.085037299	3.54E-08	1.16E-07	down	--
Solyc08g080060.4	0	0	2	29	21	41	19.19246	0.49258	37.8923385	6.215471502	3.57E-08	1.17E-07	up	--
Solyc04g050990.3	80	98	78	7	3	16	38.1703	65.9818	10.3588537	-2.621269677	3.66E-08	1.20E-07	down	--
Solyc01g104930.3	43	65	64	1	0	5	23.22008	44.1808	2.25935001	-4.155616822	4.02E-08	1.31E-07	down	AAA+-type ATPase
Solyc07g055215.1	102	85	81	13	10	17	42.86258	68.9549	16.7702368	-2.029909088	4.04E-08	1.32E-07	down	Protein involved in mRNA turnover and stability
Solyc06g035700.1	61	57	50	2	5	4	24.01963	43.2622	4.777096	-3.204225975	4.06E-08	1.32E-07	down	--
Solyc03g083730.1	63	37	65	3	1	3	22.52388	42.1535	2.89429764	-3.83673076	4.08E-08	1.33E-07	down	--
Solyc01g008420.3	112	73	131	363	432	140	250.8392	80.6646	421.013896	2.38139871	4.08E-08	1.33E-07	up	Uncharacterized membrane protein
Solyc04g074790.3	83	92	86	7	7	19	40.27	67.1322	13.4078267	-2.289895094	4.25E-08	1.38E-07	down	FOG: Predicted E3 ubiquitin ligase
Solyc12g095980.2	46	53	67	4	0	4	22.85537	42.5036	3.20713103	-3.666487693	4.40E-08	1.43E-07	down	--
Solyc04g150112.1	72	83	100	10	10	16	40.21665	65.3396	15.0936722	-2.105348431	4.48E-08	1.45E-07	down	--
Solyc07g018143.1	10	14	25	51	51	46	38.23727	12.4692	64.0053796	2.34977217	4.50E-08	1.46E-07	up	--
novel.1795	2	4	6	24	28	25	18.17856	3.05936	33.2977604	3.43145224	4.51E-08	1.46E-07	up	--
Solyc02g087510.3	105	75	101	17	10	14	44.71166	71.9967	17.4266252	-2.048427119	4.61E-08	1.49E-07	down	Putative serine/threonine protein kinase
Solyc11g069020.3	92	105	158	4	18	9	52.24115	90.6521	13.8301788	-2.744646699	4.62E-08	1.50E-07	down	Apoptotic ATPase
Solyc01g110720.2	11	16	11	34	32	50	29.2746	9.81194	48.7372627	2.319631286	4.68E-08	1.51E-07	up	--
Solyc02g082560.1	105	72	136	17	6	6	46.1872	79.8187	12.5556932	-2.681150429	4.69E-08	1.52E-07	down	CTP synthase (UTP-ammonia lyase)
Solyc00g118690.1	0	1	4	25	18	29	15.7772	1.25118	30.3032249	4.571724187	4.70E-08	1.52E-07	up	NADH dehydrogenase subunit 1
Solyc04g055255.1	84	97	42	0	0	1	29.12438	57.8844	0.36439181	-6.977126351	4.71E-08	1.52E-07	down	--
Solyc12g009730.3	67	35	78	1	0	3	23.69438	45.8582	1.53056638	-4.783375155	4.71E-08	1.52E-07	down	FOG: Leucine rich repeat
Solyc04g074680.1	488	311	283	9	52	43	161.8692	278.708	45.0307338	-2.635325129	4.81E-08	1.55E-07	down	--
Solyc05g006220.3	598	473	218	68	34	81	205.0706	334.259	75.8825997	-2.134029784	4.81E-08	1.55E-07	down	--
Solyc02g093580.4	22	19	42	65	75	66	55.12138	21.0913	89.151474	2.070329763	4.82E-08	1.55E-07	up	--
Solyc04g079990.4	53	61	47	4	3	3	22.91347	41.5174	4.30958733	-3.284184691	4.96E-08	1.60E-07	down	--
Solyc01g086680.4	100	41	36	193	554	521	295.3959	45.6492	545.142539	3.578090295	4.99E-08	1.61E-07	up	Glutathione S-transferase
Solyc11g011350.3	46	43	59	187	94	85	98.29984	37.873	158.726652	2.0641698	5.13E-08	1.65E-07	up	--
Solyc10g084510.1	104	96	84	1	1	14	39.58271	73.1376	6.02782569	-3.514414817	5.20E-08	1.67E-07	down	--
Solyc12g006440.2	182	159	89	15	18	26	68.07398	111.312	24.83614	-2.159802059	5.20E-08	1.67E-07	down	--
Solyc01g009830.3	88	88	41	5	4	6	31.30398	56.2789	6.32910309	-3.146959506	5.40E-08	1.73E-07	down	Nucleotide-sugar transporter VRG4/SQV-7
Solyc03g113980.3	204	105	180	17	29	27	78.25251	125.051	31.4537568	-2.000472883	5.45E-08	1.74E-07	down	--
Solyc02g084670.4	19	10	8	40	34	49	30.76104	9.54697	51.9751153	2.448884989	5.72E-08	1.83E-07	up	--
Solyc01g086670.3	280	87	45	1245	1559	1740	1023.772	106.679	1940.86555	4.185512537	5.77E-08	1.84E-07	up	--
Solyc02g071430.3	7	0	3	32	38	24	21.93608	2.55017	41.3219899	4.006365421	5.77E-08	1.84E-07	up	Iron/ascorbate family oxidoreductases
Solyc08g082110.4	254	158	275	4	10	42	98.71474	175.486	21.9435136	-2.973690579	5.88E-08	1.87E-07	down	--
Solyc11g006955.1	2	1	4	24	14	37	16.29693	1.7687	30.8251709	4.115410858	5.97E-08	1.90E-07	up	--
Solyc08g082485.1	64	52	76	0	0	1	24.7381	49.1118	0.36439181	-6.741845075	5.98E-08	1.91E-07	down	--

Solyc01g091320.3	0	0	3	24	42	20	19.52998	0.73887	38.3210926	5.640790622	6.07E-08	1.93E-07	up	C-4 sterol methyl oxidase
Solyc02g063350.1	96	50	93	5	7	11	35.3324	61.0469	9.61791027	-2.656491461	6.11E-08	1.94E-07	down	Calmodulin and related proteins (EF-Hand superfamily)
Solyc01g079300.4	12	3	4	28	42	36	25.39462	4.88832	45.9009254	3.227783126	6.15E-08	1.96E-07	up	--
Solyc05g009490.3	18	22	19	62	82	40	48.48874	15.1897	81.7877596	2.42182339	6.21E-08	1.97E-07	up	--
Solyc09g090480.2	1	0	0	25	22	30	16.44109	0.25876	32.6234142	6.884734339	6.35E-08	2.02E-07	up	--
Solyc04g050755.1	58	68	81	6	9	7	31.31139	53.0471	9.57563271	-2.494419751	6.35E-08	2.02E-07	down	--
Solyc07g065990.1	110	101	122	131	253	782	275.668	85.3792	465.956802	2.44964157	6.42E-08	2.04E-07	up	--
Solyc06g009840.3	13	6	18	37	34	52	30.57466	9.3932	51.7561179	2.456629321	6.49E-08	2.06E-07	up	Fumarase
Solyc09g010605.1	67	62	49	4	4	7	26.07733	45.8986	6.25610396	-2.858347003	6.93E-08	2.19E-07	down	--
Solyc12g009875.1	117	145	85	3	7	21	51.08506	89.7831	12.3870465	-2.822944454	7.22E-08	2.28E-07	down	--
Solyc02g087450.3	79	31	82	1	0	4	25.38964	48.8843	1.89495819	-4.56724452	7.22E-08	2.28E-07	down	--
Solyc01g095905.1	1	0	0	31	21	25	16.5978	0.25876	32.9368514	6.896251209	7.38E-08	2.33E-07	up	Predicted membrane protein
Solyc01g080970.3	0	0	0	24	20	10	11.96014	0	23.9202883	7.390526934	7.58E-08	2.39E-07	up	--
Solyc09g091510.3	577	2823	3127	16090	15190	16321	11041.22	1670.44	20412	3.611101566	7.89E-08	2.48E-07	up	--
Solyc03g031910.3	235	149	90	221	492	523	325.2077	122.612	527.803408	2.106152837	8.06E-08	2.53E-07	up	tRNA-splicing endonuclease positive effector (SEN1)
Solyc03g031800.3	259	235	196	23	13	62	108.4078	177.807	39.0086259	-2.165998457	8.17E-08	2.57E-07	down	--
Solyc01g091840.4	80	72	138	10	13	12	44.47266	73.8424	15.102953	-2.303809533	8.17E-08	2.57E-07	down	UDP-galactose transporter related protein
Solyc04g081870.4	11	8	25	53	62	43	40.1486	11.1318	69.1654291	2.6222956	8.18E-08	2.57E-07	up	--
Solyc12g006590.3	80	82	50	8	5	7	31.66193	54.8292	8.49461711	-2.690028864	8.22E-08	2.58E-07	down	RNA-binding Ran Zn-finger protein and related proteins
Solyc06g060870.1	72	44	51	0	2	1	22.11936	42.8964	1.34229056	-5.067018934	8.26E-08	2.59E-07	down	Predicted lipase
novel.483	0	1	0	12	41	39	19.88646	0.26603	39.5068963	7.158201614	8.26E-08	2.59E-07	up	--
Solyc11g005280.1	42	66	68	0	2	6	24.16874	45.1732	3.16424962	-3.765642953	8.29E-08	2.60E-07	down	FOG: Predicted E3 ubiquitin ligase
Solyc08g016583.1	4	4	3	26	15	33	16.78467	2.838	30.731335	3.448229193	8.34E-08	2.61E-07	up	--
Solyc01g108480.1	1	0	0	19	20	42	16.82631	0.25876	33.3938715	6.924686227	8.65E-08	2.71E-07	up	Serine carboxypeptidases (lysosomal cathepsin A)
Solyc03g071850.1	4	0	0	19	55	24	22.49154	1.03503	43.948047	5.409018668	8.70E-08	2.72E-07	up	UDP-glucuronosyl and UDP-glucosyl transferase
Solyc01g102380.5.1	0	0	1	31	19	26	16.28482	0.24629	32.3233445	6.870721463	8.72E-08	2.73E-07	up	--
Solyc02g079080.2	55	43	53	4	0	2	20.60123	38.7241	2.4783474	-3.934369763	9.19E-08	2.87E-07	down	--
novel.392	13	5	12	18	58	135	46.53722	7.64944	85.4249953	3.48172777	9.51E-08	2.96E-07	up	--
Solyc01g100020.4	45	47	61	90	192	101	104.609	39.171	170.047038	2.112670618	9.54E-08	2.97E-07	up	Phospholipase D1
Solyc04g051470.1	124	116	54	6	11	10	43.94567	76.2446	11.6467069	-2.72188456	9.67E-08	3.01E-07	down	--
novel.639	2	3	2	36	9	42	18.62962	1.80817	35.4510745	4.306681979	9.70E-08	3.02E-07	up	MADS box transcription factor
Solyc01g081185.1	95	89	82	10	16	9	41.96532	68.454	15.4766257	-2.177107046	9.75E-08	3.03E-07	down	--
Solyc08g007980.3	16	11	12	37	60	36	34.3302	10.0219	58.6385326	2.540072119	9.93E-08	3.09E-07	up	--
Solyc06g069460.3	0	0	1	16	35	29	17.46257	0.24629	34.6788457	6.967567752	9.94E-08	3.09E-07	up	--
Solyc07g041230.3	7	7	13	31	23	84	31.14455	6.87524	55.4138671	3.015408421	1.00E-07	3.11E-07	up	--
Solyc06g062830.4	77	66	115	7	8	16	39.30444	65.8053	12.8036006	-2.343158877	1.00E-07	3.11E-07	down	--
novel.1138	59	83	66	6	9	4	31.04222	53.602	8.48245727	-2.70365136	1.00E-07	3.12E-07	down	--
Solyc08g081480.3	42	38	56	148	135	66	94.78044	34.769	154.791885	2.148810632	1.02E-07	3.15E-07	up	--
Solyc09g075320.1	61	47	46	3	3	4	21.92666	39.6167	4.2365882	-3.224039832	1.02E-07	3.16E-07	down	FOG: Predicted E3 ubiquitin ligase
Solyc05g056080.4	81	40	98	4	5	8	31.42307	55.7367	7.10944514	-2.961184433	1.08E-07	3.33E-07	down	--
Solyc06g050140.1	44	48	52	2	2	4	20.13594	36.9616	3.31024788	-3.453985255	1.11E-07	3.43E-07	down	--
novel.2062	34	79	74	0	1	5	25.17508	48.0393	2.31090843	-4.279041147	1.12E-07	3.45E-07	down	--
Solyc02g068610.2	1	6	0	18	25	60	21.9076	1.85492	41.9602801	4.531091436	1.12E-07	3.46E-07	up	FOG: Ankyrin repeat
Solyc06g006010.3	48	54	61	2	3	7	23.3509	41.8094	4.89237269	-3.056106328	1.14E-07	3.50E-07	down	--

Solyc03g093700.3	66	55	40	3	3	4	22.8988	41.561	4.2365882	-3.292582057	1.14E-07	3.50E-07	down	Histone H3 (Lys9) methyltransferase SUV39H1/Clr4
Solyc07g006480.4	248	70	181	11	6	26	72.29554	127.372	17.2191837	-2.862646854	1.14E-07	3.52E-07	down	--
Solyc06g084180.3	0	0	2	33	32	19	18.74815	0.49258	37.0037255	6.17083535	1.19E-07	3.66E-07	up	--
novel.1254	55	44	61	3	0	0	21.13631	40.9604	1.31217283	-5.002007305	1.21E-07	3.71E-07	down	--
Solyc10g075103.1	63	35	32	62	122	280	111.1468	33.4939	188.79977	2.49792958	1.24E-07	3.81E-07	up	--
Solyc09g064740.4	96	96	125	5	19	13	48.68973	81.1654	16.2140864	-2.346540442	1.27E-07	3.89E-07	down	3'-5' exonuclease
Solyc05g011820.3	75	77	93	14	8	15	39.14833	62.7957	15.5009454	-2.008222791	1.30E-07	3.98E-07	down	Predicted serine protease
Solyc06g006090.1	73	48	39	2	2	0	21.55826	41.2638	1.85268064	-4.579101528	1.33E-07	4.06E-07	down	Uncharacterized conserved protein
novel.1014	63	80	75	12	4	9	33.26977	56.0555	10.4840151	-2.407857338	1.33E-07	4.06E-07	down	--
Solyc01g081586.1	6	7	3	22	21	42	19.6743	4.1536	35.1949937	3.098808241	1.35E-07	4.12E-07	up	1,3-beta-glucan synthase/callose synthase catalytic subunit
Solyc04g016200.1	16	9	9	38	24	78	32.76458	8.75096	56.7782022	2.70815965	1.35E-07	4.12E-07	up	UDP-glucuronosyl and UDP-glucosyl transferase
novel.1379	41	19	22	44	142	112	75.2849	21.0819	129.487896	2.61665993	1.38E-07	4.21E-07	up	--
Solyc04g005770.3	64	45	51	3	5	3	22.97125	41.0924	4.85009514	-3.123034795	1.38E-07	4.23E-07	down	FOG: PPR repeat
Solyc01g107760.4	6	6	4	24	31	24	19.26704	4.13386	34.4002167	3.053594675	1.48E-07	4.49E-07	up	Cysteine proteinase Cathepsin L
novel.938	71	54	39	0	4	2	22.51353	42.3425	2.68458112	-4.037738699	1.49E-07	4.54E-07	down	--
Solyc04g015820.1	24	10	9	65	29	70	39.60221	11.087	68.1173701	2.624919196	1.50E-07	4.56E-07	up	--
Solyc08g083270.3	53	60	48	3	4	6	23.47597	41.4976	5.4543212	-2.916591104	1.52E-07	4.61E-07	down	Glutaredoxin-related protein
Solyc01g108440.2	59	37	129	2	0	6	29.97102	56.8809	3.06113276	-4.124642174	1.52E-07	4.61E-07	down	--
Solyc03g113480.1	186	507	680	47	66	71	214.5904	350.481	78.6998517	-2.156862506	1.53E-07	4.63E-07	down	--
Solyc10g086740.3	0	0	2	34	13	41	18.33014	0.49258	36.1676983	6.152190823	1.54E-07	4.69E-07	up	--
Solyc05g024380.1	74	62	121	6	12	9	38.60695	65.4426	11.7712645	-2.497553199	1.56E-07	4.73E-07	down	--
Solyc12g021160.1	108	103	97	11	9	27	49.1435	79.2366	19.0504237	-2.025000885	1.58E-07	4.79E-07	down	--
Solyc08g013950.1	2	2	0	23	12	37	15.22973	1.04957	29.4098812	4.841335819	1.59E-07	4.82E-07	up	--
Solyc02g079490.3	21	48	30	61	188	104	91.04597	25.5919	156.500078	2.610715421	1.59E-07	4.84E-07	up	--
Solyc06g071540.3	37	26	12	65	62	76	52.94262	19.4462	86.4390503	2.15541895	1.62E-07	4.91E-07	up	--
Solyc12g013830.2	17	35	19	44	61	140	59.23764	18.3893	100.085967	2.451801359	1.66E-07	5.03E-07	up	--
Solyc04g077535.1	0	0	0	28	4	34	13.29603	0	26.5920655	7.558427022	1.68E-07	5.08E-07	up	--
Solyc05g018870.3	48	40	55	1	0	3	19.06894	36.6073	1.53056638	-4.453870167	1.73E-07	5.23E-07	down	Endosomal membrane proteins, EMP70
novel.103	125	43	48	4	1	5	29.83307	55.6057	4.06047221	-3.737456775	1.74E-07	5.25E-07	down	--
novel.507	66	34	68	2	0	5	22.78363	42.8705	2.69674095	-3.890948154	1.74E-07	5.26E-07	down	--
Solyc03g006470.3	13	8	14	34	30	39	26.34558	8.9401	43.751054	2.286396848	1.75E-07	5.28E-07	up	--
Solyc07g055720.4	100	73	125	19	9	9	46.03614	76.0818	15.9904986	-2.26269416	1.75E-07	5.28E-07	down	Molecular chaperone (small heat-shock protein Hsp26/Hsp42)
Solyc02g084005.1	262	146	120	9	16	36	80.53339	136.189	24.8778137	-2.43751579	1.76E-07	5.31E-07	down	--
Solyc11g071990.2	83	40	43	1	1	1	21.99954	42.7083	1.29073213	-5.066009935	1.78E-07	5.36E-07	down	NADH-dehydrogenase (ubiquinone)
Solyc08g069020.1	35	57	56	2	0	2	19.80789	38.0122	1.60356551	-4.498387482	1.80E-07	5.41E-07	down	--
Solyc07g043490.1	123	83	102	20	11	8	48.03511	79.0288	17.0413965	-2.231841142	1.80E-07	5.43E-07	down	UDP-glucuronosyl and UDP-glucosyl transferase
Solyc02g076920.3	19	16	17	50	30	49	33.87648	13.3597	54.3932272	2.028307967	1.80E-07	5.43E-07	up	--
Solyc11g070140.2	63	66	103	6	7	14	35.18785	59.2272	11.1484766	-2.389738549	1.82E-07	5.49E-07	down	Protein kinase PCTAIRE and related kinases
Solyc05g015850.4	42	23	48	66	176	99	89.90298	28.8083	150.997681	2.384371952	1.83E-07	5.50E-07	up	--
Solyc07g032390.2	48	62	38	2	3	2	20.67171	38.273	3.07041363	-3.675673834	1.86E-07	5.59E-07	down	--
Solyc02g088210.3	56	47	59	115	257	92	125.5044	41.5247	209.483994	2.330523881	1.87E-07	5.62E-07	up	Protein involved in vacuolar polyphosphate accumulation
Solyc03g079960.4	28	13	11	49	41	61	38.55985	13.4127	63.7069811	2.25141156	2.10E-07	6.30E-07	up	--
Solyc06g074240.3	67	68	93	12	8	12	35.9322	58.3314	13.5329881	-2.106123373	2.14E-07	6.41E-07	down	--
Solyc06g007275.1	106	134	130	5	22	8	55.4762	95.0934	15.8589754	-2.620054219	2.16E-07	6.45E-07	down	--

Solyc08g080040.4	122	81	174	11	21	19	58.98674	95.9708	22.0026817	-2.136514993	2.28E-07	6.79E-07	down	Iron/ascorbate family oxidoreductases
Solyc04g017740.1	2	0	0	32	18	25	16.21245	0.51751	31.9073942	5.955511795	2.31E-07	6.89E-07	up	--
Solyc07g045570.3	42	49	55	3	4	3	20.90508	37.449	4.36114576	-3.132438222	2.36E-07	7.03E-07	down	--
Solyc10g075150.2	43	13	45	147	530	242	218.6452	25.6679	411.622455	4.001673621	2.48E-07	7.35E-07	up	--
Solyc10g081520.1	0	1	0	19	21	25	13.97709	0.26603	27.6881601	6.646334762	2.59E-07	7.68E-07	up	--
Solyc02g077790.1	38	38	84	3	1	2	21.57998	40.63	2.52990583	-4.000040654	2.60E-07	7.71E-07	down	--
Solyc02g078700.3	65	55	96	9	8	4	32.20005	55.0944	9.30568073	-2.601810791	2.62E-07	7.77E-07	down	LisH motif-containing protein
Solyc02g077840.2	56	40	48	1	0	2	19.05976	36.9533	1.16617457	-4.874616479	2.68E-07	7.92E-07	down	--
novel.1920	0	0	1	24	21	20	14.14972	0.24629	28.0531557	6.662000247	2.71E-07	8.00E-07	up	FOG: Transposon-encoded proteins with TYA
Solyc12g036810.3	76	66	75	8	12	10	34.35271	55.695	13.0104382	-2.117889734	2.74E-07	8.09E-07	down	Kinesin (KAR3 subfamily)
Solyc01g079615.1	2	7	9	23	50	30	25.01776	4.5963	45.4392148	3.294565126	2.75E-07	8.11E-07	up	FOG: Transposon-encoded proteins with TYA
Solyc01g067370.3	2	1	0	23	30	15	15.48895	0.78354	30.1943501	5.27806198	2.76E-07	8.16E-07	up	--
novel.317	119	93	86	16	17	10	47.83388	76.7135	18.9543126	-2.042676939	2.78E-07	8.20E-07	down	--
Solyc01g079550.2	7	4	5	25	16	41	18.90244	4.10685	33.6980279	3.045717583	2.78E-07	8.20E-07	up	--
Solyc07g017950.3	11	8	11	30	31	32	23.81171	7.68372	39.9396969	2.371082093	2.79E-07	8.22E-07	up	--
Solyc12g006620.2	37	46	51	1	1	2	18.01356	34.372	1.65512394	-4.346634961	2.81E-07	8.27E-07	down	Uncharacterized conserved protein
Solyc06g051250.1	60	75	61	5	5	12	29.75274	50.5011	9.00440333	-2.456283487	2.82E-07	8.30E-07	down	FOG: Predicted E3 ubiquitin ligase
Solyc07g042230.1	23	35	40	0	0	0	12.55696	25.1139	0	-6.735673043	2.91E-07	8.55E-07	down	--
Solyc04g074830.1	206	197	379	783	2559	1111	1098.796	199.055	1998.53786	3.327370541	2.92E-07	8.57E-07	up	Clathrin assembly protein AP180 and related proteins
Solyc02g076860.3	0	4	8	42	36	21	23.32962	3.03442	43.6248252	3.829742488	2.92E-07	8.57E-07	up	--
Solyc09g061830.1	69	80	70	3	9	13	33.41322	56.3766	10.4498108	-2.421050012	3.00E-07	8.81E-07	down	--
Solyc03g112620.4	156	83	111	13	10	27	55.09928	89.7844	20.4141549	-2.114895439	3.02E-07	8.85E-07	down	--
Solyc09g075660.3	19	7	10	32	50	43	31.67714	9.24146	54.1128268	2.544550948	3.02E-07	8.85E-07	up	--
Solyc09g072640.3	0	0	2	30	21	21	15.76724	0.49258	31.0418932	5.920488548	3.09E-07	9.05E-07	up	--
Solyc01g005390.3	12	14	19	29	71	50	38.56414	11.509	65.6193335	2.503214041	3.13E-07	9.17E-07	up	Diadenosine and diphosphoinositol polyphosphate phosphohydrolase
novel.1375	48	45	60	4	5	2	22.04599	39.1689	4.92309427	-3.047087098	3.15E-07	9.20E-07	down	--
Solyc10g082020.2	3	5	4	15	29	29	17.19966	3.09156	31.3077585	3.339222364	3.15E-07	9.21E-07	up	--
Solyc08g081700.1	15	8	4	33	25	82	31.76625	6.99473	56.5377641	3.026945262	3.22E-07	9.41E-07	up	--
Solyc08g007150.1	6	7	9	23	32	29	20.95253	5.63133	36.2737342	2.678427239	3.27E-07	9.56E-07	up	--
novel.1862	57	47	42	1	3	5	20.66138	37.5966	3.72619813	-3.310432783	3.31E-07	9.65E-07	down	--
Solyc03g120050.3	59	43	44	1	0	5	19.90095	37.5425	2.25935001	-3.922856665	3.45E-07	1.01E-06	down	--
novel.2063	32	52	93	0	1	0	22.75372	45.0185	0.48894937	-6.616615738	3.51E-07	1.02E-06	down	--
Solyc07g063560.4	21	15	25	61	63	38	43.45654	15.5815	71.331547	2.184195965	3.56E-07	1.04E-06	up	--
Solyc04g076970.3	86	43	44	1	5	1	23.88776	44.529	3.24652962	-3.864857949	3.66E-07	1.06E-06	down	--
Solyc02g067010.3	74	74	77	11	5	16	35.44229	57.7983	13.0863162	-2.112552869	3.76E-07	1.09E-06	down	Pattern-formation protein/guanine nucleotide exchange factor
novel.1767	460	428	226	37	27	101	177.3689	288.549	66.188671	-2.112541554	3.78E-07	1.10E-06	down	--
Solyc11g005710.2	46	62	53	4	5	6	23.91524	41.4498	6.38066152	-2.702519485	3.87E-07	1.12E-06	down	WD40 repeat-containing protein
Solyc02g089960.3	13	22	14	40	32	55	32.92403	12.6645	53.1835674	2.078488246	3.89E-07	1.13E-06	up	--
novel.761	87	79	42	5	1	9	29.91379	53.8721	5.9554304	-3.122634938	3.97E-07	1.15E-06	down	--
Solyc02g088120.4	50	58	86	8	6	7	29.26591	49.5483	8.98356648	-2.471940745	4.07E-07	1.18E-06	down	PolyC-binding proteins alphaCP-1
Solyc07g055870.4	65	92	104	14	13	11	41.69793	66.9077	16.488125	-2.037953002	4.13E-07	1.19E-06	down	Tyrosine kinase specific for activated (GTP-bound) p21cdc42Hs
Solyc01g107220.2	2	1	2	21	12	26	12.90145	1.27612	24.5267894	4.264274912	4.14E-07	1.20E-06	up	--
Solyc12g011033.1	231	126	89	8	5	28	65.67943	115.212	16.1468452	-2.802785627	4.31E-07	1.24E-06	down	--
Solyc11g040330.2	19	20	38	54	97	59	56.07111	19.5959	92.5463171	2.230265332	4.32E-07	1.24E-06	up	--
Solyc11g007390.1	30	78	100	141	196	159	134.2931	53.1417	215.444498	2.016667569	4.36E-07	1.26E-06	up	UDP-glucuronosyl and UDP-glucosyl transferase

Solyc11g005970.1	75	74	66	8	8	15	34.11222	55.3478	12.8765997	-2.086013828	4.38E-07	1.26E-06	down	FOG: PPR repeat
Solyc02g068760.4	59	61	72	6	10	7	29.64585	49.2271	10.0645821	-2.318912273	4.42E-07	1.27E-06	down	FOG: PPR repeat
Solyc11g008050.1	61	61	76	9	5	12	30.74187	50.7298	10.7539671	-2.216306817	4.52E-07	1.30E-06	down	1,4-alpha-glucan branching enzyme/starch branching enzyme II
Solyc01g087860.2	49	52	62	6	4	7	24.45665	41.7824	7.13088584	-2.540517817	4.56E-07	1.31E-06	down	--
Solyc01g067160.4	46	54	82	4	4	9	26.72443	46.464	6.98488758	-2.707335913	4.65E-07	1.34E-06	down	--
Solyc10g005550.3	1	5	1	22	13	38	15.83051	1.83518	29.8258315	4.050293381	4.69E-07	1.35E-06	up	Transcription factor, Myb superfamily
Solyc03g034230.3	10	13	5	25	32	45	25.12808	7.27737	42.9787851	2.571720318	4.80E-07	1.38E-06	up	--
Solyc12g056810.2	0	3	3	22	21	18	13.99327	1.53695	26.4495902	4.096838374	4.83E-07	1.38E-06	up	Cytochrome P450 CYP2 subfamily
Solyc10g005320.3	113	55	25	1	0	0	25.23283	50.0283	0.43739094	-6.767173085	4.84E-07	1.39E-06	down	Tryptophan synthase beta chain
Solyc07g063780.2	0	0	1	13	23	28	13.69059	0.24629	27.1348886	6.61823579	4.86E-07	1.39E-06	up	--
novel.1215	45	48	69	0	0	2	21.06804	41.4073	0.72878362	-5.627874868	4.97E-07	1.42E-06	down	--
Solyc09g150117.1	29	21	45	0	0	0	12.08676	24.1735	0	-6.681725804	5.00E-07	1.43E-06	down	--
Solyc03g095940.1	64	57	67	7	8	10	29.4213	48.2253	10.6172497	-2.185473934	5.08E-07	1.45E-06	down	--
Solyc09g011227.1	8	4	4	33	29	19	19.8281	4.11932	35.5368774	3.102426211	5.12E-07	1.46E-06	up	--
Solyc07g065110.1	27	13	32	68	111	48	59.9164	18.326	101.506772	2.460785414	5.14E-07	1.47E-06	up	--
Solyc01g108750.2	71	63	77	11	8	13	33.77784	54.0957	13.4599889	-2.000118394	5.27E-07	1.50E-06	down	--
Solyc10g083900.2	1	0	0	24	13	25	13.11114	0.25876	25.9635198	6.558802863	5.27E-07	1.50E-06	up	Transcription factor, Myb superfamily
Solyc09g010780.3	45	61	84	7	0	6	26.90403	48.56	5.24808748	-3.166649723	5.34E-07	1.52E-06	down	Protein phosphatase 2C/pyruvate dehydrogenase (lipoamide) phosphatase
Solyc09g008080.3	145	82	72	4	9	18	44.88798	77.0668	12.7091608	-2.580841974	5.49E-07	1.56E-06	down	--
Solyc09g092350.1	0	0	1	22	25	15	13.77925	0.24629	27.3122123	6.619305322	5.54E-07	1.58E-06	up	--
Solyc06g071610.3	2	2	2	20	12	26	12.81577	1.54215	24.0893985	3.973399325	5.59E-07	1.59E-06	up	Dehydrogenases with different specificities
Solyc02g084570.4	71	87	114	11	6	21	42.49513	69.593	15.3972247	-2.145184167	5.68E-07	1.61E-06	down	Cytochrome P450 CYP2 subfamily
novel.409	2	0	2	8	36	33	17.06816	1.01009	33.1262348	5.01579382	5.69E-07	1.61E-06	up	--
Solyc01g099170.4	4	9	9	38	18	35	21.91076	5.64587	38.175658	2.760569435	5.72E-07	1.62E-06	up	--
Solyc06g063340.3	103	73	77	17	8	11	40.19587	65.0362	15.355551	-2.085645879	5.74E-07	1.63E-06	down	--
Solyc02g068670.3	88	31	60	4	4	2	25.11446	45.7948	4.43414489	-3.408234634	5.78E-07	1.64E-06	down	FOG: Ankyrin repeat
novel.527	73	74	85	2	4	16	34.08533	59.5098	8.66084838	-2.727300083	5.94E-07	1.68E-06	down	DNA-binding protein C1D
Solyc09g010430.1	1306	731	544	141	53	211	415.4284	666.384	164.473112	-2.014330306	5.95E-07	1.68E-06	down	--
Solyc09g025220.2	1	0	1	27	10	39	15.70769	0.50505	30.9103299	5.923677666	6.09E-07	1.72E-06	up	--
novel.1247	7	16	2	40	34	45	28.53893	6.56031	50.517548	2.957061722	6.17E-07	1.74E-06	up	--
Solyc01g110940.3	14	11	13	34	32	34	26.32882	9.75065	42.9069937	2.132503986	6.19E-07	1.75E-06	up	--
Solyc05g005340.3	26	12	34	32	95	158	68.15722	18.2938	118.020607	2.688338221	6.40E-07	1.80E-06	up	--
novel.1196	20	29	8	55	40	105	48.36793	14.8602	81.8756171	2.472582941	6.52E-07	1.83E-06	up	--
Solyc10g085310.1	37	36	26	63	140	67	72.98865	25.5545	120.422793	2.232162896	6.73E-07	1.89E-06	up	--
Solyc02g082410.4	12	5	12	39	21	41	24.82847	7.39068	42.266248	2.514783736	6.80E-07	1.91E-06	up	Predicted transporter (major facilitator superfamily)
Solyc04g064610.3	27	59	54	75	159	111	93.4882	35.9816	150.994762	2.06600601	6.87E-07	1.93E-06	up	Multitransmembrane protein
novel.1026	8	14	19	30	41	46	30.2023	10.4739	49.930676	2.247802668	6.95E-07	1.95E-06	up	--
Solyc01g006400.4	5	10	33	54	50	103	48.84026	12.0816	85.5989363	2.820212479	6.98E-07	1.96E-06	up	--
Solyc05g050010.3	161	331	384	836	3147	1617	1358.947	224.29	2493.60407	3.474648852	7.02E-07	1.97E-06	up	1-aminocyclopropane-1-carboxylate synthase
Solyc04g079860.1	100	66	124	9	17	15	45.84392	73.9733	17.714535	-2.076313509	7.27E-07	2.04E-06	down	--
Solyc04g058080.3	9	6	5	30	15	46	21.18721	5.15642	37.2179923	2.864213068	7.51E-07	2.10E-06	up	--
Solyc10g080690.2	51	41	53	4	5	2	21.04006	37.157	4.92309427	-2.970297743	7.54E-07	2.11E-06	down	Ca2+-independent phospholipase A2
Solyc03g006300.3	50	28	67	1	1	4	19.63593	36.888	2.38390757	-3.884297983	7.68E-07	2.14E-06	down	--
Solyc10g078170.3	14	11	20	35	34	42	29.35604	11.4747	47.2374179	2.03533094	7.69E-07	2.15E-06	up	Phosphatidylinositol 4-kinase
novel.607	87	32	39	2	0	1	20.93458	40.63	1.2391737	-5.002709053	8.12E-07	2.26E-06	down	--
Solyc06g068890.1	48	59	34	3	3	3	20.18098	36.4898	3.87219639	-3.249626569	8.39E-07	2.33E-06	down	--
Solyc02g079430.4	13	6	12	23	32	48	25.55632	7.91547	43.1971787	2.44718802	8.59E-07	2.39E-06	up	--

Solyc03g116190.2	3	10	3	21	47	28	23.27211	4.17541	42.3688011	3.346562313	8.62E-07	2.39E-06	up	Predicted chitinase
Solyc11g020210.2	98	63	76	10	13	12	37.9694	60.8358	15.102953	-2.024023529	8.80E-07	2.44E-06	down	--
Solyc06g068650.4	23	19	68	109	60	212	91.0086	27.7536	154.26364	2.474208898	8.85E-07	2.45E-06	up	Acyl-CoA synthetase
Solyc04g015710.3	53	47	51	4	6	5	22.64167	38.7781	6.50521908	-2.598053192	9.19E-07	2.55E-06	down	--
Solyc06g076910.3	8	4	6	40	15	30	20.18677	4.6119	35.7616327	2.956039486	9.21E-07	2.55E-06	up	--
Solyc12g013850.2	15	20	19	31	37	66	34.79075	13.8814	55.7001057	2.010996302	9.29E-07	2.57E-06	up	Branching enzyme
novel.780	57	51	59	8	6	5	25.55118	42.8476	8.25478286	-2.398151027	9.38E-07	2.59E-06	down	--
Solyc10g076550.1	95	51	162	12	11	9	45.97736	78.0481	13.9066607	-2.503738993	9.40E-07	2.60E-06	down	--
Solyc03g025670.3	5	2	11	31	57	21	26.80824	4.53501	49.0814616	3.417100418	9.57E-07	2.64E-06	up	--
Solyc10g011990.1	12	9	5	27	21	89	30.61957	6.73077	54.5083636	3.030892831	9.66E-07	2.67E-06	up	--
novel.1786	54	43	41	5	0	2	19.21281	35.5099	2.91573835	-3.584973481	9.75E-07	2.69E-06	down	--
Solyc01g090870.1	1	1	1	10	21	29	12.99014	0.77107	25.2092088	5.033733304	9.78E-07	2.70E-06	up	--
Solyc02g093480.4	220	135	176	34	8	38	84.40841	136.187	32.629776	-2.045466815	9.85E-07	2.71E-06	down	--
Solyc07g062170.3	88	64	103	6	16	9	39.44558	65.1641	13.7270619	-2.27833352	1.00E-06	2.76E-06	down	Serine/threonine protein kinase
Solyc04g082170.3	1	0	0	23	22	13	12.90636	0.25876	25.5539715	6.523572844	1.00E-06	2.77E-06	up	Alcohol dehydrogenase, class III
Solyc06g084130.3	4	6	7	29	69	15	28.12147	4.35521	51.8877213	3.564353964	1.02E-06	2.81E-06	up	N-methyl-D-aspartate receptor glutamate-binding subunit
Solyc01g021730.2	33	67	174	0	1	8	36.31056	69.217	3.40408387	-4.266081685	1.06E-06	2.90E-06	down	--
Solyc11g011580.2	108	138	91	4	19	17	52.15203	87.0698	17.2342627	-2.346437951	1.08E-06	2.95E-06	down	--
Solyc06g068200.1	14	15	13	46	56	27	34.07724	10.8148	57.3397273	2.39818278	1.09E-06	2.99E-06	up	--
Solyc09g064730.3	19	27	34	38	92	78	55.24985	20.4729	90.0267596	2.131747053	1.09E-06	2.99E-06	up	Copper chaperone
Solyc12g005450.1	51	36	38	0	1	3	16.85734	32.1326	1.58212481	-4.262020007	1.11E-06	3.03E-06	down	Serine/threonine protein kinase
Solyc03g097760.2	23	28	28	0	0	0	10.14813	20.2963	0	-6.42796526	1.20E-06	3.27E-06	down	RNA-binding protein Bicaudal-C
Solyc02g080600.1	25	27	23	35	54	134	54.92838	19.3163	90.540452	2.235695009	1.20E-06	3.27E-06	up	--
Solyc03g095980.3	36	32	77	1	0	0	18.61487	36.7924	0.43739094	-6.325997781	1.21E-06	3.29E-06	down	--
Solyc02g083930.1	19	35	29	0	0	0	10.68486	21.3697	0	-6.501966042	1.21E-06	3.29E-06	down	--
Solyc02g092580.3	5	4	37	77	141	63	68.52411	11.4706	125.577649	3.443078739	1.21E-06	3.30E-06	up	--
Solyc03g082700.4	1	1	0	30	8	35	15.15591	0.52478	29.7870367	5.860652808	1.22E-06	3.32E-06	up	Sexual differentiation process protein ISP4
novel.163	11	8	14	32	20	84	31.4035	8.42259	54.3844099	2.698082713	1.29E-06	3.49E-06	up	--
novel.1570	5	8	6	16	25	42	19.71309	4.89974	34.5264456	2.825032682	1.29E-06	3.50E-06	up	--
Solyc03g113600.4	8	7	3	32	26	21	19.51627	4.67111	34.361422	2.880097668	1.33E-06	3.59E-06	up	--
Solyc08g006740.3	6	17	19	21	66	107	45.60014	10.7545	80.4457924	2.903941696	1.35E-06	3.64E-06	up	Glutamate decarboxylase and related proteins
Solyc06g008270.2	12	8	8	23	35	32	23.01868	7.20361	38.8337578	2.42580423	1.37E-06	3.69E-06	up	FOG: Leucine rich repeat
Solyc11g069700.2	27325	15702	10390	72179	46502	46008	42439.57	13806.6	71072.5032	2.363927802	1.37E-06	3.72E-06	up	Translation elongation factor EF-1 alpha/Tu
Solyc06g073980.4	72	40	76	6	6	9	28.41355	47.9895	8.83756822	-2.436180768	1.38E-06	3.73E-06	down	--
Solyc01g007200.2	67	50	81	10	4	11	30.46274	50.5875	10.3380169	-2.27061789	1.38E-06	3.74E-06	down	--
Solyc04g049640.3	3	1	0	13	19	24	12.38191	1.0423	23.7215239	4.524192528	1.39E-06	3.75E-06	up	Serine/threonine protein kinase
Solyc11g066950.2	22	9	6	35	32	90	36.65748	9.56463	63.7503261	2.745441404	1.40E-06	3.77E-06	up	--
Solyc01g091360.4	139	68	81	13	5	18	44.34817	74.0065	14.6898818	-2.309017709	1.42E-06	3.83E-06	down	--
Solyc01g108240.3	41	35	49	1	0	0	16.21276	31.9881	0.43739094	-6.123278757	1.43E-06	3.87E-06	down	--
Solyc03g005580.2	405	382	372	47	14	108	182.3977	298.038	66.7569813	-2.145484044	1.45E-06	3.91E-06	down	--
Solyc11g056620.3	67	66	89	8	14	7	34.85469	56.8142	12.8951615	-2.176266458	1.51E-06	4.05E-06	down	--
novel.419	117	90	194	7	24	18	61.6763	101.997	21.3555742	-2.2704066	1.53E-06	4.11E-06	down	--
Solyc01g014373.1	119	85	122	20	5	24	51.6948	83.4516	19.9379692	-2.041205325	1.56E-06	4.19E-06	down	--
Solyc03g043700.3	292	139	186	18	43	20	97.26512	158.345	36.1856963	-2.146555943	1.61E-06	4.32E-06	down	FOG: Armadillo/beta-catenin-like repeats
Solyc01g108390.3	7	16	6	48	16	84	33.48617	7.54547	59.4268675	2.990677792	1.61E-06	4.32E-06	up	--
Solyc05g056430.3	10	10	3	29	18	92	30.49809	5.98671	55.0094728	3.214844579	1.70E-06	4.54E-06	up	--
Solyc08g075570.4	8	4	3	23	21	24	16.47318	3.87303	29.0733321	2.910004402	1.70E-06	4.55E-06	up	Urea transporter
Solyc06g062920.3	675	391	660	5	41	30	237.1969	441.228	33.1656334	-3.737808292	1.73E-06	4.63E-06	down	Serine/threonine protein kinase
Solyc11g012570.3	109	84	106	24	8	10	47.35515	76.6574	18.0528958	-2.092863075	1.77E-06	4.73E-06	down	FOG: PPR repeat

novel.1054	23	16	6	50	30	70	36.86552	11.6856	62.0454553	2.418222046	1.84E-06	4.91E-06	up	--
Solyc04g072760.3	10	3	22	37	34	78	35.01715	8.804	61.230305	2.793025883	1.89E-06	5.04E-06	up	Sulfate/bicarbonate/oxalate exchanger SAT-1
Solyc01g108470.3	2	0	0	10	32	26	15.006	0.51751	29.4944765	5.837216919	1.91E-06	5.08E-06	up	Serine carboxypeptidases (lysosomal cathepsin A)
Solyc02g078370.1	10	21	22	34	57	49	37.09455	13.5925	60.5966052	2.150774296	1.91E-06	5.09E-06	up	--
novel.327	249	231	224	20	8	65	108.6982	181.051	36.3448817	-2.294128513	1.92E-06	5.11E-06	down	--
Solyc10g080900.3	2019	4335	4729	7686	15758	10966	8951.465	2840.36	15062.5716	2.406795543	1.95E-06	5.18E-06	up	Reductases with broad range of substrate specificities
Solyc07g054770.1	602	841	459	5	4	45	256.5438	492.547	20.5403838	-4.569809073	1.95E-06	5.18E-06	down	--
Solyc01g106600.2	0	2	4	23	26	13	14.51349	1.51721	27.509769	4.157238223	1.97E-06	5.22E-06	up	Defense-related protein containing SCP domain
Solyc12g005480.2	1	5	3	22	23	16	14.51323	2.32776	26.6987054	3.517924536	2.00E-06	5.32E-06	up	--
Solyc06g082300.4	71	45	82	4	5	13	29.73503	50.5386	8.9314042	-2.468176106	2.01E-06	5.32E-06	down	UDP-glucuronosyl and UDP-glucosyl transferase
Solyc03g095820.4	127	118	108	11	5	32	54.88456	90.8525	18.9165852	-2.226478988	2.01E-06	5.33E-06	down	--
Solyc03g025125.1	116	66	64	11	12	8	38.46496	63.3361	13.5938274	-2.242127039	2.02E-06	5.34E-06	down	--
Solyc08g068520.3	41	40	49	1	0	1	17.06003	33.3183	0.80178276	-5.302604644	2.06E-06	5.44E-06	down	--
novel.1835	79	27	35	1	0	0	18.34102	36.2446	0.43739094	-6.302756865	2.10E-06	5.54E-06	down	--
Solyc02g084130.4	40	39	41	4	2	2	17.13971	30.8232	3.45624615	-3.175939629	2.16E-06	5.71E-06	down	--
Solyc05g053330.3	3	0	6	18	21	25	14.75239	2.254	27.2507691	3.572601609	2.18E-06	5.76E-06	up	Transcription factor, Myb superfamily
Solyc10g049600.3	83	56	58	9	8	11	31.25776	50.6591	11.8564234	-2.093800105	2.40E-06	6.31E-06	down	--
Solyc03g079900.3	7	7	2	21	28	24	17.89363	4.16607	31.6211958	2.928534392	2.44E-06	6.41E-06	up	GTPase Rab11/YPT3, small G protein superfamily
Solyc10g079860.2	0	0	7	25	60	18	24.2774	1.72402	46.8307886	4.73024274	2.63E-06	6.87E-06	up	--
Solyc11g071760.3	54	37	43	2	3	6	19.46714	34.4063	4.52798088	-2.897231224	2.63E-06	6.89E-06	down	Calmodulin and related proteins (EF-Hand superfamily)
Solyc04g045300.1	54	35	36	3	0	0	16.7312	32.1502	1.31217283	-4.649154557	2.64E-06	6.91E-06	down	--
Solyc02g081690.1	73	45	55	7	7	7	26.72075	44.4064	9.03512491	-2.309797706	2.71E-06	7.09E-06	down	UDP-glucuronosyl and UDP-glucosyl transferase
Solyc03g121420.4	5	2	4	20	70	11	24.89679	2.81099	46.982585	4.051486147	2.79E-06	7.26E-06	up	Predicted haloacid dehalogenase-like hydrolase
Solyc07g055950.3	5	5	7	32	12	32	17.93619	4.34794	31.5244407	2.861978338	2.81E-06	7.33E-06	up	--
novel.628	1	5	1	30	8	36	15.99331	1.83518	30.1514286	4.062846688	2.83E-06	7.36E-06	up	--
Solyc03g111540.2	10	1	10	30	21	48	23.09848	5.31648	40.8804722	2.93934359	2.86E-06	7.45E-06	up	--
Solyc01g087980.4	321	255	497	15	58	79	168.5051	273.303	63.706881	-2.099830326	2.96E-06	7.70E-06	down	--
Solyc08g015730.3	58	33	45	4	2	5	19.70961	34.8698	4.54942158	-2.913985853	2.98E-06	7.75E-06	down	--
Solyc08g069140.4	51	27	50	0	2	4	17.56462	32.6938	2.435466	-3.703464011	3.03E-06	7.86E-06	down	K+-channel ERG and related proteins
Solyc02g067870.3	3	6	9	24	15	42	18.86256	4.58903	33.1360794	2.856679039	3.05E-06	7.91E-06	up	--
novel.1183	10	6	7	16	28	43	21.13272	5.90775	36.3576855	2.625162181	3.10E-06	8.03E-06	up	--
Solyc06g007240.4	22	23	38	63	33	117	53.74753	21.1703	86.3248008	2.033017815	3.13E-06	8.11E-06	up	BCL2-associated athanogene-like proteins
Solyc02g090480.4	65	70	87	11	5	18	35.34166	56.8682	13.8150999	-2.00947723	3.17E-06	8.20E-06	down	HSP90 co-chaperone CPR7/Cyclophilin
Solyc06g008080.2	1	1	4	26	9	24	13.01403	1.50994	24.5181124	4.007503417	3.19E-06	8.25E-06	up	Cullins
Solyc03g005630.3	15	14	13	37	38	29	28.0692	10.8075	45.3309037	2.060679639	3.21E-06	8.31E-06	up	--
Solyc02g092783.1	6	7	7	40	14	28	19.84133	5.13875	34.5438997	2.751936169	3.24E-06	8.37E-06	up	--
Solyc11g044860.3	8	5	2	24	27	20	17.43981	3.89277	30.986852	2.993087306	3.29E-06	8.49E-06	up	Clathrin assembly protein AP180 and related proteins
Solyc12g044450.2	56	51	92	10	8	8	30.95849	50.7163	11.2006389	-2.192344932	3.43E-06	8.84E-06	down	--
Solyc04g045440.3	17	10	19	37	24	63	31.30678	11.7386	50.8749341	2.121305156	3.44E-06	8.87E-06	up	P-type ATPase
Solyc05g018820.1	14	28	34	0	0	0	9.722588	19.4452	0	-6.366792803	3.48E-06	8.95E-06	down	--
Solyc03g115790.1	53	62	75	8	11	7	30.05389	48.6795	11.4283133	-2.120661929	3.49E-06	8.99E-06	down	FOG: PPR repeat
Solyc08g042060.2	63	37	40	2	5	4	20.38667	35.9962	4.777096	-2.936895882	3.54E-06	9.10E-06	down	--
novel.611	38	36	48	3	3	4	17.7341	31.2316	4.2365882	-2.882128314	3.55E-06	9.12E-06	down	--

Solyc03g013340.3	48	44	29	1	2	4	17.07038	31.2679	2.87285694	-3.409720007	3.67E-06	9.42E-06	down	Aquaporin (major intrinsic protein family)
Solyc07g052950.3	4	0	3	21	10	33	13.93676	1.77389	26.0996334	3.875996639	3.69E-06	9.47E-06	up	--
Solyc05g051480.2	29	14	31	0	0	0	9.431642	18.8633	0	-6.323617097	3.82E-06	9.80E-06	down	--
Solyc07g039347.1	55	53	61	3	10	4	25.50696	43.3547	7.65923382	-2.55220445	3.82E-06	9.80E-06	down	--
Solyc09g007010.1	13	3	32	44	149	61	63.18486	12.0432	114.326559	3.238297906	3.87E-06	9.91E-06	up	Defense-related protein containing SCP domain
Solyc03g112090.3	13	10	5	34	21	35	22.57425	7.25556	37.8929424	2.393228194	3.88E-06	9.95E-06	up	--
Solyc01g005510.3	0	0	9	294	339	268	197.1102	2.2166	392.003781	7.456176034	4.10E-06	1.05E-05	up	Multicopper oxidases
Solyc08g077870.4	54	21	68	0	1	1	18.58021	36.3071	0.85334119	-5.421308585	4.11E-06	1.05E-05	down	--
novel.106	27	17	25	0	0	0	8.83306	17.6661	0	-6.22839726	4.16E-06	1.06E-05	down	--
Solyc08g066990.4	43	56	60	4	5	10	24.31981	40.8014	7.83822877	-2.356578067	4.31E-06	1.10E-05	down	K+-channel ERG and related proteins
Solyc03g119840.3	10	9	7	20	43	28	23.34072	6.70584	39.9756127	2.569178203	4.36E-06	1.11E-05	up	--
novel.353	83	73	85	4	8	21	37.57237	61.8314	13.3133868	-2.18381594	4.43E-06	1.13E-05	down	--
Solyc03g115600.4	2	6	3	15	19	25	13.90662	2.85254	24.9606976	3.138381597	4.60E-06	1.17E-05	up	--
Solyc06g074870.3	35	29	42	1	2	2	14.62974	27.1154	2.14407332	-3.675169437	4.69E-06	1.19E-05	down	Sister chromatid cohesion complex Cohesin
Solyc10g080360.1	323	267	134	14	9	60	109.999	187.611	32.3875263	-2.514241882	4.75E-06	1.20E-05	down	--
Solyc12g096860.3	52	52	37	1	0	7	19.6948	36.4015	2.98813363	-3.486188597	4.75E-06	1.21E-05	down	Acyl-CoA thioesterase
Solyc03g095880.1	21	46	88	90	231	132	119.8784	39.3446	200.412209	2.34492563	5.01E-06	1.27E-05	up	--
Solyc03g119230.1	31	41	40	0	0	3	14.93665	28.7801	1.09317544	-4.532678238	5.04E-06	1.27E-05	down	Serine/threonine protein kinase
Solyc08g062290.4	25	25	9	42	39	80	40.9635	15.3362	66.5907902	2.128124014	5.05E-06	1.28E-05	up	--
Solyc11g005860.2	10	5	5	17	24	35	18.53665	5.14915	31.9241444	2.636500021	5.06E-06	1.28E-05	up	Uncharacterized conserved protein
Solyc04g064630.3	55	51	53	10	4	5	24.502	40.8523	8.151666	-2.334053109	5.10E-06	1.29E-05	down	Multitransmembrane protein
Solyc11g065220.2	103	75	121	22	4	15	46.72461	76.4049	17.0442754	-2.152425218	5.12E-06	1.29E-05	down	Hexokinase
Solyc06g065050.2	40	29	42	1	3	0	15.15672	28.4092	1.90423906	-4.03174823	5.26E-06	1.33E-05	down	Uncharacterized conserved protein
Solyc02g086223.1	33	42	53	5	1	4	18.44945	32.7654	4.13347134	-2.961187686	5.29E-06	1.33E-05	down	--
Solyc11g071550.2	51	43	45	4	1	8	20.43621	35.7188	5.15364765	-2.729809298	5.33E-06	1.34E-05	down	Amidases
novel.539	24	24	18	0	0	0	8.51401	17.028	0	-6.173899197	5.34E-06	1.35E-05	down	--
Solyc09g090685.1	46	50	32	2	1	6	18.31775	33.0854	3.55008213	-3.148496603	5.40E-06	1.36E-05	down	--
Solyc06g064720.1	33	35	37	0	1	3	14.27237	26.9626	1.58212481	-4.009760055	5.50E-06	1.38E-05	down	Apoptotic ATPase
Solyc08g066250.4	198	788	960	2532	5392	3209	2705.261	497.3	4913.22222	3.304408605	5.55E-06	1.40E-05	up	Glutamate decarboxylase
Solyc12g036820.2	32	28	45	0	0	1	13.58818	26.812	0.36439181	-5.868996912	5.59E-06	1.40E-05	down	--
Solyc06g072013.1	84	81	108	12	17	4	42.45069	69.883	15.0183979	-2.258459698	5.67E-06	1.42E-05	down	--
Solyc12g006840.2	77	37	103	2	10	6	31.54283	55.135	7.9506265	-2.823337474	5.70E-06	1.43E-05	down	--
Solyc11g017320.3	42	37	29	0	1	3	14.71765	27.8532	1.58212481	-4.057260182	5.75E-06	1.44E-05	down	FOG: PPR repeat
Solyc01g100220.3	34	37	36	2	3	1	15.10658	27.5071	2.70602182	-3.413255819	5.77E-06	1.45E-05	down	GATA-4/5/6 transcription factors
novel.1720	49	34	36	4	0	0	16.16999	30.5904	1.74956378	-4.158744809	5.84E-06	1.47E-05	down	--
Solyc12g096295.1	8	4	3	30	24	16	17.27991	3.87303	30.6867823	2.982060693	5.96E-06	1.49E-05	up	--
Solyc09g090130.3	46	31	48	1	2	6	17.78658	31.9715	3.60164056	-3.09458367	6.17E-06	1.55E-05	down	Transcription factor, Myb superfamily
Solyc08g062930.3	42	31	44	0	2	0	15.46462	29.9513	0.97789875	-5.120817395	6.19E-06	1.55E-05	down	--
Solyc06g068815.1	57	59	29	6	1	1	20.53241	37.5871	3.47768685	-3.452229041	6.40E-06	1.60E-05	down	--
Solyc04g064860.1	66	41	63	9	1	7	25.23874	43.5013	6.97621056	-2.613252301	6.41E-06	1.60E-05	down	MADS box transcription factor
Solyc04g079450.4	122	40	106	5	13	9	40.06943	68.316	11.8228229	-2.550254556	6.43E-06	1.61E-05	down	Serpin
Solyc01g090140.3	36	36	35	0	0	3	14.30276	27.5123	1.09317544	-4.467096871	6.61E-06	1.65E-05	down	Iron/ascorbate family oxidoreductases
Solyc03g083770.1	20	13	33	58	63	39	43.57239	16.761	70.383766	2.058909192	6.63E-06	1.65E-05	up	--
Solyc04g064880.4	5	1	2	14	170	44	53.66525	2.05239	105.278106	5.678721907	6.81E-06	1.70E-05	up	--
Solyc01g020347.1	38	38	58	3	5	0	18.99173	34.2265	3.7569197	-3.281927534	7.47E-06	1.85E-05	down	--
Solyc02g092520.3	60	53	41	8	3	7	23.61971	39.7227	7.51671836	-2.386766586	7.53E-06	1.87E-05	down	--
Solyc03g044980.1	29	31	38	1	0	0	12.77358	25.1098	0.43739094	-5.773752714	7.67E-06	1.90E-05	down	--
Solyc01g099910.4	5	5	12	19	21	42	19.7311	5.57939	33.8828209	2.600871576	7.67E-06	1.90E-05	up	Soluble epoxide hydrolase
Solyc05g015030.3	6	15	9	16	54	49	29.50813	7.75955	51.2567201	2.724202674	7.73E-06	1.91E-05	up	--
Solyc01g065643.1	85	37	30	2	4	4	21.75708	39.226	4.28814663	-3.203227923	8.01E-06	1.98E-05	down	--

Solyc09g091550.3	14	94	45	1	2	1	20.74592	39.7122	1.7796815	-4.525787169	8.12E-06	2.00E-05	down	--
novel.546	1	1	0	25	6	26	11.93372	0.52478	23.342657	5.508196184	8.56E-06	2.11E-05	up	--
Solyc03g013320.1	49	32	37	3	1	5	16.96386	30.3046	3.62308127	-3.011801819	8.70E-06	2.14E-05	down	--
Solyc04g071000.1	40	44	37	0	5	1	16.98865	31.1682	2.80913868	-3.57165293	8.75E-06	2.15E-05	down	Serine/threonine protein kinase
Solyc01g065680.2	35	24	58	3	0	2	15.88342	29.7259	2.04095646	-3.823968269	8.84E-06	2.17E-05	down	--
Solyc03g025100.1	31	11	27	0	0	0	8.798781	17.5976	0	-6.223238921	9.04E-06	2.22E-05	down	--
Solyc04g071600.3	1	6	7	30	211	43	67.76892	3.57894	131.958894	5.201806717	9.17E-06	2.25E-05	up	--
Solyc09g011630.3	18	2	1	108	324	110	125.5884	5.43597	245.740918	5.49924983	9.20E-06	2.26E-05	up	Glutathione S-transferase
Solyc03g044725.1	45	31	28	2	2	1	14.50203	26.787	2.21707245	-3.643446742	9.20E-06	2.26E-05	down	--
Solyc08g062960.4	1024	1309	1445	145	148	45	560.6339	969.084	152.183826	-2.67336739	9.25E-06	2.27E-05	down	Heat shock transcription factor
Solyc04g005260.3	14	7	4	38	16	43	23.29142	6.46994	40.1128938	2.642319388	9.26E-06	2.27E-05	up	--
Solyc04g025530.3	42	22	43	0	1	2	14.26427	27.3108	1.217733	-4.437228813	9.53E-06	2.33E-05	down	Glutamate decarboxylase/sphingosine phosphate lyase
novel.1072	53	47	49	0	3	9	21.51596	38.2856	4.74637443	-2.954826154	9.83E-06	2.40E-05	down	--
Solyc02g078450.3	4	3	2	27	8	24	13.39612	2.32569	24.466554	3.406801519	9.88E-06	2.41E-05	up	--
Solyc01g096750.1	44	54	53	9	5	5	23.50365	38.8041	8.20322443	-2.256607976	9.99E-06	2.44E-05	down	Translation initiation factor 2C (eIF-2C)
novel.228	0	0	0	22	23	1	10.61641	0	21.2328282	7.219579056	1.03E-05	2.52E-05	up	--
Solyc10g009365.1	5	9	6	25	14	33	17.48538	5.16576	29.8049946	2.540959004	1.04E-05	2.53E-05	up	--
Solyc02g086120.2	54	51	51	6	7	10	24.89595	40.101	9.6909094	-2.0434732	1.07E-05	2.59E-05	down	--
novel.1803	0	1	0	4	23	26	11.36781	0.26603	22.4695865	6.345870334	1.08E-05	2.62E-05	up	--
Solyc06g007910.4	15	40	36	105	71	49	60.94275	23.3888	98.4966535	2.070988477	1.08E-05	2.63E-05	up	--
novel.1165	31	26	35	1	0	0	11.99784	23.5583	0.43739094	-5.681874296	1.18E-05	2.86E-05	down	--
novel.1640	22	42	15	58	40	119	54.4247	20.5601	88.2892753	2.111870563	1.19E-05	2.88E-05	up	--
novel.1664	305	741	580	1057	2150	1485	1236.79	418.895	2054.68522	2.294150459	1.19E-05	2.89E-05	up	--
novel.166	33	31	43	2	0	0	14.12551	27.3762	0.87478189	-5.006558995	1.20E-05	2.91E-05	down	--
Solyc11g064800.3	3	11	13	25	27	34	21.71503	6.90432	36.5257283	2.400268693	1.36E-05	3.26E-05	up	--
Solyc10g011730.3	53	36	23	0	1	1	14.90454	28.9557	0.85334119	-5.092569954	1.40E-05	3.36E-05	down	--
Solyc11g005100.3	11	4	10	19	25	35	19.8306	6.37332	33.2878757	2.381522117	1.40E-05	3.37E-05	up	Predicted sugar kinase
Solyc11g062230.1	8	2	0	16	23	33	16.43557	2.60211	30.2690205	3.550323108	1.43E-05	3.42E-05	up	--
Solyc01g095330.2	275	719	750	1161	2245	1582	1314.559	447.148	2181.97008	2.286651859	1.43E-05	3.43E-05	up	--
Solyc01g088320.2	34	38	56	2	3	7	18.79566	32.6989	4.89237269	-2.706274036	1.43E-05	3.44E-05	down	--
Solyc02g084290.3	36	31	46	1	3	5	16.30879	28.8914	3.72619813	-2.933269268	1.44E-05	3.46E-05	down	--
Solyc06g048700.1	41	18	38	0	1	0	12.62272	24.7565	0.48894937	-5.753854273	1.44E-05	3.46E-05	down	--
novel.1328	36	28	26	0	0	1	11.76596	23.1675	0.36439181	-5.656702774	1.45E-05	3.48E-05	down	--
Solyc02g084300.1	38	43	37	3	3	6	17.675	30.3846	4.96537183	-2.589169759	1.49E-05	3.56E-05	down	CTP synthase (UTP-ammonia lyase)
novel.774	140	47	21	270	698	622	369.9676	53.9013	686.033925	3.670114245	1.56E-05	3.73E-05	up	--
Solyc11g011030.2	101	57	27	4	5	7	27.34643	47.9478	6.74505333	-2.825249523	1.62E-05	3.87E-05	down	--
novel.1585	33	31	35	1	0	1	13.10386	25.4059	0.80178276	-4.912539353	1.63E-05	3.89E-05	down	ATP sulfurylase (sulfate adenylyltransferase)
Solyc02g069930.1	30	69	140	111	196	289	155.1464	60.599	249.693706	2.041857361	1.68E-05	3.99E-05	up	Lysophospholipase
Solyc09g005745.1	41	49	33	2	2	7	18.08766	31.7719	4.40342332	-2.796875996	1.69E-05	4.02E-05	down	--
novel.577	36	37	25	0	1	3	13.4488	25.3155	1.58212481	-3.920961567	1.72E-05	4.09E-05	down	--
Solyc02g070430.3	51	55	52	11	3	7	24.73201	40.6351	8.82889119	-2.193623437	1.72E-05	4.10E-05	down	Iron/ascorbate family oxidoreductases
novel.287	69	66	125	19	6	14	41.27186	66.1981	16.3456096	-2.011054705	1.75E-05	4.15E-05	down	--
Solyc06g083470.4	4	1	10	15	37	27	19.12726	3.76394	34.4905699	3.176451981	1.79E-05	4.24E-05	up	Reductases with broad range of substrate specificities
novel.424	52	25	34	2	0	4	15.40611	28.4799	2.33234914	-3.520935378	1.79E-05	4.25E-05	down	--
Solyc01g081640.4	31	32	38	0	0	4	13.67544	25.8933	1.45756725	-3.979737114	1.80E-05	4.28E-05	down	--
Solyc05g047530.3	51	71	125	10	14	11	39.04907	62.8706	15.2275106	-2.06250525	1.80E-05	4.28E-05	down	Cytochrome P450 CYP2 subfamily
Solyc08g066320.4	45	39	51	0	1	8	18.99197	34.5799	3.40408387	-3.245435126	1.81E-05	4.29E-05	down	--
Solyc03g033610.1	31	30	25	1	0	0	11.29845	22.1595	0.43739094	-5.592300512	1.84E-05	4.37E-05	down	--

Solyc10g081700.3	55	63	41	8	7	7	25.28085	41.0892	9.47251585	-2.127892616	1.86E-05	4.40E-05	down	--
Solyc12g096880.3	37	21	41	0	2	1	13.30035	25.2584	1.34229056	-4.299935394	1.87E-05	4.41E-05	down	--
novel.1422	39	22	41	0	0	3	13.56757	26.042	1.09317544	-4.39674355	2.01E-05	4.74E-05	down	--
Solyc02g092540.2	58	42	72	7	5	13	27.07871	43.9138	10.243577	-2.074363837	2.04E-05	4.79E-05	down	--
Solyc03g115120.1	60	43	53	10	6	5	24.57373	40.0179	9.12956474	-2.151863042	2.06E-05	4.85E-05	down	Molecular chaperone (DnaJ superfamily)
Solyc03g098580.1	40	32	29	1	2	4	14.43919	26.0055	2.87285694	-3.144434307	2.07E-05	4.87E-05	down	FOG: PPR repeat
Solyc08g063030.2	42	36	49	4	6	5	19.50907	32.5129	6.50521908	-2.343476696	2.19E-05	5.14E-05	down	Mitochondrial ADP/ATP carrier proteins
Solyc10g006120.2	52	67	65	6	13	5	29.0453	47.288	10.8026466	-2.173098848	2.20E-05	5.16E-05	down	--
Solyc07g066560.1	36	30	57	3	5	3	18.09231	31.3345	4.85009514	-2.729446695	2.21E-05	5.18E-05	down	--
Solyc07g008600.1	24	38	60	2	4	3	17.51014	31.0965	3.92375482	-3.013919211	2.21E-05	5.18E-05	down	FOG: Leucine rich repeat
Solyc04g072940.4	35	38	68	4	1	8	20.5334	35.9132	5.15364765	-2.745498884	2.29E-05	5.35E-05	down	--
Solyc03g058430.3	2	0	8	21	22	20	14.85888	2.48782	27.2299323	3.425398504	2.35E-05	5.48E-05	up	--
Solyc10g008610.1	32	35	36	4	2	3	15.1391	26.4576	3.82063796	-2.792242733	2.39E-05	5.58E-05	down	--
Solyc06g053550.3	16	8	5	26	20	50	23.43526	7.49977	39.3707426	2.403709915	2.41E-05	5.61E-05	up	Peroxisomal membrane protein MPV17
Solyc03g119370.2	1	5	5	20	21	14	13.46879	2.82034	24.1172411	3.086088163	2.48E-05	5.77E-05	up	Transcription factor, Myb superfamily
Solyc08g150125.1	35	49	37	4	5	4	18.4282	31.2045	5.65187789	-2.487297119	2.58E-05	6.00E-05	down	--
Solyc03g007380.2	23	10	25	0	0	0	7.38445	14.7689	0	-5.970755709	2.59E-05	6.01E-05	down	--
Solyc01g092950.3	6	0	0	20	12	34	14.27854	1.55254	27.004533	4.13131528	2.61E-05	6.06E-05	up	MADS box transcription factor
novel.540	51	62	64	5	6	16	28.20185	45.4528	10.95092	-2.020311433	2.75E-05	6.37E-05	down	--
Solyc05g052360.2	28	27	25	0	1	0	10.53705	20.5852	0.48894937	-5.48624008	2.82E-05	6.53E-05	down	Multicopper oxidases
Solyc03g006810.3	110	61	86	2	11	19	39.52422	65.8718	13.1766694	-2.30815357	2.88E-05	6.65E-05	down	--
Solyc02g005070.3	8	4	11	27	22	21	18.03101	5.84334	30.2186698	2.357653625	2.89E-05	6.66E-05	up	--
Solyc05g047590.4	84	48	83	6	4	19	33.22522	54.9469	11.5035876	-2.215258416	2.89E-05	6.68E-05	down	--
Solyc03g026015.1	40	51	44	7	6	4	21.10369	34.7544	7.4530001	-2.249363118	2.91E-05	6.72E-05	down	--
Solyc09g014720.3	17	23	55	0	1	0	12.27616	24.0634	0.48894937	-5.713623495	2.92E-05	6.73E-05	down	--
Solyc12g096490.1	56	38	34	0	6	2	18.31786	32.9732	3.66247986	-3.238026142	2.93E-05	6.75E-05	down	--
Solyc05g024190.3	14	7	6	44	27	19	23.1664	6.96252	39.3702791	2.495653629	2.96E-05	6.82E-05	up	--
Solyc08g077370.4	44	41	65	7	3	10	23.23685	38.3012	8.17250285	-2.198849435	2.99E-05	6.89E-05	down	--
Solyc08g007850.1	87	175	188	310	944	424	433.5152	115.369	751.66153	2.703327873	3.06E-05	7.02E-05	up	Uncharacterized membrane protein
Solyc08g076960.1	42	60	49	3	7	10	23.63816	38.8976	8.37873657	-2.205691852	3.07E-05	7.07E-05	down	--
Solyc06g062550.4	5	14	10	56	541	35	154.6251	7.48105	301.769217	5.334005568	3.14E-05	7.22E-05	up	Predicted haloacid dehalogenase-like hydrolase
novel.1188	106	63	46	14	6	5	33.19819	55.5172	10.8791285	-2.364506563	3.18E-05	7.31E-05	down	--
novel.1268	36	46	23	2	0	4	14.77475	27.2171	2.33234914	-3.455105839	3.19E-05	7.33E-05	down	--
Solyc03g013295.1	9	1	2	20	14	34	15.53492	3.08742	27.9824317	3.187242606	3.26E-05	7.47E-05	up	Transporter, ABC superfamily (Breast cancer resistance protein)
novel.752	33	35	39	3	4	4	16.09037	27.4552	4.72553758	-2.552064298	3.31E-05	7.56E-05	down	--
novel.1619	109	81	61	4	17	13	39.78757	64.7763	14.7987967	-2.14503984	3.31E-05	7.57E-05	down	--
Solyc09g092150.1	57	43	65	6	11	7	26.37531	42.1971	10.5535315	-2.029364283	3.33E-05	7.61E-05	down	FOG: PPR repeat
novel.1353	37	34	40	5	1	5	16.48417	28.4705	4.49786316	-2.62644563	3.33E-05	7.62E-05	down	--
Solyc02g084173.1	60	32	28	1	4	4	17.39257	30.9344	3.85075569	-3.01503669	3.34E-05	7.63E-05	down	--
Solyc03g007700.1	28	30	32	2	1	3	12.78208	23.1073	2.4569067	-3.195083085	3.36E-05	7.69E-05	down	--
Solyc06g062320.3	35	45	59	7	6	4	21.50588	35.5588	7.4530001	-2.282678401	3.51E-05	7.99E-05	down	Flavin-containing monooxygenase
Solyc05g046130.4	59	34	51	8	4	7	22.43899	36.8723	8.00566773	-2.198162202	3.71E-05	8.44E-05	down	--
Solyc05g012430.1	6	0	6	23	13	27	14.64259	3.03027	26.2549125	3.107202207	3.72E-05	8.44E-05	up	--
Solyc03g046390.1	63	86	72	16	4	2	33.29783	56.9128	9.68283623	-2.572994276	3.72E-05	8.45E-05	down	--
novel.595	16	9	6	28	30	26	22.20085	8.01209	36.3896148	2.180841627	3.80E-05	8.62E-05	up	--
Solyc08g067796.1	9	13	5	25	22	28	19.45662	7.01861	31.8946306	2.191239627	3.84E-05	8.71E-05	up	FOG: Leucine rich repeat
Solyc07g056510.3	2383	2299	2404	6583	7857	2941	4806.494	1820.29	7792.69613	2.097884558	3.87E-05	8.76E-05	up	Glutathione S-transferase
Solyc05g014360.1	47	47	64	10	8	5	25.2674	40.4273	10.1074635	-2.02840184	4.05E-05	9.15E-05	down	--
Solyc07g045510.4	42	40	90	7	7	8	26.53719	43.6749	9.39951672	-2.223572224	4.05E-05	9.15E-05	down	FOG: PPR repeat

Solyc06g050950.3	9	4	13	170	143	13	77.80399	6.59467	149.013314	4.494699843	4.06E-05	9.19E-05	up	--
Solyc11g012050.1	42	49	50	8	2	9	21.98706	36.2176	7.75655261	-2.190359207	4.12E-05	9.31E-05	down	Tyrosine kinase specific for activated (GTP-bound) p21cdc42Hs
Solyc11g065390.3	1	3	16	40	25	32	23.18868	4.99746	41.3799101	3.035989418	4.15E-05	9.37E-05	up	Galactosyltransferases
Solyc05g043240.1	32	30	36	1	2	5	14.18234	25.1274	3.23724875	-2.910220577	4.28E-05	9.66E-05	down	--
Solyc05g006550.4	44	36	85	7	6	8	25.4037	41.8968	8.91056735	-2.235675896	4.29E-05	9.67E-05	down	--
novel.1168	10	22	11	8	128	91	55.19682	11.1493	99.2443023	3.154507707	4.34E-05	9.78E-05	up	FOG: Transposon-encoded proteins with TYA
Solyc06g008765.1	4	7	3	23	10	26	14.02988	3.63609	24.4236726	2.763966302	4.35E-05	9.81E-05	up	--
Solyc02g069390.2	16	28	20	94	58	25	47.54913	16.5147	78.5836077	2.247176725	4.44E-05	1.00E-04	up	--
Solyc04g071083.1	8	12	35	20	61	159	55.19726	13.8825	96.5120288	2.796616232	4.45E-05	1.00E-04	up	--
Solyc09g075170.1	128	99	130	28	16	5	56.68361	91.4751	21.8920955	-2.082179901	4.46E-05	1.00E-04	down	FOG: PPR repeat
Solyc01g016720.2	28	33	26	2	2	0	12.14014	22.4276	1.85268064	-3.694468107	4.49E-05	1.01E-04	down	--
Solyc11g011510.1	15	13	6	28	19	48	23.92262	8.81744	39.0277915	2.159894384	4.52E-05	1.02E-04	up	--
Solyc10g050880.2	6	0	1	5	38	37	18.02418	1.79883	34.249528	4.249866626	4.54E-05	1.02E-04	up	Arylacetamide deacetylase
Solyc07g006670.1	3	5	9	14	21	26	15.0943	4.32301	25.8655972	2.573444411	4.55E-05	1.02E-04	up	--
Solyc03g120650.4	4	1	8	20	20	17	13.99642	3.27136	24.7214672	2.896868546	4.56E-05	1.02E-04	up	--
Solyc04g008910.3	12	11	14	29	16	50	24.10327	9.47943	38.727118	2.041094217	4.60E-05	1.03E-04	up	--
Solyc07g054170.3	0	0	6	20	21	15	12.97968	1.47773	24.4816329	4.012445475	4.62E-05	1.04E-04	up	--
Solyc09g031595.1	5	15	7	25	21	33	20.11793	7.00822	33.2276402	2.254387316	4.62E-05	1.04E-04	up	--
Solyc10g079100.1	41	44	47	2	1	10	19.44873	33.8898	5.00764938	-2.679424154	4.73E-05	1.06E-04	down	--
Solyc08g078770.1	8	4	6	10	26	34	17.04391	4.6119	29.4759148	2.675606013	4.74E-05	1.06E-04	up	--
Solyc04g055080.3	33	35	74	5	6	3	21.14456	36.0753	6.2138264	-2.574646104	4.77E-05	1.07E-04	down	Sugar transporter/spinster transmembrane protein
Solyc11g073260.2	75	64	44	3	8	14	28.79724	47.2692	10.3252532	-2.177522514	4.85E-05	1.08E-04	down	Histone 2A
Solyc05g051920.3	126	148	227	5	37	12	76.26686	127.883	24.6507833	-2.395896864	5.31E-05	1.18E-04	down	Sugar transporter/spinster transmembrane protein
Solyc05g032620.1	22	28	32	1	1	2	11.33889	21.0227	1.65512394	-3.639484088	5.34E-05	1.19E-04	down	--
Solyc10g018010.2	39	27	24	0	2	0	12.08154	23.1852	0.97789875	-4.742455413	5.44E-05	1.21E-04	down	--
Solyc07g054410.1	27	25	32	1	0	3	11.52446	21.5184	1.53056638	-3.696243102	5.49E-05	1.22E-04	down	--
Solyc04g081250.1	15	26	28	67	68	28	45.22543	17.6941	72.7567214	2.032213705	5.63E-05	1.25E-04	up	--
Solyc08g016587.1	7	9	12	27	23	21	18.93431	7.16101	30.7076191	2.090715641	5.76E-05	1.27E-04	up	--
Solyc01g106150.1	42	51	42	5	7	8	21.65202	34.7793	8.52473483	-2.032739502	5.88E-05	1.30E-04	down	Interferon-related protein PC4 like
Solyc01g150150.1	43	37	39	6	4	6	18.67066	30.5748	6.76649403	-2.172984966	5.96E-05	1.31E-04	down	ATP-dependent DNA helicase
novel.1678	27	28	26	2	1	0	11.10122	20.8387	1.36373126	-4.014168296	5.98E-05	1.32E-04	down	--
Solyc03g025860.3	10	7	12	35	16	26	20.00564	7.40523	32.6060601	2.136136336	6.10E-05	1.34E-04	up	Synaptobrevin/VAMP-like protein SEC22
Solyc01g073890.3	45	35	72	1	7	8	22.73149	38.6878	6.77517106	-2.515819316	6.15E-05	1.35E-04	down	--
Solyc11g011210.2	288	560	540	772	1866	1018	988.7447	356.493	1620.9962	2.184695559	6.17E-05	1.36E-04	up	--
Solyc08g082490.3	32	25	28	0	2	0	11.40244	21.827	0.97789875	-4.657850304	6.44E-05	1.41E-04	down	--
Solyc12g100030.2	38	21	29	0	0	3	11.82744	22.5617	1.09317544	-4.192293768	6.47E-05	1.42E-04	down	FOG: Leucine rich repeat
Solyc03g112410.2	37	25	22	2	1	0	11.50339	21.643	1.36373126	-4.066456994	6.59E-05	1.44E-04	down	Histone deacetylase complex, catalytic component RPD3
Solyc04g056260.2	27	29	29	1	3	0	11.87392	21.8436	1.90423906	-3.646795489	6.59E-05	1.45E-04	down	Mitochondrial solute carrier protein
Solyc12g017480.3	27	35	24	0	2	0	11.59311	22.2083	0.97789875	-4.680045601	6.76E-05	1.48E-04	down	FOG: Leucine rich repeat
Solyc05g015290.2	35	35	36	5	4	3	16.23489	27.2338	5.23592765	-2.405436638	6.91E-05	1.51E-04	down	--
Solyc08g005130.1	23	43	55	5	1	5	17.71716	30.9365	4.49786316	-2.75077059	6.95E-05	1.52E-04	down	--
Solyc03g006910.3	28	24	29	1	0	1	10.787	20.7722	0.80178276	-4.623518475	6.98E-05	1.53E-04	down	--
novel.1491	22	29	31	0	1	1	10.94787	21.0424	0.85334119	-4.633155665	7.01E-05	1.53E-04	down	--
Solyc08g082460.4	7	5	11	20	19	24	16.31694	5.85061	26.7832605	2.186805187	7.10E-05	1.55E-04	up	--
Solyc05g006000.3	43	41	45	2	6	9	20.10233	33.1167	7.08800444	-2.211321759	7.22E-05	1.57E-04	down	H+/oligopeptide symporter
Solyc12g044420.1	41	34	43	6	2	7	18.19868	30.2444	6.1529871	-2.267280821	7.36E-05	1.60E-04	down	--

Solyc01g059950.1	28	30	26	2	1	3	12.04321	21.6295	2.4569067	-3.099681916	7.40E-05	1.61E-04	down	--
novel.328	53	66	53	2	2	15	25.82189	44.3252	7.31855782	-2.535960706	7.55E-05	1.64E-04	down	--
Solyc03g118450.1	51	45	57	3	4	14	23.78787	39.2063	8.3694557	-2.1825296	7.57E-05	1.65E-04	down	--
Solyc08g066240.3	1853	4961	5642	9022	15552	8917	8994.181	3188.8	14799.5635	2.214439872	7.62E-05	1.66E-04	up	Glutamate decarboxylase and related proteins
Solyc12g096890.1	54	25	55	1	5	7	19.80116	34.1694	5.4328805	-2.644451593	7.68E-05	1.67E-04	down	--
Solyc11g071750.2	32	15	51	0	2	0	12.90463	24.8314	0.97789875	-4.834513786	7.75E-05	1.68E-04	down	Calmodulin and related proteins (EF-Hand superfamily)
Solyc12g019550.2	53	34	31	4	1	7	17.59163	30.394	4.78925584	-2.612863023	7.89E-05	1.71E-04	down	--
Solyc03g034410.1	24	14	37	0	0	1	9.705811	19.0472	0.36439181	-5.376447261	7.91E-05	1.72E-04	down	--
Solyc03g119390.4	13	14	12	33	14	58	26.2288	10.0437	42.4139175	2.091433811	8.23E-05	1.78E-04	up	--
Solyc12g056200.2	48	38	31	5	0	6	17.26881	30.1643	4.37330559	-2.728917196	8.27E-05	1.79E-04	down	Galactosyltransferases
Solyc05g050240.3	5	5	7	21	10	27	14.13061	4.34794	23.9132825	2.466460581	8.41E-05	1.82E-04	up	--
Solyc01g110970.3	33	26	21	1	2	1	11.20372	20.6278	1.7796815	-3.588241045	8.42E-05	1.82E-04	down	--
Solyc05g008980.2	42	14	40	2	2	1	13.3304	24.4437	2.21707245	-3.507985151	8.54E-05	1.85E-04	down	Serine/threonine protein kinase
Solyc06g066410.3	12	10	10	21	33	23	20.9649	8.22824	33.7015508	2.024236597	8.58E-05	1.85E-04	up	Profilin
Solyc07g006320.4	27	20	36	1	2	2	11.65872	21.1734	2.14407332	-3.318234387	8.66E-05	1.87E-04	down	--
Solyc12g042340.2	23	31	30	1	3	0	11.74558	21.5869	1.90423906	-3.627721255	8.71E-05	1.88E-04	down	Uncharacterized conserved protein
novel.1145	69	41	63	13	2	8	26.92833	44.2775	9.57911552	-2.195318888	9.16E-05	1.97E-04	down	--
Solyc04g011770.2	60	30	55	3	4	11	22.1642	37.0521	7.27628026	-2.31419426	9.60E-05	2.06E-04	down	--
Solyc01g095240.3	13	10	13	19	20	59	24.4072	9.22587	39.5885323	2.111935099	9.89E-05	2.12E-04	up	--
Solyc03g098720.3	16	9	4	19	35	33	22.48405	7.51951	37.4485858	2.317344807	1.02E-04	2.18E-04	up	--
Solyc02g092450.3	66	36	32	3	7	5	20.54648	34.5362	6.55677751	-2.420746889	1.06E-04	2.25E-04	down	Calcium transporting ATPase
Solyc07g006020.3	11	9	8	21	29	20	18.93173	7.21088	30.6525779	2.07855342	1.08E-04	2.31E-04	up	Predicted membrane proteins, contain hemolysin III domain
Solyc06g050610.3	29	12	30	0	1	0	9.286945	18.0849	0.48894937	-5.301191087	1.10E-04	2.34E-04	down	--
Solyc10g084600.2	40	117	49	110	509	381	244.6826	53.5436	435.821515	3.025212176	1.10E-04	2.35E-04	up	--
Solyc01g073820.4	9	28	43	0	0	1	10.36619	20.368	0.36439181	-5.47234655	1.10E-04	2.35E-04	down	--
Solyc11g005930.2	1	14	15	31	27	46	25.60012	7.67747	43.5227757	2.503769435	1.11E-04	2.35E-04	up	--
Solyc05g052230.1	12	4	7	10	26	58	22.05727	5.89321	38.2213183	2.702227489	1.11E-04	2.36E-04	up	--
Solyc04g014530.1	68	40	76	4	8	16	29.22297	46.9545	11.4914278	-2.011693539	1.12E-04	2.38E-04	down	--
Solyc06g072760.4	0	6	8	30	21	16	16.3932	3.56647	29.2199342	3.025389037	1.13E-04	2.40E-04	up	SNARE protein PEP12/VAM3/Syntaxin 7/Syntaxin 17
Solyc09g064820.1	22	37	62	3	3	6	17.88547	30.8056	4.96537183	-2.615091705	1.14E-04	2.42E-04	down	Predicted E3 ubiquitin ligase
Solyc07g052310.1	37	51	16	1	2	4	14.97744	27.082	2.87285694	-3.206981345	1.19E-04	2.51E-04	down	--
Solyc02g064710.3	38	18	40	1	4	1	13.61519	24.4728	2.75758025	-3.227838136	1.19E-04	2.53E-04	down	--
Solyc01g104430.3	59	44	53	4	3	15	24.35373	40.0252	8.68228908	-2.154198169	1.21E-04	2.55E-04	down	--
Solyc09g074590.1	28	59	32	4	0	6	17.37898	30.822	3.93591465	-2.906644002	1.23E-04	2.60E-04	down	Glutaredoxin and related proteins
Solyc12g057100.2	44	40	38	7	6	5	19.60138	31.3854	7.81739191	-2.025382576	1.24E-04	2.61E-04	down	--
Solyc03g007930.4	44	79	48	4	1	14	25.78166	44.2233	7.33999852	-2.532091177	1.25E-04	2.64E-04	down	--
novel.784	24	30	41	0	2	5	13.54434	24.2888	2.79985781	-3.063785278	1.26E-04	2.65E-04	down	--
novel.217	10	24	42	0	1	0	9.902648	19.3163	0.48894937	-5.396178006	1.26E-04	2.65E-04	down	--
Solyc06g051040.4	40	36	32	7	2	3	16.47066	27.8085	5.13281079	-2.443108232	1.26E-04	2.66E-04	down	Mitotic checkpoint serine/threonine protein kinase
novel.816	27	29	18	1	1	1	10.21258	19.1344	1.29073213	-3.905528191	1.26E-04	2.67E-04	down	--
Solyc03g083680.1	22	24	27	1	0	2	9.946639	18.7271	1.16617457	-3.901734672	1.29E-04	2.71E-04	down	--
Solyc08g077100.3	77	50	35	11	3	2	24.42635	41.8458	7.00693213	-2.595481082	1.31E-04	2.75E-04	down	--
Solyc06g063190.3	43	21	37	1	3	5	14.776	25.8258	3.72619813	-2.77356298	1.31E-04	2.75E-04	down	Glutamate-gated kainate-type ion channel receptor
Solyc06g067910.3	3	4	10	22	27	13	15.9323	4.30327	27.5613274	2.659555946	1.33E-04	2.80E-04	up	--
Solyc07g055170.1	73	59	50	15	7	4	29.17019	46.8993	11.441077	-2.056241911	1.36E-04	2.85E-04	down	DNA repair protein RHP57

Solyc05g053370.3	45	28	60	6	6	1	19.89629	33.8701	5.92243372	-2.569080723	1.36E-04	2.85E-04	down	Fe2+/Zn2+ regulated transporter
Solyc04g026300.1	44	37	66	10	3	1	21.84426	37.4834	6.20514937	-2.623034742	1.38E-04	2.90E-04	down	--
Solyc04g080250.3	14	29	13	24	104	43	45.77805	14.5391	77.0169654	2.402490433	1.40E-04	2.93E-04	up	dUTPase
novel.1947	4	16	3	21	28	33	20.46553	6.03033	34.9007221	2.54249673	1.42E-04	2.97E-04	up	--
novel.382	47	17	38	3	0	4	14.40638	26.043	2.76974008	-3.170292512	1.42E-04	2.98E-04	down	--
Solyc09g150109.1	37	25	23	2	2	3	12.41759	21.8893	2.94585607	-2.885500095	1.43E-04	3.00E-04	down	--
novel.1062	16	13	5	30	16	54	24.72599	8.82991	40.6220762	2.215839529	1.48E-04	3.09E-04	up	--
Solyc01g020190.2	26	38	32	1	2	6	14.1598	24.718	3.60164056	-2.725377927	1.50E-04	3.14E-04	down	Protein involved in mRNA turnover and stability
Solyc02g079790.3	33	27	33	2	1	6	13.69966	23.8492	3.55008213	-2.679294327	1.55E-04	3.24E-04	down	--
Solyc11g071240.2	30	34	19	0	3	0	11.47699	21.4871	1.46684812	-4.025216994	1.56E-04	3.25E-04	down	--
Solyc01g008500.4	48	30	52	9	4	5	20.46122	33.2082	7.71427505	-2.114322441	1.70E-04	3.53E-04	down	Serine/threonine protein kinase
Solyc06g062420.4	52	26	52	0	4	8	19.02501	33.1791	4.87093199	-2.738727826	1.72E-04	3.56E-04	down	--
Solyc02g067750.4	5	25	6	27	180	109	74.48068	9.4222	139.53915	3.890451609	1.72E-04	3.57E-04	up	Predicted carbonic anhydrase
Solyc01g006540.4	193	381	683	846	2250	1261	1124.589	319.512	1929.6669	2.594184136	1.79E-04	3.70E-04	up	--
Solyc11g072250.2	50	36	56	4	2	13	21.88578	36.307	7.46455608	-2.227555357	1.84E-04	3.79E-04	down	--
Solyc09g008970.1	1517	1302	1180	4252	4505	1485	2816.574	1029.52	4603.62506	2.160713017	1.90E-04	3.91E-04	up	--
Solyc02g082125.1	10	16	4	31	22	27	20.99187	7.82916	34.1545844	2.132268245	1.91E-04	3.94E-04	up	FOG: Transposon-encoded proteins with TYA
novel.1377	33	34	32	1	5	5	15.08462	25.4651	4.70409687	-2.445540964	1.93E-04	3.99E-04	down	--
Solyc01g105890.3	70	39	18	1	5	4	18.63048	32.9213	4.33970506	-2.940889181	1.98E-04	4.07E-04	down	--
Solyc08g077825.1	44	36	27	4	2	7	16.44515	27.6121	5.27820521	-2.348350682	1.98E-04	4.07E-04	down	--
Solyc12g045035.1	6	7	19	14	31	56	24.89053	8.09422	41.6868453	2.362349161	2.01E-04	4.14E-04	up	--
Solyc05g054190.4	36	38	41	8	4	5	18.3995	29.5221	7.27688411	-2.02774014	2.06E-04	4.23E-04	down	--
novel.1376	96	49	40	12	1	8	28.19017	47.7276	8.6527752	-2.445401029	2.07E-04	4.24E-04	down	--
Solyc03g117630.1	2133	1059	1184	5875	5747	1865	3592.256	1125.26	6059.25458	2.428832909	2.19E-04	4.48E-04	up	Molecular chaperones HSP70/HSC70, HSP70 superfamily
Solyc02g091050.3	4	0	9	28	11	25	14.99341	3.25163	26.7351848	3.02708991	2.21E-04	4.52E-04	up	Uncharacterized membrane protein, predicted efflux pump
Solyc10g086500.1	99	100	58	12	18	3	40.82369	66.5044	15.1429555	-2.167321737	2.22E-04	4.54E-04	down	Steroid reductase
Solyc01g020340.2	41	38	52	7	8	2	20.6136	33.5251	7.70211522	-2.171616424	2.24E-04	4.57E-04	down	FOG: PPR repeat
Solyc11g022590.1	124	43	55	493	242	126	218.4719	57.0709	379.872852	2.734310979	2.33E-04	4.75E-04	up	--
Solyc11g066255.1	48	20	42	4	5	2	16.50405	28.085	4.92309427	-2.556662541	2.40E-04	4.88E-04	down	--
Solyc01g150157.1	34	14	37	0	3	0	11.55082	21.6348	1.46684812	-4.03083389	2.41E-04	4.90E-04	down	GATA-4/5/6 transcription factors
novel.1925	58	37	38	6	3	11	21.1547	34.2099	8.09950372	-2.04287392	2.43E-04	4.93E-04	down	--
Solyc02g064970.4	52	37	43	5	2	12	20.71317	33.8888	7.53755521	-2.118024866	2.44E-04	4.95E-04	down	--
Solyc03g112130.1	94	68	51	17	1	6	32.54234	54.9737	10.1109463	-2.436778377	2.46E-04	4.99E-04	down	--
novel.959	10	1	0	21	18	28	15.52143	2.8536	28.1892693	3.310733378	2.51E-04	5.09E-04	up	--
Solyc06g035930.1	24	33	20	3	0	2	10.9779	19.9148	2.04095646	-3.244280422	2.60E-04	5.26E-04	down	--
novel.1319	58	70	33	8	4	13	25.97468	41.7573	10.1920186	-2.007140512	2.67E-04	5.40E-04	down	--
Solyc08g080180.1	31	23	29	1	3	4	12.32214	21.2825	3.36180631	-2.655245804	2.72E-04	5.49E-04	down	Molybdenum cofactor sulfurase
Solyc04g014645.1	25	17	27	1	2	0	9.528236	17.6412	1.41528969	-3.760685365	2.79E-04	5.62E-04	down	--
Solyc09g074180.3	43	45	22	6	2	0	16.05919	28.5161	3.60224441	-3.027149292	2.82E-04	5.68E-04	down	--
novel.1245	32	34	20	2	4	1	12.72295	22.2509	3.19497119	-2.867051153	2.86E-04	5.75E-04	down	--
Solyc06g062310.3	48	34	58	11	2	7	22.04497	35.75	8.33994182	-2.08715789	2.90E-04	5.83E-04	down	Flavin-containing monooxygenase
Solyc02g094230.1	27	41	40	7	2	5	16.80335	27.7451	5.86159442	-2.230788435	2.90E-04	5.83E-04	down	--
Solyc03g112780.1	13	33	44	1	0	4	12.4372	22.9794	1.89495819	-3.495237603	2.94E-04	5.90E-04	down	--
Solyc09g090790.3	19	31	30	2	1	4	11.68659	20.5519	2.82129851	-2.814675986	3.10E-04	6.19E-04	down	Transcription factor, Myb superfamily
Solyc04g076120.3	23	19	50	3	2	3	13.3518	23.3204	3.38324702	-2.784812429	3.16E-04	6.31E-04	down	Serine carboxypeptidases (lysosomal cathepsin A)
Solyc10g084100.2	32	33	63	4	8	5	20.02921	32.5753	7.48311783	-2.152294201	3.18E-04	6.34E-04	down	Predicted E3 ubiquitin ligase

Solyc02g067680.1	50	30	50	6	0	9	19.56849	33.2331	5.90387197	-2.439349358	3.19E-04	6.37E-04	down	FOG: Predicted E3 ubiquitin ligase
Solyc05g007170.3	27	3	17	45	50	34	34.24541	11.9714	56.5193828	2.232549314	3.24E-04	6.47E-04	up	Apoptotic ATPase
Solyc12g042730.1	11	3	10	5	71	39	28.61047	6.1073	51.1136409	3.058291454	3.26E-04	6.51E-04	up	--
novel.1164	11	29	7	32	27	69	32.31316	12.2851	52.3411783	2.102489283	3.31E-04	6.60E-04	up	--
Solyc02g085590.4	40	26	305	3	8	16	51.71952	92.385	11.0540368	-3.054767987	3.35E-04	6.68E-04	down	--
Solyc11g007540.2	12	5	9	21	11	47	19.17094	6.65182	31.6900681	2.263059398	3.43E-04	6.82E-04	up	Cytochrome P450 CYP2 subfamily
Solyc06g062460.3	51	22	34	1	3	7	15.939	27.423	4.45498175	-2.586922311	3.44E-04	6.85E-04	down	--
Solyc07g008630.1	34	25	61	1	4	8	17.89018	30.472	5.30832294	-2.496432057	3.47E-04	6.89E-04	down	FOG: Leucine rich repeat
Solyc12g013520.3	51	49	33	3	5	12	21.24455	34.3595	8.12962145	-2.048513153	3.54E-04	7.03E-04	down	--
Solyc09g098610.2	48	30	36	7	3	7	18.17344	29.2675	7.07932741	-2.032081715	3.59E-04	7.11E-04	down	Cytochrome P450 CYP2 subfamily
novel.647	1	8	2	2	53	34	21.02899	2.87955	39.1784203	3.771357845	3.64E-04	7.22E-04	up	--
novel.1638	32	27	19	2	0	4	11.2374	20.1424	2.33234914	-3.023680994	3.95E-04	7.79E-04	down	--
Solyc06g071300.1	36	63	56	3	11	1	23.46107	39.8671	7.05500775	-2.558357604	3.95E-04	7.80E-04	down	MADS box transcription factor
Solyc05g015020.1	8	4	1	7	22	32	14.42981	3.38045	25.4791608	2.923015583	4.08E-04	8.04E-04	up	--
Solyc03g112500.4	49	20	49	400	267	39	174.8925	30.0678	319.717141	3.40956982	4.17E-04	8.19E-04	up	--
Solyc05g008210.4	70	43	38	1	5	13	23.26518	38.9111	7.61923137	-2.319412428	4.24E-04	8.33E-04	down	--
Solyc07g052885.1	32	31	11	1	2	0	10.32577	19.2362	1.41528969	-3.873013882	4.27E-04	8.38E-04	down	--
novel.979	27	31	28	1	5	3	13.05234	22.1294	3.97531325	-2.515181283	4.30E-04	8.43E-04	down	--
novel.1012	52	59	61	3	3	19	26.93852	44.1746	9.70246538	-2.136539183	4.32E-04	8.48E-04	down	--
Solyc05g007350.3	26	23	18	0	0	3	9.186341	17.2795	1.09317544	-3.812312357	4.41E-04	8.64E-04	down	Apoptotic ATPase
Solyc08g062870.1	21	28	29	1	4	0	11.20911	20.025	2.39318844	-3.180368988	4.42E-04	8.66E-04	down	--
novel.89	31	40	35	3	6	7	17.03964	27.2827	6.79661176	-2.007057979	4.61E-04	9.00E-04	down	FOG: Reverse transcriptase
Solyc03g082520.1	8	9	10	10	47	24	21.51356	6.92719	36.0999335	2.372212401	4.66E-04	9.08E-04	up	--
novel.1867	57	40	37	2	3	13	20.79082	34.5029	7.07872357	-2.234521764	4.71E-04	9.17E-04	down	--
Solyc06g053640.1	26	14	25	0	0	2	8.669032	16.6093	0.72878362	-4.329017941	4.77E-04	9.30E-04	down	FOG: Predicted E3 ubiquitin ligase
Solyc02g090560.3	68	18	57	1	8	4	21.11449	36.4224	5.80655318	-2.682897413	4.98E-04	9.68E-04	down	Calcium transporting ATPase
Solyc02g089250.4	112	81	357	587	500	432	398.5472	138.454	658.640434	2.249365384	5.00E-04	9.71E-04	up	--
Solyc01g096880.4	45	21	50	4	5	7	18.14506	29.5451	6.74505333	-2.128762107	5.06E-04	9.82E-04	down	H+/oligopeptide symporter
Solyc09g014480.3	932	377	521	77	11	87	270.2649	469.77	70.7596335	-2.728057617	5.22E-04	1.01E-03	down	--
Solyc01g099500.1	31	44	37	3	4	10	17.87562	28.8393	6.91188845	-2.027453298	5.36E-04	1.04E-03	down	ATP-dependent DNA helicase
Solyc09g005260.4	37	16	10	24	106	489	128.4035	16.2933	240.513612	3.884978507	5.50E-04	1.06E-03	up	Ca2+/H+ antiporter VCX1 and related proteins
Solyc03g025460.3	31	19	28	3	3	2	11.73994	19.9721	3.50780458	-2.541772891	5.60E-04	1.08E-03	down	--
Solyc08g067380.1	35	19	31	3	0	5	12.44005	21.746	3.13413189	-2.723852117	5.93E-04	1.14E-03	down	--
Solyc08g068610.3	47	19	32	2	5	4	14.93722	25.0973	4.777096	-2.414299839	6.21E-04	1.19E-03	down	Glutamate decarboxylase and related proteins
Solyc12g010030.3	34	26	10	0	2	0	9.577618	18.1773	0.97789875	-4.371881481	6.44E-04	1.23E-03	down	Predicted aminopeptidase of the M17 family
Solyc05g015477.1	29	30	42	1	6	6	15.69317	25.8289	5.55743806	-2.225829507	6.50E-04	1.24E-03	down	--
Solyc02g072170.3	32	34	23	2	1	7	13.45213	22.9898	3.91447395	-2.484503396	6.71E-04	1.28E-03	down	--
Solyc03g117345.1	32	28	18	3	3	2	11.835	20.1622	3.50780458	-2.552274092	6.88E-04	1.31E-03	down	--
Solyc10g011960.3	44	34	80	2	2	15	23.72594	40.1333	7.31855782	-2.403085369	6.91E-04	1.32E-03	down	Copper chaperone
Solyc03g120160.1	35	23	28	2	4	5	13.36187	22.0712	4.65253844	-2.245025803	7.31E-04	1.39E-03	down	--
Solyc09g014710.4	32	25	42	2	5	7	15.57265	25.275	5.87027144	-2.099619339	7.79E-04	1.47E-03	down	--
Solyc03g096610.2	31	45	25	7	0	3	15.15241	26.1499	4.15491205	-2.634669107	7.86E-04	1.48E-03	down	FOG: PPR repeat
novel.94	24	10	31	2	0	0	8.690085	16.5054	0.87478189	-4.271364148	8.08E-04	1.52E-03	down	--
Solyc04g074840.3	656	1733	3137	4050	11238	4811	5211.356	1403.38	9019.33539	2.68406881	8.26E-04	1.55E-03	up	Uncharacterized membrane protein
Solyc07g009510.1	2	8	49	123	195	132	105.9789	14.7139	197.243933	3.742006925	8.32E-04	1.56E-03	up	Predicted chitinase
Solyc10g080210.2	3686	10494	15244	23328	53384	18904	25346.94	7499.89	43193.9921	2.525873987	8.90E-04	1.67E-03	up	--
Solyc12g056270.2	26	22	28	3	4	1	11.55436	19.4764	3.63236214	-2.485850261	8.92E-04	1.67E-03	down	Halotolerance protein HAL3
Solyc12g096950.2	24	29	36	4	3	6	14.09706	22.7914	5.40276277	-2.059713285	9.09E-04	1.70E-03	down	Oxidation resistance protein
Solyc06g076830.2	16	24	27	3	1	2	9.852233	17.1746	2.52990583	-2.756882629	9.17E-04	1.71E-03	down	Nucleotide-sugar transporter VRG4/SQV-7
novel.563	27	34	30	1	4	7	14.18198	23.42	4.94393112	-2.220957064	9.19E-04	1.72E-03	down	--

Solyc10g018813.1	31	23	31	4	4	4	13.46899	21.775	5.16292852	-2.090318917	9.25E-04	1.73E-03	down	--
Solyc02g067280.1	36	29	34	2	4	9	15.75698	25.4039	6.11010569	-2.024306222	9.89E-04	1.84E-03	down	FOG: Predicted E3 ubiquitin ligase
Solyc08g042025.1	19	24	43	1	2	6	12.74655	21.8914	3.60164056	-2.557848742	1.00E-03	1.87E-03	down	--
Solyc01g096360.3	38	28	29	7	2	5	15.14275	24.4239	5.86159442	-2.046813239	1.01E-03	1.88E-03	down	GTPase-activator protein
Solyc09g072810.3	14	33	44	3	2	5	13.67511	23.2382	4.11203064	-2.474856423	1.02E-03	1.89E-03	down	--
novel.1616	24	33	14	0	0	4	9.94734	18.4371	1.45756725	-3.51442385	1.03E-03	1.91E-03	down	--
novel.838	47	27	30	6	6	3	16.6921	26.733	6.65121734	-2.040722952	1.03E-03	1.92E-03	down	--
Solyc07g007755.1	2725	5419	3331	5971	6226	26559	9150.422	2967.1	15333.7423	2.369606561	1.11E-03	2.04E-03	up	--
novel.1855	39	39	19	2	1	8	14.71247	25.1461	4.27886576	-2.488812238	1.11E-03	2.05E-03	down	60S ribosomal protein L22
Solyc11g068620.2	52	25	26	1	7	3	15.73138	26.5096	4.95321199	-2.464037537	1.14E-03	2.10E-03	down	--
Solyc06g065910.3	17	6	3	20	33	20	19.45244	6.7339	32.1709845	2.252603576	1.14E-03	2.10E-03	up	Myosin assembly protein/sexual cycle protein
Solyc08g079140.1	32	29	31	0	2	8	13.7615	23.63	3.89303324	-2.536381844	1.15E-03	2.11E-03	down	--
novel.2033	37	20	46	7	4	3	16.16727	26.2238	6.11070954	-2.123820807	1.17E-03	2.14E-03	down	--
Solyc03g096545.1	2	8	65	168	179	306	145.581	18.6545	272.507511	3.866979467	1.19E-03	2.17E-03	up	--
Solyc05g053570.4	48	33	50	11	0	5	20.07346	33.5137	6.63325945	-2.321120142	1.21E-03	2.22E-03	down	Pleiotropic drug resistance proteins (PDR1-15)
Solyc02g087580.3	39	23	34	5	6	3	15.3989	24.584	6.2138264	-2.021445489	1.25E-03	2.28E-03	down	--
novel.1324	34	50	37	2	3	13	19.14525	31.2118	7.07872357	-2.090618704	1.27E-03	2.32E-03	down	--
Solyc09g018490.4	16	10	4	30	12	34	19.58199	7.78554	31.3784424	2.022054561	1.30E-03	2.36E-03	up	--
Solyc05g045700.1	25	28	29	2	6	2	12.79866	21.0601	4.53726175	-2.272848644	1.35E-03	2.45E-03	down	--
Solyc05g055200.4	36	19	33	2	4	6	13.75711	22.4973	5.01693026	-2.155287399	1.35E-03	2.46E-03	down	Chaperone HSP104
novel.1176	6	14	3	31	10	27	17.15149	6.01579	28.2871919	2.246313787	1.41E-03	2.56E-03	up	--
Solyc04g079460.1	26	21	40	1	6	3	13.31503	22.1658	4.46426262	-2.355867192	1.50E-03	2.71E-03	down	Serpin
Solyc03g059390.3	36	26	22	6	0	3	12.68392	21.6503	3.7175211	-2.517813863	1.56E-03	2.80E-03	down	--
novel.1403	20	26	44	2	3	7	13.91046	22.9285	4.89237269	-2.199840894	1.62E-03	2.91E-03	down	--
Solyc09g011590.4	25	6	11	11	113	24	39.79112	10.7743	68.8079831	2.669712168	1.64E-03	2.95E-03	up	Glutathione S-transferase
Solyc08g068660.1	53	23	39	2	8	7	18.38756	29.438	7.33711956	-2.018391534	1.74E-03	3.12E-03	down	Glutamate decarboxylase and related proteins
Solyc03g006080.4	40	23	67	2	6	11	20.39352	32.9702	7.81678806	-2.061394302	1.76E-03	3.14E-03	down	--
novel.1973	10	0	4	7	66	195	54.98076	3.57272	106.388799	4.895702552	1.91E-03	3.40E-03	up	--
Solyc04g072380.3	31	21	30	1	3	7	12.72584	20.9967	4.45498175	-2.200648378	1.93E-03	3.43E-03	down	--
Solyc08g080730.3	22	38	34	2	3	9	14.89833	24.1755	5.62115632	-2.063659132	2.04E-03	3.61E-03	down	--
Solyc08g008610.3	23	29	31	0	5	5	12.78393	21.3012	4.26670593	-2.327211909	2.06E-03	3.64E-03	down	Predicted hydrolase/acyltransferase
Solyc07g062480.2	46	13	18	24	83	457	118.7008	19.7944	217.607239	3.459476738	2.07E-03	3.65E-03	up	--
Solyc03g083998.1	0	1	12	28	25	11	15.85024	3.22149	28.4789907	3.125129292	2.08E-03	3.67E-03	up	--
novel.516	26	23	31	0	2	7	12.00495	20.4813	3.52864143	-2.47636223	2.13E-03	3.75E-03	down	--
Solyc12g005820.3	50	15	50	4	7	5	18.11843	29.2427	6.99416845	-2.085268687	2.24E-03	3.93E-03	down	--
Solyc07g040710.3	60	32	38	1	11	6	20.69972	33.3973	8.00218492	-2.089688559	2.37E-03	4.15E-03	down	--
Solyc01g111540.4	6	0	0	65	54	13	30.56166	1.55254	59.5707711	5.263037308	2.37E-03	4.15E-03	up	Beta-galactosidase
Solyc01g095650.4	32	23	32	2	1	9	13.46167	22.2801	4.64325757	-2.193377606	2.61E-03	4.54E-03	down	Histones H3 and H4
Solyc12g088500.2	36	26	26	1	1	9	13.42067	22.6355	4.20586663	-2.352294082	2.62E-03	4.55E-03	down	G2/Mitotic-specific cyclin A
Solyc12g077660.3	34	35	30	7	5	0	15.50192	25.4974	5.50648348	-2.260904842	2.67E-03	4.63E-03	down	DNA replication factor/protein phosphatase inhibitor SET/SPR-2
Solyc03g116970.3	4	6	14	26	6	38	17.11599	6.07923	28.1527497	2.215756855	2.68E-03	4.65E-03	up	Sister chromatid cohesion complex Cohesin, subunit PDS5
Solyc05g005170.4	1	5	35	98	153	54	73.77986	10.209	137.350725	3.746377446	2.79E-03	4.83E-03	up	--
Solyc07g150144.1	25	26	20	4	1	5	11.18594	18.3114	4.06047221	-2.133289592	2.99E-03	5.15E-03	down	--
Solyc08g080670.1	8	38	257	793	520	702	466.1915	75.4753	856.907745	3.504610545	3.05E-03	5.26E-03	up	--
Solyc12g008900.2	279	106	272	548	851	230	453.4894	167.383	739.596271	2.143013779	3.11E-03	5.36E-03	up	Proteins containing the FAD binding domain
Solyc09g055830.1	9	23	25	3	0	0	7.958415	14.6047	1.31217283	-3.502065527	3.11E-03	5.36E-03	down	--
Solyc03g113910.3	27	29	46	1	1	11	15.48258	26.0305	4.93465025	-2.329587127	3.21E-03	5.51E-03	down	--
novel.1159	33	27	25	6	5	1	13.65621	21.8789	5.43348434	-2.057320916	3.28E-03	5.63E-03	down	--

Solyc03g082840.3	24	18	26	5	2	2	10.6479	17.4022	3.89363709	-2.17708537	3.29E-03	5.64E-03	down	--
Solyc04g081890.1	416	392	455	278	1521	1203	813.8185	323.987	1303.65003	2.008417651	3.32E-03	5.70E-03	up	FOG: Predicted E3 ubiquitin ligase
Solyc10g082066.1	23	22	47	1	1	9	13.79272	23.3796	4.20586663	-2.405898392	3.33E-03	5.72E-03	down	--
Solyc08g068730.1	3	4	11	4	22	32	14.35827	4.54956	24.166988	2.404679268	4.20E-03	7.09E-03	up	Diamine acetyltransferase
Solyc01g007870.3	34	38	47	1	11	1	18.33128	30.4823	6.18022586	-2.362220575	4.24E-03	7.14E-03	down	--
novel.1390	26	29	22	2	2	8	12.31432	19.8608	4.76781513	-2.006006104	4.26E-03	7.17E-03	down	--
novel.789	34	31	22	0	5	7	13.72921	22.4629	4.99548955	-2.157619284	4.48E-03	7.51E-03	down	Calmodulin and related proteins (EF-Hand superfamily)
novel.873	33	30	23	0	6	6	13.65224	22.1844	5.12004711	-2.121345737	4.98E-03	8.28E-03	down	--
Solyc02g065260.4	89	214	242	198	1207	337	469.5632	139.561	799.565341	2.517971072	5.63E-03	9.29E-03	up	--
novel.2052	66	30	45	4	0	16	21.8608	36.1418	7.57983277	-2.202737491	5.63E-03	9.29E-03	down	--
Solyc10g047540.3	64	61	34	2	1	20	24.90675	41.1619	8.65156751	-2.201843733	5.66E-03	9.34E-03	down	--
Solyc04g076630.3	8	2	11	7	22	24	13.93766	5.31129	22.5640263	2.078298146	5.95E-03	9.78E-03	up	--
novel.773	266	79	43	250	465	341	280.7014	100.436	460.966803	2.198301066	6.53E-03	1.07E-02	up	--
Solyc05g025890.3	23	31	36	0	1	10	13.59876	23.0646	4.1328675	-2.407518566	6.68E-03	1.09E-02	down	--
Solyc08g082770.3	29	23	22	2	0	8	11.41542	19.0409	3.78991639	-2.246497122	6.81E-03	1.11E-02	down	Multitransmembrane protein
Solyc07g150133.1	26	16	43	2	2	9	13.35337	21.5745	5.13220694	-2.025721258	7.14E-03	1.16E-02	down	Protein kinase ATM/Tel1
Solyc01g057300.2	32	27	24	4	0	9	13.20149	21.3739	5.02909009	-2.023902132	7.66E-03	1.24E-02	down	--
Solyc10g079630.1	0	0	10	31	30	110	35.38679	2.46289	68.3106998	4.786855253	8.02E-03	1.29E-02	up	AAA+-type ATPase
Solyc01g106620.2	44	305	862	1065	1445	2101	1121.382	304.824	1937.9404	2.668377883	8.35E-03	1.34E-02	up	Defense-related protein containing SCP domain
Solyc08g067360.3	8	0	0	19	85	30	31.43647	2.07006	60.802879	4.876858577	8.64E-03	1.38E-02	up	--
Solyc06g043250.4	7	0	17	23	20	21	16.74471	5.99821	27.4912072	2.185510021	9.25E-03	1.47E-02	up	Serine/threonine protein kinase
Solyc12g006760.1	9	47	29	1	7	2	13.28164	21.9745	4.58882018	-2.303345264	1.27E-02	1.97E-02	down	--
Solyc09g092767.1	27	2	59	1	0	6	12.33663	22.0495	2.62374182	-3.008469398	1.48E-02	2.28E-02	down	--
Solyc04g150160.1	28	5	492	2	9	9	69.15209	129.749	8.55485256	-3.9244511	1.51E-02	2.33E-02	down	--
Solyc03g025810.4	4	4	100	127	120	401	143.5358	26.728	260.343691	3.28314261	1.72E-02	2.62E-02	up	--
novel.731	40	23	16	7	4	0	12.71353	20.4095	5.0175341	-2.06137927	1.92E-02	2.90E-02	down	--
Solyc07g009500.3	26	212	1734	2682	4039	3281	2416.854	490.19	4343.51857	3.147370952	1.95E-02	2.95E-02	up	Predicted chitinase
Solyc02g077060.2	33	17	27	0	1	10	11.92205	19.7112	4.1328675	-2.187092218	1.99E-02	3.01E-02	down	--
Solyc03g150103.1	26	37	16	0	8	3	12.75804	20.5113	5.00477042	-2.073596912	2.06E-02	3.10E-02	down	--
Solyc08g078900.1	8	19	76	55	223	54	89.30594	25.8425	152.76937	2.561311901	2.08E-02	3.13E-02	up	--
Solyc08g074720.2	23	8	3	41	39	3	23.45686	8.8185	38.0952297	2.107104962	2.63E-02	3.89E-02	up	--

**Index**	***yfm* -55 DPA-1**	***yfm* -55 DPA-2**	***yfm* -55 DPA-3**	**OH 88119-55 DPA-1**	**OH 88119-55 DPA-2**	**OH 88119-55 DPA-3**	**baseMean**	***yfm* -55 DPA**	**55 DPA OH 88119-55 DPA**	**log2FoldChange**	**pvalue**	**padj**	**regulated**	**Annotation**
Solyc01g005140.3	1357	1429	1637	7797	9847	8686	6146.156	1148.77	11143.5376	3.277962939	0	0	up	Cytochrome b
Solyc01g086660.2	48	37	48	6789	8634	9482	5259.941	34.4921	10485.3898	8.246273592	0	0	up	--
Solyc01g104740.3	406	348	361	10186	9764	12094	6967.901	290.833	13644.9691	5.553197591	0	0	up	Transcription factor MBF1
Solyc01g106700.3	176	240	274	3181	4560	4121	2582.566	178.947	4986.18635	4.799967454	0	0	up	MADS box transcription factor
Solyc01g107820.2	1285	1211	1222	12740	15770	16291	9941.826	970.015	18913.6373	4.286227416	0	0	up	UDP-glucuronosyl and UDP-glucosyl transferase
Solyc02g036370.3	532	580	643	7271	10237	9531	5912.475	456.185	11368.765	4.639840209	0	0	up	Zuotin and related molecular chaperones (DnaJ superfamily)
Solyc02g062390.3	1119	1217	1446	19433	20534	19743	13219.3	980.956	25457.6479	4.697498219	0	0	up	--
Solyc02g071730.4	501	480	567	4371	5811	5463	3498.581	401.933	6595.22874	4.036370507	0	0	up	MADS box transcription factor
Solyc02g076800.1	311	368	346	4047	5163	4711	3076.023	267.657	5884.38942	4.46094682	0	0	up	--
Solyc02g082450.3	136	148	126	5418	6740	6570	4011.125	107.413	7914.83785	6.210698637	0	0	up	Predicted membrane protein
Solyc02g089610.2	968	872	1088	16296	20002	20552	12386.39	759.309	24013.4726	4.982455504	0	0	up	S-adenosylmethionine decarboxylase
Solyc02g091930.3	1396	1276	1514	5635	8003	7154	4916.455	1087.09	8745.81711	3.008280833	0	0	up	Transcription factor HEX, contains HOX and HALZ domains
Solyc02g092800.3	1939	1834	2106	9504	12864	11696	7942.61	1527.91	14357.3069	3.232336494	0	0	up	Mn2+ and Fe2+ transporters of the NRAMP family
Solyc02g093520.3	3293	3300	3829	13670	19950	19129	12416.87	2706.69	22127.039	3.031339498	0	0	up	Copine
Solyc02g094030.3	775	649	762	4703	6129	5333	3700.373	568.233	6832.5129	3.588004591	0	0	up	--

Solyc02g150147.1	307	274	378	4560	6938	5879	3770.392	247.938	7292.84676	4.876558839	0	0	up	--
Solyc03g025720.3	8321	8561	8821	41164	59635	53052	35678.61	6698.34	64658.8887	3.271114578	0	0	up	Acyl-CoA synthetase
Solyc03g093140.3	240	222	328	25579	34074	27173	18464.04	203.738	36724.336	7.490568201	0	0	up	Permease of the major facilitator superfamily
Solyc03g115230.3	4592	4177	4682	33030	42004	46241	27277.62	3499.28	51055.9511	3.867087885	0	0	up	Chaperone HSP104 and related ATP-dependent Clp proteases
Solyc03g119300.4	162	153	175	1525	2204	1943	1255.924	127.364	2384.48364	4.227764231	0	0	up	Zn-finger protein
Solyc04g009440.3	197	212	249	5485	4896	5253	3439.567	170.743	6708.39159	5.295345705	0	0	up	--
Solyc04g011440.4	1471	1491	1711	10380	13462	14613	8696.497	1213.94	16179.0491	3.736509886	0	0	up	Molecular chaperones HSP70/HSC70, HSP70 superfamily
Solyc04g051800.3	4546	4318	5197	15798	20725	19682	13676.49	3649.02	23703.9578	2.699491395	0	0	up	Predicted transporter (ABC superfamily)
Solyc05g055870.3	852	823	900	5167	6945	7613	4476.442	670.119	8282.76469	3.628398294	0	0	up	--
Solyc06g036290.3	1995	1990	2335	25747	32534	38948	21253.17	1640.85	40865.4927	4.638376645	0	0	up	Molecular chaperone (HSP90 family)
Solyc06g053840.3	755	712	849	9047	10457	9275	6417.941	601.235	12234.6476	4.346577766	0	0	up	--
Solyc06g061020.3	1528	1529	1800	14279	19018	17500	11341.24	1260.86	21421.6167	4.086524631	0	0	up	Predicted E3 ubiquitin ligase
Solyc06g068970.3	363	373	397	2402	3460	3098	2030.571	294.98	3766.16271	3.676007145	0	0	up	Predicted steroid reductase
Solyc07g008840.3	1762	1776	2145	7411	9647	9422	6319.603	1474.04	11165.1695	2.920970311	0	0	up	Ypt/Rab-specific GTPase-activating protein GYP6
Solyc07g054780.1	11595	9390	13164	104	86	112	4480.551	8831.66	129.438443	-6.105018808	0	0	down	--
Solyc07g055060.3	1714	1906	2138	20683	31077	25681	17016.09	1495.97	32536.2	4.442988498	0	0	up	--
Solyc08g060920.4	1291	1287	1475	6218	7846	6937	4970.579	1053.04	8888.11865	3.077267886	0	0	up	Protein involved in vacuolar polyphosphate accumulation
Solyc08g062220.3	121	98	102	3626	3579	3376	2306.861	83.7502	4529.97155	5.759609333	0	0	up	UDP-glucuronosyl and UDP-glucosyl transferase
Solyc08g075540.5.1	2903	3208	3190	26393	39478	37079	22789.37	2425.51	43153.2225	4.153450458	0	0	up	--
Solyc08g080640.2	12469	10562	14099	64421	79252	81902	52436.5	9613.49	95259.5179	3.308639412	0	0	up	--
Solyc08g082870.3	876	709	774	6744	8067	7943	5123.953	614.646	9633.25995	3.970852394	0	0	up	Dehydrogenases with different specificities
Solyc09g064940.2	1957	1832	1785	18719	22988	23086	14418.98	1455.76	27382.2027	4.234075853	0	0	up	Predicted PhzC/PhzF-type epimerase
Solyc09g090570.2	410	389	383	4667	6988	5770	3815.453	308.587	7322.31863	4.570646983	0	0	up	--
Solyc10g005080.3	64	95	80	6556	10208	8993	5426.176	62.5594	10789.7924	7.437302057	0	0	up	Zuotin and related molecular chaperones (DnaJ superfamily)
Solyc10g051020.2	1610	1486	1340	43480	60013	56000	34141.47	1161.15	67121.8005	5.85401781	0	0	up	Cytochrome P450 CYP4/CYP19/CYP26
Solyc10g054440.3	2446	2494	2969	9377	11278	11828	7890.254	2052.17	13728.3403	2.741767327	0	0	up	--
Solyc11g008010.2	1464	1408	1788	20043	29199	28236	16852.27	1207.21	32497.3301	4.75022661	0	0	up	--
Solyc11g008540.3	508	410	474	14188	18821	17681	10866.69	362.099	21371.2732	5.883850672	0	0	up	dsRNA-specific nuclease Dicer and related ribonucleases
Solyc11g010380.2	460	431	495	16119	18049	17491	11156.89	360.234	21953.5495	5.92958271	0	0	up	Uncharacterized membrane protein, predicted efflux pump
Solyc11g011170.2	1568	1747	2122	10919	15841	15232	9513.826	1409	17618.6476	3.644233203	0	0	up	--
Solyc11g065000.2	1559	1439	1809	6858	9147	8954	5878.389	1246.07	10510.7106	3.076093569	0	0	up	--
Solyc11g069800.1	702	765	863	4521	5174	5528	3526.215	605.321	6447.10927	3.412801588	0	0	up	--
Solyc11g073120.2	1355	1453	1414	13508	18315	16759	10786.87	1101.9	20471.8519	4.216470653	0	0	up	Transcription factor, Myb superfamily
novel.774	168	187	190	4730	5389	5441	3371.121	142.027	6600.21525	5.540587053	0	0	up	--
Solyc03g096670.3	542	589	790	4370	5868	5801	3623.491	496.327	6750.65617	3.764400964	6.05E-304	2.18E-301	up	Serine/threonine protein phosphatase
Solyc03g122300.3	4734	5180	6008	21520	25996	26642	17742.07	4132.75	31351.3912	2.923307869	1.65E-302	5.82E-300	up	--
Solyc07g006140.4	410	276	346	3824	5140	5513	3175.107	268.202	6082.01175	4.503447544	4.62E-301	1.60E-298	up	Cytochrome P450 CYP4/CYP19/CYP26
Solyc09g092490.3	688	641	858	3398	4651	4294	2881.773	565.762	5197.78465	3.198523909	7.38E-299	2.51E-296	up	UDP-glucuronosyl and UDP-glucosyl transferase
novel.773	150	169	180	1516	1800	1810	1149.893	129.843	2169.94231	4.063851214	3.03E-298	1.01E-295	up	--
Solyc07g006370.1	733	735	889	4417	6681	5601	3811.541	611.345	7011.73758	3.519639718	2.09E-297	6.84E-295	up	K+-dependent Na+:Ca2+ antiporter
Solyc11g012850.2	181	177	198	2118	3594	3076	1906.663	144.583	3668.74186	4.666661396	9.84E-296	3.16E-293	up	--
Solyc02g089400.4	320	355	407	2032	2712	2612	1689.834	280.924	3098.74391	3.463566132	8.13E-295	2.57E-292	up	Amino acid transporter protein
Solyc02g078210.3	290	261	311	1931	2490	2189	1509.308	223.855	2794.76055	3.641791472	8.38E-295	2.60E-292	up	Ubiquitin-conjugating enzyme
Solyc08g081010.3	5817	5957	6651	16748	21399	20857	14846.54	4790.13	24902.9387	2.37825924	2.46E-294	7.52E-292	up	--
Solyc06g075530.1	62	58	57	1889	2444	1881	1340.182	46.2158	2634.14764	5.837332702	1.35E-293	4.04E-291	up	--
Solyc11g008720.3	755	731	719	4651	5765	5133	3580.667	575.616	6585.71737	3.517324474	3.28E-290	9.69E-288	up	Beta-glucosidase, lactase phlorizinhydrolase
Solyc03g118420.4	1379	1429	1689	7082	10883	9885	6416.884	1166.98	11666.787	3.321669349	6.63E-288	1.93E-285	up	Transcription factor NF-X1
Solyc01g005560.3	1752	1798	2072	6894	8686	9569	6026.174	1460.16	10592.1832	2.858908254	3.02E-282	8.62E-280	up	NADP-dependent isocitrate dehydrogenase

Solyc03g098320.4	181	174	165	1999	3147	3219	1813.962	135.894	3492.0307	4.687515125	9.51E-280	2.68E-277	up	Zuotin and related molecular chaperones (DnaJ superfamily)
Solyc06g035960.3	540	467	551	4382	4370	4903	3111.179	404.837	5817.5201	3.844692225	1.15E-279	3.19E-277	up	Acyl-CoA synthetase
Solyc08g014130.3	7840	9283	11078	55834	80292	75494	48088.93	7309.52	88868.3444	3.603800412	3.07E-279	8.38E-277	up	Alpha-isopropylmalate synthase/homocitrate synthase
Solyc01g086870.3	791	629	823	3391	4618	4412	2904.764	581.4	5228.12762	3.168257585	2.90E-277	7.80E-275	up	--
Solyc11g007590.3	2084	2580	2804	17436	20616	19415	13154.85	1940.93	24368.7713	3.65023062	3.20E-276	8.46E-274	up	OTU (ovarian tumor)-like cysteine protease
Solyc04g005020.3	958	879	1042	3969	5304	5808	3541.13	747.669	6334.59099	3.08306233	4.14E-276	1.08E-273	up	FOG: WD40 repeat
Solyc10g078590.2	1952	1829	2048	6865	9585	8390	5988.977	1516.17	10461.7887	2.786955335	4.30E-276	1.11E-273	up	--
Solyc02g089720.3	1378	1593	1703	15042	27700	23914	14468.66	1215.88	27721.4366	4.511158032	1.14E-274	2.89E-272	up	Predicted endo-1,3-beta-glucanase
Solyc06g082080.3	345	368	393	1862	2539	2536	1602.37	287.857	2916.88267	3.342604996	1.02E-273	2.56E-271	up	Serine/threonine protein phosphatase
Solyc02g087740.3	3699	3291	4049	69	48	60	1470.445	2864.2	76.687225	-5.250029194	1.21E-273	2.98E-271	down	Uncharacterized conserved protein
Solyc10g076370.3	39	31	19	1549	1911	2196	1202.904	23.5285	2382.27846	6.681981328	3.24E-271	7.88E-269	up	--
Solyc03g120570.3	6870	7305	9087	32657	47810	43713	29079.73	6025.73	52133.7304	3.112966481	1.82E-268	4.36E-266	up	H+/oligopeptide symporter
Solyc11g013810.3	2172	1912	1962	12957	20812	19708	11957.73	1577.25	22338.2007	3.82447875	8.15E-268	1.93E-265	up	Sulfite oxidase, molybdopterin-binding component
novel.1111	469	567	709	4216	5193	5145	3301.592	451.545	6151.6383	3.767054511	1.01E-267	2.36E-265	up	--
Solyc07g049530.3	27999	33053	32305	148267	192629	183612	122822.8	24350	221295.62	3.184016441	1.63E-265	3.76E-263	up	Iron/ascorbate family oxidoreductases
Solyc11g066100.2	3033	2856	3334	7985	10710	10850	7412.559	2395.87	12429.245	2.375303932	3.92E-265	8.94E-263	up	Molecular chaperones HSP70/HSC70, HSP70 superfamily
Solyc03g119530.3	6722	4753	6255	78	96	130	2363.502	4599.45	127.553078	-5.168092701	2.34E-262	5.28E-260	down	--
Solyc07g009473.1	2087	1974	1894	15171	15632	17130	10977.04	1555.86	20398.2197	3.713050849	2.58E-262	5.74E-260	up	--
Solyc10g084170.1	66	81	82	1616	1605	1892	1117.83	59.6528	2176.00623	5.190781679	4.50E-260	9.89E-258	up	--
Solyc02g087975.1	197	147	172	1442	1977	1806	1167.439	134.257	2200.62181	4.036195795	3.28E-258	7.12E-256	up	--
Solyc12g056600.3	151	169	236	1602	2086	2140	1299.034	143.431	2454.63649	4.093304108	7.35E-257	1.57E-254	up	Reductases with broad range of substrate
Solyc02g083420.3	1160	1079	1409	4445	5743	6195	3919.738	944.458	6895.01829	2.867492166	4.28E-256	9.05E-254	up	Protein phosphatase 2C/pyruvate dehydrogenase phosphatase
Solyc03g117860.3	214	141	140	4295	4134	4780	2881.589	129.475	5633.70349	5.445799021	6.22E-255	1.30E-252	up	Predicted E3 ubiquitin ligase
Solyc05g051425.2	24	17	21	1389	2002	1789	1096.725	16.1131	2177.3376	7.079514445	4.67E-254	9.67E-252	up	Phospholipase/carboxyhydrolase
Solyc08g006330.3	603	547	687	2216	2750	2771	1872.016	476.262	3267.76942	2.777620499	1.05E-253	2.15E-251	up	UDP-glucuronosyl and UDP-glucosyl transferase
Solyc01g100200.3	601	778	920	6990	11110	10530	6280.241	595.728	11964.7555	4.327852234	5.86E-253	1.19E-250	up	--
Solyc06g051800.3	53150	56381	71290	3928	4927	5336	26398.2	46815.8	5980.5888	-2.968563453	1.05E-251	2.10E-249	down	--
novel.274	1405	1339	1200	8829	10654	10277	6815.999	1032.38	12599.6149	3.610237815	2.00E-251	3.95E-249	up	--
Solyc07g054760.1	4722	3571	4785	100	89	109	1758.167	3388.9	127.435815	-4.7433759	4.39E-249	8.59E-247	down	--
Solyc11g045225.1	725	748	723	3625	4285	4739	2958.594	573.362	5343.82565	3.221724006	1.92E-247	3.73E-245	up	FOG: Ankyrin repeat
Solyc07g043310.3	3153	2904	3607	8419	10137	10605	7415.226	2506.07	12324.3844	2.297759929	6.24E-246	1.20E-243	up	Alanine-glyoxylate aminotransferase AGT2
Solyc10g044520.3	5413	4700	5281	16570	23493	21736	14992.1	4005.72	25978.4705	2.697352752	2.82E-245	5.34E-243	up	--
Solyc09g082240.4	83	70	76	1232	1993	2070	1133.366	59.6599	2207.07196	5.212617959	3.52E-244	6.61E-242	up	--
Solyc03g115920.3	1010	673	699	10065	13154	13221	7987.151	622.282	15352.0199	4.625392037	4.02E-242	7.46E-240	up	Predicted E3 ubiquitin ligase
Solyc11g017470.2	6573	6266	7327	13969	18393	18206	13270.65	5237.84	21303.452	2.024114046	3.56E-240	6.54E-238	up	--
Solyc03g118840.3	453	497	509	2285	2941	2652	1854.768	380.154	3329.38235	3.131776886	3.89E-238	7.07E-236	up	FOG: Predicted E3 ubiquitin ligase
Solyc02g092150.3	1051	1232	1502	7396	11408	10038	6534.102	980.436	12087.7681	3.623824074	5.10E-237	9.18E-235	up	--
Solyc12g056590.2	707	676	650	3061	4012	4095	2616.928	531.097	4702.75963	3.148271391	7.85E-234	1.40E-231	up	--
Solyc07g150145.1	1229	1268	1513	4245	6044	5692	3877.212	1040.33	6714.09891	2.690248148	9.60E-233	1.69E-230	up	--
Solyc05g046310.3	962	911	1028	2895	3897	4001	2646.356	754.343	4538.36895	2.589616315	4.81E-229	8.41E-227	up	--
Solyc02g089350.3	243	228	240	2090	3051	3670	1931.729	185.274	3678.18469	4.31314957	1.19E-228	2.07E-226	up	--
Solyc12g088670.2	610	613	810	3107	4311	4625	2788.807	525.828	5051.78607	3.26343391	2.89E-228	4.95E-226	up	Cysteine proteinase Cathepsin L
Solyc02g069100.4	6608	6392	7200	14106	18728	17876	13311.8	5252.12	21371.4692	2.024841697	4.80E-227	8.16E-225	up	Cysteine proteinase Cathepsin L
Solyc08g061270.1	1986	1883	2388	11247	11923	11177	8135.716	1621.17	14650.265	3.175397496	5.96E-224	1.00E-221	up	--
Solyc03g007890.3	3706	2853	3091	19271	23816	27289	16078.62	2515.72	29641.5137	3.558758864	1.16E-223	1.94E-221	up	Molecular chaperone (HSP90 family)
Solyc08g005610.3	2992	2523	2764	47	74	58	1115.715	2156.32	75.1114371	-4.837505273	2.26E-223	3.73E-221	down	Cytochrome P450 CYP4/CYP19/CYP26
Solyc07g008280.4	58	55	64	818	1201	1201	697.5701	45.9818	1349.15837	4.876341422	3.12E-223	5.11E-221	up	--
Solyc03g115720.3	2013	2109	2548	8450	11493	9622	7104.49	1729.56	12479.4208	2.850913482	3.23E-223	5.24E-221	up	Predicted E3 ubiquitin ligase
Solyc06g048520.3	719	765	761	2735	3699	3584	2401.198	585.563	4216.83234	2.849811452	5.33E-223	8.56E-221	up	Uncharacterized conserved protein
Solyc04g077650.3	5804	5892	6277	18129	22650	20340	15275.46	4679.54	25871.3804	2.467001421	7.31E-223	1.16E-220	up	Serine carboxypeptidases (lysosomal cathepsin A)
Solyc05g054830.3	1326	1205	1390	3373	4431	4381	3076.614	1019.18	5134.04669	2.333069047	5.12E-222	8.09E-220	up	FOG: Ankyrin repeat

Solyc05g015060.4	12	14	13	1641	2440	2590	1400.084	10.1885	2789.97883	8.10553688	9.87E-220	1.54E-217	up	AAA+-type ATPase
Solyc06g059860.3	84	61	59	918	1196	1248	734.398	53.3652	1415.43074	4.735929106	3.21E-216	4.98E-214	up	--
Solyc08g066870.4	1214	1208	1399	3376	4121	4310	2989.54	992.456	4986.62424	2.328952319	3.29E-215	5.05E-213	up	Casein kinase (serine/threonine/tyrosine protein kinase)
Solyc06g081980.1	6941	7035	8675	20116	23818	24703	17454.88	5871.08	29038.6743	2.306144993	5.31E-215	8.10E-213	up	Stationary phase-induced protein, SOR/SNZ family
Solyc05g007140.3	354	243	309	2065	2486	2689	1646.41	235.323	3057.4962	3.699528008	6.95E-215	1.05E-212	up	Serine/threonine protein kinase
Solyc03g114360.4	518	457	410	3536	5143	5575	3163.719	362.661	5964.77662	4.041912448	8.37E-215	1.26E-212	up	--
Solyc02g068280.4	257	218	322	1515	2041	1797	1231.968	205.701	2258.23497	3.453382632	1.05E-213	1.57E-211	up	--
Solyc03g113420.4	239	173	272	1677	2546	2178	1431.317	176.455	2686.17865	3.925419599	1.43E-213	2.11E-211	up	H+/oligopeptide symporter
Solyc03g097050.3	3852	3273	4634	165	163	187	1629.163	3038.93	219.392106	-3.798287399	5.27E-213	7.71E-211	down	--
Solyc01g010430.4	946	1086	1078	4649	6972	6976	4297.565	810.905	7784.22421	3.263899689	2.31E-212	3.36E-210	up	--
Solyc07g053140.3	221	284	382	2472	3101	3139	1953.037	228.874	3677.19883	4.003762269	1.62E-211	2.33E-209	up	--
Solyc06g036110.1	2791	3038	3730	12894	15775	18425	11156.48	2476.77	19836.1915	3.001496023	2.09E-210	2.99E-208	up	--
Solyc03g026370.1	54	62	107	1075	1515	1436	874.579	57.1076	1692.0503	4.8783061	1.17E-209	1.66E-207	up	--
Solyc08g074270.4	496	499	543	2018	2818	2409	1726.507	400.207	3052.80654	2.932235805	4.44E-209	6.24E-207	up	Deoxyribodipyrimidine photolyase/cryptochrome
Solyc02g090360.3	4194	4547	5865	126	98	144	1968.138	3778.58	157.692549	-4.592024584	4.97E-208	6.93E-206	down	Multicopper oxidases
Solyc05g009720.3	423	307	439	2027	2457	2437	1615.044	302.44	2927.64705	3.273210661	7.63E-208	1.06E-205	up	--
Solyc03g115930.3	116	148	206	1740	1911	2076	1275.37	121.142	2429.59913	4.321682664	3.46E-207	4.75E-205	up	--
Solyc06g069760.3	44	39	43	787	1255	1140	681.7451	32.8006	1330.68972	5.345994546	1.23E-206	1.68E-204	up	--
Solyc03g005010.4	834	837	943	6331	7233	5701	4447.912	679.488	8216.33536	3.595849302	4.61E-206	6.24E-204	up	--
Solyc02g071510.3	1533	1409	1635	3966	4700	4751	3434.473	1189.39	5679.55585	2.255396265	1.50E-205	2.01E-203	up	Transcription initiation factor TFIID
Solyc08g075210.2	1574	1668	2030	7448	12158	10453	6968.344	1366.63	12570.0607	3.201316798	3.28E-205	4.38E-203	up	--
Solyc06g008130.4	1527	1048	1148	10920	12216	13433	8232.827	971.038	15494.6166	3.996397056	3.58E-205	4.74E-203	up	Histone H3 (Lys9) methyltransferase
Solyc06g069570.3	1204	1229	1381	4760	6120	7234	4300.345	991.39	7609.29949	2.940582847	5.78E-205	7.60E-203	up	SNAP-25 (synaptosome-associated protein)
Solyc07g064620.2	11002	11778	13725	39856	44847	44587	32186.33	9475.27	54897.3896	2.534444388	1.84E-203	2.40E-201	up	Translation initiation factor 1 (eIF-1/SUI1)
Solyc09g074560.3	871	756	967	3650	4006	3911	2796.16	672.373	4919.94656	2.870300267	5.97E-203	7.74E-201	up	--
Solyc03g119140.4	645	653	687	2397	3582	3212	2186.678	517.028	3856.32831	2.900295041	6.99E-203	8.99E-201	up	Tyrosine kinase specific for activated (GTP-bound) p21cdc42Hs
Solyc07g042190.3	4500	3259	3856	86	84	112	1570.759	3021.79	119.725763	-4.661816823	2.04E-202	2.61E-200	down	--
Solyc07g049150.2	569	482	560	3930	7282	7170	4021.912	418.862	7624.96209	4.186717016	3.09E-202	3.92E-200	up	dsRNA-specific nuclease Dicer and related ribonucleases
Solyc05g051240.1	4476	3778	6286	86	73	64	1917.532	3738.61	96.4503774	-5.293997098	5.78E-202	7.27E-200	down	Aspartyl protease
Solyc11g006300.2	93	169	153	1993	2967	2589	1638.99	108.301	3169.67843	4.873871615	7.45E-202	9.31E-200	up	Steroid reductase
Solyc01g057770.3	2157	1892	2326	39	31	35	849.7844	1654.29	45.2831283	-5.216723943	7.60E-202	9.43E-200	down	Na+-independent Cl/HCO3 exchanger AE1
Solyc05g052050.1	874	933	1130	3444	4474	4932	3082.714	761.418	5404.00967	2.827138263	4.49E-201	5.53E-199	up	--
Solyc01g080460.3	5475	5940	6383	27627	48379	41443	26796.06	4630.91	48961.2023	3.402377324	7.13E-201	8.73E-199	up	--
Solyc08g081550.4	1296	1161	1801	6967	8741	9669	5893.059	1096.71	10689.4072	3.284034639	2.21E-199	2.68E-197	up	1-aminocyclopropane-1-carboxylate synthase
Solyc12g056650.2	3864	3978	4262	294	316	330	1775.133	3150.65	399.614591	-2.982956941	3.23E-199	3.90E-197	down	--
Solyc04g007270.3	1441	1224	1452	4166	5947	5983	3910.973	1069.74	6752.20085	2.658490648	4.48E-199	5.38E-197	up	PHD Zn-finger proteins
Solyc09g083090.4	398	306	318	2425	2699	2763	1807.597	266.742	3348.45109	3.651243679	4.73E-199	5.64E-197	up	Serine/threonine protein kinase
Solyc11g006270.2	930	995	1168	3436	5114	4718	3183.735	802.64	5564.82907	2.793750308	9.04E-199	1.07E-196	up	Steroid reductase
Solyc09g013150.4	1818	2458	2284	16956	25509	19490	13889.94	1712.57	26067.3056	3.928307774	2.49E-198	2.93E-196	up	Permease of the major facilitator superfamily
Solyc03g113380.4	1463	1569	1923	6158	9563	8946	5803.172	1284.06	10322.2842	3.006978975	2.88E-198	3.36E-196	up	FOG: PPR repeat
Solyc04g007210.3	415	324	334	2033	2861	2662	1728.591	280.089	3177.09328	3.505742217	2.62E-196	3.04E-194	up	GATA-4/5/6 transcription factors
Solyc05g013530.4	405	381	567	2588	3537	2919	2083.238	348.801	3817.67379	3.450000025	3.47E-196	4.01E-194	up	--
Solyc02g083400.3	446	447	502	1508	1881	1879	1293.748	362.658	2224.8377	2.617388444	5.96E-196	6.83E-194	up	FOG: Predicted E3 ubiquitin ligase
Solyc05g014300.1	54	61	59	797	1082	1261	680.7493	45.4083	1316.09027	4.862523252	2.04E-195	2.33E-193	up	--
Solyc01g107850.4	163	230	249	1635	2055	1897	1264.78	166.756	2362.80446	3.824710371	6.89E-195	7.81E-193	up	UDP-glucuronosyl and UDP-glucosyl transferase
Solyc07g007560.3	416	519	551	2425	3251	2864	1995.016	386.482	3603.55124	3.221464158	1.49E-193	1.67E-191	up	Permease of the major facilitator superfamily
Solyc12g042830.3	890	750	735	7519	6883	8394	5177.679	620.54	9734.8181	3.9721312	3.73E-193	4.18E-191	up	Molecular chaperone (small heat-shock protein Hsp26/Hsp42)
Solyc01g105370.4	202	225	307	1557	1940	2230	1300.252	189.501	2411.00159	3.666976399	2.83E-191	3.14E-189	up	--
Solyc09g082230.1	161	191	221	1319	1448	1464	973.3853	148.664	1798.10693	3.594967635	6.75E-190	7.45E-188	up	--
Solyc07g019460.3	3629	4392	5025	19287	29990	26062	17478	3385.56	31570.4347	3.221141661	2.35E-189	2.56E-187	up	NADP/FAD dependent oxidoreductase
Solyc02g092790.3	7136	7321	9742	335	300	286	3326.455	6256.59	396.32052	-3.987166518	2.91E-189	3.16E-187	down	--

Solyc07g063640.1	4295	3812	4604	239	218	282	1807.536	3299.94	315.134524	-3.393885684	1.93E-188	2.08E-186	down	--
Solyc01g010870.3	3009	2696	3178	7758	11604	10292	7375.985	2307.67	12444.2949	2.431178628	1.62E-187	1.74E-185	up	--
Solyc10g086420.1	920	1014	1296	4161	4932	4960	3392.592	835.752	5949.43269	2.830791188	5.49E-187	5.85E-185	up	--
Solyc04g014810.3	1245	1056	1273	3393	4004	4249	2926.774	928.218	4925.32884	2.407484221	2.35E-186	2.48E-184	up	FOG: Armadillo/beta-catenin-like repeats
Solyc04g076220.3	1178	844	1096	4780	5552	5944	3848.312	809.085	6887.53904	3.089346306	8.57E-186	9.03E-184	up	--
Solyc04g009900.4	144	190	201	3586	3198	2845	2145.342	139.117	4151.56713	4.899234084	7.49E-184	7.84E-182	up	Ca2+/calmodulin-dependent protein kinase
Solyc01g106690.3	1471	1854	2053	8177	12393	11891	7494.531	1396.77	13592.2974	3.28278238	1.21E-183	1.26E-181	up	--
Solyc00g500063.1	107	96	109	840	1048	915	634.225	81.1433	1187.30663	3.871454789	1.38E-183	1.43E-181	up	F0F1-type ATP synthase, beta subunit
Solyc07g006030.4	5478	4967	5334	14967	21095	22258	14282.49	4110.19	24454.7792	2.573065875	1.95E-183	2.01E-181	up	Uncharacterized protein CLU1/cluA/TIF31
Solyc01g086650.3	319	303	330	1206	1729	1575	1071.632	247.806	1895.45823	2.937104222	9.04E-182	9.24E-180	up	Uroporphyrin III methyltransferase
Solyc03g063600.4	11984	11667	13797	24721	34770	34306	24556.66	9721.83	39391.4969	2.01864297	2.52E-180	2.56E-178	up	Guanylate kinase
Solyc07g007140.3	1098	1099	1259	2976	4192	4268	2848.542	897.916	4799.16769	2.418718669	3.15E-180	3.19E-178	up	Tyrosine kinase specific for activated (GTP-bound) p21cdc42Hs
Solyc09g008780.4	6424	6314	7300	25847	32895	25778	20525.64	5205.31	35845.9765	2.783721401	4.47E-180	4.50E-178	up	--
Solyc08g079090.4	1683	1729	1936	36	34	51	720.7578	1390.22	51.2928911	-4.765303522	6.48E-180	6.48E-178	down	Multicopper oxidases
Solyc04g051500.3	376	409	469	1403	1852	1845	1236.797	325.62	2147.97473	2.722021951	1.09E-179	1.09E-177	up	Glucose-6-phosphate/phosphate
Solyc04g007880.4	6639	6930	8393	745	913	886	3385.307	5694.54	1076.07017	-2.404607338	1.33E-179	1.32E-177	down	Serine/threonine protein kinase
Solyc06g073920.3	2108	1969	2314	4669	6320	5676	4342.862	1659.95	7025.76828	2.081601235	5.30E-179	5.21E-177	up	--
Solyc05g054860.1	2018	2070	2356	121	131	139	920.2	1674.3	166.095188	-3.338976013	7.97E-179	7.80E-177	down	Serine/threonine protein kinase
Solyc01g107825.1	3026	3388	3898	9429	11501	11479	8189.946	2676.89	13703.0042	2.355785441	6.01E-177	5.85E-175	up	UDP-glucuronosyl and UDP-glucosyl transferase
Solyc01g086670.3	7	8	11	2114	2079	2281	1384.247	6.70959	2761.78452	8.675950009	7.43E-177	7.20E-175	up	--
Solyc03g097440.3	333	241	236	1982	2503	2580	1595.819	211.827	2979.81155	3.816647395	1.17E-176	1.12E-174	up	Predicted dehydrogenase
Solyc10g006970.3	1008	998	1133	2581	3139	3055	2264.358	815.84	3712.8758	2.1860632	1.52E-176	1.45E-174	up	Thioredoxin-like protein
Solyc08g062437.1	968	657	727	7699	7489	8623	5382.132	613.332	10150.9316	4.049089933	4.88E-176	4.65E-174	up	Molecular chaperone (small heat-shock protein Hsp26/Hsp42)
Solyc06g035940.3	7	7	3	3368	4311	3985	2466.335	4.52678	4928.14291	10.12331292	1.56E-174	1.47E-172	up	--
Solyc11g071830.2	807	759	864	3132	3245	3705	2458.253	631.732	4284.77409	2.761573796	1.78E-174	1.68E-172	up	Molecular chaperone (DnaJ superfamily)
Solyc06g083310.3	5068	5975	6338	403	528	474	2557.671	4522.04	593.302612	-2.930300991	5.69E-173	5.33E-171	down	--
Solyc02g021220.1	867	944	1114	2819	3968	3646	2573.361	758.83	4387.89144	2.531742122	7.33E-173	6.84E-171	up	--
Solyc07g006220.2	3366	4202	5432	65	119	129	1744.601	3359.53	129.670184	-4.684402808	7.52E-172	6.98E-170	down	UDP-glucose 4-epimerase/UDP-sulfoquinovose synthase
Solyc09g063010.4	515	538	648	2131	3044	3367	2008.535	441.128	3575.94206	3.019277246	1.68E-171	1.55E-169	up	--
Solyc12g014530.3	285	242	314	1034	1368	1319	893.0286	217.932	1568.12481	2.846081148	3.19E-171	2.93E-169	up	Succinate dehydrogenase, flavoprotein subunit
Solyc10g086180.2	354	455	435	3883	8202	6258	3961.996	324.552	7599.44043	4.550444072	4.03E-171	3.68E-169	up	Phenylalanine and histidine ammonia-lyase
Solyc02g087960.3	35	34	54	589	754	745	456.2668	31.6331	880.900558	4.789172848	2.85E-169	2.59E-167	up	Transcription factor, Myb superfamily
Solyc01g007100.3	144	113	136	769	1096	996	652.4764	102.132	1202.82096	3.559029005	3.29E-169	2.97E-167	up	--
Solyc05g007880.4	41	47	28	835	1524	1363	789.1218	30.6721	1547.57159	5.673438119	5.11E-169	4.60E-167	up	--
Solyc01g102610.3	529	687	708	2704	3582	3438	2299.425	500.759	4098.09049	3.033421986	1.64E-168	1.47E-166	up	Ferric reductase, NADH/NADPH oxidase
Solyc09g059170.3	133	145	163	873	1007	1160	699.602	114.582	1284.62224	3.487027081	1.69E-168	1.50E-166	up	UDP-glucuronosyl and UDP-glucosyl transferase
Solyc12g006260.1	1770	1642	1872	44	27	39	710.7718	1373.76	47.787871	-4.877543906	2.23E-168	1.98E-166	down	--
Solyc08g083130.4	2049	2465	2836	82	111	110	1017.298	1907.12	127.472898	-3.900716886	4.52E-168	3.99E-166	down	Transcription factor HEX, contains HOX and HALZ domains
Solyc02g085830.4	465	441	536	1790	1942	2037	1412.589	374.109	2451.06816	2.710607306	5.48E-168	4.81E-166	up	Spindle pole body protein
Solyc08g078650.3	63	71	74	1098	2370	1683	1094.288	54.1589	2134.41689	5.302994536	3.71E-167	3.25E-165	up	Glycosyl transferase, family 8 - glycogenin
Solyc04g007890.3	3239	3160	4200	225	222	228	1514.947	2741.51	288.383976	-3.255886124	6.61E-167	5.75E-165	down	--
Solyc08g075870.3	7153	7357	9068	836	1110	1087	3694.167	6110.81	1277.52697	-2.257527302	1.13E-165	9.77E-164	down	--
Solyc11g017410.1	57	94	101	1203	2311	1857	1148.891	65.4195	2232.36302	5.093385826	1.57E-165	1.36E-163	up	--
Solyc08g006460.3	929	952	1053	2255	2876	2899	2074.933	762.997	3386.86886	2.15070934	3.31E-165	2.84E-163	up	FOG: Predicted E3 ubiquitin ligase
Solyc01g110370.4	205	232	219	1050	1503	1573	950.2391	171.317	1729.16111	3.339086663	1.15E-162	9.78E-161	up	--
Solyc01g150156.1	1680	1614	1732	58	91	76	701.5341	1308.75	94.3169905	-3.787679475	1.53E-162	1.30E-160	down	--
Solyc02g084430.3	50	98	72	1162	1461	1242	847.6091	57.7813	1637.43698	4.831997192	1.25E-160	1.05E-158	up	--
Solyc05g053600.3	298	291	361	1293	1414	1519	1019.983	246.257	1793.70886	2.86291179	5.76E-160	4.85E-158	up	Pleiotropic drug resistance proteins (PDR1-15)
Solyc05g012230.4	111	100	140	866	1484	1490	844.1515	90.6976	1597.60541	4.137115489	1.01E-158	8.48E-157	up	--
Solyc10g074730.3	194	189	203	985	1354	1579	896.8885	152.575	1641.2021	3.429432507	3.24E-158	2.70E-156	up	--

Solyc01g111250.3	369	541	554	2895	4165	3711	2454.491	380.878	4528.10445	3.572097295	5.29E-158	4.39E-156	up	Glycosylphosphatidylinositol-specific phospholipase C
Solyc03g116530.3	1063	1051	1239	2702	3780	3299	2494.872	870.46	4119.28366	2.242665087	1.82E-157	1.51E-155	up	--
Solyc05g056300.4	222	186	243	968	1270	1102	790.0056	168.679	1411.33181	3.06307852	1.95E-157	1.60E-155	up	Thioredoxin
Solyc08g076860.3	3242	2793	3294	7191	10284	10453	7068.146	2424.18	11712.1095	2.272680262	1.37E-156	1.12E-154	up	--
Solyc04g040180.3	2959	3240	3487	10499	12155	10888	8387.556	2519.97	14255.1422	2.49997396	1.80E-156	1.47E-154	up	Methyltransferase
Solyc05g015510.3	1578	1405	1684	4657	7699	6936	4633.795	1211.86	8055.72479	2.733116511	1.06E-155	8.62E-154	up	--
Solyc04g049670.4	3475	3519	3698	342	387	403	1632.497	2785.01	479.98681	-2.538828787	4.79E-155	3.87E-153	down	GATA-4/5/6 transcription factors
Solyc07g052990.1	1932	1920	2770	75	68	77	901.1267	1708.07	94.1850316	-4.192821477	2.12E-153	1.71E-151	down	--
Solyc10g085680.2	1146	1285	1416	3666	4759	4173	3162.501	999.985	5325.01764	2.412884633	3.16E-152	2.53E-150	up	Protein kinase PCTAIRE and related kinases
Solyc04g051490.3	197	142	112	1923	2071	2178	1370.857	118.584	2623.13045	4.471650547	2.14E-151	1.70E-149	up	--
Solyc05g007480.1	110	156	135	1111	1976	1630	1035.616	104.894	1966.3382	4.232837218	7.57E-151	5.95E-149	up	--
Solyc03g081330.1	85	118	164	989	1239	1289	789.094	94.5423	1483.64565	3.967570999	3.83E-150	3.00E-148	up	--
Solyc03g115220.4	705	878	944	3520	4850	5641	3262.858	656.97	5868.74566	3.159606731	8.42E-150	6.57E-148	up	Cytochrome P450 CYP2 subfamily
Solyc01g010640.3	1590	1660	1795	3790	5478	5326	3717.814	1312.73	6122.90172	2.222334932	1.77E-149	1.37E-147	up	--
Solyc06g053260.1	9	4	2	2662	3133	3358	1937.29	3.98087	3870.59859	9.955408758	3.31E-149	2.55E-147	up	--
Solyc08g007800.3	445	475	545	1847	2145	1923	1446.915	380.448	2513.38066	2.723109245	3.72E-149	2.85E-147	up	Predicted transporter/transmembrane protein
Solyc10g051030.2	95	81	68	693	857	809	530.9953	64.0137	997.976824	3.969905945	1.28E-147	9.77E-146	up	--
Solyc02g038805.1	503	463	532	1260	1670	1650	1159.158	389.385	1928.93087	2.309321587	1.78E-147	1.35E-145	up	FOG: Predicted E3 ubiquitin ligase
Solyc01g096630.3	130	153	147	707	950	912	597.0664	112.215	1081.91748	3.273071453	2.67E-147	2.02E-145	up	--
Solyc03g113270.3	11726	11203	12889	23707	35752	32185	23869.83	9307.69	38431.9691	2.045898494	8.40E-146	6.33E-144	up	Transcription factor HEX
Solyc01g005440.4	898	798	964	1926	2626	2460	1821.524	690.559	2952.48952	2.096298879	1.66E-144	1.24E-142	up	--
Solyc02g089520.2	1261	1209	1500	44	41	43	542.0285	1029.23	54.8268133	-4.247130823	2.50E-144	1.86E-142	down	GATA-4/5/6 transcription factors
Solyc04g074180.4	1252	1408	1397	4289	6880	6458	4211.373	1057.96	7364.79047	2.800182888	2.85E-144	2.12E-142	up	Deoxyribodipyrimidine photolyase/cryptochrome
Solyc01g102270.2	109	99	161	782	982	901	611.0718	94.8838	1127.25981	3.56352787	4.70E-144	3.48E-142	up	Anaphase-promoting complex (APC), subunit 11
Solyc03g019950.3	1201	1143	1225	2800	4008	4064	2744.425	929.444	4559.40659	2.29543463	6.04E-144	4.45E-142	up	--
Solyc01g095090.3	173	209	233	1441	1548	1356	1007.319	159.732	1854.90656	3.536681227	1.13E-143	8.30E-142	up	Predicted E3 ubiquitin ligase
Solyc03g098010.3	2	5	12	1388	1798	1818	1056.793	4.78288	2108.80274	8.741212238	1.86E-142	1.36E-140	up	Purple (tartrate-resistant) acid phosphatase
Solyc01g100760.2	436	448	562	1805	1992	1909	1400.963	374.56	2427.36553	2.694409958	1.92E-141	1.39E-139	up	--
Solyc12g099250.2	155	98	120	864	1134	1011	683.8816	97.0505	1270.71277	3.711605011	3.20E-141	2.30E-139	up	Tyrosine kinase specific for activated (GTP-bound) p21cdc42Hs
Solyc06g082410.1	264	389	417	2250	3082	2517	1795.758	277.952	3313.5642	3.575611749	5.58E-141	3.99E-139	up	--
Solyc01g006680.4	1293	1224	1350	37	59	56	534.8669	1006.22	63.5110368	-3.974860963	8.42E-141	5.99E-139	down	Uncharacterized conserved protein, contains JmjC domain
Solyc03g097980.4	256	259	281	858	1187	1113	767.9413	207.141	1328.74181	2.68317789	9.00E-141	6.38E-139	up	G-protein alpha subunit (small G protein superfamily)
Solyc01g099160.4	2255	2729	3047	9878	16642	15850	9872.213	2085.75	17658.6798	3.081889283	1.33E-140	9.42E-139	up	--
Solyc02g079170.3	2438	2744	2887	6325	7639	8105	5711.497	2100.41	9322.58421	2.150261166	1.62E-140	1.14E-138	up	NADH-dehydrogenase (ubiquinone)
Solyc03g082660.3	2805	3194	3693	242	249	266	1418.828	2515.28	322.376515	-2.968505789	1.38E-139	9.68E-138	down	Predicted sugar transporter
Solyc01g095080.3	7078	10128	12078	53785	76364	67802	45429.57	7581.5	83277.6495	3.457351183	1.53E-139	1.07E-137	up	1-aminocyclopropane-1-carboxylate synthase
Solyc02g077610.3	120	144	125	728	918	860	580.5552	101.813	1059.29688	3.384057788	3.83E-139	2.66E-137	up	--
Solyc05g050010.3	2601	2572	2474	7362	11166	9499	6880.332	1997.31	11763.3531	2.558699846	1.00E-138	6.93E-137	up	1-aminocyclopropane-1-carboxylate synthase
Solyc07g045190.3	528	478	514	1461	1917	2113	1352.018	395.926	2308.11024	2.544918276	1.79E-138	1.23E-136	up	FOG: Predicted E3 ubiquitin ligase
Solyc02g093290.3	556	487	693	1864	2951	2623	1780.663	448.454	3112.87117	2.794492481	4.34E-137	2.97E-135	up	Nicotinic acid phosphoribosyltransferase
Solyc07g042550.3	4439	3986	4798	9064	11316	12934	8729.124	3432.92	14025.3297	2.030576377	8.75E-137	5.97E-135	up	Glycosyltransferase
Solyc08g081990.3	526	492	680	1730	2671	2403	1644.505	438.802	2850.20805	2.698729831	8.80E-137	5.98E-135	up	--
Solyc11g013330.3	1361	1493	1799	34	45	21	624.4923	1206.26	42.7201155	-4.832568622	4.45E-136	3.01E-134	down	--
novel.953	261	228	241	1201	1236	1328	896.7016	190.286	1603.117	3.075116771	9.36E-136	6.31E-134	up	--
Solyc02g062790.3	35	49	47	480	778	729	431.9951	34.16	829.830108	4.608033575	3.02E-135	2.02E-133	up	Serine/threonine protein kinase
Solyc05g041910.3	2019	2299	2610	5304	6821	6551	4840.659	1799	7882.31539	2.131422928	7.51E-135	5.01E-133	up	Predicted membrane protein, contains DoH
Solyc02g092460.3	1519	1158	1616	37	50	55	585.3059	1111	59.6076352	-4.215167673	9.14E-134	6.07E-132	down	CREB binding protein/P300
Solyc05g053210.4	8675	10026	11890	30793	41272	34205	26415.52	7931.84	44899.2002	2.500929403	1.09E-133	7.21E-132	up	Serine/threonine protein kinase
Solyc07g056420.4	6815	7365	9684	20365	27408	25438	18511.09	6169.95	30852.2336	2.321940171	1.66E-133	1.09E-131	up	Glutathione S-transferase
Solyc08g066705.1	939	962	1195	3650	5318	3943	3123.098	802.228	5443.96704	2.76228993	6.02E-133	3.94E-131	up	--

Solyc03g123370.3	1913	1975	2400	4704	6857	7023	4707.831	1630.37	7785.29035	2.255703938	6.90E-133	4.50E-131	up	Predicted guanosine polyphosphate pyrophosphohydrolase
Solyc03g114340.3	854	807	877	1925	2735	2446	1824.822	660.706	2988.93861	2.178607547	1.98E-132	1.28E-130	up	--
Solyc05g052950.4	1	5	11	1489	1798	2220	1161.116	4.27973	2317.95268	9.038881439	3.42E-132	2.21E-130	up	FOG: RCC1 domain
Solyc02g084420.3	34	61	83	806	1209	931	642.6634	45.8136	1239.51318	4.752156595	8.31E-132	5.34E-130	up	--
Solyc04g007220.4	855	675	810	2331	2716	2633	1932.569	608.138	3257.00001	2.420747421	2.02E-131	1.29E-129	up	--
Solyc07g008560.3	66	35	73	836	790	990	579.544	44.6551	1114.4329	4.6316413	7.41E-131	4.73E-129	up	Purple acid phosphatase
Solyc05g007770.3	1879	2295	2447	10304	10956	9725	7476.478	1721.97	13230.9823	2.941714798	1.00E-130	6.37E-129	up	--
Solyc02g067230.3	87	107	91	668	1034	1147	631.4474	74.6306	1188.26427	3.999440284	1.70E-130	1.08E-128	up	--
Solyc09g061280.4	238	201	265	1950	1716	1714	1248.482	182.35	2314.61511	3.663956511	2.08E-130	1.32E-128	up	--
Solyc09g056340.3	1613	1525	2193	80	73	90	739.7714	1375.78	103.757852	-3.738746342	4.19E-130	2.64E-128	down	GTPase Rab11/YPT3, small G protein superfamily
Solyc04g080300.4	1743	1669	1977	4185	4713	4535	3553.493	1399.12	5707.8633	2.027897087	1.37E-129	8.61E-128	up	--
Solyc11g069700.2	1112	972	731	7291	7764	9346	5541.077	740.516	10341.6374	3.804760795	2.45E-129	1.53E-127	up	Translation elongation factor EF-1 alpha/Tu
Solyc10g078240.2	433	705	684	4133	7060	7009	4025.056	474.618	7575.49308	3.997094597	5.84E-129	3.63E-127	up	Cytochrome P450 CYP2 subfamily
Solyc04g009910.3	102	119	105	873	955	832	609.9485	85.2938	1134.60315	3.736773348	2.10E-128	1.30E-126	up	Ca2+/calmodulin-dependent protein kinase
Solyc08g062820.3	817	607	829	2792	3180	2900	2177.916	583.575	3772.25681	2.691409421	9.99E-128	6.18E-126	up	--
Solyc03g120580.3	83	53	66	749	1431	995	687.7989	52.5295	1323.06838	4.656197828	1.43E-127	8.80E-126	up	H+/oligopeptide symporter
Solyc01g010230.2	646	466	615	2460	2581	2631	1857.756	447.899	3267.6126	2.866048864	2.82E-127	1.73E-125	up	--
Solyc01g099580.3	141	134	193	704	904	820	573.4363	120.767	1026.10601	3.082562035	5.37E-127	3.29E-125	up	--
Solyc06g062780.4	391	450	546	1514	2018	1755	1295.768	359.377	2232.15972	2.634125	8.92E-127	5.44E-125	up	P-type ATPase
Solyc12g006970.1	1872	1854	2366	163	165	171	895.2202	1577.59	212.849696	-2.897309538	1.18E-126	7.16E-125	down	--
Solyc03g095770.3	209	168	199	786	901	990	641.307	149.732	1132.88163	2.919472175	3.34E-126	2.02E-124	up	--
Solyc08g068600.3	51	64	58	502	570	585	373.9639	45.2131	702.714708	3.962722698	5.04E-126	3.05E-124	up	Glutamate decarboxylase and related proteins
Solyc07g045180.4	980	980	1245	2403	3070	2796	2162.608	830.029	3495.18708	2.073325532	9.40E-126	5.66E-124	up	--
Solyc09g008175.1	1071	1096	1341	38	54	58	486.1377	909.426	62.8497054	-3.847663994	6.20E-125	3.72E-123	down	--
Solyc12g009610.2	2887	2730	3064	5592	8482	7889	5729.811	2257.7	9201.92517	2.027514012	2.17E-124	1.30E-122	up	Ypt/Rab-specific GTPase-activating protein GYP7
Solyc12g044240.3	1138	1197	1429	58	73	59	528.4687	976.361	80.5764205	-3.603874611	1.03E-123	6.16E-122	down	Dynein light chain type 1
Solyc02g089640.3	2183	2025	2747	177	208	247	1032.569	1798.52	266.621703	-2.754021953	1.13E-123	6.71E-122	down	--
Solyc01g103590.4	1095	975	1225	23	43	31	447.4062	854.375	40.4372887	-4.387333903	3.39E-123	2.01E-121	down	--
Solyc06g073180.3	1130	1489	1457	4314	5985	5728	3901.05	1062.51	6739.58806	2.665739525	5.09E-123	2.99E-121	up	--
Solyc11g068960.2	175	99	150	846	1090	1072	689.2665	109.772	1268.76093	3.529079918	8.94E-123	5.24E-121	up	--
Solyc04g081890.1	1766	2422	2790	9698	11569	11349	7810.342	1809.1	13811.5829	2.932417538	2.55E-122	1.49E-120	up	FOG: Predicted E3 ubiquitin ligase
Solyc07g040680.3	382	353	434	995	1355	1222	904.2819	303.233	1505.33106	2.311313936	5.72E-122	3.32E-120	up	Heat shock transcription factor
Solyc01g104650.3	8374	7608	8665	914	1016	1261	3878.679	6408.93	1348.43185	-2.249014771	6.91E-122	4.00E-120	down	--
Solyc08g006820.4	2237	2598	2850	205	277	260	1155.022	1997.49	312.5536	-2.67490756	1.78E-121	1.02E-119	down	Endosomal membrane proteins, EMP70
Solyc12g056940.2	4776	5243	5950	13157	21646	20184	13549.25	4147.69	22950.814	2.46829309	6.59E-121	3.75E-119	up	Acetyl-CoA carboxylase
Solyc01g106580.3	1132	1161	1285	63	82	78	512.265	930.448	94.0817771	-3.305436511	8.52E-121	4.84E-119	down	--
novel.1018	1221	985	1420	29	34	44	491.0161	936.982	45.0497913	-4.376369055	1.51E-120	8.56E-119	down	--
Solyc01g091700.4	93	108	108	545	728	635	442.981	80.546	805.415948	3.324572048	2.23E-120	1.26E-118	up	--
Solyc12g096070.2	347	281	357	937	1240	1144	828.1879	255.507	1400.86874	2.454356695	5.29E-120	2.97E-118	up	--
Solyc02g072190.4	122	167	179	813	1291	1343	779.7738	121.62	1437.9281	3.565099806	9.22E-120	5.16E-118	up	--
Solyc01g094810.3	511	422	566	1487	1648	1690	1218.487	388.137	2048.83672	2.398265598	1.21E-119	6.74E-118	up	Ubiquitin-protein ligase
Solyc05g052240.3	2814	3679	4092	11014	13966	14656	9727.644	2748.15	16707.1429	2.603952503	3.15E-119	1.75E-117	up	--
Solyc01g096230.4	1011	1243	1294	3280	4848	4878	3185.22	923.415	5447.02634	2.561059791	1.35E-118	7.48E-117	up	AAA+-type ATPase
Solyc04g071770.3	913	930	1036	2640	2772	2909	2144.96	748.56	3541.36041	2.241632818	1.49E-118	8.21E-117	up	--
Solyc07g063590.4	66	76	86	476	697	582	398.6654	59.207	738.123691	3.640750337	2.01E-118	1.11E-116	up	Myosin class V heavy chain
Solyc03g098480.1	1033	994	1253	31	37	21	444.0079	849.903	38.113195	-4.495785053	1.30E-117	7.12E-116	down	--
Solyc01g098110.4	3586	4251	4895	10453	15300	13682	9933.59	3303.82	16563.3631	2.325841575	1.96E-117	1.07E-115	up	--
Solyc06g005480.3	297	275	268	996	1143	1291	834.9441	219.394	1450.49411	2.727098899	8.71E-117	4.75E-115	up	--
Solyc01g108300.3	556	551	587	1317	1572	1584	1167.019	441.123	1892.91442	2.101859434	9.54E-117	5.19E-115	up	--
Solyc03g093080.3	1390	1381	1469	96	88	101	613.019	1104.14	121.896234	-3.190003892	2.15E-116	1.17E-114	down	--
Solyc07g054730.1	4221	3535	4507	515	573	593	1946.658	3179.81	713.500386	-2.158242994	3.27E-116	1.77E-114	down	--
Solyc05g005340.3	41	24	29	405	483	522	310.1129	24.4817	595.744154	4.607407889	4.15E-115	2.24E-113	up	--
Solyc06g007620.3	2583	2141	2824	159	261	185	1104.584	1955.35	253.819446	-2.942612971	5.36E-115	2.88E-113	down	--
Solyc03g121540.3	1455	1389	2079	6286	11722	11078	6669.713	1268.75	12070.6813	3.249783514	7.30E-115	3.91E-113	up	Beta-galactosidase

Solyc04g054740.3	41	18	32	831	749	681	498.6272	23.5185	973.735807	5.365569042	9.95E-115	5.32E-113	up	Myo-inositol-1-phosphate synthase
Solyc06g075370.3	33	35	48	533	1073	706	495.984	29.9546	962.013323	5.002072527	4.11E-114	2.18E-112	up	--
Solyc06g072430.3	430	380	492	1226	1370	1578	1051.978	337.309	1766.64779	2.387497496	4.19E-114	2.22E-112	up	--
Solyc03g117310.1	1020	1093	1399	18	20	14	465.563	908.859	22.2664967	-5.369257691	1.27E-113	6.73E-112	down	--
Solyc09g015770.3	1118	1122	1437	4970	4515	5478	3672.655	951.998	6393.31272	2.746845081	1.32E-113	6.95E-112	up	--
Solyc06g076750.3	890	722	1122	3480	3856	3485	2656.691	704.787	4608.59518	2.707580074	5.11E-113	2.68E-111	up	H+/oligopeptide symporter
Solyc09g091580.3	1940	1950	2356	4644	7584	6491	4724.247	1620.08	7828.41587	2.272870124	1.55E-112	8.10E-111	up	Predicted unusual protein kinase
novel.7	30	9	25	408	516	526	314.0488	16.4202	611.677349	5.205585241	2.52E-112	1.32E-110	up	--
Solyc03g117660.4	210	224	253	675	918	925	618.6015	178.496	1058.70666	2.569515225	4.87E-112	2.53E-110	up	--
Solyc01g005130.2	463	417	533	1085	1586	1527	1063.421	366.157	1760.68414	2.265816069	1.35E-111	6.99E-110	up	--
Solyc12g006973.1	1723	1860	2169	110	193	173	845.5649	1492.88	198.250865	-2.904245103	2.75E-111	1.42E-109	down	--
Solyc09g090930.3	563	630	699	1421	1942	1806	1334.132	491.705	2176.55827	2.146773081	4.41E-111	2.27E-109	up	--
Solyc01g081540.4	1107	1060	1077	2192	3030	2911	2133.238	846.103	3420.37245	2.016513668	4.87E-111	2.50E-109	up	Myosin class V heavy chain
novel.1030	1288	1044	1193	40	66	62	493.6689	917.236	70.1017066	-3.698885386	2.54E-110	1.29E-108	down	FOG: Transposon-encoded proteins with TYA
Solyc07g006890.1	1283	1030	1392	2652	3171	3357	2419.697	959.342	3880.05092	2.015240754	7.58E-110	3.86E-108	up	Cytochrome P450 CYP4/CYP19/CYP26
Solyc10g076360.1	880	1028	1000	2578	3350	3849	2433.176	758.632	4107.71982	2.43787886	2.57E-109	1.30E-107	up	Prenylated rab acceptor 1
Solyc02g083250.3	1052	980	1196	2682	3106	2750	2233.477	837.468	3629.48552	2.114822197	8.25E-109	4.17E-107	up	Hismacro and SEC14 domain-containing proteins
Solyc12g019700.1	1972	1640	1971	118	156	196	823.6128	1450.33	196.898577	-2.876037759	1.55E-108	7.84E-107	down	--
Solyc12g057160.1	1034	902	1262	40	36	49	439.9313	826.596	53.2663849	-3.966254512	5.00E-108	2.51E-106	down	--
Solyc02g093840.3	457	515	590	1300	1860	1633	1210.814	405.517	2016.11133	2.314112571	6.64E-108	3.33E-106	up	--
Solyc01g109780.3	1904	2273	2487	5305	8063	7107	5159.403	1731.97	8586.83539	2.309974819	9.84E-108	4.92E-106	up	--
Solyc01g007070.3	3003	3009	3898	7871	13305	12116	8224.88	2564.86	13884.8989	2.436598282	1.18E-107	5.86E-106	up	Transcription factor MEIS1
Solyc11g007770.2	662	730	853	2615	4547	5053	2825.728	582.551	5068.9058	3.121613021	2.75E-107	1.36E-105	up	Acetylglucosaminyltransferase EXT2/exostosin 2
Solyc02g069260.3	785	521	713	2348	3176	3639	2183.068	523.453	3842.68329	2.875929731	3.90E-107	1.93E-105	up	Translation initiation factor 2C (eIF-2C)
Solyc12g005850.2	555	505	696	1638	1771	2016	1377.305	453.934	2300.67675	2.339671282	5.77E-107	2.84E-105	up	Uncharacterized membrane protein
Solyc04g005540.3	411	338	462	1371	2483	2363	1448.169	313.394	2582.94325	3.043049446	1.22E-106	5.97E-105	up	Apoptotic ATPase
Solyc12g036330.1	24	8	18	471	497	488	316.6949	12.8839	620.505825	5.578865281	1.85E-106	9.04E-105	up	Serine/threonine protein kinase
Solyc05g017980.3	1554	1577	1848	156	193	159	754.0655	1292.59	215.541882	-2.588057994	2.01E-106	9.80E-105	down	--
Solyc02g089440.3	1341	1351	1618	120	155	164	651.5264	1118.21	184.842691	-2.59513433	2.67E-106	1.30E-104	down	--
Solyc04g081910.4	1291	1334	1681	111	122	128	634.2294	1115.19	153.273072	-2.868058795	3.33E-106	1.61E-104	down	Ca2+/calmodulin-dependent protein kinase
Solyc01g088810.4	18	22	25	405	459	414	280.2688	16.8708	543.666853	5.007687927	4.34E-106	2.09E-104	up	FOG: Ankyrin repeat
Solyc02g092000.3	1539	1750	1729	119	160	132	741.1434	1308.65	173.636489	-2.914316292	5.74E-106	2.76E-104	down	--
Solyc03g116710.3	149	139	174	641	1158	1079	658.0301	119.766	1196.2947	3.321224587	2.40E-105	1.15E-103	up	--
novel.1218	957	797	1083	24	22	25	382.3077	734.24	30.3751623	-4.61167238	4.41E-105	2.10E-103	down	--
Solyc09g007510.3	46	31	42	336	474	461	282.3595	30.8572	533.861885	4.112381806	6.03E-105	2.86E-103	up	--
Solyc01g086680.4	21	21	33	562	468	654	369.6611	19.2903	720.031992	5.211374244	9.22E-105	4.37E-103	up	Glutathione S-transferase
Solyc03g031430.1	1344	1392	1764	113	148	151	669.349	1165.2	173.497782	-2.746299457	1.14E-104	5.41E-103	down	--
Solyc07g005760.3	5839	7798	8163	23224	38657	32644	22588.16	5670.23	39506.0812	2.800665651	1.36E-104	6.38E-103	up	--
Solyc08g067970.3	497	496	550	1345	1503	1806	1184.518	401.299	1967.73694	2.294009112	1.36E-104	6.38E-103	up	--
Solyc04g074580.1	2924	2679	3401	407	468	436	1445.152	2333.43	556.870102	-2.070238564	1.63E-104	7.62E-103	down	Histones H3 and H4
Solyc12g044190.3	979	837	996	16	31	37	382.608	730.556	34.6596974	-4.372004457	2.65E-104	1.24E-102	down	Apoptotic ATPase
Solyc03g097670.4	1080	1044	1036	2829	4816	4619	2966.494	824.715	5108.27231	2.631959039	3.98E-104	1.85E-102	up	--
Solyc03g113480.1	1096	1021	1632	21	30	42	501.5287	964.328	38.7292969	-4.625398918	7.57E-104	3.51E-102	down	--
Solyc01g079610.3	1270	1450	1589	94	81	96	618.1729	1120.15	116.197435	-3.280966596	8.13E-104	3.76E-102	down	Molecular chaperone (DnaJ superfamily)
Solyc07g063880.4	12	20	14	402	457	437	281.3001	12.1033	550.49692	5.521413485	1.15E-103	5.33E-102	up	Beta-glucosidase, lactase phlorizinhydrolase
Solyc01g010490.4	1845	1714	2017	201	286	260	881.1726	1448.27	314.076269	-2.202670522	1.25E-103	5.77E-102	down	Cytochrome P450 CYP2 subfamily
Solyc12g089210.2	589	469	642	2598	2625	2303	1831.77	440.043	3223.49783	2.871509411	1.32E-103	6.06E-102	up	Ornithine carbamoyltransferase OTC/ARG3
Solyc11g072623.1	9	7	10	518	552	645	367.0487	6.72265	727.374732	6.75077523	3.00E-103	1.38E-101	up	--
Solyc07g042270.4	2191	2359	2646	290	352	356	1145.952	1869.96	421.945908	-2.148782324	4.52E-103	2.06E-101	down	ATP-dependent RNA helicase
Solyc06g072330.3	210	133	176	971	984	1155	728.5298	134.744	1322.31602	3.293734584	5.74E-103	2.62E-101	up	Molecular chaperone (DnaJ superfamily)
Solyc06g082010.4	1336	1119	1605	2960	3497	3521	2636.897	1048.95	4224.84408	2.0088453	6.81E-103	3.09E-101	up	CCCH-type Zn-finger protein
Solyc06g074800.1	29	22	26	400	881	658	411.106	20.0262	802.185673	5.327092901	8.19E-103	3.70E-101	up	FOG: Zn-finger
Solyc10g024420.3	8381	8456	9546	18012	28613	22745	17973.11	6857.39	29088.8192	2.084817519	1.26E-102	5.66E-101	up	Multidrug resistance-associated protein
Solyc10g052880.1	635	706	971	4320	3898	3960	2913.252	596.756	5229.74865	3.130387583	1.72E-102	7.74E-101	up	FOG: Leucine rich repeat
Solyc02g087970.1	1678	1374	1532	3239	4497	4040	3075.967	1193.56	4958.37393	2.055058951	2.98E-102	1.33E-100	up	--

novel.1954	1227	1129	1269	78	76	105	526.3817	942.895	109.868317	-3.105427681	4.03E-102	1.80E-100	down	--
Solyc09g007790.1	2596	1885	2625	196	229	213	1055.344	1839.9	270.78619	-2.767494326	4.57E-102	2.04E-100	down	--
Solyc01g067010.3	415	467	629	1720	1892	1925	1371.388	390.24	2352.53475	2.589854197	7.45E-102	3.27E-100	up	--
Solyc01g098520.3	825	824	906	20	25	32	348.4967	664.665	32.3283781	-4.355378569	1.36E-101	5.95E-100	down	DNA mismatch repair protein - MLH3 family
Solyc01g086975.1	246	278	272	973	1573	1183	885.8048	207.657	1563.9521	2.915203243	6.80E-101	2.96E-99	up	--
Solyc02g093710.1	428	398	459	1075	1629	1749	1097.129	333.958	1860.30059	2.479228156	1.41E-100	6.13E-99	up	--
Solyc06g073550.3	1276	1191	1351	114	119	130	573.5562	992.729	154.383829	-2.690951934	2.77E-100	1.20E-98	down	RNA-binding protein LARP/SRO9
Solyc05g056200.1	135	159	156	535	745	691	473.2956	117.36	829.231685	2.82422793	2.83E-100	1.22E-98	up	Serine/threonine protein kinase
Solyc07g051840.4	714	775	979	1845	2747	2585	1823.898	638.897	3008.89898	2.235524765	3.99E-100	1.72E-98	up	--
Solyc08g006500.4	57	76	90	421	556	512	342.9764	57.7716	628.181195	3.441029882	1.06E-99	4.55E-98	up	Glutamate-gated kainate-type ion channel receptor
Solyc10g005040.4	42	27	65	409	627	663	371.8911	34.1504	709.631784	4.363580386	1.16E-99	4.99E-98	up	--
Solyc02g085360.4	70	90	90	513	630	526	386.6761	65.1325	708.219828	3.443985379	1.19E-99	5.08E-98	up	Cytochrome P450 CYP4/CYP19/CYP26
Solyc01g095330.2	1589	1172	1380	4020	4453	4600	3313.844	1077.34	5550.352	2.365028145	2.28E-99	9.71E-98	up	--
Solyc07g064500.3	3039	3242	3965	397	511	552	1635.013	2655.47	614.554601	-2.110437894	2.75E-99	1.17E-97	down	Purple acid phosphatase
Solyc05g011960.4	2165	1964	2415	259	309	259	1024.567	1697.71	351.429749	-2.276236579	3.78E-99	1.60E-97	down	--
Solyc06g068460.3	613	809	1071	3086	4221	3889	2679.318	643.5	4715.13571	2.872640771	3.78E-99	1.60E-97	up	--
Solyc04g073950.2	1370	1689	2354	6524	8188	7559	5407.404	1395.47	9419.33304	2.754384879	5.02E-99	2.12E-97	up	--
Solyc03g095860.3	1243	1409	1599	110	135	137	632.6556	1103.91	161.403892	-2.774728033	5.18E-99	2.18E-97	down	Fibrillins and related proteins
Solyc10g007280.4	15	11	18	494	488	438	309.9843	11.3353	608.633385	5.73496845	8.73E-99	3.67E-97	up	AAA+-type ATPase
Solyc02g079490.3	10	14	12	576	561	478	351.5124	9.42013	693.604588	6.208722902	8.86E-99	3.72E-97	up	--
Solyc08g077230.3	1796	2163	2668	5563	8381	7599	5374.547	1715.65	9033.44785	2.396484355	3.33E-98	1.39E-96	up	GATA-4/5/6 transcription factors
Solyc01g111380.3	161	212	222	785	887	896	622.2573	154.771	1089.7438	2.815748369	5.98E-98	2.48E-96	up	Actin depolymerizing factor
Solyc12g039080.3	179	133	156	573	743	805	507.0854	121.763	892.407702	2.875168123	8.16E-98	3.38E-96	up	--
Solyc03g006400.3	1962	2249	2503	178	276	266	1022.796	1744.45	301.137601	-2.529549867	1.02E-97	4.22E-96	down	Tyrosine kinase specific for activated (GTP-bound) p21cdc42Hs
Solyc04g006970.4	716	779	787	1973	3123	2445	1879.228	594.866	3163.59069	2.412038203	1.91E-97	7.84E-96	up	--
Solyc12g011030.3	1260	1245	1259	10	4	11	496.2812	981.689	10.8733284	-6.53624994	3.12E-97	1.28E-95	down	--
Solyc06g072840.3	1054	911	1197	72	88	80	460.3149	818.952	101.678073	-3.013660703	8.92E-97	3.64E-95	down	--
Solyc02g030225.1	1034	1008	1279	79	99	104	489.5999	860.266	118.933441	-2.854423362	9.58E-97	3.90E-95	down	UDP-glucose 4-epimerase/UDP-sulfoquinovose synthase
Solyc06g073320.3	10347	9667	11623	22539	39500	37609	24835.11	8211.45	41458.767	2.336036327	3.21E-96	1.30E-94	up	Predicted hydrolase (HIT family)
Solyc09g074050.3	68	57	83	393	454	471	306.0122	53.7135	558.310937	3.371979253	4.02E-96	1.63E-94	up	Uncharacterized conserved protein
Solyc04g050930.3	4293	4093	4638	457	754	676	2087.064	3386.03	788.095353	-2.099878134	6.12E-96	2.47E-94	down	--
Solyc12g010540.1	979	891	1374	40	49	53	447.748	835.58	59.9155697	-3.802342317	7.87E-96	3.16E-94	down	UDP-glucose 4-epimerase/UDP-sulfoquinovose synthase
Solyc06g074670.4	1113	1131	1323	106	117	122	536.2672	926.065	146.469343	-2.665449248	1.41E-95	5.63E-94	down	dTDP-glucose 4-6-dehydratase/UDP-glucuronic acid decarboxylase
Solyc04g071600.3	58	56	52	608	504	637	396.7869	43.4063	750.167524	4.113903145	3.62E-95	1.44E-93	up	--
Solyc06g150137.1	2880	3876	4315	12094	21605	18499	12307.24	2873.77	21740.7211	2.919467108	4.08E-95	1.62E-93	up	Cytochrome P450 CYP2 subfamily
Solyc02g090870.1	144	104	173	821	1836	1650	942.09	108.419	1775.76063	4.031981865	5.73E-95	2.27E-93	up	--
Solyc03g096900.4	127	122	145	442	618	581	396.1716	102.28	690.063511	2.754936009	9.70E-95	3.83E-93	up	Predicted guanosine polyphosphate pyrophosphohydrolase
Solyc06g076570.4	6663	4780	5621	18002	19093	19706	14305.85	4440.51	24171.1841	2.44446311	1.62E-94	6.39E-93	up	Molecular chaperone (small heat-shock protein Hsp26/Hsp42)
Solyc08g075020.3	2845	3012	3268	6214	9447	7482	6049.818	2373.91	9725.72872	2.034782564	2.31E-94	9.08E-93	up	Pectin acetylesterase and similar proteins
Solyc07g049550.3	891	756	956	2356	3085	4026	2319.669	675.061	3964.27684	2.554129605	2.44E-94	9.56E-93	up	Iron/ascorbate family oxidoreductases
Solyc10g083380.2	1435	1352	1668	164	192	172	689.791	1155.32	224.265018	-2.36978465	4.96E-94	1.93E-92	down	--
Solyc10g086710.3	1313	1804	1730	85	87	103	690.4383	1264.04	116.838489	-3.439523329	7.55E-94	2.94E-92	down	--
Solyc06g071680.4	1754	1625	1995	231	294	281	867.2072	1394.02	340.390211	-2.034624817	2.85E-93	1.10E-91	down	Histone deacetylase complex
Solyc09g011560.3	3226	3886	4630	8702	11312	12213	8301.086	3043.27	13558.8984	2.155523236	3.32E-93	1.28E-91	up	Glutathione S-transferase
Solyc02g085910.4	1724	1021	1341	11	11	11	537.8791	1061.66	14.0979175	-6.249177116	3.90E-93	1.49E-91	down	--
Solyc09g092380.3	165243	199168	210509	21155	32094	30079	92241.28	149576	34906.6112	-2.099248559	1.34E-92	5.09E-91	down	S-adenosylhomocysteine hydrolase
Solyc03g097030.3	312	438	474	1552	2789	2594	1600.678	317.94	2883.41698	3.181684583	1.35E-92	5.12E-91	up	Acyl-CoA synthetase
Solyc05g005720.4	2166	2331	2686	224	335	247	1102.232	1865.02	339.446386	-2.457010323	1.38E-92	5.19E-91	down	--
Solyc07g064250.3	1698	1763	2033	222	288	259	875.8318	1426.78	324.882225	-2.135443505	2.73E-92	1.03E-90	down	COPII vesicle protein

novel.1062	12	20	23	384	368	436	260.3622	14.2446	506.479757	5.147220304	3.78E-92	1.42E-90	up	--
Solyc10g005250.3	65	43	58	350	502	429	291.0608	43.0571	539.064409	3.6456183	6.14E-92	2.29E-90	up	Uncharacterized membrane protein
Solyc11g073060.3	18	14	11	409	379	462	272.3818	11.3041	533.459459	5.572201328	9.46E-92	3.52E-90	up	FOG: Zn-finger
Solyc03g006960.4	2102	2855	3207	9531	10943	9859	7506.871	2118.45	12895.2939	2.605647833	1.03E-91	3.83E-90	up	Serine/threonine protein phosphatase
Solyc05g005320.1	90	55	64	488	880	667	451.7493	54.4692	849.029428	3.96546202	1.97E-91	7.30E-90	up	--
Solyc02g081830.4	1718	1209	1861	104	123	111	689.8961	1236.33	143.463885	-3.111379346	3.18E-91	1.17E-89	down	Predicted hydrolase (HAD superfamily)
Solyc12g005270.2	4667	4152	4627	617	623	712	2164.893	3499.1	830.684078	-2.076897766	3.22E-91	1.18E-89	down	Histone 2A
Solyc11g008250.2	514	572	626	1277	1951	1990	1313.26	445.131	2181.38831	2.294269334	3.65E-91	1.34E-89	up	--
Solyc03g078360.1	980	1089	1058	2482	4200	3770	2588.061	816.003	4360.11894	2.418874032	5.16E-91	1.89E-89	up	--
Solyc10g083990.2	710	764	902	22	22	32	324.2971	616.442	32.1516796	-4.263811277	7.12E-91	2.61E-89	down	--
Solyc11g010930.2	51	53	71	332	422	410	268.3331	45.2316	491.434542	3.437350701	1.47E-90	5.37E-89	up	Protein involved in membrane traffic
Solyc02g085600.1	1401	2017	2282	6569	10967	9768	6438.443	1478.24	11398.648	2.946990501	1.54E-90	5.61E-89	up	--
Solyc02g089500.4	767	702	919	19	29	40	327.4164	618.277	36.5557967	-4.063018408	6.74E-90	2.44E-88	down	GATA-4/5/6 transcription factors
Solyc06g010030.4	162	104	176	705	747	814	538.4721	113.907	963.036869	3.074803522	1.64E-89	5.94E-88	up	--
Solyc08g081230.1	397	420	670	1676	2189	2067	1442.357	382.085	2502.62908	2.709323555	2.23E-89	8.06E-88	up	SETA binding protein SB1
Solyc04g076880.3	13919	18486	20629	45312	55549	58638	40550.5	13766.3	67334.6728	2.290204492	1.64E-88	5.86E-87	up	--
Solyc06g076140.4	2642	2429	3680	11763	10984	10803	8323.781	2255.14	14392.4182	2.673557684	9.76E-88	3.48E-86	up	Predicted metallothionein
Solyc10g081930.1	47	48	41	359	397	522	287.5158	35.6358	539.395813	3.927272224	1.61E-87	5.70E-86	up	--
Solyc12g088240.2	721	888	944	13	21	13	341.8941	664.009	19.7792349	-5.06564213	1.83E-87	6.47E-86	down	--
Solyc03g115740.2	2574	2693	3028	288	471	402	1320.652	2155.77	485.531356	-2.146715634	1.83E-87	6.47E-86	down	Subunit of Golgi mannosyltransferase complex
Solyc04g081930.3	1787	1417	1936	166	220	184	785.7621	1330.61	240.917815	-2.466714871	2.49E-87	8.78E-86	down	Prolyl 4-hydroxylase alpha subunit
Solyc03g121090.4	576	559	1006	2993	3379	3627	2392.756	548.349	4237.16212	2.948134748	4.94E-87	1.74E-85	up	Nuclear localization sequence binding protein
Solyc10g077040.2	521	458	509	1177	2000	1832	1237.823	387.29	2088.35538	2.432528279	6.93E-87	2.44E-85	up	--
novel.1664	1701	1842	2148	4961	5133	4924	3943.79	1477.02	6410.56246	2.117284784	9.64E-87	3.38E-85	up	--
Solyc11g006410.2	548	590	729	1373	2205	1968	1401.506	483.685	2319.32739	2.261910671	1.23E-86	4.29E-85	up	--
Solyc03g031420.1	701	681	840	18	38	32	306.2549	576.107	36.4026511	-3.96224586	1.47E-86	5.13E-85	down	Molybdenum cofactor sulfurase
Solyc02g083860.3	2775	3332	3874	6964	8887	9897	6711.985	2588.95	10835.0162	2.06527832	1.70E-86	5.94E-85	up	Iron/ascorbate family oxidoreductases
Solyc08g076720.4	363	297	384	888	1013	1190	788.4595	270.646	1306.27262	2.270009159	2.60E-86	9.01E-85	up	Multidrug/pheromone exporter, ABC superfamily
novel.775	7	0	5	817	889	1104	595.8661	3.04619	1188.68606	8.576258394	3.57E-86	1.24E-84	up	--
Solyc07g064970.4	1831	1927	2376	244	328	307	979.9385	1589.5	370.378891	-2.100876086	4.39E-86	1.52E-84	down	Microtubule-associated protein
Solyc12g007070.2	2177	2481	3253	5816	8823	8206	5808.078	2044.76	9571.40101	2.226706592	7.01E-86	2.41E-84	up	Heat shock transcription factor
Solyc02g092490.3	139	150	168	485	772	656	459.9277	118.76	801.095332	2.755923785	3.13E-85	1.07E-83	up	--
Solyc12g100120.2	96	112	113	460	869	793	482.1695	83.6493	880.689703	3.400129585	5.69E-85	1.95E-83	up	--
Solyc01g007190.3	157	148	157	594	669	897	515.3698	120.358	910.381447	2.920886449	8.05E-85	2.75E-83	up	--
Solyc07g062670.1	23	17	43	330	368	396	242.4694	21.082	463.856883	4.43724983	2.72E-84	9.23E-83	up	--
novel.1001	927	781	945	61	56	62	382.8027	688.979	76.626158	-3.181624858	3.16E-84	1.07E-82	down	--
Solyc12g089110.1	3469	2915	3604	478	494	495	1608.91	2592.24	625.579653	-2.054624046	3.30E-84	1.12E-82	down	--
Solyc06g005500.4	433	424	544	943	1226	1338	918.9535	362.774	1475.13324	2.023285282	3.88E-84	1.31E-82	up	Serine/threonine protein kinase
novel.275	225	227	178	871	942	1037	687.5541	165.47	1209.63854	2.873528474	4.63E-84	1.56E-82	up	Karyopherin (importin) alpha
Solyc06g051400.3	1872	1997	2605	5509	10467	9165	6056.793	1674.42	10439.1677	2.640317989	4.82E-84	1.62E-82	up	--
Solyc07g006880.1	2157	1778	2427	135	231	254	951.9823	1646.45	257.511163	-2.669258658	1.43E-83	4.77E-82	down	--
Solyc06g076580.1	13	4	18	374	402	362	247.1719	8.84843	485.495391	5.743983339	1.48E-83	4.93E-82	up	--
Solyc06g068270.3	1803	1697	1795	176	260	268	837.1305	1379.56	294.700077	-2.221856759	1.72E-83	5.74E-82	down	Iron/ascorbate family oxidoreductases
Solyc09g091510.3	5262	7520	7968	19198	25273	26580	17642.9	5393.1	29892.692	2.470643627	1.86E-83	6.19E-82	up	--
Solyc10g078440.2	52	80	110	707	677	675	472.0409	62.3217	881.760181	3.816854408	2.82E-83	9.35E-82	up	--
Solyc02g072370.1	924	1035	1222	78	76	92	464.9009	825.076	104.725719	-2.984958013	3.74E-83	1.24E-81	down	--
Solyc03g115940.4	34	30	40	277	514	531	287.3906	26.9191	547.862166	4.347167826	4.50E-83	1.48E-81	up	--
Solyc03g120980.3	21316	25612	30866	60431	102065	85076	61790.93	20155.4	103426.455	2.359374041	5.51E-83	1.81E-81	up	Pleiotropic drug resistance proteins (PDR1-15)
Solyc02g086530.4	11	17	17	289	451	328	230.3363	11.7134	448.959244	5.263772489	8.28E-83	2.72E-81	up	--
novel.1368	916	837	1072	55	54	84	406.75	731.928	81.5717795	-3.167048494	1.69E-82	5.55E-81	down	--
Solyc10g007100.3	164	145	166	743	715	699	523.7245	123.518	923.931322	2.90136409	1.94E-82	6.34E-81	up	Uncharacterized membrane protein, predicted efflux pump
Solyc07g065410.1	406	335	269	1465	1641	1847	1181.304	265.312	2097.29686	2.985064018	2.77E-82	9.04E-81	up	--
Solyc07g054770.1	1645	896	1755	6	6	11	556.9676	1104.27	9.66769549	-6.831130751	1.98E-81	6.43E-80	down	--

Solyc02g088090.1	80	127	183	863	935	1024	649.0246	100.252	1197.79735	3.574459782	2.67E-81	8.65E-80	up	Calmodulin and related proteins (EF-Hand superfamily)
Solyc06g050170.4	251	275	289	723	1226	1022	726.5676	212.19	1240.94542	2.550379455	6.22E-81	2.00E-79	up	--
Solyc03g005100.3	830	971	1082	50	30	40	400.6397	748.949	52.3304495	-3.867975008	1.04E-80	3.31E-79	down	Arylacetamide deacetylase
Solyc01g095320.4	18999	14691	18448	42292	82236	70947	47320.37	13534.1	81106.5925	2.583242501	1.20E-80	3.83E-79	up	--
Solyc03g119740.3	1264	1377	1530	155	191	196	656.5101	1084.12	228.902705	-2.244065676	1.22E-80	3.88E-79	down	Zuotin and related molecular chaperones (DnaJ superfamily)
Solyc02g091030.4	1551	1433	1606	178	257	240	738.7094	1193.97	283.447014	-2.071173979	1.73E-80	5.52E-79	down	CCAAT-binding factor, subunit C (HAP5)
Solyc12g014120.3	775	738	786	48	56	50	332.1226	598.818	65.4274141	-3.200234791	1.80E-80	5.73E-79	down	--
Solyc11g012440.1	1166	1093	1238	133	163	158	550.659	909.279	192.038974	-2.244884223	6.97E-80	2.20E-78	down	FOG: Armadillo/beta-catenin-like repeats
Solyc12g096570.1	1217	1274	1451	146	168	142	609.0896	1024.07	194.110141	-2.405218257	9.43E-80	2.97E-78	down	--
Solyc09g097960.3	8	23	22	1604	1444	1788	1039.972	13.7842	2066.15905	7.228861649	1.45E-79	4.55E-78	up	Voltage-gated shaker-like K+ channel, subunit beta/KCNAB
Solyc01g028803.1	513	542	614	1155	1919	1691	1211.858	433.626	1990.08931	2.199561602	1.60E-79	5.00E-78	up	Cytochrome P450 CYP4/CYP19/CYP26
Solyc03g113720.3	70	61	65	335	378	394	260.2621	51.0794	469.444724	3.201496363	2.51E-79	7.83E-78	up	GATA-4/5/6 transcription factors
Solyc08g006470.4	12	5	5	465	401	614	317.8624	5.76979	629.95503	6.781310878	4.06E-79	1.26E-77	up	C2H2-type Zn-finger protein
Solyc08g005120.3	109	137	144	459	770	765	465.9955	101.46	830.531063	3.036086194	5.98E-79	1.85E-77	up	Flavonol reductase/cinnamoyl-CoA reductase
Solyc01g100230.3	687	709	768	40	45	53	310.7231	563.09	58.3565399	-3.272052097	1.03E-78	3.18E-77	down	Molecular chaperone (DnaJ superfamily)
Solyc10g083880.2	1031	685	918	4526	3782	6103	3408.909	683.309	6134.50939	3.166088469	1.40E-78	4.32E-77	up	Aquaporin (major intrinsic protein family)
Solyc08g008490.4	2100	2426	2909	5338	8946	7305	5476.406	1927.12	9025.69442	2.227701901	1.64E-78	5.04E-77	up	WD40 repeat-containing protein
Solyc11g013110.2	9034	11175	12963	22508	28722	31682	21750.71	8603.47	34897.9472	2.020154272	2.13E-78	6.56E-77	up	Iron/ascorbate family oxidoreductases
Solyc02g088630.4	1011	1073	1392	98	124	105	518.8598	899.217	138.502414	-2.70183098	2.73E-78	8.35E-77	down	--
Solyc02g150143.1	1073	828	1013	47	86	62	419.1941	757.017	81.3708644	-3.208584646	1.32E-77	4.03E-76	down	DNA mismatch repair protein - MLH3 family
Solyc03g117980.3	224	140	278	1056	1215	1047	788.2459	164.68	1411.81126	3.095274384	1.51E-77	4.60E-76	up	Ferric reductase, NADH/NADPH oxidase
Solyc05g015980.3	1445	1718	1986	167	225	220	796.7614	1335.92	257.603929	-2.373251515	2.18E-77	6.58E-76	down	--
Solyc06g076030.3	30	20	26	227	337	340	199.1183	19.7325	378.504117	4.263369185	2.36E-77	7.13E-76	up	--
Solyc06g073970.3	1765	1918	1956	236	300	316	914.3044	1469.55	359.055646	-2.031952577	4.37E-77	1.32E-75	down	--
Solyc01g095140.4	2638	3306	3165	250	209	303	1351.031	2376.67	325.393137	-2.872205313	8.63E-77	2.58E-75	down	--
Solyc06g065560.2	245	230	300	592	795	715	543.5702	200.638	886.501941	2.14209988	3.03E-76	8.97E-75	up	--
Solyc11g066820.2	2653	2322	3598	220	342	367	1298.154	2208.65	387.659676	-2.506744313	3.54E-76	1.05E-74	down	--
Solyc11g066720.3	1467	1436	1506	185	227	198	703.6765	1148.74	258.613751	-2.153660437	3.85E-76	1.14E-74	down	dTDP-glucose 4-6-dehydratase
novel.1323	11	8	10	243	357	336	200.0951	7.5326	392.657519	5.705711622	4.05E-76	1.19E-74	up	--
Solyc03g114450.3	292	236	281	630	904	959	627.3139	210.261	1044.36702	2.314031915	4.09E-76	1.20E-74	up	--
Solyc01g059965.1	8	2	13	410	366	438	262.5843	5.77372	519.394872	6.446775933	5.45E-76	1.60E-74	up	--
Solyc12g010440.3	340	480	476	1184	1759	1607	1123.236	337.58	1908.89134	2.500692519	5.76E-76	1.69E-74	up	Permease of the major facilitator superfamily
Solyc06g084290.3	1805	1829	2214	243	282	345	941.828	1516.67	366.98597	-2.046950259	6.45E-76	1.89E-74	down	--
Solyc01g096320.3	662	720	853	1707	2258	1782	1505.961	579.756	2432.16567	2.068239094	6.77E-76	1.98E-74	up	Transcription factor HEX
Solyc07g065090.2	135	109	133	551	553	548	401.8417	97.9131	705.770266	2.847161458	7.37E-76	2.15E-74	up	--
novel.283	2147	2338	3630	170	244	265	1185.413	2086.53	284.299483	-2.873151372	8.43E-76	2.46E-74	down	Fatty acyl-CoA elongase
Solyc01g111970.4	964	934	1129	74	129	101	456.1444	785.331	126.957977	-2.620930579	2.32E-75	6.71E-74	down	Multicopper oxidases
Solyc01g105410.4	1308	1233	1515	125	118	174	614.4158	1051.97	176.858945	-2.575568812	4.06E-75	1.17E-73	down	--
Solyc12g013680.3	8	22	22	304	394	298	217.9123	13.5047	422.319839	4.966198575	5.35E-75	1.54E-73	up	FOG: Leucine rich repeat
Solyc04g077440.4	1445	1498	1817	193	269	229	762.7204	1234.22	291.216978	-2.082827564	5.89E-75	1.69E-73	down	Squalene monooxygenase
Solyc06g150108.1	31	35	44	333	449	309	245.4864	28.4725	462.50025	4.018230719	9.62E-75	2.75E-73	up	--
Solyc07g051880.1	1948	2563	2946	202	255	296	1125.314	1933.9	316.731115	-2.608953084	1.08E-74	3.08E-73	down	MEKK and related serine/threonine protein kinases
Solyc03g093130.3	1257	1088	1433	108	145	177	579.2716	978.411	180.132271	-2.436720563	1.69E-74	4.82E-73	down	--
novel.1374	812	816	977	67	89	62	384.1695	675.873	92.4658757	-2.874244455	2.20E-74	6.27E-73	down	--
Solyc06g050520.3	128	111	118	387	473	496	332.8375	93.0468	572.628218	2.623710235	2.32E-74	6.60E-73	up	--
Solyc08g068850.3	4	8	3	539	465	595	344.0596	4.01058	684.108636	7.450597776	2.62E-74	7.43E-73	up	--
Solyc12g099930.2	120	50	70	626	746	853	500.6622	62.4562	938.868237	3.910641686	2.71E-74	7.67E-73	up	Alanine-glyoxylate aminotransferase AGT1
novel.378	1117	1058	1299	131	132	159	540.1864	901.013	179.359504	-2.333914469	2.82E-74	7.96E-73	down	Tam3-transposase (Ac family)
Solyc03g083360.3	135	122	124	405	660	662	409.7591	99.4052	720.113048	2.86151965	3.02E-74	8.50E-73	up	--
novel.883	737	589	669	18	19	30	273.7326	519.259	28.2060906	-4.199325177	4.62E-74	1.29E-72	down	--
Solyc04g078750.3	2049	1911	2395	281	255	272	996.9054	1647.37	346.443757	-2.256593541	7.98E-74	2.23E-72	down	--

Solyc06g150132.1	826	1189	1564	36	28	48	485.6295	923.491	47.767593	-4.28113013	1.09E-73	3.03E-72	down	Calmodulin and related proteins (EF-Hand superfamily)
Solyc04g025650.3	36	21	40	296	305	337	212.0115	24.9342	399.088924	3.989093234	1.11E-73	3.08E-72	up	Kynurenine 3-monooxygenase
Solyc06g008960.3	1052	1045	1217	124	169	159	525.4687	860.631	190.306521	-2.17529188	1.49E-73	4.15E-72	down	--
Solyc05g005290.4	2739	3242	2641	6555	9460	9555	6490.489	2260.9	10720.076	2.245880659	2.56E-73	7.09E-72	up	--
Solyc01g095510.3	246	274	316	599	849	826	585.9703	217.008	954.932727	2.138629823	4.20E-73	1.16E-71	up	Mitochondrial carrier protein CGI-69
Solyc03g007370.3	1241	1893	1943	5171	8612	7206	5046.982	1320.49	8773.47265	2.732303767	5.07E-73	1.40E-71	up	--
novel.1546	1063	749	1096	57	42	45	407.2459	752.032	62.45947	-3.611989039	6.14E-73	1.69E-71	down	--
Solyc03g096050.3	183	304	365	1179	1623	1571	1029.751	220.341	1839.16185	3.06059722	8.25E-73	2.26E-71	up	Iron/ascorbate family oxidoreductases
Solyc01g005690.3	930	1151	1300	65	84	110	493.079	877.645	108.512569	-3.010683479	9.62E-73	2.63E-71	down	--
Solyc08g078960.4	122	128	155	466	509	505	367.218	105.01	629.426416	2.580577162	1.11E-72	3.03E-71	up	Oxysterol-binding protein
Solyc05g051660.1	243	250	275	576	928	862	594.1499	199.75	988.550007	2.309669996	1.22E-72	3.34E-71	up	Arylacetamide deacetylase
Solyc07g007930.3	8559	6652	7747	596	1127	932	3538.063	5972.4	1103.72839	-2.433928517	1.99E-72	5.39E-71	down	--
Solyc06g082160.4	669	600	784	44	42	49	294.624	531.658	57.5900775	-3.217651768	2.14E-72	5.81E-71	down	Predicted integral membrane protein
Solyc09g007500.3	1631	1627	1845	221	198	228	801.6586	1326.27	277.044649	-2.266633013	3.89E-72	1.05E-70	down	--
Solyc09g082690.3	23650	33125	36980	75827	100964	96072	69658.44	24328.9	114988.013	2.240743382	4.50E-72	1.21E-70	up	--
Solyc12g038910.1	4	5	3	681	568	680	415.7616	3.17211	828.35108	8.050324984	4.70E-72	1.26E-70	up	--
Solyc04g082480.3	180	178	194	507	809	636	481.0845	143.646	818.522982	2.51272429	5.50E-72	1.48E-70	up	--
Solyc01g112105.1	1132	922	1217	116	135	128	504.1469	847.472	160.821354	-2.402019372	6.30E-72	1.69E-70	down	--
Solyc08g077790.3	1060	1242	1194	111	124	116	530.8228	912.34	149.305573	-2.616089741	9.79E-72	2.61E-70	down	--
Solyc07g065860.3	1304	1262	1367	164	226	206	637.386	1023.81	250.966644	-2.026537223	1.12E-71	2.98E-70	down	--
Solyc10g055800.2	301	201	560	21152	21538	24059	14339.33	269.244	28409.4242	6.720337632	1.13E-71	3.00E-70	up	Predicted chitinase
Solyc03g078630.3	863	1107	1144	2676	5178	4522	2972.762	810.463	5135.06091	2.664189916	1.27E-71	3.37E-70	up	Ubiquitin and ubiquitin-like proteins
Solyc10g076480.2	68	65	79	308	368	348	244.4741	54.9977	433.950516	2.977915514	1.55E-71	4.12E-70	up	Ammonia permease
Solyc12g019410.3	187	231	246	547	725	732	508.149	172.687	843.610942	2.289749967	2.54E-71	6.73E-70	up	Tyrosine kinase specific for activated (GTP-bound) p21cdc42Hs
Solyc07g056510.3	3390	2391	3047	11501	9585	13985	8634.094	2292.32	14975.8701	2.707653545	3.23E-71	8.54E-70	up	Glutathione S-transferase
Solyc07g047850.3	46	33	34	238	377	337	213.9398	29.5129	398.366818	3.761476764	4.02E-71	1.06E-69	up	--
Solyc03g032040.3	1320	1294	1504	97	184	179	630.1259	1069.59	190.664824	-2.477151407	6.63E-71	1.75E-69	down	Predicted transporter (major facilitator superfamily)
Solyc11g006740.3	761	606	739	3484	2536	3697	2363.497	547.03	4179.96439	2.933324247	7.26E-71	1.91E-69	up	--
Solyc02g094400.4	3594	3374	4034	11338	10700	9612	8227.824	2855.98	13599.664	2.251267736	1.71E-70	4.49E-69	up	Predicted starch-binding protein
Solyc03g034320.3	104	62	81	474	743	1046	501.3973	64.1834	938.611149	3.871626232	2.36E-70	6.17E-69	up	--
Solyc10g005030.4	39	43	32	246	359	416	228.274	29.9753	426.572652	3.84322138	2.94E-70	7.66E-69	up	GATA-4/5/6 transcription factors
Solyc10g055680.1	79	75	82	368	663	822	413.8186	61.4239	766.213217	3.64410054	3.31E-70	8.60E-69	up	SAM-dependent methyltransferases
Solyc06g072845.1	18354	13913	20719	2442	2893	2407	8488.803	13685.9	3291.67285	-2.056307064	3.31E-70	8.60E-69	down	--
Solyc08g076790.3	392	478	537	1213	2230	2244	1362.087	365.326	2358.84673	2.691754392	6.46E-70	1.67E-68	up	Flavonol reductase/cinnamoyl-CoA reductase
Solyc07g043130.3	7941	11208	11914	24883	38164	32890	24153.9	8073.22	40234.5846	2.317256893	7.01E-70	1.81E-68	up	--
Solyc06g074750.1	654	697	716	5	9	10	274.2784	538.612	9.94509645	-5.734459987	1.14E-69	2.94E-68	down	Histone H2B
Solyc08g068390.3	331	262	385	2097	1548	1665	1277.661	252.615	2302.7064	3.186297329	1.34E-69	3.45E-68	up	Hydroxyacyl-CoA dehydrogenase/enoyl-CoA hydratase
Solyc03g025380.3	283	332	378	767	882	982	685.3322	257.782	1112.88211	2.109562786	1.49E-69	3.83E-68	up	--
Solyc07g065980.3	8934	10993	12119	825	1568	1156	4901.576	8325.28	1477.87206	-2.492763977	1.50E-69	3.84E-68	down	--
Solyc10g085240.1	281	329	357	1143	1264	1006	854.6423	251.417	1457.86737	2.534860296	1.58E-69	4.03E-68	up	UDP-glucuronosyl and UDP-glucosyl transferase
Solyc09g092390.2	2547	3096	3170	380	451	503	1429.189	2295.03	563.348637	-2.026445436	1.58E-69	4.03E-68	down	S-adenosylhomocysteine hydrolase
Solyc06g075610.1	35	47	94	414	586	556	349.2943	44.783	653.805626	3.856573164	3.69E-69	9.37E-68	up	Exocyst component protein and related proteins
novel.674	861	772	1003	59	100	100	395.2864	682.758	107.815122	-2.652074043	4.44E-69	1.12E-67	down	--
Solyc05g053620.3	214	307	300	847	983	973	701.1636	213.937	1188.39031	2.474380253	5.79E-69	1.47E-67	up	--
Solyc11g066740.2	1083	947	1055	113	125	112	476.0133	802.921	149.105567	-2.435523825	5.87E-69	1.48E-67	down	--
Solyc07g045160.3	161	167	155	430	593	624	408.8139	126.254	691.374263	2.45791885	6.75E-69	1.70E-67	up	Pyrophosphate-dependent phosphofructo-1-kinase
Solyc10g085020.3	3687	4572	4524	593	641	647	2066.065	3332.05	800.075836	-2.059872988	1.08E-68	2.70E-67	down	Beta tubulin
Solyc04g074830.1	366	438	504	974	1384	1146	907.7736	339.4	1476.1476	2.120818453	2.41E-68	6.01E-67	up	Clathrin assembly protein AP180
Solyc05g009520.2	194	225	280	573	737	814	537.2927	180.956	893.629418	2.303340323	2.81E-68	6.99E-67	up	--
Solyc02g082770.3	818	614	751	42	49	53	314.0736	567.239	60.9081436	-3.221231001	3.05E-68	7.59E-67	down	Putative phosphoinositide phosphatase
Solyc06g076490.3	229	217	255	618	794	637	525.1076	182.055	868.160056	2.252715778	3.54E-68	8.80E-67	up	--

Solyc10g084370.3	8	7	10	219	381	315	194.1255	6.45742	381.793538	5.881459231	4.60E-68	1.14E-66	up	Zuotin and related molecular chaperones (DnaJ superfamily)
novel.1498	619	512	792	21	13	22	259.9482	495.705	24.191731	-4.383267064	6.99E-68	1.72E-66	down	--
Solyc01g099960.4	71	87	99	306	402	422	271.0917	66.7005	475.482946	2.83410271	7.18E-68	1.77E-66	up	--
Solyc03g117340.1	37	67	37	1288	2149	2164	1185.097	37.3421	2332.85114	5.971500669	8.15E-68	2.00E-66	up	--
Solyc05g013960.3	1548	1905	2219	164	254	186	862.4522	1470.94	253.968163	-2.53252349	1.02E-67	2.50E-66	down	Oxysterol-binding protein
Solyc03g122190.3	221	227	258	526	865	823	553.5976	183.442	923.753146	2.334522644	1.69E-67	4.14E-66	up	--
Solyc10g050890.2	1251	1396	1415	162	210	211	652.1677	1058.62	245.715873	-2.105863795	3.37E-67	8.20E-66	down	Sulfite reductase (ferredoxin)
Solyc05g009120.3	426	596	495	1724	1730	2071	1373.233	397.331	2349.13452	2.564768938	8.74E-67	2.12E-65	up	--
Solyc01g081570.3	735	686	903	60	72	87	346.8833	601.511	92.2556018	-2.703814027	9.83E-67	2.38E-65	down	--
Solyc08g083110.4	92	153	181	589	1002	974	589.1874	110.226	1068.14925	3.276537967	1.67E-66	4.03E-65	up	Cystathionine beta-lyases/cystathionine gamma-synthases
Solyc12g005730.2	1418	1427	2214	142	195	184	760.4671	1301.67	219.262991	-2.568888803	1.71E-66	4.11E-65	down	Galactosyltransferases
Solyc05g008780.4	3034	3940	4040	412	598	559	1762.874	2867.07	658.676895	-2.120498662	1.94E-66	4.67E-65	down	--
Solyc02g088560.4	1416	1589	1572	2938	4978	3952	3077.665	1193.68	4961.65346	2.056158185	3.63E-66	8.65E-65	up	K+-channel ERG and related proteins
Solyc02g088070.3	40	44	47	234	288	288	188.1992	34.0887	342.309618	3.329292172	4.36E-66	1.04E-64	up	--
Solyc01g096620.4	94	120	138	384	618	512	362.6431	91.3026	633.98354	2.796664442	8.71E-66	2.07E-64	up	--
Solyc08g081400.4	178	135	174	520	627	528	418.8275	126.339	711.315734	2.491218485	9.44E-66	2.24E-64	up	Transcription factor MEIS1
Solyc10g081430.2	42	57	62	270	506	399	265.4371	41.8213	489.052949	3.550373799	1.91E-65	4.51E-64	up	Acetylglucosaminyltransferase EXT1/exostosin 1
Solyc04g080873.1	346	284	363	787	1506	1343	883.1651	257.508	1508.82249	2.551823375	2.06E-65	4.86E-64	up	Cysteine proteinase Cathepsin L
Solyc09g098130.3	158	156	155	412	658	563	403.0141	122.384	683.64468	2.486064501	2.22E-65	5.21E-64	up	Apoptotic ATPase
novel.463	32	28	37	254	265	266	179.842	25.1159	334.568077	3.730053337	2.50E-65	5.87E-64	up	--
novel.2007	772	636	727	52	62	66	315.7788	555.477	76.080458	-2.869214189	2.84E-65	6.66E-64	down	FOG: Transposon-encoded proteins with TYA
Solyc10g055810.2	198	326	318	4431	3924	4219	2808.356	219.286	5397.42657	4.621570085	3.37E-65	7.89E-64	up	Predicted chitinase
Solyc08g083050.1	867	511	895	21	15	21	305.1418	585.708	24.5756614	-4.596664108	4.14E-65	9.67E-64	down	--
Solyc07g007700.3	619	621	695	47	60	63	287.362	503.091	71.632755	-2.810414543	4.33E-65	1.01E-63	down	Farnesyl cysteine-carboxyl methyltransferase
Solyc02g076700.1	52	41	42	248	476	374	245.8979	35.2435	456.552301	3.701646419	4.64E-65	1.08E-63	up	FOG: PPR repeat
novel.1090	1322	1461	1515	176	250	234	698.3809	1119.41	277.352631	-2.010114203	4.77E-65	1.11E-63	down	Histones H3 and H4
Solyc11g012260.3	884	1046	1277	99	106	97	479.7224	830.626	128.818391	-2.696188731	5.36E-65	1.25E-63	down	--
Solyc04g008650.4	305	223	274	659	819	967	618.6024	208.41	1028.79463	2.304085959	6.35E-65	1.47E-63	up	--
Solyc03g031440.4	1013	1132	1424	126	149	156	553.084	923.851	182.317192	-2.343014122	7.85E-65	1.82E-63	down	1,4-benzoquinone reductase-like
Solyc09g060110.4	41	35	52	231	288	282	185.7377	33.0281	338.44725	3.351015825	8.02E-65	1.85E-63	up	--
Solyc12g009050.3	327	225	311	1071	1004	1041	779.1251	223.607	1334.64323	2.575265214	8.85E-65	2.05E-63	up	CCAAT-binding factor, subunit B (HAP2)
Solyc05g050007.1	52	26	31	335	554	351	274.733	28.4341	521.031904	4.1989546	1.04E-64	2.40E-63	up	--
Solyc03g025260.1	36	45	54	255	286	306	197.0227	34.9727	359.072649	3.35665362	1.15E-64	2.64E-63	up	Predicted E3 ubiquitin ligase
Solyc01g080870.3	846	926	944	76	119	96	414.9292	707.783	122.075054	-2.530562236	1.47E-64	3.38E-63	down	H+/oligopeptide symporter
novel.164	935	675	783	45	52	64	345.4255	622.933	67.9177059	-3.196621123	4.30E-64	9.78E-63	down	--
novel.885	968	651	725	23	44	32	326.2009	611.179	41.2226306	-3.878169273	4.54E-64	1.03E-62	down	--
Solyc07g017520.4	576	610	708	43	57	56	278.707	491.705	65.7092436	-2.901836447	7.81E-64	1.77E-62	down	Subunit of cis-Golgi transport vesicle tethering complex
Solyc05g012850.3	538	514	672	21	7	16	232.8549	446.23	19.4796795	-4.573137973	9.43E-64	2.13E-62	down	--
Solyc12g009480.2	147	114	162	2896	2302	1978	1613.164	109.393	3116.93473	4.831505974	1.19E-63	2.67E-62	up	Protein involved in vacuolar polyphosphate accumulation
Solyc07g056410.3	404	298	417	852	1055	1302	819.3665	289.651	1349.08156	2.21909358	3.11E-63	6.95E-62	up	--
Solyc08g007130.4	4	6	7	253	854	701	370.0607	4.40326	735.718172	7.383482965	7.03E-63	1.55E-61	up	--
Solyc03g006330.4	2135	2351	3173	322	427	403	1231.95	1978.25	485.651369	-2.026176263	7.63E-63	1.69E-61	down	FOG: Leucine rich repeat
Solyc11g006010.3	24	14	14	215	233	271	159.1639	13.6092	304.71857	4.491636782	9.86E-63	2.17E-61	up	Predicted hydrolase
Solyc12g094540.1	697	639	842	46	23	34	304.5135	563.784	45.2434928	-3.674742025	1.14E-62	2.51E-61	down	Predicted 3'-5' exonuclease
Solyc08g066070.1	837	733	883	85	76	74	369.0108	636.942	101.079207	-2.670640043	1.64E-62	3.59E-61	down	Exocyst component protein and related proteins
Solyc08g082210.4	702	911	1085	38	72	68	386.3825	698.944	73.8211842	-3.23039316	2.10E-62	4.60E-61	down	--
Solyc01g095470.3	1283	1734	1828	3492	5244	4309	3370.664	1259.83	5481.49557	2.121588503	2.22E-62	4.84E-61	up	Uncharacterized enzymes
novel.124	527	464	522	19	23	15	208.9893	393.651	24.3276404	-4.02889759	2.93E-62	6.38E-61	down	--
Solyc10g005640.4	123	110	123	327	450	407	295.6551	92.6307	498.679563	2.430370054	6.34E-62	1.37E-60	up	Serine/threonine protein phosphatase
Solyc10g084690.2	466	619	631	14	14	21	233.7186	446.725	20.7118953	-4.432235219	7.21E-62	1.56E-60	down	ADP-ribosylation factor GTPase activator
Solyc10g006330.3	1452	1419	1397	2735	5100	4569	3133.305	1114.08	5152.53323	2.210305899	7.74E-62	1.67E-60	up	--

Solyc01g010220.3	118	96	119	443	415	512	335.2919	86.44	584.143706	2.754138861	9.09E-62	1.96E-60	up	FOG: Transposon-encoded proteins with TYA
Solyc07g008180.4	372	386	469	934	1755	1314	992.7425	318.131	1667.35433	2.390626275	9.61E-62	2.07E-60	up	--
Solyc04g080790.3	239	199	252	550	782	959	568.0383	178.963	957.11365	2.420050672	1.04E-61	2.25E-60	up	Transcription factor MEIS1
novel.1742	660	610	723	49	49	72	294.7256	517.553	71.898257	-2.849531692	1.96E-61	4.21E-60	down	--
novel.2063	769	565	817	1	6	11	281.7182	556.25	7.18626075	-6.201388813	3.31E-61	7.06E-60	down	--
Solyc08g078670.2	126	83	130	396	522	434	329.6061	87.5456	571.66668	2.703377237	3.41E-61	7.28E-60	up	Aspartyl protease
Solyc08g075230.1	11	27	24	272	311	245	184.6482	16.1737	353.122812	4.450891278	5.66E-61	1.20E-59	up	--
Solyc02g092230.3	733	728	904	84	85	77	359.1173	612.957	105.277491	-2.553050057	7.64E-61	1.62E-59	down	Predicted membrane proteins
Solyc08g067310.1	727	1002	1115	56	75	48	407.0787	738.145	76.0119325	-3.283988269	1.09E-60	2.30E-59	down	Serine/threonine protein kinase
Solyc09g059430.3	94	116	111	343	434	526	315.6098	83.7609	547.458586	2.712202434	1.43E-60	3.02E-59	up	--
Solyc11g013080.3	357	340	416	943	949	926	746.4379	288.686	1204.18963	2.058147135	1.54E-60	3.24E-59	up	Inner membrane protein translocase
Solyc11g013500.1	440	498	630	17	23	23	216.1365	405.773	26.4997422	-3.933492334	3.20E-60	6.73E-59	down	--
novel.444	17	7	13	274	251	240	169.1551	9.55824	328.752016	5.094711556	4.08E-60	8.55E-59	up	--
Solyc03g112700.1	103	118	135	357	405	444	301.5413	92.4169	510.665711	2.465017669	4.70E-60	9.83E-59	up	--
Solyc03g063730.4	550	280	490	1601	1869	1887	1305.096	340.712	2269.47997	2.734534048	5.36E-60	1.12E-58	up	Uncharacterized membrane protein
Solyc08g075550.4	5	8	7	287	246	307	182.4933	5.22747	359.759118	6.109911076	1.05E-59	2.18E-58	up	--
Solyc03g115870.3	1232	1155	1636	137	104	140	601.3552	1038.8	163.907912	-2.674197457	1.18E-59	2.45E-58	down	Thioredoxin
Solyc06g069420.3	1500	2108	2308	5906	6116	5714	4555.656	1536.12	7575.19693	2.301786711	2.39E-59	4.93E-58	up	--
Solyc08g013730.3	367	294	327	661	870	968	653.684	257.308	1050.06042	2.030630114	3.00E-59	6.19E-58	up	Aquaporin (major intrinsic protein family)
Solyc10g007570.4	30	31	27	227	317	228	174.7241	23.0448	326.403508	3.830962785	3.44E-59	7.08E-58	up	Iron/ascorbate family oxidoreductases
Solyc02g067750.4	20	13	33	214	234	289	164.2609	16.7892	311.732561	4.194491028	5.73E-59	1.18E-57	up	Predicted carbonic anhydrase
Solyc01g111340.4	879	1226	1185	2313	3539	3105	2306.637	857.72	3755.5531	2.131147778	6.95E-59	1.43E-57	up	--
Solyc04g079350.1	97	148	246	841	998	1043	672.2843	125.619	1218.94986	3.274398967	7.16E-59	1.47E-57	up	--
Solyc11g007840.3	476	536	565	4	23	19	214.4717	410.478	18.465674	-4.417210395	9.46E-59	1.93E-57	down	--
Solyc02g081150.1	30	29	36	198	245	319	172.2869	24.627	319.946835	3.699271087	1.35E-58	2.75E-57	up	--
Solyc03g114100.1	480	496	575	31	32	40	223.2094	402.738	43.6805124	-3.209732275	1.58E-58	3.20E-57	down	--
Solyc03g123630.4	168	88	149	565	955	671	511.3304	104.603	918.05767	3.132206186	2.06E-58	4.18E-57	up	--
Solyc04g072900.1	2020	2027	2930	293	352	403	1120.704	1799.38	442.027238	-2.025479151	2.08E-58	4.20E-57	down	--
novel.574	446	421	570	8	15	12	193.0664	371.569	14.563671	-4.656502472	2.21E-58	4.45E-57	down	FOG: PPR repeat
Solyc03g121620.1	960	986	1316	122	157	164	514.9541	843.293	186.61478	-2.175353376	2.64E-58	5.32E-57	down	--
Solyc06g073615.1	612	561	675	53	71	63	279.3024	479.707	78.8978087	-2.603974145	3.00E-58	6.03E-57	down	Molybdopterin converting factor subunit 2
Solyc07g047950.1	244	254	319	536	825	719	541.7931	211.601	871.984933	2.043294994	3.10E-58	6.23E-57	up	--
Solyc07g056580.3	1052	864	1219	101	138	92	475.4123	810.519	140.305281	-2.533559122	3.62E-58	7.26E-57	down	Sensory transduction histidine kinase
Solyc10g084125.1	19	22	23	164	235	251	144.468	16.6602	272.275761	4.0352407	4.02E-58	8.03E-57	up	FOG: Transposon-encoded proteins with TYA
Solyc11g066400.1	36	46	44	244	316	248	187.6177	32.873	342.362318	3.383264025	4.24E-58	8.47E-57	up	FOG: Zn-finger
Solyc06g083350.3	847	867	1059	116	118	134	437.744	718.919	156.568984	-2.205032051	1.04E-57	2.07E-56	down	Transcription factor of the Forkhead/HNF3 family
Solyc09g097870.4	1258	1309	1731	191	247	211	692.9355	1111.34	274.52922	-2.018945843	3.31E-57	6.52E-56	down	--
Solyc12g096370.2	452	456	515	26	30	22	201.5782	369.857	33.2990425	-3.485685439	4.35E-57	8.55E-56	down	Exocyst component protein and related proteins
novel.352	746	601	675	56	64	67	302.8341	526.428	79.2407052	-2.734990968	6.11E-57	1.20E-55	down	--
Solyc12g005660.2	8	10	1	197	278	265	158.0528	5.15465	310.950984	5.97094406	7.41E-57	1.45E-55	up	--
Solyc02g082140.3	2050	1615	2207	213	213	316	916.954	1520.18	313.732148	-2.277457018	1.30E-56	2.53E-55	down	Extracellular protein with conserved cysteines
Solyc09g075230.1	434	415	443	1081	1005	1178	865.3426	336.494	1394.19093	2.050002499	1.43E-56	2.78E-55	up	--
Solyc01g087020.2	819	1006	1413	48	97	84	464.7103	834.563	94.8573415	-3.126615472	2.49E-56	4.82E-55	down	--
Solyc08g080630.4	3207	2106	3636	30048	23990	31850	19583.17	2304.26	36862.0767	3.999651767	3.43E-56	6.61E-55	up	--
Solyc02g032200.4	791	683	952	89	95	84	370.8045	627.183	114.425591	-2.463810142	3.60E-56	6.93E-55	down	--
Solyc05g054620.4	1031	926	1139	114	158	186	497.4909	803.245	191.7371	-2.061489093	5.14E-56	9.87E-55	down	--
Solyc03g115850.3	46	23	50	237	276	284	184.0318	30.5246	337.539052	3.456117463	9.52E-56	1.82E-54	up	--
novel.1533	563	405	493	14	13	12	198.2859	379.81	16.7618777	-4.523102933	1.05E-55	2.01E-54	down	--
Solyc12g006230.3	1199	958	993	112	129	127	489.0568	822.012	156.102077	-2.399308764	1.33E-55	2.54E-54	down	FOG: Predicted E3 ubiquitin ligase
novel.372	55	56	89	564	387	517	343.3363	51.4135	635.259141	3.619878606	2.55E-55	4.85E-54	up	--
Solyc01g110110.3	26	23	25	176	442	394	217.3758	19.2721	415.479576	4.435658506	2.64E-55	5.02E-54	up	Cysteine proteinase Cathepsin F
Solyc04g077220.3	129	173	232	591	634	709	479.3219	137.763	820.881196	2.57182942	2.69E-55	5.12E-54	up	Transcription factor HEX
Solyc03g123620.4	391	454	528	13	13	19	187.6234	356.212	19.034682	-4.228748798	2.89E-55	5.50E-54	down	--
Solyc11g066040.2	703	727	877	85	111	91	359.8713	598.297	121.445654	-2.302892955	3.57E-55	6.76E-54	down	FOG: Armadillo/beta-catenin-like repeats
novel.1953	14	12	23	445	236	389	239.626	12.5391	466.712807	5.20344564	7.93E-55	1.49E-53	up	--

Solyc09g075280.2	1021	1093	1292	132	174	206	549.2429	883.668	214.818073	-2.036923856	1.09E-54	2.05E-53	down	Exocyst complex, subunit SEC15
Solyc09g056430.4	22	25	34	219	207	259	156.3672	20.9114	291.82301	3.795135798	2.33E-54	4.34E-53	up	Putative serine/threonine protein kinase
Solyc10g080840.1	386	527	502	1177	2481	2151	1385.562	369.103	2402.02016	2.703581852	2.49E-54	4.62E-53	up	Cytochrome P450 CYP4/CYP19/CYP26
Solyc01g107170.2	1602	2456	3225	7098	8850	9816	6366.827	1878.6	10855.0553	2.530463928	2.65E-54	4.91E-53	up	FOG: Zn-finger
Solyc12g019710.1	623	504	713	49	61	62	274.177	475.734	72.6195019	-2.712846325	2.66E-54	4.93E-53	down	--
Solyc10g085960.2	89	81	81	297	322	339	236.2587	65.5152	407.002257	2.636530883	2.97E-54	5.49E-53	up	Defense-related protein containing SCP domain
Solyc08g008250.3	890	849	927	121	117	128	425.0878	693.888	156.287154	-2.158427114	3.06E-54	5.66E-53	down	RNA polymerase II elongator associated protein
Solyc01g060130.3	760	767	839	102	106	110	375.5019	615.554	135.44985	-2.190873839	3.75E-54	6.91E-53	down	Vesicle coat complex COPII, GTPase subunit SAR1
novel.1221	458	423	508	15	33	32	196.7626	360.56	32.965003	-3.426064575	3.93E-54	7.22E-53	down	--
Solyc07g065970.1	427	415	495	9	6	11	179.0829	347.009	11.1565563	-4.980364044	4.07E-54	7.47E-53	down	Molecular chaperone (DnaJ superfamily)
Solyc02g078410.1	1037	1057	1178	154	187	145	528.7001	850.728	206.672581	-2.045923488	4.17E-54	7.66E-53	down	HGG motif-containing thioesterase
Solyc04g072800.4	143	199	209	494	848	676	493.1828	143.27	843.095171	2.558818809	4.79E-54	8.77E-53	up	Phosphoglycerate mutase
Solyc03g026110.4	219	188	224	437	751	730	481.1421	163.922	798.361902	2.286528545	6.31E-54	1.15E-52	up	--
Solyc03g095780.3	25	31	54	201	306	323	187.4678	28.1423	346.793239	3.613984374	6.43E-54	1.17E-52	up	--
Solyc06g076040.3	1085	1046	1230	130	99	148	517.2029	872.756	161.649792	-2.441495564	7.13E-54	1.30E-52	down	FOG: Armadillo/beta-catenin-like repeats
Solyc08g080750.4	118	124	161	358	422	410	304.2981	104.258	504.338003	2.270743761	7.50E-54	1.36E-52	up	--
Solyc08g007170.1	1242	1302	1380	125	203	230	626.8873	1021.63	232.141058	-2.130070751	7.61E-54	1.38E-52	down	Transcription factor GT-2 and related proteins
Solyc01g102770.1	45	37	39	186	312	263	174.7538	31.5552	317.952412	3.338769177	7.76E-54	1.41E-52	up	--
Solyc08g061250.3	21	13	25	166	206	211	130.6465	15.1511	246.141991	4.010255949	9.03E-54	1.63E-52	up	Serine/threonine protein kinase
Solyc01g109320.4	40	34	36	203	324	232	173.74	28.6768	318.803261	3.478847924	1.12E-53	2.03E-52	up	Uncharacterized membrane protein
Solyc02g069180.4	1632	1842	2151	187	332	312	902.5286	1459.43	345.627487	-2.072523534	1.65E-53	2.96E-52	down	Predicted E3 ubiquitin ligase
novel.1963	16	22	27	163	255	207	139.4925	16.8161	262.168907	3.960647394	2.45E-53	4.40E-52	up	--
novel.1657	488	365	510	6	12	17	183.5812	352.783	14.379747	-4.585098588	3.07E-53	5.51E-52	down	--
Solyc10g085140.2	482	517	575	43	43	46	232.7174	409.138	56.2967946	-2.871227572	4.16E-53	7.45E-52	down	Cis-prenyltransferase
Solyc08g045640.3	565	487	536	32	51	34	231.3488	413.489	49.2086843	-3.067338861	4.44E-53	7.95E-52	down	--
Solyc07g041555.1	1127	988	1153	63	49	114	472.4134	849.366	95.4608216	-3.153687248	4.47E-53	8.00E-52	down	--
Solyc05g054880.3	435	421	450	20	26	30	186.0144	340.102	31.9269666	-3.407626574	5.87E-53	1.05E-51	down	Putative u4/u6 small nuclear ribonucleoprotein
Solyc02g081190.4	393	352	389	825	1390	1717	962.7914	295.165	1630.41812	2.467284202	7.12E-53	1.27E-51	up	Iron/ascorbate family oxidoreductases
Solyc01g099200.3	123	55	112	394	595	630	375.6514	74.6418	676.660954	3.177481857	7.15E-53	1.27E-51	up	--
Solyc03g031650.4	154	174	149	434	491	500	364.7388	124.926	604.551677	2.277728546	8.15E-53	1.45E-51	up	Microtubule-associated anchor protein
Solyc08g081310.4	801	764	987	97	113	80	392.3152	660.801	123.829183	-2.424320849	8.72E-53	1.55E-51	down	--
novel.990	497	459	537	31	39	51	219.3128	387.865	50.7602437	-2.927842763	1.93E-52	3.41E-51	down	--
Solyc02g062130.4	715	530	688	40	48	69	283.6549	501.455	65.8551636	-2.923870962	3.34E-52	5.88E-51	down	NADP/FAD dependent oxidoreductase
Solyc03g005170.3	966	1081	1213	119	194	150	520.4699	846.931	194.008762	-2.12191348	5.18E-52	9.09E-51	down	--
Solyc07g032170.3	873	750	866	103	96	118	391.2053	647.197	135.213238	-2.266252952	6.45E-52	1.13E-50	down	Predicted hydrolase/acyltransferase
Solyc04g074350.3	465	413	513	13	36	29	196.3832	360.812	31.9549481	-3.466771164	8.05E-52	1.40E-50	down	UDP-glucuronosyl and UDP-glucosyl transferase
Solyc03g115730.3	490	456	555	40	48	50	223.8959	389.453	58.3390589	-2.74074912	9.18E-52	1.59E-50	down	--
Solyc08g013900.3	63	55	55	226	307	257	189.3242	45.1667	333.481596	2.888055481	1.09E-51	1.88E-50	up	--
Solyc02g088190.4	423	427	482	19	37	24	189.7769	346.209	33.344505	-3.36305332	1.94E-51	3.34E-50	down	Transcription factor, Myb superfamily
Solyc10g083460.1	153	156	197	424	518	699	409.9417	131.05	688.833566	2.393908173	2.15E-51	3.70E-50	up	Predicted Zn-finger protein
Solyc09g005570.3	541	614	844	51	49	57	291.4265	515.896	66.957064	-2.95465026	2.62E-51	4.48E-50	down	--
Solyc02g092240.3	91	68	87	244	320	338	221.6819	63.8398	379.523949	2.572001191	2.96E-51	5.06E-50	up	--
novel.1299	669	571	718	75	78	75	302.5709	507.85	97.2914373	-2.393309793	3.46E-51	5.92E-50	down	--
Solyc01g079660.2	488	422	568	29	31	46	213.5921	382.513	44.6716878	-3.097219317	3.58E-51	6.11E-50	down	--
Solyc09g005100.4	673	660	867	84	100	105	345.7177	569.235	122.200218	-2.221876306	5.33E-51	9.00E-50	down	Polynucleotide kinase 3' phosphatase
Solyc09g015040.1	2337	2575	3092	344	360	516	1295.159	2075.16	515.156976	-2.010311556	5.98E-51	1.01E-49	down	--
Solyc08g014290.1	631	611	772	64	77	98	311.1698	521.799	100.540966	-2.373474386	7.04E-51	1.19E-49	down	Serine/threonine specific protein phosphatase PP1
novel.1450	203	197	206	517	509	611	427.2903	157.912	696.669003	2.141012789	7.44E-51	1.25E-49	up	--
Solyc02g082633.1	457	433	578	37	29	33	211.2319	379.744	42.7198705	-3.174072729	8.29E-51	1.39E-49	down	--
Solyc11g069600.2	168	188	209	372	528	506	368.7016	146.827	590.576419	2.009767078	1.01E-50	1.70E-49	up	--
Solyc12g096710.2	648	606	889	63	67	86	322.0731	552.746	91.4000909	-2.599162262	1.10E-50	1.84E-49	down	--
Solyc08g066790.4	696	807	898	79	75	107	367.2808	623.796	110.766015	-2.497265218	1.13E-50	1.89E-49	down	Phospholipase D1
Solyc02g089510.3	382	379	480	5	20	13	168.4314	321.444	15.4191819	-4.335795486	1.53E-50	2.55E-49	down	GATA-4/5/6 transcription factors
Solyc09g018280.1	689	901	1277	2206	2935	2829	2048.118	738.38	3357.85558	2.184414642	2.40E-50	3.99E-49	up	Serine/threonine protein kinase
Solyc11g032190.1	465	347	431	13	11	16	169.9624	322.856	17.0684137	-4.254215601	4.65E-50	7.67E-49	down	--

Solyc09g064730.3	90	101	132	399	398	382	293.8796	83.5039	504.255224	2.589101627	6.44E-50	1.06E-48	up	Copper chaperone
novel.381	12	4	16	183	203	181	124.8247	8.10737	241.542063	4.866860371	7.01E-50	1.15E-48	up	--
Solyc01g067830.3	609	572	655	56	62	86	281.6023	477.227	85.977295	-2.471312697	1.14E-49	1.85E-48	down	U1 snRNP-specific protein C
Solyc04g080450.1	832	779	953	121	114	137	411.9024	665.127	158.678142	-2.075029828	1.16E-49	1.89E-48	down	--
Solyc04g074410.2	875	707	1095	98	116	123	416.3486	690.192	142.504875	-2.278143265	1.43E-49	2.33E-48	down	--
Solyc06g076450.3	634	778	765	63	86	94	334.7868	567.603	101.970158	-2.472020273	2.97E-49	4.80E-48	down	GTPase Rab11/YPT3, small G protein superfamily
Solyc09g008440.1	342	442	514	10	16	9	175.6452	336.531	14.7592489	-4.510390394	5.08E-49	8.18E-48	down	FOG: Zn-finger
Solyc03g113370.3	594	583	658	1079	2011	1505	1195.844	477.037	1914.65048	2.006095535	5.25E-49	8.44E-48	up	--
Solyc06g008690.1	136	105	126	289	453	457	298.0821	95.395	500.769154	2.395059595	5.98E-49	9.59E-48	up	FOG: Kelch repeat
Solyc11g020960.2	519	412	543	37	41	46	217.2658	381.992	52.539558	-2.866169944	6.17E-49	9.88E-48	down	--
Solyc08g005150.3	682	583	643	63	75	53	289.1363	496.809	81.4638634	-2.616499306	6.29E-49	1.00E-47	down	Predicted E3 ubiquitin ligase
Solyc04g063240.3	187	151	185	355	534	473	353.6195	135.815	571.423798	2.074411561	6.38E-49	1.02E-47	up	--
novel.805	8	10	15	127	245	247	132.3569	8.48545	256.228381	4.909872727	8.05E-49	1.28E-47	up	--
Solyc08g077020.1	152	189	211	411	544	485	375.5995	143.338	607.860455	2.084269929	8.93E-49	1.42E-47	up	--
Solyc01g058720.4	2	4	11	179	208	211	128.8194	4.26548	253.373236	5.847585026	1.09E-48	1.72E-47	up	Calmodulin and related proteins (EF-Hand superfamily)
Solyc10g078530.2	133	129	180	336	601	556	367.548	114.154	620.941606	2.443739125	1.28E-48	2.03E-47	up	Ca2+-independent phospholipase A2
novel.1456	3	4	3	260	298	276	178.4955	2.62739	354.36364	7.08948055	1.95E-48	3.07E-47	up	--
Solyc04g076960.4	173	142	158	338	524	461	338.7526	123.163	554.342332	2.173272082	2.68E-48	4.20E-47	up	Sucrose transporter and related proteins
Solyc04g049680.2	402	448	479	32	27	31	192.2313	345.795	38.667752	-3.178037088	3.83E-48	5.99E-47	down	GATA-4/5/6 transcription factors
Solyc02g150146.1	684	711	904	92	104	81	356.7224	595.209	118.235516	-2.340104338	3.84E-48	5.99E-47	down	--
Solyc10g076930.2	431	381	434	25	31	34	180.9972	324.055	37.9395265	-3.093020708	7.91E-48	1.23E-46	down	--
Solyc05g054210.3	268	284	295	605	769	1063	620.5625	220.642	1020.48337	2.211347041	9.13E-48	1.41E-46	up	--
Solyc11g008590.2	364	387	429	11	27	22	165.7285	306.772	24.6854659	-3.607588769	1.41E-47	2.17E-46	down	Predicted UDP-galactose transporter
Solyc12g088390.1	29	53	72	254	347	306	210.993	39.6344	382.351567	3.264855057	1.56E-47	2.40E-46	up	FOG: Zn-finger
Solyc09g005730.4	381	492	579	26	33	30	206.9735	376.314	37.6329905	-3.323921903	1.62E-47	2.48E-46	down	--
Solyc08g081710.3	6	5	9	161	198	173	115.3202	5.13006	225.510287	5.441327157	1.64E-47	2.51E-46	up	--
Solyc09g061410.1	54	56	61	187	270	302	180.9967	44.4866	317.50668	2.839246543	1.84E-47	2.81E-46	up	--
Solyc08g065140.1	38	46	60	214	279	225	170.5822	37.2101	303.954241	3.025538374	1.98E-47	3.02E-46	up	--
Solyc01g094700.4	12	8	14	156	177	292	135.334	8.74949	261.918496	4.895701143	2.30E-47	3.51E-46	up	--
Solyc10g085310.1	44	40	64	192	273	237	166.7603	38.0763	295.444397	2.949558031	2.85E-47	4.33E-46	up	--
Solyc08g007470.2	541	503	611	52	75	53	252.7217	429.439	76.004707	-2.498975881	2.97E-47	4.51E-46	down	--
Solyc06g082070.3	86	103	88	255	416	367	253.2027	72.5337	433.871771	2.587523461	3.16E-47	4.80E-46	up	--
novel.1231	497	337	462	4	8	10	172.4916	335.924	9.05905205	-5.182168126	4.02E-47	6.09E-46	down	--
Solyc07g006630.4	23	34	19	164	214	229	137.7557	20.1233	255.387997	3.683499517	4.03E-47	6.10E-46	up	GATA-4/5/6 transcription factors
Solyc02g080640.4	878	725	882	1532	3108	3233	1947.973	645.343	3250.60234	2.333287609	4.28E-47	6.46E-46	up	Phosphoadenosine phosphosulfate reductase
Solyc06g064940.3	3236	3732	4772	357	679	637	1865.238	3036.67	693.807055	-2.126936161	4.45E-47	6.73E-46	down	Phosphatidylinositol transfer protein
Solyc01g099990.3	371	310	470	739	1390	1373	874.2593	296.862	1451.65638	2.2899064	5.44E-47	8.20E-46	up	--
Solyc09g010980.1	454	397	472	36	28	31	192.3552	343.668	41.0426572	-3.088583308	5.77E-47	8.69E-46	down	--
Solyc06g076780.3	667	690	853	80	115	121	352.5442	572.698	132.390783	-2.10793235	7.29E-47	1.09E-45	down	--
Solyc08g065190.3	920	1148	1261	1878	3357	3272	2199.835	864.876	3534.79502	2.031740891	8.25E-47	1.24E-45	up	Fe2+/Zn2+ regulated transporter
Solyc10g083230.1	483	417	568	40	42	28	213.5433	379.789	47.297656	-3.022965136	8.49E-47	1.27E-45	down	--
Solyc02g088390.4	1567	1608	2230	215	148	192	817.9613	1395.58	240.338014	-2.54622956	1.96E-46	2.91E-45	down	--
Solyc04g082070.4	580	583	675	75	71	84	287.7459	477.368	98.1233952	-2.291106942	2.32E-46	3.44E-45	down	Pseudouridylate synthase
Solyc01g080900.4	82	84	96	249	284	334	217.2288	68.0658	366.391778	2.428224052	2.48E-46	3.67E-45	up	Cytochrome P450 CYP4/CYP19/CYP26
Solyc02g086960.2	5	0	3	412	335	352	238.1623	2.0399	474.284701	7.833241326	2.64E-46	3.90E-45	up	--
novel.2062	645	456	596	10	43	27	236.3607	440.318	32.4032205	-3.729339259	2.81E-46	4.14E-45	down	--
Solyc03g080120.1	47	50	87	282	353	275	216.7309	47.1389	386.323028	3.025495112	3.41E-46	5.01E-45	up	--
Solyc10g052490.2	384	567	559	1098	1892	1500	1134.517	393.313	1875.72087	2.254812941	4.12E-46	6.03E-45	up	--
novel.1142	481	407	538	28	52	32	208.0741	369.326	46.822125	-2.971477169	4.18E-46	6.11E-45	down	--
Solyc01g099660.4	550	369	372	3813	3361	4883	2735.734	337.513	5133.95587	3.927350822	4.30E-46	6.28E-45	up	Molecular chaperones GRP78/BiP/KAR2
Solyc10g018350.2	671	620	761	90	100	79	323.5994	532.306	114.892743	-2.221145411	5.65E-46	8.25E-45	down	VAMP-associated protein involved in inositol metabolism
Solyc09g082250.3	83	79	64	280	292	367	228.6347	59.3203	397.94902	2.751228252	8.58E-46	1.25E-44	up	--
novel.2017	465	359	435	25	32	30	181.9544	327.162	36.7469461	-3.155083943	9.36E-46	1.36E-44	down	--

Solyc08g079040.1	478	493	603	48	64	66	241.4529	408.031	74.8748251	-2.443580183	9.78E-46	1.42E-44	down	Acetylglucosaminyltransferase EXT1/exostosin 1
Solyc04g016180.3	3	1	8	157	349	323	172.3473	2.9785	341.716184	6.788522083	1.03E-45	1.49E-44	up	Uncharacterized conserved protein
Solyc10g009190.1	21	16	29	161	170	223	125.6588	16.9412	234.376301	3.778800623	1.06E-45	1.53E-44	up	--
Solyc06g075960.1	550	462	597	56	53	63	245.2035	417.036	73.3710353	-2.516383574	1.14E-45	1.65E-44	down	Histone H4
Solyc06g063370.3	883	556	604	1609	2263	2680	1637.004	533.295	2740.71318	2.362340134	1.15E-45	1.67E-44	up	--
Solyc07g042580.3	3	3	5	190	284	225	148.4079	2.82373	293.992141	6.687363945	1.19E-45	1.71E-44	up	--
Solyc10g086760.2	852	1100	1090	128	125	119	476.0301	792.741	159.318962	-2.322434043	1.53E-45	2.20E-44	down	Beta tubulin
Solyc07g055020.3	475	494	468	36	50	45	215.2759	375.396	55.1555037	-2.763719003	2.27E-45	3.27E-44	down	--
Solyc10g079930.1	128	149	180	854	562	722	523.4514	118.418	928.484725	2.968366505	2.87E-45	4.13E-44	up	UDP-glucuronosyl and UDP-glucosyl transferase
Solyc06g051010.1	324	438	426	15	10	13	163.0935	309.703	16.4844768	-4.261704142	4.29E-45	6.16E-44	down	--
Solyc02g082920.4	39	16	32	321	238	269	190.4559	22.4291	358.482605	3.989910269	4.38E-45	6.29E-44	up	Predicted chitinase
Solyc05g055340.4	519	519	619	55	50	73	252.82	429.979	75.6613206	-2.512626224	4.74E-45	6.80E-44	down	--
Solyc11g006730.1	26	16	11	159	311	207	147.9975	13.9849	282.010177	4.34910241	4.92E-45	7.04E-44	up	--
Solyc06g071620.3	158	159	205	337	564	514	362.76	135.118	590.40232	2.128614729	6.90E-45	9.85E-44	up	RNA helicase BRR2, DEAD-box superfamily
Solyc04g071780.3	119	89	235	583	818	797	517.8923	112.347	923.437705	3.032930632	7.95E-45	1.13E-43	up	Cytochrome P450 CYP2 subfamily
novel.1379	11	13	3	158	164	211	116.5333	7.26464	225.801882	4.995989645	8.76E-45	1.25E-43	up	--
Solyc10g006010.3	571	703	762	68	95	85	316.8088	529.218	104.39915	-2.339798207	1.12E-44	1.59E-43	down	Tandem pore domain K+ channel
Solyc01g109460.3	697	732	1022	93	103	79	375.0757	632.601	117.550877	-2.436582524	1.24E-44	1.76E-43	down	Predicted transporter (major facilitator superfamily)
Solyc03g111860.1	4	4	4	155	239	227	131.5024	3.13054	259.874182	6.383539774	1.59E-44	2.24E-43	up	--
Solyc09g083390.3	236	318	341	584	973	846	618.1654	232.601	1003.73004	2.111035644	2.06E-44	2.90E-43	up	--
novel.1539	679	486	417	22	25	20	221.8539	415.134	28.5739385	-3.869201302	2.39E-44	3.37E-43	down	--
Solyc07g063600.3	4	1	6	227	260	202	148.335	2.7679	293.902137	6.6863842	2.47E-44	3.48E-43	up	--
Solyc01g109760.3	396	420	403	17	24	35	174.9664	318.296	31.6365132	-3.316075192	2.51E-44	3.53E-43	down	--
Solyc10g006510.3	340	367	406	19	28	26	159.9861	289.344	30.6278568	-3.233483169	2.59E-44	3.64E-43	down	--
Solyc02g082930.3	246	142	299	903	828	878	647.1301	176.071	1118.18944	2.66278788	3.50E-44	4.89E-43	up	Predicted chitinase
Solyc01g095390.3	88	95	144	278	393	425	271.7087	84.1515	459.265848	2.445244436	5.24E-44	7.32E-43	up	--
Solyc11g006900.1	323	332	371	15	21	19	144.9358	266.726	23.1453156	-3.523178313	5.76E-44	8.03E-43	down	--
Solyc10g081970.2	52	66	88	387	358	300	251.7232	53.1748	450.271557	3.07592353	6.74E-44	9.37E-43	up	--
Solyc01g098530.3	341	306	440	17	10	12	148.8656	280.65	17.0814662	-4.081106464	8.20E-44	1.13E-42	down	CTP synthase (UTP-ammonia lyase)
Solyc05g010516.1	30	40	60	532	1305	1092	619.0239	33.4114	1204.63637	5.169598465	1.02E-43	1.40E-42	up	--
Solyc11g067080.3	230	184	189	396	631	599	418.4083	157.395	679.421678	2.113679945	1.15E-43	1.58E-42	up	Putative serine/threonine protein kinase
Solyc08g016801.1	445	431	538	20	24	49	202.5746	366.486	38.6635565	-3.231349097	1.15E-43	1.59E-42	down	WD40 repeat protein
Solyc07g041970.4	867	649	1002	99	95	116	390.8906	649.734	132.047164	-2.305080319	1.27E-43	1.75E-42	down	--
Solyc07g054470.1	119	83	152	500	413	505	349.9033	90.9231	608.883456	2.736880096	1.78E-43	2.45E-42	up	--
Solyc04g079590.3	649	649	704	52	108	83	310.8746	521.015	100.734237	-2.357731043	1.85E-43	2.53E-42	down	--
Solyc11g012680.3	36	30	32	141	227	258	143.0292	25.5463	260.512193	3.356634075	1.95E-43	2.67E-42	up	--
Solyc02g064690.3	740	609	728	56	106	100	324.1732	539.682	108.664806	-2.299989484	2.42E-43	3.30E-42	down	--
Solyc06g063020.4	519	484	535	48	28	44	226.1763	400.212	52.1406986	-2.964008275	3.00E-43	4.08E-42	down	--
Solyc03g093120.5.1	490	511	577	61	55	63	243.3453	410.059	76.631985	-2.431637074	3.37E-43	4.57E-42	down	--
Solyc03g082520.1	12	12	17	137	147	164	100.3713	10.5812	190.16151	4.159181729	3.97E-43	5.38E-42	up	--
Solyc01g094170.3	725	566	803	68	102	108	328.8773	541.529	116.225895	-2.214123826	6.14E-43	8.28E-42	down	--
Solyc02g080890.3	166	212	238	433	624	762	459.7199	159.904	759.53626	2.249430613	6.83E-43	9.20E-42	up	--
Solyc09g092360.3	471	517	607	49	61	74	245.6001	413.834	77.3665154	-2.416308177	1.04E-42	1.39E-41	down	--
Solyc11g005150.3	985	1064	1177	149	127	187	518.0372	838.654	197.420246	-2.091774414	2.08E-42	2.77E-41	down	--
Solyc03g112300.3	429	437	555	48	43	50	214.162	367.963	60.3605672	-2.620808932	2.85E-42	3.78E-41	down	--
Solyc03g121400.1	547	627	725	62	76	98	295.9838	492.809	99.1586346	-2.310015589	5.33E-42	7.05E-41	down	--
Solyc02g081450.2	25	40	61	205	272	236	166.7169	32.3231	301.110785	3.211309655	5.34E-42	7.05E-41	up	--
Solyc08g007790.3	168	132	212	355	588	493	366.1358	131.889	600.38239	2.185020823	5.92E-42	7.81E-41	up	Hydroxymethylglutaryl-CoA synthase
Solyc01g044270.3	841	757	1017	131	111	129	417.9493	676.592	159.307063	-2.09707848	6.19E-42	8.15E-41	down	--
Solyc05g052610.3	533	545	564	63	48	50	248.8135	427.873	69.7536587	-2.636749026	6.60E-42	8.69E-41	down	Zuotin and related molecular chaperones (DnaJ superfamily)
Solyc03g078640.1	339	339	486	21	12	23	162.242	300.287	24.197558	-3.658061741	7.25E-42	9.53E-41	down	--
novel.1169	436	407	506	41	53	58	206.8631	349.777	63.9488088	-2.448936032	1.21E-41	1.59E-40	down	--
Solyc12g006120.3	294	352	408	5	19	14	144.4261	273.427	15.4250089	-4.104300495	1.63E-41	2.13E-40	down	CCAAT-binding factor, subunit A (HAP3)
Solyc11g013010.2	452	370	401	24	36	38	179.8367	318.699	40.9743647	-2.950029554	1.76E-41	2.29E-40	down	Junctional membrane complex protein Junctophilin

Solyc05g040050.3	334	366	375	21	28	28	156.2548	280.098	32.4115996	-3.108023158	2.20E-41	2.86E-40	down	--
Solyc03g098020.4	243	235	197	568	567	688	476.0229	177	775.045573	2.132478888	2.79E-41	3.63E-40	up	Lysophospholipase
Solyc08g016210.3	14	6	23	198	174	172	122.4927	10.8622	234.12314	4.400458088	2.84E-41	3.69E-40	up	FOG: Leucine rich repeat
Solyc05g008060.4	193	240	270	552	676	534	465.5862	182.504	748.668538	2.035152565	3.19E-41	4.14E-40	up	--
Solyc03g121140.4	362	362	458	32	30	38	174.3798	306.153	42.6061155	-2.854724909	4.15E-41	5.37E-40	down	--
Solyc11g061980.2	7	12	4	109	214	237	118.7095	6.16214	231.25689	5.267148097	4.63E-41	5.97E-40	up	UDP-glucuronosyl and UDP-glucosyl transferase
Solyc02g089490.4	330	337	392	26	18	19	151.2058	274.976	27.4351996	-3.357664308	5.43E-41	6.99E-40	down	--
Solyc03g033840.4	113	115	131	370	327	401	281.4927	93.2791	469.706228	2.329409958	5.95E-41	7.63E-40	up	AAA+-type ATPase
Solyc06g005230.3	645	687	834	97	104	129	350.6043	561.504	139.705005	-2.009277776	5.99E-41	7.69E-40	down	Serine/threonine protein kinase
Solyc09g031920.1	1090	1488	1413	168	173	221	639.6914	1041.15	238.228413	-2.129169468	6.63E-41	8.49E-40	down	--
Solyc02g093230.4	24	8	8	197	210	167	128.0925	10.5048	245.6802	4.554894912	7.29E-41	9.32E-40	up	O-methyltransferase
novel.1078	419	369	524	32	33	49	193.5286	338.93	48.126817	-2.817160922	7.35E-41	9.39E-40	down	--
Solyc06g066770.1	301	283	351	11	7	8	126.895	242.438	11.3521343	-4.458776737	8.99E-41	1.14E-39	down	FOG: Kelch repeat
Solyc08g082590.3	57	46	21	230	356	392	220.4698	32.9709	407.968761	3.642727033	1.10E-40	1.39E-39	up	Glutaredoxin-related protein
Solyc01g087230.3	526	526	547	62	70	81	253.3785	416.662	90.0951541	-2.21052083	1.20E-40	1.52E-39	down	--
Solyc06g068160.3	187	135	124	389	579	483	363.3116	116.831	609.792485	2.387628729	1.22E-40	1.54E-39	up	5'-AMP-activated protein kinase, gamma subunit
Solyc06g076820.1	172	183	152	344	491	505	347.3965	132.929	561.863774	2.085095657	1.26E-40	1.60E-39	up	--
Solyc07g006360.1	17	20	23	128	172	148	102.3401	15.5707	189.109512	3.60156712	1.45E-40	1.83E-39	up	FOG: Predicted E3 ubiquitin ligase
Solyc04g064880.4	4	14	21	211	167	195	128.4575	9.96997	246.945011	4.613199655	1.59E-40	2.00E-39	up	--
Solyc07g047900.3	647	528	590	68	75	87	278.4697	459.544	97.3951698	-2.240418888	1.66E-40	2.09E-39	down	Poly(A)-specific exoribonuclease PARN
Solyc09g008830.3	2436	1128	1563	15	9	17	675.4522	1333.23	17.6770572	-6.248813747	2.17E-40	2.73E-39	down	--
Solyc05g010570.3	624	691	824	94	121	92	342.439	554.673	130.205396	-2.094413595	2.48E-40	3.11E-39	down	Transcription initiation factor TFIID, subunit
Solyc06g011575.1	410	340	427	7	6	0	155.5865	305.36	5.81255338	-5.792313921	2.53E-40	3.16E-39	down	--
Solyc01g097240.3	187	125	218	3276	2242	2345	1781.859	136.4	3427.31782	4.650120064	2.88E-40	3.59E-39	up	Predicted chitinase
novel.555	20	18	17	117	158	160	98.66037	14.38	182.940765	3.676993479	3.46E-40	4.31E-39	up	FOG: Transposon-encoded proteins with TYA
Solyc09g007910.4	2447	3228	3805	5186	9748	8923	6179.687	2456.47	9902.89994	2.011414446	3.60E-40	4.48E-39	up	Phenylalanine and histidine ammonia-lyase
Solyc09g065200.4	696	673	976	101	87	83	360.8844	604.901	116.867391	-2.38521904	3.67E-40	4.57E-39	down	Cyclin
Solyc12g038420.2	358	272	359	7	8	5	132.4775	256.385	8.56999059	-4.922499319	4.21E-40	5.23E-39	down	--
Solyc12g044610.2	178	125	151	320	430	481	317.378	118.072	516.683655	2.13140453	4.45E-40	5.52E-39	up	--
Solyc06g072145.1	497	506	530	55	78	58	239.9883	399.336	80.6407625	-2.307862579	5.47E-40	6.77E-39	down	--
Solyc10g044510.2	130	84	81	347	393	369	274.2926	77.2282	471.356917	2.611818205	5.86E-40	7.24E-39	up	--
novel.1333	347	269	343	13	18	18	134.7051	248.822	20.5878848	-3.590577425	5.86E-40	7.24E-39	down	--
Solyc01g010650.3	548	553	738	74	99	84	292.0126	475.485	108.540317	-2.132478176	7.32E-40	9.03E-39	down	UDP-galactose transporter related protein
Solyc05g013320.1	541	520	585	59	94	68	260.4111	428.004	92.8178742	-2.201169008	8.71E-40	1.07E-38	down	Serine/threonine protein kinase
Solyc10g083370.1	374	319	369	19	34	22	153.764	276.144	31.3840637	-3.128627988	9.53E-40	1.17E-38	down	--
Solyc03g044900.3	5	7	7	245	136	210	131.309	4.94798	257.670053	5.701357574	1.12E-39	1.38E-38	up	Calmodulin and related proteins (EF-Hand superfamily)
Solyc03g121940.3	118	109	131	259	344	301	237.3071	92.9284	381.685807	2.03770276	1.14E-39	1.40E-38	up	CCAAT-binding factor, subunit B (HAP2)
Solyc08g076010.4	502	518	612	58	87	86	260.1194	423.525	96.7138053	-2.124348368	1.27E-39	1.55E-38	down	--
Solyc08g078150.1	501	482	520	55	73	76	238.5612	391.31	85.8124955	-2.185765991	1.27E-39	1.55E-38	down	FOG: PPR repeat
Solyc02g088200.4	65	63	83	209	257	229	174.5376	54.5947	294.48048	2.427416014	1.32E-39	1.61E-38	up	Transcription coactivator complex, P50 component
Solyc06g053190.3	704	671	926	90	142	102	367.4648	594.569	140.360999	-2.080767002	1.38E-39	1.69E-38	down	Ypt/Rab-specific GTPase-activating protein GYP7
Solyc06g061030.3	53	44	42	168	231	200	144.4371	36.3472	252.527072	2.801882173	1.44E-39	1.75E-38	up	GATA-4/5/6 transcription factors
Solyc11g008530.3	772	438	413	6096	7010	6990	4474.067	425.433	8522.70039	4.324566596	2.41E-39	2.92E-38	up	dsRNA-specific nuclease Dicer and related ribonucleases
novel.155	293	306	327	16	13	19	130.7789	241.034	20.5235429	-3.567844091	2.78E-39	3.37E-38	down	--
Solyc09g082460.3	5651	7975	8275	601	1205	1172	3464.019	5696.49	1231.55118	-2.207995015	2.86E-39	3.46E-38	down	Homocysteine S-methyltransferase
Solyc12g008490.2	338	472	574	30	25	32	197.7105	358.13	37.2912476	-3.276907475	2.99E-39	3.61E-38	down	Ceramide glucosyltransferase
Solyc07g066570.4	105	67	120	256	361	319	234.5341	75.1248	393.943343	2.386132669	3.09E-39	3.73E-38	up	--
Solyc08g067340.4	87	69	89	1501	860	1274	823.8135	63.5342	1584.09273	4.639214785	3.94E-39	4.74E-38	up	--
Solyc04g058000.2	0	3	2	290	513	551	281.5751	1.31429	561.835828	8.759022355	4.50E-39	5.41E-38	up	Multicopper oxidases
Solyc09g089620.1	60	70	91	211	258	325	195.4836	57.1283	333.83892	2.545174132	4.86E-39	5.83E-38	up	--
Solyc02g077790.1	330	426	448	3	4	5	159.1	313.174	5.02581297	-5.951043162	5.62E-39	6.71E-38	down	--
novel.1488	322	330	390	21	7	18	145.3466	270.422	20.2708484	-3.78266181	5.71E-39	6.82E-38	down	--
novel.558	420	390	453	44	33	44	190.1387	328.173	52.1043381	-2.672712737	6.57E-39	7.84E-38	down	--

novel.1654	329	271	329	9	6	17	127.4032	241.276	13.5300631	-4.158496943	6.61E-39	7.89E-38	down	--
Solyc03g114970.3	846	713	714	94	112	128	367.2353	593.532	140.938619	-2.073471546	6.63E-39	7.89E-38	down	--
Solyc02g081340.3	3	9	4	119	217	153	104.2113	4.26275	204.159937	5.614899195	7.01E-39	8.35E-38	up	Glutathione S-transferase
Solyc02g087560.1	360	329	419	18	28	40	161.3955	287.121	35.6697523	-2.9923985	7.27E-39	8.65E-38	down	FOG: PPR repeat
Solyc01g106290.3	404	398	471	37	57	37	192.8315	330.448	55.2154172	-2.580319305	8.20E-39	9.72E-38	down	--
Solyc04g007660.1	315	363	389	15	17	32	152.1399	277.551	26.7288837	-3.365458634	8.53E-39	1.01E-37	down	--
Solyc03g098140.3	524	603	695	81	84	83	289.3179	472.863	105.772379	-2.168146802	8.99E-39	1.06E-37	down	--
Solyc08g014570.3	29	46	28	159	206	234	139.4881	27.2098	251.766425	3.223501954	1.14E-38	1.34E-37	up	FOG: Ankyrin repeat
novel.913	450	400	405	33	49	34	188.2152	327.505	48.9254563	-2.741621508	1.34E-38	1.57E-37	down	--
Solyc09g075920.1	34	37	39	164	189	169	125.2733	28.6376	221.908993	2.953562229	1.39E-38	1.63E-37	up	--
Solyc02g088380.4	332	264	356	9	3	7	127.4722	246.539	8.40494613	-4.934191693	2.00E-38	2.35E-37	down	--
novel.1997	374	252	376	4	6	9	133.4837	259.084	7.88395268	-5.017859135	2.24E-38	2.62E-37	down	--
novel.1432	42	45	43	146	200	203	132.33	33.947	230.713031	2.77054686	2.92E-38	3.40E-37	up	--
Solyc03g120260.4	67	138	77	422	494	494	336.0257	74.6593	597.391999	3.006671534	3.18E-38	3.70E-37	up	Vesicle coat complex COPI, beta' subunit
novel.690	489	439	505	57	59	44	220.6151	372.54	68.6897623	-2.453064387	3.38E-38	3.93E-37	down	--
Solyc06g059800.3	150	89	113	288	451	431	290.3758	91.5435	489.208156	2.420023032	3.58E-38	4.15E-37	up	--
Solyc07g063450.3	307	284	329	18	24	19	132.4391	239.075	25.8034488	-3.214233334	5.08E-38	5.88E-37	down	Amine oxidase
Solyc11g012360.2	163	369	250	4403	8890	7310	4373.83	205.843	8541.81763	5.375556173	5.70E-38	6.60E-37	up	Na+/dicarboxylate
Solyc07g065690.3	485	432	493	51	58	72	221.5334	366.668	76.398648	-2.2626923	8.02E-38	9.25E-37	down	--
novel.1435	48	37	31	156	211	196	133.8208	30.4475	237.194142	2.970012932	8.54E-38	9.84E-37	up	--
Solyc12g049175.1	6	9	11	96	149	151	86.08725	6.72384	165.450662	4.619359186	1.06E-37	1.22E-36	up	--
Solyc09g008370.1	307	335	381	17	28	33	149.0523	265.7	32.4043741	-3.019790345	1.17E-37	1.34E-36	down	--
Solyc06g075170.1	282	279	296	12	8	15	119.1013	223.195	15.0072699	-3.912336666	1.86E-37	2.13E-36	down	--
Solyc10g079810.1	95	77	100	380	335	296	253.3799	70.5091	436.250789	2.62456903	2.05E-37	2.34E-36	up	--
Solyc06g076900.4	396	360	435	37	51	50	183.5799	309.14	58.0194704	-2.410299316	2.89E-37	3.27E-36	down	FOG: PPR repeat
Solyc02g091180.1	334	530	872	24	15	19	234.7255	444.178	25.2733534	-4.160167689	3.31E-37	3.75E-36	down	--
Solyc03g026280.3	286	315	394	11	8	24	137.8523	257.633	18.0712431	-3.829343545	3.32E-37	3.75E-36	down	--
Solyc06g069360.3	332	302	370	24	33	20	146.5877	260.491	32.684572	-3.00060272	3.47E-37	3.92E-36	down	Topoisomerase I-binding arginine-serine-rich
novel.1842	341	303	347	18	34	24	144.6821	257.685	31.6789456	-3.011393152	4.17E-37	4.71E-36	down	--
Solyc07g054270.3	547	428	607	38	63	77	241.4952	409.117	73.8736272	-2.457424549	4.81E-37	5.41E-36	down	--
Solyc12g006060.2	411	407	485	49	36	49	197.9417	338.15	57.7329675	-2.567676007	5.35E-37	6.02E-36	down	--
Solyc11g066450.2	469	427	529	61	73	67	227.4109	369.592	85.229957	-2.120796324	7.42E-37	8.32E-36	down	--
Solyc10g081450.2	108	73	102	224	388	316	230.3569	73.3149	387.398859	2.402737265	8.36E-37	9.36E-36	up	Predicted hydrolase/acyltransferase
Solyc05g055860.4	3	5	11	96	161	159	89.05131	4.8102	173.292427	5.144647405	1.30E-36	1.44E-35	up	Serine/threonine protein kinase
Solyc03g082830.2	482	415	508	52	63	75	222.3602	364.69	80.0304756	-2.186531164	1.87E-36	2.08E-35	down	FOG: PPR repeat
Solyc02g093020.3	55	41	48	186	185	218	144.0593	37.4667	250.651914	2.740730295	1.94E-36	2.15E-35	up	--
Solyc12g006250.3	259	247	306	5	9	11	110.4356	210.53	10.3406809	-4.32317141	2.38E-36	2.63E-35	down	Membrane protein involved in ER to Golgi transport
novel.562	511	329	497	29	10	21	186.1629	345.729	26.5971697	-3.741596264	2.81E-36	3.10E-35	down	--
Solyc10g085210.2	30	71	34	967	1533	1169	787.8678	35.8897	1539.84589	5.428208966	2.83E-36	3.12E-35	up	--
Solyc01g090160.4	327	301	389	17	32	35	149.08	263.405	34.7545728	-2.903132887	2.88E-36	3.17E-35	down	Iron/ascorbate family oxidoreductases
Solyc07g056280.3	39	30	31	129	175	197	118.1314	26.104	210.15871	3.015051941	3.29E-36	3.61E-35	up	--
Solyc02g083110.1	529	609	672	56	101	96	287.7641	470.394	105.13368	-2.150836288	4.12E-36	4.51E-35	down	--
Solyc12g011023.1	462	552	573	3	2	2	208.0996	413.14	3.05954468	-7.125505225	4.48E-36	4.90E-35	down	--
Solyc06g071540.3	12	20	14	107	175	137	93.80434	12.1033	175.505329	3.872660172	5.27E-36	5.76E-35	up	--
novel.1995	313	283	380	21	32	28	143.2454	252.52	33.9706294	-2.888588395	5.33E-36	5.81E-35	down	--
Solyc01g086730.4	32	34	51	191	170	211	137.3208	30.1237	244.517896	3.01166098	6.34E-36	6.91E-35	up	Uncharacterized conserved protein
Solyc11g011020.2	247	308	300	6	9	14	117.4963	222.969	12.0237212	-4.191465591	7.77E-36	8.45E-35	down	--
Solyc07g047690.1	422	351	471	26	55	47	187.5092	322.086	52.9325904	-2.58838389	9.02E-36	9.80E-35	down	--
Solyc01g099410.3	494	354	453	42	33	43	194.2274	337.739	50.7161797	-2.750277465	9.29E-36	1.01E-34	down	Histone 2A
Solyc02g091140.3	459	417	555	60	74	66	227.5292	370.331	84.7278431	-2.131772477	1.04E-35	1.13E-34	down	--
Solyc00g500296.1	6	6	6	137	151	112	87.92298	4.69581	171.150147	5.189482993	1.07E-35	1.16E-34	up	--
novel.438	121	113	116	600	421	420	359.6394	91.2733	628.005532	2.78068879	1.16E-35	1.25E-34	up	K+-channel ERG and related proteins
Solyc01g150164.1	60	46	58	341	236	264	204.1102	42.5694	365.650907	3.097938478	1.23E-35	1.33E-34	up	--
Solyc01g005570.4	372	368	468	40	25	23	175.7779	312.862	38.693857	-3.050454762	1.65E-35	1.78E-34	down	--
Solyc06g009290.4	5	11	8	143	110	187	97.06025	6.30385	187.816648	4.906766744	1.69E-35	1.82E-34	up	Multidrug/pheromone exporter, ABC superfamily

Solyc12g043040.1	517	367	478	34	53	57	206.7497	353.42	60.0792157	-2.547453482	1.74E-35	1.86E-34	down	Sulfate/bicarbonate/oxalate exchanger SAT-1
Solyc11g006560.3	309	298	340	22	18	31	138.166	246.135	30.1970654	-3.034476938	2.14E-35	2.29E-34	down	--
Solyc11g066460.1	360	390	443	39	49	53	184.6496	309.88	59.4192828	-2.381338055	2.16E-35	2.31E-34	down	--
Solyc02g067520.4	453	584	639	57	42	32	246.3577	435.398	57.316872	-2.949157868	2.37E-35	2.54E-34	down	--
novel.1750	647	497	600	58	90	97	277.7469	453.259	102.234507	-2.140473445	3.12E-35	3.34E-34	down	FOG: Reverse transcriptase
Solyc07g043580.4	48	27	42	142	194	191	125.956	30.2697	221.642325	2.870280222	4.19E-35	4.45E-34	up	--
Solyc03g117350.1	366	403	410	30	37	53	178.7646	307.254	50.2756107	-2.604469345	4.61E-35	4.89E-34	down	Amino acid transporter protein
Solyc03g116740.3	384	434	543	39	65	47	207.8082	352.334	63.2818953	-2.472049204	4.88E-35	5.17E-34	down	--
Solyc02g062100.4	368	386	452	22	53	44	181.0031	313.025	48.9811743	-2.654697806	5.87E-35	6.19E-34	down	--
Solyc09g015457.1	290	234	282	10	16	10	112.2821	209.409	15.1548334	-3.785635506	5.96E-35	6.29E-34	down	--
Solyc03g114730.3	244	282	299	7	15	17	115.357	214.669	16.0453063	-3.713032989	7.63E-35	8.02E-34	down	--
Solyc06g005680.4	245	252	272	9	10	7	105.6294	200.126	11.1332483	-4.185174644	9.76E-35	1.02E-33	down	--
Solyc06g054150.1	56	65	53	171	312	395	204.1772	45.6292	362.725256	2.998893723	1.03E-34	1.08E-33	up	--
Solyc02g088953.1	273	225	303	13	13	14	112.219	207.381	17.0567597	-3.616142942	1.03E-34	1.08E-33	down	DNA mismatch repair protein - MLH3 family
Solyc07g053980.3	842	943	908	117	96	157	430.249	702.909	157.589049	-2.161047337	1.04E-34	1.09E-33	down	1,3-beta-glucan synthase
Solyc09g007340.4	272	293	374	18	18	29	135.2169	243.013	27.4207486	-3.147789213	1.52E-34	1.59E-33	down	--
Solyc02g071900.3	417	433	509	60	67	59	215.9745	352.719	79.2304497	-2.162161055	1.53E-34	1.60E-33	down	Uncharacterized conserved protein
Solyc08g005333.1	246	248	300	7	8	4	107.0544	205.934	8.17440613	-4.679544037	1.57E-34	1.63E-33	down	--
Solyc02g065765.1	677	633	605	95	93	96	310.8935	500.416	121.370812	-2.050936114	1.78E-34	1.85E-33	down	--
Solyc04g008820.3	9	5	6	105	129	115	76.54653	5.21201	147.881054	4.829051423	2.25E-34	2.33E-33	up	HMG box-containing protein
Solyc04g078420.1	1173	1001	1385	101	223	206	569.4637	920.396	218.531293	-2.065668288	2.52E-34	2.60E-33	down	Transcription factor, Myb superfamily
Solyc02g086770.3	36	28	40	147	155	168	113.3577	26.8906	199.824777	2.887474338	2.57E-34	2.66E-33	up	Flavonol reductase/cinnamoyl-CoA reductase
novel.1812	88	89	85	188	329	299	204.1247	68.4376	339.811903	2.318511631	2.64E-34	2.73E-33	up	--
Solyc05g015380.2	39	59	73	166	290	264	172.0246	44.2016	299.847593	2.762430711	2.68E-34	2.76E-33	up	HORMA domain
novel.327	367	244	476	20	16	20	151.428	278.782	24.0735475	-3.551946924	2.77E-34	2.86E-33	down	--
Solyc04g007640.3	520	713	784	76	90	101	318.2353	523.721	112.75001	-2.215991626	2.84E-34	2.93E-33	down	FOG: Armadillo/beta-catenin-like repeats
novel.582	476	332	508	33	29	51	193.8781	339.901	47.855243	-2.831673195	3.04E-34	3.13E-33	down	--
Solyc00g500065.1	7	4	3	148	172	110	93.84568	3.68832	184.003042	5.654526594	3.06E-34	3.15E-33	up	3-Methylcrotonyl-CoA carboxylase
Solyc06g053290.1	492	403	599	52	60	78	232.8433	385.639	80.0479566	-2.267954137	3.10E-34	3.18E-33	down	--
Solyc07g051930.3	12	11	12	89	145	124	79.42448	9.11213	149.736843	4.04389953	3.83E-34	3.93E-33	up	MEKK and related serine/threonine protein kinases
Solyc07g005235.1	110	90	80	246	317	272	213.3006	73.3626	353.238676	2.271437861	5.91E-34	6.03E-33	up	--
Solyc12g098910.2	12	22	18	109	132	145	88.2585	13.614	162.903008	3.588585995	9.30E-34	9.42E-33	up	Serine/threonine protein kinase
Solyc07g054790.1	1974	780	1626	12	6	11	570.5313	1128.42	12.6454172	-6.498882423	1.04E-33	1.05E-32	down	--
Solyc06g074710.1	1766	3407	3496	16587	28353	24409	15595.45	2252.37	28938.5259	3.683497869	1.05E-33	1.06E-32	up	--
Solyc08g080780.4	390	346	415	44	38	50	177.6522	298.878	56.4266321	-2.416247672	1.48E-33	1.49E-32	down	--
novel.1284	219	312	360	13	10	8	122.2247	230.935	13.5139806	-4.130252443	1.50E-33	1.51E-32	down	--
Solyc01g099035.1	59	54	73	208	220	196	157.3088	48.1088	266.50888	2.463570909	1.91E-33	1.91E-32	up	--
Solyc09g008850.3	455	452	518	51	82	83	230.1765	370.249	90.104256	-2.02945241	2.38E-33	2.37E-32	down	--
Solyc02g067440.3	338	253	295	6	2	5	118.1394	230.544	5.73515891	-5.386203253	2.75E-33	2.73E-32	down	Uncharacterized conserved protein
novel.90	753	577	690	3	1	1	263.7097	525.145	2.27420276	-7.956486293	3.07E-33	3.04E-32	down	Tam3-transposase (Ac family)
Solyc02g088240.4	289	218	418	9	16	14	126.6348	237.029	16.2408843	-3.855314996	3.38E-33	3.34E-32	down	Predicted small molecule transporter
Solyc05g041530.3	675	495	677	70	101	111	298.2308	478.446	118.015465	-2.014269793	4.17E-33	4.10E-32	down	--
Solyc09g005860.3	361	320	455	36	39	50	173.141	293.436	52.846094	-2.475662905	4.56E-33	4.48E-32	down	FOG: Ankyrin repeat
Solyc08g077830.1	424	503	588	54	74	82	240.507	392.935	88.0794727	-2.153024454	5.69E-33	5.56E-32	down	HEAT repeat-containing protein
Solyc11g062030.1	446	347	429	46	40	31	184.0118	317.341	50.6826162	-2.667470632	5.94E-33	5.81E-32	down	--
Solyc05g026215.1	332	305	309	22	35	23	140.2373	246.816	33.6582664	-2.870720312	5.98E-33	5.84E-32	down	--
Solyc06g074530.1	164	210	304	463	1030	885	577.9199	174.517	981.323285	2.491251999	7.26E-33	7.06E-32	up	Prephenate dehydratase
Solyc04g077300.3	48	31	40	158	160	205	126.3906	30.9118	221.869345	2.842027762	8.32E-33	8.07E-32	up	--
Solyc04g078950.1	92	91	96	184	281	292	194.5119	72.6745	316.349306	2.126857577	9.50E-33	9.19E-32	up	--
Solyc03g112060.3	33	37	34	129	162	155	107.8301	27.1828	188.477316	2.798340589	1.06E-32	1.03E-31	up	--
Solyc12g005010.2	509	405	559	63	76	82	237.2577	381.19	93.3255702	-2.031322752	1.20E-32	1.15E-31	down	--
Solyc06g008510.3	318	307	436	31	23	37	156.4316	273.877	38.9859419	-2.826573608	1.22E-32	1.18E-31	down	DNA replication factor
Solyc09g090380.1	233	283	357	14	8	19	121.7059	225.83	17.5821817	-3.699933654	1.30E-32	1.25E-31	down	--
Solyc12g049390.2	416	414	477	47	70	75	209.9035	339.53	80.2773431	-2.0726955	1.47E-32	1.41E-31	down	Predicted membrane protein
novel.1424	4	0	5	177	128	180	105.5937	2.2505	208.936947	6.48447541	1.55E-32	1.49E-31	up	--

Solyc03g120040.3	240	261	271	4	13	17	107.4269	201.077	13.7769306	-3.821121661	1.78E-32	1.70E-31	down	--
Solyc02g092840.1	582	640	788	50	105	112	315.3792	520.714	110.04434	-2.228981092	1.91E-32	1.82E-31	down	Acetylglucosaminyltransferase EXT1/exostosin 1
Solyc03g115550.3	456	405	487	62	75	59	216.6719	350.003	83.3410832	-2.076394761	1.95E-32	1.86E-31	down	--
Solyc07g054370.3	393	360	443	27	62	42	182.2136	310.248	54.1792572	-2.501249021	1.96E-32	1.87E-31	down	--
novel.874	562	449	568	54	94	89	254.1648	409.686	98.6437131	-2.04443679	2.15E-32	2.05E-31	down	--
Solyc00g500083.1	7	4	13	90	117	127	73.28707	6.06746	140.506674	4.504396314	2.35E-32	2.23E-31	up	--
Solyc12g056980.1	57	33	64	170	199	206	141.4944	39.5678	243.420914	2.612946416	3.37E-32	3.19E-31	up	--
Solyc01g044365.1	6	1	6	125	116	126	80.19487	3.29837	157.091376	5.541007856	3.62E-32	3.43E-31	up	--
Solyc01g104900.3	15	7	4	448	1279	817	525.4577	6.88655	1044.02884	7.253636105	3.87E-32	3.66E-31	up	--
Solyc09g082810.3	26	18	42	190	147	201	126.5103	21.9192	231.101343	3.382083796	4.34E-32	4.10E-31	up	--
Solyc11g008520.3	2	7	4	88	148	129	77.91315	3.43854	152.38775	5.495572989	5.83E-32	5.49E-31	up	dsRNA-specific nuclease Dicer
Solyc12g014490.3	520	347	597	49	32	33	213.3912	376.938	49.8445863	-2.943580626	8.69E-32	8.13E-31	down	Microtubule-associated protein
Solyc04g072490.4	206	299	308	8	13	14	113.0289	211.482	14.575325	-3.844341205	1.17E-31	1.10E-30	down	Mitochondrial transcription termination factor, mTERF
Solyc07g064740.3	353	442	482	38	60	58	198.5118	331.835	65.1882501	-2.339941947	1.69E-31	1.57E-30	down	Uncharacterized conserved protein
Solyc10g009160.3	219	225	244	8	12	10	95.81248	179.022	12.6032297	-3.822530399	1.99E-31	1.84E-30	down	--
Solyc11g069570.2	21	23	25	96	146	184	97.64082	17.946	177.335676	3.309847352	2.03E-31	1.88E-30	up	--
novel.2029	264	213	288	11	15	21	108.8422	198.072	19.6127919	-3.324670965	2.81E-31	2.59E-30	down	--
Solyc03g031880.3	19	14	23	120	145	110	87.00391	14.4243	159.583556	3.457822966	3.12E-31	2.87E-30	up	Amine oxidase
Solyc08g076780.2	35	45	49	133	402	309	189.2211	33.5179	344.92426	3.367270128	3.47E-31	3.19E-30	up	Flavonol reductase/cinnamoyl-CoA reductase
novel.944	431	354	361	33	29	49	173.1024	299.141	47.0640741	-2.671074123	5.71E-31	5.20E-30	down	FOG: Transposon-encoded proteins with TYA
Solyc06g069710.3	47	21	46	197	210	169	137.8753	29.2792	246.471369	3.063596125	6.32E-31	5.75E-30	up	--
Solyc05g010540.2	288	250	255	15	16	23	114.8529	206.927	22.7788661	-3.18195971	7.44E-31	6.76E-30	down	--
Solyc06g065190.1	8	8	12	75	125	122	70.70762	7.21273	134.202507	4.212987387	8.60E-31	7.79E-30	up	--
Solyc11g066510.3	337	223	256	9	17	13	114.4251	212.615	16.2350573	-3.696520787	8.62E-31	7.80E-30	down	FOG: Predicted E3 ubiquitin ligase
Solyc08g078700.2	653	528	353	5275	4060	5877	3464.964	404.75	6525.1788	4.011329634	8.84E-31	7.99E-30	up	Molecular chaperone (small heat-shock protein Hsp26/Hsp42)
Solyc01g100020.4	35	39	34	110	217	196	122.4879	28.2722	216.703487	2.948315628	9.17E-31	8.30E-30	up	Phospholipase D1
Solyc10g055390.2	686	729	799	59	141	121	353.946	575.79	132.102451	-2.110687689	9.19E-31	8.30E-30	down	--
Solyc12g056300.3	301	341	336	29	41	35	149.6487	255.08	44.2178333	-2.525468406	9.72E-31	8.78E-30	down	--
novel.161	11	6	7	82	108	122	68.65526	6.25988	131.050639	4.393938074	9.99E-31	9.02E-30	up	FOG: Transposon-encoded proteins with TYA
Solyc01g081270.2	122	135	114	262	359	497	281.8835	97.2114	466.555584	2.268084577	1.21E-30	1.09E-29	up	Glutathione S-transferase
Solyc08g006760.4	277	247	318	18	28	31	125.1345	218.16	32.1094921	-2.753013663	1.58E-30	1.42E-29	down	--
novel.1577	327	363	357	30	23	44	157.1896	273.12	41.2587462	-2.732559804	1.63E-30	1.46E-29	down	--
Solyc01g104110.4	5	9	10	122	99	112	74.82958	6.2207	143.438455	4.520108894	1.70E-30	1.52E-29	up	--
Solyc10g085800.2	45	41	61	146	177	172	123.6964	37.9072	209.485491	2.460172602	1.95E-30	1.73E-29	up	--
Solyc01g089950.3	250	206	277	11	15	4	101.3363	189.785	12.8878561	-3.900818373	2.00E-30	1.78E-29	down	--
Solyc02g072470.4	238	245	249	3	6	4	98.12511	190.84	5.40974343	-5.121591378	2.40E-30	2.13E-29	down	--
Solyc06g059870.1	125	94	100	287	265	343	232.3114	83.2173	381.40555	2.196167782	2.94E-30	2.60E-29	up	--
Solyc12g055730.3	10	21	32	118	153	143	95.44908	16.1348	174.763329	3.42610838	3.03E-30	2.67E-29	up	Predicted lipase
Solyc05g054405.1	264	285	288	23	10	17	120.1159	218.195	22.0371102	-3.349241439	3.10E-30	2.74E-29	down	--
Solyc09g007410.3	533	457	563	58	75	105	251.2967	403.04	99.5528206	-2.01126492	3.14E-30	2.77E-29	down	--
Solyc10g075110.2	44	30	57	228	190	183	146.6076	33.616	259.599297	2.9375045	3.76E-30	3.31E-29	up	--
Solyc02g078180.3	257	266	346	21	24	33	128.8287	224.827	32.8304921	-2.773823141	5.12E-30	4.48E-29	down	--
Solyc05g041700.1	265	243	337	17	15	30	122.265	218.379	26.1507738	-3.062055639	6.05E-30	5.29E-29	down	--
Solyc07g026930.3	24	13	20	84	215	216	112.8447	14.7572	210.9322	3.839678247	6.58E-30	5.75E-29	up	Predicted transporter ADD1 (major facilitator superfamily)
Solyc03g114950.2	132	86	142	228	388	358	249.4145	92.8304	405.998554	2.128323282	6.82E-30	5.95E-29	up	Peptide exporter, ABC superfamily
Solyc09g007840.3	2	2	5	98	141	127	78.05508	2.27901	153.831149	6.044920128	6.97E-30	6.08E-29	up	K+-channel ERG and related proteins
Solyc12g042600.2	1	0	2	470	511	445	304.5985	0.74106	608.456011	9.608210595	8.33E-30	7.25E-29	up	UDP-glucuronosyl and UDP-glucosyl transferase
Solyc04g077460.3	243	201	252	8	10	18	97.78564	180.583	14.9883904	-3.575834322	1.07E-29	9.27E-29	down	Amino acid transporter protein
Solyc01g100400.3	350	282	372	25	48	39	153.3472	260.151	46.5433256	-2.469726414	1.22E-29	1.05E-28	down	RNA pseudouridylate synthases
novel.545	251	208	247	6	17	16	99.70227	183.472	15.9329498	-3.489942187	1.37E-29	1.18E-28	down	--
Solyc06g084320.1	385	465	464	60	53	59	208.1212	342.469	73.7738453	-2.226058752	1.40E-29	1.20E-28	down	Predicted E3 ubiquitin ligase
Solyc07g065420.1	231	219	269	11	19	21	103.8236	186.475	21.1718218	-3.121556393	1.53E-29	1.32E-28	down	--

Solyc10g086520.2	222	365	415	5	3	1	131.8377	259.629	4.04629157	-6.085327918	1.66E-29	1.43E-28	down	--
Solyc06g083200.3	321	296	275	20	34	23	132.7852	233.294	32.2759351	-2.846306502	1.73E-29	1.49E-28	down	FOG: PPR repeat
Solyc12g070120.1	260	208	298	14	22	22	111.1089	197.992	24.2255394	-3.021305263	2.44E-29	2.09E-28	down	--
Solyc06g060690.3	446	460	668	72	68	81	250.0317	405.785	94.2785086	-2.113707449	2.82E-29	2.42E-28	down	Serine/threonine protein kinase
Solyc02g068040.3	246	233	287	20	26	21	113.508	198.649	28.3667065	-2.811558236	3.23E-29	2.75E-28	down	--
Solyc02g031780.1	314	246	342	3	1	4	118.4323	233.404	3.46095614	-6.109778818	3.55E-29	3.02E-28	down	--
novel.107	234	182	230	7	8	7	88.50629	167.651	9.36115951	-4.172721592	3.56E-29	3.04E-28	down	--
Solyc01g058270.3	33	42	33	145	171	140	111.1671	28.3423	193.991957	2.780095726	3.76E-29	3.20E-28	up	O-acetyltransferase
Solyc09g010810.3	6	4	8	72	111	112	63.95693	4.61266	123.301198	4.72795614	4.52E-29	3.84E-28	up	Kinesin-like protein
Solyc11g005750.2	13	22	13	82	149	166	88.56303	12.6896	164.436411	3.715049632	4.71E-29	4.00E-28	up	--
Solyc04g078290.4	63	55	97	269	216	268	189.4323	55.1592	323.705435	2.54378808	5.36E-29	4.54E-28	up	Cytochrome P450 CYP2 subfamily
novel.1514	224	196	237	4	4	7	88.4453	170.577	6.31326884	-4.752449905	5.46E-29	4.62E-28	down	--
Solyc10g084820.2	412	403	483	63	49	69	207.2407	336.822	77.6595209	-2.129085037	6.82E-29	5.75E-28	down	Predicted DHHC-type Zn-finger protein
Solyc09g009080.4	395	391	537	47	80	64	210.815	341.807	79.8234886	-2.091962677	6.97E-29	5.87E-28	down	--
Solyc03g005580.2	196	312	283	12	13	14	111.538	206.516	16.5604728	-3.645825701	7.91E-29	6.64E-28	down	--
Solyc08g081100.4	281	299	363	34	37	40	145.7891	244.46	47.1181606	-2.380812532	8.95E-29	7.50E-28	down	Kinesin (KAR3 subfamily)
Solyc12g096310.2	311	315	308	23	27	43	141.3759	243.804	38.9481829	-2.638335423	9.46E-29	7.92E-28	down	--
Solyc01g110510.3	17	17	14	83	121	117	73.61345	12.591	134.635851	3.430064803	1.01E-28	8.41E-28	up	Equilibrative nucleoside transporter protein
Solyc02g076920.3	10	6	10	97	99	94	65.30958	6.70839	123.910761	4.196214126	1.26E-28	1.05E-27	up	--
Solyc06g071780.4	146	143	124	380	366	326	284.1964	108.192	460.200804	2.08869459	1.30E-28	1.08E-27	up	--
novel.126	251	208	208	11	9	9	93.36008	174.193	12.5272337	-3.822764226	1.33E-28	1.10E-27	down	--
novel.375	208	193	223	9	7	5	85.66858	162.164	9.17280704	-4.185553286	1.42E-28	1.18E-27	down	--
Solyc07g037950.1	232	238	278	20	25	19	110.689	194.192	27.1857801	-2.843220571	1.62E-28	1.34E-27	down	Type I phosphodiesterase/nucleotide pyrophosphatase
Solyc01g081380.4	332	361	357	28	41	55	162.7604	273.888	51.6332356	-2.395277455	1.65E-28	1.37E-27	down	--
Solyc01g111010.4	71	87	61	200	239	224	169.34	57.6597	281.020341	2.291373017	1.73E-28	1.43E-27	up	--
Solyc09g061791.1	206	304	412	7	13	22	127.4333	237.623	17.2437137	-3.759723002	1.78E-28	1.47E-27	down	Cytochrome P450 CYP2 subfamily
Solyc03g117630.1	1248	1012	817	7359	5398	7282	4722.48	808.227	8636.73245	3.417796554	1.89E-28	1.56E-27	up	Molecular chaperones HSP70/HSC70, HSP70 superfamily
Solyc12g005760.2	244	289	302	24	17	28	123.476	217.339	29.6131284	-2.890760186	2.14E-28	1.76E-27	down	--
Solyc12g009300.3	362	221	449	872	816	848	675.4322	264.604	1086.25993	2.034154794	2.18E-28	1.79E-27	up	Glycosyltransferase
Solyc11g071850.1	470	417	420	30	73	54	202.916	341.13	64.7024636	-2.381191635	2.21E-28	1.82E-27	down	--
Solyc05g054040.4	245	307	290	22	29	18	124.5608	219.78	29.3417994	-2.911246344	2.90E-28	2.37E-27	down	Endosomal membrane proteins, EMP70
novel.2009	353	258	348	32	29	39	145.5705	248.529	42.6119425	-2.552729816	3.18E-28	2.60E-27	down	--
Solyc04g016460.3	54	70	67	229	182	270	170.6102	49.827	291.393373	2.547526038	3.30E-28	2.69E-27	up	--
Solyc01g107070.4	271	242	311	10	29	29	120.6216	213.505	27.7377851	-2.912613375	3.45E-28	2.81E-27	down	--
Solyc02g072450.4	323	276	328	36	24	32	140.3619	240.845	39.8792118	-2.617545456	3.82E-28	3.11E-27	down	--
novel.904	316	290	332	27	39	48	145.7204	243.852	47.5883425	-2.347338289	4.16E-28	3.38E-27	down	--
Solyc12g094550.2	378	387	436	24	64	55	185.3814	312.15	58.6125093	-2.391131759	4.23E-28	3.44E-27	down	--
Solyc04g077000.3	285	232	289	22	32	23	120.8393	209.19	32.4889941	-2.686806883	4.58E-28	3.71E-27	down	Predicted E3 ubiquitin ligase
novel.1568	447	365	471	60	52	38	198.8532	332.63	65.0768142	-2.373315069	5.16E-28	4.18E-27	down	--
Solyc09g064540.3	590	554	679	101	95	67	293.2616	472.867	113.656099	-2.070224237	5.31E-28	4.30E-27	down	--
Solyc07g056685.1	20	22	19	164	111	139	97.80706	15.9738	179.640377	3.491705462	5.57E-28	4.50E-27	up	--
Solyc04g076680.2	218	190	247	15	11	12	93.08341	169.688	16.4786498	-3.394311582	5.71E-28	4.61E-27	down	MADS box transcription factor
novel.1283	217	181	215	4	10	7	83.97304	159.294	8.65181358	-4.173338222	6.03E-28	4.87E-27	down	--
Solyc05g052040.1	1932	2779	4210	11440	17500	15908	10540.99	2290.75	18791.2358	3.036089789	6.80E-28	5.48E-27	up	--
Solyc02g084980.3	297	183	227	1816	2007	3931	1711.235	183.927	3238.54283	4.138274358	6.93E-28	5.58E-27	up	Glycosyl transferase, family 8 - glycogenin
Solyc06g008810.3	295	351	280	25	33	22	138.4659	242.96	33.9720279	-2.842174318	7.42E-28	5.97E-27	down	Leucine rich repeat proteins, some proteins contain F-box
Solyc06g060820.2	42	23	43	108	221	241	131.4348	27.7983	235.071243	3.078924885	7.69E-28	6.18E-27	up	--
Solyc01g008910.2	340	270	382	28	43	48	153.0838	256.524	49.6436593	-2.360381799	7.72E-28	6.20E-27	down	--
Solyc08g005335.1	325	298	418	1	1	4	135.7022	268.936	2.46838225	-6.732977832	9.55E-28	7.65E-27	down	--
Solyc01g005120.3	320	303	362	27	52	48	154.1697	255.684	52.6551894	-2.264565105	1.45E-27	1.15E-26	down	--
novel.1398	211	173	237	11	10	8	86.61125	160.701	12.5214067	-3.70866886	1.46E-27	1.16E-26	down	--
Solyc04g050960.3	499	347	494	53	65	75	214.0845	346.863	81.3062775	-2.092203098	1.77E-27	1.40E-26	down	Predicted helicase

Solyc12g057090.1	299	275	320	31	27	42	137.4095	232.296	42.5228941	-2.456209213	1.79E-27	1.42E-26	down	--
Solyc07g005390.3	73	48	34	167	288	241	165.6662	40.8664	290.465923	2.838186247	1.94E-27	1.54E-26	up	Aldehyde dehydrogenase
Solyc04g058150.3	219	143	198	351	448	646	374.7576	145.16	604.355621	2.058391428	2.05E-27	1.62E-26	up	Predicted metallothionein
Solyc12g096030.1	367	320	423	36	67	46	174.7955	287.414	62.1769649	-2.200359332	2.15E-27	1.70E-26	down	Predicted mitochondrial carrier protein
Solyc01g096730.2	89	69	94	203	209	222	167.6398	65.2543	270.025309	2.044171782	2.28E-27	1.80E-26	up	Permease of the major facilitator superfamily
Solyc12g005900.3	380	352	407	59	60	55	185.2113	295.999	74.4235227	-2.002425096	3.04E-27	2.39E-26	down	--
Solyc02g090120.1	2389	975	1885	45	31	44	703.2147	1354.61	51.8211101	-4.714017147	3.67E-27	2.88E-26	down	--
novel.1230	313	237	262	22	14	25	118.9272	211.59	26.2645288	-3.027855575	4.00E-27	3.13E-26	down	--
Solyc08g006080.1	249	267	238	22	18	19	111.3699	197.29	25.4500518	-2.974200957	4.40E-27	3.44E-26	down	Acetylglucosaminyltransferase EXT1/exostosin 1
Solyc04g082270.3	244	276	331	23	16	33	125.655	220.605	30.7050063	-2.854344554	4.80E-27	3.75E-26	down	--
Solyc02g005606.1	997	726	1012	148	97	110	431.4426	708.114	154.771232	-2.206124125	4.86E-27	3.80E-26	down	--
Solyc05g007895.1	97	67	100	211	224	229	175.4278	68.2446	282.611058	2.044774703	5.82E-27	4.53E-26	up	FOG: Predicted E3 ubiquitin ligase
Solyc01g007267.1	220	216	220	3	2	6	87.85175	171.062	4.64188252	-5.202713419	5.85E-27	4.55E-26	down	FOG: PPR repeat
Solyc02g031990.1	71	61	86	245	199	224	172.0519	56.3409	287.762955	2.345433705	5.90E-27	4.59E-26	up	--
Solyc08g076120.3	80	75	100	235	245	203	179.1966	65.9716	292.421655	2.142255317	6.27E-27	4.88E-26	up	Uncharacterized conserved protein
Solyc02g077690.4	518	471	734	88	74	93	276.4815	443.658	109.304658	-2.031024599	7.19E-27	5.58E-26	down	--
Solyc10g084640.1	382	260	382	39	34	37	156.0562	264.869	47.2435696	-2.50161793	7.31E-27	5.68E-26	down	Diamine acetyltransferase
Solyc03g082530.1	232	412	476	776	948	1070	733.9072	289.93	1177.88411	2.022116392	7.56E-27	5.86E-26	up	--
Solyc01g008260.4	378	458	542	56	80	50	217.9824	357.213	78.7518887	-2.18385514	8.15E-27	6.31E-26	down	Uncharacterized conserved protein
Solyc09g008170.3	293	393	385	41	44	34	165.0479	279.149	50.9469646	-2.464416942	8.35E-27	6.45E-26	down	--
Solyc04g072500.1	342	329	395	45	53	65	172.6701	276.637	68.7030478	-2.008613814	1.09E-26	8.38E-26	down	Mitochondrial transcription termination factor, mTERF
Solyc09g075720.4	222	230	307	12	18	29	110.3233	196.204	24.4430269	-2.987829819	1.27E-26	9.73E-26	down	Serine/threonine protein kinase
Solyc11g040330.2	18	17	19	88	100	119	71.8847	14.0458	129.723548	3.208191119	1.38E-26	1.06E-25	up	--
Solyc08g080000.3	317	288	311	37	41	42	144.7598	238.562	50.9572201	-2.231832034	1.40E-26	1.07E-25	down	--
Solyc06g063280.1	103	135	177	242	448	411	282.2293	107.161	457.297995	2.094191102	2.09E-26	1.59E-25	up	--
Solyc07g006855.2	202	235	287	18	15	23	105.708	187.538	23.8779695	-2.983820368	2.19E-26	1.67E-25	down	--
Solyc06g033850.3	368	214	299	18	14	27	126.8114	228.552	25.0705499	-3.194362997	2.37E-26	1.80E-25	down	--
Solyc08g007510.3	230	210	259	22	15	12	101.4137	181.316	21.5116882	-3.114708411	2.39E-26	1.82E-25	down	ER vesicle integral membrane protein
novel.1302	1078	877	1096	155	81	119	473.6773	791.785	155.569382	-2.359578895	2.53E-26	1.92E-25	down	--
Solyc06g052020.2	7	3	4	84	109	80	59.73258	3.64674	115.818423	4.990838279	2.70E-26	2.05E-25	up	--
Solyc02g083310.3	346	341	419	39	63	68	178.7858	286.762	70.8096541	-2.007634006	3.36E-26	2.54E-25	down	--
Solyc11g066900.2	329	277	356	33	48	52	152.5166	249.377	55.6562191	-2.156863868	3.55E-26	2.68E-25	down	Xanthine/uracil transporters
Solyc03g118630.3	66	92	61	170	256	273	174.9361	57.731	292.141248	2.34861342	3.87E-26	2.91E-25	up	--
Solyc03g093110.3	271	230	317	29	32	22	123.5732	211.579	35.5674182	-2.587232476	4.21E-26	3.16E-25	down	--
Solyc12g011280.2	13	11	15	76	94	104	62.79345	10.0911	115.495793	3.513490383	5.48E-26	4.10E-25	up	--
Solyc02g068550.2	213	221	216	13	21	14	94.9128	169.651	20.1748194	-3.066790533	5.89E-26	4.39E-25	down	--
novel.478	5	0	2	87	159	114	76.02351	1.80199	150.245029	6.372504616	5.95E-26	4.44E-25	up	--
Solyc04g081950.4	297	294	333	38	50	41	147.3673	240.169	54.5657397	-2.139816625	6.38E-26	4.75E-25	down	FOG: PPR repeat
Solyc06g084430.4	234	246	319	24	33	30	121.6767	206.713	36.6404166	-2.495017945	7.65E-26	5.68E-25	down	Histone 2A
Solyc01g091260.3	121	70	82	261	248	308	209.5985	71.1662	348.030757	2.289587445	8.24E-26	6.11E-25	up	--
Solyc09g008970.1	1800	1160	1383	7503	5805	7210	4984.503	1130.66	8838.34697	2.966564865	8.40E-26	6.22E-25	up	--
Solyc04g074840.3	1610	2161	2286	6225	13105	8567	6580.5	1574.87	11586.1298	2.879163794	8.94E-26	6.61E-25	up	Uncharacterized membrane protein
Solyc02g089880.4	192	173	213	4	9	14	80.49145	149.952	11.0311473	-3.727840164	9.28E-26	6.86E-25	down	--
novel.1270	194	178	219	10	11	16	84.44335	153.307	15.5795529	-3.296402629	9.36E-26	6.92E-25	down	--
novel.2008	203	170	233	10	8	16	85.5997	156.789	14.4102805	-3.448944027	9.66E-26	7.14E-25	down	--
novel.768	212	195	249	17	21	18	96.85618	169.97	23.742305	-2.845007689	1.05E-25	7.77E-25	down	--
Solyc03g119920.1	327	332	346	48	44	51	161.4926	261.839	61.1459091	-2.108398391	1.12E-25	8.24E-25	down	--
Solyc07g065320.4	16	13	25	74	124	137	76.53757	13.8249	139.25023	3.324787278	1.19E-25	8.74E-25	up	Multidrug resistance-associated protein
Solyc10g024490.2	79	73	85	142	251	258	165.9707	61.5787	270.362659	2.139118191	1.24E-25	9.09E-25	up	H+/oligopeptide symporter
Solyc01g108800.4	256	147	255	2	9	5	88.0653	169.652	6.47831331	-4.662849888	1.30E-25	9.58E-25	down	--
Solyc10g079640.2	12	12	12	63	110	115	64.51161	9.39161	119.631611	3.680292412	1.35E-25	9.89E-25	up	--
Solyc10g083190.3	310	307	355	38	39	57	154.5461	252.484	56.6077591	-2.157818472	1.58E-25	1.16E-24	down	--
Solyc05g008860.4	194	163	230	10	7	13	82.28282	151.732	12.8337697	-3.579446308	1.61E-25	1.18E-24	down	--
Solyc02g086260.1	200	150	232	6	4	6	78.538	150.166	6.91025828	-4.47110214	1.70E-25	1.24E-24	down	--

Solyc01g010390.3	71	56	83	143	205	193	140.7234	54.2297	227.217113	2.065044608	1.71E-25	1.25E-24	up	Beta-glucosidase, lactase phlorizinhydrolase
Solyc05g009340.1	227	186	276	20	18	16	100.5638	177.857	23.2707246	-2.955848294	1.86E-25	1.36E-24	down	--
Solyc08g077970.2	46	49	56	114	178	165	115.2219	39.2188	191.224975	2.288842051	2.35E-25	1.71E-24	up	--
Solyc01g113620.3	181	203	214	11	16	8	85.25836	155.657	14.8599514	-3.394997705	2.37E-25	1.73E-24	down	--
Solyc07g056400.1	5	1	4	113	125	74	68.31533	2.5573	134.073357	5.689778131	2.99E-25	2.16E-24	up	Putative serine/threonine protein kinase
Solyc07g056240.3	55	43	50	120	187	154	115.9303	38.5015	193.359085	2.331426162	3.04E-25	2.20E-24	up	--
novel.1125	286	164	358	639	604	864	550.5958	206.866	894.325838	2.109154269	3.10E-25	2.24E-24	up	FOG: Transposon-encoded proteins with TYA
Solyc09g063150.3	288	224	279	27	32	29	121.3571	205.37	37.3439356	-2.464430146	3.12E-25	2.25E-24	down	Glutathione S-transferase
Solyc07g056480.3	7	14	10	96	78	124	67.62285	8.1486	127.097102	3.972053132	3.36E-25	2.43E-24	up	Glutathione S-transferase
Solyc08g075560.1	68	57	77	205	168	225	154.2553	52.286	256.224581	2.287289256	3.50E-25	2.52E-24	up	--
Solyc05g007590.4	278	289	349	31	24	46	140.2373	237.539	42.9359595	-2.473966691	3.58E-25	2.58E-24	down	Cysteine desulfurase NFS1
Solyc07g045470.3	354	362	390	53	35	54	174.5799	287.853	61.3062801	-2.247402306	3.93E-25	2.82E-24	down	N-acetylglucosaminyltransferase
novel.538	325	246	298	26	42	35	134.4858	225.853	43.1187299	-2.381982536	4.02E-25	2.88E-24	down	--
Solyc04g078120.3	237	190	230	19	17	18	96.92943	170.683	23.1758491	-2.897398775	4.51E-25	3.23E-24	down	Guanidinoacetate methyltransferase
Solyc11g008905.1	56	78	73	152	236	191	148.4979	54.0208	242.975008	2.173725484	5.25E-25	3.75E-24	up	--
Solyc02g067350.3	27	18	25	80	125	149	82.75231	18.1399	147.364723	3.024454897	5.63E-25	4.02E-24	up	--
Solyc08g079440.1	290	244	298	35	24	27	126.7079	216.011	37.4050026	-2.55580651	5.82E-25	4.15E-24	down	UDP-glucose 4-epimerase/UDP-sulfoquinovose synthase
Solyc01g099440.4	235	217	292	27	17	16	109.4022	192.449	26.3549757	-2.905531887	7.38E-25	5.24E-24	down	ATP-dependent DNA helicase
Solyc02g088955.1	322	317	380	46	38	24	155.772	264.41	47.13401	-2.512937127	9.89E-25	6.99E-24	down	--
novel.1527	227	209	275	1	2	4	93.45264	184.047	2.8581397	-5.964360212	1.35E-24	9.51E-24	down	--
Solyc03g096760.1	3	8	14	58	153	220	90.90426	6.36241	175.446115	4.773354957	1.38E-24	9.73E-24	up	--
Solyc07g042400.2	73	78	93	439	266	263	244.4361	63.288	425.584167	2.745107058	1.58E-24	1.11E-23	up	--
Solyc01g150169.1	257	175	224	12	15	20	95.04066	170.368	19.7134944	-3.105315823	1.84E-24	1.29E-23	down	Sexual differentiation process protein ISP4
Solyc07g007755.1	638	547	783	4667	3197	3284	2684.855	508.385	4861.32515	3.257016068	1.86E-24	1.30E-23	up	--
Solyc01g058470.1	188	180	212	3	2	5	77.42781	150.609	4.24629806	-5.156522921	1.86E-24	1.30E-23	down	--
Solyc04g012030.3	4	10	11	78	83	89	56.37007	6.47287	106.267268	4.031998963	2.00E-24	1.40E-23	up	DHHC-type Zn-finger proteins
Solyc12g011450.2	9	6	15	63	120	94	61.42732	7.63273	115.221912	3.900322024	2.04E-24	1.43E-23	up	--
Solyc09g015140.2	17	25	32	80	145	146	86.54127	19.1094	153.973118	3.010932461	2.12E-24	1.48E-23	up	--
novel.1565	374	281	290	36	35	38	146.634	246.728	46.5400506	-2.414690094	2.21E-24	1.54E-23	down	--
Solyc02g062710.1	9	14	21	73	118	100	66.53745	11.2961	121.778773	3.422887121	2.35E-24	1.64E-23	up	--
Solyc04g082500.4	39	39	71	148	156	204	126.5439	38.136	214.951862	2.485784135	2.50E-24	1.74E-23	up	Serine/threonine protein kinase
Solyc03g114160.1	17	55	48	470	655	572	373.0605	31.3007	714.820311	4.514565511	2.60E-24	1.81E-23	up	FOG: Armadillo/beta-catenin-like repeats
Solyc03g083400.3	33	25	23	90	127	128	83.00586	21.2119	144.799833	2.779297811	2.72E-24	1.89E-23	up	--
Solyc07g049610.1	196	196	165	7	9	4	77.29258	146.021	8.56416359	-4.107101503	2.89E-24	2.00E-23	down	Acetylglucosaminyltransferase EXT1/exostosin 1
Solyc05g052990.4	166	157	191	7	6	5	70.57013	133.35	7.79047568	-4.128278596	3.60E-24	2.49E-23	down	--
Solyc04g074420.1	66	61	76	154	165	188	133.8718	52.6356	215.108049	2.02796607	3.91E-24	2.70E-23	up	--
Solyc09g098510.3	125	74	186	312	358	408	276.9307	98.0882	455.773157	2.209999947	4.05E-24	2.79E-23	up	--
Solyc08g007863.1	25	22	17	511	259	348	254.5186	16.8241	492.213204	4.873670029	4.27E-24	2.94E-23	up	Cytochrome P450 CYP4/CYP19/CYP26
Solyc00g500325.1	6	3	9	69	95	75	52.75534	4.57109	100.939592	4.439474648	4.68E-24	3.22E-23	up	--
Solyc04g082030.1	19	22	35	82	132	149	85.30038	19.5152	151.085599	2.948426879	4.70E-24	3.23E-23	up	Ornithine decarboxylase
novel.1060	164	185	279	9	14	14	88.52143	161.581	15.4613694	-3.376921132	4.70E-24	3.23E-23	down	--
Solyc10g008620.3	197	163	201	4	1	6	75.18823	145.628	4.74841201	-4.967834553	4.93E-24	3.38E-23	down	--
Solyc04g054830.4	148	206	222	6	5	2	77.68179	149.646	5.7176779	-4.76069945	5.73E-24	3.92E-23	down	Inositol polyphosphate 5-phosphatase
Solyc02g082635.1	507	449	531	77	55	40	230.8847	386.295	75.4741336	-2.376787423	6.04E-24	4.12E-23	down	--
Solyc06g083740.1	30	27	39	117	113	131	89.35577	24.7818	153.92973	2.626956649	6.37E-24	4.34E-23	up	--
Solyc07g043170.4	48	30	35	96	203	197	117.0686	29.4428	204.694449	2.803929542	6.70E-24	4.56E-23	up	UDP-glucuronosyl and UDP-glucosyl transferase
Solyc07g017530.4	315	271	310	25	50	45	141.3696	233.043	49.6963473	-2.213430428	6.94E-24	4.72E-23	down	Subunit of cis-Golgi transport vesicle tethering complex
Solyc03g116880.3	322	363	359	53	47	55	169.3246	272.27	66.3789541	-2.046528542	8.80E-24	5.97E-23	down	--
Solyc05g011890.1	176	344	256	1248	2295	1847	1224.117	203.731	2244.50397	3.46250965	9.51E-24	6.45E-23	up	Sulfotransferase
Solyc01g108210.4	186	148	218	8	14	8	77.57715	142.563	12.5915757	-3.496234731	9.74E-24	6.59E-23	down	Cytochrome P450 CYP4/CYP19/CYP26
novel.515	53	60	49	173	147	204	133.1678	42.4844	223.851218	2.399882282	1.17E-23	7.91E-23	up	FOG: Transposon-encoded proteins with TYA
Solyc07g053410.4	5	1	4	79	100	74	55.00648	2.5573	107.455665	5.371876673	1.28E-23	8.66E-23	up	--
Solyc06g084250.4	345	282	305	34	40	18	141.2346	242.885	39.5845748	-2.631563434	1.42E-23	9.55E-23	down	FOG: Armadillo/beta-catenin-like repeats

Solyc11g066090.1	171	170	189	9	4	12	74.30305	137.833	10.7726259	-3.700585242	1.48E-23	9.93E-23	down	--
novel.262	53	26	39	148	136	177	113.5393	30.6026	196.475932	2.679240062	1.51E-23	1.01E-22	up	Dehydrogenases with different specificities
Solyc09g010780.3	162	157	251	10	5	9	78.51783	146.564	10.4719169	-3.845070583	1.52E-23	1.02E-22	down	Protein phosphatase 2C/pyruvate dehydrogenase phosphatase
Solyc02g086980.3	63	73	73	261	178	235	173.175	54.48	291.870069	2.418556949	2.00E-23	1.34E-22	up	K+-channel ERG and related proteins
Solyc08g023270.3	185	198	303	11	23	18	99.01938	176.495	21.5440982	-3.018504456	2.05E-23	1.37E-22	down	--
Solyc02g093980.3	260	252	299	31	45	31	127.8574	210.528	45.1870992	-2.220783572	2.19E-23	1.46E-22	down	--
novel.1151	183	189	194	3	2	4	75.6834	147.516	3.8507136	-5.276999945	2.26E-23	1.51E-22	down	--
Solyc06g011530.3	265	254	257	31	35	32	122.0528	202.42	41.6851091	-2.285789456	2.45E-23	1.63E-22	down	Hydroxymethylglutaryl-CoA lyase
Solyc06g075690.3	307	255	315	25	11	36	129.2871	227.639	30.9355463	-2.893189985	2.59E-23	1.73E-22	down	--
Solyc07g064380.4	5	2	0	65	125	110	63.18888	1.88513	124.492624	6.095632512	2.84E-23	1.88E-22	up	--
Solyc04g079540.3	312	427	437	37	71	48	185.5429	306.062	65.0234506	-2.226341792	2.88E-23	1.91E-22	down	--
novel.56	2	3	2	81	126	81	61.59789	1.84476	121.351024	6.058893118	3.48E-23	2.29E-22	up	--
novel.1257	263	239	279	32	39	35	123.9295	202.932	44.9271793	-2.179101505	3.56E-23	2.35E-22	down	--
Solyc09g090020.4	225	203	191	15	20	17	91.90965	161.855	21.9643892	-2.88102624	3.96E-23	2.60E-22	down	--
Solyc05g055840.4	364	236	348	31	40	47	147.4327	245.298	49.5676633	-2.304110825	5.25E-23	3.42E-22	down	UDP-glucuronosyl and UDP-glucosyl transferase
Solyc05g055400.4	32	23	19	148	98	210	107.0777	19.436	194.719436	3.329157041	5.31E-23	3.46E-22	up	Cytochrome P450 CYP2 subfamily
Solyc02g085710.4	113	128	98	195	388	322	232.2207	89.0613	375.380044	2.082719967	5.36E-23	3.50E-22	up	Geranylgeranyl pyrophosphate synthase
Solyc11g068550.1	328	267	346	43	37	53	150.3326	243.938	56.7273411	-2.113078459	5.63E-23	3.67E-22	down	--
Solyc05g051330.1	154	180	190	9	4	11	73.36718	136.357	10.3770414	-3.742571777	5.80E-23	3.78E-22	down	--
Solyc09g090360.3	22	5	9	85	111	207	88.35361	9.37377	167.333452	4.161418973	5.82E-23	3.79E-22	up	Predicted small molecule transporter
Solyc09g065240.3	40	45	35	110	159	131	99.9489	31.5132	168.384564	2.42609295	5.87E-23	3.82E-22	up	Ca2+-independent phospholipase A2
Solyc05g014250.3	106	85	72	168	423	451	247.8266	69.0009	426.652203	2.634236327	6.15E-23	3.99E-22	up	Predicted E3 ubiquitin ligase
Solyc02g068000.2	379	367	424	68	61	47	190.043	303.971	76.115187	-2.014318701	6.17E-23	4.01E-22	down	--
Solyc01g107690.4	310	264	284	23	49	29	132.7795	223.574	41.9846645	-2.400189507	6.28E-23	4.07E-22	down	TATA-binding protein-interacting protein
Solyc05g018310.1	22	17	19	119	117	85	76.69561	15.1068	138.284448	3.191085637	6.77E-23	4.37E-22	up	FOG: Predicted E3 ubiquitin ligase
Solyc07g062730.1	197	173	222	9	19	21	86.79919	153.419	20.1792479	-2.902200911	6.89E-23	4.45E-22	down	--
Solyc03g098660.3	94	74	132	274	252	239	202.8824	77.0186	328.746189	2.085305806	7.71E-23	4.96E-22	up	--
Solyc05g014230.3	21	13	19	76	96	100	64.20828	13.7236	114.69297	3.060612	8.50E-23	5.46E-22	up	Predicted E3 ubiquitin ligase
Solyc03g080170.3	239	243	246	27	28	15	110.0399	189.833	30.2467233	-2.669918186	8.59E-23	5.52E-22	down	--
novel.2071	181	161	165	9	9	10	72.09534	132.26	11.9302442	-3.480437265	9.22E-23	5.92E-22	down	FOG: Transposon-encoded proteins with TYA
Solyc03g120070.4	169	163	176	7	1	5	69.04769	132.254	5.8416884	-4.578186534	9.37E-23	6.01E-22	down	FOG: PPR repeat
Solyc09g074280.1	201	211	291	1	2	3	91.98964	181.517	2.46255524	-6.166696115	9.66E-23	6.19E-22	down	FOG: Ankyrin repeat
Solyc12g010890.2	329	255	292	29	49	30	136.6799	228.002	45.3579707	-2.325516251	9.85E-23	6.30E-22	down	Transcription factor GT-2 and related proteins
Solyc01g008540.4	139	459	305	8	11	6	124.1737	237.716	10.6311344	-4.488767106	1.06E-22	6.79E-22	down	Flavonol reductase/cinnamoyl-CoA reductase
Solyc01g009160.2	276	206	356	32	27	26	126.083	215.476	36.6898297	-2.573619997	1.21E-22	7.71E-22	down	--
novel.1234	67	41	50	175	151	183	129.6104	41.1253	218.095548	2.40420445	1.38E-22	8.81E-22	up	FOG: Reverse transcriptase
novel.560	246	164	233	18	8	15	92.25107	166.517	17.9849916	-3.247650981	1.43E-22	9.11E-22	down	--
Solyc08g014140.1	0	1	1	337	545	454	279.8896	0.5174	559.26186	10.07410039	1.56E-22	9.88E-22	up	--
Solyc12g005720.1	63	65	82	228	164	227	160.6283	54.3853	266.87132	2.289159201	2.13E-22	1.34E-21	up	--
Solyc02g086970.4	1121	587	879	4991	4152	4386	3250.393	670.511	5830.27456	3.120075134	2.32E-22	1.46E-21	up	Aldehyde dehydrogenase
Solyc02g093525.1	23	29	25	123	93	143	87.00633	20.1534	153.859316	2.934049124	2.42E-22	1.52E-21	up	--
Solyc04g015590.4	16	16	19	88	102	80	64.15558	13.2359	115.075269	3.116097631	2.43E-22	1.53E-21	up	Flavonol reductase/cinnamoyl-CoA reductase
Solyc01g097650.3	40	17	31	238	672	706	341.0259	22.7359	659.315933	4.857108037	2.61E-22	1.64E-21	up	Tumor differentially expressed (TDE) protein
novel.677	190	155	188	9	6	16	75.78855	138.443	13.1344786	-3.401906939	2.83E-22	1.77E-21	down	--
Solyc01g008790.4	191	200	210	21	15	15	89.36055	156.519	22.2021547	-2.847206541	2.83E-22	1.78E-21	down	--
Solyc11g068710.3	192	236	362	23	9	13	111.5369	203.009	20.0650149	-3.384598146	3.15E-22	1.97E-21	down	--
Solyc01g090490.1	190	144	164	9	5	6	69.22359	129.658	8.78887658	-3.928900839	3.33E-22	2.08E-21	down	Mitochondrial transcription termination factor, mTERF
Solyc01g008660.4	247	236	297	29	13	30	116.7294	202.132	31.3267023	-2.714875586	3.51E-22	2.19E-21	down	--
novel.392	43	31	43	232	113	282	150.5177	30.2994	270.735982	3.155788512	4.35E-22	2.71E-21	up	--
Solyc09g015010.2	148	145	150	565	361	367	340.8757	115.467	566.284065	2.291499478	4.43E-22	2.75E-21	up	Molecular chaperone (small heat-shock protein Hsp26/Hsp42)
Solyc02g065000.1	58	33	34	108	193	241	128.4268	32.6956	224.158034	2.784177346	4.50E-22	2.80E-21	up	Calmodulin and related proteins (EF-Hand superfamily)

Solyc04g050050.3	19	22	36	73	162	157	90.61474	19.7531	161.476415	3.028724077	5.03E-22	3.12E-21	up	--
Solyc03g031910.3	302	253	229	767	1854	1654	981.426	205.293	1757.55911	3.098789885	5.24E-22	3.24E-21	up	tRNA-splicing endonuclease positive effector (SEN1)
Solyc03g026300.4	223	229	253	22	37	21	108.4942	183.342	33.6466124	-2.442774307	6.24E-22	3.85E-21	down	Predicted membrane protein
novel.878	10	19	7	63	117	110	65.00499	9.62799	120.381991	3.672742505	6.41E-22	3.95E-21	up	FOG: Transposon-encoded proteins with TYA
Solyc06g064880.3	3	1	3	73	79	99	53.98579	1.78893	106.182648	5.869638067	6.80E-22	4.18E-21	up	Carbon-nitrogen hydrolase
Solyc10g086570.3	427	343	398	27	70	66	185.2999	303.808	66.7913439	-2.166330967	7.02E-22	4.31E-21	down	--
Solyc08g150135.1	4	3	8	53	89	233	78.48275	3.80271	153.162801	5.313463781	1.05E-21	6.44E-21	up	--
Solyc03g113460.1	164	173	165	7	14	11	72.1937	131.105	13.2820421	-3.284056292	1.06E-21	6.44E-21	down	Reductases with broad range of substrate
Solyc10g050910.1	177	156	170	13	7	8	71.66817	130.992	12.3447082	-3.45426454	1.12E-21	6.86E-21	down	--
Solyc08g081420.4	326	258	310	40	41	53	144.5623	232.327	56.7975101	-2.035566874	1.19E-21	7.26E-21	down	Galactosyltransferases
Solyc03g123550.1	150	169	151	4	8	7	65.40783	122.943	7.87229867	-3.942069642	1.20E-21	7.28E-21	down	--
Solyc10g076760.3	155	145	215	6	11	14	72.79579	132.788	12.8032362	-3.352707401	1.31E-21	7.96E-21	down	Serine/threonine protein kinase
Solyc01g096720.4	0	6	1	1188	699	1049	639.4562	1.91484	1276.99746	9.415506033	1.31E-21	7.96E-21	up	Permease of the major facilitator superfamily
Solyc10g079280.2	180	162	214	12	21	16	82.20111	143.933	20.4697013	-2.804303355	1.46E-21	8.86E-21	down	Glucose-6-phosphate/phosphate
Solyc06g076560.2	4000	2844	3567	12513	9512	11577	8600.763	2704.43	14497.0928	2.422300029	1.62E-21	9.81E-21	up	Molecular chaperone (small heat-shock protein Hsp26/Hsp42)
Solyc07g044785.1	7	12	7	65	90	70	50.95181	6.87588	95.027735	3.806539601	1.63E-21	9.86E-21	up	--
Solyc04g008950.4	265	240	255	16	32	41	117.3318	198.032	36.6317927	-2.412923773	1.65E-21	9.98E-21	down	--
Solyc08g077090.3	401	374	404	39	39	81	186.801	307.004	66.5980731	-2.199578153	1.90E-21	1.15E-20	down	Predicted haloacid dehalogenase-like hydrolase (eyes absent)
novel.219	241	158	158	2	3	4	74.70732	145.67	3.74418411	-5.262851163	1.97E-21	1.19E-20	down	--
Solyc06g066420.4	77	75	180	261	409	346	255.0115	84.2091	425.813917	2.332350803	2.07E-21	1.25E-20	up	Nuclear localization sequence binding protein
Solyc12g099950.3	1	1	7	85	89	67	52.79354	2.21012	103.376963	5.476008789	2.09E-21	1.26E-20	up	FOG: Leucine rich repeat
Solyc06g008360.3	33	35	46	88	141	138	91.34926	29.4788	153.219708	2.378733912	2.15E-21	1.29E-20	up	--
Solyc10g085260.1	11	6	10	67	81	69	49.54527	6.97362	92.1169073	3.715792003	2.63E-21	1.58E-20	up	--
novel.1	5	4	2	52	78	84	46.17852	2.91994	89.4370976	4.965936389	2.79E-21	1.67E-20	up	--
Solyc12g008600.2	291	243	255	7	35	26	116.5835	205.766	27.4007156	-2.871836332	2.91E-21	1.74E-20	down	--
novel.1444	1	1	0	412	381	311	238.2697	0.54472	475.994581	9.835609208	3.05E-21	1.82E-20	up	--
Solyc10g054080.3	166	152	159	7	13	9	68.2201	124.339	12.1011157	-3.347879396	3.15E-21	1.88E-20	down	Kinesin-like protein
Solyc03g098100.4	16	23	56	119	167	248	123.1239	23.9952	222.252588	3.200038997	3.76E-21	2.24E-20	up	Aldo/keto reductase family proteins
Solyc04g009840.4	206	252	267	17	38	21	110.0734	188.592	31.5549351	-2.565421695	4.08E-21	2.43E-20	down	--
Solyc10g050160.2	23	17	9	82	110	230	93.77307	12.9929	174.553276	3.762912584	4.16E-21	2.47E-20	up	O-methyltransferase
Solyc08g016500.3	26	45	39	145	114	173	106.7909	28.7517	184.830069	2.685315668	4.59E-21	2.72E-20	up	K+-channel ERG and related proteins
novel.1608	342	318	362	59	39	44	163.7994	265.712	61.887187	-2.123995596	4.74E-21	2.81E-20	down	--
Solyc02g067660.4	4	5	3	64	71	67	44.55571	3.17211	85.939303	4.778015984	5.04E-21	2.98E-20	up	--
novel.629	2	0	0	318	361	335	215.7865	0.53046	431.042486	9.694579291	5.70E-21	3.36E-20	up	--
novel.227	267	230	279	31	28	45	122.7884	201.477	44.0994049	-2.195712963	5.94E-21	3.50E-20	down	--
Solyc10g007460.4	246	211	244	23	33	37	110.5917	182.27	38.9132209	-2.21982184	6.60E-21	3.88E-20	down	--
Solyc04g016010.4	146	167	190	11	15	11	73.12954	130.602	15.6569473	-3.063183597	6.66E-21	3.91E-20	down	Thioredoxin reductase
Solyc08g007867.1	3	5	9	103	61	79	55.23915	4.33437	106.143933	4.588801513	6.90E-21	4.05E-20	up	Cytochrome P450 CYP4/CYP19/CYP26
Solyc01g099980.4	30	48	52	105	165	141	102.9707	33.7439	172.197519	2.353415519	7.07E-21	4.15E-20	up	--
Solyc02g094450.2	207	184	226	23	24	25	95.37793	160.097	30.6583903	-2.393247676	8.12E-21	4.76E-20	down	--
Solyc01g111050.4	41	49	36	98	147	153	99.79462	33.1343	166.454889	2.339566069	8.49E-21	4.97E-20	up	--
Solyc00g500301.1	3	2	4	58	91	66	46.33374	2.30633	90.3611459	5.276754188	9.27E-21	5.42E-20	up	--
Solyc09g065580.4	178	195	236	20	23	24	93.12174	157.859	28.3841875	-2.479480641	9.42E-21	5.51E-20	down	--
Solyc01g005470.3	13	7	18	106	84	78	62.94426	9.68689	116.201631	3.562606865	9.60E-21	5.61E-20	up	--
Solyc03g114740.4	247	266	223	28	33	28	115.3727	192.911	37.8343955	-2.354541019	1.05E-20	6.12E-20	down	--
Solyc11g010870.2	270	238	299	35	39	19	124.6769	209.267	40.0866888	-2.400864129	1.21E-20	7.03E-20	down	--
Solyc06g073380.3	173	204	237	22	21	14	91.96392	159.286	24.6414019	-2.714705146	1.22E-20	7.11E-20	down	Predicted methyltransferase
Solyc07g063460.3	259	194	300	20	16	37	112.5443	194.29	30.7984833	-2.657806027	1.26E-20	7.29E-20	down	--
Solyc10g083360.2	197	217	242	13	34	22	99.44063	170.475	28.406342	-2.56320278	1.37E-20	7.91E-20	down	--
Solyc10g079490.2	234	238	288	26	45	40	121.6839	197.102	46.2659246	-2.080587647	1.40E-20	8.08E-20	down	Branching enzyme
Solyc01g096510.2	2	9	10	101	66	81	56.65816	5.425	107.891315	4.303086798	1.49E-20	8.61E-20	up	--
novel.617	157	156	209	12	18	14	76.73747	134.966	18.50926	-2.863994981	1.50E-20	8.66E-20	down	--

Solyc05g007470.4	149	169	181	8	16	14	72.78008	129.816	15.7445973	-3.023954348	1.51E-20	8.68E-20	down	AAA+-type ATPase
novel.839	150	126	168	4	10	8	62.00866	114.97	9.04739804	-3.638527069	1.68E-20	9.67E-20	down	--
Solyc02g088210.3	87	71	78	301	187	239	189.1439	61.4762	316.811702	2.361862657	1.73E-20	9.94E-20	up	Protein involved in vacuolar polyphosphate accumulation
Solyc07g006910.4	198	177	239	13	30	15	91.46254	158.847	24.0782209	-2.707608529	1.86E-20	1.07E-19	down	Glucose-6-phosphate/phosphate
Solyc07g032155.1	327	271	295	31	58	49	145.0157	232.657	57.3744664	-2.007677088	1.98E-20	1.14E-19	down	--
novel.502	24	16	11	87	98	88	64.81953	13.4544	116.184628	3.119925034	2.07E-20	1.19E-19	up	--
novel.1600	63	57	69	139	207	139	126.8532	49.0565	204.649919	2.060302266	2.52E-20	1.44E-19	up	--
Solyc02g062380.3	1	1	2	93	106	122	68.37541	1.02055	135.730281	7.030312429	2.54E-20	1.46E-19	up	GATA-4/5/6 transcription factors
Solyc06g009110.3	244	328	337	25	45	52	143.5413	236.566	50.5166512	-2.213238056	2.58E-20	1.47E-19	down	Aspartyl protease
Solyc03g044100.4	64	35	62	155	161	148	119.8648	41.5076	198.221928	2.248185939	2.60E-20	1.48E-19	up	--
novel.1049	276	250	333	36	21	45	133.077	222.302	43.8525375	-2.355770778	2.64E-20	1.51E-19	down	--
Solyc06g064430.1	134	156	160	7	7	11	63.88064	117.208	10.5537399	-3.472854927	2.73E-20	1.56E-19	down	--
Solyc02g061770.4	4	2	1	127	47	149	71.07347	1.85782	140.289128	6.259667785	2.74E-20	1.56E-19	up	Predicted chitinase
Solyc05g007130.4	233	227	268	32	25	39	115.0284	189.004	41.0529127	-2.214007935	2.75E-20	1.57E-19	down	--
Solyc02g080600.1	4	5	0	46	100	89	49.73516	2.45837	97.0119622	5.366875207	2.80E-20	1.60E-19	up	--
Solyc08g077060.3	325	247	320	48	43	40	143.8856	231.367	56.4047226	-2.051644819	2.90E-20	1.65E-19	down	--
Solyc08g007240.4	192	143	205	7	20	15	78.43335	139.664	17.2029247	-2.992569604	2.94E-20	1.67E-19	down	Predicted NUDIX hydrolase FGF-2 and related proteins
Solyc04g071900.4	17	7	32	106	190	95	89.15974	14.0786	164.240857	3.525007261	3.04E-20	1.72E-19	up	--
Solyc03g121470.4	6	4	9	49	82	70	44.40983	4.85057	83.9690841	4.09870096	3.17E-20	1.80E-19	up	Phospholipase D1
Solyc06g073990.2	124	164	180	8	5	10	65.71218	121.549	9.87492747	-3.640342857	3.21E-20	1.82E-19	down	--
Solyc01g090920.3	186	181	250	6	27	16	89.61482	159.399	19.8305244	-2.969864054	3.56E-20	2.02E-19	down	--
novel.620	172	144	147	10	3	7	64.87041	120.84	8.90123308	-3.821154244	3.71E-20	2.10E-19	down	--
novel.1033	187	192	239	3	1	1	81.19792	160.122	2.27420276	-6.240915321	4.47E-20	2.52E-19	down	--
novel.1243	35	41	68	135	134	166	110.9068	36.9203	184.893258	2.315130082	4.80E-20	2.70E-19	up	--
Solyc05g007150.4	300	272	389	34	37	65	152.5734	248.139	57.0077721	-2.117930487	4.80E-20	2.70E-19	down	UDP-galactose transporter related protein
Solyc05g054650.1	3	0	2	86	89	86	56.33044	1.27152	111.389355	6.421308379	4.98E-20	2.80E-19	up	FOG: Zn-finger
Solyc10g081680.1	174	163	167	10	12	19	74.29734	131.439	17.1560637	-2.927968561	5.01E-20	2.81E-19	down	--
novel.162	4	4	16	72	75	80	51.29837	5.98551	96.6112264	3.972118345	5.09E-20	2.86E-19	up	--
Solyc05g009820.4	190	189	241	0	4	1	81.25466	160.555	1.95461429	-6.255158762	5.14E-20	2.88E-19	down	--
novel.683	148	158	145	4	12	8	63.86897	117.911	9.82691295	-3.550013368	5.20E-20	2.92E-19	down	--
novel.864	2	2	6	68	80	57	44.99668	2.51693	87.4764233	5.078767958	5.75E-20	3.22E-19	up	--
Solyc10g009400.3	212	202	252	31	25	21	103.0382	172.64	33.4361055	-2.39336725	5.77E-20	3.23E-19	down	Predicted methyltransferase
Solyc07g056190.3	15	21	49	102	137	156	93.61738	21.5055	165.729216	2.932189071	6.07E-20	3.40E-19	up	--
Solyc08g081505.1	255	297	317	42	54	37	141.2943	226.061	56.5275795	-2.004812439	6.58E-20	3.67E-19	down	--
Solyc04g080260.4	495	544	566	1399	3804	3485	1986.773	417.991	3555.55465	3.088836198	6.82E-20	3.81E-19	up	--
Solyc01g111070.3	244	181	185	14	18	25	91.58567	159.318	23.853263	-2.731117008	6.94E-20	3.87E-19	down	--
Solyc01g096150.4	235	263	378	9	42	27	128.6419	225.767	31.5171761	-2.811145285	8.06E-20	4.47E-19	down	--
Solyc03g114860.4	14	13	23	65	112	81	60.38623	12.8186	107.953828	3.066275564	8.33E-20	4.62E-19	up	--
Solyc12g006380.2	2	1	4	64	65	88	46.83482	1.76161	91.9080319	5.664111149	9.04E-20	5.01E-19	up	Iron/ascorbate family oxidoreductases
Solyc08g008495.1	24	12	19	80	87	90	61.72713	14.2398	109.214456	2.934102843	9.16E-20	5.08E-19	up	--
Solyc06g062560.2	0	0	0	187	221	215	131.9964	0	263.992716	10.84267397	9.21E-20	5.10E-19	up	Predicted haloacid dehalogenase-like hydrolase
Solyc04g078500.1	57	59	61	139	162	133	115.4291	46.1208	184.737327	2.00093624	9.59E-20	5.31E-19	up	--
Solyc10g079150.3	5	3	6	55	66	63	40.76685	3.59211	77.9415953	4.424286034	1.17E-19	6.46E-19	up	CCAAT-binding factor, subunit B (HAP2)
Solyc08g080980.4	174	142	183	5	18	14	72.20563	129.376	15.0352514	-3.067096372	1.24E-19	6.85E-19	down	--
Solyc11g066790.2	210	231	243	30	33	33	109.4393	178.074	40.8048917	-2.131309398	1.30E-19	7.14E-19	down	Helicase-like transcription factor HLTF/DNA helicase
novel.1286	0	2	1	115	140	154	86.67797	0.79689	172.55905	7.790216578	1.40E-19	7.67E-19	up	--
Solyc11g056650.2	110	53	72	154	280	302	183.0725	61.1182	305.026785	2.322298323	1.47E-19	8.09E-19	up	--
Solyc09g150108.1	7	1	4	80	67	60	46.31977	3.08777	89.5517731	4.841260911	1.50E-19	8.20E-19	up	CCCH-type Zn-finger protein
Solyc10g007290.4	16	28	25	100	85	117	73.52935	18.0172	129.041461	2.840982688	1.54E-19	8.43E-19	up	--
Solyc01g101030.4	177	229	286	26	28	22	105.756	178.992	32.5195276	-2.472558359	1.59E-19	8.72E-19	down	Global transcriptional regulator, cell division control protein
Solyc03g044720.2	189	223	271	22	27	36	106.3062	176.93	35.6828048	-2.305761984	1.64E-19	8.98E-19	down	--

Solyc05g053970.2	0	5	2	89	71	68	50.30767	1.87327	98.7420612	5.751167999	1.66E-19	9.09E-19	up	Amino acid transporters
Solyc01g068360.4	18	11	21	64	88	88	56.8586	12.8447	100.872453	2.965391448	1.69E-19	9.26E-19	up	--
Solyc09g091670.3	10	11	18	216	79	97	93.18483	10.0092	176.360513	4.126072546	1.74E-19	9.48E-19	up	Pleiotropic drug resistance proteins (PDR1-15)
Solyc02g083570.3	28	16	28	74	117	209	91.78196	18.5599	165.004009	3.151791607	1.95E-19	1.06E-18	up	Ubiquitin-protein ligase
Solyc03g095200.1	223	217	282	35	36	35	116.0671	186.888	45.2467677	-2.05673298	2.00E-19	1.09E-18	down	FOG: PPR repeat
Solyc06g060760.3	15	4	9	50	98	89	52.72763	7.23766	98.2175951	3.762437264	2.07E-19	1.13E-18	up	Aquaporin (major intrinsic protein family)
novel.425	203	282	223	24	25	30	109.6175	185.713	33.522357	-2.472578379	2.09E-19	1.14E-18	down	Putative phosphoinositide phosphatase
Solyc02g092940.3	160	139	169	6	7	17	66.96222	121.493	12.4309597	-3.269562509	2.30E-19	1.25E-18	down	--
Solyc10g045290.2	290	206	210	17	21	34	107.2627	184.454	30.0716564	-2.607596713	2.36E-19	1.28E-18	down	--
Solyc08g066250.4	2170	1178	1442	7009	7181	6260	5000.772	1247.86	8753.68224	2.810413929	2.45E-19	1.33E-18	up	Glutamate decarboxylase and related proteins
novel.1294	163	128	131	6	7	6	59.12679	110.174	8.07953065	-3.777106367	2.50E-19	1.35E-18	down	--
Solyc05g006380.1	2	5	3	48	63	134	52.01323	2.64165	101.384811	5.284773904	2.52E-19	1.36E-18	up	--
Solyc06g082180.3	205	188	238	20	21	35	97.74802	163.54	31.9561017	-2.352540889	2.67E-19	1.44E-18	down	Glutaredoxin-related protein
Solyc06g072017.1	141	121	145	5	3	8	56.26437	105.713	6.81538279	-3.966145353	2.84E-19	1.53E-18	down	AAA+-type ATPase
Solyc08g067370.1	29	23	32	86	91	142	78.02741	21.7332	134.321599	2.625834676	2.95E-19	1.59E-18	up	--
Solyc01g008420.3	42	31	30	97	106	136	85.09746	26.9413	143.253611	2.414875138	3.05E-19	1.64E-18	up	Uncharacterized membrane protein
novel.1175	359	235	278	30	28	48	135.914	227.038	44.7898713	-2.342152896	3.13E-19	1.68E-18	down	--
Solyc12g011230.1	162	116	169	10	5	9	63.03382	115.596	10.4719169	-3.5012588	3.48E-19	1.87E-18	down	--
Solyc09g010260.4	24	27	27	79	93	103	68.26737	20.3354	116.199312	2.517113905	3.63E-19	1.95E-18	up	--
Solyc10g007680.4	172	163	184	14	20	21	79.00157	134.953	23.0504401	-2.542429149	3.78E-19	2.02E-18	down	FOG: RCC1 domain
Solyc03g025560.4	167	179	218	17	20	26	86.35234	146.187	26.5172232	-2.460686064	3.88E-19	2.08E-18	down	Cis-prenyltransferase
novel.1017	150	130	141	3	1	6	56.95815	109.664	4.25212506	-4.697603157	4.03E-19	2.15E-18	down	--
Solyc11g007930.1	203	243	219	24	30	14	101.5014	173.861	29.1417929	-2.588428295	4.03E-19	2.15E-18	down	Histone H2B
Solyc03g025230.3	228	189	221	26	32	21	99.77909	165.875	33.6829729	-2.308831041	4.07E-19	2.17E-18	down	Uncharacterized membrane protein
Solyc08g082060.3	253	194	243	21	18	38	105.8037	179.138	32.4698696	-2.463172745	4.53E-19	2.41E-18	down	--
novel.144	3	8	10	53	92	61	45.85115	5.41075	86.2915464	3.98987367	4.56E-19	2.42E-18	up	--
Solyc02g070300.4	247	225	253	25	34	47	116.4204	188.589	44.2513969	-2.081996332	4.66E-19	2.47E-18	down	Predicted telomere binding protein
Solyc03g026270.3	147	113	140	4	6	5	55.09044	103.879	6.30161484	-4.037632124	4.73E-19	2.51E-18	down	--
Solyc04g007340.4	242	271	257	26	44	46	124.6605	201.071	48.2496739	-2.046358546	5.42E-19	2.87E-18	down	--
Solyc03g044160.3	149	139	166	8	12	16	66.41947	117.862	14.9767364	-2.961285583	5.43E-19	2.88E-18	down	Serine/threonine protein kinase
Solyc06g011450.3	178	180	221	14	24	29	88.93619	150.098	27.7741456	-2.41792453	6.12E-19	3.23E-18	down	Clathrin assembly protein AP180
Solyc05g007430.4	127	155	164	10	8	12	64.42552	116.023	12.8279427	-3.190460698	6.17E-19	3.25E-18	down	--
Solyc02g037590.3	157	136	156	5	16	8	64.32433	116.766	11.8822297	-3.265270547	6.28E-19	3.31E-18	down	--
Solyc05g005865.1	22	15	21	80	73	116	64.53334	15.0236	114.043048	2.920882094	6.64E-19	3.50E-18	up	--
Solyc10g009410.1	252	186	307	33	33	31	116.6828	191.863	41.5025836	-2.221194071	7.19E-19	3.78E-18	down	Aspartyl protease
Solyc03g097400.1	169	174	181	18	10	19	78.43224	136.518	20.3468444	-2.769627263	7.27E-19	3.82E-18	down	--
Solyc05g054480.3	5	8	20	58	92	86	53.49148	8.32036	98.6625926	3.545219698	7.83E-19	4.11E-18	up	Actin and related proteins
Solyc07g052230.3	58	47	42	93	195	220	123.8489	38.5118	209.185971	2.450239919	8.02E-19	4.21E-18	up	Multicopper oxidases
novel.809	118	193	143	5	2	4	62.05182	119.26	4.8432875	-4.670758736	8.24E-19	4.32E-18	down	tRNA delta(2)-isopentenylpyrophosphate transferase
Solyc05g007940.3	15	8	32	66	151	126	77.6398	13.8276	141.451957	3.338146749	8.39E-19	4.40E-18	up	Ribonuclease, T2 family
Solyc01g088750.3	222	213	228	33	22	26	103.9471	172.657	35.2373293	-2.317467959	8.98E-19	4.70E-18	down	--
Solyc02g084305.1	250	256	303	43	38	24	127.7951	209.945	45.6451492	-2.223322478	9.34E-19	4.88E-18	down	--
Solyc03g117750.3	221	207	224	30	22	32	102.9425	169.763	36.1219752	-2.248001558	9.64E-19	5.04E-18	down	--
Solyc12g055850.2	154	147	188	15	13	18	73.14503	126.658	19.6316714	-2.701357273	1.14E-18	5.94E-18	down	--
Solyc06g066010.3	153	136	152	8	15	11	64.46097	114.754	14.1680865	-3.004081676	1.15E-18	5.99E-18	down	--
Solyc07g041340.2	215	202	204	23	33	19	96.90435	162.016	31.7927006	-2.352415075	1.32E-18	6.84E-18	down	--
Solyc01g065690.3	123	84	133	162	401	412	243.7076	87.7431	399.672023	2.189221091	1.39E-18	7.22E-18	up	Copper chaperone
Solyc04g081940.3	303	229	341	26	47	57	139.6336	225.497	53.7703754	-2.054616967	1.40E-18	7.27E-18	down	--
Solyc12g056160.2	193	153	187	14	25	16	80.73137	138.441	23.0213051	-2.580576332	1.49E-18	7.71E-18	down	Predicted K+/H+-antiporter
Solyc11g010760.1	395	373	471	14	45	72	187.0213	321.073	52.969184	-2.57810726	1.49E-18	7.74E-18	down	UDP-glucuronosyl and UDP-glucosyl transferase
Solyc03g117960.4	34	19	24	84	93	112	71.13955	20.0381	122.241007	2.609419794	1.53E-18	7.92E-18	up	--
Solyc01g096210.4	308	244	345	27	63	34	141.6857	231.967	51.404339	-2.162464707	1.61E-18	8.31E-18	down	FOG: PPR repeat
Solyc03g116910.3	3	5	13	123	60	77	60.08739	5.28603	114.888746	4.409225344	1.70E-18	8.81E-18	up	Flavonol reductase/cinnamoyl-CoA reductase
Solyc08g077210.4	334	201	285	18	36	43	126.2724	212.57	39.9745653	-2.394256334	1.89E-18	9.77E-18	down	Inositol polyphosphate 5-phosphatase
Solyc09g091000.4	15	5	39	1147	698	963	618.4471	14.6546	1222.23967	6.373540582	1.98E-18	1.02E-17	up	--

Solyc03g113320.1	5	2	2	56	55	72	40.03589	2.36096	77.7108104	5.050607836	2.00E-18	1.03E-17	up	--
Solyc12g098630.2	141	132	181	12	14	12	66.75586	117.353	16.1590613	-2.869313542	2.22E-18	1.14E-17	down	--
Solyc01g107780.4	55	35	65	181	125	182	125.1891	39.8343	210.543992	2.392814266	2.22E-18	1.14E-17	up	UDP-glucuronosyl and UDP-glucosyl transferase
Solyc07g007350.1	39	56	55	226	805	570	345.2397	39.0807	651.398745	4.06039653	2.26E-18	1.16E-17	up	--
Solyc01g150146.1	221	221	250	36	22	22	107.5028	179.862	35.1438523	-2.386873461	2.42E-18	1.24E-17	down	N-acetylglucosaminyltransferase complex
Solyc12g043100.2	230	213	214	13	30	36	101.9167	171.448	32.3854946	-2.379487184	2.47E-18	1.27E-17	down	FOG: PPR repeat
Solyc03g059110.1	149	111	162	7	7	0	57.64359	109.085	6.20231084	-4.19353841	2.64E-18	1.35E-17	down	--
Solyc07g063860.4	138	129	127	7	4	8	55.53441	102.871	8.19771415	-3.673506333	2.72E-18	1.39E-17	down	--
novel.97	1	0	3	102	60	158	68.74402	0.97898	136.509061	7.045238255	2.74E-18	1.40E-17	up	--
Solyc12g010500.3	3	4	3	52	53	71	38.58898	2.62739	74.5505632	4.83913915	3.03E-18	1.55E-17	up	FOG: Predicted E3 ubiquitin ligase
Solyc02g070800.2	328	215	291	21	47	44	131.2327	216.319	46.1463426	-2.212123974	3.18E-18	1.62E-17	down	--
Solyc06g011350.3	31	33	49	93	118	111	82.57953	29.1031	136.055941	2.218855547	3.60E-18	1.83E-17	up	Aquaporin (major intrinsic protein family)
Solyc09g010660.3	140	157	167	16	10	10	68.26891	120.744	15.7940103	-2.972659457	3.71E-18	1.88E-17	down	Branching enzyme
Solyc02g090930.3	4	4	7	41	61	82	40.20259	3.84428	76.5608955	4.305999812	3.71E-18	1.88E-17	up	Predicted lipase
novel.1849	176	158	185	19	12	21	78.63396	134.854	22.4138152	-2.608553012	3.88E-18	1.96E-17	down	--
Solyc01g107240.4	39	68	59	128	144	190	119.0985	43.3862	194.810851	2.170409314	3.92E-18	1.99E-17	up	Peroxisomal membrane protein MPV17
Solyc10g083950.1	266	212	291	25	36	52	123.0225	199.036	47.0088341	-2.071524779	3.96E-18	2.00E-17	down	--
Solyc02g084200.3	39	38	46	102	104	129	87.04755	31.9087	142.18644	2.152939854	4.36E-18	2.21E-17	up	--
Solyc01g103650.3	45	41	95	279	172	213	167.8791	45.9963	289.761831	2.644495711	4.72E-18	2.39E-17	up	Alpha/beta hydrolase
Solyc07g018230.2	206	185	189	24	26	23	91.22596	151.309	31.1430232	-2.289233247	4.85E-18	2.45E-17	down	Uncharacterized conserved protein
Solyc10g085850.1	157	82	164	246	307	483	268.1934	103.577	432.809423	2.061589634	5.43E-18	2.74E-17	up	--
Solyc08g041930.1	157	151	117	2	4	2	57.51143	111.68	3.34277264	-5.047692094	5.46E-18	2.75E-17	down	--
Solyc10g050990.2	1143	375	840	16	7	9	311.0226	607.816	14.2291535	-5.43830648	6.31E-18	3.17E-17	down	--
Solyc04g009180.3	119	112	166	5	10	5	55.358	102.359	8.35693161	-3.604863702	6.68E-18	3.35E-17	down	--
Solyc07g053840.1	1	1	1	118	117	97	71.6589	0.78263	142.535175	7.510565751	7.87E-18	3.92E-17	up	--
Solyc06g066650.4	8	8	6	86	49	73	48.22085	5.78525	90.6564586	3.97373968	8.06E-18	4.02E-17	up	--
novel.285	2	1	1	50	148	93	60.16784	1.04787	119.287806	6.847448188	8.95E-18	4.46E-17	up	--
Solyc02g065240.3	73	174	113	697	1172	778	602.6748	94.8772	1110.47245	3.550458121	1.01E-17	5.02E-17	up	--
Solyc11g068910.3	134	142	162	14	12	11	64.87353	113.771	15.9765358	-2.855375597	1.07E-17	5.32E-17	down	--
Solyc01g079750.3	143	139	147	12	10	14	63.57082	111.75	15.3912004	-2.872704605	1.25E-17	6.18E-17	down	MEKK and related serine/threonine protein kinases
Solyc04g056640.1	12	12	17	68	76	63	49.43604	10.5812	88.2909002	3.051579743	1.35E-17	6.69E-17	up	FOG: Leucine rich repeat
Solyc05g053060.1	251	154	230	24	26	16	96.3543	164.335	28.373932	-2.54948446	1.41E-17	6.95E-17	down	--
Solyc02g067850.2	2	1	6	71	53	59	40.73522	2.23744	79.2330017	5.088788318	1.47E-17	7.23E-17	up	FOG: Armadillo/beta-catenin-like repeats
Solyc02g085130.4	130	135	201	8	6	17	66.53282	120.032	13.0337762	-3.200525352	1.58E-17	7.78E-17	down	Tub family proteins
Solyc06g054620.4	146	99	147	8	6	6	55.02447	101.367	8.68234709	-3.577490696	1.76E-17	8.65E-17	down	CCCH-type Zn-finger protein
novel.994	177	145	145	10	19	11	69.34455	121.969	16.7196902	-2.85665976	1.95E-17	9.50E-17	down	--
Solyc03g123680.1	6	12	4	88	64	60	49.12485	5.89691	92.3527963	3.990067244	2.00E-17	9.75E-17	up	FOG: Predicted E3 ubiquitin ligase
Solyc01g060020.4	18	2	15	394	296	402	239.416	8.90186	469.930218	5.714515126	2.12E-17	1.04E-16	up	--
Solyc05g015370.3	32	40	67	95	244	169	122.3545	35.6072	209.101853	2.552373722	2.21E-17	1.08E-16	up	HORMA domain
Solyc03g031980.4	0	1	3	80	68	92	51.79673	0.99323	102.600233	6.627222394	2.31E-17	1.12E-16	up	--
Solyc06g016790.3	96	168	152	6	2	6	57.35501	108.579	6.13074337	-4.186930371	2.73E-17	1.32E-16	down	--
Solyc09g074370.4	159	196	216	19	27	27	89.48738	148.341	30.6336838	-2.270601441	3.01E-17	1.46E-16	down	Mitochondrial fatty acid anion carrier protein
Solyc01g094830.4	281	200	284	24	45	47	123.019	197.995	48.0424419	-2.029626764	3.25E-17	1.57E-16	down	--
novel.1822	149	169	194	17	24	19	79.10781	132.908	25.3071618	-2.39239824	3.66E-17	1.76E-16	down	--
Solyc09g098320.1	0	1	1	170	165	188	111.783	0.5174	223.04864	8.743672332	4.50E-17	2.16E-16	up	NADH:flavin oxidoreductase/
Solyc09g092010.1	182	139	230	16	14	26	82.76189	141.841	23.6823915	-2.585633809	4.55E-17	2.18E-16	down	Serine/threonine protein kinase
novel.461	262	176	238	29	36	30	107.7977	175.304	40.2911238	-2.125628687	4.76E-17	2.28E-16	down	--
Solyc06g076700.1	131	151	165	13	17	8	66.22313	116.204	16.2422828	-2.85117059	5.21E-17	2.49E-16	down	--
Solyc06g048590.3	220	229	248	39	25	34	111.9528	181.357	42.5489991	-2.113039107	5.52E-17	2.63E-16	down	Predicted membrane protein
Solyc06g005430.1	132	119	177	12	13	11	62.87714	110.381	15.3737194	-2.857413115	6.15E-17	2.92E-16	down	Histone H4
Solyc08g082470.1	103	112	157	4	7	8	51.92613	95.9741	7.87812567	-3.588098516	6.20E-17	2.94E-16	down	--
Solyc08g006740.3	5	8	30	77	134	95	69.36081	10.6995	128.022118	3.555008591	6.31E-17	2.99E-16	up	Glutamate decarboxylase and related proteins
Solyc05g047530.3	149	115	120	9	6	7	54.89231	100.21	9.5742185	-3.419961581	7.03E-17	3.33E-16	down	Cytochrome P450 CYP2 subfamily
Solyc11g011340.2	18	13	14	114	54	110	66.43811	11.7383	121.137905	3.364342492	7.90E-17	3.73E-16	up	Alcohol dehydrogenase, class V
Solyc05g043405.1	159	130	146	13	13	15	65.34658	113.241	17.4523442	-2.705906892	8.03E-17	3.80E-16	down	--

Solyc05g009760.3	162	159	230	19	27	23	85.58893	142.127	29.0513459	-2.289919291	8.18E-17	3.86E-16	down	Apoptotic ATPase
novel.84	2	2	3	171	28	101	68.76775	1.80318	135.732308	6.220780086	8.62E-17	4.06E-16	up	--
Solyc01g111700.2	0	3	2	53	95	56	43.3986	1.31429	85.4828964	6.040806544	9.23E-17	4.34E-16	up	26S proteasome regulatory complex
Solyc03g033510.3	137	138	111	5	8	10	55.43498	101.315	9.555339	-3.387565193	9.68E-17	4.55E-16	down	--
Solyc03g007510.4	182	170	193	21	15	29	84.7215	141.703	27.7403371	-2.362243468	9.73E-17	4.57E-16	down	--
Solyc05g054870.3	100	115	138	6	4	6	49.20339	91.4965	6.91025828	-3.754003111	9.90E-17	4.64E-16	down	--
Solyc05g049980.3	105	118	134	469	1906	1184	768.3589	92.7095	1444.00829	3.961567512	1.04E-16	4.88E-16	up	--
Solyc09g011630.3	32	19	32	230	83	147	113.0289	21.411	204.646783	3.248589244	1.12E-16	5.21E-16	up	Glutathione S-transferase
Solyc02g087780.3	57	51	46	99	126	159	100.728	40.3162	161.139777	2.005346926	1.21E-16	5.65E-16	up	Predicted mutarotase
Solyc02g085660.1	10	26	13	64	90	95	58.71648	13.0119	104.42106	3.022394305	1.29E-16	6.01E-16	up	UDP-glucuronosyl and UDP-glucosyl transferase
Solyc02g080040.4	292	228	384	40	20	47	139.3846	232.53	46.2390967	-2.345235336	1.32E-16	6.13E-16	down	--
Solyc04g082040.3	195	161	208	20	31	29	89.84209	146.204	33.4801695	-2.119641112	1.34E-16	6.21E-16	down	Predicted mechanosensitive ion channel
novel.1745	136	91	138	4	6	1	49.52821	94.3371	4.71927699	-4.342052352	1.37E-16	6.36E-16	down	--
Solyc12g088400.1	27	52	107	501	711	576	400.3827	47.1515	753.613962	3.995329818	1.45E-16	6.70E-16	up	--
Solyc02g080150.3	235	204	259	38	20	20	107.7653	180.965	34.5657424	-2.423344638	1.48E-16	6.85E-16	down	--
Solyc04g150161.1	320	155	497	1813	2467	1894	1428.488	246.438	2610.53685	3.40415042	1.65E-16	7.61E-16	up	--
Solyc02g082560.1	129	122	143	13	9	7	57.53143	102.334	12.7286387	-3.047722751	1.71E-16	7.87E-16	down	CTP synthase (UTP-ammonia lyase)
Solyc01g020495.1	147	154	188	11	26	15	74.1424	126.758	21.5266172	-2.540615023	1.77E-16	8.16E-16	down	FOG: Transposon-encoded proteins with TYA
Solyc12g070127.1	140	180	186	17	17	23	77.92681	131.692	24.1611975	-2.449799463	1.84E-16	8.49E-16	down	Transcription-associated recombination protein - Thp1p
novel.598	222	181	202	23	23	37	96.27159	157.528	35.0156463	-2.168647373	1.94E-16	8.93E-16	down	--
Solyc08g036507.1	147	98	122	6	4	8	51.55295	95.4045	7.7014272	-3.645870898	1.96E-16	9.01E-16	down	--
Solyc05g026460.1	186	124	165	6	6	20	68.23674	123.246	13.2279556	-3.200654468	1.98E-16	9.08E-16	down	--
Solyc03g118000.1	32	42	40	98	110	100	80.40519	29.7425	131.067887	2.139754949	2.02E-16	9.25E-16	up	Inositol polyphosphate multikinase
Solyc12g096350.2	139	119	133	1	0	5	52.12157	101.769	2.47420925	-5.331030449	2.57E-16	1.17E-15	down	--
Solyc06g009840.3	45	24	25	88	119	104	77.89309	24.591	131.195173	2.419781026	2.69E-16	1.23E-15	up	Fumarase
Solyc01g016460.4	210	182	188	9	33	13	86.88231	151.293	22.4711766	-2.72495515	2.83E-16	1.28E-15	down	--
Solyc09g066410.3	14	20	10	63	76	72	50.52594	11.6822	89.3697256	2.951967769	2.90E-16	1.32E-15	up	Inorganic phosphate transporter
novel.2055	183	129	203	10	23	22	77.75909	132.888	22.6301491	-2.532726259	2.96E-16	1.34E-15	down	--
Solyc07g066190.3	115	98	138	3	10	4	48.84624	90.7237	6.96877325	-3.670511113	3.60E-16	1.63E-15	down	Prenylated rab acceptor 1
novel.525	145	109	129	1	3	2	51.03525	99.6138	2.45672824	-5.301307426	3.63E-16	1.64E-15	down	--
Solyc08g036600.3	111	120	109	3	4	2	46.37552	88.912	3.83905959	-4.545672148	3.78E-16	1.71E-15	down	WD40 repeat protein
Solyc02g083120.3	139	98	128	8	8	7	52.28378	94.7101	9.85744646	-3.28111508	3.86E-16	1.74E-15	down	--
Solyc05g006850.4	73	30	36	111	176	256	130.6331	36.3115	224.954785	2.63542963	4.14E-16	1.87E-15	up	Thioredoxin
Solyc06g048930.4	58	21	55	129	145	139	104.93	34.338	175.522088	2.345636524	4.18E-16	1.88E-15	up	GATA-4/5/6 transcription factors
Solyc04g072200.3	123	159	157	11	14	2	63.06082	114.415	11.7069297	-3.314158317	5.78E-16	2.59E-15	down	--
Solyc04g015530.3	37	37	41	112	95	243	109.3236	29.9091	188.73812	2.659372814	6.02E-16	2.69E-15	up	--
Solyc04g010030.1	16	31	34	83	115	85	70.31777	20.9969	119.638603	2.50894989	6.19E-16	2.76E-15	up	Thioredoxin-like protein
Solyc04g008790.1	188	157	223	25	32	23	90.388	146.798	33.9778549	-2.117791803	6.31E-16	2.82E-15	down	Uncharacterized conserved protein
Solyc05g012910.4	29	19	29	68	83	99	62.58088	19.9015	105.260243	2.400303766	6.46E-16	2.88E-15	up	Apoptotic ATPase
novel.1675	154	111	146	13	9	13	60.85327	106.604	15.1021454	-2.841481922	7.42E-16	3.31E-15	down	--
Solyc05g014480.4	106	123	121	7	7	9	50.52092	91.2793	9.76257098	-3.230215754	7.50E-16	3.34E-15	down	Glyceraldehyde 3-phosphate dehydrogenase
novel.1339	116	110	109	1	2	7	45.74408	87.4433	4.04489308	-4.380842147	8.78E-16	3.89E-15	down	--
Solyc07g039343.1	163	145	172	21	20	12	73.82202	124.68	22.9641886	-2.464533793	9.23E-16	4.09E-15	down	FOG: Transposon-encoded proteins with TYA
Solyc07g064280.3	181	161	201	23	27	31	87.51327	140.825	34.2011694	-2.042567062	9.63E-16	4.26E-15	down	Tryptophan synthase beta chain
Solyc01g098070.1	2	0	1	64	104	78	51.96055	0.76838	103.152728	7.051437911	1.18E-15	5.19E-15	up	MADS box transcription factor
Solyc03g019960.3	111	116	129	7	12	4	51.14288	92.5523	9.73343596	-3.252136611	1.27E-15	5.60E-15	down	DNA replication factor
Solyc08g078100.1	122	137	163	10	15	2	60.51431	109.428	11.6004003	-3.255356484	1.29E-15	5.66E-15	down	Amino acid transporters
Solyc07g056200.3	10	3	14	102	49	76	53.30269	6.82159	99.7838031	3.843040627	1.33E-15	5.84E-15	up	--
Solyc04g082530.3	134	101	147	11	9	11	56.0306	98.7428	13.3184026	-2.909443699	1.47E-15	6.44E-15	down	PHD finger protein BR140/LIN-49
novel.1999	122	135	143	14	9	13	59.8547	104.111	15.5984324	-2.764061496	1.50E-15	6.56E-15	down	--
Solyc05g150116.1	184	156	237	20	27	35	91.5416	148.789	34.2946464	-2.111150625	1.82E-15	7.96E-15	down	--
Solyc08g150101.1	126	125	134	9	15	13	57.84566	100.236	15.4555424	-2.68621916	2.05E-15	8.93E-15	down	--
Solyc06g008620.1	17	9	13	87	59	65	51.00143	10.1172	91.8856444	3.174001453	2.19E-15	9.50E-15	up	--
Solyc08g016720.1	39	18	39	87	95	166	85.26222	24.6535	145.870943	2.560309112	2.21E-15	9.61E-15	up	Beta, beta-carotene 15,15'-dioxygenase

Solyc01g010310.4	21	6	12	59	77	69	48.34467	10.1018	86.5875819	3.096783537	2.23E-15	9.70E-15	up	--
Solyc10g078270.2	5	13	2	52	56	147	55.60979	5.43533	105.784255	4.318641244	2.25E-15	9.76E-15	up	--
novel.1709	11	16	16	70	53	84	49.91116	11.196	88.6263263	2.982639932	2.26E-15	9.80E-15	up	--
Solyc10g008320.1	142	147	160	19	18	11	68.80526	116.814	20.7965153	-2.513633132	2.34E-15	1.01E-14	down	--
Solyc03g005060.4	17	12	14	76	66	59	48.98744	11.1936	86.7812834	2.949300267	2.36E-15	1.02E-14	up	--
Solyc10g076380.2	0	1	1	105	127	151	80.92999	0.5174	161.34258	8.279754584	2.38E-15	1.03E-14	up	--
Solyc11g072720.2	115	151	158	4	18	12	62.02133	110.295	13.7477955	-2.963369005	2.45E-15	1.06E-14	down	--
Solyc03g033480.4	196	153	193	22	22	32	86.40815	140.665	32.1516796	-2.131473762	2.46E-15	1.07E-14	down	Predicted BBOX Zn-finger protein
Solyc02g071500.2	29	23	26	110	99	71	70.78489	20.3057	121.264049	2.572816306	2.53E-15	1.09E-14	up	Iron/ascorbate family oxidoreductases
Solyc03g034410.1	121	123	131	2	0	3	49.90811	97.6369	2.17932728	-5.528140485	2.62E-15	1.13E-14	down	--
Solyc05g005760.4	151	117	160	6	19	16	63.76445	110.816	16.7124647	-2.696958793	2.63E-15	1.14E-14	down	--
Solyc05g007930.3	115	122	184	15	8	8	61.05127	108.375	13.7270396	-3.027577684	2.65E-15	1.15E-14	down	Galactosyltransferases
Solyc12g019080.3	4	9	3	40	61	53	34.44136	4.29006	64.5926592	3.946650555	2.66E-15	1.15E-14	up	Inositol polyphosphate 5-phosphatase
Solyc02g092580.3	11	4	18	296	157	171	142.0279	8.31796	275.7378	5.037684797	2.71E-15	1.17E-14	up	--
Solyc12g036850.2	136	120	122	0	4	1	50.29514	98.6357	1.95461429	-5.553734197	3.24E-15	1.39E-14	down	--
Solyc01g095500.3	2	2	2	42	55	52	32.20819	1.56527	62.8511039	5.333289646	3.25E-15	1.39E-14	up	--
Solyc06g083330.2	7	1	2	58	54	43	34.72681	2.61194	66.8416775	4.677635228	3.37E-15	1.45E-14	up	--
Solyc12g056345.1	223	167	164	19	29	24	87.53281	144.839	30.2264453	-2.255158309	3.63E-15	1.55E-14	down	--
Solyc05g009560.4	62	47	36	143	131	124	104.6125	38.1452	171.079734	2.166670406	3.70E-15	1.58E-14	up	TBP-1 interacting protein
Solyc02g071310.3	6	8	5	68	46	50	38.23622	5.01687	71.4555785	3.839111335	3.78E-15	1.61E-14	up	--
Solyc01g109550.2	2	8	3	60	58	41	36.04111	3.48011	68.6021123	4.326391765	4.05E-15	1.73E-14	up	Mitochondrial transcription termination factor, mTERF
novel.557	118	95	116	4	6	10	46.86317	85.4468	8.27953714	-3.346054951	4.16E-15	1.78E-14	down	FOG: Reverse transcriptase
Solyc12g036160.3	21	26	27	92	80	76	63.08183	19.2603	106.903415	2.467367866	4.36E-15	1.86E-14	up	Multidrug resistance-associated protein
Solyc06g007275.1	122	108	116	6	10	11	50.6839	90.1411	11.2267253	-2.98879477	4.54E-15	1.94E-14	down	--
Solyc00g500062.1	8	6	15	57	65	50	40.38466	7.3675	73.4018138	3.29368267	4.66E-15	1.98E-14	up	NADH-ubiquinone oxidoreductase
Solyc01g090460.3	51	42	22	87	147	181	101.2858	30.4994	172.072098	2.508359472	4.70E-15	2.00E-14	up	Transcription factor HEX, contains HOX and HALZ domains
Solyc02g093600.3	335	110	167	3014	1762	2654	1695.885	159.328	3232.44266	4.342502395	4.91E-15	2.09E-14	up	Molecular chaperone (small heat-shock protein Hsp26/Hsp42)
Solyc03g006050.4	94	88	125	3	3	4	41.75327	79.2661	4.24047106	-4.231486874	5.25E-15	2.22E-14	down	FOG: Leucine rich repeat
Solyc05g044555.1	121	120	129	6	9	16	54.56874	96.3226	12.8148902	-2.888420112	5.27E-15	2.23E-14	down	FOG: Transposon-encoded proteins with TYA
Solyc01g005410.4	21	13	40	142	73	136	85.72216	18.7198	152.724528	3.011374062	5.29E-15	2.24E-14	up	--
Solyc05g005170.4	6	4	4	42	38	122	43.78857	3.661	83.9161393	4.527349814	5.36E-15	2.27E-14	up	--
Solyc04g005900.3	176	186	228	31	30	17	93.35633	152.91	33.8025549	-2.19846742	5.45E-15	2.31E-14	down	--
Solyc05g055343.2	169	142	155	14	20	25	73.0105	121.388	24.6327779	-2.290511776	6.07E-15	2.56E-14	down	UDP-glucuronosyl and UDP-glucosyl transferase
Solyc01g150174.1	240	161	223	23	41	27	99.89179	161.708	38.075436	-2.080438598	6.84E-15	2.88E-14	down	--
Solyc11g010420.3	129	141	195	15	13	23	70.81283	120.016	21.6095937	-2.478091043	7.22E-15	3.03E-14	down	Phospholipase/carboxyhydrolase
novel.1297	1	3	1	46	49	60	33.502	1.34161	65.6623826	5.653733244	8.18E-15	3.43E-14	up	--
Solyc08g007480.2	110	78	126	1	4	5	42.49302	80.9528	4.03323908	-4.273227833	8.27E-15	3.46E-14	down	--
novel.1986	108	119	111	0	5	1	45.3285	88.3126	2.34437174	-5.133688776	9.10E-15	3.80E-14	down	--
Solyc06g005300.4	187	159	166	3	0	0	67.50981	133.531	1.48886085	-6.710741105	9.53E-15	3.98E-14	down	--
Solyc00g500130.1	4	4	1	37	57	45	30.39844	2.41679	58.3800928	4.634109364	9.95E-15	4.15E-14	up	--
Solyc04g078700.3	268	193	196	29	34	38	107.1654	171.655	42.6762845	-2.007857515	1.03E-14	4.28E-14	down	Serine/threonine protein kinase
Solyc02g077950.3	15	31	27	58	102	107	64.96687	19.0663	110.867441	2.548122206	1.03E-14	4.29E-14	up	--
Solyc02g082630.2	125	127	159	15	18	11	62.64437	106.477	18.8113675	-2.513794507	1.04E-14	4.31E-14	down	Putative phosphoinositide phosphatase
Solyc10g017970.1	321	290	517	3405	2225	1766	1772.431	289.193	3255.66956	3.49234423	1.04E-14	4.32E-14	up	Predicted chitinase
Solyc12g099210.2	1	10	14	85	69	54	48.41507	6.39092	90.439216	3.806055281	1.12E-14	4.63E-14	up	--
Solyc07g047710.3	9	6	20	77	62	60	47.46822	8.82231	86.114125	3.25814445	1.14E-14	4.71E-14	up	--
novel.584	103	112	122	10	8	8	49.44636	87.6471	11.2456048	-2.987806914	1.23E-14	5.09E-14	down	--
Solyc06g072460.1	93	129	140	8	10	11	53.12394	94.0286	12.2192992	-2.942215269	1.45E-14	5.98E-14	down	--
Solyc09g091990.3	152	200	246	23	35	30	95.83175	154.74	36.9236446	-2.063393174	1.48E-14	6.09E-14	down	Serine/threonine protein kinase
Solyc09g089680.4	12	17	13	48	73	62	43.91364	11.027	76.8003044	2.811128362	1.62E-14	6.66E-14	up	Iron/ascorbate family oxidoreductases
Solyc02g079570.4	13	16	14	65	57	63	45.32364	11.2506	79.3966477	2.819242383	1.76E-14	7.24E-14	up	--
Solyc11g013850.2	132	98	114	9	8	10	50.53158	89.5227	11.5404868	-2.968210141	1.87E-14	7.67E-14	down	--

Solyc06g082225.1	10	21	25	101	71	75	60.96802	14.4694	107.466596	2.883088835	1.99E-14	8.13E-14	up	--
Solyc12g011310.2	4627	2279	2770	12661	11766	14611	9586.232	2523.2	16649.2598	2.722130063	2.06E-14	8.42E-14	up	Glutathione S-transferase
novel.782	166	137	144	16	22	19	70.3047	116.578	24.03136	-2.276497019	2.20E-14	8.96E-14	down	--
Solyc12g010160.1	15	15	6	61	60	66	44.6829	9.59828	79.7675256	3.072223932	2.20E-14	8.99E-14	up	--
Solyc01g007130.3	132	120	142	14	14	17	60.7313	102.333	19.1295575	-2.426374507	2.25E-14	9.16E-14	down	--
Solyc07g007770.2	95	131	142	12	9	7	53.9131	95.5938	12.2323517	-2.999981434	2.25E-14	9.19E-14	down	--
Solyc02g063380.1	2	0	1	89	61	63	46.81747	0.76838	92.8665643	6.891150005	2.31E-14	9.41E-14	up	--
Solyc10g008100.3	89	129	109	8	3	5	46.35489	85.5923	7.11749026	-3.643276783	2.40E-14	9.76E-14	down	Protein kinase C substrate, 80 KD protein, heavy chain
Solyc12g038810.3	107	84	136	4	1	1	43.49184	84.2132	2.77048971	-5.043058389	2.51E-14	1.02E-13	down	Tyrosine kinase specific for activated (GTP-bound) p21cdc42Hs
Solyc08g081120.4	88	124	167	3	12	10	53.92523	97.7287	10.1217949	-3.231858308	2.63E-14	1.07E-13	down	Kinesin (KAR3 subfamily)
Solyc02g093760.4	13	8	10	50	65	48	38.59985	8.06306	69.1366362	3.099369504	2.74E-14	1.11E-13	up	Predicted E3 ubiquitin ligase
Solyc02g080530.3	22	31	26	85	70	98	64.45985	20.685	108.23469	2.388652629	2.81E-14	1.14E-13	up	--
novel.1570	4	1	4	76	28	65	38.31804	2.29207	74.3440068	4.992004582	2.88E-14	1.17E-13	up	--
Solyc09g061794.1	88	107	106	3	2	2	40.76206	78.4646	3.05954468	-4.724597491	2.90E-14	1.18E-13	down	--
novel.448	1	0	0	89	139	149	78.77657	0.26523	157.287909	9.138143956	2.91E-14	1.18E-13	up	--
Solyc11g068390.1	2	4	3	33	86	38	33.64549	2.36216	64.9288201	4.796798847	3.24E-14	1.31E-13	up	--
Solyc09g074990.4	122	124	178	15	14	19	64.89031	109.364	20.4170134	-2.429893646	3.26E-14	1.32E-13	down	DNA mismatch repair protein - MLH2/PMS1/Pms2 family
Solyc02g092845.1	216	170	243	32	19	38	100.4675	162.616	38.3187835	-2.100386198	3.67E-14	1.48E-13	down	--
Solyc02g078650.4	4	3	18	42	107	67	47.61706	6.18186	89.0522585	3.816073739	3.79E-14	1.53E-13	up	--
Solyc08g081620.4	317	467	476	860	1996	2157	1192.943	327.847	2058.03834	2.650573051	3.84E-14	1.54E-13	up	--
Solyc07g065190.4	151	199	254	24	11	30	92.0821	156.098	28.0657526	-2.491872397	3.96E-14	1.59E-13	down	Copper chaperone
Solyc06g009330.3	146	165	152	13	28	15	72.15055	121.002	23.298706	-2.363359426	4.51E-14	1.80E-13	down	Tandem pore domain K+ channel
Solyc02g070850.1	91	79	121	5	4	4	40.31306	75.0033	5.62280241	-3.76779327	4.51E-14	1.81E-13	down	--
Solyc07g007750.3	13	6	8	82	41	66	44.90621	7.02826	82.7841599	3.551769406	4.80E-14	1.92E-13	up	--
Solyc12g096900.2	51	38	78	148	117	144	109.3605	42.7047	176.016253	2.031704679	5.43E-14	2.16E-13	up	--
Solyc11g018710.1	8	5	6	37	62	43	32.24225	4.94678	59.5377112	3.59368229	5.46E-14	2.17E-13	up	--
novel.695	5	0	1	43	51	45	30.29167	1.56407	59.0192698	5.239984227	5.58E-14	2.22E-13	up	--
novel.1748	142	151	180	20	24	26	76.12768	122.69	29.5651139	-2.054018055	5.65E-14	2.24E-13	down	--
novel.564	4	7	1	31	70	49	32.65341	3.25526	62.0515559	4.301716425	5.67E-14	2.25E-13	up	--
novel.656	171	131	122	11	20	13	64.69502	110.993	18.3969035	-2.583388001	5.84E-14	2.32E-13	down	FOG: Transposon-encoded proteins with TYA
Solyc09g090560.2	111	132	143	13	16	14	59.29049	100.355	18.2260321	-2.464905263	6.11E-14	2.42E-13	down	--
Solyc10g055375.1	108	118	133	4	14	14	53.1236	93.2673	12.9799346	-2.805856876	6.16E-14	2.44E-13	down	--
Solyc07g042160.3	152	121	148	11	24	12	64.45249	109.345	19.5603489	-2.470459466	6.39E-14	2.53E-13	down	--
Solyc04g055080.3	124	99	126	4	15	10	51.16132	90.5353	11.7873542	-2.903918973	6.42E-14	2.54E-13	down	Sugar transporter/spinster transmembrane protein
Solyc06g082400.1	0	0	0	59	94	49	42.65088	0	85.3017694	9.21377103	6.52E-14	2.58E-13	up	--
Solyc05g056170.3	12	2	25	246	456	361	226.1558	9.68962	442.62198	5.50163566	6.53E-14	2.58E-13	up	Phenylalanine and histidine ammonia-lyase
novel.877	7	7	4	64	34	61	36.95473	4.7647	69.1447703	3.868927652	6.59E-14	2.61E-13	up	--
Solyc06g068510.1	80	128	119	6	8	7	47.08487	85.3049	8.86487257	-3.265848156	7.87E-14	3.10E-13	down	MEKK and related serine/threonine protein kinases
novel.236	135	108	108	7	14	10	52.28611	91.6858	12.8864576	-2.814811019	8.03E-14	3.16E-13	down	--
Solyc04g072760.3	19	22	32	64	73	85	56.32037	18.8014	93.8393382	2.312958894	8.12E-14	3.20E-13	up	Sulfate/bicarbonate/oxalate exchanger SAT-1
Solyc03g005190.3	276	156	232	20	38	38	105.8845	172	39.7687318	-2.100488982	8.25E-14	3.24E-13	down	--
Solyc08g006270.1	96	82	127	1	8	7	42.48943	78.5954	6.38343782	-3.561154487	8.56E-14	3.36E-13	down	--
Solyc03g115830.2	4	1	2	40	47	41	28.10264	1.81624	54.3890413	4.900586665	8.67E-14	3.40E-13	up	--
Solyc12g100240.1	4	0	2	92	25	71	42.51279	1.53676	83.4888324	5.741694063	8.85E-14	3.48E-13	up	--
novel.1767	230	218	248	1	1	0	90.91029	180.935	0.88604441	-7.742562318	9.62E-14	3.77E-13	down	--
Solyc01g079170.3	7	18	16	79	54	70	49.31926	10.694	87.9444838	3.036720148	9.70E-14	3.80E-13	up	Glycosyl transferase, family 8 - glycogenin
Solyc08g061950.1	125	118	135	12	19	10	57.78436	98.252	17.3166797	-2.505004556	1.01E-13	3.96E-13	down	--
Solyc11g070040.3	101	99	101	6	7	9	43.87669	78.4871	9.26628403	-3.079278011	1.01E-13	3.96E-13	down	FOG: PPR repeat
Solyc10g084830.2	135	104	99	9	4	10	49.20402	88.4266	9.98145697	-3.173662768	1.07E-13	4.19E-13	down	Amino acid transporters
Solyc11g009030.1	193	132	156	19	25	20	76.14095	125.197	27.0850776	-2.210624056	1.08E-13	4.24E-13	down	--
novel.828	0	2	1	54	79	52	39.47881	0.79689	78.1607262	6.646045184	1.16E-13	4.53E-13	up	--
Solyc08g028780.1	84	87	104	2	2	4	37.34624	71.3381	3.35442665	-4.402546295	1.21E-13	4.74E-13	down	--

Solyc03g120920.1	0	0	4	42	57	54	32.68672	0.95166	64.4217877	5.967064776	1.23E-13	4.81E-13	up	--
Solyc01g108490.3	6	2	10	54	36	63	35.14105	4.52951	65.7525847	3.826317022	1.38E-13	5.35E-13	up	Serine carboxypeptidases (lysosomal cathepsin A)
Solyc07g045520.4	121	156	179	13	18	26	71.01623	118.28	23.7525605	-2.305332953	1.43E-13	5.56E-13	down	--
Solyc01g099630.4	24	31	21	64	77	91	58.89889	20.0259	97.7718747	2.297889314	1.52E-13	5.88E-13	up	--
novel.879	15	10	17	53	89	48	45.39879	10.8179	79.979676	2.880654266	1.66E-13	6.40E-13	up	--
Solyc08g007980.3	7	13	11	62	51	50	39.26684	8.10703	70.4266441	3.117533027	1.89E-13	7.27E-13	up	--
Solyc10g008460.3	85	118	101	6	8	6	44.01148	79.5537	8.46928811	-3.233683739	1.94E-13	7.46E-13	down	Lanthionine synthetase C-like protein 1
Solyc03g013304.2	16	10	17	49	64	57	41.44699	11.0831	71.8108519	2.689168339	2.21E-13	8.48E-13	up	--
Solyc12g039100.2	27	23	26	47	130	95	65.67487	19.7753	111.57448	2.504443323	2.32E-13	8.88E-13	up	--
Solyc06g060270.4	91	112	141	8	6	14	50.41587	88.9847	11.8470228	-2.91208352	2.47E-13	9.44E-13	down	--
Solyc02g091380.1	12	3	4	44	44	62	34.24255	4.9729	63.5121904	3.680414482	2.49E-13	9.54E-13	up	--
Solyc02g079580.4	138	130	115	13	17	13	59.25791	100.296	18.2202051	-2.462631868	2.54E-13	9.71E-13	down	--
Solyc02g086410.3	113	81	95	6	0	3	39.68804	75.2116	4.16447507	-4.282604973	2.55E-13	9.77E-13	down	--
Solyc01g020500.2	94	122	132	5	16	7	50.96036	90.4341	11.4866453	-2.949466107	2.65E-13	1.01E-12	down	--
novel.1616	80	90	137	2	8	0	41.5387	78.9668	4.11063355	-4.235301409	2.72E-13	1.04E-12	down	--
Solyc10g076810.1	95	97	94	4	6	9	41.27764	74.6713	7.88395268	-3.223808729	2.95E-13	1.12E-12	down	--
Solyc04g150112.1	89	115	104	7	10	6	45.11749	80.4899	9.74508997	-3.048895031	3.05E-13	1.16E-12	down	--
Solyc09g091230.4	180	151	162	20	33	23	80.18638	128.487	31.8861776	-2.005020714	3.07E-13	1.17E-12	down	Branching enzyme
Solyc11g005760.3	4	3	7	38	45	40	27.89308	3.56479	52.221368	3.849972896	3.11E-13	1.19E-12	up	Glycosyl transferase, family 8 - glycogenin
Solyc12g042960.3	13	16	8	59	57	55	41.53869	9.82314	73.2542503	2.911038158	3.32E-13	1.26E-12	up	--
Solyc04g015470.3	104	93	85	2	0	6	38.58266	73.7992	3.36608066	-4.449261499	3.34E-13	1.27E-12	down	Phosphatidylinositol-4-phosphate 5-kinase
novel.486	127	68	93	3	2	4	39.33319	74.8157	3.8507136	-4.296330949	3.51E-13	1.33E-12	down	--
Solyc02g084303.1	133	133	158	12	23	5	63.46802	110.038	16.8977872	-2.704296329	3.66E-13	1.39E-12	down	--
novel.453	3	1	0	71	43	42	34.84284	1.07518	68.6104913	6.029404941	4.18E-13	1.58E-12	up	--
Solyc01g087360.3	9	17	8	56	68	48	41.16267	9.0417	73.2836303	3.034401257	4.30E-13	1.63E-12	up	Putative transcription factor HALR/MLL3
Solyc03g120930.1	215	329	273	662	1956	1913	1030.794	213.927	1847.66062	3.111123324	4.33E-13	1.64E-12	up	--
Solyc08g006410.4	1	4	0	52	41	42	29.89235	1.38318	58.4015244	5.470495339	4.68E-13	1.77E-12	up	UDP-glucuronosyl and UDP-glucosyl transferase
Solyc12g098220.2	119	115	122	14	14	6	53.75371	92.7293	14.7781284	-2.676750828	4.73E-13	1.78E-12	down	--
novel.876	24	15	20	52	92	62	50.75351	15.3162	86.1908439	2.494853248	4.86E-13	1.83E-12	up	--
Solyc12g099860.2	30	20	30	70	95	66	59.27988	20.6842	97.8756192	2.237921532	5.11E-13	1.92E-12	up	Conserved ATP/GTP binding protein
Solyc06g053700.1	194	168	162	30	25	25	85.73664	136.951	34.5221564	-2.004206405	5.19E-13	1.95E-12	down	--
Solyc09g014990.4	24	30	85	435	329	409	270.441	34.973	505.90907	3.849509243	5.23E-13	1.96E-12	up	--
Solyc06g067980.3	26	15	34	94	72	79	62.57106	19.1774	105.964682	2.450188156	5.29E-13	1.99E-12	up	--
Solyc11g068720.2	12	13	17	42	56	71	40.80882	10.8607	70.7569661	2.703375143	5.50E-13	2.06E-12	up	--
Solyc07g049495.1	21	25	28	70	79	65	55.2313	19.2187	91.2439154	2.243403132	5.61E-13	2.10E-12	up	--
Solyc09g014720.3	93	78	93	5	1	2	36.12752	68.5927	3.66236112	-4.326767869	6.08E-13	2.27E-12	down	--
Solyc09g074820.4	15	22	18	53	61	140	59.93496	14.4097	105.460238	2.880002036	6.48E-13	2.42E-12	up	--
novel.1214	1	1	1	48	50	62	34.30926	0.78263	67.8358829	6.442154512	6.98E-13	2.60E-12	up	--
Solyc03g097100.1	87	105	175	10	9	12	53.63709	94.0565	13.2177001	-2.84250616	9.03E-13	3.34E-12	down	Calmodulin and related proteins (EF-Hand superfamily)
Solyc08g005337.1	111	108	145	0	0	0	47.06153	94.1231	0	-8.651033863	9.05E-13	3.35E-12	down	--
Solyc01g049770.3	88	82	83	3	2	5	35.12581	66.0053	4.24629806	-3.964618285	9.24E-13	3.42E-12	down	--
Solyc05g015150.3	128	133	162	13	27	19	67.07752	109.664	24.4912864	-2.148651067	9.51E-13	3.51E-12	down	--
Solyc03g034090.3	121	106	153	17	15	9	57.98161	98.1197	17.8435001	-2.488016975	9.60E-13	3.55E-12	down	--
Solyc06g066410.3	25	55	38	86	145	105	85.88764	31.0434	140.731877	2.188630321	1.04E-12	3.82E-12	up	Profilin
Solyc08g081110.2	101	132	158	8	18	18	59.6889	101.271	18.1064501	-2.461699802	1.04E-12	3.84E-12	down	Kinesin (KAR3 subfamily)
Solyc04g014740.3	131	124	143	20	9	7	59.81316	103.424	16.2026473	-2.728357518	1.11E-12	4.07E-12	down	--
Solyc10g075103.1	6	9	18	42	58	58	37.39157	8.38925	66.393883	2.968731951	1.23E-12	4.51E-12	up	--
Solyc01g100010.3	33	59	64	154	105	145	107.5907	40.469	174.71247	2.105090611	1.28E-12	4.69E-12	up	--
novel.1738	1	3	1	36	59	33	27.62896	1.34161	53.9163073	5.369278585	1.34E-12	4.90E-12	up	--
Solyc06g069460.3	0	0	1	63	107	86	53.61415	0.23791	106.990389	8.581855456	1.37E-12	5.03E-12	up	--
Solyc03g116190.2	4	3	6	34	36	46	26.21439	3.32688	49.1019099	3.867093467	1.48E-12	5.40E-12	up	Predicted chitinase
novel.1728	137	97	161	11	18	19	60.87112	101.751	19.9908954	-2.337655146	1.55E-12	5.66E-12	down	--
Solyc01g091590.3	172	338	431	40	47	50	150.2887	242.628	57.9493014	-2.067508361	1.61E-12	5.88E-12	down	--
Solyc04g074700.4	144	87	149	10	11	18	57.16441	97.9581	16.3707218	-2.577609413	1.63E-12	5.93E-12	down	Transcription factor HEX

Solyc09g008145.1	3	2	3	52	26	47	28.30075	2.06842	54.5330848	4.707395949	1.64E-12	5.96E-12	up	--
novel.1574	78	138	140	5	11	13	52.23843	92.5655	11.9113647	-2.933115487	1.64E-12	5.97E-12	down	--
Solyc09g031595.1	9	9	11	37	55	47	32.95564	7.51954	58.3917468	2.957444169	1.71E-12	6.22E-12	up	--
Solyc08g074670.3	172	145	158	16	22	34	76.85063	123.736	29.9651269	-2.033849778	1.75E-12	6.36E-12	down	--
novel.1682	139	104	135	12	11	21	58.30124	98.0524	18.5500491	-2.401370147	1.76E-12	6.38E-12	down	--
Solyc06g011403.1	102	106	95	7	10	11	45.50214	79.2813	11.7230123	-2.7478794	1.89E-12	6.85E-12	down	Uncharacterized conserved protein
Solyc07g062170.3	87	62	107	4	4	3	35.29561	65.8603	4.730931	-3.823149676	2.14E-12	7.75E-12	down	Serine/threonine protein kinase
Solyc10g007560.2	4	13	12	32	91	59	41.11892	7.54925	74.6885941	3.314885062	2.17E-12	7.85E-12	up	Iron/ascorbate family oxidoreductases
Solyc06g074900.3	111	108	123	15	8	13	52.29695	88.8889	15.7049619	-2.53157002	2.18E-12	7.90E-12	down	--
Solyc01g081400.3	229	188	156	26	10	28	89.13695	150.396	27.8774001	-2.450588425	2.21E-12	7.98E-12	down	--
Solyc10g005060.4	16	5	8	37	72	53	37.4678	7.54446	67.3911304	3.163958461	2.27E-12	8.21E-12	up	Hydroxyindole-O-methyltransferase
Solyc11g042710.3	115	100	113	0	0	0	42.6674	85.3348	0	-8.508468691	2.35E-12	8.47E-12	down	Dehydrogenases with different specificities
Solyc07g026650.3	927	427	827	39	14	28	298.9273	561.967	35.8881603	-3.979516286	2.39E-12	8.61E-12	down	Iron/ascorbate family oxidoreductases
Solyc06g071300.1	139	176	136	18	25	11	70.72102	118.414	23.0285306	-2.368841536	2.48E-12	8.93E-12	down	MADS box transcription factor
Solyc11g013270.2	7	8	9	38	40	53	30.82447	6.23376	55.4151787	3.151459071	2.50E-12	8.98E-12	up	--
novel.993	103	78	142	7	5	13	46.7341	82.9028	10.5653939	-2.975360992	2.52E-12	9.06E-12	down	--
Solyc03g083190.3	148	124	150	21	24	16	67.85178	109.598	26.1055562	-2.081945883	2.61E-12	9.37E-12	down	--
novel.1386	10	9	12	127	36	60	54.40873	8.02269	100.794779	3.640561869	2.73E-12	9.80E-12	up	--
Solyc09g090980.3	1180	883	1999	6318	4333	5265	3971.232	1035.35	6907.11218	2.737706089	2.78E-12	9.97E-12	up	--
Solyc10g074540.1	132	87	202	11	18	13	62.5011	107.385	17.6173886	-2.605577046	2.80E-12	1.00E-11	down	--
novel.735	102	66	105	4	0	7	37.61756	70.4809	4.75423902	-3.917293039	2.96E-12	1.06E-11	down	--
Solyc02g082120.3	39	46	29	80	90	115	75.18668	30.1	120.27334	2.007579871	3.19E-12	1.14E-11	up	--
Solyc02g083785.1	119	105	106	14	11	9	50.46169	86.1278	14.7956094	-2.569210039	3.39E-12	1.21E-11	down	--
Solyc03g078030.1	100	71	88	2	6	7	36.70176	67.3033	6.10020986	-3.422604957	3.66E-12	1.30E-11	down	--
Solyc02g085630.3	3	1	2	26	44	43	24.30697	1.55101	47.0629205	4.921927867	3.68E-12	1.31E-11	up	Transcription factor HEX
Solyc08g060970.4	15	7	18	70	44	66	44.10767	10.2174	77.9979889	2.913975165	3.75E-12	1.34E-11	up	--
Solyc09g011590.4	29	34	58	165	79	138	99.1311	30.9934	167.268841	2.421739179	4.37E-12	1.55E-11	up	Glutathione S-transferase
Solyc05g006770.4	113	73	89	6	8	4	39.61316	71.5482	7.67811919	-3.231131733	4.40E-12	1.56E-11	down	Serine/threonine protein kinase
Solyc01g006950.3	19	13	14	56	49	64	42.10557	12.0036	72.20759	2.587774485	4.44E-12	1.57E-11	up	SNARE protein Syntaxin 1 and related proteins
novel.821	0	0	0	44	30	62	29.02779	0	58.055586	8.656134481	4.52E-12	1.60E-11	up	--
Solyc05g050510.3	5	10	9	28	49	79	35.2538	6.26227	64.2453223	3.369264044	4.62E-12	1.63E-11	up	Predicted Yippee-type zinc-binding protein
novel.420	0	0	1	82	66	75	48.16314	0.23791	96.0883564	8.41847578	4.90E-12	1.73E-11	up	--
Solyc02g067800.4	7	14	12	50	55	44	36.14058	8.62443	63.6567238	2.884925657	5.16E-12	1.82E-11	up	--
Solyc02g070380.1	4	5	4	32	53	31	26.10574	3.41003	48.8014458	3.850140923	5.30E-12	1.86E-11	up	--
Solyc06g084280.1	95	77	114	8	10	7	42.23841	73.8399	10.6369614	-2.805068536	5.50E-12	1.93E-11	down	--
Solyc08g068230.4	1	4	9	35	39	47	27.34374	3.52442	51.1630536	3.828950569	5.51E-12	1.94E-11	up	FOG: PPR repeat
Solyc10g080370.1	5135	5946	7537	843	488	638	2838.959	4816.96	860.954422	-2.484977284	5.57E-12	1.96E-11	down	--
Solyc08g081840.3	113	114	129	8	21	15	55.30639	92.5238	18.0889691	-2.330588814	5.73E-12	2.01E-11	down	--
Solyc02g078350.3	173	135	145	13	27	28	73.08257	118.114	28.0515465	-2.054789816	5.79E-12	2.03E-11	down	FOG: PPR repeat
Solyc05g006430.3	23	19	23	59	97	53	52.46801	16.8826	88.0533797	2.383301379	5.81E-12	2.04E-11	up	Rho GTPase effector BNI1 and related formins
Solyc02g020880.2	162	136	150	22	29	16	72.60787	116.665	28.5506305	-2.038225874	5.82E-12	2.04E-11	down	--
Solyc04g009610.2	115	116	155	11	10	24	59.3249	99.799	18.850758	-2.39868414	5.83E-12	2.05E-11	down	--
novel.871	89	112	114	13	9	7	47.3796	82.0306	12.7286387	-2.724761521	5.92E-12	2.07E-11	down	--
novel.663	6	1	6	43	39	35	26.84235	3.29837	50.3863357	3.903520515	5.97E-12	2.09E-11	up	--
Solyc12g017480.3	97	72	86	7	1	2	35.4831	66.3113	4.65493502	-3.942989346	6.62E-12	2.31E-11	down	FOG: Leucine rich repeat
Solyc09g082220.1	3	3	5	25	35	57	25.71037	2.82373	48.5969989	4.098162061	6.72E-12	2.35E-11	up	--
Solyc10g084670.3	107	89	128	9	15	13	49.58142	83.7073	15.4555424	-2.428693966	7.39E-12	2.57E-11	down	Translation initiation factor 3, subunit a (eIF-3a)
Solyc01g110907.1	100	116	143	11	17	18	56.08557	92.9656	19.2055535	-2.265985631	7.72E-12	2.68E-11	down	--
Solyc09g082110.4	198	130	121	21	14	16	69.92248	117.637	22.2079817	-2.427297787	8.38E-12	2.90E-11	down	--
Solyc02g150145.1	95	121	135	16	13	10	54.04842	91.1336	16.9632827	-2.45255147	8.52E-12	2.95E-11	down	--
Solyc01g087630.2	9	7	18	58	41	60	38.56287	8.62597	68.4997664	2.966114133	8.95E-12	3.09E-11	up	Flavonol reductase/cinnamoyl-CoA reductase
Solyc06g051090.3	3	1	3	43	29	37	24.53443	1.78893	47.2799301	4.697512805	9.20E-12	3.18E-11	up	FOG: Armadillo/beta-catenin-like repeats
Solyc07g007620.3	2	3	5	20	44	56	25.89315	2.5585	49.2277968	4.258800784	9.28E-12	3.20E-11	up	--
Solyc08g023540.1	89	68	77	4	2	4	32.63862	60.9302	4.34700055	-3.843268203	9.30E-12	3.21E-11	down	--
novel.1370	1	0	1	54	83	56	40.90262	0.50315	81.3020939	7.292625661	9.51E-12	3.28E-11	up	FOG: Reverse transcriptase

novel.1496	430	241	307	48	22	62	155.6843	254.446	56.9226741	-2.168972706	1.01E-11	3.46E-11	down	FOG: Transposon-encoded proteins with TYA
Solyc01g091250.3	2	1	2	37	27	51	25.17333	1.28578	49.0608759	5.238782808	1.01E-11	3.49E-11	up	--
Solyc10g007300.2	97	86	81	7	8	2	38.20889	69.0345	7.38323721	-3.253821248	1.16E-11	3.97E-11	down	--
Solyc02g092680.1	92	89	100	6	5	13	41.56816	73.0672	10.069107	-2.852904106	1.16E-11	3.99E-11	down	--
novel.1469	130	114	124	11	23	16	58.29805	95.8432	20.7529293	-2.19190874	1.17E-11	4.01E-11	down	--
Solyc00g500066.1	3	5	2	37	33	36	24.06731	2.66897	45.4656537	4.114261565	1.17E-11	4.01E-11	up	--
Solyc04g010050.4	109	127	149	9	13	25	59.63879	99.8545	19.423041	-2.344311853	1.21E-11	4.15E-11	down	FOG: PPR repeat
Solyc12g010750.2	4	4	6	26	54	35	25.70109	3.60637	47.7958194	3.725273692	1.33E-11	4.55E-11	up	Secretory carrier membrane protein
Solyc02g088980.3	86	64	103	7	3	5	35.91179	65.2024	6.62120331	-3.34962962	1.40E-11	4.79E-11	down	--
Solyc08g078990.3	81	65	90	6	3	2	33.00049	61.0628	4.93816298	-3.699696246	1.44E-11	4.90E-11	down	Uncharacterized conserved protein containing PAP2 domain
novel.414	155	107	153	17	7	22	63.64256	107.417	19.8680384	-2.453171633	1.44E-11	4.90E-11	down	--
Solyc01g005590.2	19	19	31	74	55	79	53.56905	17.725	89.4130667	2.322250153	1.46E-11	4.97E-11	up	--
Solyc08g079790.1	83	90	72	5	6	4	35.35017	64.298	6.40231732	-3.338304442	1.46E-11	4.98E-11	down	Glucose-6-phosphate/phosphate
Solyc02g093250.3	1	1	8	27	60	37	26.93493	2.44804	51.42182	4.337610327	1.55E-11	5.28E-11	up	O-methyltransferase
Solyc01g108220.3	80	81	101	8	7	7	38.67707	67.8865	9.46768901	-2.862959441	1.56E-11	5.29E-11	down	--
Solyc01g087870.4	141	158	148	26	16	24	72.70088	116.768	28.633607	-2.04825887	1.61E-11	5.47E-11	down	Glucosamine 6-phosphate synthetases
Solyc02g086230.2	75	75	94	7	2	5	34.7247	63.218	6.23144586	-3.401378408	1.63E-11	5.54E-11	down	FOG: PPR repeat
novel.1705	91	83	106	9	11	7	42.03778	72.5525	11.5230058	-2.666416865	1.67E-11	5.66E-11	down	--
Solyc06g062980.3	141	124	131	8	24	22	62.62417	103.221	22.0273327	-2.19904832	1.68E-11	5.69E-11	down	Small Nuclear ribonucleoprotein splicing factor
Solyc03g063650.1	119	123	125	19	12	17	58.25521	95.6789	20.8314773	-2.222910694	1.71E-11	5.80E-11	down	--
Solyc01g058530.4	98	105	92	7	5	14	44.09404	77.2271	10.9609784	-2.813957012	1.80E-11	6.08E-11	down	Mitochondrial/chloroplast ribosomal protein S18
Solyc12g056000.1	2	4	0	38	44	28	24.36651	1.64842	47.084597	4.896662947	1.81E-11	6.11E-11	up	Cysteine proteinase Cathepsin L
Solyc02g071940.1	108	124	134	19	16	11	57.59956	95.1821	20.0170004	-2.275713422	1.87E-11	6.31E-11	down	--
Solyc01g098140.4	430	1414	1166	49	53	63	428.2758	786.654	69.8970267	-3.492882448	1.89E-11	6.38E-11	down	Arylacetamide deacetylase
Solyc01g111270.3	1	13	4	32	79	52	36.04633	4.85024	67.2424134	3.825951064	1.89E-11	6.39E-11	up	--
Solyc12g087980.3	109	149	214	15	16	29	73.31005	121.468	25.1523729	-2.268099635	1.97E-11	6.62E-11	down	--
Solyc04g074280.1	128	143	190	14	13	33	72.0947	119.12	25.0691514	-2.241551392	2.01E-11	6.75E-11	down	--
Solyc10g005360.4	112	199	207	20	31	20	82.24617	134.572	29.9199093	-2.16815903	2.04E-11	6.85E-11	down	Iron/ascorbate family oxidoreductases
Solyc04g080120.1	95	87	104	7	10	13	43.38489	74.2556	12.5141812	-2.557345667	2.09E-11	7.01E-11	down	FOG: PPR repeat
novel.1073	80	62	83	3	5	2	31.26126	58.2937	4.22881705	-3.788072743	2.12E-11	7.12E-11	down	--
Solyc08g016220.1	0	0	2	53	75	51	38.09283	0.47583	75.709825	7.192464504	2.26E-11	7.58E-11	up	FOG: Leucine rich repeat
Solyc08g008080.1	15	18	21	45	62	59	41.92141	14.0055	69.8373581	2.318377821	2.36E-11	7.89E-11	up	FOG: Predicted E3 ubiquitin ligase
Solyc12g056730.1	21	22	39	67	65	89	57.39488	20.9973	93.7924772	2.148902682	2.41E-11	8.06E-11	up	--
Solyc06g007180.3	10	22	29	57	64	79	50.09229	15.7006	84.4840056	2.422251894	2.44E-11	8.16E-11	up	Asparagine synthase (glutamine-hydrolyzing)
Solyc06g054610.2	105	67	95	2	8	9	38.4239	69.1769	7.67089369	-3.127780601	2.45E-11	8.17E-11	down	--
Solyc03g113260.1	81	65	78	3	5	1	31.02053	58.2078	3.83323259	-3.935568642	2.48E-11	8.27E-11	down	--
novel.959	1	0	3	57	29	37	27.60346	0.97898	54.2279473	5.709564865	2.74E-11	9.13E-11	up	--
novel.1007	0	0	0	43	30	35	23.43926	0	46.8785186	8.342661802	3.21E-11	1.06E-10	up	--
Solyc05g009490.3	16	18	31	85	52	78	54.97861	16.6498	93.3073663	2.471730157	3.21E-11	1.07E-10	up	--
Solyc10g051050.3	3	2	0	33	32	39	22.81609	1.35467	44.2775019	5.078770587	3.22E-11	1.07E-10	up	Apoptotic ATPase
Solyc07g018390.3	161	110	113	15	17	19	60.95831	100.33	21.5862857	-2.217852857	3.96E-11	1.31E-10	down	--
novel.151	91	62	77	3	6	4	32.59676	59.7838	5.40974343	-3.450360591	3.99E-11	1.32E-10	down	--
Solyc09g064460.1	0	0	0	30	42	32	21.95856	0	43.9171244	8.255970913	4.02E-11	1.33E-10	up	--
Solyc09g074675.1	28	47	31	104	100	76	74.2959	27.9378	120.654007	2.112601368	4.17E-11	1.37E-10	up	--
Solyc10g005960.1	38	20	23	64	67	127	64.62797	21.1406	108.115341	2.358405731	4.18E-11	1.38E-10	up	--
novel.824	13	6	13	30	52	66	34.7412	8.21783	61.2645706	2.895519901	4.26E-11	1.41E-10	up	--
novel.1956	103	92	89	1	0	2	37.74681	74.2062	1.28745587	-5.870463448	4.30E-11	1.41E-10	down	--
Solyc03g114650.3	2	0	6	29	39	35	22.69813	1.95795	43.4383184	4.4061663	4.39E-11	1.44E-10	up	--
Solyc12g044730.2	81	76	87	7	6	7	36.00254	63.4234	8.5816446	-2.903006497	4.43E-11	1.46E-10	down	40S ribosomal protein S15/S22
Solyc08g005580.3	5	3	5	14	52	90	33.0861	3.3542	62.8180064	4.229533505	4.45E-11	1.46E-10	up	Transporter, ABC superfamily
Solyc01g097470.4	1	5	4	39	29	36	23.75676	2.61433	44.8991978	4.102564529	4.53E-11	1.49E-10	up	--
Solyc08g015820.1	77	76	88	0	0	0	31.30021	62.6004	0	-8.061791259	4.59E-11	1.51E-10	down	--
Solyc07g150148.1	66	66	94	2	0	5	30.64299	58.3155	2.9704962	-4.302762236	4.62E-11	1.52E-10	down	--
Solyc02g005110.3	88	95	105	3	12	13	43.09067	74.8728	11.3085483	-2.684316021	4.62E-11	1.52E-10	down	--

Solyc09g091770.1	0	0	1	64	59	52	37.78318	0.23791	75.3284466	8.067233822	4.76E-11	1.56E-10	up	--
novel.1457	0	0	0	40	25	40	22.7094	0	45.4187928	8.298208793	4.90E-11	1.61E-10	up	--
Solyc01g099450.4	86	68	82	7	4	5	34.16754	61.3241	7.01096077	-3.169065401	4.99E-11	1.63E-10	down	--
novel.1825	140	78	115	6	12	16	50.13842	86.2927	13.9841625	-2.603314556	5.26E-11	1.72E-10	down	--
Solyc12g010780.2	3	6	4	26	45	34	23.65835	3.42428	43.8924179	3.69770284	5.29E-11	1.73E-10	up	FOG: Predicted E3 ubiquitin ligase
Solyc07g048090.2	106	75	82	1	11	3	37.27777	68.5852	5.97037235	-3.461632233	5.50E-11	1.80E-10	down	--
novel.1298	2	2	0	30	38	37	22.71273	1.08944	44.3360168	5.406512333	5.54E-11	1.81E-10	up	--
novel.471	1	0	0	63	62	48	37.34216	0.26523	74.4190942	8.049992358	5.56E-11	1.82E-10	up	--
Solyc01g058160.3	105	86	113	10	16	10	46.96225	78.7697	15.1548334	-2.376160758	5.60E-11	1.83E-10	down	--
Solyc05g026500.1	113	66	104	9	3	9	41.17831	73.1605	9.19611505	-3.027846191	5.89E-11	1.92E-10	down	--
novel.512	72	71	75	5	1	4	30.61873	56.7839	4.45353004	-3.733827947	5.93E-11	1.93E-10	down	--
Solyc02g079530.4	13	9	16	70	39	53	40.33832	9.77004	70.9066036	2.843047882	5.96E-11	1.94E-10	up	--
novel.992	94	57	75	3	3	5	31.67112	58.7062	4.63605552	-3.660990397	5.99E-11	1.95E-10	down	--
Solyc02g084540.3	79	86	59	4	2	3	31.48883	59.0262	3.95141609	-3.943782013	6.07E-11	1.98E-10	down	Uncharacterized conserved protein
Solyc01g104090.4	83	69	97	6	6	9	36.62658	64.3766	8.87652657	-2.861123082	6.08E-11	1.98E-10	down	FOG: PPR repeat
Solyc11g007600.2	0	0	0	36	30	33	21.30667	0	42.613341	8.20681233	6.56E-11	2.13E-10	up	--
Solyc01g105110.1	1	1	0	37	54	74	34.61374	0.54472	68.6827698	7.04713893	6.56E-11	2.13E-10	up	--
Solyc07g008820.3	63	91	76	2	7	2	32.36829	60.2245	4.51204501	-3.705299429	6.62E-11	2.15E-10	down	--
Solyc09g092520.3	72	76	64	2	2	4	29.45937	55.5643	3.35442665	-4.040239935	7.04E-11	2.28E-10	down	--
Solyc01g006660.3	113	88	84	5	14	7	42.62904	74.551	10.7071304	-2.77524648	7.25E-11	2.35E-10	down	--
novel.842	67	82	81	0	0	0	29.97982	59.9596	0	-7.998882134	7.64E-11	2.47E-10	down	--
Solyc01g104290.3	2	2	11	36	38	42	26.49908	3.7065	49.2916608	3.685796969	7.72E-11	2.50E-10	up	--
Solyc09g014940.3	4	4	1	42	34	27	23.59669	2.41679	44.7765858	4.237583403	7.75E-11	2.51E-10	up	--
Solyc03g083460.3	34	17	38	89	62	99	65.15365	22.8099	107.497362	2.22508051	8.03E-11	2.60E-10	up	--
novel.1893	108	76	88	1	1	1	36.05212	70.8226	1.28162887	-5.804259497	8.69E-11	2.80E-10	down	--
Solyc12g014020.2	87	70	77	0	8	5	33.02737	60.9587	5.09598195	-3.499352966	8.80E-11	2.84E-10	down	Predicted nucleic acid binding protein
Solyc02g092375.1	5	3	3	48	33	26	24.92367	2.87837	46.9689656	4.024952666	9.09E-11	2.93E-10	up	--
Solyc01g108040.1	104	81	95	10	12	8	42.81458	72.8245	12.8046346	-2.518432631	9.60E-11	3.09E-10	down	Vacuolar sorting protein VPS1
Solyc01g095620.3	20	7	20	64	43	78	45.69843	12.0193	79.3775232	2.70973022	9.88E-11	3.18E-10	up	UDP-glucuronosyl and UDP-glucosyl transferase
Solyc11g033283.1	99	86	85	9	7	11	41.03149	70.5167	11.5463138	-2.622108811	9.94E-11	3.20E-10	down	H+/oligopeptide symporter
novel.647	19	17	22	47	67	52	42.51723	15.0248	70.0096281	2.218584573	1.14E-10	3.65E-10	up	--
Solyc10g005750.3	18	14	18	49	46	62	39.8713	12.9695	66.77314	2.360252374	1.18E-10	3.78E-10	up	Calmodulin and related proteins (EF-Hand superfamily)
Solyc09g090910.3	17	13	26	39	61	83	45.14599	14.3281	75.963906	2.402064866	1.23E-10	3.93E-10	up	--
Solyc11g008260.2	152	60	59	0	2	3	36.54386	71.1214	1.96626829	-5.091398414	1.33E-10	4.25E-10	down	Cysteine proteinase Cathepsin F
Solyc04g057950.2	9	12	13	36	47	47	31.80562	8.83383	54.7774002	2.633579476	1.34E-10	4.26E-10	up	--
Solyc01g090790.4	17	11	11	27	71	70	39.4819	10.2004	68.7634392	2.765973786	1.37E-10	4.37E-10	up	--
Solyc01g099620.3	103	70	63	6	4	1	33.40199	61.8716	4.93233598	-3.712342534	1.39E-10	4.41E-10	down	Ferric reductase, NADH/NADPH oxidase
Solyc06g062520.1	68	63	77	3	5	4	29.49146	53.9629	5.01998597	-3.416428997	1.39E-10	4.42E-10	down	--
novel.264	11	9	6	32	40	52	29.45115	6.86043	52.0418725	2.938383302	1.40E-10	4.46E-10	up	MADS box transcription factor
Solyc06g064560.4	83	79	112	11	10	6	41.23523	70.7402	11.7302378	-2.621542773	1.41E-10	4.46E-10	down	--
Solyc12g099550.1	173	159	149	32	15	22	78.10169	125.773	30.4304023	-2.076014234	1.50E-10	4.77E-10	down	--
Solyc01g095160.3	161	100	130	15	17	24	62.57211	101.58	23.564208	-2.105536458	1.51E-10	4.77E-10	down	FOG: PPR repeat
Solyc07g049290.3	124	79	112	6	15	15	48.18629	81.6147	14.7578504	-2.441979014	1.54E-10	4.89E-10	down	H+/oligopeptide symporter
Solyc03g096545.1	55	25	94	300	239	351	212.4136	43.9389	380.888262	3.11133166	1.61E-10	5.09E-10	up	--
novel.409	0	0	0	31	35	25	19.45801	0	38.9160179	8.077736142	1.61E-10	5.10E-10	up	--
Solyc12g013710.2	7	11	14	39	41	49	31.49034	8.2618	54.7188853	2.721064948	1.72E-10	5.41E-10	up	Dehydrogenases with different specificities
Solyc01g094090.4	0	0	2	72	38	47	34.80587	0.47583	69.1359133	7.056808457	1.82E-10	5.74E-10	up	--
Solyc07g054340.1	79	86	127	6	15	3	42.60764	75.2044	10.0108369	-2.900224144	1.97E-10	6.18E-10	down	--
Solyc01g107680.4	127	86	118	7	19	3	48.93024	85.7943	12.0661537	-2.818806847	2.21E-10	6.93E-10	down	Putative ATP-dependent Clp-type protease
Solyc12g014560.3	115	132	122	22	16	12	59.16056	96.4197	21.9014457	-2.167001099	2.34E-10	7.32E-10	down	--
Solyc08g080560.3	1	1	0	35	59	49	30.14705	0.54472	59.7493717	6.845270629	2.34E-10	7.33E-10	up	--
Solyc02g062690.3	25	32	18	55	93	64	54.35875	19.8569	88.8606311	2.175051807	2.37E-10	7.40E-10	up	--
Solyc06g072013.1	79	78	110	4	14	7	39.56742	68.924	10.2108434	-2.724691573	2.40E-10	7.51E-10	down	--
novel.1519	84	66	56	2	4	2	28.69583	54.0489	3.34277264	-4.001639484	2.50E-10	7.80E-10	down	--

Solyc07g040960.1	1567	2490	4010	253	277	420	1232.625	2065.58	399.668887	-2.369579565	2.52E-10	7.85E-10	down	Predicted transposase
Solyc08g080040.4	80	89	150	9	6	16	47.45733	81.7802	13.1344786	-2.643640611	2.69E-10	8.37E-10	down	Iron/ascorbate family oxidoreductases
Solyc11g006950.3	7	13	5	50	39	40	31.2589	6.67954	55.8382667	3.078208681	2.69E-10	8.37E-10	up	--
Solyc12g015770.3	24	13	17	54	70	46	43.16143	14.0435	72.2794024	2.361842624	2.87E-10	8.91E-10	up	--
Solyc03g026210.4	110	67	96	8	5	12	40.70354	70.741	10.6660964	-2.740032278	2.94E-10	9.13E-10	down	--
Solyc12g036340.1	87	67	81	5	3	10	34.33925	61.0719	7.60655171	-3.006256283	3.18E-10	9.86E-10	down	--
Solyc03g115640.3	64	68	81	4	4	7	30.78219	55.2511	6.31326884	-3.126752094	3.28E-10	1.01E-09	down	--
Solyc09g008030.1	83	47	80	1	4	1	28.31712	54.1833	2.45090123	-4.42790116	3.31E-10	1.02E-09	down	--
novel.1123	0	0	0	30	22	33	18.25878	0	36.5175597	7.984842189	3.44E-10	1.06E-09	up	--
Solyc11g040173.1	15	35	36	59	96	76	59.54378	22.3255	96.7620648	2.117921409	3.70E-10	1.14E-09	up	--
Solyc07g054100.3	102	97	121	12	21	13	51.1173	82.9517	19.282948	-2.09948086	4.33E-10	1.33E-09	down	FOG: PPR repeat
Solyc01g090040.3	62	66	71	0	5	1	27.06345	51.7825	2.34437174	-4.369441369	4.68E-10	1.44E-09	down	--
Solyc10g007780.3	63	68	100	6	3	8	33.40896	59.5063	7.31166974	-3.045641617	4.72E-10	1.45E-09	down	--
novel.778	11	15	8	57	38	47	35.3524	9.01319	61.6916091	2.781109001	4.76E-10	1.46E-09	up	--
Solyc02g067960.3	160	129	164	7	36	19	71.26521	117.509	25.0213818	-2.201395441	4.80E-10	1.47E-09	down	Mitochondrial transcription termination factor, mTERF
Solyc08g029090.4	121	77	103	3	13	15	45.30415	78.1188	12.4894747	-2.604496972	4.80E-10	1.47E-09	down	--
Solyc11g012980.1	127	163	281	33	25	16	89.27289	146.095	32.4507571	-2.193646922	4.92E-10	1.51E-09	down	--
Solyc10g081320.1	4	9	10	41	44	31	27.85784	5.95547	49.7602113	3.055202664	4.95E-10	1.52E-09	up	Transcription factor, Myb superfamily
Solyc05g010240.4	1	2	5	19	35	39	20.25627	2.01378	38.4987569	4.229631652	5.21E-10	1.59E-09	up	Mitochondrial chaperonin, Cpn60/Hsp60p
Solyc11g007570.2	79	46	84	0	3	2	27.87751	53.7946	1.96044129	-4.68725271	5.26E-10	1.61E-09	down	--
Solyc06g051140.4	0	2	5	27	24	45	21.15189	1.74855	40.5552273	4.493868688	5.41E-10	1.65E-09	up	Ubiquitin-conjugating enzyme
Solyc01g066560.3	121	84	88	9	10	15	45.40222	76.5065	14.297924	-2.415480684	5.45E-10	1.66E-09	down	Uncharacterized membrane protein
Solyc09g082550.3	0	1	0	28	48	65	29.29844	0.27949	58.3173824	7.709108736	5.53E-10	1.68E-09	up	Sulfate/bicarbonate/oxalate exchanger SAT-1
Solyc04g071790.3	0	0	2	30	55	50	28.29016	0.47583	56.1044916	6.767001104	5.53E-10	1.68E-09	up	Cytochrome P450 CYP2 subfamily
Solyc03g019920.1	3	2	1	35	22	33	20.29579	1.59259	38.9989944	4.630521348	5.60E-10	1.70E-09	up	--
Solyc05g024380.1	67	77	73	7	0	1	30.26424	56.6589	3.8695931	-4.021529583	5.72E-10	1.74E-09	down	--
novel.1536	21	16	29	48	55	73	45.53865	16.9412	74.1360992	2.122622764	5.73E-10	1.74E-09	up	--
Solyc05g039990.4	125	113	114	10	15	26	56.47641	91.8584	21.0944273	-2.104829269	5.79E-10	1.76E-09	down	--
Solyc12g017575.1	95	74	88	8	10	11	39.51746	66.8156	12.2192992	-2.449243275	5.91E-10	1.79E-09	down	--
Solyc09g090685.1	70	73	74	6	5	7	32.13507	56.5745	7.6956002	-2.890691067	6.01E-10	1.82E-09	down	--
Solyc11g016970.2	12	8	23	44	45	63	37.59413	10.8907	64.2975323	2.543714723	6.34E-10	1.92E-09	up	Uncharacterized membrane protein
Solyc02g064960.3	4	1	7	36	27	34	22.42274	3.00582	41.8396531	3.757439196	6.37E-10	1.93E-09	up	--
Solyc02g021410.4	3	3	2	28	30	27	19.18976	2.10999	36.2695387	4.116352706	6.49E-10	1.96E-09	up	Predicted K+/H+-antiporter
Solyc10g080550.2	108	85	148	11	17	20	53.8048	87.6129	19.9967224	-2.122767852	6.58E-10	1.99E-09	down	--
Solyc06g065820.3	77	44	96	4	2	5	30.15135	55.5601	4.74258501	-3.575806164	6.73E-10	2.03E-09	down	--
Solyc12g096130.2	80	77	97	5	11	12	38.66631	65.8168	11.5157803	-2.490180569	6.84E-10	2.07E-09	down	--
Solyc07g039310.1	4	0	5	27	32	31	20.1928	2.2505	38.1351045	4.041349428	7.11E-10	2.14E-09	up	Flavin-containing amine oxidase
Solyc09g090780.1	107	108	121	16	11	22	54.14148	87.3522	20.9307813	-2.071094431	7.24E-10	2.18E-09	down	FOG: PPR repeat
Solyc01g049880.3	84	57	65	2	1	1	27.72632	53.6747	1.77791581	-4.982475041	7.56E-10	2.27E-09	down	--
Solyc07g064820.1	57	124	199	6	8	16	54.77245	97.1198	12.4251327	-2.954237201	7.72E-10	2.32E-09	down	MEKK and related serine/threonine protein kinases
Solyc01g112040.2	21	19	38	57	62	75	51.02152	19.9209	82.1221529	2.032197255	7.80E-10	2.35E-09	up	--
Solyc07g055900.1	115	86	97	8	12	18	46.69162	77.6153	15.7679054	-2.283633205	7.92E-10	2.38E-09	down	DNA helicase PIF1/RRM3
novel.1334	71	62	98	0	1	2	30.32814	59.4753	1.18092638	-5.562208544	8.10E-10	2.43E-09	down	--
Solyc06g069380.4	72	62	67	5	4	5	29.19181	52.3652	6.01838687	-3.140561274	8.39E-10	2.52E-09	down	--
Solyc01g109810.2	179	125	148	5	23	31	70.66641	117.624	23.7089745	-2.278143298	8.55E-10	2.56E-09	down	--
Solyc12g005240.1	107	44	109	7	4	6	37.00825	66.61	7.40654523	-3.200513562	8.87E-10	2.66E-09	down	--
Solyc07g053270.3	2	6	5	24	35	34	21.19962	3.39697	39.0022694	3.529798052	9.02E-10	2.70E-09	up	Predicted haloacid-halidohydrolase
Solyc06g007970.3	11	2	7	20	69	48	30.47449	5.14193	55.8070576	3.439790061	9.05E-10	2.71E-09	up	--
Solyc08g074720.2	42	26	13	124	68	93	73.16588	21.4993	124.832443	2.540291099	9.26E-10	2.77E-09	up	--
Solyc05g025907.1	62	77	73	4	7	6	31.20984	55.3327	7.08695675	-2.950780704	9.46E-10	2.83E-09	down	--
novel.1088	101	102	100	1	0	0	39.79197	79.0877	0.49628695	-7.436328414	9.65E-10	2.88E-09	down	--
novel.1462	1	0	1	45	51	31	27.4884	0.50315	54.4736612	6.710852185	9.87E-10	2.94E-09	up	--
Solyc02g089050.4	88	55	70	3	7	5	30.78067	55.3663	6.19508534	-3.137017577	1.01E-09	3.02E-09	down	--
Solyc01g108420.2	120	174	188	20	12	36	77.01528	125.187	28.843869	-2.120680313	1.01E-09	3.02E-09	down	--

Solyc02g088710.3	5	6	2	34	27	34	22.163	3.47892	40.8470792	3.575735222	1.02E-09	3.04E-09	up	Acyl-CoA synthetase
Solyc01g099170.4	4	4	5	31	24	37	21.37208	3.36845	39.3756994	3.540136733	1.05E-09	3.13E-09	up	--
Solyc03g070430.1	77	48	89	1	7	0	29.11863	55.0127	3.22458914	-4.042829475	1.07E-09	3.18E-09	down	--
Solyc01g100750.2	2	0	6	27	26	38	20.2618	1.95795	38.565651	4.236150661	1.14E-09	3.37E-09	up	--
Solyc01g107440.2	127	74	107	11	16	12	48.13285	79.8234	16.4422893	-2.279107416	1.14E-09	3.39E-09	down	--
Solyc02g064980.1	50	68	89	1	3	0	27.55337	53.4412	1.66555932	-4.987757255	1.15E-09	3.40E-09	down	MEKK and related serine/threonine protein kinases
Solyc02g084590.4	103	90	94	15	12	8	45.06142	74.8368	15.2860694	-2.321122122	1.16E-09	3.45E-09	down	GATA-4/5/6 transcription factors
Solyc01g095690.2	73	46	70	1	4	2	25.85944	48.8724	2.8464857	-4.058183714	1.16E-09	3.45E-09	down	FOG: PPR repeat
Solyc08g080650.3	399	176	727	2772	2045	2300	1705.293	327.981	3082.60568	3.231871533	1.25E-09	3.69E-09	up	--
Solyc10g009420.3	109	87	152	10	11	24	54.06649	89.3887	18.7442286	-2.245062157	1.27E-09	3.76E-09	down	Zn finger protein
novel.737	119	90	91	12	17	9	47.25415	78.3667	16.1415803	-2.284045715	1.32E-09	3.89E-09	down	--
Solyc09g005260.4	10	13	11	29	44	54	30.90297	8.90272	52.9032104	2.582360143	1.37E-09	4.04E-09	up	Ca2+/H+ antiporter VCX1 and related proteins
Solyc07g064200.4	115	105	154	10	11	29	58.60445	96.4868	20.7221509	-2.206372778	1.39E-09	4.11E-09	down	--
novel.2035	112	78	89	10	15	7	43.12937	72.6804	13.5783226	-2.424681182	1.44E-09	4.23E-09	down	--
Solyc12g010270.1	95	78	65	4	11	6	35.55376	62.4615	8.64598658	-2.827198334	1.54E-09	4.52E-09	down	--
Solyc07g055215.1	79	73	80	9	5	9	35.18237	60.3891	9.97562996	-2.624101969	1.61E-09	4.73E-09	down	Protein involved in mRNA turnover and stability
Solyc05g052660.1	56	52	89	2	6	1	27.14373	50.5608	3.7267031	-3.742968365	1.62E-09	4.75E-09	down	--
Solyc12g008530.2	26	24	26	40	68	82	49.29121	19.7895	78.7929107	2.001393056	1.66E-09	4.86E-09	up	--
Solyc08g007225.1	97	110	103	4	18	17	48.35105	80.9764	15.7257178	-2.325020079	1.68E-09	4.92E-09	down	Predicted lipase
Solyc06g065490.3	157	64	159	15	14	14	57.89806	97.357	18.4390911	-2.413789947	1.71E-09	5.00E-09	down	--
Solyc08g079590.3	14	14	12	42	36	59	34.34793	10.4811	58.2148034	2.477618268	1.72E-09	5.02E-09	up	FOG: PPR repeat
Solyc02g065116.1	85	70	65	6	8	1	32.03234	57.5733	6.49136581	-3.172935721	1.75E-09	5.11E-09	down	--
novel.1484	74	60	81	3	0	0	28.57819	55.6675	1.48886085	-5.43996984	1.78E-09	5.19E-09	down	--
Solyc08g044280.1	120	111	105	19	13	19	54.92221	87.832	22.0124037	-2.013449456	1.90E-09	5.55E-09	down	--
Solyc03g113130.3	84	47	65	1	0	4	26.47924	50.8798	2.07862479	-4.594757636	1.90E-09	5.56E-09	down	Uncharacterized conserved protein
Solyc10g083330.1	74	86	90	8	10	12	38.84516	65.0754	12.6148837	-2.362849936	1.96E-09	5.71E-09	down	--
Solyc12g044920.3	19	6	27	52	54	66	43.05121	13.14	72.9623984	2.455331927	1.97E-09	5.74E-09	up	P-type ATPase
Solyc07g055220.3	58	67	67	2	7	3	27.47853	50.0494	4.90762947	-3.31487084	1.98E-09	5.76E-09	down	FOG: PPR repeat
Solyc11g006310.2	13	16	10	32	43	72	35.7109	10.299	61.1228341	2.584983714	2.10E-09	6.12E-09	up	--
Solyc12g100260.1	3	1	9	82	23	30	32.37195	3.21642	61.5274851	4.212423227	2.14E-09	6.21E-09	up	--
Solyc01g108750.2	58	52	70	4	3	2	25.25822	46.5708	3.94558909	-3.606069727	2.18E-09	6.34E-09	down	--
Solyc06g062550.4	1	1	4	20	36	27	18.06708	1.49638	34.6377878	4.490515913	2.19E-09	6.34E-09	up	Predicted haloacid dehalogenase-like hydrolase
Solyc04g055000.3	16	11	8	30	52	49	31.88051	9.22139	54.5396347	2.579328521	2.20E-09	6.37E-09	up	FOG: TPR repeat
Solyc11g073150.1	13	25	26	37	67	95	49.33894	16.621	82.0568904	2.309997558	2.25E-09	6.53E-09	up	--
novel.65	0	0	0	16	23	32	14.78186	0	29.5637154	7.689406567	2.40E-09	6.94E-09	up	--
Solyc08g061847.1	106	93	112	10	23	9	49.12047	80.7534	17.4875511	-2.196107532	2.43E-09	7.03E-09	down	FOG: Transposon-encoded proteins with TYA
Solyc11g068700.2	39	73	95	1	2	1	27.50998	53.3486	1.67138632	-4.984942224	2.45E-09	7.09E-09	down	--
Solyc02g075620.4	0	2	4	30	23	30	18.6156	1.51064	35.7205638	4.523290323	2.47E-09	7.14E-09	up	--
Solyc08g067410.2	108	72	112	4	13	17	44.59577	75.4146	13.7769306	-2.418734113	2.52E-09	7.26E-09	down	--
Solyc06g007740.1	100	86	92	8	17	13	44.09303	72.4473	15.7387703	-2.184811572	2.59E-09	7.46E-09	down	FOG: PPR repeat
Solyc08g078280.1	63	49	67	4	2	3	25.1481	46.3448	3.95141609	-3.598192899	2.70E-09	7.77E-09	down	FOG: PPR repeat
Solyc05g052650.2	64	57	88	0	2	1	27.50862	53.8421	1.17509937	-5.419660109	2.71E-09	7.81E-09	down	--
Solyc01g088670.3	101	70	85	10	6	12	39.31187	66.5753	12.0484277	-2.483564318	2.77E-09	7.98E-09	down	FOG: PPR repeat
Solyc02g072100.3	50	60	70	2	1	5	25.02258	46.6849	3.36025365	-3.790815564	2.88E-09	8.26E-09	down	Uncharacterized conserved protein
Solyc07g043110.2	0	0	0	25	18	25	14.65621	0	29.3124194	7.666652784	2.93E-09	8.40E-09	up	UDP-glucuronosyl and UDP-glucosyl transferase
Solyc02g077370.1	22	46	28	106	64	93	69.84671	25.3532	114.340249	2.174329705	3.10E-09	8.88E-09	up	--
Solyc06g005850.3	9	2	10	36	34	36	25.34217	5.32521	45.3591242	3.066068344	3.18E-09	9.11E-09	up	Protein involved in mRNA turnover and stability
Solyc01g021730.2	39	56	116	3	1	3	28.32942	53.5935	3.06537168	-4.171680664	3.26E-09	9.31E-09	down	--
Solyc04g011990.3	61	70	71	0	2	1	26.90517	52.6352	1.17509937	-5.384509293	3.30E-09	9.43E-09	down	Apoptotic ATPase
Solyc06g075580.4	75	81	90	4	6	15	37.10035	63.9432	10.2574594	-2.613466305	3.37E-09	9.61E-09	down	Kinesin-like protein
novel.1814	0	2	2	17	31	38	18.29319	1.03481	35.5515688	5.104575394	3.37E-09	9.62E-09	up	--
Solyc04g017740.1	0	0	0	23	16	29	14.56133	0	29.1226685	7.659391812	3.48E-09	9.92E-09	up	--
Solyc01g079150.4	8	11	10	28	38	47	27.43733	7.57537	47.2992875	2.650432323	3.50E-09	9.98E-09	up	Na+-independent Cl/HCO3 exchanger AE1
Solyc02g070040.1	17	19	32	52	67	52	44.96177	17.4325	72.4910629	2.04618797	3.51E-09	1.00E-08	up	--
Solyc07g061800.4	796	512	696	89	40	73	304.2242	519.811	88.6375023	-2.558055537	3.53E-09	1.00E-08	down	--

Solyc12g005940.2	11	5	12	20	82	46	33.62635	7.16996	60.0827356	3.066024378	3.76E-09	1.07E-08	up	Iron/ascorbate family oxidoreductases
Solyc04g071070.2	2110	902	1785	5381	4331	4281	3644.236	1236.41	6052.05669	2.291120057	3.80E-09	1.08E-08	up	--
Solyc02g076770.1	0	0	2	41	28	43	24.37347	0.47583	48.2711055	6.541598365	3.82E-09	1.09E-08	up	--
Solyc02g005602.1	130	108	93	18	13	17	53.75792	86.7909	20.7249478	-2.082391023	4.00E-09	1.13E-08	down	K+-channel ERG and related proteins
Solyc07g055160.4	135	89	128	21	19	12	56.85411	91.1338	22.5744311	-2.035835615	4.11E-09	1.17E-08	down	Predicted lipase
Solyc05g055970.4	11	11	16	37	45	40	30.76182	9.79855	51.7250811	2.392842892	4.18E-09	1.18E-08	up	FOG: Ankyrin repeat
Solyc08g077340.2	89	65	91	10	9	10	37.92455	63.4226	12.4265312	-2.366759729	4.19E-09	1.19E-08	down	--
Solyc04g014510.3	1	1	2	19	21	50	19.20707	1.02055	37.3935816	5.178739229	4.29E-09	1.21E-08	up	Glutamine synthetase
Solyc07g009530.1	222	90	415	1455	1450	1198	971.963	182.77	1761.15601	3.267448009	4.58E-09	1.29E-08	up	Predicted chitinase
Solyc11g065910.1	0	1	0	32	36	36	22.21649	0.27949	44.1534914	7.300178887	4.75E-09	1.34E-08	up	--
Solyc03g114750.3	203	334	384	530	1575	1142	783.6039	238.55	1328.65753	2.477893937	4.78E-09	1.35E-08	up	--
Solyc03g034330.1	57	71	71	5	5	7	29.52656	51.8538	7.19931325	-2.851212819	4.78E-09	1.35E-08	down	--
Solyc05g012180.3	8	19	19	46	53	45	36.62008	11.9525	61.2876455	2.356059527	5.01E-09	1.41E-08	up	--
Solyc02g014840.3	83	67	106	7	13	12	39.62338	65.9589	13.2878691	-2.298403904	5.07E-09	1.43E-08	down	--
Solyc07g055690.1	80	76	51	7	2	4	30.21457	54.5933	5.8358614	-3.284710233	5.21E-09	1.47E-08	down	--
Solyc09g074620.3	71	62	74	6	4	9	30.9312	53.7654	8.09701166	-2.74094744	5.25E-09	1.48E-08	down	--
Solyc01g096560.3	6	7	11	50	23	45	28.87247	6.16487	51.5800696	3.047718435	5.27E-09	1.48E-08	up	--
Solyc09g065780.3	60	57	94	5	7	7	31.09376	54.2087	7.97882816	-2.761717508	5.82E-09	1.63E-08	down	--
Solyc09g025310.4	0	1	2	16	30	48	19.68834	0.75532	38.621369	5.645061939	5.89E-09	1.65E-08	up	--
Solyc10g080850.1	0	0	0	13	26	26	13.43531	0	26.8706202	7.553805479	5.96E-09	1.66E-08	up	--
novel.290	39	60	90	0	4	3	25.63571	48.5256	2.74578321	-4.061077839	5.96E-09	1.66E-08	down	--
Solyc09g010600.1	62	42	68	2	4	2	23.85192	44.3611	3.34277264	-3.720217566	6.34E-09	1.77E-08	down	FOG: Predicted E3 ubiquitin ligase
Solyc10g086440.2	14	14	16	28	54	54	33.8686	11.4327	56.3044981	2.30777894	6.62E-09	1.84E-08	up	--
Solyc01g087860.2	81	87	98	9	11	17	42.29686	69.1149	15.4788504	-2.150507438	6.73E-09	1.87E-08	down	--
Solyc05g014280.4	784	403	566	1759	1168	1828	1253.284	455.235	2051.33385	2.171651133	6.73E-09	1.87E-08	up	Molecular chaperone
Solyc06g062540.3	8	1	19	114	126	118	79.64342	6.92172	152.365118	4.443201407	6.93E-09	1.92E-08	up	Predicted haloacid dehalogenase-like hydrolase
Solyc08g075920.4	27	18	23	63	55	51	45.27081	17.6641	72.8775454	2.037018727	7.01E-09	1.94E-08	up	--
Solyc04g055090.1	47	56	64	2	4	1	23.14548	43.3438	2.94718818	-3.875838976	7.44E-09	2.06E-08	down	--
Solyc04g071890.3	257	99	321	1134	786	942	706.9919	172.204	1241.77932	2.849183022	7.47E-09	2.07E-08	up	--
Solyc06g066065.1	2	2	8	39	20	35	21.99428	2.99276	40.9957962	3.731957684	7.87E-09	2.18E-08	up	--
novel.208	75	60	87	10	5	7	33.52049	57.3602	9.68074799	-2.607966649	8.06E-09	2.22E-08	down	--
novel.722	45	63	59	1	2	2	22.82356	43.5801	2.06697078	-4.371223365	8.09E-09	2.23E-08	down	--
Solyc06g073480.3	12	8	7	30	37	39	25.91076	7.08409	44.7374283	2.666862713	8.10E-09	2.23E-08	up	--
novel.320	70	85	97	10	14	6	39.09671	65.4004	12.7929806	-2.363563941	8.13E-09	2.24E-08	down	--
Solyc12g036800.1	201	87	98	7	7	23	58.1217	100.943	15.3007534	-2.708081017	8.21E-09	2.26E-08	down	--
Solyc03g097510.1	4	0	0	30	20	34	18.59728	1.06093	36.1336292	5.10869973	8.29E-09	2.28E-08	up	--
novel.47	0	0	0	21	16	24	13.07609	0	26.1521723	7.503613619	8.31E-09	2.29E-08	up	--
Solyc07g052885.1	60	28	59	0	0	0	18.88826	37.7765	0	-7.334968329	8.33E-09	2.29E-08	down	--
Solyc02g092210.1	59	49	90	0	3	0	25.96259	50.7559	1.16927237	-5.337523188	8.61E-09	2.37E-08	down	Subunit of Golgi mannosyltransferase complex
Solyc01g150170.1	108	80	80	8	16	11	42.29754	70.0372	14.557844	-2.25240249	8.66E-09	2.38E-08	down	Vacuolar sorting protein VPS1
Solyc03g025670.3	3	1	10	38	18	60	26.53197	3.45433	49.6096059	3.802444499	9.01E-09	2.47E-08	up	--
Solyc07g055870.4	96	64	84	9	11	10	38.02203	63.3343	12.7097592	-2.320840122	9.15E-09	2.51E-08	down	Tyrosine kinase specific for activated (GTP-bound) p21cdc42Hs
novel.167	87	75	72	7	13	7	36.23828	61.1666	11.3099468	-2.426855523	9.30E-09	2.55E-08	down	--
Solyc02g069250.4	1	1	0	24	40	36	21.14347	0.54472	41.7422256	6.327004143	9.40E-09	2.58E-08	up	Alcohol dehydrogenase, class V
Solyc10g080360.1	70	50	117	7	9	4	34.4704	60.3766	8.56416359	-2.834268332	9.93E-09	2.72E-08	down	--
Solyc05g010735.1	1	2	1	32	25	20	17.29947	1.06212	33.536808	4.997678049	1.02E-08	2.79E-08	up	--
Solyc02g084850.3	5	0	8	54	58	14	29.08654	3.22948	54.9436102	4.051250938	1.02E-08	2.80E-08	up	--
Solyc04g015280.3	59	55	53	2	4	4	23.88197	43.63	4.13394156	-3.37496716	1.09E-08	2.98E-08	down	--
novel.1458	0	0	0	21	14	25	12.88412	0	25.7682418	7.481998008	1.12E-08	3.07E-08	up	--
novel.1420	78	49	98	5	9	8	33.42627	57.6986	9.15392753	-2.645349326	1.13E-08	3.08E-08	down	--
Solyc06g062830.4	61	60	99	8	7	7	32.98483	56.502	9.46768901	-2.598359781	1.14E-08	3.10E-08	down	--
Solyc05g056550.3	8	7	9	25	36	37	23.64728	6.2195	41.0750672	2.72434088	1.14E-08	3.10E-08	up	Serine/threonine protein kinase
Solyc07g063320.3	95	46	55	1	1	1	26.21019	51.1388	1.28162887	-5.333581524	1.20E-08	3.25E-08	down	Lanthionine synthetase C-like protein 1
Solyc11g010160.1	6	0	5	25	26	34	19.38585	2.78096	35.9907392	3.6670725	1.24E-08	3.38E-08	up	Apoptotic ATPase

Solyc01g090890.3	1	0	8	56	143	125	67.572	2.16855	132.975443	5.898874159	1.35E-08	3.67E-08	up	Protein involved in vacuolar polyphosphate accumulation
Solyc09g072820.4	0	1	2	26	30	23	17.22497	0.75532	33.6946269	5.436755981	1.36E-08	3.69E-08	up	--
novel.1803	0	0	0	30	10	23	13.94231	0	27.8846256	7.590267284	1.38E-08	3.73E-08	up	--
Solyc03g118900.3	2	0	2	25	20	30	16.53807	1.00629	32.0698567	4.951504203	1.38E-08	3.74E-08	up	--
novel.1619	91	84	71	12	9	9	38.76427	64.505	13.0235206	-2.331388919	1.43E-08	3.87E-08	down	--
Solyc02g093270.4	77	33	143	812	561	387	419.1989	63.6677	774.730122	3.602810458	1.45E-08	3.93E-08	up	O-methyltransferase
Solyc03g026000.4	55	59	49	2	4	3	23.23686	42.7354	3.7383571	-3.494777749	1.51E-08	4.08E-08	down	--
Solyc09g009570.3	12	7	3	42	33	33	26.30664	5.85294	46.7603351	3.008453697	1.52E-08	4.10E-08	up	Acyl-CoA reductase
Solyc12g088750.2	97	98	95	18	13	13	47.4309	75.7192	19.14261	-2.008209939	1.54E-08	4.15E-08	down	Serine/threonine protein kinase
Solyc03g124110.2	58	81	133	4	3	15	39.37641	69.6646	9.08818707	-2.922294269	1.57E-08	4.22E-08	down	--
Solyc01g107220.2	1	2	0	26	21	32	17.28564	0.82421	33.74707	5.422093699	1.60E-08	4.31E-08	up	--
Solyc10g019170.2	85	82	81	11	12	12	39.80853	64.7338	14.8832594	-2.126474372	1.63E-08	4.39E-08	down	--
Solyc10g086480.3	74	69	99	6	11	14	37.63431	62.4654	12.8032362	-2.268053633	1.64E-08	4.42E-08	down	Pectin acetylesterase and similar proteins
Solyc03g115330.2	80	70	65	8	8	8	33.25009	56.2472	10.2530309	-2.465738691	1.64E-08	4.42E-08	down	--
novel.410	1	0	0	33	26	32	19.71755	0.26523	39.1698659	7.123359361	1.64E-08	4.42E-08	up	--
Solyc12g056890.1	55	54	66	1	7	0	24.30353	45.3825	3.22458914	-3.76409385	1.66E-08	4.47E-08	down	Molecular chaperone (DnaJ superfamily)
Solyc04g078960.1	17	18	23	32	58	61	38.81476	15.0118	62.6177669	2.065104689	1.67E-08	4.50E-08	up	--
Solyc03g025710.3	2	3	4	20	24	30	16.73402	2.32059	31.1474517	3.740261468	1.68E-08	4.51E-08	up	--
Solyc11g069590.2	19	16	16	36	41	72	37.82316	13.3178	62.328467	2.232972946	1.71E-08	4.59E-08	up	Serine/threonine protein kinase
Solyc12g042730.1	4	6	10	50	19	42	26.97564	5.117	48.8342864	3.234511091	1.79E-08	4.79E-08	up	--
novel.811	0	1	0	30	30	29	19.16638	0.27949	38.0532815	7.08342296	1.84E-08	4.95E-08	up	--
Solyc10g050980.1	86	45	47	1	3	0	24.11721	46.5689	1.66555932	-4.78787828	1.87E-08	5.02E-08	down	--
Solyc12g010800.2	6	10	25	34	48	70	36.80358	10.3341	63.2730264	2.600220703	1.92E-08	5.13E-08	up	--
Solyc02g083760.4	63	95	80	4	11	12	36.65682	62.2941	11.0194933	-2.467461964	2.07E-08	5.54E-08	down	--
Solyc01g102740.3	1	4	0	17	25	35	16.70473	1.38318	32.0262706	4.607573422	2.09E-08	5.60E-08	up	--
Solyc12g008650.2	75	43	59	5	4	2	25.38948	45.9473	4.83163349	-3.29196365	2.15E-08	5.74E-08	down	Aldehyde reductase
novel.1397	107	84	158	19	22	11	55.90143	89.4473	22.3555451	-2.016422767	2.19E-08	5.84E-08	down	--
Solyc06g008950.2	2	7	11	33	36	29	23.49231	5.10394	41.8806871	3.018994886	2.24E-08	5.96E-08	up	Copper chaperone
novel.1815	6	5	4	20	27	38	19.71094	3.94049	35.4813998	3.184861211	2.30E-08	6.12E-08	up	FOG: Reverse transcriptase
Solyc01g111800.4	55	40	63	3	3	1	21.9048	40.7559	3.05371768	-3.77873926	2.34E-08	6.23E-08	down	Amino acid transporters
Solyc03g031955.1	55	68	87	0	0	1	27.34355	54.2915	0.39558446	-6.895403922	2.37E-08	6.32E-08	down	--
novel.685	59	58	60	3	5	7	26.1703	46.1339	6.20673935	-2.872174363	2.43E-08	6.46E-08	down	FOG: Transposon-encoded proteins with TYA
Solyc01g090050.4	67	77	78	12	5	5	33.8653	57.8485	9.88215297	-2.60518154	2.50E-08	6.63E-08	down	--
Solyc09g010080.3	111	45	98	2	3	13	36.31888	65.3333	7.30444425	-3.128966281	2.57E-08	6.82E-08	down	Beta-fructofuranosidase (invertase)
Solyc02g082520.3	10	17	12	43	32	53	32.51857	10.2586	54.7785538	2.420987231	2.66E-08	7.05E-08	up	Amino acid transporters
Solyc02g068630.1	93	76	75	6	16	9	38.26267	63.7512	12.7741011	-2.296927025	2.68E-08	7.09E-08	down	FOG: PPR repeat
Solyc05g015865.1	77	56	36	3	1	1	23.45665	44.6391	2.27420276	-4.380187499	2.84E-08	7.51E-08	down	--
Solyc01g087580.3	37	54	125	3	3	6	29.83845	54.6453	5.03163998	-3.438020272	2.88E-08	7.59E-08	down	--
Solyc05g010720.4	78	84	113	10	20	9	43.68385	71.0494	16.3182788	-2.116368846	2.95E-08	7.78E-08	down	--
Solyc11g013150.1	113	96	104	4	20	21	49.81636	81.5452	18.0875706	-2.134779909	2.97E-08	7.83E-08	down	--
Solyc02g063320.4	6	15	18	44	43	40	32.24288	10.0662	54.4195748	2.425872988	2.98E-08	7.85E-08	up	FOG: Armadillo/beta-catenin-like repeats
novel.1603	3	5	6	23	26	30	18.51823	3.62062	33.4158275	3.201638182	3.10E-08	8.16E-08	up	--
Solyc11g069230.3	80	60	72	4	11	9	32.47521	55.1177	9.83273996	-2.458414513	3.16E-08	8.31E-08	down	--
Solyc01g104930.3	55	64	50	0	2	1	22.77291	44.3707	1.17509937	-5.138495227	3.19E-08	8.38E-08	down	AAA+-type ATPase
Solyc10g005340.3	50	66	66	6	5	5	27.1573	47.4102	6.90443128	-2.801548829	3.23E-08	8.50E-08	down	--
Solyc01g097570.2	4	9	8	30	29	33	22.36275	5.47964	39.2458619	2.840652406	3.26E-08	8.57E-08	up	--
Solyc09g011910.3	64	69	105	5	11	13	36.57596	61.2405	11.9113647	-2.33974113	3.32E-08	8.72E-08	down	--
Solyc06g083550.1	76	72	58	3	8	10	31.32128	54.0798	8.5627651	-2.623976107	3.52E-08	9.21E-08	down	--
Solyc04g076260.2	83	61	84	9	11	10	35.8788	59.0478	12.7097592	-2.220319421	3.53E-08	9.25E-08	down	--
Solyc11g012570.3	65	66	71	3	5	12	30.38144	52.5782	8.18466165	-2.653143804	3.67E-08	9.61E-08	down	FOG: PPR repeat
Solyc09g011520.3	3	0	1	30	17	26	16.41665	1.03361	31.7996812	4.928126874	3.68E-08	9.62E-08	up	Glutathione S-transferase
Solyc12g019070.1	0	0	1	23	30	29	17.40859	0.23791	34.5792729	6.949783949	3.80E-08	9.94E-08	up	Inositol polyphosphate 5-phosphatase
Solyc08g059730.1	84	75	99	10	18	11	41.56228	66.7946	16.3299328	-2.026146048	3.86E-08	1.01E-07	down	--
Solyc03g121420.4	7	4	1	45	42	14	23.7267	3.21249	44.2409083	3.79954026	3.91E-08	1.02E-07	up	Predicted haloacid dehalogenase-like hydrolase

novel.1946	62	64	68	6	1	9	28.71877	50.5098	6.92773929	-2.890161405	3.93E-08	1.02E-07	down	--
Solyc02g072020.3	87	63	89	6	8	16	37.14122	61.8573	12.4251327	-2.299654803	4.12E-08	1.07E-07	down	FOG: PPR repeat
Solyc05g009253.1	49	52	51	3	3	3	21.75413	39.6634	3.8448866	-3.379528787	4.18E-08	1.09E-07	down	FOG: PPR repeat
Solyc02g089690.3	111	75	111	19	8	13	47.25048	76.8108	17.6901097	-2.154875073	4.33E-08	1.13E-07	down	--
Solyc08g005100.3	53	47	52	1	5	1	21.20272	39.5648	2.84065869	-3.753840276	4.34E-08	1.13E-07	down	--
Solyc12g006620.2	52	68	99	3	8	11	32.65457	56.3508	8.95834956	-2.622046292	4.36E-08	1.13E-07	down	Uncharacterized conserved protein
Solyc07g064650.3	2	3	7	28	18	36	19.09352	3.03433	35.1527093	3.505796581	4.43E-08	1.15E-07	up	--
Solyc02g067400.3	1	0	2	18	38	20	16.19835	0.74106	31.6556376	5.358502971	4.48E-08	1.16E-07	up	Glutaredoxin-related protein
novel.1680	16	10	13	28	43	47	29.68978	10.1315	49.2480748	2.285681724	4.55E-08	1.18E-07	up	--
Solyc04g078970.3	21	12	15	30	47	61	34.91515	12.4924	57.337861	2.205119102	4.76E-08	1.23E-07	up	--
Solyc03g117880.1	49	34	32	0	0	0	15.05611	30.1122	0	-7.004589132	4.78E-08	1.24E-07	down	--
Solyc02g085240.4	65	73	72	3	10	11	32.25521	54.7725	9.73786447	-2.453649727	4.82E-08	1.25E-07	down	Clathrin adaptor complex, small subunit
Solyc06g083160.1	0	1	1	24	26	34	18.00593	0.5174	35.4944523	6.094829759	4.83E-08	1.25E-07	up	--
Solyc10g050880.2	2	4	5	54	16	23	22.48602	2.83799	42.1340571	3.873570548	5.06E-08	1.31E-07	up	Arylacetamide deacetylase
Solyc10g049580.2	1	5	5	28	19	31	18.2084	2.85225	33.5645445	3.549815646	5.37E-08	1.39E-07	up	--
Solyc04g079910.4	1	0	0	30	25	24	17.1959	0.26523	34.1265719	6.923366089	5.40E-08	1.40E-07	up	Uncharacterized conserved protein
Solyc09g018805.1	8	5	6	28	31	26	20.60525	4.94678	36.2637117	2.87172203	5.49E-08	1.42E-07	up	--
Solyc06g024310.1	35	79	105	7	4	0	30.68838	56.3437	5.03303847	-3.558338878	5.51E-08	1.42E-07	down	--
Solyc04g079340.3	79	45	68	0	4	9	27.41387	49.7085	5.11928997	-3.211906713	5.62E-08	1.45E-07	down	--
Solyc01g093990.3	114	69	104	6	21	13	45.28472	74.2642	16.3052263	-2.160888624	5.76E-08	1.48E-07	down	Glyoxylate/hydroxypyruvate reductase
Solyc06g073830.1	47	49	17	88	76	132	77.85866	30.2054	125.511967	2.063523436	5.77E-08	1.49E-07	up	Calmodulin and related proteins
Solyc04g079760.4	3	6	7	34	19	34	20.93352	4.13803	37.7290196	3.177267966	5.95E-08	1.53E-07	up	--
Solyc11g027860.2	0	0	1	18	38	25	16.93574	0.23791	33.6335599	6.914422354	5.96E-08	1.53E-07	up	--
Solyc09g008080.3	65	56	60	4	4	9	27.13535	47.1663	7.10443776	-2.720188563	5.97E-08	1.54E-07	down	--
Solyc08g061240.3	1	7	4	25	21	36	19.00321	3.17331	34.8331209	3.470986201	6.03E-08	1.55E-07	up	Predicted hydrolase/acyltransferase (alpha/beta hydrolase superfamily)
novel.436	6	12	4	19	43	45	24.94362	5.89691	43.9903234	2.929630536	6.04E-08	1.56E-07	up	--
Solyc12g005320.3	26	30	37	63	46	152	66.70363	24.0835	109.323759	2.182896002	6.05E-08	1.56E-07	up	Pectin acetylesterase and similar proteins
Solyc06g060410.3	7	4	10	38	22	35	23.31637	5.35372	41.2790242	2.923474846	6.43E-08	1.65E-07	up	Junctional membrane complex protein Junctophilin
Solyc01g104750.4	4	2	7	27	28	22	18.15056	3.28531	33.0158145	3.298436635	6.45E-08	1.66E-07	up	--
Solyc04g081630.2	3	2	6	22	25	25	16.66701	2.78216	30.5518608	3.4292351	6.51E-08	1.67E-07	up	Endoplasmic reticulum glucose-regulated protein
Solyc11g020210.2	85	57	57	6	9	5	30.25005	52.0366	8.4634611	-2.621548126	6.64E-08	1.70E-07	down	--
Solyc01g065570.2	0	2	1	22	26	21	15.07809	0.79689	29.3592804	5.228868236	6.77E-08	1.74E-07	up	Molecular chaperone (DnaJ superfamily)
Solyc02g062590.3	1	0	2	20	20	30	15.16474	0.74106	29.5884219	5.256347312	6.93E-08	1.78E-07	up	Glycosyl transferase, family 8 - glycogenin
Solyc01g005980.4	75	75	84	9	12	14	37.76034	60.8388	14.6818545	-2.045068949	6.95E-08	1.78E-07	down	--
novel.1495	7	9	5	30	47	21	23.53804	5.56159	41.5144826	2.913652373	6.98E-08	1.79E-07	up	--
Solyc07g048060.3	2	1	1	18	29	19	14.40005	1.04787	27.7522361	4.738318278	7.17E-08	1.84E-07	up	Predicted membrane protein
Solyc08g075930.4	66	76	70	11	5	8	32.98652	55.4004	10.5726194	-2.427083955	7.21E-08	1.85E-07	down	--
Solyc07g039347.1	43	52	64	2	3	6	22.85012	41.1649	4.53535303	-3.157438452	7.62E-08	1.94E-07	down	--
Solyc02g065610.3	12	17	24	35	49	51	35.1435	13.644	56.642966	2.05025078	7.66E-08	1.95E-07	up	--
Solyc01g008520.3	86	50	64	5	7	9	30.39043	52.0109	8.76999708	-2.558082915	8.43E-08	2.14E-07	down	DNA repair protein
novel.664	2	1	4	17	23	26	14.72405	1.76161	27.6864956	3.9427881	8.44E-08	2.14E-07	up	--
Solyc03g118050.3	57	33	27	142	54	124	85.66856	30.765	140.572122	2.190290846	8.48E-08	2.15E-07	up	--
Solyc01g079300.4	0	0	0	4	26	30	11.99319	0	23.9863755	7.394848614	8.94E-08	2.27E-07	up	--
Solyc09g073030.4	37	43	95	3	4	3	24.33405	44.4335	4.23464405	-3.398588256	8.95E-08	2.27E-07	down	--
Solyc03g114040.3	103	58	75	9	6	13	36.66024	61.3728	11.9477253	-2.36986726	9.63E-08	2.43E-07	down	--
Solyc03g071850.1	4	1	1	22	18	26	14.89874	1.57833	28.2191431	4.169225018	9.75E-08	2.46E-07	up	UDP-glucuronosyl and UDP-glucosyl transferase
Solyc07g044800.2	5	3	6	17	37	25	18.16981	3.59211	32.7475155	3.183859925	9.75E-08	2.46E-07	up	Apoptotic ATPase
Solyc03g007850.1	55	45	43	1	4	2	20.12076	37.395	2.8464857	-3.671130125	1.04E-07	2.61E-07	down	Cell cycle control protein (crooked neck)
Solyc11g042950.1	179	66	92	14	11	16	52.68776	87.8108	17.5647007	-2.331057845	1.05E-07	2.65E-07	down	--
Solyc02g069910.2	56	72	77	9	9	7	32.01952	53.2955	10.7434909	-2.32715483	1.06E-07	2.66E-07	down	--
Solyc03g116250.2	86	56	49	4	3	9	28.41688	50.1191	6.71468031	-2.892814666	1.06E-07	2.67E-07	down	Alpha-1,4-N-acetylglucosaminyltransferase
Solyc03g114890.4	19	9	23	43	54	39	35.42093	13.0268	57.8150354	2.136808596	1.10E-07	2.77E-07	up	--
Solyc10g008910.1	124	47	55	6	6	7	33.59766	59.11	8.08535765	-2.87609174	1.13E-07	2.83E-07	down	Histones H3 and H4
Solyc05g007600.1	67	62	52	2	6	9	27.18086	47.4703	6.89137878	-2.74277167	1.19E-07	2.99E-07	down	Cysteine desulfurase NFS1

Solyc09g007520.3	30	4	63	400	409	573	304.3295	24.0635	584.595475	4.598652452	1.22E-07	3.04E-07	up	--
Solyc02g080170.1	97	59	98	12	8	15	40.27009	65.5329	15.0072699	-2.140962936	1.22E-07	3.05E-07	down	FOG: PPR repeat
Solyc04g051690.4	9	10	12	59	23	38	30.65725	8.03694	53.277561	2.715785174	1.23E-07	3.08E-07	up	--
Solyc03g007180.1	72	63	73	7	9	12	32.90052	54.0722	11.7288393	-2.197518919	1.23E-07	3.09E-07	down	--
Solyc02g071830.3	60	52	75	1	9	8	27.72983	48.2909	7.16877974	-2.697248115	1.30E-07	3.25E-07	down	--
Solyc03g025860.3	4	9	5	14	42	40	21.95355	4.76589	39.1412089	3.062715974	1.32E-07	3.28E-07	up	Synaptobrevin/VAMP-like protein SEC22
Solyc12g006220.3	79	43	63	7	6	4	27.6774	47.9599	7.39489122	-2.726512499	1.42E-07	3.52E-07	down	--
Solyc06g069400.4	1	0	1	17	26	34	16.26179	0.50315	32.0204436	5.954809979	1.43E-07	3.55E-07	up	Asparaginase
Solyc04g071000.1	39	26	35	0	0	0	12.96887	25.9377	0	-6.791030825	1.43E-07	3.57E-07	down	Serine/threonine protein kinase
Solyc08g006920.3	2	4	2	21	28	17	15.09221	2.12425	28.0601705	3.743347007	1.54E-07	3.81E-07	up	Leucine rich repeat proteins
Solyc07g064660.1	4	2	1	22	28	16	15.00935	1.85782	28.160873	3.940179931	1.54E-07	3.83E-07	up	Geranylgeranyl pyrophosphate synthase
Solyc07g055880.1	72	56	97	9	12	10	35.46263	57.8257	13.0995166	-2.1459127	1.63E-07	4.04E-07	down	--
novel.1806	35	59	57	4	0	0	20.6596	39.334	1.9851478	-4.500483173	1.64E-07	4.06E-07	down	--
novel.1461	12	0	7	256	123	173	124.137	4.84818	243.425738	5.644621367	1.70E-07	4.21E-07	up	FOG: Transposon-encoded proteins with TYA
novel.1375	44	56	72	1	2	9	24.64372	44.4514	4.836062	-3.154811505	1.76E-07	4.34E-07	down	--
Solyc01g099470.3	78	49	61	8	7	4	28.58836	48.8958	8.28093563	-2.591837173	1.77E-07	4.37E-07	down	ATP-dependent DNA helicase
Solyc12g150125.1	5	1	13	83	37	16	33.32037	4.69854	61.942194	3.685652125	1.87E-07	4.59E-07	up	Arylacetamide deacetylase
Solyc05g025890.3	46	37	52	2	1	1	18.3456	34.9133	1.77791581	-4.361398754	1.87E-07	4.61E-07	down	--
Solyc09g018030.4	66	51	55	5	4	8	26.02481	44.8445	7.20514025	-2.641077617	1.88E-07	4.62E-07	down	--
Solyc03g059360.1	83	66	66	4	15	7	33.3817	56.1628	10.6006009	-2.374297883	1.92E-07	4.71E-07	down	Calcium transporting ATPase
Solyc09g015145.1	8	7	3	24	31	27	19.73308	4.79201	34.6741483	2.874750412	1.92E-07	4.72E-07	up	--
Solyc05g012820.2	33	50	65	1	5	1	20.51608	38.1915	2.84065869	-3.706350432	1.93E-07	4.76E-07	down	--
novel.709	92	61	103	12	17	10	41.24623	65.9553	16.5371647	-2.001651735	2.05E-07	5.03E-07	down	--
Solyc06g069650.4	3	0	4	18	25	21	14.36586	1.74735	26.9843752	3.907715896	2.07E-07	5.07E-07	up	--
novel.694	79	60	74	3	13	11	33.1177	55.3283	10.9071368	-2.30380704	2.09E-07	5.12E-07	down	--
Solyc03g118510.3	49	36	54	4	0	1	19.14302	35.9053	2.38073226	-4.057168619	2.35E-07	5.73E-07	down	--
Solyc01g081185.1	67	65	43	7	5	3	26.38856	46.1676	6.60954931	-2.842276415	2.37E-07	5.77E-07	down	--
novel.1072	61	29	50	1	1	3	19.12641	36.18	2.07279779	-4.105236866	2.46E-07	5.98E-07	down	--
Solyc03g150118.1	11	11	17	18	50	66	32.28304	10.0365	54.5296123	2.446372626	2.51E-07	6.11E-07	up	--
Solyc03g006810.3	46	54	82	5	4	9	27.20137	46.802	7.60072471	-2.624769715	2.53E-07	6.16E-07	down	--
Solyc07g005100.4	87	54	247	820	606	613	491.287	96.9324	885.641591	3.189841524	2.60E-07	6.32E-07	up	Chitinase
Solyc02g088950.3	50	51	64	1	8	6	24.36493	42.742	5.98785336	-2.780313706	2.82E-07	6.84E-07	down	--
Solyc10g055540.2	72	41	77	2	8	9	28.27301	48.8751	7.67089369	-2.632647015	2.91E-07	7.05E-07	down	Ras-related small GTPase, Rho type
novel.147	62	54	61	8	7	5	27.36302	46.0495	8.67652009	-2.434258446	2.93E-07	7.07E-07	down	--
novel.139	64	48	67	6	8	7	27.5977	46.3305	8.86487257	-2.386983625	2.95E-07	7.13E-07	down	--
Solyc03g044850.1	3	2	8	20	28	24	16.79548	3.25799	30.3329748	3.187496956	3.07E-07	7.40E-07	up	--
Solyc03g095903.1	40	58	40	2	3	3	19.84238	36.3362	3.34859965	-3.427742996	3.07E-07	7.41E-07	down	Iron/ascorbate family oxidoreductases
novel.442	52	27	64	1	4	1	19.50783	36.5648	2.45090123	-3.864948314	3.22E-07	7.74E-07	down	--
Solyc02g086227.1	54	56	51	6	5	5	24.50596	42.1075	6.90443128	-2.629092821	3.27E-07	7.86E-07	down	--
Solyc07g041235.1	73	97	86	12	19	7	41.53143	66.9329	16.1299263	-2.056333293	3.30E-07	7.95E-07	down	--
Solyc05g054360.4	8	9	10	21	25	67	26.84326	7.01639	46.6701212	2.739812254	3.31E-07	7.96E-07	up	--
Solyc01g066810.4	15	17	27	51	29	98	45.26717	15.1535	75.3808777	2.309124531	3.32E-07	7.98E-07	up	--
Solyc08g015640.1	50	39	46	1	5	1	18.97318	35.1057	2.84065869	-3.582705984	3.34E-07	8.02E-07	down	--
Solyc01g057430.3	97	68	80	10	6	18	39.09388	63.7658	14.4219345	-2.148010627	3.35E-07	8.05E-07	down	--
Solyc05g015477.1	53	24	79	1	0	2	20.42385	39.5602	1.28745587	-4.965572406	3.38E-07	8.12E-07	down	--
Solyc01g073840.1	29	27	33	0	0	0	11.54454	23.0891	0	-6.623096095	3.39E-07	8.14E-07	down	--
Solyc01g011050.3	27	17	33	56	35	150	60.26749	19.7637	100.771249	2.347543368	3.69E-07	8.85E-07	up	FOG: Leucine rich repeat
Solyc09g010605.1	46	41	42	0	3	1	17.60847	33.6521	1.56485683	-4.330986978	3.70E-07	8.86E-07	down	--
Solyc03g025125.1	73	41	55	0	7	6	24.50402	43.9062	5.10180896	-3.034740039	3.75E-07	8.98E-07	down	--
novel.15	1	1	2	18	23	16	12.62374	1.02055	24.2269379	4.546014648	4.02E-07	9.60E-07	up	--
Solyc06g069850.3	52	52	57	6	7	3	24.38967	41.8866	6.89277727	-2.623517698	4.08E-07	9.74E-07	down	Transcription factor, Myb superfamily
Solyc04g007630.2	53	44	58	0	0	2	20.47249	40.1538	0.79116892	-5.578268359	4.24E-07	1.01E-06	down	Copper chaperone
novel.89	67	78	83	14	10	3	35.67493	59.3175	12.0323452	-2.344672829	4.29E-07	1.02E-06	down	FOG: Reverse transcriptase
Solyc02g084770.3	85	50	65	10	8	3	30.62561	51.9835	9.26768252	-2.526237604	4.33E-07	1.03E-06	down	Mitochondrial FAD carrier protein
Solyc05g053330.3	6	6	13	24	25	45	22.90867	6.36121	39.4561239	2.619955889	4.41E-07	1.05E-06	up	Transcription factor, Myb superfamily

Solyc12g077520.1	6	7	3	16	30	32	18.27678	4.26155	32.2920176	2.949333485	4.52E-07	1.08E-06	up	--
Solyc06g068290.1	51	46	54	1	7	5	22.21659	39.2307	5.20251144	-2.859838438	4.63E-07	1.10E-06	down	--
Solyc04g014990.3	42	45	68	1	1	0	20.39047	39.8949	0.88604441	-5.559693472	4.66E-07	1.11E-06	down	UDP-glucuronosyl and UDP-glucosyl transferase
Solyc02g067450.4	44	43	58	85	55	219	93.87064	37.4872	150.254048	2.002940111	4.70E-07	1.12E-06	up	--
Solyc09g098350.4	57	47	31	2	3	2	19.29126	35.6295	2.95301519	-3.589439831	4.74E-07	1.13E-06	down	--
Solyc09g082560.4	0	0	2	18	31	19	14.50379	0.47583	28.531751	5.794359264	4.86E-07	1.15E-06	up	--
Solyc02g084300.1	93	66	91	4	19	14	39.84586	64.763	14.9287219	-2.082041469	4.86E-07	1.15E-06	down	CTP synthase (UTP-ammonia lyase)
Solyc01g073860.3	43	62	70	4	6	9	26.6356	45.3873	7.88395268	-2.509370367	4.99E-07	1.18E-06	down	--
Solyc06g066820.4	61	34	48	4	3	3	20.7214	37.1016	4.34117355	-3.125387343	5.01E-07	1.19E-06	down	Iron/ascorbate family oxidoreductases
Solyc01g008680.4	59	70	77	11	6	11	32.8407	53.5323	12.1491302	-2.163680059	5.03E-07	1.19E-06	down	--
Solyc11g066250.2	9	0	4	32	29	21	19.41508	3.33874	35.4914223	3.394544072	5.04E-07	1.19E-06	up	Serine carboxypeptidases (lysosomal cathepsin A)
novel.217	78	63	28	2	0	0	22.975	44.9574	0.9925739	-5.706425821	5.12E-07	1.21E-06	down	--
Solyc09g059470.4	42	40	40	1	2	2	16.95141	31.8358	2.06697078	-3.918885171	5.19E-07	1.23E-06	down	--
Solyc02g032950.3	11	10	16	26	33	46	26.7407	9.51906	43.9623419	2.203956957	5.27E-07	1.25E-06	up	--
Solyc04g018140.1	46	43	38	0	3	3	17.80771	33.2594	2.35602575	-3.733208552	5.32E-07	1.26E-06	down	--
Solyc03g098090.2	3	5	7	20	19	36	17.71535	3.85854	31.5721712	3.025675685	5.32E-07	1.26E-06	up	--
Solyc01g068140.4	58	46	72	5	10	5	26.86334	45.3697	8.35693161	-2.433139387	5.33E-07	1.26E-06	down	--
Solyc07g007920.3	44	57	57	1	5	8	23.38595	41.1622	5.60974991	-2.822847944	5.37E-07	1.27E-06	down	--
Solyc08g080660.1	9	1	22	39	30	87	36.68223	7.9007	65.4637627	3.027929088	5.51E-07	1.30E-06	up	--
Solyc07g055470.3	61	31	63	3	3	6	22.43176	39.8319	5.03163998	-2.980374403	5.58E-07	1.32E-06	down	Cytochrome P450 CYP4/CYP19/CYP26
Solyc04g072070.3	3	0	3	14	23	22	13.06237	1.50944	24.6152969	3.997349992	5.67E-07	1.34E-06	up	--
Solyc01g103130.2	11	14	7	38	38	27	26.42313	8.49578	44.3504678	2.39217334	5.71E-07	1.34E-06	up	Vacuolar sorting protein VPS1
novel.834	77	86	63	9	10	15	36.87268	59.4474	14.297924	-2.049899071	5.71E-07	1.34E-06	down	--
Solyc06g060510.2	44	39	40	1	3	2	17.27177	32.0868	2.45672824	-3.670562681	5.81E-07	1.37E-06	down	--
Solyc06g084620.1	35	29	38	67	39	206	78.18559	26.429	129.942165	2.29856204	5.89E-07	1.38E-06	up	--
Solyc08g074250.3	5	4	5	33	24	16	17.84734	3.63368	32.0609996	3.128363714	6.21E-07	1.46E-06	up	Apoptotic ATPase
Solyc08g007210.4	256	313	518	39	12	36	158.4461	278.619	38.273321	-2.872328884	6.44E-07	1.51E-06	down	--
Solyc01g081250.3	15	15	25	442	68	143	158.2748	14.1187	302.430916	4.418332079	6.48E-07	1.52E-06	up	Glutathione S-transferase
Solyc02g072170.3	39	50	64	6	0	4	22.05252	39.545	4.56005953	-3.193710951	6.55E-07	1.53E-06	down	--
Solyc05g009280.4	2	1	7	23	17	27	15.59831	2.47535	28.721257	3.488253617	6.81E-07	1.59E-06	up	--
novel.621	53	43	40	3	1	5	19.7242	35.5919	3.8565406	-3.22069715	6.83E-07	1.60E-06	down	--
Solyc07g041230.3	5	13	8	16	42	46	24.68506	6.86282	42.5072895	2.650925668	7.06E-07	1.65E-06	up	--
Solyc02g069500.1	64	38	40	5	2	2	20.58204	37.112	4.05211858	-3.260938268	7.16E-07	1.67E-06	down	FOG: PPR repeat
Solyc04g050720.4	104	76	56	8	15	10	37.96045	62.1484	13.772502	-2.162878533	7.25E-07	1.69E-06	down	--
novel.218	68	52	46	8	1	4	24.7278	43.5132	5.94239089	-2.944246225	7.48E-07	1.74E-06	down	--
Solyc03g116540.1	66	54	56	1	6	11	26.55356	45.9209	7.18626075	-2.623820841	7.64E-07	1.78E-06	down	FOG: PPR repeat
Solyc12g096480.1	70	57	75	1	13	10	30.92981	52.3406	9.51897848	-2.406410224	7.77E-07	1.81E-06	down	--
novel.268	44	35	198	929	529	745	515.251	68.5594	961.942693	3.808560927	8.16E-07	1.90E-06	up	Predicted chitinase
Solyc03g062900.3	77	45	99	10	5	12	34.10601	56.5534	11.6586703	-2.299155611	8.33E-07	1.93E-06	down	--
Solyc01g068120.4	13	13	26	52	24	87	41.42204	13.2671	69.5769483	2.380892647	8.39E-07	1.95E-06	up	--
Solyc04g009860.4	20	5	24	50	35	58	36.90589	12.412	61.3997571	2.289609321	8.49E-07	1.97E-06	up	Iron/ascorbate family oxidoreductases
Solyc01g099500.1	54	49	65	2	4	11	25.19246	43.4819	6.90303278	-2.619236123	8.88E-07	2.06E-06	down	ATP-dependent DNA helicase
Solyc03g043920.4	41	33	49	1	1	4	17.1119	31.7554	2.46838225	-3.656847497	8.89E-07	2.06E-06	down	Subunit of Golgi mannosyltransferase complex
Solyc03g118450.1	26	48	63	2	0	4	18.9375	35.3001	2.57491174	-3.796337075	8.99E-07	2.08E-06	down	--
Solyc10g054400.1	63	57	72	8	12	8	30.79117	49.7703	11.8120607	-2.07524996	9.10E-07	2.11E-06	down	Transcription initiation factor IIF, small subunit (RAP30)
Solyc08g082485.1	17	41	26	0	0	0	11.07687	22.1537	0	-6.560605813	9.36E-07	2.16E-06	down	--
Solyc08g014430.3	1	0	1	2	47	47	19.2034	0.50315	37.903644	6.206918956	9.41E-07	2.17E-06	up	Rho GTPase effector BNI1 and related formins
Solyc01g109580.4	58	64	59	9	3	9	28.25188	47.3076	9.19611505	-2.394454752	9.48E-07	2.19E-06	down	Adenylate cyclase-associated protein (CAP/Srv2p)
novel.784	37	50	94	6	7	4	26.72016	46.152	7.28836173	-2.679967332	9.62E-07	2.22E-06	down	--
Solyc11g005980.3	9	9	8	20	17	112	33.83144	6.8058	60.8570753	3.168597972	9.97E-07	2.30E-06	up	1,3-beta-glucan synthase/callose synthase catalytic subunit
Solyc02g086620.3	37	42	53	4	1	4	19.0594	34.1616	3.95724309	-3.154258714	1.01E-06	2.33E-06	down	--
Solyc07g008100.4	75	54	64	8	10	10	31.01749	50.2113	11.8237148	-2.086482752	1.05E-06	2.41E-06	down	--
Solyc06g073980.4	31	44	47	1	1	1	16.49164	31.7017	1.28162887	-4.643911981	1.05E-06	2.42E-06	down	--

Solyc07g005380.4	2	0	1	101	26	22	34.86496	0.76838	68.9615338	6.472410146	1.07E-06	2.46E-06	up	--
novel.1701	2	4	12	25	21	38	20.06384	4.50339	35.6242898	2.955303292	1.10E-06	2.53E-06	up	--
Solyc03g059350.3	59	59	43	5	1	8	24.20233	42.3688	6.03586788	-2.82859893	1.11E-06	2.54E-06	down	Calcium transporting ATPase
Solyc11g066950.2	4	0	2	18	29	13	13.45774	1.53676	25.3787293	4.030347524	1.11E-06	2.55E-06	up	--
Solyc02g050330.1	68	67	64	7	9	14	32.254	51.988	12.5200082	-2.042428308	1.14E-06	2.61E-06	down	--
Solyc05g012890.1	22	8	17	38	40	41	31.39186	12.1155	50.6681652	2.056170785	1.15E-06	2.64E-06	up	Apoptotic ATPase
Solyc08g008670.3	205	205	207	2	5	22	86.28005	160.916	11.6442193	-3.770895764	1.17E-06	2.68E-06	down	Phosphomannomutase
Solyc05g024230.3	211	193	219	16	4	2	86.14959	162.008	10.2907899	-4.011601157	1.19E-06	2.72E-06	down	--
Solyc05g012800.1	66	42	54	6	5	7	24.89339	42.0912	7.6956002	-2.463678289	1.20E-06	2.75E-06	down	--
Solyc12g070123.1	54	74	95	2	16	10	34.39553	57.6065	11.1845378	-2.322402891	1.21E-06	2.77E-06	down	Iron/ascorbate family oxidoreductases
Solyc10g006160.1	17	37	26	0	0	0	10.51789	21.0358	0	-6.486273246	1.22E-06	2.78E-06	down	--
Solyc01g106620.2	149	152	436	892	824	642	601.7729	185.733	1017.81333	2.452974651	1.24E-06	2.83E-06	up	Defense-related protein containing SCP domain
Solyc08g075190.1	36	40	63	1	0	1	18.30418	35.7165	0.89187141	-5.398827028	1.25E-06	2.85E-06	down	--
Solyc09g090670.3	31	40	49	3	0	2	16.66978	31.0595	2.28002977	-3.858387632	1.32E-06	3.02E-06	down	Oxidation resistance protein
Solyc10g055630.2	984	499	552	1642	1380	3000	1535.652	531.781	2539.52184	2.255725654	1.36E-06	3.10E-06	up	Aquaporin (major intrinsic protein family)
Solyc08g006765.1	37	41	43	3	3	2	17.47611	31.5029	3.44930214	-3.214354185	1.38E-06	3.14E-06	down	Diamine acetyltransferase
Solyc12g009220.2	9	7	7	25	22	33	20.02251	6.0089	34.0361249	2.503291776	1.39E-06	3.16E-06	up	--
Solyc04g045300.1	45	37	36	3	0	2	16.56072	30.8414	2.28002977	-3.846469211	1.39E-06	3.16E-06	down	--
Solyc05g012780.4	15	10	9	21	46	35	25.55546	8.91459	42.196325	2.256215334	1.42E-06	3.22E-06	up	--
Solyc09g010050.1	40	44	48	1	0	1	17.60926	34.3266	0.89187141	-5.339584452	1.46E-06	3.30E-06	down	Cullins
Solyc02g063350.1	35	65	69	8	4	4	25.48881	43.866	7.11166326	-2.671117348	1.48E-06	3.36E-06	down	Calmodulin and related proteins (EF-Hand superfamily)
Solyc10g080760.2	56	41	40	5	2	4	20.33593	35.8286	4.8432875	-2.928610374	1.50E-06	3.40E-06	down	Diadenosine and diphosphoinositol polyphosphate phosphohydrolase
Solyc09g082260.1	4	6	10	24	17	44	20.52974	5.117	35.9424798	2.801352657	1.50E-06	3.40E-06	up	--
Solyc03g122360.3	2	8	2	14	27	31	16.48839	3.2422	29.7345869	3.236117833	1.55E-06	3.50E-06	up	Cytochrome P450 CYP2 subfamily
Solyc01g028800.3	5	5	9	17	30	27	17.83761	4.86483	30.8103822	2.656754216	1.58E-06	3.57E-06	up	Translational repressor MPT5/PUF4
Solyc01g090440.2	6	6	15	29	45	21	23.53786	6.83704	40.2386807	2.53919632	1.60E-06	3.62E-06	up	--
Solyc11g066160.1	51	51	51	7	6	4	23.65462	39.9144	7.39489122	-2.460576546	1.61E-06	3.63E-06	down	Histone H4
novel.353	48	58	37	4	2	6	21.44122	37.7443	5.13816947	-2.887768258	1.62E-06	3.66E-06	down	--
Solyc08g007060.4	30	45	47	2	1	4	17.34029	31.7159	2.96466919	-3.422704446	1.63E-06	3.68E-06	down	H+/oligopeptide symporter
Solyc08g080580.4	61	50	55	6	3	10	25.67084	43.2388	8.10283866	-2.424658823	1.64E-06	3.69E-06	down	Transcription factor, Myb superfamily
novel.566	49	36	49	3	4	5	19.87078	34.7157	5.02581297	-2.780822078	1.65E-06	3.71E-06	down	--
novel.2019	9	10	21	34	22	88	35.21901	10.1782	60.2598528	2.55797169	1.66E-06	3.73E-06	up	--
novel.445	2	3	2	7	30	26	13.64834	1.84476	25.4519283	3.812739001	1.77E-06	3.98E-06	up	--
Solyc07g007705.1	65	58	66	6	14	8	30.37587	49.1527	11.5990018	-2.066417795	1.81E-06	4.07E-06	down	--
Solyc12g008580.2	53	38	33	2	2	4	17.94172	32.529	3.35442665	-3.268454298	1.83E-06	4.10E-06	down	--
novel.270	48	8	33	0	0	0	11.4091	22.8182	0	-6.607555378	1.86E-06	4.18E-06	down	--
Solyc02g085530.3	6	22	5	41	39	40	30.15069	8.9297	51.3716841	2.54307578	1.92E-06	4.31E-06	up	FOG: PPR repeat
novel.1215	31	43	58	0	1	1	17.41228	34.0392	0.78534192	-5.343083044	1.99E-06	4.45E-06	down	--
novel.1361	42	29	46	0	1	0	15.28936	30.189	0.38975746	-6.049253472	2.00E-06	4.48E-06	down	--
novel.2027	46	39	50	3	6	4	20.20308	34.9964	5.40974343	-2.679649786	2.05E-06	4.59E-06	down	--
Solyc03g111070.3	64	43	41	4	6	5	22.52446	38.7473	6.30161484	-2.614248245	2.08E-06	4.64E-06	down	Mitochondrial/plastidial beta-ketoacyl-ACP reductase
Solyc10g084880.3	376	710	1298	82	96	124	367.0709	606.977	127.164719	-2.254938995	2.13E-06	4.74E-06	down	--
Solyc03g026140.4	65	54	46	6	5	9	25.88163	43.2765	8.48676912	-2.353001116	2.21E-06	4.92E-06	down	Cytochrome P450 CYP2 subfamily
Solyc04g051390.2	40	36	50	1	1	0	16.72631	32.5666	0.88604441	-5.265627393	2.25E-06	5.01E-06	down	--
Solyc10g083550.1	27	18	26	0	0	0	9.18891	18.3778	0	-6.294353515	2.32E-06	5.16E-06	down	Glutamine synthetase
Solyc11g007550.1	59	49	56	8	8	5	25.86654	42.6668	9.06627754	-2.255384	2.32E-06	5.16E-06	down	Predicted spermine/spermidine synthase
Solyc06g064530.1	28	39	43	2	1	1	15.16739	28.5569	1.77791581	-4.068388625	2.46E-06	5.46E-06	down	--
Solyc02g084130.4	30	36	61	3	1	4	17.99614	32.5313	3.46095614	-3.261293574	2.48E-06	5.50E-06	down	--
Solyc03g063370.3	18	19	8	44	39	36	31.63298	11.9878	51.2782071	2.10463207	2.49E-06	5.53E-06	up	FOG: PPR repeat
Solyc12g006210.1	65	40	54	6	7	6	24.67325	41.267	8.07953065	-2.360025089	2.53E-06	5.60E-06	down	F0F1-type ATP synthase, beta subunit
novel.1324	47	35	43	2	0	0	16.73544	32.4783	0.9925739	-5.24533993	2.55E-06	5.65E-06	down	--
Solyc04g076720.3	41	32	42	3	1	3	16.43795	29.8105	3.06537168	-3.323245563	2.58E-06	5.71E-06	down	Uncharacterized conserved protein

novel.446	1	6	4	13	27	24	14.68151	2.89382	26.4692087	3.211579228	2.60E-06	5.75E-06	up	--
Solyc11g008860.3	25	19	43	62	32	156	63.5623	22.1714	104.953205	2.238330071	2.65E-06	5.86E-06	up	Multicopper oxidases
Solyc01g057260.4	10	6	7	18	34	27	19.43017	5.99465	32.865699	2.462909413	2.66E-06	5.87E-06	up	--
Solyc01g010020.3	34	41	66	2	7	3	20.54344	36.1793	4.90762947	-2.853546545	2.66E-06	5.87E-06	down	Calmodulin and related proteins (EF-Hand superfamily)
Solyc03g078770.3	8	4	6	16	24	32	17.31038	4.66729	29.9534729	2.685994664	2.69E-06	5.94E-06	up	UDP-glucuronosyl and UDP-glucosyl transferase
Solyc05g006720.1	43	74	52	9	2	5	25.84133	44.4586	7.22401975	-2.676723673	2.73E-06	6.03E-06	down	--
Solyc12g007040.3	43	43	50	5	6	2	20.46492	35.3187	5.6111484	-2.676772754	2.76E-06	6.08E-06	down	--
Solyc08g068680.4	56	108	103	19	9	6	42.42684	69.5429	15.3107759	-2.224328401	2.76E-06	6.08E-06	down	Glutamate decarboxylase
Solyc07g054800.1	48	27	38	1	0	0	14.90717	29.3181	0.49628695	-6.005944281	2.76E-06	6.08E-06	down	--
Solyc07g040890.1	6	1	4	29	11	28	16.28928	2.82254	29.7560184	3.378934487	2.90E-06	6.38E-06	up	Arylacetamide deacetylase
Solyc07g008545.1	8	9	14	16	33	53	24.86831	7.96805	41.7685636	2.393007754	2.97E-06	6.54E-06	up	Purple acid phosphatase
Solyc09g074180.3	43	31	36	0	2	1	14.90456	28.634	1.17509937	-4.511722434	3.04E-06	6.67E-06	down	--
Solyc02g089270.1	79	46	61	2	14	4	28.17703	48.3225	8.03151613	-2.548464493	3.08E-06	6.75E-06	down	--
Solyc02g094230.1	50	44	31	0	2	0	16.85696	32.9344	0.77951491	-5.293662404	3.16E-06	6.93E-06	down	--
Solyc03g026015.1	39	58	53	6	5	6	23.23192	39.1638	7.30001574	-2.438618837	3.17E-06	6.95E-06	down	--
Solyc12g096870.1	2	7	5	14	27	26	15.71656	3.67645	27.7566646	2.932497235	3.17E-06	6.95E-06	up	UDP-glucuronosyl and UDP-glucosyl transferase
novel.1640	24	16	17	82	43	30	42.10228	14.8819	69.3226342	2.210594938	3.18E-06	6.98E-06	up	--
Solyc08g075950.3	38	24	54	1	2	3	16.04823	29.6339	2.46255524	-3.5609332	3.22E-06	7.06E-06	down	--
Solyc06g060540.3	44	34	50	5	1	4	18.76103	33.0685	4.45353004	-2.948451964	3.27E-06	7.15E-06	down	--
Solyc08g007780.3	9	1	0	28	27	19	17.30108	2.66657	31.9355906	3.596315239	3.27E-06	7.15E-06	up	--
Solyc03g121190.4	42	40	53	6	0	4	19.7444	34.9287	4.56005953	-3.013723297	3.37E-06	7.37E-06	down	--
Solyc08g079310.4	64	45	83	11	7	9	30.52321	49.2987	11.7477188	-2.097466532	3.39E-06	7.42E-06	down	Cytochrome P450 CYP2 subfamily
Solyc11g020930.1	43	42	36	0	2	0	16.24395	31.7084	0.77951491	-5.23893152	3.40E-06	7.43E-06	down	--
Solyc12g010710.1	2	17	24	50	39	51	35.59071	10.9917	60.1896957	2.44299426	3.49E-06	7.62E-06	up	--
Solyc11g069070.3	9	2	1	19	20	31	16.33585	3.18397	29.4877194	3.227656887	3.58E-06	7.82E-06	up	Copper chaperone
Solyc03g117345.1	39	34	43	1	2	5	16.66534	30.077	3.25372416	-3.168269439	3.60E-06	7.85E-06	down	--
Solyc02g083370.1	50	47	50	8	5	2	22.50175	38.2933	6.71025179	-2.566607805	3.68E-06	8.02E-06	down	--
Solyc06g060450.3	38	68	83	10	4	8	29.25875	48.8309	9.686575	-2.368466477	3.83E-06	8.34E-06	down	emp24/gp25L/p24 family
novel.1917	47	34	56	5	4	5	20.65505	35.2917	6.01838687	-2.572127966	3.90E-06	8.48E-06	down	FOG: Reverse transcriptase
Solyc01g058350.1	47	23	42	1	0	2	15.08699	28.8865	1.28745587	-4.509787221	3.96E-06	8.60E-06	down	--
Solyc12g099410.2	8	5	7	30	9	61	23.85589	5.1847	42.5270776	3.033572852	4.11E-06	8.92E-06	up	--
Solyc08g081690.3	12	9	9	20	31	35	21.84654	7.8394	35.8536762	2.20264998	4.19E-06	9.08E-06	up	Ferric reductase, NADH/NADPH oxidase
novel.1016	54	27	46	4	3	3	18.57697	32.8128	4.34117355	-2.948004614	4.23E-06	9.16E-06	down	--
Solyc02g090400.4	3	5	11	21	18	39	18.83783	4.8102	32.8654541	2.754949603	4.42E-06	9.56E-06	up	--
Solyc05g015290.2	43	49	37	1	7	3	19.15703	33.9027	4.41134252	-2.88872305	4.72E-06	1.02E-05	down	--
Solyc11g017310.3	42	27	38	3	0	1	14.80555	27.7267	1.88444531	-4.009059374	4.78E-06	1.03E-05	down	FOG: PPR repeat
Solyc05g053320.1	38	71	77	11	1	3	27.63878	48.2419	7.03566727	-2.857720018	4.81E-06	1.04E-05	down	FOG: Transposon-encoded proteins with TYA
Solyc02g032520.1	12	27	29	0	0	0	8.814244	17.6285	0	-6.233749125	4.82E-06	1.04E-05	down	Nucleoside phosphatase
Solyc02g069300.1	8	8	7	22	19	37	19.49175	6.02316	32.9603296	2.45781721	4.84E-06	1.04E-05	up	--
Solyc11g067180.2	5	11	8	20	28	30	19.50516	6.30385	32.7064816	2.386441638	4.89E-06	1.05E-05	up	Acyl-CoA reductase
Solyc05g005910.4	34	19	10	299	79	128	123.2614	16.7073	229.815448	3.783160551	4.91E-06	1.06E-05	up	H+/oligopeptide symporter
Solyc03g006030.4	106	38	65	0	11	9	31.02357	54.1995	7.84759216	-2.735344697	5.10E-06	1.10E-05	down	--
Solyc11g006040.2	22	10	14	20	41	75	33.76771	11.9608	55.5746292	2.224162569	5.17E-06	1.11E-05	up	--
Solyc08g066610.3	10	9	9	32	23	27	21.41766	7.30894	35.5263843	2.274738556	5.18E-06	1.11E-05	up	--
Solyc10g077090.2	62	56	52	7	7	11	27.5105	44.4673	10.5537399	-2.073376077	5.25E-06	1.13E-05	down	--
Solyc06g082910.3	52	59	41	7	6	0	22.92445	40.0363	5.81255338	-2.832600195	5.33E-06	1.14E-05	down	Rho GTPase effector BNI1 and related formins
Solyc04g025550.1	38	31	34	1	2	0	14.05392	26.832	1.27580186	-4.403661451	5.40E-06	1.16E-05	down	--
Solyc03g031630.3	47	38	22	1	2	1	14.99597	28.3206	1.67138632	-4.067779361	5.54E-06	1.19E-05	down	--
Solyc01g106605.1	11	2	28	56	38	44	35.07335	10.1381	60.0085687	2.539122113	5.56E-06	1.19E-05	up	Defense-related protein containing SCP domain
Solyc10g009380.3	41	27	38	2	1	3	15.01526	27.4614	2.56908473	-3.434931891	5.77E-06	1.23E-05	down	--
Solyc03g043860.2	28	28	49	1	2	2	14.48847	26.91	2.06697078	-3.681338731	5.78E-06	1.24E-05	down	--
Solyc11g011330.2	1	0	1	19	8	26	11.66793	0.50315	22.8327076	5.459461291	6.11E-06	1.30E-05	up	Alcohol dehydrogenase, class V
Solyc01g058320.4	52	54	54	10	6	2	24.91219	41.7318	8.09258315	-2.420395533	6.11E-06	1.30E-05	down	--
Solyc05g053610.2	18	9	34	88	37	42	45.04374	15.3787	74.7088247	2.260196384	6.28E-06	1.34E-05	up	Pleiotropic drug resistance proteins (PDR1-15)

Solyc06g074770.4	37	35	38	0	1	1	14.71088	28.6364	0.78534192	-5.092109784	6.31E-06	1.34E-05	down	Ubiquitin fusion degradation protein-2
Solyc10g008610.1	52	27	37	0	0	2	15.46612	30.1411	0.79116892	-5.166781842	6.37E-06	1.36E-05	down	--
Solyc09g011750.3	1	2	10	21	31	17	16.2164	3.20336	29.2294429	3.148856371	6.39E-06	1.36E-05	up	--
novel.976	27	29	46	2	1	1	13.9942	26.2105	1.77791581	-3.946059285	6.51E-06	1.38E-05	down	--
Solyc11g005070.2	51	42	73	8	7	8	26.24818	42.6331	9.86327347	-2.128881103	6.54E-06	1.39E-05	down	Amino acid transporters
Solyc09g061830.1	60	62	49	8	3	11	27.19549	44.9	9.49099702	-2.258155437	6.73E-06	1.43E-05	down	--
Solyc02g081570.4	0	13	19	148	287	273	150.7296	8.15373	293.305416	5.166539024	6.77E-06	1.44E-05	up	--
Solyc10g074470.3	49	22	39	2	3	1	15.49059	28.4238	2.55743073	-3.486510194	7.04E-06	1.49E-05	down	--
Solyc03g097410.1	4	4	1	26	9	25	14.35884	2.41679	26.3008893	3.461537316	7.35E-06	1.55E-05	up	--
Solyc01g097280.2	10	3	21	69	21	37	32.77616	8.48699	57.0653311	2.722814781	7.44E-06	1.57E-05	up	Predicted chitinase
Solyc03g112440.1	85	29	21	158	186	247	142.1318	35.646	248.617586	2.803799619	7.60E-06	1.60E-05	up	--
Solyc03g005570.3	8	2	6	32	29	12	18.01974	4.10832	31.9311621	2.940672508	7.74E-06	1.63E-05	up	Transcription factor, Myb superfamily
Solyc09g018550.1	38	30	60	0	4	6	18.33543	32.7383	3.93253659	-2.990028663	7.93E-06	1.67E-05	down	--
Solyc07g021020.4	16	13	16	31	19	103	37.60959	11.6837	63.5354865	2.445657615	8.12E-06	1.71E-05	up	--
Solyc11g012090.2	35	46	55	2	6	7	20.66254	35.2249	6.10020986	-2.494566042	8.29E-06	1.74E-05	down	--
Solyc01g107590.3	11	5	29	34	37	54	31.93543	11.2145	52.656343	2.211367796	8.45E-06	1.78E-05	up	Alcohol dehydrogenase, class V
Solyc10g017980.1	2858	920	4426	11152	9834	8604	7419.623	2068.17	12771.0756	2.626370466	8.56E-06	1.80E-05	up	Predicted chitinase
Solyc08g013680.4	35	40	62	4	7	4	20.75457	35.2133	6.29578783	-2.480897001	8.59E-06	1.80E-05	down	Inorganic pyrophosphatase/Nucleosome remodeling factor
novel.1191	34	32	40	0	1	1	14.13171	27.4781	0.78534192	-5.033746782	8.62E-06	1.81E-05	down	--
Solyc02g085720.1	9	11	12	20	32	32	21.68656	8.31643	35.0566803	2.081029671	9.30E-06	1.95E-05	up	Geranylgeranyl pyrophosphate synthase
novel.1867	43	25	57	1	6	3	17.98744	31.9533	4.02158507	-2.945435463	9.42E-06	1.97E-05	down	--
Solyc01g073820.4	57	30	46	7	1	2	19.55093	34.4469	4.65493502	-2.978529235	9.63E-06	2.01E-05	down	--
Solyc06g074370.3	41	26	38	1	2	4	15.02004	27.1819	2.8581397	-3.213950824	1.05E-05	2.18E-05	down	Sialyltransferase
novel.1794	6	3	7	9	27	36	16.66317	4.09526	29.2310744	2.836405077	1.05E-05	2.19E-05	up	--
Solyc03g150103.1	45	51	49	4	10	5	22.85389	37.8471	7.86064466	-2.247943528	1.09E-05	2.28E-05	down	--
Solyc07g062560.3	9	15	15	34	15	89	34.03763	10.1481	57.9271351	2.514999261	1.11E-05	2.31E-05	up	--
Solyc06g071610.3	4	7	6	18	22	22	15.32776	4.44483	26.2106872	2.564920586	1.12E-05	2.32E-05	up	Dehydrogenases with different specificities
Solyc02g072350.1	27	20	59	1	1	2	14.2326	26.788	1.67721333	-3.994673688	1.13E-05	2.34E-05	down	--
Solyc01g101150.3	25	36	33	1	2	1	13.10747	24.5435	1.67138632	-3.863137072	1.22E-05	2.54E-05	down	FOG: PPR repeat
novel.580	49	47	55	6	6	10	24.24486	39.2176	9.27211103	-2.079176106	1.28E-05	2.64E-05	down	--
Solyc05g054440.4	12	23	30	37	28	132	49.12071	16.7484	81.4929746	2.283181722	1.28E-05	2.66E-05	up	--
Solyc12g014240.3	9	18	26	55	24	56	36.20317	13.6037	58.8026909	2.0999723	1.29E-05	2.66E-05	up	--
Solyc07g062310.4	50	38	57	8	0	5	21.69574	37.4433	5.94821789	-2.724475558	1.30E-05	2.68E-05	down	--
Solyc06g051240.1	35	25	47	0	4	3	15.09904	27.4523	2.74578321	-3.24525088	1.31E-05	2.71E-05	down	--
Solyc11g005860.2	6	9	8	30	18	25	18.90198	6.0101	31.7938542	2.396664116	1.34E-05	2.76E-05	up	--
Solyc07g006100.4	58	56	46	5	6	12	25.77292	41.9788	9.566993	-2.118483093	1.37E-05	2.82E-05	down	Oxysterol-binding protein
Solyc03g053040.1	42	37	40	4	6	2	18.05612	30.9974	5.11486145	-2.60874777	1.38E-05	2.84E-05	down	--
Solyc03g078310.1	49	31	22	0	0	3	14.04068	26.8946	1.18675338	-4.421834072	1.47E-05	3.02E-05	down	Long-chain acyl-CoA synthetases (AMP-forming)
Solyc09g092150.1	48	49	50	8	4	8	23.50789	38.3218	8.6940011	-2.165742916	1.48E-05	3.04E-05	down	FOG: PPR repeat
novel.1344	58	35	51	5	8	6	22.63608	37.2992	7.97300116	-2.22154002	1.50E-05	3.09E-05	down	--
Solyc10g008510.3	43	50	42	4	2	9	20.84835	35.3718	6.32492285	-2.480534779	1.54E-05	3.17E-05	down	FOG: PPR repeat
Solyc04g051360.3	27	40	28	0	1	3	13.28945	25.0024	1.57651084	-3.903785833	1.57E-05	3.22E-05	down	--
Solyc06g083080.3	9	7	8	19	26	25	17.84979	6.24682	29.4527574	2.240099825	1.58E-05	3.24E-05	up	--
Solyc08g066630.2	45	27	39	0	1	6	15.76177	28.7603	2.76326422	-3.310293172	1.60E-05	3.27E-05	down	Leucine rich repeat proteins
novel.1447	24	42	29	2	0	1	13.19587	25.0036	1.38815836	-4.279249042	1.63E-05	3.33E-05	down	--
Solyc08g008430.4	31	46	27	2	4	0	15.02697	27.5023	2.55160372	-3.435236784	1.64E-05	3.36E-05	down	Reductases with broad range of substrate
Solyc01g091420.2	5	5	8	12	24	30	15.90204	4.62692	27.1771561	2.555730471	1.65E-05	3.37E-05	up	--
Solyc08g069140.4	29	29	41	0	2	0	13.16545	25.5514	0.77951491	-4.93213908	1.73E-05	3.53E-05	down	K+-channel ERG and related proteins
Solyc01g066360.3	16	16	18	36	19	85	35.94719	12.998	58.8964009	2.181078362	1.74E-05	3.55E-05	up	--
Solyc07g056530.3	35	23	27	1	0	0	11.31566	22.135	0.49628695	-5.599729822	1.76E-05	3.60E-05	down	Glucose-6-phosphate/phosphate
Solyc06g075660.4	21	34	39	2	0	1	12.86965	24.3511	1.38815836	-4.24452975	1.80E-05	3.68E-05	down	Transcription factor, Myb superfamily
Solyc01g109370.3	58	36	40	7	5	1	20.38999	34.9616	5.81838039	-2.638827646	1.82E-05	3.70E-05	down	Lysophosphatidic acid acyltransferase LPAAT
Solyc03g033460.3	28	35	36	2	1	4	14.36908	25.7735	2.96466919	-3.122964434	1.95E-05	3.96E-05	down	Sexual differentiation process protein ISP4
Solyc03g080180.4	13	5	12	20	26	37	21.19824	7.70043	34.6960579	2.167312334	1.97E-05	4.00E-05	up	Hydroxyindole-O-methyltransferase

Solyc02g072340.1	20	39	41	2	3	1	14.2583	25.9592	2.55743073	-3.354340489	2.01E-05	4.08E-05	down	--
novel.754	38	25	36	1	2	4	14.24454	25.6309	2.8581397	-3.129476682	2.07E-05	4.20E-05	down	--
novel.793	80	26	54	5	9	3	24.25431	41.3326	7.17600523	-2.527368533	2.11E-05	4.26E-05	down	--
novel.863	26	13	8	26	42	51	30.94038	12.4327	49.4480813	2.004946468	2.16E-05	4.38E-05	up	--
Solyc04g081990.3	41	45	59	4	8	9	23.07595	37.4884	8.66346759	-2.093809955	2.22E-05	4.48E-05	down	Predicted transporter ADD1 (major facilitator superfamily)
novel.1403	37	36	41	6	3	2	17.2839	29.6296	4.93816298	-2.645451416	2.27E-05	4.57E-05	down	--
Solyc04g009720.1	52	44	63	3	9	12	25.41092	41.0781	9.74369148	-2.043465604	2.27E-05	4.58E-05	down	FOG: PPR repeat
novel.474	57	43	50	2	10	9	23.74117	39.0319	8.4504086	-2.165462632	2.29E-05	4.61E-05	down	--
Solyc03g096630.1	32	25	5	0	0	0	8.332094	16.6642	0	-6.14836858	2.29E-05	4.62E-05	down	--
novel.318	43	31	42	6	3	0	17.10425	30.0615	4.14699406	-2.942351178	2.30E-05	4.63E-05	down	--
Solyc07g055720.4	57	31	48	5	8	3	20.99424	35.2022	6.78624778	-2.380492038	2.30E-05	4.64E-05	down	Molecular chaperone (small heat-shock protein Hsp26/Hsp42)
novel.360	27	42	37	4	2	3	15.82701	27.7026	3.95141609	-2.848228572	2.37E-05	4.76E-05	down	--
Solyc03g115790.1	47	26	33	2	1	5	15.47201	27.5838	3.36025365	-3.031788208	2.45E-05	4.91E-05	down	FOG: PPR repeat
novel.98	37	28	18	0	0	1	11.15864	21.9217	0.39558446	-5.583302928	2.45E-05	4.93E-05	down	--
Solyc07g009180.1	26	27	35	2	0	1	12.07869	22.7692	1.38815836	-4.148901056	2.49E-05	5.00E-05	down	Apoptotic ATPase
Solyc03g114910.4	23	29	39	2	1	2	12.82883	23.4842	2.17350027	-3.468519663	2.52E-05	5.05E-05	down	--
Solyc01g091030.3	14	7	7	17	30	33	20.25948	7.33506	33.183889	2.189396191	2.54E-05	5.09E-05	up	--
Solyc12g021160.1	45	22	27	2	1	2	13.34068	24.5079	2.17350027	-3.527285449	2.60E-05	5.21E-05	down	--
Solyc02g087670.3	31	21	39	55	26	160	62.04655	23.3701	100.72299	2.106063783	2.61E-05	5.23E-05	up	--
Solyc02g087450.3	19	38	34	1	0	2	12.51825	23.7491	1.28745587	-4.224197896	2.61E-05	5.23E-05	down	--
Solyc05g150128.1	104	59	67	6	7	23	37.40932	60.0142	14.8044665	-2.001744476	2.91E-05	5.81E-05	down	--
novel.1340	55	39	36	6	4	6	20.48148	34.0527	6.91025828	-2.320034456	2.97E-05	5.93E-05	down	--
Solyc02g092540.2	50	27	29	2	4	3	15.72282	27.7073	3.7383571	-2.872625171	3.06E-05	6.09E-05	down	--
Solyc10g017620.3	7	7	18	25	17	59	25.23402	8.0955	42.3725336	2.376130934	3.11E-05	6.20E-05	up	Ribokinase
Solyc06g071400.3	13	17	18	43	13	95	38.23474	12.4818	63.9877094	2.358013473	3.15E-05	6.27E-05	up	Multitransmembrane protein
Solyc06g072420.3	11	12	20	30	15	98	35.26597	11.0297	59.5022474	2.429283961	3.17E-05	6.31E-05	up	Purple (tartrate-resistant) acid phosphatase
Solyc10g150144.1	14	5	8	13	30	44	21.28208	7.014	35.5501703	2.350722116	3.20E-05	6.37E-05	up	Cytochrome P450 CYP4/CYP19/CYP26
Solyc03g119510.3	28	38	36	4	3	2	15.27878	26.612	3.94558909	-2.791622295	3.22E-05	6.39E-05	down	--
Solyc07g064480.1	40	23	34	3	1	3	14.09598	25.1266	3.06537168	-3.074592478	3.34E-05	6.62E-05	down	Uncharacterized conserved protein
Solyc09g083190.3	121	34	71	7	16	9	35.87897	58.4876	13.2703881	-2.129669779	3.46E-05	6.85E-05	down	--
Solyc04g050990.3	38	34	29	1	4	4	15.05929	26.4809	3.63765462	-2.817094308	3.49E-05	6.90E-05	down	--
Solyc09g082920.1	33	30	26	0	2	0	12.05129	23.3231	0.77951491	-4.798569014	3.61E-05	7.13E-05	down	--
Solyc06g062310.3	32	31	32	1	5	0	13.60494	24.7648	2.44507423	-3.302372916	3.64E-05	7.18E-05	down	Flavin-containing monooxygenase
Solyc04g050755.1	55	33	36	7	3	3	19.1029	32.3758	5.83003439	-2.527018473	3.64E-05	7.19E-05	down	--
Solyc05g005940.3	56	28	29	5	1	3	16.81805	29.5782	4.05794558	-2.926318346	3.72E-05	7.33E-05	down	H+/oligopeptide symporter
Solyc08g067796.1	9	6	11	20	31	20	18.30049	6.68107	29.9199093	2.155873208	3.81E-05	7.50E-05	up	FOG: Leucine rich repeat
Solyc11g044937.1	57	35	43	7	7	1	20.86426	35.1306	6.5978953	-2.447935301	3.91E-05	7.68E-05	down	--
Solyc10g080960.1	21	28	39	2	1	2	12.42385	22.6742	2.17350027	-3.417793494	3.99E-05	7.85E-05	down	--
Solyc08g077760.3	47	37	29	3	6	3	17.36032	29.7065	5.01415897	-2.554676538	4.05E-05	7.95E-05	down	--
Solyc09g010710.4	33	30	29	2	1	4	13.50074	24.0368	2.96466919	-3.021503638	4.45E-05	8.70E-05	down	--
novel.134	39	24	29	2	2	3	13.45506	23.9513	2.95884219	-3.018146932	4.64E-05	9.07E-05	down	--
Solyc08g150131.1	39	36	30	4	3	4	16.1399	27.543	4.73675801	-2.557177763	4.73E-05	9.23E-05	down	--
Solyc07g055460.3	155	119	147	0	12	12	59.38377	109.343	9.424103	-3.511143371	4.82E-05	9.39E-05	down	Cytochrome P450 CYP4/CYP19/CYP26
Solyc02g069700.1	4	9	9	20	18	28	16.86765	5.71755	28.0177381	2.291511674	4.97E-05	9.66E-05	up	--
novel.1199	34	20	36	1	4	0	12.61394	23.1726	2.05531677	-3.46821333	5.25E-05	1.02E-04	down	--
Solyc02g072440.4	47	37	35	1	6	7	18.36894	31.134	5.60392291	-2.423365795	5.26E-05	1.02E-04	down	--
novel.2068	36	19	49	4	3	0	14.83543	26.5164	3.15442017	-3.139265581	5.28E-05	1.02E-04	down	--
Solyc03g093760.1	50	26	36	2	5	5	17.00624	29.0932	4.91928348	-2.536549764	5.35E-05	1.04E-04	down	Histone H3 (Lys9) methyltransferase
Solyc09g098610.2	24	32	27	0	1	1	11.25911	21.7329	0.78534192	-4.69543278	5.38E-05	1.04E-04	down	Cytochrome P450 CYP2 subfamily
Solyc05g012320.1	41	38	46	2	8	7	19.65942	32.4391	6.87972477	-2.198720465	5.45E-05	1.06E-04	down	Uncharacterized conserved protein
Solyc11g066255.1	31	34	25	1	3	3	13.26248	23.6726	2.8523127	-3.01255613	5.46E-05	1.06E-04	down	--
Solyc12g014580.2	16	6	20	28	23	53	27.25268	10.6789	43.8264325	2.026570111	5.46E-05	1.06E-04	up	--
Solyc03g115120.1	22	34	51	1	6	2	15.54867	27.4713	3.62600061	-2.878664319	5.50E-05	1.06E-04	down	Molecular chaperone (DnaJ superfamily)

Solyc02g069670.4	55	59	64	1	14	13	28.69978	46.3041	11.0954893	-2.013803677	5.59E-05	1.08E-04	down	Maltase glucoamylase and related hydrolases
Solyc11g020230.1	26	31	79	2	0	8	19.25633	34.3554	4.15724958	-3.037162978	5.64E-05	1.09E-04	down	Serine/threonine protein kinase
Solyc04g008245.1	14	7	3	14	28	40	20.034	6.3834	33.6846045	2.420251594	5.82E-05	1.12E-04	up	Purple (tartrate-resistant) acid phosphatase
Solyc03g116440.3	41	37	41	7	5	3	18.7898	30.9701	6.60954931	-2.267683047	5.96E-05	1.15E-04	down	PHD Zn-finger proteins
Solyc07g032070.3	48	27	46	4	4	7	18.76732	31.2214	6.31326884	-2.30509588	5.98E-05	1.15E-04	down	--
Solyc03g120050.3	36	34	16	0	1	1	11.82146	22.8576	0.78534192	-4.768105996	6.00E-05	1.16E-04	down	--
Solyc09g059550.1	27	18	54	0	2	4	13.70064	25.0394	2.36185275	-3.338584838	6.12E-05	1.18E-04	down	--
novel.1638	42	42	42	9	2	1	19.25616	32.8706	5.64168191	-2.632327863	6.12E-05	1.18E-04	down	--
Solyc01g091140.4	14	31	27	0	0	1	9.598329	18.8011	0.39558446	-5.3635466	6.38E-05	1.23E-04	down	--
Solyc04g008850.1	9	8	10	28	24	18	18.68644	7.00214	30.3707338	2.105060838	6.48E-05	1.25E-04	up	Aspartyl protease
Solyc01g080260.3	1	8	8	14	27	22	15.28939	4.40446	26.1743267	2.57497512	6.53E-05	1.26E-04	up	--
Solyc02g084173.1	40	32	38	3	3	7	17.01043	28.5936	5.42722444	-2.386642834	6.56E-05	1.26E-04	down	--
Solyc12g036650.2	36	43	59	7	4	9	22.0983	35.6033	8.59329861	-2.067618773	6.62E-05	1.27E-04	down	--
novel.472	26	21	49	2	1	4	13.69388	24.4231	2.96466919	-3.049870003	6.80E-05	1.30E-04	down	--
Solyc02g032170.4	46	32	62	6	3	10	21.99891	35.895	8.10283866	-2.158609161	7.11E-05	1.36E-04	down	--
Solyc02g078870.1	29	17	34	0	1	2	10.85652	20.5321	1.18092638	-4.038030211	7.18E-05	1.37E-04	down	--
Solyc07g026720.3	36	39	41	4	6	6	18.45004	30.2029	6.6971993	-2.164533239	7.31E-05	1.40E-04	down	Calcium transporting ATPase
novel.1349	50	37	43	1	6	10	20.31182	33.833	6.79067629	-2.268539645	7.44E-05	1.42E-04	down	FOG: Transposon-encoded proteins with TYA
Solyc02g093420.4	11	11	3	27	17	33	19.89279	6.70566	33.0799116	2.316076735	7.62E-05	1.46E-04	up	--
Solyc01g150162.1	27	23	30	2	1	2	11.45021	20.7269	2.17350027	-3.287263689	7.66E-05	1.46E-04	down	--
Solyc01g073910.4	8	14	3	19	17	56	22.47825	6.74843	38.2080586	2.523739363	7.70E-05	1.47E-04	up	Transcription factor HEX, contains HOX and HALZ domains
Solyc09g015700.4	52	20	49	5	3	5	18.33413	31.0396	5.62862941	-2.488151468	7.86E-05	1.50E-04	down	--
Solyc04g071083.1	6	1	19	46	27	20	23.8278	6.39126	41.2643402	2.656730413	8.14E-05	1.55E-04	up	--
Solyc07g005033.1	17	17	20	26	18	121	40.90167	14.0185	67.7848146	2.277218481	8.14E-05	1.55E-04	up	Plasma membrane H+-transporting ATPase
novel.619	25	28	23	1	2	0	10.60215	19.9285	1.27580186	-3.972697431	8.31E-05	1.58E-04	down	--
novel.593	45	33	53	6	8	6	21.11865	33.768	8.46928811	-2.000488389	8.35E-05	1.59E-04	down	--
Solyc07g064870.3	38	35	30	3	5	4	16.00916	26.9983	5.01998597	-2.416256931	8.37E-05	1.59E-04	down	--
Solyc10g005690.3	41	42	41	1	10	5	19.36964	32.3675	6.37178381	-2.292691957	8.59E-05	1.63E-04	down	Cl- channel CLC-3 and related proteins (CLC superfamily)
Solyc06g062320.3	26	28	24	0	0	3	10.8092	20.4316	1.18675338	-4.026038485	8.61E-05	1.63E-04	down	Flavin-containing monooxygenase
novel.678	77	32	24	4	1	7	20.1102	35.0764	5.14399647	-2.779713824	8.63E-05	1.64E-04	down	--
Solyc11g073180.2	4	5	10	18	29	14	15.30591	4.83751	25.7743138	2.399248959	8.70E-05	1.65E-04	up	--
Solyc03g112880.1	27	28	22	1	0	1	10.55646	20.221	0.89187141	-4.570369128	8.92E-05	1.69E-04	down	--
Solyc10g005610.2	24	27	27	0	2	0	10.55748	20.3354	0.77951491	-4.602790217	8.92E-05	1.69E-04	down	--
Solyc11g065250.1	36	29	53	2	7	6	18.17868	30.263	6.09438285	-2.280144257	8.98E-05	1.70E-04	down	FOG: PPR repeat
novel.403	26	14	48	2	0	2	12.00625	22.2288	1.78374282	-3.703947097	9.05E-05	1.71E-04	down	--
Solyc03g007690.1	38	46	58	9	9	3	22.94773	36.7343	9.16115303	-2.031825021	9.42E-05	1.78E-04	down	Transporter, ABC superfamily (Breast cancer resistance protein)
novel.2048	12	31	44	2	0	0	11.65387	22.3152	0.9925739	-4.687346374	9.76E-05	1.84E-04	down	FOG: Transposon-encoded proteins with TYA,
Solyc04g055160.3	27	40	29	3	5	1	14.53677	25.2403	3.83323259	-2.725592757	1.03E-04	1.93E-04	down	--
Solyc03g034010.3	6	6	11	10	39	23	17.57362	5.88538	29.2618529	2.316505205	1.06E-04	2.00E-04	up	FOG: Armadillo/beta-catenin-like repeats
Solyc09g011227.1	3	6	13	31	24	14	17.92139	5.56552	30.2772568	2.417392615	1.13E-04	2.12E-04	up	--
Solyc06g008667.1	11	8	9	24	10	55	22.43015	7.29468	37.5656066	2.366044924	1.18E-04	2.20E-04	up	--
novel.2022	47	28	38	5	4	6	17.87314	29.3323	6.41397133	-2.205326961	1.28E-04	2.38E-04	down	--
Solyc11g011880.2	57	53	40	9	1	10	24.12992	39.4477	8.8121846	-2.191656081	1.29E-04	2.41E-04	down	--
Solyc01g016720.2	29	25	11	0	0	1	8.845783	17.296	0.39558446	-5.240269945	1.30E-04	2.42E-04	down	--
Solyc04g007910.3	31	30	21	1	1	4	12.03571	21.603	2.46838225	-3.099378793	1.33E-04	2.47E-04	down	--
Solyc06g051680.1	17	23	34	0	1	2	10.1036	19.0263	1.18092638	-3.928562649	1.34E-04	2.49E-04	down	--
novel.816	22	27	31	1	0	4	11.41763	20.7566	2.07862479	-3.303311644	1.34E-04	2.49E-04	down	--
novel.1862	36	32	30	5	0	4	14.84659	25.6294	4.06377258	-2.718511191	1.35E-04	2.50E-04	down	--
Solyc02g072010.3	29	32	36	4	5	2	14.96269	25.2003	4.725104	-2.432271312	1.37E-04	2.53E-04	down	FOG: PPR repeat
Solyc07g017230.3	7	14	15	18	19	63	25.29928	9.33818	41.2603777	2.148672233	1.47E-04	2.72E-04	up	--
Solyc05g009500.3	4	11	23	19	23	75	28.83502	9.60733	48.062708	2.317266241	1.47E-04	2.72E-04	up	H+/oligopeptide symporter
Solyc06g082230.4	17	11	8	41	26	20	23.93989	9.48662	38.393148	2.011402558	1.51E-04	2.79E-04	up	Predicted K+/H+-antiporter

Solyc03g112210.1	34	24	29	3	4	1	13.0343	22.6251	3.44347513	-2.73589534	1.53E-04	2.82E-04	down	--
Solyc06g050870.3	57	47	25	8	2	5	20.46487	34.202	6.7277328	-2.389676045	1.54E-04	2.83E-04	down	--
Solyc02g092525.1	24	21	26	1	1	2	10.0489	18.4206	1.67721333	-3.449846833	1.57E-04	2.90E-04	down	--
Solyc01g020347.1	28	23	44	1	2	6	13.98613	24.323	3.64930862	-2.700530262	1.58E-04	2.91E-04	down	--
Solyc04g007425.1	41	24	32	3	5	3	14.90994	25.1955	4.62440151	-2.442283743	1.61E-04	2.95E-04	down	--
Solyc06g036420.3	35	29	20	2	0	4	12.36073	22.1466	2.57491174	-3.118376522	1.63E-04	2.98E-04	down	Suppressor of G2 allele of skp1
Solyc10g084890.3	18	29	53	5	0	2	14.3807	25.4888	3.27260366	-3.056776156	1.69E-04	3.09E-04	down	UDP-glucuronosyl and UDP-glucosyl transferase
Solyc04g076190.1	0	5	11	20	14	30	15.63219	4.0145	27.2498772	2.740671025	1.70E-04	3.11E-04	up	Aspartyl protease
Solyc01g087180.4	53	14	33	3	0	4	14.44624	25.8213	3.07119869	-3.10761093	1.75E-04	3.21E-04	down	SAP family cell cycle dependent protein
Solyc05g009170.3	38	27	29	3	4	4	14.57737	24.5245	4.63022851	-2.401442591	1.81E-04	3.30E-04	down	--
Solyc02g082410.4	9	6	8	18	26	16	15.68177	5.96733	25.3962103	2.087320349	1.91E-04	3.48E-04	up	Predicted transporter (major facilitator superfamily)
novel.1653	28	32	32	3	4	4	14.3068	23.9834	4.63022851	-2.369263208	1.97E-04	3.58E-04	down	--
novel.29	2	7	8	10	23	24	13.90576	4.3902	23.421318	2.421217716	1.98E-04	3.61E-04	up	--
Solyc12g055710.1	4	7	23	56	18	26	26.79114	8.48938	45.0928993	2.382952697	2.01E-04	3.65E-04	up	FOG: Predicted E3 ubiquitin ligase
Solyc08g082120.2	27	11	16	15	30	116	39.53354	14.0423	65.0248253	2.217147224	2.08E-04	3.77E-04	up	--
Solyc11g073170.1	3	10	11	25	31	13	17.91995	6.20764	29.6322529	2.247807457	2.09E-04	3.79E-04	up	FOG: Predicted E3 ubiquitin ligase
novel.343	35	26	37	5	5	1	15.08922	25.3526	4.82580649	-2.430462587	2.10E-04	3.81E-04	down	--
Solyc01g058520.4	33	20	36	2	5	2	13.31993	22.9073	3.7325301	-2.602735572	2.16E-04	3.91E-04	down	--
Solyc08g029360.4	35	27	41	4	4	6	16.25074	26.5838	5.91768438	-2.170535522	2.17E-04	3.92E-04	down	--
novel.2064	19	24	33	2	0	3	10.88881	19.5983	2.17932728	-3.204470815	2.17E-04	3.93E-04	down	--
Solyc03g025580.1	10	3	7	11	24	25	14.92956	5.15618	24.7029469	2.263889298	2.26E-04	4.09E-04	up	Pectin acetylesterase and similar proteins
Solyc11g005970.1	36	50	36	1	6	10	19.43918	32.0877	6.79067629	-2.192368001	2.28E-04	4.11E-04	down	FOG: PPR repeat
novel.1012	20	43	26	1	2	5	13.38107	23.5084	3.25372416	-2.814045792	2.31E-04	4.16E-04	down	--
Solyc02g032523.1	409	234	241	24	3	2	122.5443	231.217	13.8713281	-4.077417114	2.39E-04	4.29E-04	down	--
Solyc09g075660.3	7	12	11	26	8	62	24.18765	7.82754	40.5477568	2.375117217	2.39E-04	4.30E-04	up	--
Solyc01g096450.4	38	33	37	6	4	6	17.50751	28.1048	6.91025828	-2.044477706	2.48E-04	4.46E-04	down	Aspartyl protease
Solyc01g109340.4	48	32	52	5	2	12	21.02713	34.0463	8.00796318	-2.088021343	2.56E-04	4.59E-04	down	Predicted E3 ubiquitin ligase
Solyc12g020120.2	40	23	34	2	7	2	14.81932	25.1266	4.51204501	-2.451915866	2.57E-04	4.61E-04	down	--
Solyc01g058240.1	28	29	26	0	4	4	12.4294	21.7174	3.14136767	-2.715438226	2.74E-04	4.90E-04	down	--
Solyc04g005770.3	44	34	20	1	0	7	14.59823	25.9311	3.26537817	-2.95700903	2.83E-04	5.05E-04	down	FOG: PPR repeat
novel.1439	26	30	26	4	0	3	12.31917	21.4664	3.17190118	-2.82646641	2.83E-04	5.06E-04	down	--
Solyc05g043390.2	34	26	15	0	3	2	10.90686	19.8533	1.96044129	-3.25917614	2.90E-04	5.16E-04	down	--
Solyc05g026240.3	26	32	67	8	2	5	19.25383	31.7799	6.7277328	-2.290519493	2.92E-04	5.21E-04	down	--
Solyc05g053570.4	34	32	37	6	4	5	16.63951	26.7643	6.51467382	-2.064702484	3.09E-04	5.48E-04	down	Pleiotropic drug resistance proteins (PDR1-15)
Solyc02g087320.2	41	33	31	7	5	2	16.84346	27.473	6.21396485	-2.186620877	3.42E-04	6.05E-04	down	--
Solyc09g075610.3	31	29	56	8	4	4	18.38111	29.6506	7.11166326	-2.106301842	3.43E-04	6.06E-04	down	GATA-4/5/6 transcription factors
Solyc04g016440.4	35	16	40	0	6	2	13.20061	23.2715	3.12971366	-2.826922969	3.52E-04	6.20E-04	down	Predicted membrane protein
Solyc06g065670.4	7	3	13	13	27	22	15.73301	5.78798	25.6780398	2.135876774	3.54E-04	6.24E-04	up	Pleiotropic drug resistance proteins (PDR1-15)
novel.1185	30	36	24	0	1	7	13.44366	23.7285	3.15884868	-2.845284793	3.66E-04	6.44E-04	down	--
Solyc08g079740.3	24	23	26	0	4	2	10.66488	18.9796	2.35019875	-2.93493829	3.74E-04	6.59E-04	down	--
Solyc04g014220.1	20	31	37	3	5	2	13.50021	22.7716	4.22881705	-2.431743267	3.89E-04	6.83E-04	down	FOG: Predicted E3 ubiquitin ligase
Solyc03g083730.1	22	42	28	0	1	7	13.69703	24.2352	3.15884868	-2.877275368	3.90E-04	6.84E-04	down	--
Solyc02g091710.3	45	20	35	1	7	4	15.32956	25.8522	4.80692698	-2.382469989	4.08E-04	7.14E-04	down	--
Solyc12g013520.3	26	29	38	5	1	5	14.44553	24.0419	4.8491145	-2.350280993	4.08E-04	7.14E-04	down	--
Solyc04g082280.3	41	38	37	1	11	0	17.54075	30.2979	4.78361897	-2.619066002	4.09E-04	7.16E-04	down	--
Solyc04g009260.3	36	20	28	0	0	6	12.08661	21.7997	2.37350676	-3.136950921	4.09E-04	7.16E-04	down	Apoptotic ATPase
Solyc03g112780.1	28	27	21	4	0	2	11.37259	19.9689	2.77631672	-2.934863875	4.12E-04	7.21E-04	down	--
novel.563	18	57	61	3	9	7	21.49178	35.2178	7.76576918	-2.158295158	4.12E-04	7.21E-04	down	--
Solyc01g098790.2	17	28	27	1	0	4	10.41846	18.7583	2.07862479	-3.157012182	4.22E-04	7.37E-04	down	--
Solyc09g010070.1	29	28	37	2	5	6	14.81755	24.3202	5.31486794	-2.165201343	4.32E-04	7.53E-04	down	FOG: PPR repeat
Solyc08g080150.1	28	27	31	2	5	4	13.43586	22.348	4.52369902	-2.279359684	4.50E-04	7.84E-04	down	--
Solyc02g069680.3	25	25	26	2	4	2	11.57327	19.8038	3.34277264	-2.555812966	4.54E-04	7.90E-04	down	Vacuolar assembly/sorting protein DID4
Solyc01g105430.4	28	22	45	5	3	4	14.75721	24.2814	5.23304495	-2.247599437	4.70E-04	8.16E-04	down	Transporter, ABC superfamily
Solyc10g084510.1	23	12	37	3	0	1	10.07074	18.257	1.88444531	-3.394281392	4.88E-04	8.46E-04	down	--
novel.449	27	11	26	0	2	0	8.600459	16.4214	0.77951491	-4.302436187	4.92E-04	8.52E-04	down	--

Solyc06g009900.3	30	22	27	2	3	4	12.13678	20.5294	3.74418411	-2.440573989	4.97E-04	8.61E-04	down	Uncharacterized conserved protein, contains ENT domain
Solyc09g090990.2	6	0	0	67	26	58	33.9601	1.59139	66.3288181	5.386886669	5.17E-04	8.93E-04	up	--
Solyc12g036810.3	23	22	26	2	3	2	10.69393	18.4348	2.95301519	-2.641894707	5.39E-04	9.29E-04	down	Kinesin (KAR3 subfamily)
Solyc11g005390.3	25	22	26	4	0	2	10.87081	18.9653	2.77631672	-2.863284893	5.45E-04	9.39E-04	down	--
Solyc11g150136.1	30	30	36	7	3	3	15.36828	24.9065	5.83003439	-2.149079041	5.49E-04	9.46E-04	down	--
Solyc07g043490.1	24	19	34	1	3	4	11.50641	19.7649	3.24789716	-2.569555606	5.61E-04	9.65E-04	down	UDP-glucuronosyl and UDP-glucosyl transferase
Solyc08g077550.3	31	36	21	4	0	5	13.62152	23.28	3.9630701	-2.587964351	5.85E-04	1.00E-03	down	--
Solyc03g093250.2	25	44	43	1	10	5	17.76519	29.1586	6.37178381	-2.147021537	5.91E-04	1.01E-03	down	Structural maintenance of chromosome protein 2
Solyc04g008910.3	5	4	18	7	32	45	20.23706	6.72658	33.747548	2.31845856	6.22E-04	1.07E-03	up	--
Solyc07g054110.2	19	23	36	3	4	1	11.73802	20.0326	3.44347513	-2.562162371	6.43E-04	1.10E-03	down	FOG: PPR repeat
Solyc03g093500.3	40	22	30	4	2	6	14.51681	23.8954	5.13816947	-2.230204961	6.55E-04	1.12E-03	down	Uncharacterized conserved protein WDR8, contains WD repeats
Solyc01g108440.2	33	22	31	3	2	6	13.45931	22.2767	4.64188252	-2.263182805	6.87E-04	1.17E-03	down	--
Solyc11g011110.3	23	44	29	1	6	6	15.25284	25.2973	5.20833845	-2.23268203	7.63E-04	1.29E-03	down	--
Solyc02g091080.2	27	28	23	4	4	1	12.19936	20.459	3.93976208	-2.408811932	8.11E-04	1.37E-03	down	Uncharacterized membrane protein
Solyc08g016310.3	50	21	37	4	9	3	17.30669	27.9337	6.67971828	-2.056698457	8.83E-04	1.49E-03	down	FOG: Leucine rich repeat
Solyc02g077210.1	23	28	27	2	6	1	12.0382	20.3497	3.7267031	-2.430376003	8.92E-04	1.50E-03	down	--
Solyc05g018870.3	23	21	29	5	0	0	10.67527	18.8691	2.48143474	-3.078161308	9.10E-04	1.53E-03	down	Endosomal membrane proteins, EMP70
Solyc04g081590.4	65	37	58	42	149	239	107.4214	41.3802	173.462599	2.069115406	9.26E-04	1.56E-03	up	--
Solyc10g085610.2	32	17	50	6	4	3	15.42897	25.1344	5.7235049	-2.174251848	9.61E-04	1.61E-03	down	--
Solyc01g086820.4	34	19	31	2	2	7	13.12234	21.7035	4.54118003	-2.238255784	1.26E-03	2.09E-03	down	Histones H3 and H4
Solyc06g082320.4	20	34	40	6	5	3	15.21854	24.3238	6.11326236	-2.021945883	1.26E-03	2.09E-03	down	--
Solyc01g014105.1	35	17	39	2	1	8	13.93004	23.3131	4.54700703	-2.34318935	1.28E-03	2.12E-03	down	--
Solyc05g010590.4	25	23	40	2	4	7	13.94815	22.5756	5.32069494	-2.060821587	1.35E-03	2.23E-03	down	--
Solyc10g055470.2	33	56	73	104	64	359	130.1724	41.7718	218.573141	2.387513767	1.52E-03	2.49E-03	up	Beta-galactosidase
novel.1254	27	15	33	5	1	2	11.43356	19.2048	3.66236112	-2.466329836	1.54E-03	2.52E-03	down	--
Solyc12g009240.1	34	149	323	12	8	22	72.64222	127.508	17.7763612	-2.843453875	1.57E-03	2.56E-03	down	--
Solyc05g052820.3	34	18	33	4	2	6	13.51901	21.8998	5.13816947	-2.105967739	1.60E-03	2.61E-03	down	Amino acid transporters
Solyc01g056320.1	24	24	35	1	8	2	12.9029	21.4003	4.40551552	-2.236503175	1.64E-03	2.67E-03	down	--
Solyc07g052840.1	30	34	20	3	7	2	13.61309	22.2178	5.00833196	-2.136076292	1.72E-03	2.80E-03	down	--
Solyc07g053570.4	34	15	30	2	2	6	12.24662	20.3476	4.14559557	-2.281127251	1.72E-03	2.80E-03	down	FOG: Zn-finger
Solyc12g100270.2	16	8	45	138	89	33	66.70804	17.1858	116.2303	2.751160412	1.73E-03	2.82E-03	up	--
Solyc11g071740.2	245	344	324	17	20	89	144.8245	238.21	51.4390442	-2.207647498	1.80E-03	2.92E-03	down	Calmodulin and related proteins (EF-Hand superfamily)
Solyc10g085500.2	25	28	27	4	2	6	13.00916	20.8802	5.13816947	-2.034229604	1.91E-03	3.09E-03	down	Cytochrome P450 CYP2 subfamily
Solyc04g010330.3	30	31	23	0	5	7	13.4055	22.0931	4.7178785	-2.165473356	2.15E-03	3.45E-03	down	--
Solyc07g005770.2	35	22	33	8	3	2	14.60688	23.283	5.93073688	-2.036205113	2.16E-03	3.47E-03	down	Apoptotic ATPase
Solyc01g005510.3	34	35	71	89	52	353	119.8851	35.6919	204.078241	2.514764521	2.22E-03	3.57E-03	up	Multicopper oxidases
novel.1271	28	23	33	1	2	9	13.27098	21.7059	4.836062	-2.12871144	2.54E-03	4.05E-03	down	--
Solyc02g070760.4	48	23	21	4	8	1	14.82717	24.1555	5.49879191	-2.135471714	2.68E-03	4.27E-03	down	Predicted dehydrogenase
Solyc02g083880.3	20	26	28	3	6	2	11.92576	19.2329	4.61857451	-2.053403288	2.78E-03	4.40E-03	down	--
Solyc03g095820.4	30	16	27	6	0	0	10.91509	18.8525	2.97772169	-2.792479928	2.92E-03	4.62E-03	down	--
Solyc05g050900.4	25	27	19	2	4	5	11.61344	18.6973	4.52952602	-2.019672756	3.48E-03	5.45E-03	down	--
Solyc08g080670.1	131	36	319	459	359	490	341.1284	120.702	561.555022	2.216865273	3.98E-03	6.20E-03	up	--
Solyc01g056310.3	46	54	48	75	49	296	106.0628	38.7129	173.412637	2.164478692	4.02E-03	6.26E-03	up	Multicopper oxidases
Solyc07g026730.1	24	26	14	1	3	5	10.30327	16.9631	3.64348162	-2.177633206	5.02E-03	7.72E-03	down	Calcium transporting ATPase
Solyc12g044950.3	45	3	86	2	6	8	19.86517	33.2345	6.49579432	-2.339278793	5.45E-03	8.34E-03	down	--
Solyc01g066420.3	24	27	36	40	29	187	63.8027	22.4767	105.128738	2.22633513	5.53E-03	8.46E-03	up	--
Solyc09g090790.3	11	28	30	0	0	7	10.32487	17.8807	2.76909122	-2.640511671	6.46E-03	9.78E-03	down	Transcription factor, Myb superfamily
Solyc08g150139.1	23	17	35	7	0	3	11.9197	19.1786	4.66076202	-2.11612534	7.57E-03	1.13E-02	down	--
Solyc07g009510.1	66	14	276	686	378	343	355.2746	87.0826	623.466635	2.838716919	7.70E-03	1.15E-02	up	Predicted chitinase
Solyc03g112620.4	24	11	30	0	4	5	10.05716	16.5774	3.53695213	-2.172919364	7.83E-03	1.17E-02	down	--
Solyc06g059720.3	942	761	1384	234	40	47	471.0632	791.813	150.313914	-2.399413742	8.14E-03	1.22E-02	down	--
novel.1973	8	6	5	73	1	44	29.50639	4.98835	54.024421	3.436328232	1.09E-02	1.59E-02	up	--

Solyc10g079860.2	15	0	20	81	30	82	46.53333	8.73677	84.3298923	3.265958441	1.12E-02	1.64E-02	up	--
Solyc06g084180.3	3	10	10	26	2	27	15.16674	5.96972	24.363756	2.020317424	1.28E-02	1.86E-02	up	--
Solyc10g075150.2	34	7	129	288	173	98	145.3956	41.6653	249.125958	2.577572313	1.66E-02	2.37E-02	up	--
Solyc10g055820.3	7	1	40	91	42	41	44.70179	11.6527	77.7508884	2.730869801	2.50E-02	3.47E-02	up	Predicted chitinase
novel.214	179	35	82	22	4	15	47.58932	76.7675	18.4111096	-2.070000127	2.56E-02	3.56E-02	down	--

Table S4 Differential accumulation metabolites (DAMs) between OH 88119 and *yfm* in metabolome

										0 DPA											
**Index**	**Compounds**	**Class I**	**Class II**	**Formula**	**Level**	**score**	**CAS**	**yfm-0DPA-1**	**yfm-0DPA-2**	**yfm-0DPA-3**	**OH88119-0DPA-1**	**OH88119-0DPA-2**	**OH88119-0DPA-3**	**VIP**	**P-value**	**FDR**	**Fold_Change**	**Log2FC**	**Type**	**cpd_ID**	**kegg_map**
MW0139382	Plantaginin	Heterocyclic compounds	Heterocyclic compounds	C21H20O11	2	0.9208	26046-94-6	4121.19	12121.48	16133.05	1667.33	5202.45	6737.45	1.03594251	0.21139334	0.60186131	2.379302768	1.250538867	up	C17056	-
MW0126018	N-[2-(5-hydroxy-1H-indol-3-yl)ethyl]hexadecanamid	Lipids	Free fatty acids	C26H42N2O2	2	0.8911	212707-51-2	162534.03	164387.66	159416.47	339665.08	387813.56	366730.17	1.756679	0.00434928	0.16416683	0.444465586	-1.16985638	down	-	-
MW0104181	3-Oxoglutaric acid	Organic acids	Organic acids	C5H6O5	2	0.8485	542-05-2	2588.8	7488.63	6340.86	21124.92	19615.74	19244.54	1.60032887	0.00485012	0.16746065	0.273705681	-1.86930272	down	-	-
MW0108764	Neopine	Benzene and substituted derivatives	Benzene and substituted derivatives	C18H21NO3	2	0.8401	467-14-1	27641.21	20699.3	47192	11.06	17.24	287.57	1.69064203	0.05712718	0.37920186	302.4424922	8.240517037	up	C09594	-
MW0155976	Pro-Pro-Gly	Amino acids and derivatives	Amino acids and derivatives	C12H19N3O4	2	0.8244	16875-10-8	7235.5	3267.81	3774.55	1959.78	1163.49	1377.91	1.57901543	0.11571487	0.49367245	3.172026002	1.665404597	up	-	-
MW0117084	4-Cyanoindole	Alkaloids	Plumerane	C9H6N2	2	0.7857	16136-52-0	418.06	1709.98	972.21	1613.62	3348.79	2166.85	1.28990001	0.10785987	0.48477095	0.434862805	-1.20136778	down	-	-
MW0169534	N-benzyl-1-methyl-1H-pyrazolo[3,4-d]pyrimidin-4-amine	Benzene and substituted derivatives	Benzene and substituted derivatives	C13H13N5	2	0.777	105903-56-8	9462.39	10626.09	10900.72	4102.28	2417.49	4466.53	1.66523561	0.00159243	0.13083964	2.82071307	1.496059919	up	-	-
MW0006472	Cannabidiolic acid	Others	Others	C22H30O4	2	0.7687	1244-58-2	3890.61	2120.42	1207.39	10203.57	6522.83	6472.21	1.52108638	0.02915737	0.30170986	0.311157436	-1.68428337	down	-	-
MW0138536	Isorhoifolin	Heterocyclic compounds	Heterocyclic compounds	C27H30O14	2	0.7668	552-57-8	1143.5	2705.74	1022.88	10836.29	5335.57	2874.46	1.43704006	0.17725882	0.56946912	0.255803746	-1.9668907	down	-	-
MEDL00348	Asp-Tyr	Amino acids and derivatives	Amino acids and derivatives	C13H16N2O6	2	0.7656	22840-03-5	17372.59	20957.52	20512.96	49051.66	38809.21	34523.62	1.67757125	0.03214697	0.31330981	0.480804961	-1.05647631	down	-	-
MW0169766	Resorcinol sulfoxide	Benzene and substituted derivatives	Benzene and substituted derivatives	C12H10O5S	2	0.765	62845-75-4	164.06	79.66	117.28	0.02	0.02	0.02	1.76154102	0.03879005	0.32974696	6016.666667	12.55474872	up	-	-
MW0107972	Tyr-Gly-Gly	Amino acids and derivatives	Amino acids and derivatives	C13H17N3O5	2	0.7606	21778-69-8	199246.85	191971	258207.29	10229.85	661.17	822.51	1.65875998	0.00843907	0.20100334	55.44230817	5.792915416	up	-	-
MW0112752	Kynuramine	Alcohol and	Amines	C9H12N2O	2	0.759	363-36-0	5427.37	14966.84	4554.01	2818.88	2897.59	4071.56	1.23426586	0.26800018	0.65020132	2.548849973	1.349846458	up	-	-
MW0106675	Ethyl N-acetyl-L-tyrosinate	Amino acids and derivatives	Amino acids and derivatives	C13H17NO4	2	0.7586	840-97-1	1413.68	289.47	628.4	9622.35	4710.62	3102.47	1.53786513	0.12027715	0.49926787	0.133724758	-2.9026615	down	C01657	-
MW0125062	Methyl pyrimidine-2-carboxylate	Others	Lactones	C6H6N2O2	2	0.7567	34253-03-7	1657.83	1257.48	530.15	289.47	386.83	276.83	1.48979582	0.12665444	0.50962303	3.614889889	1.853951703	up	-	-
MW0000275	Harman	Alkaloids	Alkaloids	C12H10N2	2	0.7556	486-84-0	13920.39	6958.02	9642.23	2964.81	1891.82	1763.46	1.67824321	0.05462937	0.37690654	4.610305902	2.204862479	up	C09209	-
MEDN0493	4-Methoxycinnamic	Phenolic acids	Phenolic acids	C10H10O3	2	0.7554	943-89-5	1204.25	29.65	42.21	1168.08	783.88	1017.57	1.16358652	0.28172167	0.66285701	0.429734672	-1.21848191	down	-	-
MW0102977	Triptophenolide	Terpenoids	Ditepenoids	C20H24O3	2	0.755	74285-86-2	45452.39	40570.9	41318.71	20.62	25.03	13.66	1.75829826	0.00127753	0.12524365	2147.057832	11.06814534	up	-	-
MW0115164	Populin	Benzene and substituted derivatives	Benzene and substituted derivatives	C20H22O8	2	0.7547	99-17-2	5442.18	1795.81	845.91	7167.66	15815.46	4270.62	1.28020705	0.19853866	0.5893935	0.296616171	-1.75333084	down	-	-
MW0012920	1-Octadecyl Lysophosphatidic Acid	GL	LPA	C21H45O6P	2	0.7539	52977-29-4	3788.36	2965.48	8742.19	3436.03	1597.67	1161.13	1.26340225	0.22152996	0.61514616	2.50144556	1.322762055	up	-	-
MW0007840	Monoethyl phthalate	Benzene and substituted derivatives	Benzene and substituted derivatives	C10H10O4	2	0.7513	2306-33-4	1020.26	845.64	650.21	195.39	185.45	702.98	1.33482899	0.08937173	0.45596986	2.321520179	1.215069821	up	-	-
MW0058015	1,2-Dimyristoyl-sn-glycero-3-phosphoethanolamine	GL	PE	C33H66NO8P	2	0.7489	998-07-2	5356.97	3726.6	4254.62	197.57	951.64	213.78	1.61950579	0.00513979	0.17342912	9.785977887	3.290716023	up	-	-
MW0141122	Dioleoylphosphatidylcholine	GL	PC	C44H84NO8P	2	0.7481	52088-89-8	47191.9	46216.11	32395.04	206100.08	63995.19	82459.44	1.35618384	0.23130018	0.62596031	0.356832703	-1.48668025	down	-	-
MW0011852	1,2-Didecanoyl-sn-glycero-3-phosphocholine	GL	PC	C28H56NO8P	2	0.7475	3436-44-0	8551.86	20448.5	31554.24	68005.77	47250.99	50304.86	1.43224629	0.0195418	0.27199747	0.365752642	-1.45105981	down	-	-
MW0054001	Hypaconitine	Alkaloids	Terpenoid alkaloids	C33H45NO10	2	0.7441	6900-87-4	23496.52	21641.06	26579.7	11316.16	5922.4	10435.36	1.63719882	0.00285477	0.14452248	2.59151143	1.373793757	up	C08688	-
MW0112009	2,6-Dimethyl-4-hydroxybenzaldehyde	Benzene and substituted derivatives	Benzene and substituted derivatives	C9H10O2	2	0.7418	70547-87-4	19688.8	14432.73	32937.22	17.27	85.27	41.29	1.73795095	0.05586334	0.37756499	466.2361816	8.864917157	up	-	-
MW0128411	(Z)-Resveratrol	Phenolic acids	Phenolic acids	C14H12O3	2	0.7402	61434-67-1	4841.26	3263.48	3192.92	808.19	1135.79	756.33	1.70146118	0.02870423	0.30152977	4.183838152	2.064827043	up	-	-
pme3504	Ononin	Flavonoids	Isoflavones	C22H22O9	2	0.7383	486-62-4	1506.43	1584.48	604.45	2696.96	1206.17	3906.28	1.07838212	0.21426379	0.60438945	0.473193237	-1.07949864	down	C10509	ko00943
MW0159301	Val-Tyr	Amino acids and derivatives	Amino acids and derivatives	C14H20N2O4	2	0.7374	3061-91-4	2930.99	4860.63	2358.24	22966.83	19318.85	3733.7	1.30567243	0.17796437	0.56963734	0.220556209	-2.18078172	down	-	-
MW0121600	5-Methoxytryptophol	Amino acids and derivatives	Amino acids and derivatives	C11H13NO2	2	0.7366	712-09-4	10015.26	12391.66	11896.08	29385.55	21222.26	24229.03	1.68748552	0.02211494	0.27856654	0.458370503	-1.12541389	down	-	-
MW0116893	1-Benzyl-4-hydroxy-5-oxo-2,5-dihydro-1H-pyrrole-3-carboxylic acid ethyl ester	Benzene and substituted derivatives	Benzene and substituted derivatives	C14H15NO4	2	0.73	4450-98-0	15715.23	13646.49	13641.75	1790.84	228.19	682.6	1.65336925	0.00020596	0.07934638	15.9176016	3.992551067	up	-	-
MW0055340	N-(9Z-octadecenoyl)-sphing-4-enine-1-phosphocholine	Lipids	SM	C41H81N2O6P	2	0.7278	108392-10-5	9495.99	5566.84	8678.09	5.5	746.58	62.89	1.50311541	0.02032976	0.2743902	29.1310355	4.86448508	up	-	-
MW0106445	Dihydromethysticin	Benzene and substituted derivatives	Benzene and substituted derivatives	C15H16O5	2	0.7262	19902-91-1	679.96	757.3	582.39	378.15	149.15	222.62	1.56159982	0.00901186	0.2053328	2.693153936	1.429296694	up	C09926	-

MW0121428	5-Ethyl-2-nitro-9h-carbazole	Heterocyclic compounds	Heterocyclic compounds	C14H12N2O2	2	0.7253	5419-84-1	309525.96	285901.9	242850.04	1676.12	22304.96	21834.67	1.56495592	0.00252225	0.14452248	18.29671892	4.193513054	up	-	-
MW0054763	Menthone	Terpenoids	Monoterpenoids	C10H18O	2	0.7253	14073-97-3	11806.57	11631.06	36893.39	11295.02	8064.47	7888.91	1.14228681	0.31872495	0.686129	2.214112388	1.146728455	up	C00843	ko00902,ko01110
MW0169689	Pomiferin	Flavonoids	Other Flavonoids	C25H24O6	2	0.7193	572-03-2	143055.19	49978.19	178149.49	557.35	727.69	37.34	1.66722347	0.08422893	0.44308302	280.6930459	8.132849512	up	-	-
MW0103027	Umbelliprenin	Lignans and Coumarins	Coumarins	C24H30O3	2	0.7184	532-16-1 | 30413-87-7 | 23838-17-7	12711.72	5683.5	6451.46	32047.07	29533.62	22603.91	1.61580679	0.0062069	0.1850017	0.295145193	-1.76050325	down	-	-
MW0012772	Glyceryl arachidonate	GL	MG	C23H38O4	2	0.7183	35474-99-8	2774.59	7305.42	2255.48	7535.06	8566.93	8792.65	1.3644595	0.11360499	0.49245668	0.495507868	-1.01302013	down	-	-
MW0118517	2-Ethyl-4-methylthiazole	Heterocyclic compounds	Heterocyclic compounds	C6H9NS	2	0.7153	15679-12-6	4266.96	8112.91	5430.2	23001.16	12570.92	8005.65	1.35636877	0.18674489	0.57922127	0.408696598	-1.29089786	down	-	-
MW0137107	7-Methoxyflavanone	Others	Ketone compounds	C16H14O3	2	0.7123	21785-09-1	265.03	247.59	800.07	1788.64	1143.72	1713.44	1.4993806	0.01556869	0.24803772	0.282554135	-1.82340079	down	-	-
MW0115788	tert-Butyl 1H-indol-4-ylcarbamate	Organic acids	Organic acids	C13H16N2O2	2	0.7081	819850-13-0	15738.97	8140.92	2641.96	26466.56	14097.19	13787.49	1.11677447	0.17623538	0.56921854	0.487971388	-1.03513154	down	-	-
MW0105524	Ala-Lys	Amino acids and derivatives	Amino acids and derivatives	C9H19N3O3	2	0.707	6366-77-4	4494.55	22290.06	16034.24	26719.28	30013.27	33093.85	1.23084185	0.08184946	0.43607355	0.476684471	-1.06889347	down	-	-
MW0012822	Azelaoyl PAF	GL	PA	C33H66NO9P	2	0.7043	354583-69-0	74.75	41.22	58.46	0.02	0.02	2.94	1.56949891	0.02651149	0.29249362	58.53355705	5.871192048	up	-	-
MW0003591	(3,5-Dimethoxyphenyl)(4-methoxyphenyl)methano[illegible]	Benzene and substituted derivatives	Benzene and substituted derivatives	C16H16O4	2	0.7042	94709-12-3	463.99	248.45	579.91	523.68	684.81	2146.26	1.07506208	0.31331961	0.68542982	0.385229898	-1.37620842	down	-	-
MEDL00809	Tryptamine	Alkaloids	Plumerane	C10H12N2	3	0.9821	61-54-1	7250.66	5483.86	5982.32	23463.49	6485.88	17728.03	1.22688436	0.19127534	0.58497036	0.392572582	-1.34896868	down	C00398	ko00380,ko01100,ko01110
MW0009905	4-Hydroxy-3-(2'-hydroxy-[1,1'-biphenyl]-4-yl)-6-oxo-6,7-dihydrothieno[2,3-b]pyridine-5-carbonitrile	Benzene and substituted derivatives	Benzene and substituted derivatives	C20H12N2O3S	3	0.9489	844499-71-4	10539.71	19861.7	9475.27	43101.1	24703.47	104644.84	1.43074885	0.20722049	0.59893581	0.231236976	-2.11255599	down	-	-
MEDL02018	(2E)-Decenoyl-ACP	Amino acids and derivatives	Amino acids and derivatives	C6H11NO2	3	0.9424	52-52-8	13960.91	29511.53	26341.49	61406.61	24251.03	105127.23	1.17091885	0.22340161	0.61812661	0.365930118	-1.45035993	down	C03969	-
MEDN0198	Citric acid	Organic acids	Organic acids	C6H8O7	3	0.9411	77-92-9	393.99	433.88	692.95	13294.76	10996.19	5986.46	1.72699813	0.04687092	0.35503927	0.050229528	-4.31532048	down	C00158	ko00020,ko00250,ko00630,ko01100,ko01110,ko01200,ko01210,ko01230,ko01240
MW0124001	Ethyl 4-hydroxy-5,8-dimethoxyquinoline-3-carboxylate	Others	Lactones	C14H15NO5	3	0.94	5428-19-3	26693.36	30452.8	32019.64	4614.88	475.65	4404.17	1.54768654	0.0002494	0.07934638	9.391112937	3.231296141	up	-	-
MW0141085	1,2-Anthracenediol	Benzene and substituted derivatives	Benzene and substituted derivatives	C14H10O2	3	0.9369	577-95-7 | 70143-54-3	3800.57	10831.75	2176.11	52276.1	27804.23	40977.98	1.61314478	0.02721015	0.29249362	0.138845735	-2.84844524	down	-	-
MEDP0367	γ-Aminobutyric Acid	Organic acids	Organic acids	C4H9NO2	3	0.9121	56-12-2	4737.99	1750.74	6489.34	37100.5	8547.12	7868.84	1.21680039	0.29483817	0.67656321	0.242506137	-2.04390684	down	C00334	ko00250,ko00330,ko00410,ko00650,ko00760,ko01100
MEDL01744	Heterophylliin A	Tannins	Tannin	C34H26O22	3	0.8984	87687-52-3	908.67	1529.95	698.53	1388.18	4740.37	9323.65	1.33857325	0.21508892	0.60477561	0.203022871	-2.30028584	down	-	-
MEDL02308	Nicotinic acid adenine dinucleotide	Nucleotides and derivatives	Nucleotides and derivatives	C21H27N6O15P2+	3	0.8967	6450-77-7	770414.94	263265.56	242865.62	80480.86	273705.73	221108.43	1.03434426	0.30721107	0.68381714	2.218941718	1.149871775	up	C00857	ko00760,ko01100,ko01240
MEDL02307	p-Coumaroylagmatine	Alkaloids	Phenolamine	C14H20N4O2	3	0.8586	7295-86-5	2550.08	2244.61	792.87	3648.28	3906.48	3706.58	1.32896077	0.07038421	0.40874139	0.496171859	-1.01108818	down	C04498	ko00999,ko01100,ko01110
MEDP0685	S-Methyl-L-cysteine	Amino acids and derivatives	Amino acids and derivatives	C4H9NO2S	3	0.8393	1187-84-4	910.87	13107.36	981.84	27679.81	10029.05	13042.23	1.29400293	0.16016037	0.54886804	0.295561534	-1.75846957	down	-	-
MW0059173	Peimine	Alkaloids	Alkaloids	C27H45NO3	3	0.813	23496-41-5 | 107299-20-7 | [illegible]	3720.77	8365.27	1574.66	16503.63	9866.84	6269.98	1.22033987	0.16454895	0.55155116	0.418520578	-1.25662954	down	-	-
MW0114510	Gastrodin	Phenolic acids	Phenolic acids	C13H18O7	3	0.7929	62499-27-8	3522.14	8119.28	1968.52	23755.93	6026.28	6986.42	1.12256545	0.31090057	0.68402525	0.37015086	-1.43381472	down	C16964	-
MEDP1962	IAA-L-Ala	Amino acids and derivatives	Amino acids and derivatives	C13H14N2O3	3	0.7905	57105-39-2	21071.86	3516.65	3573.69	3104.46	388.97	1876.41	1.21846565	0.32257426	0.68977114	5.244513803	2.390809033	up	-	-
MEDN0067	Phenylalanylphenylalanine	Amino acids and derivatives	Amino acids and derivatives	C18H20N2O3	3	0.787	201023-39-4	108841.59	49921.39	62242.21	26401.1	16211.92	22012.43	1.61980461	0.0971568	0.47241966	3.419785704	1.773905923	up	-	-
MW0126505	Salsolinol	Heterocyclic compounds	Heterocyclic compounds	C10H13NO2	3	0.7863	27740-96-1	38200.84	25987.14	32156.57	79030.72	60078.1	56548.79	1.6068063	0.0248079	0.28869482	0.492414019	-1.02205626	down	C09642	-
MEDL02311	Moschamine	Alkaloids	Plumerane	C20H20N2O4	3	0.7845	68573-23-9	5862.12	6915.08	1808.84	29051.79	7731.8	10119.21	1.24574118	0.24812555	0.63798289	0.310984419	-1.6850858	down	-	-
MW0000390	Oxymatrine	Alkaloids	Quinorisidine alkaloids	C15H24N2O2	3	0.7738	16837-52-8 | 54809-74-4	7680.38	4553.36	6996.92	518.47	380.96	118.7	1.68216857	0.02220693	0.27856654	18.88821663	4.239414588	up	-	-
MEDP0289	3,4,5-Trimethoxycinnamic acid	Phenolic acids	Phenolic acids	C12H14O5	3	0.772	90-50-6	660.79	1292.49	499.11	803.91	198.58	83.07	1.08440682	0.2393265	0.63224212	2.259101293	1.17574896	up	-	-
MEDP1503	N-Acetylhistamine	Alcohol and [illegible]	Amines	C7H11N3O	3	0.7709	673-49-4	12966.92	9533.31	1450.65	58802.76	11610.96	12875.93	1.07323851	0.32985227	0.69431097	0.2875613	-1.79805857	down	C05135	ko00340
MEDP1475	N-Methylhydantoin	Heterocyclic compounds	Heterocyclic compounds	C4H6N2O2	3	0.7699	616-04-6	20350.1	29066.82	56895.42	785.29	23.99	9324.49	1.35807271	0.0917448	0.45963526	10.49089727	3.391066169	up	C02565	ko00330,ko01100
MEDP0664	Tyrosine methylester	Amino acids and derivatives	Amino acids and derivatives	C10H13NO3	3	0.7691	1080-06-4	2500.55	3931.27	3525.21	3645.48	10360.05	10047.3	1.30645115	0.16054499	0.54886804	0.41396501	-1.27241927	down	C03404	-
MW0008809	N-Acetyltyramine	Phenolic acids	Phenolic acids	C10H13NO2	3	0.7615	1202-66-0	1823.01	1218.71	1635.83	109.45	1020.99	1003	1.07454626	0.08717747	0.45097379	2.192491938	1.132571538	up	-	-
MEDN1192	2',6'-Dihydroxyacetophenone	Phenolic acids	Phenolic acids	C8H8O3	3	0.7579	699-83-2	868.88	686.91	369.69	6095.81	4245.03	4244.62	1.70454369	0.01654328	0.25364283	0.132013663	-2.92124084	down	-	-
MW0117890	2,4-Diamino-6-hydroxypyrimidine	Heterocyclic compounds	Heterocyclic compounds	C4H6N4O	3	0.7543	56-06-4 | 100643-27-4 | 143504-99-8 | 195-66-4	5773.53	837.54	887.45	4297.15	10276.53	2360.45	1.01131758	0.34496393	0.70481795	0.442805151	-1.17525609	down	-	-
MEDL02423	Phosphocreatine	Organic acids	Organic acids	C4H10N3O5P	3	0.7528	67-07-2	90195.24	89173.57	81850.87	10166.29	8865.96	6462.67	1.74674563	0.00023764	0.07934638	10.24595017	3.356981876	up	C02305	ko00330,ko01100
MW0110480	2,6-Dihydroxypyridine	Others	Vitamin	C5H5NO2	3	0.7518	626-06-2	16913.63	12647.38	14870.04	5106.25	4030.93	6077.96	1.71485292	0.00653816	0.18604545	2.920186735	1.546060627	up	C03056	ko00760,ko01100
MEDP0606	3,4-Dimethoxyphenethylamine	Benzene and substituted derivatives	Benzene and substituted derivatives	C10H15NO2	3	0.7507	120-20-7	8568.53	4444.35	3910.74	10194.85	14905.29	12778.22	1.49642255	0.02551887	0.29249362	0.446788615	-1.16233567	down	-	-

MEDP0156	5-Methyluridine	Nucleotides and derivatives	Nucleotides and derivatives	C10H14N2O6	3	0.7498	1463-10-1	1840.48	4143.43	1645.58	13832.03	29656.26	28302.5	1.68578277	0.04863515	0.35923867	0.106273938	-3.23414025	down	-	-
MW0127606	Epigallocatechin 3,3',-di-O-gallate	Flavonoids	Flavanols	C29H22O15	3	0.7447	89013-66-1	6459.26	5551.09	7339.92	3178.96	179.06	1163.73	1.36619779	0.01427141	0.24261402	4.279376348	2.097400562	up	-	-
MEDP0592	3-Dehydrosphinganine	Lipids	Sphingolipids	C18H37NO2	3	0.7399	16105-69-4	1882.24	2644.73	5145.45	93.12	89.53	295.66	1.68924354	0.08901866	0.45596986	20.22207355	4.337859032	up	C02934	ko00600,ko01100
MEDP1070	Glycyl-L-valine	Amino acids and derivatives	Amino acids and derivatives	C7H14N2O3	3	0.7381	1963-21-9	112827.99	102264.05	33341.32	941.9	28665.89	489.23	1.3669096	0.08577271	0.44629867	8.254417215	3.04516636	up	-	-
MEDP1096	1,2-Dilauroyl-sn-glycero-3-phosphocholine	GL	PC	C32H64NO8P	3	0.7358	18194-25-7 | 127641-86-5	11219.19	19330.6	8075.3	16762.25	45418.49	18029.22	1.17848932	0.27358992	0.65233913	0.481549798	-1.0542431	down	-	-
MW0153971	Mucronine A	Heterocyclic compounds	Heterocyclic compounds	C29H38N4O4	3	0.7351	38840-25-4	181.75	129.03	207.59	1313.91	366.96	505.01	1.47365805	0.1998978	0.59119842	0.237144765	-2.07616007	down	C10009	-
MW0123506	Decoyinine	Heterocyclic compounds	Heterocyclic compounds	C11H13N5O4	3	0.7349	2004-04-8	400.46	235.61	1109.53	2409.8	5479.77	1663.13	1.4718805	0.149783	0.5387718	0.182733677	-2.45218555	down	-	-
MW0000171	Catharanthine	Alkaloids	Plumerane	C21H24N2O2	3	0.7318	2468-21-5	21121.86	27106.12	21615.62	44128.15	54999.43	46465.81	1.71220445	0.00569277	0.17982689	0.479716833	-1.05974503	down	-	-
MW0009985	Trimethoprim	Phenolic acids	Phenolic acids	C14H18N4O3	3	0.7309	738-70-5	47560.95	48594.56	44043.66	103633.08	118911.29	110904.15	1.75564214	0.00242284	0.14452248	0.420452219	-1.24998624	down	C01965	-
MEDN1403	10-Hydroxy-2-decenoic acid	Lipids	Free fatty acids	C10H18O3	3	0.7253	14113-05-4 | 765-01-5	9233.69	9425.72	3383.3	18190.77	11915.71	15894.84	1.33911777	0.04197958	0.33934129	0.479175598	-1.06137366	down	-	-
MW0111931	Gallacetophenone	Phenolic acids	Phenolic acids	C8H8O4	3	0.7226	528-21-2	13034334.35	12342482.71	11400662.9	2363.02	120173.09	3912.87	1.65141591	0.00140597	0.12524365	290.8483719	8.184123418	up	-	-
MW0117980	3-(4-hydroxy-5-oxo-3-phenyl-2H-furan-2-yl)propanoic acid	Organic acids	Organic acids	C13H12O5	3	0.7213	105364-56-5	208.61	1209.74	1350.38	3503.51	2560.83	1216.72	1.15416939	0.13784472	0.52754608	0.380264687	-1.39492413	down	-	-
MW0001224	1-(Diphenylphosphoryl)cyclobutanecarboxylic acid	Benzene and substituted derivatives	Benzene and substituted derivatives	C17H17O3P	3	0.7179	5116-23-4	3032.81	1819.35	2655.87	15921.18	8459.51	4633.6	1.49141711	0.16156952	0.54904711	0.258770075	-1.95025731	down	-	-
MEDP1127	1-Acetylindole	Alkaloids	Plumerane	C10H9NO	3	0.7177	576-15-8	18589.65	18810.09	14684.85	692.89	10492.44	9099.56	1.10362699	0.05706747	0.37920186	2.567654545	1.360451114	up	-	-
MW0015923	Artemisinin	Terpenoids	Sesquiterpenoids	C15H22O5	3	0.717	63968-64-9	281.6	623.24	1161.09	1805.57	3916.9	5387.81	1.50698029	0.09356992	0.46580192	0.185947609	-2.4270319	down	C09538	-
MEDP1884	Prolyl-Histidine	Amino acids and derivatives	Amino acids and derivatives	C11H16N4O3	3	0.7165	92027-43-5	12993.09	17786.43	18364.12	7954.95	7738.99	8406.03	1.67553824	0.03761412	0.32893629	2.039157725	1.027973369	up	-	-
MEDP0519	L-Norleucine	Amino acids and derivatives	Amino acids and derivatives	C6H13NO2	3	0.7159	327-57-1	3145.93	3798.44	2888.55	11206.03	5313.58	5993.07	1.49268575	0.14849262	0.5387718	0.436772521	-1.195046	down	C01933	-
mws1015	Esculin	Lignans and Coumarins	Coumarins	C15H16O9	3	0.7157	531-75-9	2509.63	5240.49	821.5	22645.82	41870.99	1401.32	1.03407756	0.24260098	0.6366467	0.130034332	-2.94303552	down	C09264	-
MW0149118	Fagaramide	Alkaloids	Alkaloids	C14H17NO3	3	0.7139	60045-88-7 | 495-86-3	1012.69	971.22	939.2	5495.6	3246.58	2078.16	1.60513882	0.11957143	0.4975454	0.270149552	-1.88816981	down	-	-
MEDP2182	Beta-Aminopropionitrile	Alcohol and amines	Polyamines	C3H6N2	3	0.713	151-18-8	14817.27	25758.24	12764.01	21260.3	54552.23	35712.74	1.3012669	0.17131096	0.56228372	0.478272951	-1.06409389	down	C05670	ko00410,ko00460
MEDL01937	2,3-Butanediol	Others	Alcohol compounds	C4H10O2	3	0.7122	513-85-9	10244.48	9857.08	7178.74	19855.87	18739.29	21000.57	1.69517424	0.00136706	0.12524365	0.45775595	-1.12734946	down	-	-
MW0153387	Manniflavanone	Others	Ketone compounds	C30H22O13	3	0.7122	73428-17-8	1327.21	432.3	2059.9	7387.7	15748.08	4017.06	1.49344985	0.15317311	0.54188487	0.140663371	-2.8296814	down	C09763	-
MEDP1183	N-Acetylcadaverine	Alkaloids	Alkaloids	C7H16N2O	3	0.7105	32343-73-0	34185.68	32632.6	31172.49	7914.77	7735.39	10719.42	1.74235787	5.6029E-05	0.06238827	3.716053498	1.893771272	up	-	-
MW0049020	Corepoxylone	Lipids	Others	C35H60O5	3	0.7104	154272-51-2	45599.87	37183.16	3706.73	267419.68	300412.72	84184.39	1.41918457	0.10237921	0.48180112	0.13264959	-2.91430788	down	-	-
MW0127431	Di-1-propenyl sulfide	Others	Others	C6H10S	3	0.7093	33922-80-4	334.74	437.77	611.68	4035.71	1988.02	1347.87	1.60015758	0.13150057	0.51786341	0.187773346	-2.4129358	down	-	-
MW0148998	L-Erythrulose 1-phosphate	Organic acids	Organic acids	C4H9O7P	3	0.7089	16409-92-0	1712.25	3474.08	3320.53	30399.94	16691.41	21251.7	1.69925393	0.03628612	0.32815889	0.124472935	-3.00609602	down	C03394	-
MADN0596	Serine alanine	Amino acids and derivatives	Amino acids and derivatives	C6H12N2O4	3	0.7077	6403-17-4	1288.32	2060.24	1621.17	3881.61	5115.48	2155.77	1.43591798	0.130624	0.51623716	0.445601397	-1.16617434	down	-	-
ZINC1520521	2-Oxazolidinethione, 5-ethyl-5-methyl-, (S)-	Others	Ketone compounds	C6H11NOS	3	0.6987	3690-37-7	3498.48	4502.1	1639.97	3011.05	716.19	1071.68	1.06401916	0.21823333	0.61055439	2.008899919	1.006405693	up	-	-
MW0160835	[2-(6-Amino-9H-purin-9-YL)-1-methylethoxy]methyl-triphosphate	Organic acids	Organic acids	C9H16N5O10P3	3	0.6972	166403-66-3	2124.62	2130.67	1839.24	444.18	395.62	1368.65	1.47346119	0.04498336	0.34946131	2.759641377	1.464480797	up	-	-
MW0129933	1-O-trans-Cinnamoyl-beta-D-glucopyranose	Phenolic acids	Phenolic acids	C15H18O7	3	0.6962	40004-96-4	109711.08	179839.42	123666.24	583138.02	349137.39	448026.6	1.6689592	0.03204366	0.31330981	0.299366904	-1.74001336	down	C04164	-
MW0138703	Kavain	Others	Lactones	C14H14O3	3	0.6953	500-64-1	83526.74	67202.64	59016.01	194549.58	224697.25	251797.94	1.72196356	0.00478987	0.16667262	0.312565419	-1.67776992	down	C09947	-
MEDP0281	1,3-Diaminopropane	Alcohol and amines	Polyamines	C3H10N2	3	0.6938	109-76-2	2284.5	1024.36	1170.64	7572.67	54071.07	11208.81	1.57003918	0.26638161	0.6495827	0.061487209	-4.02356986	down	C00986	ko00260,ko00330,ko00410,ko01100
MW0052505	Ecdysone	Organic acids	Organic acids	C27H44O6	3	0.6933	3604-87-3	4224.42	4641.13	4512.16	532.77	2962.78	2312.23	1.22844671	0.06992732	0.40873525	2.303411975	1.203772466	up	C00477	-
MW0006987	Epsilon-pyrromycinone	Benzene and substituted derivatives	Benzene and substituted derivatives	C22H20O9	3	0.6892	21288-61-9	3514.94	3927.42	3902.87	2128.25	476.23	1011.62	1.48631747	0.02693056	0.29249362	3.137421531	1.649579377	up	-	-
MW0014680	(3R,5R,7R,8R,9S,10S,12S,13R,14S,17R)-17-[(2S)-4,6-Dihydroxy-6-methylheptan-2-yl]-10,13-dimethyl-2,3,4,5,6,7,8,9,11,12,14,15,16,17-tetradecahydro-1H-cyclopenta[a]phenanthrene-3,7,12-triol	Alcohol and amines	Alcohols	C27H48O5	3	0.6891	137252-15-4	40046.46	67204.46	36663.38	21604.53	4620.06	16377.15	1.4179992	0.05313034	0.37242434	3.378131973	1.756225691	up	-	-
MEDN1096	N-Benzoyl-L-tyrosine ethyl ester	Amino acids and derivatives	Amino acids and derivatives	C18H19NO4	3	0.6885	3483-82-7	28724.46	64201.78	45587.5	7549.62	30020.45	30665.13	1.08167726	0.14618385	0.53868111	2.029945541	1.021441024	up	-	-
MW0124447	Isatin	Alkaloids	Plumerane	C8H5NO2	3	0.6815	91-56-5	212.56	461.46	494.03	1595.34	642.86	3862.72	1.36165845	0.22646256	0.62075385	0.191454731	-2.38492478	down	C11129	-
MEDL02383	Ethyl gallate	Phenolic acids	Phenolic acids	C9H10O5	3	0.6813	831-61-8	6.22	6.03	22.05	33.3	20.72	17.43	1.18923353	0.16033393	0.54886804	0.480055983	-1.05872543	down	-	-

MEDP1605	Glu-glu	Amino acids and derivatives	Amino acids and derivatives	C10H16N2O7	3	0.6807	3929-61-1	61922.68	61884.31	43246.22	1144.17	6075.25	6857.27	1.60989385	0.00996467	0.21235708	11.86736442	3.568927662	up	C01425	-
MW0158075	Triethylenephosphoramide	Heterocyclic compounds	Heterocyclic compounds	C6H12N3OP	3	0.6803	545-55-1 | 27030-72-4	319.33	1139.03	199.63	2149.67	366.47	4074.74	1.02137798	0.26105661	0.64722458	0.251558214	-1.9910358	down	C19543	-
MW0055375	Nostoxanthin	Lipids	Others	C40H56O4	3	0.6801	29307-44-6	37755.73	18967.54	14602.07	32549.01	148970.54	85979.43	1.34852612	0.18709013	0.57969291	0.266637802	-1.90704677	down	-	-
MW0113021	4-Aminophenyl b-D-thiogalactopyranoside	Benzene and substituted derivatives	Benzene and substituted derivatives	C12H17NO5S	3	0.6773	29558-05-2	2079.96	1218.24	2097.73	23146.85	1871.04	6456.9	1.13703372	0.31084239	0.68402525	0.171436569	-2.54425321	down	-	-
MEDP1152	2-Methoxy-4-[illegible]	Phenolic acids	Phenolic acids	C9H10O2	3	0.6773	7786-61-0	27657.19	28347.98	20080.59	69040.02	49621.85	62822.9	1.67040352	0.01345725	0.23505329	0.419240468	-1.25415011	down	C17883	-
MEDL00352	Glu-Val	Amino acids and derivatives	Amino acids and derivatives	C10H18N2O5	3	0.6752	5879-06-1	1444.76	5764.93	20745.81	89050.6	21511.36	18427.71	1.21424118	0.27891265	0.65903285	0.216726657	-2.20605148	down	-	-
MW0123319	Chromone	Others	Ketone compounds	C9H6O2	3	0.6737	491-38-3	2377.08	3956.29	624.31	21642.57	12616.05	17301.68	1.58386773	0.01935807	0.2702848	0.134942582	-2.88958243	down	-	-
MW0004101	3-Methoxybenzyl alcohol	Benzene and substituted derivatives	Benzene and substituted derivatives	C8H10O2	3	0.6724	6971-51-3	522094.57	478498.87	709535.62	24195.88	16360.26	16896.05	1.75656105	0.01605458	0.25019321	29.76612484	4.895599509	up	C03352	-
MEDP0068	N-Acetyl-Neuraminic Acid	Amino acids and derivatives	Amino acids and derivatives	C11H19NO9	3	0.6723	131-48-6 | 489-46-3	78942.99	113427.08	84888.54	206128.39	187233.1	189737.53	1.69828616	0.00304092	0.14883777	0.47549147	-1.07250864	down	C00270	ko00520,ko01100,ko01250
MEDN0622	Porphobilinogen	Organic acids	Organic acids	C10H14N2O4	3	0.672	487-90-1	386.02	664.16	1032.75	229.63	285.6	184.99	1.50385383	0.12980875	0.51392725	2.974679386	1.572734182	up	C00931	ko00860,ko01100,ko01110,ko01240
MW0106942	Glutamylasparagine	Amino acids and derivatives	Amino acids and derivatives	C9H15N3O6	3	0.6715	36314-37-1	6099.74	10184.24	11019.29	22014.86	14015.63	22381.6	1.4854556	0.04233231	0.34157265	0.46742498	-1.09719326	down	-	-
MW0105135	1-propyl-2-sulfanyl-4,5-dihydro-1H-imidazol-5-[illegible]	Organic acids	Organic acids	C6H10N2OS	3	0.6706	65710-56-7	1140.75	7154.59	1743.12	24087.25	7404.33	9693.23	1.37899631	0.17567933	0.56921854	0.243741807	-2.03657437	down	-	-
MEDL00369	Glutamyl-phenylalanine	Amino acids and derivatives	Amino acids and derivatives	C14H18N2O5	3	0.6703	20556-22-3	5420.3	13786.81	19709.42	10178.43	1617.93	3315.73	1.14155517	0.19378051	0.58554843	2.575191784	1.364679879	up	-	-
MW0011930	1,2-Dimyristoyl-sn-glycero-3-[illegible]	GL	PC	C36H72NO8P	3	0.6666	18194-24-6	329.2	1574.85	267.14	7784.18	7945.01	16237.6	1.64389866	0.06756481	0.40452854	0.067920176	-3.88001599	down	-	-
MW0007219	Frangulin A	Benzene and substituted derivatives	Benzene and substituted derivatives	C21H20O9	3	0.6656	521-62-0	15218.49	17697.8	16141.94	5241.85	3363.54	5430.47	1.71399073	0.00029728	0.08153915	3.495206564	1.80537772	up	-	-
MW0054008	Ethyl icosapentate	Lipids	Free fatty acids	C22H34O2	3	0.6644	86227-47-6	8444.88	3362.93	5150.98	569737.18	32316.15	25774.95	1.38853426	0.375857	0.72803247	0.027011829	-5.21026487	down	C16184	-
MW0143681	Mycaminosyltylonolide	Heterocyclic compounds	Heterocyclic compounds	C31H51NO10	3	0.6643	61257-02-1	13342.62	16104.85	16735.19	8900.63	4271.03	3730.68	1.56547743	0.0112979	0.22167778	2.732323453	1.45012828	up	-	-
MW0049160	Dethiobiotin	Organic acids	Organic acids	C10H18N2O3	3	0.6634	533-48-2	34578.26	68346.74	40404.05	111037.42	68790.79	113436.69	1.44165094	0.05455528	0.37690654	0.488735781	-1.03287336	down	C01909	ko00780,ko01100,ko01240
MEDP0215	Quinine	Alkaloids	Quinoline [illegible]	C20H24N2O2	3	0.6612	130-95-0	301058.46	322300.8	290774.44	679049.15	778195.54	704549.32	1.75633429	0.0026783	0.14452248	0.422858837	-1.24175197	down	C06526	-
MEDP1617	L-Gulose	Others	Saccharides	C6H12O6	3	0.6599	6027-89-0	7.87	134.98	19.69	323.8	504.86	287.79	1.4964483	0.0227284	0.27962521	0.145586457	-2.78005194	down	C15923	ko00053
MEDL01976	Phytic acid	Organic acids	Organic acids	C6H18O24P6	3	0.6598	83-86-3	1016.09	488.38	182.39	1454	529.8	1570.12	1.01384262	0.20904801	0.60095435	0.474647713	-1.07507096	down	C01204	ko00562,ko01100,ko04070
MW0123384	Clerodin	Heterocyclic compounds	Heterocyclic compounds	C24H34O7	3	0.6585	464-71-1	17208.82	17691.07	23523.25	633.81	7654.63	20017.21	1.00673675	0.21153607	0.60186131	2.064009835	1.045449846	up	C09075	-
MW0103666	Telbivudine	Nucleotides and derivatives	Nucleotides and derivatives	C10H14N2O5	3	0.6512	3424-98-4	7187.53	5490.7	7209.16	4795.22	3683.87	1008.59	1.19926091	0.07161701	0.41116592	2.09612782	1.067726694	up	-	-
MW0001942	2-(4-Methoxyphenoxy)acetohydrazide	Phenolic acids	Phenolic acids	C9H12N2O3	3	0.6496	21953-91-3	3045.08	1196.28	1663.5	166.64	135.16	617.83	1.56901811	0.08632714	0.44865937	6.420908409	2.682777419	up	-	-
MW0128402	(S,S)-(-)-Hydrobenzoin	Benzene and substituted derivatives	Benzene and substituted derivatives	C14H14O2	3	0.6473	2325-10-2 | 655-48-1	315500.85	498687.64	452196.4	120806.94	108141.95	360087.64	1.32035235	0.09359962	0.46580192	2.149925897	1.104286934	up	-	-
MW0008211	N-(3-hydroxyphenyl)-Arachidonoyl amide	Benzene and substituted derivatives	Benzene and substituted derivatives	C26H37NO2	3	0.6465	183718-75-4	20497.36	17242.28	10128.39	51272.31	37175.6	37148.58	1.59783604	0.01410645	0.24165426	0.381125539	-1.39166181	down	-	-
MW0153988	Muscapurpurin	Heterocyclic compounds	Heterocyclic compounds	C18H14N2O10	3	0.643	12624-19-0	275432.67	296578.94	305491.74	61250.96	24006.68	27637.22	1.70288342	0.00011253	0.07160277	7.772748467	2.95842483	up	C08563	-
MEDP1390	Carnitine C18:1(Acylcarnitine C18:1)	Lipids	CAR	C25H47NO4	3	0.6414	38677-66-6	2036.29	2489.22	5758.83	4572.15	4480.59	24075.01	1.01209555	0.3628684	0.71609031	0.310444869	-1.68759101	down	-	-
MEDP2077	1-Phenylbiguanide	Benzene and substituted derivatives	Benzene and substituted derivatives	C8H11N5	3	0.6407	102-02-3	1053.96	762.91	532.21	2392.1	2020.89	1305.79	1.52484773	0.05347981	0.37335277	0.410765933	-1.28361156	down	-	-
MW0159463	Zizyphine A	Heterocyclic compounds	Heterocyclic compounds	C33H49N5O6	3	0.6385	51059-42-8	66.24	201.03	263.48	287.54	854.59	400.75	1.28991998	0.18255635	0.57250504	0.343999533	-1.53952149	down	-	-
MW0139539	Retusin	Flavonoids	Flavones	C19H18O7	3	0.6356	1245-15-4	4840.81	5216.96	9050.69	3660.98	935.88	1082.27	1.46320057	0.05869089	0.3844253	3.364680858	1.750469671	up	-	-
MW0143899	6-tuliposide B	Others	Lactones	C11H18O9	3	0.6354	244105-18-8	622.27	3933.51	3814.69	5033.67	6775.57	11362.41	1.21972375	0.10298068	0.48180112	0.361237547	-1.46898024	down	C21186	-
MW0103020	Ubiquinol-6	Heterocyclic compounds	Heterocyclic compounds	C39H60O4	3	0.6329	5677-58-7	3301.93	2909.84	10700.95	2890.58	1120.33	2297.09	1.19529146	0.29624515	0.67792725	2.68115409	1.422854136	up	-	-
MW0116583	1,2,5-Trimethylpyrrole	Heterocyclic compounds	Heterocyclic compounds	C7H11N	3	0.6322	930-87-0	1446.41	928.84	1794.03	3455.07	4482.65	3270.28	1.60767281	0.00956602	0.20897133	0.371991435	-1.42665869	down	-	-
MW0153878	Met-Thr	Amino acids and derivatives	Amino acids and derivatives	C9H18N2O4S	3	0.6285	40883-16-7	259087.29	222846.24	215548.63	75859.99	127626.7	52530.96	1.56340692	0.00838199	0.20100334	2.724351856	1.445913043	up	-	-
MW0151985	Kaempferol 3-O-beta-D-glucosylgalactoside	Flavonoids	Flavonols	C27H30O16	3	0.6285	31512-06-8	11682.71	11771.69	9628.03	2557.64	2587.55	4148.27	1.70450319	0.00116005	0.12524365	3.559753848	1.831777484	up	-	-
ZINC8548712	Agrimol F	Phenolic acids	Phenolic acids	C34H40O12	3	0.6228	121693-16-1	61881.77	45694.53	32807.92	381.15	1319.99	1880.51	1.69888479	0.03208113	0.31330981	39.19540435	5.292612604	up	-	-
MEDP1796	Perillyl alcohol	Terpenoids	Monoterpenoids	C10H16O	3	0.6227	536-59-4	312.02	2131.17	310.77	8963.72	1125.97	3114.71	1.23113536	0.27412735	0.65327085	0.208563812	-2.26143924	down	C02452	ko00902,ko01110

MW0138164	Fisetinidol	Flavonoids	Flavanonols	C15H14O5	3	0.6203	490-49-3	31868.99	37608.87	37123.78	5882.33	17358.16	4904.62	1.53634432	0.01144038	0.22207747	3.787572335	1.921273441	up	C09735	-
MW0115372	Tolmetin glucuronide	Heterocyclic compounds	Heterocyclic compounds	C21H23NO9	3	0.6153	71595-19-2	246313.27	182550.8	239540.77	21334.87	31368.49	23597.94	1.74614689	0.00912828	0.2053328	8.760071454	3.130942638	up	-	-
MW0110392	Valylcysteine	Amino acids and derivatives	Amino acids and derivatives	C8H16N2O3S	3	0.6148	160070-15-5	7409.77	8707.01	8476.76	37966.67	23615.89	30684.33	1.71504605	0.03111697	0.31144938	0.266547837	-1.90753362	down	-	-
MW0119700	3-Methyl-4-nitro-1-(4-nitrophenyl)-1H-pyrazol-5-ol	Benzene and substituted derivatives	Benzene and substituted derivatives	C10H8N4O5	3	0.6144	132-42-3	980.84	1365.61	1888.67	13118.06	5298.58	1671.89	1.26342734	0.25767649	0.64577513	0.210822793	-2.24589724	down	-	-
MEDL00424	PAz-PC	GL	PC	C33H64NO10P	3	0.6143	117205-52-4	1451.25	1704.25	2148.48	5462.43	5661.87	6921.05	1.71456427	0.00463062	0.16605573	0.293925028	-1.76647988	down	-	-
MW0106553	Pantethine	Alcohol and	Polyamines	C22H42N4O8S2	3	0.6127	16816-67-4	836.93	39.33	1012.13	4056.42	1869.62	912	1.04891441	0.21185752	0.6021783	0.276159543	-1.85642611	down	-	-
MW0054028	Ipecoside	Others	Lactones	C27H35NO12	3	0.6107	15401-60-2	90601.85	110141.63	185768.38	62.95	12367.27	8592.47	1.35544708	0.05031269	0.36472395	18.38546161	4.200493494	up	C09464	-
MEDP2037	3-Phenylpropionylglycine	Amino acids and derivatives	Amino acids and derivatives	C11H13NO3	3	0.6076	20989-69-9	40102131.33	40736739.07	57752465.39	9810481.54	23615466.09	33092317.29	1.30936405	0.05542307	0.37695993	2.08350798	1.059014626	up	-	-
MEDP0652	Methyl cinnamate	Benzene and substituted derivatives	Benzene and substituted derivatives	C10H10O2	3	0.603	103-26-4 | 1754-62-7	4434.56	2809.77	3649.75	75.94	1639.36	2044.26	1.10444057	0.03829792	0.32974696	2.897700795	1.534908636	up	C06358	-
MW0053877	Herculin	Others	Ketone compounds	C16H29NO	3	0.6017	72362-03-9	5004.73	1254.09	7425.68	9524.29	7494.62	10851.36	1.09608114	0.1011704	0.48076303	0.491007084	-1.02618426	down	-	-
MEDTP02544	5-(6-Hydroxy-2,5,7,8-tetramethyl-chroman-2-YL)-2-methyl-pentanoic acid	Organic acids	Organic acids	C19H28O4	3	0.6006	7083-09-2	2992.97	547.53	2431	379.8	378.57	691	1.32998354	0.17613876	0.56921854	4.12006596	2.042667434	up	-	-
MW0150339	Gly-Gln-Gln	Amino acids and derivatives	Amino acids and derivatives	C12H21N5O6	3	0.6006	627882-94-4	649.16	342.92	717.76	25207.81	1915.31	4931.29	1.45899892	0.30080532	0.67873528	0.053341802	-4.22858962	down	-	-
MW0157291	Sulfazecin	Amino acids and derivatives	Amino acids and derivatives	C12H20N4O9S	3	0.5979	77912-79-9	22448.34	24421.14	17835.01	2004.92	1297.05	776.7	1.72062054	0.00761517	0.19469501	15.86411502	3.987695137	up	C20927	ko00261
MW0126336	Pyridoxamine phosphate	Organic acids	Organic acids	C8H13N2O5P	3	0.5886	529-96-4	250066.99	303915.01	235035.72	13534.69	8496.32	1789.77	1.66691277	0.00562612	0.17971833	33.12308497	5.049765142	up	C00647	ko00750,ko01100
MW0105412	H-Lys-Tyr-Lys-OH Acetate salt	Benzene and substituted derivatives	Benzene and substituted derivatives	C23H39N5O7	3	0.5866	35193-18-1	1743.19	908.69	1571.46	23.29	18.21	13.6	1.7533504	0.03192016	0.31330981	76.64863884	6.260188267	up	-	-
MW0117846	1,2,3,4-Tetrahydro-2,3-dioxoquinoxaline-6-carboxylic acid	Organic acids	Organic acids	C9H6N2O4	3	0.5834	14121-55-2	4699.88	5228.98	289.61	11510.75	13410.57	9203.38	1.22768632	0.01784776	0.26056177	0.299444977	-1.73963716	down	-	-
MEDL02754	2,6-Dimethoxy-1,4-benzoquinone	Quinones	Quinones	C8H8O4	3	0.5827	530-55-2	26508.61	25518.01	21809.48	17609.02	703.67	227.7	1.29983897	0.07637569	0.42295042	3.982445892	1.993654761	up	C10331	-
MW0108432	N-(3-Oxohexanoyl)homoserine lactone	Others	Lactones	C10H15NO4	3	0.5813	76924-95-3 | 143537-62-6	4.08	96.17	163.81	963.1	430.63	552.26	1.33626885	0.06388882	0.39280702	0.135694428	-2.88156661	down	-	-
MW0001326	2-(4-Iodo-2,5-dimethoxyphenyl)-N-((2,3-methylenedioxyphenyl)methyl)ethanamine	Benzene and substituted derivatives	Benzene and substituted derivatives	C18H20INO4	3	0.5785	919797-25-4	2272.86	4013.06	4224.63	1287.79	465.27	976.95	1.57241316	0.03863289	0.32974696	3.850004212	1.944860024	up	-	-
MW0012351	13-Tetradecynoic acid	Lipids	Free fatty acids	C14H24O2	3	0.5781	82909-47-5	2199.35	825.63	2751.76	439.67	189.3	853.21	1.41132434	0.11800556	0.4975454	3.897461847	1.9625349	up	-	-
MW0123976	Ethyl 4-(1-hydroxy-1-methylethyl)-2-propyl-imidazole-5-carboxylate	Others	Lactones	C12H20N2O3	3	0.5772	144689-93-0	8900.79	8475.44	7497.66	3741.16	3303.26	2228.41	1.66661273	0.00108061	0.12524365	2.682448616	1.423550536	up	-	-
MEDP1249	2-Oxo-4-phenylbutyric acid	Benzene and substituted derivatives	Phenolic acids	C10H10O3	3	0.5763	710-11-2	206255.59	121351.66	242024.51	10232.11	3717.83	4449.56	1.7314552	0.03551957	0.32619419	30.95908911	4.952291119	up	C20327	ko00400,ko01100,ko01210
MW0148433	Dibenzyl succinate	Benzene and substituted derivatives	Benzene and substituted derivatives	C18H18O4	3	0.5701	103-43-5	120010.78	117583.82	192151.04	311.43	668.69	1974.07	1.72766741	0.02827066	0.29980363	145.4698716	7.184576576	up	C07332	-
MW0017134	cis-11-Methyl-2-dodecenoic acid	Lipids	Free fatty acids	C13H24O2	3	0.5684	677354-23-3	3180.34	1382.83	1441.38	8513.86	11850.67	10969.2	1.66665355	0.00408653	0.15827323	0.191632148	-2.38358848	down	C18206	-
MW0114989	Neohesperidose heptaacetate	Others	Saccharides	C26H36O17	3	0.5645	19949-47-4	8674.17	7683.7	4529.14	95.01	78.72	172.26	1.74292925	0.03167949	0.31251818	60.36882569	5.915731833	up	-	-
MW0008605	N-[(4-Hydroxy-3-methoxyphenyl)methyl] octanamide	Phenolic acids	Phenolic acids	C16H25NO3	3	0.5557	58493-47-3	355.55	205.93	386.53	0.37	1.08	0.53	1.74948374	0.02986942	0.30510706	478.7929293	8.903258037	up	-	-
MW0110369	L-Arginine, L-valyl-L-threonyl-	Amino acids and derivatives	Amino acids and derivatives	C15H30N6O5	3	0.5533	362603-27-8	53.82	76.5	138.63	496.68	489.95	2587.13	1.54183025	0.25512583	0.64417825	0.075256872	-3.73203286	down	-	-
MW0109543	S-(2,4-dinitrophenyl)glutathion	Amino acids and derivatives	Amino acids and derivatives	C16H19N5O10S	3	0.5531	26289-39-4	1767.68	1467.98	1102.39	994.67	221.74	705.26	1.27851999	0.05453538	0.37690654	2.257437541	1.174686072	up	C11175	-
MEDL02650	Cyclomorusin	Flavonoids	Other Flavonoids	C25H22O6	3	0.5499	62596-34-3	135.77	182.43	361.26	210.32	665.08	568.01	1.08663454	0.19980338	0.59119842	0.470732502	-1.08702063	down	C17867	-
MW0014209	3-O-a-L-Fucopyranosyl-D-glucose	Organic acids	Organic acids	C12H22O10	3	0.5495	56822-52-7	13555.03	8253.37	10533.54	31276.14	13733.26	29885.75	1.37109754	0.11943869	0.4975454	0.431829564	-1.21146608	down	-	-
INC10000712	Dimethylcurcumin	Phenolic acids	Phenolic acids	C23H24O6	3	0.5456	52328-98-0 | 917813-54-8 | 115851-85-9	5492	1751.05	7211.86	897.61	1592.47	4018.74	1.02532074	0.24525287	0.63712686	2.220818827	1.151091704	up	-	-
MW0110031	Triiodothyronine glucuronide	Amino acids and derivatives	Amino acids and derivatives	C21H20I3NO10	3	0.5426	29919-72-0	2680.82	3234.62	2583.02	1511.41	1311.1	1271.57	1.70318871	0.01101084	0.21796572	2.075792364	1.053662142	up	-	-
MEDP0317	Hydrocinnamic acid	Phenolic acids	Phenolic acids	C9H10O2	3	0.5401	501-52-0	19219.93	17055.23	3126.98	17425.09	35648.18	28666.22	1.10016222	0.12635683	0.50962303	0.482045337	-1.05275925	down	C05629	ko00360,ko01100
MW0146861	Brevetoxin A	Heterocyclic compounds	Heterocyclic compounds	C49H70O13	3	0.5323	98112-41-5	24474.2	20632.99	21521.54	6105.84	4210.43	2117.81	1.65582565	0.00038198	0.09451911	5.358557288	2.421844629	up	-	-

MEDL01773	2,4,5-Trimethoxybenzaldehyde	Phenolic acids	Phenolic acids	C10H12O4	3	0.5266	4460-86-0	2994.51	10602.51	5289.39	22507.51	10431.79	16972.89	1.37917907	0.0781494	0.42764652	0.378392733	-1.40204371	down	-	-
MEDL02176	6-Methylcoumarin	Lignans and Coumarins	Coumarins	C10H8O2	3	0.5216	92-48-8	6176.95	7265.07	1919.1	20024.67	13641.66	15695.14	1.48478732	0.01089712	0.21667748	0.311196567	-1.68410195	down	-	-
MW0002167	2,3-Dimethoxyphenol	Phenolic acids	Phenolic acids	C8H10O3	3	0.5162	5150-42-5 | 25155-26-4	5587.24	7455.03	6125.09	23925.93	12831.29	27046.97	1.61440272	0.07244502	0.41345377	0.300409111	-1.73499953	down	-	-
MW0129464	Angoletin	Others	Ketone compounds	C18H20O4	3	0.5116	76444-55-8	249454.58	202121.08	227234.31	82945.43	134728.53	120211.87	1.60990792	0.00550398	0.17971833	2.00899212	1.006471905	up	-	-
MW0161700	2-Hydroxyprop-2-enal	Others	Aldehyde compounds	C3H4O2	3	0.5068	73623-80-0	410.73	482.57	919.78	3739.91	750.78	1423.27	1.20524103	0.26841511	0.65025613	0.306576304	-1.7056819	down	-	-
MW0146629	beta-Alanine amide	Amino acids and derivatives	Amino acids and derivatives	C3H8N2O	3	0.5066	4726-85-6	2292.7	456.79	781.31	1726.75	4253.27	3570.51	1.26419305	0.10588124	0.48180112	0.36969676	-1.43558569	down	C19779	-
MW0160847	2-Hydroxy-3-(phosphonooxy)propyl octadec-9-enoate	GL	PA	C21H41O7P	3	0.5016	22002-79-5	13076.59	16088.64	18540.19	53486.91	29568.34	15742.05	1.13989723	0.26109407	0.64722458	0.482861576	-1.05031843	down	-	-
MW0146229	Asp-Phe-Trp	Amino acids and derivatives	Amino acids and derivatives	C24H26N4O6	2	0.9055	-	12733.49	6845.2	9331.68	34758.39	28785.09	11587.06	1.33218318	0.15023677	0.5387718	0.38480184	-1.3778124	down	-	-
MW0155339	Phe-Ser-Trp	Amino acids and derivatives	Amino acids and derivatives	C23H26N4O5	2	0.8997	-	1015.88	1009.18	343.38	3176.93	4545.08	2352.12	1.57662853	0.04452176	0.34946131	0.235101195	-2.08864622	down	-	-
MW0149720	Gln-Ile-Leu	Amino acids and derivatives	Amino acids and derivatives	C17H32N4O5	2	0.8818	-	2345.37	8763.75	18017.68	24010.36	9139.24	38811.92	1.00261623	0.23607597	0.63039188	0.404755208	-1.30487845	down	-	-
MW0146032	Asp-Arg-Tyr	Amino acids and derivatives	Amino acids and derivatives	C19H28N6O7	2	0.8675	-	7637.05	15751.67	12888.76	851.53	892.58	2383.97	1.66226199	0.04071136	0.33610993	8.787978915	3.135531408	up	-	-
MW0153005	Lys-Ile-Asp-Asn-Glu	Amino acids and derivatives	Amino acids and derivatives	C25H43N7O11	2	0.8631	-	2824.45	9410.06	8297.32	31870.56	21193.04	8319.07	1.25145959	0.17588271	0.56921854	0.334489034	-1.57996918	down	-	-
MW0152308	Leu-Glu-Arg-Met-Phe	Amino acids and derivatives	Amino acids and derivatives	C31H50N8O8S	2	0.8581	-	101384.99	4136.6	22561.17	5050.43	2822.2	1681.61	1.28284562	0.31630188	0.68542982	13.40585541	3.744791374	up	-	-
MW0158529	Tyr-Leu-Thr-Arg	Amino acids and derivatives	Amino acids and derivatives	C25H41N7O7	2	0.8517	-	1356.68	1305	972.72	4891.48	3141.02	1165.02	1.19170683	0.22596131	0.62010578	0.395149997	-1.3395277	down	-	-
MW0153088	Lys-Phe-Arg-Lys	Amino acids and derivatives	Amino acids and derivatives	C27H47N9O5	2	0.8372	-	7474.69	6402.52	4518.3	20186.97	18023.54	24579.98	1.69653936	0.00770644	0.19498432	0.292966499	-1.7711924	down	-	-
MW0138534	Dactilin	Flavonoids	Flavones	C28H32O17	2	0.8335	-	2560.65	2248.42	2087.55	1596.88	338.34	1381.05	1.22479143	0.07814734	0.42764652	2.079631634	1.056328006	up	-	-
MW0151609	Ile-Thr-Val-Lys-Leu	Amino acids and derivatives	Amino acids and derivatives	C27H52N6O7	2	0.8305	-	19292.57	12055.96	16704.59	141.01	343.64	211.25	1.74502013	0.01745236	0.25570009	69.05176031	6.109606288	up	-	-
MW0008784	N(4)-Acetylsulfamethazine	Benzene and substituted derivatives	Benzene and substituted derivatives	C14H16N4O3S	2	0.828	-	590258.01	568776.03	167382.27	16026.91	7197.43	13143.37	1.69554041	0.08879873	0.45596986	36.47236271	5.188731757	up	-	-
MW0145662	Asn-Ala-Leu-Ala-His	Amino acids and derivatives	Amino acids and derivatives	C22H36N8O7	2	0.8276	-	199757.21	198709.82	197097.3	959748.15	422891.14	446702.68	1.5671174	0.14333233	0.53323181	0.325562054	-1.61899554	down	-	-
MW0150294	Gly-Arg-Ala	Amino acids and derivatives	Amino acids and derivatives	C11H22N6O4	2	0.8261	-	135023.95	73440.7	110856.2	278.66	55.33	52.52	1.74196775	0.02723774	0.29249362	826.1645236	9.6902853	up	-	-
MW0155997	Pro-Ser-Val	Amino acids and derivatives	Amino acids and derivatives	C13H23N3O5	2	0.8088	-	1739637.48	971501.79	1466276.89	387419.95	104927.46	1025859.97	1.27773718	0.06829939	0.40452854	2.751545155	1.460242004	up	-	-
MW0156054	Pro-Val-Ser	Amino acids and derivatives	Amino acids and derivatives	C13H23N3O5	2	0.8037	-	24733.46	20117.75	22089.57	7512.56	6125.31	4.64	1.03136495	0.00556748	0.17971833	4.906778885	2.29477626	up	-	-
MW0156748	Ser-Glu-Val-Lys-Met	Amino acids and derivatives	Amino acids and derivatives	C24H44N6O9S	2	0.801	-	10968.72	17608.7	13791.77	29774.1	18952.21	36688.7	1.44099261	0.09475781	0.46738791	0.496039162	-1.01147407	down	-	-
MW0156902	Ser-Pro-Val	Amino acids and derivatives	Amino acids and derivatives	C13H23N3O5	2	0.8003	-	415505.83	216448.59	367735.94	57500.09	401.62	66648.74	1.2650236	0.02918695	0.30170986	8.026388985	3.004751076	up	-	-
MW0144707	Ala-Pro-Arg-Lys-Lys	Amino acids and derivatives	Amino acids and derivatives	C26H50N10O6	2	0.7893	-	47577.23	94146.43	52035.33	90059.39	250120.79	77681.33	1.13727658	0.30938309	0.68402525	0.463691882	-1.10876163	down	-	-
MW0144772	Ala-Tyr-Gln	Amino acids and derivatives	Amino acids and derivatives	C17H24N4O6	2	0.7805	-	18904.64	19807.68	27186.47	15878.01	9170.33	4006.9	1.32396622	0.05065842	0.36569306	2.268051821	1.181453604	up	-	-
MW0156866	Ser-Phe-Ala	Amino acids and derivatives	Amino acids and derivatives	C15H21N3O5	2	0.7784	-	44533.21	25618.61	27553.59	99.17	1237.09	209.48	1.66518121	0.0329851	0.31392227	63.20947249	5.98206887	up	-	-
MW0146023	Asp-Arg-His	Amino acids and derivatives	Amino acids and derivatives	C16H26N8O6	2	0.7783	-	20418.92	21539.57	26287.84	86700.96	49106.92	42233	1.56559399	0.11555671	0.49367245	0.383318314	-1.38338517	down	-	-
MW0107254	His-Glu-Arg	Amino acids and derivatives	Amino acids and derivatives	C17H28N8O6	2	0.7765	-	45.33	58.65	44.9	46.87	115.94	193.03	1.15133545	0.24292155	0.6366467	0.418390288	-1.25707873	down	-	-
MW0114997	N-ethyl-N-Methylcathinone	Others	Ketone compounds	C12H17NO	2	0.775	-	6733.26	2548.38	3610.24	17678.1	9267.72	9356.21	1.44885474	0.09047052	0.45758514	0.35512835	-1.49358756	down	-	-
MW0005268	4-Nitroaniline	Benzene and substituted derivatives	Benzene and substituted derivatives	C6H6N2O2	2	0.7737	-	1573.3	2477.05	646.47	682.28	761.08	122.56	1.11265452	0.17740634	0.56946912	2.999399714	1.584673795	up	C02126	-
MW0159058	Val-Leu-Asp-Phe-Glu	Amino acids and derivatives	Amino acids and derivatives	C29H43N5O10	2	0.7708	-	726.35	89.03	49.66	8.46	62.55	19.53	1.20806453	0.3601356	0.71458744	9.554230175	3.256139634	up	-	-
MW0158995	Val-Glu-Phe-Asp	Amino acids and derivatives	Amino acids and derivatives	C23H32N4O9	2	0.7705	-	5268.75	2796	3169.35	12115.2	18030.88	4389.09	1.30989364	0.18429088	0.57441663	0.325294475	-1.62018178	down	-	-
MW0149994	Glu-Glu-Leu-Ile-Thr	Amino acids and derivatives	Amino acids and derivatives	C26H45N5O11	2	0.7687	-	3852.12	4499.63	110.92	11762.52	7504.78	4659.88	1.06360482	0.1163081	0.49429284	0.353684387	-1.49946556	down	-	-
MW0152386	Leu-Ile-Tyr	Amino acids and derivatives	Amino acids and derivatives	C21H33N3O5	2	0.7629	-	449379.25	404215.16	553886.58	9960.59	3230.11	3589.88	1.74270654	0.00889159	0.2053328	83.87558654	6.390179045	up	-	-

MW0149854	Gln-Val-Gln	Amino acids and derivatives	Amino acids and derivatives	C15H27N5O6	2	0.7623	-	9665.61	5645.83	7441.25	11.76	7.1	15.62	1.75979202	0.02276787	0.27962521	659.8808005	9.366061632	up	-	-
MW0153121	Lys-Pro-Tyr	Amino acids and derivatives	Amino acids and derivatives	C20H30N4O5	2	0.7608	-	14747.67	8557.37	2061.02	24859.19	23110.81	20536.98	1.309925	0.04709913	0.35555849	0.370269716	-1.43335154	down	-	-
MW0158550	Tyr-Lys-Val-Glu-Ile	Amino acids and derivatives	Amino acids and derivatives	C31H50N6O9	2	0.7602	-	35222.78	17489.12	13720.99	32391.33	65189.14	53254.73	1.37291116	0.08119831	0.43468421	0.440433599	-1.18300356	down	-	-
MW0158670	Tyr-Val-Lys-Ala-Leu	Amino acids and derivatives	Amino acids and derivatives	C29H48N6O7	2	0.7601	-	2973.68	2082.94	1240.14	267.94	423.44	1649.57	1.26994332	0.11945167	0.4975454	2.689831051	1.42751556	up	-	-
MW0159193	Val-Thr-Tyr-Lys	Amino acids and derivatives	Amino acids and derivatives	C24H39N5O7	2	0.76	-	646.8	136.2	260.23	1279.84	478.99	529.3	1.07509639	0.25538273	0.64420978	0.455931263	-1.13311176	down	-	-
MW0107729	Leu-Ile-Ile	Amino acids and derivatives	Amino acids and derivatives	C18H35N3O4	2	0.7585	-	4886.79	4245.67	5952.46	12538.2	7195.66	11222.36	1.51118572	0.07020543	0.40874139	0.487298514	-1.03712227	down	-	-
MW0150834	His-Arg-Gly	Amino acids and derivatives	Amino acids and derivatives	C14H24N8O4	2	0.7584	-	42693.15	34816.52	41313.27	21910.71	23829.15	9893.95	1.4362343	0.02232785	0.27856654	2.135804469	1.094779575	up	-	-
MW0156042	Pro-Val-Ala-Glu-Val	Amino acids and derivatives	Amino acids and derivatives	C23H39N5O8	2	0.7569	-	689.39	115.17	846.71	2649.23	845.19	1676.01	1.1599279	0.14015888	0.530528	0.319368022	-1.64670823	down	-	-
MW0151168	His-Nap-OH	Amino acids and derivatives	Amino acids and derivatives	C25H22N4O6	2	0.7568	-	46493.99	43469.58	64290.54	31876.65	18116.84	16109.22	1.55383977	0.02557813	0.29249362	2.333551983	1.222527606	up	-	-
MW0152949	Lys-Glu-Arg-Phe-Ala	Amino acids and derivatives	Amino acids and derivatives	C29H47N9O8	2	0.7567	-	4297.93	5906.53	8239.01	2034.72	3913.93	3120.24	1.40123547	0.09446237	0.46692158	2.033707543	1.024112228	up	-	-
MW0152462	Leu-Pro-Arg-Leu-Pro	Amino acids and derivatives	Amino acids and derivatives	C28H50N8O6	2	0.7561	-	4448.09	3968.52	4125.67	129.29	20.8	33.02	1.71695139	0.00066375	0.12117687	68.4958768	6.097945241	up	-	-
MW0156675	Ser-Asp-Arg-Asp	Amino acids and derivatives	Amino acids and derivatives	C17H29N7O10	2	0.7551	-	2004.48	2329.59	2635.23	7636.21	4995.41	7366.05	1.67633837	0.03057956	0.30884667	0.348505601	-1.52074625	down	-	-
MW0157725	Thr-Lys-Gln-Lys	Amino acids and derivatives	Amino acids and derivatives	C21H41N7O7	2	0.7548	-	26728.71	16754.96	30864.05	6844.76	9310.51	15785.72	1.46856906	0.05644549	0.37828204	2.327658598	1.218879471	up	-	-
MW0150845	His-Asn-Ala-Glu	Amino acids and derivatives	Amino acids and derivatives	C18H27N7O8	2	0.7545	-	13043.88	17909.68	15472.78	2389.46	1743.26	16749.54	1.21243031	0.21891545	0.61131625	2.223243078	1.152665694	up	-	-
MW0151501	Ile-Leu-Val	Amino acids and derivatives	Amino acids and derivatives	C17H33N3O4	2	0.7544	-	1395.3	1032.92	1814.29	822.82	170.16	839.04	1.23996057	0.0635284	0.39280702	2.315755287	1.211482807	up	-	-
MW0153830	Methylcarbamyl PAF C-8	Others	Lactones	C18H39N2O7P	2	0.7538	-	77722.36	113336.61	105168.09	229612.54	251391.16	227768.2	1.70940308	0.00081656	0.12524365	0.417944137	-1.25861797	down	-	-
MW0158092	Trp-Ala-Tyr	Amino acids and derivatives	Amino acids and derivatives	C23H26N4O5	2	0.7535	-	63419.42	78778.15	76411.91	30088.55	19760.31	42995.71	1.57576386	0.00924779	0.2059482	2.354574748	1.235466523	up	-	-
MW0151093	His-Thr-Gln-Glu	Amino acids and derivatives	Amino acids and derivatives	C20H31N7O9	2	0.7526	-	1438.19	3487.6	1369.15	9317.77	9639.44	4392.77	1.5476528	0.06280056	0.392745	0.269590809	-1.89115678	down	-	-
MW0151176	His-TyrMe-OH	Amino acids and derivatives	Amino acids and derivatives	C22H22N4O7	2	0.7521	-	1175.04	2084.18	1898.13	715.55	873.37	747.24	1.56759964	0.07296243	0.41345377	2.207618485	1.142490871	up	-	-
MW0109201	Phenylalanylcysteine	Amino acids and derivatives	Amino acids and derivatives	C12H16N2O3S	2	0.7511	-	124772.46	125341.64	101458.85	7235.66	2378.68	1672.21	1.70965733	0.00339928	0.14905978	31.14972689	4.961147609	up	-	-
MW0152384	Leu-Ile-Thr-Arg	Amino acids and derivatives	Amino acids and derivatives	C22H43N7O6	2	0.7498	-	15825.88	12110.87	8756.88	99.1	142.85	217.86	1.7470176	0.02737278	0.29307299	79.80172245	6.318347981	up	-	-
MW0150978	His-Ile-Phe	Amino acids and derivatives	Amino acids and derivatives	C21H29N5O4	2	0.7491	-	6129.54	17967.38	5271.57	26778.33	65334.45	162689.55	1.50470877	0.20318928	0.5952815	0.115259896	-3.11703747	down	-	-
MW0158210	Trp-Met-Trp	Amino acids and derivatives	Amino acids and derivatives	C27H31N5O4S1	2	0.7485	-	20346.13	15834.69	11012.25	154.11	407.11	488.28	1.72304369	0.0292077	0.30170986	44.9671939	5.490800953	up	-	-
MW0157735	Thr-Lys-Ser-Lys	Amino acids and derivatives	Amino acids and derivatives	C19H38N6O7	2	0.748	-	1985.88	9649.14	5153.88	12077.81	16132.11	12477.47	1.35108421	0.04873003	0.35923867	0.41263153	-1.27707403	down	-	-
MW0002156	2,3-Dihydroxy-4-methoxy-4'-ethoxybenzophenone	Benzene and substituted derivatives	Benzene and substituted derivatives	C16H16O5	2	0.7478	-	5355.28	10913.18	3787.61	416.26	1460.85	2409.99	1.40872796	0.12752343	0.50962303	4.678237037	2.225964962	up	-	-
MW0158888	Val-Arg-Ala-Glu	Amino acids and derivatives	Amino acids and derivatives	C19H35N7O7	2	0.7472	-	14370.97	40512.78	15166.24	45122.5	50419.13	64046.74	1.43354094	0.05239157	0.37040011	0.438941697	-1.18789877	down	-	-
MW0151466	Ile-Ile-Asn	Amino acids and derivatives	Amino acids and derivatives	C16H30N4O5	2	0.7471	-	3865.61	11087.58	7329.85	20118.63	10976.1	13509.83	1.29380259	0.10066255	0.47968762	0.499568654	-1.00124514	down	-	-
MW0150127	Glu-Pro-Pro-Arg-Asp	Amino acids and derivatives	Amino acids and derivatives	C25H40N8O10	2	0.747	-	5609.29	18984.16	5685.93	2316.21	3950.04	3829.84	1.25671973	0.26848249	0.65025613	2.999119461	1.584538989	up	-	-
MW0150365	Gly-Gly-Asn-Glu-Glu	Amino acids and derivatives	Amino acids and derivatives	C18H28N6O11	2	0.7463	-	33556.98	33843.41	26881.53	6022.78	15854.81	13837.69	1.5014003	0.00794227	0.19725653	2.639820267	1.400439707	up	-	-
MW0158633	Tyr-Thr-Val-Thr-Leu	Amino acids and derivatives	Amino acids and derivatives	C28H45N5O9	2	0.7451	-	9191.67	13404.02	3051.88	4516.07	1874.98	3055.74	1.13847165	0.20924678	0.60095435	2.714950793	1.44092605	up	-	-
MW0150305	Gly-Arg-Ser-Asp-Asp	Amino acids and derivatives	Amino acids and derivatives	C19H32N8O11	2	0.7447	-	12149.79	6647.33	8056.48	3727.16	463.41	8148.43	1.0746965	0.16185326	0.54904711	2.176318989	1.121890032	up	-	-
MW0159145	Val-Pro-Ser	Amino acids and derivatives	Amino acids and derivatives	C13H23N3O5	2	0.7436	-	30893.37	11680.5	13947.43	504.88	170.53	1762.93	1.63773843	0.0959809	0.47047039	23.18023737	4.534823435	up	-	-
MW0152463	Leu-Pro-Asn	Amino acids and derivatives	Amino acids and derivatives	C15H26N4O5	2	0.7434	-	8221.1	9581.05	2535.77	2323.52	1615.21	2811.11	1.28058267	0.16768776	0.55689517	3.013096607	1.59124693	up	-	-
MW0108011	Lys-Lys-Arg	Amino acids and derivatives	Amino acids and derivatives	C18H38N8O4	2	0.7433	-	3122.46	11241.27	5039.8	27475.11	10962.93	15146.13	1.35719609	0.13381114	0.52029406	0.362113102	-1.46548772	down	-	-
MW0155931	Pro-Lys-Met	Amino acids and derivatives	Amino acids and derivatives	C16H30N4O4S1	2	0.7431	-	1087398.38	640736.38	805285.75	68.13	10.18	117.5	1.74791334	0.02303435	0.28031419	12938.15694	13.6593445	up	-	-
MW0157776	Thr-Pro-Asn	Amino acids and derivatives	Amino acids and derivatives	C13H22N4O6	2	0.7427	-	14048.54	15446.86	18939.39	5950.67	5830.45	9375.29	1.63684685	0.00917405	0.2053328	2.289367147	1.194948847	up	-	-

MW0155128	Phe-Asp-Gly-Gly-Val	Amino acids and derivatives	Amino acids and derivatives	C22H31N5O8	2	0.7413	-	412.63	483.7	561.68	1811.48	516.02	1508.63	1.25079661	0.17859106	0.57035301	0.380073147	-1.395651	down	-	-
MW0155806	Pro-Asp-Asn	Amino acids and derivatives	Amino acids and derivatives	C13H20N4O7	2	0.7405	-	72349.97	76300.01	57762.35	3563.54	1709.44	1825.73	1.74273341	0.00663572	0.18706012	29.07744224	4.861828465	up	-	-
MW0109233	Phe-Trp	Amino acids and derivatives	Amino acids and derivatives	C20H21N3O3	2	0.7402	-	3943.05	1754.64	5739.93	582.27	1034.11	1718.87	1.42260939	0.13502027	0.52248503	3.429314144	1.777920069	up	-	-
MW0158652	Tyr-Tyr-Leu	Amino acids and derivatives	Amino acids and derivatives	C24H31N3O6	2	0.7398	-	295810.18	377268.28	384226.69	25344.76	56850.59	47188.58	1.70792214	0.00457932	0.16605573	8.171842902	3.03066147	up	-	-
MW0155399	Phe-Val-Arg-Arg	Amino acids and derivatives	Amino acids and derivatives	C26H44N10O5	2	0.7391	-	2474.33	1321.66	8125.75	15069.4	9992.42	5544.31	1.18533759	0.15142082	0.54040731	0.389521315	-1.36022582	down	-	-
MW0145537	Arg-Tyr-Asn-Glu	Amino acids and derivatives	Amino acids and derivatives	C24H36N8O9	2	0.7369	-	14607.73	11052.63	3584.18	1629.83	2455.95	3152.22	1.4027512	0.15016176	0.5387718	4.040417242	2.014504284	up	-	-
MW0145847	Asn-Met-Tyr	Amino acids and derivatives	Amino acids and derivatives	C18H26N4O6S1	2	0.735	-	1581.95	2332.28	1792.02	2578.03	4565.12	5081.81	1.48916046	0.09437101	0.46692158	0.466770443	-1.09921488	down	-	-
MW0155920	Pro-Lys-Ala	Amino acids and derivatives	Amino acids and derivatives	C14H26N4O4	2	0.7316	-	37000.72	36615.06	39754.99	10186.2	20752.82	18602.08	1.56426327	0.01561898	0.24803772	2.288418505	1.194350916	up	-	-
MW0158396	Tyr-Asn-His-Arg	Amino acids and derivatives	Amino acids and derivatives	C25H36N10O7	2	0.7289	-	5113.32	3995.17	3711.49	14670.13	10350.98	5979.24	1.46793281	0.13329626	0.51987875	0.413543073	-1.27389049	down	-	-
MW0109327	Pro-Met-Arg	Amino acids and derivatives	Amino acids and derivatives	C16H30N6O4S1	2	0.7281	-	63983.49	57403.77	73307.09	225314.88	153155.54	222551.26	1.69081787	0.02545733	0.29249362	0.32393898	-1.62620601	down	-	-
MW0105680	Arg-Tyr-Arg	Amino acids and derivatives	Amino acids and derivatives	C21H35N9O5	2	0.7268	-	2450.77	753.05	1928.52	5454.34	4911.34	4719.11	1.47866513	0.01166455	0.22207747	0.340232778	-1.55540596	down	-	-
MW0158395	Tyr-Asn-His	Amino acids and derivatives	Amino acids and derivatives	C19H24N6O6	2	0.7266	-	112554.5	118848.4	120110.43	2688.52	30005.85	19205.22	1.47458514	0.00366928	0.15308276	6.772950037	2.759784353	up	-	-
MW0110230	Tyr-Val-Arg	Amino acids and derivatives	Amino acids and derivatives	C20H32N6O5	2	0.7255	-	63620.84	84991.14	78321.25	1236.61	4819.45	10220.46	1.62206505	0.00322343	0.14905978	13.9423679	3.801403697	up	-	-
MW0153049	Lys-Leu-Thr-Asp-Glu	Amino acids and derivatives	Amino acids and derivatives	C25H44N6O11	2	0.7235	-	5691.2	11155.39	12385.15	3453.62	3143.79	2447.37	1.58866907	0.07921186	0.43025564	3.23189066	1.69237839	up	-	-
MW0142517	2-O-alpha-L-Rhamnopyranosyl-D-glucopyranose	Others	Saccharides	C12H22O10	2	0.7225	-	1047840.42	387245.92	306951.7	279050.46	273165.55	257509.93	1.11769684	0.31654063	0.68542982	2.151392161	1.105270527	up	C08244	-
MW0153225	Lys-HoPhe-OH	Amino acids and derivatives	Amino acids and derivatives	C21H25N3O6	2	0.7182	-	1265.65	607.3	809.12	544.11	374.18	192.81	1.37635974	0.09687042	0.47154192	2.413887139	1.271358225	up	-	-
MW0144778	Ala-Tyr-Ile-Asp	Amino acids and derivatives	Amino acids and derivatives	C22H32N4O8	2	0.7178	-	13041.31	15734.76	14523.19	6036.14	7640.2	7063.26	1.70436161	0.0026276	0.14452248	2.08775772	1.061954299	up	-	-
MW0110184	Tyr-Ala-Lys	Amino acids and derivatives	Amino acids and derivatives	C18H28N4O5	2	0.7176	-	1998.3	1766.92	1779.81	4251.21	3993.74	4229.03	1.74752866	3.4239E-05	0.06238827	0.444527729	-1.16965468	down	-	-
MW0105651	Arg-Asp-Arg	Amino acids and derivatives	Amino acids and derivatives	C16H31N9O6	2	0.7175	-	4208.46	642.9	743.43	363.32	334.44	561.78	1.2000535	0.34278164	0.70386281	4.441931181	2.151187041	up	-	-
MW0155986	Pro-Pro-Val	Amino acids and derivatives	Amino acids and derivatives	C15H25N3O4	2	0.7169	-	4313.11	1934.81	4433.99	957.76	685.76	252.55	1.55848042	0.06151812	0.39031584	5.633710781	2.494085502	up	-	-
MW0110116	Trp-His	Amino acids and derivatives	Amino acids and derivatives	C17H19N5O3	2	0.7108	-	3382.67	1524.34	3373.21	933.29	1513.55	141.16	1.21594665	0.07163555	0.41116592	3.19946677	1.677831482	up	-	-
MW0156023	Pro-Tyr-Ala-Tyr-Asp	Amino acids and derivatives	Amino acids and derivatives	C30H37N5O10	2	0.7075	-	6613.68	8802.66	2557.9	25538.05	18364.25	7575.38	1.28674764	0.15553403	0.54478132	0.349165697	-1.51801626	down	-	-
MW0156677	Ser-Asp-Gln	Amino acids and derivatives	Amino acids and derivatives	C12H20N4O8	2	0.7051	-	10392.44	4180.16	8436.31	604.09	7116.54	668.26	1.19917335	0.16246703	0.54904711	2.74278361	1.455640806	up	-	-
MW0145939	Asn-Tyr-Tyr	Amino acids and derivatives	Amino acids and derivatives	C22H26N4O7	2	0.7049	-	633797.08	451227.71	332185.77	5019.07	11344.95	2891.13	1.72044406	0.03355602	0.31732169	73.60163696	6.201665948	up	-	-
MW0151597	Ile-Thr-His-Asp	Amino acids and derivatives	Amino acids and derivatives	C20H32N6O8	2	0.7038	-	2817.39	2561.39	2174.26	5716.61	8809.74	5122.77	1.65674772	0.06820919	0.40452854	0.384395841	-1.37933537	down	-	-
MW0159071	Val-Leu-Lys-Val-Leu	Amino acids and derivatives	Amino acids and derivatives	C28H54N6O6	2	0.7022	-	430.23	13.61	351.8	924.96	459.17	856.8	1.01599623	0.06849111	0.40512535	0.35504902	-1.49390987	down	-	-
MW0011341	NCGC00385256-01_C51H84O23_(3beta,8xi,9xi,14xi,16xi)-3-{[6-Deoxy-alpha-L-mannopyranosyl-(1->2)-[6-deoxy-alpha-L-mannopyranosyl-(1->4)]-beta-D-glucopyranosyl]oxy}-17,22-dihydroxyfurost-5-en-26-yl beta-D-glucopyranoside	Terpenoids	Terpene	C51H84O23	3	0.9921	-	2148.53	1186.47	1724.96	4459.94	5958.35	6121.89	1.65999044	0.00749201	0.19469501	0.305919283	-1.70877705	down	-	-
MW0118167	2-(2',3-Dimethyl-[2,4'-bipyridin]-5-yl)-N-(5-(pyrazin-2-yl)pyridin-2-yl)acetamide	Alcohol and amines	Amines	C23H20N6O	3	0.9913	-	303328.02	324663.58	249784.3	16360.86	15331.43	20468.92	1.75495594	0.00628084	0.18526407	16.82813531	4.072803419	up	-	-
MW0165918	(-)-Dehydrodiconiferyl acid carboxylate	Organic acids	Organic acids	C20H16O8-2	3	0.9707	-	309.91	3342	1999.94	15563.55	10943.85	8628.5	1.47254264	0.0262566	0.29249362	0.160856844	-2.63615077	down	-	-

MW0129755	1-[3-(3-{2,4-dihydroxy-3-[(1E)-3-methylbut-1-en-1-yl]benzoyl}-4-(2,4-dihydroxyphenyl)-6-methyl-7-oxabicyclo[4.1.0]heptan-2-yl)-2,4-dihydroxyphenyl]-3-(2,4-dihydroxyphenyl)propan-1-one	Benzene and substituted derivatives	Benzene and substituted derivatives	C40H40O11	3	0.9633	-	4450.83	3150.94	8591.86	5203.44	16119.96	14709.88	1.10941903	0.18433028	0.57441663	0.449407603	-1.15390356	down	-	-
MEDN1530	Cellobiose	Others	Saccharides	C12H22O11	3	0.9362	-	1578086.39	5759408.29	5729226.87	1504773.03	1508707.64	1509083.71	1.2665094	0.17676613	0.56928152	2.889228423	1.530684268	up	-	-
MW0148335	Dexylosylpradimicin C	Benzene and substituted derivatives	Benzene and substituted derivatives	C34H34N2O14	3	0.8487	-	11214.35	16516.53	5516.17	2284.15	7088.01	7015.43	1.0169447	0.21348177	0.6035828	2.02879435	1.020622633	up	C06785	-
MW0162660	P1,P4-Bis(5'-adenosyl) tetraphosphate	Nucleotides and derivatives	Nucleotides and derivatives	C20H24N10O19P4-4	3	0.829	-	735.57	187.09	626.21	5815.04	996	777.12	1.12295669	0.34520813	0.70481971	0.204116677	-2.29253404	down	-	-
ZINC9591020	4-O,6-O-(4,4',5,5',6,6'-Hexahydroxybiphenyl-2,2'-diyldicarbonyl)-beta-D-glucopyranose 1-(4-hydroxy-trans-cinnamate)	Others	Lactones	C29H24O16	3	0.7965	-	2757.86	1120.09	1250.07	19871.49	3396.04	5285.03	1.36053568	0.27176541	0.65233913	0.179599307	-2.47714631	down	-	-
MW0115273	Sesamolinol 4'-O-b-D-glucosyl(1->6)-O-b-D-glucoside	Benzene and substituted derivatives	Benzene and substituted derivatives	C32H40O17	3	0.7725	-	4243.78	3084.38	7916.54	535.2	3369.37	1127.66	1.29463106	0.13080529	0.51636171	3.029412408	1.599037992	up	-	-
ZINC3383085	[(1S,3S,5R,6S)-8-methyl-6-[(E)-2-methylbut-2-enoyl]oxy-8-azabicyclo[3.2.1]octan-3-yl] (E)-2-methylbut-2-enoate	Alkaloids	Tropan alkaloids	C18H27NO4	3	0.7495	-	14229.24	11038.54	13739.05	1535.39	1067.56	4357.68	1.62300354	0.00171759	0.13189913	5.603922346	2.486436964	up	-	-
MW0140693	benzyl 2-[[(2S)-5-(acetylsulfanylmethyl)-4-(1,3-benzodioxol-5-yl)-1-hydroxy-pyrrolidine-2-carbonyl]amino]acetate	Organic acids	Organic acids	C24H26N2O7S	3	0.7463	-	14813.69	5105.96	8949.26	2002.57	6513.79	2883.09	1.2695076	0.16389754	0.55155116	2.532482707	1.340552418	up	C01315	-
MW0167145	[(2S)-2-[(Z)-hexadec-9-enoyl]oxy-3-tetradecanoyloxypropyl] docosanoate	GL	TG	C55H104O6	3	0.7416	-	1041.94	1361.78	1303.21	683.05	610.59	429.46	1.61437982	0.00711888	0.19216671	2.151314491	1.105218442	up	-	-
MW0054938	Methyl(Z)-5-(1-propynyl)-2-thiophenecarboxylate	Others	Lactones	C11H10O2S	3	0.7407	-	2114.7	6165.73	2721.83	27898.05	21501.93	2588.83	1.1062387	0.21148444	0.60186131	0.211627464	-2.24040123	down	-	-
MW0166292	Mono(methylsulfanyl)gliotoxin	Alkaloids	Plumerane	C14H18N2O4S2	3	0.7376	-	4260.87	2501.7	1239.64	905.37	872.46	1519	1.24414643	0.210752	0.60186131	2.427243746	1.279318993	up	-	-
ZINC9591161	4',5,5''-Trihydroxy-4''',7''-dimethoxy-7-(beta-D-glucopyranosyloxy)-8,3'''-biflavone	Others	Ketone compounds	C38H32O15	3	0.7369	-	1367.76	639.25	387.83	1211.23	1858.18	2042.15	1.30051367	0.08094578	0.434301	0.468514504	-1.09383438	down	-	-
MW0159186	Val-Thr-Pro-Gly-Gly	Amino acids and derivatives	Amino acids and derivatives	C18H31N5O7	3	0.7301	-	1758.88	5669.31	2449.37	1970.52	251.7	2037.23	1.00557764	0.25963999	0.64677658	2.318975455	1.21348755	up	-	-
MW0161494	Mphpv II	Others	Ketone compounds	C17H18O6	3	0.7293	-	10924.06	686.56	8091.83	53.73	830.7	429.12	1.32864426	0.18173337	0.57250504	14.99939096	3.906832017	up	-	-
MW0130644	2-{3-[(6-carboxy-3,4,5-trihydroxyoxan-2-yl)oxy]-4-methoxyphenyl}-3-hydroxy-5-sulfino-3,4-dihydro-2H-1-benzopyran-7-olate	Benzene and substituted derivatives	Benzene and substituted derivatives	C22H24O13S	3	0.7292	-	4880.46	7087.04	7004.15	5162.94	1697.1	513.52	1.22383332	0.09077839	0.45758514	2.572929494	1.363411923	up	-	-
MW0150383	Gly-Ile-Asp-Lys-Phe	Amino acids and derivatives	Amino acids and derivatives	C27H42N6O8	3	0.7252	-	8825.49	17927.32	12902.33	41542.15	24414.9	24391.41	1.48815133	0.0800175	0.43345386	0.438913292	-1.18799213	down	-	-
MW0164396	1-[(2-azaniumyl-4-methylpentanoyl)amino]ethenyl-hydroxyphosphinate	Others	Others	C8H17N2O4P	3	0.7142	-	23076.61	22798.63	15120.43	12181.8	2886.47	4438.94	1.4578712	0.02388624	0.28304669	3.126826953	1.644699379	up	-	-
MW0168059	(E,2Z)-2-[amino(carboxy)methylidene]-5-oxopent-3-enoate	Others	Others	C7H6NO5-	3	0.714	-	1907.56	1603.94	962.82	5188.89	2635.05	1362.53	1.03010685	0.29559808	0.67760877	0.48705542	-1.03784216	down	-	-
MEDP1322	LysoPC(22:5(7Z,10Z,13Z,16Z,19Z))	Lipids	LPC	C30H52NO7P	3	0.7067	-	1350.75	3439.11	1315.73	9219.12	3190.1	3296.12	1.25223927	0.24480465	0.63712686	0.388758855	-1.36305256	down	C04230	ko00564
MW0011533	(E)-5-Hydroxyundec-2-enoic acid	Lipids	Free fatty acids	C11H20O3	3	0.7049	-	1887.21	2067.25	676.63	373.35	109.49	571.15	1.3965065	0.10141199	0.48076303	4.393865217	2.135490615	up	-	-
MW0062870	PS(20:5(5Z,8Z,11Z,14Z,17Z)/22:6(4Z,7Z,10Z,13Z,16Z,19Z))	GL	PS	C48H72NO10P	3	0.7049	-	1383.02	1984.91	1075.78	220.37	590.36	321.68	1.56761397	0.03878369	0.32974696	3.92411759	1.972368274	up	-	-
MW0144275	Acetyl-maltose	Others	Saccharides	C14H24O12	3	0.7038	-	106750.08	108140.73	95605.22	1939.88	301.06	2507.88	1.69880327	0.00116838	0.12524365	65.38382798	6.030861939	up	C02130	-

MW0011403	(5E)-heptadeca-5,16-diene-1,2,4-triol	Lipids	Others	C17H32O3	3	0.6993	-	5649.04	2260.07	2050.38	8372	9012.99	8696.79	1.50406285	0.04104799	0.33610993	0.381856223	-1.38889856	down	-	-
MW0122491	7-Hydroxymethotrexate	Heterocyclic compounds	Heterocyclic compounds	C20H22N8O6	3	0.6989	-	204543.7	89805.68	32672.19	17515.26	175.43	18095.57	1.23493693	0.1932416	0.58501187	9.138187953	3.191908116	up	-	-
MW0130172	2-(3,4-dihydroxyphenyl)-5-hydroxy-3-[(3,4,5-trihydroxy-6-methyloxan-2-yl)oxy]-7-[(3,4,5-trihydroxy-6-{[(3,4,5-trihydroxy-6-methyloxan-2-yl)oxy]methyl}oxan-2-yl)oxyl-4H-chromen-4-[illegible]	Others	Ketone compounds	C33H40O20	3	0.6967	-	2106.61	1345.46	3503.55	7515.79	7313.75	4700.33	1.50673134	0.02360938	0.28304669	0.356152908	-1.48943132	down	-	-
MW0049024	Coroloside	Heterocyclic compounds	Heterocyclic compounds	C35H54O12	3	0.6953	-	79282.66	99426.87	75795.7	14988.1	5206.64	23673.67	1.60364327	0.0021922	0.13948645	5.801560394	2.536440981	up	-	-
MW0165831	4-amino-demethoxy-Q6H2	Alcohol and amines	Alcohols	C38H59NO2	3	0.6949	-	29063.36	86702.63	5301.28	71331.98	561385.34	255425.14	1.33667569	0.2129377	0.60329506	0.136315147	-2.87498222	down	-	-
MW0145880	Asn-Pro-Thr	Amino acids and derivatives	Amino acids and derivatives	C13H22N4O6	3	0.6949	-	1758.77	610.88	657.35	138.59	47.64	1113.72	1.10642092	0.32020277	0.68798029	2.328551098	1.219432541	up	-	-
MW0003308	Phome	Benzene and substituted derivatives	Benzene and substituted derivatives	C23H19NO4	3	0.6948	-	119513.91	84412.91	105037.43	31731.46	27076.99	39003.17	1.72113976	0.01304079	0.23140906	3.158768355	1.659362142	up	-	-
MW0000226	dextrorphan O-glucuronide	Heterocyclic compounds	Heterocyclic compounds	C23H31NO7	3	0.6928	-	124.33	1.33	54.06	111.58	350.92	251.74	1.0775968	0.10706171	0.48330183	0.251624104	-1.99065797	down	-	-
MW0144505	Ala-Asn-Val	Amino acids and derivatives	Amino acids and derivatives	C12H22N4O5	3	0.6923	-	15737.93	7230.75	8782.27	1027.23	3842.96	5962.22	1.30035387	0.09856712	0.47667531	2.931106744	1.551445508	up	-	-
MEDP1671	Glu-Glu-Ile	Amino acids and derivatives	Amino acids and derivatives	C16H27N3O8	3	0.692	-	4830.63	4667.42	4171.02	8100.21	16522.75	16170.28	1.60947484	0.08066038	0.434301	0.335081744	-1.57741501	down	-	-
MEDP1412	Carnitine C13:1	Lipids	CAR	C20H37NO4	3	0.69	-	573.11	576.53	474.29	1833.32	1548.01	1166.33	1.7008767	0.0338737	0.31897138	0.357091339	-1.48563495	down	-	-
MW0144799	Ala-Val-His-Val-Lys	Amino acids and derivatives	Amino acids and derivatives	C25H44N8O6	3	0.6867	-	5308.59	11280.14	7051.53	25891.23	12213.66	12453.75	1.33082444	0.17565071	0.56921854	0.467581011	-1.09671175	down	-	-
MW0154042	Myxochromide S3	Alcohol and [illegible]	Amines	C40H56N6O8	3	0.6864	-	11769.45	7415.26	6197.07	2408.25	1053.51	2589.98	1.60729276	0.05317959	0.37242434	4.194129292	2.068371336	up	-	-
MW0168296	2-[2-[(2-Amino-3-phenylpropanoyl)amino]propanoylamino]-3-hydroxypropanoic acid	Amino acids and derivatives	Amino acids and derivatives	C15H21N3O5	3	0.6847	-	3595.8	5079.65	1424.71	7156.53	16570.5	21184.89	1.49077275	0.09854506	0.47667531	0.224888181	-2.15272025	down	-	-
MW0105632	Antanapeptin A	Benzene and substituted derivatives	Benzene and substituted derivatives	C41H60N4O8	3	0.6844	-	211157.47	364666.75	134836.6	1077027.19	897571.56	869400.39	1.65256533	0.00163211	0.13083964	0.249880814	-2.00068796	down	-	-
MW0162750	(2R,3S,4S,5R,6S)-2-(hydroxymethyl)-6-[6-[[(E)-4-hydroxy-3-methylbut-2-enyl]amino]purin-7-yl]oxane-3,4,5-triol	Alcohol and amines	Alcohols	C16H23N5O6	3	0.6822	-	33661.91	29242.98	29886.31	550.2	1105.74	786.19	1.74828142	0.00185481	0.13216141	37.99601168	5.247776086	up	-	-
MW0156019	Pro-Trp-Trp	Amino acids and derivatives	Amino acids and derivatives	C27H29N5O4	3	0.6818	-	289.52	365.67	185.13	244.69	931.6	910.5	1.13258993	0.20219907	0.59445191	0.402685464	-1.3122747	down	-	-
MW0011657	(S)-Succinyldihydrolipoami[illegible]	Organic acids	Organic acids	C12H21NO4S2	3	0.6804	-	12726.66	6561.47	6956.26	5993.26	3613.28	1487.24	1.27265627	0.11222645	0.49150111	2.365685096	1.242258044	up	C01169	-
MW0058179	1-Palmitoleoyl-2-(1-enyl-vaccenoyl)-sn-glycero-3-[illegible]	GL	PE	C39H74NO7P	3	0.68	-	17152.47	5747.28	16832.97	2072.41	3198.85	1132.11	1.56933435	0.09402214	0.46678757	6.204970195	2.633424281	up	C00350	ko00563,ko00564,ko01100,ko01110,ko04136
MW0169576	N-pentadecanoyl-L-Homoserine lactone	Others	Lactones	C19H35NO3	3	0.6788	-	25401.04	22116.49	23713.59	442.31	411.08	64.06	1.69356668	0.00140596	0.12524365	77.64032917	6.27873433	up	-	-
MW0158416	Tyr-Asp-Phe	Amino acids and derivatives	Amino acids and derivatives	C22H25N3O7	3	0.6774	-	5885.65	2622.26	2412.67	2340.16	1357.27	889.37	1.29301409	0.19276155	0.58497036	2.380871196	1.251489573	up	-	-
MW0156876	Ser-Phe-Leu	Amino acids and derivatives	Amino acids and derivatives	C18H27N3O5	3	0.6757	-	28767.78	33403.42	24761.4	443.91	510.74	480.63	1.75702548	0.00758625	0.19469501	60.56839084	5.920493178	up	-	-
MW0153110	Lys-Phe-Val-Leu-Val	Amino acids and derivatives	Amino acids and derivatives	C31H52N6O6	3	0.6754	-	5569.13	8727	10223.73	25948.68	8937.16	27173.52	1.24890482	0.16161439	0.54904711	0.395103333	-1.33969808	down	-	-
MW0165186	2-azaniumylprop-2-[illegible]	Others	Lactones	C3H5NO2	3	0.6735	-	234.85	42.73	43.78	937.26	162.34	508.8	1.29723082	0.18907162	0.58157172	0.199801045	-2.32336397	down	-	-
MW0148299	Deoxyviolaceinic acid	Organic acids	Organic acids	C21H15N3O3	3	0.6728	-	3940.72	3568.76	2571.27	1285.65	1024.08	798.35	1.66980932	0.0204294	0.27467954	3.243401071	1.697507435	up	C21132	-
MW0127906	(2E)-2-cyano-3-(4-ethoxy-3-methoxyphenyl)-N-(furan-2-ylmethyl)prop-2-enamide	Alcohol and amines	Amines	C18H18N2O4	3	0.6727	-	368.06	705.2	243.45	1283.73	2399.72	843.31	1.47987997	0.13798634	0.52754608	0.2908725	-1.78154119	down	-	-
MEDP1463	FFA(20:6)	Lipids	Free fatty acids	C20H28O2	3	0.6722	-	4701.11	3803.8	1300.8	7762.47	34197.73	18302.22	1.49301201	0.15787862	0.54808369	0.162716831	-2.61956461	down	-	-
MW0148712	dTDP-3,4-didehydro-2,6-dideoxy-alpha-D-glucose	Nucleotides and derivatives	Nucleotides and derivatives	C16H22N2O14P2	3	0.6715	-	3205.44	9943.55	837.35	20762	9048.91	8041.44	1.18870218	0.1907991	0.58497036	0.36949727	-1.43636439	down	C06620	ko01100,ko01110,ko01250
MW0145756	Asn-Gly-Phe	Amino acids and derivatives	Amino acids and derivatives	C15H20N4O5	3	0.6713	-	9526.32	14625.47	7636.49	2806.03	4116.77	2604.73	1.61124272	0.06432594	0.39280702	3.33646601	1.738320806	up	-	-
MW0155077	Phe-Ala-His	Amino acids and derivatives	Amino acids and derivatives	C18H23N5O4	3	0.6676	-	973.95	1147.75	1413.27	361.26	372.82	28.59	1.32275382	0.0058594	0.18250174	4.634992854	2.212567115	up	-	-

MW0013501	2-Sulfooxy-2-deoxytartaric acid	Organic acids	Organic acids	C4H6O9S	3	0.6675	-	8028.01	11855.4	271.76	17874.28	49143.66	50734.31	1.27264462	0.08176869	0.43607355	0.171165901	-2.54653277	down	-	-
MW0155306	Phe-Phe-Thr	Amino acids and derivatives	Amino acids and derivatives	C22H27N3O5	3	0.665	-	5545.11	4594.88	5633.3	467.66	3821.12	3449.45	1.06421258	0.11710705	0.49537633	2.038358901	1.027408094	up	-	-
MW0061286	CID 131823277	GL	PG	C33H65O10P	3	0.664	-	7145.65	4867.84	7920.73	19878.91	15554.21	5625.82	1.0501706	0.23377608	0.62914723	0.485502548	-1.04244923	down	-	-
MW0128771	[4-(7-Hydroxy-8-methoxy-4-oxo-2,3-dihydrochromen-2-yl)phenyl] hydrogen sulate	Organic acids	Organic acids	C16H14O8S	3	0.6636	-	655.22	1753.77	779.67	2853.87	8811.31	3450.22	1.53696309	0.16694161	0.55563746	0.210954391	-2.24499698	down	-	-
MW0012973	1-Palmitoyl-2-oleoyl-sn-glycero-3-phosphate	GL	PA	C37H71O8P	3	0.6631	-	13590.95	9808.62	14093.49	1507.65	1451.77	1317.77	1.75548896	0.01446887	0.24503557	8.765815874	3.131888375	up	C00416	ko00561,ko00564,ko01100,ko01110,ko04070
MW0116346	3-[(E)-(2-nitrophenyl)methylideneamino]imidazolidine-2,4-dione	Benzene and substituted derivatives	Benzene and substituted derivatives	C10H8N4O4	3	0.6617	-	2177.69	2344.79	2515.69	1367.35	541.04	946	1.55079944	0.01691103	0.25446523	2.465735236	1.302017895	up	-	-
MW0003567	3,3'-Dimethylbenzidine	Benzene and substituted derivatives	Benzene and substituted derivatives	C14H16N2	3	0.6615	-	3803.07	3071.39	127.85	3236.8	8118.48	5919.09	1.02810807	0.13410363	0.52029406	0.405358343	-1.30273026	down	C14443	-
MW0012843	1-Tetracosanoyl-sn-glycero-3-phosphocholine	Lipids	LPC	C32H66NO7P	3	0.6566	-	1242.73	1719.97	2569.89	6891.56	10359.16	3190.46	1.4877791	0.13394734	0.52029406	0.270659032	-1.88545156	down	C04230	ko00564
MEDP1527	Carnitine C19:0	Lipids	CAR	C26H51NO4	3	0.656	-	203166.53	179285.14	223060.29	84761.02	80857.12	92506.34	1.74602457	0.00811884	0.19760292	2.345813772	1.230088486	up	-	-
MW0110677	2-[Bis-(2-hydroxy-ethyl)-amino]-2-hydroxymethyl-propane-1,3-diol	Alcohol and amines	Alcohols	C8H19NO5	3	0.6554	-	2764.5	3078.45	2135.34	10156.44	12378.85	13351.02	1.74037232	0.00647662	0.18604545	0.22232127	-2.16928212	down	-	-
MW0148768	dTDP-alpha-D-forosamine	Nucleotides and derivatives	Nucleotides and derivatives	C18H31N3O12P2	3	0.6547	-	821.77	2286.55	1119.92	284.51	201.25	1056.94	1.25021802	0.17721818	0.56946912	2.740805082	1.454599732	up	C18034	ko01100,ko01110,ko01250
MW0156573	Scopoloside II	Terpenoids	Triterpene	C39H62O15	3	0.654	-	40987.42	30580.57	19986.89	140807.63	153922	137057.76	1.70870547	0.00016587	0.07934638	0.212036947	-2.23761242	down	C17922	-
ZINC4430631	ethyl 5-O-caffeoyl-3-O-sinapoylquinate	Others	Lactones	C29H32O13	3	0.6515	-	10037.04	27044.38	54180.62	3324.47	2657.53	5395.12	1.54861758	0.17360824	0.56648433	8.02154148	3.003879502	up	-	-
MW0110054	Trp-Asn-Arg	Amino acids and derivatives	Amino acids and derivatives	C21H30N8O5	3	0.6504	-	1167.61	1480.96	1401.99	5820.29	3828.75	2436.29	1.58045482	0.110933	0.48968655	0.335163376	-1.57706358	down	-	-
MW0156640	Ser-Arg-Glu-Val-Ser	Amino acids and derivatives	Amino acids and derivatives	C22H40N8O10	3	0.6495	-	1237.28	2295.46	1818.72	13513.53	11918.26	7350.79	1.70447078	0.03541827	0.32593586	0.163240965	-2.61492495	down	-	-
MW0151151	His-Val-Leu-Lys	Amino acids and derivatives	Amino acids and derivatives	C23H41N7O5	3	0.6494	-	10729.96	2726.14	9933.8	95.54	148.25	108.84	1.7263746	0.09455843	0.46692158	66.32986416	6.051586666	up	-	-
MW0148752	dTDP-4-oxo-2-deoxy-alpha-D-pentos-2-ene	Nucleotides and derivatives	Nucleotides and derivatives	C15H20N2O14P2	3	0.6472	-	208890.95	270888.61	131732.01	915356.81	395049	806285.99	1.54540879	0.07885094	0.42947714	0.288899674	-1.79135952	down	C21352	ko01110
MW0143824	6-Desmethoxy hormothamnione triacetate	Others	Lactones	C27H26O11	3	0.6412	-	24.55	392.13	307.02	2041.34	921.7	2458.01	1.40450376	0.06881359	0.40595463	0.133498123	-2.90510864	down	-	-
MEDL00371	Thr-Val-Ile	Amino acids and derivatives	Amino acids and derivatives	C15H29N3O5	3	0.6387	-	18363.67	17473.52	16536.94	2751.73	7468.26	7616.18	1.52210012	0.01210934	0.22465715	2.936400023	1.554048519	up	-	-
MW0157859	Thr-Val-Glu-Thr-Trp	Amino acids and derivatives	Amino acids and derivatives	C29H42N6O10	3	0.6372	-	1465.57	4787.37	7451.14	406.95	2027.27	1394.33	1.1875591	0.19147688	0.58497036	3.579443915	1.839735475	up	-	-
MW0109304	Pro-Asn	Amino acids and derivatives	Amino acids and derivatives	C9H15N3O4	3	0.6371	-	3320.63	3466.1	3309.18	179.46	433.12	456.13	1.70199651	5.5657E-05	0.06238827	9.446819062	3.239828626	up	-	-
MW0156969	Ser-Val-Asn	Amino acids and derivatives	Amino acids and derivatives	C12H22N4O6	3	0.6371	-	13045.03	11234.66	10372.5	300.51	4003.66	3872.03	1.30770578	0.00592363	0.18257415	4.238177882	2.083444141	up	-	-
MW0105633	Antanapeptin B	Benzene and substituted derivatives	Benzene and substituted derivatives	C41H62N4O8	3	0.6353	-	5588.44	1663.24	611.76	11178.34	6972.34	2017.97	1.0024253	0.26648086	0.6495827	0.389884301	-1.35888203	down	-	-
MW0155059	Phaeophorbide b	Heterocyclic compounds	Heterocyclic compounds	C35H34N4O6	3	0.6351	-	5654.06	2465.56	3548.35	1359.88	1263.85	2534.22	1.40123839	0.13235355	0.51892846	2.262133212	1.177683889	up	-	-
MW0151428	Ile-Glu-Val-His-Gly	Amino acids and derivatives	Amino acids and derivatives	C24H39N7O8	3	0.635	-	581.19	377.35	323.14	2343.33	1215.39	995.07	1.56911632	0.11598558	0.49388123	0.281453471	-1.82903166	down	-	-
MW0105773	Asn-Glu-Arg	Amino acids and derivatives	Amino acids and derivatives	C15H27N7O7	3	0.6347	-	7012.31	4118.78	13760.71	3616.49	3191.28	2356.77	1.38137214	0.20624885	0.59767885	2.716099226	1.441536186	up	-	-
MEDP1378	Carnitine C21:2	Lipids	CAR	C28H51NO4	3	0.6307	-	101787.03	118982.47	143384.43	275187.94	235341.99	234295.47	1.67702697	0.00226684	0.1402294	0.488911804	-1.03235386	down	-	-
MW0010618	Lyoniresinol 9'-sulfate	Lignans and Coumarins	Lignans	C22H28O11S	3	0.6292	-	13661.74	3457.8	13374.52	24168.89	17824.91	33969.75	1.24230714	0.06470378	0.39316593	0.40143016	-1.31677908	down	-	-
MW0127557	S-2-Propenyl 2-propene-1-sulfonothioate	Others	Lactones	C6H10O2S2	3	0.6288	-	473.13	582.87	2144.27	3781.13	1086.96	8309.61	1.13612069	0.25178943	0.64084008	0.242854975	-2.04183306	down	-	-
ZINC1334032	4-[(2R)-3-(2,4-dihydroxyphenyl)-2-hydroxypropyl]benzene-1,2-diol	Benzene and substituted derivatives	Benzene and substituted derivatives	C15H16O5	3	0.6281	-	210.74	730.38	156.96	162.04	133.69	44.08	1.11104557	0.29961473	0.67873528	3.231452871	1.692182951	up	-	-
MW0145358	Arg-Glu-Val	Amino acids and derivatives	Amino acids and derivatives	C16H30N6O6	3	0.6278	-	27752.94	31376.79	40421.99	92906.71	90162.25	40945.07	1.40849421	0.1271968	0.50962303	0.444399487	-1.17007094	down	-	-
MW0110197	Tyr-Glu-Arg	Amino acids and derivatives	Amino acids and derivatives	C20H30N6O7	3	0.6239	-	2751.57	8259.67	5276.41	2345.74	2355.89	886.41	1.3106198	0.14514475	0.53649353	2.914733968	1.543364212	up	-	-
MW0150513	Gly-Tyr-Asp	Amino acids and derivatives	Amino acids and derivatives	C15H19N3O7	3	0.6235	-	53983.95	66499.19	73266.69	14110.34	3551.53	6275.89	1.6625355	0.00262417	0.14452248	8.093899763	3.016834982	up	-	-
MW0146142	Asp-Ile-Gln	Amino acids and derivatives	Amino acids and derivatives	C15H26N4O7	3	0.6231	-	192332.44	171005.33	143046.02	5011.44	4451.49	4306.25	1.75819619	0.00745788	0.19469501	36.77661197	5.200716673	up	-	-

MW0150952	His-His-Ala-Ala-Tyr	Amino acids and derivatives	Amino acids and derivatives	C27H35N9O7	3	0.6228	-	12928.63	15725.66	22789.12	1234.47	5625.14	7924.24	1.37373129	0.03200364	0.31330981	3.47970319	1.798964253	up	-	-
MW0130153	Taxifolin 3-O-bata-xylopyranoside	Flavonoids	Flavones	C20H20O11	3	0.6228	-	2590.3	276.5	859.36	1930.21	5226.23	6576.95	1.2492861	0.12113186	0.50042564	0.271321211	-1.88192626	down	-	-
MEDP0535	TRIETHYL PHOSPHATE	Organic acids	Organic acids	C6H15O4P	3	0.6194	-	8581.56	6775.88	10944.58	15402.99	26997.55	27925.07	1.57113671	0.0575944	0.3800377	0.374003439	-1.41887656	down	-	-
MW0158665	Tyr-Val-Glu-Asp	Amino acids and derivatives	Amino acids and derivatives	C23H32N4O10	3	0.6176	-	7436.95	7715.69	2531.17	15327.71	15799.95	8426.62	1.3087452	0.07383877	0.41630112	0.447077029	-1.16140467	down	-	-
MW0104141	{4-[(1Z)-2-hydroxy-3-oxobut-1-en-1-yl]-2-methoxyphenyl}oxidane sulfonic acid	Organic acids	Organic acids	C11H12O7S	3	0.6175	-	139.06	174.32	169.97	578.87	1457.29	1648.79	1.66698353	0.08334087	0.44033244	0.131168673	-2.9305049	down	-	-
MW0145557	Arg-Val-Asn-His-Val	Amino acids and derivatives	Amino acids and derivatives	C26H45N11O7	3	0.6148	-	27325.67	39217.92	33262.96	2421.76	5874.99	12612.76	1.54120062	0.00472478	0.16605573	4.773261066	2.254975245	up	-	-
MW0149735	Gln-Leu-Asn-Leu-Lys	Amino acids and derivatives	Amino acids and derivatives	C27H50N8O8	3	0.614	-	968.06	211.62	750.17	240.62	97.36	106.48	1.40491913	0.15368326	0.54291084	4.34201053	2.118363225	up	-	-
MW0145929	Asn-Tyr-Gln-Phe-Phe	Amino acids and derivatives	Amino acids and derivatives	C36H43N7O9	3	0.6097	-	4524.21	6643.28	2816.97	397.38	1288.88	4688.48	1.08403754	0.21463382	0.60477561	2.193730254	1.13338614	up	-	-
MW0103729	2-(2,6-Dimethylmorpholin-4-yl)-6-(3-methylpiperidin-1-yl)-5-nitropyrimidin-4-amino	Heterocyclic compounds	Heterocyclic compounds	C16H26N6O3	3	0.6095	-	2291	698	819.04	836.32	44.01	527.82	1.05355684	0.25627234	0.64457434	2.704285765	1.435247611	up	-	-
MW0057438	PC(20:5(5Z,8Z,11Z,14Z,17Z)/24:1(15Z))	GL	PC	C52H92NO8P	3	0.608	-	1059.97	611.43	151.69	704.78	1625.62	1709.96	1.09688805	0.15259509	0.54104356	0.451219693	-1.14809806	down	-	-
MW0150206	Glu-Val-Gln	Amino acids and derivatives	Amino acids and derivatives	C15H26N4O7	3	0.6071	-	309001.59	385563.14	467087.66	147861.11	108069.54	220696.02	1.58014885	0.01858368	0.26614704	2.437237492	1.285246839	up	-	-
MW0134152	8-[5,7-Dihydroxy-2-(4-hydroxyphenyl)-4-oxo-2,3-dihydrochromen-3-yl]-5-hydroxy-2-(4-hydroxy-3-methoxyphenyl)-7-[3,4,5-trihydroxy-6-(hydroxymethyl)oxan-2-yl]oxy-2,3-dihydrochromen-4-one	Benzene and substituted derivatives	Benzene and substituted derivatives	C37H34O16	3	0.6066	-	60678.51	68264.92	64488.34	26463.79	35178.26	9885.17	1.43163721	0.02419935	0.28436137	2.704309912	1.435260493	up	-	-
MW0007659	Methyl 2-(2-hydroxy-6-methoxy-4-methylbenzoyl)-3-methoxy-5-sulfooxybenzoate	Benzene and substituted derivatives	Benzene and substituted derivatives	C18H18O10S	3	0.6044	-	49.48	85.85	1203.44	1539.11	507.74	912.22	1.06671471	0.32927596	0.69375361	0.452429311	-1.1442357	down	-	-
MW0155282	Phe-Lys-Val	Amino acids and derivatives	Amino acids and derivatives	C20H32N4O4	3	0.6018	-	1710.7	1046.8	1473.77	0.77	0.77	263.79	1.45812952	0.0104948	0.21412876	15.94719783	3.995231037	up	-	-
MW0016660	CDP-DG(a-25:0/i-19:0)	GL	DG	C56H105N3O15P2	3	0.6015	-	2053.92	3220.11	3452.65	223.89	1028.01	2415.69	1.15486387	0.10393388	0.48180112	2.379404459	1.250600527	up	-	-
MW0146112	Asp-Gly-His	Amino acids and derivatives	Amino acids and derivatives	C12H17N5O6	3	0.5987	-	6900.25	6069.8	8965.24	23096.15	11807.49	9034.88	1.25684727	0.2271301	0.62216327	0.499226874	-1.0022325	down	-	-
MW0112501	3,4,5-trihydroxy-6-[3-(4-hydroxy-3-methoxyphenyl)oxirane-2-carbonyloxy]oxane-2-carboxylic acid	Organic acids	Organic acids	C16H18O11	3	0.5975	-	5.73	5.73	5.73	768.14	524.54	149.43	1.71979232	0.11854488	0.4975454	0.011920034	-6.39046786	down	-	-
MW0163505	CTP trianion	Nucleotides and derivatives	Nucleotides and derivatives	C9H13N3O14P3-3	3	0.5948	-	30636.11	33515.55	44476.81	19888.61	19964.87	2800.56	1.18589191	0.04055068	0.33610993	2.54673344	1.348647964	up	-	-
MW0150446	Gly-Phe-Asp	Amino acids and derivatives	Amino acids and derivatives	C15H19N3O6	3	0.5926	-	3155.56	1045.16	4186.56	88.56	1220.94	1445.35	1.0679639	0.1684189	0.55689517	3.04455052	1.606229253	up	-	-
MW0159211	Val-Tyr-Gln	Amino acids and derivatives	Amino acids and derivatives	C19H28N4O6	3	0.5916	-	37884.35	63162.21	54429.58	102963.03	150169.8	71214	1.44360398	0.12278535	0.50274678	0.479351502	-1.06084414	down	-	-
MW0013188	2-(Sulooxymethyl)butanoic acid	Organic acids	Organic acids	C5H10O6S	3	0.5897	-	5209.35	2635.87	13636.2	1771.44	1469.53	1137.13	1.45960319	0.22798816	0.62328279	4.906562207	2.294712551	up	-	-
MW0157849	Thr-Tyr-Thr	Amino acids and derivatives	Amino acids and derivatives	C17H25N3O7	3	0.5887	-	10251.25	5654.74	10739.44	329.56	1109.87	2817.87	1.51380004	0.02845269	0.30030403	6.258762596	2.645877454	up	-	-
MW0148737	dTDP-4-amino-2,4-dideoxy-beta-L-xylose	Nucleotides and derivatives	Nucleotides and derivatives	C15H25N3O13P2	3	0.5877	-	10113.18	9460.46	16911.98	41916.82	28978.9	17306.21	1.40951985	0.12490661	0.50714133	0.413660109	-1.27348225	down	C21354	ko01110
MW0123903	ethyl {10-[3-(morpholin-4-yl)propanoyl]-5-oxido-10H-phenothiazin-2-yl}carbamate	Others	Lactones	C22H25N3O5S	3	0.5872	-	8263.06	7659.24	8470.31	24.2	97.29	58.96	1.73620744	0.0008393	0.12524365	135.1765586	7.078701181	up	-	-
MW0057441	PC(20:5(5Z,8Z,11Z,14Z,17Z)/P-18:1(11Z))	GL	PC	C46H80NO7P	3	0.5868	-	49129.35	40515.74	25503.2	9021.02	21578.08	6000.84	1.43476684	0.04168829	0.3382143	3.1461333	1.653579798	up	-	-
MW0151012	His-Lys-Ile	Amino acids and derivatives	Amino acids and derivatives	C18H32N6O4	3	0.5856	-	50040.98	25615.91	34272.87	492.65	15.95	154.96	1.67329679	0.03639661	0.32815889	165.6666466	7.372139366	up	-	-
MW0110190	Tyr-Asp-Arg	Amino acids and derivatives	Amino acids and derivatives	C19H28N6O7	3	0.5848	-	29153.06	18243.32	35857.83	7065.12	969.39	615.23	1.55001	0.02681108	0.29249362	9.62505347	3.266794555	up	-	-

MW0155805	Pro-Asp-Ala-Lys-Ser	Amino acids and derivatives	Amino acids and derivatives	C21H36N6O9	3	0.584	-	1420.83	835.62	1722.92	607.63	370.85	398.61	1.59340501	0.06979973	0.4085256	2.889694936	1.530917196	up	-	-
MW0148029	Cys-Leu-Met	Amino acids and derivatives	Amino acids and derivatives	C14H27N3O4S2	3	0.5835	-	3076.74	540.98	3697.95	5937.41	6535.76	5695.75	1.19156612	0.05647902	0.37828204	0.402647488	-1.31241076	down	-	-
MW0016463	Cdp-DG(18:1(11Z)/22:3(10Z,13Z,16Z))	GL	DG	C52H89N3O15P2	3	0.579	-	1280728.72	1053435.44	1039645.27	13189.79	18174.27	15160.52	1.7582493	0.00491821	0.16850553	72.51670902	6.180241548	up	-	-
MW0150192	Glu-Tyr-Ile	Amino acids and derivatives	Amino acids and derivatives	C20H29N3O7	3	0.5784	-	1581.01	956.69	1234.45	6.36	5.82	21.41	1.74030144	0.02029142	0.2743902	112.2997916	6.81121144	up	-	-
ZINC1338057	Cuscuta propenamide 2	Alcohol and [illegible]	Amines	C22H27NO3	3	0.576	-	43111.52	18813.04	60097.57	8674.65	3845.01	18836.21	1.41947585	0.11468835	0.49367245	3.891524298	1.960335365	up	-	-
MW0148064	Cys-Ser-Gln	Amino acids and derivatives	Amino acids and derivatives	C11H20N4O6S1	3	0.5721	-	51247.81	94039.59	87819.92	15919.08	6557.55	2146.24	1.57673437	0.0266845	0.29249362	9.467105987	3.242923474	up	-	-
MW0108017	Lys-Pro-Lys	Amino acids and derivatives	Amino acids and derivatives	C17H33N5O4	3	0.5703	-	468.82	299.72	474.99	0.68	0.85	0.23	1.74582869	0.01870618	0.26704265	706.5511364	9.464650169	up	-	-
MW0021617	CL(8:0/10:0/10:0/11:0)	GL	CL	C48H92O17P2	3	0.5688	-	349.06	1075.53	2142.34	8336.63	7160.75	6111.93	1.55753778	0.00223114	0.13996451	0.165064502	-2.5988982	down	-	-
MW0141660	19-(4-Hydroxyphenyl)nonadecanoic acid	Lipids	Free fatty acids	C25H42O3	3	0.5686	-	195.23	209.66	152.2	902.82	468.32	435.06	1.61350951	0.10795623	0.48477095	0.308432067	-1.69697533	down	C21448	-
ZINC1337858	boeravinone F	Others	Ketone compounds	C17H10O7	3	0.5667	-	75.27	840.16	568.69	439.67	3895.89	8134.3	1.16239827	0.24073442	0.63445628	0.119016573	-3.07076562	down	-	-
MW0126657	Termitomycamide B	Alcohol and [illegible]	Amines	C28H40N2O2	3	0.5663	-	308.63	1536	1700.58	1822.7	4133.37	2615.81	1.1854956	0.11827193	0.4975454	0.413586051	-1.27374057	down	-	-
MW0128248	(5-{8-[1-(2,4-dihydroxyphenyl)-3-(3,4-dihydroxyphenyl)-2-hydroxypropyl]-3,5,7-trihydroxy-3,4-dihydro-2H-1-benzopyran-2-yl}-2-hydroxyphenyl)oxidanesulfonic acid	Organic acids	Organic acids	C30H28O14S	3	0.5644	-	3388.15	2297.06	3422.46	3879.71	11820.79	8387.91	1.37730836	0.15893971	0.54886804	0.378093448	-1.40318524	down	-	-
MW0145997	Asn-Tyr	Amino acids and derivatives	Amino acids and derivatives	C13H17N3O5	3	0.5601	-	1958.46	905.25	1886.14	8052.02	6198.19	5602.21	1.635143	0.01020257	0.21412876	0.239257985	-2.06336102	down	-	-
MW0003306	2-O-Galloyl-1,4-galactarolactone	Benzene and substituted derivatives	Benzene and substituted derivatives	C13H12O11	3	0.5547	-	1554.73	1870.63	2371.42	2675.94	5671.81	7095.71	1.44263301	0.12801146	0.50962303	0.375355005	-1.41367238	down	-	-
MW0167120	[(2S)-1-pentadecanoyloxy-3-tetradecanoyloxypropan-2-yl] octadecanoate	GL	TG	C50H96O6	3	0.5461	-	2090.87	1337.61	660.7	746.52	125.9	304.62	1.32815725	0.12873694	0.51098122	3.474121525	1.796648221	up	-	-
Hmln001682	Quercetin-3-O-(6''-acetyl)glucosyl-(1→3)-Galactoside	Flavonoids	Flavones	C29H32O18	3	0.5431	-	959.81	656.28	1211.02	1329.43	3103.42	3223.6	1.42204855	0.11114406	0.48968655	0.369245538	-1.43734761	down	-	-
ZINC8584497	1,3-Bis[(14R)-13,14-dihydro-13-methyl[1,3]benzodioxolo[5,6-c]-1,3-dioxolo[4,5-i]phenanthridin-14-yl]-2-propanone	Alkaloids	Alkaloids	C43H32N2O9	3	0.5413	-	5830.35	2434.05	1907.02	1311.32	153.27	387.29	1.43946526	0.1442515	0.53496769	5.492483314	2.457458582	up	-	-
MW0107262	His-Leu-Lys	Amino acids and derivatives	Amino acids and derivatives	C18H32N6O4	3	0.5403	-	7203.77	4120.06	8511.11	106.64	9.43	26.41	1.70904664	0.03708528	0.32893629	139.2120999	7.121140802	up	-	-
MW0061789	PI(20:3(8Z,11Z,14Z)/20:3(8Z,11Z,14Z))	GL	PI	C49H83O13P	3	0.5259	-	2797.7	2060.66	2442.76	9217.76	7870.83	6647.29	1.71606931	0.01266104	0.2293489	0.307598454	-1.70087984	down	-	-
MW0161020	(2S)-4-(2-aminophenyl)-2-azaniumyl-4-[illegible]	Others	Lactones	C10H12N2O3	3	0.5247	-	1766.05	10225.78	11421.56	7028.58	22.25	916.77	1.00805102	0.24840347	0.63798289	2.938574979	1.55511671	up	-	-
MW0060996	{2,3-bis[(15Z)-tetracos-15-enoyloxy]propoxy}[2-(dimethylamino)ethoxy]phosphinic acid	GL	PE	C55H106NO8P	3	0.5227	-	2460.24	4870.37	3860.52	1772.94	446.86	1249.57	1.44059969	0.0459037	0.3512974	3.225695155	1.689610103	up	-	-
MW0107572	1-(2-Amino-3-methylpentanoyl)pyrrolidine-2-carboxylic acid	Organic acids	Organic acids	C11H20N2O3	3	0.5192	-	1917.28	1844.99	1883.08	14610.96	2490.32	3819	1.19935286	0.31583228	0.68542982	0.269850595	-1.88976723	down	-	-
MW0154396	Neu5Ac2-3[Gal1-3GalNAc1-4]Gal1-[illegible]	Others	Saccharides	C39H65N5O29	3	0.5159	-	10062.35	11890.08	2050.68	20938.91	29077.76	16207.05	1.31101243	0.04577158	0.3512974	0.362454873	-1.46412671	down	-	-
MW0163764	2-Aminobut-2-enoate	Others	Lactones	C4H6NO2-	3	0.5128	-	24617.73	26631.44	26385.03	704.14	700.24	616.55	1.76005378	0.00061837	0.12117687	38.41508612	5.263601083	up	-	-
MW0144530	Ala-Cys-Val	Amino acids and derivatives	Amino acids and derivatives	C11H21N3O4S1	3	0.5057	-	9665.78	3194.28	8361.75	4544.33	748.61	2615.62	1.20596219	0.14063838	0.530528	2.683397483	1.424060772	up	-	-
MW0153513	Met-Asp-Gln	Amino acids and derivatives	Amino acids and derivatives	C14H24N4O7S1	3	0.5049	-	5744.32	5279.59	10424.41	5630.63	1975.89	3102.11	1.27333698	0.15394533	0.54332212	2.002900464	1.002090727	up	-	-

MW0166696	alpha-D-xylopyranosyl-(1->3)-beta-D-glucopyranuronosyl-(1->3)-alpha-D-xylopyranosyl-(1->3)-beta-D-glucopyranuronic acid	Organic acids	Organic acids	C22H34O21	3	0.501	-	6140.68	1428.86	2975.35	813.59	1002.39	2858.04	1.04901921	0.29588834	0.67791845	2.256064373	1.173808233	up	-	-
Zmhn002301	4-O-beta-D-glucosyl-4-coumaric acid	Organic acids	Organic acids	C15H18O8	2	0.9912	14364-05-7	3460103.23	1445709.11	2647355	1478850.09	746017.72	1161458.79	1.38513338	0.12878201	0.51098122	2.230489918	1.157360627	up	C04415	-
MEDN0675	Methyl stearate	Lipids	Free fatty acids	C19H38O2	2	0.9514	112-61-8	6671722.85	8511400.76	6845639.74	40403334.1	37255369.96	31348919.24	1.75237882	0.00623041	0.1850017	0.202084613	-2.30696862	down	-	-
MW0015702	alpha-Hederin	Terpenoids	Triterpene Saponin	C41H66O12	2	0.8979	27013-91-8 | 104748-88-1	1993.4	2713.02	2153.61	4435.24	5215.63	4832.16	1.71297498	0.00126656	0.12524365	0.473659863	-1.07807667	down	C08954	-
MW0105667	Thymotrinan	Amino acids and derivatives	Amino acids and derivatives	C16H31N7O6	2	0.8971	85465-82-3	6380.43	6265.36	7586.69	2653.62	1685.17	4341.9	1.52608206	0.02102755	0.27627352	2.330745597	1.220791542	up	-	-
MW0119612	3-Hydroxy-3-methyloxindole	Alkaloids	Plumerane	C9H9NO2	2	0.8902	3040-34-4	43789.01	36864.38	46143.6	13214.5	20823.52	21066.22	1.64368751	0.0033146	0.14905978	2.301038722	1.202285262	up	C05834	ko00380
MW0055346	Nonadecanoic acid	Lipids	Free fatty acids	C19H38O2	2	0.8837	646-30-0	2783467.49	3400315.48	3100273.63	7465308.12	7649419.24	6504664.65	1.74192929	0.00207518	0.13825012	0.429431901	-1.21949873	down	C16535	-
MW0114088	cis-coumarinic acid-beta-D-glucoside	Phenolic acids	Phenolic acids	C15H18O8	2	0.8701	2446-60-8	43093.46	27796.15	40104.24	9536.3	4236.93	6435.88	1.69653291	0.01565409	0.24803772	5.492268091	2.457402049	up	C05839	ko00999,ko01110
MEDL01909	1,7-Bis(4-hydroxyphenyl)-3-heptanone	Phenolic acids	Phenolic acids	C19H22O3	2	0.8645	130233-83-9	17258.16	12574	17555.61	4787.84	4221.81	4497.61	1.74326784	0.01894872	0.2687821	3.508318489	1.810779723	up	-	-
MW0103346	2'-Deoxyguanosine-5'-diphosphate	Nucleotides and derivatives	Nucleotides and derivatives	C10H15N5O10P2	2	0.8583	3493-09-2	31365.35	23088.9	15933.45	793.01	1222.21	1991.05	1.71752844	0.03756601	0.32893629	17.56938499	4.134991786	up	C00361	ko00230,ko01100,ko01232
MW0114000	beta-L-fucose 1-phosphate	Organic acids	Organic acids	C6H13O8P	2	0.8569	16562-59-7	49.07	39.32	56.18	7.54	16.22	16.03	1.63422263	0.00690601	0.18870785	3.633324956	1.861290403	up	C02985	ko00051,ko00520,ko01100,ko01250
MW0011993	10-Deacetylbaccatin III	Benzene and substituted derivatives	Benzene and substituted derivatives	C29H36O10	2	0.8149	32981-86-5	29241.55	18658.83	21071.71	82777.73	39607.78	42310.14	1.46112836	0.14361205	0.53323181	0.418785135	-1.25571786	down	-	-
MW0108880	N-omega-Propyl-L-arginine	Amino acids and derivatives	Amino acids and derivatives	C9H20N4O2	2	0.8128	137361-05-8	1458.56	4394.69	343.45	8162.8	9998.91	6577.31	1.39982308	0.01797965	0.26056177	0.25048284	-1.99721632	down	-	-
MW0000289	(1S)-((2R,4S,5R)-5-Ethylquinuclidin-2-yl)(quinolin-4-yl)methanol	Alkaloids	Alkaloids	C19H24N2O	2	0.7927	485-65-4	1972.52	627.83	1393.17	86.21	740.19	81.03	1.34782321	0.10055355	0.47968762	4.400923487	2.13780629	up	-	-
MW0139619	(2S)-2-(2-hydroxypropan-2-yl)-9-[(2S,3R,4S,5S,6R)-3,4,5-trihydroxy-6-(hydroxymethyl)oxan-2-yl]oxy-2,3-dihydrofuro[3,2-g]chromen-7-one	Lignans and Coumarins	Coumarins	C20H24O10	2	0.784	20320-81-4	52.22	2785.48	321.55	4856.13	1160.59	2142.69	1.08805779	0.30489277	0.68276972	0.387190986	-1.36888273	down	C09309	-
MW0055509	Ostruthin	Lignans and Coumarins	Coumarins	C19H22O3	2	0.7826	148-83-4	71367.88	38802.67	43199.17	9667.22	6185.85	9499.75	1.71269492	0.05108257	0.36604803	6.049414621	2.596795545	up	C09281	-
MW0137975	Dihydromunduletone	Others	Ketone compounds	C25H28O6	2	0.7779	674786-20-0	33614.01	21814.3	26961.48	90645.15	65208.95	107461.91	1.65432	0.03149222	0.31251818	0.312893204	-1.67625777	down	-	-
MW0130165	5,7,3',4'-Tetrahydroxy-6,8-dimethoxyflavone	Flavonoids	Flavones	C17H14O8	2	0.7776	57093-50-2	441.36	560.28	274.94	13757.54	10870.57	1300.28	1.53739813	0.16069239	0.54886804	0.049234835	-4.34417677	down	-	-
MW0157439	Tetrahydrofolic acid	Organic acids	Organic acids	C19H23N7O6	2	0.7769	135-16-0	48195.62	51746.11	48902.78	19065.77	23591.97	25546.3	1.7146626	0.00092079	0.12524365	2.182341545	1.125876906	up	-	-
MW0000409	Pseudoyohimbine	Alkaloids	Plumerane	C21H26N2O3	2	0.776	84-37-7	3282.89	1259.54	2245.84	553.04	342.66	279.43	1.64862261	0.0823999	0.43733122	5.776611949	2.530223583	up	-	-
MW0005557	5-Aminoimidazole-4-carboxamide	Alcohol and amines	Amines	C4H6N4O	2	0.7747	360-97-4	189925.09	134024.75	176903.88	80513.14	59144.16	79032.81	1.68148647	0.01909585	0.26949041	2.290244035	1.195501332	up	C04051	ko00230,ko01100
MW0108619	N-Acetyl-D-phenylalanine	Amino acids and derivatives	Amino acids and derivatives	C11H13NO3	2	0.7747	10172-89-1	6628.21	8713.34	6559.14	20054.45	25238.13	15408.37	1.68800415	0.03828336	0.32974696	0.360796495	-1.47074277	down	C05620	ko00470,ko01100
MW0126537	Sempervirene	Alkaloids	Plumerane	C19H16N2	2	0.7728	549-92-8	36710.18	27760.5	37630.81	12925.56	22781.09	6025.9	1.37873397	0.0326391	0.31359786	2.446567248	1.290758938	up	C09240	-
MW0114515	Gentianose	Others	Saccharides	C18H32O16	2	0.7727	25954-44-3	39455.97	39431.84	72431.53	227.16	1112.97	696.47	1.71584866	0.04545263	0.35025258	74.29998036	6.215289924	up	C08239	-
MW0112809	3'-Fucosyllactose	Others	Saccharides	C18H32O15	2	0.7674	41312-47-4	272.19	359.96	381.52	883.54	562.93	685.04	1.59822188	0.0447639	0.34946131	0.475564271	-1.07228776	down	-	-
MW0170030	Zearalenone	Others	Ketone compounds	C18H22O5	2	0.7672	17924-92-4 | 36455-70-6	8718.54	7926.39	9947.98	4138.01	4199.1	4485.03	1.73979219	0.01375951	0.23753821	2.07398375	1.05240459	up	C09981	-
MW0114263	Digalacturonate	Others	Saccharides	C12H18O13	2	0.7663	5894-59-7	59.83	313.19	106.97	2253.8	611.83	1059.89	1.54741309	0.14058389	0.530528	0.122274246	-3.03180752	down	C02273	ko00040,ko01100,ko02010
MW0115078	Paederoside	Terpenoids	Monoterpenoids	C18H22O11S	2	0.7657	20547-45-9	4702.1	12396.91	6912.36	3866.8	5832.75	2049.44	1.11472907	0.20940304	0.60095435	2.043696522	1.03118098	up	C09795	-
MW0158681	Tyr-Ala	Amino acids and derivatives	Amino acids and derivatives	C12H16N2O4	2	0.765	730-08-5	11127.95	13241.2	12529.76	5090.89	6854.18	6156.66	1.68618403	0.00169573	0.13189913	2.03841898	1.027450616	up	-	-
MW0137266	Acteoside	Heterocyclic compounds	Heterocyclic compounds	C29H36O15	2	0.761	61276-17-3 | 22323-52-0	1026.36	388.79	805.32	62.75	459.41	408.36	1.08949344	0.13859899	0.52799149	2.386267893	1.254756016	up	-	-
MW0137905	Delphinidin 3-O-beta-D-sambubioside	Flavonoids	Anthocyanidins	C26H29O16+	2	0.7419	178275-92-8	13311.78	19866.64	9856.27	28735.12	26971.5	31831.81	1.56009656	0.02159673	0.27856654	0.491609114	-1.02441643	down	C20491	ko00942
MW0114697	Lacto-N-fucopentoase-3	Others	Saccharides	C32H55NO25	2	0.7405	25541-09-7	704.42	1431.37	1574.29	795.37	275.78	206.83	1.36478451	0.07629833	0.42295042	2.903081425	1.537585037	up	-	-
MW0148190	3,8-Dihydroxy-1-pentanoyl-6-pentyl-11H-dibenzo(b,e)(1,4)dioxepin-11-one	Organic acids	Organic acids	C23H26O6	2	0.7393	6320-33-8	8401.29	4218.37	5645.61	1427.7	1039.69	1000.83	1.70546792	0.05510041	0.37690654	5.266468102	2.396835759	up	-	-
MW0006095	Methyl 2,4-dihydroxy-3,6-dimethylbenzoate	Benzene and substituted derivatives	Benzene and substituted derivatives	C10H12O4	2	0.7381	4707-47-5 | 91061-32-4	644.69	1843.19	1911.22	2814.59	7056.74	2847.28	1.34856466	0.18063405	0.57170254	0.345878991	-1.53166071	down	-	-

MW0056741	Palmitoleoyl 3-carbacyclic Phosphatidic Acid	Lipids	Free fatty acids	C20H37O5P	2	0.7365	910228-13-6	14939.05	6330.73	3199.88	98972.21	68353.34	40058.93	1.60842976	0.06432185	0.39280702	0.117991761	-3.08324197	down	-	-
MW0125626	N-Acetyl-L-tryptophanamide	Alcohol and amines	Amines	C13H15N3O2	2	0.7333	2382-79-8	1663788.64	1948130.62	2339318.61	22402.34	33191.59	29030.99	1.75665923	0.00986451	0.212217	70.32488622	6.135963408	up	-	-
MW0108193	Met-Asn	Amino acids and derivatives	Amino acids and derivatives	C9H17N3O4S	2	0.7315	36261-61-7	59545.57	49266.54	82339.11	28742.81	25638.45	29008.49	1.6649818	0.06500947	0.394214	2.292262778	1.19677244	up	-	-
MW0107785	Mimosine	Amino acids and derivatives	Amino acids and derivatives	C8H10N2O4	2	0.7294	2116-55-4 | 10182-82-8 | 27678-82-6	15954.14	4973.25	15022.59	4928.17	6343.44	5853.68	1.14443689	0.21486557	0.60477561	2.099233356	1.069862549	up	-	-
MW0145991	L-Asparaginyl-L-phenylalanine	Amino acids and derivatives	Amino acids and derivatives	C13H17N3O4	2	0.7267	22849-01-0	20.3	25.37	23.36	0	7.29	4.73	1.10578356	0.00289286	0.14452248	5.742928453	2.521786588	up	-	-
MW0138348	Gyrophoric acid	Organic acids	Organic acids	C24H20O10	2	0.7222	548-89-0	1570.64	2089.53	3654.4	1513.52	304.38	1215.92	1.21729865	0.13735446	0.52739377	2.411009882	1.269637564	up	-	-
MW0012149	11-Oxoursolic acid acetate	Others	Lactones	C32H48O5	2	0.717	35959-01-4	7307.7	3229.82	21211.74	31130.76	11452.75	29987.41	1.05438613	0.18167669	0.57250504	0.437492869	-1.19266859	down	-	-
MEDP1608	cyclic ADP-ribose	Others	Saccharides	C15H21N5O13P2	2	0.7161	119340-53-3	2130.75	657.7	2470.61	211.13	200.84	460.94	1.55615881	0.11625231	0.49429284	6.024744819	2.590900137	up	-	-
MW0108303	Met-Ile	Amino acids and derivatives	Amino acids and derivatives	C11H22N2O3S	2	0.7104	40883-17-8	885.36	377.34	1476.86	8083.95	4190.99	2909.49	1.51450779	0.11117438	0.48968655	0.180419021	-2.47057665	down	-	-
MW0111319	Thiolutin	Heterocyclic compounds	Heterocyclic compounds	C8H8N2O2S2	2	0.7066	87-11-6	88463.8	62663.61	66365.05	31972.84	31396.15	40946.58	1.66234614	0.03007229	0.30510706	2.084947242	1.060010878	up	-	-
MW0115093	p-Coumaryl alcohol 4-O-glucoside	Phenolic acids	Phenolic acids	C15H20O7	2	0.7054	120442-73-1	42079.61	23040.21	36494.35	15954.97	4057.99	14138.74	1.42208745	0.03638242	0.32815889	2.975376628	1.573072299	up	C05855	ko00940,ko01110
MW0103676	Thymidine-5'-triphosphate	Nucleotides and derivatives	Nucleotides and derivatives	C10H17N2O14P3	2	0.7001	365-08-2	193112.22	205487.73	210287.8	34901.28	9424.73	180785.76	1.24816128	0.137544	0.52754608	2.704824141	1.435534798	up	C00459	ko00240,ko01100,ko01232
MEDP0171	Inosine	Nucleotides and derivatives	Nucleotides and derivatives	C10H12N4O5	3	0.9546	58-63-9	54809.15	22531.19	39314.24	34099.4	11271.14	7393.08	1.19611032	0.16455515	0.55155116	2.210890382	1.144627497	up	C00294	ko00230,ko01100,ko01232,ko02010
MEDL02380	Phthalic acid	Phenolic acids	Phenolic acids	C8H6O4	3	0.9467	88-99-3	61889.5	57366.71	63890.27	19307.48	30308.77	27311.44	1.67792526	0.00193086	0.13437613	2.380761466	1.251423081	up	C01606	ko01100,ko02010
MEDN0152	Adenosine 5'-Diphosphate（ADP）	Nucleotides and derivatives	Nucleotides and derivatives	C10H15N5O10P2	3	0.9409	58-64-0	71924.38	101498.99	102074.12	6336.6	42487.43	24600.2	1.40727039	0.00956802	0.20897133	3.752133185	1.907711039	up	C00008	ko00190,ko00195,ko00230,ko00908,ko01100,ko01110,ko01232,ko01240
MW0111506	(S)-Multifidol 2-[apiosyl-(1->6)-glucoside]	Benzene and substituted derivatives	Benzene and substituted derivatives	C22H32O13	3	0.9399	467437-62-3	59579.62	54210.25	45040.3	3604.73	32697.22	22675.1	1.2132422	0.04112706	0.33610993	2.693084344	1.429259414	up	-	-
MW0142490	Azotochelin	Benzene and substituted derivatives	Benzene and substituted derivatives	C20H22N2O8	3	0.9237	23369-85-9	408707.61	394644.2	439233.03	52898.59	124615.68	94996.21	1.67167124	0.00051077	0.11374907	4.559769004	2.18896074	up	C06446	-
MW0015984	Astragaloside IV	Terpenoids	Triterpene Saponin	C41H68O14	3	0.9125	84687-43-4 | 83207-58-3	1557.54	1236.63	4010.3	8994.33	7449.96	7360.94	1.53707828	0.00905465	0.2053328	0.285839288	-1.80672387	down	C17799	-
MEDL01794	Betulinic acid	Terpenoids	Triterpene	C30H48O3	3	0.9111	472-15-1	902.71	793.13	513.85	400.07	371.26	167.57	1.40397279	0.04573851	0.3512974	2.353488124	1.234800573	up	C08619	-
MEDL01725	N-AcetyTryptophan	Amino acids and derivatives	Amino acids and derivatives	C13H14N2O3	3	0.9051	1218-34-4	119105.83	83026.64	94328.27	206002.44	340588.75	217535.81	1.63407478	0.061094	0.38873242	0.387973125	-1.36597138	down	-	-
MW0009218	Benzenesulfonic acid, undecyl-	Benzene and substituted derivatives	Benzene and substituted derivatives	C17H28O3S	3	0.9045	50854-94-9	77424.41	52164.78	48276.82	13803.98	16997.98	52166.06	1.29385285	0.11354317	0.49245668	2.143789981	1.100163577	up	-	-
MW0147057	Cannabisin A	Lignans and Coumarins	Lignans	C34H30N2O8	3	0.8637	130508-46-2	3464.63	5504.6	3662.7	26838.76	8796.01	8457.15	1.43143712	0.225247	0.61967273	0.286490813	-1.80343922	down	C17905	-
MW0111507	(S)-Nerolidol 3-O-[a-L-Rhamnopyranosyl-(1->4)-a-L-rhamnopyranosyl-(1->2)-[4-(4-hydroxy-3-methoxycinnamoyl)-(E)-a-L-rhamnopyranosyl-(1->6)]-b-D-glucopyranoside]	Terpenoids	Terpene	C49H74O21	3	0.8568	143376-48-1	1610.17	798.77	622.82	4316.67	3270.19	2601.08	1.57527513	0.02205958	0.27856654	0.297583221	-1.74863491	down	-	-
MEDL02347	L-Phenylalanine	Amino acids and derivatives	Amino acids and derivatives	C9H11NO2	3	0.8567	63-91-2	2987.72	2863.06	231.51	5093.78	4419.73	3731.14	1.07312104	0.10116762	0.48076303	0.459226178	-1.12272321	down	C00079	ko00360,ko00400,ko00460,ko00470,ko00940,ko00960,ko00966,ko00970,ko00996,ko00999,ko01100,ko01110,ko01210,ko01230,ko02010
MW0015125	7,8-Dehydroastaxanthianthi[illegible]	Others	Ketone compounds	C40H50O4	3	0.8545	19866-02-5	1351.49	430.29	803.97	3095.11	4066.3	3995.14	1.59806191	0.00246276	0.14452248	0.231769678	-2.10923626	down	-	-
MW0114661	Italidipyrone	Others	Ketone compounds	C29H34O10	3	0.8521	75680-22-7	2435.4	2294.13	2778.78	13839.99	3566.76	4355.49	1.24764698	0.28656257	0.66816081	0.345015495	-1.53526694	down	-	-
MEDN0506	N-Acetylglucosamine-1-phosphate	Others	Saccharides	C8H16NO9P	3	0.8446	6866-69-9	16611.97	37732.29	20840.83	4011.36	10953.6	21566.18	1.08790966	0.19608368	0.5889486	2.058109602	1.041319813	up	C04256	-
MW0013503	2-Hydroxy-3-Methylbutanoic Acid	Organic acids	Organic acids	C5H10O3	3	0.8422	4026-18-0	23367.62	17117.87	7749.28	8722.56	7189.34	6230.59	1.23198948	0.1927671	0.58497036	2.178380571	1.12325602	up	-	-

MW0141403	[(1S,2R,3R,4S,5S,6S,8R,9S,10S,13S,16S,17R,18S)-11-ethyl-4,8,9-trihydroxy-6,16,18-trimethoxy-11-azahexacyclo[7.7.2.12,5.01,10.03,8.013,17]nonadecan-13-yl]methyl 2-[(3S)-3-methyl-2,5-dioxopyrrolidin-1-	Benzene and substituted derivatives	Benzene and substituted derivatives	C36H48N2O10	3	0.8413	119347-24-9	355.08	64.24	563.38	1067.3	524.99	857.94	1.11086972	0.08494243	0.44353291	0.40106439	-1.31809422	down	-	-
MW0123014	Azactam	Heterocyclic compounds	Heterocyclic compounds	C13H17N5O8S2	3	0.8387	78110-38-0	5345.45	3224.89	1390.28	145.78	667.38	1092.88	1.36830464	0.13660636	0.52588134	5.225818975	2.38565715	up	C06840	-
MW0124205	Gambogic acid	Organic acids	Organic acids	C38H44O8	3	0.8274	2752-65-0	4437.16	1885.11	2426.54	6070.33	12168.82	5080.73	1.40853027	0.14914302	0.5387718	0.37516531	-1.41440166	down	-	-
MW0120490	8-(4-Dibenzothienyl)-2-(4-morpholinyl)-4H-1-benzopyran-4-one	Benzene and substituted derivatives	Benzene and substituted derivatives	C25H19NO3S	3	0.8248	503468-95-9	1783.14	2921.32	2554.14	23469.49	7225.12	25808.16	1.59580412	0.10633374	0.48180112	0.128464498	-2.96055838	down	-	-
MW0143147	4-(3,5-Diphenylcyclohexyl)phenol	Benzene and substituted derivatives	Benzene and substituted derivatives	C24H24O	3	0.8212	33330-65-3	13699.7	10886.35	13644.05	2853.98	3472.29	1163.19	1.6286243	0.00128319	0.12524365	5.104520219	2.351775367	up	-	-
MW0108476	N-Phenylacetylphenylalani	Amino acids and derivatives	Amino acids and derivatives	C17H17NO3	3	0.8063	738-75-0	24578.14	19680.08	25542.63	402.77	1012.56	2434.94	1.67608103	0.00351422	0.14907003	18.12881954	4.180213082	up	-	-
MW0126778	Thieno[3,2-b][1]benzothiophene-2-carboxylic acid	Organic acids	Organic acids	C11H6O2S2	3	0.8063	30126-05-7 | 30376-45-5	19252.14	24447.78	45614.75	53632.16	170389.22	39046.54	1.19869494	0.29671754	0.67792725	0.339511826	-1.55846627	down	-	-
ZINC3918304	Tenuifoliside A	Phenolic acids	Phenolic acids	C31H38O17	3	0.8015	139726-35-5	2738.83	1680.91	4192.64	2193.1	1060	279.6	1.15256346	0.14313417	0.53323181	2.43790302	1.285640737	up	-	-
MEDN0236	Raffinose	Others	Saccharides	C18H32O16	3	0.7946	512-69-6	5632.62	5295.56	4621.61	560.15	3554.64	1795.67	1.29838247	0.053612	0.37368988	2.630893365	1.395552775	up	C00492	ko00052,ko01100,ko02010
MW0138304	Glyzaglabrin	Heterocyclic compounds	Heterocyclic compounds	C16H10O6	3	0.7913	65242-64-0	2626.98	1883.3	877.21	3100.43	5932.46	8636.19	1.42762324	0.11373449	0.49245668	0.304910612	-1.71354173	down	-	-
MW0146780	Bisibutiamine	Heterocyclic compounds	Heterocyclic compounds	C32H46N8O6S2	3	0.7864	3286-46-2	42025.29	35690.52	36590.22	7920.75	18347.54	22597.42	1.44951038	0.02314089	0.28084333	2.339186927	1.226007154	up	-	-
MEDL01891	3-Hydroxycinnamic acid	Phenolic acids	Phenolic acids	C9H8O3	3	0.7853	14755-02-3	504.57	466.5	379.3	515.55	2.54	37.47	1.13228731	0.24748362	0.63798289	2.430646555	1.281340123	up	C12621	ko00360,ko01100
MW0102906	cis,cis-Muconic acid	Organic acids	Organic acids	C6H6O4	3	0.7838	1119-72-8	15902.36	7895.53	15220.6	30762.48	27138.57	28804.14	1.52141193	0.01461094	0.24650433	0.450013315	-1.15196041	down	C02480	ko01100
MEDN1602	3'-Deoxyguanosine	Nucleotides and derivatives	Nucleotides and derivatives	C10H13N5O4	3	0.7801	3608-58-0	10120.73	21230.15	7374.23	344.22	326.81	162.53	1.71251566	0.09643904	0.47047039	46.45749556	5.537839478	up	-	-
MEDL00336	Uridine diphosphate glucuronic acid	Nucleotides and derivatives	Nucleotides and derivatives	C15H22N2O18P2	3	0.7799	2616-64-0	0.08	0.03	0.14	0	0	0	1.62009662	N/A	N/A	#DIV/0!	#DIV/0!	up	C00167	ko00040,ko00053,ko00520,ko01100,ko01240,ko01250
MW0011917	1,2-Dipalmitoyl-sn-glycero-3-phosphoglycerol	GL	PG	C38H75O10P	3	0.7722	74313-95-4	4198.37	6182.4	7141.13	31592.35	10215.9	6673.81	1.12863289	0.31557987	0.68542982	0.361409973	-1.46829178	down	-	-
MEDL01970	Corilagin	Tannins	Tannin	C27H22O18	3	0.7704	23094-69-1	814.26	1165.43	640.94	460.31	41.52	318.9	1.26030488	0.04088131	0.33610993	3.193047653	1.674934084	up	C10219	-
MEDL02393	N-Methylnicotinamide	Alkaloids	Pyridine alkaloids	C7H8N2O	3	0.7701	114-33-0	38710.45	22799.33	36424.75	16217.73	19492	7428.31	1.38860281	0.0458952	0.3512974	2.270259149	1.18285699	up	-	-
MEDL01834	Capsianoside I	Terpenoids	Ditepenoids	C32H52O14	3	0.7696	121924-04-7	60448.2	49057.61	47333.07	30372.04	22739.29	23376.96	1.67675954	0.00902733	0.2053328	2.050495311	1.035972445	up	-	-
MW0157039	Sesartemin	Lignans and Coumarins	Lignans	C23H26O8	3	0.7638	77394-27-5	111308.13	103118.27	89374.71	24803.79	1130.17	87510.07	1.11038534	0.1255925	0.50853545	2.67798235	1.421146452	up	-	-
MW0139812	Thalsimine	Heterocyclic compounds	Heterocyclic compounds	C38H40N2O7	3	0.7625	5525-36-0	124.58	104.46	73.18	302	212.18	241.68	1.63615921	0.0137326	0.23753821	0.399835948	-1.32251991	down	C09661	-
MEDN1223	N-acetylneuraminic acid 9-phosphate	Organic acids	Organic acids	C11H20NO12P	3	0.7624	37992-17-9	1720.06	5232.84	2187.94	232165.25	806556.2	537851.5	1.73873632	0.08777251	0.45299974	0.005797918	-7.43024946	down	C06241	ko00520,ko01100,ko01250
MW0122967	Aurasperone D	Others	Ketone compounds	C31H24O10	3	0.7554	67924-64-5	23965.34	44769.18	25395.65	4048.02	7099.78	18111.1	1.41938324	0.06346191	0.39280702	3.217146578	1.685781669	up	C08995	-
MW0141945	2,2-Dimethyl-3-(4-methoxyphenyl)-4-ethyl-6-(1-pyrrolidinylmethyl)-2H-1-benzopyran-7-ol	Benzene and substituted derivatives	Benzene and substituted derivatives	C25H31NO3	3	0.7516	16797-60-7	34310.49	71213.24	68853.58	30759.61	27381.28	13275.31	1.36796147	0.08530454	0.44490214	2.441705243	1.287889052	up	-	-
MW0147078	Caracurine V	Alkaloids	Alkaloids	C38H40N4O2	3	0.7495	630-87-5	7087.81	7104.66	7808.79	5370.49	592.3	490.13	1.3582704	0.08143103	0.43540672	3.409504534	1.769562104	up	C09100	-
MW0143673	5-Nitrosalicylic acid	Benzene and substituted derivatives	Benzene and substituted derivatives	C7H5NO5	3	0.7481	96-97-9	11208.04	18906.95	18756.13	3094.15	7808.79	3832.41	1.54244678	0.02706603	0.29249362	3.316590376	1.729700839	up	C19789	-
MW0145019	Amataine	Alkaloids	Alkaloids	C43H48N4O6	3	0.7439	31148-60-4	1599.68	931.6	761.82	3243.32	2676.73	1188.01	1.23819579	0.16244192	0.54904711	0.463290968	-1.11000954	down	C08433	-
MW0146543	Aconitan-14-one, 7,8-dihydroxy-20-ethyl-4-(((2-(3-methyl-2,5-dioxo-1-pyrrolidinyl)benzoyl)oxy)methyl)-1,6,16-trimethoxy-, (1-alpha,4(S),6-beta,16-	Benzene and substituted derivatives	Benzene and substituted derivatives	C36H46N2O10	3	0.7416	123497-99-4	14297.34	12171.07	9563.93	3287.91	3208.87	3859.68	1.72303438	0.02260667	0.27962521	3.47921394	1.798761394	up	C08662	-
MW0148876	Elatin	Benzene and substituted derivatives	Benzene and substituted derivatives	C38H50N2O10	3	0.7415	26000-16-8	1454.59	10908.01	13248.41	22479.02	25074.61	19576.56	1.21065587	0.04487779	0.34946131	0.38151255	-1.39019758	down	C08681	-
MEDN0442	Pantetheine	Alkaloids	Alkaloids	C11H22N2O4S	3	0.7384	496-65-1	2967.61	733.58	5879.59	93997.17	4442.6	41656.72	1.26164193	0.23564967	0.63038056	0.06838701	-3.87013389	down	C00831	ko00770,ko01100,ko01110
MW0151872	3H-isoindol-1-yl (3S,4S,5R)-3,4,5-trihydroxy-5-(hydroxymethyl)tetrahydrofuran-2-carboxylate	Alkaloids	Plumerane	C14H15NO7	3	0.7369	20307-14-6	1236581.91	755390.03	986294.07	84028.41	236125.4	407445.83	1.50932364	0.01482375	0.24697482	4.093275816	2.033255883	up	C08494	-

MW0006469	Candesartan	Benzene and substituted derivatives	Benzene and substituted derivatives	C24H20N6O3	3	0.7326	139481-59-7	10494.55	4344.81	7108.41	247.27	3054.9	4819.07	1.08955284	0.11216889	0.49150111	2.702514641	1.434302434	up	C07468	-
MW0110838	N-Cyclohexylhydrazinecarbothioamide	Alcohol and amines	Polyamines	C7H15N3S	3	0.7297	21198-18-5	8462.99	6142.38	8281.9	26227.24	27359.86	19852.06	1.70608144	0.01241351	0.22752996	0.311649398	-1.68200417	down	-	-
MEDN1045	Xanthopterin	Heterocyclic compounds	Pteridines and derivatives	C6H5N5O2	3	0.7239	119-44-8 | 5979-01-1 | 46176-54-9	1067706.54	1551361.67	1237103.2	6235.53	234671.87	1096324.69	1.07841441	0.11196577	0.49132565	2.883696434	1.527919301	up	-	-
MW0054489	1-(9Z,12Z-octadecadienoyl)-glycero-3-phosphate	GL	PA	C21H39O7P	3	0.7234	65528-85-0	18121.06	14339.65	11618.19	5025.01	520.4	1109.52	1.529313	0.00753369	0.19469501	6.623495664	2.727592825	up	-	-
MEDL01815	Verbasoside	Phenolic acids	Phenolic acids	C20H30O12	3	0.7226	61548-34-3	788.4	11716.41	2039.96	39064.87	18619.5	18287.64	1.42808652	0.07754798	0.42641815	0.191449061	-2.38496751	down	-	-
MEDN1197	8-Methoxyalkynurate	Organic acids	Organic acids	C11H9NO4	3	0.718	2929-14-8	300.41	427.73	63.07	4768.84	580.58	537.84	1.22069182	0.3496049	0.70682716	0.134393589	-2.89546378	down	C05830	ko00380
MEDL01730	Methotrexate	Nucleotides and derivatives	Nucleotides and derivatives	C20H22N8O5	3	0.7137	59-05-2	827.18	8077.53	7051.76	13976.14	14690.29	15324.3	1.22522785	0.0503604	0.36472395	0.362723465	-1.46305802	down	C01937	-
MW0141698	1-{[3-(3,4-Dihydroxyphenyl)acryloyl]oxy}-3,5-dihydroxycyclohexane-1-carboxylic acid	Organic acids	Organic acids	C16H18O8	3	0.71	153444-59-8	69533.67	50118.11	47171.94	54196.29	7747.89	17062.71	1.19994356	0.16392874	0.55155116	2.111508503	1.078274057	up	-	-
MW0009240	Triton X-100	Benzene and substituted derivatives	Benzene and substituted derivatives	C16H26O2	3	0.7096	2315-67-5 | 9002-93-1 | 63869-93-2	2122.53	1314.07	1953.85	316.96	919.63	556.72	1.52106719	0.02018245	0.2743902	3.005866247	1.587780815	up	-	-
MW0146965	Calafatimine	Lignans and Coumarins	Lignans	C38H40N2O7	3	0.7051	77793-42-1	1905.89	2309.56	3342.11	1409.27	995.28	447.44	1.42726225	0.04544596	0.35025258	2.64992514	1.405951604	up	C09369	-
MW0149004	Esmeraldic acid	Organic acids	Organic acids	C30H22N4O5	3	0.7031	119936-20-8	17940.45	16690.02	3687.98	4561.48	2910.59	726.3	1.24607019	0.15200726	0.54090463	4.67391079	2.224630198	up	C12116	-
MEDP1552	N-Acetylthreonine	Amino acids and derivatives	Amino acids and derivatives	C6H11NO4	3	0.7022	17093-74-2	6735.99	25543.43	20381.08	47185.18	59276.11	38450.77	1.42638579	0.02047454	0.27467954	0.363396256	-1.46038454	down	-	-
MW0139172	Novobiocin	Heterocyclic compounds	Heterocyclic compounds	C31H36N2O11	3	0.7021	303-81-1	5619.28	5593.8	4051.03	10177.23	11338.85	12775.09	1.69010198	0.00345604	0.14907003	0.445132377	-1.16769366	down	C05080	ko01100,ko01110
MW0146885	Bruceantinol	Alcohol and [illegible]	Alcohols	C30H38O13	3	0.7013	53729-52-5	9261.74	8916.9	12085.81	151.42	6194.27	4813.14	1.04911903	0.05283113	0.37232569	2.712152618	1.439438364	up	C08750	-
MEDL02309	Inosinic acid	Nucleotides and derivatives	Nucleotides and derivatives	C10H13N4O8P	3	0.6998	131-99-7 | 30918-54-8 | 25249-22-3	5728.51	4289.26	4439.21	110.56	83.63	3373.71	1.34438976	0.06345129	0.39280702	4.051957734	2.018619126	up	C00130	ko00230,ko01100,ko01110,ko01232,ko01240
MW0146375	Aspulvinone G	Phenolic acids	Phenolic acids	C17H12O6	3	0.6994	55215-40-2	12688.82	14708.61	14126.43	7382.41	108.68	4571.79	1.14492014	0.03533527	0.32593586	3.442284098	1.783366171	up	C02007	-
MW0012635	17-Hydroxypregnenolone [illegible]	Others	Lactones	C21H32O6S	3	0.6951	2477-77-2	1274.07	2175.8	2134.66	16160.94	2981.09	3526.28	1.2339265	0.31673918	0.68542982	0.246358463	-2.02116907	down	-	-
MW0148256	Demethylalangiside	Alkaloids	Alkaloids	C24H29NO10	3	0.6949	47763-23-5	7094.16	1466.65	4539.99	28877.57	12535.65	8787.97	1.32742859	0.17636983	0.56924001	0.260965925	-1.93806665	down	C11813	ko00950
MEDP1243	4-Hydroxytryptamine	Others	Tryptamines	C10H12N2O	3	0.6933	570-14-9	668.62	1069.56	2766.12	268.29	633.03	923.53	1.0808033	0.29733768	0.67810652	2.468312464	1.303525037	up	C21762	ko01100,ko01110
MW0103327	2-Aminoadenosine	Nucleotides and derivatives	Nucleotides and derivatives	C10H14N6O4	3	0.6926	2096-10-8	109151.35	83467.69	104003.73	37046.12	52975.16	45500.79	1.67339431	0.00785561	0.19656685	2.188741435	1.130101533	up	C00939	-
MW0152116	Lankamycin	Heterocyclic compounds	Heterocyclic compounds	C42H72O16	3	0.6917	30042-37-6	69349.84	100740.2	69247.01	14186.35	46697.28	47045.85	1.28381104	0.04431518	0.3493879	2.217531762	1.148954769	up	-	-
MEDN0074	gamma-Glutamylcysteine	Amino acids and derivatives	Amino acids and derivatives	C8H14N2O5S	3	0.6906	636-58-8	517.48	835.21	899.05	469.43	403.87	111.49	1.22399525	0.05923713	0.38573418	2.286517938	1.193152237	up	C00669	ko00480,ko01100,ko01240
MW0156169	Pseudaconitine	Terpenoids	Ditepenoids	C36H51NO12	3	0.6886	127-29-7	2000.47	2144.26	36971.73	11645.61	54249.85	46212.2	1.08538437	0.24852069	0.63798289	0.366758703	-1.44709689	down	-	-
MW0138375	(S)-7-(((2-O-6-Deoxy-alpha-L-mannopyranosyl)-beta-D-glucopyranosyl)oxy)-2,3-dihydro-5-hydroxy-2-(3-hydroxy-4-methoxyphenyl)-4H-1-benzopyran-4-one	Flavonoids	Flavanones	C28H34O15	3	0.6883	13241-33-3	25030.68	21967.57	8817.16	8882.78	7523.28	725.91	1.13685303	0.10483745	0.48180112	3.257967998	1.703972433	up	C09806	ko00941
MW0103313	1-Methylguanosine	Nucleotides and derivatives	Nucleotides and derivatives	C11H15N5O5	3	0.688	2140-65-0	16787.56	14285.43	15166.79	11652.51	2543.78	8633.59	1.23747341	0.0913614	0.4592818	2.025406178	1.018211257	up	C04545	-
MW0121803	6,6'-(1,2-phenylene)bis(1,3,5-triazine-2,4-diamine)	Benzene and substituted derivatives	Benzene and substituted derivatives	C12H12N10	3	0.6852	5118-79-6	565.87	626.28	602.25	1696.64	1696.56	1812.91	1.758199	0.00019167	0.07934638	0.344671934	-1.53670427	down	-	-
MW0153811	Methyl farnesoate	Lipids	Others	C16H26O2	3	0.6834	10485-70-8 | 3675-00-1 | 4176-79-8	2860.67	1632.36	2222.33	529.74	860.46	622.92	1.66099153	0.03941531	0.33249206	3.335797171	1.73803157	up	-	-
ZINC2169825	5-[(3aR,4R,6aS)-2-oxo-1,3,3a,4,6,6a-hexahydrothieno[3,4-d]imidazol-4-yl]pentanoic acid	Organic acids	Organic acids	C10H16N2O3S	3	0.6823	21788-37-4	230.98	719.71	1232.18	5155.49	567.29	2802.21	1.03312201	0.24865515	0.63798289	0.256055432	-1.96547193	down	-	-
MW0153432	Megaphone	Alcohol and [illegible]	Alcohols	C22H30O6	3	0.6817	64332-37-2	14036.78	14748.39	5504.33	800.19	9729.46	3113.97	1.08034731	0.16101414	0.54904711	2.513225962	1.329540389	up	-	-
MW0002360	2,6-diamino-4-(4-methoxyphenyl)-4H-thiopyran-3,5-[illegible]	Phenolic acids	Phenolic acids	C14H12N4OS	3	0.6813	102423-74-5	4415.07	4523.82	3350.79	797.67	477.66	1018.77	1.70009835	0.00564407	0.17971833	5.357081208	2.421447166	up	-	-
MW0063676	Stevioside	Terpenoids	Ditepenoids	C38H60O18	3	0.6779	57817-89-7	8714.3	32365.41	34805.01	45581.55	72863.44	58134.33	1.29334864	0.04303828	0.34331994	0.429748625	-1.21843507	down	C09189	ko00904,ko01110
MW0125969	Oleoyl oxazolopyridine	Heterocyclic compounds	Heterocyclic compounds	C24H36N2O2	3	0.6765	288862-58-8	10615.67	11259.47	8314.6	8874.89	2043.78	2988.43	1.29781361	0.11137236	0.48968655	2.170814907	1.118236721	up	-	-

MEDN1332	Adenosine-5'-Diphosphoglucose	Nucleotides and derivatives	Nucleotides and derivatives	C16H25N5O15P2	3	0.6756	102129-65-7	1727.55	6246.72	1712.02	7886.66	5660.73	7202.26	1.32214314	0.11955878	0.4975454	0.466817031	-1.0990709	down	-	-
MW0154503	Picras-2-ene-1,16-dione, 11-(acetyloxy)-13-hydroxy-2,12-dimethoxy-, (11alpha,12beta)	Terpenoids	Ditepenoids	C24H34O8	3	0.6744	28360-79-4	23.28	6.05	7.84	32.59	28.35	21.36	1.29854925	0.09221728	0.46085323	0.45164034	-1.14675374	down	-	-
MEDN1417	13-Hpode	Lipids	Free fatty acids	C18H32O4	3	0.6736	23017-93-8	41468.7	70188.74	46770.78	746825.76	74420.03	134099.13	1.20117717	0.34181948	0.70329636	0.16583353	-2.59219236	down	C04717	ko00591,ko01100
MW0000452	Strychnine	Alkaloids	Alkaloids	C21H22N2O2	3	0.6727	57-24-9	20479.25	28760.63	27205.59	59211.41	51852.01	50449.35	1.6895974	0.00161982	0.13083964	0.473309138	-1.07914532	down	C06522	ko01110
MW0114190	Deoxyribose 5-phosphate	Nucleotides and derivatives	Nucleotides and derivatives	C5H11O7P	3	0.6704	102916-66-5	1546.5	2043.8	1535.34	4609.16	2948.96	4333.92	1.63300562	0.0376195	0.32893629	0.431014359	-1.21419216	down	C00673	ko00030,ko01100
MW0115136	Photobarbatusin I	Others	Lactones	C24H30O8	3	0.6691	62288-03-3	15853.73	20502.65	5318.92	21211.96	49720.09	26692.33	1.25759828	0.15413713	0.5435683	0.426894388	-1.2280489	down	-	-
MW0156216	2'-Deoxynebularine	Nucleotides and derivatives	Nucleotides and derivatives	C10H12N4O3	3	0.6668	4546-68-3	257.43	2024.71	1716.94	2060.45	4766.27	3006.36	1.14406803	0.12230698	0.50268793	0.406696579	-1.29797524	down	C20463	-
MW0148881	Eleganin	Heterocyclic compounds	Heterocyclic compounds	C22H26O9	3	0.6651	57498-84-7	199698.56	167015.49	184683.28	77462.91	86744.09	74942.53	1.73981757	0.00373091	0.15364676	2.305659267	1.205179325	up	C09401	-
MEDL02244	Podofilox	Lignans and Coumarins	Lignans	C22H22O8	3	0.6643	477-47-4	2584.18	7192.02	10286.52	105786.25	123602.66	3739.02	1.08832989	0.19712856	0.58909363	0.086058843	-3.53853274	down	C10874	ko00999,ko01100,ko01110
MW0001859	4,5-Dihydro-2-(3-nitrophenyl)-1H-imidazole	Benzene and substituted derivatives	Benzene and substituted derivatives	C9H9N3O2	3	0.6633	31659-42-4	4510.92	893.57	5826.6	799.61	686.42	1882.12	1.13394588	0.21256032	0.60263758	3.334498167	1.737469656	up	-	-
MW0118099	2-(5-Amino-[1,3,4]thiadiazol-2-ylsulfanyl)-N-(1,5-dimethyl-3-oxo-2-phenyl-2,3-dihydro-1H-pyrazol-4-yl)-acetamide	Alcohol and amines	Amines	C15H16N6O2S2	3	0.6586	329921-17-7	2369.47	3610.5	8719.6	2610.28	2341.13	1021.86	1.14566458	0.27034847	0.65233913	2.460891605	1.299181113	up	-	-
MW0151672	Ile-Leu	Amino acids and derivatives	Amino acids and derivatives	C12H24N2O3	3	0.6554	26462-22-6	2350.33	1141.06	2172.75	744.91	197.49	696.38	1.45866726	0.05257207	0.37108719	3.456315064	1.789234734	up	-	-
MEDP1679	Glu-Val-Phe	Amino acids and derivatives	Amino acids and derivatives	C19H27N3O6	3	0.6549	31461-61-7	120.84	166.42	34.9	2276.99	3097.63	1455.36	1.69088997	0.04377193	0.34812392	0.047168513	-4.40603207	down	-	-
MW0169896	TAXININE M	Others	Others	C35H44O14	3	0.6545	135730-55-1	8660.92	7127.55	6289.86	924.67	698.64	387.57	1.71565763	0.00810886	0.19760292	10.97943686	3.456732155	up	-	-
MW0003469	Phenol, 3-(3-phenoxyphenoxy)-	Benzene and substituted derivatives	Benzene and substituted derivatives	C18H14O3	3	0.654	14200-84-1	83786.95	78049.34	97184.54	30133.75	17119.67	17787.86	1.70622234	0.00113642	0.12524365	3.982406712	1.993640567	up	-	-
MEDN1244	4-(Cytidine 5'-diphosphate) -2-C-methyl-D-erythritol	Nucleotides and derivatives	Nucleotides and derivatives	C14H25N3O14P2	3	0.6517	263016-94-0	160535.18	203648.67	164836.67	14581.22	18874.53	15341.68	1.74880721	0.00684719	0.18870785	10.84115536	3.438446611	up	C11435	ko00900,ko01100,ko01110
MW0103715	Zeatin riboside	Nucleotides and derivatives	Nucleotides and derivatives	C15H21N5O5	3	0.6505	6025-53-2 | 28542-78-1	448.99	3363.48	1381.56	17181.94	12063.71	8750.86	1.56509775	0.03524411	0.32593586	0.136697555	-2.87094066	down	-	-
MW0123298	Chelidonic acid	Organic acids	Organic acids	C7H4O6	3	0.646	99-32-1	11357.64	4685.28	17358.34	6187.53	3695.66	2369.12	1.27012991	0.18662047	0.57922127	2.726119401	1.446848752	up	C08476	-
MW0154115	N,N'-Diethylthiourea	Others	Others	C5H12N2S	3	0.6452	105-55-5	4118.89	3252.22	3951.24	2784.36	656.15	1451.04	1.36578857	0.05789575	0.38089756	2.314675307	1.210809833	up	C19400	-
MEDP1467	5-Methyl-2'-deoxycytidine	Nucleotides and derivatives	Nucleotides and derivatives	C10H15N3O4	3	0.642	838-07-3	2228.64	713.3	484	43.05	138.47	133.13	1.55983967	0.19825955	0.5893935	10.88809789	3.444680037	up	C03592	-
MW0153825	Methyl-2-alpha-L-fucopyranosyl-beta-D-galactoside	Others	Saccharides	C13H24O10	3	0.642	37288-45-2	817621.82	661516.74	581291.27	44618.2	8843.74	5068.04	1.67239169	0.00890588	0.2053328	35.20298196	5.137625736	up	C04698	-
MW0146616	(2r,3s)-3-Phenylisoserine ethyl ester	Benzene and substituted derivatives	Benzene and substituted derivatives	C11H15NO3	3	0.6417	143615-00-3 | 153433-80-8	57789.22	11568.75	52754.51	16755.83	3643.07	13659.74	1.21177876	0.17617537	0.56921854	3.585359838	1.84211792	up	-	-
MEDL02157	Coniferin	Phenolic acids	Phenolic acids	C16H22O8	3	0.6407	124151-33-3	3788.78	9155.49	1727.94	49109.1	8948.48	10012.84	1.25095457	0.30932138	0.68402525	0.215544579	-2.21394182	down	C00761	ko00940,ko01110
MW0157166	Spinoside A	Terpenoids	Triterpene	C39H56O12	3	0.6386	119626-74-3	27462.42	16665.99	27475.81	1836.17	16721.17	15613.56	1.04125978	0.1110279	0.48968655	2.095473634	1.067276369	up	C08809	-
MW0127096	Tribufos	Others	Lactones	C12H27OPS3	3	0.636	78-48-8	1082.83	11089.02	8972.6	55041.23	8406.54	11752.12	1.00941462	0.35277914	0.7090606	0.281176608	-1.83045152	down	-	-
MW0141199	2-(p-Hydroxyphenyl)-3-benzofuranyl methyl ketone	Benzene and substituted derivatives	Benzene and substituted derivatives	C16H12O3	3	0.6315	2726-47-8	156334.15	37340.29	61991.83	215561.67	186458.36	205490.34	1.34751252	0.07670481	0.42295042	0.420842643	-1.2486472	down	-	-
MEDL02722	Polydatin	Others	Stilbene	C20H22O8	3	0.6282	27208-80-6	2130.4	18918.45	5181.99	79120.24	49987.3	46458.04	1.5440562	0.02408886	0.28436137	0.149407646	-2.74267412	down	C10275	-
MW0007080	Ethyl 4-hydroxy-3,5-dimethoxybenzoate	Benzene and substituted derivatives	Benzene and substituted derivatives	C11H14O5	3	0.6247	3943-80-4	7151.51	6229.53	6107.77	1666.81	4264.95	3582.69	1.40645235	0.03541113	0.32593586	2.048338054	1.034453835	up	-	-
MW0154695	9-Octadecenedioic acid, (9Z)-	Lipids	Free fatty acids	C18H32O4	3	0.6235	20701-68-2 | 4494-16-0	7250.19	12964.13	9868.42	112980.06	10400.02	22789.77	1.07122002	0.35389062	0.71033295	0.205806738	-2.28063788	down	C19618	ko00073
MEDN1475	D-Sorbitol-6-phosphate	Others	Saccharides	C6H15O9P	3	0.6233	20479-58-7	161374.02	111463.95	155647.35	30835.86	32828.65	58620.23	1.66961294	0.00957121	0.20897133	3.503996656	1.809001398	up	C01096	ko00051,ko01100
ZINC582286[illegible]	Philonotisflavone	Flavonoids	Flavones	C30H18O12	3	0.621	124615-12-9	2775.29	18608.05	12921.16	1960.99	2720.89	2823.63	1.26543444	0.19311052	0.58501187	4.570575484	2.192375827	up	-	-
MW0105034	3-Hydroxyadipic acid	Organic acids	Organic acids	C6H10O5	3	0.619	14292-29-6	2384.7	5245.9	4364.19	5160.59	17915.72	12814.19	1.35497937	0.15903676	0.54886804	0.334205152	-1.58119412	down	-	-
MEDN1574	Pyritinol	Heterocyclic compounds	Heterocyclic compounds	C16H20N2O4S2	3	0.6182	1098-97-1	762.56	7834.57	546.56	1953.19	11988.92	6925.24	1.0113804	0.35908724	0.71458744	0.438181657	-1.190399	down	C71346	-
MW0000482	Leurocristine	Alkaloids	Alkaloids	C46H56N4O10	3	0.6153	57-22-7 | 132142-73-5	21034.1	17991.24	10733.62	53212.89	43293.53	44771.55	1.64618249	0.00216917	0.13948645	0.35220608	-1.50550828	down	-	-
MW0109976	Threonyl-valine	Amino acids and derivatives	Amino acids and derivatives	C9H18N2O4	3	0.6136	99032-17-4	109483.71	227706.39	184764.1	4000.92	7384.32	12040	1.70863743	0.04006762	0.33487182	22.28170128	4.477787486	up	-	-
MW0159659	Cellotriose	Others	Saccharides	C18H32O16	3	0.6109	9061-30-7	113928.91	146187.37	73437.35	72945.99	31029.37	4094.2	1.17709112	0.06103336	0.38873242	3.086471621	1.625958526	up	C01898	ko00500,ko01100
MW0157091	S-Octylglutathione	Amino acids and derivatives	Amino acids and derivatives	C18H33N3O6S	3	0.606	24435-27-6	60774.93	1562.69	509.77	74052.55	53671.29	62908.74	1.23238133	0.15754642	0.54735706	0.329678117	-1.60086997	down	-	-

MW0149199	(4aS,6aR,6aS,6bR,8aR,10S,12aR,14bS)-10-[(2S,3S,5R)-5-hydroxy-4-oxo-3-[(2S,3R,4S,5S,6R)-3,4,5-trihydroxy-6-(hydroxymethyl)oxan-2-yl]oxyoxan-2-yl]oxy-2,2,6a,6b,9,9,12a-heptamethyl-1,3,4,5,6,6a,7,8,8a,10,11,12,13,14b-tetradecahydropicene-4a-carboxylic acid	Heterocyclic compounds	Heterocyclic compounds	C41H64O12	3	0.6048	117585-05-4	547.5	971.76	562.53	2453.97	2252.32	2056.55	1.69414906	0.00114511	0.12524365	0.307827776	-1.69980468	down	C08943	-
mws5038	Isomaltulose	Others	Saccharides	C12H22O11	3	0.6041	58166-27-1 | 15132-06-6	27084.89	29742.23	34713.26	3658.24	16431.23	24226.08	1.13899443	0.10602076	0.48180112	2.065649191	1.046595262	up	C01742	-
MW0133966	5,6,7-Trimethoxycoumarin	Lignans and Coumarins	Coumarins	C12H12O5	3	0.6032	55085-47-7	49780.11	57943.08	19103.81	3580.32	1293.43	1763.62	1.67577841	0.07664564	0.42295042	19.10802019	4.256106402	up	C09313	-
MW0157047	Shikimate-3-phosphate	Organic acids	Organic acids	C7H11O8P	3	0.6029	63959-45-5	3808.39	858.2	4634.36	7643.6	14674.02	11169.99	1.37131818	0.03755226	0.32893629	0.277743022	-1.84817743	down	C03175	ko00400,ko01100,ko01110,ko01230
MEDP1367	Ala-Tyr	Amino acids and derivatives	Amino acids and derivatives	C12H16N2O4	3	0.6026	3061-88-9	4090894.56	1776227.09	6272522.34	50348.75	3030079.84	1233199.38	1.01430171	0.18034841	0.57165026	2.814253819	1.492752452	up	-	-
MW0147482	Cilazaprilat	Organic acids	Organic acids	C20H27N3O5	3	0.6025	90139-06-3	169.32	182.75	722.5	67.03	19.87	69.48	1.48252735	0.23432305	0.62985809	6.871530886	2.780631548	up	C17309	-
MW0137554	Cajanin	Flavonoids	Isoflavones	C16H12O6	3	0.5986	32884-36-9	411.24	2169.16	715.87	3120.53	3431.25	3210.24	1.44625025	0.05419611	0.37658263	0.337662697	-1.56634529	down	C10203	-
MEDL00815	Cytidine-5'-diphosphate	Nucleotides and derivatives	Nucleotides and derivatives	C9H15N3O11P2	3	0.5974	63-38-7	878.85	6416.95	6257.65	15138.81	13456.81	13400.5	1.31323157	0.0262699	0.29249362	0.322731005	-1.63159591	down	C00112	ko00240,ko01100,ko01232,ko01240
MW0143316	5-Hydroxy-1-(4-hydroxy-3-methoxyphenyl)octan-3-[illegible]	Phenolic acids	Phenolic acids	C15H22O4	3	0.5945	77398-90-4	12861.01	8939.3	3957.81	16367.46	22806.67	21458.22	1.41926492	0.02575653	0.29249362	0.424824702	-1.23506044	down	-	-
MW0015654	All-trans-heptaprenyl diphosphate	Organic acids	Organic acids	C35H60O7P2	3	0.5945	76078-18-7	233.85	373.11	215.07	3636.6	3242.28	6021.82	1.73419113	0.04308843	0.34331994	0.063719798	-3.97211449	down	C04216	ko00900,ko01110
MW0145631	Artecanin	Others	Lactones	C15H18O5	3	0.5929	29431-84-3	896234.46	778437.99	913891.75	21630.44	20856.87	18353.27	1.7612599	0.00252494	0.14452248	42.54667197	5.410974383	up	C09302	-
MW0144464	Ala-Ala-Tyr	Amino acids and derivatives	Amino acids and derivatives	C15H21N3O5	3	0.5885	67131-52-6	7071.91	5300.58	9494.02	4878.41	1962.03	2984.75	1.44345958	0.06067321	0.38873242	2.225555943	1.154165766	up	-	-
MW0012238	12-Methyltridecanoic [illegible]	Lipids	Free fatty acids	C14H28O2	3	0.5879	2724-57-4	1264.76	1207.89	1006.86	689.05	464.52	330.81	1.5679607	0.00858131	0.20222829	2.344083052	1.229023686	up	-	-
MW0108775	N-Feruloylglycyl-L-phenylalanine	Amino acids and derivatives	Amino acids and derivatives	C21H22N2O6	3	0.5846	40747-82-8	8164.73	3628.35	47237.56	274542.63	270139.35	21492.06	1.25720732	0.17761818	0.56955461	0.104262357	-3.26170971	down	-	-
MEDP0617	N-(2-Furoyl)glycine	Amino acids and derivatives	Amino acids and derivatives	C7H7NO4	3	0.5693	5657-19-2	1847.02	5540.19	3515.06	517.83	2747.92	907.5	1.17894805	0.16428206	0.55155116	2.612417181	1.385385301	up	-	-
MEDL01787	N-trans-Feruloyloctopamine	Alkaloids	Phenolamine	C18H19NO5	3	0.5691	66648-44-0	84989.53	83704.33	86908.82	451882.96	119547.54	111258.84	1.11908273	0.33213934	0.69681989	0.374405553	-1.41732626	down	-	-
MW0141949	2,2-Dimethyl-3,4-bis(4-methoxyphenyl)-2H-1-benzopyran-7-ol acetate	Benzene and substituted derivatives	Benzene and substituted derivatives	C27H26O5	3	0.5653	5867-70-9	40402.2	33284.92	43627.67	20402.86	14754.15	11022.4	1.63673314	0.00459859	0.16605573	2.540413357	1.345063261	up	-	-
MW0126863	Trichodimerol	Alcohol and [illegible]	Alcohols	C28H32O8	3	0.5608	145174-90-9	8410.93	12776.91	18004.55	11205.79	3986.03	1698.64	1.18852135	0.1357793	0.52451084	2.320386182	1.214364933	up	-	-
MW0111826	1-Kestose	Others	Saccharides	C18H32O16	3	0.5597	470-69-9	1054.84	579.46	348.64	4023.54	882.2	914.82	1.06729173	0.34388073	0.70386281	0.34067856	-1.55351694	down	C03661	-
MW0129713	Tetrahydrobisdemethoxydiferuloylmethane	Others	Ketone compounds	C19H20O4	3	0.5489	113482-94-3	68.57	77.3	77.39	162.57	171.09	158.25	1.75341126	7.3654E-05	0.0656108	0.453863512	-1.13966959	down	-	-
MW0008791	2-(acetylsulfamoyl)benzoic Acid	Benzene and substituted derivatives	Benzene and substituted derivatives	C9H9NO5S	3	0.5417	849067-18-1	20313.09	16183.65	20898.39	4968.25	7224.55	9843.12	1.62812222	0.00454057	0.16605573	2.604616916	1.381071198	up	-	-
MW0158037	Trichilin A	Terpenoids	Triterpene	C35H46O13	3	0.5319	77182-69-5	10348.71	3923.33	5197.41	15158.28	12033.73	11920.47	1.37819089	0.05902072	0.38573418	0.497781015	-1.00641689	down	C08786	-
MW0000319	Levallorphan	Alkaloids	Isoquinoline alkaloids	C19H25NO	3	0.5308	152-02-3	12497.4	18384.96	22499.5	8713.87	7947	6848.89	1.59449433	0.07085543	0.41039021	2.270625476	1.183089763	up	C07069	-
MEDTN0035[illegible]	Proglumide	Alcohol and amines	Amines	C18H26N2O4	3	0.5265	6620-60-6 | 24485-90-3	3381.42	3097.67	3426.79	8293.74	7843.01	6978.5	1.74218386	0.00499512	0.16983407	0.428543061	-1.22248792	down	-	-
MW0103678	trans-Zeatin-O-glucoside riboside	Nucleotides and derivatives	Nucleotides and derivatives	C21H31N5O10	3	0.5262	62512-97-4	1872.57	3871.45	3908.48	12659.66	5846.92	5156.15	1.34092095	0.1836953	0.57375796	0.407919965	-1.29364197	down	-	-
MEDN0835	D-Glucuronolactone	Others	Saccharides	C6H8O6	3	0.5147	32449-92-6	6546.33	28475.95	6948.82	4624.21	6811.81	703.23	1.05571856	0.3018393	0.67932907	3.457470602	1.789716984	up	C02670	ko00053,ko01100
MW0114251	[illegible]ide-O-methyl-4-O-alpha-D-glucopyranosylsimmond[illegible]	Others	Saccharides	C20H31NO14	3	0.5129	370068-00-1	430.6	3176.01	1651.62	5083.04	6518.33	5525.9	1.39154884	0.02101889	0.27627352	0.307009231	-1.70364606	down	-	-
MW0138677	Kaempferol 3-O-beta-D-glucopyranosyl-7-O-alpha-L-[illegible]	Flavonoids	Flavonols	C27H30O15	2	0.9182	-	564125.85	580694.85	724723.7	71677.65	70671.12	59582.92	1.75567692	0.00802928	0.19758229	9.258301161	3.210747492	up	-	-
MW0157843	Thr-Tyr-Ile-Arg	Amino acids and derivatives	Amino acids and derivatives	C25H41N7O7	2	0.9174	-	1565.66	1065.04	595.71	3725.59	2678.26	4457.16	1.5738112	0.02143018	0.27827994	0.297063533	-1.75115658	down	-	-
MW0155169	Phe-Gln-Thr-Lys	Amino acids and derivatives	Amino acids and derivatives	C24H38N6O7	2	0.9107	-	34053.83	29270.16	29993.03	19975.75	13067.11	13452.38	1.63971799	0.00669862	0.18764551	2.007023085	1.00505721	up	-	-
MW0157536	Thr-Ala-Phe-Lys	Amino acids and derivatives	Amino acids and derivatives	C22H35N5O6	2	0.9055	-	5637.82	15152.86	16203.1	3313.83	5475.19	3129.21	1.41097869	0.12452916	0.50653232	3.103965941	1.634112727	up	-	-
MW0145959	Asn-Val-Phe-Lys	Amino acids and derivatives	Amino acids and derivatives	C24H38N6O6	2	0.9	-	9064.85	13058.64	9190.55	6693.42	6564.42	1724.34	1.1892344	0.06305464	0.392745	2.090085688	1.063562091	up	-	-

MEDTP02401	Cyclocreatine	Nucleotides and derivatives	Nucleotides and derivatives	C5H9N3O2	2	0.8946	-	1399.7	6310.49	7788.07	16442.51	14418.62	11995.5	1.32786012	0.02224249	0.27856654	0.361630394	-1.46741216	down	-	-
MW0151208	HoPhe-Abu-OH	Others	Lactones	C19H20N2O6	2	0.8893	-	2630.46	19846.86	3094.99	14175.63	32949.06	8223.27	1.05431267	0.35263999	0.7090606	0.462028049	-1.11394766	down	-	-
MW0015331	2-[(1R,5S,6R,11R,13R,16S)-6-(furan-3-yl)-11-hydroxy-1,5,15,15-tetramethyl-8,14,17-trioxo-7-oxatetracyclo[11.3.1.02,11.05,10]heptadec-9-en-16-yl]acetic acid	Benzene and substituted derivatives	Benzene and substituted derivatives	C26H30O8	2	0.886	-	85503.81	22996.97	76744	21190.11	21425.69	9812.87	1.36732012	0.14711464	0.5387718	3.53327254	1.821005036	up	-	-
MW0000410	Psychotrin	Alkaloids	Alkaloids	C28H36N2O4	2	0.8685	-	114020.66	104190.49	24768.1	17342.02	41552.26	34647.32	1.00916719	0.21519226	0.60477561	2.597552853	1.377153105	up	-	-
MW0138976	Mollicellin I	Benzene and substituted derivatives	Benzene and substituted derivatives	C21H22O6	2	0.8217	-	733.91	577.18	493.24	4238.84	1267.03	1876.05	1.51547137	0.17596202	0.56921854	0.244425569	-2.03253288	down	-	-
MW0145271	Arg-Asn-Gln-Glu	Amino acids and derivatives	Amino acids and derivatives	C20H35N9O9	2	0.8144	-	15053.49	19099.9	17362.11	79154.3	19756.69	30582.17	1.1722689	0.29045049	0.67244942	0.397824101	-1.32979741	down	-	-
MW0157571	Thr-Asn-Ile-Asp	Amino acids and derivatives	Amino acids and derivatives	C18H31N5O9	2	0.796	-	672.16	1278.34	3297.65	8285.55	3863.65	3536.31	1.31612946	0.13698815	0.5264411	0.334585869	-1.57955158	down	-	-
MW0155414	Phe-Val-Ser-Arg	Amino acids and derivatives	Amino acids and derivatives	C23H37N7O6	2	0.7909	-	663.47	4139	515.7	279.17	445.5	191.87	1.18252241	0.34083457	0.70329636	5.802441792	2.536660145	up	-	-
MW0159024	Val-His-Ile-Glu	Amino acids and derivatives	Amino acids and derivatives	C22H36N6O7	2	0.7895	-	387.73	379.69	591.38	286.22	53.26	10.34	1.29328392	0.0401607	0.33487182	3.884283346	1.957648444	up	-	-
MW0150134	Glu-Ser-Arg-Asp	Amino acids and derivatives	Amino acids and derivatives	C18H31N7O10	2	0.7687	-	765.83	766.07	2263.55	5911.8	2464.22	2129.56	1.27708727	0.19719031	0.58909363	0.361279434	-1.46881296	down	-	-
MW0105263	4-Nitrophenyl [illegible]	Others	Lactones	C6H6NO6P	2	0.7644	-	2249.87	3052.64	2052.19	16614.95	8901.78	6674.02	1.6354946	0.10931773	0.48754925	0.228472465	-2.12990779	down	C03360	-
MW0159180	Val-Thr-Ile-Asp	Amino acids and derivatives	Amino acids and derivatives	C19H34N4O8	2	0.7623	-	891.8	78.06	114.86	7194.84	578.51	883.4	1.18751461	0.36224487	0.71581129	0.125303376	-2.99650281	down	-	-
MW0156968	Ser-Val-Arg-Glu	Amino acids and derivatives	Amino acids and derivatives	C19H35N7O8	2	0.7587	-	21857.64	7398.33	14043.8	2946.89	166.98	4704.36	1.31088062	0.09421689	0.46678757	5.538308543	2.469445429	up	-	-
MW0149927	Glu-Asn-Gln-Glu	Amino acids and derivatives	Amino acids and derivatives	C19H30N6O11	2	0.7567	-	3964.68	3698.81	2479.12	11926.32	7925.92	3696.45	1.2711276	0.19724562	0.58909363	0.430708035	-1.21521786	down	-	-
MW0150931	His-Glu-Tyr-Lys	Amino acids and derivatives	Amino acids and derivatives	C26H37N7O8	2	0.7556	-	3012.53	4681.36	5448.83	807.04	1279.74	1142.18	1.67187224	0.03990703	0.33487182	4.07026411	2.025122411	up	-	-
MW0158715	TyrMe-Trp-OH	Amino acids and derivatives	Amino acids and derivatives	C27H25N3O7	2	0.7519	-	41742.57	39435.49	31153.48	2271.46	24335.51	20163.55	1.11506897	0.06537717	0.39510168	2.401759484	1.264091685	up	-	-
MW0169719	5-(7-Acetyloxy-4-methoxycarbonyl-9-methyl-11-oxo-10-oxatricyclo[6.3.2.01,7]tridec-3-en-9-yl)-2-methylpenta-2,4-dienoic acid	Organic acids	Organic acids	C23H28O8	2	0.7469	-	7661.45	2208.01	11510.53	13828.97	18176.75	18374.41	1.2366695	0.04928947	0.36048486	0.424373458	-1.23659367	down	-	-
MW0156688	Ser-Asp-Thr-Glu	Amino acids and derivatives	Amino acids and derivatives	C16H26N4O11	2	0.7463	-	217849.85	293400.55	347342.85	829057.94	617073.67	616411.2	1.64326021	0.01479617	0.24697482	0.416278996	-1.26437733	down	-	-
MW0158485	Tyr-His-Leu-Glu	Amino acids and derivatives	Amino acids and derivatives	C26H36N6O8	2	0.7437	-	10376.49	11677.31	8865.34	30652.81	11439.73	31424.89	1.30533254	0.16005322	0.54886804	0.420568837	-1.24958614	down	-	-
MW0144694	Ala-Phe-Lys-Arg	Amino acids and derivatives	Amino acids and derivatives	C24H40N8O5	2	0.7386	-	15666.97	14537.7	8312.78	57140.68	12006.28	57726.28	1.16679419	0.18881299	0.58157172	0.303590024	-1.71980371	down	-	-
MW0153090	Lys-Phe-Asn-Lys	Amino acids and derivatives	Amino acids and derivatives	C25H41N7O6	2	0.7363	-	5766.05	1782.77	2324.04	312.16	941.32	133.43	1.48464016	0.14689301	0.5387718	7.118601784	2.831593899	up	-	-
MW0156803	Ser-Ile-Phe-Glu	Amino acids and derivatives	Amino acids and derivatives	C23H34N4O8	2	0.7332	-	3861.38	5596.91	8335.07	496.06	557.69	3414.54	1.42131825	0.05667932	0.37869809	3.982140819	1.993544239	up	-	-
MW0106263	Cys-Met	Amino acids and derivatives	Amino acids and derivatives	C8H16N2O3S2	2	0.7284	-	508452.23	393703.39	347485	995283.43	847948.11	772111.82	1.65862245	0.00642716	0.18604545	0.477811304	-1.06548711	down	-	-
MW0146251	Asp-Ser-Asn-Lys	Amino acids and derivatives	Amino acids and derivatives	C17H30N6O9	2	0.7284	-	11038.68	33497.53	10674.42	62258.3	23446.85	30153.96	1.15930782	0.23896627	0.63210635	0.476532488	-1.06935352	down	-	-
MW0151212	HoPhe-Gly-OH	Others	Lactones	C17H16N2O6	2	0.7225	-	28783.77	100925.21	72659.65	203871.24	157132.38	164321.71	1.44615684	0.01724208	0.25570009	0.385225342	-1.37622548	down	-	-
MW0158710	TyrMe-Phe-OH	Amino acids and derivatives	Amino acids and derivatives	C25H24N2O7	2	0.7149	-	31629.82	6236.03	3922.92	4282.56	2701.74	1533.02	1.17775915	0.33764579	0.70238553	4.906328516	2.294643837	up	-	-
MW0150157	Glu-Thr-Asp-Arg	Amino acids and derivatives	Amino acids and derivatives	C19H33N7O10	2	0.713	-	140.05	288.72	461.93	768.59	275.8	1150.43	1.05766771	0.22184498	0.61560003	0.405819156	-1.30109113	down	-	-
MW0123471	9H-Pyrrolo[1',2':2,3]isoindolo[4,5,6-cd]indol-9-one, 10-acetyl-2,6,6a,7,11a,11b-hexahydro-11-hydroxy-7,7-dimethyl-, (6aR,11aS,11bR)-	Organic acids	Organic acids	C20H20N2O3	2	0.7046	-	227323.82	196538.25	184357.51	98381.6	80462.2	105440.65	1.70946599	0.00418864	0.15945462	2.139475374	1.097257073	up	-	-
MW0004443	Bis(4-cyanophenyl)methanol	Benzene and substituted derivatives	Benzene and substituted derivatives	C15H10N2O	2	0.7041	-	3751.88	5955.58	7762.78	4668.87	2902.11	1079.66	1.13656953	0.13259272	0.51940894	2.019531503	1.014020651	up	-	-

MW0011879	PE(18:3(9Z,12Z,15Z)/18:3(9Z,12Z,15Z))	GL	PE	C41H70NO8P	3	0.9641	-	1729.87	766.87	1105.58	2840.45	4501.67	1742.34	1.37996377	0.13917773	0.52937457	0.396536503	-1.33447442	down	-	-
MW0011241	4-[(E)-3-[4,5-dihydroxy-6-(hydroxymethyl)-3-[3,4,5-trihydroxy-6-(hydroxymethyl)oxan-2-yl]oxyoxan-2-yl]oxybut-1-enyl]-4-hydroxy-3,5,5-trimethylcyclohex-2-en-1-one	Others	Hydrocarbon derivatives	C25H40O13	3	0.961	-	2200.79	1558.66	9264.12	27435.97	4025.36	9644.53	1.01991721	0.31551231	0.68542982	0.316830009	-1.65821911	down	-	-
MADN0549	N-Acetyl-DL-tryptophan	Amino acids and derivatives	Amino acids and derivatives	C13H14N2O3	3	0.9545	-	450831.87	392185.06	466715.44	147748.83	210859.99	193717.34	1.7008106	0.00117877	0.12524365	2.371302438	1.245679677	up	-	-
MW0011668	(Z)-2-Methyl-2-butene-1,4-diol 4-O-beta-D-Glucopyranoside	Others	Saccharides	C11H20O7	3	0.9466	-	10585.96	10339.08	8631.72	19473.27	22076.85	18346.98	1.72491939	0.00348838	0.14907003	0.493458949	-1.01899802	down	-	-
ZINC9591029	Dahuribirin B	Others	Ketone compounds	C34H34O13	3	0.9268	-	103059.79	92534.29	174824.38	63544.51	55695.15	33482.1	1.49958499	0.09414375	0.46678757	2.425446511	1.278250364	up	-	-
MW0013059	[3-hydroxy-2-[3,4,5-trihydroxy-6-(hydroxymethyl)oxan-2-yl]oxypropyl] (7Z,10Z,13Z)-hexadeca-7,10,13-trienoate	GL	DG	C25H42O9	3	0.912	-	14250.18	12268.68	10477.2	6829.69	5710.06	4482.84	1.63729055	0.01055072	0.21412876	2.173350824	1.119921074	up	-	-
MW0145312	Arg-Cys-Gly	Amino acids and derivatives	Amino acids and derivatives	C11H22N6O4S1	3	0.9055	-	8062.78	69391.36	56004.92	6361.73	5114.71	435.87	1.3174263	0.16031448	0.54886804	11.2034576	3.485872139	up	-	-
MEDN1251	LPI(16:0/0:0)	GL	PI	C25H49O12P	3	0.8974	-	92961.07	185381.82	180702.94	55873.16	55754.87	63873.47	1.56875285	0.0868378	0.45026257	2.615623399	1.387154835	up	-	-
ZINC9591030	Dantaxusin A	Others	Lactones	C35H40O10	3	0.8879	-	389.99	145.26	206.89	1535.15	620.95	7210.18	1.39898517	0.2977299	0.67834292	0.079235299	-3.65771289	down	-	-
MW0145842	Asn-Met-His	Amino acids and derivatives	Amino acids and derivatives	C15H24N6O5S1	3	0.8841	-	1335.24	1440.89	936.26	5.08	22.96	30.07	1.70139195	0.01536999	0.24803772	63.88556187	5.997418014	up	-	-
MW0128915	{[(3E)-4-(5-hydroxy-1-oxo-1H-isochromen-3-yl)but-3-en-2-yl]oxy}sulfonic acid	Organic acids	Organic acids	C13H12O7S	3	0.8818	-	21909.01	15175.07	17590.78	3.66	3.71	4.38	1.76210823	0.01148375	0.22207747	4653.179574	12.18400115	up	-	-
MW0054903	Methyl nomilinate 17-glucoside	Others	Lactones	C35H50O16	3	0.8767	-	42192.32	31125.4	48869.03	12591.09	14487.46	12986.02	1.717631	0.0325625	0.31359786	3.049745698	1.608688949	up	-	-
MW0130148	2-(3,4-dihydroxyphenyl)-5,7-dihydroxy-3,6-bis[3,4,5-trihydroxy-6-(hydroxymethyl)oxan-2-yl]-4H-chromen-4-one	Others	Ketone compounds	C27H30O16	3	0.8764	-	15186.96	13290.07	12632.21	47002.6	22043.3	17300.02	1.28169156	0.24276207	0.6366467	0.476099392	-1.07066531	down	-	-
MW0128914	{[(3E)-4-(5-hydroxy-1-oxo-1H-isochromen-3-yl)but-3-en-1-yl]oxy}sulfonic acid	Organic acids	Organic acids	C13H12O7S	3	0.871	-	1320.88	707.74	1675.94	1.43	1.02	0.83	1.75884173	0.04872759	0.35923867	1129.439024	10.14139067	up	-	-
MW0025806	CL(8:0/8:0/8:0/10:0)	GL	CL	C43H82O17P2	3	0.8678	-	3994.38	5187.5	2859.48	9222.17	11516.27	12268.38	1.66594748	0.00468368	0.16605573	0.364814302	-1.45476581	down	-	-
MW0128256	(5Z)-4-[(E)-3-(4-hydroxy-3-methoxyphenyl)prop-2-enoyl]oxy-2-methyl-5-[2-[(2R,3R,4S,5S,6R)-3,4,5-trihydroxy-6-(hydroxymethyl)oxan-2-yl]oxyethylidene]cyclopentene-1-carboxylic acid	Organic acids	Organic acids	C25H30O12	3	0.8655	-	36830.19	30094.36	34764.56	82.48	341.23	2331.7	1.61975776	0.00148221	0.12944673	36.90525548	5.205754372	up	-	-
MW0129754	1-[3-(3,7-dimethylocta-2,6-dien-1-yl)-2,4,6-trihydroxyphenyl]-3-phenylpropan-1-one	Others	Others	C25H30O4	3	0.8148	-	9144.11	2779	15834.52	29602.73	15856.05	17551.42	1.14927098	0.11130374	0.48968655	0.440525978	-1.182701	down	-	-
MW0130947	3-(1,2-dihydroxybutyl)-7-hydroxy-1H-isochromen-1-one	Others	Ketone compounds	C13H14O5	3	0.813	-	40673.99	34777.57	37784.55	116888.17	100805.96	104806.16	1.74377346	0.00212184	0.13898055	0.351119405	-1.50996636	down	-	-
MW0137152	8-[1-(2,4-dihydroxyphenyl)-3-(3,4-dihydroxyphenyl)-2-hydroxypropyl]-2-(4-hydroxy-3-methoxyphenyl)-3,4-dihydro-2H-1-benzopyran-3,5,7-triol	Benzene and substituted derivatives	Benzene and substituted derivatives	C31H30O11	3	0.8004	-	1365.59	13023.86	8798.25	42725.97	34568.14	12980.04	1.27919188	0.11431203	0.49367245	0.256858691	-1.96095321	down	-	-

MW0134170	5,7-dihydroxy-6-[3,4,5-trihydroxy-6-(hydroxymethyl)oxan-2-yl]-8-(3,4,5-trihydroxyoxan-2-yl)-2-(2,4,5-trihydroxyphenyl)-4H-chromen-4-one	Others	Ketone compounds	C26H28O16	3	0.7997	-	3159.08	2854.66	3061.08	559.82	1508.24	521.21	1.56159178	0.01600783	0.25019321	3.504779339	1.809323615	up	-	-
MW0129321	[4-[5,7-Dihydroxy-8-(3-methylbut-2-enyl)-4-oxo-2,3-dihydrochromen-3-yl]-2-hydroxy-6-(3-methylbut-2-enyl)phenyl] hydrogen	Organic acids	Organic acids	C25H28O9S	3	0.7951	-	301541.89	168333.32	177384.67	82711.85	89360.38	108410.87	1.58509454	0.10019291	0.47968762	2.307660889	1.206431236	up	-	-
MW0119085	3-(6,7-dimethoxy-2H-1,3-benzodioxol-5-yl)-2-hydroxypropanal	Benzene and substituted derivatives	Benzene and substituted derivatives	C12H14O6	3	0.7822	-	321557.45	245023.64	213940.88	637886.33	528905.03	497976.59	1.65353122	0.00638986	0.18604545	0.468847307	-1.09280995	down	-	-
MW0133510	4-[5-(3-Hydroxypropyl)-1-benzofuran-2-yl]-2-methoxyphenol	Benzene and substituted derivatives	Benzene and substituted derivatives	C18H18O4	3	0.7715	-	27901.48	30084.48	53538.12	227.91	747.02	326.01	1.73186665	0.04638804	0.3534266	85.72576752	6.421657011	up	-	-
MW0055351	Nonic acid	Organic acids	Organic acids	C9H16O4	3	0.7697	-	1400.96	3562.63	1351.11	553.4	1415.53	880.48	1.14310571	0.24959599	0.63943748	2.216142991	1.148050971	up	-	-
MW0155522	Phospho-anandamide	Alcohol and [illegible]	Amines	C22H37NO5P-	3	0.7667	-	2485.28	5346.45	3383.29	18763.69	4505	6577.3	1.15947581	0.29603758	0.67791845	0.375763042	-1.41210492	down	C19913	-
MW0112538	3,4,5-trihydroxy-6-[4-hydroxy-3-(3-oxoprop-1-en-1-yl)phenoxy]oxane-2-carboxylic acid	Benzene and substituted derivatives	Benzene and substituted derivatives	C15H16O9	3	0.7558	-	1090.61	14004.77	918.24	155.5	484.79	496.4	1.2261292	0.37098238	0.72249913	14.08793954	3.816388718	up	-	-
MW0126941	Vasicinone	Others	Ketone compounds	C11H10N2O2	3	0.7549	-	3094.69	1938.11	3560.64	863.8	67.43	1482.39	1.24516439	0.03241094	0.31359786	3.56039476	1.832037209	up	-	-
MW0055955	PA(20:2(11Z,14Z)/16:1(9Z))	GL	PA	C39H71O8P	3	0.7521	-	3278.75	5191.92	3126.47	22357.42	14520.04	10185.36	1.64655742	0.07537944	0.42178392	0.246418298	-2.02081871	down	-	-
MW0141294	11-Methoxy-vinorine	Alkaloids	Alkaloids	C22H24N2O3	3	0.7513	-	72042.43	88583.63	86076.01	44191.1	25101.5	29150.38	1.64819387	0.00327219	0.14905978	2.506040248	1.325409585	up	C12073	-
ZINC3461983	Petalosa	Others	Saccharides	C10H18O10	3	0.7498	-	1395.09	372.96	1397.54	23800.32	24774.34	7416.91	1.62318811	0.08829545	0.45517121	0.056536904	-4.14466332	down	-	-
MW0142457	2-Methoxyestrone 3-sulfate	Others	Lactones	C19H24O6S	3	0.7487	-	14211.42	37216.47	40019.7	67101.97	43935.3	93964.55	1.28160276	0.10251584	0.48180112	0.446081845	-1.16461966	down	C08358	-
MW0004918	2-Dodecylbenzenesulfonic acid	Benzene and substituted derivatives	Benzene and substituted derivatives	C18H30O3S	3	0.7469	-	229598.29	175206.07	210008.01	71485.85	62104.05	88305.33	1.72143251	0.00581015	0.18224241	2.770732701	1.470267538	up	-	-
MW0119216	3,5,7-Trihydroxy-2-(1-hydroxy-3-methoxy-4-oxocyclohexyl)-6-methoxychromen-4-one	Others	Ketone compounds	C17H18O9	3	0.7453	-	29683.46	13157.46	18378.52	6099.49	10440.46	7998.14	1.49134891	0.12123019	0.50042564	2.494873888	1.318966891	up	-	-
MW0129990	CID 85189935	Benzene and substituted derivatives	Benzene and substituted derivatives	C21H22O7	3	0.7407	-	28618.93	22058.66	17740.55	3312.8	7066	4236.67	1.66120389	0.02007624	0.2743902	4.68121381	2.22688266	up	-	-
MW0138447	Irigenin, 7-benzyl ether	Flavonoids	Flavones	C25H22O8	3	0.7376	-	8380.07	7669.82	8237.82	514.12	3367.4	2102.74	1.44697908	0.0132801	0.23420683	4.058598724	2.020981707	up	-	-
MW0154359	3-[(4-Hydroxy-3,5-dimethoxyphenyl)methyl]-4-[[4-[4-[[4-[(4-hydroxy-3,5-dimethoxyphenyl)methyl]-5-oxooxolan-3-yl]methyl]-2-methoxyphenyl]-3-methoxyphenyl]methyl]oxolan-2-one	Lignans and Coumarins	Lignans	C42H46O12	3	0.7353	-	47432.81	32375.16	39285.58	16296.27	15154.95	17936.05	1.71123637	0.02982948	0.30510706	2.411422012	1.269884153	up	C17683	-
MW0113366	6-(4-ethenyl-3-hydroxyphenoxy)-3,4,5-trihydroxyoxane-2-carboxylic acid	Organic acids	Organic acids	C14H16O8	3	0.7346	-	15587.69	10855.72	17623.75	3259.11	9167.17	8571.95	1.32256323	0.0490836	0.36016204	2.098613074	1.069436198	up	-	-
MW0131219	3-(3,7-dimethylocta-2,6-dien-1-yl)-7-hydroxy-8-(3-methylbut-2-en-1-yl)-2-(2,4,5-trihydroxyphenyl)-4H-chromen-4-one	Others	Others	C30H34O6	3	0.7334	-	39624.3	172335.06	53176.17	22073.11	32368.02	37999.5	1.14845441	0.30490098	0.68276972	2.868170955	1.520131017	up	-	-
MW0153198	Lys-Val-Glu-Ile-Asp	Amino acids and derivatives	Amino acids and derivatives	C26H46N6O10	3	0.7313	-	2865.78	2114.03	1893.53	751.08	362.69	735.49	1.64822603	0.01734776	0.25570009	3.716805641	1.894063249	up	-	-
MW0164191	curcumin 4'-O-beta-D-gentiobioside	Others	Ketone compounds	C33H40O16	3	0.7276	-	6927.47	5320.58	23514.89	50100.87	56070.56	50979.04	1.53131878	0.01344898	0.23505329	0.227571321	-2.13560934	down	-	-
MEDP1089	Humic acid	Organic acids	Organic acids	C9H9NO6	3	0.7259	-	11964.1	21495.47	30327.51	10228.66	684.29	13171.86	1.08757621	0.11921397	0.4975454	2.648436089	1.405140694	up	-	-

MW0130246	2-(3-hydroxyphenyl)-4-[3,5,7-trihydroxy-2-(4-hydroxy-3-methoxyphenyl)-3,4-dihydro-2H-1-benzopyran-8-yl]-8-[3,4,5-trihydroxy-6-(hydroxymethyl)oxan-2-yl]-3,4-dihydro-2H-1-benzopyran-3,5,7-triol	Benzene and substituted derivatives	Benzene and substituted derivatives	C37H38O16	3	0.7254	-	356.23	621.33	740.83	0.53	0.46	0.46	1.75792159	0.0372421	0.32893629	1185.096552	10.21078889	up	-	-
MW0130540	2-[2,3-dihydroxy-4-(methoxymethyl)phenyl]-3,4-dihydro-2H-1-benzopyran-3,5,7-triol	Benzene and substituted derivatives	Benzene and substituted derivatives	C17H18O7	3	0.7234	-	1147290.31	906617.37	1072301.04	18626.68	14211.23	12251.83	1.75908896	0.00473486	0.16605573	69.3330394	6.115471101	up	-	-
MW0130544	2-[2,5-dihydroxy-4-(methoxymethyl)phenyl]-3,4-dihydro-2H-1-benzopyran-3,5,7-triol	Benzene and substituted derivatives	Benzene and substituted derivatives	C17H18O7	3	0.7218	-	2744982.1	2263837.54	2910315.03	53137.7	47434.86	37014.49	1.75907782	0.00551957	0.17971833	57.55726771	5.846926203	up	-	-
MW0137174	Dioonflavone	Others	Ketone compounds	C36H30O10	3	0.7215	-	47475.3	22262.02	39677.14	11562.76	4944.35	8062.42	1.63056363	0.0551551	0.37690654	4.453258162	2.154861249	up	-	-
MW0143391	4-Methyl-5-hydroxyethylthiazole phosphate	Organic acids	Organic acids	C6H10NO4PS	3	0.7207	-	567.45	96.18	728.06	1562.05	2417.85	2913.37	1.40776823	0.02694099	0.29249362	0.20189112	-2.30835064	down	C04327	ko00730,ko01100,ko01240
MW0130566	2-[3-hydroxy-4-(methoxymethyl)phenyl]-3,4-dihydro-2H-1-benzopyran-3,5,6,7-tetrol	Benzene and substituted derivatives	Benzene and substituted derivatives	C17H18O7	3	0.719	-	365769.59	287434.43	242888.81	7166.04	105.55	343.72	1.63623906	0.01409969	0.24165426	117.6699084	6.878601618	up	-	-
MW0128929	{[1-(2,4-dihydroxyphenyl)-3-(3-hydroxyphenyl)-1-[3,5,7-trihydroxy-2-(3-hydroxyphenyl)-3,4-dihydro-2H-1-benzopyran-8-yl]propan-2-	Organic acids	Organic acids	C30H28O12S	3	0.7141	-	70.31	406.34	349.06	175.38	45.51	40.47	1.07581702	0.20390396	0.5952815	3.159282216	1.659596818	up	-	-
MW0152204	Leu-Ala-Met-Glu-Arg	Amino acids and derivatives	Amino acids and derivatives	C25H46N8O8S	3	0.7137	-	722	685.28	1592.35	3529.43	1291.83	1524.55	1.13340883	0.25349459	0.64187885	0.472694581	-1.08101977	down	-	-
MW0008543	Salicylhydroxamic acid	Benzene and substituted derivatives	Benzene and substituted derivatives	C7H7NO3	3	0.7098	-	69620.44	63408.34	112377.92	20280.29	31190.36	46720.89	1.50369509	0.06661286	0.40147997	2.499265212	1.321504002	up	C11343	-
MW0128193	(4-{3-[2,4-dihydroxy-5-(3-methylbut-2-en-1-yl)phenyl]-2-hydroxy-3-oxopropyl}-2-hydroxy-6-(3-methylbut-2-en-1-yl)phenyl)oxidanesulfonic acid	Organic acids	Organic acids	C25H30O9S	3	0.7085	-	106302.22	73648.04	123802.37	337792.64	207414.76	165125.83	1.47258332	0.1116898	0.490598	0.427619907	-1.22559908	down	-	-
MW0146202	Asp-Lys-Val-Lys	Amino acids and derivatives	Amino acids and derivatives	C21H40N6O7	3	0.7084	-	23148.89	11699.01	14248	2357.13	1454.44	1873.48	1.7254964	0.05225242	0.37000361	8.635966262	3.110357607	up	-	-
MW0130185	2-(3,4-dihydroxyphenyl)-8-[1-(2,4-dihydroxyphenyl)-2-hydroxy-3-(3-hydroxyphenyl)propyl]-3,4-dihydro-2H-1-benzopyran-3,5,7-triol	Benzene and substituted derivatives	Benzene and substituted derivatives	C30H28O10	3	0.7062	-	3391.3	4222.6	2904.49	1170.11	1262.85	1030.36	1.70499644	0.02295883	0.28016065	3.037082915	1.602686296	up	-	-
ZINC8553093	[(2S,3R,4S,5R,6S)-3,4-dihydroxy-6-methyl-5-[(2S,3R,4R,5R,6S)-3,4,5-trihydroxy-6-methyloxan-2-yl]oxyoxan-2-yl] (11S)-11-	Others	Lactones	C28H52O11	3	0.7061	-	715.14	113.5	798.74	262.29	56.76	69.02	1.1940992	0.18842101	0.58157172	4.193521787	2.068162352	up	-	-
MW0131915	3,4,5-trihydroxy-6-[4-(5,6,7,8-tetrahydroxy-4-oxo-3,4-dihydro-2H-1-benzopyran-2-yl)phenoxy]oxane-2-carboxylic acid	Organic acids	Organic acids	C21H20O13	3	0.7059	-	1784.62	2028.81	3266.72	966.88	189.67	2064.75	1.12462846	0.14697328	0.5387718	2.19791699	1.1361369	up	-	-
MW0134245	5-[(E)-2-(4-{6-[2,4-dihydroxy-3-(3-methylbut-2-en-1-yl)benzoyl]-5-(2,4-dihydroxyphenyl)-3-methylcyclohex-2-en-1-yl}-3,5-dihydroxyphenyl)ethenyl]benzene-1,2,4-triol	Benzene and substituted derivatives	Benzene and substituted derivatives	C39H38O10	3	0.7017	-	35296.16	19745.76	26823.98	16185.26	8802.5	11888.32	1.53033731	0.06062965	0.38873242	2.220027183	1.150577342	up	-	-

MW0131709	CID 131839423	Organic acids	Organic acids	C15H14O9	3	0.7003	-	628.37	1088.18	1480.79	4817.9	4477.21	2636.24	1.59996366	0.03766884	0.32893629	0.267978058	-1.89981322	down	-	-
MW0110259	Tyr-Ile	Amino acids and derivatives	Amino acids and derivatives	C15H22N2O4	3	0.696	-	100730.37	100337.47	87722.42	16389.58	17387.86	3461.48	1.58266271	0.00017486	0.07934638	7.755065399	2.955138948	up	-	-
MW0144489	Ala-Asn-Asp-Asp	Amino acids and derivatives	Amino acids and derivatives	C15H23N5O10	3	0.693	-	795714.83	672608.83	728217.82	290766.81	306815.11	261642.11	1.7442727	0.00279158	0.14452248	2.556424638	1.354127497	up	-	-
MW0146130	Asp-His-Phe-Asp	Amino acids and derivatives	Amino acids and derivatives	C23H28N6O9	3	0.6918	-	43235.56	51078.35	53159.2	2744.08	17771.18	14466.62	1.43659836	0.00390997	0.15609579	4.215699957	2.07577219	up	-	-
MW0143657	5-Methyldeoxycytidine 5'-(trihydrogen diphosphate)	Organic acids	Organic acids	C10H17N3O10P2	3	0.6911	-	4005.82	7511.1	5303.52	477.11	2197.05	2126.99	1.39314505	0.03932097	0.33249206	3.503418973	1.80876353	up	C04187	-
MW0134077	CID 74977687	Others	Ketone compounds	C27H30O15	3	0.6898	-	146995.8	149751.06	185207.06	18417.65	17070.75	14308.64	1.75514584	0.0067935	0.18870785	9.678364818	3.274763322	up	-	-
MW0143260	4-Benzyloxy-2'-hydroxy-3',4',5',6'-tetramethoxychalcone	Benzene and substituted derivatives	Benzene and substituted derivatives	C26H26O7	3	0.6887	-	1445.39	8553.08	307.18	297223.39	1545.24	133791.26	1.13916569	0.24143783	0.63571533	0.023824793	-5.39139249	down	-	-
MW0154346	N-dodecanoyl-L-Homoserine lactone-3-hydrazone-fluorescein	Amino acids and derivatives	Amino acids and derivatives	C37H40N4O8S	3	0.6882	-	28808.65	23889.76	27701.54	2258.45	10351.82	4684.68	1.55620073	0.00341358	0.14905978	4.64875296	2.216843761	up	-	-
MW0119895	4-(3-Pyridyl)-3-butenoic acid	Organic acids	Organic acids	C9H9NO2	3	0.6879	-	3132.31	2872.08	3670.12	109.29	1496.38	1576.21	1.18519425	0.02808366	0.2989387	3.040501213	1.604309165	up	-	-
MW0134065	[5,7-dihydroxy-2-(4-hydroxy-3-methoxyphenyl)-3,4-dihydro-2H-chromen-3-yl] 3,4,5-trihydroxybenzoate	Benzene and substituted derivatives	Benzene and substituted derivatives	C23H20O10	3	0.6836	-	1069.77	3744.25	1246.19	435.57	1235.88	393.49	1.187379	0.25926284	0.64658621	2.934811665	1.553267925	up	-	-
MW0138672	Kaempferol3-rhamninoside	Flavonoids	Flavones	C33H40O19	3	0.6828	-	4182.97	6843.14	3606.99	1700.09	2217.11	1672.68	1.57378082	0.08984584	0.45681891	2.617784282	1.388346217	up	-	-
MW0131355	3-(4-Hydroxyphenyl)-2-sulfooxypropanoic acid	Organic acids	Organic acids	C9H10O7S	3	0.6775	-	9421.5	8102.63	7458.73	2866.93	2495.83	4351.87	1.66064029	0.00327237	0.14905978	2.571673857	1.36270769	up	-	-
MW0155114	Phe-Asn-Thr-Lys	Amino acids and derivatives	Amino acids and derivatives	C23H36N6O7	3	0.6763	-	135010.36	111028.19	100770.72	1190.23	1825.72	865.92	1.74879677	0.00774859	0.19498432	89.34077391	6.481246847	up	-	-
MW0137155	8-[2-(acetyloxy)-1-hydroxypropan-2-yl]-2-oxo-2H,8H,9H-furo[2,3-h]chromen-9-yl(2E)-2-methylbut-2-enoate	Lignans and Coumarins	Coumarins	C21H22O8	3	0.6763	-	54838.23	8742.93	8104.13	8412.58	3613.77	5061.88	1.11832845	0.36043909	0.71458744	4.195009665	2.068674134	up	-	-
MW0128569	[2-(acetyloxy)-2-(9-{[(2E)-2-methylbut-2-enoyl]oxy}-2-oxo-2H,8H,9H-furo[2,3-h]chromen-8-yl)propoxy]sulfonic acid	Organic acids	Organic acids	C21H22O11S	3	0.6763	-	147724.28	157197.76	161501	15320.33	5587.49	3759.1	1.70123782	1.2571E-05	0.05599079	18.90884796	4.240989563	up	-	-
MW0113281	6-[2-(Acetyloxymethyl)-4,5,6-trihydroxyoxan-3-yl]oxy-3,4,5-trihydroxyoxane-2-carboxylic acid	Organic acids	Organic acids	C14H22O13	3	0.6744	-	9022.33	6567.72	7864.84	3039.86	973.97	7267.25	1.18899548	0.14819692	0.5387718	2.079135154	1.055983543	up	-	-
MW0107687	S-Glutathionyl-L-cysteine	Amino acids and derivatives	Amino acids and derivatives	C13H22N4O8S2	3	0.6739	-	24924.19	30960.08	29092.49	93099.26	73437.53	65519.21	1.71540094	0.02266453	0.27962521	0.366190747	-1.44933276	down	C05526	ko00270,ko01100
MW0157072	S-Inosyl-L-homocysteine	Amino acids and derivatives	Amino acids and derivatives	C14H19N5O6S	3	0.6709	-	3820.25	2787.82	2458.02	443.72	1785.17	1065.08	1.39941153	0.02719371	0.29249362	2.752329256	1.460653067	up	C03431	ko00270,ko01100
MW0131745	3,4,5-trihydroxy-6-[(5-hydroxy-4-methyl-2-oxo-2H-chromen-7-yl)oxy]oxane-2-carboxylic acid	Organic acids	Organic acids	C16H16O10	3	0.67	-	364.58	475.64	54733.19	63764.7	42901.91	45385.04	1.19896215	0.21066016	0.60186131	0.365490345	-1.4520948	down	-	-
MW0150185	Glu-Tyr-Asn-Lys	Amino acids and derivatives	Amino acids and derivatives	C24H36N6O9	3	0.6695	-	1540.85	732.06	1634.34	5599.01	3555.91	4385.72	1.58161619	0.01806758	0.26056177	0.288557262	-1.79307046	down	-	-
MW0155378	Phe-Tyr-Asn-Glu	Amino acids and derivatives	Amino acids and derivatives	C27H33N5O9	3	0.6668	-	10514.16	16754.14	15924.02	58717.84	19961.17	16037.82	1.05218826	0.33399541	0.69939612	0.456015261	-1.13284599	down	-	-
MW0157916	Thr-TyrMe-OH	Amino acids and derivatives	Amino acids and derivatives	C20H22N2O8	3	0.6653	-	17326.08	19299.74	16639.05	92566.4	23840.83	34645.55	1.27975233	0.26606925	0.6495827	0.352624228	-1.50379649	down	-	-
MW0152192	Leu-Ala-Arg-Glu	Amino acids and derivatives	Amino acids and derivatives	C20H37N7O7	3	0.6641	-	46.24	40.22	32.22	21.38	18.45	12.7	1.59404866	0.01497725	0.24697482	2.259280411	1.175863342	up	-	-
MW0012595	7,8,17-trihydroxy-4,9,11,13,15,19-docosahexaenoic acid	Lipids	Others	C22H32O5	3	0.6625	-	33081.41	10426.87	7546.78	5611.12	4356.56	8934.78	1.1423514	0.31475589	0.68542982	2.700974371	1.43347995	up	-	-
MW0116191	[3-(6,7-dimethoxy-2H-1,3-benzodioxol-5-yl)propoxy]sulfonic acid	Organic acids	Organic acids	C12H16O8S	3	0.6605	-	4258.14	4260.35	15665.15	75200.54	10489.47	13867.12	1.08110721	0.354374	0.71066267	0.242912185	-2.04149323	down	-	-

MW0144943	[hydroxy-[(2R,3R,4S,5R,6R)-3,4,5-trihydroxy-6-(hydroxymethyl)tetrahydropyran-2-yl]oxy-phosphoryl] [(2Z,6Z,10Z,14Z,18Z,22Z,26Z,30Z,34E,38E)-3,7,11,15,19,23,27,31,35,39,43-undecamethyltetratetraconta-2,6,10,14,18,22,26,30,34,38,42-undecaenyl] hydrogen phosphate	Alcohol and amines	Alcohols	C61H102O12P2	3	0.6596	-	2972.58	2824.03	3829.08	347.64	385.53	978.36	1.66263061	0.00367756	0.15308276	5.624026456	2.491603381	up	C04507	-
MW0159000	Val-Glu-Thr-Glu	Amino acids and derivatives	Amino acids and derivatives	C19H32N4O10	3	0.6595	-	8373.59	14885.74	5198.28	23776.6	17245.78	20200.57	1.43571052	0.04042411	0.3359123	0.46481932	-1.10525806	down	-	-
MW0109195	4-Hydroxyphenylacetylglutamate	Amino acids and derivatives	Amino acids and derivatives	C13H15NO6	3	0.6588	-	14348.12	12132.74	10876.37	25748.19	26067.57	24211.79	1.71857136	0.00125098	0.12524365	0.49136438	-1.02513482	down	C05595	ko00350
MW0130695	CID 131835378	Others	Lactones	C16H16O6	3	0.6575	-	27282.82	20394.28	26287.9	9780.71	4577.12	18721.53	1.35655539	0.06091879	0.38873242	2.235986428	1.160911431	up	-	-
MW0014950	6-[(2Z)-2-carboxy-2-(phenylmethylidene)ethoxy]-3,4,5-trihydroxyoxane-2-carboxylic acid	Organic acids	Organic acids	C16H18O9	3	0.6567	-	25948.52	77966.07	18407.89	13895.41	9290.85	7390.22	1.36144144	0.24314413	0.6366467	4.000541593	2.000195325	up	-	-
MW0158430	Tyr-Gln-Asn-Glu	Amino acids and derivatives	Amino acids and derivatives	C23H32N6O10	3	0.6549	-	19716.39	12607.23	17865.96	2735.08	9104.2	10967.08	1.26430104	0.05095271	0.36604803	2.200683494	1.137951669	up	-	-
MW0129281	{4-[1-Oxo-1-(2,4,6-trihydroxyphenyl)propan-2-yl]phenyl}oxidanesulfonic acid	Organic acids	Organic acids	C15H14O8S	3	0.6539	-	3889.37	3012.83	5458.38	1003.4	328.56	201.49	1.61807328	0.02673183	0.29249362	8.060634517	3.010893409	up	-	-
MW0109856	Thr-Asn	Amino acids and derivatives	Amino acids and derivatives	C8H15N3O5	3	0.6538	-	727.27	683.22	4349.27	97.35	1201.11	374.37	1.00131003	0.37969877	0.72875989	3.44312333	1.783717858	up	-	-
MW0165099	Desmethyldescarbamoyl novobiocin(1-)	Benzene and substituted derivatives	Benzene and substituted derivatives	C29H32NO10-	3	0.6534	-	470.71	1475.89	4908.65	657.4	705.99	322.57	1.03309322	0.32796548	0.69268022	4.066081046	2.023638972	up	-	-
MW0008616	Tenovin-6	Benzene and substituted derivatives	Benzene and substituted derivatives	C25H34N4O2S	3	0.6524	-	7759.58	3613.26	5829.64	17791.5	12030.32	8814.66	1.39945479	0.09575825	0.47047039	0.445239318	-1.1673471	down	-	-
MW0104618	2-amino-4-({1-[(carboxymethyl)-C-hydroxycarbonimidoyl]-2-{[2-hydroxy-1-(4-methoxyphenyl)-4-methyl-3-oxopentyl]sulfanyl}ethyl}-C-hydroxycarbonimidoyl)butanoic acid	Organic acids	Organic acids	C23H33N3O9S	3	0.6488	-	5824.45	14342.94	16108.2	2380.6	4232.38	4516.08	1.45400036	0.11279792	0.49245668	3.259537643	1.704667336	up	-	-
MW0112592	3,4,5-Trihydroxy-6-[3-(3-hydroxyphenyl)propanoyloxy]oxane-2-carboxylic acid	Organic acids	Organic acids	C15H18O9	3	0.648	-	1610.22	791.72	3662.88	42073.26	38756.14	32933.09	1.67967392	0.00286582	0.14452248	0.053311245	-4.22941631	down	-	-
MW0135539	6-[4-(3-{3-[6-carboxy-5-(2,4-dihydroxyphenyl)-3-methylcyclohex-2-en-1-yl]-2,4-dihydroxyphenyl}-3-oxopropyl)-3-hydroxyphenoxy]-3,4,5-trihydroxyoxane-2-	Organic acids	Organic acids	C35H36O15	3	0.6444	-	12378.13	8984.86	10309.18	3842.78	5180.03	3650.68	1.68039843	0.01138813	0.22207747	2.499088254	1.321401851	up	-	-
MW0159462	Zizybeoside II	Benzene and substituted derivatives	Benzene and substituted derivatives	C25H38O16	3	0.6426	-	289590.49	120583.36	218781.18	123013.57	68198.3	107082.67	1.36052138	0.14312496	0.53323181	2.108503327	1.076219298	up	C17565	-
MW0118164	[6-(2,4-Dihydroxyphenyl)-2-(2,6-dihydroxyphenyl)-4-methylcyclohex-3-en-1-yl]-(5-hydroxy-2,2-dimethylchromen-6-yl)methanone	Benzene and substituted derivatives	Benzene and substituted derivatives	C31H30O7	3	0.6426	-	10699.35	67271.58	12396.91	8790.99	8718.27	7782.24	1.02598241	0.36338132	0.71615061	3.573051816	1.837156836	up	-	-
MW0158483	Tyr-His-Ile-Glu	Amino acids and derivatives	Amino acids and derivatives	C26H36N6O8	3	0.6408	-	4240.15	2237.16	2492.2	10035.04	11402.39	9508.83	1.67340521	0.00102871	0.12524365	0.289841486	-1.78666398	down	-	-

MW0158704	TyrMe-Ile-OH	Amino acids and derivatives	Amino acids and derivatives	C22H26N2O7	3	0.64	-	611155.41	541395.57	606437.12	79380.73	73904.51	100761.61	1.75719866	0.00068006	0.12117687	6.9238729	2.791579242	up	-	-
MW0011512	2-(hydroxymethyl)-6-[(E)-2-methylbut-2-enoxy]oxane-3,4,5-triol	Others	Saccharides	C11H20O6	3	0.64	-	4291.59	3560.63	3779.61	1348.93	1348.87	604.51	1.62021112	0.00117348	0.12524365	3.522331338	1.816530627	up	-	-
MW0148753	dTDP-4-keto-2-deoxy-beta-L-xylose	Nucleotides and derivatives	Nucleotides and derivatives	C15H22N2O14P2	3	0.6376	-	1006.9	5915.42	998.19	4168.79	4700.78	8771.67	1.19682081	0.21402138	0.60408824	0.448976943	-1.15528674	down	C21353	ko01110
MW0132580	3,4-dihydroxy-5-[3-hydroxy-4-(sulfooxy)-5-(3,4,5-trihydroxybenzoyloxy)benzoyloxy]benzoic acid	Organic acids	Organic acids	C21H14O16S	3	0.636	-	449.02	449.56	309.06	939.4	1687.95	3476.99	1.55445957	0.16192032	0.54904711	0.197833017	-2.33764487	down	-	-
MW0135004	6-(4-{5,7-dihydroxy-4-oxo-6,8-bis[3,4,5-trihydroxy-6-(hydroxymethyl)oxan-2-yl]-4H-chromen-2-yl}-2,5-dihydroxyphenoxy)-3,4,5-trihydroxyoxane-2-carboxylic acid	Organic acids	Organic acids	C33H38O23	3	0.636	-	5496.39	19964.07	21709.94	8531.68	3456.55	6916.76	1.09309255	0.20192798	0.59445191	2.495129593	1.319114749	up	-	-
MW0129954	2-(1,2-dihydroxypropan-2-yl)-9-hydroxy-2H,3H,7H-furo[3,2-g]chromen-7-one	Others	Ketone compounds	C14H14O6	3	0.6351	-	32604.05	31934.04	35795.23	2267.88	8885.96	17406.3	1.39721484	0.02529839	0.29191454	3.513054208	1.812725838	up	-	-
ZINC2731639	Tzfnsitzszgmkw-qkqmvzjrsa-	Others	Lactones	C30H42O13	3	0.6339	-	17583.96	15301.72	13785.59	73002.27	24385.21	23805.51	1.28688649	0.26674602	0.6495827	0.385098759	-1.37669962	down	-	-
MW0106884	Glu-Ala-Trp	Amino acids and derivatives	Amino acids and derivatives	C19H24N4O6	3	0.6323	-	106531.51	111145.59	135011.72	213003.65	283218.98	234702.33	1.68598445	0.01488465	0.24697482	0.482523979	-1.05132746	down	-	-
MW0147747	Condurangogenin A	Benzene and substituted derivatives	Benzene and substituted derivatives	C32H42O7	3	0.6312	-	2247.22	2195.02	3812.9	782.07	945.94	1535.74	1.54547862	0.07154421	0.41116592	2.529342015	1.33876213	up	C17767	-
MW0104507	2-amino-4-({1-[(carboxymethyl)-C-hydroxycarbonimidoyl]-2-({2,3,4,6-tetrahydroxy-5-[(2E)-3-(4-hydroxyphenyl)prop-2-enoyl]phenyl}sulfanyl)ethyl}-C-hydroxycarbonimidoyl)butanoic acid	Organic acids	Organic acids	C25H27N3O12S	3	0.6298	-	2177.47	693.41	1359.12	11.05	367.59	252.61	1.29908954	0.10021994	0.47968762	6.700990099	2.744374275	up	-	-
MW0057082	PC(18:3(6Z,9Z,12Z)/15 0)	GL	PC	C41H76NO8P	3	0.6298	-	1434.54	3756.75	3467.63	25.12	1256.44	58.37	1.35288784	0.05853208	0.38394972	6.462218176	2.692029459	up	-	-
MW0158011	trans-Zeatin riboside triphosphate	Nucleotides and derivatives	Nucleotides and derivatives	C15H24N5O14P3	3	0.6294	-	1021.24	1652.86	1272.56	600.47	327.42	709.69	1.51685189	0.03171491	0.31251818	2.410056303	1.26906685	up	-	-
MW0143534	12-Dehydrotetracycline	Benzene and substituted derivatives	Benzene and substituted derivatives	C22H22N2O8	3	0.6278	-	21305.65	24918.55	32264.7	828.88	9815.06	8510.6	1.31528083	0.01027017	0.21412876	4.097665619	2.034802261	up	C03206	ko01100,ko01110
MW0013810	3,4,5-trihydroxy-6-{[5-(4-methoxyphenyl)-3-oxopentyl]oxy}oxane-2-carboxylic acid	Organic acids	Organic acids	C18H24O9	3	0.626	-	3371.26	717.89	3541.63	3879.95	4311.44	7248.25	1.04756085	0.13769012	0.52754608	0.494233026	-1.01673668	down	-	-
ZINC8604739	Terrestrin F	Benzene and substituted derivatives	Benzene and substituted derivatives	C36H26O10	3	0.6238	-	11808.67	12532.33	13464.21	8142.31	5526.5	2863.25	1.45393628	0.03365215	0.31755651	2.286781563	1.193318564	up	-	-
MW0112507	3,4,5-trihydroxy-6-[3-hydroxy-2-methoxy-4-(prop-2-en-1-yl)phenoxy]oxane-2-carboxylic acid	Organic acids	Organic acids	C16H20O9	3	0.6234	-	1527527.34	1247387.79	1267086.3	324894.23	550819.22	646435.33	1.61994705	0.00309617	0.14905978	2.655457524	1.408960452	up	-	-
MW0104617	2-amino-4-({1-[(carboxymethyl)-C-hydroxycarbonimidoyl]-2-{[2-hydroxy-1-(4-methoxyphenyl)-3-oxopropyl]sulfanyl}ethyl}-C-hydroxycarbonimidoyl)butanoic acid	Organic acids	Organic acids	C20H27N3O9S	3	0.6233	-	20003.74	23453.96	19365.98	1358.59	11470.05	14906.8	1.09494776	0.09152332	0.45957708	2.265104862	1.179577841	up	-	-
MW0107248	His-Asp-Lys	Amino acids and derivatives	Amino acids and derivatives	C16H26N6O6	3	0.6187	-	17396.18	11619.37	20427.34	2873.26	1801.57	3642.91	1.7069264	0.02945348	0.30296951	5.944269717	2.571499579	up	-	-

MW0131761	3,4,5-trihydroxy-6-[(6-hydroxy-4-methyl-2-oxo-2H-chromen-7-yl)oxy]oxane-2-carboxylic acid	Organic acids	Organic acids	C16H16O10	3	0.6165	-	25042.74	32455.92	17730.44	54229.42	48580.3	66294.98	1.60384319	0.01067279	0.21412876	0.444866997	-1.16855402	down	-	-
MW0109885	Thr-Lys-Tyr	Amino acids and derivatives	Amino acids and derivatives	C19H30N4O6	3	0.6161	-	33605.7	33758.91	58655.59	18222.29	23609.99	19843.3	1.53206391	0.11860614	0.4975454	2.043275475	1.030883722	up	-	-
MW0104621	2-amino-4-({1-[(carboxymethyl)-C-hydroxycarbonimidoyl]-2-{[2-hydroxy-3-(4-hydroxy-2-methoxyphenyl)-1-phenylpropyl]sulfanyl}ethyl}-C-hydroxycarbonimidoyl)butanoic acid	Organic acids	Organic acids	C26H33N3O9S	3	0.6123	-	124054.84	57295.62	96169.61	790.62	42215.9	36392.88	1.09693105	0.05517357	0.37690654	3.4952414	1.805392099	up	-	-
MW0104543	2-amino-4-({1-[(carboxymethyl)-C-hydroxycarbonimidoyl]-2-[(2-{3,5-dihydroxy-4-[(1E)-3-methylbut-1-en-1-yl]phenyl}-1-(3,4-dihydroxyphenyl)-2-hydroxyethyl)sulfanyl]ethyl}-C-hydroxycarbonimidoyl)butanoic acid	Organic acids	Organic acids	C29H37N3O11S	3	0.611	-	5081.61	4324.83	6046.54	13764.68	9991.31	8790.24	1.59560972	0.05101522	0.36604803	0.474800922	-1.07460536	down	-	-
MW0054718	4-(1,3-Butadienyl)-3,5,5-trimethylcyclohex-2-en-1-one	Others	Aldehyde compounds	C13H18O	3	0.6107	-	3643.43	6058.14	7040.02	5901.45	881.69	303.54	1.14444079	0.20865785	0.60095435	2.362402423	1.240254741	up	-	-
MW0112352	3,4,5-Trihydroxy-6-[2-[4-(2-methylpropyl)phenyl]propanoyloxy]oxane-2-carboxylic acid	Organic acids	Organic acids	C19H26O8	3	0.6058	-	49540.21	14445.2	3143.24	70009.76	62972.46	53731.26	1.23778001	0.09164428	0.45963526	0.359527603	-1.47582555	down	-	-
MW0058173	PE(16:1(9Z)/22:5(7Z,10Z,13Z,16Z,19Z))	GL	PE	C43H74NO8P	3	0.6049	-	9760.35	7621.52	7561.73	101693.64	15170.07	12680.45	1.15793464	0.3556934	0.71211706	0.192549012	-2.37670238	down	C00350	ko00563,ko00564,ko01100,ko01110,ko04136
MW0000673	(3-Ethoxyphenyl)-(2,3,4-trihydroxyphenyl)metha[illegible]	Benzene and substituted derivatives	Benzene and substituted derivatives	C15H14O5	3	0.6036	-	728.08	538.83	1796.79	3126.55	1395.27	3298.16	1.26467882	0.10496617	0.48180112	0.391778496	-1.35188988	down	-	-
MW0109906	Thr-Val-Arg	Amino acids and derivatives	Amino acids and derivatives	C15H30N6O5	3	0.6007	-	381513.01	128457.16	128864.77	61387.59	21655.36	94106.04	1.35621071	0.20442137	0.5952815	3.606201424	1.850479981	up	-	-
MW0113343	6-(3-carboxy-5-methoxyphenoxy)-3,4,5-trihydroxyoxane-2-carboxylic acid	Organic acids	Organic acids	C14H16O10	3	0.5999	-	6428.2	1951.47	795.84	9210.98	3615.5	10567.04	1.13808015	0.16124519	0.54904711	0.392224428	-1.3502487	down	-	-
ZINC9591021	Cowagarcinone E	Others	Lactones	C31H36O8	3	0.5996	-	815.94	440.93	381.01	225.63	241.69	214.96	1.52722137	0.14366371	0.53323181	2.400597995	1.26339383	up	-	-
MW0058138	PE(16:0/22:5(4Z,7Z,10Z,13Z,16Z))	GL	PE	C43H76NO8P	3	0.5981	-	66502.83	80953.96	70128.24	52917.28	1265.82	407.72	1.29244665	0.08089662	0.434301	3.98574394	1.994849029	up	-	-
MW0130137	2-(3,4-dihydroxyphenyl)-4-[3,5,7-trihydroxy-2-(4-hydroxy-3-methoxyphenyl)-3,4-dihydro-2H-1-benzopyran-8-yl]-6-[3,4,5-trihydroxy-6-(hydroxymethyl)oxan-2-yl]-3,4-dihydro-2H-1-	Benzene and substituted derivatives	Benzene and substituted derivatives	C37H38O17	3	0.5969	-	11443.79	12809.13	9772.66	150.37	1309.49	1695.17	1.53551605	0.00181816	0.13216141	10.78455038	3.430894126	up	-	-
MW0058215	1-Vaccenoyl-2-myristoyl-sn-glycero-3-phosphoethanolamine	GL	PE	C37H72NO8P	3	0.5961	-	99687.94	963898.76	70607.23	31990.77	30682.32	16637.71	1.30953851	0.35301588	0.70921638	14.300624	3.838006195	up	-	-
MW0058054	1-Myristoleoyl-2-vaccenoyl-sn-glycero-3-phosphoethanolamine	GL	PE	C37H70NO8P	3	0.596	-	21110.68	49546.11	20460.71	6051.05	6884.23	342.9	1.32363609	0.10782429	0.48477095	6.862197982	2.77867075	up	-	-
MW0058238	PE(18:1(11Z)/22:4(7Z,10Z,13Z,16Z))	GL	PE	C45H80NO8P	3	0.5942	-	11543.04	28600.14	11285.58	3532.53	229.75	3975.34	1.36462504	0.12073422	0.49954518	6.646586418	2.732613585	up	-	-
MW0058172	PE(16:1(9Z)/22:5(4Z,7Z,10Z,13Z,16Z))	GL	PE	C43H74NO8P	3	0.5932	-	1930.07	2356.73	1892.52	10.07	2.01	2.01	1.73731481	0.00519513	0.17366357	438.5606813	8.776632664	up	-	-
MW0000462	1-(1,2,3,4,5-Pentahydroxypent-1-yl)-1,2,3,4-tetrahydro-beta-carboline-3-carboxylate	Alkaloids	Alkaloids	C17H22N2O7	3	0.5884	-	11175.36	7519.71	3895.93	7746.26	286.19	1504.24	1.08509294	0.23658124	0.63043731	2.368851247	1.244187607	up	-	-

MW0058067	PE(14:1(9Z)/20:5(5Z,8Z,11Z,14Z,17Z))	GL	PE	C39H66NO8P	3	0.5861	-	80.21	48.21	85.25	2124.67	2054.35	255.31	1.55934806	0.14811745	0.5387718	0.048185408	-4.37525987	down	-	-
MW0161375	2-amino-2-deoxy-beta-D-glucopyranosyl-(1->4)-2-amino-2-deoxy-beta-D-glucopyranosyl-(1->4)-2-amino-2-deoxy-D-glucopyranose	Alcohol and amines	Alcohols	C18H35N3O13	3	0.5859	-	4652.62	4744.41	2658.45	7643.3	7509.78	9150.28	1.55804629	0.01045067	0.21412876	0.4960417	-1.01146669	down	-	-
MW0058231	PE(18:1(11Z)/20:3(8Z,11Z,14Z))	GL	PE	C43H78NO8P	3	0.5854	-	60237.34	55903.83	51988.14	139252.2	115958.63	116085.93	1.73106469	0.00816647	0.19768185	0.452816529	-1.14300147	down	-	-
ZINC7045454	1beta-Hydroxy-7-deacetylbaccatin I	Others	Lactones	C30H42O13	3	0.5772	-	19.89	16.08	6.49	64.68	29.51	24.74	1.34229856	0.17191572	0.56343828	0.357016733	-1.4859364	down	-	-
MW0148772	dTDP-alpha-D-mycaminose	Nucleotides and derivatives	Nucleotides and derivatives	C18H31N3O14P2	3	0.5762	-	174.24	337.79	501.32	2288.11	1770.11	287.99	1.15506922	0.20255438	0.59510371	0.233157165	-2.10062533	down	-	-
MW0136416	6-{4-[2,3-dioxo-3-(2,4,6-trihydroxyphenyl)propyl]phenoxy}-3,4,5-trihydroxyoxane-2-carboxylic acid	Organic acids	Organic acids	C21H20O12	3	0.5757	-	11694.5	8090.77	16134.91	938.76	5050.21	2524.44	1.47392539	0.0399256	0.33487182	4.219247047	2.076985563	up	-	-
MW0134314	Viniferin	Benzene and substituted derivatives	Benzene and substituted derivatives	C28H22O6	3	0.5748	-	1213.61	2710.56	1241.59	2768.29	4368.3	4692.11	1.4634662	0.04718787	0.35562566	0.436714094	-1.195239	down	-	-
MW0104554	2-amino-4-({1-[(carboxymethyl)-C-hydroxycarbonimidoyl]-2-[(3-hydroxy-5-oxo-1,7-diphenylheptan-4-yl)sulfanyl]ethyl}-C-hydroxycarbonimidoyl)butanoic acid	Organic acids	Organic acids	C29H37N3O8S	3	0.5721	-	2429.35	6011.81	1948.19	12767.5	3437.74	9615.86	1.15064185	0.19288506	0.58497036	0.402358924	-1.31344507	down	-	-
MW0156659	Ser-Asn-Gln-Asp	Amino acids and derivatives	Amino acids and derivatives	C16H26N6O10	3	0.5611	-	15561.68	29310.32	20916.18	19181.89	9531.68	3935.05	1.13866575	0.1395622	0.52993183	2.015037083	1.010806389	up	-	-
MW0057107	PC(18:3(6Z,9Z,12Z)/24 [illegible]	GL	PC	C50H94NO8P	3	0.5591	-	16273.98	14469.89	12832.86	65026.34	46207.56	34426.74	1.67512156	0.06055827	0.38873242	0.29916613	-1.74098124	down	-	-
MW0151819	Isodonal	Others	Lactones	C22H28O7	3	0.5577	-	11631.03	7480.09	15112.3	1990.72	5097	9996.39	1.15043891	0.14982806	0.5387718	2.003231073	1.002328846	up	C09117	-
MW0145380	Arg-His-Val	Amino acids and derivatives	Amino acids and derivatives	C17H30N8O4	3	0.557	-	1153.72	3119.47	1540.27	9593.98	3513.26	2547.62	1.21220747	0.27243745	0.65233913	0.371351772	-1.42914163	down	-	-
MEDL01900	3,4,6-trihydroxy-5-oxo-1-(3,5,7-trihydroxy-3,4-dihydro-2H-chromen-2-yl)benzo[7]annulene-8-carboxylic acid	Tannins	Proanthocyanidins	C21H16O10	3	0.5509	-	226.46	281.56	561.82	2385.92	550.79	439.2	1.06641388	0.347034	0.70635074	0.316904183	-1.65788139	down	-	-
MW0149953	Glu-Asp-Thr-Glu	Amino acids and derivatives	Amino acids and derivatives	C18H28N4O12	3	0.5437	-	38105.07	22624.03	27425.47	127.46	366.05	728.13	1.71785825	0.02389438	0.28304669	72.16084116	6.173144251	up	-	-
MW0011908	Dioleoyl phosphatidylserine	GL	PS	C42H78NO10P	3	0.5402	-	24.63	24.63	24.63	1515.45	777.54	817.07	1.74838657	0.05175865	0.3682636	0.023758384	-5.39541947	down	-	-
MW0113418	6-[(2-{[3,4-dihydroxy-2,5-bis(hydroxymethyl)oxolan-2-yl]oxy}-3,5-dihydroxy-6-(hydroxymethyl)oxan-4-yl)oxy]-3,4,5-trihydroxyoxane-2-[illegible]	Organic acids	Organic acids	C18H30O17	3	0.5359	-	30484.95	23668.23	26826.96	2492.75	4846.14	1245.06	1.66161123	0.00154988	0.13083964	9.433901642	3.237854559	up	-	-
MW0132101	3,4,5-Trihydroxy-6-[4-hydroxy-2-oxo-3-(3-oxo-1-phenylbutyl)chromen-7-yl]oxyoxane-2-carboxylic acid	Organic acids	Organic acids	C25H24O11	3	0.5344	-	1300.46	3272.24	1164.31	11379.37	5987.56	3379.16	1.43493443	0.15966546	0.54886804	0.276534518	-1.85446852	down	-	-
MW0105834	Asn-Ser	Amino acids and derivatives	Amino acids and derivatives	C7H13N3O5	3	0.5246	-	1291.49	5710.85	2122.9	18592.94	6366.7	16734.71	1.45999877	0.09067117	0.45758514	0.218860349	-2.19191749	down	-	-
MW0135875	6-{[3-(3,4-dimethoxyphenyl)-7-methoxy-8-methyl-4-oxo-4H-chromen-5-yl]oxy}-3,4,5-trihydroxyoxane-2-carboxylic acid	Organic acids	Organic acids	C25H26O12	3	0.5219	-	19228.74	16367.2	17044.8	207.09	2230.12	3243.8	1.48672021	0.00022952	0.07934638	9.266088248	3.211960422	up	-	-
MW0058107	PE(15:0/22:6(4Z,7Z,10Z,13Z,16Z,19Z))	GL	PE	C42H72NO8P	3	0.5151	-	309.39	1375.02	2088.67	7545.01	3172.73	4977.74	1.38950685	0.07263339	0.41345377	0.240392775	-2.05653456	down	-	-
MW0133578	3,4,5-trihydroxy-6-[[3-hydroxy-2-(4-hydroxy-3-methoxyphenyl)-5-sulino-3,4-dihydro-2H-chromen-7-yl]oxy]oxane-2-carboxylic acid	Benzene and substituted derivatives	Benzene and substituted derivatives	C22H24O13S	3	0.513	-	37291.24	41692.02	34529.17	1078.38	146.74	654.02	1.70630227	0.00274252	0.14452248	60.40658493	5.916633921	up	-	-

Index	Compounds	Class I	Class II	Formula	Level	score	CAS	yfm-45DPA-1	yfm-45DPA-2	45 DPA yfm-45DPA-3	88119-45DP	88119-45DPA	88119-45DP	VIP	P-value	FDR	Fold_Change	Log2FC	Type	cpd_ID	kegg_map
MW0145988	Asn-Nap-OH	Amino acids and derivatives	Amino acids and derivatives	C22H19N3O7	3	0.5105	-	53177.51	33512.87	56495.89	17318.76	13990.04	17779.81	1.68602387	0.04516439	0.34946131	2.91689396	1.54443294	up	-	-
MW0113649	6-{[3-(3,4-dihydroxyphenyl)-2-{[(2E)-3-(3,4-dihydroxyphenyl)prop-2-enoyl]oxy}propanoyl]oxy}-3,4,5-trihydroxyoxane-2-	Organic acids	Organic acids	C24H24O14	3	0.5102	-	1079.5	583.77	841.57	1993.96	1942.41	1090.69	1.39061634	0.08458374	0.44353291	0.498271355	-1.00499646	down	-	-
MW0135012	6-(4-{5,7-dihydroxy-4-oxo-6-[3,4,5-trihydroxy-6-(hydroxymethyl)oxan-2-yl]-8-(3,4,5-trihydroxyoxan-2-yl)-4H-chromen-2-yl}-2-hydroxyphenoxy)-3,4,5-trihydroxyoxane-2-carboxylic acid	Organic acids	Organic acids	C32H36O21	3	0.5082	-	41.1	47.26	39.56	94.52	97.62	74.8	1.71375071	0.01551704	0.24803772	0.479208811	-1.06127366	down	-	-
MW0139382	Plantaginin	Heterocyclic compounds	Heterocyclic compounds	C21H20O11	2	0.9208	26046-94-6	32.34	109.23	54.97	103.52	248.48	73.89	1.05645058	0.29209276	0.66139816	0.461480664	-1.11565789	down	C17056	-
MW0126018	N-[2-(5-hydroxy-1H-indol-3-yl)ethyl]hexadecanamid	Lipids	Free Lipidstty acids	C26H42N2O2	2	0.8911	212707-51-2	125272.81	118407.76	93828.72	60506.29	46051.74	44020.53	1.6726571	0.00972596	0.24036471	2.24141664	1.164410845	up	-	-
MW0108512	N-[(3s)-2-Oxotetrahydrofuran-3-Yl]hexanamide	Others	Lactones	C10H17NO3	2	0.8491	147852-83-3	32353.29	32050.5	29481.73	13844.69	13102.09	8120.86	1.66708453	0.00242324	0.18110516	2.677269414	1.420762325	up	C21197	-
MW0006843	Diisodecyl phthalate	Benzene and substituted derivatives	Benzene and substituted derivatives	C28H46O4	2	0.8421	26761-40-0 | 89-16-7 | 68515-49-1 | 19269-67-1	11293.58	5971.67	12687.94	5188.85	7201.3	1089.9	1.10507925	0.11521651	0.50205214	2.222038494	1.15188381	up	-	-
MW0169538	N-dodecanoyl-L-Homoserine lactone	Others	Lactones	C16H29NO3	2	0.8418	137173-46-7	27.38	121.75	36.58	37.32	9.75	13.61	1.16376515	0.29786691	0.66384274	3.060481213	1.613758512	up	-	-
MW0108764	Neopine	Benzene and substituted derivatives	Benzene and substituted derivatives	C18H21NO3	2	0.8401	467-14-1	117.89	393.05	130.36	72.73	75.13	50.49	1.38542809	0.24092133	0.63032074	3.233173683	1.69295101	up	C09594	-
MW0144895	Allopurinol riboside	Nucleotides and derivatives	Nucleotides and derivatives	C10H12N4O5	2	0.8279	16220-07-8	169.62	2662.43	388.14	1995.71	2553.51	2853.11	1.17012502	0.216371	0.60875495	0.435023837	-1.20083364	down	-	-
MW0137821	Cyanidin 3-sambubioside 5-glucoside	Flavonoids	Anthocyanidins	C32H39O20+	2	0.8106	73036-94-9	3211.72	1262.25	1055.56	285.05	1401.96	777.5	1.05879788	0.27555888	0.65100051	2.243663041	1.165856024	up	C20493	ko00942
MW0112142	2'-Fucosyl-D-lactose	Others	Saccharides	C18H32O15	2	0.8101	41263-94-9	17322.07	7547.77	17382.9	28421.59	61476.67	39037.71	1.49322568	0.08504165	0.47658307	0.327703278	-1.60953799	down	-	-
MW0132689	Dihydrokaempferol	Benzene and substituted derivatives	Benzene and substituted derivatives	C15H12O6	2	0.8075	104486-98-8 | 5150-32-3 | 724434-08-6	18.33	9.7	13.58	23.76	91.95	123.04	1.45309371	0.15333871	0.5475101	0.174282723	-2.52049854	down	-	-
MW0006408	Brazilein	Benzene and substituted derivatives	Benzene and substituted derivatives	C16H12O5	2	0.7901	600-76-0	631.43	669.72	591.34	1556.28	1984.69	1114.86	1.64061684	0.0658117	0.44856776	0.40647747	-1.2987527	down	-	-
Lmhp002001	Val-Phe	Amino acids and derivatives	Amino acids and derivatives	C14H20N2O3	2	0.7831	3918-92-1	8122.74	4017.88	5382.27	16347.15	56120.41	4737.76	1.03200415	0.32913193	0.68387843	0.2269648	-2.13945953	down	-	-
MW0118718	2-Methyl-3-N,N-dimethylaminomethylindole	Alkaloids	Plumerane	C12H16N2	2	0.7808	37125-92-1	4533.9	1380.46	2628.09	5114.67	7953.49	5998.31	1.36824307	0.04811725	0.40294196	0.448035216	-1.15831596	down	-	-
MW0114212	alpha-D-Galactose 1-phosphate	Others	Saccharides	C6H13O9P	2	0.7793	2255-14-3	1946.22	6825.84	5237.53	15988.74	17104.23	18297.51	1.54748582	0.00530517	0.19663582	0.272610608	-1.87508639	down	C00446	ko00052,ko00520,ko01100,ko01250
MW0127920	2,5-Dihydroxycinnamic acid	Organic acids	Organic acids	C9H8O4	2	0.7791	636-01-1 | 38489-67-7	5152.48	3386.25	10136.82	2066.83	4566.78	2035.74	1.16921522	0.23570574	0.62573958	2.154204179	1.107154997	up	-	-
MW0169534	N-benzyl-1-methyl-1H-pyrazolo[3,4-d]pyrimidin-4-amine	Benzene and substituted derivatives	Benzene and substituted derivatives	C13H13N5	2	0.777	105903-56-8	207.77	186.16	385.63	30.22	13.02	226.29	1.22947788	0.14240757	0.53210743	2.892293993	1.532214205	up	-	-
MW0106444	Dihydrofolic acid	Organic acids	Organic acids	C19H21N7O6	2	0.7769	4033-27-6	123250.93	106019.85	146974.92	25392.45	45633.54	42940.44	1.66628372	0.0069986	0.22054168	3.301373045	1.723066168	up	C00415	ko00670,ko00790,ko01100,ko01240
MW0130752	Murrangatin	Others	Ketone compounds	C15H16O5	2	0.7745	88546-96-7 | 37126-91-3	40273.13	57609.68	48418.62	20258.66	23685.22	14901.22	1.65189712	0.01420899	0.27261581	2.486212616	1.313949678	up	-	-
MW0015735	alpha-Terpineol acetate	Terpenoids	Terpene	C12H20O2	2	0.774	10581-37-0	367.17	576.25	447.69	186.83	184.78	212.03	1.67845614	0.04501864	0.39546247	2.383506956	1.253085836	up	C12300	-
MW0109339	Pro-Tyr	Amino acids and derivatives	Amino acids and derivatives	C14H18N2O4	2	0.772	19786-36-8	5850.85	1235.29	3706.15	11332.19	6457.15	15112.73	1.34798009	0.07927539	0.46796065	0.328012493	-1.60817733	down	-	-
MEDL02736	Salvigenin	Flavonoids	Flavones	C18H16O6	2	0.7706	19103-54-9	2.5	9.81	5.97	1.54	4.39	1.32	1.14306083	0.21797249	0.60939029	2.52137931	1.33421317	up	-	-
MW0007335	Phenylacetaldehyde	Phenolic acids	Phenolic acids	C8H8O	2	0.7661	122-78-1	863.54	633.75	54.31	38.79	140.25	21.53	1.17049139	0.19999041	0.59487081	7.735952535	2.951578943	up	C00601	ko00360,ko01100
MW0008581	Rodaplutin	Benzene and substituted derivatives	Benzene and substituted derivatives	C24H39N9O7	2	0.7627	108351-49-1 | 113667-06-4	1002.46	469.45	546.75	110.75	210.02	116.25	1.62889809	0.08172241	0.47136621	4.619147865	2.20762673	up	-	-

MW0000241	3-Hydroxy-8-methyl-8-azabicyclo[3.2.1]octane-2-carboxylic acid	Alkaloids	Tropan alkaloids	C9H15NO3	2	0.7618	481-37-8	3539.68	4698.51	4129.76	1280	1325.98	642.74	1.65692718	0.00283387	0.18110516	3.807022458	1.928663081	up	-	-
MW0107972	Tyr-Gly-Gly	Amino acids and derivatives	Amino acids and derivatives	C13H17N3O5	2	0.7606	21778-69-8	66723.28	56730.16	70916.31	278.84	198.3	48.74	1.73470618	0.00420596	0.18300962	369.6085609	8.529854363	up	-	-
MW0016414	Carvyl acetate	Terpenoids	Monoterpenoids	C12H18O2	2	0.7592	97-42-7	8.62	15.27	4.52	29.37	9.91	43.02	1.17825072	0.1950444	0.58845253	0.345200486	-1.5344936	down	-	-
MW0053831	Hecogenin	Terpenoids	Steroidal [illegible]	C27H42O4	2	0.7592	467-55-0	2504	2233.23	1758.15	1206.11	714.5	776.09	1.61669382	0.01163173	0.25665182	2.40864019	1.268218895	up	C08902	-
MW0106675	Ethyl N-acetyl-L-tyrosinate	Amino acids and derivatives	Amino acids and derivatives	C13H17NO4	2	0.7586	840-97-1	140900.61	91688.66	106265.96	297051.48	315300.18	266851.34	1.69830841	0.0008976	0.12883555	0.385411822	-1.37552727	down	C01657	-
MW0126471	8-Demethyl-8-(dimethylamino)riboflav[illegible]	Heterocyclic compounds	Heterocyclic compounds	C18H23N5O6	2	0.7566	51093-55-1	3940.22	5466.61	51066.82	7896.99	616.12	1826.03	1.04405001	0.3928664	0.71920029	5.849001948	2.54819047	up	C21647	ko01100,ko01110
MEDN0493	4-Methoxycinnamic [illegible]	Phenolic acids	Phenolic acids	C10H10O3	2	0.7554	943-89-5	176.7	197.76	346.61	346.25	1001.2	649.32	1.39875846	0.14542688	0.53579335	0.361118206	-1.46945694	down	-	-
MW0012920	1-Octadecyl Lysophosphatidic Acid	GL	LPA	C21H45O6P	2	0.7539	52977-29-4	115.3	105.39	79.3	137.86	456.66	171.94	1.2746825	0.26302272	0.64268675	0.391396811	-1.35329609	down	-	-
MW0159299	Val-trp	Amino acids and derivatives	Amino acids and derivatives	C16H21N3O3	2	0.7485	24587-37-9	1589.96	3337	4504.87	10937.63	31075.97	14798.26	1.57907155	0.12216961	0.50965057	0.16601868	-2.59058252	down	-	-
MW0141122	Dioleoylphosphatidylcholine	GL	PC	C44H84NO8P	2	0.7481	52088-89-8	4704.29	4186.91	18450.48	3494.79	1581.14	2334.87	1.27760364	0.29013919	0.66011135	3.689437038	1.883400696	up	-	-
MW0116568	1,2,3,6-Tetrahydrophthalimide	Alcohol and amines	Amines	C8H9NO2	2	0.7454	85-40-5	3202.72	5422.46	2968.31	4799.8	19740.27	9219.39	1.27234439	0.23500862	0.62573958	0.343414557	-1.5419769	down	-	-
MW0000153	Bicuculline	Alkaloids	Isoquinoline alkaloids	C20H17NO6	2	0.7429	485-49-4	3.71	33	64.48	172.15	40.54	121	1.10164026	0.16952923	0.56577827	0.303245527	-1.72144173	down	C09364	-
MW0112009	2,6-Dimethyl-4-hydroxybenzaldehyde	Benzene and substituted derivatives	Benzene and substituted derivatives	C9H10O2	2	0.7418	70547-87-4	114.27	278.24	93.77	74.19	14.17	13.84	1.4227201	0.14917401	0.54058806	4.758121331	2.250392061	up	-	-
MW0108317	Met-Ser	Amino acids and derivatives	Amino acids and derivatives	C8H16N2O4S	2	0.7403	14517-43-2	139504.07	96590.46	98195.75	268.6	29713.59	433.66	1.44247796	0.00579526	0.20125311	10.99066046	3.45820618	up	-	-
MW0005826	6-Methoxy-2-naphthylacetic acid	Benzene and substituted derivatives	Benzene and substituted derivatives	C13H12O3	2	0.7375	23981-47-7	4531.2	19635.83	11590.02	26844.73	91353.21	83561.2	1.46667685	0.10668725	0.49924019	0.17722642	-2.49633441	down	-	-
MW0150469	Gly-Pro-Met	Amino acids and derivatives	Amino acids and derivatives	C12H21N3O4S1	2	0.7371	109456-28-2	4214.32	6510.38	4850.06	1429.33	2862.98	1249.51	1.55843929	0.02006843	0.31365607	2.810405246	1.490778175	up	-	-
MW0127419	Butylate	Others	Lactones	C11H23NOS	2	0.7354	2008-41-5	2390.64	2316.91	2783.65	1346.55	1155.08	721.38	1.60631145	0.00448369	0.18300962	2.324286924	1.216788175	up	-	-
MW0107300	His-Glu	Amino acids and derivatives	Amino acids and derivatives	C11H16N4O5	2	0.7345	53634-28-9	4011.09	2999.99	2703.18	1969.22	269.59	1120.54	1.30546517	0.03025732	0.34952497	2.891708217	1.531921987	up	-	-
MW0153885	Methionylvaline	Amino acids and derivatives	Amino acids and derivatives	C10H20N2O3S	2	0.7343	14486-13-6	71.19	84.75	150.58	186.44	5303.81	4409.83	1.38421411	0.17991892	0.57366954	0.030961366	-5.01338707	down	-	-
MEDN1677	2'-Adenylic acid	Nucleotides and derivatives	Nucleotides and derivatives	C10H14N5O7P	2	0.7299	130-49-4	10585.04	10220.06	10788.54	4839.78	1686.33	5291.47	1.464251	0.02624231	0.33612848	2.67344414	1.418699533	up	C00946	-
MW0113991	beta-D-Glucosamine	Alcohol and [illegible]	Amines	C6H13NO5	2	0.7298	14257-69-3	78260.91	61550.62	40096.72	36558.52	14600.78	34544.77	1.3027636	0.08600578	0.47762164	2.099179771	1.069825722	up	C08349	-
MW0121428	5-Ethyl-2-nitro-9h-carbazole	Heterocyclic compounds	Heterocyclic compounds	C14H12N2O2	2	0.7253	5419-84-1	263.76	1635.44	418.44	7038.03	12144.39	15434.75	1.63244353	0.04406011	0.39546247	0.066950591	-3.90075939	down	-	-
MW0009814	Sulfometuron-methyl	Benzene and substituted derivatives	Benzene and substituted derivatives	C15H16N4O5S	2	0.7228	74222-97-2	4825.77	11286.63	8323.2	60.63	34.61	2034.76	1.46251523	0.04507729	0.39546247	11.47211268	3.520059194	up	C10955	-
MW0149468	Ganoderic acid H	Lipids	Free Lipidstty acids	C32H44O9	2	0.7207	98665-19-1	5557.46	4845.32	3096.78	14390.57	6530.56	6227.62	1.24871137	0.22593735	0.61697947	0.497244256	-1.00797339	down	-	-
MW0115288	Acetonitrile, ((2S,3R,4S,6R)-6-(beta-D-glucopyranosyloxy)-2-hydroxy-3,4-dimethoxycyclohexylidene)-, (2Z)-	Others	Saccharides	C16H25NO9	2	0.7183	51771-52-9	476.25	146.05	88.61	783.16	2394.92	503.1	1.36996224	0.23199521	0.62319286	0.193120141	-2.37242946	down	-	-
FDATN01334	Dioxybenzone	Benzene and substituted derivatives	Benzene and substituted derivatives	C14H12O4	2	0.7108	131-53-3	798780.17	400219.04	247182.58	900727.52	1629618.95	1141564.57	1.41211595	0.05543275	0.42471427	0.393849898	-1.34428219	down	-	-
MW0106509	DL-Homocysteine thiolactone	Amino acids and derivatives	Amino acids and derivatives	C4H7NOS	2	0.7051	3622-59-1	960.95	1210.88	871.04	1745.57	5079.23	2053.15	1.42927741	0.20804538	0.60236834	0.342744665	-1.54479388	down	-	-
MW0003591	(3,5-Dimethoxyphenyl)(4-methoxyphenyl)methano[illegible]	Benzene and substituted derivatives	Benzene and substituted derivatives	C16H16O4	2	0.7042	94709-12-3	500.55	746.07	844.71	1124.41	3424	738.73	1.06234524	0.33119938	0.68478989	0.395550335	-1.3380668	down	-	-
MW0010965	Secalciferol	Alcohol and [illegible]	Alcohols	C27H44O3	2	0.7034	55721-11-4	806.8	151.11	279.1	209.97	100.8	75.31	1.16233938	0.29141161	0.66130889	3.204025073	1.679885437	up	-	-
MW0009433	Phenyltoloxamine	Benzene and substituted derivatives	Benzene and substituted derivatives	C17H21NO	2	0.7004	92-12-6	29.52	426.71	3600.06	5901.94	6740.4	2321.15	1.16657043	0.11062516	0.50205214	0.27107914	-1.883214	down	-	-
MW0111391	4-O-feruloyl-D-quinic [illegible]	Organic acids	Organic acids	C17H20O9	3	0.9836	76272-56-5	101.92	243.78	229.58	1127.1	222.29	413.17	1.15991761	0.28592198	0.6560916	0.326388889	-1.61533615	down	-	-
MEDP0179	Uridine	Nucleotides and derivatives	Nucleotides and derivatives	C9H12N2O6	3	0.9817	58-96-8	33347.01	40766.72	26696.63	47853.55	91377.01	69713.25	1.48867941	0.09141408	0.48462583	0.482475935	-1.05147111	down	C00299	ko00240,ko01100,ko01232,ko02010
MEDP0160	Adenosine	Nucleotides and derivatives	Nucleotides and derivatives	C10H13N5O4	3	0.977	58-61-7	437407.59	590220.33	613424.29	804493.41	1654805.41	971534	1.4493473	0.14349206	0.53396876	0.478324738	-1.06393769	down	C00212	ko00230,ko01100,ko01232,ko02010
MEDL02489	Naringenin chalcone	Flavonoids	Chalcones	C15H12O5	3	0.9672	5071-40-9	277877.37	692640.1	2124933.47	3625241.88	6599224.24	5342539.4	1.4806317	0.0210221	0.32127574	0.19884691	-2.33026995	down	C06561	ko00941,ko01100,ko01110
MEDN1426	9(S),12(S),13(S)-TriHOME	Lipids	Free Lipidstty acids	C18H34O5	3	0.9625	97134-11-7	2795.94	1281.53	6052.51	1979.83	858.89	1049.96	1.10654157	0.27493338	0.65066506	2.604991925	1.381278901	up	C14833	ko00591

MEDL01737	5-Aminoimidazole ribonucleotide	Nucleotides and derivatives	Nucleotides and derivatives	C8H14N3O7P	3	0.9613	25635-88-5	1046079.96	709189.92	862875.52	513448.24	337923.89	388533.63	1.60800879	0.02431611	0.33365018	2.11156806	1.078314749	up	C03373	ko00230,ko00730,ko00860,ko01100,ko01110,ko01240
MEDN0198	Citric acid	Organic acids	Organic acids	C6H8O7	3	0.9411	77-92-9	48802.97	40711.01	8020.74	53723.97	74939.77	72965.38	1.12763524	0.08965193	0.48162102	0.483733302	-1.04771623	down	C00158	ko00020,ko00250,ko00630,ko01100,ko01110,ko01200,ko01210,ko01230,ko01240
MW0124001	Ethyl 4-hydroxy-5,8-dimethoxyquinoline-3-carboxylate	Others	Lactones	C14H15NO5	3	0.94	5428-19-3	61020.18	47933.89	42969.08	16216.53	1376.79	306.1	1.43531252	0.00389863	0.18188156	8.487601833	3.085356979	up	-	-
MEDP1339	LPC(18:1/0:0)	Lipids	LPC	C26H53NO7P	3	0.9376	3542-29-8	278666.85	252749.18	272123.23	48258.97	126014.8	109445.07	1.5520734	0.01167738	0.25665182	2.832167437	1.50190656	up	-	-
pmb2211	Cocamidopropyl betaine	Alkaloids	Alkaloids	C19H38N2O3	3	0.928	61789-40-0 | 4292-10-8 | 86438-79-1	76935.85	73130.9	53378.44	69780.34	8853.97	15244.7	1.19065613	0.19090143	0.58370725	2.167099866	1.115765638	up	-	-
MEDL02719	Methylparaben	Phenolic acids	Phenolic acids	C8H8O3	3	0.926	99-76-3	936.66	576.74	416.79	124.73	244.77	125.37	1.58742827	0.08124135	0.47136621	3.900398084	1.963621377	up	-	-
MEDP0151	Isoguanine	Nucleotides and derivatives	Nucleotides and derivatives	C5H5N5O	3	0.9229	3373-53-3	762.92	615.53	2979.24	300.42	549.8	392.22	1.23162392	0.30692004	0.67088493	3.507364541	1.810387386	up	-	-
MEDL00902	LPC(0:0/14:0)	Lipids	LPC	C22H46NO7P	3	0.9024	20559-16-4	48967.37	39658.76	67852.84	18722.36	25176.57	22977.89	1.61776332	0.06267143	0.44396349	2.339808771	1.226390625	up	-	-
MW0108521	N-[(R)-4-phosphopantothenoyl]-L-cysteine	Amino acids and derivatives	Amino acids and derivatives	C12H23N2O9PS	3	0.8991	7196-09-0	485.78	1138.19	187.75	1018.42	2161.55	675.01	1.0214403	0.28016129	0.65528903	0.469968716	-1.08936337	down	C04352	ko00770,ko01100,ko01240
MEDL01744	Heterophylliin A	Tannins	Tannin	C34H26O22	3	0.8984	87687-52-3	7298.12	10748.4	14334.35	25515.23	35514.84	23616.62	1.59081172	0.02392478	0.33093994	0.382541479	-1.38631191	down	-	-
MW0015017	6-Geranylgeranyl 8'-methyl 6,8'-diapocarotene-6,8'-dioate	Others	Lactones	C43H60O4	3	0.8907	247030-33-7	19188.89	7452.15	6667.86	472.8	244.09	395.25	1.71362858	0.11769934	0.50254671	29.95027604	4.904497395	up	-	-
MEDL00442	Soyasaponin I	Terpenoids	Triterpene Saponin	C48H78O18	3	0.8873	51330-27-9	34.05	24.57	25.06	1363.34	1213.98	28.03	1.24853221	0.1848816	0.57832633	0.032118525	-4.96045053	down	C08983	-
MEDL02516	L-Homoarginine	Amino acids and derivatives	Amino acids and derivatives	C7H16N4O2	3	0.8739	156-86-5	169.95	112.14	74.38	267.37	1018.41	155.78	1.20364437	0.313057	0.67242769	0.247280724	-2.01577831	down	C01924	-
MEDP1083	Oxypurinol	Heterocyclic compounds	Heterocyclic compounds	C5H4N4O2	3	0.8556	2465-59-0 | 184764-63-4 | 187486-05-1 | 187486-06-2	20399.74	29230.78	22306.45	10246.26	12625.66	2140.02	1.3197435	0.02070889	0.31868681	2.876105172	1.524116433	up	C07599	-
MEDP1271	Bis(2-ethylhexyl) phthalate	Phenolic acids	Phenolic acids	C24H38O4	3	0.8467	117-81-7 | 15495-94-0 | 8033-53-2 | 50885-87-5 | [illegible]	141608.78	431679.85	327292.4	90744.71	53666.58	41800.47	1.55850215	0.10283257	0.49377461	4.836327362	2.273911901	up	C03690	-
MEDP0881	Phosphocholine	Alkaloids	Alkaloids	C5H15NO4P+	3	0.813	107-73-3	14231.47	14465.75	9512.44	27459.45	26364.09	47250.71	1.58268477	0.08363557	0.4737568	0.378035553	-1.40340617	down	C00588	ko00564,ko01100
MEDP1675	Leu-Leu-Gly	Amino acids and derivatives	Amino acids and derivatives	C14H27N3O4	3	0.8001	4464-36-2	1795.79	16316.75	3210.01	772.42	311.77	445.16	1.49359592	0.28960671	0.66011135	13.94223036	3.801389465	up	-	-
MW0112812	3-Glucosyl-2,3',4,4',6-pentahydroxybenzophenone	Benzene and substituted derivatives	Benzene and substituted derivatives	C19H20O11	3	0.7963	92631-83-9	26702.53	4097.48	3040.18	38685.79	34075.65	10462.53	1.0868337	0.23191992	0.62319286	0.406615906	-1.29826144	down	-	-
MEDP1018	5'-Deoxyadenosine	Nucleotides and derivatives	Nucleotides and derivatives	C10H13N5O3	3	0.7941	4754-39-6	4755.01	682.04	4867.9	421.81	320.67	307.52	1.46342332	0.15426642	0.5475101	9.814238095	3.294876272	up	C05198	-
MEDP0537	LPC(O-16:0/2:0)	Lipids	LPC	C26H54NO7P	3	0.7854	74389-68-7	80003.57	70034.36	72202.77	32801.24	32875.99	21826.65	1.68646869	0.00083195	0.12883555	2.539781093	1.344704155	up	-	-
MW0138241	Sophoricoside	Flavonoids	Isoflavones	C21H20O10	3	0.7842	152-95-4	411.46	1526.46	2860	20551.44	3210.26	15156.22	1.39388282	0.1542483	0.5475101	0.123283053	-3.0199536	down	-	-
MEDP1211	N-Acetylpyrrolidine	Alkaloids	Alkaloids	C6H11NO	3	0.7837	4030-18-6	16.68	16.32	55.44	51.66	51.7	81.98	1.26345167	0.12528237	0.51699391	0.47717708	-1.06740335	down	-	-
MEDP1904	1-(9Z,12Z-octadecadienoyl)-glycero-3-phosphoethanolamine	GL	LPE	C23H44NO7P	3	0.7804	85046-18-0	10922.05	9529.41	9655.03	759.17	5699.72	5748.56	1.18847792	0.06047694	0.43825617	2.466239059	1.302312651	up	-	-
MW0111691	1,2-Cyclopentanedione	Others	Ketone compounds	C5H6O2	3	0.7798	3008-40-0	28.75	55.98	42.88	16.12	19.82	13.52	1.58870072	0.0733979	0.46133776	2.580064699	1.367407244	up	-	-
MEDP0208	Succinic acid	Organic acids	Organic acids	C4H6O4	3	0.7785	110-15-6	286.48	2910.35	308.51	140.38	83.85	104.04	1.33244183	0.34805041	0.6972136	10.6782222	3.416599569	up	C00042	ko00020,ko00190,ko00250,ko00310,ko00350,ko00360,ko00620,ko00630,ko00640,ko00650,ko00760,ko00920,ko01100,ko01110,ko01200
MEDN1397	Succinylacetone	Organic acids	Organic acids	C7H10O4	3	0.7756	51568-18-4	1027.93	96.38	1366.43	50.25	38.32	79.02	1.39588048	0.17813052	0.57366954	14.86210394	3.893566459	up	-	-
MW0000390	Oxymatrine	Alkaloids	Quinorisidine alkaloids	C15H24N2O2	3	0.7738	16837-52-8 | 54809-74-4	21.31	44.58	25.29	18.63	2.06	10.51	1.23317733	0.09129899	0.48459536	2.922435897	1.54717138	up	-	-
MEDP0289	3,4,5-Trimethoxycinnamic acid	Phenolic acids	Phenolic acids	C12H14O5	3	0.772	90-50-6	169.19	21.01	79.47	2.97	6.17	50	1.18773469	0.23998659	0.62973391	4.559857964	2.188988886	up	-	-
MEDP1475	N-Methylhydantoin	Heterocyclic compounds	Heterocyclic compounds	C4H6N2O2	3	0.7699	616-04-6	32905.17	41632.32	40537.18	13804.76	19041.88	23738.97	1.59109735	0.00802591	0.22093693	2.033638411	1.024063185	up	C02565	ko00330,ko01100
MW0126326	Pyridine-3,4-dicarboxylic acid	Organic acids	Organic acids	C7H5NO4	3	0.7679	490-11-9	88229.88	350799.76	99188.17	5236.4	12027.17	3553.35	1.65927761	0.18188541	0.57620883	25.85482434	4.692361598	up	-	-
MEDN1502	2-Phenylbutyric acid	Organic acids	Organic acids	C10H12O2	3	0.754	90-27-7	46.36	218.89	235.46	72.37	43.06	76.3	1.00169257	0.22818682	0.61968381	2.611537057	1.384899175	up	-	-
MEDL02423	Phosphocreatine	Organic acids	Organic acids	C4H10N3O5P	3	0.7528	67-07-2	8046.36	5582.94	16095.72	1058.55	1104.34	1763.56	1.65754697	0.11278041	0.50205214	7.570456774	2.92038035	up	C02305	ko00330,ko01100
MW0110480	2,6-Dihydroxypyridine	Others	Vitamin	C5H5NO2	3	0.7518	626-06-2	76.27	176.8	64.75	118.3	1329.29	1492.69	1.27713201	0.18046831	0.57412883	0.108091746	-3.20967173	down	C03056	ko00760,ko01100
MEDP0156	5-Methyluridine	Nucleotides and derivatives	Nucleotides and derivatives	C10H14N2O6	3	0.7498	1463-10-1	24618.61	19273.17	22095.37	50172.86	75870.73	36971.68	1.57161886	0.10278878	0.49377461	0.40479122	-1.3047501	down	-	-
MEDN1562	Monobutyl phthalate	Others	Lactones	C12H14O4	3	0.7481	131-70-4	72.58	189.59	69.04	63.05	38.19	22.3	1.29216331	0.21691567	0.60875495	2.68099401	1.422767996	up	-	-
MW0118307	2-benzyl-5-iodopyridazin-3(2H)-one	Benzene and substituted derivatives	Benzene and substituted derivatives	C11H9IN2O	3	0.7476	825633-93-0	5848.05	6698.82	3125.08	3021.71	847.88	304.85	1.35101716	0.05158375	0.41145636	3.754264045	1.908530121	up	-	-
MEDP0365	cotinine N-oxide	Alkaloids	Pyridine alkaloids	C10H12N2O2	3	0.7453	36508-80-2	7789	8193.24	9342.11	312.74	4858.65	3732.37	1.16913238	0.04523468	0.39546247	2.844230977	1.508038629	up	-	-
MEDL01821	3-Furoic acid	Organic acids	Organic acids	C5H4O3	3	0.744	488-93-7	3189.67	38056.97	4126.11	2398.28	1202.88	2105.31	1.18419836	0.3681574	0.70440483	7.951106376	2.991155622	up	-	-

MW0118057	2,7-Naphthyridine	Heterocyclic compounds	Heterocyclic compounds	C8H6N2	3	0.7439	253-45-2	1751.33	545.72	2552.38	4634.44	2318.54	3345.74	1.17169212	0.11164444	0.50205214	0.470876963	-1.08657795	down	-	-
MEDP1831	Ethyl acetoacetate	Others	Lactones	C6H10O3	3	0.7434	141-97-9	43.57	34.86	17.24	14.59	19.31	8.29	1.28891596	0.1346855	0.52638988	2.267598957	1.181165511	up	C03500	-
MEDP0592	3-Dehydrosphinganine	Lipids	Sphingolipids	C18H37NO2	3	0.7399	16105-69-4	73.63	371.06	108.59	1950.36	921.51	663.56	1.51951535	0.12027988	0.50624923	0.156495815	-2.67580402	down	C02934	ko00600,ko01100
MEDP1600	4-Propylphenol	Benzene and substituted derivatives	Benzene and substituted derivatives	C9H12O	3	0.7398	645-56-7	2699.6	18939.07	37718.18	55321.34	54528.81	26210.73	1.05118999	0.14054589	0.53210743	0.436252139	-1.19676589	down	C14311	-
MEDP1070	Glycyl-L-valine	Amino acids and derivatives	Amino acids and derivatives	C7H14N2O3	3	0.7381	1963-21-9	136584.22	135840.81	173472.51	99.83	29470.31	275.47	1.44821596	0.0011843	0.13399001	14.94013826	3.901121595	up	-	-
pme0088	Luteolin	Flavonoids	Flavones	C15H10O6	3	0.7374	491-70-3	1083.34	722.27	811.28	30.12	20.02	715.79	1.28696216	0.09845573	0.48888534	3.416617707	1.77256883	up	C01514	ko00941,ko00944,ko01100,ko01110
MW0146384	Astragaloside II	Terpenoids	Triterpene Saponin	C43H70O15	3	0.7359	91739-01-4	567.21	65.39	298.05	1076.87	963.83	349.86	1.09971024	0.15632936	0.5503776	0.389302088	-1.36103801	down	C17798	-
MW0009985	Trimethoprim	Phenolic acids	Phenolic acids	C14H18N4O3	3	0.7309	738-70-5	835.91	1091.38	4467.31	9315.51	14050.93	4319.71	1.40204114	0.11261012	0.50205214	0.230967469	-2.11423843	down	C01965	-
MW0116854	1-Acetyl-2-methyl-1,2,3,4-[illegible]	Heterocyclic compounds	Heterocyclic compounds	C12H15NO	3	0.7277	16078-42-5	806.28	714.75	1040.13	3007.42	4446.54	5247.78	1.70865351	0.03323066	0.35212062	0.201638516	-2.31015686	down	-	-
MW0111205	N-ethyl-2-formylhydrazinecarbothioamide	Alcohol and amines	Amines	C4H9N3OS	3	0.7276	31409-15-1	2506.05	916.75	2937.76	947.17	569.16	79.44	1.24636851	0.10790123	0.49971175	3.985887691	1.99490106	up	-	-
MEDP0298	Aminocaproic acid	Organic acids	Organic acids	C6H13NO2	3	0.7273	60-32-2	1396.51	1431.95	694.27	410.84	552.31	369.6	1.53068907	0.08653814	0.47762164	2.643203902	1.402287721	up	C02378	ko01100
MW0111931	Gallacetophenone	Phenolic acids	Phenolic acids	C8H8O4	3	0.7226	528-21-2	2367917.85	2675853.28	2702580.21	1523568.25	527.55	497.82	1.30194065	0.04963213	0.40584796	5.080928608	2.345092193	up	-	-
MEDP1076	Kojibiose	Others	Saccharides	C12H22O11	3	0.7218	2140-29-6	313.98	1765.63	402.94	2241.99	2069.53	2196.97	1.34022557	0.10207805	0.49313285	0.38143256	-1.3905001	down	C19632	-
MW0005752	Hexanoic acid, 6-[[(4-methylphenyl)sulfonyl]amino]-	Benzene and substituted derivatives	Benzene and substituted derivatives	C13H19NO4S	3	0.7209	78521-39-8	316.87	42.49	152.55	2647.11	11614.84	14473.13	1.64401318	0.11838029	0.50367581	0.01781481	-5.81077907	down	-	-
MW0004065	3-(Hydroxy(phenyl)phosphoryl)propanoic acid	Benzene and substituted derivatives	Benzene and substituted derivatives	C9H11O4P	3	0.7195	14657-64-8	6818.54	2900.05	5465.71	1207.68	1796.63	705.2	1.55688585	0.0705432	0.45866819	4.093343865	2.033279867	up	-	-
MEDL02411	5-hydroxy-L-tryptophan	Amino acids and derivatives	Amino acids and derivatives	C11H12N2O3	3	0.7193	4350-09-8	16780.69	7248.15	19661.59	11350.49	3753.43	1459.95	1.18279873	0.13602104	0.52749511	2.637694573	1.39927752	up	C00643	ko00380,ko01100
MEDP0846	4-Methylbenzoic acid	Phenolic acids	Phenolic acids	C8H8O2	3	0.7189	99-94-5	6859.6	5232.05	6106.3	8662.37	18275.76	11267.4	1.46812933	0.14242841	0.53210743	0.476317172	-1.07000553	down	C01454	ko01100
MEDP1478	4-Pentenoic acid	Organic acids	Organic acids	C5H8O2	3	0.717	591-80-0	99.35	112.92	57.94	81.55	30.24	7.74	1.12013123	0.14591664	0.53579335	2.260604032	1.176708312	up	-	-
MEDP1286	Geranyl acetate	Others	Lactones	C12H20O2	3	0.7166	105-87-3	488.38	845.52	3124.31	255.28	410.9	749.81	1.0477983	0.34379548	0.69543658	3.148475625	1.654653498	up	C09861	-
MEDP1884	Prolyl-Histidine	Amino acids and derivatives	Amino acids and derivatives	C11H16N4O3	3	0.7165	92027-43-5	3674.16	3623.78	2515.41	1256.9	408.54	1958.31	1.35826688	0.02561228	0.33612848	2.70806485	1.437262288	up	-	-
MW0153387	Manniflavanone	Others	Ketone compounds	C30H22O13	3	0.7122	73428-17-8	189013.17	103888.88	145327.1	350150.81	346724.34	307025.5	1.62884246	0.00583205	0.20125311	0.436526413	-1.19585914	down	C09763	-
MEDP1183	N-Acetylcadaverine	Alkaloids	Alkaloids	C7H16N2O	3	0.7105	32343-73-0	17637.31	12305.64	22369.61	38123	37365.47	30290.4	1.56558136	0.01018146	0.24391477	0.494546406	-1.01582219	down	-	-
MADP0540	2-Pyrimidin-2-yl-propionic acid	Heterocyclic compounds	Heterocyclic compounds	C7H8N2O2	3	0.7091	819850-16-3	34636.82	9529.56	12645.8	262.04	157.01	18.82	1.66276342	0.14046404	0.53210743	129.7466828	7.019553844	up	-	-
MW0148998	L-erythrulose 1-[illegible]	Organic acids	Organic acids	C4H9O7P	3	0.7089	16409-92-0	71177.14	36024.38	66377.72	191679.52	217774.5	150819.74	1.63672411	0.00901592	0.23644112	0.309811475	-1.69053751	down	C03394	-
MADN0596	Serine alanine	Amino acids and derivatives	Amino acids and derivatives	C6H12N2O4	3	0.7077	6403-17-4	353	210.63	130.03	88.02	52.4	17.5	1.44242816	0.10048511	0.49047358	4.392477204	2.135034799	up	-	-
MW0133346	4-Dimethylaminocinnamaldehyde	Others	Aldehyde compounds	C11H13NO	3	0.7073	6203-18-5 | 20432-35-3	3082.44	2974.05	4193.29	7192.72	5743.46	9080.68	1.60500765	0.04090904	0.38991154	0.465542316	-1.10301579	down	-	-
MW0119848	4-(2-Hydroxyethyl)piperazin-1-ylethanesulphonic [illegible]	Benzene and substituted derivatives	Benzene and substituted derivatives	C8H18N2O4S	3	0.7065	7365-45-9	3093.6	93.99	396.04	99.2	79.34	91.72	1.12135121	0.36636653	0.70436456	13.25993488	3.729001785	up	-	-
MW0108809	Nitroarginine	Amino acids and derivatives	Amino acids and derivatives	C6H13N5O4	3	0.705	2149-70-4	119.05	67.58	32.63	33.17	25.13	18.28	1.32681914	0.19568433	0.58918	2.863149647	1.517603077	up	C03417	-
MW0104489	2-amino-3-(1H-pyrazol-1-yl)propanoic acid	Organic acids	Organic acids	C6H9N3O2	3	0.6998	2734-48-7	150080.22	79728.56	108116.44	286071.11	328875.8	269567.99	1.64520241	0.00271256	0.18110516	0.38204582	-1.38818242	down	-	-
MW0112116	2-Deoxy-D-ribose	Others	Saccharides	C5H10O4	3	0.6997	533-67-5	94.54	2466.65	160.36	30.62	11.89	52.99	1.33099392	0.3783735	0.71084714	28.49790576	4.832783998	up	-	-
MW0110229	H-Tyr-tyr-tyr-OH	Amino acids and derivatives	Amino acids and derivatives	C27H29N3O7	3	0.6989	7390-78-5	361.56	507.16	452.32	954.29	1471.94	1195.89	1.67687848	0.02843489	0.33858973	0.364714587	-1.45516019	down	-	-
MW0127304	3-Mercapto-2-methyl-1-butanol	Benzene and substituted derivatives	Benzene and substituted derivatives	C5H12OS	3	0.6976	227456-33-9	1114.27	1918.05	4227.77	6918.07	4166.89	5890.41	1.34056418	0.05945233	0.4369697	0.427683756	-1.22538368	down	-	-
MW0160835	[2-(6-Amino-9H-purin-9-YL)-1-methylethoxy]methyl-triphosphate	Organic acids	Organic acids	C9H16N5O10P3	3	0.6972	166403-66-3	479.22	205.31	682.52	167.94	15.41	71.05	1.37606091	0.10407184	0.49596385	5.373624214	2.425895435	up	-	-
MW0004432	2,2-Bis(4-(3,4-dicarboxyphenoxy)phenyl)propane dianhydride	Benzene and substituted derivatives	Benzene and substituted derivatives	C31H20O8	3	0.6963	38103-06-9	1127.16	1618.68	3816.38	63.61	195.42	372.56	1.56596545	0.1377008	0.5286681	10.38999984	3.377123727	up	-	-
MW0015861	Arachidonylcyclopropylamide	Benzene and substituted derivatives	Benzene and substituted derivatives	C23H37NO	3	0.6934	229021-64-1	22491.32	12678.43	14453.84	12608.84	2847.6	4465.92	1.32357911	0.0812811	0.47136621	2.490848976	1.316637551	up	-	-
MEDL02667	Perlolyrine	Alkaloids	Plumerane	C16H12N2O2	3	0.6932	29700-20-7	45519.6	32817.69	67291.21	26955.91	13254.67	18656.75	1.47928875	0.08765038	0.47802518	2.473842452	1.306753625	up	C09231	-
MW0126750	Thalidomide arene [illegible]	Alcohol and [illegible]	Amines	C13H10N2O5	3	0.6928	1488-25-1	13456.97	5502.78	12801.66	19726.67	21781.28	36158.17	1.41738832	0.07892952	0.46796065	0.408948072	-1.29001043	down	-	-
MEDL01977	psi-Pelletierine	Alkaloids	Piperidine alkaloids	C9H15NO	3	0.6909	552-70-5	6810.67	6679.46	6459.54	3189.07	2596.76	1830.23	1.67242737	0.00619323	0.20791718	2.619421328	1.389248133	up	C10865	ko00960,ko01110

MW0003383	4-(pyridin-4-yl)-N-(m-tolyl)thiazol-2-amine	Benzene and substituted derivatives	Benzene and substituted derivatives	C15H13N3S	3	0.6902	315702-99-9	33582.25	19158.73	9354.92	329.03	71.35	5836.82	1.40659418	0.10955167	0.50091108	9.955733342	3.315527589	up	-	-
MW0131207	3,5-Dimethoxycinnamic acid	Organic acids	Organic acids	C11H12O4	3	0.6892	16909-11-8 | 20767-04-8	29.24	61.98	27.91	13.99	31.57	8.84	1.20948781	0.18885321	0.58166266	2.189889706	1.13085821	up	-	-
MEDN1096	N-Benzoyl-L-tyrosine ethyl ester	Amino acids and derivatives	Amino acids and derivatives	C18H19NO4	3	0.6885	3483-82-7	637.87	554.81	934.18	48.09	140.99	287.44	1.48820409	0.02212161	0.32980774	4.463317384	2.1581164	up	-	-
MEDP1681	Leu-Gly-Leu	Amino acids and derivatives	Amino acids and derivatives	C14H27N3O4	3	0.6819	19408-48-1	84.62	169.59	132.55	163.42	451.12	634.42	1.33728152	0.16734463	0.56572953	0.309665642	-1.69121677	down	-	-
MEDP2164	2,2-Dihydroxymethyl-1-azabicyclo[2.2.2]octan-3-one	Heterocyclic compounds	Heterocyclic compounds	C9H15NO3	3	0.6807	5608-24-2	955.91	609.18	1824.87	202.58	605.13	279.62	1.37841838	0.15792451	0.5507933	3.117691961	1.640478392	up	-	-
MEDP1605	Glu-glu	Amino acids and derivatives	Amino acids and derivatives	C10H16N2O7	3	0.6807	3929-61-1	24235.59	9561.87	14803.87	673.25	113.74	110.11	1.68392801	0.06551255	0.44856776	54.17604503	5.759583172	up	C01425	-
MW0000006	Hyoscyamine	Alkaloids	Tropan alkaloids	C17H23NO3	3	0.68	101-31-5	38980.7	56325.45	60299.87	25170.92	21637.48	7780.35	1.45377673	0.01756673	0.29823307	2.850514437	1.511222309	up	C02046	ko00960,ko01100,ko01110
MW0004101	3-Methoxybenzyl alcohol	Benzene and substituted derivatives	Benzene and substituted derivatives	C8H10O2	3	0.6724	6971-51-3	120902.03	65165.58	123455.34	51.56	185.52	53864.25	1.31566351	0.03128349	0.34952497	5.72117081	2.516310418	up	C03352	-
MEDN0622	Porphobilinogen	Organic acids	Organic acids	C10H14N2O4	3	0.672	487-90-1	6266.33	7367.51	5421.7	38.67	435.65	521.95	1.58014889	0.0058334	0.20125311	19.12688327	4.257529901	up	C00931	ko00860,ko01100,ko01110,ko01240
MW0105135	1-propyl-2-sulLipidsnyl-4,5-dihydro-1H-imidazol-5-one	Organic acids	Organic acids	C6H10N2OS	3	0.6706	65710-56-7	42458.21	41544.84	15241.74	10913.26	3517.79	4837.83	1.5259079	0.08805765	0.4783049	5.150521982	2.36471865	up	-	-
MEDL00369	Glutamyl-phenylalanine	Amino acids and derivatives	Amino acids and derivatives	C14H18N2O5	3	0.6703	20556-22-3	2793.53	2655.27	2102.66	355.3	1187.9	810.36	1.52640339	0.0059253	0.2013045	3.208526657	1.68191097	up	-	-
MW0114378	D-xylulose	Others	Saccharides	C5H10O5	3	0.6684	551-84-8 | 5962-29-[illegible]	1629.57	20814.56	966.24	234.9	237.35	272.18	1.41733894	0.36552629	0.7041771	31.44737584	4.97486773	up	-	-
MW0053847	Hellebrin	Heterocyclic compounds	Heterocyclic compounds	C36H52O15	3	0.667	13289-18-4	384.71	29.53	34.61	24.69	1.47	16.63	1.1687967	0.36859384	0.70440483	10.48960037	3.390887811	up	C08868	-
MEDL02128	Pyridoxal phosphate	Others	Vitamin	C8H10NO6P	3	0.6634	54-47-7	68002.07	69815.9	63471.6	31774.1	25764.44	35900.83	1.70493226	0.00110667	0.13399001	2.154226532	1.107169967	up	C00018	ko00730,ko00750,ko01100,ko01240
MEDN0553	2-Methylbenzoic acid	Phenolic acids	Phenolic acids	C8H8O2	3	0.6624	118-90-1	11.49	18.7	10.24	323.98	9.54	402.23	1.14847216	0.19310697	0.58740569	0.054950731	-4.18571753	down	C07215	ko01100
MEDL01832	(-)-Arctigenin	Lignans and Coumarins	Lignans	C21H24O6	3	0.6618	7770-78-7	26226.32	15353.95	6521.42	3716.59	5955.61	5792.26	1.33307133	0.19476965	0.5880239	3.110466838	1.637131125	up	C10545	-
MEDP0215	Quinine	Alkaloids	Quinoline [illegible]	C20H24N2O2	3	0.6612	130-95-0	8006.03	9712.32	24604.19	62222.25	92060.86	33886.16	1.51821005	0.09033368	0.48325238	0.224917384	-2.15253292	down	C06526	-
MW0114766	LS-Tetrasaccharide b	Others	Saccharides	C37H62N2O29	3	0.6599	64003-54-9	18514.4	17955.35	24602.08	7931.26	9926.03	5433.31	1.6286277	0.01177562	0.25665182	2.622166453	1.39075927	up	-	-
MEDL02636	Ephedrine	Alkaloids	Alkaloids	C10H15NO	3	0.6594	299-42-3	2238.52	7211.58	2167.89	14085.64	21276.5	12833.43	1.54615288	0.02374657	0.33093994	0.241059292	-2.05254005	down	C01575	ko00996,ko01100,ko01110
MW0118261	2-Amino-5-[illegible]	Alkaloids	Pyridine [illegible]	C11H10N2	3	0.6593	33421-40-8	3853.51	4822.16	1865.82	8214.53	9806.4	8288.57	1.51501843	0.01126178	0.25665182	0.400672381	-1.31950503	down	-	-
MW0103864	(S)-2-Amino-2-(2,3-dihydro-1H-inden-2-yl)acetic acid	Organic acids	Organic acids	C11H13NO2	3	0.6579	155239-51-3	357.73	562.49	421.66	1083.56	863.3	2049.65	1.53535458	0.13226099	0.52284849	0.335762953	-1.57448504	down	-	-
MEDL00392	1-(9Z-octadecenoyl)-sn-glycero-3-[illegible]	Lipids	LPC	C26H52NO7P	3	0.6572	19420-56-5	5497.38	6869.81	6464.43	1011.9	2842.34	1500.89	1.60204997	0.00361952	0.18110516	3.516557021	1.814163611	up	-	-
MW0000324	Lupinine	Alkaloids	Alkaloids	C10H19NO	3	0.6561	486-70-4 | 10248-30-3	59.77	23.05	54.84	900.9	2034.91	1398.57	1.72077421	0.05070973	0.40876228	0.031760021	-4.97664432	down	-	-
MEDN0576	1,2,3-[illegible]	Phenolic acids	Phenolic acids	C6H6O3	3	0.6546	87-66-1	648.52	2691.67	2139.67	2789.38	6651.59	4306.2	1.2449047	0.11757995	0.50254671	0.398617315	-1.32692371	down	C01108	-
MW0108799	(2S,4R)-1-hexadecyl-4-hydroxypyrrolidine-2-carboxylic acid	Amino acids and derivatives	Amino acids and derivatives	C21H41NO3	3	0.6503	76652-69-2	15.58	3.84	91.31	13.24	1.47	1.71	1.0401907	0.37001337	0.70479083	6.743605359	2.753520111	up	-	-
MEDP1505	5-Methoxytryptophan	Amino acids and derivatives	Amino acids and derivatives	C12H14N2O3	3	0.6502	2504-22-5	5051.36	7265.4	9820.99	9980.98	17279.04	20808.36	1.39744698	0.09722032	0.48888534	0.460547037	-1.11857958	down	-	-
MW0118712	Methyl 2-methyl-3-furyl disulfide	Heterocyclic compounds	Heterocyclic compounds	C6H8OS2	3	0.6499	65505-17-1	47402.06	50288.62	50630.22	16568.27	250.53	14391.96	1.19492002	0.01398416	0.27083212	4.752236088	2.24860651	up	-	-
MW0001942	2-(4-Methoxyphenoxy)acetohydrazide	Phenolic acids	Phenolic acids	C9H12N2O3	3	0.6496	21953-91-3	10949.04	6828.22	8607.13	715.98	378.16	412.04	1.73644873	0.01940073	0.31365607	17.51742156	4.130718531	up	-	-
MW0006111	Azilsartan	Benzene and substituted derivatives	Benzene and substituted derivatives	C25H20N4O5	3	0.6494	147403-03-0	68024.18	70523.95	50665.46	78805.56	214586.05	117415.59	1.31973792	0.20669096	0.60108551	0.460589761	-1.11844576	down	-	-
MW0006323	Benzylamine	Alkaloids	Alkaloids	C7H9N	3	0.6472	100-46-9	1588.87	1721.58	2418.39	1192.4	204.55	1110.14	1.18063275	0.06046971	0.43825617	2.285055582	1.192229258	up	C15562	-
MEDN0658	Hexadecanedioic acid	Lipids	Free Lipidstty acids	C16H30O4	3	0.6469	505-54-4	13538	16460.02	13348.55	7660.57	4715.8	7816.34	1.6043657	0.00563529	0.20125311	2.146644507	1.102083295	up	C19615	ko00073,ko01100
MW0008211	N-(3-hydroxyphenyl)-Arachidonoyl amide	Benzene and substituted derivatives	Benzene and substituted derivatives	C26H37NO2	3	0.6465	183718-75-4	5595.45	5561.38	4132.49	749.86	1345.85	557.74	1.68378117	0.00478571	0.18937746	5.762053176	2.526582975	up	-	-
MW0054292	Lauroyl diethanolamide	Lipids	Others	C16H33NO3	3	0.6438	120-40-1	2822.91	3435.5	4769.84	1567.13	1729.18	601.55	1.46802925	0.03301125	0.35164075	2.829308903	1.500449698	up	-	-
MW0153988	Muscapurpurin	Heterocyclic compounds	Heterocyclic compounds	C18H14N2O10	3	0.643	12624-19-0	73558.75	111681.85	114399.13	633.01	26129.48	2716.66	1.47172702	0.0075127	0.22054168	10.16446302	3.345462096	up	C08563	-
MEDL02503	Carnitine C16:0	Alkaloids	Alkaloids	C23H45NO4	3	0.6426	2364-67-2	53.63	35.76	25.52	190.82	76.6	44.49	1.18494888	0.27552825	0.65100051	0.368407553	-1.44062545	down	C02990	ko00071,ko01212
MW0129527	1-(2-Hydroxy-4-methoxyphenyl)-3-phenylpropan-1-one	Others	Ketone compounds	C16H16O3	3	0.6416	22141-31-7	4881.1	3571.41	10143.32	13705.99	80283.81	9329.14	1.19712811	0.34351627	0.69542972	0.179984715	-2.4740537	down	-	-
MW0142566	Propargyl alcohol	Others	Hydrocarbon derivatives	C3H4O	3	0.6401	107-19-7 | 30306-19-5	2342.5	2455.88	3320.11	1033.47	926.41	266.9	1.49483199	0.00859802	0.23235632	3.645842876	1.866252387	up	C05986	-

MEDP0711	4-Ethyl-2-[illegible]	Phenolic acids	Phenolic acids	C9H12O2	3	0.6374	2785-89-9	18.54	265.74	186.98	1246.38	3388.23	1002.43	1.47274199	0.1498218	0.54119721	0.083600613	-3.58034267	down	-	-
MW0052594	Ercalcitriol	Alcohol and [illegible]	Alcohols	C28H44O3	3	0.6345	60133-18-8	2.2	9.12	4.21	1.04	0.99	2.47	1.33405848	0.21110801	0.6050101	3.451111111	1.787060923	up	-	-
MW0143552	5alpha-Cyprinol	Alcohol and [illegible]	Alcohols	C27H48O5	3	0.6334	2952-70-7	399.11	226.78	414.17	591.98	489.45	1858.36	1.22421535	0.28594953	0.6560916	0.353787175	-1.49904634	down	C16890	-
MW0109969	Thr-Ser	Amino acids and [illegible]	Amino acids and derivatives	C7H14N2O5	3	0.631	61043-86-5	109.93	325.32	1045.23	5010.06	9764.91	1261.92	1.42956315	0.18587619	0.57833032	0.092317151	-3.43725748	down	-	-
MEDN0806	Aceglutamide	Amino acids and derivatives	Amino acids and derivatives	C7H12N2O4	3	0.6302	2490-97-3	32.57	9.79	15.91	75.49	114.29	52.4	1.54308531	0.06246009	0.44396349	0.240606161	-2.05525451	down	-	-
MW0153878	Met-Thr	Amino acids and derivatives	Amino acids and derivatives	C9H18N2O4S	3	0.6285	40883-16-7	122895.12	171507.02	107932.99	64157.12	58429.06	59242.35	1.65672547	0.06071509	0.43825617	2.212717278	1.145819128	up	-	-
MEDL00367	Glutaminylphenylalanine	Amino acids and derivatives	Amino acids and derivatives	C14H19N3O4	3	0.6243	92327-75-8	86529.88	101740.93	106931.98	39157.7	17922.91	29147.47	1.65206581	0.00129999	0.13399001	3.423511111	1.775476695	up	-	-
ZINC8548712	Agrimol F	Phenolic acids	Phenolic acids	C34H40O12	3	0.6228	121693-16-1	112.97	140.49	93.59	303.06	622.48	323.61	1.6256176	0.09794655	0.48888534	0.277828924	-1.84773129	down	-	-
MW0138164	Fisetinidol	Flavonoids	Flavanonols	C15H14O5	3	0.6203	490-49-3	17983.64	17575.9	19090.82	1792.48	1071.3	799.81	1.73576175	2.3156E-05	0.03690435	14.91716049	3.898901037	up	C09735	-
MW0103342	2'-Deoxycytidine-5'-triphosphate	Nucleotides and derivatives	Nucleotides and derivatives	C9H16N3O13P3	3	0.6183	2056-98-6	5192.53	527.62	7318.13	15803.39	10678.54	11403.71	1.19045543	0.03433008	0.3606623	0.344148337	-1.53889756	down	C00458	ko00240,ko01100,ko01232
MW0161291	K252a	Others	Lactones	C27H21N3O5	3	0.6174	171333-67-8	5898.47	2939.46	3961.82	1830.98	1157.95	464	1.4829953	0.0518486	0.4118154	3.706924264	1.89022264	up	-	-
MEDL01873	[8]-Gingerdione	Phenolic acids	Phenolic acids	C19H28O4	3	0.6169	77334-06-6	327.62	297.01	309.57	195	196.93	7.66	1.02200686	0.1021138	0.49313285	2.337896344	1.225210966	up	-	-
MEDP1304	2-Piperidinone	Alkaloids	Piperidine alkaloids	C5H9NO	3	0.6162	675-20-7	1351.96	17496.74	1831.68	1063.29	833.49	1063.08	1.0931382	0.38128258	0.71355082	6.986945328	2.804661852	up	-	-
MW0011806	1-(3-Methyl-2-butenoyl)-6-[illegible]	Others	Saccharides	C16H26O11	3	0.6153	467242-32-6	4177.41	4979.34	5985.29	4996.9	640.61	565.25	1.23744847	0.16930247	0.56577827	2.441177798	1.287577375	up	-	-
MW0110392	Valylcysteine	Amino acids and derivatives	Amino acids and derivatives	C8H16N2O3S	3	0.6148	160070-15-5	7371.39	9933.35	5202.67	38566.55	29948.21	31071.34	1.70110859	0.00362983	0.18110516	0.226009554	-2.14554434	down	-	-
MW0119700	3-Methyl-4-nitro-1-(4-nitrophenyl)-1H-pyrazol-5-ol	Benzene and substituted derivatives	Benzene and substituted derivatives	C10H8N4O5	3	0.6144	132-42-3	1006.6	2256.76	544.44	3005.21	2814.52	2642.33	1.33569081	0.08810492	0.4783049	0.449984992	-1.15205121	down	-	-
MW0168258	Dihydrorobinetin	Flavonoids	Flavones	C15H12O7	3	0.614	4382-33-6 | 93432-80-5	388699.63	1544250.5	425928.44	99314.41	86452.95	83997.47	1.60360577	0.20763574	0.60235963	8.744203498	3.128326976	up	-	-
MW0148550	Dimethylallyl phosphate	Organic acids	Organic acids	C5H11O4P	3	0.6125	10379-43-8	227.62	2438.77	98.02	104.11	43.7	63.64	1.16467627	0.37903263	0.71090673	13.07358714	3.708583138	up	C21214	ko00740
MW0132715	2,3-Dihydro-2-(3,4-dihydroxyphenyl)-3,5-bis(3,4,5-trihydroxybenzoyloxy)-4H-1-benzopyran-7-ol	Flavonoids	Flavones	C29H22O14	3	0.6058	37484-74-5	5246.34	4875.49	7483.95	582.79	2091.08	2061.05	1.50140548	0.0166285	0.29597391	3.718284575	1.89463719	up	-	-
MW0125337	N-(2-Oxo-1-propyl-1,2,3,4-tetrahydroquinolin-6-yl)-1-(p-tolyl)methanesulfonami[illegible]	Alcohol and amines	Amines	C20H24N2O3S	3	0.604	946270-26-4	600.79	591.57	1115.39	2934.16	2357.74	4422.02	1.64352567	0.04797061	0.40294196	0.237571444	-2.07356666	down	-	-
MEDL01767	Damascenone	Others	Ketone compounds	C13H18O	3	0.6039	23696-85-7	900.69	603.36	1004.73	623.36	42.78	93.63	1.30675061	0.06782753	0.45272832	3.302025613	1.723351311	up	-	-
MEDP0313	Glycocyamine	Alkaloids	Alkaloids	C3H7N3O2	3	0.6034	352-97-6	60268.85	58952.29	98377.21	18111.53	44001.36	41355.33	1.34829638	0.07929568	0.46796065	2.103045264	1.072479901	up	C00581	ko00260,ko00330,ko01100
MEDP0652	Methyl cinnamate	Benzene and substituted derivatives	Benzene and substituted derivatives	C10H10O2	3	0.603	103-26-4 | 1754-62-7	6935.49	549.27	1870.17	2600.26	7569.67	18724.59	1.02210026	0.30577116	0.67088493	0.323761391	-1.62699714	down	C06358	-
MEDTP0254[illegible]	5-(6-Hydroxy-2,5,7,8-tetramethyl-chroman-2-YL)-2-methyl-pentanoic acid	Organic acids	Organic acids	C19H28O4	3	0.6006	7083-09-2	212.78	307.48	417.13	1202.68	2044.23	606.4	1.50780007	0.14251762	0.53210743	0.243268774	-2.03937695	down	-	-
MW0150339	Gly-Gln-Gln	Amino acids and derivatives	Amino acids and derivatives	C12H21N5O6	3	0.6006	627882-94-4	6052.82	1152.73	3850.54	15654.91	12645.41	8601.44	1.40679303	0.03093114	0.34952497	0.299608745	-1.73884836	down	-	-
MEDL01926	Albanin A	Flavonoids	Flavones	C20H18O6	3	0.6	73343-42-7	877.65	1387.75	788.73	477.9	832.68	197.32	1.19313419	0.12052786	0.50681166	2.025419458	1.018220716	up	-	-
MW0157291	SulLipidszecin	Amino acids and derivatives	Amino acids and derivatives	C12H20N4O9S	3	0.5979	77912-79-9	307.57	993.96	600.37	496.18	88.12	318.01	1.00752128	0.23849542	0.6289157	2.107812171	1.075746313	up	C20927	ko00261
MW0124805	N-(3-(9H-carbazol-9-yl)-2-hydroxypropyl)-N-(furan-2-ylmethyl)methanesulfonamide	Alcohol and amines	Amines	C21H22N2O4S	3	0.5948	309928-48-1	4071.23	3555.42	8616.19	157.98	181.05	168.38	1.73823107	0.08250366	0.47220697	32.01127294	5.000508142	up	-	-
MW0115052	(6aR,9R,9aR)-9-acetyl-3-[(1E,3E,5S)-3,5-dimethylhepta-1,3-dienyl]-6a-methyl-9,9a-dihydrofuro[2,3-h]isochromene-6,8-dione	Others	Ketone compounds	C23H26O5	3	0.5926	53014-38-3	3801.07	1353.61	4110.33	41.55	136.52	174.16	1.66566129	0.0761014	0.46149681	26.30386395	4.717202837	up	-	-
MEDP0359	Curcumin	Phenolic acids	Phenolic acids	C21H20O6	3	0.5907	458-37-7	9448.53	11470.37	11327.59	4562.37	2688.45	1637.17	1.61159098	0.00250007	0.18110516	3.628097016	1.859213034	up	C10443	ko00945,ko01110
MEDL01845	Homodihydrocapsaicin	Alkaloids	Phenolamine	C19H31NO3	3	0.5887	20279-06-5	133.63	168.12	359.44	75.54	216.24	28.12	1.03267893	0.27835398	0.65377045	2.066864645	1.047443913	up	-	-
MW0126336	Pyridoxamine phosphate	Organic acids	Organic acids	C8H13N2O5P	3	0.5886	529-96-4	366860.16	471025.67	419163.55	1900.48	1913.42	4451.37	1.75041827	0.00514941	0.19663582	152.0881205	7.24876366	up	C00647	ko00750,ko01100
MEDL02754	2,6-Dimethoxy-1,4-benzoquinone	Quinones	Quinones	C8H8O4	3	0.5827	530-55-2	26935.36	15489.3	22870.06	191.05	49.6	91.95	1.73982518	0.02309393	0.33077008	196.3160553	7.617034355	up	C10331	-
MW0001326	2-(4-Iodo-2,5-dimethoxyphenyl)-N-((2,3-methylenedioxyphenyl)methyl)ethanamine	Benzene and substituted derivatives	Benzene and substituted derivatives	C18H20INO4	3	0.5785	919797-25-4	87.07	45.35	49.1	139.26	433.08	274.67	1.56169255	0.11724367	0.50205214	0.214306797	-2.22225049	down	-	-

MW0012351	13-Tetradecynoic acid	Lipids	Free Lipidstty acids	C14H24O2	3	0.5781	82909-47-5	775.28	40.37	72.99	2015.73	1483.51	1145.71	1.41159607	0.0230628	0.33077008	0.191313147	-2.38599208	down	-	-
MW0123976	Ethyl 4-(1-hydroxy-1-methylethyl)-2-propyl-imidazole-5-carboxylate	Others	Lactones	C12H20N2O3	3	0.5772	144689-93-0	57.75	65.34	160.81	74.46	608.06	397.06	1.08818906	0.22608013	0.61697947	0.262972638	-1.9270154	down	-	-
MEDP0668	m-Cresol	Phenolic acids	Phenolic acids	C7H8O	3	0.5734	108-39-4	5802.9	6530.21	8113.49	987.33	3830.16	2950.87	1.39519051	0.01890176	0.30912398	2.632035591	1.396178997	up	C01467	-
MEDL02512	6,7,4'-Trihydroxyisoflavone	Flavonoids	Isoflavones	C15H10O5	3	0.5732	17817-31-1	11931.06	18889.23	13900.64	30440.96	34033.16	46794.38	1.62829926	0.0320867	0.35051264	0.401919052	-1.31502313	down	C14314	ko00943,ko01110
MEDP0650	4,4'-Methylenedianiline	Alkaloids	Alkaloids	C13H14N2	3	0.5727	101-77-9	45.87	1746.21	181.97	26.45	23.79	60.23	1.18424706	0.37283134	0.70675299	17.86955735	4.159431992	up	C14288	-
MW0110854	5-(2-aminoethyl)-1H-imidazol-2-amine	Alcohol and amines	Amines	C5H10N4	3	0.571	39050-13-0	3837.63	2109.42	9104.16	20899.29	34544.06	38148.75	1.59494122	0.02533336	0.33603105	0.160817099	-2.63650729	down	-	-
MW0148433	Dibenzyl succinate	Benzene and substituted derivatives	Benzene and substituted derivatives	C18H18O4	3	0.5701	103-43-5	25792.37	28195.96	24557.85	20.46	190.43	10.78	1.70337327	0.00162459	0.14694232	354.3383408	8.468983769	up	C07332	-
MW0114989	Neohesperidose heptaacetate	Others	Saccharides	C26H36O17	3	0.5645	19949-47-4	464.18	116.58	558.56	88.57	68.17	33.07	1.4485521	0.14076946	0.53210743	6.002423476	2.585545106	up	-	-
MW0146896	Bryotoxin A	Others	Others	C32H42O12	3	0.5644	101329-50-4	205.21	281.69	193.16	684.65	486.88	671.04	1.67557966	0.01459429	0.27628422	0.369082314	-1.43798549	down	C08853	-
MW0061611	Phorbol 12-myristate 13-acetate	Heterocyclic compounds	Heterocyclic compounds	C36H56O8	3	0.5642	16561-29-8 | 11016-13-0	10085.66	5052.05	10933.83	16495.98	28195.33	43541.05	1.49680444	0.1112231	0.50205214	0.295487279	-1.75883207	down	C05151	-
MW0133028	3 -Galloylprodelphinidin [illegible]	Flavonoids	Anthocyanidins	C37H30O18	3	0.5615	86588-88-7	1713.41	1673.13	1500.87	850.65	1001.81	92.25	1.15204188	0.06659693	0.44856776	2.513181914	1.329515103	up	-	-
ZINC1335935	Ancistrocladidine	Heterocyclic compounds	Heterocyclic compounds	C25H27NO4	3	0.5555	52659-52-6	6961.58	966.52	6208.89	8070.9	35770.79	16438.12	1.26279073	0.19694172	0.59081764	0.234522803	-2.09219989	down	-	-
MW0110369	L-Arginine, L-valyl-L-threonyl-	Amino acids and derivatives	Amino acids and derivatives	C15H30N6O5	3	0.5533	362603-27-8	5201.44	740.16	1530.75	16428.1	17719.42	13199.39	1.55771213	0.00230037	0.18110516	0.157821281	-2.66363634	down	-	-
MW0109543	S-(2,4-dinitrophenyl)glutathion	Amino acids and derivatives	Amino acids and derivatives	C16H19N5O10S	3	0.5531	26289-39-4	1742.9	3879.68	4145.02	1323.73	685.02	746.84	1.53987835	0.08276212	0.47220697	3.54464924	1.825642873	up	C11175	-
MW0007885	N-(1-Hydroxy-2-oxo-2-(2-thienyl)ethyl)benzenesulfonamide	Benzene and substituted derivatives	Benzene and substituted derivatives	C12H11NO4S2	3	0.5513	303104-25-8	25782.77	39280.1	27731	1619.59	2292.93	1633.64	1.74879457	0.0200989	0.31365607	16.73119239	4.064468361	up	-	-
MEDL02650	Cyclomorusin	Flavonoids	Other Flavonoids	C25H22O6	3	0.5499	62596-34-3	312.44	3158.82	531.16	132.85	181.3	206.63	1.30242936	0.33211517	0.68525811	7.685433388	2.942126617	up	C17867	-
MW0109398	Pro-Lys	Amino acids and derivatives	Amino acids and derivatives	C11H21N3O3	3	0.5499	71227-70-8	2359.48	575.39	1475.13	622.38	534.21	535.09	1.21869247	0.22028045	0.61059856	2.606876005	1.382321963	up	-	-
MW0014209	3-O-a-L-Fucopyranosyl-D-glucose	Organic acids	Organic acids	C12H22O10	3	0.5495	56822-52-7	10.99	7.3	19.01	611.21	748.09	45.77	1.51614561	0.16795509	0.56577827	0.02654672	-5.23532257	down	-	-
MW0049106	Cyclopamine	Alkaloids	Terpenoid alkaloids	C27H41NO2	3	0.5372	4449-51-8 | 11051-96-0	136.63	463.14	501.58	74.75	246.2	139.11	1.08031213	0.19790943	0.59185871	2.393926879	1.259379087	up	C10798	-
MW0132813	3,8'-Biapigenin	Flavonoids	Flavones	C30H18O10	3	0.531	101140-06-1	43.85	49.41	28.39	12.5	8.39	10.03	1.68672667	0.03662697	0.37270445	3.934346701	1.976124096	up	-	-
MEDP1230	Glycidyl oleate	GL	MG	C21H38O3	3	0.5296	5431-33-4	56.52	44.34	109.16	54.52	11.38	36.9	1.06764232	0.21586057	0.60827732	2.042996109	1.030686456	up	C19426	-
MEDL01773	2,4,5-Trimethoxybenzaldehyde	Phenolic acids	Phenolic acids	C10H12O4	3	0.5266	4460-86-0	65257.78	58653.2	62282.11	23685.28	28260.42	24780.33	1.74445752	0.0001868	0.06517318	2.426726497	1.279011519	up	-	-
MADN0502	N-Lactoyl ethanolamine	Alcohol and [illegible]	Amines	C5H11NO3	3	0.5141	5422-34-4	2356.05	2307.61	4569.45	211.5	532.24	3085.43	1.15780352	0.20304727	0.59870897	2.411256225	1.269784963	up	-	-
MW0160477	Hydrogen oxalate	Organic acids	Organic acids	C2HO4-	3	0.5138	920-52-5	635.67	532.77	314.2	1504.03	1388.23	594.75	1.31747307	0.13363737	0.52553754	0.425189489	-1.23382216	down	-	-
MW0011587	(R)-2-Hydroxysterculic acid	Organic acids	Organic acids	C19H34O3	3	0.5109	14602-38-1	3765.04	2927.28	2746.4	18837.63	4172.39	4123.38	1.07584256	0.35149698	0.69843207	0.347863519	-1.52340671	down	-	-
MEDP1093	Allopurinol	Nucleotides and derivatives	Nucleotides and derivatives	C5H4N4O	3	0.508	315-30-0 | 180749-08-0 | 180749-06-8 | 73334-58-4 | 180749-09-1 | 180749-07-9 | 184789-03-5 | 291279-53-3	11492.67	265329.32	1017887.03	791631.2	3513897.6	1352435.48	1.13392646	0.21507696	0.60753414	0.228829479	-2.12765517	down	-	-
MW0146629	beta-Alanine amide	Amino acids and derivatives	Amino acids and derivatives	C3H8N2O	3	0.5066	4726-85-6	200.2	348.35	104.93	19.94	12.24	35.31	1.62979778	0.10884331	0.4998897	9.682619647	3.275397424	up	C19779	-
MW0160847	2-Hydroxy-3-(phosphonooxy)propyl octadec-9-enoate	GL	PA	C21H41O7P	3	0.5016	22002-79-5	21380.32	14673.74	16848.78	40028.32	62380.21	33421.44	1.596712	0.08092101	0.47136621	0.389478405	-1.36038476	down	-	-
MEDL02597	6"-O-Malonylglycitin	Flavonoids	Isoflavones	C25H24O13	3	0.5016	137705-39-6	97972.1	126588.92	63725.74	54787.08	45382.21	43669.69	1.48331336	0.1130368	0.50205214	2.004232511	1.003049885	up	C16197	ko00943
MW0150111	Glu-Phe-Thr	Amino acids and derivatives	Amino acids and derivatives	C18H25N3O7	2	0.9055	-	539.32	42.7	35.11	1273.69	1607.93	2653.68	1.50224301	0.04380428	0.39461781	0.111489892	-3.16501518	down	-	-
MW0158916	Val-Asn-His-Val-Thr	Amino acids and derivatives	Amino acids and derivatives	C24H40N8O8	2	0.9039	-	11303.19	4514.32	9974.95	14575.21	10039.94	48344.55	1.09442832	0.32213318	0.67752893	0.35351653	-1.50015042	down	-	-
MW0156730	Ser-Glu-Glu-Glu-Ile	Amino acids and derivatives	Amino acids and derivatives	C24H39N5O13	2	0.8894	-	4.62	5.84	8.47	99.67	27.37	8.62	1.28147476	0.29593873	0.662229	0.139540027	-2.84124908	down	-	-
MW0157752	Thr-Phe-Asp-Glu-Ile	Amino acids and derivatives	Amino acids and derivatives	C28H41N5O11	2	0.8797	-	3.88	103.74	8.05	881.32	1287.22	972.33	1.5901424	0.01050214	0.24844069	0.036827376	-4.76307759	down	-	-
MW0151083	His-Ser-Leu-Ser-Glu	Amino acids and derivatives	Amino acids and derivatives	C23H37N7O10	2	0.8719	-	550.11	350.56	262.66	174.82	206.97	180.69	1.47534383	0.14090081	0.53210743	2.068215759	1.048386697	up	-	-
MW0153174	Lys-Tyr-Gln	Amino acids and derivatives	Amino acids and derivatives	C20H31N5O6	2	0.8678	-	1.16	13.17	2.35	42.68	64.62	7.12	1.27989644	0.18633928	0.57833032	0.14577871	-2.77814806	down	-	-
MW0158702	TyrMe-His-OH	Amino acids and derivatives	Amino acids and derivatives	C22H22N4O7	2	0.8582	-	4808.73	5066.56	7151.23	12923.08	25074.09	13536.04	1.60000542	0.09583064	0.48888534	0.33039898	-1.59771886	down	-	-
MW0109092	Phe-Arg-Phe	Amino acids and derivatives	Amino acids and derivatives	C24H32N6O4	2	0.8515	-	97.54	47.31	56.73	26.29	20.28	29.49	1.55987396	0.10903928	0.50002306	2.650276098	1.406142663	up	-	-

MW0150824	His-Ala-Phe-Lys	Amino acids and derivatives	Amino acids and derivatives	C24H35N7O5	2	0.8379	-	151.84	154.59	170.41	3.13	18.95	3.06	1.65564353	4.5971E-05	0.04593672	18.96738266	4.245448707	up	-	-
MW0153088	Lys-Phe-Arg-Lys	Amino acids and derivatives	Amino acids and derivatives	C27H47N9O5	2	0.8372	-	14.69	27.78	106.61	1.11	9.5	3.12	1.43696903	0.25609286	0.64181203	10.85797524	3.440683194	up	-	-
MW0144702	Ala-Phe-Tyr-Arg	Amino acids and derivatives	Amino acids and derivatives	C27H37N7O6	2	0.8342	-	6203.13	6195.43	6224.62	1956.54	2349.66	3401.52	1.65896479	0.01373588	0.27083212	2.416172357	1.272723373	up	-	-
MW0151609	Ile-Thr-Val-Lys-Leu	Amino acids and derivatives	Amino acids and derivatives	C27H52N6O7	2	0.8305	-	413	366.09	184.1	248.38	76.44	77.25	1.27697648	0.10959248	0.50091108	2.395577884	1.260373719	up	-	-
MW0008784	N(4)-AcetylsulLipidsmethazine	Benzene and substituted derivatives	Benzene and substituted derivatives	C14H16N4O3S	2	0.828	-	97578.08	72850.4	93247.08	395.59	1250.83	1459.99	1.73224259	0.00753152	0.22054168	84.88112001	6.407371788	up	-	-
MW0145662	Asn-Ala-Leu-Ala-His	Amino acids and derivatives	Amino acids and derivatives	C22H36N8O7	2	0.8276	-	181077.38	221232.9	163042.18	49659.67	101789.94	64868.27	1.60286319	0.00755616	0.22054168	2.613526261	1.385997655	up	-	-
MW0150138	Glu-Ser-Gln-Arg-Gln	Amino acids and derivatives	Amino acids and derivatives	C24H42N10O11	2	0.8106	-	6746.67	69596	53854.19	121623.28	121436.64	95857.91	1.1755241	0.04886791	0.40525092	0.384154649	-1.38024088	down	-	-
MW0157661	Thr-His-Gly	Amino acids and derivatives	Amino acids and derivatives	C12H19N5O5	2	0.8054	-	12.36	29.64	14.65	249.99	208.11	197.75	1.71915724	0.003164	0.18110516	0.086376458	-3.53321803	down	-	-
MW0150524	Gly-Tyr-Tyr-Ser-Lys	Amino acids and derivatives	Amino acids and derivatives	C29H40N6O9	2	0.7926	-	345.29	882.7	647.89	119.06	81.71	12.82	1.50821979	0.06537984	0.44856776	8.782620909	3.134651533	up	-	-
MW0158135	Trp-Gln-Met	Amino acids and derivatives	Amino acids and derivatives	C21H29N5O5S1	2	0.7905	-	1481.32	1695.75	3537.35	7386.57	8388.05	12195.42	1.62424306	0.02549282	0.33612848	0.240057576	-2.05854763	down	-	-
MW0150450	Gly-Phe-His	Amino acids and derivatives	Amino acids and derivatives	C17H21N5O4	2	0.79	-	138.5	82.02	84.16	15.16	4.21	25.77	1.55462146	0.03218832	0.35051264	6.7496677	2.754816477	up	-	-
MW0103299	(-)-Neplanocin A	Nucleotides and derivatives	Nucleotides and derivatives	C11H13N5O3	2	0.7876	-	15580.06	18741.93	13168.05	7525.75	2429.33	4261	1.55901042	0.00735866	0.22054168	3.340586153	1.740101266	up	-	-
MW0005085	4-Hydroxyamphetamine	Benzene and substituted derivatives	Benzene and substituted derivatives	C9H13NO	2	0.7789	-	5671.04	5029.91	6081.73	13133.39	18591.74	17556.36	1.71815435	0.02022439	0.31450694	0.340547333	-1.55407276	down	-	-
MW0110058	Trp-Gln-Arg	Amino acids and derivatives	Amino acids and derivatives	C22H32N8O5	2	0.7786	-	378.18	304.76	274.3	101.7	74.09	165.56	1.59486188	0.00780558	0.22054168	2.804277135	1.487628932	up	-	-
MW0105857	Asp-Gly-Lys	Amino acids and derivatives	Amino acids and derivatives	C12H22N4O6	2	0.7773	-	1039.68	331.08	184.49	1133.66	859.35	1628.74	1.24412026	0.11986723	0.50547245	0.42941948	-1.21954046	down	-	-
MW0151107	His-Trp-Ala	Amino acids and derivatives	Amino acids and derivatives	C20H24N6O4	2	0.7763	-	2472.3	897.89	372.99	3092.01	6053.08	2147	1.26993074	0.15407979	0.5475101	0.331486908	-1.5929762	down	-	-
MW0153100	Lys-Phe-Leu-Glu	Amino acids and derivatives	Amino acids and derivatives	C26H41N5O7	2	0.7758	-	4443.23	10341.22	14450.35	5334.07	4641.73	1894.58	1.1979442	0.17508965	0.57311473	2.462836068	1.300320602	up	-	-
MW0149411	GalNAc1-3Gal1- [illegible]	Others	Saccharides	C28H48N4O21	2	0.7684	-	6223.99	4255.72	2172.74	872.76	1905.03	1389.38	1.43264892	0.12930046	0.52049015	3.036221224	1.602276912	up	-	-
MW0156821	Ser-Leu-Leu-Ser-Phe	Amino acids and derivatives	Amino acids and derivatives	C27H43N5O8	2	0.7664	-	24559	30753.05	35552.23	14692.88	17088.29	12618.58	1.6425832	0.02659077	0.33612848	2.046504316	1.033161709	up	-	-
MW0110070	Trp-Leu-Leu	Amino acids and derivatives	Amino acids and derivatives	C23H34N4O4	2	0.7644	-	60031.66	61003.67	51087.31	43472.93	18486.15	14454.77	1.42148781	0.05942064	0.4369697	2.252505796	1.171530818	up	-	-
MW0152386	Leu-Ile-Tyr	Amino acids and derivatives	Amino acids and derivatives	C21H33N3O5	2	0.7629	-	321805.6	397701.97	428697.78	124268.14	462.4	22.61	1.33311378	0.00351836	0.18110516	9.203818501	3.202232534	up	-	-
MW0016968	Cedrol	Alcohol and [illegible]	Alcohols	C15H26O	2	0.7612	-	20667.68	17624.54	14536.74	15731.16	1630.31	6019.02	1.1677196	0.12858189	0.51982213	2.259531772	1.176023843	up	C09631	-
MW0158949	Val-Asp-Tyr-Val-Leu	Amino acids and derivatives	Amino acids and derivatives	C29H45N5O9	2	0.7596	-	1025.3	7001.51	10033.42	1644.06	814.52	774.53	1.21323417	0.20158384	0.59684301	5.586023983	2.481821768	up	-	-
MW0151338	Ile-Arg-Thr-Asp	Amino acids and derivatives	Amino acids and derivatives	C20H37N7O8	2	0.7593	-	29620.1	32413.7	18447.65	6242.25	4792.03	5349.61	1.71144309	0.03651596	0.37270445	4.912230856	2.296378363	up	-	-
MW0145924	Asn-Tyr-Arg-Asp	Amino acids and derivatives	Amino acids and derivatives	C23H34N8O9	2	0.7592	-	20.83	102.73	97.62	25.41	23.38	10.11	1.23955816	0.17433461	0.57211065	3.755178268	1.908881398	up	-	-
MW0107729	Leu-Ile-Ile	Amino acids and derivatives	Amino acids and derivatives	C18H35N3O4	2	0.7585	-	3173.04	4406.16	4313.65	926.85	1338.24	837.98	1.70475973	0.00989065	0.24036471	3.832607708	1.938326337	up	-	-
MW0109872	Thr-His-Arg	Amino acids and derivatives	Amino acids and derivatives	C16H28N8O5	2	0.7579	-	561.65	427.93	125.2	235.44	102.27	85.25	1.10045539	0.20881095	0.6025099	2.635662947	1.398165888	up	-	-
MW0152990	Lys-His-Ile-Glu-Glu	Amino acids and derivatives	Amino acids and derivatives	C28H46N8O10	2	0.7575	-	14145.06	20792.48	12808.42	4027.34	4312.3	4474.68	1.71641222	0.04179438	0.39256349	3.72598468	1.897621742	up	-	-
MW0110098	Trp-Asn	Amino acids and derivatives	Amino acids and derivatives	C15H18N4O4	2	0.7562	-	391.54	478.38	140.82	25.29	168.45	217.58	1.0280675	0.17972188	0.57366954	2.457308179	1.297078802	up	-	-
MW0156675	Ser-Asp-Arg-Asp	Amino acids and derivatives	Amino acids and derivatives	C17H29N7O10	2	0.7551	-	640.93	1603.91	490.28	384.01	256.36	542.22	1.17626205	0.27370443	0.65054534	2.312821857	1.209654148	up	-	-
MW0157810	Thr-Thr-Asp-Glu-Tyr	Amino acids and derivatives	Amino acids and derivatives	C26H37N5O13	2	0.755	-	3613.25	3482.28	145.99	7.62	129.47	42.62	1.40506289	0.17357276	0.57211065	40.29558734	5.332549957	up	-	-
MW0155871	Pro-Gly-Phe	Amino acids and derivatives	Amino acids and derivatives	C16H21N3O4	2	0.7521	-	223524.63	239887.16	226477.88	113331.89	107346.53	114932.98	1.75575848	0.00034472	0.08987031	2.055620488	1.039573937	up	-	-
MW0150100	Glu-Phe-Glu	Amino acids and derivatives	Amino acids and derivatives	C19H25N3O8	2	0.7518	-	396.99	69.32	76.07	20.85	26.31	57.46	1.29180623	0.30914499	0.67088493	5.184285987	2.374145306	up	-	-
MW0109201	Phenylalanylcysteine	Amino acids and derivatives	Amino acids and derivatives	C12H16N2O3S	2	0.7511	-	62125.39	56209.92	54154.73	257.82	78.72	6492.39	1.53854475	7.2553E-05	0.04593672	25.25872135	4.658709704	up	-	-
MW0146296	Asp-Tyr-Asn-Leu-Leu	Amino acids and derivatives	Amino acids and derivatives	C29H44N6O10	2	0.7508	-	1099.03	3516.94	17009.65	38348.71	49627.96	26885.86	1.41844519	0.02218369	0.32980774	0.188273931	-2.40909484	down	-	-
MW0153085	Lys-Met-Val-Ser-Arg	Amino acids and derivatives	Amino acids and derivatives	C25H49N9O7S	2	0.7498	-	57.07	27.74	19.99	77.68	128.46	31.81	1.06012653	0.24875257	0.6354302	0.440428661	-1.18301974	down	-	-

MW0153149	Lys-Thr-Glu-Lys-Ala	Amino acids and derivatives	Amino acids and derivatives	C24H45N7O9	2	0.7495	-	1437.68	2390.7	2217.94	12711.06	10963.86	6480.04	1.67422974	0.04634605	0.39597854	0.200508308	-2.31826608	down	-	-
MW0153190	Lys-Tyr-Val-Lys	Amino acids and derivatives	Amino acids and derivatives	C26H44N6O6	2	0.7491	-	268.11	184.07	274.2	127.69	119.24	20.93	1.26303672	0.02838721	0.33858973	2.711789741	1.439245323	up	-	-
MW0106254	Cys-Glu-Arg	Amino acids and derivatives	Amino acids and derivatives	C14H26N6O6S1	2	0.7489	-	34147.1	37440.06	35509.84	3142.17	13744.35	15370.15	1.42529283	0.01799775	0.29968703	3.32015053	1.731248652	up	-	-
MW0150926	His-Glu-Phe-Gly-Asp	Amino acids and derivatives	Amino acids and derivatives	C26H33N7O10	2	0.7487	-	1710.53	2942.25	1877.75	1971.55	9800.88	15956.72	1.16823976	0.22206122	0.61243021	0.235511366	-2.08613141	down	-	-
MW0158210	Trp-Met-Trp	Amino acids and derivatives	Amino acids and derivatives	C27H31N5O4S1	2	0.7485	-	86.99	80.83	250.57	5.32	3.95	1.17	1.67908566	0.13416745	0.52622135	40.0756705	5.324654753	up	-	-
MW0110226	Tyr-Tyr-Arg	Amino acids and derivatives	Amino acids and derivatives	C24H32N6O6	2	0.7479	-	12883.26	10450.78	10502.95	2929.73	7617.93	5721.84	1.42409665	0.02996184	0.34901483	2.079780571	1.056431324	up	-	-
MW0151648	Ile-Val-Ile-Phe-Asn	Amino acids and derivatives	Amino acids and derivatives	C30H48N6O7	2	0.7461	-	5807.33	27215.19	35534.45	87649.79	79169.36	65717.11	1.38598641	0.00952806	0.24021401	0.294822709	-1.76208044	down	-	-
MW0155911	Pro-Leu-His	Amino acids and derivatives	Amino acids and derivatives	C17H27N5O4	2	0.7457	-	309.74	953.76	663.44	177.76	135.17	479.33	1.20396976	0.17124662	0.5671048	2.432206599	1.282265781	up	-	-
MW0155297	Phe-Phe-Ala	Amino acids and derivatives	Amino acids and derivatives	C21H25N3O4	2	0.7452	-	2420.03	5723	2055.94	336.9	449.02	182.08	1.65921316	0.1180293	0.50298639	10.53612603	3.397272603	up	-	-
MW0158633	Tyr-Thr-Val-Thr-Leu	Amino acids and derivatives	Amino acids and derivatives	C28H45N5O9	2	0.7451	-	1292.02	365.63	399.84	517.15	152.32	185.61	1.06794702	0.3178598	0.67428155	2.406195911	1.266754111	up	-	-
MW0156957	Ser-Tyr-His-Asp	Amino acids and derivatives	Amino acids and derivatives	C22H28N6O9	2	0.7451	-	6617.99	3772.57	361.69	13848.95	5593.5	13907.87	1.14055344	0.09523865	0.48768821	0.322403203	-1.63306202	down	-	-
MW0150305	Gly-Arg-Ser-Asp-Asp	Amino acids and derivatives	Amino acids and derivatives	C19H32N8O11	2	0.7447	-	3507.98	813.26	1638.66	545.05	1113.65	648.2	1.15204467	0.26366141	0.64421071	2.583510339	1.369332657	up	-	-
MW0159145	Val-Pro-Ser	Amino acids and derivatives	Amino acids and derivatives	C13H23N3O5	2	0.7436	-	3806.89	4580.39	2673.79	8677.89	14220.93	5544.74	1.44148038	0.14422168	0.53525358	0.388877834	-1.36261109	down	-	-
MW0155931	Pro-Lys-Met	Amino acids and derivatives	Amino acids and derivatives	C16H30N4O4S1	2	0.7431	-	2367.34	799.54	950.87	263.06	426.09	58.46	1.41781853	0.14811192	0.53947123	5.507885127	2.461498471	up	-	-
MW0155128	Phe-Asp-Gly-Gly-Val	Amino acids and derivatives	Amino acids and derivatives	C22H31N5O8	2	0.7413	-	156.44	234.82	358.76	35.58	58.15	156.12	1.37539123	0.08674853	0.47762164	3.001881129	1.585866849	up	-	-
MW0155806	Pro-Asp-Asn	Amino acids and derivatives	Amino acids and derivatives	C13H20N4O7	2	0.7405	-	9879.73	9337.01	8284.91	163.04	252.16	1431.04	1.62359119	0.0001807	0.06517318	14.89603194	3.896856166	up	-	-
MW0158652	Tyr-Tyr-Leu	Amino acids and derivatives	Amino acids and derivatives	C24H31N3O6	2	0.7398	-	618389.1	625279.87	682231.45	1704	55.38	662.8	1.69765485	0.00098836	0.12883555	795.1103634	9.635011314	up	-	-
MW0011860	1,2-Dimyristoleoyl-sn-glycero-3-phosphocholine	GL	PC	C36H68NO8P	2	0.7379	-	349.34	476.52	363.29	1088.91	666.14	666.46	1.5422511	0.09052349	0.48325238	0.491077881	-1.02597625	down	-	-
MW0155134	Phe-Asp-Phe-Gln-Asn	Amino acids and derivatives	Amino acids and derivatives	C31H39N7O10	2	0.7337	-	989.38	1118.63	550.94	116.36	73.25	35.08	1.675193	0.03982714	0.38624483	11.83385998	3.564848826	up	-	-
MW0107448	Ile-Leu-Asn	Amino acids and derivatives	Amino acids and derivatives	C16H30N4O5	2	0.7302	-	2216.57	12050.56	6695.13	3329.67	2455.99	1750.24	1.10021353	0.25433694	0.64131789	2.781653154	1.47594254	up	-	-
MW0110186	Tyr-Asn	Amino acids and derivatives	Amino acids and derivatives	C13H17N3O5	2	0.7272	-	453.3	2110.91	1555.15	289.61	44.74	254.69	1.41170961	0.13378375	0.5256468	6.993345104	2.8059827	up	-	-
MW0158395	Tyr-Asn-His	Amino acids and derivatives	Amino acids and derivatives	C19H24N6O6	2	0.7266	-	87441.16	67426.87	85801.13	407.41	1186.44	683.5	1.74554571	0.00640243	0.21018946	105.6794783	6.72355144	up	-	-
MW0110042	Tris(2-butoxyethyl) phosphate	Others	Lactones	C18H39O7P	2	0.7256	-	2934.5	3176.05	1513.83	916.99	695.93	908.1	1.59212078	0.07898118	0.46796065	3.024323488	1.596612462	up	C14446	-
MW0110230	Tyr-Val-Arg	Amino acids and derivatives	Amino acids and derivatives	C20H32N6O5	2	0.7255	-	125376.34	117934.79	137308.87	28431.07	61.51	21.56	1.4048685	0.00120693	0.13399001	13.34846501	3.738601945	up	-	-
MW0153552	Met-Glu-Trp	Amino acids and derivatives	Amino acids and derivatives	C21H28N4O6S1	2	0.7229	-	57444.38	43512.23	22746.93	762.12	3977.44	2414.33	1.64856873	0.06005864	0.43825617	17.29178671	4.112015041	up	-	-
MW0153545	Met-Glu-Leu-Ser-Glu	Amino acids and derivatives	Amino acids and derivatives	C24H41N5O11S	2	0.7201	-	12.22	2.74	6.44	46.33	8.97	29.25	1.26619642	0.18469839	0.57832633	0.253104672	-1.98219396	down	-	-
MW0148055	Cys-Phe-His	Amino acids and derivatives	Amino acids and derivatives	C18H23N5O4S1	2	0.7197	-	28077.71	24892.72	32863.37	45864.91	63199.74	77752.75	1.60294881	0.05981909	0.43748876	0.45945292	-1.12201106	down	-	-
MW0153225	Lys-HoPhe-OH	Amino acids and derivatives	Amino acids and derivatives	C21H25N3O6	2	0.7182	-	540.06	824.83	1060.85	1903.95	1770.85	3392.09	1.55022929	0.0867963	0.47762164	0.343254246	-1.54265053	down	-	-
MW0159233	Val-Val-Asn-Trp-Asp	Amino acids and derivatives	Amino acids and derivatives	C29H41N7O9	2	0.7122	-	401.06	427.92	296.76	486.53	752.45	1061.4	1.41186089	0.13578739	0.52749511	0.489371321	-1.03099854	down	-	-
MW0148074	Cys-Trp-Ala	Amino acids and derivatives	Amino acids and derivatives	C17H22N4O4S1	2	0.7044	-	33769.61	7011.68	28737.83	9837.38	8441.1	196.31	1.03771205	0.1638008	0.56075761	3.762918009	1.911851854	up	-	-
MW0151505	Ile-Lys-Arg-Asp-Phe	Amino acids and derivatives	Amino acids and derivatives	C31H51N9O8	2	0.7038	-	238.65	106.55	157.01	55.16	97.71	77.46	1.39927542	0.13247215	0.52284849	2.180393349	1.124588424	up	-	-
MW0159071	Val-Leu-Lys-Val-Leu	Amino acids and derivatives	Amino acids and derivatives	C28H54N6O6	2	0.7022	-	19348.74	6355.46	5871.02	282.4	43.61	323.14	1.64950765	0.14455456	0.53525358	48.64086883	5.604097093	up	-	-
MW0151559	Ile-Pro-His	Amino acids and derivatives	Amino acids and derivatives	C17H27N5O4	2	0.7021	-	3123.37	4783.41	5120.63	9856.93	12348.5	9890.42	1.64803851	0.00449562	0.18300962	0.405890793	-1.30083648	down	-	-
MW0112382	3,4,5-trihydroxy-6-(2-oxoethoxy)oxane-2-carboxylic acid	Organic acids	Organic acids	C8H12O8	3	0.9866	-	1323.15	15063.66	1579.23	993.6	280.2	834.14	1.20505983	0.36420644	0.7041771	8.523031965	3.091366743	up	-	-
MW0165255	1-Oleoylglycerone 3-phosphate(2-)	Organic acids	Organic acids	C21H37O7P-2	3	0.9824	-	13468.06	10481.73	10698.96	57055.57	23263.55	17524.31	1.37687876	0.22919499	0.62089986	0.354124441	-1.49767167	down	-	-
MW0165918	(-)-Dehydrodiconiferyl acid carboxylate	Organic acids	Organic acids	C20H16O8-2	3	0.9707	-	62	396.31	980.06	61.98	61.98	61.98	1.21513145	0.25993251	0.64322773	7.73566742	2.95152577	up	-	-

MW0069387	TG(15:0/18:4(6Z,9Z,12Z,15Z)/o-18:0)	GL	TG	C54H98O5	3	0.9546	-	3174.5	2109.29	2748.39	251.68	1060.6	641.29	1.53499993	0.00780402	0.22054168	4.111539387	2.039678649	up	-	-
ZINC9590888	Acanfolioside	Lignans and Coumarins	Lignans	C37H46O17	3	0.9531	-	3.09	4.67	0.36	0.19	1	0.29	1.10695534	0.21743937	0.6087753	5.486486486	2.455882552	up	-	-
MW0075975	TG(18:1(11Z)/22:6(4Z,7Z,10Z,13Z,16Z,19Z)/o-18:0)	GL	TG	C61H106O5	3	0.9241	-	927.28	1275.52	1399.26	339.63	1077.57	336.37	1.28596179	0.11336637	0.50205214	2.054129576	1.038527191	up	-	-
MW0084251	TG(22:0/24:0/24:1(15Z))	GL	TG	C73H140O6	3	0.8995	-	584.11	909.98	614.88	105.23	458.62	480.88	1.14346987	0.09253978	0.48592036	2.018674681	1.013408433	up	-	-
MW0132077	3,4,5-trihydroxy-6-{[3-hydroxy-2-(2-hydroxypropan-2-yl)-7-oxo-2H,3H,7H-furo[3,2-g]chromen-9-yl]oxy}oxane-2-carboxylic acid	Organic acids	Organic acids	C20H22O12	3	0.8956	-	375.18	2029.25	1648.43	3333.64	3754.78	2627.97	1.24410278	0.04206412	0.39256349	0.417115822	-1.26148006	down	-	-
MW0060675	PE-NMe2(20:4(5Z,8Z,11Z,14Z)/22:6(4Z,7Z,10Z,13Z,16Z,19Z))	GL	PE	C49H78NO8P	3	0.8602	-	927.04	2342.49	1042.66	177.47	924.42	487.02	1.24304392	0.17304561	0.57148894	2.713929675	1.440383337	up	-	-
MW0132871	3-[2-(3,4-dihydroxyphenyl)-5,7-dihydroxy-4-oxo-3,4-dihydro-2H-1-benzopyran-8-yl]-5,7-dihydroxy-2-phenyl-3,4-dihydro-2H-1-benzopyran-4-one	Benzene and substituted derivatives	Benzene and substituted derivatives	C30H22O10	3	0.8364	-	238444.46	119573.75	100459.79	415525.65	412444.35	250465.82	1.45116903	0.04379674	0.39461781	0.425132392	-1.23401591	down	-	-
MW0136081	6-{[7-({6-[(acetyloxy)methyl]-3-{[3,4-dihydroxy-4-(hydroxymethyl)oxolan-2-yl]oxy}-4,5-dihydroxyoxan-2-yl}oxy)-2-(4-hydroxyphenyl)-4-oxo-4H-chromen-5-yl]oxy}-3,4,5-trihydroxyoxane-2-carboxylic acid	Organic acids	Organic acids	C34H38O21	3	0.8107	-	901.5	693.63	1744.31	2838.58	3502.58	1611.67	1.37503193	0.09011304	0.48292743	0.41990587	-1.25186214	down	-	-
MW0161844	Cocaine(1+)	Organic acids	Organic acids	C17H22NO4+	3	0.8077	-	32298.86	15687.15	19129.53	60422.35	67398.71	46393.81	1.58508118	0.01206494	0.25665182	0.38524576	-1.37614902	down	-	-
ZINC9591020	4-O,6-O-(4,4',5,5',6,6'-Hexahydroxybiphenyl-2,2'-diyldicarbonyl)-beta-D-glucopyranose 1-(4-hydroxy-trans-cinnamate)	Others	Lactones	C29H24O16	3	0.7965	-	10440.78	4965.47	13358.52	24754.94	33415.39	26709.32	1.55276848	0.00658026	0.21133131	0.338888886	-1.56111577	down	-	-
MEDP1664	Glu-Leu-Arg	Amino acids and derivatives	Amino acids and derivatives	C17H32N6O6	3	0.7862	-	126237.62	173186.46	166562.05	118212.56	57799.35	48067.63	1.4247288	0.04526632	0.39546247	2.079556795	1.056276087	up	-	-
MEDP1433	Carnitine ph-C1	Lipids	CAR	C14H19NO4	3	0.7586	-	1935.98	2338.87	1536.17	1281.35	183.53	258.27	1.3930153	0.03992516	0.38629132	3.372323942	1.753743127	up	-	-
MW0105145	4-{Methyl[(methylcarbamoyl)methyl]sulLipidsmoyl}butanoic acid	Organic acids	Organic acids	C8H16N2O5S	3	0.7495	-	709.28	305.93	3061.49	5442.48	1904.52	3620.21	1.18630703	0.16250692	0.55831836	0.371717146	-1.42772286	down	-	-
MEDP1024	Leu-Val	Amino acids and derivatives	Amino acids and derivatives	C11H22N2O3	3	0.7449	-	576.62	33.24	368.17	9.86	43.59	13.27	1.34612681	0.19443339	0.58779711	14.65872302	3.873687525	up	-	-
MEDP1407	Carnitine C14:2	Lipids	CAR	C21H37NO4	3	0.7436	-	267002.7	238239.75	257893.01	120096.84	127142.34	83149.59	1.67586656	0.00186895	0.15624092	2.30981053	1.207774515	up	-	-
MW0150336	Gly-Cys-His	Amino acids and derivatives	Amino acids and derivatives	C11H17N5O4S1	3	0.7423	-	997.53	1250.69	246.36	6131.26	3896.81	1528.88	1.36343886	0.14499566	0.53579335	0.215851068	-2.21189186	down	-	-
ZINC9591161	4',5,5''-Trihydroxy-4''',7''-dimethoxy-7-(beta-D-glucopyranosyloxy)-8,3'''-biflavone	Others	Ketone compounds	C38H32O15	3	0.7369	-	4.08	5.9	5.36	38.58	4.92	10.12	1.04157412	0.34683032	0.69613395	0.286087281	-1.80547274	down	-	-
MEDP1444	FLipids(18:5)	Lipids	Free Lipidstty acids	C18H26O2	3	0.7341	-	3507.96	1490.68	2283.57	1286.47	842.37	390.73	1.39396002	0.09735525	0.48888534	2.890259052	1.531198806	up	-	-
MW0150237	Glucoarabin	Organic acids	Organic acids	C17H33NO10S3	3	0.7334	-	37114.35	8035.55	197023.54	200749.6	376629.43	168252.14	1.20664092	0.12796295	0.51982213	0.324789856	-1.62242152	down	C17272	-
MW0140064	(-)-Threo-isodihomocitric acid	Organic acids	Organic acids	C8H12O7	3	0.7314	-	13624.02	15467.14	15502.39	8731.51	4188.52	7483.79	1.54343557	0.01472565	0.27628422	2.185549079	1.127995776	up	-	-
MW0161494	Mphpv II	Others	Ketone compounds	C17H18O6	3	0.7293	-	1200.56	1121.85	1874.31	289.01	671	585.58	1.51533845	0.04721427	0.39857834	2.715286719	1.441104547	up	-	-
MW0130644	2-{3-[(6-carboxy-3,4,5-trihydroxyoxan-2-yl)oxy]-4-methoxyphenyl}-3-hydroxy-5-sulfino-3,4-dihydro-2H-1-benzopyran-7-olate	Benzene and substituted derivatives	Benzene and substituted derivatives	C22H24O13S	3	0.7292	-	13897.91	12281.09	11758.52	1175.37	1186.41	2588.57	1.70777905	0.00027533	0.08134988	7.663603584	2.938022936	up	-	-

MW0139879	Tricin 7-[sinapoyl-(->2)-glucuronyl-(1->2)-glucuronide]	Flavonoids	Flavones	C40H40O23	3	0.7278	-	53.11	67.31	115.31	25.2	20.76	38.91	1.51960043	0.10644809	0.4992359	2.777542123	1.473808791	up	-	-
MEDP1428	Carnitine C8:1(2-Octenoylcarnitine)	Lipids	CAR	C15H27NO4	3	0.719	-	2953.03	3671.92	1012.02	6226.68	8090.11	8270.79	1.46051944	0.00917275	0.23913909	0.338104835	-1.56445745	down	-	-
MW0165814	dTDP-4-keto-5-methyl-beta-L-rhamnose	Nucleotides and derivatives	Nucleotides and derivatives	C17H24N2O15P2-2	3	0.7151	-	266.48	7480.61	3641.55	293.31	56.06	80.05	1.38731021	0.22158116	0.61186772	26.52098179	4.729062279	up	-	-
MEDP1322	LysoPC(22:5(7Z,10Z,13Z,16Z,19Z))	Lipids	LPC	C30H52NO7P	3	0.7067	-	40.16	59.28	42.39	57.48	306.35	115.98	1.24570615	0.27208254	0.64977045	0.295596173	-1.7583005	down	C04230	ko00564
MW0144275	Acetyl-maltose	Others	Saccharides	C14H24O12	3	0.7038	-	12236.27	16830.5	12598.7	2074.79	2300.59	1532.04	1.7391384	0.01335151	0.26897231	7.053073931	2.818252162	up	C02130	-
MW0138358	Helonioside B	Benzene and substituted derivatives	Benzene and substituted derivatives	C34H40O18	3	0.7005	-	35317.43	29135.47	31394.32	9507.17	6534.28	26759.05	1.33568392	0.09743814	0.48888534	2.239394867	1.163108937	up	-	-
MW0144525	Ala-Cys-Glu	Amino acids and derivatives	Amino acids and derivatives	C11H19N3O6S1	3	0.6983	-	1936.52	3151.64	4678.98	3293.61	17108.05	7640.4	1.12920018	0.27187907	0.64977045	0.348303227	-1.52158425	down	-	-
MW0155321	Phe-Pro-Tyr	Amino acids and derivatives	Amino acids and derivatives	C23H27N3O5	3	0.6956	-	5.98	45.27	10.91	1.6	6.77	0.55	1.30397276	0.2862999	0.6560916	6.968609865	2.800870888	up	-	-
MW0000941	Isobutyric acid 2-methoxy-4-[1-(acetyloxy)-2-[illegible]	Phenolic acids	Phenolic acids	C16H20O5	3	0.6953	-	418.24	1043.92	719.69	81.09	496.53	77.87	1.33217928	0.09383322	0.48592036	3.328578621	1.734906245	up	-	-
ZINC9591002	Carvacrol 2-O-beta-glucopyranosyl(1-2)-beta-glucopyranoside	Alcohol and amines	Alcohols	C22H34O11	3	0.6947	-	119174.89	40108.48	95362.34	134172.31	311413.81	117143.08	1.16453768	0.23474385	0.62573958	0.452519098	-1.14394941	down	-	-
MW0114004	NCGC00179726-03_C40H44O22_1,4-Ethanonaphthalene-5,10-dicarboxylic acid, 1,4,4a,5,6,8a-hexahydro-5,10-dihydroxy-6,9-dioxo-, bis[[2-(beta-D-glucopyranosyloxy)-3-hydroxyphenyl]methyl] ester	Benzene and substituted derivatives	Benzene and substituted derivatives	C40H44O22	3	0.6942	-	2788.74	1272.66	1039.41	9788.81	19650.28	7447.27	1.62598958	0.10225103	0.49313285	0.13828445	-2.85428916	down	-	-
ZINC5958579	Dichotomoside E	Others	Lactones	C20H30O9	3	0.6941	-	5703.76	4478.33	2298.4	1264.86	894.23	556.04	1.58790233	0.07665709	0.46149681	4.596645464	2.200581397	up	-	-
MW0006925	4-Dodecylbenzenesulfonic acid	Benzene and substituted derivatives	Benzene and substituted derivatives	C18H30O3S	3	0.6938	-	272.62	123.9	167.77	53.77	37.1	84.57	1.53160611	0.08768828	0.47802518	3.216427269	1.685459066	up	-	-
MW0148061	Cys-Pro-Glu	Amino acids and derivatives	Amino acids and derivatives	C13H21N3O6S1	3	0.693	-	2414.79	3635.24	3289.92	145.03	778.15	1080.47	1.4473021	0.00709662	0.22054168	4.661467821	2.220784308	up	-	-
MW0152497	Leu-Ser-Phe-Gln-Asp	Amino acids and derivatives	Amino acids and derivatives	C27H40N6O10	3	0.6903	-	29213.46	17801.59	37077.92	14684.22	16229.56	9837.82	1.40433313	0.1108226	0.50205214	2.063550143	1.045128495	up	-	-
MW0158462	Tyr-Glu-Phe	Amino acids and derivatives	Amino acids and derivatives	C23H27N3O7	3	0.6894	-	38.64	61.81	28.62	171.34	111.5	35.15	1.03629856	0.24766604	0.63413128	0.405893267	-1.30082769	down	-	-
MW0152962	Lys-Glu-Phe-Lys	Amino acids and derivatives	Amino acids and derivatives	C26H42N6O7	3	0.6862	-	11929.27	7898.16	5850.98	504.67	348.52	152.26	1.70891082	0.0436381	0.39461781	25.53922124	4.674642629	up	-	-
MW0162750	(2R,3S,4S,5R,6S)-2-(hydroxymethyl)-6-[6-[[(E)-4-hydroxy-3-methylbut-2-enyl]amino]purin-7-yl]oxane-3,4,5-triol	Alcohol and amines	Alcohols	C16H23N5O6	3	0.6822	-	216.85	292.33	319.79	27.75	121.76	24.7	1.52723095	0.00791203	0.22054168	4.758452442	2.250492452	up	-	-
MW0153678	Met-Thr-Ile	Amino acids and derivatives	Amino acids and derivatives	C15H29N3O5S1	3	0.6819	-	18.76	16.85	15.28	5.71	9.34	1.43	1.36300165	0.02344638	0.33077008	3.087985437	1.626665949	up	-	-
MW0155968	Pro-Phe-Tyr-Leu-Arg	Amino acids and derivatives	Amino acids and derivatives	C35H50N8O7	3	0.6782	-	386.76	681.4	428.94	116.92	58.31	384.34	1.29803625	0.08383457	0.47392197	2.675447218	1.419780068	up	-	-
MW0158416	Tyr-Asp-Phe	Amino acids and derivatives	Amino acids and derivatives	C22H25N3O7	3	0.6774	-	2097.95	2515.47	3012.99	6997.23	32329.37	4378.91	1.31600363	0.30963398	0.67088493	0.17449539	-2.51873917	down	-	-
MW0153110	Lys-Phe-Val-Leu-Val	Amino acids and derivatives	Amino acids and derivatives	C31H52N6O6	3	0.6754	-	1608.02	12167.08	11713.63	21217.87	32101.19	16885.37	1.201999	0.06302778	0.44396349	0.363064411	-1.46170258	down	-	-
MEDP1446	Octadecadienamide	Alcohol and amines	Amines	C18H33NO	3	0.6733	-	160.55	104.49	268.27	466.72	441.46	204.98	1.23514518	0.13247591	0.52284849	0.479095548	-1.06161469	down	-	-
MW0127906	(2E)-2-cyano-3-(4-ethoxy-3-methoxyphenyl)-N-(furan-2-ylmethyl)prop-2-enamide	Alcohol and amines	Amines	C18H18N2O4	3	0.6727	-	5504.58	8367.34	9135.13	3583.19	6108.7	1635.13	1.24234139	0.08575478	0.47762164	2.031165302	1.022307655	up	-	-
ZINC9591166	Glochidacuminoside C	Others	Lactones	C22H32O10	3	0.672	-	14084.46	20380.62	16047.66	30522.46	51900.67	42434.34	1.62088447	0.04718138	0.39857834	0.404563219	-1.30556293	down	-	-
MW0144801	Ala-Val-Leu	Amino acids and derivatives	Amino acids and derivatives	C14H27N3O4	3	0.6712	-	5234.89	13938.37	4191.98	2664.27	3354.94	5007.41	1.08391895	0.31336736	0.67242769	2.118984784	1.083373228	up	-	-
MW0150201	Glu-Tyr-Val	Amino acids and derivatives	Amino acids and derivatives	C19H27N3O7	3	0.6703	-	6123.16	3716.08	6818.07	2925.54	1943.49	2648.22	1.5245415	0.07234482	0.4595545	2.215878147	1.147878549	up	-	-
MW0146248	Asp-Pro-Tyr	Amino acids and derivatives	Amino acids and derivatives	C18H23N3O7	3	0.6697	-	3123.26	1181.27	2273.36	327.54	369.69	542.02	1.6331459	0.08500538	0.47658307	5.30796046	2.408157624	up	-	-
MEDP1245	3-Hydroxycaprolactone	Others	Lactones	C12H16N2O4	3	0.6681	-	16.64	44.23	62.7	644.73	480.36	1049.92	1.68083465	0.05531147	0.42471427	0.056813532	-4.1376216	down	C03068	-

MW0145438	Arg-Lys-His-Arg	Amino acids and derivatives	Amino acids and derivatives	C24H45N13O5	3	0.6681	-	9046.58	6773.04	7039.99	2343.86	2902.34	1705.21	1.68718139	0.00782438	0.22054168	3.288485358	1.717423247	up	-	-
MW0143452	Azacyclonol	Benzene and substituted derivatives	Benzene and substituted derivatives	C18H21NO	3	0.6678	-	29.44	77.42	156.67	240.73	1619.55	325.7	1.38635327	0.28702546	0.65741439	0.120554625	-3.05224109	down	-	-
MEDP1473	2H-Pyran-2-one, tetrahydro-4-hydroxy-4-methyl-, (R)-	Others	Lactones	C6H10O3	3	0.6665	-	442.06	261.92	223.01	33.1	58.42	86.71	1.62659222	0.05941537	0.4369697	5.201088481	2.378813581	up	-	-
MW0155306	Phe-Phe-Thr	Amino acids and derivatives	Amino acids and derivatives	C22H27N3O5	3	0.665	-	10973.66	12737.07	4638.12	4717.95	5512.33	2239.27	1.21664574	0.15249426	0.54704545	2.273446115	1.18488081	up	-	-
MW0147991	Cys-Asp-His	Amino acids and derivatives	Amino acids and derivatives	C13H19N5O6S1	3	0.6622	-	25.6	135.46	119.3	120.36	134.58	629.83	1.01166418	0.35181844	0.69843207	0.316873312	-1.65802194	down	-	-
MW0116346	3-[(E)-(2-nitrophenyl)methylideneamino]imidazolidine-2,4-dione	Benzene and substituted derivatives	Benzene and substituted derivatives	C10H8N4O4	3	0.6617	-	28.64	166.21	94.83	79.5	13.47	4.46	1.07547024	0.25294082	0.64095696	2.973211536	1.572022111	up	-	-
MW0160868	N-(hexacosanoyl)-1-beta-glucosyl-sphinganine	Lipids	Cer	C50H99NO8	3	0.6611	-	81.24	1062.58	814.49	4724.44	1090.46	888.12	1.02184969	0.33142471	0.68478989	0.292153388	-1.77520208	down	-	-
MW0147988	Cys-Asn-Ser-Ala-Arg	Amino acids and derivatives	Amino acids and derivatives	C19H35N9O8S	3	0.6607	-	3181.88	1209.33	4785.98	4513.27	8900.26	6090.18	1.21706457	0.10810748	0.49971175	0.470535606	-1.0876242	down	-	-
MEDP0797	1-[illegible]	Quinones	Anthraquinone	C14H8O3	3	0.6596	-	215.78	179.23	180.69	69.26	89.7	87.79	1.71153529	0.00356691	0.18110516	2.333130699	1.222267128	up	C02980	-
MW0154292	Nap-Phe-OH	Amino acids and derivatives	Amino acids and derivatives	C27H22N2O6	3	0.658	-	196.85	102.49	178.88	72.61	9.43	99.68	1.14995095	0.06651367	0.44856776	2.631631081	1.395957257	up	-	-
MW0063566	C26:1 Sphingomyelin	Lipids	SM	C49H97N2O6P	3	0.6578	-	818.04	1743.95	2260.96	585.62	304.71	945.74	1.3050985	0.12795852	0.51982213	2.626778935	1.393294791	up	C00550	ko00600,ko01100
MW0146049	Asp-Asp-Ala	Amino acids and derivatives	Amino acids and derivatives	C11H17N3O8	3	0.657	-	4.43	12.82	180.98	284.09	488.13	231.78	1.34766623	0.05552728	0.42471427	0.197440239	-2.34051205	down	-	-
MW0151457	Ile-His-Pro-Lys-Asn	Amino acids and derivatives	Amino acids and derivatives	C27H45N9O7	3	0.6568	-	66402.5	27964.27	60933.77	15216.7	23069.8	30643.93	1.32501902	0.12607124	0.5176181	2.25300408	1.171849926	up	-	-
MW0001405	1,9-Pyrazoloanthrone	Benzene and substituted derivatives	Benzene and substituted derivatives	C14H8N2O	3	0.6562	-	24313.55	41751.95	27119.61	14763.03	12172.87	12267.19	1.62614632	0.0762555	0.46149681	2.376983804	1.249132073	up	-	-
MW0106024	(2S)-2-[[(2R,3R)-3-azaniumyl-2-hydroxy-4-phenylbutanoyl]amino]-4-methylpentanoate	Amino acids and derivatives	Amino acids and derivatives	C16H24N2O4	3	0.6521	-	30.1	87.41	150.73	118.55	604.28	139.43	1.06886532	0.33744967	0.69016011	0.311089463	-1.68459857	down	-	-
MW0151011	His-Lys-His	Amino acids and derivatives	Amino acids and derivatives	C18H28N8O4	3	0.6504	-	30073.62	30576.23	34931.83	13429.7	22373.99	11871.66	1.54649605	0.02404392	0.33093994	2.004844852	1.003490596	up	-	-
MW0110054	Trp-Asn-Arg	Amino acids and derivatives	Amino acids and derivatives	C21H30N8O5	3	0.6504	-	507.76	2943.32	728.36	221.48	19.24	247.88	1.33973348	0.25383556	0.64102518	8.553909128	3.096583881	up	-	-
MEDP2189	Hexadecylamine	Alcohol and [illegible]	Amines	C16H35N	3	0.6482	-	3644.81	2571.67	5156.49	2560.79	823.21	349.69	1.32374888	0.06546952	0.44856776	3.046040244	1.606935003	up	-	-
MW0145298	Arg-Asp-Leu-Tyr-Ser	Amino acids and derivatives	Amino acids and derivatives	C28H44N8O10	3	0.6472	-	2809.71	11082.88	19635.37	21043.77	19592.87	26867.32	1.11711926	0.13052146	0.52255746	0.49668138	-1.00960743	down	-	-
MW0148752	dTDP-4-oxo-2-deoxy-alpha-D-pentos-2-ene	Nucleotides and derivatives	Nucleotides and derivatives	C15H20N2O14P2	3	0.6472	-	212583.27	119356.82	147131.44	37148.12	53649.2	48562.37	1.67072021	0.05083331	0.40888066	3.437662139	1.78142776	up	C21352	ko01110
MW0062696	PS(20:1(11Z)/22:6(4Z,7Z,10Z,13Z,16Z,19Z))	GL	PS	C48H80NO10P	3	0.6468	-	888.62	9306.05	7841.94	9636.05	19193.54	19849.9	1.13538259	0.0753351	0.46149681	0.370517645	-1.43238585	down	-	-
MW0105851	Asp-Gln-Arg	Amino acids and derivatives	Amino acids and derivatives	C15H27N7O7	3	0.6451	-	5188.71	7015.1	3909.02	9439.69	26952.13	19318.35	1.51139145	0.11707285	0.50205214	0.289226007	-1.78973081	down	-	-
MW0112080	2-amino-8H-indeno[1,2-d][1,3]thiazol-8-one	Others	Ketone compounds	C10H6N2OS	3	0.6435	-	696.7	732.44	3128.46	3878	2987.39	3835.71	1.30730118	0.11314616	0.50205214	0.425900141	-1.23141289	down	-	-
MW0143824	6-Desmethoxy hormothamnione triacetate	Others	Lactones	C27H26O11	3	0.6412	-	37472.02	68342.55	45895.65	23457.09	17255.12	32200.18	1.45417889	0.08633626	0.47762164	2.08071934	1.057082379	up	-	-
MW0144605	Ala-His-Val	Amino acids and derivatives	Amino acids and derivatives	C14H23N5O4	3	0.6396	-	195.52	230.67	84.74	67.32	67.36	47.08	1.44900475	0.12687754	0.51824528	2.811014525	1.491090908	up	-	-
MW0109034	palmyramide A	Lipids	Free Lipidstty acids	C36H53N3O9	3	0.6396	-	8.33	10.5	16.24	2.2	6.6	4.49	1.42174074	0.0714147	0.45866819	2.638826185	1.399896326	up	-	-
MW0128477	[(5-{[2,2-dimethyl-7-(2-methylbut-3-en-2-yl)-5-[(2-methylbut-3-en-2-yl)oxy]-8-oxo-2H,8H-pyrano[3,2-g]chromen-10-yl]oxy}-2-methyl-7,10-bis(2-methylbut-3-en-2-yl)-8-oxo-2H,8H-pyrano[3,2-g]chromen-2-yl)methoxy]sulfonic acid	Organic acids	Organic acids	C48H54O12S	3	0.6396	-	21.14	17.57	11.55	10.08	9.5	2.61	1.24126837	0.06552014	0.44856776	2.264984227	1.179501004	up	-	-
MW0155095	Phe-Arg-Leu-Phe-Leu	Amino acids and derivatives	Amino acids and derivatives	C36H54N8O6	3	0.6369	-	925.65	1055.8	1673.25	169.31	415.05	840.06	1.31214956	0.07156722	0.45866819	2.565746058	1.359378388	up	-	-
MW0137929	Deoxysappanone B 7,3'-dimethyl ether	Others	Ketone compounds	C18H18O5	3	0.6353	-	4803.28	1032.23	832.48	6129.66	5136.36	4337.43	1.27403023	0.13452305	0.52638988	0.427340748	-1.22654121	down	-	-
MW0155059	Phaeophorbide b	Heterocyclic compounds	Heterocyclic compounds	C35H34N4O6	3	0.6351	-	3468.87	2211.34	3118.83	5704.18	4824.5	9284.82	1.50848414	0.10581744	0.49914367	0.444093169	-1.17106572	down	-	-

MW0006507	Carmine	Benzene and substituted derivatives	Benzene and substituted derivatives	C22H20O13	3	0.6333	-	181574.9	647.14	191.97	220506.91	318527.51	322863.33	1.3016474	0.04380685	0.39461781	0.211642286	-2.24030019	down	-	-
MW0012198	11(E)-(3,4-dimethyl-5-(pent-1-en-1-yl)furan-2-yl)-undecanoic acid	Lipids	Free Lipidstty acids	C22H36O3	3	0.629	-	70620.91	90243.08	54018.18	84575.8	201025.33	185893.25	1.34164248	0.13543383	0.52745406	0.455747044	-1.1336948	down	-	-
MW0109091	Phe-Arg-Lys	Amino acids and derivatives	Amino acids and derivatives	C21H35N7O4	3	0.629	-	25486.89	8869.71	9099.02	6329.66	5919.06	9262.59	1.08905123	0.31395887	0.6726196	2.020128946	1.014447384	up	-	-
MW0155830	Pro-Gln-Gln	Amino acids and derivatives	Amino acids and derivatives	C15H25N5O6	3	0.629	-	15388.64	14717.38	13354.29	39249.04	51665.34	39045.55	1.73188826	0.01863004	0.30580858	0.334413153	-1.5802965	down	-	-
MW0149637	Gln-Asn-Met	Amino acids and derivatives	Amino acids and derivatives	C14H25N5O6S1	3	0.6269	-	19.73	217.34	65.02	1.63	24.44	36.8	1.04239907	0.31284699	0.67242769	4.804994433	2.26453476	up	-	-
MW0157059	Sibiricoside B	Terpenoids	Steroidal	C50H80O24	3	0.6264	-	34824.06	53494.4	27879.54	19573.66	9104	4839.48	1.47406589	0.04814794	0.40294196	3.466823243	1.793614282	up	C17537	-
MW0146024	Asp-Arg-His-Lys	Amino acids and derivatives	Amino acids and derivatives	C22H38N10O7	3	0.6253	-	7728.89	5295.64	20919.4	43033.64	40820.73	27172.73	1.47838492	0.02080181	0.31900915	0.30572653	-1.70968635	down	-	-
MW0150513	Gly-Tyr-Asp	Amino acids and derivatives	Amino acids and derivatives	C15H19N3O7	3	0.6235	-	20929.6	24432.66	24916.22	363.68	184.28	5458.13	1.51484847	0.00086993	0.12883555	11.70120328	3.54858499	up	-	-
MW0146142	Asp-Ile-Gln	Amino acids and derivatives	Amino acids and derivatives	C15H26N4O7	3	0.6231	-	46912.07	53299.02	54350	11.22	22325.73	16901.04	1.00646854	0.02007482	0.31365607	3.93906747	1.977854128	up	-	-
MW0150351	Gly-Glu-Gln	Amino acids and derivatives	Amino acids and derivatives	C12H20N4O7	3	0.6217	-	7068.12	2914.99	3927.17	376.42	2086.89	420.34	1.46364868	0.08143837	0.47136621	4.82384478	2.270183485	up	-	-
MW0150529	Gly-Val-Asp	Amino acids and derivatives	Amino acids and derivatives	C11H19N3O6	3	0.6188	-	14927.32	8316.29	14542.02	2570.69	9026.64	1673.35	1.34404596	0.06104292	0.43919191	2.847301721	1.50959538	up	-	-
MEDP0154	5,6-dihydrothymine	Nucleotides and derivatives	Nucleotides and derivatives	C5H8N2O2	3	0.6123	-	154.92	363.75	1194.81	2656.54	1807.87	2530.61	1.44206622	0.01398624	0.27083212	0.244957127	-2.02939883	down	C00906	-
MW0103729	2-(2,6-Dimethylmorpholin-4-yl)-6-(3-methylpiperidin-1-yl)-5-nitropyrimidin-4-amine	Heterocyclic compounds	Heterocyclic compounds	C16H26N6O3	3	0.6095	-	84.06	27.85	39.83	3.58	16.89	22.18	1.29077867	0.15794746	0.5507933	3.557796014	1.830983796	up	-	-
MW0057438	PC(20:5(5Z,8Z,11Z,14Z,17Z)/24:1(15Z))	GL	PC	C52H92NO8P	3	0.608	-	360.71	526.41	42.35	1499.91	1415.8	978.78	1.33595754	0.01046159	0.24844069	0.238662829	-2.06695421	down	-	-
MW0167227	[(2S)-1-[(4Z,7Z,10Z,13Z,16Z,19Z)-docosa-4,7,10,13,16,19-hexaenoyl]oxy-3-pentadecanoyloxypropan-2-yl] docosanoate	GL	TG	C62H108O6	3	0.6067	-	2581.76	1070.9	1043.51	86.02	204.36	1025.54	1.29045038	0.14500948	0.53579335	3.568735181	1.83541285	up	-	-
MW0007659	Methyl 2-(2-hydroxy-6-methoxy-4-methylbenzoyl)-3-methoxy-5-sulfooxybenzoate	Benzene and substituted derivatives	Benzene and substituted derivatives	C18H18O10S	3	0.6044	-	2791.45	1558.77	2301.66	875.78	949.86	30.33	1.15368057	0.02773637	0.33858973	3.584045001	1.841588752	up	-	-
MW0161842	cephalosporin C(1-)	Organic acids	Organic acids	C16H20N3O8S-	3	0.6027	-	4678.33	2338.96	2347.87	1805.48	332.9	388.08	1.407785	0.07994892	0.47056257	3.706830902	1.890186304	up	-	-
MEDP1400	Carnitine C16:3	Lipids	CAR	C23H39NO4	3	0.6019	-	309.61	337.28	156.2	224.26	66.76	106.03	1.1816333	0.14174999	0.53210743	2.022641985	1.01624098	up	-	-
MW0168256	3,7-Dimethyl-9-(2,6,6-trimethylcyclohex-1-en-1-yl)nona-2,4,6,8-tetraenoic acid	Others	Vitamin	C20H28O2	3	0.6018	-	5989.28	6632.56	5585.81	1151.37	1956.37	2944.45	1.59351333	0.00524266	0.19663582	3.00843992	1.589015546	up	-	-
MW0146112	Asp-Gly-His	Amino acids and derivatives	Amino acids and derivatives	C12H17N5O6	3	0.5987	-	11785.14	4386.1	8131.93	12885.73	17546.23	19944.19	1.37942959	0.04327513	0.39461781	0.482434049	-1.05159636	down	-	-
MW0144559	Ala-Glu-Gln-Tyr-Leu	Amino acids and derivatives	Amino acids and derivatives	C28H42N6O10	3	0.5978	-	953.76	4094.12	1262.39	12963.96	13023.32	7343.54	1.55620666	0.02357538	0.33077008	0.189322375	-2.40108317	down	-	-
MW0127867	(2-{9-hydroxy-2-oxo-2H,8H,9H-furo[2,3-h]chromen-8-yl}-2-[(3-methylbut-2-enoyl)oxy]propoxy)sulfonic acid	Organic acids	Organic acids	C19H20O10S	3	0.5944	-	1573.8	60.53	998.08	8126.18	9354.63	462.76	1.0118476	0.2056076	0.60068239	0.14670492	-2.76901084	down	-	-
MW0159211	Val-Tyr-Gln	Amino acids and derivatives	Amino acids and derivatives	C19H28N4O6	3	0.5916	-	41503.52	24304.15	37302.46	9757.12	15005.22	15835.69	1.58486077	0.04339892	0.39461781	2.53978161	1.344704448	up	-	-
MW0013188	2-(Sulooxymethyl)butanoic acid	Organic acids	Organic acids	C5H10O6S	3	0.5897	-	4313.64	16863.61	14782.43	5373.27	3873.52	373.51	1.15700974	0.14008901	0.53210743	3.737895908	1.902226394	up	-	-
MEDP1429	Carnitine C5:DC	Lipids	CAR	C12H21NO6	3	0.5877	-	1498.23	1054.87	1387	93.05	535.12	350.67	1.46478692	0.00595011	0.2013045	4.025274815	2.009087283	up	-	-
MW0148737	dTDP-4-amino-2,4-dideoxy-beta-L-xylose	Nucleotides and derivatives	Nucleotides and derivatives	C15H25N3O13P2	3	0.5877	-	7114.62	26192.15	27304.21	4199.28	435.94	3558.93	1.39931417	0.11235853	0.50205214	7.396859955	2.886912961	up	C21354	ko01110
MW0123903	ethyl {10-[3-(morpholin-4-yl)propanoyl]-5-oxido-10H-phenothiazin-2-yl}carbamate	Others	Lactones	C22H25N3O5S	3	0.5872	-	176.41	65.04	217.64	22.54	14.61	25.48	1.61617269	0.10046545	0.49047358	7.330193198	2.873851223	up	-	-

MW0134125	5,7-Dihydroxy-2-phenyl-8-[3,4,5-trihydroxy-6-(hydroxymethyl)oxan-2-yl]-6-(3,4,5-trihydroxyoxan-2-yl)chromen-4-one	Others	Ketone compounds	C26H28O13	3	0.5849	-	74558.39	88657.27	72966.02	102.86	6817.22	666.19	1.55902309	0.00129026	0.13399001	31.13278067	4.960362534	up	-	-
MW0110190	Tyr-Asp-Arg	Amino acids and derivatives	Amino acids and derivatives	C19H28N6O7	3	0.5848	-	28878.51	45689.4	28371.76	3160.05	1576.61	950.47	1.70704064	0.02809634	0.33858973	18.10046016	4.17795447	up	-	-
MW0155805	Pro-Asp-Ala-Lys-Ser	Amino acids and derivatives	Amino acids and derivatives	C21H36N6O9	3	0.584	-	3931.2	1471.16	4346.94	632.93	1823.86	646.96	1.34750609	0.11713952	0.50205214	3.141135723	1.651286281	up	-	-
MW0003446	3-(3,4-dimethoxyphenyl)-N-[2-(3,4-dimethoxyphenyl)-2-(sulfooxy)ethyl]propanimidic acid	Benzene and substituted derivatives	Benzene and substituted derivatives	C21H27NO9S	3	0.5804	-	12147	11035.58	4538.11	3639.15	633.72	3801.4	1.27958786	0.09365719	0.48592036	3.433213157	1.779559432	up	-	-
MW0016463	Cdp-DG(18:1(11Z)/22:3(10Z,13Z,16Z))	GL	DG	C52H89N3O15P2	3	0.579	-	17320.79	14960.01	11015.92	130225.63	120670.05	119848.87	1.74815061	7.0086E-05	0.04593672	0.11678316	-3.09809584	down	-	-
MW0150484	Gly-Thr-Ala	Amino acids and derivatives	Amino acids and derivatives	C9H17N3O5	3	0.5777	-	157.32	94.51	142.28	27.87	20.37	18.11	1.71632053	0.02638238	0.33612848	5.939864356	2.570429986	up	-	-
MW0135986	6-{[4,5-dihydroxy-2-({7-oxo-7H-furo[3,2-g]chromen-9-yl}oxy)oxan-3-yl]oxy}-3,4,5-trihydroxyoxane-2-carboxylic acid	Organic acids	Organic acids	C22H22O14	3	0.5771	-	481656.52	462029.72	401461.51	924771.09	1144156.62	840992.41	1.69776592	0.02262427	0.33066403	0.462262775	-1.1132149	down	-	-
ZINC4440470	2",6"-Diacetyloninin	Others	Lactones	C26H26O11	3	0.5732	-	7834.01	8615	1540.5	1696.98	3600.01	990.86	1.01386588	0.21725531	0.60875495	2.860995412	1.516517184	up	-	-
MW0156143	Pro-TGF-alpha	Amino acids and derivatives	Amino acids and derivatives	C52H87N15O17	3	0.5729	-	637.6	2404.21	1342.07	498.52	698.54	247.77	1.29426336	0.19101203	0.58370725	3.034183952	1.601308554	up	-	-
MW0139843	Torvanol A	Heterocyclic compounds	Heterocyclic compounds	C20H20O10S	3	0.5705	-	3032.06	136.08	2811.78	12075.98	19215.74	10145.93	1.36603209	0.03914398	0.38297158	0.144311272	-2.7927441	down	-	-
MW0113729	6-{5-[(1E)-3-[(3-carboxy-5,6-dihydroxycyclohex-3-en-1-yl)oxy]-3-oxoprop-1-en-1-yl]-2-hydroxyphenoxy}-3,4,5-trihydroxyoxane-2-carboxylic acid	Organic acids	Organic acids	C22H24O14	3	0.5695	-	676995.37	855787.89	773810.46	81.98	7734.95	482.72	1.64860346	0.00444377	0.18300962	277.9145771	8.118497698	up	-	-
MW0146084	Asp-Gln-Leu-Thr-Glu	Amino acids and derivatives	Amino acids and derivatives	C24H40N6O12	3	0.5693	-	24544.15	41947.1	50970.65	21956.62	7675.68	17292.69	1.37684613	0.07384563	0.46133776	2.503184337	1.323764537	up	-	-
MW0001005	{[1-(4-hydroxy-3-methoxyphenyl)-3-oxobutan-2-yl]oxy}sulfonic acid	Phenolic acids	Phenolic acids	C11H14O7S	3	0.5687	-	27121.03	9314.09	18667.26	47379.32	49978.14	31898.7	1.41748083	0.03214597	0.35051264	0.426303706	-1.2300465	down	-	-
ZINC1337858	boeravinone F	Others	Ketone compounds	C17H10O7	3	0.5667	-	2009.9	1320.12	804.63	8179.13	4135.97	5921.34	1.61624544	0.04701527	0.39857834	0.226724624	-2.14098701	down	-	-
ZINC3898355	(16R)-17-Hydroxykaura-18-oic acid	Organic acids	Organic acids	C20H32O3	3	0.5618	-	41005.28	53353.6	72769.22	1411	35937.91	32123.88	1.02219997	0.08717776	0.47762164	2.405662706	1.266434379	up	-	-
MW0145997	Asn-Tyr	Amino acids and derivatives	Amino acids and derivatives	C13H17N3O5	3	0.5601	-	38601.98	38087.99	40254.7	11470.05	9789.68	8402.7	1.74602862	2.498E-05	0.03690435	3.942518196	1.979117413	up	-	-
MW0156854	Ser-Lys-Val-Glu	Amino acids and derivatives	Amino acids and derivatives	C19H35N5O8	3	0.5549	-	5050.04	3771.01	5229.46	8071.52	20757.91	8743.54	1.43498424	0.19612914	0.59011837	0.373952605	-1.41907266	down	-	-
MW0166505	prenylated FMNH2	Nucleotides and derivatives	Nucleotides and derivatives	C22H29N4O9P-2	3	0.554	-	932.15	985.48	1046.63	1434.99	3888.12	1542.54	1.32014786	0.24556487	0.63275786	0.431752274	-1.21172432	down	-	-
ZINC5958755	Tonkinensine B	Others	Ketone compounds	C28H26N2O6	3	0.5517	-	87232.36	40418.54	45683.6	20031.79	20154.69	7223.84	1.49272658	0.09529287	0.48768821	3.656049991	1.870285797	up	-	-
MW0014120	3-Hydroxysintaxanthin	Others	Ketone compounds	C31H42O2	3	0.5514	-	319.65	155.84	129.99	62.69	4.5	42.6	1.35687955	0.09885141	0.48888534	5.514892067	2.463332651	up	-	-
MW0155388	Phe-Tyr-Lys-Arg	Amino acids and derivatives	Amino acids and derivatives	C30H44N8O6	3	0.5513	-	566.69	154.63	157.35	63.2	71.17	161.69	1.20307049	0.2898926	0.66011135	2.967878133	1.569431853	up	-	-
MW0166443	(+)-sesaminol 2-O-beta-D-gentiobioside	Lignans and Coumarins	Lignans	C32H38O17	3	0.5498	-	892.11	325.92	1598.5	356.25	94.07	443.29	1.1432883	0.21842036	0.60939029	3.151855955	1.656201603	up	-	-
MW0161445	(2S)-3,4-dihydro-2H-pyrrole-2-carboxylate	Organic acids	Organic acids	C5H6NO2-	3	0.5478	-	62186.24	37574.11	35617.43	24571.75	18758.9	19495.86	1.55371663	0.0995956	0.48935482	2.154787525	1.107545618	up	-	-
MW0167120	[(2S)-1-pentadecanoyloxy-3-tetradecanoyloxypropan-2-yl] octadecanoate	GL	TG	C50H96O6	3	0.5461	-	95.32	1417.45	683.74	2651.05	1129.75	2277.23	1.13225853	0.09926091	0.48888534	0.362578264	-1.46363565	down	-	-
MW0158585	Tyr-Pro-Gln	Amino acids and derivatives	Amino acids and derivatives	C19H26N4O6	3	0.5429	-	583.96	125.59	93.57	50.54	1.51	2.31	1.43704353	0.25502539	0.64155925	14.7740986	3.884998206	up	-	-
MW0145854	Asn-Phe-Glu-Tyr-Ile	Amino acids and derivatives	Amino acids and derivatives	C33H44N6O10	3	0.5396	-	9216.34	13128.43	12705.69	3417.81	7834.75	3318.42	1.48581014	0.02570889	0.33612848	2.405497777	1.266335466	up	-	-

MW0154136	N1,N5,N10-tricaffeoyl spermidine	Heterocyclic compounds	Heterocyclic compounds	C34H37N3O9	3	0.5367	-	8624.23	6363.23	11172.43	19598	34585.83	8100.08	1.10803248	0.25483339	0.64155925	0.420010401	-1.25150304	down	C18070	ko00940,ko01110
MW0144811	Ala-Val-Val	Amino acids and derivatives	Amino acids and derivatives	C13H25N3O4	3	0.5347	-	59.82	39.99	43.61	625.66	60.87	292.08	1.31320466	0.23143117	0.62319286	0.146554807	-2.7704878	down	-	-
MW0113639	6-{[3-(1,2-dihydroxybutyl)-1-oxo-1H-isochromen-5-yl]oxy}-3,4,5-trihydroxyoxane-2-carboxylic acid	Organic acids	Organic acids	C19H22O11	3	0.5331	-	386.03	1158.5	1456.09	1913.68	2808.67	2111.6	1.30965274	0.03925432	0.38320518	0.439075498	-1.18745907	down	-	-
MW0068675	TG(14:1(9Z)/22:2(13Z,16Z)/o-18:0)	GL	TG	C57H106O5	3	0.532	-	1022.05	241.73	1037.95	3877.97	1010.19	3288.5	1.25804626	0.14549158	0.53579335	0.281500026	-1.82879304	down	-	-
MW0127719	({6-[5,7-dihydroxy-2-(4-hydroxyphenyl)-4-oxo-3,4-dihydro-2H-1-benzopyran-8-yl]-3,4,5-trihydroxyoxan-2-yl}methoxy)sulfonic acid	Organic acids	Organic acids	C21H22O13S	3	0.532	-	900.95	1036.46	1152.5	1760.67	2731.71	4073.67	1.56019568	0.11094359	0.50205214	0.360715849	-1.47106528	down	-	-
MW0166608	cyanidin 3-O-beta-D-(caffeoyl)-sambubioside	Flavonoids	Anthocyanidins	C35H34O18	3	0.5317	-	835.03	4699.36	3485.66	1383.68	558.79	297.88	1.23571141	0.17905238	0.57366954	4.026178945	2.009411295	up	-	-
MW0161020	(2S)-4-(2-aminophenyl)-2-azaniumyl-4-	Others	Lactones	C10H12N2O3	3	0.5247	-	25121.33	26122.23	19982.38	10380.36	10465.3	93.23	1.06348966	0.02195335	0.32870685	3.401610114	1.766217792	up	-	-
MW0062551	PS(16:2(9Z,12Z)/22:6(4Z,7Z,10Z,13Z,16Z,19Z))	GL	PS	C46H74NO10P	3	0.5246	-	641.56	183.3	1402.24	5034.21	2204.8	977.71	1.19527393	0.23350997	0.62494939	0.271044894	-1.88339627	down	-	-
MW0108246	methyl N-[(2-nitrophenyl)sulfonyl]glycinate	Others	Lactones	C9H10N2O6S	3	0.5191	-	3716.04	1813.25	2613.19	578.45	767.6	846.12	1.6507963	0.06657443	0.44856776	3.714346971	1.893108589	up	-	-
MW0152399	Leu-Leu-Leu-Leu-Ser	Amino acids and derivatives	Amino acids and derivatives	C27H51N5O7	3	0.5172	-	131776.59	116917.39	136898.25	60206.33	55960.7	52254.41	1.74098761	0.0029194	0.18110516	2.2894486	1.195000176	up	-	-
MW0110238	L-Tyrosyl-L-Asparagine	Amino acids and derivatives	Amino acids and derivatives	C13H17N3O5	3	0.5169	-	61151.73	90510	76105.16	52064	25416.83	18331.56	1.4501946	0.03144831	0.34952497	2.377217498	1.249273905	up	-	-
MW0105251	alpha-AMINO-3-HYDROXY-5-METHYL-4-ISOXAZOLEPROPIONIC ACID	Organic acids	Organic acids	C7H10N2O4	3	0.5157	-	1416.86	1209.63	508.28	1942.89	3103.02	2700.99	1.43573423	0.02634114	0.33612848	0.404648311	-1.30525952	down	C13672	-
MW0163764	2-Aminobut-2-enoate	Others	Lactones	C4H6NO2-	3	0.5128	-	17968.31	17072.48	17080.81	363.32	492.14	159.11	1.73710173	8.4585E-05	0.04686027	51.37309402	5.68294106	up	-	-
MW0056974	1-Octadecanoyl-2-tetracosanoyl-sn-glycero-3-phosphocholine	GL	PC	C50H100NO8P	3	0.5112	-	145.19	764.04	261.35	804.37	1346.27	2409.21	1.38930194	0.12507945	0.51699391	0.256714585	-1.96176283	down	-	-
MW0059289	PE-NMe(14:1(9Z)/22:6(4Z,7Z,10Z,13Z,16Z,19Z))	GL	PE	C42H70NO8P	3	0.5078	-	13014.8	6985.86	13847.51	492.76	5946.89	2551.16	1.31319486	0.04108	0.39037745	3.764752008	1.912554835	up	-	-
MW0011391	(5R,9S,15R)-5,9-dimethyl-15-[(Z)-2-methylbut-2-enoyl]oxy-14-methylidenetetracyclo[11.2.1.01,10.04,9]hexadecane-5-carboxylic acid	Organic acids	Organic acids	C25H36O4	3	0.506	-	656.74	1149.01	873.56	1183.38	2391.4	2363.94	1.41778133	0.09902381	0.48888534	0.451159509	-1.1482905	down	-	-
pme0376	Naringenin	Flavonoids	Flavanones	C15H12O5	2	0.9991	480-41-1	223.8	4441.16	192497.66	218745.61	813029.24	308360.8	1.27294857	0.16618649	0.56418482	0.14712139	-2.76492108	down	C00509	ko00941,ko00943,ko01100,ko01110
MEDL00410	1,3-Dihydroxy-6-methoxy-8-methyl-9-xanthenone	Flavonoids	Other Flavonoids	C15H12O5	2	0.9308	22938-77-8	14855.14	3057.02	57922.19	58687.61	686439.55	157026.89	1.33951598	0.29283166	0.66139816	0.084059203	-3.57245042	down	-	-
MW0112732	Isochlorogenic acid A	Phenolic acids	Phenolic acids	C25H24O12	2	0.9071	2450-53-5 | 89919-62-0 | 879305-14-3	8577.77	10700.33	15381.79	48485.99	114014.55	72885.93	1.67778438	0.07130422	0.45866819	0.147246739	-2.76369242	down	C02733	-
MW0008260	N-Caffeoylputrescine	Alkaloids	Phenolamine	C13H18N2O3	2	0.8959	29554-26-5 | 26148-06-1	1902.1	18225.95	4616.19	10047.43	28379.08	14827.4	1.05550882	0.27210604	0.64977045	0.464646446	-1.10579472	down	C03002	-
MW0119612	3-Hydroxy-3-methyloxindole	Alkaloids	Plumerane	C9H9NO2	2	0.8902	3040-34-4	823.32	823.13	1652.69	1222.58	141.65	161.79	1.12962519	0.26474093	0.64511688	2.161924483	1.11231613	up	C05834	ko00380
MW0055346	Nonadecanoic acid	Lipids	Free Lipidstty acids	C19H38O2	2	0.8837	646-30-0	2876.37	50292.87	1576.44	188706.22	418899.35	160681.2	1.5024681	0.09561477	0.48877125	0.07125683	-3.81082788	down	C16535	-
MW0000104	7-Ethyl-10-(4-N-aminopentanoic acid)-1-piperidino)carbonyloxycamptothecin	Alkaloids	Quinoline alkaloids	C33H38N4O8	2	0.8831	181467-56-1	9451.28	8660.3	8887.94	408.75	9218.55	319.17	1.23638974	0.1932818	0.58753425	2.714482625	1.44067725	up	-	-
MW0053624	Ginsenoside Rg3	Terpenoids	Triterpene Saponin	C42H72O13	2	0.8784	14197-60-5 | 11019-45-7	735.85	671.03	119.09	3733.63	2555.76	826.64	1.3021241	0.15206382	0.54659112	0.214441198	-2.22134599	down	C20778	ko00999,ko01110
MW0139710	Silicristin	Flavonoids	Flavanonols	C25H22O10	2	0.869	33889-69-9	16597.96	12726.84	9053.43	4188.74	3606.23	5103.32	1.64673444	0.05491923	0.42471427	2.975451009	1.573108364	up	C08717	-
MW0103346	2'-Deoxyguanosine-5'-diphosphate	Nucleotides and derivatives	Nucleotides and derivatives	C10H15N5O10P2	2	0.8583	3493-09-2	9787.08	11394.93	7031.06	4578.18	2111.72	5833.15	1.39608379	0.03681412	0.37270445	2.252891269	1.171777686	up	C00361	ko00230,ko01100,ko01232
MW0011760	[6]-Gingerdiol 3,5-diacetate	Others	Lactones	C21H32O6	2	0.8543	195204-05-8	2799.58	551.26	477.93	271.02	414.29	114.29	1.23520602	0.31577074	0.67380641	4.788356678	2.259530621	up	-	-
MEDN1742	3-Hydroxycoumarin	Lignans and Coumarins	Coumarins	C9H6O3	2	0.8521	939-19-5	397.48	868.69	5498.61	13598.03	27701.1	9425.24	1.46225624	0.10788967	0.49971175	0.133363509	-2.90656412	down	-	-

MW0111715	Xylobiose	Others	Saccharides	C10H18O9	2	0.8457	6860-47-5 | 83113-52-4	12.76	22.47	29.86	154.86	163.99	201.49	1.70906106	0.00456179	0.18379856	0.125091286	-2.9989468	down	C01630	ko02010
MW0169501	Myricitrin	Flavonoids	Flavonols	C21H20O12	2	0.828	17912-87-7	1118.57	1271.55	948.37	171.09	908.63	235.68	1.3337216	0.08850192	0.47951163	2.538003649	1.343694143	up	C10108	-
MW0138806	Lonchocarpic acid	Organic acids	Organic acids	C26H26O6	2	0.8201	5490-47-1	1008.62	2669.51	2226.93	639.08	791.19	1064.5	1.35971836	0.14227791	0.53210743	2.366975713	1.243044903	up	-	-
MW0011993	10-Deacetylbaccatin III	Benzene and substituted derivatives	Benzene and substituted derivatives	C29H36O10	2	0.8149	32981-86-5	6087.69	637.56	5278.93	740.62	934.3	397	1.19523365	0.18956306	0.58166266	5.793746863	2.534496653	up	-	-
MW0108880	N-omega-Propyl-L-arginine	Amino acids and derivatives	Amino acids and derivatives	C9H20N4O2	2	0.8128	137361-05-8	764.9	807.38	681.81	22.77	65.01	243.08	1.522251	0.00321535	0.18110516	6.812821133	2.76825233	up	-	-
MW0109227	Phenylalanylserine	Amino acids and derivatives	Amino acids and derivatives	C12H16N2O4	2	0.7993	16053-39-7	37.06	48.06	16.23	5974.84	5846.46	3529.4	1.74319691	0.02356351	0.33077008	0.006602305	-7.24281455	down	-	-
MW0108192	H-Met-arg-phe-ala-OH	Amino acids and derivatives	Amino acids and derivatives	C23H37N7O5S	2	0.7985	67368-29-0	37938.42	35209.36	41962.67	13552.09	19925.98	16017.65	1.69286709	0.0012791	0.13399001	2.325664724	1.217643128	up	-	-
MW0144813	H-Ala-Abu-OH	Amino acids and derivatives	Amino acids and derivatives	C7H14N2O3	2	0.7862	39537-33-2	36.9	46.13	65.22	19.7	12.47	20.83	1.60601786	0.05180937	0.4118154	2.797169811	1.48396784	up	-	-
MEDN0441	Eudesmic acid	Phenolic acids	Phenolic acids	C10H12O5	2	0.7832	118-41-2	117.43	89.94	34.77	20.62	12.24	43.29	1.31625255	0.13834002	0.52946715	3.179776756	1.668925481	up	-	-
MW0132545	3,7,4'-Trihydroxyflavone	Flavonoids	Flavones	C15H10O5	2	0.7809	2034-65-3	114.69	83.96	5879.11	6937.21	15145.2	5635.8	1.30397341	0.12335696	0.51286873	0.21926957	-2.18922248	down	C10037	-
MW0015605	Agnuside	Phenolic acids	Phenolic acids	C22H26O11	2	0.7787	11027-63-7	2215.5	3187.59	2364.35	1254.78	697.97	1256.83	1.57841813	0.01899179	0.30945448	2.420079886	1.275054671	up	C09765	-
MW0005557	5-Aminoimidazole-4-carboxamide	Alcohol and amines	Amines	C4H6N4O	2	0.7747	360-97-4	8118.3	2838.55	11403.41	2387.9	2675.28	2918.72	1.26903896	0.19424528	0.58775794	2.801370601	1.486132854	up	C04051	ko00230,ko01100
MW0133358	1-(2-Hydroxyphenyl)-3-(4-hydroxyphenyl)-2-propen-1-one	Others	Ketone compounds	C15H12O3	2	0.7735	13323-66-5	204.66	129.72	130.71	21.67	55.03	5.88	1.47963482	0.01829883	0.30148847	5.631993219	2.493645597	up	-	-
MW0138479	Isoginkgetin	Flavonoids	Biflavones	C32H22O10	2	0.7712	548-19-6	203.49	541.71	26868.86	39131.44	151689.49	62799.89	1.38162025	0.15193844	0.54658373	0.10887931	-3.19919826	down	-	-
MEDP2160	Oroxylin A	Flavonoids	Flavones	C16H12O5	2	0.771	480-11-5	1509.29	1801.28	64508.8	47733.96	124346.01	72501	1.26701339	0.12866536	0.51982213	0.277288008	-1.85054287	down	-	-
MW0002264	2,4-Dihydroxy-3,6-dimethylbenzoic acid	Benzene and substituted derivatives	Benzene and substituted derivatives	C9H10O4	2	0.7685	4707-46-4	87.79	589.36	337.36	145.3	57.86	108.67	1.09171412	0.24498627	0.63271123	3.253407305	1.70195145	up	-	-
MW0005958	AcetylsulLipidsmethoxazole	Benzene and substituted derivatives	Benzene and substituted derivatives	C12H13N3O4S	2	0.7684	21312-10-7	5112.57	2154.01	16202.48	17719.93	42116.08	18670.84	1.31539277	0.13393492	0.52577462	0.298942831	-1.74205848	down	C13061	-
MW0168807	Baicalin	Flavonoids	Flavones	C21H18O11	2	0.7682	21967-41-9 | 31564-28-0	7445.4	36927.5	83987.32	67957.6	200961.62	88287.15	1.13090478	0.20085741	0.5961949	0.359344712	-1.47655964	down	C10025	-
MW0158681	Tyr-Ala	Amino acids and derivatives	Amino acids and derivatives	C12H16N2O4	2	0.765	730-08-5	469.06	247.43	285.17	27.37	18.35	28.73	1.73067786	0.04543743	0.39546247	13.45413029	3.749977229	up	-	-
MW0109576	S-allyl-L-cysteine	Amino acids and derivatives	Amino acids and derivatives	C6H11NO2S	2	0.7645	21593-77-1	4176.3	7401.86	5190.09	2464.24	1416.89	752.45	1.54021965	0.03246249	0.3516194	3.618854104	1.855532946	up	-	-
MEDL02603	7,3',4'-Trihydroxyflavone	Flavonoids	Flavones	C15H10O5	2	0.7615	2150-11-0 | 104666-14-0	133.08	46.59	66.59	53.36	23.72	12.99	1.2711169	0.17429327	0.57211065	2.734095703	1.451063743	up	-	-
MW0137266	Acteoside	Heterocyclic compounds	Heterocyclic compounds	C29H36O15	2	0.761	61276-17-3 | 22323-52-0	1785.85	51.82	873.94	4640.25	6843.44	2192.66	1.24723525	0.09886769	0.48888534	0.198270006	-2.33446165	down	-	-
MW0000559	(2,3-Dihydroxy-4-methoxyphenyl)(phenyl)methanone	Benzene and substituted derivatives	Benzene and substituted derivatives	C14H12O4	2	0.7597	35836-41-0	2476.56	795.18	391.15	2433.15	4090.84	1235.24	1.03492013	0.26576726	0.64647667	0.472068749	-1.08293112	down	-	-
MW0132828	3,9- [illegible]	Flavonoids	Isoflavones	C15H12O4	2	0.7582	61135-91-9	111.26	477.27	5930.89	2866.22	7761.91	3756.78	1.07705857	0.34052461	0.6939065	0.453212429	-1.14174067	down	C04271	ko00943,ko01110
MW0168471	2-(1h-1,2,4-triazol-5-yl)-1h-isoindole- [illegible]	Alkaloids	Plumerane	C10H6N4O2	2	0.7475	59208-47-8	11530.25	20099.41	124435.71	116260.1	469834.27	211272.89	1.35675587	0.17118222	0.5671048	0.195725832	-2.35309392	down	-	-
MW0126431	Resorcinolnaphthalein	Heterocyclic compounds	Heterocyclic compounds	C24H14O5	2	0.7465	41307-63-5	4714.99	5071.56	1675.83	204.7	4159.14	466.1	1.09617907	0.25814486	0.64187626	2.373193042	1.246829458	up	-	-
MW0010059	Vialinin A	Benzene and substituted derivatives	Benzene and substituted derivatives	C34H26O8	2	0.7464	858134-23-3	293.76	146.39	106.85	287.83	949.44	517.05	1.37838965	0.16582802	0.56418482	0.311801724	-1.68129919	down	-	-
MW0148485	[illegible]	Alkaloids	Alkaloids	C35H44N2O9	2	0.746	6335-49-5	4843.54	5857.97	4291.3	1072.26	1697.39	1828.03	1.68016095	0.00691242	0.2204017	3.260951175	1.70529284	up	-	-
MW0011607	(R)-dihydrolipoic acid	Organic acids	Organic acids	C8H16O2S2	2	0.7379	119365-69-4	10776.47	3822.03	6584.54	58133.49	22851.85	26457.19	1.56912277	0.11998928	0.50550617	0.197156936	-2.34258363	down	C02147	ko00785,ko01100
MW0006499	Carboxytolbutamide	Benzene and substituted derivatives	Benzene and substituted derivatives	C12H16N2O5S	2	0.7378	2224-10-4	12000.19	1003.49	4706.58	209.56	2314.72	797.26	1.12046971	0.27400497	0.65054534	5.331942412	2.414661199	up	-	-
MW0103360	3',5'-Cyclic IMP	Nucleotides and derivatives	Nucleotides and derivatives	C10H11N4O7P	2	0.7377	3545-76-4	331.37	145.94	582.57	154.02	74.88	96.35	1.34506489	0.18908703	0.58166266	3.258662567	1.70427997	up	C00943	-
MW0137947	Dicumarol	Lignans and Coumarins	Coumarins	C19H12O6	2	0.7373	66-76-2	1236.6	1376	3514.78	1471.53	999.14	249.28	1.04321811	0.26281827	0.64368675	2.252754646	1.171690194	up	C00796	-
MW0126911	Uncarine F	Alkaloids	Plumerane	C21H24N2O4	2	0.7341	14019-66-0	6170	7091.9	2487.37	1094.26	1078.74	1211.73	1.6015281	0.09927726	0.48888534	4.65303584	2.2181723	up	C17598	-
MW0125626	N-Acetyl-L-tryptophanamide	Alcohol and amines	Amines	C13H15N3O2	2	0.7333	2382-79-8	219902.82	236487.03	261796.67	86368.22	74661.88	60329.06	1.72884382	0.0008343	0.12883555	3.244440031	1.6979695	up	-	-
MW0159267	Val-His	Amino acids and derivatives	Amino acids and derivatives	C11H18N4O3	2	0.7331	13589-07-6 | 51077-05-5	19051.82	23586.66	22543.96	11216.67	2695.81	11372.76	1.33979755	0.02711497	0.33851703	2.577884964	1.366187886	up	-	-
MEDP1879	Phe-ala	Amino acids and derivatives	Amino acids and derivatives	C12H16N2O3	2	0.7298	3918-87-4	188.83	253.52	354.78	168.37	9.04	170.91	1.01158717	0.10736816	0.49971175	2.288499081	1.194401713	up	-	-
MW0113887	alpha,beta-Trehalose	Others	Saccharides	C12H22O11	2	0.7259	585-91-1	3734.79	8724.55	11315.62	460.54	7070.89	2224.55	1.06988411	0.19238201	0.58600486	2.436962765	1.285084208	up	-	-
MW0116617	Atalaphylline	Heterocyclic compounds	Heterocyclic compounds	C23H25NO4	2	0.7207	28233-35-4 | 28233-34-3	9675.7	19876.25	20573.74	1728.23	12742.66	4600.94	1.23117639	0.09882095	0.48888534	2.628258012	1.39410691	up	C10645	-
MW0120912	5-(Tetradecyloxy)-2-furoic acid	Organic acids	Organic acids	C19H32O4	2	0.7195	54857-86-2	618.03	107.29	78.79	738.06	1157.25	1531.73	1.42318041	0.04227148	0.39256349	0.234636888	-2.09149825	down	-	-

MEDP1608	cyclic ADP-ribose	Others	Saccharides	C15H21N5O13P2	2	0.7161	119340-53-3	2292.3	2657.69	7091.03	8783.28	10367.84	8059.41	1.39675676	0.0643712	0.44624322	0.442513248	-1.17620745	down	-	-
MW0109216	L-phenylalanyl-L-valine	Amino acids and derivatives	Amino acids and derivatives	C14H20N2O3	2	0.7135	3918-90-9	48006.08	63722.73	55599.51	25609.48	41902.81	13033.07	1.35153666	0.05379243	0.42047275	2.077442077	1.054808253	up	-	-
MW0015406	(-)-9,10-Dihydrojasmonic acid	Organic acids	Organic acids	C12H20O3	2	0.711	76968-33-7 | 98674-52-3	38.81	49.29	151.56	800.64	931.96	482.28	1.63969807	0.03157981	0.34952497	0.108204508	-3.20816749	down	-	-
MW0122798	alpha-Mangostin	Flavonoids	Other Flavonoids	C24H26O6	2	0.7081	6147-11-1	626.67	2729.75	6905.1	5450	13492.11	12349.94	1.21454783	0.09372847	0.48592036	0.327927381	-1.60855173	down	C10080	-
MW0110425	Val-Thr	Amino acids and derivatives	Amino acids and derivatives	C9H18N2O4	2	0.7036	72636-02-3	191.01	201.83	104.07	817.58	1118.39	730.38	1.69184518	0.01981469	0.31365607	0.186363381	-2.42380969	down	-	-
MW0124060	Ethyl 2-amino-4-methylthiazole-5-carboxylate	Others	Lactones	C7H10N2O2S	2	0.7015	7210-76-6	213164	262019.95	672756.98	245315.55	158036.99	153974.71	1.09925039	0.30802137	0.67088493	2.059725108	1.042451807	up	-	-
MADP0567	Zebularine	Nucleotides and derivatives	Nucleotides and derivatives	C9H12N2O5	2	0.701	3690-10-6	23117.6	22263.41	240174.06	338933.22	897092.67	380732.72	1.4150906	0.11786986	0.50279038	0.176621957	-2.50126339	down	-	-
MW0049195	Dehydrotomatin	Alkaloids	Steroid alkaloids	C50H81NO21	3	0.9974	157604-98-3	165777.71	151713.12	143922.53	77328.8	70150.26	71736.29	1.74468257	0.00308074	0.18110516	2.10484056	1.073710954	up	-	-
MW0141195	1-[(3R)-1-(1-adamantylmethyl)-2,4-dioxo-5-phenyl-1,5-benzodiazepin-3-yl]-3-phenylurea	Benzene and substituted derivatives	Benzene and substituted derivatives	C33H34N4O3	3	0.9958	167355-22-8	11784.52	12580.38	16470.89	26980.78	42266.93	12786.08	1.11675826	0.24599648	0.63276996	0.497792312	-1.00638415	down	-	-
MW0049137	Cynarasaponin D	Terpenoids	Steroid	C47H74O19	3	0.9851	117804-10-1	5.02	84.53	13.54	667.29	639.47	723.64	1.59995079	5.3915E-05	0.04593672	0.050773247	-4.29978767	down	-	-
MEDL02027	Ferulic acid	Phenolic acids	Phenolic acids	C10H10O4	3	0.9793	537-98-4	2634.1	1428.63	3967.78	342.49	989.22	843.56	1.46024209	0.10841424	0.4998897	3.691730222	1.88429713	up	C01494	ko00940,ko01100,ko01110
MW0015595	16-[5-[5-[4,5-Dihydroxy-6-(hydroxymethyl)-3-(3,4,5-trihydroxyoxan-2-yl)oxyoxan-2-yl]oxy-3,4-dihydroxy-6-(hydroxymethyl)oxan-2-yl]oxy-3,4-dihydroxy-6-(hydroxymethyl)oxan-2-yl]oxy-5',7,9,13-tetramethylspiro[5-oxapentacyclo[10.8.0.02,9.04,8.013,18]icosane-6,2'-oxane]-10-one	Terpenoids	Steroid	C50H80O23	3	0.9776	58546-17-1	2935.26	3026.83	665.13	1601.28	114.36	117.01	1.1975935	0.16885553	0.56577827	3.616195127	1.854472527	up	-	-
MEDL02751	2',3,4,4',6'-Pentahydroxychalcone	Flavonoids	Chalcones	C15H12O6	3	0.9651	14917-41-0 | 73692-51-0	5781.16	6697.69	25962.59	24684.94	66449.82	21835.13	1.24648736	0.2214326	0.61183871	0.340280406	-1.55520402	down	-	-
MEDL00096	2-Methylglutaric acid	Organic acids	Organic acids	C6H10O4	3	0.9629	617-62-9	5081.61	4425.72	8692.4	995.15	2870.05	1841.21	1.50157245	0.07225582	0.4595545	3.18934847	1.673261736	up	-	-
MEDN0531	Glucaric acid	Others	Saccharides	C6H10O8	3	0.9474	87-73-0	29226.97	30842.17	29574.52	16756.69	19800.89	935.43	1.05494561	0.09595137	0.48888534	2.390943272	1.257579901	up	C00818	ko00053,ko01100
MEDL00440	(+)-Catechin gallate	Flavonoids	Flavanols	C22H18O10	3	0.9464	1257-08-5	845.62	4906.68	870.82	407.77	689.03	81.3	1.23074288	0.31022401	0.67088493	5.621865716	2.491048994	up	-	-
MW0111506	(S)-Multifidol 2-[apiosyl-(1->6)-glucoside]	Benzene and substituted derivatives	Benzene and substituted derivatives	C22H32O13	3	0.9399	467437-62-3	9.67	71.53	124.91	138.54	1390.24	1631.3	1.36283227	0.16647614	0.56418482	0.065223032	-3.93847467	down	-	-
MEDL02152	Cyclic AMP	Nucleotides and derivatives	Nucleotides and derivatives	C10H12N5O6P	3	0.9311	60-92-4	16165.87	17834.59	20720.83	4406.93	8115.18	3562.74	1.63515406	0.00264443	0.18110516	3.402039186	1.766399759	up	C00575	ko00230,ko01100
MEDN0491	9S-HOTrE	Lipids	Free Lipidstty acids	C18H30O3	3	0.9247	89886-42-0	20119.42	13693.21	22169.28	9960.42	5651.41	3184.12	1.50007266	0.02069681	0.31868681	2.978402794	1.574538875	up	-	-
MW0123217	Tioxidazole	Organic acids	Organic acids	C12H14N2O3S	3	0.9152	61570-90-9	208.78	122.65	309.22	8.63	82.78	106.4	1.19202379	0.09294748	0.48592036	3.238713917	1.695421037	up	-	-
MEDL01792	Azukisaponin I	Terpenoids	Triterpene Saponin	C42H68O13	3	0.8974	82793-02-0	24847.01	28243.3	24968.83	17657.07	105.72	332.51	1.30350868	0.07033168	0.45866819	4.313779821	2.108952542	up	-	-
MEDN0204	Pyruvic acid	Organic acids	Organic acids	C3H4O3	3	0.8903	127-17-3	188614.04	636937.32	697731.29	210468.24	184189.44	202916.71	1.20494883	0.19426156	0.58775794	2.54910966	1.349993438	up	C00022	ko00010,ko00020,ko00030,ko00040,ko00053,ko00250,ko00260,ko00261,ko00270,ko00290,ko00330,ko00350,ko00360,ko00430,ko00440,ko00470,ko00620,ko00630,ko00650,ko00660,ko00710,ko00730,ko00760,ko00770,ko00785,ko00900,ko01100,ko01110,ko01200.ko01210.ko01230.ko012 [illegible]
MEDN1369	3-Hydroxyisovaleric [illegible]	Organic acids	Organic acids	C5H10O3	3	0.8679	625-08-1	135.22	174.51	212.49	99.27	26.3	89.98	1.3260311	0.03308533	0.35164075	2.422732545	1.276635149	up	-	-
MEDL02396	Sucrose	Others	Saccharides	C12H22O11	3	0.8655	57-50-1	941540.9	650414.21	673760	1785879.85	3812713.98	1744547.88	1.57403395	0.12885169	0.52010079	0.308548466	-1.69643097	down	C00089	ko00052,ko00500,ko01100,ko01110,ko02010
MW0015125	7,8-Dehydroastaxanthianthi [illegible]	Others	Ketone compounds	C40H50O4	3	0.8545	19866-02-5	27.91	64.41	44.88	122.57	227.7	55.68	1.26787489	0.21174997	0.60547084	0.337972657	-1.56502156	down	-	-
MEDL01917	cis-3-Hexenyl acetate	Lipids	Free Lipidstty acids	C8H14O2	3	0.8486	3681-71-8	11586.82	8153.85	9579.05	3745.78	3707.43	3880.76	1.71962932	0.02621804	0.33612848	2.586888795	1.371218037	up	C19757	ko00592,ko01110

MW0141403	[(1S,2R,3R,4S,5S,6S,8R,9S,10S,13S,16S,17R,18S)-11-ethyl-4,8,9-trihydroxy-6,16,18-trimethoxy-11-azahexacyclo[7.7.2.12,5.01,10.03,8.013,17]nonadecan-13-yl]methyl 2-[(3S)-3-methyl-2,5-dioxopyrrolidin-1-[illegible]	Benzene and substituted derivatives	Benzene and substituted derivatives	C36H48N2O10	3	0.8413	119347-24-9	3307.42	2661.37	3047.88	8829.38	8706.64	6064.64	1.69264932	0.0284631	0.33858973	0.382051604	-1.38816058	down	-	-
MW0123014	Azactam	Heterocyclic compounds	Heterocyclic compounds	C13H17N5O8S2	3	0.8387	78110-38-0	88.32	147.57	87.43	9.39	14.93	85.7	1.30063129	0.09063237	0.48325238	2.938738411	1.555196945	up	C06840	-
MW0103535	2',3'-Dideoxyinosine	Nucleotides and derivatives	Nucleotides and derivatives	C10H12N4O3	3	0.8353	69655-05-6	2099.8	539.54	2514.3	3495.34	6722.66	2484.86	1.17310852	0.17781959	0.57366954	0.405707061	-1.30148968	down	C06953	-
MW0157172	Spiradine A	Alkaloids	Terpenoid alkaloids	C20H25NO2	3	0.8292	19741-46-9	12288.2	7526.9	7968.42	6498.61	2286.85	2361.81	1.41280376	0.05498756	0.42471427	2.492405764	1.317538958	up	C08708	-
MW0108476	N-Phenylacetylphenylalani[illegible]	Amino acids and derivatives	Amino acids and derivatives	C17H17NO3	3	0.8063	738-75-0	3786.74	1903.14	4226.11	9852.05	41130.51	17754.58	1.55652345	0.17133423	0.5671048	0.144259566	-2.7932611	down	-	-
MEDP2093	4'-Hydroxyflavone	Flavonoids	Flavones	C15H10O3	3	0.7998	4143-63-9	659.34	664.56	11172.67	5688.09	15601.26	6334.03	1.09504045	0.34841814	0.6974448	0.452391054	-1.14435769	down	-	-
MW0009992	Triphenylphosphine oxide	Benzene and substituted derivatives	Benzene and substituted derivatives	C18H15OP	3	0.7996	791-28-6	1925.53	1501.93	1573.68	921.28	851.32	629.68	1.64763504	0.00782757	0.22054168	2.081830594	1.057852676	up	-	-
MW0138304	Glyzaglabrin	Heterocyclic compounds	Heterocyclic compounds	C16H10O6	3	0.7913	65242-64-0	680.09	3091.67	815.03	11354.2	1806.11	10436.2	1.27294342	0.16604657	0.56418482	0.194384254	-2.36301673	down	-	-
MEDL01843	Matairesinol	Lignans and Coumarins	Lignans	C20H22O6	3	0.7888	580-72-3	4257.63	4854.14	5586.84	3361.62	778.76	999.33	1.42515732	0.04349013	0.39461781	2.859813102	1.515920865	up	C10682	ko00999,ko01100,ko01110
MW0063587	Solanesyl-PP	Terpenoids	Terpene	C45H76O7P2	3	0.7875	146340-00-3	1458.9	4357.68	440.34	354.57	67.05	765.01	1.14954022	0.28506256	0.65528903	5.272848318	2.398582496	up	C17438	-
MEDL01892	3-Hydroxybenzoic acid	Benzene and substituted derivatives	Benzene and substituted derivatives	C7H6O3	3	0.7856	99-06-9	760.32	1441.51	872.91	1055.79	4822.96	1862.94	1.08998158	0.3061024	0.67088493	0.39716651	-1.33218412	down	C00587	ko00400
MW0142688	3,4-Dihydroxyphthalic acid	Benzene and substituted derivatives	Benzene and substituted derivatives	C8H6O6	3	0.785	82784-82-5	271.57	296.92	747.43	570.83	1058.17	1874.33	1.26596222	0.18600025	0.57833032	0.375619767	-1.41265511	down	C03223	ko01100
MW0121592	5-Methoxy-dl-tryptophan	Amino acids and derivatives	Amino acids and derivatives	C12H14N2O3	3	0.7809	28052-84-8	9113.73	7278.23	6962.53	3520.38	1782.84	2556.78	1.64496913	0.00450044	0.18300962	2.97130916	1.571098723	up	-	-
MEDL00626	Butyrate	Organic acids	Organic acids	C4H7O2-	3	0.7809	461-55-2	2761.22	4449.85	4100.79	9327.4	5986.54	12294.03	1.51571799	0.08693244	0.47762164	0.409731683	-1.28724864	down	-	-
MEDN1602	3'-Deoxyguanosine	Nucleotides and derivatives	Nucleotides and derivatives	C10H13N5O4	3	0.7801	3608-58-0	1081.41	16474.7	7380.44	27731.92	47453.59	29928.33	1.3256034	0.02965295	0.34767691	0.237233746	-2.07561885	down	-	-
MEDN0203	cis-Aconitic acid	Organic acids	Organic acids	C6H6O6	3	0.7768	585-84-2	13658.58	14285.4	15299.6	5211.41	4133.27	285.24	1.30537231	0.01133048	0.25665182	4.490544054	2.166890246	up	C00417	ko00020,ko00630,ko00660,ko01100,ko01110,ko0121[illegible]
MEDP0272	5-Hydroxyindoleacetic acid	Alkaloids	Plumerane	C10H9NO3	3	0.7722	54-16-0	23817.71	18801.79	30925.31	10992.72	15871.08	9217.29	1.50975697	0.05009095	0.40809391	2.038320073	1.027380613	up	C05635	ko00380,ko01100
MEDL01970	Corilagin	Tannins	Tannin	C27H22O18	3	0.7704	23094-69-1	109.86	31.84	53.98	19.64	37.23	16.85	1.27139805	0.21681725	0.60875495	2.654367878	1.408368333	up	C10219	-
MEDN0092	3,4-Dihydroxybenzeneacetic acid	Phenolic acids	Phenolic acids	C8H8O4	3	0.7702	102-32-9	478.55	2070.65	4341.99	3816.6	11434.99	10642.01	1.27573294	0.10340237	0.49490204	0.266134875	-1.90977052	down	C01161	ko00350,ko01100
MEDL02393	N-Methylnicotinamide	Alkaloids	Pyridine [illegible]	C7H8N2O	3	0.7701	114-33-0	827.22	1051.26	1043.63	2476.53	2517.36	1901.65	1.69501526	0.01333669	0.26897231	0.423768117	-1.23865305	down	-	-
MEDL00689	Phenylpyruvic acid	Organic acids	Organic acids	C9H8O3	3	0.7695	156-06-9	382.82	577.8	452.27	123.82	150.78	121.86	1.72206157	0.02469102	0.33384987	3.563764314	1.833401929	up	C00166	ko00360,ko00400,ko00470,ko00960,ko01100,ko01110,ko01210,ko01230
MEDL02036	Epigallocatechin gallate	Flavonoids	Flavanols	C22H18O11	3	0.7677	989-51-5	215.05	170.58	21.68	3.94	12.14	17.81	1.40656168	0.16645902	0.56418482	12.01858955	3.587195693	up	C09731	-
MEDP0291	3,7-Dimethyluric acid	Amino acids and derivatives	Amino acids and derivatives	C7H8N4O3	3	0.766	13087-49-5	955.86	1413.09	3776.66	627.93	1172.06	1055.68	1.00674287	0.33573668	0.68792647	2.152072894	1.105726945	up	C16360	ko00232
MW0145614	Aricine	Alkaloids	Plumerane	C22H26N2O4	3	0.7626	482-91-7	1905.98	974.38	1827.46	291.52	472.25	1034.13	1.36786503	0.06467248	0.44715824	2.618510484	1.388746381	up	C09037	-
MEDL01801	6-Hydroxyluteolin	Flavonoids	Flavones	C15H10O7	3	0.7612	2035-15-6	24.17	94.27	197.8	156.34	1236.88	162.81	1.07193412	0.36870512	0.70440483	0.203235156	-2.29877811	down	C10072	-
MW0146406	Atractylon	Terpenoids	Sesquiterpenoids	C15H20O	3	0.7597	6989-21-5	13224.15	12684.32	13232.1	6407.44	4039.91	3797.99	1.67779258	0.0076487	0.22054168	2.747605182	1.45817471	up	C16919	-
MEDL00926	Paraxanthine	Nucleotides and derivatives	Nucleotides and derivatives	C7H8N4O2	3	0.7561	611-59-6	12278.8	11649.51	12484.63	4789.63	6204.48	5240.03	1.72919506	0.00050935	0.10742261	2.242985462	1.165420269	up	C13747	ko00232,ko01100,ko01110
MW0146440	Auriculoside	Terpenoids	Steroid	C22H26O10	3	0.7555	75871-96-4	7644.16	3117.99	3432.17	5834.21	29233.5	42780.95	1.31053856	0.18618725	0.57833032	0.182332233	-2.45535847	down	C09478	-
MW0108427	N-(3-methoxypropyl)-2-piperazin-1-ylacetamide	Alcohol and amines	Amines	C10H21N3O2	3	0.7553	871217-92-4	23665	7924.11	18290.97	586.45	4106.4	329.99	1.51162734	0.0751076	0.46149681	9.930652778	3.311888554	up	-	-
MW0149306	Isocromadurine	Heterocyclic compounds	Heterocyclic compounds	C16H23NO5	3	0.7525	57495-69-9 | 6029-87-4	8.76	274.1	5.77	179.61	322.97	167.44	1.1601861	0.29738482	0.66331632	0.430778186	-1.2149829	down	C10304	-
MW0106097	(±)-Camphoric acid	Organic acids	Organic acids	C10H16O4	3	0.752	5394-83-2	2049.3	1702.66	3561.45	1026.88	589.15	1163.85	1.48562114	0.10757005	0.49971175	2.630836583	1.395521637	up	-	-
MW0055278	Nigakihemiacetal B	Terpenoids	Ditepenoids	C22H30O6	3	0.7512	76-77-7	5487.61	749.51	5027.11	8555.79	9467.76	8793.66	1.18768117	0.07086141	0.45866819	0.420037357	-1.25141045	down	C08771	-
MW0122906	Arborine	Alkaloids	Alkaloids	C16H14N2O	3	0.7508	6873-15-0	163.56	1819.06	360.84	111.44	209.15	156.66	1.07968716	0.3556113	0.70140421	4.910340492	2.295823067	up	C10642	-
MW0116587	1,2-Di(2-pyridyl)ethylene	Alkaloids	Pyridine alkaloids	C12H10N2	3	0.7506	1437-15-6 | 13341-40-7	5974.45	2509.09	21317.82	1265.94	945.01	1918.23	1.3777699	0.27668733	0.65233228	7.217258632	2.851450955	up	-	-
MW0147078	Caracurine V	Alkaloids	Alkaloids	C38H40N4O2	3	0.7495	630-87-5	11334.5	14473.67	9193.09	24867.42	107337.58	87464.28	1.53218924	0.13102396	0.52284849	0.159336162	-2.64985436	down	C09100	-
MEDP0247	Nicotinuric acid	Amino acids and derivatives	Amino acids and derivatives	C8H8N2O3	3	0.7473	583-08-4	10855.17	9711.67	15958.58	996.96	1729.06	948.05	1.72329276	0.02766498	0.33858973	9.94140558	3.313449844	up	C05380	ko00760
MW0160171	Avenacin A 1	Alkaloids	Terpenoid alkaloids	C55H83NO21	3	0.7432	90547-90-3	22066.02	61986.89	16336.79	13064.27	6073.34	11309.72	1.33006638	0.2443074	0.63271123	3.29715939	1.721223631	up	-	-

MW0139901	7-Hydroxycoumarinyl Arachidonate	Others	Others	C29H36O4	3	0.7418	161180-11-6	26399.06	25277.64	29836.21	1212.11	8678.17	10934.7	1.36642276	0.00982298	0.24036471	3.914189113	1.968713461	up	-	-
MW0150558	Glycerophosphoglycerol	Organic acids	Organic acids	C6H15O8P	3	0.7414	6418-92-4	33339.15	27329.38	31528.2	14131.28	18433.84	11411.18	1.6348571	0.00430037	0.18300962	2.096509484	1.067989356	up	C03274	-
MW0052910	Finasteride Carboxylic Acid	Organic acids	Organic acids	C23H34N2O4	3	0.7412	116285-37-1	717.52	425.54	2718.68	167.79	883.99	248.19	1.00595146	0.35886696	0.70355685	2.970637784	1.570772705	up	-	-
MW0004470	4,5-Diphenyl-1,2,3-thiadiazole	Benzene and substituted derivatives	Benzene and substituted derivatives	C14H10N2S	3	0.7407	5393-99-7	179.7	148.84	108.4	134.75	46.28	19.55	1.18714676	0.13959721	0.53158009	2.17838269	1.123257424	up	-	-
MEDN0442	Pantetheine	Alkaloids	Alkaloids	C11H22N2O4S	3	0.7384	496-65-1	22158.5	22786.18	27843.82	39567.84	62794.35	67075.91	1.61213367	0.0581445	0.4343505	0.42958756	-1.21897588	down	C00831	ko00770,ko01100,ko01110
MW0105439	Adouetine X	Amino acids and derivatives	Amino acids and derivatives	C28H44N4O4	3	0.7374	19542-37-1	6099.37	6097.83	5510.16	1895.72	2220.4	1444.73	1.7194069	0.00019117	0.06517318	3.184290171	1.670971809	up	C09993	-
MW0155528	phosphoribosyl-AMP	Nucleotides and derivatives	Nucleotides and derivatives	C15H23N5O14P2	3	0.737	1109-75-7	1706.71	2386.73	4647.49	634.97	2351.86	410.28	1.2142941	0.1832888	0.57807562	2.573048856	1.363478851	up	C02741	ko00340,ko01100,ko01110,ko01230
MW0130029	Cudraflavanone B	Flavonoids	Flavones	C20H20O6	3	0.7351	597542-74-0	1424.93	1573.14	1335.77	618.88	815.65	603.31	1.69537655	0.00141125	0.13924282	2.126683155	1.088605108	up	-	-
MW0006988	Alphazurine	Benzene and substituted derivatives	Benzene and substituted derivatives	C37H36N2O9S3	3	0.7321	68921-42-6	992.94	1754.48	282.28	3881.71	3948.71	3426.13	1.44545686	0.01390255	0.27083212	0.269149962	-1.89351788	down	-	-
MEDN0641	Ribose-1-phosphate	Others	Saccharides	C5H11O8P	3	0.7311	14075-00-4	958.45	980.19	772.29	283.34	257.61	476.22	1.63255744	0.00411695	0.18300962	2.665169047	1.414227044	up	C00620	ko00030,ko00230,ko01100
MEDP0150	2'-Deoxyinosine	Nucleotides and derivatives	Nucleotides and derivatives	C10H12N4O4	3	0.7284	890-38-0	6617.58	7356.69	43208.34	48672.01	115889.18	63061.65	1.39303076	0.08990516	0.48239668	0.251216486	-1.99299695	down	C05512	ko00230,ko01100,ko01232,ko02010
MW0054489	1-(9Z,12Z-octadecadienoyl)-glycero-3-phosphate	GL	PA	C21H39O7P	3	0.7234	65528-85-0	2052.92	1965.34	2244.39	9085.76	1896.12	4208.51	1.06940995	0.2952875	0.66163509	0.412277104	-1.27831375	down	-	-
MW0106111	Carbenicillin	Benzene and substituted derivatives	Benzene and substituted derivatives	C17H18N2O6S	3	0.7231	4697-36-3	418.25	329.52	2960.16	13046.82	11755.09	8890.78	1.56193761	0.00385393	0.18170858	0.110051468	-3.1837497	down	C06869	-
MEDN1197	8-Methoxyalkynurate	Organic acids	Organic acids	C11H9NO4	3	0.718	2929-14-8	1413.22	1115.57	1020.63	577.93	646.5	421.26	1.62514302	0.01652563	0.29532899	2.156797453	1.108890698	up	C05830	ko00380
MW0003096	2-Hydroxy-3,4-dimethoxybenzoic acid	Benzene and substituted derivatives	Benzene and substituted derivatives	C9H10O5	3	0.7128	5653-46-3	180.94	384.95	380.3	92.67	17.02	231.62	1.17131277	0.093924	0.48592036	2.772230524	1.471047229	up	-	-
MW0148692	D-Ribitol-5-phosphate	Organic acids	Organic acids	C5H13O8P	3	0.7114	35320-17-3 | 3506-18-1 | 51584-96-4	304855.34	158793.05	178292.91	105739.78	95480.72	119509.99	1.4777089	0.14134587	0.53210743	2.001497581	1.001079872	up	C01068	ko00040,ko01100
MEDN1160	Mevalonate 5-phosphate	Organic acids	Organic acids	C6H13O7P	3	0.7105	73566-35-5	16915.38	11388.62	25046.48	7029.58	10833.91	6995.27	1.41826007	0.12848622	0.51982213	2.146144055	1.101746917	up	C01107	ko00900,ko01100,ko01110
MW0156059	Proacacipetalin	Others	Saccharides	C11H17NO6	3	0.7101	66871-89-4	52484.4	76839.67	67349.12	26862.19	26166.75	25528.59	1.7109151	0.03059509	0.34952497	2.503556184	1.323978832	up	C08338	-
MEDL02353	Rosmarinic acid	Phenolic acids	Phenolic acids	C18H16O8	3	0.7057	20283-92-5	28465.75	13337.69	8247.61	9916.35	6805.87	4008.02	1.18533604	0.24462666	0.63271123	2.414398	1.271663516	up	C01850	ko00350,ko01100
MW0146965	CalaLipidstimine	Lignans and Coumarins	Lignans	C38H40N2O7	3	0.7051	77793-42-1	463.57	1212.02	889.37	990.07	7595.17	3158.92	1.2325581	0.25523731	0.64155925	0.218403019	-2.1949353	down	C09369	-
MEDL02031	Gallic acid	Phenolic acids	Phenolic acids	C7H6O5	3	0.7036	149-91-7	4848.55	3359.2	4054.51	7390.15	14538.35	15278.86	1.5887386	0.07662336	0.46149681	0.329565441	-1.60136313	down	C01424	ko00999
MEDN0417	Mevalonate	Organic acids	Organic acids	C6H12O4	3	0.7036	150-97-0	153.83	131.85	307.31	47.34	92.73	78.69	1.4543427	0.14569534	0.53579335	2.710687511	1.438658809	up	C00418	ko00900,ko01100,ko01110
MW0110034	Trimethyl glycine	Alcohol and	Amines	C5H12NO2+	3	0.7022	6915-17-9	201.59	150.31	53.03	4.38	33.59	16.03	1.46803451	0.11023534	0.50173323	7.498703704	2.906641219	up	-	-
FDATN00564	Nordihydroguaiaretic acid	Benzene and substituted derivatives	Benzene and substituted derivatives	C18H22O4	3	0.7021	500-38-9 | 27686-84-6	1322.58	1450.55	871.6	30.31	842.8	34.23	1.32648321	0.05645023	0.42642523	4.016939626	2.006096778	up	-	-
MEDL00375	OgluLipidsnide	Amino acids and derivatives	Amino acids and derivatives	C16H19N3O5	3	0.6996	38101-59-6	1763.7	433.85	4400.32	5157.07	7388.23	3363.28	1.14565271	0.13267441	0.52284849	0.414736576	-1.26973281	down	-	-
MW0052593	Equilin sulLipidste	Others	Lactones	C18H20O5S	3	0.6993	27540-07-4	245.25	230.93	82.88	76.27	89.48	26	1.26944119	0.12990381	0.52149789	2.915567145	1.543776548	up	-	-
MW0106579	Edetic acid	Organic acids	Organic acids	C10H16N2O8	3	0.6974	62-33-9	1098.13	1070.8	846.7	351.26	343.2	263.66	1.72143696	0.00751884	0.22054168	3.147444996	1.654181166	up	C00284	-
MW0107770	Leu-Val-Leu	Amino acids and derivatives	Amino acids and derivatives	C17H33N3O4	3	0.6968	58337-01-2	195.31	63.86	203.44	43.15	73.35	5.46	1.20831003	0.11346985	0.50205214	3.793128895	1.923388397	up	-	-
MW0144280	Acetylspiramycin	Heterocyclic compounds	Heterocyclic compounds	C45H76N2O15	3	0.696	24916-51-6	2691.94	3578.97	7532.04	1917.93	990.35	3014.09	1.21513679	0.21246787	0.60587521	2.330646346	1.220730105	up	-	-
MW0148256	Demethylalangiside	Alkaloids	Alkaloids	C24H29NO10	3	0.6949	47763-23-5	14050.01	10409.89	6821.4	20944.62	35646.9	51359.34	1.54433111	0.09340017	0.48592036	0.289773514	-1.78700236	down	C11813	ko00950
MW0118226	2-Amino-4,6-diphenylpyridine-3-carbonitrile	Heterocyclic compounds	Heterocyclic compounds	C18H13N3	3	0.6942	4604-06-2	113.15	829.45	1301.93	2091.52	2922.75	1644.1	1.21308209	0.04505493	0.39546247	0.33709902	-1.56875566	down	-	-
MW0169507	N-1-Naphthylbenzamide	Benzene and substituted derivatives	Benzene and substituted derivatives	C17H13NO	3	0.693	634-42-4	25347.33	24286.44	23488.87	8232.29	11050.73	11370.12	1.71279835	0.00097874	0.12883555	2.38548612	1.254283292	up	-	-
MW0106789	gamma-Glutamyltyrosine	Amino acids and derivatives	Amino acids and derivatives	C14H18N2O6	3	0.6923	7432-23-7	18.9	13.9	47.6	505.73	63.24	149.26	1.43385868	0.25663625	0.64181203	0.111941857	-3.15917851	down	-	-
MEDN0260	3-Indolebutyric acid	Alkaloids	Plumerane	C12H13NO2	3	0.6916	133-32-4	88.31	17.32	70.77	17.61	21.77	5.27	1.23124471	0.17047677	0.56680649	3.950727884	1.98211848	up	C11284	-
MEDN0317	Hippuric acid	Organic acids	Organic acids	C9H9NO3	3	0.6913	495-69-2	2760.52	2769.21	17687.84	13586.1	35920.01	16582.3	1.24112253	0.17904863	0.57366954	0.351310767	-1.5091803	down	C01586	-
MW0103525	Deoxyuridine-5'-diphosphate	Nucleotides and derivatives	Nucleotides and derivatives	C9H14N2O11P2	3	0.6907	4208-67-7	6018.97	3951.35	1526.81	10310.66	11367.57	7676.09	1.38440903	0.02598982	0.33612848	0.391667393	-1.35229907	down	C01346	ko00240,ko01100,ko01232
MEDN0074	gamma-Glutamylcysteine	Amino acids and derivatives	Amino acids and derivatives	C8H14N2O5S	3	0.6906	636-58-8	68848.96	47080.26	54664.12	26111.7	21182.21	21885	1.69148049	0.02821426	0.33858973	2.465973228	1.302157137	up	C00669	ko00480,ko01100,ko01240
MW0111613	1-(3,4-Dimethoxyphenyl)-1,2-ethanediol 1-O-b-D-glucoside	Alcohol and amines	Alcohols	C16H24O9	3	0.6895	251905-98-3	5646.61	23194.87	1396.73	13770.8	19257.64	28326.74	1.04505168	0.2713412	0.64977045	0.492838746	-1.02081241	down	-	-
MW0155474	Phenisopham	Benzene and substituted derivatives	Benzene and substituted derivatives	C19H22N2O4	3	0.6889	57375-63-0	1689.69	1475.38	3540.18	295.49	117.08	1597.87	1.27123988	0.13125125	0.52284849	3.335215177	1.737779842	up	C19061	-

MW0010818	(10E,12Z)-9-HODE	Lipids	Free Lipidstty acids	C18H32O3	3	0.687	98524-19-7	5276.89	3700.78	7359.99	4117.54	2078.67	1158.05	1.2848576	0.09724781	0.48888534	2.221523308	1.151549278	up	-	-
MW0153811	Methyl Lipidsrnesoate	Lipids	Others	C16H26O2	3	0.6834	10485-70-8 | 3675-00-1 | 4176-79-8	70.97	97.05	13.91	9.52	8.77	11.05	1.38682727	0.17397387	0.57211065	6.20074983	2.632442685	up	-	-
ZINC2169825[illegible]	5-[(3aR,4R,6aS)-2-oxo-1,3,3a,4,6,6a-hexahydrothieno[3,4-d]imidazol-4-yl]pentanoic acid	Organic acids	Organic acids	C10H16N2O3S	3	0.6823	21788-37-4	319.96	504.88	418.81	56.8	30.2	27.32	1.72149582	0.01715859	0.29823307	10.8786739	3.443430799	up	-	-
MEDN0036	L-Alanyl-L-alanine	Amino acids and derivatives	Amino acids and derivatives	C6H12N2O3	3	0.6818	1948-31-8	1055.12	790.92	910.21	97.59	712.29	291.71	1.26921441	0.07684672	0.46149681	2.502065197	1.323119383	up	-	-
MW0006086	Aspulvinone E	Phenolic acids	Phenolic acids	C17H12O5	3	0.6814	49637-60-7	381.54	938.14	1299.18	6952.22	20187.12	12919.86	1.66215481	0.08184	0.47136621	0.065374745	-3.93512277	down	C02006	-
MW0123019	Bamnidazole	Heterocyclic compounds	Heterocyclic compounds	C7H10N4O4	3	0.6801	31478-45-2	423.97	1746.88	563.94	3743.93	2727.29	3402.88	1.50712245	0.01254407	0.26101095	0.276966002	-1.8522192	down	-	-
MW0157939	Tinyatoxin	Terpenoids	Ditepenoids	C36H38O8	3	0.68	58821-95-7	45.98	29.99	143.83	121.19	82.69	274.12	1.05404829	0.29009941	0.66011135	0.459832636	-1.12081923	down	-	-
MW0113931	Amygdalin	Alkaloids	Alkaloids	C20H27NO11	3	0.6778	29883-15-6	137.82	2387.82	1662.8	6948.55	7359.18	1923.08	1.14982669	0.13618723	0.52760645	0.258054897	-1.95425009	down	C08325	ko00460,ko01100,ko01110
MEDL01875	[10]-Shogaol	Phenolic acids	Phenolic acids	C21H32O3	3	0.6753	36752-54-2	138.1	152.45	336.42	253.28	277.04	1043.12	1.03153894	0.34773335	0.6972136	0.398470866	-1.32745385	down	-	-
MW0138808	Lonchocarpol A	Flavonoids	Flavanones	C25H28O5	3	0.6743	68236-11-3	717.64	372.87	1260.13	2796.92	2771.49	1723.07	1.49172823	0.02319496	0.33077008	0.322381739	-1.63315807	down	C09724	-
MEDL01793	Jubanine A	Alkaloids	Pyrrole alkaloids	C40H49N5O6	3	0.674	60375-07-7	17.27	284.24	271.8	8.12	44.19	42.35	1.0052306	0.20630159	0.60099477	6.056518065	2.598488616	up	-	-
MEDL02714	3-Phosphoglyceric acid	Others	Saccharides	C3H7O7P	3	0.6731	3443-58-1	2712.01	2727.89	27106.84	38248.53	73076.81	51855.96	1.42944408	0.03058713	0.34952497	0.199451408	-2.32589079	down	C00197	ko00010,ko00030,ko00260,ko00270,ko00561,ko00630,ko00710,ko01100,ko01110,ko01200,ko01230,ko01240
MW0169215	Geniposide	Terpenoids	Monoterpenoids	C17H24O10	3	0.6713	24512-63-8 | 169799-41-1 | [illegible]	30126.53	73047.28	26524.02	16244.32	29943.39	7399.15	1.18118262	0.22616112	0.61697947	2.420328976	1.275203155	up	C09781	-
MW0112859	3-Methyl-1-(2,4,6-trihydroxyphenyl)-1-butanone	Others	Ketone compounds	C11H14O4	3	0.6682	26103-97-9	2.34	2.01	195.01	390.98	338.97	582.32	1.37587015	0.02004545	0.31365607	0.151919955	-2.71861671	down	C07350	-
MW0147810	Cotylenin F	Heterocyclic compounds	Heterocyclic compounds	C33H54O11	3	0.6663	58045-03-7	154.93	77	110.16	31.93	39.48	56.08	1.55157549	0.07549384	0.46149681	2.683269276	1.423991842	up	C09078	-
MW0148881	Eleganin	Heterocyclic compounds	Heterocyclic compounds	C22H26O9	3	0.6651	57498-84-7	5861.93	21172.11	23834.92	35497.25	50395.6	25732.66	1.19908023	0.0939792	0.48592036	0.455710885	-1.13380926	down	C09401	-
MW0113884	Aloeresin A	Flavonoids	Other Flavonoids	C28H28O11	3	0.6638	74545-79-2	1807.07	2164.22	1386.02	360.87	94.85	196.6	1.65462615	0.01265524	0.26209353	8.212702355	3.037857013	up	C17781	-
MW0143257	Paptga	Benzene and substituted derivatives	Benzene and substituted derivatives	C12H15NO6S	3	0.6635	62205-44-1	11933.96	964.66	10791.5	19629.79	127632.31	40663.26	1.32396114	0.23864193	0.6289157	0.126061326	-2.98780236	down	-	-
MW0152081	Lacto-N-triaose	Others	Saccharides	C20H35NO16	3	0.6601	75645-27-1	3632.47	915.8	6846	6514.9	18709.17	7844.63	1.19510903	0.19377066	0.58775794	0.34456359	-1.53715783	down	-	-
MEDP1669	Alanylphenylalanine	Amino acids and derivatives	Amino acids and derivatives	C12H16N2O3	3	0.6597	3061-90-3	7946.81	6419.65	10362.19	1549.74	1648.19	1959	1.72707622	0.02840017	0.33858973	4.795227005	2.261599114	up	-	-
MW0007535	Maclurin	Flavonoids	Other Flavonoids	C13H10O6	3	0.6583	519-34-6	78.95	335.2	1071.75	3281.52	19289.49	6565.47	1.52734878	0.19922127	0.59377853	0.050997924	-4.29341766	down	C09951	-
MW0154318	N-Benzoyl-D-arginine	Benzene and substituted derivatives	Benzene and substituted derivatives	C13H18N4O3	3	0.6569	61088-55-9	223.68	4416.47	382.18	5878.67	12236.87	8626.33	1.39644881	0.03836016	0.37864641	0.187807734	-2.41267162	down	C03001	-
MEDTP0167[illegible]	cis-5-Dodecenoic acid	Lipids	Free Lipidstty acids	C12H22O2	3	0.6563	2430-94-6	1657.6	2013.25	2649.91	1486.26	607.21	115.57	1.22794705	0.05590786	0.42551183	2.861315322	1.516678494	up	-	-
MW0148386	Docosahexaenoic acid	Lipids	Free Lipidstty acids	C22H32O2	3	0.6561	6217-54-5	245.24	190.43	110.8	63.02	149.05	47.74	1.23234913	0.13272566	0.52284849	2.103344752	1.072685337	up	C06429	ko01040
MW0000053	1,2,3,4-Tetrahydro-b-carboline-1,3-dicarboxylic acid	Alkaloids	Alkaloids	C13H12N2O4	3	0.6539	59132-30-8	13531.5	14702.84	13019.8	7674.65	7263.79	4520.8	1.61066674	0.00758598	0.22054168	2.120028326	1.084083541	up	-	-
MEDN1244	4-(Cytidine 5'-diphosphate) -2-C-methyl-D-erythritol	Nucleotides and derivatives	Nucleotides and derivatives	C14H25N3O14P2	3	0.6517	263016-94-0	16784.7	34702.68	40780.8	9601.75	18936.46	11761.6	1.34839157	0.12464256	0.51634495	2.289543797	1.195060163	up	C11435	ko00900,ko01100,ko01110
MEDL02107	Desaminotyrosine	Phenolic acids	Phenolic acids	C9H10O3	3	0.6511	501-97-3	123.19	244.47	132.81	59.32	29.46	128.26	1.25224102	0.12980372	0.52149789	2.305888316	1.205322639	up	C01744	ko01100
MW0011996	10E,12Z-Octadecadienoic acid	Lipids	Free Lipidstty acids	C18H32O2	3	0.6494	2420-56-6	1883.63	1136.54	3062.75	1038.7	811.66	417.74	1.36693625	0.14141012	0.53210743	2.681945241	1.423279781	up	-	-
pme0321	Kaempferol-7-[illegible]	Flavonoids	Flavonols	C21H20O10	3	0.6488	20196-89-8	1033.08	1508.3	967.6	255.17	299.25	117.25	1.64935517	0.02246055	0.33066403	5.224261914	2.385227227	up	-	-
MW0123298	Chelidonic acid	Organic acids	Organic acids	C7H4O6	3	0.646	99-32-1	19811.18	4848.29	6953.22	22599.17	32536.31	38002.24	1.43161511	0.03434104	0.3606623	0.339418766	-1.55886176	down	C08476	-
MW0127152	2-(1-Methyl-1H-tetrazol-5-ylsulLipidsnyl)-ethylamine	Heterocyclic compounds	Heterocyclic compounds	C4H9N5S	3	0.646	774191-08-1	987.06	543.32	589.05	54.17	650.81	137.3	1.19837048	0.14808414	0.53947123	2.516300993	1.331304503	up	-	-
MW0109829	Thapsigargin	Heterocyclic compounds	Heterocyclic compounds	C34H50O12	3	0.6446	67526-95-8 | 123269-03-4	2026.72	2134.09	2599.06	2394.27	3194.38	11030.35	1.0160833	0.35517668	0.70127119	0.406755521	-1.29776617	down	C09561	-
MW0153825	Methyl-2-alpha-L-fucopyranosyl-beta-D-galactoside	Others	Saccharides	C13H24O10	3	0.642	37288-45-2	54440.29	98611.01	93600.03	58	207.38	49008.49	1.30281282	0.03859439	0.37926907	5.005722709	2.323578375	up	C04698	-
MEDP0370	Formylanthranilic acid	Phenolic acids	Phenolic acids	C8H7NO3	3	0.6411	3342-77-6	1117.21	1680.09	2217.2	4036.84	5796.34	1836.24	1.23753855	0.18591356	0.57833032	0.429712873	-1.2185551	down	C05653	ko00380,ko01100
MEDL02157	Coniferin	Phenolic acids	Phenolic acids	C16H22O8	3	0.6407	124151-33-3	123984.89	38831.89	139077.23	128422.76	153837.28	564396.92	1.064915	0.32654558	0.68070395	0.356571816	-1.48773542	down	C00761	ko00940,ko01110
MW0146553	Beiwutine	Benzene and substituted derivatives	Benzene and substituted derivatives	C33H45NO12	3	0.639	76918-93-9	1658.25	7431.53	5479.38	596.7	3129.37	5.35	1.05619015	0.1559671	0.5503776	3.904454604	1.965121039	up	C08663	-
MW0014757	5-Geranyloxy-7-methoxycoumarin	Lignans and Coumarins	Coumarins	C20H24O4	3	0.6382	7380-39-4	473.64	779.91	760.43	203.34	543.36	219.89	1.33118778	0.07923218	0.46796065	2.083592837	1.059073382	up	-	-

MW0127096	Tribufos	Others	Lactones	C12H27OPS3	3	0.636	78-48-8	15944.01	14350.58	8668.18	24730.68	31219.65	29301.9	1.57995598	0.0065492	0.21133131	0.457029335	-1.12964132	down	-	-
MW0104734	2-Deoxyribonic acid	Organic acids	Organic acids	C5H10O5	3	0.6359	29625-75-0	28005.04	28688.5	29797.61	10689.32	10742.04	8210.36	1.73701013	0.00014927	0.06517318	2.917885669	1.544923356	up	-	-
MADN0561	N,N',N''-triacetylchitotriose	Others	Saccharides	C24H41N3O16	3	0.635	38864-21-0	27653.13	42773.58	57085.28	15493.01	16187.77	23191.61	1.51103987	0.09440535	0.4865169	2.323791437	1.216480591	up	-	-
MW0157928	Tiapamil	Benzene and substituted derivatives	Benzene and substituted derivatives	C26H37NO8S2	3	0.6336	57010-31-8	815.61	932.14	347.37	383.61	304.58	88.19	1.23232678	0.1169879	0.50205214	2.69857544	1.432198019	up	-	-
MEDL01997	Prunasin	Alkaloids	Alkaloids	C14H17NO6	3	0.6297	99-18-3	4610.8	4160.15	6285.83	3564.16	852.14	2149.43	1.30975535	0.05147648	0.41145636	2.293237766	1.197385944	up	C00844	ko00460,ko01100,ko01110
MW0146370	Asp-Trp	Amino acids and derivatives	Amino acids and derivatives	C15H17N3O5	3	0.6286	71835-79-5	683.18	5646.63	8236.62	16077.72	30252.97	12666.79	1.28563982	0.09513828	0.48768821	0.24689919	-2.01800599	down	-	-
MW0127814	Isovitexin 2''-O-arabinoside	Alcohol and amines	Alcohols	C26H28O14	3	0.6285	53382-71-1	1036.35	7751.87	3468.8	12331.39	24694.01	11951.22	1.38710357	0.08176654	0.47136621	0.250262676	-1.99848495	down	-	-
MW0157233	Streptobiosamine	Alcohol and [illegible]	Amines	C13H23NO9	3	0.6259	126-05-6	1115.71	2479.79	2917.01	151.5	1473.53	428.95	1.26237751	0.09832516	0.48888534	3.17067839	1.664791549	up	C07655	-
MW0154029	Myricoside	Benzene and substituted derivatives	Benzene and substituted derivatives	C34H44O19	3	0.623	76076-04-5	76.79	1179.64	933.25	1158.87	14781.85	4698.81	1.24185457	0.27037323	0.64977045	0.106091563	-3.23661816	down	C10479	-
MW0012592	17R-hydroxy-4Z,7Z,10Z,13Z,15E,19Z-docosahexaenoic acid	Lipids	Free Lipidstty acids	C22H32O3	3	0.6216	155976-53-7	681.11	321.34	670.99	190.68	183.42	67.78	1.50145417	0.06161367	0.44168098	3.787091518	1.921090286	up	-	-
MEDN1233	4-Hydroxyphenylacetylglycine	Amino acids and derivatives	Amino acids and derivatives	C10H11NO4	3	0.62	28116-23-6	571.6	1073.94	121.37	5235.99	13042.27	13480.42	1.60144971	0.06340905	0.44396349	0.055635499	-4.16785049	down	C05596	ko00350
MW0127213	Isopropyl methyl sulfide	Alcohol and [illegible]	Alcohols	C4H10S	3	0.6194	1551-21-9	2062.02	3199.24	2078.26	318.43	638.24	801.22	1.62316827	0.02679481	0.33655369	4.175187298	2.061840918	up	-	-
MEDN1375	3-Phenylbutyric acid	Organic acids	Organic acids	C10H12O2	3	0.6146	4593-90-2	2066.67	925.93	1605.46	537.97	905.39	691.36	1.39806683	0.12065814	0.50687854	2.153940564	1.10697844	up	-	-
MW0151793	Isobutrin	Heterocyclic compounds	Heterocyclic compounds	C27H32O15	3	0.6145	536-01-6	154.81	508.21	930.36	245.34	161.23	60.22	1.14355112	0.23206541	0.62319286	3.413483579	1.771244812	up	C08649	-
MEDP1614	Isopentenyl [illegible]	Organic acids	Organic acids	C5H12O7P2	3	0.6101	358-71-4	945.89	1743.84	1436.26	834.49	907.84	231.5	1.18555733	0.08619102	0.47762164	2.090347193	1.063742584	up	C00129	ko00900,ko01100,ko01110
MW0105527	L-Alanyl-L-methionine	Amino acids and derivatives	Amino acids and derivatives	C8H16N2O3S	3	0.6095	14486-05-6	616.2	186.73	915.45	92.17	124.06	239.7	1.29915447	0.18038322	0.57412883	3.768955761	1.914164861	up	-	-
MW0159385	Volkenin	Heterocyclic compounds	Heterocyclic compounds	C12H17NO7	3	0.6084	66575-40-4	2399.41	456.95	449.48	28.32	39.8	400.69	1.33500656	0.28057364	0.65528903	7.051556067	2.817941652	up	C08344	-
MW0014781	5-HPETE	Organic acids	Organic acids	C20H32O4	3	0.608	74581-83-2	2471.36	3439.43	1934.51	500.37	747.03	439.93	1.68740034	0.03804146	0.3763387	4.649535064	2.217086459	up	C05356	ko00590,ko01100
MW0145611	Arg-Tyr	Amino acids and derivatives	Amino acids and derivatives	C15H23N5O4	3	0.6069	74863-12-0	139.11	167	59.79	3.27	16.09	16.27	1.57392383	0.07308297	0.46133776	10.26943587	3.360285027	up	-	-
MW0157956	Toonacilin	Terpenoids	Sesquiterpenoids	C31H38O9	3	0.6068	66610-71-7	7408.44	8092.28	14472.95	5581.84	3145.29	3905.86	1.48780166	0.11295371	0.50205214	2.372650497	1.2464996	up	C08785	-
MW0149199	(4aS,6aR,6aS,6bR,8aR,10S,12aR,14bS)-10-[(2S,3S,5R)-5-hydroxy-4-oxo-3-[(2S,3R,4S,5S,6R)-3,4,5-trihydroxy-6-(hydroxymethyl)oxan-2-yl]oxyoxan-2-yl]oxy-2,2,6a,6b,9,9,12a-heptamethyl-1,3,4,5,6,6a,7,8,8a,10,11,12,13,14b-tetradecahydropicene-4a-carboxylic acid	Heterocyclic compounds	Heterocyclic compounds	C41H64O12	3	0.6048	117585-05-4	2.75	7.36	3.41	29.13	30.38	14.49	1.61057726	0.04951512	0.40584796	0.182702703	-2.45243012	down	C08943	-
MW0133966	5,6,7-Trimethoxycoumarin	Lignans and Coumarins	Coumarins	C12H12O5	3	0.6032	55085-47-7	17102.79	10980.92	23081.79	334.01	9413.19	6860.44	1.11225243	0.06333299	0.44396349	3.080841107	1.623324278	up	C09313	-
MW0002404	2,6-Dimethoxyphenol	Phenolic acids	Phenolic acids	C8H10O3	3	0.6017	91-10-1	883.62	659.81	1177.79	687.71	167.38	127.35	1.33709013	0.07106667	0.45866819	2.769858719	1.469812391	up	C10787	-
MW0123525	Dimethyl 2,6-dimethyl-4-(2-nitrophenyl)-3,5-pyridinedicarboxylate	Heterocyclic compounds	Heterocyclic compounds	C17H16N2O6	3	0.6007	67035-22-7	3439.53	787.4	1975.2	1132.3	628.38	1002.02	1.02523899	0.27159451	0.64977045	2.244952402	1.166684857	up	-	-
MEDP1314	Monoisobutyl phthalate	Others	Lactones	C12H14O4	3	0.5998	30833-53-5	1045.09	747.68	668.4	47.71	74.77	419.67	1.4479509	0.01823212	0.30148847	4.539647699	2.182580341	up	-	-
MADN0605	DL-threo-3-isopropylmalic acid	Organic acids	Organic acids	C7H12O5	3	0.5925	113473-21-5	880.69	561.51	716.8	384.89	219.51	302.01	1.58928956	0.0275638	0.33858973	2.381924295	1.25212756	up	-	-
MW0122950	Asticolorin C	Heterocyclic compounds	Heterocyclic compounds	C33H28O9	3	0.5915	93395-44-9	94.3	268.52	261.42	1029.67	1045.21	252.89	1.28061591	0.15684033	0.5507933	0.268170824	-1.89877581	down	-	-
MW0156999	Methyl 10-acetyloxy-3,4-dihydroxy-1,16-dimethyl-11,19-dioxo-6,12,20-trioxahexacyclo[13.7.0.02,8.05,9.08,13.017,21]docosa-17,21-diene-5-carboxylate	Terpenoids	Triterpene	C25H28O11	3	0.5897	82290-17-3	397.41	935.7	522.35	71.36	30.42	97.97	1.64850643	0.07491954	0.46149681	9.288911139	3.215509491	up	-	-
MW0007500	Levosimendan	Benzene and substituted derivatives	Benzene and substituted derivatives	C14H12N6O	3	0.587	141505-33-1	2679.89	2221.08	2063.09	911.53	770.74	880.99	1.73055429	0.01216085	0.25665182	2.716876166	1.441948809	up	-	-
MW0109127	Phenylalanyl-phenylalanyl-[illegible]	Amino acids and derivatives	Amino acids and derivatives	C27H29N3O4	3	0.5803	2578-81-6	89.03	234.89	232.01	33.12	65.58	71.17	1.44583515	0.10863851	0.4998897	3.272679108	1.71047215	up	-	-
MW0125248	Myristicin	Lignans and Coumarins	Lignans	C11H12O3	3	0.5726	607-91-0	74.74	59.48	74.9	668.88	755.98	45.58	1.074232	0.20088439	0.5961949	0.142215935	-2.81384497	down	C10480	-

MEDP0617	N-(2-Furoyl)glycine	Amino acids and derivatives	Amino acids and derivatives	C7H7NO4	3	0.5693	5657-19-2	154.56	81.75	112.69	431.07	864.57	349.35	1.61508065	0.11158131	0.50205214	0.212159344	-2.23677987	down	-	-
MEDN1163	3-Hydroxyhexadecanoic acid	Lipids	Free Lipidstty acids	C16H32O3	3	0.5649	2398-34-7 | 928-17-6 | 20595-04-4	265.22	81.4	2449.66	63.57	107.04	96.92	1.03118318	0.38320812	0.714505	10.45221097	3.385736244	up	-	-
pme0170	N-Acetyl-L-arginine	Amino acids and derivatives	Amino acids and derivatives	C8H16N4O3	3	0.5637	155-84-0	400.22	277.02	235.85	184.79	114.45	109.87	1.52994945	0.05746268	0.4309215	2.231893623	1.158268267	up	-	-
MW0010434	Acanthoside B	Lignans and Coumarins	Lignans	C28H36O13	3	0.5637	7374-79-0	231.97	306.35	1349.83	2178.02	3040.35	974.5	1.29809624	0.12465909	0.51634495	0.304890947	-1.71363478	down	-	-
MW0154656	2-Trimethylaminoethylphosphonic acid	Others	Lactones	C5H15NO3P+	3	0.5516	5148-60-7	35.89	82.76	207.91	603.73	544.38	202.93	1.38185556	0.09630982	0.48888534	0.24171009	-2.0486504	down	C06459	ko00440,ko01100
MW0124076	Eugenitol	Heterocyclic compounds	Heterocyclic compounds	C11H10O4	3	0.5483	491-48-5	2762.97	2674.83	1717.93	1486.15	434.42	38.66	1.21818904	0.03683316	0.37270445	3.652317492	1.868812183	up	-	-
MEDN0834	O-Succinyl-L-Homoserine	Amino acids and derivatives	Amino acids and derivatives	C8H13NO6	3	0.5413	1492-23-5	163035.49	251614.73	1571808.39	1642164.07	5974112.66	2556207.79	1.37687212	0.16473036	0.56203613	0.195277624	-2.35640145	down	C01118	ko00270,ko00920,ko01100,ko01110,ko01230
MW0000095	6-Acetylmorphine	Alkaloids	Isoquinoline alkaloids	C19H21NO4	3	0.5371	2784-73-8	1096.09	808.21	584.12	40.17	23.02	52.78	1.72785924	0.03298514	0.35164075	21.45744589	4.423406455	up	-	-
MEDL00817	Cysteinylglycine	Amino acids and derivatives	Amino acids and derivatives	C5H10N2O3S	3	0.5258	19246-18-5	3027.43	2348.32	4807.53	3145.74	1256.24	684.3	1.1487742	0.17912645	0.57366954	2.002107631	1.001519534	up	C01419	ko00480,ko01100
MW0111379	3-O-beta-D-Glucopyranosylandrographolide	Benzene and substituted derivatives	Benzene and substituted derivatives	C26H40O10	3	0.5244	869384-84-9	14148.13	15187.08	20331.86	36588.67	17476.58	54618.55	1.23506912	0.20507414	0.60017369	0.456986874	-1.12977537	down	-	-
MW0147362	Chaulmoogric acid	Lipids	Free Lipidstty acids	C18H32O2	3	0.522	29106-32-9	65.12	15.9	128.12	81.37	144.53	285.62	1.0629559	0.2359122	0.62573958	0.408859869	-1.29032163	down	C08282	-
MW0155498	Phe-Phe	Amino acids and derivatives	Amino acids and derivatives	C18H20N2O3	3	0.5197	2577-40-4	593.12	768.7	249.6	238.82	226	191.67	1.33108054	0.1709969	0.5671048	2.454599461	1.295487626	up	-	-
MW0012826	1-Hexanol	Alcohol and [illegible]	Alcohols	C6H14O	3	0.5167	111-27-3	35913.09	22391.39	47962.26	58193.53	152032.54	103927.87	1.46098422	0.11623528	0.50205214	0.338263273	-1.56378155	down	C00854	-
MW0114251	Dide-O-methyl-4-O-alpha-D-glucopyranosylsimmond[illegible]	Others	Saccharides	C20H31NO14	3	0.5129	370068-00-1	342.41	253.36	424.27	701.28	688.33	777.37	1.63725285	0.00567084	0.20125311	0.470719619	-1.08706011	down	-	-
MW0111798	1-deoxy-D-xylulose 5-phosphate	Others	Saccharides	C5H11O7P	3	0.5011	190079-18-6	10.36	12.74	16.7	137.82	19.54	81.54	1.3869037	0.1911625	0.58370725	0.166596902	-2.58556652	down	C11437	ko00270,ko00730,ko00900,ko01100,ko01110,ko0124[illegible]
MW0149981	Glu-Gln-Phe-Arg	Amino acids and derivatives	Amino acids and derivatives	C25H38N8O8	2	0.8965	-	108.27	9.62	41.83	4.62	3.58	10.62	1.34157849	0.24679507	0.63381695	8.486716259	3.085206444	up	-	-
MW0151208	HoPhe-Abu-OH	Others	Lactones	C19H20N2O6	2	0.8893	-	564322.01	84554.62	333664.26	641820.65	1139981.17	423272.44	1.08768378	0.19428371	0.58775794	0.445581769	-1.16623789	down	-	-
MW0015331	2-[(1R,5S,6R,11R,13R,16S)-6-(furan-3-yl)-11-hydroxy-1,5,15,15-tetramethyl-8,14,17-trioxo-7-oxatetracyclo[11.3.1.02,11.05,10]heptadec-9-en-16-yl]acetic acid	Benzene and substituted derivatives	Benzene and substituted derivatives	C26H30O8	2	0.886	-	1612.39	1876.98	3623.26	660.87	705.45	526.3	1.62668042	0.10909799	0.50002306	3.758086674	1.90999834	up	-	-
MW0153025	Lys-Leu-Arg-Asp	Amino acids and derivatives	Amino acids and derivatives	C22H42N8O7	2	0.8838	-	13660.96	6264.22	5317.24	5692.7	1363.46	2904.91	1.23752603	0.18401668	0.57807562	2.53410728	1.341477601	up	-	-
MW0158719	Tyr-Nap-OH	Amino acids and derivatives	Amino acids and derivatives	C28H24N2O7	2	0.876	-	6724.31	4545.55	2111.19	27446.4	29961.7	12197.21	1.55893137	0.07037701	0.45866819	0.192241799	-2.37900604	down	-	-
MW0150445	Gly-Phe-Asn-Thr-Phe	Amino acids and derivatives	Amino acids and derivatives	C28H36N6O8	2	0.8721	-	19280.86	34656.29	126551.8	131139.63	276855.72	87794.57	1.19853676	0.20454762	0.60017369	0.364043202	-1.45781843	down	-	-
MW0146256	Asp-Ser-Glu-Asp	Amino acids and derivatives	Amino acids and derivatives	C16H24N4O12	2	0.8233	-	1517.29	2347.39	18315.94	16732.82	30936.37	16938.94	1.2613572	0.12291496	0.51199163	0.343310045	-1.54241603	down	-	-
MW0159290	Val-Nap-OH	Amino acids and derivatives	Amino acids and derivatives	C23H22N2O6	2	0.8173	-	927.01	5765.81	1643.75	406.59	560.74	1353.78	1.15364193	0.31386971	0.6726196	3.591630728	1.844639028	up	-	-
MW0151402	Ile-Gln-Phe-Arg	Amino acids and derivatives	Amino acids and derivatives	C26H42N8O6	2	0.7909	-	273.76	245.51	420.36	629.4	734.86	1111.71	1.58238857	0.05888979	0.4369697	0.379499752	-1.39782915	down	-	-
MW0144792	Ala-Val-Asp-Asp	Amino acids and derivatives	Amino acids and derivatives	C16H26N4O9	2	0.7898	-	1792.85	1815.32	1917.23	470.74	312.98	630.26	1.69163859	0.00145187	0.13924282	3.907693178	1.966317195	up	-	-
MW0159029	Val-His-Phe-Glu	Amino acids and derivatives	Amino acids and derivatives	C25H34N6O7	2	0.7893	-	232.45	117.21	246.37	189.96	13.79	16.2	1.2164425	0.16157105	0.55635821	2.709843146	1.438209346	up	-	-
MW0159149	Val-Ser-Ala-Lys	Amino acids and derivatives	Amino acids and derivatives	C17H33N5O6	2	0.7783	-	24.24	12.52	39.22	137.08	328.48	204	1.64967404	0.06918483	0.45765247	0.113477508	-3.13952173	down	-	-
MW0156986	Ser-Val-Thr-Lys	Amino acids and derivatives	Amino acids and derivatives	C18H35N5O7	2	0.7753	-	221.16	45.09	370.93	526.1	647.01	617.53	1.28221121	0.04113409	0.39037745	0.355839253	-1.49070243	down	-	-
MW0151214	HoPhe-HoPhe-OH	Others	Lactones	C25H24N2O6	2	0.775	-	13604.68	723.41	1086.92	19386.91	45594.22	74726.25	1.45536029	0.11384323	0.50205214	0.110337836	-3.1800005	down	-	-
MW0168445	10-Hydroxydecanoic acid	Lipids	Free Lipidstty acids	C10H20O3	2	0.7738	-	17561.09	18846.74	28004.95	8424.57	3629.74	7786.94	1.57590987	0.0297317	0.34768046	3.246407358	1.69884404	up	C02774	-
MW0158439	Tyr-Gln-Leu-Lys	Amino acids and derivatives	Amino acids and derivatives	C26H42N6O7	2	0.7728	-	13749.21	7421.81	8107.85	82.25	365.36	2713.28	1.46303125	0.03443057	0.36074776	9.262856347	3.21145714	up	-	-
MW0011889	1,2-Dimyristoyl-sn-glycero-3-phospho-l-[illegible]	GL	PS	C34H66NO10P	2	0.7678	-	42.59	46.66	117.45	75.63	8.92	11.92	1.0611579	0.32367297	0.67853969	2.142635016	1.099386117	up	-	-
MW0158490	Tyr-His-Ser-Asp	Amino acids and derivatives	Amino acids and derivatives	C22H28N6O9	2	0.7637	-	1732.05	1060.23	1661.43	4507.68	4901.1	3437.09	1.66179746	0.01147586	0.25665182	0.346703649	-1.52822508	down	-	-

MW0159180	Val-Thr-Ile-Asp	Amino acids and derivatives	Amino acids and derivatives	C19H34N4O8	2	0.7623	-	5685.31	7773.75	7404.1	1459.53	3469.55	3249.92	1.51755038	0.00951203	0.24021401	2.550820394	1.350961321	up	-	-
MW0145739	Asn-Glu-Ile-Lys	Amino acids and derivatives	Amino acids and derivatives	C21H38N6O8	2	0.7618	-	5045.65	5443.54	3726.35	2438.34	114.65	614.56	1.35049506	0.01633872	0.29436263	4.487866016	2.166029605	up	-	-
MW0153003	Lys-Ile-Asn-Glu	Amino acids and derivatives	Amino acids and derivatives	C21H38N6O8	2	0.76	-	15.17	59.19	40.34	125.01	98.54	148.6	1.4757438	0.01163589	0.25665182	0.308209055	-1.69801884	down	-	-
MW0156968	Ser-Val-Arg-Glu	Amino acids and derivatives	Amino acids and derivatives	C19H35N7O8	2	0.7587	-	1478.79	2237.3	1222.88	87.57	170.35	44.89	1.69086552	0.03507952	0.36581753	16.3104587	4.027725451	up	-	-
MW0146227	Asp-Phe-Ser-Arg	Amino acids and derivatives	Amino acids and derivatives	C22H33N7O8	2	0.7568	-	1915.5	3851.09	3785.43	9855.03	17412.66	5271.35	1.43098169	0.15909289	0.55258596	0.29355568	-1.76829393	down	-	-
MW0149927	Glu-Asn-Gln-Glu	Amino acids and derivatives	Amino acids and derivatives	C19H30N6O11	2	0.7567	-	3641.51	2610.03	1972.98	6612.03	8278.56	4899.99	1.56077013	0.03976868	0.38624483	0.415577512	-1.26681051	down	-	-
MW0155439	Phe-Abu-OH	Amino acids and derivatives	Amino acids and derivatives	C18H18N2O6	2	0.7553	-	151.88	115.78	129.44	88.55	58.93	33.94	1.45570656	0.0247333	0.33384987	2.188843567	1.130168851	up	-	-
MW0156960	Ser-Tyr-Lys-Asp	Amino acids and derivatives	Amino acids and derivatives	C22H33N5O9	2	0.7546	-	526.83	736.74	609.83	551.22	159.56	67.18	1.24194158	0.11864565	0.50367581	2.408092961	1.267891086	up	-	-
MW0158880	Val-Ala-Ser-Asp	Amino acids and derivatives	Amino acids and derivatives	C15H26N4O8	2	0.7507	-	1068.83	3016.18	2660.69	1150.34	688.76	441.73	1.3760722	0.11886881	0.50379673	2.957563694	1.564409239	up	-	-
MW0158300	Trp-HoPhe-OH	Amino acids and derivatives	Amino acids and derivatives	C27H25N3O6	2	0.7449	-	4345.68	8142.46	7635.49	14201.16	40352.69	7367.48	1.14590334	0.29939568	0.66510003	0.324987044	-1.62154589	down	-	-
MW0156703	Ser-Gln-Asp-Glu	Amino acids and derivatives	Amino acids and derivatives	C17H27N5O11	2	0.7429	-	6.93	9.03	18.77	274.8	494.58	238.32	1.72042615	0.05556616	0.42471427	0.034464622	-4.85873998	down	-	-
MW0146107	Asp-Glu-Thr-Asp	Amino acids and derivatives	Amino acids and derivatives	C17H26N4O12	2	0.7429	-	225.78	73.4	191.07	48.79	161.71	25.82	1.01904506	0.24683395	0.63381695	2.074517603	1.052775899	up	-	-
MW0154345	N-dodecanoyl-L-Homoserine lactone-3-hydrazone-biotin	Amino acids and derivatives	Amino acids and derivatives	C26H43N5O5S	2	0.7411	-	13976.95	86917.33	180820.9	660384.24	1005216.83	150955.15	1.28425401	0.17124272	0.5671048	0.155082005	-2.68889681	down	-	-
MW0144694	Ala-Phe-Lys-Arg	Amino acids and derivatives	Amino acids and derivatives	C24H40N8O5	2	0.7386	-	4941.04	4100.94	4702.32	1876.33	2216.09	2099.59	1.73087247	0.00439007	0.18300962	2.219683108	1.150353725	up	-	-
MW0153090	Lys-Phe-Asn-Lys	Amino acids and derivatives	Amino acids and derivatives	C25H41N7O6	2	0.7363	-	35.84	11.03	398.24	639.34	336.02	427.63	1.24027802	0.11400374	0.50205214	0.317258142	-1.65627091	down	-	-
MW0106263	Cys-Met	Amino acids and derivatives	Amino acids and derivatives	C8H16N2O3S2	2	0.7284	-	171.34	330.56	390.53	25992.88	154.9	23705	1.11212887	0.186756	0.57840853	0.017901309	-5.80379114	down	-	-
MW0105971	Asp-Ser	Amino acids and derivatives	Amino acids and derivatives	C7H12N2O6	2	0.7192	-	227.14	213.67	3423.23	2675.98	9023.46	4213.55	1.29745537	0.15995794	0.5530994	0.242823002	-2.042023	down	-	-
MW0123471	9H-Pyrrolo[1',2':2,3]isoindolo[4,5,6-cd]indol-9-one, 10-acetyl-2,6,6a,7,11a,11b-hexahydro-11-hydroxy-7,7-dimethyl-, (6aR,11aS,11bR)-	Organic acids	Organic acids	C20H20N2O3	2	0.7046	-	9948.85	8565.54	13063.91	10453.43	288.89	138.11	1.23188798	0.17043714	0.56680649	2.902302574	1.537197933	up	-	-
MW0012817	1-Hexadecyl-sn-glycerol 3-phosphate	GL	LPA	C19H41O6P	2	0.7038	-	6628.83	4058.18	7874.53	2445.82	4981.59	1834.23	1.27318244	0.1059272	0.49914367	2.004131018	1.002976826	up	-	-
pmn001691	9,12,13-Trihydroxy-10,15-octadecadienoic acid	Lipids	Free Lipidstty acids	C18H32O5	3	0.9984	-	9230.8	7077.14	19691.56	7870.75	2914.13	3919.05	1.27101991	0.20221848	0.59709014	2.448291035	1.291775065	up	-	-
MW0013337	26-Desglucoavenacoside A	Terpenoids	Triterpene Saponin	C45H72O18	3	0.9765	-	26.87	132.08	120.78	481.42	1593.85	583.74	1.54154781	0.15391508	0.5475101	0.105200808	-3.24878231	down	-	-
MEDN1221	Mannose 6-phosphate	Others	Saccharides	C6H13O9P	3	0.9657	-	49878.06	41627.62	57415.09	103435.7	126301.65	149226.69	1.68614248	0.01947557	0.31365607	0.392968077	-1.34751598	down	C00275	ko00051,ko00520,ko01100,ko01110,ko01240,ko01250
MW0011241	4-[(E)-3-[4,5-dihydroxy-6-(hydroxymethyl)-3-[3,4,5-trihydroxy-6-(hydroxymethyl)oxan-2-yl]oxyoxan-2-yl]oxybut-1-enyl]-4-hydroxy-3,5,5-trimethylcyclohex-2-en-1-one	Others	Hydrocarbon derivatives	C25H40O13	3	0.961	-	4956.16	22615.78	16194.43	37665.76	55741.76	43135.68	1.41984015	0.01418886	0.27261581	0.320531304	-1.64146283	down	-	-
MW0011668	(Z)-2-Methyl-2-butene-1,4-diol 4-O-beta-D-Glucopyranoside	Others	Saccharides	C11H20O7	3	0.9466	-	59.96	403.89	310.36	39.88	19.13	146.29	1.13148998	0.19790713	0.59185871	3.771115441	1.914991315	up	-	-
MW0061234	PG(20:4(5Z,8Z,11Z,14Z)/22:5(4Z,7Z,10Z,13Z,16Z))	GL	PG	C48H77O10P	3	0.9296	-	11226.41	16032.39	35691.69	761.18	1392.38	4464.57	1.56598446	0.12573799	0.51748183	9.511824337	3.249722072	up	-	-
MEDL00448	1-Palmitoylglycerol 3-phosphate	Lipids	Glycerol ester	C19H39O7P	3	0.911	-	29991.75	18069.72	20944.58	174885.36	45606.92	32208.57	1.22701394	0.31031068	0.67088493	0.273074072	-1.87263576	down	C04036	-
MW0145696	Asn-Asp-Asp	Amino acids and derivatives	Amino acids and derivatives	C12H18N4O9	3	0.9083	-	2280.22	2037.09	2809.5	286.03	1727.36	77.6	1.2887039	0.0663555	0.44856776	3.40834246	1.7690703	up	-	-
MEDN1251	LPI(16:0/0:0)	GL	PI	C25H49O12P	3	0.8974	-	19987.15	15918.34	15634.48	62884.14	23433.08	19338.93	1.08391501	0.32309738	0.67833614	0.487808518	-1.03561314	down	-	-
MW0012959	1-Palmitoyl-2-docosahexaenoyl-sn-glycero-3-phospho-(1'-rac-glycerol)	GL	PG	C44H75O10P	3	0.8835	-	3974.2	8133.05	9649.31	380.13	771.05	5649.15	1.26262682	0.1061518	0.4992359	3.199338856	1.677773803	up	-	-

MEDL02559	3,4-Bis[3-(3,4-dihydroxyphenyl)prop-2-enoyloxy]-1,5-dihydroxycyclohexane-1-carboxylic acid	Phenolic acids	Phenolic acids	C25H24O12	3	0.8832	-	156130.22	295572.68	622992.85	1284531.26	2839255.87	728969.82	1.41158684	0.17953175	0.57366954	0.221460865	-2.17487632	down	-	-
ZINC9591202	(2S)-2alpha-[(E)-2,4,5-Trimethoxystyryl]-3abeta-(4-methoxyphenyl)-6-methoxy-8,9abeta-dihydroxy-2,3,3a,9a-tetrahydro-9H-furo[3,2-b][1]benzopyran-9-one	Others	Ketone compounds	C30H30O10	3	0.8772	-	196.28	591.95	532.24	6619.84	9670.49	7855.93	1.70915881	0.01205964	0.25665182	0.054686316	-4.19267632	down	-	-
MEDN1261	LPS(18:3/0:0)	GL	LPS	C24H42NO9P	3	0.8651	-	111.1	371.03	981.78	7105.28	905.15	909.53	1.16909086	0.3518207	0.69843207	0.164116207	-2.60721038	down	-	-
MW0133347	N,N,2-trimethyl-3,4-diphenyl-3-propanoyloxybutan-1-amine oxide	Others	Lactones	C22H29NO3	3	0.8649	-	23538.2	12184.06	7824.9	15519.59	2541.21	3427.07	1.03230038	0.30750781	0.67088493	2.026592678	1.019056152	up	-	-
MW0147063	Cannabisin G	Lignans and Coumarins	Lignans	C36H36N2O8	3	0.8567	-	8246.2	3326.05	12929.13	3311.16	4426.26	2051.34	1.17422643	0.21431901	0.60749018	2.503011617	1.323664987	up	C17911	-
MW0112439	3,4,5-trihydroxy-6-[(4-methoxy-1-benzofuran-6-yl)oxy]oxane-2-carboxylic acid	Organic acids	Organic acids	C15H16O9	3	0.8314	-	244.66	127.72	760.66	1360.71	385.56	926.29	1.08809774	0.21720936	0.60875495	0.423953064	-1.23802354	down	-	-
MW0130947	3-(1,2-dihydroxybutyl)-7-hydroxy-1H-isochromen-1-one	Others	Ketone compounds	C13H14O5	3	0.813	-	3143.68	1311.78	1579.76	561.3	528.57	370.63	1.61275423	0.11484066	0.50205214	4.132297159	2.046944004	up	-	-
MW0133939	5,6,7-trihydroxy-2-(4-hydroxyphenyl)-8-(3,4,5-trihydroxyoxan-2-yl)-4H-chromen-4-one	Others	Ketone compounds	C20H18O10	3	0.8071	-	234.87	334.98	418.56	96.63	278.13	77.32	1.28959263	0.10024026	0.49047358	2.186360821	1.128531513	up	-	-
MW0134075	CID 14730445	Others	Ketone compounds	C28H32O16	3	0.8045	-	5234.97	2021.94	4445.72	6361.23	8253.37	9028.58	1.37196687	0.0352147	0.36636516	0.494968528	-1.0145913	down	-	-
MW0130946	3-(1,2-dihydroxybutyl)-6-hydroxy-1H-isochromen-1-one	Others	Ketone compounds	C13H14O5	3	0.8034	-	961.82	659.75	671.96	329.23	418.25	308.76	1.63355518	0.04122901	0.3904422	2.171409907	1.118632096	up	-	-
MEDP1124	15-Hydroxy-5,8,11,13-eicosatetraenoic acid	Lipids	Free Lipidstty acids	C20H32O3	3	0.7982	-	777.84	877.73	628.86	536.86	77.59	362.12	1.20186772	0.06238825	0.44396349	2.339238355	1.226038871	up	-	-
MW0135343	6-[1-(3,4-dihydroxyphenyl)-3-(4-methoxy-1-benzofuran-5-yl)-1,3-dioxopropan-2-yl]-3,4,5-trihydroxyoxane-2-carboxylic acid	Organic acids	Organic acids	C24H22O12	3	0.7948	-	469.97	605.58	433.58	314.57	11.87	254.85	1.06830362	0.05827072	0.4343505	2.596174027	1.376387093	up	-	-
MEDL00494	9-[(3S,4S,5R)-3,4-dihydroxy-5-(hydroxymethyl)oxolan-2-yl]-1H-purin-6-one	Nucleotides and derivatives	Nucleotides and derivatives	C10H12N4O5	3	0.7851	-	2568.78	4359.77	1207.25	2731.02	7142.54	7922.42	1.10382718	0.17659079	0.57342844	0.457170664	-1.12919526	down	-	-
MW0133510	4-[5-(3-Hydroxypropyl)-1-benzofuran-2-yl]-2-methoxyphenol	Benzene and substituted derivatives	Benzene and substituted derivatives	C18H18O4	3	0.7715	-	3155.27	2704.59	3363.74	281.89	842.15	487.43	1.66195104	0.00065321	0.12131435	5.723718096	2.516952618	up	-	-
MW0145073	Ancistrobrevine A	Heterocyclic compounds	Heterocyclic compounds	C27H33NO4	3	0.7613	-	12527.72	13715.5	5814.7	17780.58	29633.48	16971.05	1.31547147	0.1017149	0.49313285	0.497908911	-1.00604626	down	C12342	-
MW0162756	2-[(3,4-dihydroxy-2-oxo-5-phosphonatooxypentyl)amino]benzoate	Others	Others	C12H13NO9P-3	3	0.7608	-	108188.82	118419.1	379873.33	178529.37	576310.45	495473.09	1.02394788	0.23306963	0.6241478	0.485063575	-1.04375425	down	-	-
MW0054498	LysoPA(20:5(5Z,8Z,11Z,14Z,17Z)/0:0)	GL	LPA	C23H37O7P	3	0.7585	-	5360.59	5267.52	2197.98	7474.51	15799.82	6590.16	1.27650605	0.18385559	0.57807562	0.429476278	-1.21934965	down	-	-
MW0156428	(1R,3R,8R,12S,13R,17R,18Z,20Z,24R,25S,26S)-12-hydroxy-17-[(1R)-1-hydroxyethyl]-5,13,25-trimethylspiro[2,10,16,23-tetraoxatetracyclo[22.2.1.03,8.08,25]heptacosa-4,18,20-triene-26,2'-	Terpenoids	Sesquiterpenoids	C29H40O9	3	0.7572	-	43593.89	19173.72	38939.29	62442.45	157358.32	69836.69	1.40178377	0.17021444	0.56680649	0.351152437	-1.50983065	down	-	-
MW0127704	{[6-[3-(4-hydroxyphenyl)-3-oxopropyl]-5-methoxy-2-methyl-2H-chromen-2-yl]methoxy}sulfonic	Organic acids	Organic acids	C21H22O8S	3	0.7569	-	38.73	99.13	105.65	123.59	102.96	263.53	1.10593073	0.24028937	0.62977439	0.496878061	-1.00903625	down	-	-
MW0112394	Quinol glucuronide	Organic acids	Organic acids	C12H14O8	3	0.7516	-	182656.29	173976.42	223703	217589.17	603214.05	371082.89	1.27749982	0.20878857	0.6025099	0.486905339	-1.03828678	down	-	-
ZINC3461983	Petalosa	Others	Saccharides	C10H18O10	3	0.7498	-	272.94	1812.08	427.55	278.17	111.63	67.99	1.3007543	0.29574917	0.662229	5.488477249	2.456405936	up	-	-

MW0130945	3-(1,2-dihydroxybutyl)-5-hydroxy-1H-isochromen-1-one	Others	Ketone compounds	C13H14O5	3	0.7497	-	343.64	117.67	265.76	14.52	97.43	81.43	1.30173863	0.10110438	0.49241167	3.759799359	1.910655675	up	-	-
MW0166427	2-Keto-3,6-dideoxy-6-sulfogluconate	Organic acids	Organic acids	C6H8O8S-2	3	0.7493	-	2031.03	1662.73	1910.08	206.22	1317.75	907.19	1.21692545	0.07034887	0.45866819	2.305006663	1.204770921	up	-	-
MW0054490	LysoPA(18:3(6Z,9Z,12Z)/0:0)	GL	LPA	C21H37O7P	3	0.7464	-	2243.33	1943.87	2482	8373.07	2610.84	3924.95	1.23982564	0.25528228	0.64155925	0.447331318	-1.16058433	down	-	-
Zmhn001793	6-O-Caffeoyl-D-glucose	Phenolic acids	Phenolic acids	C15H18O9	3	0.7449	-	653179.75	704170.85	864060.09	533092.74	225891.77	329594.74	1.45503923	0.03164135	0.34952497	2.040651326	1.029029699	up	-	-
MW0110087	Trp-Val-Asn	Amino acids and derivatives	Amino acids and derivatives	C20H27N5O5	3	0.7423	-	937.46	853.36	620.3	252.64	503.08	353.76	1.51862847	0.02478263	0.33384987	2.173198255	1.119819794	up	-	-
MW0134622	NCGC00384692-01_C32H42O17_beta-D-Glucopyranoside, 6-[[6-deoxy-3-O-[(2E)-3-(3,4-dimethoxyphenyl)-1-oxo-2-propen-1-yl]-alpha-L-mannopyranosyl]oxy]-1a,1b,2,5a,6,6a-hexahydro-1a-(hydroxymethyl)oxireno[4,5]cyclopenta[1,2-c]pyran-2-yl	Benzene and substituted derivatives	Benzene and substituted derivatives	C32H42O17	3	0.7409	-	120.24	31.78	29.19	14.62	41.05	12.42	1.06343753	0.33471849	0.68687581	2.661330592	1.412147734	up	-	-
MW0112547	3,4,5-trihydroxy-6-[5-hydroxy-4-methoxy-2-(3-oxobut-1-en-1-yl)phenoxy]oxane-2-carboxylic acid	Benzene and substituted derivatives	Benzene and substituted derivatives	C17H20O10	3	0.7381	-	48.87	444.3	70.52	488.53	570.35	244.6	1.14896786	0.20613204	0.60099477	0.432450057	-1.20939457	down	-	-
MW0154359	3-[(4-Hydroxy-3,5-dimethoxyphenyl)methyl]-4-[[4-[4-[[4-[(4-hydroxy-3,5-dimethoxyphenyl)methyl]-5-oxooxolan-3-yl]methyl]-2-methoxyphenyl]-3-methoxyphenyl]methyl]oxolan-2-one	Lignans and Coumarins	Lignans	C42H46O12	3	0.7353	-	1872.29	2084.57	2410.23	1269.59	1171.09	49.6	1.06064273	0.0651573	0.44856776	2.556776748	1.354326193	up	C17683	-
MW0148610	Ditrans,polycis-heptaprenyl diphosphate	Organic acids	Organic acids	C35H60O7P2	3	0.7351	-	404.73	53.73	292.28	29.66	12.39	24.57	1.49280077	0.15807967	0.5507933	11.26898829	3.494286094	up	C17436	-
MW0056357	PA(22:6(4Z,7Z,10Z,13Z,16Z,19Z)/15:0)	GL	PA	C40H67O8P	3	0.7335	-	7958.17	19565.76	16340.97	3507.6	3142.02	3460.13	1.64044277	0.08277463	0.47220697	4.338870892	2.117319658	up	-	-
MW0127983	(2-hydroxy-5-{3,5,6,7-tetrahydroxy-8-[3,4,5-trihydroxy-6-(hydroxymethyl)oxan-2-yl]-3,4-dihydro-2H-1-benzopyran-2-yl}phenyl)oxidanesulfonic acid	Organic acids	Organic acids	C21H24O15S	3	0.7329	-	116.04	2051.82	72.71	171.3	20.17	20.97	1.03559895	0.40911953	0.72930919	10.54683675	3.39873846	up	-	-
MW0061562	PGP(i-12:0/i-12:0)	GL	PG-P	C30H60O13P2	3	0.7327	-	96.13	206.29	82.58	991.4	651.79	688.64	1.66935338	0.0171219	0.29823307	0.165106376	-2.59853226	down	-	-
MW0015564	[(1S,6S,7S,8R,9R,13R,14R,16S,18R)-8-acetyloxy-11-ethyl-5,7,14-trihydroxy-6,16,18-trimethoxy-13-(methoxymethyl)-11-azahexacyclo[7.7.2.12,5.01,10.03,8.013,17]nonadecan-4-yl] benzoate	Benzene and substituted derivatives	Benzene and substituted derivatives	C34H47NO11	3	0.7305	-	11324.62	10664.78	10661.29	7949.38	2531.39	5628.64	1.34983703	0.06962792	0.45866819	2.026808555	1.019209823	up	-	-
MW0003445	3-(3,4-dimethoxyphenyl)-N-[2-(3,4-dimethoxyphenyl)-2-(sulfooxy)ethyl]prop-2-enimidic acid	Organic acids	Organic acids	C21H25NO9S	3	0.7294	-	0.09	0.09	0.09	0.09	0.65	0.65	1.24483779	0.18350342	0.57807562	0.194244604	-2.36405357	down	-	-
MW0134150	5,7-dihydroxy-3-[3-hydroxy-4-methoxy-5-(3-methylbut-2-en-1-yl)phenyl]-8-(3-methylbut-2-en-1-yl)-3,4-dihydro-2H-1-benzopyran-4-one	Benzene and substituted derivatives	Benzene and substituted derivatives	C26H30O6	3	0.7285	-	19480.98	11175.5	30112.34	4136.79	7233.14	5139.42	1.55706252	0.11097995	0.50205214	3.68087296	1.880047958	up	-	-
MEDP1089	Humic acid	Organic acids	Organic acids	C9H9NO6	3	0.7259	-	14296.69	10887.88	14187.33	137.86	1587.69	470.21	1.61937624	0.0035233	0.18110516	17.93087587	4.164374056	up	-	-
FDATN00542	2-Hydroxy-1,4-naphthoquinone	Benzene and substituted derivatives	Benzene and substituted derivatives	C10H6O3	3	0.7244	-	162.83	292.35	3685.59	4844.2	5587.4	2282.4	1.23184216	0.13603947	0.52749511	0.325685858	-1.61844702	down	-	-

MW0112461	3,4,5-trihydroxy-6-[2-hydroxy-3,4-dimethoxy-5-(3-oxoprop-1-en-1-yl)phenoxy]oxane-2-carboxylic acid	Benzene and substituted derivatives	Benzene and substituted derivatives	C17H20O11	3	0.7233	-	3637.52	9570.48	2448.41	13959.51	11677.24	10377.79	1.35233582	0.07270181	0.46096484	0.434724697	-1.20182604	down	-	-
MW0128125	(3-{8-[1-(2,4-dihydroxyphenyl)-3-(3,4-dihydroxyphenyl)-2-hydroxypropyl]-3,5,7-trihydroxy-3,4-dihydro-2H-1-benzopyran-2-yl}phenyl)oxidanesulfonic acid	Organic acids	Organic acids	C30H28O13S	3	0.7223	-	753.55	191.63	873.95	48.82	30.41	53.29	1.62362051	0.11587151	0.50205214	13.72721099	3.778966632	up	-	-
MW0130544	2-[2,5-dihydroxy-4-(methoxymethyl)phenyl]-3,4-dihydro-2H-1-benzopyran-3,5,7-triol	Benzene and substituted derivatives	Benzene and substituted derivatives	C17H18O7	3	0.7218	-	160871.2	202176.43	255729.92	231.52	129.98	606.46	1.74451957	0.01731651	0.29823307	639.2594219	9.320257709	up	-	-
MW0139072	N-(4-aminobutyl)-3-[3-methoxy-4-(sulfooxy)phenyl]prop-2-enimidic acid	Organic acids	Organic acids	C14H20N2O6S	3	0.7214	-	242.59	2890.59	3951.85	251.15	226.15	117.49	1.30675773	0.18868716	0.58166266	11.91181762	3.574321665	up	-	-
MW0144445	2-[4,5-Dihydroxy-3-(1-hydroxy-3-oxopent-1-enyl)-9,10-dioxoanthracen-2-yl]acetic acid	Benzene and substituted derivatives	Benzene and substituted derivatives	C21H16O8	3	0.7207	-	135.7	426.24	126.23	270.49	1099.2	339.4	1.05070988	0.3294517	0.68422208	0.402652874	-1.31239146	down	-	-
ZINC1448950	(1S,2S,3R,4S)-2-[(Benzoyloxy)methyl]-5-cyclohexene-1,2,3,4-tetraol 3,4-diacetate	Benzene and substituted derivatives	Benzene and substituted derivatives	C18H20O8	3	0.7141	-	15167.09	9187.57	9636.81	7494.06	190.19	2834.88	1.1564512	0.05343674	0.41861832	3.231395562	1.692157365	up	-	-
MW0146202	Asp-Lys-Val-Lys	Amino acids and derivatives	Amino acids and derivatives	C21H40N6O7	3	0.7084	-	1131.42	1301.48	1389.03	815.04	432.65	49.36	1.20110834	0.05035501	0.40874247	2.946632744	1.55906726	up	-	-
MW0128573	[2-(methoxymethyl)-5-(3,5,7-trihydroxy-3,4-dihydro-2H-1-benzopyran-2-yl)phenyl]oxidanesulfonic acid	Organic acids	Organic acids	C17H18O9S	3	0.7083	-	212.76	101.74	409.43	78.33	199.24	17.93	1.02041909	0.2588696	0.64187626	2.449847716	1.292692073	up	-	-
MW0131269	3-(3-hydroxyphenyl)-2-phenyl-4-[(E)-2-phenylethenyl]-2,3-dihydro-1-benzofuran-6-ol	Benzene and substituted derivatives	Benzene and substituted derivatives	C28H22O3	3	0.7065	-	3322.87	2823.42	1834.81	949.99	1091.49	1670.85	1.48208257	0.06334571	0.44396349	2.149889692	1.104262639	up	-	-
ZINC8553093	[(2S,3R,4S,5R,6S)-3,4-dihydroxy-6-methyl-5-[(2S,3R,4R,5R,6S)-3,4,5-trihydroxy-6-methyloxan-2-yl]oxyoxan-2-yl] (11S)-11-[illegible]	Others	Lactones	C28H52O11	3	0.7061	-	1022.1	263.82	566.94	1183.15	2306.72	751.39	1.14433819	0.22300914	0.61283622	0.43686546	-1.19473905	down	-	-
MW0062176	Prieuranin acetate	Terpenoids	Triterpene	C40H52O17	3	0.7041	-	13.84	47.48	207.61	283.61	69.45	838.22	1.05490707	0.31021283	0.67088493	0.225748774	-2.14720994	down	-	-
MW0129189	{3-[2-(3,5-dihydroxyphenyl)ethyl]phenyl}oxidanesulfonic acid	Organic acids	Organic acids	C14H14O6S	3	0.7038	-	1299.09	11061.62	21235.6	16974.19	50727.71	23799.72	1.07537812	0.19649404	0.59041464	0.367166286	-1.4454945	down	-	-
MW0128746	[4-(3-{5-[(3,3-dimethyloxiran-2-yl)methyl]-2,4-dihydroxyphenyl}-2-hydroxy-3-oxopropyl)-2-(3-methylbut-2-en-1-yl)phenyl]oxidanesulfonic acid	Organic acids	Organic acids	C25H30O9S	3	0.7031	-	32740.84	24030.16	15189.64	3765.05	1830.42	11812.9	1.4388375	0.04827667	0.40294196	4.133680523	2.047426892	up	-	-
MW0000672	(3E)-4-phenylbuta-1,3-dien-2-ol	Benzene and substituted derivatives	Benzene and substituted derivatives	C10H10O	3	0.7004	-	3995.95	4036.7	4457.91	121.19	849.57	182.63	1.61712395	0.0004435	0.10041972	10.82943324	3.436885836	up	-	-
MW0131709	CID 131839423	Organic acids	Organic acids	C15H14O9	3	0.7003	-	103.07	2274.64	173.08	1217.61	8508.15	2097.91	1.20024206	0.30956239	0.67088493	0.215735892	-2.21266187	down	-	-

MW0104588	2-amino-4-({1-[(carboxymethyl)-C-hydroxycarbonimidoyl]-2-{[1-(6,7-dimethoxy-2H-1,3-benzodioxol-5-yl)-2-hydroxy-3-oxopropyl]sulLipidsnyl}ethyl}-C-hydroxycarbonimidoyl)butanoic acid	Organic acids	Organic acids	C22H29N3O12S	3	0.6979	-	60606.29	87383.91	114733.23	134644.92	586120.67	227922.97	1.35447503	0.23741008	0.62780518	0.276933275	-1.85238968	down	-	-
MW0128192	(4-{3-[2,4-dihydroxy-5-(3-methylbut-2-en-1-yl)phenyl]-2-hydroxy-3-oxopropyl}-2-[(3,3-dimethyloxiran-2-yl)methyl]phenyl)oxidanesulfonic acid	Organic acids	Organic acids	C25H30O9S	3	0.6978	-	8653.31	1888.47	12047.75	21849.23	22698.25	18966	1.31920471	0.0321199	0.35051264	0.355665128	-1.49140857	down	-	-
MW0130689	2-{6-[2,4-dihydroxy-5-methoxy-3-(3-methylbut-2-en-1-yl)benzoyl]-5-(2,4-dihydroxyphenyl)-3-methylcyclohex-2-en-1-yl}-5-[(E)-2-(2,4-dihydroxyphenyl)ethenyl]benzene-1,3-diol	Benzene and substituted derivatives	Benzene and substituted derivatives	C40H40O10	3	0.6966	-	917.28	3320	1516.56	4514.45	6457.99	2906.67	1.3014232	0.10495029	0.49854201	0.414568369	-1.27031805	down	-	-
ZINC9591248	(2S,3S,4S,5R,6S)-6-[5,7-dihydroxy-2-(4-hydroxyphenyl)-4-oxochromen-8-yl]oxy-4-hydroxy-3,5-disulfooxyoxane-2-carboxylic acid	Organic acids	Organic acids	C21H18O18S2	3	0.6948	-	2360.13	687.8	685.71	2312.4	3937.7	3756.39	1.39239553	0.05142381	0.41145636	0.373121844	-1.42228127	down	-	-
MW0105312	5-Hydroxyindoleacetylglycine	Amino acids and derivatives	Amino acids and derivatives	C12H12N2O4	3	0.6947	-	1146.71	181.28	55.46	56.85	32.55	92.23	1.05687348	0.36505766	0.7041771	7.616858448	2.929196085	up	C05832	ko00380
MW0134554	5-Hydroxy-7-[3,4,5-trihydroxy-6-(hydroxymethyl)oxan-2-yl]oxy-2-(3,4,5-trimethoxyphenyl)chromen-4-one	Others	Ketone compounds	C24H26O12	3	0.6942	-	5868.26	2632.56	13009.54	10951.85	23332.87	9579.25	1.08025059	0.24323285	0.632619	0.490387897	-1.02800472	down	-	-
MW0158969	Val-Gln-Ile-Asp	Amino acids and derivatives	Amino acids and derivatives	C20H35N5O8	3	0.6931	-	1496.99	5151.13	2653.22	1552.34	1502.34	412.74	1.18344874	0.20601173	0.60099477	2.682495919	1.423575976	up	-	-
MW0131403	3-(5-hydroxy-2,2-dimethyl-2H-chromen-6-yl)-1-phenylpropan-1-one	Others	Ketone compounds	C20H20O3	3	0.6929	-	272.61	151.57	167.25	63.34	34.03	37.03	1.65669864	0.04993171	0.40754576	4.400520833	2.137674287	up	-	-
MW0114006	[4,5-Diacetyloxy-6-[4-[(ethoxycarbonylamino)methyl]phenoxy]-2-methyloxan-3-yl] acetate	Others	Lactones	C22H29NO10	3	0.6923	-	492.29	478.31	510.07	5256.26	14019.68	16013.44	1.71657424	0.07624929	0.46149681	0.041957949	-4.57491204	down	-	-
MW0118191	Ncgc00347814-02_C29H37NO13_	Benzene and substituted derivatives	Benzene and substituted derivatives	C29H37NO13	3	0.6923	-	7489.95	2007.86	5458.27	14923.74	93943.51	66330.74	1.51956676	0.1468017	0.53780685	0.085366733	-3.55018222	down	-	-
MW0151760	Ipodate	Organic acids	Organic acids	C12H13I3N2O2	3	0.692	-	4168.13	6584.64	3740.69	33463.76	83645.93	16178.95	1.57615035	0.18924753	0.58166266	0.108737399	-3.20107987	down	-	-
MW0130532	2-[2-(3,7-dimethylocta-2,6-dien-1-yl)-5-hydroxy-3-methoxyphenyl]-1-benzofuran-4,6-diol	Others	Others	C25H28O5	3	0.6909	-	8575.65	5930.41	1636.57	571.01	1648.06	1520.07	1.31092532	0.17441275	0.57211065	4.317203956	2.110097251	up	-	-
MW0111541	Stearoylglycerone phosphate	Lipids	Free Lipidstty acids	C21H41O7P	3	0.6889	-	5620.52	4333.53	3044.46	10708.45	8120.51	7723.07	1.54059774	0.02128645	0.32374651	0.489548633	-1.03047591	down	C03805	-
MW0109423	Prolylprolyllysine	Amino acids and derivatives	Amino acids and derivatives	C16H28N4O4	3	0.6882	-	1552.24	822.23	824.2	439.23	247.78	19.69	1.27759465	0.05658023	0.42642523	4.526206311	2.178302346	up	-	-
MW0108507	N,O-Bis-(trimethylsilyl)phenylalanine	Amino acids and derivatives	Amino acids and derivatives	C15H27NO2Si2	3	0.6834	-	6.28	12.02	1.4	87.54	51.76	56.88	1.57180645	0.02723705	0.33858973	0.100417983	-3.31591044	down	-	-
MW0134402	5-{6-[2,4-dihydroxy-3-(3-methylbut-2-en-1-yl)benzoyl]-5-{4-[(E)-2-(2,4-dihydroxyphenyl)ethenyl]-2,6-dihydroxyphenyl}-3-methylcyclohex-3-en-1-yl}benzene-1,2,4-triol	Benzene and substituted derivatives	Benzene and substituted derivatives	C39H38O10	3	0.6833	-	2748.37	4567.91	3042.36	8898.49	5924.15	7234.51	1.56866193	0.02518015	0.33594919	0.469627309	-1.09041179	down	-	-

MW0134795	6-({8-[2-(acetyloxy)propan-2-yl]-2-oxo-2H,8H,9H-furo[2,3-h]chromen-9-yl}oxy)-3,4,5-trihydroxyoxane-2-carboxylic acid	Organic acids	Organic acids	C22H24O12	3	0.6829	-	6956.66	10060.05	23160.47	72400.84	74934.02	24525.63	1.44541875	0.10545527	0.49914367	0.233777874	-2.0967897	down	-	-
MW0156970	Ser-Val-Asn-Glu	Amino acids and derivatives	Amino acids and derivatives	C17H29N5O9	3	0.6791	-	17019.14	17683.34	14245.76	2102.69	2783.58	1966.01	1.74730247	0.00382703	0.18170858	7.143350826	2.836600977	up	-	-
MW0151224	HoPhe-Trp-OH	Others	Lactones	C26H23N3O6	3	0.6776	-	46.09	16.85	51.15	100.04	40.17	111.81	1.13714218	0.16244405	0.55831836	0.452702166	-1.14336588	down	-	-
MW0134117	5,7-dihydroxy-2-phenyl-6,8-bis[3,4,5-trihydroxy-6-(hydroxymethyl)oxan-2-yl]-3,4-dihydro-2H-1-benzopyran-4-one	Benzene and substituted derivatives	Benzene and substituted derivatives	C27H32O14	3	0.6771	-	3880.26	1020.09	2584.41	146.64	2215.1	243.78	1.16033566	0.2048485	0.60017369	2.872654979	1.522384728	up	-	-
MW0128569	[2-(acetyloxy)-2-(9-{[(2E)-2-methylbut-2-enoyl]oxy}-2-oxo-2H,8H,9H-furo[2,3-h]chromen-8-yl)propoxy]sulfonic acid	Organic acids	Organic acids	C21H22O11S	3	0.6763	-	1343.27	831.29	1548.87	94.78	318.14	494.36	1.46521083	0.02912861	0.3433457	4.103948065	2.037012474	up	-	-
MW0131647	3,4,5-trihydroxy-6-(3-{3,5,7-trihydroxy-8-[3,4,5-trihydroxy-6-(hydroxymethyl)oxan-2-yl]-3,4-dihydro-2H-1-benzopyran-2-yl}phenoxy)oxane-2-carboxylic acid	Organic acids	Organic acids	C27H32O16	3	0.6753	-	56988.87	45201.89	83824.42	1532.53	2249.45	68864.65	1.19941555	0.229543	0.62108339	2.560547957	1.35645258	up	-	-
MW0144203	Abu-His-OH	Amino acids and derivatives	Amino acids and derivatives	C15H16N4O6	3	0.6747	-	2367.07	861.12	1201.26	3462.13	3673.69	4993.17	1.52222033	0.01791064	0.29968703	0.365195288	-1.45325994	down	-	-
MW0112353	3,4,5-trihydroxy-6-({2-hydroxy-3-[4-hydroxy-3-(3-methylbut-2-en-1-yl)phenyl]propanoyl}oxy)oxane-2-carboxylic acid	Organic acids	Organic acids	C20H26O10	3	0.6741	-	6520.19	9099.28	7850.71	6497.69	84273.25	32000.71	1.10144333	0.28501405	0.65528903	0.191169378	-2.38707665	down	-	-
MEDN1252	LPI(16:1/0:0)	GL	PI	C25H47O12P	3	0.674	-	1727.61	1698.47	2277.64	1248.15	794.38	575.41	1.51357203	0.01982213	0.31365607	2.178705394	1.123471128	up	-	-
MW0158512	Tyr-Leu-Ala-Lys	Amino acids and derivatives	Amino acids and derivatives	C24H39N5O6	3	0.674	-	787.79	1699.23	814.6	329.55	131.65	76.27	1.56835909	0.08318113	0.47221936	6.142891696	2.618917948	up	-	-
MW0104544	2-amino-4-({1-[(carboxymethyl)-C-hydroxycarbonimidoyl]-2-[(2-{3,5-dihydroxy-4-[(1E)-3-methylbut-1-en-1-yl]phenyl}-2-hydroxy-1-(4-hydroxyphenyl)ethyl)sulLipidsnyl]ethyl}-C-hydroxycarbonimidoyl)butanoic acid	Organic acids	Organic acids	C29H37N3O10S	3	0.6736	-	25490.87	39253.04	36129.43	17553.18	22897.12	3322.53	1.17054492	0.06310122	0.44396349	2.304473803	1.204437367	up	-	-
MW0128777	[4,5-dihydroxy-2-({7-oxo-7H-furo[3,2-g]chromen-9-yl}oxy)oxan-3-yl]oxidanesulfonic acid	Organic acids	Organic acids	C16H14O11S	3	0.6733	-	491.03	448.06	99.82	2185.78	1929.38	937.54	1.46444259	0.06058763	0.43825617	0.20561482	-2.28198384	down	-	-
MW0132157	3,4,5-trihydroxy-6-{[5-hydroxy-2-(4-hydroxyphenyl)-7-methoxy-4-oxo-8-[3,4,5-trihydroxy-6-(hydroxymethyl)oxan-2-yl]-4H-chromen-6-yl]oxy}oxane-2-carboxylic acid	Organic acids	Organic acids	C28H30O17	3	0.6716	-	303.56	32.7	5.01	402.61	401.95	238.44	1.19964372	0.11762567	0.50254671	0.327200384	-1.61175366	down	-	-
MW0158035	Ethyl 3-aminobenzoate	Benzene and substituted derivatives	Benzene and substituted derivatives	C9H11NO2	3	0.6704	-	78.46	106.03	108.16	72.24	2.78	34.84	1.11386489	0.07508631	0.46149681	2.663844894	1.413510082	up	C18090	-
MW0150185	Glu-Tyr-Asn-Lys	Amino acids and derivatives	Amino acids and derivatives	C24H36N6O9	3	0.6695	-	2746.08	2306.41	3114.13	860.99	953.07	1259.47	1.69094099	0.00756262	0.22054168	2.657081597	1.409842532	up	-	-
MW0146643	4-deoxy-alpha-L-threo-hex-4-enopyranuronosyl-(1->4)-beta-D-glucopyranosyl-(1->4)-alpha-L-rhamnopyranosyl-(1-	Others	Saccharides	C24H38O20	3	0.6667	-	26.99	12.09	165.09	162.52	59.47	203.36	1.00731572	0.32002645	0.67617274	0.480004702	-1.05887956	down	C19965	-

MW0148729	dTDP-3-O-methyl-4-oxo-2,6-dideoxy-L-mannose	Nucleotides and derivatives	Nucleotides and derivatives	C17H26N2O14P2	3	0.665	-	186.13	111.57	38.42	24.37	5.77	94.64	1.03993501	0.24754983	0.63413128	2.693700914	1.429589675	up	C21071	ko01100,ko01110,ko01250
MW0012595	7,8,17-trihydroxy-4,9,11,13,15,19-docosahexaenoic acid	Lipids	Others	C22H32O5	3	0.6625	-	5987.07	5684.58	17439.24	52475.85	10763.97	15768.32	1.0578309	0.33302157	0.68616996	0.368454314	-1.44044235	down	-	-
MW0127979	(2-hydroxy-1,2-diphenylethoxy)sulfonic acid	Organic acids	Organic acids	C14H14O5S	3	0.6618	-	669.67	347.06	15435.66	13975.24	44041.32	19988.69	1.3102008	0.14187081	0.53210743	0.210913881	-2.24527405	down	-	-
MW0132145	3,4,5-Trihydroxy-6-[5-hydroxy-2-(4-hydroxyphenyl)-4-oxochromen-7-yl]oxyoxane-2-carboxylic acid	Organic acids	Organic acids	C21H18O11	3	0.6603	-	1615.81	7640.29	17142.33	14728.61	45910.86	21571.26	1.18697128	0.17836216	0.57366954	0.321106868	-1.63887457	down	-	-
MW0144943	[hydroxy-[(2R,3R,4S,5R,6R)-3,4,5-trihydroxy-6-(hydroxymethyl)tetrahydropyran-2-yl]oxy-phosphoryl] [(2Z,6Z,10Z,14Z,18Z,22Z,26Z,30Z,34E,38E)-3,7,11,15,19,23,27,31,35,39,43-undecamethyltetratetraconta-2,6,10,14,18,22,26,30,34,38,42-undecaenyl] hydrogen phosphate	Alcohol and amines	Alcohols	C61H102O12P2	3	0.6596	-	157.19	916.01	189.02	58.15	30	117.33	1.35263016	0.29053311	0.66032961	6.142787619	2.618893504	up	C04507	-
MW0165609	(1E,4Z,6E)-5-hydroxy-1-(4-hydroxy-3-methoxyphenyl)-7-[3-methoxy-4-[(2S,3R,4S,5S,6R)-3,4,5-trihydroxy-6-[[(2R,3R,4S,5S,6R)-3,4,5-trihydroxy-6-[[(2R,3R,4S,5S,6R)-3,4,5-trihydroxy-6-(hydroxymethyl)oxan-2-yl]oxymethyl]oxan-2-yl]oxymethyl]oxan-2-yl]oxyphenyl]hepta-1,4,6-trien-3-one	Others	Ketone compounds	C39H50O21	3	0.6593	-	167.64	124.62	156.85	11.68	33.75	173.13	1.11538371	0.26477157	0.64511688	2.054859078	1.039039457	up	-	-
MW0003312	2-Oxo-3-(3,4,5-trihydroxyphenyl)propanoic acid	Benzene and substituted derivatives	Benzene and substituted derivatives	C9H8O6	3	0.6587	-	175.61	123.37	159.5	53.71	42.76	43.17	1.71982187	0.01645866	0.29532304	3.283299914	1.715146539	up	-	-
MW0128077	(3,4,5,6-Tetrahydroxyoxan-2-yl)methyl 3-(4-hydroxy-3-methoxyphenyl)prop-2-enoate	Others	Lactones	C16H20O9	3	0.6585	-	237.79	167.16	47.34	690.28	215.73	472.83	1.25316177	0.14043968	0.53210743	0.328022106	-1.60813505	down	-	-
MW0130999	3-(2,4-Dihydroxyphenyl)-5-hydroxy-6-(2-hydroxy-3-methylbut-3-enyl)-8,8-dimethylpyrano[2,3-b]chromen-4-one	Others	Ketone compounds	C25H24O7	3	0.6569	-	313.14	1268.74	870.72	210.19	572.33	241.89	1.09617377	0.22182165	0.61215041	2.394158589	1.259518719	up	-	-
MW0014950	6-[(2Z)-2-carboxy-2-(phenylmethylidene)ethoxy]-3,4,5-trihydroxyoxane-2-carboxylic acid	Organic acids	Organic acids	C16H18O9	3	0.6567	-	35151.33	13664.62	35653.91	90586.91	125659.08	38050.7	1.3211149	0.14795052	0.53947123	0.332170505	-1.59000412	down	-	-
MW0157627	Thr-Glu-Gln-Asp	Amino acids and derivatives	Amino acids and derivatives	C18H29N5O11	3	0.6547	-	34879.65	40238.37	44817.78	52641.51	131639.56	100942.39	1.44065368	0.13672688	0.5286681	0.420497669	-1.24983029	down	-	-
MW0156621	Ser-Ala-Asn-Asp	Amino acids and derivatives	Amino acids and derivatives	C14H23N5O9	3	0.6545	-	3926.88	34828.92	115599.09	2210.01	11618.32	1870.2	1.13695603	0.29902291	0.66470991	9.832442273	3.297549811	up	-	-
MW0112622	3,4,5-trihydroxy-6-{[4-hydroxy-5-(3-hydroxy-4-methoxyphenyl)pentanoyl]oxy}oxane-2-carboxylic acid	Organic acids	Organic acids	C18H24O11	3	0.6528	-	419.2	540.24	330.84	207.13	142.38	175.88	1.63571188	0.04232976	0.39256349	2.455851843	1.296223528	up	-	-
MEDL02723	Resveratrol-3-O-sulLipidste	Others	Stilbene	C14H12O6S	3	0.6498	-	164.95	459.91	8493.52	11137.35	11882.25	13899.82	1.31278141	0.06628215	0.44856776	0.246980586	-2.01753045	down	-	-

MW0010817	[(2E)-2,11-dimethyl-7-methylidene-6,12-dioxo-5,14-dioxatricyclo[9.2.1.04,8]tetradeca-1(13),2-dien-9-yl] (E)-4-acetyloxy-2-methylbut-2-enoate	Others	Lactones	C22H24O8	3	0.6488	-	887.13	414.93	1064.44	2022.58	4847.87	10220.72	1.51431078	0.17721598	0.57366954	0.138463312	-2.85242433	down	-	-
MW0113593	6-{[(16R)-5,11-dihydroxy-8,8,12,16-tetramethyl-3-[1-(2-methyl-1,3-thiazol-4-yl)prop-1-en-2-yl]-10-methylidene-9-oxo-17-oxa-4-azabicyclo[14.1.0]heptadec-4-en-7-yl]oxy}-3,4,5-trihydroxyoxane-2-carboxylic acid	Benzene and substituted derivatives	Benzene and substituted derivatives	C33H48N2O11S	3	0.6484	-	1606.91	2110.15	1313.82	482.98	367.75	512.14	1.70390868	0.0306257	0.34952497	3.691386559	1.884162823	up	-	-
MW0128595	[2,6-dihydroxy-4-(3,5,7-trihydroxy-3,4-dihydro-2H-1-benzopyran-2-yl)phenyl]oxidanesulfonic acid	Organic acids	Organic acids	C15H14O10S	3	0.6475	-	7350.42	9259.58	12914.7	8921.27	24290.4	27588.03	1.10971341	0.20573914	0.60068239	0.485606015	-1.0421418	down	-	-
MW0137932	Deoxyschizandrin	Lignans and Coumarins	Lignans	C24H32O6	3	0.6462	-	65.15	555.36	183.4	131.09	40.42	49.63	1.06134309	0.3179844	0.67428155	3.635298906	1.862073992	up	C16951	-
MW0129185	{3-[(E)-2-{3,5-dihydroxy-4-[(1E)-3-methylbut-1-en-1-yl]phenyl}ethenyl]-2,6-dihydroxyphenyl}oxidanesulfonic acid	Organic acids	Organic acids	C19H20O8S	3	0.644	-	411.24	92.5	625.32	62.95	110.91	64.18	1.29358433	0.19390927	0.58775794	4.743152411	2.245846227	up	-	-
MW0104278	1-Hydroxyoct-6-enoylglycine	Amino acids and derivatives	Amino acids and derivatives	C10H17NO4	3	0.6432	-	42.86	39.53	186.98	6.65	23.12	11.72	1.42999642	0.25780629	0.64187626	6.492407809	2.698753623	up	-	-
MW0128247	(5-{8-[1-(2,4-dihydroxyphenyl)-2-hydroxy-3-(3-hydroxyphenyl)propyl]-3,5,7-trihydroxy-3,4-dihydro-2H-1-benzopyran-2-yl}-2-hydroxyphenyl)oxidanesulfonic acid	Organic acids	Organic acids	C30H28O13S	3	0.6426	-	337.53	37.29	53.13	399.94	293.15	382.62	1.26018257	0.14695038	0.53780685	0.39783027	-1.32977504	down	-	-
MW0129336	{5-[(E)-2-{3,5-dihydroxy-4-[(1E)-3-methylbuta-1,3-dien-1-yl]phenyl}ethenyl]-2-hydroxyphenyl}oxidanesulfonic acid	Organic acids	Organic acids	C19H18O7S	3	0.6417	-	5424.82	2638	4383.24	2487.97	1821.61	373.76	1.21983151	0.06915761	0.45765247	2.657517925	1.410079423	up	-	-
MW0148753	dTDP-4-keto-2-deoxy-beta-L-xylose	Nucleotides and derivatives	Nucleotides and derivatives	C15H22N2O14P2	3	0.6376	-	7166.09	6921.46	7623.73	13988.62	21931.79	28740.22	1.63297266	0.07803275	0.46534609	0.335772788	-1.57444278	down	C21353	ko01110
MW0130608	2-{2,6-dihydroxy-3-[3-(2,4,5-trihydroxyphenyl)propanoyl]phenyl}-6-(2,4-dihydroxyphenyl)-4-methylcyclohex-3-ene-1-carboxylic acid	Organic acids	Organic acids	C29H28O10	3	0.6368	-	4568.35	5688.68	3828.64	777.13	1943.29	1001.34	1.6217951	0.00883724	0.23453093	3.784679829	1.920171258	up	-	-
MW0151682	Ile-Nap-OH	Amino acids and derivatives	Amino acids and derivatives	C24H24N2O6	3	0.6363	-	7675.65	6056.2	7420.45	1845.25	1464.46	3619.31	1.59171495	0.0058155	0.20125311	3.052711639	1.610091318	up	-	-
MW0112429	3,4,5-trihydroxy-6-[(3-phenyloxiran-2-yl)methoxy]oxane-2-carboxylic acid	Organic acids	Organic acids	C15H18O8	3	0.6353	-	3584.96	5622.22	3068.5	4480.4	7998.92	13035.82	1.20483039	0.2111802	0.6050101	0.481113566	-1.05555061	down	-	-
MW0129954	2-(1,2-dihydroxypropan-2-yl)-9-hydroxy-2H,3H,7H-furo[3,2-g]chromen-7-one	Others	Ketone compounds	C14H14O6	3	0.6351	-	803.14	576.9	1355.27	3351.22	1743.12	2305.98	1.50348516	0.06168784	0.44168098	0.369620503	-1.43588331	down	-	-
MW0058192	PE(18:0/18:3(9Z,12Z,15Z))	GL	PE	C41H76NO8P	3	0.635	-	145.36	38.34	16.89	153.06	63.94	285.03	1.05151695	0.26700409	0.6487731	0.399557795	-1.32352389	down	-	-
ZINC2731639	Tzfnsitzszgmkw-qkqmvzjrsa-	Others	Lactones	C30H42O13	3	0.6339	-	466.71	271.49	204.32	59.33	166.78	35.56	1.42832346	0.08321375	0.47221936	3.601941377	1.848774701	up	-	-
MW0130505	2-[(1Z)-1-{4-[2-(methylamino)ethoxy]phenyl}-2-phenylbut-1-en-1-yl]phenol	Benzene and substituted derivatives	Benzene and substituted derivatives	C25H27NO2	3	0.6339	-	262.98	156.6	276.77	112.64	41.2	16.75	1.44322765	0.0240037	0.33093994	4.082009496	2.029279539	up	-	-

MW0145313	Arg-Cys-His	Amino acids and derivatives	Amino acids and derivatives	C15H26N8O4S1	3	0.6321	-	526.74	2544.18	456.26	8518.51	30347.83	24694.77	1.61118181	0.09071886	0.48325238	0.055492738	-4.17155721	down	-	-
MW0104507	2-amino-4-({1-[(carboxymethyl)-C-hydroxycarbonimidoyl]-2-({2,3,4,6-tetrahydroxy-5-[(2E)-3-(4-hydroxyphenyl)prop-2-enoyl]phenyl}sulLipidsnyl)ethyl}-C-hydroxycarbonimidoyl)butanoic acid	Organic acids	Organic acids	C25H27N3O12S	3	0.6298	-	202.72	289.35	15.05	12.28	43.14	22.63	1.02579952	0.21813988	0.60939029	6.497373479	2.699856636	up	-	-
MW0106919	Glu-Phe-Lys	Amino acids and derivatives	Amino acids and derivatives	C20H30N4O6	3	0.628	-	57233.79	63074.92	65123.58	25837.85	21228.48	21221.62	1.74174848	0.00039024	0.09608544	2.715446722	1.441189558	up	-	-
MW0110372	Val-Trp-Gln	Amino acids and derivatives	Amino acids and derivatives	C21H29N5O5	3	0.6263	-	3887.14	4398.89	4125.28	1000.3	2307.65	2253.52	1.48619077	0.02346263	0.33077008	2.231659975	1.158117229	up	-	-
MW0113730	6-{5-[(1E)-3-[(6-{[3,4-dihydroxy-2,5-bis(hydroxymethyl)oxolan-2-yl]oxy}-3,4,5-trihydroxyoxan-2-yl)methoxy]-3-oxoprop-1-en-1-yl]-2-hydroxyphenoxy}-3,4,5-trihydroxyoxane-2-carboxylic acid	Organic acids	Organic acids	C27H36O20	3	0.6258	-	5002099.7	4844004.97	5861171.69	5707.47	6184620.22	8874.06	1.21997123	0.26214993	0.64368675	2.533757892	1.341278677	up	-	-
MW0150880	His-Asp-Tyr-Lys	Amino acids and derivatives	Amino acids and derivatives	C25H35N7O8	3	0.6258	-	149429.5	153250.19	140524.8	66999.67	51970.95	65910.07	1.72885408	0.00021394	0.06772687	2.397245975	1.261377948	up	-	-
MW0105462	Ala-Glu-Arg	Amino acids and derivatives	Amino acids and derivatives	C14H26N6O6	3	0.6256	-	23187.9	34790.71	32807.82	9380.27	14227.88	8577.29	1.64592657	0.01746055	0.29823307	2.820729808	1.49606848	up	-	-
MW0006824	Difenacoum	Benzene and substituted derivatives	Benzene and substituted derivatives	C31H24O3	3	0.6255	-	5875.01	19452.97	37938.25	720.94	1325.95	1505.66	1.61281222	0.16537976	0.56381776	17.80868109	4.154508769	up	C16807	-
MW0136151	6-{2-[(E)-2-{4-[6-carboxy-5-(2,4-dihydroxyphenyl)-3-methylcyclohex-2-en-1-yl]-3,5-dihydroxyphenyl}ethenyl]-5-hydroxyphenoxy}-3,4,5-trihydroxyoxane-2-carboxylic acid	Organic acids	Organic acids	C34H34O14	3	0.6231	-	1371.34	227.06	1459.97	2025.03	3393.78	1394.25	1.07605143	0.16338985	0.56041959	0.448898146	-1.15553996	down	-	-
MW0000264	CID 10044441	Alkaloids	Alkaloids	C23H30N2O2	3	0.621	-	114.44	111.48	486.49	38.9	81.97	8.89	1.30478967	0.2573212	0.64187626	5.4902127	2.456862043	up	C20438	ko01100,ko01110
MW0132082	3,4,5-trihydroxy-6-{[3-hydroxy-2-(3-methoxyphenyl)-5-sulfino-3,4-dihydro-2H-1-benzopyran-7-yl]oxy}oxane-2-carboxylate	Organic acids	Organic acids	C22H24O12S	3	0.6192	-	761.39	973.24	1657.36	6447.08	19335.46	6385.23	1.63396175	0.15527837	0.55011491	0.105446849	-3.2454121	down	-	-
MW0135312	6-[(6-carboxy-3,4,5-trihydroxyoxan-2-yl)oxy]-2H-1,3-benzodioxole-5-carboxylic acid	Organic acids	Organic acids	C14H14O11	3	0.6187	-	91.92	52.75	146.55	320.28	126.39	463.57	1.31545473	0.16158339	0.55635821	0.319937599	-1.64413755	down	-	-
MW0107248	His-Asp-Lys	Amino acids and derivatives	Amino acids and derivatives	C16H26N6O6	3	0.6187	-	302.32	152.47	205.74	512.92	711.04	697.07	1.63211966	0.00787187	0.22054168	0.343841585	-1.54018406	down	-	-
MW0153022	Lys-Ile-Val-Lys	Amino acids and derivatives	Amino acids and derivatives	C23H46N6O5	3	0.6161	-	178.17	438.93	281.7	4.86	4.86	59.11	1.55048853	0.06055067	0.43825617	13.05825948	3.706890709	up	-	-
MW0130777	2-[illegible]	Flavonoids	Isoflavones	C15H12O5	3	0.6128	-	25208.18	22243.61	256224.94	261939.01	874499.75	394549.74	1.36829683	0.14644729	0.53729669	0.198353371	-2.33385518	down	C03567	ko00943,ko01110
MW0135792	6-[2-(3,4-Dihydroxyphenyl)-4-oxo-6,8-bis[3,4,5-trihydroxy-6-(hydroxymethyl)oxan-2-yl]-7-(3,4,5-trihydroxy-6-methyloxan-2-yl)oxychromen-5-yl]oxy-3,4,5-trihydroxyoxane-2-[illegible]	Organic acids	Organic acids	C39H48O26	3	0.6124	-	661.33	116.85	1442.48	1775.36	3138.16	2416.8	1.27818355	0.0364407	0.37270445	0.302941754	-1.72288766	down	-	-
MW0132300	3,4,5-trihydroxy-6-{2,3,5-trihydroxy-4-[3-(4-methoxyphenyl)propanoyl]phenoxy}oxane-2-carboxylic acid	Organic acids	Organic acids	C22H24O12	3	0.6105	-	464.17	230.03	191.81	740.24	504.12	1280.99	1.42653052	0.1282294	0.51982213	0.350846417	-1.51108846	down	-	-

MW0002559	2-[4,6-dihydroxy-2-methoxy-3-(3-methylbut-2-en-1-yl)phenyl]acetic acid	Benzene and substituted derivatives	Benzene and substituted derivatives	C14H18O5	3	0.6065	-	78.46	102.41	78.02	9.48	27.2	2.53	1.50671467	0.00263749	0.18110516	6.602652385	2.723045693	up	-	-
MW0113604	6-{[(1S,16R)-5,11-dihydroxy-8,8,10,12,16-pentamethyl-3-[1-(2-methyl-1,3-thiazol-4-yl)prop-1-en-2-yl]-9-oxo-17-oxa-4-azabicyclo[14.1.0]heptadec-4-en-7-yl]oxy}-3,4,5-trihydroxyoxane-2-carboxylic acid	Benzene and substituted derivatives	Benzene and substituted derivatives	C33H50N2O11S	3	0.603	-	213.4	165.91	61.38	24.08	14.78	56.17	1.44442024	0.1152375	0.50205214	4.63737767	2.213309225	up	-	-
MW0156492	S-Adenosylhomocysteine-d4	Nucleotides and derivatives	Nucleotides and derivatives	C14H16D4N6O5S	3	0.6012	-	69008.89	87688.55	101802.78	19187.47	30352.72	29975.11	1.68000783	0.01486203	0.27628422	3.25094944	1.700861119	up	-	-
MW0109906	Thr-Val-Arg	Amino acids and derivatives	Amino acids and derivatives	C15H30N6O5	3	0.6007	-	3354.65	2521.51	3790.35	18960.41	3825.31	8653.84	1.26937712	0.24490495	0.63271123	0.307463272	-1.70151401	down	-	-
MW0113343	6-(3-carboxy-5-methoxyphenoxy)-3,4,5-trihydroxyoxane-2-carboxylic acid	Organic acids	Organic acids	C14H16O10	3	0.5999	-	1421.2	1144.28	851.58	433.64	421.97	405.17	1.68274697	0.04824363	0.40294196	2.710274592	1.438439026	up	-	-
ZINC9591021	Cowagarcinone E	Others	Lactones	C31H36O8	3	0.5996	-	720.81	600.04	66.68	43.25	11.82	48.34	1.405229	0.16650581	0.56418482	13.41775457	3.746071355	up	-	-
MW0051045	DG(22:5(4Z,7Z,10Z,13Z,16Z)/20:3(8Z,11Z,14Z)/0:0)	GL	DG	C45H72O5	3	0.5978	-	56.39	173.64	96.63	22.74	64.72	55.98	1.16960754	0.21073698	0.6048377	2.2773285	1.187342412	up	-	-
MW0130137	2-(3,4-dihydroxyphenyl)-4-[3,5,7-trihydroxy-2-(4-hydroxy-3-methoxyphenyl)-3,4-dihydro-2H-1-benzopyran-8-yl]-6-[3,4,5-trihydroxy-6-(hydroxymethyl)oxan-2-yl]-3,4-dihydro-2H-1-	Benzene and substituted derivatives	Benzene and substituted derivatives	C37H38O17	3	0.5969	-	49.15	89.56	75.86	20.93	37.87	26.06	1.55448004	0.05143113	0.41145636	2.528517558	1.338291797	up	-	-
MW0058215	1-Vaccenoyl-2-myristoyl-sn-glycero-3-phosphoethanolamine	GL	PE	C37H72NO8P	3	0.5961	-	603.22	30313.4	256.22	27158.49	29936.04	19702.06	1.1890907	0.26290197	0.64368675	0.405914377	-1.30075265	down	-	-
MW0058238	PE(18:1(11Z)/22:4(7Z,10Z,13Z,16Z))	GL	PE	C45H80NO8P	3	0.5942	-	1144.54	142.29	415.08	1780.54	1014.01	1246.13	1.20875859	0.11153928	0.50205214	0.421193957	-1.24744336	down	-	-
MW0106910	Glu-Ile-Val	Amino acids and derivatives	Amino acids and derivatives	C16H29N3O6	3	0.5923	-	28.35	748.94	26.48	348.86	683.1	1085.61	1.16540112	0.24548606	0.63275786	0.379571868	-1.39755502	down	-	-
MW0151688	Isoleucylthreonine	Amino acids and derivatives	Amino acids and derivatives	C10H20N2O4	3	0.5913	-	29087.85	29055.48	55895.24	12478.52	16000.62	11356.17	1.59103847	0.10624325	0.4992359	2.862750911	1.517402146	up	-	-
MW0149387	Gal1-3GlcNAcSp	Others	Saccharides	C16H28N4O11	3	0.5911	-	100.04	421.3	204.15	343.44	890.02	825.25	1.29517284	0.10597793	0.49914367	0.35240029	-1.50471299	down	-	-
MW0000462	1-(1,2,3,4,5-Pentahydroxypent-1-yl)-1,2,3,4-tetrahydro-beta-carboline-3-carboxylate	Alkaloids	Alkaloids	C17H22N2O7	3	0.5884	-	341.84	549.56	778.35	193.4	150.36	159.57	1.61690053	0.08945048	0.48112197	3.317406076	1.73005562	up	-	-
MW0058067	PE(14:1(9Z)/20:5(5Z,8Z,11Z,14Z,17Z))	GL	PE	C39H66NO8P	3	0.5861	-	2891.56	4140.83	2195.21	6478.14	10303.13	8455.18	1.60799756	0.02333072	0.33077008	0.365645723	-1.45148161	down	-	-
MW0150527	Gly-Val-Ala-Asp-Val	Amino acids and derivatives	Amino acids and derivatives	C19H33N5O8	3	0.586	-	6619.98	6313.97	7185.29	30810.3	42430.07	37828.18	1.74919276	0.01183283	0.25665182	0.181142547	-2.46480265	down	-	-
MW0107762	Leu-Thr-Ser	Amino acids and derivatives	Amino acids and derivatives	C13H25N3O6	3	0.5848	-	1407.73	1478.97	1790.67	4734.99	5498.17	1338.54	1.11787701	0.21324573	0.60587521	0.404207679	-1.30683136	down	-	-
MW0151683	Ile-Phe	Amino acids and derivatives	Amino acids and derivatives	C15H22N2O3	3	0.5806	-	37767.07	50722.65	46618.3	77161.51	167684.96	49795.45	1.17818822	0.27333369	0.65054534	0.45854989	-1.12484939	down	-	-
MW0057093	PC(18:3(6Z,9Z,12Z)/20:1(11Z))	GL	PC	C46H84NO8P	3	0.5682	-	59016.68	11248.46	23704.01	3864.53	6719.95	19116.98	1.14493367	0.26981396	0.64977045	3.163788918	1.661653349	up	-	-
MW0113634	6-{[2-(3,4-dihydroxyphenyl)-7-hydroxy-3-[(3,4,5-trihydroxyoxan-2-yl)oxy]-8aH-chromen-5-yl]oxy}-3,4,5-trihydroxyoxane-2-carboxylic acid	Organic acids	Organic acids	C26H27O16	3	0.567	-	31240.38	15558.92	37942.87	18115.88	19438.67	2237.21	1.0003434	0.15998949	0.5530994	2.129641162	1.090610361	up	-	-
MW0113275	6-[(10-Butanoyl-3-hydroxy-2,2-dimethyl-8-oxo-6-propyl-3,4-dihydropyrano[3,2-g]chromen-5-yl)oxy]-3,4,5-trihydroxyoxane-2-carboxylic acid	Organic acids	Organic acids	C27H34O12	3	0.5666	-	14257.22	8890.45	4957.4	21394.39	25981.34	10974.37	1.21118327	0.13921517	0.53143981	0.481662756	-1.05390472	down	-	-

MW0113588	6-{[(16R)-5,11-dihydroxy-12-(hydroxymethyl)-8,8,10,16-tetramethyl-3-[1-(2-methyl-1,3-thiazol-4-yl)prop-1-en-2-yl]-9-oxo-17-oxa-4-azabicyclo[14.1.0]heptadec-4-en-7-yl]oxy}-3,4,5-trihydroxyoxane-2-carboxylic acid	Benzene and substituted derivatives	Benzene and substituted derivatives	C33H50N2O12S	3	0.5619	-	10.61	8.61	64.49	152.48	44.24	72.15	1.24723397	0.19161776	0.58407835	0.311340053	-1.68343691	down	-	-
MW0151819	Isodonal	Others	Lactones	C22H28O7	3	0.5577	-	310.63	465.8	297.14	1190.45	780.64	359.82	1.18039454	0.21868216	0.60955933	0.460579774	-1.11847704	down	C09117	-
MW0128739	[4-(3,5,7-trihydroxy-3,4-dihydro-2H-1-benzopyran-2-yl)phenyl]oxidanesulfonic acid	Organic acids	Organic acids	C15H14O8S	3	0.5439	-	699.94	146.21	184.1	843.14	733.84	623.76	1.25963734	0.14983055	0.54119721	0.46813799	-1.09499425	down	-	-
MW0135379	6-[2-(6-carboxy-5-{3-[3-(2,4-dihydroxyphenyl)propanoyl]-2,6-dihydroxyphenyl}-3-methylcyclohex-3-en-1-yl)-5-hydroxyphenoxy]-3,4,5-trihydroxyoxane-2-carboxylic acid	Organic acids	Organic acids	C35H36O15	3	0.5427	-	106.03	281.29	103.49	97.83	41	93.2	1.11300975	0.27757435	0.65377045	2.115286816	1.080853295	up	-	-
MW0145866	Asn-Phe-Trp	Amino acids and derivatives	Amino acids and derivatives	C24H27N5O5	3	0.5353	-	4238.3	7587.63	7971.01	8410.65	17730.53	15547.9	1.34886836	0.10678694	0.49924019	0.474871117	-1.07439208	down	-	-
ZINC9591035	(1aR,2R,8aS)-1,1aalpha,2,5,5a,6,10,10aalpha-Octahydro-5beta,5abeta-dihydroxy-4-hydroxymethyl-1,1,7,9alpha-tetramethyl-6beta-[[(2E,4Z)-1-oxodeca-2,4-dienyl]oxy]-9H-2,8aalpha-methanocyclopenta[a]cyclopropa[e]cyclodecen-11-one	Others	Lactones	C30H42O6	3	0.5315	-	387.98	1195.78	446.24	51.4	215.42	40.79	1.50094751	0.15266184	0.54704545	6.599265303	2.722305418	up	-	-
MW0145898	Asn-Thr-Asp-Arg	Amino acids and derivatives	Amino acids and derivatives	C18H32N8O9	3	0.5308	-	2.34	20.14	7.41	8.16	38.46	36.81	1.02936274	0.20638855	0.60099477	0.358264413	-1.48090335	down	-	-
MW0146176	Asp-Leu-Phe-Arg	Amino acids and derivatives	Amino acids and derivatives	C25H39N7O7	3	0.5266	-	2246.95	3598.24	13056.63	7167.09	12062.92	32070.13	1.09365847	0.2929148	0.66139816	0.368455525	-1.44043761	down	-	-
MW0051082	DG(22:5(7Z,10Z,13Z,16Z,19Z)/22:5(4Z,7Z,10Z,13Z,16Z)/0:0)	GL	DG	C47H72O5	3	0.525	-	1881	1416.51	2433.65	1372.93	153.51	532.37	1.26608774	0.06044298	0.43825617	2.783724579	1.477016478	up	-	-
MW0105834	Asn-Ser	Amino acids and derivatives	Amino acids and derivatives	C7H13N3O5	3	0.5246	-	487.84	163.15	584.58	68.61	66.3	328.23	1.15241581	0.17987248	0.57366954	2.667811029	1.415656479	up	-	-
MW0012508	16(17)-EpDPE	Lipids	Free Lipidstty acids	C22H32O3	3	0.523	-	1167.23	439.02	1274.47	1116.13	3156.13	1717	1.11498288	0.22317637	0.61283622	0.480980956	-1.05594832	down	-	-
MW0149497	GDP-4-oxo-L-rhamnose	Nucleotides and derivatives	Nucleotides and derivatives	C16H23N5O15P2	3	0.5221	-	14311.63	15059.81	13094.91	57553.93	73933.63	34136.65	1.66177103	0.07047518	0.45866819	0.256401827	-1.96352155	down	C05344	-
MW0132484	3,4,5-trihydroxy-6-{4-[3-(5-hydroxy-2,2-dimethyl-2H-chromen-6-yl)prop-2-enoyl]phenoxy}oxane-2-carboxylic acid	Benzene and substituted derivatives	Benzene and substituted derivatives	C26H26O10	3	0.5221	-	149320.98	117826.22	165694.74	69228.2	85619.43	48771.78	1.57416441	0.01434247	0.27281471	2.125740076	1.087965202	up	-	-
MW0158713	TyrMe-Thr-OH	Amino acids and derivatives	Amino acids and derivatives	C20H22N2O8	3	0.5176	-	25.01	131.67	423.79	531.92	201.1	1551.16	1.08633747	0.29506259	0.66146556	0.254126207	-1.97638293	down	-	-
MW0109010	Homoserine, O-succinyl-	Amino acids and derivatives	Amino acids and derivatives	C8H13NO6	3	0.517	-	497678.94	406357.8	4896745.47	5538593.92	13550042.6	6744858.84	1.3677417	0.09788893	0.48888534	0.224545	-2.1549235	down	-	-
MW0058107	PE(15:0/22:6(4Z,7Z,10Z,13Z,16Z,19Z))	GL	PE	C42H72NO8P	3	0.5151	-	2789.91	2128.78	4124.56	2742.36	9142.28	8301.37	1.12227753	0.19919056	0.59377853	0.447995914	-1.15844252	down	-	-
MW0104594	2-amino-4-({1-[(carboxymethyl)-C-hydroxycarbonimidoyl]-2-{[1,3-dihydroxy-4-(7-methoxy-2-oxo-2H-chromen-8-yl)-2-methylbutan-2-yl]sulLipidsnyl}ethyl}-C-hydroxycarbonimidoyl)butanoic acid	Organic acids	Organic acids	C25H33N3O11S	3	0.5125	-	16859.69	18604.97	53730.06	64138.93	72349.49	77345.16	1.41771897	0.06289531	0.44396349	0.417122138	-1.26145821	down	-	-

MW0128214	(4-{8-[4,5-dihydroxy-6-(hydroxymethyl)-3-[(3,4,5-trihydroxy-6-methyloxan-2-yl)oxy]oxan-2-yl]-5,7-dihydroxy-4-oxo-3,4-dihydro-2H-1-benzopyran-2-yl}phenyl)oxidanesulfonic acid	Organic acids	Organic acids	C27H32O17S	3	0.5118	-	163.72	971.01	72.63	18.42	16.42	44.69	1.42071724	0.31850143	0.67428155	15.18118949	3.924212929	up	-	-
MW0107728	Leu-Ile-Arg	Amino acids and derivatives	Amino acids and derivatives	C18H36N6O4	3	0.5066	-	6.85	15.33	13.85	331.79	236.43	278.32	1.73569013	0.00977226	0.24036471	0.042561486	-4.55430768	down	-	-
MW0103098	Vinaginsenoside R12	Terpenoids	Triterpene Saponin	C36H64O11	3	0.5035	-	563.3	844.72	591.39	2833.03	2658.29	740.92	1.29985675	0.1677704	0.56577827	0.320817234	-1.64017645	down	-	-
MW0165428	(E)-1-(glutathion-S-yl)-N-hydroxy-omega-(methylsulLipidsnyl)heptan-1-imine	Others	Lactones	C18H31N4O7S2-	3	0.5015	-	148.33	1419.86	1021.81	1777.34	2261.67	3443.97	1.20815042	0.06281759	0.44396349	0.346118792	-1.53066082	down	-	-

Index	Compounds	Class I	Class II	Formula	Level	score	CAS	yfm-55DPA-1	yfm-55DPA-2	55 DPA yfm-55DPA-3	H88119-55DPA	H88119-55DPA	H88119-55DPA	VIP	P-value	FDR	Fold_Change	Log2FC	Type	cpd_ID	kegg_map
MW0127974	N-Feruloyl dopamine, trans-	Alkaloids	Alkaloids	C18H19NO5	2	0.9047	142350-99-0	47399.18	30522.96	48013.02	69577.95	156083.27	57343.31	1.19601389	0.23146372	0.51669106	0.444993442	-1.16814402	down	-	-
MW0126804	3',5'-Cyclic dtmp	Organic acids	Organic acids	C10H13N2O7P	2	0.9025	6453-60-7	40000.94	8969.36	32528.32	7023.46	4437.16	2665.32	1.360556	0.13587509	0.4126664	5.769429857	2.528428757	up	-	-
MW0138125	(E)-Ethyl 3-(4-hydroxyphenyl)acrylate	Others	Lactones	C11H12O3	2	0.8763	7362-39-2 | 2979-06-8	28182.98	33902.87	37134.68	16405.28	10578.88	16309.41	1.51992685	0.00589967	0.13106004	2.291807536	1.196485893	up	-	-
MW0006843	Diisodecyl phthalate	Benzene and substituted derivatives	Benzene and substituted derivatives	C28H46O4	2	0.8421	26761-40-0 | 89-16-7 | 68515-49-1 | 19269-67-1	39603.22	14956.74	29825.58	6831.08	3490.13	7026.76	1.47441029	0.08572397	0.33324725	4.864289021	2.282228952	up	-	-
MW0144895	Allopurinol riboside	Nucleotides and derivatives	Nucleotides and derivatives	C10H12N4O5	2	0.8279	16220-07-8	56.68	428.49	451.53	2019.02	454.88	653.18	1.0299059	0.27312209	0.55388084	0.299544623	-1.73915716	down	-	-
MW0112142	2'-Fucosyl-D-lactose	Others	Saccharides	C18H32O15	2	0.8101	41263-94-9	2726.98	842.31	1974.14	3040.07	20348.93	10803.83	1.28129194	0.19536012	0.48243539	0.162122585	-2.62484301	down	-	-
MW0132689	Dihydrokaempferol	Benzene and substituted derivatives	Benzene and substituted derivatives	C15H12O6	2	0.8075	104486-98-8 | 5150-32-3 | 724434-08-6	149.15	85.09	88.87	5.38	51.52	14.85	1.32141041	0.03495006	0.22565754	4.503275261	2.170974665	up	-	-
MW0139542	Vincetoxicoside A	Flavonoids	Flavones	C27H30O16	2	0.7959	18016-58-5 | 163559-06-6	3120.49	3304.93	3297.84	2390.73	163.59	210.54	1.24346142	0.08640117	0.33497534	3.516727791	1.814233669	up	-	-
MW0061609	Phomenone	Terpenoids	Sesquiterpenoids	C15H20O4	2	0.7894	55785-58-5	85809.21	78400.48	81956.49	178953.4	199565.35	180449.97	1.62425384	0.00195226	0.08476296	0.44039348	-1.18313498	down	C09708	-
MW0011719	Heudelottin C	Others	Lactones	C39H54O11	2	0.7847	11052-28-1	3086.49	2264.53	2143.64	18	310.27	936.06	1.21899817	0.0067751	0.13456926	5.927772022	2.567489964	up	-	-
MW0005999	alpha-Hydroxyhippuric acid	Benzene and substituted derivatives	Benzene and substituted derivatives	C9H9NO4	2	0.781	16555-77-4	1944.91	1152.51	1286.89	619.88	238.47	15.59	1.1903081	0.02137915	0.18323322	5.016717395	2.326743669	up	-	-
MW0139541	3-[(2S,3R,4S,5S,6R)-4,5-dihydroxy-6-(hydroxymethyl)-3-[(2S,3R,4R,5R,6S)-3,4,5-trihydroxy-6-methyloxan-2-yl]oxyoxan-2-yl]oxy-2-(3,4-dihydroxyphenyl)-5-hydroxy-7-[(2S,3R,4R,5R,6S)-3,4,5-trihydroxy-6-methyloxan-2-	Flavonoids	Flavones	C33H40O20	2	0.7809	161993-01-7	1071.37	835.84	541.42	5051.53	3181.02	1209.77	1.30277752	0.1680759	0.45767138	0.259325039	-1.94716658	down	-	-
MW0127920	2,5-Dihydroxycinnamic acid	Organic acids	Organic acids	C9H8O4	2	0.7791	636-01-1 | 38489-67-7	22552.59	19228.62	52121.45	9273.9	9270.72	18135.11	1.24089668	0.20370175	0.49044405	2.560069553	1.356183007	up	-	-
MW0106444	Dihydrofolic acid	Organic acids	Organic acids	C19H21N7O6	2	0.7769	4033-27-6	170413.89	171990.43	193718.76	19784.33	22884.68	45516.08	1.57374756	0.00018036	0.04188261	6.079520699	2.603957588	up	C00415	ko00670,ko00790,ko01100,ko01240
MW0000973	Gingerol	Phenolic acids	Phenolic acids	C17H26O4	2	0.7769	23513-14-6 | 1391-73-7	1573.14	1431.96	1065.61	4325.16	5184.56	6682.75	1.58447041	0.02352746	0.18873301	0.251395247	-1.99197072	down	C10462	ko00945,ko01110
MW0009430	Phenylpropiolic acid	Benzene and substituted derivatives	Benzene and substituted derivatives	C9H6O2	2	0.7766	637-44-5	337597.78	288847.41	357931.65	121896.93	5292.11	1210.27	1.29826581	0.00770553	0.13950182	7.666527491	2.938573265	up	-	-
MW0114815	Melilotoside	Benzene and substituted derivatives	Benzene and substituted derivatives	C15H18O8	2	0.7723	618-67-7	88557.82	101940.33	116126.95	6571.21	6451	6761.11	1.63051373	0.00685335	0.13456926	15.49917304	3.954119337	up	C05158	ko00999,ko01110
MW0112865	Isovalerophenone	Others	Ketone compounds	C11H14O	2	0.7714	582-62-7	8619.68	6555.17	6866.71	1770.79	2340.71	4830.87	1.38210477	0.02325672	0.1872421	2.464845449	1.30149719	up	-	-
MW0113918	alpha-Pyrrolidinopropiopheno	Others	Ketone compounds	C13H17NO	2	0.7703	19134-50-0	430.85	333.95	391.52	46.36	70.05	64.25	1.61311246	0.00504815	0.12160208	6.400531385	2.678191686	up	-	-
FDATN00282	Neohesperidin dihydrochalcone	Flavonoids	Chalcones	C28H36O15	2	0.7685	20702-77-6	207490.56	254698.21	257723.76	90785.39	146511.77	121625.2	1.49543465	0.0062761	0.13374656	2.005761162	1.004149826	up	-	-
MW0009147	Norcocaine	Benzene and substituted derivatives	Benzene and substituted derivatives	C16H19NO4	2	0.7671	18717-72-1	19189.69	17125.16	34703.12	3479.23	9561.29	1142.69	1.34651484	0.05831059	0.28147299	5.007185961	2.324000037	up	-	-

MW0138536	Isorhoifolin	Heterocyclic compounds	Heterocyclic compounds	C27H30O14	2	0.7668	552-57-8	95077.68	60326.44	95613.56	28421.19	44229.54	40654.58	1.4501078	0.04372153	0.24666834	2.215409675	1.147573508	up	-	-
MW0007335	Phenylacetaldehyde	Phenolic acids	Phenolic acids	C8H8O	2	0.7661	122-78-1	1433.68	3298.7	1902.2	511.89	321.31	1805.36	1.15441698	0.14395427	0.42625922	2.514470014	1.330254349	up	C00601	ko00360,ko01100
MW0107365	His-Val	Amino acids and derivatives	Amino acids and derivatives	C11H18N4O3	2	0.7641	76019-15-3	935.72	232.26	575.81	32.37	465.85	128.05	1.01650123	0.21007125	0.4976598	2.784406087	1.477369634	up	-	-
MW0107972	Tyr-Gly-Gly	Amino acids and derivatives	Amino acids and derivatives	C13H17N3O5	2	0.7606	21778-69-8	107397.7	73601.28	81732.97	22698.89	25683.13	393.66	1.13076355	0.00620974	0.13374656	5.386535872	2.429357761	up	-	-
MW0053831	Hecogenin	Terpenoids	Steroidal [illegible]	C27H42O4	2	0.7592	467-55-0	222.36	115.01	291.31	5.47	34.54	81.43	1.26357919	0.06403325	0.29035184	5.17687747	2.372082172	up	C08902	-
MW0010784	(+)-Isomenthone	Others	Ketone compounds	C10H18O	2	0.7547	1196-31-2 | 491-07-6	49.83	32.95	40.51	74.06	180.45	155.55	1.4478379	0.09362269	0.3476964	0.300663318	-1.73377923	down	-	-
MW0007840	Monoethyl phthalate	Benzene and substituted derivatives	Benzene and substituted derivatives	C10H10O4	2	0.7513	2306-33-4	11278.94	2567.53	9904.52	287.12	2142.74	3349.06	1.10631841	0.147087	0.43166641	4.109935767	2.039115846	up	-	-
MW0058015	1,2-Dimyristoyl-sn-glycero-3-phosphoethanolamine	GL	PE	C33H66NO8P	2	0.7489	998-07-2	14.2	67.77	53.09	81.32	141.4	209.76	1.23090241	0.10020841	0.35973922	0.312291898	-1.67903295	down	-	-
MW0141122	Dioleoylphosphatidylcholine	GL	PC	C44H84NO8P	2	0.7481	52088-89-8	5016.67	11379.95	12522.81	5378.93	3398.63	2452.81	1.27713978	0.11445459	0.37996512	2.57510928	1.364633657	up	-	-
MW0011852	1,2-Didecanoyl-sn-glycero-3-[illegible]	GL	PC	C28H56NO8P	2	0.7475	3436-44-0	6947.33	2933.27	4410.57	695.23	283.65	686.59	1.53719735	0.06771516	0.29786568	8.58086306	3.101122761	up	-	-
MW0152360	Leu-His-Leu	Amino acids and derivatives	Amino acids and derivatives	C18H31N5O4	2	0.7414	126828-33-9	19526.14	22528.23	33672.22	9089.15	8163.34	7823.4	1.55890814	0.05820126	0.28143735	3.019896402	1.594499059	up	-	-
MW0108317	Met-Ser	Amino acids and derivatives	Amino acids and derivatives	C8H16N2O4S	2	0.7403	14517-43-2	209672.37	154177.69	181869.9	569.1	504.11	30346.26	1.39891254	0.00180632	0.08476296	17.36884677	4.118430062	up	-	-
pme3504	Ononin	Flavonoids	Isoflavones	C22H22O9	2	0.7383	486-62-4	29099.27	21568.67	30256.85	8227.36	13127.37	10389.49	1.53326621	0.01274257	0.15141102	2.549276372	1.350087787	up	C10509	ko00943
MW0116893	1-Benzyl-4-hydroxy-5-oxo-2,5-dihydro-1H-pyrrole-3-carboxylic acid ethyl ester	Benzene and substituted derivatives	Benzene and substituted derivatives	C14H15NO4	2	0.73	4450-98-0	3578.77	4908.35	4302.49	2555	867.56	2576.98	1.2357377	0.03594296	0.22604178	2.131765102	1.092048477	up	-	-
MEDN1677	2'-Adenylic acid	Nucleotides and derivatives	Nucleotides and derivatives	C10H14N5O7P	2	0.7299	130-49-4	3927.08	6133.1	3480.33	892.05	1796.44	1929.06	1.43383938	0.05312878	0.26758468	2.932401382	1.55208259	up	C00946	-
MW0113991	beta-D-Glucosamine	Alcohol and [illegible]	Amines	C6H13NO5	2	0.7298	14257-69-3	476.73	551.37	1151.56	410.29	166.05	204.96	1.30515588	0.15010587	0.43473176	2.789786254	1.480154591	up	C08349	-
MW0052352	Digitoxin	Terpenoids	Steroid	C41H64O13	2	0.7238	71-63-6	107.27	65.28	34.73	140.88	233.22	162.31	1.34063712	0.03853365	0.23094812	0.386420835	-1.37175521	down	C06955	-
MW0049032	Cortisol 21-sulfate	Others	Lactones	C21H30O8S	2	0.7233	1253-43-6	9807.03	26149.43	22173.26	59078.33	34642.24	34033.36	1.24489866	0.08754764	0.33783172	0.455013165	-1.13601981	down	-	-
MW0009814	Sulfometuron-methyl	Benzene and substituted derivatives	Benzene and substituted derivatives	C15H16N4O5S	2	0.7228	74222-97-2	8776.2	12012.6	6605.37	75.15	28.23	11.87	1.60255985	0.02856139	0.20752525	237.693449	7.892958332	up	C10955	-
MW0012963	1-Palmitoyl-sn-glycero-3-phosphoethanolamine	Lipids	LPE	C21H44NO7P	2	0.714	53862-35-4	954.99	578.7	702.62	82.64	339.19	177.99	1.40104126	0.01955581	0.17648958	3.728301824	1.898518658	up	-	-
MW0006870	Dimoxystrobin	Benzene and substituted derivatives	Benzene and substituted derivatives	C19H22N2O3	2	0.7124	149961-52-4	2709.5	2958.56	6178.64	1261.36	118.38	439.74	1.36159432	0.08517958	0.33287184	6.51103612	2.702887142	up	-	-
MW0003591	(3,5-Dimethoxyphenyl)(4-methoxyphenyl)methano[illegible]	Benzene and substituted derivatives	Benzene and substituted derivatives	C16H16O4	2	0.7042	94709-12-3	267.98	676.85	384.55	899.12	4940.22	497.65	1.00393836	0.36032934	0.62418104	0.209780984	-2.25304418	down	-	-
MW0010965	Secalciferol	Alcohol and [illegible]	Alcohols	C27H44O3	2	0.7034	55721-11-4	1172.11	191.91	263.2	6.64	66.49	326.64	1.01542046	0.32364609	0.59517183	4.070390475	2.0251672	up	-	-
MEDN0275	1-Caffeoylquinic acid	Phenolic acids	Phenolic acids	C16H18O9	3	0.9949	1241-87-8 | 928005-87-2	10216.98	11448.67	10464.72	5087.16	5225.26	5247.76	1.6254082	0.00405839	0.11299759	2.064909917	1.046078845	up	-	-
MW0111391	4-O-feruloyl-D-quinic [illegible]	Organic acids	Organic acids	C17H20O9	3	0.9836	76272-56-5	184.92	19.84	33.67	5.79	8.88	18.67	1.19195277	0.32505245	0.59517183	7.151469706	2.838239762	up	-	-
mws0059	Rutin	Flavonoids	Flavonols	C27H30O16	3	0.9765	153-18-4	10042555.17	8368531.44	6034406.88	5411477.7	2827206.6	3963800.36	1.35234334	0.05099173	0.26226703	2.003320977	1.002393592	up	C05625	ko00944,ko01100,ko01110
pmp000596	Quercetin-3-O-(2"-O-galactosyl)glucoside	Flavonoids	Flavonols	C27H30O17	3	0.9728	56782-99-1	155268.04	18627.86	71071.52	16384.96	17218.78	6617.29	1.22103336	0.22784362	0.51357456	6.09053075	2.606567955	up	-	-
MEDL01831	n-cis-Feruloyltyramine	Alkaloids	Phenolamine	C18H19NO4	3	0.9632	80510-09-4	40638.3	14507.61	16411.54	157494.85	485741.17	186679.46	1.5212378	0.13664802	0.41402683	0.086222575	-3.53579054	down	-	-
MEDL00568	Hyperoside	Flavonoids	Flavonols	C21H20O12	3	0.958	482-36-0	473006.45	334688.3	503600.06	103532.07	173080.14	261462.14	1.38270612	0.0209868	0.18177631	2.43701416	1.285114634	up	C10073	-
MEDN0198	Citric acid	Organic acids	Organic acids	C6H8O7	3	0.9411	77-92-9	69649.23	14869.35	71312.52	171736.83	78562.51	90433.7	1.03543186	0.16294167	0.45100293	0.457340738	-1.12865866	down	C00158	ko00020,ko00250,ko00630,ko01100,ko01110,ko01200,ko01210,ko01230,ko01240
MW0124001	Ethyl 4-hydroxy-5,8-dimethoxyquinoline-3-carboxylate	Others	Lactones	C14H15NO5	3	0.94	5428-19-3	96776.18	79783.51	83383.39	283.94	9320.79	11575.76	1.32184605	0.00046938	0.05342558	12.27276045	3.617387879	up	-	-
MEDP1339	LPC(18:1/0:0)	Lipids	LPC	C26H53NO7P	3	0.9376	3542-29-8	154303.41	42801.43	125852.54	46702.48	44422.19	36631.89	1.14376252	0.19024122	0.47658128	2.527912304	1.337946416	up	-	-
MEDN1545	Guanosine-5'-monophosphate	Nucleotides and derivatives	Nucleotides and derivatives	C10H14N5O8P	3	0.9275	85-32-5 | 128952-18-1 | 29593-02-0	10865.5	3657.85	4781.85	18419.98	10931.33	12355.73	1.24769602	0.08042116	0.32462776	0.462876291	-1.11130143	down	C00144	ko00230,ko01100,ko01232
MEDP1310	4-Hydroxy-l-tryptophan	Amino acids and derivatives	Amino acids and derivatives	C11H12N2O3	3	0.9254	25242-90-4	12155.58	14517.11	21155.37	20229.87	31011.16	48596.81	1.23700986	0.16174654	0.44882123	0.47905744	-1.06172945	down	C21777	-
MEDP0151	Isoguanine	Nucleotides and derivatives	Nucleotides and derivatives	C5H5N5O	3	0.9229	3373-53-3	15790.4	3145.42	2380.14	19137.63	26774.9	15401.96	1.23257882	0.07616454	0.31624084	0.347649634	-1.52429402	down	-	-
MEDL00902	LPC(0:0/14:0)	Lipids	LPC	C22H46NO7P	3	0.9024	20559-16-4	162997.26	115885.57	147869.66	29177.24	22536.26	31168.78	1.60893631	0.01212209	0.14858307	5.148899017	2.364263975	up	-	-
MW0108521	N-[(R)-4-phosphopantothenoyl]-L-cysteine	Amino acids and derivatives	Amino acids and derivatives	C12H23N2O9PS	3	0.8991	7196-09-0	21743.3	19737.62	23882.99	3729.03	571.85	2107.14	1.49920311	0.00029665	0.05012965	10.20032865	3.350543731	up	C04352	ko00770,ko01100,ko01240
MEDL01744	Heterophylliin A	Tannins	Tannin	C34H26O22	3	0.8984	87687-52-3	2325.53	1176.33	3491.16	4952.03	20126.04	11195.3	1.37017962	0.15414622	0.43914832	0.192786609	-2.37492325	down	-	-

MW0015017	6-Geranylgeranyl 8'-methyl 6,8'-diapocarotene-6,8'-[illegible]	Others	Lactones	C43H60O4	3	0.8907	247030-33-7	9318.59	12025.04	12430.56	206.05	639.25	623.96	1.58926703	0.00716601	0.13569	22.98721125	4.522759547	up	-	-
MW0011215	(3beta,5alpha,6alpha,7alpha,22E,24R)-5,6-Epoxyergosta-8,14,22-triene-3,7-diol	Alcohol and amines	Alcohols	C28H42O3	3	0.8775	276864-98-3	9190.48	5570.39	8582.88	750.34	674.44	1594.46	1.56491333	0.02052477	0.18074908	7.73166426	2.950778991	up	-	-
MEDP1348	1-(9Z-hexadecenoyl)-sn-glycero-3-[illegible]	Lipids	LPC	C24H48NO7P	3	0.8658	76790-27-7	29399.37	43698.27	41927.67	16557.07	16268.93	15997.9	1.56539334	0.03900989	0.2317402	2.355922202	1.236291899	up	C04230	ko00564
MEDL01000	Octopamine	Alcohol and [illegible]	Polyamines	C8H11NO2	3	0.8536	104-14-3	320730.8	340695.11	303611.78	1156690.28	858498.13	842083.48	1.60101845	0.02424332	0.19303267	0.337747938	-1.56598113	down	-	-
MEDN0302	Chlorogenic Acid	Phenolic acids	Phenolic acids	C16H18O9	3	0.84	202650-88-2	6225.05	6066.96	3210.26	3014.08	2267.84	1154.03	1.28361195	0.07078566	0.30409573	2.40869957	1.268254462	up	C00852	ko00940,ko00941,ko00945,ko01110
MEDL02193	Methyl 4-hydroxycinnamate	Others	Lactones	C10H10O3	3	0.8267	19367-38-5 | 3943-97-3	6078.16	5297.71	8049.9	2336.09	2189.49	4592.38	1.35898107	0.0384451	0.23094812	2.130495198	1.0911888	up	-	-
MEDL02312	Nb-p-Coumaroyltryptamine	Others	Tryptamines	C19H18N2O2	3	0.8265	53905-12-7	5499.76	2425.49	3063.43	10872.51	4691.79	12559.15	1.2425778	0.12628895	0.4002779	0.390730156	-1.35575549	down	-	-
MEDL02488	Quercetin	Flavonoids	Flavonols	C15H10O7	3	0.8203	117-39-5	2034516.18	531824.32	624298.83	490206.18	328004.35	290353.44	1.14410517	0.28882021	0.56731868	2.878173399	1.525153512	up	C00389	ko00941,ko00944,ko01100,ko01110
MW0159335	Versicolorin A	Benzene and substituted derivatives	Benzene and substituted derivatives	C18H10O7	3	0.8167	6807-96-1	2951.39	3405.44	4643.06	5389.99	20621.28	9546.76	1.28195332	0.21231842	0.49813497	0.309350377	-1.6926863	down	C20583	ko01100,ko01110
MEDN0234	Globotriose	Others	Saccharides	C18H32O16	3	0.8133	1109-28-0 | 33404-34-1 | 66580-68-5	68219.67	73510.05	70105.32	11568.38	18892.6	9432.23	1.58705448	0.00034418	0.05012965	5.310052513	2.408726128	up	C00420	-
MEDP1660	Bis(2-ethylhexyl) [illegible]	Others	Lactones	C22H42O4	3	0.8042	103-23-1	8132.59	7066.97	4690.27	23897.93	8723.44	16110.35	1.27492398	0.15351069	0.43832974	0.408149558	-1.2928302	down	C14240	-
MEDP1962	IAA-L-Ala	Amino acids and derivatives	Amino acids and derivatives	C13H14N2O3	3	0.7905	57105-39-2	1250.22	7831.85	6152.43	1731.36	1103.48	1714.93	1.03573831	0.21195301	0.49794285	3.348411019	1.74347663	up	-	-
MEDL02494	2-Aminoisobutyric acid	Organic acids	Organic acids	C4H9NO2	3	0.7889	62-57-7	7416.61	3522.99	6421.22	1603.07	3684.05	1561.81	1.30482153	0.07536433	0.31398244	2.534822228	1.341884572	up	C03665	-
MEDN0555	Hydroxyphenyllactic acid	Benzene and substituted derivatives	Phenolic acids	C9H10O4	3	0.7886	306-23-0	169551.18	142181.67	159888.63	29156.01	22881.22	21321.73	1.62209573	0.00189912	0.08476296	6.428955372	2.684584336	up	C03672	-
MEDN0067	Phenylalanylphenylalanine	Amino acids and derivatives	Amino acids and derivatives	C18H20N2O3	3	0.787	201023-39-4	411.71	1161.27	2452.16	7650.93	2633.77	3943.28	1.23705002	0.13934766	0.41851727	0.282903125	-1.82161998	down	-	-
MEDP0537	LPC(O-16:0/2:0)	Lipids	LPC	C26H54NO7P	3	0.7854	74389-68-7	44710.84	41303.7	29264.94	10042.55	8824.2	6756.12	1.59021216	0.02019312	0.1788997	4.499085387	2.169631748	up	-	-
MEDP1597	Securinine	Alkaloids	Terpenoid alkaloids	C13H15NO2	3	0.7832	5610-40-2	360547.02	488647.98	509948.52	22172.74	32708.12	26415.64	1.62328516	0.01152052	0.14569982	16.7183522	4.063360754	up	-	-
MW0108971	O-benzyl-L-serine	Amino acids and derivatives	Amino acids and derivatives	C10H13NO3	3	0.7808	4726-96-9 | 30916-68-8	2515.33	1837.19	2338.05	320.8	629.95	1240	1.37511004	0.01339364	0.1555072	3.054008901	1.610704267	up	-	-
MEDP1904	1-(9Z,12Z-octadecadienoyl)-glycero-3-phosphoethanolamine	GL	LPE	C23H44NO7P	3	0.7804	85046-18-0	6790.98	8989.84	8816.21	1859.96	1588.91	2058.27	1.61379271	0.00998451	0.14566058	4.466389088	2.159108936	up	-	-
MEDP1475	N-Methylhydantoin	Heterocyclic compounds	Heterocyclic compounds	C4H6N2O2	3	0.7699	616-04-6	47292.9	43794.82	66174.44	42.71	92.05	9349.45	1.357729	0.00957058	0.14545906	16.58147173	4.051500157	up	C02565	ko00330,ko01100
MW0126326	Pyridine-3,4-dicarboxylic acid	Organic acids	Organic acids	C7H5NO4	3	0.7679	490-11-9	99219.99	112401.53	142637.88	41345.03	1414.84	6653.65	1.33351709	0.00480745	0.11959415	7.169280796	2.841828399	up	-	-
MW0147843	Cryptogenin	Others	Ketone compounds	C27H42O4	3	0.7673	468-99-5	268.8	194.33	248.84	28.15	197.15	8.05	1.15964211	0.10340959	0.3645986	3.051082066	1.609320985	up	C17891	-
MEDP0437	Ergothioneine	Alkaloids	Alkaloids	C9H15N3O2S	3	0.7655	497-30-3	333.7	174.95	149.05	327.45	372.32	998.7	1.16866725	0.24666226	0.5293161	0.387230861	-1.36873416	down	C05570	ko00340,ko01100
MW0117890	2,4-Diamino-6-hydroxypyrimidine	Heterocyclic compounds	Heterocyclic compounds	C4H6N4O	3	0.7543	56-06-4 | 100643-27-4 | 143504-99-8 | 195-66-4	22123.94	10604.52	26608.58	37053.58	37797.13	45164.38	1.31021134	0.03202346	0.21871128	0.494413161	-1.01621095	down	-	-
MEDL02423	Phosphocreatine	Organic acids	Organic acids	C4H10N3O5P	3	0.7528	67-07-2	27572.49	25949.2	29113.57	752.86	782.44	1986.25	1.60292311	0.00021163	0.04446273	23.46559328	4.552475031	up	C02305	ko00330,ko01100
MW0110480	2,6-Dihydroxypyridine	Others	Vitamin	C5H5NO2	3	0.7518	626-06-2	154.13	269.86	113.33	131.1	45.68	51.49	1.20168337	0.14777296	0.43205719	2.353879178	1.23504027	up	C03056	ko00760,ko01100
MEDP0606	3,4-Dimethoxyphenethylamine	Benzene and substituted derivatives	Benzene and substituted derivatives	C10H15NO2	3	0.7507	120-20-7	303.2	161.51	202.88	1899.97	1133.73	651.67	1.49800499	0.10761897	0.37038694	0.181145991	-2.46477522	down	-	-
MEDP0574	Oleamide	Lipids	Free fatty acids	C18H35NO	3	0.7483	301-02-0	18748.74	20917.81	24279.56	11749.75	8008.76	7837.11	1.53942869	0.00483206	0.11959415	2.317255782	1.2124173	up	C19670	-
MEDN1056	Iminodiacetic acid	Organic acids	Organic acids	C4H7NO4	3	0.7476	142-73-4	1045.94	821.68	668.08	110.23	341.76	581.02	1.19482892	0.04798716	0.25569785	2.4546713	1.295529849	up	C19911	-
MW0118307	2-benzyl-5-iodopyridazin-3(2H)-one	Benzene and substituted derivatives	Benzene and substituted derivatives	C11H9IN2O	3	0.7476	825633-93-0	957.14	6154.46	5003.79	436.75	921.83	1177.77	1.18093931	0.17787265	0.46740569	4.776702742	2.2560151	up	-	-
MW0114868	Methylpicraquassioside A	Lignans and Coumarins	Coumarins	C19H24O10	3	0.7468	334974-07-1	5832.34	855.84	3763.52	10100	11194.54	16356.78	1.28779357	0.02264135	0.18567593	0.277591861	-1.84896282	down	-	-
MW0007582	Menadione	Others	Vitamin	C11H8O2	3	0.7451	58-27-5	16468.42	12738.73	16179.62	939.12	2571.94	6849.46	1.36726507	0.00774659	0.13950182	4.380742472	2.131175406	up	C05377	-
MW0127606	Epigallocatechin 3,3',-di-O-gallate	Flavonoids	Flavanols	C29H22O15	3	0.7447	89013-66-1	685.67	2367.58	915.3	397.2	564.63	688.27	1.11218792	0.27855047	0.55860069	2.405036058	1.266058524	up	-	-
MEDL01821	3-Furoic acid	Organic acids	Organic acids	C5H4O3	3	0.744	488-93-7	611.97	641.89	847.06	64.16	356.29	304.33	1.23616119	0.01821541	0.17357752	2.898700295	1.535406177	up	-	-
MW0157436	4-[2,2-Bis(diethoxyphosphoryl)ethenyl]-2,6-ditert-butylphenol	Organic acids	Organic acids	C24H42O7P2	3	0.7395	126411-39-0	302.52	258.43	139.7	793.42	637.41	356.02	1.32689762	0.09117815	0.34265588	0.392114615	-1.35065268	down	-	-
MEDP1070	Glycyl-L-valine	Amino acids and derivatives	Amino acids and derivatives	C7H14N2O3	3	0.7381	1963-21-9	179545.97	174968.78	188391.7	153.69	318.88	46.94	1.6137049	0.00047226	0.05342558	1045.03561	10.02933639	up	-	-

MEDL02179	Coumarin	Lignans and Coumarins	Coumarins	C9H6O2	3	0.7371	91-64-5	267353.66	293475.12	175809.18	67925.2	37903.31	79840.63	1.52017834	0.02544682	0.1973135	3.967476555	1.988221698	up	C05851	ko00999,ko01110
MW0146384	Astragaloside II	Terpenoids	Triterpene Saponin	C43H70O15	3	0.7359	91739-01-4	9137.06	10647.75	20031.53	5745.28	9759.86	1717.08	1.08072309	0.15133247	0.43582171	2.311916814	1.209089489	up	C17798	-
MW0153971	Mucronine A	Heterocyclic compounds	Heterocyclic compounds	C29H38N4O4	3	0.7351	38840-25-4	259.1	121.75	1156.98	3161.34	9670.24	1140.45	1.28153823	0.24738117	0.53047175	0.110064894	-3.18357371	down	C10009	-
MEDL00543	3-Methyladipic acid	Organic acids	Organic acids	C7H12O4	3	0.7346	3058-01-3 | 61898-58-6	22838.7	31384.6	7426.04	43921.06	48827.28	50332.04	1.22866883	0.05196327	0.26504273	0.430872073	-1.2146685	down	-	-
MW0151112	His-Trp-Gly	Amino acids and derivatives	Amino acids and derivatives	C19H22N6O4	3	0.7344	89032-48-4	464.57	200.14	1175.7	825.55	2290.43	1664.39	1.12170871	0.13880517	0.4174563	0.384993212	-1.37709509	down	-	-
MW0151993	Kanamycin 3'-	Organic acids	Organic acids	C18H37N4O14P	3	0.7331	17029-36-6	7783.7	12587.41	4520.97	1834.81	5024.02	2834.28	1.22571804	0.15120288	0.43582171	2.568017901	1.360655259	up	C03281	-
MEDP1800	Sanguinarine	Alkaloids	Isoquinoline alkaloids	C20H14NO4+	3	0.7326	2447-54-3	10254.5	6653.61	9121.34	1813.78	1349.65	1993.05	1.59593642	0.01983248	0.17676954	5.04791059	2.335686357	up	C06162	ko00950,ko01100
MW0009854	2-Anthracenesulfonic acid, 1-amino-4-(cyclohexylamino)-9,10-dihydro-9,10-dioxo-	Benzene and substituted derivatives	Benzene and substituted derivatives	C20H20N2O5S	3	0.732	5617-28-7	8020.23	11851.45	5987.34	1228.57	741.63	4041.01	1.37462545	0.04026209	0.23653307	4.301799471	2.104940275	up	-	-
MW0116854	1-Acetyl-2-methyl-1,2,3,4-	Heterocyclic compounds	Heterocyclic compounds	C12H15NO	3	0.7277	16078-42-5	1477.87	2060.74	6347.47	18947.81	17963.11	17974.69	1.48305518	0.0080816	0.14010122	0.180121529	-2.47295746	down	-	-
MEDP2122	11-Carbonyl-beta-acetyl-boswellic-acid	Organic acids	Organic acids	C32H48O5	3	0.7256	67416-61-9	598.02	308.01	273.52	211.83	77.69	130.69	1.32463806	0.11990344	0.39041622	2.807048857	1.489054174	up	-	-
MW0146889	Bruceoside A	Others	Lactones	C32H42O16	3	0.7245	63306-30-9	2885.94	1096.3	1638.19	647.01	329.74	1018.69	1.26421221	0.13809746	0.41632027	2.816636932	1.493973611	up	C08753	-
MW0000043	10-	Alkaloids	Quinoline	C20H16N2O5	3	0.7238	19685-09-7	3655.27	1935.6	6882.34	1954.84	1195.9	1144.58	1.25206379	0.19770962	0.48461731	2.903907043	1.537995272	up	C17939	-
MW0111931	Gallacetophenone	Phenolic acids	Phenolic acids	C8H8O4	3	0.7226	528-21-2	4111065.6	3191809.33	4118384.23	1732.71	1138.32	390989.5	1.38528254	0.00258697	0.09724636	28.99823234	4.857893055	up	-	-
MEDP1076	Kojibiose	Others	Saccharides	C12H22O11	3	0.7218	2140-29-6	35897.84	31638.17	39221.93	7594.52	19220.91	21013.56	1.31060721	0.02535401	0.19715214	2.232075986	1.158386141	up	C19632	-
MW0005752	Hexanoic acid, 6-[[(4-methylphenyl)sulfonyl]amino]-	Benzene and substituted derivatives	Benzene and substituted derivatives	C13H19NO4S	3	0.7209	78521-39-8	2033.23	1114.82	674.6	16764.24	11122.41	22829.8	1.56966885	0.04203244	0.24052804	0.075372981	-3.72980875	down	-	-
MEDN1410	N-Palmitoylglycine	Amino acids and derivatives	Amino acids and derivatives	C18H35NO3	3	0.7208	158305-64-7	63161.48	40052.09	62634.91	24596.01	22123.2	28139.88	1.50180753	0.05137085	0.26293296	2.215475502	1.147616374	up	-	-
MW0004065	3-(Hydroxy(phenyl)phosphoryl)propanoic acid	Benzene and substituted derivatives	Benzene and substituted derivatives	C9H11O4P	3	0.7195	14657-64-8	2697.02	8090.91	4235.65	2182.55	432.67	630.22	1.3429865	0.12244361	0.39403444	4.629135033	2.210742647	up	-	-
MW0007107	Felbamate	Benzene and substituted derivatives	Benzene and substituted derivatives	C11H14N2O4	3	0.7167	25451-15-4	4.35	22.61	11.6	20.59	27.54	47.58	1.14044667	0.13151875	0.4072005	0.402883711	-1.31156462	down	C07501	-
MEDP1884	Prolyl-Histidine	Amino acids and derivatives	Amino acids and derivatives	C11H16N4O3	3	0.7165	92027-43-5	9634.25	7361.89	9473.67	1512.63	765.16	4853.42	1.34765557	0.01841142	0.17394261	3.711825903	1.892129045	up	-	-
MEDN0101	Theobromine	Alkaloids	Alkaloids	C7H8N4O2	3	0.7158	83-67-0	1746.37	1437.88	7618.23	951.9	1396.33	138.88	1.0726739	0.30074372	0.57540385	4.343386501	2.118820338	up	C07480	ko00232,ko01100,ko01110
MEDL02217	Moupinamide	Alkaloids	Phenolamine	C18H19NO4	3	0.7137	66648-43-9	3482.19	3001.61	2025.66	9868.77	8747.03	8367.23	1.57008229	0.00059145	0.06185252	0.315363397	-1.66491287	down	C02717	-
MEDP1183	N-Acetylcadaverine	Alkaloids	Alkaloids	C7H16N2O	3	0.7105	32343-73-0	21575.23	19674.52	28526.53	8611.45	5974.35	14364.97	1.40329028	0.02070382	0.18081323	2.410170092	1.269134965	up	-	-
MADP0540	2-Pyrimidin-2-yl-propionic acid	Heterocyclic compounds	Heterocyclic compounds	C7H8N2O2	3	0.7091	819850-16-3	15440.83	16719.62	18776.57	84.25	123.25	342.38	1.60760742	0.00315739	0.10261813	92.63297447	6.533453935	up	-	-
MW0108611	N-Acetylcysteine amide	Alcohol and amines	Amines	C5H10N2O2S	3	0.708	38520-57-9 | 15394-73-7	2112.21	1164.71	1120.73	3466.87	6056.84	3963.94	1.48113673	0.04688035	0.25471913	0.326050127	-1.61683431	down	-	-
MW0002036	2-(Methylamino)benzoic acid	Benzene and substituted derivatives	Benzene and substituted derivatives	C8H9NO2	3	0.7076	119-68-6	43952.67	48280.96	43680.3	19062.22	13692.23	20484.75	1.57731292	0.00068	0.06250295	2.552892042	1.35213253	up	C03005	ko00996,ko01110
MEDP1645	Benzamide	Phenolic acids	Phenolic acids	C7H7NO	3	0.7067	55-21-0	803.75	1034.53	296.58	1217.7	2631.14	1486.05	1.2120761	0.11679322	0.38397295	0.400169451	-1.32131706	down	C09815	-
MW0104956	3,3'-Thiodipropionodihydraz	Alcohol and amines	Amines	C6H14N4O2S	3	0.7055	6292-68-8	5222.73	1295.41	1684.51	31206.34	29235.3	36905.08	1.55406612	0.00128465	0.07855195	0.084262212	-3.56897039	down	-	-
MW0003649	3,4-Dimethylbenzoic	Phenolic acids	Phenolic acids	C9H10O2	3	0.7048	619-04-5	7028.61	7116.21	413.42	10674.84	15387.84	29097.35	1.10318456	0.12005768	0.39061195	0.26392734	-1.92178729	down	-	-
MW0110910	Aminophylline	Alkaloids	Alkaloids	C16H24N10O4	3	0.7046	317-34-0 | 95646-60-9	17560.03	15244.09	18154.26	7673.77	256.12	27.7	1.23723552	0.01990825	0.17708711	6.403745355	2.67891594	up	-	-
MEDL02688	p-Coumaroyltyramine	Alkaloids	Phenolamine	C17H17NO3	3	0.7002	36417-86-4	1.03	4.55	1.56	44.87	23.31	5.92	1.36141371	0.18499877	0.47289372	0.096356275	-3.37547756	down	-	-
MW0146360	Asp-Lys	Amino acids and derivatives	Amino acids and derivatives	C10H19N3O5	3	0.6999	5891-51-0	2965.7	2643.74	3385.81	308.09	2178.92	1914.71	1.01504228	0.10676052	0.3691444	2.043576148	1.031096002	up	-	-
ZINC1520521	2-Oxazolidinethione, 5-ethyl-5-methyl-, (S)-	Others	Ketone compounds	C6H11NOS	3	0.6987	3690-37-7	1211.89	612.16	519.16	117.85	13.59	96.75	1.4216223	0.08016676	0.3241941	10.26867961	3.360178781	up	-	-
MW0127304	3-Mercapto-2-methyl-1-butanol	Benzene and substituted derivatives	Benzene and substituted derivatives	C5H12OS	3	0.6976	227456-33-9	2183.86	4341.56	3284.01	4382.74	10101.32	10197.88	1.29193459	0.11201122	0.3775421	0.397433508	-1.33121458	down	-	-
MEDP1799	Quinaldic acid	Organic acids	Organic acids	C10H7NO2	3	0.6972	93-10-7	1948.8	3077.85	2990.28	8645.22	8701.4	7865.57	1.57982688	0.00035223	0.05012965	0.317978327	-1.65299966	down	C06325	-
MW0160835	[2-(6-Amino-9H-purin-9-YL)-1-methylethoxy]methyl-triphosphate	Organic acids	Organic acids	C9H16N5O10P3	3	0.6972	166403-66-3	501.64	238.45	455.88	213.51	39.8	203.63	1.1303908	0.07493649	0.31279072	2.617345822	1.388104556	up	-	-
MW0004432	2,2-Bis(4-(3,4-dicarboxyphenoxy)phenyl)propane dianhydride	Benzene and substituted derivatives	Benzene and substituted derivatives	C31H20O8	3	0.6963	38103-06-9	11814.93	12771.29	12006.07	246.06	42.46	272.86	1.5765225	0.00029963	0.05012965	65.18274609	6.026418228	up	-	-
MW0126851	3-(3-methylbut-2-en-1-yl)-3H-purin-6-amine	Heterocyclic compounds	Heterocyclic compounds	C10H13N5	3	0.6958	10091-84-6	8667.85	5942.8	9425.99	1124.9	2497.2	3121.04	1.45481822	0.01593034	0.16425584	3.5646064	1.833742785	up	C08435	-

MW0107880	L-Homophenylalanine	Amino acids and derivatives	Amino acids and derivatives	C10H13NO2	3	0.6953	943-73-7	10024.93	5227.73	3679.27	21442.91	14063.52	17135.89	1.41384006	0.01771567	0.17028658	0.359633276	-1.47540158	down	C17235	ko00400,ko00966,ko01100,ko01110,ko01210
MW0006987	Epsilon-pyrromycinone	Benzene and substituted derivatives	Benzene and substituted derivatives	C22H20O9	3	0.6892	21288-61-9	60728.01	73451.22	78580.82	18497.24	35873.68	45755.65	1.3445671	0.022389	0.18480239	2.124911	1.087402417	up	-	-
MW0014680	(3R,5R,7R,8R,9S,10S,12S,13R,14S,17R)-17-[(2S)-4,6-Dihydroxy-6-methylheptan-2-yl]-10,13-dimethyl-2,3,4,5,6,7,8,9,11,12,14,15,16,17-tetradecahydro-1H-cyclopenta[a]phenanthrene-3,7,12-triol	Alcohol and amines	Alcohols	C27H48O5	3	0.6891	137252-15-4	710.2	2472.7	347.09	2318.04	7836.1	1600.71	1.06540908	0.29694189	0.57385353	0.300300727	-1.73552013	down	-	-
MW0126293	Pyrazinamide	Organic acids	Organic acids	C5H5N3O	3	0.6865	98-96-4	3934.67	19634.86	6705.98	39998.72	35465.44	32162.35	1.37427618	0.01921973	0.17648958	0.281301605	-1.82981031	down	C01956	-
MEDP1605	Glu-glu	Amino acids and derivatives	Amino acids and derivatives	C10H16N2O7	3	0.6807	3929-61-1	9255.17	13856.96	8563.38	2756.47	119.27	4979.77	1.11277371	0.02282939	0.18652454	4.032266524	2.011591001	up	C01425	-
MW0137454	Avenanthramide A	Alkaloids	Phenolamine	C16H13NO5	3	0.6775	108605-70-5	9222.53	2867.26	5310.7	2077.39	1322.57	1119.34	1.39619779	0.14301398	0.42418205	3.850262209	1.944956699	up	C08472	-
MW0113021	4-Aminophenyl b-D-thiogalactopyranoside	Benzene and substituted derivatives	Benzene and substituted derivatives	C12H17NO5S	3	0.6773	29558-05-2	3734.5	3323.75	653.55	589.96	381.19	171.77	1.3056903	0.14954785	0.43451026	6.74745389	2.754343213	up	-	-
MW0054126	Isopropyl linoleate	Others	Lactones	C21H38O2	3	0.6771	22882-95-7	29.25	35.88	21.57	726.94	134.95	19884.7	1.28800176	0.39964851	0.64980829	0.004179	-7.90262652	down	-	-
MEDL00352	Glu-Val	Amino acids and derivatives	Amino acids and derivatives	C10H18N2O5	3	0.6752	5879-06-1	36009.81	44638.91	40951.5	100391.87	89628.51	91150.97	1.60925103	0.00035165	0.05012965	0.432477278	-1.20930376	down	-	-
MW0169779	Rifaximin (Xifaxan)	Benzene and substituted derivatives	Benzene and substituted derivatives	C43H51N3O11	3	0.6733	80621-81-4	29927.62	24547.57	13814.02	10162.74	4389.61	9186.47	1.36436774	0.0739522	0.3113331	2.876689322	1.52440942	up	-	-
MW0126367	Queuine	Alkaloids	Alkaloids	C12H15N5O3	3	0.6728	72496-59-4 | 69565-92-0	20204.93	22660	28549.46	11154.21	11320.52	9983.99	1.57301078	0.03145807	0.21754388	2.200160388	1.137608698	up	C01449	-
MW0104039	(Z)-S-1-Propenyl methanesulfinothioate	Others	Others	C4H8OS2	3	0.6727	119052-99-2	7257.2	5441.69	9624.91	4309.07	2958.59	3758.28	1.41648471	0.07794369	0.32019325	2.024661843	1.017680971	up	-	-
MW0004101	3-Methoxybenzyl alcohol	Benzene and substituted derivatives	Benzene and substituted derivatives	C8H10O2	3	0.6724	6971-51-3	184693.31	118629.23	169965	43315.35	53535.31	148.23	1.01983318	0.00919056	0.14331421	4.879308825	2.286676798	up	C03352	-
MW0009257	Olmesartan	Benzene and substituted derivatives	Benzene and substituted derivatives	C24H26N6O3	3	0.6722	144689-63-4	1909.81	696.73	1594.68	312.71	501.79	699.93	1.2815505	0.12256004	0.39412163	2.774126239	1.47203344	up	-	-
MEDN0622	Porphobilinogen	Organic acids	Organic acids	C10H14N2O4	3	0.672	487-90-1	5426.08	6473.7	7027.53	77.34	92.48	624.72	1.53219587	0.00248636	0.09520967	23.82172074	4.574205724	up	C00931	ko00860,ko01100,ko01110,ko01240
MW0106942	Glutamylasparagine	Amino acids and derivatives	Amino acids and derivatives	C9H15N3O6	3	0.6715	36314-37-1	189631.56	119733.6	181868.82	51655.07	86923.34	65656.84	1.47236506	0.03289484	0.22066836	2.405236021	1.26617847	up	-	-
MW0114378	D-xylulose	Others	Saccharides	C5H10O5	3	0.6684	551-84-8 | 5962-29-[illegible]	167.98	377.64	34.62	19.17	53.8	35.09	1.09067258	0.2548779	0.53660748	5.369609476	2.424817167	up	-	-
MW0147868	Cucurbitacin O	Others	Ketone compounds	C30H46O7	3	0.6673	2163-40-8 | 25383-23-7	166295.48	181909.1	201670.98	77482.93	80404.29	90466.76	1.60679656	0.0049153	0.11959415	2.214079919	1.146707299	up	C08803	-
MEDN0722	Orcinol	Phenolic acids	Phenolic acids	C7H8O2	3	0.6666	504-15-4	34.28	34.74	48.04	203.28	300.26	78.57	1.42553338	0.13630135	0.41330691	0.201096013	-2.31404362	down	C00727	-
MW0007219	Frangulin A	Benzene and substituted derivatives	Benzene and substituted derivatives	C21H20O9	3	0.6656	521-62-0	38596.43	38729.66	40901.8	11807.6	24819.31	17174.65	1.45016571	0.02582708	0.19834267	2.197480705	1.135850498	up	-	-
MEDL01916	Phenol	Phenolic acids	Phenolic acids	C6H6O	3	0.6637	108-95-2	13	8.95	8.71	15.33	20.84	105.49	1.12725782	0.33234196	0.59912952	0.216433715	-2.20800285	down	C15584	-
MEDP1027	Maltol	Heterocyclic compounds	Heterocyclic compounds	C6H6O3	3	0.6625	118-71-8	379.65	413.69	629.87	50.11	69.01	298.59	1.30637811	0.04012782	0.23637376	3.40717244	1.768574965	up	C11918	ko01110
MEDN0553	2-Methylbenzoic acid	Phenolic acids	Phenolic acids	C8H8O2	3	0.6624	118-90-1	4.8	10.25	2.46	219.65	185.14	4.1	1.08177327	0.19010367	0.47658128	0.042823253	-4.54546179	down	C07215	ko01100
MW0114766	LS-Tetrasaccharide b	Others	Saccharides	C37H62N2O29	3	0.6599	64003-54-9	7237.32	3752.52	8845.73	2776.12	1173.06	1263.6	1.42555142	0.07045724	0.30358605	3.805180729	1.927964977	up	-	-
MEDL02636	Ephedrine	Alkaloids	Alkaloids	C10H15NO	3	0.6594	299-42-3	12619.9	14580.84	16138.59	68578.46	53238.77	54398.98	1.61371598	0.00979576	0.14545906	0.245944059	-2.02359789	down	C01575	ko00996,ko01100,ko01110
MW0103864	(S)-2-Amino-2-(2,3-dihydro-1H-inden-2-yl)acetic acid	Organic acids	Organic acids	C11H13NO2	3	0.6579	155239-51-3	18.1	442.97	481.58	1209.52	665.54	1019.45	1.0296225	0.04072311	0.23740616	0.325668248	-1.61852503	down	-	-
MEDL00392	1-(9Z-octadecenoyl)-sn-glycero-3-[illegible]	Lipids	LPC	C26H52NO7P	3	0.6572	19420-56-5	3851.23	1599.63	4023.47	114.58	500.74	1250.76	1.28811802	0.06633918	0.29624338	5.077129598	2.344013087	up	-	-
MEDL00349	Ser-Leu	Amino acids and derivatives	Amino acids and derivatives	C9H18N2O4	3	0.6564	6665-16-3	1360.66	1236.37	2757.4	74.64	59.71	258.75	1.52848073	0.07462687	0.31230283	13.6210379	3.767764734	up	-	-
MW0000324	Lupinine	Alkaloids	Alkaloids	C10H19NO	3	0.6561	486-70-4 | 10248-30-3	1387	1730.8	1450.1	12.62	54.77	75.17	1.56473888	0.00413594	0.11299759	32.04194725	5.001889921	up	-	-
MEDL02339	Diethyl hydrogen phosphate	Organic acids	Organic acids	C4H11O4P	3	0.653	598-02-7	30990.51	40468.84	37640.09	664.67	5039.57	3223.24	1.49285779	0.0023377	0.09291822	12.22063113	3.611246889	up	C06608	-
MW0103367	3-Deaza-2'-deoxyadenosine	Nucleotides and derivatives	Nucleotides and derivatives	C11H14N4O3	3	0.6504	78582-17-9	151.04	119.65	303.2	297.01	384.98	819.08	1.22996244	0.18717314	0.47487514	0.382320611	-1.38714512	down	-	-
MEDP1505	5-Methoxytryptophan	Amino acids and derivatives	Amino acids and derivatives	C12H14N2O3	3	0.6502	2504-22-5	1621.61	983.87	283.16	5196.44	3028.41	1263.71	1.12732942	0.18442279	0.47251646	0.304433971	-1.71579874	down	-	-
MW0001942	2-(4-Methoxyphenoxy)acetohydrazide	Phenolic acids	Phenolic acids	C9H12N2O3	3	0.6496	21953-91-3	15178.11	10746.88	12055.89	691.02	893.69	958.98	1.62385066	0.01191819	0.14824085	14.93141067	3.900278568	up	-	-

MW0128402	(S,S)-(-)-Hydrobenzoin	Benzene and substituted derivatives	Benzene and substituted derivatives	C14H14O2	3	0.6473	2325-10-2 | 655-48-1	26515.19	31310.49	36071.49	8420.14	6840.49	6782.4	1.61365378	0.01077329	0.14566058	4.259721554	2.090759129	up	-	-
MW0006323	Benzylamine	Alkaloids	Alkaloids	C7H9N	3	0.6472	100-46-9	2587.36	1668.08	808.52	635.93	517.25	779.17	1.25859038	0.17671926	0.46631902	2.620622558	1.389909581	up	C15562	-
MW0052336	Diethyl fumarate	Others	Lactones	C8H12O4	3	0.6444	623-91-6 | 26298-06-6	14457.35	15318.95	22044.38	2805.82	2913.83	4583.47	1.58343969	0.0236223	0.18914989	5.029610448	2.330446665	up	-	-
MW0009518	Procaine	Benzene and substituted derivatives	Benzene and substituted derivatives	C13H20N2O2	3	0.6439	59-46-1	24082.27	31588.39	31265.55	233.74	350.31	94.69	1.6126878	0.00714167	0.13569	128.0847011	7.000954355	up	C07375	-
MW0054292	Lauroyl diethanolamide	Lipids	Others	C16H33NO3	3	0.6438	120-40-1	790.26	1084.17	2254.28	1161.04	229.11	98.17	1.09936264	0.19574772	0.48243539	2.774074124	1.472006337	up	-	-
MW0153988	Muscapurpurin	Heterocyclic compounds	Heterocyclic compounds	C18H14N2O10	3	0.643	12624-19-0	119902.11	122496.89	93874.8	38225.19	1015.23	543.58	1.32600419	0.00412927	0.11299759	8.452488438	3.079376138	up	C08563	-
MEDL02503	Carnitine C16:0	Alkaloids	Alkaloids	C23H45NO4	3	0.6426	2364-67-2	95.53	81.86	22.73	227.66	132.32	165.66	1.2348445	0.04108946	0.23822166	0.38071684	-1.39320971	down	C02990	ko00071,ko01212
MW0142566	Propargyl alcohol	Others	Hydrocarbon derivatives	C3H4O	3	0.6401	107-19-7 | 30306-19-5	3174.1	2900.86	2771.26	997.92	1726.92	408.76	1.33384676	0.02891777	0.20834886	2.823021445	1.497240088	up	C05986	-
MW0127388	1,2,4-Triazin-5(2H)-one, 6-(1,1-dimethylethyl)-3-(methylthio)	Others	Ketone compounds	C8H13N3OS	3	0.6361	35045-02-4	824.07	3958.29	3542.04	6261.06	7521.98	6654.73	1.19683792	0.04090575	0.23778152	0.407304711	-1.29581959	down	-	-
MW0139539	Retusin	Flavonoids	Flavones	C19H18O7	3	0.6356	1245-15-4	1494.79	971.44	935.75	18.7	13.22	444.96	1.32638498	0.01489055	0.16129107	7.133828217	2.834676476	up	-	-
MW0052594	Ercalcitriol	Alcohol and [illegible]	Alcohols	C28H44O3	3	0.6345	60133-18-8	6.04	6.49	5.69	1.66	2.31	0.57	1.43043681	0.00493339	0.11959415	4.013215859	2.004758757	up	-	-
MEDN0636	Ginkgolic acid	Phenolic acids	Phenolic acids	C22H34O3	3	0.6334	22910-60-7	1974.66	1940.19	2211.1	316.91	1282.39	715.92	1.32162886	0.03608943	0.22604178	2.645947253	1.403784302	up	-	-
MW0103335	2'-Deoxyadenosine-5'-diphosphate	Nucleotides and derivatives	Nucleotides and derivatives	C10H15N5O9P2	3	0.6298	2793-06-8	1.9	17.03	17.6	0.85	2.19	4.83	1.0033477	0.19972493	0.48684331	4.641677255	2.214646213	up	C00206	ko00230,ko01100,ko01232
MW0153878	Met-Thr	Amino acids and derivatives	Amino acids and derivatives	C9H18N2O4S	3	0.6285	40883-16-7	17677.93	18856.32	12858.73	2335.19	2190.05	872.73	1.56551423	0.01140433	0.14569982	9.150287979	3.193817149	up	-	-
MW0151985	Kaempferol 3-O-beta-D-glucosylgalactoside	Flavonoids	Flavonols	C27H30O16	3	0.6285	31512-06-8	181.24	155.88	341.58	91.13	20.94	7.66	1.33735577	0.0675735	0.29786568	5.668587656	2.502989328	up	-	-
MW0005393	5-(3-Carboxybenzoyl)-2-((6-(4-methoxyphenyl)-5-hexenyl)oxy)benzenepropanoic acid	Benzene and substituted derivatives	Benzene and substituted derivatives	C30H30O7	3	0.6266	117423-74-2	2883.5	3951.63	5153.02	3893.48	10841.61	11076.54	1.0927331	0.18301139	0.47026574	0.464447615	-1.10641221	down	-	-
MEDP1796	Perillyl alcohol	Terpenoids	Monoterpenoids	C10H16O	3	0.6227	536-59-4	18711.41	19751.78	24044.5	57076.76	37245.98	34636.8	1.46495437	0.08206614	0.32678322	0.484707762	-1.04481291	down	C02452	ko00902,ko01110
MW0138164	Fisetinidol	Flavonoids	Flavanonols	C15H14O5	3	0.6203	490-49-3	15398.01	15474.21	13604.9	569.89	1159.4	1080.43	1.61156222	0.00088845	0.06912669	15.82973392	3.984565101	up	C09735	-
MEDL02748	Auraptene	Lignans and Coumarins	Coumarins	C19H22O3	3	0.6188	495-02-3	237.51	112.51	112.65	405.68	292.11	351.51	1.39678709	0.0231041	0.18703724	0.44093205	-1.18137175	down	-	-
MW0103342	2'-Deoxycytidine-5'-triphosphate	Nucleotides and derivatives	Nucleotides and derivatives	C9H16N3O13P3	3	0.6183	2056-98-6	4154.02	4713.98	6145.01	23598.23	17793.94	6652.53	1.2974956	0.1554086	0.43991021	0.312480045	-1.67816403	down	C00458	ko00240,ko01100,ko01232
MW0010043	Venlafaxine	Phenolic acids	Phenolic acids	C17H27NO2	3	0.6181	93413-69-5	1.95	3.93	3.42	1.22	1.98	0.53	1.22924262	0.06979683	0.30220177	2.493297587	1.318055086	up	C07187	-
MW0161291	K252a	Others	Lactones	C27H21N3O5	3	0.6174	171333-67-8	10421.24	2416.78	9042.05	375.71	87.65	1452.01	1.36141432	0.11079582	0.37538116	11.42341689	3.513922338	up	-	-
MEDN1793	protoveratrine A	Alkaloids	Steroid alkaloids	C41H63NO14	3	0.6167	143-57-7 | 1380-61-[illegible]	1111.82	482.33	538.63	700.81	227.82	130.83	1.00559691	0.2528881	0.53498055	2.013082136	1.009406037	up	-	-
MEDP1304	2-Piperidinone	Alkaloids	Piperidine alkaloids	C5H9NO	3	0.6162	675-20-7	299.77	239.18	164.38	45.54	122.37	73.97	1.39991278	0.03851336	0.23094812	2.907764181	1.539910272	up	-	-
MW0110392	Valylcysteine	Amino acids and derivatives	Amino acids and derivatives	C8H16N2O3S	3	0.6148	160070-15-5	1265.18	3882.06	1693.94	8954.76	14780.43	9499.66	1.49165994	0.02720984	0.20385331	0.205843565	-2.28037975	down	-	-
MW0014685	5alpha-Androstane-3alpha,17beta-diol disulfate	Others	Lactones	C19H32O8S2	3	0.6144	21152-49-8	288.73	919.73	856.96	232.37	237.67	251.02	1.27413237	0.15508929	0.43991021	2.864421823	1.518243964	up	-	-
MW0150114	Glu-Phe-Tyr	Amino acids and derivatives	Amino acids and derivatives	C23H27N3O7	3	0.6141	70421-70-4	933.73	671.23	817.39	68.91	25.99	868	1.10654519	0.21214187	0.4981213	2.515681795	1.330949449	up	-	-
MW0168258	Dihydrorobinetin	Flavonoids	Flavones	C15H12O7	3	0.614	4382-33-6 | 93432-80-5	406870.6	504091.22	1701173.53	2119.58	29931	63394.66	1.40526777	0.18116575	0.46907066	27.36789546	4.774412593	up	-	-
MW0054028	Ipecoside	Others	Lactones	C27H35NO12	3	0.6107	15401-60-2	18441.89	7636.88	14706.35	23901.17	45242	40813.29	1.3724405	0.052216	0.26602426	0.370920635	-1.43081756	down	C09464	-
MW0107878	L-Homocysteine sulfonic acid	Organic acids	Organic acids	C3H9NO3S2	3	0.6106	59729-28-1	2250.04	3513.73	4710.56	4301.33	13520.71	10063.93	1.22409083	0.15643027	0.44088176	0.375612898	-1.41268149	down	-	-
MEDP2037	3-Phenylpropionylglycine	Amino acids and derivatives	Amino acids and derivatives	C11H13NO3	3	0.6076	20989-69-9	7397597.52	13943158.88	10372319.96	6661073.37	3134657.37	2680418.58	1.35036877	0.05608842	0.27711323	2.541896185	1.34590511	up	-	-
MW0109688	Ser-Ser	Amino acids and derivatives	Amino acids and derivatives	C6H12N2O5	3	0.6075	6620-95-7	26521.06	41331.86	54205.44	7094.76	27337.55	15970	1.213232	0.07945948	0.3228133	2.421681863	1.27600935	up	-	-
MW0132715	2,3-Dihydro-2-(3,4-dihydroxyphenyl)-3,5-bis(3,4,5-trihydroxybenzoyloxy)-4H-1-benzopyran-7-ol	Flavonoids	Flavones	C29H22O14	3	0.6058	37484-74-5	12052.61	11724.76	9958.6	1484.02	1264.42	1087.12	1.62506885	0.00337901	0.10399056	8.795578742	3.136778509	up	-	-
MW0006304	Benzyl benzoate	Benzene and substituted derivatives	Benzene and substituted derivatives	C14H12O2	3	0.6032	120-51-4	155153.95	52095.53	165168.37	45449.04	17524.57	46278.12	1.25934894	0.12812363	0.40364464	3.40880506	1.769266098	up	C12537	-
MW0139797	Tephrosin	Flavonoids	Other Flavonoids	C23H22O7	3	0.6031	76-80-2	496303.08	570439.69	638390.36	144912.87	226330.75	225616.99	1.56020246	0.00292288	0.10260237	2.856836423	1.514418433	up	-	-
MEDTP0254[illegible]	5-(6-Hydroxy-2,5,7,8-tetramethyl-chroman-2-YL)-2-methyl-pentanoic acid	Organic acids	Organic acids	C19H28O4	3	0.6006	7083-09-2	5993.13	3469.03	1125.99	4893.79	14662.78	15074.41	1.17673044	0.12332496	0.3945943	0.305742142	-1.70961268	down	-	-
MEDL01926	Albanin A	Flavonoids	Flavones	C20H18O6	3	0.6	73343-42-7	939	236.81	980.82	478	152.45	193.92	1.0058225	0.19897204	0.48595854	2.616094715	1.387414774	up	-	-

MW0157291	Sulfazecin	Amino acids and derivatives	Amino acids and derivatives	C12H20N4O9S	3	0.5979	77912-79-9	862.15	1445.5	448.98	235.28	458.08	328.49	1.24714328	0.17848607	0.46794913	2.69768557	1.431722204	up	C20927	ko00261
Lmzn006284	Corosolic acid	Terpenoids	Triterpene	C30H48O4	3	0.5952	4547-24-4	34.98	12.57	10.06	177.56	178.71	113.8	1.53359307	0.01464251	0.16000982	0.122556215	-3.02848445	down	-	-
MW0124805	N-(3-(9H-carbazol-9-yl)-2-hydroxypropyl)-N-(furan-2-ylmethyl)methanesulfonamide	Alcohol and amines	Amines	C21H22N2O4S	3	0.5948	309928-48-1	5111.44	8963.54	6084.64	365.01	3757.23	770.2	1.29946262	0.03223795	0.21915843	4.120565607	2.042842382	up	-	-
MW0139880	Tricin 7-	Flavonoids	Flavones	C29H30O19	3	0.5933	83097-44-3	4620.69	2400.64	4497.18	1039.34	1178.79	2203.58	1.36867367	0.06187276	0.28817563	2.604989925	1.381277793	up	-	-
MEDN1083	Sphingosine 1-	Lipids	Sphingolipids	C18H38NO5P	3	0.5931	26993-30-6	742.7	406.84	944.88	218.06	389.83	378.26	1.23568373	0.13110391	0.4069309	2.123835116	1.086671767	up	C06124	ko00600,ko01100
MW0115052	(6aR,9R,9aR)-9-acetyl-3-[(1E,3E,5S)-3,5-dimethylhepta-1,3-dienyl]-6a-methyl-9,9a-dihydrofuro[2,3-h]isochromene-6,8-dione	Others	Ketone compounds	C23H26O5	3	0.5926	53014-38-3	1293.11	2503.44	2093.07	44.98	36.55	287.47	1.50154477	0.03000093	0.2125747	15.96102981	3.996481833	up	-	-
MW0126336	Pyridoxamine phosphate	Organic acids	Organic acids	C8H13N2O5P	3	0.5886	529-96-4	381056.19	414786.55	408815.29	1653.38	173869.11	1656.03	1.28206902	0.02394306	0.19102488	6.799120063	2.765348046	up	C00647	ko00750,ko01100
MEDN1507	3-Methylpentanoic acid	Organic acids	Organic acids	C6H12O2	3	0.5837	105-43-1	2900.26	2203.11	3809.14	453.32	336.4	1069.39	1.48576012	0.0214084	0.18323322	4.79396593	2.261219656	up	-	-
MEDL02754	2,6-Dimethoxy-1,4-benzoquinone	Quinones	Quinones	C8H8O4	3	0.5827	530-55-2	42416.16	25830.65	23139.53	54.41	66.25	5798.14	1.36468123	0.03238165	0.21978922	15.44001149	3.948601921	up	C10331	-
MW0001326	2-(4-Iodo-2,5-dimethoxyphenyl)-N-((2,3-methylenedioxyphenyl)methyl)ethanamine	Benzene and substituted derivatives	Benzene and substituted derivatives	C18H20INO4	3	0.5785	919797-25-4	133.06	98.83	37.87	297.67	243.44	373.39	1.40159471	0.01228808	0.14864691	0.294980864	-1.76130673	down	-	-
MW0123976	Ethyl 4-(1-hydroxy-1-methylethyl)-2-propyl-imidazole-5-carboxylate	Others	Lactones	C12H20N2O3	3	0.5772	144689-93-0	707.69	375.26	645.13	105.09	279.51	35.39	1.31199827	0.02978736	0.2125747	4.114574157	2.040743125	up	-	-
MEDP1249	2-Oxo-4-phenylbutyric acid	Benzene and substituted derivatives	Phenolic acids	C10H10O3	3	0.5763	710-11-2	109906.78	123879.95	137082.82	108.11	913.62	101907.82	1.15459361	0.11160239	0.37702125	3.60313972	1.849254597	up	C20327	ko00400,ko01100,ko01210
MW0055194	N-(1,3-Dihydroxyoctadecan-2-YL)-6-[(7-nitro-2,1,3-benzoxadiazol-4-YL)amino]hexanamide	Lipids	Cer	C30H51N5O6	3	0.5717	114301-95-0	9889611.6	10563881.65	13488212.61	1914487.91	2566563.93	2592016.11	1.60886655	0.01229466	0.14864691	4.798724698	2.262651048	up	-	-
MW0148433	Dibenzyl succinate	Benzene and substituted derivatives	Benzene and substituted derivatives	C18H18O4	3	0.5701	103-43-5	42995.24	26286.37	36884.42	52.93	176.52	87.85	1.61962041	0.01860106	0.17461247	334.5919634	8.386258984	up	C07332	-
MW0115745	Cannabichromene	Others	Others	C21H30O2	3	0.5701	20675-51-8 | 18793-28-7	1493.61	1697.27	3621.81	934.1	389.57	1759.61	1.06489049	0.20550904	0.49134567	2.2095593	1.143758651	up	C08998	-
MW0000632	(2-Methylbenzyl)(pyridin-4-ylmethyl)amine	Benzene and substituted derivatives	Benzene and substituted derivatives	C14H16N2	3	0.5668	880809-54-1	443.11	336.78	450.87	106.36	148.17	45.01	1.46902038	0.00324698	0.10290142	4.108833545	2.038728886	up	-	-
MW0117708	2-(Hydroxymethyl)-6-methylpyridin-3-ol	Alkaloids	Pyridine alkaloids	C7H9NO2	3	0.566	42097-42-7	3652.17	7331.2	9568.49	15050.83	14853.46	12069.24	1.30221018	0.033813	0.22399846	0.489638589	-1.03021083	down	-	-
MW0011639	(S)-Abscisic acid	Organic acids	Organic acids	C15H20O4	3	0.5612	21293-29-8	92278.83	98845.55	118593.41	15620.93	19301.05	22404.04	1.61472714	0.00615886	0.13374656	5.402743641	2.433692228	up	C06082	ko00906,ko01100,ko01110,ko04075
MW0008605	N-[(4-Hydroxy-3-methoxyphenyl)methyl]octanamide	Phenolic acids	Phenolic acids	C16H25NO3	3	0.5557	58493-47-3	0.47	0.75	0.15	0.57	3.62	1.06	1.06345161	0.30363033	0.57816876	0.260952381	-1.93814153	down	-	-
ZINC1335935	Ancistrocladidine	Heterocyclic compounds	Heterocyclic compounds	C25H27NO4	3	0.5555	52659-52-6	2256.27	7763.48	11855.86	12488.88	47209.33	64796.92	1.26433658	0.15204686	0.43673877	0.175714584	-2.50869416	down	-	-
MW0007885	N-(1-Hydroxy-2-oxo-2-(2-thienyl)ethyl)benzenesulfonamide	Benzene and substituted derivatives	Benzene and substituted derivatives	C12H11NO4S2	3	0.5513	303104-25-8	24219.97	34882.12	11275.94	250.46	1097.79	3605.23	1.43142472	0.082532	0.32686822	14.20779533	3.828610799	up	-	-
MW0109398	Pro-Lys	Amino acids and derivatives	Amino acids and derivatives	C11H21N3O3	3	0.5499	71227-70-8	405.88	43.3	240.3	325.77	433.02	1069.89	1.0058088	0.23972841	0.52360483	0.377036988	-1.40722203	down	-	-
INC10000712	Dimethylcurcumin	Phenolic acids	Phenolic acids	C23H24O6	3	0.5456	52328-98-0 | 917813-54-8 | 115851-85-9	824.7	636.44	585.41	169.56	110.52	292.62	1.4908817	0.00705815	0.13569	3.573511437	1.837342406	up	-	-
MW0133087	3-Hydroxy-3',4'-dimethoxyflavone	Flavonoids	Flavones	C17H14O5	3	0.5406	6889-80-1	29782.43	18043.4	20992.08	6903.33	10169.51	3976.2	1.4574666	0.0281134	0.20604042	3.269408486	1.709029641	up	-	-
MEDP1337	LysoPC 18:0	Lipids	LPC	C26H54NO7P	3	0.5335	19420-57-6	17701.4	20676.06	16802.86	7851.04	6052.95	10833.11	1.49877613	0.00553336	0.12649316	2.230670531	1.157477444	up	C04230	ko00564
MEDP1230	Glycidyl oleate	GL	MG	C21H38O3	3	0.5296	5431-33-4	67.72	111.71	135.55	6.5	45.29	52.26	1.1525027	0.05078241	0.2620491	3.027198462	1.597983261	up	C19426	-
MW0150673	Gymnemic acid I	Organic acids	Organic acids	C43H66O14	3	0.5293	122168-40-5	3452.51	12485.3	12659.75	713.25	7046.37	2246.8	1.0356948	0.17272575	0.4624187	2.857921215	1.514966146	up	C08947	-
MEDL01773	2,4,5-Trimethoxybenzaldehyde	Phenolic acids	Phenolic acids	C10H12O4	3	0.5266	4460-86-0	69866.5	60718.01	66704.17	28522.48	36528.26	26844.48	1.57727223	0.00099037	0.06912669	2.146887292	1.102246454	up	-	-
MEDL02176	6-Methylcoumarin	Lignans and Coumarins	Coumarins	C10H8O2	3	0.5216	92-48-8	12946.22	7792.75	16446.03	3247.47	2124.41	3108.3	1.53808176	0.05994995	0.28410222	4.384930509	2.132553979	up	-	-
MW0107842	Leuprorelin	Benzene and substituted derivatives	Benzene and substituted derivatives	C59H84N16O12	3	0.5137	53714-56-0	1467.91	1632.97	547.82	214.74	310.37	898.05	1.11383301	0.14987289	0.43473176	2.563801681	1.358284669	up	C07612	-

MW0160847	2-Hydroxy-3-(phosphonooxy)propyl octadec-9-enoate	GL	PA	C21H41O7P	3	0.5016	22002-79-5	115477.99	67900.36	79995.62	41570.39	28923.47	45558.29	1.45063488	0.0620312	0.28817563	2.269444987	1.182339516	up	-	-
MEDL02597	6"-O-Malonylglycitin	Flavonoids	Isoflavones	C25H24O13	3	0.5016	137705-39-6	159859.07	121928.63	137196.07	7189.1	5359.22	48357.8	1.41741456	0.00315373	0.10261813	6.879173554	2.782235254	up	C16197	ko00943
MW0138670	kaempferol 7-O-[illegible]	Flavonoids	Flavonols	C21H20O11	2	0.9425	-	40134.4	73157.81	81651.86	7041.43	17188.29	19481.45	1.45853514	0.04777367	0.25522731	4.459822741	2.15698637	up	-	-
MW0156730	Ser-Glu-Glu-Glu-Ile	Amino acids and derivatives	Amino acids and derivatives	C24H39N5O13	2	0.8894	-	0.73	3.11	5.56	4.32	16.23	8.12	1.08308046	0.20099354	0.48887189	0.327868852	-1.60880924	down	-	-
MW0151213	HoPhe-His-OH	Others	Lactones	C21H20N4O6	2	0.8858	-	21371.26	26190.19	19625.52	3882.99	6546.86	15443.38	1.31271603	0.03842645	0.23094812	2.596775509	1.376721298	up	-	-
MW0110180	Tumonoic acid I	Organic acids	Organic acids	C27H47NO7	2	0.8744	-	15618.41	15989.98	14985.83	7245.59	5273.2	8712.28	1.53379458	0.00906682	0.14235878	2.194624199	1.133973918	up	-	-
MW0158163	Trp-His-Leu	Amino acids and derivatives	Amino acids and derivatives	C23H30N6O4	2	0.8741	-	3368.75	2709.08	2642.64	15352.74	12806.11	8338.68	1.57326286	0.0441128	0.24760945	0.238933155	-2.06532103	down	-	-
MW0153174	Lys-Tyr-Gln	Amino acids and derivatives	Amino acids and derivatives	C20H31N5O6	2	0.8678	-	4.36	891.3	1022.76	1.01	3.03	4.61	1.22469282	0.18480287	0.47289372	221.782659	7.793002756	up	-	-
MW0158647	Tyr-Tyr-Asp	Amino acids and derivatives	Amino acids and derivatives	C22H25N3O8	2	0.8543	-	9170.13	8126.63	10613.32	22350.76	13568.99	20476.38	1.45973965	0.06208943	0.28817563	0.494893533	-1.01480991	down	-	-
MW0054380	Ethyl alpha-linolenate	Lipids	Free fatty acids	C20H34O2	2	0.852	-	3681.21	2140.61	1523.65	1207.18	1049.82	1190.31	1.30240267	0.17938935	0.46794913	2.130783132	1.091383765	up	-	-
MW0158529	Tyr-Leu-Thr-Arg	Amino acids and derivatives	Amino acids and derivatives	C25H41N7O7	2	0.8517	-	1446.52	306.32	973.24	3287.06	8367.33	7558.58	1.44093905	0.06762645	0.29786568	0.141887485	-2.81718075	down	-	-
MW0110983	D-erythro-Sphingosine C-20	Lipids	SPH	C20H41NO2	2	0.8387	-	5829.88	13261.77	8225.96	954.07	2518.35	703.63	1.48529853	0.06384732	0.29035184	6.541494953	2.709620378	up	-	-
MW0144702	Ala-Phe-Tyr-Arg	Amino acids and derivatives	Amino acids and derivatives	C27H37N7O6	2	0.8342	-	8468.21	7493.93	12893.44	1121.78	2622.9	1501.44	1.54445276	0.03431866	0.22531832	5.500365985	2.459527616	up	-	-
MW0158578	Tyr-Phe-Thr-Lys	Amino acids and derivatives	Amino acids and derivatives	C28H39N5O7	2	0.8283	-	20311.37	23851.85	22226.19	7535.64	12858.81	6122.33	1.47916883	0.01081328	0.14566058	2.503675409	1.324047535	up	-	-
MW0008784	N(4)-Acetylsulfamethazine	Benzene and substituted derivatives	Benzene and substituted derivatives	C14H16N4O3S	2	0.828	-	80240.58	60052.09	115588.51	71.87	594.2	443.36	1.58790684	0.0346264	0.22565754	230.6420234	7.849511588	up	-	-
MW0150294	Gly-Arg-Ala	Amino acids and derivatives	Amino acids and derivatives	C11H22N6O4	2	0.8261	-	9980.52	2740.09	2529.31	7225.79	16291.38	8231.67	1.0679707	0.22029739	0.50486336	0.48032999	-1.05790221	down	-	-
MW0150368	Gly-Gly-Lys-Pro-Asn	Amino acids and derivatives	Amino acids and derivatives	C19H33N7O7	2	0.8139	-	203.46	288.58	282.59	102.74	151.33	26.39	1.22685153	0.02538133	0.19715214	2.761998146	1.465712351	up	-	-
MW0157661	Thr-His-Gly	Amino acids and derivatives	Amino acids and derivatives	C12H19N5O5	2	0.8054	-	19.06	257.94	15.79	2.93	6.1	1.35	1.31156548	0.36122578	0.62453259	28.20712909	4.817987932	up	-	-
MW0150450	Gly-Phe-His	Amino acids and derivatives	Amino acids and derivatives	C17H21N5O4	2	0.79	-	41.55	10.37	11.75	4.13	7.95	7.67	1.16220224	0.2859203	0.56516477	3.223797468	1.688761111	up	-	-
MW0144707	Ala-Pro-Arg-Lys-Lys	Amino acids and derivatives	Amino acids and derivatives	C26H50N10O6	2	0.7893	-	47305.23	33207.4	26171.44	8973.8	6258.51	9159.25	1.56802029	0.04489717	0.24947897	4.373810859	2.128890834	up	-	-
MW0103299	(-)-Neplanocin A	Nucleotides and derivatives	Nucleotides and derivatives	C11H13N5O3	2	0.7876	-	6787.88	6300.66	5216.86	262.58	775.98	156.76	1.55315812	0.00250325	0.09520967	15.31422548	3.936800499	up	-	-
MW0144772	Ala-Tyr-Gln	Amino acids and derivatives	Amino acids and derivatives	C17H24N4O6	2	0.7805	-	392.33	1274.79	1534.69	574.02	460.6	352.93	1.0076954	0.21950167	0.50486336	2.307527657	1.206347939	up	-	-
MW0156866	Ser-Phe-Ala	Amino acids and derivatives	Amino acids and derivatives	C15H21N3O5	2	0.7784	-	2334.98	1784.9	1097.42	3845.54	11432.62	3871.83	1.33682942	0.20524373	0.49133768	0.272444006	-1.87596834	down	-	-
MW0146023	Asp-Arg-His	Amino acids and derivatives	Amino acids and derivatives	C16H26N8O6	2	0.7783	-	2674.24	6886.6	6938.7	11314.52	14103.97	14481.48	1.35114489	0.01367685	0.15713627	0.413522617	-1.27396186	down	-	-
MW0149665	Gln-Gln-Gln-Glu-Gln	Amino acids and derivatives	Amino acids and derivatives	C25H41N9O12	2	0.7715	-	500.55	362.13	754.67	47.78	122.67	183.01	1.42566691	0.05474247	0.27218393	4.575765292	2.194013053	up	-	-
MW0155367	Phe-Trp-Glu	Amino acids and derivatives	Amino acids and derivatives	C25H28N4O6	2	0.7685	-	28915.24	24149.8	25868.77	7830.43	14732.08	12644.3	1.47683617	0.00608905	0.13374656	2.242004033	1.164788873	up	-	-
MW0149411	GalNAc1-3Gal1-[illegible]	Others	Saccharides	C28H48N4O21	2	0.7684	-	4416.39	2348.95	3872.83	1602.74	2307.37	1303.54	1.32292886	0.08155085	0.32588632	2.040445753	1.028884356	up	-	-
MW0156821	Ser-Leu-Leu-Ser-Phe	Amino acids and derivatives	Amino acids and derivatives	C27H43N5O8	2	0.7664	-	14102.68	14006.29	16564.84	3005.98	4161.75	7101.01	1.50231492	0.00365043	0.10719376	3.13088682	1.646571357	up	-	-
MW0110070	Trp-Leu-Leu	Amino acids and derivatives	Amino acids and derivatives	C23H34N4O4	2	0.7644	-	41388.38	43788.22	33612.29	10994.12	3021.1	26340.84	1.21679054	0.04504942	0.24969604	2.943520502	1.557542676	up	-	-
MW0145402	Arg-Ile-Thr-Val-Lys	Amino acids and derivatives	Amino acids and derivatives	C27H53N9O7	2	0.763	-	27260.53	4987.55	6457.75	1373.92	1773.97	3631.23	1.28138666	0.27649815	0.55700777	5.709565548	2.513380972	up	-	-
MW0152386	Leu-Ile-Tyr	Amino acids and derivatives	Amino acids and derivatives	C21H33N3O5	2	0.7629	-	272231.95	293223.38	274153.29	253.03	21355.97	308.56	1.44261006	9.7693E-06	0.02155107	38.30757712	5.259557876	up	-	-
MW0158670	Tyr-Val-Lys-Ala-Leu	Amino acids and derivatives	Amino acids and derivatives	C29H48N6O7	2	0.7601	-	297836.83	234560.71	337101.23	136203.37	103290.44	122036.72	1.5615245	0.02152212	0.18323322	2.405049361	1.266066504	up	-	-
MW0139564	trans-Resveratrol 4'-O-glucuronide	Others	Stilbene	C20H20O9	2	0.7601	-	38.5	28.68	48.7	6.78	8.22	24.69	1.33489913	0.03565033	0.22604178	2.91962711	1.545784122	up	-	-
MW0158949	Val-Asp-Tyr-Val-Leu	Amino acids and derivatives	Amino acids and derivatives	C29H45N5O9	2	0.7596	-	79.37	224.86	437.66	809.31	7261.7	2437.15	1.37320998	0.23465062	0.51815744	0.070601323	-3.82416097	down	-	-
MW0151338	Ile-Arg-Thr-Asp	Amino acids and derivatives	Amino acids and derivatives	C20H37N7O8	2	0.7593	-	3979.05	3915.18	4664.99	1969.23	1454.99	2271.31	1.53955196	0.0024914	0.09520967	2.205101193	1.140844863	up	-	-
MW0151465	Ile-Ile-Arg-Ser-Ser	Amino acids and derivatives	Amino acids and derivatives	C24H46N8O8	2	0.7591	-	44220.18	45787.98	53000.29	22318.99	11206.76	29573.44	1.36484914	0.02169456	0.18323322	2.266407065	1.180407004	up	-	-
MW0107729	Leu-Ile-Ile	Amino acids and derivatives	Amino acids and derivatives	C18H35N3O4	2	0.7585	-	1742.95	2511.98	2251.52	765.2	947.67	670.71	1.56693926	0.01690222	0.16736536	2.729696507	1.448740558	up	-	-
MW0109872	Thr-His-Arg	Amino acids and derivatives	Amino acids and derivatives	C16H28N8O5	2	0.7579	-	203.44	227.29	270.47	79.91	113.01	88.38	1.56938002	0.00815379	0.14010122	2.492712407	1.317716443	up	-	-

MW0152990	Lys-His-Ile-Glu-Glu	Amino acids and derivatives	Amino acids and derivatives	C28H46N8O10	2	0.7575	-	10873.22	13042.38	12133.06	1359.08	5110.83	5217.8	1.34015799	0.01126127	0.14569982	3.084321907	1.624953346	up	-	-
MW0145888	Asn-Ser-Leu-Arg	Amino acids and derivatives	Amino acids and derivatives	C19H36N8O7	2	0.7572	-	2840.52	1245.34	1634.72	92271.24	64729.08	48134.6	1.61046362	0.03534969	0.22604178	0.027886915	-5.16426786	down	-	-
MW0145779	Asn-His-Val-Asp	Amino acids and derivatives	Amino acids and derivatives	C19H29N7O8	2	0.7568	-	1721.86	1839.68	887.37	1033.13	140.34	202.68	1.23648899	0.06942083	0.30146134	3.232867057	1.692814182	up	-	-
MW0150496	Gly-Thr-Phe-Thr-Asp	Amino acids and derivatives	Amino acids and derivatives	C23H33N5O10	2	0.7566	-	176730.31	165740.97	161645.69	481047.66	459942.67	467514.82	1.63053981	6.0252E-06	0.02155107	0.357909213	-1.48233441	down	-	-
MW0155260	Phe-Leu-Tyr-Asp	Amino acids and derivatives	Amino acids and derivatives	C28H36N4O8	2	0.7563	-	66.4	83.55	67.06	811.95	506.92	742.48	1.61573334	0.02147713	0.18323322	0.105275669	-3.24775605	down	-	-
MW0145340	Arg-Glu-Arg-Val-Tyr	Amino acids and derivatives	Amino acids and derivatives	C31H51N11O9	2	0.7561	-	17484.79	8434.2	37274.28	10498.79	3482.07	5344.22	1.17561469	0.22381903	0.50849101	3.270013371	1.709296535	up	-	-
MW0152462	Leu-Pro-Arg-Leu-Pro	Amino acids and derivatives	Amino acids and derivatives	C28H50N8O6	2	0.7561	-	17.67	7.95	4.86	13.92	24.52	29.71	1.1695126	0.10850808	0.37111447	0.447248716	-1.16085075	down	-	-
MW0146129	Asp-His-Phe	Amino acids and derivatives	Amino acids and derivatives	C19H23N5O6	2	0.7559	-	32.01	111.32	219.62	45.28	27.9	15.19	1.13026725	0.23230545	0.51669106	4.107163064	2.038142227	up	-	-
MW0156675	Ser-Asp-Arg-Asp	Amino acids and derivatives	Amino acids and derivatives	C17H29N7O10	2	0.7551	-	258.91	731.98	348.64	121.72	228.71	121.48	1.30474248	0.1786279	0.46794913	2.838528533	1.505143244	up	-	-
MW0144458	Ala-Ala-Leu-Arg-Ala	Amino acids and derivatives	Amino acids and derivatives	C21H40N8O6	2	0.755	-	9680.12	11819.67	11130.55	3601.84	1186.27	7223.19	1.24313513	0.04648085	0.25471913	2.716636834	1.441821716	up	-	-
MW0150845	His-Asn-Ala-Glu	Amino acids and derivatives	Amino acids and derivatives	C18H27N7O8	2	0.7545	-	18029.63	36016.7	31782.15	1578.84	3001.62	1788.63	1.59436878	0.03875497	0.23106342	13.47578382	3.752297285	up	-	-
MW0151093	His-Thr-Gln-Glu	Amino acids and derivatives	Amino acids and derivatives	C20H31N7O9	2	0.7526	-	28357.05	25425.78	9732.87	7548.11	7485.64	6185.81	1.34229497	0.13413849	0.41013099	2.993261877	1.581718504	up	-	-
MW0157621	Thr-Glu-Arg-Glu	Amino acids and derivatives	Amino acids and derivatives	C20H35N7O10	2	0.7525	-	1609.31	2047.61	2383.88	7672.33	11660.54	4989.67	1.51544445	0.08608413	0.33403976	0.248362219	-2.00948237	down	-	-
MW0150223	Glu-Val-Tyr-Asp	Amino acids and derivatives	Amino acids and derivatives	C23H32N4O10	2	0.7524	-	45410	30615.99	16183.5	117724.69	70876.57	65206.77	1.36119883	0.06407926	0.29035184	0.363304069	-1.46075057	down	-	-
MW0155871	Pro-Gly-Phe	Amino acids and derivatives	Amino acids and derivatives	C16H21N3O4	2	0.7521	-	246256.78	231404.48	222848.2	93740.16	104873.05	114667.33	1.6120681	0.00015948	0.04138927	2.236045239	1.160949377	up	-	-
MW0150100	Glu-Phe-Glu	Amino acids and derivatives	Amino acids and derivatives	C19H25N3O8	2	0.7518	-	86.14	65.6	179.7	33.57	11.94	12.61	1.45470547	0.11670278	0.3839697	5.702684102	2.511641117	up	-	-
MW0109201	Phenylalanylcysteine	Amino acids and derivatives	Amino acids and derivatives	C12H16N2O3S	2	0.7511	-	51584.96	36164.76	46871.84	285.69	256.77	137.63	1.62566915	0.01027482	0.14566058	197.9466835	7.628968086	up	-	-
MW0155244	Phe-Leu-Asp-Lys	Amino acids and derivatives	Amino acids and derivatives	C25H39N5O7	2	0.7502	-	417.63	609.6	470.54	1144.06	1651.49	499.56	1.11079122	0.21144434	0.4976598	0.454543247	-1.13751053	down	-	-
MW0153085	Lys-Met-Val-Ser-Arg	Amino acids and derivatives	Amino acids and derivatives	C25H49N9O7S	2	0.7498	-	168.71	119.58	133.83	24.19	55.5	125.53	1.12527187	0.12163256	0.39305682	2.056914531	1.040481848	up	-	-
MW0150978	His-Ile-Phe	Amino acids and derivatives	Amino acids and derivatives	C21H29N5O4	2	0.7491	-	71247.01	51337.58	129300.53	39809.92	42275.84	35541.6	1.27573006	0.19501436	0.48243539	2.141382073	1.098542229	up	-	-
MW0153190	Lys-Tyr-Val-Lys	Amino acids and derivatives	Amino acids and derivatives	C26H44N6O6	2	0.7491	-	344.72	527.53	431.23	150.98	47.53	207.84	1.33692517	0.01372523	0.15713627	3.207776547	1.681573648	up	-	-
MW0158210	Trp-Met-Trp	Amino acids and derivatives	Amino acids and derivatives	C27H31N5O4S1	2	0.7485	-	87.65	119.23	27.26	178.15	266.47	145.22	1.20784471	0.06329355	0.28975661	0.396955106	-1.33295224	down	-	-
MW0149677	Gln-Glu-Asp-Glu-Arg	Amino acids and derivatives	Amino acids and derivatives	C25H41N9O13	2	0.7474	-	1463.63	637.22	9798.96	13486.21	11184.22	12731	1.20832504	0.0940067	0.34853575	0.318164573	-1.65215489	down	-	-
MW0151466	Ile-Ile-Asn	Amino acids and derivatives	Amino acids and derivatives	C16H30N4O5	2	0.7471	-	23687.38	22858.83	24721.83	3918.37	14609.13	16644.58	1.12605895	0.0902337	0.3408345	2.026267426	1.018824593	up	-	-
MW0150365	Gly-Gly-Asn-Glu-Glu	Amino acids and derivatives	Amino acids and derivatives	C18H28N6O11	2	0.7463	-	1085.6	1663.72	768.53	2166.24	2718.68	3662.87	1.40516402	0.04055395	0.23740616	0.411550822	-1.2808575	down	-	-
MW0151101	His-Thr-Phe-Cys-Ala	Amino acids and derivatives	Amino acids and derivatives	C25H35N7O7S	2	0.7459	-	319.88	518.2	95.41	20.57	42.87	69.46	1.36033249	0.1588126	0.4444937	7.02400301	2.812293463	up	-	-
MW0158633	Tyr-Thr-Val-Thr-Leu	Amino acids and derivatives	Amino acids and derivatives	C28H45N5O9	2	0.7451	-	2909.94	3306.2	2825.91	16609.36	19847.33	15402.88	1.62458807	0.00792785	0.14010122	0.17435644	-2.51988844	down	-	-
MW0058413	1-(9Z-nonadecenoyl)-glycero-3-phosphoethanolamine	GL	PE	C24H48NO7P	2	0.7446	-	873.8	561.55	1072.21	386.38	76.22	189.83	1.35715119	0.03250014	0.21978922	3.843416152	1.942389195	up	-	-
MW0158364	Tyr-Ala-Thr-Asp	Amino acids and derivatives	Amino acids and derivatives	C20H28N4O9	2	0.7445	-	263466.92	368541.6	331726.98	34620.85	29034.77	26119.47	1.62385724	0.01059604	0.14566058	10.73499898	3.42425015	up	-	-
MW0159145	Val-Pro-Ser	Amino acids and derivatives	Amino acids and derivatives	C13H23N3O5	2	0.7436	-	36779.72	27409.5	15448.95	35709.03	80090.89	87853.83	1.25990788	0.11231952	0.37770758	0.391046912	-1.3545864	down	-	-
MW0150080	Glu-Lys-Phe	Amino acids and derivatives	Amino acids and derivatives	C20H30N4O6	2	0.7432	-	10788.89	26402.85	9230.48	1507.94	3190.99	8237.74	1.24888163	0.16879364	0.45772436	3.588421132	1.843349213	up	-	-
MW0126110	1-Phenylpiperazine	Heterocyclic compounds	Heterocyclic compounds	C10H14N2	2	0.743	-	3990.96	44.73	17.03	4414.73	1407.5	2912.23	1.05646666	0.38725709	0.64353232	0.46399205	-1.10782801	down	-	-
MW0149992	Glu-Glu-Gly-Gln-Arg	Amino acids and derivatives	Amino acids and derivatives	C23H39N9O11	2	0.7429	-	5701.41	4758.72	4053.82	3467.69	1954.45	1176.39	1.30667322	0.03813128	0.23077529	2.199573238	1.137223639	up	-	-
MW0146048	Asp-Asn-Val	Amino acids and derivatives	Amino acids and derivatives	C13H22N4O7	2	0.7413	-	329816.89	311737.1	516017.17	125646.01	69692.76	100026.19	1.54534527	0.04114921	0.23825498	3.919121483	1.970530294	up	-	-
MW0155806	Pro-Asp-Asn	Amino acids and derivatives	Amino acids and derivatives	C13H20N4O7	2	0.7405	-	20873.56	7661.77	12206.25	265.79	1141.38	2165.54	1.45186071	0.08267447	0.32703025	11.40355081	3.511411212	up	-	-
MW0109233	Phe-Trp	Amino acids and derivatives	Amino acids and derivatives	C20H21N3O3	2	0.7402	-	27.87	116.85	74.36	40.88	23.85	29.58	1.01232311	0.24439046	0.52751991	2.322977415	1.215975128	up	-	-

MW0158652	Tyr-Tyr-Leu	Amino acids and derivatives	Amino acids and derivatives	C24H31N3O6	2	0.7398	-	332185.62	315962.51	383843.04	958.26	681	1064.07	1.63118064	0.00353852	0.10620363	381.7481292	8.576477277	up	-	-
MW0151387	Ile-Cys-Phe-Arg	Amino acids and derivatives	Amino acids and derivatives	C24H39N7O5S	2	0.7366	-	132057.57	135591.45	112786.3	45769	40867.61	61698.06	1.5711097	0.0012997	0.07855195	2.564709383	1.358795358	up	-	-
MW0152982	Lys-His-Ala-Val-Ser	Amino acids and derivatives	Amino acids and derivatives	C23H40N8O7	2	0.7327	-	4067.47	4016.6	6609.19	1231.01	596.64	817.81	1.56123699	0.0373751	0.22934484	5.554141813	2.473564014	up	-	-
MW0153183	Lys-Tyr-Phe	Amino acids and derivatives	Amino acids and derivatives	C24H32N4O5	2	0.7327	-	604.95	318.5	1615.33	3790.61	6242.3	1763.53	1.31804565	0.13034695	0.40613752	0.215215777	-2.21614425	down	-	-
MW0155920	Pro-Lys-Ala	Amino acids and derivatives	Amino acids and derivatives	C14H26N4O4	2	0.7316	-	5535.02	4713.27	13174.69	1613.01	1602.47	1878.28	1.48628992	0.15130749	0.43582171	4.598367414	2.201121743	up	-	-
MW0107448	Ile-Leu-Asn	Amino acids and derivatives	Amino acids and derivatives	C16H30N4O5	2	0.7302	-	22619.02	4814.5	3765.89	1504.13	1447.66	2716.28	1.2455611	0.2984781	0.57431373	5.504415083	2.460589267	up	-	-
MW0152387	Leu-Ile-Tyr-Thr-Asn	Amino acids and derivatives	Amino acids and derivatives	C29H46N6O9	2	0.7273	-	9351.31	12521.64	10405.14	4312.52	2903.62	4832.38	1.54122578	0.00630495	0.13374656	2.679008708	1.421699271	up	-	-
MW0158395	Tyr-Asn-His	Amino acids and derivatives	Amino acids and derivatives	C19H24N6O6	2	0.7266	-	138649.07	101706.07	156628.29	9.66	19357.85	474.62	1.29041311	0.00858089	0.14010122	20.00709752	4.322439982	up	-	-
MW0159064	Val-Leu-His-Arg	Amino acids and derivatives	Amino acids and derivatives	C23H41N9O5	2	0.7264	-	37.73	37.73	37.73	262.78	188.64	37.73	1.14790586	0.19893107	0.48595854	0.231401411	-2.11153044	down	-	-
MW0110042	Tris(2-butoxyethyl) phosphate	Others	Lactones	C18H39O7P	2	0.7256	-	832.77	1223.99	786.81	333.61	380.82	75.4	1.31988902	0.0194185	0.17648958	3.600230429	1.848089248	up	C14446	-
MW0110230	Tyr-Val-Arg	Amino acids and derivatives	Amino acids and derivatives	C20H32N6O5	2	0.7255	-	70874.41	65903.73	76666.71	7190.05	289.76	23.43	1.38207458	0.00010148	0.04121132	28.44702422	4.830205838	up	-	-
MW0144533	Ala-Gln-Asp	Amino acids and derivatives	Amino acids and derivatives	C12H20N4O7	2	0.7241	-	213051.85	225630.32	225820.54	99475.66	82154.31	98621.6	1.61522155	9.2337E-05	0.04121132	2.371093621	1.245552628	up	-	-
MW0142517	2-O-alpha-L-Rhamnopyranosyl-D-glucopyranose	Others	Saccharides	C12H22O10	2	0.7225	-	71474.21	48023.39	79062.05	9949.29	20552.32	13856.74	1.5451219	0.02253801	0.18529393	4.476263206	2.16229487	up	C08244	-
MW0109104	Phe-Gln-Lys	Amino acids and derivatives	Amino acids and derivatives	C20H31N5O5	2	0.7221	-	15468.73	11621.45	17817.22	5403.18	6673.82	6426.35	1.55331334	0.03467574	0.22565754	2.426987545	1.279166705	up	-	-
MW0149384	Gal1-3Gal1-4GlcSp	Others	Saccharides	C20H35N3O16	2	0.7217	-	5596.23	4262.7	2594.93	629.03	898.82	1076.94	1.53757526	0.06037936	0.28544343	4.781137827	2.257353995	up	-	-
MW0158119	Trp-Asp-Val	Amino acids and derivatives	Amino acids and derivatives	C20H26N4O6	2	0.7198	-	14942.44	9746.76	11906.6	2516.63	9736.08	2406.73	1.24734135	0.07461033	0.31230283	2.496398225	1.319848091	up	-	-
MW0148055	Cys-Phe-His	Amino acids and derivatives	Amino acids and derivatives	C18H23N5O4S1	2	0.7197	-	7711.86	11842.26	7288.95	14446.88	16311.29	27130.37	1.36571087	0.10692805	0.3691444	0.46370266	-1.10872809	down	-	-
MW0155986	Pro-Pro-Val	Amino acids and derivatives	Amino acids and derivatives	C15H25N3O4	2	0.7169	-	1023.52	992.29	638.06	2970.21	3839.32	2499.42	1.55922594	0.02181885	0.18323322	0.285088007	-1.81052075	down	-	-
MW0150409	Gly-Leu-Ser-Pro-Lys	Amino acids and derivatives	Amino acids and derivatives	C22H40N6O7	2	0.715	-	609870.68	634092.29	736415.7	260454.04	267419.93	309974.67	1.60652787	0.00467982	0.11959415	2.363647293	1.241014771	up	-	-
MW0155158	Phe-Gln-Asp-Arg	Amino acids and derivatives	Amino acids and derivatives	C24H36N8O8	2	0.7145	-	3686.82	10126.96	12821.8	2959.05	3538.83	5153.43	1.0779368	0.2017399	0.48887189	2.286058821	1.192862525	up	-	-
MW0138858	Malvidin 3-(6''-p-coumarylglucoside)	Flavonoids	Anthocyanidins	C32H31O14+	2	0.7144	-	1048.19	1500.17	728.16	1323.94	6231.61	1919.12	1.06241332	0.31250504	0.58671158	0.345818905	-1.53191136	down	-	-
MW0150871	His-Asp-Glu	Amino acids and derivatives	Amino acids and derivatives	C15H21N5O8	2	0.7128	-	14413.17	25817.44	31202.33	56849.01	64915.93	68522.13	1.47933403	0.00409656	0.11299759	0.375395659	-1.41351613	down	-	-
MW0002271	2,4-Dihydroxybenzophenone	Benzene and substituted derivatives	Benzene and substituted derivatives	C13H10O3	2	0.7076	-	528.01	381.04	337.14	176.09	193.86	137.26	1.52592092	0.04182164	0.23972398	2.45695077	1.296868951	up	C14215	-
MW0153070	Lys-Met-Ala	Amino acids and derivatives	Amino acids and derivatives	C14H28N4O4S1	2	0.7055	-	7326.37	14383.04	14286.89	3382.02	11746.94	749.08	1.01300314	0.18159602	0.4693624	2.267049334	1.180815786	up	-	-
MW0156677	Ser-Asp-Gln	Amino acids and derivatives	Amino acids and derivatives	C12H20N4O8	2	0.7051	-	875	401.59	436.36	300.21	375.1	49.01	1.02053583	0.15545129	0.43991021	2.364907776	1.241783924	up	-	-
MW0151505	Ile-Lys-Arg-Asp-Phe	Amino acids and derivatives	Amino acids and derivatives	C31H51N9O8	2	0.7038	-	375.56	198.67	132.18	66.35	176.71	56.34	1.12965619	0.19619743	0.48304857	2.359418838	1.238431545	up	-	-
MW0159071	Val-Leu-Lys-Val-Leu	Amino acids and derivatives	Amino acids and derivatives	C28H54N6O6	2	0.7022	-	8944.43	9857.95	10621.49	1304.48	87.88	71.14	1.47076467	0.00014945	0.04121132	20.10513837	4.32949236	up	-	-
MW0152268	Leu-Asp-Pro-Asp-Tyr	Amino acids and derivatives	Amino acids and derivatives	C28H39N5O11	2	0.7001	-	9.28	953.09	694.61	3.44	8.07	1.9	1.27835027	0.19110889	0.47744759	123.5630127	6.949103142	up	-	-
MEDN1283	LPE(0:0/18:3)	GL	LPE	C23H42NO7P	3	0.9942	-	205310.94	207382.69	218515.32	96504.43	99011.8	104298.96	1.62796023	0.00012248	0.04121132	2.105326785	1.074044183	up	-	-
pmp001106	Vitexin-2''-O-glucoside	Flavonoids	Flavones	C27H30O16	3	0.9845	-	77843.17	125197.46	72380.35	33336.02	37689.04	31038.14	1.52985881	0.07298612	0.30844326	2.698533654	1.432175679	up	-	-
MW0155849	Pro-Glu-Gln	Amino acids and derivatives	Amino acids and derivatives	C15H23N3O8	3	0.9688	-	11382.93	8030.96	12955.31	23297.64	16040.62	27636.63	1.40338453	0.05944403	0.2829206	0.483303519	-1.0489986	down	-	-
MW0049494	1-Myristoyl-3-a-linolenoyl-sn-glycerol	GL	DG	C35H62O5	3	0.9464	-	564.92	408.82	298.45	298.64	81.05	56.07	1.25941693	0.06298045	0.28941965	2.919474022	1.545708474	up	-	-
MW0132554	3,4,8,9,10-pentahydroxy-6-oxo-2-{5,6,7-trihydroxy-1-oxo-3-[1,2,3-trihydroxy-4-(sulfooxy)butyl]-1H-isochromen-8-yl}-6H-benzo[c]chromene-1-carboxylic acid	Organic acids	Organic acids	C27H20O21S	3	0.9413	-	1244.71	4377.47	6372.85	9823.15	12540.39	5422.8	1.08474329	0.11544862	0.38182858	0.431688016	-1.21193905	down	-	-
MEDN1530	Cellobiose	Others	Saccharides	C12H22O11	3	0.9362	-	371137.06	363618.77	484000.02	118940.35	174326.29	178965.03	1.54772092	0.01131348	0.14569982	2.580843106	1.36784244	up	-	-
MW0061134	PG(18:0/22:5(4Z,7Z,10Z,13Z,16Z))	GL	PG	C46H81O10P	3	0.9102	-	22810.99	42253.5	67452.23	65178.33	142421.41	76326.2	1.15921081	0.16032352	0.44740503	0.46672988	-1.09934026	down	-	-

MW0132077	3,4,5-trihydroxy-6-{[3-hydroxy-2-(2-hydroxypropan-2-yl)-7-oxo-2H,3H,7H-furo[3,2-g]chromen-9-yl]oxy}oxane-2-carboxylic acid	Organic acids	Organic acids	C20H22O12	3	0.8956	-	926.93	438.07	936.55	1910.56	1337.92	1358.93	1.30688619	0.03772185	0.2307381	0.499532275	-1.0013502	down	-	-
MEDP1343	LPC(0:0/18:3)	Lipids	LPC	C26H49NO7P	3	0.8749	-	91270.71	88749.34	62514.19	10937.8	7812.19	9771.8	1.61575988	0.01547961	0.16393407	8.503471907	3.088052004	up	-	-
MW0060675	PE-NMe2(20:4(5Z,8Z,11Z,14Z)/22:6(4Z,7Z,10Z,13Z,16Z,19Z))	GL	PE	C49H78NO8P	3	0.8602	-	46975.76	28610.28	35563.69	6446.84	2345.08	7415.4	1.52324316	0.02062351	0.1807673	6.857995646	2.777786988	up	-	-
MW0132871	3-[2-(3,4-dihydroxyphenyl)-5,7-dihydroxy-4-oxo-3,4-dihydro-2H-1-benzopyran-8-yl]-5,7-dihydroxy-2-phenyl-3,4-dihydro-2H-1-benzopyran-4-one	Benzene and substituted derivatives	Benzene and substituted derivatives	C30H22O10	3	0.8364	-	155344.16	8581.69	60447.99	170277.87	195592.77	183106.63	1.05655222	0.12429954	0.39596359	0.408712441	-1.29084194	down	-	-
MW0161844	Cocaine(1+)	Organic acids	Organic acids	C17H22NO4+	3	0.8077	-	5654.22	7251.78	7194.22	19365.87	23103.9	13821.96	1.54692621	0.04212835	0.2407646	0.357072344	-1.48571169	down	-	-
MEDP1664	Glu-Leu-Arg	Amino acids and derivatives	Amino acids and derivatives	C17H32N6O6	3	0.7862	-	219766.75	113614.2	177704.66	57017.21	37230.78	82621.01	1.42443442	0.05241707	0.26669928	2.889627973	1.530883764	up	-	-
MW0053678	beta-D-glucosyl-N-(hexacosanoyl)sphingosi[illegible]	Lipids	Cer	C50H97NO8	3	0.7509	-	2906.7	1679.84	2904.92	210.11	455.04	1017.2	1.40571728	0.0229848	0.18655341	4.452973519	2.154769032	up	C01190	ko00600,ko01100
ZINC3383085	[(1S,3S,5R,6S)-8-methyl-6-[(E)-2-methylbut-2-enoyl]oxy-8-azabicyclo[3.2.1]octan-3-yl] (E)-2-methylbut-2-enoate	Alkaloids	Tropan alkaloids	C18H27NO4	3	0.7495	-	3672.33	1278.24	341.69	4258.34	5711.84	3957.99	1.1451466	0.08154747	0.32588632	0.379968079	-1.39604987	down	-	-
MW0154958	(2R,3R,4S,5S,6R)-2-[[(1R,2S,6S,9S,10S,11S,14S,15S,17S,18S,20S,23R,24S)-10,17-dihydroxy-6,10,23-trimethyl-4-azahexacyclo[12.11.0.02,11.04,9.015,24.018,23]pentacosan-20-yl]oxy]-6-(hydroxymethyl)oxane-	Alkaloids	Alkaloids	C33H55NO8	3	0.7491	-	165688.2	180502.63	225781.46	49003.34	57069.98	60564.23	1.60698616	0.01486925	0.16129107	3.432433386	1.779231722	up	C17882	-
MEDP1024	Leu-Val	Amino acids and derivatives	Amino acids and derivatives	C11H22N2O3	3	0.7449	-	95.38	65.59	224.77	495.11	262.77	185.65	1.16718913	0.17411686	0.46285781	0.408826428	-1.29043963	down	-	-
MEDP1407	Carnitine C14:2	Lipids	CAR	C21H37NO4	3	0.7436	-	21518.5	21334.56	33209.85	6323.22	1682.61	3329.86	1.51417064	0.0220661	0.1840371	6.710037942	2.746320924	up	-	-
MW0150336	Gly-Cys-His	Amino acids and derivatives	Amino acids and derivatives	C11H17N5O4S1	3	0.7423	-	11701.42	15794.77	17023.21	34978.4	45553.85	31077.91	1.54080757	0.02428222	0.19303267	0.398883041	-1.32596231	down	-	-
MW0166807	4-Dodecanoylsucrose	Others	Lactones	C24H44O12	3	0.737	-	4.87	61.12	51.46	119.27	226.61	189.19	1.23259603	0.02844398	0.20742952	0.21950399	-2.18768093	down	-	-
MW0166080	2-Acetyl-3-isobutanoyl-3',4-[illegible]	Others	Lactones	C28H46O15	3	0.737	-	185.47	276.79	240.22	86.23	145.55	68.41	1.42036875	0.01975129	0.17658189	2.340117925	1.226581233	up	-	-
ZINC9591161	4',5,5''-Trihydroxy-4''',7''-dimethoxy-7-(beta-D-glucopyranosyloxy)-8,3''' biflavone	Others	Ketone compounds	C38H32O15	3	0.7369	-	11.71	5.8	12.01	53.89	39.36	39.69	1.54865656	0.0097195	0.14545906	0.222055062	-2.17101063	down	-	-
MW0161494	Mphpv II	Others	Ketone compounds	C17H18O6	3	0.7293	-	1169.3	2410.11	3860.54	335.53	110.81	89.02	1.51776145	0.09624784	0.35181896	13.89709728	3.79671167	up	-	-
MW0130644	2-{3-[(6-carboxy-3,4,5-trihydroxyoxan-2-yl)oxy]-4-methoxyphenyl}-3-hydroxy-5-sulfino-3,4-dihydro-2H-1-benzopyran-7-olate	Benzene and substituted derivatives	Benzene and substituted derivatives	C22H24O13S	3	0.7292	-	17876.67	16532.97	14232.88	1656.1	1365.18	4519.96	1.52898896	0.00073798	0.06511911	6.450201824	2.689344302	up	-	-
MW0145720	Asn-Gln-Gln	Amino acids and derivatives	Amino acids and derivatives	C14H24N6O7	3	0.7284	-	490.42	703.62	311.24	48.89	512.53	135.73	1.01906153	0.2163071	0.50316295	2.159190992	1.110490863	up	-	-
MW0131533	3,4,5-trihydroxy-6-({4-hydroxy-2-oxo-2H-furo[2,3-h]chromen-8-yl}oxy)oxane-2-carboxylic acid	Organic acids	Organic acids	C17H14O11	3	0.7263	-	3249.66	228.91	1726.92	3559.26	10856.99	8452.17	1.19170504	0.09595201	0.35181896	0.227627882	-2.13525081	down	-	-
MW0150383	Gly-Ile-Asp-Lys-Phe	Amino acids and derivatives	Amino acids and derivatives	C27H42N6O8	3	0.7252	-	1844.09	1228.82	1476.89	451.82	1196.61	429.46	1.27686829	0.06246522	0.28828091	2.189625052	1.130683846	up	-	-
MW0009319	P,P-Dioctyldiphenylamine	Benzene and substituted derivatives	Benzene and substituted derivatives	C28H43N	3	0.7227	-	40.04	113.14	7.66	2306.29	1764.28	2837.44	1.52846267	0.01764116	0.17028658	0.023283116	-5.42457202	down	-	-
MEDP1438	Carnitine C6:1	Lipids	CAR	C13H23NO4	3	0.7188	-	351335.71	410807.39	500083.19	163603.25	192736.51	201511.79	1.56616985	0.02585593	0.19834267	2.262656239	1.178017415	up	-	-

MW0167761	[(2R)-2-icosanoyloxy-3-[(6Z,9Z,12Z,15Z)-octadeca-6,9,12,15-tetraenoyl]oxypropyl] (7Z,10Z,13Z,16Z,19Z)-docosa-7,10,13,16,19-pentaenoate	GL	TG	C63H104O6	3	0.7174	-	2662.27	2845.48	1704.59	295.93	620.38	659.75	1.52124034	0.02464653	0.19487546	4.576183648	2.19414495	up	-	-
MW0143154	4-(8-Methyl-9H-1,3-dioxolo(4,5-h)(2,3)benzodiazepin-5-yl)benzenamine	Benzene and substituted derivatives	Benzene and substituted derivatives	C17H15N3O2	3	0.7154	-	10064.27	7678.93	20231.84	111769.26	35582.41	62609.13	1.4492094	0.11989136	0.39041622	0.180867286	-2.46699661	down	C15040	-
MW0165814	dTDP-4-keto-5-methyl-beta-L-rhamnose	Nucleotides and derivatives	Nucleotides and derivatives	C17H24N2O15P2-2	3	0.7151	-	927.73	3487.15	2396.62	424.55	767.19	258.94	1.35878801	0.13250834	0.40854422	4.695384234	2.231243222	up	-	-
MW0168059	(E,2Z)-2-[amino(carboxy)methylidene]-5-oxopent-3-enoate	Others	Others	C7H6NO5-	3	0.714	-	1617.44	1718.58	6058.68	4371.01	11347.86	11030.33	1.20281891	0.11100444	0.37557638	0.351214242	-1.50957675	down	-	-
MW0057738	PC(24:1(15Z)/P-18:1(11Z))	GL	PC	C50H96NO7P	3	0.7131	-	3667.85	2777.35	5683.22	592.39	715.03	696.82	1.5855583	0.05890193	0.28189599	6.051381072	2.597264438	up	-	-
MW0003283	2-Naphthoic acid	Benzene and substituted derivatives	Benzene and substituted derivatives	C11H8O2	3	0.7067	-	194885.9	202688.72	236352.91	59885.94	58084.45	59772.78	1.62606785	0.00683614	0.13456926	3.566536649	1.834523799	up	C14101	-
MEDP1322	LysoPC(22:5(7Z,10Z,13Z,16Z,19Z))	Lipids	LPC	C30H52NO7P	3	0.7067	-	62.28	124.26	12.96	205.33	266.92	123.82	1.1520235	0.06922098	0.3008896	0.334692234	-1.57909302	down	C04230	ko00564
MW0009706	N-[3-[(2-oxo-1-naphthalenylidene)methylamino]phenyl]-2-phenylpropanamide	Benzene and substituted derivatives	Benzene and substituted derivatives	C26H22N2O2	3	0.704	-	38.33	179.34	19.75	294.2	307.86	188.78	1.25329067	0.04694099	0.25471913	0.300212432	-1.73594437	down	C71361	-
MW0104523	2-amino-4-({1-[(carboxymethyl)-C-hydroxycarbonimidoyl]-2-[(1,3-dihydroxy-1-phenylpropan-2-yl)sulfanyl]ethyl}-C-hydroxycarbonimidoyl)butanoic acid	Organic acids	Organic acids	C19H27N3O8S	3	0.7038	-	101.89	88.34	45.95	188.37	349.73	391.47	1.46960981	0.05621959	0.27745061	0.254074465	-1.97667671	down	-	-
MW0144275	Acetyl-maltose	Others	Saccharides	C14H24O12	3	0.7038	-	20297.3	14427.61	18298.61	83.81	2185.06	3522.15	1.28506348	0.00329269	0.10290142	9.156162472	3.194743063	up	C02130	-
MW0141699	1-Caffeoyl-beta-D-glucose	Phenolic acids	Phenolic acids	C15H18O9	3	0.7019	-	11080.52	6249.49	36119.1	6304.79	3665.56	3367.63	1.18045051	0.28491981	0.56421284	4.007286711	2.002625734	up	C10433	-
MW0140260	(25S)-3-ketocholest-4-en-26-al	Others	Ketone compounds	C27H42O2	3	0.7016	-	1961225.21	2009006.52	2594964.69	381676.7	519269.84	544611.17	1.60494395	0.01041181	0.14566058	4.541635643	2.183211969	up	C21304	-
MW0112053	NCGC00381092-01_C15H20O8_2-Acetyl-5-hydroxy-3-methylphenyl beta-D-glucopyranoside	Benzene and substituted derivatives	Benzene and substituted derivatives	C15H20O8	3	0.7014	-	9451.66	12856.05	16221.93	3331.44	479.52	2017.61	1.42422717	0.0185674	0.17461247	6.610479071	2.72475483	up	-	-
MW0000450	6-Phosphogluconic acid tri(cyclohexylammonium) salt, Grade V	Alkaloids	Alkaloids	C15H26N2	3	0.7012	-	347804.96	379904.61	127192.48	122049.03	133798.84	122675.02	1.158107	0.1833456	0.47057636	2.258521407	1.175378588	up	-	-
MW0122491	7-Hydroxymethotrexate	Heterocyclic compounds	Heterocyclic compounds	C20H22N8O6	3	0.6989	-	288564.81	319164.94	313034.44	136932.74	1520.32	224.15	1.24081413	0.02500804	0.1956303	6.639621536	2.731101009	up	-	-
MW0150043	Glu-Ile-Trp	Amino acids and derivatives	Amino acids and derivatives	C22H30N4O6	3	0.6983	-	5773.53	3239.87	2280.75	1197.65	1280.56	1480.24	1.41847985	0.14260679	0.42367711	2.853174854	1.512568164	up	-	-
MW0146267	Asp-Thr-Arg-Asp	Amino acids and derivatives	Amino acids and derivatives	C18H31N7O10	3	0.6963	-	26253.87	12027.19	21982.95	8668.14	5513.12	8442.51	1.40766047	0.08937915	0.33936387	2.663747466	1.413457315	up	-	-
MW0165831	4-amino-demethoxy-Q6H2	Alcohol and amines	Alcohols	C38H59NO2	3	0.6949	-	151441.41	75745.61	122077.86	19888.26	2779.69	34616.02	1.32517789	0.03301023	0.22066836	6.097078816	2.608118196	up	-	-
MW0146223	Asp-Phe-Met	Amino acids and derivatives	Amino acids and derivatives	C18H25N3O6S1	3	0.6946	-	1580.45	2513.15	3150.87	452.09	573.01	577.63	1.56669192	0.05300127	0.2674595	4.520081361	2.176348741	up	-	-
MW0155247	Phe-Leu-Gln-Lys	Amino acids and derivatives	Amino acids and derivatives	C26H42N6O6	3	0.6937	-	2704.29	3462.05	3464.01	861.03	1242.41	1156.8	1.58257397	0.00591137	0.13106004	2.953877629	1.56261006	up	-	-
MW0153616	Met-Met-Asn	Amino acids and derivatives	Amino acids and derivatives	C14H26N4O5S2	3	0.6926	-	6221.86	5502.38	6794.32	1928.06	3079.26	826.66	1.39593022	0.00933476	0.14350153	3.174258396	1.666419574	up	-	-
MW0151247	3-[(1R,2S,3S,5Z,7S,8S,9Z,13S,14Z,17R,18R)-3,13,17-tris(2-carboxyethyl)-2,7,18-tris(carboxymethyl)-1,2,5,7,12,12,15,17-octamethyl-3,8,13,18,19,22-hexahydrocorrin-8-yl]propanoic acid	Organic acids	Organic acids	C45H60N4O14	3	0.6913	-	4021.22	2240.07	3342.66	4650.62	12437.87	5745.41	1.2383755	0.20781628	0.4943857	0.420600511	-1.24947749	down	C06399	ko00860,ko01100,ko01240

MW0152497	Leu-Ser-Phe-Gln-Asp	Amino acids and derivatives	Amino acids and derivatives	C27H40N6O10	3	0.6903	-	9563.76	3489.28	9957.2	3351.87	1086.97	875.81	1.3391486	0.09198579	0.34422501	4.329587085	2.114229441	up	-	-
MW0155317	Phe-Pro-Phe	Amino acids and derivatives	Amino acids and derivatives	C23H27N3O4	3	0.6862	-	266.87	170.46	45.85	178.06	586.31	766.09	1.07761063	0.17297157	0.46253718	0.315709003	-1.66333269	down	-	-
MW0105632	Antanapeptin A	Benzene and substituted derivatives	Benzene and substituted derivatives	C41H60N4O8	3	0.6844	-	101274.24	219.71	143.62	80333.75	97750.56	86881.33	1.13653136	0.24589462	0.5293161	0.383587736	-1.3823715	down	-	-
MW0114447	2-Methoxy-4-[2-propenyl]phenyl 6-O-beta-D-glucopyranosyl-beta-D-glucopyranoside	Benzene and substituted derivatives	Benzene and substituted derivatives	C22H32O12	3	0.6842	-	52534.92	36379.63	35760.93	13303.3	24788.73	18506.88	1.43297777	0.03316727	0.22077332	2.202789418	1.139331583	up	-	-
MW0162750	(2R,3S,4S,5R,6S)-2-(hydroxymethyl)-6-[6-[[(E)-4-hydroxy-3-methylbut-2-enyl]amino]purin-7-yl]oxane-3,4,5-triol	Alcohol and amines	Alcohols	C16H23N5O6	3	0.6822	-	28.76	41.64	101.69	91.45	171.55	142.44	1.23524628	0.07470389	0.31230283	0.424452447	-1.23632516	down	-	-
MW0157794	Thr-Ser-Arg-Ile-Ile	Amino acids and derivatives	Amino acids and derivatives	C25H48N8O8	3	0.6816	-	19292.8	18582.41	16840.73	4903.98	1795.8	4925.7	1.5109516	0.00062444	0.06185252	4.706553192	2.2346709	up	-	-
MW0106965	Glutamylisoleucyllysine	Amino acids and derivatives	Amino acids and derivatives	C17H32N4O6	3	0.6812	-	7191.67	4283.83	8984.66	4020.18	867.66	515.81	1.28759336	0.04901614	0.25767713	3.78635922	1.920811289	up	-	-
MW0145979	Asn-HoPhe-OH	Amino acids and derivatives	Amino acids and derivatives	C19H19N3O7	3	0.6777	-	160378.51	166600.03	161485.92	63025.42	81269.1	53941.14	1.57950696	0.00481221	0.11959415	2.464059494	1.30103709	up	-	-
MW0158416	Tyr-Asp-Phe	Amino acids and derivatives	Amino acids and derivatives	C22H25N3O7	3	0.6774	-	6040.35	6050.33	5466.38	5713.76	21389.18	30698.15	1.12764595	0.20703495	0.49365814	0.303749635	-1.71904542	down	-	-
MW0165186	2-azaniumylprop-2- [illegible]	Others	Lactones	C3H5NO2	3	0.6735	-	2450.53	216.11	2464.94	6813.65	4663.98	5129.29	1.1468827	0.01884605	0.17541932	0.309002512	-1.69430953	down	-	-
MW0145756	Asn-Gly-Phe	Amino acids and derivatives	Amino acids and derivatives	C15H20N4O5	3	0.6713	-	8529.06	4786.64	8230.67	4175.42	2528.88	1430.09	1.33723105	0.0436921	0.24666834	2.648799726	1.405338766	up	-	-
MW0149385	Gal1-3GalNAc1-3Gal1-4Gal1-4GlcSp	Others	Saccharides	C34H58N4O26	3	0.6687	-	26148.53	34729.32	28384.47	3374.09	11958.91	4729.66	1.47556025	0.00339407	0.10399056	4.449176729	2.153538406	up	-	-
MEDP1245	3-Hydroxycaprolactone	Others	Lactones	C12H16N2O4	3	0.6681	-	585.91	601.23	1248.32	348.36	231.64	446.83	1.306401	0.15648704	0.44088176	2.371823963	1.245996937	up	C03068	-
MW0143452	Azacyclonol	Benzene and substituted derivatives	Benzene and substituted derivatives	C18H21NO	3	0.6678	-	7101.41	9453.63	9549.06	18139.8	13961.53	22392.77	1.48157524	0.04880887	0.25758942	0.47902617	-1.06182362	down	-	-
MEDP1473	2H-Pyran-2-one, tetrahydro-4-hydroxy-4-methyl-, (R)-	Others	Lactones	C6H10O3	3	0.6665	-	105.41	265.77	152.73	17.58	169.75	35.24	1.06141447	0.21158674	0.4976598	2.353911129	1.235059853	up	-	-
MW0155306	Phe-Phe-Thr	Amino acids and derivatives	Amino acids and derivatives	C22H27N3O5	3	0.665	-	8301.1	13277.11	8663.88	5187.5	6824.13	2634.35	1.25949427	0.06544512	0.29463659	2.064873091	1.046053115	up	-	-
MW0154289	Nap-Met-OH	Amino acids and derivatives	Amino acids and derivatives	C23H22N2O6S	3	0.665	-	28253.92	19744.36	18812.32	9993.85	7409.31	10245.3	1.52728729	0.03966721	0.2343993	2.41643115	1.27287789	up	-	-
MW0152410	Leu-Leu-Val-Val-Tyr	Amino acids and derivatives	Amino acids and derivatives	C31H51N5O7	3	0.663	-	15673.29	12361.09	10376.96	992.87	1704.17	1953.11	1.59352367	0.01576662	0.16425584	8.260236767	3.046183135	up	-	-
MW0155273	Phe-Lys-His	Amino acids and derivatives	Amino acids and derivatives	C21H30N6O4	3	0.6615	-	20.92	77.83	24.58	119.84	290.79	404.3	1.42174562	0.10178373	0.36157643	0.151338152	-2.72415236	down	-	-
MW0151592	Ile-Thr-Gln	Amino acids and derivatives	Amino acids and derivatives	C15H28N4O6	3	0.6613	-	19	31.82	15.07	47.93	55.23	53.64	1.46469297	0.01501534	0.16197473	0.420216837	-1.25079413	down	-	-
MW0147988	Cys-Asn-Ser-Ala-Arg	Amino acids and derivatives	Amino acids and derivatives	C19H35N9O8S	3	0.6607	-	11368.7	13112.04	12263.36	29765.17	18693.35	31536.05	1.48692842	0.06751109	0.29786568	0.459332427	-1.12238946	down	-	-
MEDP0797	1- [illegible]	Quinones	Anthraquinone	C14H8O3	3	0.6596	-	73.43	117.59	132.44	52.48	76.38	9.97	1.06373008	0.07984506	0.32345454	2.329899878	1.220267959	up	C02980	-
MW0140356	2-Amino-3-hydroxy-4-methylbenzoyl-AMP	Nucleotides and derivatives	Nucleotides and derivatives	C18H21N6O9P	3	0.6573	-	6477.83	1938.7	1771.95	475.14	661.49	187.28	1.41740879	0.19444441	0.48243539	7.695749711	2.944061879	up	C21460	-
MW0001405	1,9-Pyrazoloanthrone	Benzene and substituted derivatives	Benzene and substituted derivatives	C14H8N2O	3	0.6562	-	39910.6	27317.53	51253.33	940.94	1051.65	677.65	1.62133674	0.03057447	0.21312989	44.37109024	5.471548097	up	-	-
MEDP1527	Carnitine C19:0	Lipids	CAR	C26H51NO4	3	0.656	-	8632.5	9727.71	7358.62	2495.15	1168.4	4167.25	1.41504709	0.00662819	0.13456926	3.284317056	1.715593406	up	-	-
MW0148768	dTDP-alpha-D-forosamine	Nucleotides and derivatives	Nucleotides and derivatives	C18H31N3O12P2	3	0.6547	-	6043.72	2039.29	9668.17	1102.61	3393.09	1560.82	1.08039084	0.21259931	0.49813497	2.930920727	1.551353948	up	C18034	ko01100,ko01110,ko01250
MW0163756	[(2S)-2-azaniumyl-2-carboxyethyl] [(2R)-2,3-bis[[(Z)-octadec-9-enoyl]oxy]propyl] phosphate	GL	DG	C42H78NO10P	3	0.6546	-	2862.89	6041.84	9774.22	15981.54	15569.98	11889.06	1.28950637	0.03276246	0.22066836	0.429988504	-1.21763001	down	-	-
MW0153333	Mallotinic acid	Organic acids	Organic acids	C34H26O23	3	0.6523	-	1475.05	1960.51	2115.73	1704.83	7906.48	4241.86	1.02928901	0.26327238	0.54380044	0.400723445	-1.31932118	down	C17521	-
MW0151011	His-Lys-His	Amino acids and derivatives	Amino acids and derivatives	C18H28N8O4	3	0.6504	-	23725.6	26380.01	26002.52	3264.84	7158.05	6646.35	1.55290038	0.00038738	0.05026788	4.45878844	2.156651749	up	-	-
MW0011962	NCGC00381263-01_C31H40O7_3,9-Diacetoxy-2,6,6,13-tetramethyl-12-oxotetracyclo[11.2.1.0~1,10~.0~2,7~]hexadec-8-yl benzoate	Benzene and substituted derivatives	Benzene and substituted derivatives	C31H40O7	3	0.6476	-	40359.82	39664.64	47171	9764.78	11643.47	12427.01	1.61808492	0.00298857	0.10260237	3.75925765	1.910447797	up	-	-

MW0148752	dTDP-4-oxo-2-deoxy-alpha-D-pentos-2-ene	Nucleotides and derivatives	Nucleotides and derivatives	C15H20N2O14P2	3	0.6472	-	5098.28	2673.58	8388.97	3356.32	1190.58	1561.06	1.19183684	0.16989376	0.45845338	2.645863758	1.403738776	up	C21352	ko01110
MW0150906	His-Gln-Thr-Glu	Amino acids and derivatives	Amino acids and derivatives	C20H31N7O9	3	0.6448	-	81.74	190.08	113.19	35.9	37.83	100.71	1.17024567	0.15388567	0.43887754	2.207119927	1.142165022	up	-	-
MW0169579	N-tridecanoyl-L-Homserine lactone	Others	Lactones	C17H31NO3	3	0.6442	-	320.13	505.5	599.31	240.61	132.71	83.69	1.40181472	0.0385268	0.23094812	3.117962408	1.640603534	up	-	-
MW0114191	CID 72682693	Organic acids	Organic acids	C18H31NO14S	3	0.6417	-	1440.75	1096.65	703.74	264.41	232.93	508.13	1.43873043	0.05698059	0.27904928	3.223507414	1.688631302	up	-	-
MW0143824	6-Desmethoxy hormothamnione [illegible]	Others	Lactones	C27H26O11	3	0.6412	-	4436.4	6961.78	6218.81	572.58	2438.67	462.64	1.41963553	0.00930032	0.14350153	5.071257294	2.342343472	up	-	-
MW0109304	Pro-Asn	Amino acids and derivatives	Amino acids and derivatives	C9H15N3O4	3	0.6371	-	165.38	107.2	133.4	28.06	21.73	108.72	1.23558226	0.07870119	0.32180689	2.561226421	1.356834798	up	-	-
MW0137929	Deoxysappanone B 7,3'-dimethyl ether	Others	Ketone compounds	C18H18O5	3	0.6353	-	3063.33	3380.42	2450.47	1060.2	1199.44	1660.12	1.51865413	0.01032848	0.14566058	2.269072596	1.182102767	up	-	-
MW0140138	(+)-Sceletium A4	Heterocyclic compounds	Heterocyclic compounds	C20H24N2O2	3	0.634	-	6653.26	5470.75	8689.51	2200.09	299.32	1099.16	1.40576789	0.01105754	0.14569982	5.783830799	2.532025348	up	C12259	-
MW0152327	Leu-Glu-Pro-His-Ile	Amino acids and derivatives	Amino acids and derivatives	C28H45N7O8	3	0.6316	-	23881.51	14901.76	48068.45	14057.12	8018.31	4816.45	1.27494808	0.17422455	0.46285781	3.229663378	1.691383803	up	-	-
MW0145407	Arg-Leu-Arg-Glu-Lys	Amino acids and derivatives	Amino acids and derivatives	C29H56N12O8	3	0.6272	-	8284.66	3644.12	4634.39	21986.85	14301.09	18663.72	1.49587725	0.01254165	0.14955072	0.30141346	-1.73018425	down	-	-
MW0152278	Leu-Cys-Ile	Amino acids and derivatives	Amino acids and derivatives	C15H29N3O4S1	3	0.6248	-	920.07	578.37	1616.01	879.07	101.26	324.28	1.03433764	0.19548746	0.48243539	2.38726516	1.255358819	up	-	-
MW0150048	Glu-Leu-Asn	Amino acids and derivatives	Amino acids and derivatives	C15H26N4O7	3	0.6246	-	22959.36	8955.57	15242.85	33647.54	37859.21	26675.67	1.31926785	0.03282506	0.22066836	0.480307778	-1.05796892	down	-	-
MW0003169	2-iodo-N-(6-methyl-1,3-benzothiazol-2-yl)benzamide	Benzene and substituted derivatives	Benzene and substituted derivatives	C15H11IN2OS	3	0.6242	-	11402.73	69.4	82.67	8603.91	9007.13	17535.9	1.14649849	0.17902088	0.46794913	0.328756927	-1.6049068	down	-	-
MEDL01752	Asparagoside C	Terpenoids	Steroidal [illegible]	C39H64O13	3	0.6237	-	125545.39	105106.46	148410.51	7856.86	32737.4	36570.83	1.41997366	0.0039441	0.11299604	4.912355574	2.296414992	up	-	-
MW0150513	Gly-Tyr-Asp	Amino acids and derivatives	Amino acids and derivatives	C15H19N3O7	3	0.6235	-	27396.64	29545.81	20358.61	8944.97	352.52	9120.22	1.14019361	0.00810375	0.14010122	4.197104852	2.069394505	up	-	-
MW0130153	Taxifolin 3-O-bata-xylopyranoside	Flavonoids	Flavones	C20H20O11	3	0.6228	-	178.95	644.92	399.48	129.14	210.69	108.07	1.20121536	0.18978288	0.47658128	2.73130163	1.449588646	up	-	-
MW0155234	Phe-Ile-Phe-Met-Gly	Amino acids and derivatives	Amino acids and derivatives	C31H43N5O6S	3	0.6219	-	18751.47	16923.61	23113.28	4766.05	4449.39	5998.53	1.60433915	0.01157113	0.14569982	3.864103847	1.950133867	up	-	-
MW0150351	Gly-Glu-Gln	Amino acids and derivatives	Amino acids and derivatives	C12H20N4O7	3	0.6217	-	26885.08	12462.19	6915.19	5517.28	553.56	4432.3	1.17204927	0.17708256	0.46644075	4.404631377	2.139021285	up	-	-
ZINC8604738	Terrestrin G	Benzene and substituted derivatives	Benzene and substituted derivatives	C41H28O11	3	0.6208	-	2047.87	942.98	967.63	323.53	693.32	382.44	1.35516153	0.13395951	0.41013099	2.828920381	1.500251573	up	-	-
MEDP1374	Clupanodonyl 肉碱 [illegible]	Lipids	CAR	C27H54NO5	3	0.6186	-	59761.56	69698.26	92019.14	18468.2	18067.75	16482.11	1.60609659	0.02725044	0.20385331	4.177424825	2.062613866	up	-	-
MW0148609	ditrans,heptacis-Decaprenyl diphosphate	Organic acids	Organic acids	C50H84O7P2	3	0.6184	-	692.26	1501	793.61	4618.64	4215.69	1391.47	1.28776755	0.13343396	0.40911094	0.292091572	-1.77550736	down	C04509	-
MW0159751	Thermozeaxanthin	Lipids	Others	C61H94O8	3	0.6177	-	1064.37	551	3210.47	2514.01	3939.79	4979.08	1.14714608	0.11306872	0.37822354	0.422101868	-1.24433688	down	C15983	ko00906
MW0158665	Tyr-Val-Glu-Asp	Amino acids and derivatives	Amino acids and derivatives	C23H32N4O10	3	0.6176	-	173.7	125.7	716.28	1649.18	1689.85	771.77	1.32385585	0.05355515	0.26942452	0.247075995	-2.01697324	down	-	-
MW0158415	Tyr-Asp-Leu-Glu	Amino acids and derivatives	Amino acids and derivatives	C24H34N4O10	3	0.6173	-	31054.86	56738	72530.34	8316.36	9880.7	13324.55	1.5366161	0.06884491	0.29955003	5.086136146	2.346570081	up	-	-
MW0002528	2-{[2-(4-Sulfamoylphenoxy)ethyl]sulfanyl}acetic acid	Benzene and substituted derivatives	Benzene and substituted derivatives	C10H13NO5S2	3	0.6164	-	3861.66	1559.04	2690.95	11019.76	8795.58	6254.79	1.45352207	0.03177394	0.21827962	0.311147279	-1.68433047	down	-	-
MW0167355	(3-octadecanoyloxy-2-pentadecanoyloxypropyl) octadecanoate	GL	TG	C54H104O6	3	0.6161	-	954.04	3451.76	1582.65	8772.02	2200.04	5619.7	1.09969077	0.19517714	0.48243539	0.36092916	-1.47021239	down	-	-
MW0012979	1-Palmitoylglycerophosphocholine	GL	PC	C24H51NO7P+	3	0.6142	-	8064.28	8239.16	7991.52	2103.35	1619.32	3833.38	1.50977254	0.01338749	0.1555072	3.215298999	1.684952904	up	C04102	-
MW0139530	Quercetin 3-xylosyl-(1->6)-glucoside	Flavonoids	Flavones	C26H28O16	3	0.6115	-	32970.78	78411.87	67638.15	13635.94	20743.26	24132.58	1.40988367	0.09283698	0.34623466	3.059568518	1.613328208	up	-	-
MW0145929	Asn-Tyr-Gln-Phe-Phe	Amino acids and derivatives	Amino acids and derivatives	C36H43N7O9	3	0.6097	-	18238.85	17006.76	21094.02	33784.06	51154.94	44315.08	1.54898622	0.03458919	0.22565754	0.435882798	-1.19798783	down	-	-
MW0150511	Gly-Tyr-Asn	Amino acids and derivatives	Amino acids and derivatives	C15H20N4O6	3	0.6076	-	2107.08	7483.44	7383.75	290.89	1579.85	1173.78	1.30898185	0.11456719	0.38005298	5.57535178	2.479062835	up	-	-
MW0150206	Glu-Val-Gln	Amino acids and derivatives	Amino acids and derivatives	C15H26N4O7	3	0.6071	-	24580.71	25469.16	85958.68	7306.13	5708.33	4190.44	1.48240536	0.19031965	0.47658128	7.905221768	2.982805936	up	-	-

MW0168436	[6-[2-[2-(3,4-dihydroxyphenyl)-7-hydroxy-5-[3,4,5-trihydroxy-6-(hydroxymethyl)oxan-2-yl]oxychromenylium-3-yl]oxy-6-[[(E)-3-(3,4-dihydroxyphenyl)prop-2-enoyl]oxymethyl]-4,5-dihydroxyoxan-3-yl]oxy-3,4,5-trihydroxyoxan-2-yl]methyl (E)-3-(3,4-dihydroxyphenyl)prop-2-enoate	Others	Lactones	C51H53O27+	3	0.6065	-	11944.6	8343.49	6492.87	2552.18	1268.58	3237.61	1.47015471	0.04181518	0.23972398	3.794213112	1.923800714	up	-	-
MW0144737	Ala-Ser-Trp	Amino acids and derivatives	Amino acids and derivatives	C17H22N4O5	3	0.6059	-	15286.55	27211.43	15272.18	7884.03	10632.25	7393.35	1.4173776	0.10847615	0.37111447	2.229679081	1.156836077	up	-	-
MW0007659	Methyl 2-(2-hydroxy-6-methoxy-4-methylbenzoyl)-3-methoxy-5-sulfooxybenzoate	Benzene and substituted derivatives	Benzene and substituted derivatives	C18H18O10S	3	0.6044	-	7573.19	3200.83	3693.73	1284.23	330.28	317.69	1.46566973	0.08738363	0.33759772	7.487708312	2.904524235	up	-	-
MW0135856	6-[2,2-Dimethyl-6-(3-oxo-3-phenylpropyl)chromen-5-yl]oxy-3,4,5-trihydroxyoxane-2-carboxylic acid	Organic acids	Organic acids	C26H28O9	3	0.6044	-	973.51	4348.43	4175.44	1407.2	139.65	667.89	1.14084384	0.14724004	0.43166641	4.28825957	2.100392235	up	-	-
MW0156038	Pro-Tyr-Trp	Amino acids and derivatives	Amino acids and derivatives	C25H28N4O5	3	0.6042	-	236872.85	135338.89	209113.78	74714.16	100931.7	74546.91	1.4721767	0.0581241	0.28143735	2.323510468	1.216306144	up	-	-
MW0161842	cephalosporin C(1-)	Organic acids	Organic acids	C16H20N3O8S-	3	0.6027	-	3186.59	2249.5	5740.38	385.56	370.18	632.52	1.55690215	0.08784636	0.33783172	8.050703759	3.009114903	up	-	-
MW0168256	3,7-Dimethyl-9-(2,6,6-trimethylcyclohex-1-en-1-yl)nona-2,4,6,8-tetraenoic acid	Others	Vitamin	C20H28O2	3	0.6018	-	3637.47	2146.47	3488.33	940.14	704.47	2336.3	1.25363933	0.06463198	0.29216831	2.329183528	1.219824321	up	-	-
MW0150047	Glu-Leu-Ala-Met-Met	Amino acids and derivatives	Amino acids and derivatives	C24H43N5O8S2	3	0.6015	-	8074.31	5380.82	9149.68	1175.5	1265.35	5958.81	1.23086514	0.0777836	0.31983339	2.691157737	1.428226954	up	-	-
MW0016660	CDP-DG(a-25:0/i-19:0)	GL	DG	C56H105N3O15P2	3	0.6015	-	5311	4625.68	6633.57	2520.09	1619.54	1327.14	1.52547863	0.00985228	0.14545906	3.031085998	1.599834785	up	-	-
MW0146112	Asp-Gly-His	Amino acids and derivatives	Amino acids and derivatives	C12H17N5O6	3	0.5987	-	5176.68	28589.35	33137.62	59050.6	117206.14	72405.39	1.2632474	0.05574427	0.27603113	0.269054439	-1.89402998	down	-	-
MW0112501	3,4,5-trihydroxy-6-[3-(4-hydroxy-3-methoxyphenyl)oxirane-2-carbonyloxy]oxane-2-carboxylic acid	Organic acids	Organic acids	C16H18O11	3	0.5975	-	5.73	5.73	5.73	108.2	5.73	28.67	1.0866144	0.31046588	0.58451078	0.120546985	-3.05233253	down	-	-
MW0150321	Gly-Asp-Asn	Amino acids and derivatives	Amino acids and derivatives	C10H16N4O7	3	0.5959	-	2966.21	6057.96	4681.43	944.82	1098.78	1733.56	1.50015603	0.05730976	0.27999531	3.628546315	1.859391685	up	-	-
MW0155177	Phe-Glu-His	Amino acids and derivatives	Amino acids and derivatives	C20H25N5O6	3	0.593	-	14028.76	14144.28	20205.06	3882.05	4650.87	3838.82	1.60134989	0.02605687	0.19924248	3.91037154	1.96730569	up	-	-
MW0013188	2-(Sulooxymethyl)butanoic acid	Organic acids	Organic acids	C5H10O6S	3	0.5897	-	31832.17	26369.81	27858.35	4296.14	16318.86	9372.83	1.36416254	0.01894935	0.17600951	2.869841866	1.520971244	up	-	-
MW0157849	Thr-Tyr-Thr	Amino acids and derivatives	Amino acids and derivatives	C17H25N3O7	3	0.5887	-	41.19	25.58	56.74	27.77	19.47	5.55	1.10634134	0.10753737	0.37038694	2.339647661	1.226291283	up	-	-
MEDP1429	Carnitine C5:DC	Lipids	CAR	C12H21NO6	3	0.5877	-	899.64	730.06	885.3	113.78	220.08	528.47	1.329456	0.03201404	0.21871128	2.916516879	1.544246423	up	-	-
MW0123903	ethyl {10-[3-(morpholin-4-yl)propanoyl]-5-oxido-10H-phenothiazin-2-yl}carbamate	Others	Lactones	C22H25N3O5S	3	0.5872	-	172.9	91.24	617.31	99.94	108.59	53.67	1.0011076	0.33364851	0.59946402	3.361746758	1.74921105	up	-	-
MW0150873	His-Asp-His	Amino acids and derivatives	Amino acids and derivatives	C16H21N7O6	3	0.586	-	146.81	192.69	297.99	73.18	4.54	101.15	1.10367918	0.05476099	0.27218393	3.563985017	1.833491272	up	-	-
MW0134125	5,7-Dihydroxy-2-phenyl-8-[3,4,5-trihydroxy-6-(hydroxymethyl)oxan-2-yl]-6-(3,4,5-trihydroxyoxan-2-yl)chromen-4-one	Others	Ketone compounds	C26H28O13	3	0.5849	-	80448.49	90084.88	121375.79	209.37	192.47	467.78	1.62437993	0.01582747	0.16425584	335.6743865	8.390918647	up	-	-
MW0110190	Tyr-Asp-Arg	Amino acids and derivatives	Amino acids and derivatives	C19H28N6O7	3	0.5848	-	49637.91	34902.9	39006.38	987.82	1461.34	651.55	1.61761499	0.01157114	0.14569982	39.84480651	5.316319786	up	-	-
MW0150192	Glu-Tyr-Ile	Amino acids and derivatives	Amino acids and derivatives	C20H29N3O7	3	0.5784	-	19.93	3.25	15.23	73.53	21.77	65.85	1.22675314	0.11622576	0.38324967	0.238349364	-2.06885032	down	-	-
MW0150484	Gly-Thr-Ala	Amino acids and derivatives	Amino acids and derivatives	C9H17N3O5	3	0.5777	-	5.52	72.94	149.08	2.72	18.1	5.69	1.00839568	0.24649305	0.5293161	8.58317616	3.101511608	up	-	-

MW0135986	6-{[4,5-dihydroxy-2-({7-oxo-7H-furo[3,2-g]chromen-9-yl}oxy)oxan-3-yl]oxy}-3,4,5-trihydroxyoxane-2-carboxylic acid	Organic acids	Organic acids	C22H22O14	3	0.5771	-	110364.34	110376.15	104465.71	134796.67	392488.44	242880.41	1.27372262	0.18544244	0.47320151	0.422254946	-1.24381377	down	-	-
ZINC1338057	Cuscuta propenamide 2	Alcohol and [illegible]	Amines	C22H27NO3	3	0.576	-	8476.77	7222.53	9219.08	5100.1	3603.1	2318.09	1.42727106	0.01189553	0.14824085	2.260931343	1.176917183	up	-	-
MW0150882	His-Cys-Asn	Amino acids and derivatives	Amino acids and derivatives	C13H20N6O5S1	3	0.5735	-	12836.95	17130.82	11794.55	47405.08	31611.56	31002.41	1.53029277	0.04173479	0.23972398	0.379591716	-1.39747959	down	-	-
MW0134898	6-(3,4-dihydroxy-6-methyl-5-oxooxan-2-yl)-5,7-dihydroxy-2-(3-methoxyphenyl)-4H-chromen-4-one	Others	Ketone compounds	C22H20O9	3	0.5733	-	1497.71	3604.76	2166.83	4312.25	17003.25	3542.73	1.11226484	0.31053791	0.58451078	0.292430314	-1.77383522	down	-	-
MW0148064	Cys-Ser-Gln	Amino acids and derivatives	Amino acids and derivatives	C11H20N4O6S1	3	0.5721	-	4229.02	7157.12	1280.55	1922.58	257.87	549.51	1.19094957	0.1827052	0.47026574	4.63988117	2.214087858	up	-	-
MW0108017	Lys-Pro-Lys	Amino acids and derivatives	Amino acids and derivatives	C17H33N5O4	3	0.5703	-	0.6	0.84	0.17	4.91	2.76	0.91	1.2522945	0.17902075	0.46794913	0.187645688	-2.41391696	down	-	-
MW0113729	6-{5-[(1E)-3-[(3-carboxy-5,6-dihydroxycyclohex-3-en-1-yl)oxy]-3-oxoprop-1-en-1-yl]-2-hydroxyphenoxy}-3,4,5-trihydroxyoxane-2-carboxylic acid	Organic acids	Organic acids	C22H24O14	3	0.5695	-	713532.75	779577.61	763500.95	5454.61	1173.7	4401.74	1.60900077	0.00067349	0.06250295	204.5875866	7.676574802	up	-	-
ZINC1337858	boeravinone F	Others	Ketone compounds	C17H10O7	3	0.5667	-	553.89	584.44	1149.16	1871.55	2373.24	1102.89	1.30070443	0.09141436	0.34325119	0.427753717	-1.2251477	down	-	-
MW0128248	(5-{8-[1-(2,4-dihydroxyphenyl)-3-(3,4-dihydroxyphenyl)-2-hydroxypropyl]-3,5,7-trihydroxy-3,4-dihydro-2H-1-benzopyran-2-yl}-2-hydroxyphenyl)oxidanesulfonic acid	Organic acids	Organic acids	C30H28O14S	3	0.5644	-	912.63	1530.09	872.55	461.2	380.65	631.45	1.41235598	0.08919252	0.33912614	2.250234168	1.170075142	up	-	-
MW0158634	Tyr-Trp-Asp	Amino acids and derivatives	Amino acids and derivatives	C24H26N4O7	3	0.5622	-	251.83	132.81	316.87	21.1	157.78	97.97	1.07679079	0.10782605	0.37038694	2.533899223	1.341359148	up	-	-
MW0145997	Asn-Tyr	Amino acids and derivatives	Amino acids and derivatives	C13H17N3O5	3	0.5601	-	31299.07	52748.13	41845.9	4102.91	7458.78	4796.84	1.59249076	0.02499623	0.1956303	7.695868761	2.944084197	up	-	-
MW0014120	3-Hydroxysintaxanthin	Others	Ketone compounds	C31H42O2	3	0.5514	-	50.64	50.36	43.35	341.37	60.76	87.74	1.08208606	0.3264638	0.59557833	0.294670014	-1.76282784	down	-	-
MW0050838	1-Behenoyl-3-nervonoyl-sn-glycerol	GL	DG	C49H94O5	3	0.5484	-	5382.68	10122.02	14424.44	2208.97	3595.85	1609.45	1.43721478	0.09619797	0.35181896	4.036694105	2.013174265	up	-	-
MW0113379	6-(5-{[(2E)-3-(3,4-dihydroxyphenyl)prop-2-enoyl]oxy}-3,4-dihydroxycyclohex-1-ene-1-carbonyloxy)-3,4,5-trihydroxyoxane-2-carboxylic acid	Organic acids	Organic acids	C22H24O14	3	0.5453	-	1280.56	744.9	757.36	6644.66	12106.39	2933.75	1.48252592	0.14112779	0.42131453	0.128330443	-2.96206464	down	-	-
MW0056818	1-(9Z-tetradecenoyl)-2-(9Z-hexadecenoyl)-glycero-3-phosphocholine	GL	PC	C38H72NO8P	3	0.5418	-	121.72	197.16	191.43	0.19	9.47	0.95	1.48117397	0.01928305	0.17648958	48.09707823	5.587877352	up	-	-
MW0068675	TG(14:1(9Z)/22:2(13Z,16Z)/o-18:0)	GL	TG	C57H106O5	3	0.532	-	584.45	286.57	1210.64	666.8	3679.19	3247.45	1.08484843	0.18295192	0.47026574	0.274139257	-1.86701916	down	-	-
MW0127719	({6-[5,7-dihydroxy-2-(4-hydroxyphenyl)-4-oxo-3,4-dihydro-2H-1-benzopyran-8-yl]-3,4,5-trihydroxyoxan-2-yl}methoxy)sulfonic acid	Organic acids	Organic acids	C21H22O13S	3	0.532	-	423.92	597.88	586.82	11421.58	4586.49	5118.18	1.58507841	0.09738395	0.35362798	0.076143187	-3.71514124	down	-	-
MW0057843	1-Octadecyl-2-docosanoyl-sn-glycero-3-phosphocholine	GL	PC-O	C48H98NO7P	3	0.5256	-	3263	4065.17	2486.84	121.43	488.4	3000.42	1.11047488	0.13526549	0.41129659	2.718651063	1.442890994	up	-	-
MW0104573	2-amino-4-({1-[(carboxymethyl)-C-hydroxycarbonimidoyl]-2-{[1-(2-hydroxy-4-methoxyphenyl)-3-oxopropyl]sulfanyl}ethyl}-C-hydroxycarbonimidoyl)butanoic acid	Organic acids	Organic acids	C20H27N3O9S	3	0.525	-	3225.7	853.89	3458.98	10995.02	7038.03	4822.3	1.25198056	0.08813062	0.33783172	0.329838309	-1.60016912	down	-	-

MW0161020	(2S)-4-(2-aminophenyl)-2-azaniumyl-4-oxobutanoate	Others	Lactones	C10H12N2O3	3	0.5247	-	28667.23	18378.67	21576.7	308.17	504.42	176.29	1.61385496	0.01762361	0.17028658	69.39426422	6.116744517	up	-	-
ZINC3229679	4-(beta-D-Glucopyranosyloxy)-3-pyridinecarboxylic acid	Organic acids	Organic acids	C12H15NO8	3	0.5208	-	304.88	90.31	153.31	80.05	42.16	124.98	1.02793589	0.25131196	0.5338413	2.218940896	1.14987124	up	-	-
MW0107572	1-(2-Amino-3-methylpentanoyl)pyrrolidine-2-carboxylic acid	Organic acids	Organic acids	C11H20N2O3	3	0.5192	-	3349.23	2587.69	3715.87	793.3	832.32	1066.16	1.59416099	0.01523446	0.1631418	3.586024861	1.84238549	up	-	-
MW0108246	methyl N-[(2-nitrophenyl)sulfonyl]glycinate	Others	Lactones	C9H10N2O6S	3	0.5191	-	1468.03	1594.08	4939.45	1341.87	1023.96	974.51	1.0861266	0.30458558	0.57894731	2.3954328	1.260286342	up	-	-
MW0063133	CID 131820051	GL	PS	C54H102NO10P	3	0.5188	-	1808.2	1015.6	1057.38	5143.17	1252.97	4407.28	1.10799266	0.18835954	0.47556339	0.359254755	-1.47692084	down	-	-
MW0152399	Leu-Leu-Leu-Leu-Ser	Amino acids and derivatives	Amino acids and derivatives	C27H51N5O7	3	0.5172	-	80167.39	71117.11	69382.92	19301.26	18377.28	23114.94	1.61975469	0.00113891	0.07389496	3.629787602	1.859885131	up	-	-
MW0110238	L-Tyrosyl-L-Asparagine	Amino acids and derivatives	Amino acids and derivatives	C13H17N3O5	3	0.5169	-	87645.05	51400.4	67739.14	21782.59	15646.69	33615.11	1.46423188	0.03169934	0.21827962	2.91063925	1.54133604	up	-	-
MW0163764	2-Aminobut-2-enoate	Others	Lactones	C4H6NO2-	3	0.5128	-	22577.81	22125.98	20343.14	622.97	119.42	490.97	1.58565086	0.00063216	0.06185252	52.73961374	5.7208151	up	-	-
MW0167765	[(2R)-2-[(5Z,8Z,11Z,14Z)-icosa-5,8,11,14-tetraenoyl]oxy-3-[(6Z,9Z,12Z,15Z)-octadeca-6,9,12,15-tetraenoyl]oxypropyl] (7Z,10Z,13Z,16Z,19Z)-docosa-7,10,13,16,19-pentaenoate	GL	TG	C63H96O6	3	0.5061	-	1391.63	937.31	1462.21	4329.9	3323.17	1662.28	1.30444365	0.13609558	0.41296677	0.406978804	-1.29697444	down	-	-
MW0011391	(5R,9S,15R)-5,9-dimethyl-15-[(Z)-2-methylbut-2-enoyl]oxy-14-methylidenetetracyclo[11.2.1.01,10.04,9]hexadecane-5-carboxylic acid	Organic acids	Organic acids	C25H36O4	3	0.506	-	3745.17	1923.89	1670.12	5160.76	8305.66	3014.58	1.17362527	0.17557714	0.46469486	0.445311571	-1.167113	down	-	-
Zmhn002301	4-O-beta-D-glucosyl-4-coumaric acid	Organic acids	Organic acids	C15H18O8	2	0.9912	14364-05-7	3839692.83	4074750.29	6060499.82	10546.85	634152.46	67526.53	1.40978088	0.01831953	0.17381882	19.62150508	4.294363804	up	C04415	-
MW0014140	2-Hydroxy-3-isopropylsuccinic acid	Organic acids	Organic acids	C7H12O5	2	0.9466	16048-89-8	186.93	113.6	13.34	830.28	929.93	995.5	1.39122152	0.00030869	0.05012965	0.113898052	-3.13418503	down	-	-
MW0157043	Farnesylthioacetic acid	Terpenoids	Sesquiterpenoids	C17H28O2S	2	0.8892	135784-48-4	326.15	190.01	403.24	676.86	1118.02	980.96	1.47476064	0.02557718	0.1976296	0.331215056	-1.59415984	down	-	-
MW0168267	Chrysanthellin A	Terpenoids	Steroid	C58H94O25	2	0.8864	73039-13-1	21785.93	34822.82	28286.42	5455.05	4875.5	3971.98	1.60617175	0.0234171	0.18818988	5.935675017	2.569412105	up	-	-
MW0053624	Ginsenoside Rg3	Terpenoids	Triterpene Saponin	C42H72O13	2	0.8784	14197-60-5 | 11019-45-7	121.91	63.78	148.69	1746.7	376.6	224.08	1.25975763	0.29992089	0.57523965	0.142448176	-2.81149094	down	C20778	ko00999,ko01110
MW0016358	Methyl decanoate	Others	Lactones	C11H22O2	2	0.8736	110-42-9	80549.99	73014.33	104920.75	200927.25	194423.07	175304.35	1.56918391	0.00132518	0.07898695	0.452962332	-1.14253701	down	-	-
MW0114088	cis-coumarinic acid-beta-D-glucoside	Phenolic acids	Phenolic acids	C15H18O8	2	0.8701	2446-60-8	53142.75	53467.52	32599.77	91146.2	118747.69	96580.78	1.48867355	0.00767273	0.13950182	0.454230165	-1.13850458	down	C05839	ko00999,ko01110
MW0011760	[6]-Gingerdiol 3,5-diacetate	Others	Lactones	C21H32O6	2	0.8543	195204-05-8	5823.22	2560.04	7570.78	2520.96	272.9	329.11	1.30199765	0.0815661	0.32588632	5.108611354	2.352931184	up	-	-
MEDP2286	Celastrol	Benzene and substituted derivatives	Benzene and substituted derivatives	C29H38O4	2	0.8458	34157-83-0	1075.37	1352.15	2376.87	3684.07	5780.86	3668.48	1.43754613	0.03793591	0.2307381	0.365814362	-1.45081638	down	-	-
MW0169501	Myricitrin	Flavonoids	Flavonols	C21H20O12	2	0.828	17912-87-7	1124.22	481.06	119.37	1401.41	1685.15	1366.32	1.15181237	0.07764898	0.31983339	0.387311133	-1.36843512	down	C10108	-
MW0104828	2-Ketohexanoic acid	Organic acids	Organic acids	C6H10O3	2	0.8209	2492-75-3	6785.32	14262.62	8860.06	19293.42	33357.13	20045.07	1.38725163	0.07015784	0.30287318	0.411414058	-1.281337	down	C00902	-
MW0108880	N-omega-Propyl-L-arginine	Amino acids and derivatives	Amino acids and derivatives	C9H20N4O2	2	0.8128	137361-05-8	1853.55	2996.89	3104.74	1090.95	606.66	1174.64	1.45208578	0.03550732	0.22604178	2.769668378	1.469713248	up	-	-
MW0138673	2''-p-[illegible]	Flavonoids	Flavones	C30H26O13	2	0.8031	137018-32-7	115710.02	90459.86	128234.42	28763.27	35425.82	37274.91	1.59477886	0.01589927	0.16425584	3.295792596	1.720625457	up	-	-
MW0108192	H-Met-arg-phe-ala-OH	Amino acids and derivatives	Amino acids and derivatives	C23H37N7O5S	2	0.7985	67368-29-0	16102.98	12869.21	15383.07	9425.67	1660.28	1672.48	1.32106199	0.04174211	0.23972398	3.476545312	1.797654393	up	-	-
MW0144813	H-Ala-Abu-OH	Amino acids and derivatives	Amino acids and derivatives	C7H14N2O3	2	0.7862	39537-33-2	27.57	14.2	53.06	4.73	3.84	17.64	1.22730955	0.17249755	0.46208816	3.6180847	1.855226182	up	-	-
MW0105076	3-Mercaptopyruvic acid	Organic acids	Organic acids	C3H4O3S	2	0.7849	2464-23-5	7571.16	6010.58	8036.72	8730.02	23098.82	12359.78	1.19579308	0.221498	0.50529946	0.48923139	-1.03141112	down	C00957	ko00270,ko01100
MW0130165	5,7,3',4'-Tetrahydroxy-6,8-dimethoxyflavone	Flavonoids	Flavones	C17H14O8	2	0.7776	57093-50-2	30127.73	11199.1	29137.84	54518.04	67704.15	59885.05	1.3790809	0.0108689	0.14566058	0.386940519	-1.36981628	down	-	-
MW0157439	Tetrahydrofolic acid	Organic acids	Organic acids	C19H23N7O6	2	0.7769	135-16-0	74.98	5993.33	6673.41	12266.52	18196.52	56880.68	1.08897373	0.21538625	0.5024535	0.145880208	-2.77714394	down	-	-
MW0108619	N-Acetyl-D-phenylalanine	Amino acids and derivatives	Amino acids and derivatives	C11H13NO3	2	0.7747	10172-89-1	164.76	550.76	422.57	169.34	101.76	78.77	1.29476959	0.14010953	0.42018117	3.252893932	1.701723781	up	C05620	ko00470,ko01100
MEDN1098	2-Ethyl-2-Hydroxybutyric acid	Organic acids	Organic acids	C6H12O3	2	0.7703	3639-21-2	27.02	85.06	61.72	171.43	263.14	142.43	1.39347843	0.04817709	0.25598818	0.301213172	-1.73114324	down	-	-
MW0113988	beta-D-Galp-(1->3)-D-GalpNAc	Others	Saccharides	C14H25NO11	2	0.7695	20972-29-6	107711.78	112645.17	81241.93	195786.1	213600.3	213350.87	1.56878922	0.00173516	0.08476296	0.484311594	-1.04599255	down	C07278	-
MW0002264	2,4-Dihydroxy-3,6-dimethylbenzoic acid	Benzene and substituted derivatives	Benzene and substituted derivatives	C9H10O4	2	0.7685	4707-46-4	28921.14	24018.47	32580.79	5536.4	3640.28	10188.13	1.51778992	0.00269229	0.09898666	4.416278807	2.142831255	up	-	-
MW0111951	2,3-Diphosphoglyceric acid	Others	Lactones	C3H8O10P2	2	0.7634	138-81-8	11402.04	10077.03	10918.28	2316.05	6788.85	5261.52	1.32448853	0.03576783	0.22604178	2.255074681	1.173175212	up	C03339	-

MW0138674	kaempferol 3-O-[alpha-L-rhamnopyranosyl(1->2)-beta-D-glucopyranosyl]-7-O-alpha-L-rhamnopyranoside	Flavonoids	Flavonols	C33H40O19	2	0.7626	162062-89-7	844.74	317.03	507.92	1738.62	2908.32	1906.69	1.48716234	0.03207708	0.21873894	0.254773309	-1.97271395	down	-	-
MW0139580	Rhapontin	Others	Stilbene	C21H24O9	2	0.7619	155-58-8	197.81	62	57.32	189.64	169.18	532.84	1.14543906	0.24117311	0.52442374	0.355662472	-1.49141934	down	-	-
MW0110913	Anandamide	Alcohol and [illegible]	Amines	C22H37NO2	2	0.7521	94421-68-8	789.79	817.09	517.63	258.67	300.33	127.66	1.45125679	0.02042523	0.18038854	3.093976641	1.629462305	up	C11695	-
MW0107643	Lactacystin	Amino acids and derivatives	Amino acids and derivatives	C15H24N2O7S	2	0.7457	133343-34-7	246.98	159.24	572.94	431.74	5024.37	5090.44	1.2300602	0.17373087	0.46285781	0.092841735	-3.42908271	down	-	-
MW0168670	9,12-Octadecadiynoic Acid	Lipids	Free fatty acids	C18H28O2	2	0.7448	2012-14-8	52.59	166.08	105.63	10.42	19.72	57.72	1.25930987	0.12333308	0.3945943	3.691099476	1.884050619	up	-	-
MW0137905	Delphinidin 3-O-beta-D-sambubioside	Flavonoids	Anthocyanidins	C26H29O16+	2	0.7419	178275-92-8	2115.18	2361.83	1913.16	513.31	864.74	232.25	1.45278898	0.00304324	0.10260237	3.968310253	1.988524824	up	C20491	ko00942
MW0129533	1-(2-Hydroxyphenyl)-3-phenylpropane-1,3-dione	Others	Ketone compounds	C15H12O3	2	0.7406	1469-94-9	611.99	873.68	1562.78	2407.57	1335.98	4000.03	1.20136115	0.17076894	0.45861823	0.393674502	-1.34492482	down	C22066	-
MW0123493	Dauricine	Alkaloids	Isoquinoline alkaloids	C38H44N2O6	2	0.7391	524-17-4	32079.72	18132.59	29358.11	10546.86	13939.66	12678.05	1.44213053	0.07349083	0.310279	2.141028942	1.098304298	up	-	-
MW0006499	Carboxytolbutamide	Benzene and substituted derivatives	Benzene and substituted derivatives	C12H16N2O5S	2	0.7378	2224-10-4	218955.14	156728.81	333860.84	15249.41	21813.61	21554.13	1.60057735	0.0524694	0.26669928	12.10473027	3.597499027	up	-	-
MW0139490	[6-[2-(3,4-Dihydroxyphenyl)-5,7-dihydroxy-4-oxochromen-3-yl]oxy-3,4,5-trihydroxyoxan-2-yl]methyl acetate	Flavonoids	Flavones	C23H22O13	2	0.7355	54542-51-7	2502.59	804.55	306.86	95.41	209.98	484.5	1.09301215	0.29105978	0.56921796	4.575320614	2.193872843	up	-	-
MW0125626	N-Acetyl-L-tryptophanamide	Alcohol and amines	Amines	C13H15N3O2	2	0.7333	2382-79-8	262033.98	256457.97	99685.24	55457.72	64920.17	55274.74	1.42976723	0.1086996	0.37126682	3.519316449	1.815295244	up	-	-
MW0110142	Trp-tyr	Amino acids and derivatives	Amino acids and derivatives	C20H21N3O4	2	0.7319	19653-76-0	778.01	379.23	556.48	538.69	151.75	104.93	1.10199817	0.16536236	0.45328035	2.154619862	1.107433358	up	-	-
MW0124445	Irisxanthone	Flavonoids	Other Flavonoids	C20H20O11	2	0.7314	51419-56-8	667581.28	649971.77	772126.03	50782.23	71595.29	65825.45	1.62552289	0.00294642	0.10260237	11.1033268	3.472920099	up	C10067	-
MEDP1879	Phe-ala	Amino acids and derivatives	Amino acids and derivatives	C12H16N2O3	2	0.7298	3918-87-4	296.72	164.07	266.55	23.54	148.69	17.61	1.28848361	0.03801715	0.2307381	3.831331648	1.937845914	up	-	-
MW0115294	Glucosinalbin	Organic acids	Organic acids	C14H19NO10S2	2	0.7287	19253-84-0 | 20196-67-2	8605.03	2561.61	3512.7	15478.62	8054.32	12102.92	1.25966872	0.07153294	0.30584544	0.411926077	-1.27954264	down	-	-
MW0126958	Vulpinic acid	Organic acids	Organic acids	C19H14O5	2	0.7273	521-52-8 | 73622-57-8	7656.58	5083.75	7995.17	3415.13	2234.32	3416.42	1.46669825	0.03608858	0.22604178	2.287204648	1.193585457	up	-	-
FDATN00937	Vidarabine monohydrate	Nucleotides and derivatives	Nucleotides and derivatives	C10H15N5O5	2	0.724	24356-66-9	31.58	17.67	2064.21	1896.38	1505.81	1861.62	1.11613617	0.26043837	0.54145363	0.401507653	-1.31650061	down	C07195	-
MW0005020	Hydroxymethyltolbutamide	Benzene and substituted derivatives	Benzene and substituted derivatives	C12H18N2O4S	2	0.72	1185112-19-9	315712.76	320409.64	342323.74	164698.22	94791.53	179981.97	1.47129234	0.01415869	0.15930201	2.226414341	1.154722107	up	-	-
MEDL00356	Phe-Asp	Amino acids and derivatives	Amino acids and derivatives	C13H16N2O5	2	0.7199	22828-05-3	87709.5	56253.9	86553.57	20134.86	30739.55	27306.18	1.53657159	0.03010515	0.2125747	2.948519191	1.559990584	up	-	-
MW0109216	L-phenylalanyl-L-valine	Amino acids and derivatives	Amino acids and derivatives	C14H20N2O3	2	0.7135	3918-90-9	31470.76	16126.59	34590.92	6362.34	2398.28	1241.74	1.47900019	0.04377636	0.24666834	8.216887814	3.03859207	up	-	-
MADN0597	Asp-Val	Amino acids and derivatives	Amino acids and derivatives	C9H16N2O5	2	0.7128	13433-04-0	7367.33	4154.87	1335.7	16594.37	30636.43	27984.22	1.43160001	0.02719836	0.20385331	0.170948569	-2.54836575	down	-	-
MW0119764	3-Pyrimidin-2-yl-2-pyrimidin-2-ylmethyl-propionic acid	Organic acids	Organic acids	C12H12N4O2	2	0.7091	936643-76-4	144036.07	194542.17	209803.77	51837.48	67289.84	109697.46	1.43528794	0.01616173	0.16485394	2.396514967	1.26093795	up	-	-
MW0111319	Thiolutin	Heterocyclic compounds	Heterocyclic compounds	C8H8N2O2S2	2	0.7066	87-11-6	199884.42	98807	161328.3	52768.18	55613.44	48336.38	1.50977889	0.07464558	0.31230283	2.935334295	1.553524816	up	-	-
MEDL02149	Acetaminophen glucuronide	Others	Ketone compounds	C14H17NO8	2	0.7064	16110-10-4	156251.78	194896.3	205212.15	45985.09	30948.17	37463.02	1.60819404	0.00642663	0.13374656	4.863446871	2.281979158	up	-	-
MW0124060	Ethyl 2-amino-4-methylthiazole-5-carboxylate	Others	Lactones	C7H10N2O2S	2	0.7015	7210-76-6	332819.94	313203.4	172074.49	650835.67	587974.71	531794.8	1.43774641	0.00924336	0.14350153	0.462044186	-1.11389727	down	-	-
MW0002160	Biphenyl-2,3-diol	Benzene and substituted derivatives	Benzene and substituted derivatives	C12H10O2	2	0.7006	1133-63-7	14571.8	14614.04	17268.3	32423.86	47121.98	33258.01	1.56276697	0.03945481	0.23365724	0.411813427	-1.27993723	down	C02526	ko01100
MW0062178	Pristanic acid	Terpenoids	Ditepenoids	C19H38O2	2	0.7004	1189-37-3	144494.62	194625.74	178892.9	53583.33	79931.32	96584.9	1.4797678	0.00832088	0.14010122	2.251257162	1.170730866	up	-	-
MW0108082	Lys-Tyr	Amino acids and derivatives	Amino acids and derivatives	C15H23N3O4	2	0.7002	35978-98-4	5244.79	4108.44	5245.67	1074.75	2791.96	2052.85	1.39716409	0.01154078	0.14569982	2.466213705	1.302297819	up	-	-
MW0049195	Dehydrotomatin	Alkaloids	Steroid alkaloids	C50H81NO21	3	0.9974	157604-98-3	7157.78	16811.01	21250.94	4508.86	2371.86	5836.6	1.34763531	0.11400867	0.37876977	3.55575939	1.830157703	up	-	-
MW0103175	Yayoisaponin C	Terpenoids	Steroidal [illegible]	C51H84O25	3	0.9962	218934-75-9	10859.61	7618.55	7744.53	4007.76	58.11	96.91	1.28552413	0.01321477	0.15433097	6.299321607	2.655196469	up	-	-
MEDL02027	Ferulic acid	Phenolic acids	Phenolic acids	C10H10O4	3	0.9793	537-98-4	2469.55	915.15	1817.13	767.8	764.53	891.81	1.23151211	0.17524039	0.46430045	2.145845537	1.101546231	up	C01494	ko00940,ko01100,ko01110
MEDL02180	Esculetin	Lignans and Coumarins	Coumarins	C9H6O4	3	0.9725	305-01-1	3586.04	3790.07	5694.12	765.15	493.93	4818.64	1.07748854	0.2337992	0.51726398	2.150515325	1.104682412	up	C09263	ko00999,ko01110
MEDL02140	4-Hydroxybenzoic acid	Phenolic acids	Phenolic acids	C7H6O3	3	0.9679	99-96-7	242618.43	207768.07	242750.47	54284.49	76554.94	54876.73	1.60483514	0.00064488	0.06185252	3.732238325	1.900041114	up	C00156	ko00130,ko00790,ko01100,ko01110,ko01240
MW0015883	Congmunoside V	Terpenoids	Triterpene Saponin	C54H88O23	3	0.9642	340963-86-2	6.33	38.09	20.58	1417.29	739.84	33.47	1.23791575	0.21808755	0.50443785	0.029672236	-5.07474255	down	-	-

MEDN0533	Xanthosine	Nucleotides and derivatives	Nucleotides and derivatives	C10H12N4O6	3	0.9641	146-80-5	165474.54	155969.27	204313.93	5168.39	6735.42	9798.01	1.62031644	0.00727015	0.13569	24.22643539	4.598510241	up	C01762	ko00230,ko00232,ko01100,ko01110,ko01232,ko02010
MEDN0311	Dodecanedioic Aicd	Lipids	Free fatty acids	C12H22O4	3	0.9568	693-23-2	2198.01	1377.53	1285.37	264.2	1564.82	288.88	1.13153153	0.16238167	0.45001755	2.295155579	1.198591951	up	C02678	-
MEDP0507	2-Hydroxycinnamic acid	Phenolic acids	Phenolic acids	C9H8O3	3	0.9561	614-60-8	1909927.68	2251714.52	2371588.83	481670.8	352301.85	370039.08	1.61809045	0.00350992	0.10606676	5.426218755	2.43994721	up	C01772	ko00360,ko00999,ko01100,ko01110
MEDN0685	Undecanedioic acid	Lipids	Free fatty acids	C11H20O4	3	0.9472	1852-04-6	1544.12	372.93	1895.32	556.11	466.85	90.86	1.04047266	0.18219082	0.46991768	3.422788242	1.77517204	up	-	-
MEDL00440	(+)-Catechin gallate	Flavonoids	Flavanols	C22H18O10	3	0.9464	1257-08-5	11837.02	7358.79	8325	5208.24	3017.15	4054.55	1.44547422	0.04691792	0.25471913	2.241119256	1.164219421	up	-	-
MEDP0137	3,4-Dihydroxybenzaldehyde	Phenolic acids	Phenolic acids	C7H6O3	3	0.9411	139-85-5	4460.97	4454.42	2319.04	1327.07	2621.93	1549.54	1.25053131	0.09726789	0.35349748	2.043166004	1.030806426	up	C16700	ko00950
MW0133983	5,6-Dihydroxyflavone	Others	Ketone compounds	C15H10O4	3	0.9313	6665-66-3	112.77	47.28	148.37	203.76	436.94	192.97	1.24837956	0.14744363	0.43166641	0.369954538	-1.4345801	down	-	-
MEDN0491	9S-HOTrE	Lipids	Free fatty acids	C18H30O3	3	0.9247	89886-42-0	16983.19	22551.15	42440.1	8562.09	9230.44	12205.9	1.38961083	0.1515604	0.43601352	2.732624341	1.450287143	up	-	-
MEDL02156	Isoeugenol	Phenolic acids	Phenolic acids	C10H12O2	3	0.9148	5932-68-3	3221.05	3070.73	1673.37	2122.82	746.99	135.69	1.07364778	0.09942908	0.35781493	2.650191316	1.406096511	up	C10469	ko00940
MEDL02471	Flavin adenine dinucleotide	Nucleotides and derivatives	Nucleotides and derivatives	C27H33N9O15P2	3	0.9131	146-14-5	14.93	77.63	3.96	88.75	150.29	96.23	1.19318506	0.05867903	0.28188244	0.287887374	-1.79642358	down	C00016	ko00740,ko01100,ko01110,ko01240
MW0102824	Tomatine	Alkaloids	Steroid alkaloids	C50H83NO21	3	0.9073	17406-45-0	689077.97	738370.32	805546.99	153197.56	203095.03	192541.54	1.61773786	0.00093791	0.06912669	4.068615922	2.024538096	up	-	-
MEDN1155	Ketoleucine	Organic acids	Organic acids	C6H10O3	3	0.9026	816-66-0	30677.1	52874.65	30473.2	223004.35	301129.07	247136.63	1.59808384	0.00649153	0.13398007	0.1478405	-2.75788655	down	C00233	ko00280,ko00290,ko00785,ko00966,ko01100,ko01110,ko01210,ko01230
MEDL01890	3-Methylbenzaldehyde	Others	Aldehyde compounds	C8H8O	3	0.895	620-23-5	122909.01	142126.55	157729.38	32647.95	25275.53	26173.04	1.61901394	0.00574712	0.13003217	5.02713953	2.329737732	up	C07209	ko01100
MEDL02396	Sucrose	Others	Saccharides	C12H22O11	3	0.8655	57-50-1	142949.73	77795.37	110329.01	213068.08	809341.5	376143.76	1.37519285	0.18223689	0.46991768	0.236726123	-2.07870918	down	C00089	ko00052,ko00500,ko01100,ko01110,ko02010
MW0107859	Levulinic acid	Organic acids	Organic acids	C5H8O3	3	0.8578	123-76-2	112262.58	112993.57	87803.97	236026.04	255153.44	268515.31	1.60015268	0.0003171	0.05012965	0.412086701	-1.27898019	down	-	-
MEDL02347	L-Phenylalanine	Amino acids and derivatives	Amino acids and derivatives	C9H11NO2	3	0.8567	63-91-2	6589.95	9824.52	8939.94	19910.37	17911.37	16454.19	1.54354889	0.00228761	0.09175376	0.467139117	-1.09807584	down	C00079	ko00360,ko00400,ko00460,ko00470,ko00940,ko00960,ko00966,ko00970,ko00996,ko00999,ko01100,ko01110,ko01210,ko01230,ko02010
MW0015125	7,8-Dehydroastaxanthianthi[illegible]	Others	Ketone compounds	C40H50O4	3	0.8545	19866-02-5	14.06	43.99	62.97	143.1	92.67	74.6	1.20556193	0.07226402	0.30686128	0.389921706	-1.35874363	down	-	-
MW0154950	1-O-(4-Coumaroyl)-beta-D-glucose	Phenolic acids	Phenolic acids	C15H18O8	3	0.8529	7139-64-2	2155753.95	2447683.73	2580974.73	1140900.75	1121728.5	1033251.29	1.61335243	0.00630561	0.13374656	2.17981578	1.124206215	up	-	-
MW0000196	Corynantheine	Alkaloids	Plumerane	C22H26N2O3	3	0.8315	18904-54-6	44694.48	88610.31	39501.77	21078.35	15461.71	28700.93	1.36670003	0.14113846	0.42131453	2.648742148	1.405307406	up	C17872	-
MEDN0724	Aspirin	Organic acids	Organic acids	C9H8O4	3	0.8275	50-78-2	21131.97	21424.71	21468.83	8043.57	10761.44	10351.86	1.59148545	0.00472349	0.11959415	2.195897914	1.134810986	up	C01405	-
MW0120490	8-(4-Dibenzothienyl)-2-(4-morpholinyl)-4H-1-benzopyran-4-one	Benzene and substituted derivatives	Benzene and substituted derivatives	C25H19NO3S	3	0.8248	503468-95-9	67450.86	87802.87	103552.25	36754.38	47921.12	37467	1.51318229	0.03730256	0.22934484	2.118885564	1.083305673	up	-	-
MW0007154	Flavidulol C	Phenolic acids	Phenolic acids	C34H42O4	3	0.8169	117568-34-0	413.59	431.46	417.41	97.19	222.92	170.77	1.46645384	0.01775594	0.1703026	2.571830183	1.362795385	up	-	-
MW0155603	Plantagoside	Flavonoids	Flavanones	C21H22O12	3	0.813	78708-33-5	109710.65	79733.21	98487.25	39248.06	42620.85	39309.99	1.59153486	0.02239694	0.18480239	2.376082882	1.248585161	up	C17531	-
MW0149406	Gallocatechin-(4alpha->8)-epigallocatechin	Flavonoids	Flavones	C30H26O14	3	0.81	68964-95-4	21217.19	14107.32	28974.65	7806.41	7930.15	8916.46	1.48138655	0.09033988	0.3408345	2.608165653	1.383035503	up	-	-
MW0147854	Cuauchichicine	Heterocyclic compounds	Heterocyclic compounds	C22H33NO2	3	0.8098	545-60-8	1022.4	1098.83	2224.3	745.12	694.54	675.63	1.29977339	0.1954941	0.48243539	2.054342431	1.038676679	up	-	-
MEDL01973	Maslinic acid 3-O-b-D-glucoside	Terpenoids	Triterpene Saponin	C36H58O9	3	0.801	163634-06-8	218.56	296.23	18.48	609.9	517.51	440.39	1.09073391	0.03257982	0.21978922	0.340139048	-1.55580346	down	-	-
MW0009992	Triphenylphosphine oxide	Benzene and substituted derivatives	Benzene and substituted derivatives	C18H15OP	3	0.7996	791-28-6	1315.28	923.07	1186.15	68.05	93.24	352.34	1.47375356	0.00328022	0.10290142	6.667250745	2.737091986	up	-	-
MEDN1744	3-Methoxysalicylic acid	Benzene and substituted derivatives	Benzene and substituted derivatives	C8H8O4	3	0.7982	877-22-5	16142.88	13036.89	15060.53	2888.06	3382.92	8097.27	1.44238215	0.0122974	0.14864691	3.079031893	1.622476811	up	-	-
MEDN1078	Epoxyoleic acid	Lipids	Free fatty acids	C18H34O3	3	0.7865	24560-98-3	344.75	872.4	928.41	99.36	277.92	160.54	1.38104055	0.09276152	0.34623466	3.989364471	1.996158935	up	-	-
MW0142688	3,4-Dihydroxyphthalic acid	Benzene and substituted derivatives	Benzene and substituted derivatives	C8H6O6	3	0.785	82784-82-5	283.01	773.27	321.31	5473.64	2324.04	4120.64	1.53221318	0.05743331	0.27999531	0.115585922	-3.11296241	down	C03223	ko01100
MW0102906	cis,cis-Muconic acid	Organic acids	Organic acids	C6H6O4	3	0.7838	1119-72-8	16616.06	23319.09	53720.32	70806.59	76680.12	64764.45	1.30729956	0.06379222	0.29035184	0.441248331	-1.18033727	down	C02480	ko01100
MW0121592	5-Methoxy-dl-tryptophan	Amino acids and derivatives	Amino acids and derivatives	C12H14N2O3	3	0.7809	28052-84-8	1179.21	1372.03	1298.1	32.75	28.46	23.57	1.63070429	0.00195837	0.08476296	45.40386884	5.504743329	up	-	-
MEDL00626	Butyrate	Organic acids	Organic acids	C4H7O2-	3	0.7809	461-55-2	6978.13	7602.06	6725.86	9771.65	21262.1	11827.25	1.3157566	0.1787073	0.46794913	0.497096428	-1.00840236	down	-	-
MEDL02710	Indole-3-acetamide	Alkaloids	Plumerane	C10H10N2O	3	0.7784	879-37-8	835.71	3175.42	2369.68	221.5	883.74	661.24	1.21352775	0.14601245	0.4294713	3.612160908	1.852862161	up	C02693	ko00380,ko01100
MEDP0272	5-Hydroxyindoleacetic acid	Alkaloids	Plumerane	C10H9NO3	3	0.7722	54-16-0	9461.02	19852.97	23411.9	436.35	3644.74	4087.87	1.32766483	0.0620829	0.28817563	6.454418922	2.690287219	up	C05635	ko00380,ko01100
MEDN0221	D-Mannose	Others	Saccharides	C6H12O6	3	0.7706	530-26-7 | 46032-76-2	21810.18	12785.64	17678.02	8732.8	7788.02	4227.25	1.41276164	0.03710592	0.22896686	2.519455545	1.333112	up	C00159	ko00051,ko00052,ko00520,ko01100,ko01250,ko02010
MEDL01834	Capsianoside I	Terpenoids	Ditepenoids	C32H52O14	3	0.7696	121924-04-7	5112.06	12971.65	12719.46	38279.11	46173.41	30635.71	1.4740873	0.01041694	0.14566058	0.267648308	-1.90158956	down	-	-
MEDL00689	Phenylpyruvic acid	Organic acids	Organic acids	C9H8O3	3	0.7695	156-06-9	187.06	461.59	345.55	85.46	100.24	88.84	1.49485492	0.09418727	0.34891205	3.621330225	1.856519741	up	C00166	ko00360,ko00400,ko00470,ko00960,ko01100,ko01110,ko01210,ko01230
MEDN1303	D-Mannose 1-[illegible]	Others	Saccharides	C6H13O9P	3	0.768	27251-84-9	7508.82	6243.27	12636.53	22085.26	21878.72	18356.29	1.46866746	0.01049329	0.14566058	0.423435585	-1.23978558	down	-	-
MEDL02036	Epigallocatechin gallate	Flavonoids	Flavanols	C22H18O11	3	0.7677	989-51-5	46.65	27.53	24.02	105.86	147.72	31.55	1.09724811	0.20425673	0.4907574	0.344404307	-1.53782491	down	C09731	-
MEDN0733	Phenyl beta-D-glucopyranoside	Phenolic acids	Phenolic acids	C12H16O6	3	0.7617	1464-44-4	13533.05	13756.22	15103.24	2669.39	1995.17	3765.25	1.59305268	9.29E-05	0.04121132	5.028880841	2.330237369	up	C03097	-

MW0139681	Scropolioside B	Benzene and substituted derivatives	Benzene and substituted derivatives	C41H46O17	3	0.7616	116084-90-3	753.26	244.29	1363.98	4289.32	9104.48	4470.47	1.45364849	0.07619311	0.31624084	0.132192919	-2.91928319	down	-	-
MW0123724	Diosmetin	Flavonoids	Flavones	C16H12O6	3	0.7584	520-34-3	1616.45	2009.72	1599.72	1030.95	973.49	463.68	1.35914637	0.01755619	0.17028658	2.11735653	1.082264218	up	C10038	-
MEDN1041	9(S)-HpOTrE	Lipids	Free fatty acids	C18H30O4	3	0.7582	111004-08-1	30268.48	56763.81	34654.29	17735.64	17086.42	19390.81	1.4596201	0.11016317	0.37445293	2.244606862	1.166462782	up	-	-
MEDL00926	Paraxanthine	Nucleotides and derivatives	Nucleotides and derivatives	C7H8N4O2	3	0.7561	611-59-6	11254.43	13839.15	11428.14	3868.9	3895.68	3225.69	1.61495833	0.00649858	0.13398007	3.323095793	1.732527881	up	C13747	ko00232,ko01100,ko01110
MW0146440	Auriculoside	Terpenoids	Steroid	C22H26O10	3	0.7555	75871-96-4	12888.52	31480.35	7946.45	25007.37	86334.27	78895.15	1.23886378	0.12792613	0.40364464	0.275001066	-1.86249089	down	C09478	-
MW0108427	N-(3-methoxypropyl)-2-piperazin-1-ylacetamide	Alcohol and amines	Amines	C10H21N3O2	3	0.7553	871217-92-4	29598.04	37038.87	39617.25	18024.68	16196.17	18179.33	1.57636756	0.02287682	0.18655341	2.02774418	1.019875653	up	-	-
MW0149306	Isocromadurine	Heterocyclic compounds	Heterocyclic compounds	C16H23NO5	3	0.7525	57495-69-9 | 6029-87-4	188.3	157.27	151.79	482.74	551.03	218.02	1.3407514	0.12992338	0.40526903	0.397319039	-1.33163017	down	C10304	-
MW0106097	(±)-Camphoric acid	Organic acids	Organic acids	C10H16O4	3	0.752	5394-83-2	95.68	440.5	114.2	518.63	790.24	593.31	1.29749379	0.04422399	0.24760945	0.341912963	-1.54829897	down	-	-
MW0154306	Narcotoline	Alkaloids	Isoquinoline alkaloids	C21H21NO7	3	0.7518	521-40-4	14572.87	22786.05	26684.46	51971.74	40337.69	43160.76	1.45513761	0.00891147	0.14144336	0.472748876	-1.08085407	down	C09593	ko00950,ko01110
MW0122906	Arborine	Alkaloids	Alkaloids	C16H14N2O	3	0.7508	6873-15-0	692.34	281.89	327.91	158.22	55.86	235.66	1.21599638	0.14802426	0.4322191	2.895317294	1.53372146	up	C10642	-
MW0116587	1,2-Di(2-pyridyl)ethylene	Alkaloids	Pyridine alkaloids	C12H10N2	3	0.7506	1437-15-6 | 13341-40-7	3006.28	7034.14	2192.23	6173.45	37459.09	28181.75	1.27506084	0.16256998	0.45025659	0.170337269	-2.55353397	down	-	-
MEDP0247	Nicotinuric acid	Amino acids and derivatives	Amino acids and derivatives	C8H8N2O3	3	0.7473	583-08-4	65101.93	99595.62	149810.67	56453.14	30941.29	40004.73	1.35365689	0.11651698	0.383923	2.468683624	1.303741959	up	C05380	ko00760
MW0000222	Deserpidine	Alkaloids	Plumerane	C32H38N2O8	3	0.7453	131-01-1	56.73	9.01	12.18	7.86	1.85	7.77	1.08873826	0.32053253	0.5917111	4.457665904	2.156288493	up	C06541	-
MW0145019	Amataine	Alkaloids	Alkaloids	C43H48N4O6	3	0.7439	31148-60-4	20.03	9.77	221.76	329.94	307.45	290.24	1.23246045	0.07858211	0.32161807	0.27118571	-1.88264694	down	C08433	-
MW0154530	Nitrovin	Others	Ketone compounds	C14H12N6O6	3	0.7396	804-36-4	6400.2	6593.06	5044.22	2652.27	1974.56	2189.19	1.58491701	0.00849799	0.14010122	2.646336132	1.403996321	up	C19487	-
MEDN1006	Uric acid	Nucleotides and derivatives	Nucleotides and derivatives	C5H4N4O3	3	0.7396	69-93-2	254603.06	193104.61	260190.79	100809.12	101925.52	95905.33	1.59129169	0.023243	0.1872421	2.370407618	1.245135168	up	C00366	ko00230,ko01100
MEDN0364	1-Octadecanoyl-sn-glycero-3-phosphoethanolamine	Lipids	LPE	C23H48NO7P	3	0.7395	69747-55-3	7596.91	7754.6	8935.73	1496.48	2919.31	4747.92	1.38317759	0.01960703	0.17654332	2.650371956	1.406194843	up	-	-
MW0105439	Adouetine X	Amino acids and derivatives	Amino acids and derivatives	C28H44N4O4	3	0.7374	19542-37-1	4384.27	2671.62	3590.88	435.48	776.33	335.08	1.56725493	0.01974732	0.17658189	6.882693663	2.782973299	up	C09993	-
MEDL00643	Phenylacetylglutamine	Amino acids and derivatives	Amino acids and derivatives	C13H16N2O4	3	0.7371	28047-15-6	141160.33	124025.34	119259.11	32329.93	23064.65	35954.47	1.60224236	0.00075655	0.06544864	4.208525212	2.07331476	up	C04148	ko00360
MW0151872	3H-isoindol-1-yl (3S,4S,5R)-3,4,5-trihydroxy-5-(hydroxymethyl)tetrahydrofuran-2-carboxylate	Alkaloids	Plumerane	C14H15NO7	3	0.7369	20307-14-6	153511.89	20381.81	8082.15	257145.06	255428.5	217636.22	1.26196075	0.05049905	0.26113586	0.249210371	-2.00456399	down	C08494	-
MW0130029	Cudraflavanone B	Flavonoids	Flavones	C20H20O6	3	0.7351	597542-74-0	1091.2	1119.1	942.97	588.54	228.44	718.96	1.2354185	0.0527829	0.26722901	2.052990351	1.037726847	up	-	-
MW0151970	JWH 309	Others	Lactones	C30H27NO	3	0.7344	914458-42-7	2444.09	2288.15	902.54	1387.77	211.91	523.28	1.10735729	0.13147782	0.4072005	2.654209217	1.408282095	up	-	-
MEDP0426	Xanthurenic acid	Organic acids	Organic acids	C10H7NO4	3	0.7326	59-00-7	2662.71	1107.74	2792.26	642.71	1323.24	534.44	1.24485335	0.11366729	0.37822354	2.624674551	1.392138545	up	C02470	ko00380
MW0151749	Integerressine	Benzene and substituted derivatives	Benzene and substituted derivatives	C33H38N4O4	3	0.7316	17948-40-2	7768.07	13897.49	7244.04	1483.29	3788.4	1965.08	1.46823136	0.065444	0.29463659	3.994820894	1.998130822	up	C10006	-
MW0154722	Oleanoglycotoxin-A	Terpenoids	Triterpene Saponin	C48H78O18	3	0.7258	50657-29-9	2.24	3.14	81.31	66.22	104.24	43.69	1.08592252	0.2592451	0.54003277	0.404809713	-1.30468419	down	C08963	-
ZINC1330853	Aeginetolide	Heterocyclic compounds	Heterocyclic compounds	C11H18O3	3	0.7253	53537-93-2	11.3	296	4.11	301.27	288.15	238.81	1.11927036	0.21160693	0.4976598	0.375994591	-1.41121619	down	-	-
MW0151998	Kandelin A-1	Flavonoids	Flavones	C39H32O15	3	0.7245	88903-77-9	303.37	445.53	726.92	303.24	22.61	144.04	1.12231571	0.09788604	0.35419276	3.140777629	1.651121802	up	-	-
MEDL01815	Verbasoside	Phenolic acids	Phenolic acids	C20H30O12	3	0.7226	61548-34-3	7424.85	7284.62	5684.19	3855.33	3414.52	489.99	1.12761878	0.03801434	0.2307381	2.628103157	1.394021905	up	-	-
MW0141958	2,3,3-Triphenylacrylonitrile	Heterocyclic compounds	Heterocyclic compounds	C21H15N	3	0.7223	6304-33-2 | 7136-23-4	5153.95	10932.5	14905.36	1509.7	5200.67	81.98	1.11916733	0.08511888	0.33287184	4.562752214	2.189904309	up	-	-
MW0000487	Voacamine	Alkaloids	Alkaloids	C43H52N4O5	3	0.7206	3371-85-5	1481.92	1551.44	1752.95	547.21	455.48	381.32	1.60585031	0.00084642	0.06912669	3.458291486	1.790059473	up	C09252	-
MEDN1197	8-Methoxyalkynurate	Organic acids	Organic acids	C11H9NO4	3	0.718	2929-14-8	589.67	1353.44	449.66	555.44	127.42	305.21	1.07062321	0.23258124	0.51669106	2.421660409	1.275996569	up	C05830	ko00380
MEDN1476	Allantoic acid	Organic acids	Organic acids	C4H8N4O4	3	0.7156	99-16-1	1145.14	562.42	1108.27	187.3	433.61	89.18	1.35561729	0.04477123	0.24940742	3.965455083	1.987486442	up	C00499	ko00230,ko01100
MW0153335	Mallotophenone	Benzene and substituted derivatives	Benzene and substituted derivatives	C21H24O8	3	0.7144	98569-63-2	5329.07	7324.08	4800.63	3072.49	1777.96	2957.7	1.44281777	0.03333784	0.22151588	2.235328471	1.160486844	up	C10708	-
MEDL02741	Xanthoxylin	Phenolic acids	Phenolic acids	C10H12O4	3	0.7115	90-24-4	1585.92	1080.95	1173.54	3504.24	5077.66	5080.69	1.57817122	0.01812968	0.17351004	0.281089457	-1.83089875	down	C10726	-
MW0000811	2-Methoxy-1-(2-methoxynaphthalen-1-yl)naphthalene	Benzene and substituted derivatives	Benzene and substituted derivatives	C22H18O2	3	0.7093	75640-87-8 | 2960-93-2 | 35294-28-1 | 75685-01-7	5189.02	1881.79	1953.78	19605.02	57780.45	22249.72	1.51888476	0.13249838	0.40854422	0.090576332	-3.46472208	down	-	-
MEDL02353	Rosmarinic acid	Phenolic acids	Phenolic acids	C18H16O8	3	0.7057	20283-92-5	29255.02	14160.87	20230.94	5189.73	5130.43	2824.69	1.53073249	0.05759019	0.28045023	4.841959399	2.275590982	up	C01850	ko00350,ko01100
MEDN0417	Mevalonate	Organic acids	Organic acids	C6H12O4	3	0.7036	150-97-0	300.87	147.46	135.72	48.71	105.61	42.99	1.34460159	0.12347128	0.39475022	2.960062845	1.565627806	up	C00418	ko00900,ko01100,ko01110
MW0149004	Esmeraldic acid	Organic acids	Organic acids	C30H22N4O5	3	0.7031	119936-20-8	51980.69	104274.23	59028.38	39947.1	28675.37	28340.75	1.3808198	0.13134182	0.4072005	2.220257331	1.150726896	up	C12116	-
FDATN0056	Nordihydroguaiaretic acid	Benzene and substituted derivatives	Benzene and substituted derivatives	C18H22O4	3	0.7021	500-38-9 | 27686-84-6	2092.08	1808.13	1124.4	4333.97	5579.24	4214.21	1.52109019	0.00677578	0.13456926	0.355663667	-1.49141449	down	-	-
MW0104363	Acetamide, 2-(dimethylamino)-N-(5-ethyl-1,3,4-thiadiazol-2-[illegible]	Alcohol and amines	Amines	C8H14N4OS	3	0.7006	111750-46-0	32.43	3.77	12.11	58.06	86.99	55.67	1.32154952	0.01918912	0.17648958	0.240683539	-2.05479062	down	-	-
MEDN1616	Val-Pro-Leu	Amino acids and derivatives	Amino acids and derivatives	C16H29N3O4	3	0.6998	90614-49-6	39319.85	24472.76	31231.71	6096.19	4514.39	14031.76	1.45820131	0.01418987	0.15930201	3.856140285	1.947157537	up	-	-

MW0009787	Sulfadimethoxine	Benzene and substituted derivatives	Benzene and substituted derivatives	C12H14N4O4S	3	0.6979	122-11-2	4991	15626.18	14608.28	23055.25	26286.8	21421.63	1.19740668	0.05651635	0.27829256	0.497790109	-1.00639053	down	-	-
MW0003614	3,4-Dihydro-8-hydroxy-3-(3-hydroxy-4-methoxyphenyl) 1H-2-benzopyran-1-one	Phenolic acids	Phenolic acids	C16H14O5	3	0.6969	21499-23-0	1494.49	848.17	476.52	51.96	121.11	18.28	1.4897498	0.09704566	0.35349748	14.73310687	3.880989788	up	-	-
MW0107834	Leu-Met	Amino acids and derivatives	Amino acids and derivatives	C11H22N2O3S	3	0.6969	36077-39-1	3524.29	2394.86	2134.54	5854.78	6103.49	7732.79	1.5255191	0.00769563	0.13950182	0.409002359	-1.28981893	down	-	-
MW0107770	Leu-Val-Leu	Amino acids and derivatives	Amino acids and derivatives	C17H33N3O4	3	0.6968	58337-01-2	46.52	101.65	143.52	24	21.9	30.79	1.4267345	0.12409387	0.39559405	3.803494589	1.927325552	up	-	-
MW0148256	Demethylalangiside	Alkaloids	Alkaloids	C24H29NO10	3	0.6949	47763-23-5	19541.75	34705.06	17364.1	50046.71	51330.55	48796.15	1.43353452	0.03865006	0.23094812	0.476854791	-1.06837808	down	C11813	ko00950
MW0141891	2-(Formamido)-N1-(5-phospho-D-ribosyl)acetamidine	Others	Others	C8H16N3O8P	3	0.6934	6157-85-3	2499.99	6366	6567.55	2681.78	1348.31	1903.52	1.26213918	0.12997168	0.40526903	2.601037143	1.379087001	up	C04640	ko00230,ko01100,ko01110
MEDN0754	17-HDoHE	Lipids	Free fatty acids	C22H32O3	3	0.6934	90780-52-2	772.25	401.89	133.47	1913.75	831.66	850.36	1.1560311	0.15453824	0.43914832	0.363652291	-1.45936843	down	-	-
MW0141012	1-(2-Hydroxy-3,4,5,6-tetramethoxyphenyl)-3-(2,3,4,6-tetramethoxyphenyl)-2-propen-1-one	Others	Ketone compounds	C23H28O10	3	0.6908	93827-71-5	12.37	28.62	29.4	19.15	4.43	7.34	1.10215982	0.14249213	0.42363564	2.276520052	1.186830167	up	-	-
MW0138375	(S)-7-(((2-O-6-Deoxy-alpha-L-mannopyranosyl)-beta-D-glucopyranosyl)oxy)-2,3-dihydro-5-hydroxy-2-(3-hydroxy-4-methoxyphenyl)-4H-1-benzopyran-4-one	Flavonoids	Flavanones	C28H34O15	3	0.6883	13241-33-3	15216.66	21724.69	20642.28	819.3	2670.7	1869.13	1.56570311	0.0093129	0.14350153	10.7449586	3.425588019	up	C09806	ko00941
MW0159351	Vinblastine	Alkaloids	Plumerane	C46H58N4O9	3	0.6878	865-21-4	2856.43	1048.14	2327.71	385.28	460.62	637.33	1.46283359	0.09560322	0.35179433	4.20182979	2.071017722	up	-	-
MW0010818	(10E,12Z)-9-HODE	Lipids	Free fatty acids	C18H32O3	3	0.687	98524-19-7	5516.86	7135.51	13537	3821.6	3062.53	5568.65	1.20898655	0.19558941	0.48243539	2.103094249	1.072513505	up	-	-
MW0146926	Butrin	Flavonoids	Flavones	C27H32O15	3	0.6814	492-13-7	3498260.44	1700418.54	3475524.73	1072071.76	1869057.79	1184953.29	1.2635468	0.11086166	0.37538116	2.102285399	1.071958538	up	C09616	-
MW0002360	2,6-diamino-4-(4-methoxyphenyl)-4H-thiopyran-3,5-dicarbonitrile	Phenolic acids	Phenolic acids	C14H12N4OS	3	0.6813	102423-74-5	30978.87	17918.73	25134.42	213.07	463.35	366.9	1.61900856	0.02322867	0.1872421	70.95811448	6.148895769	up	-	-
MW0114728	Lex-lactose	Others	Saccharides	C32H55NO25	3	0.6813	213250-53-4	1148.27	1099.26	100.54	2760.42	2114.83	2090.35	1.11893643	0.02563023	0.19769335	0.337095153	-1.56877221	down	-	-
MW0113931	Amygdalin	Alkaloids	Alkaloids	C20H27NO11	3	0.6778	29883-15-6	76290.62	76378.27	120540.45	38795.94	29734.25	31331.05	1.54211289	0.05478226	0.27218393	2.735889721	1.452010079	up	C08325	ko00460,ko01100,ko01110
MW0053734	Glycerophospho-N-Palmitoyl Ethanolamine	GL	PE	C21H44NO7P	3	0.677	100575-09-5	17766.18	18051.56	19094.51	7568.13	7386.41	11246.15	1.53516013	0.01089483	0.14566058	2.095832209	1.06752322	up	-	-
MW0125969	Oleoyl oxazolopyridine	Heterocyclic compounds	Heterocyclic compounds	C24H36N2O2	3	0.6765	288862-58-8	342.17	444.33	444.04	1770.66	494.47	1944.79	1.25117379	0.16125054	0.44827043	0.292295341	-1.77450126	down	-	-
MW0144229	Acanthoside D	Lignans and Coumarins	Lignans	C34H46O18	3	0.6755	66791-77-3	6181	640.8	6820.77	124.91	935.68	84.44	1.26493111	0.16535459	0.45328035	11.91459612	3.574658143	up	-	-
MW0153874	Met-phe	Amino acids and derivatives	Amino acids and derivatives	C14H20N2O3S	3	0.6749	14492-14-9	33.9	76.54	2343.69	1355.38	1457.63	2286.87	1.05095856	0.37116518	0.62890784	0.481213283	-1.05525163	down	-	-
MEDN1335	Cinnamoylglycine	Amino acids and derivatives	Amino acids and derivatives	C11H11NO3	3	0.6722	16534-24-0	24448.85	30211.55	27513.01	62377.87	55135.16	54099.86	1.59910231	0.00137828	0.08001267	0.478830058	-1.06241438	down	-	-
MW0001180	(±)-threo-1-(4-Hydroxyphenyl)-1,2,3-propanetriol	Phenolic acids	Phenolic acids	C9H12O4	3	0.67	25743-67-3	2383.79	2066.58	3033.98	643.96	1055.58	1235.62	1.47722616	0.01603083	0.16485394	2.549895065	1.350437878	up	-	-
MW0112859	3-Methyl-1-(2,4,6-trihydroxyphenyl)-1-butanone	Others	Ketone compounds	C11H14O4	3	0.6682	26103-97-9	4.77	6.1	451.17	389.75	925.83	588.24	1.19681806	0.09006831	0.3408345	0.242691011	-2.04280742	down	C07350	-
MEDN1615	Ile-Pro-Ile	Amino acids and derivatives	Amino acids and derivatives	C17H31N3O4	3	0.6677	90614-48-5	6216.9	7448.11	14264.2	4735.64	4697.5	4162.05	1.28817826	0.19583903	0.48243539	2.054344956	1.038678452	up	-	-
MW0146895	Bryophyllin A	Others	Others	C26H32O8	3	0.6668	105608-32-0	859.63	981.52	1164.48	379	514.57	525.16	1.53489724	0.01280061	0.15141102	2.118535592	1.083067366	up	-	-
MEDL02192	Vestitol	Flavonoids	Other Flavonoids	C16H16O4	3	0.6637	56701-24-7	26835.12	24260.51	9575.5	84838.88	298982.95	110589.25	1.46399518	0.16469399	0.45301115	0.122713937	-3.02662899	down	-	-
MW0118129	2-[2-(1H-indol-3-yl)ethyl]hexahydro-1H-isoindole-1,3(2H)-dione	Alkaloids	Plumerane	C18H20N2O2	3	0.6634	432015-53-7	1869.44	1788.94	1096.78	3491.03	2442.92	4590.63	1.37922287	0.07474882	0.31230283	0.451814704	-1.14619687	down	-	-
MEDN0222	Melezitose	Others	Saccharides	C18H32O16	3	0.6631	597-12-6	7970.36	3287.7	10809.65	39924.36	62768.82	48959.22	1.52959031	0.01538456	0.16355823	0.145515073	-2.7807595	down	C08243	-
MW0114793	Maltopentaose	Others	Saccharides	C30H52O26	3	0.6603	34620-76-3	8548.61	8865.17	11207.43	25116.03	18680.9	25232.32	1.56470749	0.01515048	0.16263723	0.41462438	-1.27012314	down	-	-
MW0152081	Lacto-N-triaose	Others	Saccharides	C20H35NO16	3	0.6601	75645-27-1	335437.86	274123.72	336180.16	94420.3	134465.98	92989.26	1.58224882	0.00197883	0.08476296	2.938221836	1.554943323	up	-	-
MEDP1669	Alanylphenylalanine	Amino acids and derivatives	Amino acids and derivatives	C12H16N2O3	3	0.6597	3061-90-3	21820.19	18657.41	26888.92	8387.58	8632.49	9623.25	1.58495441	0.02734487	0.20385331	2.528458165	1.338257908	up	-	-
MW0010988	(25S)-Spirostane-3b,5b,6a-triol 3-[4"-rhamnosylglucoside]	Terpenoids	Terpene	C39H64O14	3	0.6584	265092-98-6	2782.52	957.66	429.53	3009	2635.63	4296.95	1.16162113	0.0997242	0.35858449	0.419421259	-1.25352811	down	-	-
MW0152108	Lanceotoxin B	Others	Others	C32H44O11	3	0.657	93802-98-3	557177.57	250975.85	345567.52	127136.6	104546.12	158732.71	1.45911738	0.10260513	0.36273543	2.955111021	1.563212332	up	C08873	-
MEDTP01677	cis-5-Dodecenoic acid	Lipids	Free fatty acids	C12H22O2	3	0.6563	2430-94-6	41292.63	67397.25	58443.18	1154.28	2669.05	1042.8	1.60634763	0.01907239	0.17648958	34.34619708	5.102078463	up	-	-
MW0147254	Catheduline E2	Others	Lactones	C38H40N2O11	3	0.6555	61231-06-9	124.8	154.21	88.68	388.83	410.97	267.45	1.52070761	0.02169573	0.18323322	0.344520965	-1.53733632	down	C09935	-

MW0003469	Phenol, 3-(3-phenoxyphenoxy)-	Benzene and substituted derivatives	Benzene and substituted derivatives	C18H14O3	3	0.654	14200-84-1	3117.69	5213.14	3596.78	1258.29	2356.06	1806.54	1.40319677	0.0564248	0.27815218	2.200304747	1.137703354	up	-	-
MW0103542	Diguanosine-5'-triphosphate	Nucleotides and derivatives	Nucleotides and derivatives	C20H27N10O18P3	3	0.652	6674-45-9 | 102783-44-8	31629.53	6256.57	35038.79	65165.84	39087.34	42881.74	1.01638985	0.11315568	0.37822354	0.495632784	-1.01265648	down	-	-
MEDN1244	4-(Cytidine 5'-diphosphate) -2-C-methyl-D-erythritol	Nucleotides and derivatives	Nucleotides and derivatives	C14H25N3O14P2	3	0.6517	263016-94-0	15063.23	38552.65	34036.23	954.74	16430.52	10115.08	1.07070501	0.08992031	0.34083195	3.187310048	1.672339365	up	C11435	ko00900,ko01100,ko01110
MEDL02107	Desaminotyrosine	Phenolic acids	Phenolic acids	C9H10O3	3	0.6511	501-97-3	106	220.25	150.91	16.23	45.33	20.1	1.5094423	0.04951377	0.25944745	5.84325251	2.546771635	up	C01744	ko01100
MW0105602	Amastatin	Amino acids and derivatives	Amino acids and derivatives	C21H38N4O8	3	0.6509	67655-94-1	32279.5	74267.03	36381.77	168037.05	199476.03	77846.75	1.34539985	0.09602259	0.35181896	0.320927687	-1.63967984	down	C01552	-
MW0146549	Bayogenin 3-O-cellobioside	Organic acids	Organic acids	C42H68O15	3	0.6502	92622-05-4	41.57	43.99	64.95	300.08	346.49	269.04	1.6089049	0.00414905	0.11299759	0.164382215	-2.60487388	down	C08932	-
pme0321	Kaempferol-7-[illegible]	Flavonoids	Flavonols	C21H20O10	3	0.6488	20196-89-8	628.31	314.53	1923.28	202.05	164.49	138.42	1.31713249	0.25092008	0.53381884	5.675934728	2.504857999	up	-	-
MW0109829	Thapsigargin	Heterocyclic compounds	Heterocyclic compounds	C34H50O12	3	0.6446	67526-95-8 | 123269-03-4	27341.79	4550.15	354.42	47348.75	53087.17	43239.4	1.17395434	0.03621617	0.22604178	0.224439103	-2.15560404	down	C09561	-
MEDL01889	4-Methylbenzaldehyde	Others	Aldehyde compounds	C8H8O	3	0.6444	104-87-0	2608.44	1676.36	2757.96	334.28	377.19	362.16	1.60854574	0.02758373	0.20441439	6.559764537	2.71364403	up	C06758	ko01100
MW0157392	Terminalin	Organic acids	Organic acids	C28H10O16	3	0.6426	155144-63-1	1417.74	762.23	838.84	1462.3	3573.98	2344.06	1.3129324	0.12973643	0.40526903	0.409034001	-1.28970732	down	-	-
MW0146616	(2r,3s)-3-Phenylisoserine ethyl ester	Benzene and substituted derivatives	Benzene and substituted derivatives	C11H15NO3	3	0.6417	143615-00-3 | 153433-80-8	17709.59	14386.31	3492.01	40108.63	128529.89	61885.02	1.3855551	0.13115497	0.4069309	0.154378637	-2.69545497	down	-	-
MW0143420	4-Nitrophenyl-beta-D-galactopyranoside	Others	Saccharides	C12H15NO8	3	0.6416	3150-24-1	17325.81	16044.01	41643.22	208.6	3344.2	841.83	1.44847189	0.10380679	0.36522771	17.06925043	4.093327801	up	C04452	-
MEDP0370	Formylanthranilic acid	Phenolic acids	Phenolic acids	C8H7NO3	3	0.6411	3342-77-6	181815.47	206682	224324.46	45254.28	32796.64	34245.05	1.61791877	0.00282641	0.10221403	5.457203228	2.448161771	up	C05653	ko00380,ko01100
MEDN0670	O-Desmethylnaproxen	Benzene and substituted derivatives	Benzene and substituted derivatives	C13H12O3	3	0.6366	52079-10-4	1892.9	3740.74	5440.48	9697.28	8769.91	6641.65	1.31419231	0.02734477	0.20385331	0.441044668	-1.18100332	down	-	-
MEDN1037	p-Cresol	Phenolic acids	Phenolic acids	C7H8O	3	0.6365	106-44-5	351.31	228.3	297.44	79.7	227.6	81.41	1.27725008	0.06042705	0.28544343	2.256309331	1.173964869	up	C01468	ko01100
MW0015153	7-Acetyltaxol	Alcohol and [illegible]	Alcohols	C49H53NO15	3	0.6357	92950-39-5	3261.44	23123.6	4271.63	14099.76	33976.62	30421.66	1.11959778	0.14776856	0.43205719	0.390540579	-1.35645564	down	-	-
MEDL02408	Phloretin	Flavonoids	Chalcones	C15H14O5	3	0.6356	60-82-2	27797.89	20517.29	27476.76	6418.41	3949.85	4600.46	1.59686127	0.0085627	0.14010122	5.063354783	2.340093576	up	C00774	ko00941,ko01100,ko01110
MW0017157	cis-4-Octenedioic acid	Lipids	Free fatty acids	C8H12O4	3	0.6311	38561-68-1	68201.74	29987.37	48897.63	9105.78	5113.53	25308.06	1.33178203	0.06241401	0.28828091	3.721136519	1.89574332	up	-	-
mws0895	Genistin	Flavonoids	Isoflavones	C21H20O10	3	0.6248	529-59-9	1383.2	451.52	2780.24	399.18	254.79	190.56	1.29749698	0.20353244	0.49043425	5.464530567	2.450097566	up	C09126	ko00943
ZINC5822860	Philonotisflavone	Flavonoids	Flavones	C30H18O12	3	0.621	124615-12-9	14517.8	9276.76	35246.06	56462.88	83153.99	47395.74	1.36093788	0.03709672	0.22896686	0.315703952	-1.66335578	down	-	-
MEDN1233	4-Hydroxyphenylacetylglycine	Amino acids and derivatives	Amino acids and derivatives	C10H11NO4	3	0.62	28116-23-6	533.18	54.24	517.82	12068.43	4658.39	7677.35	1.47870804	0.06814275	0.29819913	0.045288981	-4.4646961	down	C05596	ko00350
MW0156618	Cashmiradelphine	Terpenoids	Ditepenoids	C37H52N2O11	3	0.6193	69787-05-9	11.61	2.28	1.25	13.49	18.76	18.42	1.26628233	0.04966126	0.25991159	0.298796132	-1.74276662	down	C08668	-
MW0138186	Forsythiaside	Phenolic acids	Phenolic acids	C29H36O15	3	0.6186	79916-77-1	535.28	529.52	507.08	294.18	120.43	370.84	1.23846056	0.06952985	0.30163786	2.001247692	1.000899739	up	-	-
MW0155643	Podorhizol beta-D-glucoside	Lignans and Coumarins	Lignans	C28H34O13	3	0.6185	17187-73-4	7758.2	9302.82	7975.08	827	2603.99	3183.89	1.41666005	0.00321107	0.10290142	3.784815446	1.920222954	up	-	-
MW0152626	Leu-Ser	Amino acids and derivatives	Amino acids and derivatives	C9H18N2O4	3	0.614	6209-12-7	1667.36	603.83	727.04	40.09	317.43	215.27	1.2990812	0.1309708	0.4069309	5.234431467	2.388032849	up	-	-
MW0158814	Undulatone	Terpenoids	Triterpene	C27H34O11	3	0.6139	70993-77-0	8561.4	6441.7	8458.73	26657.55	23955.14	24645.31	1.61109897	0.00010038	0.04121132	0.311751973	-1.6815294	down	C08787	-
MW0157448	Tetraneurin A	Terpenoids	Sesquiterpenoids	C17H22O6	3	0.6137	22621-72-3	67273.17	105748.97	66657.6	59423.67	16926.98	28710.91	1.2479533	0.06829949	0.29819913	2.281326681	1.189873052	up	C09559	-
MW0123053	O6-Cyclohexylmethoxy-2-(4'-sulphamoylanilino)purine	Alcohol and amines	Amines	C18H22N6O3S	3	0.6123	444722-95-6	14103.02	17349.67	12189.9	2262.29	1905.88	5453.7	1.50471784	0.00486113	0.11959415	4.535770074	2.181347509	up	-	-
MW0105527	L-Alanyl-L-methionine	Amino acids and derivatives	Amino acids and derivatives	C8H16N2O3S	3	0.6095	14486-05-6	31.1	30.91	429	569.23	425.96	384.32	1.16699146	0.14230897	0.42362422	0.355930729	-1.4903316	down	-	-
MW0114621	Indican	Alkaloids	Plumerane	C14H17NO6	3	0.6085	487-60-5	11215.82	3351.12	11268.2	5086.09	1868.55	1738.69	1.16284581	0.14969528	0.43451026	2.971834728	1.571353886	up	-	-
MW0159385	Volkenin	Heterocyclic compounds	Heterocyclic compounds	C12H17NO7	3	0.6084	66575-40-4	508.54	927.42	500.11	477.93	96.23	95.43	1.23736899	0.09117591	0.34265588	2.891426097	1.531781228	up	C08344	-
MW0014781	5-HPETE	Organic acids	Organic acids	C20H32O4	3	0.608	74581-83-2	692.47	693.88	3342.77	93.99	189.4	147.71	1.44512871	0.24615589	0.5293161	10.96989098	3.455477283	up	C05356	ko00590,ko01100
MW0054215	Kirenol	Terpenoids	Ditepenoids	C20H34O4	3	0.6074	52659-56-0	127.65	43.36	42.37	604.71	183.42	335.92	1.40564182	0.12624351	0.4002779	0.189831413	-2.39720935	down	-	-
MW0157956	Toonacilin	Terpenoids	Sesquiterpenoids	C31H38O9	3	0.6068	66610-71-7	3603.7	3109.51	2535.53	1529.33	614.58	2025.99	1.25668788	0.03413667	0.22466028	2.21797645	1.149244048	up	C08785	-
MEDL02381	4-Ethylbenzoic acid	Phenolic acids	Phenolic acids	C9H10O2	3	0.604	619-64-7	1657.38	599.31	271.52	364.26	15.19	162.38	1.07455321	0.25090194	0.53381884	4.66605762	2.222204123	up	-	-
MEDL02671	Sudachiin B	Flavonoids	Flavones	C30H34O17	3	0.604	98891-96-4	7523.8	5659.1	8034.32	2681.77	940.34	4998.88	1.20995927	0.04880612	0.25758942	2.461111775	1.299310181	up	-	-
MW0133966	5,6,7-Trimethoxycoumarin	Lignans and Coumarins	Coumarins	C12H12O5	3	0.6032	55085-47-7	17778.17	20371.38	24704.04	7855.98	8529.45	10834.42	1.55419134	0.01552064	0.16393407	2.30910861	1.207336033	up	C09313	-
MW0002404	2,6-Dimethoxyphenol	Phenolic acids	Phenolic acids	C8H10O3	3	0.6017	91-10-1	1033.77	1916.22	4072.06	1338.23	527.59	1114.59	1.01811348	0.2711423	0.55153518	2.35606846	1.23638146	up	C10787	-
MW0123525	Dimethyl 2,6-dimethyl-4-(2-nitrophenyl)-3,5-pyridinedicarboxylate	Heterocyclic compounds	Heterocyclic compounds	C17H16N2O6	3	0.6007	67035-22-7	11407.54	9680.37	11674.78	3397.44	3123.95	5244.72	1.55246847	0.00158469	0.08134154	2.78449632	1.477416386	up	-	-
MW0147064	Canthiumine	Organic acids	Organic acids	C33H36N4O4	3	0.6003	26195-90-4	2968.99	2598.76	3039.82	5497.2	6200.36	6005.56	1.61094228	0.00060775	0.06185252	0.486217684	-1.04032573	down	C10000	-
MEDP1314	Monoisobutyl phthalate	Others	Lactones	C12H14O4	3	0.5998	30833-53-5	514.54	535.02	697.1	40.37	469.47	126.65	1.16616763	0.08905491	0.33912614	2.744206508	1.456389052	up	-	-
MW0112040	beta-N-Acetylglucosamine	Others	Saccharides	C8H15NO6	3	0.5967	14131-68-1	9.43	24.37	1707	3247.24	791.47	1553.75	1.11260886	0.23892487	0.52278164	0.311276254	-1.68373257	down	C03878	-
MW0145631	Artecanin	Others	Lactones	C15H18O5	3	0.5929	29431-84-3	61002.83	53269.56	43549.32	174161.07	173668.8	154969.93	1.60760471	0.00019106	0.04214726	0.313885785	-1.6716884	down	C09302	-
MW0148235	Delphinine	Benzene and substituted derivatives	Benzene and substituted derivatives	C33H45NO9	3	0.5927	561-07-9	13573.18	12437.48	13060.58	24435.25	29369.56	24426.39	1.60427404	0.01291992	0.15200711	0.499432963	-1.00163705	down	-	-

MW0103482	Adenylosuccinic acid	Nucleotides and derivatives	Nucleotides and derivatives	C14H18N5O11P	3	0.5909	19046-78-7	75.39	70.27	46.38	191.77	355.48	321.63	1.55303067	0.04183759	0.23972398	0.221020164	-2.1777501	down	C03794	ko00230,ko00250,ko01100,ko01232,ko01240
MW0007539	Magnolol	Phenolic acids	Phenolic acids	C18H18O2	3	0.5903	528-43-8	1768.08	1962.55	2210.66	259.14	65.92	1240.64	1.23550873	0.04450105	0.24836985	3.794654148	1.923968402	up	C10651	-
MW0007500	Levosimendan	Benzene and substituted derivatives	Benzene and substituted derivatives	C14H12N6O	3	0.587	141505-33-1	1087.7	1122.98	1185.93	104.34	317.31	411.16	1.43598756	0.00671072	0.13456926	4.078493294	2.02803628	up	-	-
MW0107309	His-Ile	Amino acids and derivatives	Amino acids and derivatives	C12H20N4O3	3	0.5743	129050-48-2	3123.39	3204.29	2283.12	2560.4	13123.52	7341.49	1.02726808	0.25560208	0.53680988	0.373969454	-1.41900766	down	-	-
MW0105581	Alitame	Organic acids	Organic acids	C14H25N3O4S	3	0.5688	80863-62-3	280.21	188	272.4	60.28	33.47	13.38	1.4962293	0.00900939	0.14235878	6.913189583	2.78935149	up	-	-
pme0170	N-Acetyl-L-arginine	Amino acids and derivatives	Amino acids and derivatives	C8H16N4O3	3	0.5637	155-84-0	191.23	132.87	132.01	3.43	55.66	30.5	1.26989887	0.00919264	0.14331421	5.091081594	2.347972188	up	-	-
MADN0626	1,6-di-O-phosphono-D-fructose	Organic acids	Organic acids	C6H14O12P2	3	0.5551	4937-84-2	284.7	159.8	35.46	2133.03	376.82	200.49	1.0330813	0.35115952	0.61456398	0.177084794	-2.49748776	down	-	-
MW0146886	Bruceine B	Terpenoids	Triterpene	C23H28O11	3	0.5521	25514-29-8	55.01	960.89	65.39	33.58	42.38	17.23	1.06332023	0.38708513	0.64348892	11.603069	3.536434542	up	C08751	-
MW0154656	2-Trimethylaminoethylphosphonic acid	Others	Lactones	C5H15NO3P+	3	0.5516	5148-60-7	693.16	5993.05	5576.49	27302.35	22474.16	21131.44	1.35175363	0.00157051	0.08134154	0.172938295	-2.53167072	down	C06459	ko00440,ko01100
MEDP1652	Leucovorin	Heterocyclic compounds	Pteridines and derivatives	C20H23N7O7	3	0.5498	58-05-9 | 1492-18-8	5857.34	4214.56	2989.76	6998.75	28111.28	8382.42	1.20166447	0.27435513	0.55474557	0.300320171	-1.73542672	down	-	-
MW0124076	Eugenitol	Heterocyclic compounds	Heterocyclic compounds	C11H10O4	3	0.5483	491-48-5	214.59	156.25	411.03	141.5	97.49	118.93	1.23934146	0.20558117	0.49134567	2.184482566	1.127291592	up	-	-
MW0001616	1-hydroxy-N-(3-nitrophenyl)naphthalene-2-carboxamide	Benzene and substituted derivatives	Benzene and substituted derivatives	C17H12N2O4	3	0.5424	71835-65-9	67.69	27.74	69.64	4.52	25.79	24.08	1.14965613	0.09615497	0.35181896	3.034932892	1.601664616	up	-	-
MW0132544	3',4',6-TRIMETHOXYISOFLAVONE-7-O-beta-D-GLUCOPYRANOSIDE	Flavonoids	Flavones	C24H26O11	3	0.535	59183-50-5	1815.45	2226.63	3001.6	83.11	8.95	107.29	1.51514568	0.02180181	0.18323322	35.33323301	5.142953857	up	-	-
MW0153814	Methylhesperidin	Flavonoids	Flavanones	C29H36O15	3	0.5315	11013-97-1 | 28034-80-2	10818.02	7296.67	11476.06	2618.7	5114.94	5011.83	1.39728099	0.0289312	0.20834886	2.321668012	1.215161688	up	C17393	-
MW0155445	Phe-gly	Amino acids and derivatives	Amino acids and derivatives	C11H14N2O3	3	0.5306	721-90-4	639786.63	592758.33	629279.81	248047.43	294146.38	304854.58	1.60947579	0.0001469	0.04121132	2.198014649	1.136201002	up	-	-
MW0111379	3-O-beta-D-Glucopyranosylandrographolide	Benzene and substituted derivatives	Benzene and substituted derivatives	C26H40O10	3	0.5244	869384-84-9	10102.99	4895.04	7829.74	15303	15815.47	14581.15	1.41468424	0.03126138	0.21652308	0.49951772	-1.00139224	down	-	-
MEDN0835	D-Glucuronolactone	Others	Saccharides	C6H8O6	3	0.5147	32449-92-6	78943.28	79506.62	86067.84	54979.19	17143.34	37067.33	1.30575326	0.04871338	0.25758942	2.239381386	1.163100252	up	C02670	ko00053,ko01100
MW0138677	kaempferol 3-O-beta-D-glucopyranosyl-7-O-alpha-L-rhamnopyranoside	Flavonoids	Flavonols	C27H30O15	2	0.9182	-	33144.65	44377.68	44458.34	21758.68	12408.77	22184.4	1.43992859	0.0120367	0.14858307	2.164625829	1.114117666	up	-	-
MW0157843	Thr-Tyr-Ile-Arg	Amino acids and derivatives	Amino acids and derivatives	C25H41N7O7	2	0.9174	-	1686.49	3336.06	2316.12	12652.78	14063.42	14308.11	1.59130017	9.3987E-05	0.04121132	0.178885885	-2.48288854	down	-	-
MW0145959	Asn-Val-Phe-Lys	Amino acids and derivatives	Amino acids and derivatives	C24H38N6O6	2	0.9	-	9314.57	2540.74	5356.72	71164.42	65916.75	49328.57	1.55936447	0.00876426	0.14010122	0.092334392	-3.43698808	down	-	-
MW0145812	Asn-Leu-Lys-Asp	Amino acids and derivatives	Amino acids and derivatives	C20H36N6O8	2	0.8977	-	9505.42	10805.84	11465.63	3723.62	7695.37	949.83	1.16529892	0.070486	0.30358605	2.569112494	1.361270063	up	-	-
MW0149981	Glu-Gln-Phe-Arg	Amino acids and derivatives	Amino acids and derivatives	C25H38N8O8	2	0.8965	-	25.34	24.11	266.31	5.47	5.95	7.23	1.31873211	0.3437979	0.60874501	16.9308311	4.081580889	up	-	-
MW0159124	Val-Phe-Lys-Asp	Amino acids and derivatives	Amino acids and derivatives	C24H37N5O7	2	0.8965	-	750.25	1689.23	50.29	3764.46	3472.21	1204.39	1.0333658	0.11830351	0.38749449	0.294959401	-1.7614117	down	-	-
MW0015331	2-[(1R,5S,6R,11R,13R,16S)-6-(furan-3-yl)-11-hydroxy-1,5,15,15-tetramethyl-8,14,17-trioxo-7-oxatetracyclo[11.3.1.02,11.05,10]heptadec-9-en-16-yl]acetic acid	Benzene and substituted derivatives	Benzene and substituted derivatives	C26H30O8	2	0.886	-	7940.75	4952.78	11180.7	2794.03	1075	798.03	1.45267385	0.05660581	0.27842233	5.158328798	2.366903735	up	-	-
MW0150116	Glu-Phe-Tyr-Lys	Amino acids and derivatives	Amino acids and derivatives	C29H39N5O8	2	0.8755	-	11009.12	7944.45	8403.07	14.33	22.91	64.27	1.61330341	0.01083979	0.14566058	269.4969954	8.074125378	up	-	-
MW0158456	Tyr-Glu-Gln-Asp	Amino acids and derivatives	Amino acids and derivatives	C23H31N5O11	2	0.8669	-	11093.41	14543.81	11827.15	4269.42	4746.26	8547.84	1.41532793	0.02009654	0.17840231	2.133078677	1.092937179	up	-	-
MW0154273	Nap-His-OH	Amino acids and derivatives	Amino acids and derivatives	C24H20N4O6	2	0.8574	-	50.26	116.6	58.83	652.5	931.96	53.8	1.03012634	0.21007352	0.4976598	0.137762016	-2.85974994	down	-	-
MW0145472	Arg-Phe-Tyr-Arg	Amino acids and derivatives	Amino acids and derivatives	C30H44N10O6	2	0.8551	-	1165.72	766.64	1628.41	252.13	330.22	158.1	1.52053541	0.05847378	0.28164447	4.808927004	2.265715027	up	-	-
MW0157545	Thr-Arg-Gln-Glu	Amino acids and derivatives	Amino acids and derivatives	C20H36N8O9	2	0.8289	-	80.93	117.73	241.37	407.07	482.47	682.5	1.44578527	0.02498895	0.1956303	0.27991018	-1.83696414	down	-	-
MW0155363	Phe-Thr-Tyr-Asp	Amino acids and derivatives	Amino acids and derivatives	C26H32N4O9	2	0.8218	-	7.13	16.25	19.3	88.15	43.14	202.15	1.42544067	0.17639006	0.46600774	0.12799904	-2.9657951	down	-	-
MW0153739	Met-Abu-OH	Amino acids and derivatives	Amino acids and derivatives	C14H18N2O6S	2	0.7976	-	60344.95	41608.06	61798.69	13818.77	7554.79	7755.14	1.57699773	0.01381597	0.15721285	5.621661797	2.490996663	up	-	-
MW0149956	Glu-Asp-Val-Asp	Amino acids and derivatives	Amino acids and derivatives	C18H28N4O11	2	0.7947	-	4336.78	818.51	3130.58	780.18	732.51	807.73	1.17358747	0.1938467	0.48183191	3.570849243	1.836267226	up	-	-

MEDP1345	LysoPC(18:3(9Z,12Z,15 Z))	Lipids	LPC	C26H48NO7P	2	0.792	-	58453.95	57927.5	58445.39	22542.88	21955.05	28535.27	1.60472926	0.00362512	0.10719376	2.393799532	1.259302339	up	-	-
MW0155414	Phe-Val-Ser-Arg	Amino acids and derivatives	Amino acids and derivatives	C23H37N7O6	2	0.7909	-	1100.85	244.5	191.16	20826.33	18054.32	13863.42	1.56880843	0.01239899	0.14878189	0.029131427	-5.10127984	down	-	-
MW0144792	Ala-Val-Asp-Asp	Amino acids and derivatives	Amino acids and derivatives	C16H26N4O9	2	0.7898	-	3123.05	722.94	1159.91	467.2	60.21	406.98	1.23034396	0.20455305	0.4907574	5.357398945	2.421532732	up	-	-
MW0168445	10-Hydroxydecanoic [illegible]	Lipids	Free fatty acids	C10H20O3	2	0.7738	-	9450.56	8989.62	10896.36	1771.19	3287.48	1170.1	1.52947503	0.00086448	0.06912669	4.709844801	2.235679521	up	C02774	-
MW0158703	TyrMe-HoPhe-OH	Amino acids and derivatives	Amino acids and derivatives	C26H26N2O7	2	0.7726	-	19159.93	10986.1	16012.52	5162.39	4551.1	9283.18	1.399404	0.04128481	0.23857599	2.429823227	1.28085136	up	-	-
MW0109387	Prolylhistidine	Amino acids and derivatives	Amino acids and derivatives	C11H16N4O3	2	0.7718	-	46891.06	31179.86	54286.29	11870.16	13514.17	19903.75	1.50296771	0.03857865	0.23094812	2.922561743	1.547233504	up	-	-
MW0015865	Arachidonoyl-1-thioglycerol	Lipids	Others	C23H38O3S	2	0.7681	-	1763.09	865.27	934.01	4390.26	4930.37	4987.53	1.5574971	0.00099746	0.06912669	0.248974711	-2.00592888	down	-	-
MW0155398	Phe-Tyr-Val-Lys	Amino acids and derivatives	Amino acids and derivatives	C29H41N5O6	2	0.7641	-	3046.56	2037.09	3352.02	201.37	314.87	1104.66	1.4246252	0.01221888	0.14864691	5.204312419	2.379707571	up	-	-
MW0157815	Thr-Thr-Gly-Leu-Ile	Amino acids and derivatives	Amino acids and derivatives	C22H41N5O8	2	0.7615	-	4985.46	4800.31	5268.5	1408.28	1525.49	2601.89	1.52956496	0.0079271	0.14010122	2.719507701	1.443345511	up	-	-
MW0158488	Tyr-His-Phe-Lys	Amino acids and derivatives	Amino acids and derivatives	C30H39N7O6	2	0.7591	-	626.11	722.8	773.67	173.74	128.44	72.95	1.56744297	0.00073091	0.06511911	5.658251806	2.500356382	up	-	-
MW0109142	Phe-Val	Amino acids and derivatives	Amino acids and derivatives	C14H20N2O3	2	0.7578	-	303.44	468.15	276.55	928.62	1928.06	717.95	1.41241788	0.14950361	0.43451026	0.293216361	-1.76996249	down	-	-
MW0146227	Asp-Phe-Ser-Arg	Amino acids and derivatives	Amino acids and derivatives	C22H33N7O8	2	0.7568	-	38642.57	31558.82	13462.57	37016.6	90920.31	85120.98	1.20881036	0.11206299	0.3775421	0.39268182	-1.34856729	down	-	-
MW0156960	Ser-Tyr-Lys-Asp	Amino acids and derivatives	Amino acids and derivatives	C22H33N5O9	2	0.7546	-	107.35	469.07	473.48	40.04	121.21	85	1.23093026	0.15353482	0.43832974	4.263553299	2.092056292	up	-	-
MW0158715	TyrMe-Trp-OH	Amino acids and derivatives	Amino acids and derivatives	C27H25N3O7	2	0.7519	-	868.42	7465.74	3768.7	17910.64	13889.05	13916.2	1.28817032	0.01129141	0.14569982	0.264740772	-1.9173477	down	-	-
MW0158880	Val-Ala-Ser-Asp	Amino acids and derivatives	Amino acids and derivatives	C15H26N4O8	2	0.7507	-	32647.38	23555.65	31493.47	6643.64	2240.07	7532.16	1.49226469	0.00448456	0.11919209	5.342178027	2.417428054	up	-	-
MW0109785	2-(3-(1-(((4E,10E)-2,9-Dioxo-12-isopropyl-1,8-diazacyclododecane-4,10-diene-3-yl)carbamoyl)-2-methylpropyl)ureido)-3-methylbutyric acid	Heterocyclic compounds	Heterocyclic compounds	C24H39N5O6	2	0.7498	-	133061.28	113500.97	126852.86	30412.43	33101.97	43755.42	1.60423433	0.00042328	0.0518758	3.481082657	1.799536071	up	-	-
MW0151128	His-Tyr-Glu-Asp	Amino acids and derivatives	Amino acids and derivatives	C24H30N6O10	2	0.7462	-	263180.45	138966.04	267698.39	92405.09	132294.49	98608.53	1.3397426	0.10149989	0.36143464	2.071846821	1.050917344	up	-	-
MW0005023	4-Hydroxy-2-(1-methylethenyl)-2,3-dihydrobenzofuran-5-carboxylic acid	Benzene and substituted derivatives	Benzene and substituted derivatives	C12H12O4	2	0.746	-	1152.71	2454.53	1790.38	7408.1	10096.73	9074.22	1.56731056	0.00455437	0.11959415	0.203077988	-2.29989422	down	-	-
MW0149376	Gal(1-3)[Fuc(1-2)]Gal(1-4)GlcSp	Others	Saccharides	C26H45N3O20	2	0.7449	-	322.95	93.12	641.87	1114.77	555.97	953.78	1.12311078	0.0857986	0.33324725	0.403098471	-1.31079578	down	-	-
MW0144694	Ala-Phe-Lys-Arg	Amino acids and derivatives	Amino acids and derivatives	C24H40N8O5	2	0.7386	-	1897.4	1336.14	1959.06	550.41	1523.27	499.99	1.2035579	0.10259992	0.36273543	2.017585782	1.012630014	up	-	-
MW0146199	Asp-Lys-Tyr-Asp	Amino acids and derivatives	Amino acids and derivatives	C23H33N5O10	2	0.7367	-	3402.09	4002.97	4156.6	1821.23	1250.47	1718.8	1.56643658	0.00191852	0.08476296	2.413455798	1.271100404	up	-	-
MW0153090	Lys-Phe-Asn-Lys	Amino acids and derivatives	Amino acids and derivatives	C25H41N7O6	2	0.7363	-	118.6	211.41	262.84	368.14	247.43	674.22	1.1465996	0.201776	0.48887189	0.45964847	-1.12139716	down	-	-
MW0151043	His-Phe-Glu-Asp	Amino acids and derivatives	Amino acids and derivatives	C24H30N6O9	2	0.7362	-	12991.79	9118.64	16945.03	7233	2217.32	7787.51	1.1779098	0.0679596	0.29819913	2.265683094	1.179946082	up	-	-
MW0151212	HoPhe-Gly-OH	Others	Lactones	C17H16N2O6	2	0.7225	-	85354.36	78158.02	88369.04	45533.85	26768.16	37697.58	1.52905195	0.00407699	0.11299759	2.289839626	1.195246559	up	-	-
MW0155501	Phe-TyrMe-OH	Amino acids and derivatives	Amino acids and derivatives	C24H22N2O7	2	0.7187	-	16746.45	9405.99	15587.32	2723.69	2588.29	2449.47	1.59332497	0.03802053	0.2307381	5.377830173	2.427024197	up	-	-
MW0139605	Rotenonic acid, methyl ether	Organic acids	Organic acids	C24H26O6	2	0.717	-	46412.69	16297.42	35425.37	14562.38	4666.52	5238.2	1.35898851	0.09440721	0.34919158	4.010915883	2.00393171	up	-	-
MW0152575	Leu-Abu-OH	Amino acids and derivatives	Amino acids and derivatives	C15H20N2O6	2	0.713	-	1289.43	1580.1	2257.01	304.5	729.93	502.21	1.46869242	0.03725202	0.22934484	3.336201062	1.738206238	up	-	-
MW0146333	Asp-Arg	Amino acids and derivatives	Amino acids and derivatives	C10H19N5O5	2	0.711	-	7.38	5.04	7.93	19.77	16.5	20.92	1.56408498	0.00260088	0.09724636	0.355831439	-1.49073411	down	-	-
MW0114671	[(1aS,1bS,5S,5aS,6S)-6-hydroxy-6-(hydroxymethyl)-2-[[(2R,3R,4S,5S,6R)-3,4,5-trihydroxy-6-(hydroxymethyl)oxan-2-yl]oxymethyl]-1b,5,5a,6a-tetrahydro-1aH-oxireno[3,4]cyclopenta[1,3-d]pyran-5-yl] 3-methylbutanoate	Terpenoids	Terpene	C21H32O12	2	0.708	-	152378.61	170493.99	162715.55	956199.06	285066.32	242057.98	1.18446993	0.28689138	0.56536204	0.327364999	-1.61102801	down	-	-
MW0013337	26-Desglucoavenacoside A	Terpenoids	Triterpene Saponin	C45H72O18	3	0.9765	-	14244.67	9737.34	15810.32	4618.41	1106.84	5497.19	1.32069352	0.01618158	0.16485394	3.545782379	1.826103994	up	-	-

MEDN1221	Mannose 6-phosphate	Others	Saccharides	C6H13O9P	3	0.9657	-	63160.03	52404.42	56721.07	155328.34	110158.54	108903.44	1.54859218	0.04287584	0.2434597	0.460176214	-1.11974168	down	C00275	ko00051,ko00520,ko01100,ko01110,ko01240,ko01250
pmp001276	1-Linolenoyl-rac-glycerol-diglucoside	Lipids	Free fatty acids	C33H56O14	3	0.9641	-	1258.23	1304.15	1140.44	253.66	595.32	645.72	1.3926744	0.01643102	0.16551064	2.477299793	1.308768468	up	-	-
MW0011241	4-[(E)-3-[4,5-dihydroxy-6-(hydroxymethyl)-3-[3,4,5-trihydroxy-6-(hydroxymethyl)oxan-2-yl]oxyoxan-2-yl]oxybut-1-enyl]-4-hydroxy-3,5,5-trimethylcyclohex-2-en-1-one	Others	Hydrocarbon derivatives	C25H40O13	3	0.961	-	96725.7	67782.55	79692.37	149994.17	160512.39	181058.71	1.55190847	0.00271976	0.09916999	0.496781679	-1.00931613	down	-	-
MW0114982	N-di-Demethyl roxithromycin	Others	Lactones	C40H74N2O14	3	0.9418	-	117225.52	91143.31	19220.68	148385.45	160686.98	154543.97	1.0588391	0.1131896	0.37822354	0.490900473	-1.02649754	down	-	-
MW0105560	Alanylserylarginine	Amino acids and derivatives	Amino acids and derivatives	C12H24N6O5	3	0.9313	-	57602.67	44357.24	134687.21	31903.93	40527.27	38712.24	1.11597931	0.27521169	0.55571348	2.129204567	1.090314566	up	-	-
MEDN1231	1-O-vanillyl-β-D-glucose	Benzene and substituted derivatives	Benzene and substituted derivatives	C14H18O9	3	0.9285	-	271660.24	226399.32	262902.72	91497.55	147020.77	109432.75	1.52905816	0.00331188	0.10290142	2.186980715	1.128940499	up	C20470	-
MEDN1285	LysoPE(0:0/16:0)	GL	LPE	C21H44NO7P	3	0.9213	-	76237.69	87008.69	86587.29	34847.49	32323.54	56939.49	1.46149755	0.01952913	0.17648958	2.0129935	1.009342514	up	-	-
MW0145312	Arg-Cys-Gly	Amino acids and derivatives	Amino acids and derivatives	C11H22N6O4S1	3	0.9055	-	76963.29	77774.31	73977.8	160263.91	192764.9	151818.19	1.60528712	0.01722282	0.16886016	0.45303904	-1.14229272	down	-	-
ZINC9591030	Dantaxusin A	Others	Lactones	C35H40O10	3	0.8879	-	2268.68	2223.27	2477.35	5278.73	6159.36	5670.83	1.62079782	0.00309295	0.10260237	0.407348915	-1.29566303	down	-	-
MW0012959	1-Palmitoyl-2-docosahexaenoyl-sn-glycero-3-phospho-(1'-rac-glycerol)	GL	PG	C44H75O10P	3	0.8835	-	15497.42	6621.37	9448.44	6822.8	1501.13	895.39	1.24570523	0.08870997	0.33912614	3.424030189	1.775695422	up	-	-
ZINC9591202	(2S)-2alpha-[(E)-2,4,5-Trimethoxystyryl]-3abeta-(4-methoxyphenyl)-6-methoxy-8,9abeta-dihydroxy-2,3,3a,9a-tetrahydro-9H-furo[3,2-b][1]benzopyran-9-one	Others	Ketone compounds	C30H30O10	3	0.8772	-	1617.48	1631.68	742.98	12093.82	13442.67	12473.07	1.5927487	4.408E-05	0.04121132	0.105029892	-3.2511281	down	-	-
MW0054903	Methyl nomilinate 17-glucoside	Others	Lactones	C35H50O16	3	0.8767	-	10075.14	8474.85	6540.82	5479.5	3991.74	1135.51	1.17867397	0.04421746	0.24760945	2.365551182	1.242176376	up	-	-
MW0128256	(5Z)-4-[(E)-3-(4-hydroxy-3-methoxyphenyl)prop-2-enoyl]oxy-2-methyl-5-[2-[(2R,3R,4S,5S,6R)-3,4,5-trihydroxy-6-(hydroxymethyl)oxan-2-yl]oxyethylidene]cyclopentene-1-carboxylic acid	Organic acids	Organic acids	C25H30O12	3	0.8655	-	308.99	351.69	357.38	96.29	129.49	84.05	1.59314631	0.00034558	0.05012965	3.285866443	1.716273842	up	-	-
MW0110066	Trp-Ile-Lys	Amino acids and derivatives	Amino acids and derivatives	C23H35N5O4	3	0.8655	-	48.65	28.95	337.4	1220.07	997.42	758.05	1.39910673	0.00842729	0.14010122	0.139470483	-2.84196827	down	-	-
MW0137160	8-[3,7-dihydroxy-2-(3-hydroxyphenyl)-3,4-dihydro-2H-1-benzopyran-4-yl]-2-(3-hydroxyphenyl)-3,4-dihydro-2H-1-benzopyran-3,5,7-triol	Benzene and substituted derivatives	Benzene and substituted derivatives	C30H26O9	3	0.8593	-	33150.79	28394.24	32726.06	3786.76	1775.4	26643.17	1.18931995	0.11726733	0.38495793	2.927189071	1.549515934	up	-	-
MW0147063	Cannabisin G	Lignans and Coumarins	Lignans	C36H36N2O8	3	0.8567	-	3079.2	566.63	538.28	3634.41	63650.81	15328.39	1.31904343	0.29054248	0.56911419	0.05064674	-4.30338679	down	C17911	-
MW0112443	3,4,5-trihydroxy-6-[(7-hydroxy-2,2-dimethyl-2H-chromen-5-yl)oxy]oxane-2-carboxylic acid	Organic acids	Organic acids	C17H20O9	3	0.8517	-	786.31	388.91	24.17	282.12	8850.04	6279.23	1.04855545	0.20219729	0.48897804	0.077824907	-3.68362423	down	-	-
MW0112439	3,4,5-trihydroxy-6-[(4-methoxy-1-benzofuran-6-yl)oxy]oxane-2-carboxylic acid	Organic acids	Organic acids	C15H16O9	3	0.8314	-	135.31	516.76	426.17	239.95	59.76	90.57	1.08772212	0.17425359	0.46285781	2.762734447	1.466096898	up	-	-
ZINC1471164	(2S)-7-hydroxy-2-(4-hydroxy-2-methoxyphenyl)-5-methoxy-8-[(2S)-5-methyl-2-prop-1-en-2-ylhex-4-enyl]-2,3-dihydrochromen-4-one	Others	Ketone compounds	C27H32O6	3	0.8253	-	3075.1	1189.44	383.85	25780.75	26724.4	21411.64	1.52122838	0.00124934	0.0776351	0.062886795	-3.99109908	down	-	-
MW0130947	3-(1,2-dihydroxybutyl)-7-hydroxy-1H-isochromen-1-one	Others	Ketone compounds	C13H14O5	3	0.813	-	1221.85	347.02	742.71	162.02	259.49	264.34	1.32532625	0.16331234	0.45101702	3.370387111	1.752914304	up	-	-

MW0133939	5,6,7-trihydroxy-2-(4-hydroxyphenyl)-8-(3,4,5-trihydroxyoxan-2-yl)-4H-chromen-4-one	Others	Ketone compounds	C20H18O10	3	0.8071	-	417.64	56.21	39.39	381.52	488.21	560.31	1.18988039	0.11688106	0.3839756	0.358899052	-1.47834998	down	-	-
MW0127643	({2,3-dihydroxy-5-[(2E)-3-(4-hydroxyphenyl)prop-2-enoyl]-4,6-dioxo-3-[3,4,5-trihydroxy-6-(hydroxymethyl)oxan-2-yl]cyclohex-1-en-1-yl}(3,4,5-trihydroxyoxan-2-	Organic acids	Organic acids	C27H32O19S	3	0.8048	-	150008.56	160498.74	166268.15	61509.34	69396.93	31631.77	1.49685365	0.00542112	0.12636259	2.933316102	1.552532548	up	-	-
MW0134075	CID 14730445	Others	Ketone compounds	C28H32O16	3	0.8045	-	3623.29	1789.49	2962.72	9001.78	7031.42	2487.86	1.0106549	0.21634182	0.50316295	0.452214938	-1.14491945	down	-	-
MW0104652	2-amino-4-{[2-({4-[3-(carboxymethyl)-4,6-dihydroxy-2-methoxyphenyl]-3-hydroxy-2-methylbutan-2-yl}sulfanyl)-1-[(carboxymethyl)-C-hydroxycarbonimidoyl]ethyl]-C-hydroxycarbonimidoyl}butanoic acid	Organic acids	Organic acids	C24H35N3O12S	3	0.8042	-	2350.25	4727.16	5690.31	2006.84	1125.54	1886.85	1.32438875	0.11240482	0.37770758	2.543760696	1.346962956	up	-	-
MW0135340	6-[1-(2H-1,3-benzodioxol-5-yl)-3-(4-hydroxy-1-benzofuran-5-yl)-1,3-dioxopropan-2-yl]-3,4,5-trihydroxyoxane-2-carboxylic acid	Organic acids	Organic acids	C24H20O12	3	0.8011	-	14.86	5.85	36.83	50.3	39.53	53.38	1.200308	0.07212781	0.30686128	0.401787585	-1.31549511	down	-	-
MW0134556	5-hydroxy-7-methoxy-2-phenyl-6,8-bis[3,4,5-trihydroxy-6-(hydroxymethyl)oxan-2-yl]-4H-chromen-4-one	Others	Ketone compounds	C28H32O14	3	0.7982	-	9067.7	8519.78	10651.06	5898.03	4599.59	2944.5	1.43056203	0.01183017	0.14824085	2.100750477	1.070904812	up	-	-
MW0152122	2(3H)-Furanone, 3-(((2S,3R)-2,3-dihydro-2-(4-hydroxy-3-methoxyphenyl)-3-(hydroxymethyl)-7-methoxy-5-benzofuranyl)methyl)-4-((3,4-dimethoxyphenyl)methyl)dihydro-, (3R,4R)-	Lignans and Coumarins	Lignans	C31H34O9	3	0.797	-	234322.12	113483.54	182697.78	67880.05	51677.86	70403.08	1.47612131	0.07954301	0.32285533	2.792696753	1.481658926	up	C17685	-
MW0129321	[4-[5,7-Dihydroxy-8-(3-methylbut-2-enyl)-4-oxo-2,3-dihydrochromen-3-yl]-2-hydroxy-6-(3-methylbut-2-enyl)phenyl] hydrogen	Organic acids	Organic acids	C25H28O9S	3	0.7951	-	89179.58	59846.75	90292.08	4624.5	1379.02	17814.38	1.43758077	0.00796521	0.14010122	10.04783839	3.32881326	up	-	-
MW0135343	6-[1-(3,4-dihydroxyphenyl)-3-(4-methoxy-1-benzofuran-5-yl)-1,3-dioxopropan-2-yl]-3,4,5-trihydroxyoxane-2-carboxylic acid	Organic acids	Organic acids	C24H22O12	3	0.7948	-	68.02	17.59	440.56	266.5	652.13	367.68	1.03988794	0.22564012	0.51052525	0.409053805	-1.28963747	down	-	-
MW0148506	Dihydrogeranylgeranyl diphosphate	Organic acids	Organic acids	C20H38O7P2	3	0.7838	-	207.99	11.12	48.38	228.55	346.39	236.06	1.15556332	0.07560375	0.31468277	0.329827374	-1.60021696	down	C17439	-
MW0110207	Tyr-Ile-Lys	Amino acids and derivatives	Amino acids and derivatives	C21H34N4O5	3	0.7822	-	4731.4	8866.49	5566.92	7979.66	10965.32	32560.48	1.0879509	0.29739928	0.57402435	0.372092784	-1.42626568	down	-	-
MW0119085	3-(6,7-dimethoxy-2H-1,3-benzodioxol-5-yl)-2-hydroxypropanal	Benzene and substituted derivatives	Benzene and substituted derivatives	C12H14O6	3	0.7822	-	55.25	19524	32.07	21696.05	19141.93	25942.6	1.16767668	0.12662153	0.40104393	0.293668009	-1.76774198	down	-	-
MW0124292	Hematoxylin	Phenolic acids	Phenolic acids	C16H14O6	3	0.7786	-	231.57	14997.51	2629.53	52595.84	60207.93	67606.05	1.32153063	0.00101841	0.06912669	0.098989124	-3.33658617	down	-	-
MEDN1282	LysoPE(0:0/18:2(9Z,12Z))	GL	LPE	C23H44NO7P	3	0.7722	-	5230.36	5415.27	5227.59	2036.69	842.25	777.09	1.51982794	0.00876152	0.14010122	4.341654746	2.118245005	up	-	-
MW0133510	4-[5-(3-Hydroxypropyl)-1-benzofuran-2-yl]-2-methoxyphenol	Benzene and substituted derivatives	Benzene and substituted derivatives	C18H18O4	3	0.7715	-	4432.87	3645.21	3356.2	482.41	293.53	331.21	1.61598562	0.00715308	0.13569	10.32767014	3.368442923	up	-	-
MW0162756	2-[(3,4-dihydroxy-2-oxo-5-phosphonatooxypentyl)amino]benzoate	Others	Others	C12H13NO9P-3	3	0.7608	-	291405.13	230767.79	500665.43	911533.87	870239.7	805450.28	1.47627536	0.01456062	0.16000982	0.395342038	-1.33882672	down	-	-

ZINC3461983	Petalosa	Others	Saccharides	C10H18O10	3	0.7498	-	2459.71	1349.3	335.37	236.17	461.55	187.46	1.16826908	0.21685197	0.5035531	4.681962991	2.22711353	up	-	-
MW0166427	2-Keto-3,6-dideoxy-6-sulfogluconate	Organic acids	Organic acids	C6H8O8S-2	3	0.7493	-	805.17	1442.63	1220.17	388.5	375.6	110	1.39920434	0.02702616	0.20385331	3.967475117	1.988221175	up	-	-
MW0001492	14-Methoxy-4,4-bisnor-4,8,11,13-podocarpatetraen-3-one	Benzene and substituted derivatives	Benzene and substituted derivatives	C16H18O2	3	0.7476	-	22453.1	15582.23	14251.12	6082.06	918.3	4907.4	1.33342577	0.01629729	0.16485394	4.390955982	2.134535072	up	-	-
MW0137151	8-[1-(2,4-dihydroxyphenyl)-3-(3,4-dihydroxyphenyl)-2-hydroxypropyl]-2-(3-hydroxyphenyl)-3,4-dihydro-2H-1-benzopyran-3,5,7-triol	Benzene and substituted derivatives	Benzene and substituted derivatives	C30H28O10	3	0.747	-	878260.21	297911.12	422465.3	155157.07	176408.79	157576.7	1.40208735	0.17078643	0.45861823	3.268242759	1.708515148	up	-	-
MW0119216	3,5,7-Trihydroxy-2-(1-hydroxy-3-methoxy-4-oxocyclohexyl)-6-methoxychromen-4-one	Others	Ketone compounds	C17H18O9	3	0.7453	-	1512.6	5310.32	3652.53	10214.24	9587.44	8128.53	1.34343762	0.01720996	0.16886016	0.375058046	-1.4148142	down	-	-
Zmhn001793	6-O-Caffeoyl-D-glucose	Phenolic acids	Phenolic acids	C15H18O9	3	0.7449	-	3656960.32	2988076.42	3797952.09	1681003.22	1187492.54	1221375.76	1.57080503	0.00369389	0.10722006	2.553378212	1.352407249	up	-	-
MEDN1248	2,7-Dihydroxy-5-methyl-1-naphthoic acid	Benzene and substituted derivatives	Phenolic acids	C12H10O4	3	0.7437	-	10189.24	68.6	144.13	7841.81	8024.37	10699.04	1.12134677	0.24615584	0.5293161	0.39156348	-1.35268188	down	C20857	ko01100,ko01110
MW0110087	Trp-Val-Asn	Amino acids and derivatives	Amino acids and derivatives	C20H27N5O5	3	0.7423	-	1034.3	1611.13	522.53	348.16	490.13	323.19	1.29739107	0.16408837	0.45219107	2.727520061	1.447589807	up	-	-
ZINC1330102	2-isopropyl-7,8-dimethyl-9,10-dihydrophenanthrene-3-ol	Alcohol and amines	Alcohols	C19H22O	3	0.738	-	69.77	21.85	27.88	69.85	90.85	90.66	1.25384671	0.08242352	0.32686822	0.475413749	-1.07274447	down	-	-
MW0143921	7,8-dihydro-D-neopterin 2',3'-cyclic phosphate	Heterocyclic compounds	Pteridines and derivatives	C9H12N5O6P	3	0.7359	-	72476.46	30613.57	27358.62	25640.54	20338.35	18785.07	1.11949902	0.2695434	0.55056735	2.014216703	1.010218907	up	C20567	ko00790,ko01100
MW0105692	Arginylaspartylarginine	Amino acids and derivatives	Amino acids and derivatives	C16H31N9O6	3	0.7351	-	9499.09	5527.33	4621.94	3932.38	674.88	2126.55	1.21770055	0.08373422	0.32926505	2.917866705	1.544913979	up	-	-
MW0056357	PA(22:6(4Z,7Z,10Z,13Z,16Z,19Z)/15:0)	GL	PA	C40H67O8P	3	0.7335	-	6697.15	2081.46	1885.65	340.38	1246.19	2071.26	1.0487929	0.27272248	0.5537283	2.915460806	1.543723928	up	-	-
MW0106351	D-4-Hydroxy-2-oxoglutarate	Organic acids	Organic acids	C5H6O6	3	0.7325	-	15465.24	2245.01	9736.08	25679.31	22897.35	18526.66	1.14658568	0.05407857	0.27069704	0.409015977	-1.2897709	down	C05946	ko00330,ko01100
MW0128624	[2-hydroxy-6-(3-methylbut-2-en-1-yl)-4-{1-oxo-1-[2,4,6-trihydroxy-3-(3-methylbut-2-en-1-yl)phenyl]propan-2-yl}phenyl]oxidanesulfonic acid	Organic acids	Organic acids	C25H30O9S	3	0.7304	-	578925.66	191370.81	170516.98	89751.07	78642.29	107807.61	1.30610234	0.2368528	0.52019121	3.406264105	1.768190299	up	-	-
MW0003445	3-(3,4-dimethoxyphenyl)-N-[2-(3,4-dimethoxyphenyl)-2-(sulfooxy)ethyl]prop-2-enimidic acid	Organic acids	Organic acids	C21H25NO9S	3	0.7294	-	0.09	0.09	0.09	0.5	0.43	0.09	1.15361033	N/A	N/A	0.264705882	-1.91753784	down	-	-
MEDP1089	Humic acid	Organic acids	Organic acids	C9H9NO6	3	0.7259	-	57112.29	71257.51	73208.67	204305.93	244333.91	191061.05	1.60520585	0.00722607	0.13569	0.315113631	-1.66605593	down	-	-
MW0128919	Sulfuric acid (2Z)-1-methyl-2,3-diphenyl-3-[4-[2-(dimethylamino)ethoxy]phenyl]-2-propenyl ester	Organic acids	Organic acids	C26H29NO5S	3	0.7236	-	99628.69	135752.79	116316.6	2128.92	15119.09	14036	1.47439746	0.00421902	0.11419814	11.24210355	3.490840104	up	-	-
MW0003452	3-(3,4-dimethoxyphenyl)-N-[2-(3,4-dimethoxyphenyl)ethyl]-3-hydroxypropanimidic	Benzene and substituted derivatives	Benzene and substituted derivatives	C21H27NO6	3	0.7233	-	15531.13	10716.47	10187.88	5661.99	4683.37	7317.08	1.45221817	0.04901926	0.25767713	2.062879194	1.044659337	up	-	-
MW0167024	4-O-deacetylvindoline	Alkaloids	Alkaloids	C23H31N2O5+	3	0.7231	-	27876.46	15405.27	24345.9	6758.28	2353.9	12681.97	1.27847173	0.03486486	0.22565754	3.103017553	1.633671858	up	-	-
MW0142898	3'-Deoxydihydrostreptomycin 3'alpha,6-bisphosphate	Organic acids	Organic acids	C21H43N7O17P2	3	0.7189	-	295.62	391.55	50.75	643.15	2872.61	2984.33	1.32901329	0.1256098	0.3989852	0.113524582	-3.13892337	down	C04709	-
MW0141964	2',3,4,4',6'-Pentahydroxychalcone 4'-O-beta-D-glucoside	Others	Ketone compounds	C21H22O11	3	0.7188	-	38.22	45.44	69.9	383.14	371.84	118.74	1.45082781	0.10686565	0.3691444	0.175754246	-2.50836855	down	-	-
MW0149067	Eudesobovatol B	Alcohol and amines	Alcohols	C33H44O4	3	0.7113	-	8020.79	6384.78	8853.57	3203.97	1338.62	2363.31	1.49946557	0.00489931	0.11959415	3.368009962	1.751896406	up	C17677	-
MW0008543	Salicylhydroxamic acid	Benzene and substituted derivatives	Benzene and substituted derivatives	C7H7NO3	3	0.7098	-	63.51	3272.8	84.58	3577.58	3251.87	1950.18	1.11039216	0.23150708	0.51669106	0.389639427	-1.35978843	down	C11343	-
MW0144049	8-Epideoxyloganin	Others	Lactones	C17H26O9	3	0.7091	-	10937.37	6201.99	14283.52	23479.06	27629.53	22487.33	1.42499566	0.0107032	0.14566058	0.426964973	-1.22781037	down	-	-

MW0113633	6-{[2-(3,4-dihydroxyphenyl)-5-hydroxy-3-{[3,4,5-trihydroxy-6-(hydroxymethyl)oxan-2-yl]oxy}-5H-chromen-7-yl]oxy}-3,4,5-trihydroxyoxane-2-carboxylic acid	Organic acids	Organic acids	C27H29O17	3	0.709	-	73905.2	68437.45	99708.17	37982.42	42897.82	36376.23	1.5411459	0.04488155	0.24947897	2.064285408	1.045642451	up	-	-
MW0131269	3-(3-hydroxyphenyl)-2-phenyl-4-[(E)-2-phenylethenyl]-2,3-dihydro-1-benzofuran-6-[illegible]	Benzene and substituted derivatives	Benzene and substituted derivatives	C28H22O3	3	0.7065	-	1928.73	1300.47	1578.26	974.14	712.04	216.34	1.2335197	0.02966925	0.2125012	2.52689065	1.337363234	up	-	-
MW0142518	2-O-caffeoylglucaric [illegible]	Phenolic acids	Phenolic acids	C15H16O11	3	0.7062	-	6437.11	4768.58	5553.11	626.42	819.55	566.35	1.61861443	0.00822296	0.14010122	8.328098911	3.057987203	up	C03062	-
MW0131915	3,4,5-trihydroxy-6-[4-(5,6,7,8-tetrahydroxy-4-oxo-3,4-dihydro-2H-1-benzopyran-2-yl)phenoxy]oxane-2-carboxylic acid	Organic acids	Organic acids	C21H20O13	3	0.7059	-	368.24	494.74	624.76	426.96	138.6	55.93	1.13771098	0.10929326	0.37264441	2.393827737	1.259319338	up	-	-
MW0136861	7,8-dihydroxy-3-(7-hydroxy-5-methoxy-2,2-dimethyl-3,4-dihydro-2H-1-benzopyran-6-yl)-4H-chromen-4-one	Benzene and substituted derivatives	Benzene and substituted derivatives	C21H20O7	3	0.7055	-	10358.67	13606.99	16473.16	632.76	4098.34	2005.94	1.43008311	0.01002636	0.14566058	6.002461021	2.58555413	up	-	-
MW0129997	2-(2,4-Dihydroxyphenyl)-3-[(3E)-3,7-dimethyl-2,6-octadienyl]-5,7-dihydroxy-3,4-dihydro-2H-1-benzopyran-4-one	Others	Others	C25H28O6	3	0.7051	-	3606.33	2923.31	2323.83	12956.54	5099.53	12089.69	1.42429456	0.10091293	0.36015642	0.293688731	-1.76764019	down	-	-
MW0130562	7,3',4'-Trihydroxyethylquerceti[illegible]	Others	Ketone compounds	C21H22O10	3	0.7049	-	640.2	880.75	935.07	2796.07	4367.53	2210.68	1.53966439	0.06720215	0.29786568	0.261995588	-1.93238558	down	-	-
MW0062176	Prieuranin acetate	Terpenoids	Triterpene	C40H52O17	3	0.7041	-	10.58	249.71	18.8	293.09	242.17	367.93	1.18860255	0.10055493	0.36015642	0.30900475	-1.69429908	down	-	-
MW0130990	3-(2,4-dihydroxyphenyl)-1-{3-[5-(2,4-dihydroxyphenyl)-6-(5-hydroxy-2,2-dimethyl-2H-chromene-6-carbonyl)-3-methylcyclohex-2-en-1-yl]-2,4-dihydroxyphenyl}propa[illegible]	Others	Ketone compounds	C40H38O10	3	0.7024	-	10695.55	2388.92	8857.48	20094.38	19215.65	14817.71	1.22015208	0.03011314	0.2125747	0.405373474	-1.30267641	down	-	-
MW0000672	(3E)-4-phenylbuta-1,3-dien-2-ol	Benzene and substituted derivatives	Benzene and substituted derivatives	C10H10O	3	0.7004	-	2836.95	2898.93	2510.92	6370.58	9744	6512.62	1.56762481	0.04745674	0.25471913	0.364464008	-1.45615174	down	-	-
MW0158717	TyrMe-Tyr-OH	Amino acids and derivatives	Amino acids and derivatives	C25H24N2O8	3	0.6981	-	11138.43	10459.2	13802.66	1868.85	3444.89	1179.01	1.53592765	0.00250253	0.09520967	5.452279851	2.446859614	up	-	-
MW0128192	(4-{3-[2,4-dihydroxy-5-(3-methylbut-2-en-1-yl)phenyl]-2-hydroxy-3-oxopropyl}-2-[(3,3-dimethyloxiran-2-yl)methyl]phenyl)oxidanesulfonic acid	Organic acids	Organic acids	C25H30O9S	3	0.6978	-	14135.81	20965.26	8135.68	5771.3	10347.26	3962.54	1.14754971	0.16141246	0.44827043	2.153106653	1.106419784	up	-	-
MW0168269	Podocarpa-8,11,13-trien-16-oic acid, 12-[illegible]	Terpenoids	Ditepenoids	C17H22O3	3	0.6975	-	6118.78	9674.86	5641.39	385.82	351.21	271.52	1.6196221	0.03311502	0.22077332	21.25331416	4.409615922	up	-	-
MW0104472	N-Acetylvanilalanine	Amino acids and derivatives	Amino acids and derivatives	C12H15NO5	3	0.693	-	26910.97	25096.35	27475.44	10412.52	9306.64	9089.96	1.62511094	0.00017637	0.04188261	2.75894439	1.464116378	up	-	-
MW0131403	3-(5-hydroxy-2,2-dimethyl-2H-chromen-6-yl)-1-phenylpropan-1-[illegible]	Others	Ketone compounds	C20H20O3	3	0.6929	-	341.62	152.35	20.16	775.15	403.78	298.68	1.05399455	0.14731871	0.43166641	0.347947023	-1.52306043	down	-	-
MW0114006	[4,5-Diacetyloxy-6-[4-[(ethoxycarbonylamino)methyl]phenoxy]-2-methyloxan-3-yl] acetate	Others	Lactones	C22H29NO10	3	0.6923	-	15219.04	10239.76	3956.46	2080.53	3375.51	2321.21	1.33473377	0.15543824	0.43991021	3.782218651	1.919232768	up	-	-
MW0118191	Ncgc00347814-02_C29H37NO13_	Benzene and substituted derivatives	Benzene and substituted derivatives	C29H37NO13	3	0.6923	-	3926.39	14149.33	4040.59	11915.66	16864.19	56843.59	1.17089213	0.27255016	0.55363319	0.258297377	-1.9528951	down	-	-
MW0131142	3-(3,4-dimethoxyphenyl)-6-hydroxy-7-methoxy-3,4-dihydro-2H-1-benzopyran-4-one	Benzene and substituted derivatives	Benzene and substituted derivatives	C18H18O6	3	0.69	-	50.49	378.94	445.86	730.64	788.44	1044.92	1.15262149	0.02454272	0.19440299	0.341376755	-1.55056327	down	-	-

MW0158701	TyrMe-Gly-OH	Amino acids and derivatives	Amino acids and derivatives	C18H18N2O7	3	0.6893	-	1289.53	1178.64	2101.98	719.93	1024.47	116.54	1.06719139	0.0846155	0.33184319	2.455828775	1.296209977	up	-	-
MW0115014	Niazinin A	Benzene and substituted derivatives	Benzene and substituted derivatives	C15H21NO6S	3	0.6889	-	5325.52	1707.11	7187.12	52382.26	9792.25	12619.94	1.22107876	0.27892051	0.55860069	0.190117716	-2.39503512	down	-	-
MW0143260	4-Benzyloxy-2'-hydroxy-3',4',5',6'-tetramethoxychalcone	Benzene and substituted derivatives	Benzene and substituted derivatives	C26H26O7	3	0.6887	-	634193.88	670074.05	745807.13	256348.04	187230.97	192970.6	1.60514471	0.00059115	0.06185252	3.220605319	1.687331871	up	-	-
MW0109423	Prolylprolyllysine	Amino acids and derivatives	Amino acids and derivatives	C16H28N4O4	3	0.6882	-	723.34	598.24	1741.37	138.27	478.8	134.45	1.32476974	0.158113	0.44404953	4.075673302	2.027038413	up	-	-
MW0140435	(5,12,13-Triaza-indeno[1,2-b]anthracen-13-yl)-acetic acid ethylester	Organic acids	Organic acids	C22H17N3O2	3	0.6878	-	5432.86	6778.81	9764.37	2127.54	219.58	2619.39	1.24090726	0.02794439	0.20581543	4.424845616	2.14562712	up	-	-
MW0138618	Kaempferol 3-[6"-(3-hydroxy-3-methylglutaryl)glucoside]-7-glucoside	Flavonoids	Flavonols	C33H38O20	3	0.6862	-	72249.46	64559.2	71774.03	25035.01	44888.25	22895.06	1.45930876	0.02152769	0.18323322	2.247214666	1.168137945	up	-	-
MW0108507	N,O-Bis-(trimethylsilyl)phenylalanine	Amino acids and derivatives	Amino acids and derivatives	C15H27NO2Si2	3	0.6834	-	68.11	41.62	46.73	109.8	131.48	94.88	1.48960058	0.01279176	0.15141102	0.465433127	-1.1033542	down	-	-
MW0134402	5-{6-[2,4-dihydroxy-3-(3-methylbut-2-en-1-yl)benzoyl]-5-{4-[(E)-2-(2,4-dihydroxyphenyl)ethenyl]-2,6-dihydroxyphenyl}-3-methylcyclohex-3-en-1-yl}benzene-1,2,4-triol	Benzene and substituted derivatives	Benzene and substituted derivatives	C39H38O10	3	0.6833	-	89846.01	106640.76	134772.36	62165.62	44577.17	43928.27	1.50910554	0.02859822	0.20752525	2.198558436	1.136557879	up	-	-
MW0138672	Kaempferol3-rhamninoside	Flavonoids	Flavones	C33H40O19	3	0.6828	-	25139.92	23531.07	19748.99	5740.41	16881.03	9941.88	1.29413125	0.04763424	0.25522731	2.101136493	1.071169885	up	-	-
MW0136468	6-{4-[3-(3,7-dimethylocta-2,6-dien-1-yl)-7-hydroxy-4-oxo-4H-chromen-2-yl]-3-hydroxyphenoxy}-3,4,5-trihydroxyoxane-2-carboxylic acid	Organic acids	Organic acids	C31H34O11	3	0.679	-	793.99	553.8	1054.63	6391.93	2002.17	42740.76	1.29139574	0.33529069	0.59967453	0.046982039	-4.41174685	down	-	-
MW0151224	HoPhe-Trp-OH	Others	Lactones	C26H23N3O6	3	0.6776	-	48.9	33.05	104.61	5.07	7.69	23.89	1.3621467	0.1399256	0.41996717	5.090313779	2.34775459	up	-	-
MW0150099	Glu-Phe-Asp-Glu	Amino acids and derivatives	Amino acids and derivatives	C23H30N4O11	3	0.6776	-	86.22	86.4	95.77	200.98	83.13	285.53	1.06300394	0.22895682	0.51463066	0.471157222	-1.08571954	down	-	-
MW0014970	6-{[1-(2-{[2-(3,4-dimethoxyphenyl)ethyl]-C-hydroxycarbonimidoyl}ethyl)-3-methoxy-4-oxocyclohexa-2,5-dien-1-yl]oxy}-3,4,5-trihydroxyoxane-2-carboxylic acid	Organic acids	Organic acids	C26H33NO12	3	0.6776	-	27266.87	15827.88	20509.34	10650.99	7814.27	12924.6	1.39227406	0.0662321	0.29624338	2.026262303	1.018820945	up	-	-
MW0131355	3-(4-Hydroxyphenyl)-2-sulfooxypropanoic acid	Organic acids	Organic acids	C9H10O7S	3	0.6775	-	601.41	519.29	391.98	64.48	61.44	210.82	1.45618928	0.00835891	0.14010122	4.492130427	2.167399816	up	-	-
MW0155131	Phe-Asp-Ile-Glu	Amino acids and derivatives	Amino acids and derivatives	C24H34N4O9	3	0.6764	-	4950.45	6919	5347.87	1259.51	1453.66	512.42	1.52215679	0.00692367	0.13456926	5.337727362	2.416225618	up	-	-
MW0128569	[2-(acetyloxy)-2-(9-{[(2E)-2-methylbut-2-enoyl]oxy}-2-oxo-2H,8H,9H-furo[2,3-h]chromen-8-yl)propoxy]sulfonic acid	Organic acids	Organic acids	C21H22O11S	3	0.6763	-	9934.64	4269.94	8273.96	42.66	112.72	80.35	1.61017923	0.04774673	0.25522731	95.35714589	6.57526915	up	-	-
MW0156737	Ser-Glu-Lys-Arg	Amino acids and derivatives	Amino acids and derivatives	C20H38N8O8	3	0.6751	-	41321.8	41425.93	33315.5	60575.05	74479.73	126573.83	1.39908039	0.13465055	0.41087189	0.443618265	-1.17260933	down	-	-
MW0158512	Tyr-Leu-Ala-Lys	Amino acids and derivatives	Amino acids and derivatives	C24H39N5O6	3	0.674	-	680.14	345.68	1042.74	357.52	32.86	211.1	1.13522454	0.12030301	0.39061195	3.439116845	1.782038133	up	-	-
MW0107687	S-Glutathionyl-L-cysteine	Amino acids and derivatives	Amino acids and derivatives	C13H22N4O8S2	3	0.6739	-	23128.68	21413.91	29873.08	7621.51	9171.5	10351.25	1.57781063	0.01865614	0.17470383	2.741488256	1.454959293	up	C05526	ko00270,ko01100
MW0128777	[4,5-dihydroxy-2-({7-oxo-7H-furo[3,2-g]chromen-9-yl}oxy)oxan-3-yl]oxidanesulfonic acid	Organic acids	Organic acids	C16H14O11S	3	0.6733	-	1231.04	548.55	3512.42	3626.71	7230.89	3337.9	1.1660929	0.13358465	0.40928854	0.3727949	-1.42354597	down	-	-

MW0132157	3,4,5-trihydroxy-6-{[5-hydroxy-2-(4-hydroxyphenyl)-7-methoxy-4-oxo-8-[3,4,5-trihydroxy-6-(hydroxymethyl)oxan-2-yl]-4H-chromen-6-yl]oxy}oxane-2-carboxylic acid	Organic acids	Organic acids	C28H30O17	3	0.6716	-	101.54	332.54	453.71	20.96	15.19	33.52	1.506217	0.118114	0.38744904	12.74278743	3.67160899	up	-	-
MW0141217	(1S,4aS,7aS)-7-methyl-1-[(2S,3R,4S,5S,6R)-3,4,5-trihydroxy-6-(hydroxymethyl)tetrahydropyran-2-yl]oxy-1,4a,5,7a-tetrahydrocyclopenta[c]pyran-4-carboxylic acid	Terpenoids	Monoterpenoids	C16H22O9	3	0.6707	-	1729.21	1125.26	1727.73	4917.88	2175.17	4642.79	1.35887724	0.1050276	0.36659951	0.390444996	-1.35680877	down	C11672	ko00902,ko01110
MW0158035	Ethyl 3-aminobenzoate	Benzene and substituted derivatives	Benzene and substituted derivatives	C9H11NO2	3	0.6704	-	30.7	29.88	17.49	84.73	45.61	64.39	1.42435795	0.06055266	0.28552881	0.400914086	-1.31863499	down	C18090	-
MW0150185	Glu-Tyr-Asn-Lys	Amino acids and derivatives	Amino acids and derivatives	C24H36N6O9	3	0.6695	-	1493.09	750.76	1519.06	2608.53	2407.78	2672.96	1.39299251	0.02626345	0.20047466	0.489371553	-1.03099785	down	-	-
MW0123749	CID 317610	Others	Ketone compounds	C20H28O6	3	0.6679	-	103.14	139.03	140.44	270.26	118.65	430.34	1.03556054	0.24581785	0.5293161	0.467024718	-1.09842919	down	-	-
MW0158716	TyrMe-TyrMe-OH	Amino acids and derivatives	Amino acids and derivatives	C26H26N2O8	3	0.6668	-	1540.06	2264.08	4564.82	3159	4661.86	9011.71	1.01950052	0.24878888	0.53242342	0.497188486	-1.00813521	down	-	-
MW0150921	His-Glu-Ile-Glu	Amino acids and derivatives	Amino acids and derivatives	C22H34N6O9	3	0.6659	-	27.03	1597.39	172.99	1577.17	1141.75	1686.75	1.05098438	0.21860498	0.50443785	0.407976539	-1.2934419	down	-	-
MW0157916	Thr-TyrMe-OH	Amino acids and derivatives	Amino acids and derivatives	C20H22N2O8	3	0.6653	-	839.08	1305.9	1580.05	9985.99	4895.07	9286.88	1.55597056	0.04824583	0.25598818	0.154131051	-2.69777056	down	-	-
MW0148729	dTDP-3-O-methyl-4-oxo-2,6-dideoxy-L-[illegible]	Nucleotides and derivatives	Nucleotides and derivatives	C17H26N2O14P2	3	0.665	-	149.69	101.61	115.83	123.86	22.24	16.3	1.12712857	0.18003879	0.46795959	2.260652709	1.176739376	up	C21071	ko01100,ko01110,ko01250
MW0135830	6-{[2-(3,4-dihydroxyphenyl)-7-methoxy-4-oxo-8-[3,4,5-trihydroxy-6-(hydroxymethyl)oxan-2-yl]-4H-chromen-5-yl]oxy}-3,4,5-trihydroxyoxane-2-carboxylic acid	Organic acids	Organic acids	C28H30O17	3	0.6648	-	10625.37	10857.9	16047.86	1541.79	7943.92	5590.09	1.19029856	0.0439531	0.24734834	2.489495085	1.315853167	up	-	-
MW0055631	PA(16:0/22:6(4Z,7Z,10Z,13Z,16Z,19Z))	GL	PA	C41H69O8P	3	0.6646	-	5308.85	390.87	811.16	10933.76	10943.44	4756.38	1.24677371	0.06508351	0.29360781	0.244461315	-2.03232191	down	-	-
MW0158533	Tyr-Lys-Arg-Glu	Amino acids and derivatives	Amino acids and derivatives	C26H42N8O8	3	0.6637	-	6687.46	5469.37	6966.8	15953.87	22398.87	17300.83	1.58912763	0.02044294	0.18038854	0.343619107	-1.54111784	down	-	-
MEDL01996	3-Methylpentyl glucosinolate	Others	Glucosinolates	C13H25NO9S2	3	0.6636	-	22107.4	25012.42	26683.71	3360.88	225.95	9843.37	1.22163061	0.00891234	0.14144336	5.495341097	2.458209032	up	-	-
MW0145908	Asn-Thr-Leu-Lys	Amino acids and derivatives	Amino acids and derivatives	C20H38N6O7	3	0.6635	-	2330.86	6046.17	2302.61	6486.79	11814.45	9325.27	1.33241553	0.04856139	0.25720632	0.386572173	-1.3711903	down	-	-
MW0127979	(2-hydroxy-1,2-diphenylethoxy)sulfonic acid	Organic acids	Organic acids	C14H14O5S	3	0.6618	-	33755.47	35409.09	39466.23	2383.8	15483.45	15001.5	1.25209127	0.01704866	0.16827443	3.304986956	1.724644578	up	-	-
MW0109195	4-Hydroxyphenylacetylglutamate	Amino acids and derivatives	Amino acids and derivatives	C13H15NO6	3	0.6588	-	87.17	44.61	75.11	64.19	277.94	219.68	1.07292448	0.20123703	0.48887189	0.368256172	-1.44121839	down	C05595	ko00350
MW0111404	(2E)-3-[4-({2-O-[(2S,3R,4R)-3,4-Dihydroxy-4-(hydroxymethyl)tetrahydro-2-furanyl]-beta-D-glucopyranosyl}oxy)-3-methoxyphenyl]acrylic acid	Organic acids	Organic acids	C21H28O13	3	0.6578	-	19873	14122.34	17950.3	1311.28	370.46	520.68	1.58902992	0.00868907	0.14010122	23.58571026	4.559841142	up	-	-
MEDL02558	Salvianolic acid G	Phenolic acids	Phenolic acids	C18H12O7	3	0.6571	-	1892.91	1656.45	1506.46	4776.29	3296.44	2724.33	1.47100243	0.08411511	0.33017426	0.468258952	-1.09462152	down	-	-
MW0112623	3,4,5-Trihydroxy-6-[4-hydroxy-5-(3-hydroxyphenyl)pentanoyl]oxyoxane-2-carboxylic acid	Organic acids	Organic acids	C17H22O10	3	0.6571	-	3890.75	10826.36	6502.75	14417.09	16556.55	12209.35	1.28434161	0.04709502	0.25471913	0.491393949	-1.025048	down	-	-
MW0130999	3-(2,4-Dihydroxyphenyl)-5-hydroxy-6-(2-hydroxy-3-methylbut-3-enyl)-8,8-dimethylpyrano[2,3-h]chromen-4-one	Others	Ketone compounds	C25H24O7	3	0.6569	-	2410.91	2285.27	2910.66	721.46	683.23	335.13	1.54686784	0.00197175	0.08476296	4.372199423	2.128359206	up	-	-

MW0158449	Tyr-Glu-Arg-Arg	Amino acids and derivatives	Amino acids and derivatives	C26H42N10O8	3	0.6568	-	35138.24	23897.26	26687.69	7645.38	4931.85	4978.21	1.59054889	0.01626382	0.16485394	4.88299866	2.287767382	up	-	-
MW0052784	(1R,4R,5R,7R,8R,11R,17S,18S)-4,5,7,8,17-pentahydroxy-14,18-dimethyl-6-methylidene-3,10-dioxapentacyclo[9.8.0.01,7.04,19.013,18]nonadec-14-ene-9,16-dione	Others	Ketone compounds	C20H24O9	3	0.6568	-	4879.69	7502.95	5596.77	3113.74	2777.6	2205.74	1.51166711	0.0406677	0.23740616	2.220480717	1.150872042	up	-	-
MW0157627	Thr-Glu-Gln-Asp	Amino acids and derivatives	Amino acids and derivatives	C18H29N5O11	3	0.6547	-	32885.61	65211.82	41360.26	130143.64	133883.83	133473.78	1.5304452	0.0114978	0.14569982	0.350835853	-1.51113191	down	-	-
MW0146315	Asp-Val-Asn-His-Phe	Amino acids and derivatives	Amino acids and derivatives	C28H38N8O9	3	0.6542	-	193.35	799.44	825.27	1317.87	1190.28	1271.81	1.12580756	0.08246527	0.32686822	0.480973344	-1.05597116	down	-	-
MW0012966	1-Palmitoyl-2-linoleoyl-sn-glycero-3-phosphate	GL	PA	C37H69O8P	3	0.6542	-	10399.45	4779.09	8005.28	2435.38	5892.11	2903.02	1.18750844	0.12169438	0.39305682	2.06436039	1.045694854	up	C00416	ko00561,ko00564,ko01100,ko01110,ko04070
MW0154214	N-Acetyl-alpha-D-glucosamine 1,6-bisphosphate	Organic acids	Organic acids	C8H17NO12P2	3	0.6538	-	90789.95	74197.73	77665.06	22937.44	27490.75	15165.78	1.57223434	0.00109829	0.07341887	3.699314739	1.887258051	up	C04461	-
MW0109856	Thr-Asn	Amino acids and derivatives	Amino acids and derivatives	C8H15N3O5	3	0.6538	-	32167.52	29542.27	40758.02	15475.09	6752.12	22399.72	1.2850444	0.03045637	0.21312989	2.296098118	1.199184293	up	-	-
MW0013765	3,4,5-trihydroxy-6-[(2Z)-3-oxo-2-(phenylmethylidene)propoxy]oxane-2-carboxylic acid	Organic acids	Organic acids	C16H18O8	3	0.6516	-	1727597.43	1773131.88	1850038.79	855678.11	487985.62	914590.76	1.49244574	0.01192781	0.14824085	2.36942653	1.244537927	up	-	-
MEDL02723	Resveratrol-3-O-sulfate	Others	Stilbene	C14H12O6S	3	0.6498	-	12196.29	19565.36	22949.81	82078.26	25582.23	29030.26	1.13403914	0.27200497	0.553035	0.400257223	-1.32100066	down	-	-
MW0149650	Gln-Asp-Leu-Glu	Amino acids and derivatives	Amino acids and derivatives	C20H33N5O10	3	0.6489	-	1862.31	2447.19	1216.77	1356.93	395.57	519.68	1.24247075	0.08228874	0.32678482	2.43214446	1.282228922	up	-	-
MW0010817	[(2E)-2,11-dimethyl-7-methylidene-6,12-dioxo-5,14-dioxatricyclo[9.2.1.04,8]tetradeca-1(13),2-dien-9-yl] (E)-4-acetyloxy-2-methylbut-2-enoate	Others	Lactones	C22H24O8	3	0.6488	-	3230.12	5748.36	3872.47	24877.62	40747.14	20850.27	1.57325766	0.05411487	0.27069704	0.148608795	-2.7504086	down	-	-
MW0113593	6-{[(16R)-5,11-dihydroxy-8,8,12,16-tetramethyl-3-[1-(2-methyl-1,3-thiazol-4-yl)prop-1-en-2-yl]-10-methylidene-9-oxo-17-oxa-4-azabicyclo[14.1.0]heptadec-4-en-7-yl]oxy}-3,4,5-trihydroxyoxane-2-carboxylic acid	Benzene and substituted derivatives	Benzene and substituted derivatives	C33H48N2O11S	3	0.6484	-	1289.4	1518.01	1066.98	280.17	261.07	519.49	1.53653411	0.00620188	0.13374656	3.652569457	1.868911707	up	-	-
MW0112592	3,4,5-Trihydroxy-6-[3-(3-hydroxyphenyl)propanoyloxy]oxane-2-carboxylic acid	Organic acids	Organic acids	C15H18O9	3	0.648	-	48128.16	45846.26	50749.36	16964.26	22589	16790.66	1.59685139	0.00038089	0.05026788	2.568578473	1.36097015	up	-	-
MW0128595	[2,6-dihydroxy-4-(3,5,7-trihydroxy-3,4-dihydro-2H-1-benzopyran-2-yl)phenyl]oxidanesulfonic acid	Organic acids	Organic acids	C15H14O10S	3	0.6475	-	20109.01	14139.89	18474.23	56422.88	50265.08	21139.61	1.27215207	0.14472234	0.42757737	0.412455075	-1.27769111	down	-	-
MW0148714	dTDP-3-acetamido-3,6-dideoxy-alpha-D-galactopyranose	Nucleotides and derivatives	Nucleotides and derivatives	C18H29N3O15P2	3	0.6437	-	13.01	185.84	68.85	14.76	15.61	7.39	1.12117652	0.27104048	0.55153518	7.089512712	2.825686469	up	C20156	ko01100,ko01110,ko01250
MW0104278	1-Hydroxyoct-6-enoylglycine	Amino acids and derivatives	Amino acids and derivatives	C10H17NO4	3	0.6432	-	56.87	166.77	25.73	216.63	236.26	237.07	1.25376542	0.07205058	0.30686128	0.361426749	-1.46822481	down	-	-
MW0159462	Zizybeoside II	Benzene and substituted derivatives	Benzene and substituted derivatives	C25H38O16	3	0.6426	-	313462.18	321422.61	471261.05	12397.98	3165.08	7146.93	1.59904625	0.01937544	0.17648958	48.70745606	5.60607073	up	C17565	-
MW0131703	3,4,5-trihydroxy-6-[(16-hydroxy-3-methyl-1,7-dioxo-3,4,5,6,7,8,9,10-octahydro-1H-2-benzoxacyclotetradecin-14-yl)oxy]oxane-2-carboxylic acid	Organic acids	Organic acids	C24H30O11	3	0.6422	-	12951.67	6730.35	6598.79	14416.3	17701.24	35223.17	1.29960437	0.15893517	0.4444937	0.390266304	-1.35746919	down	-	-
MW0158704	TyrMe-Ile-OH	Amino acids and derivatives	Amino acids and derivatives	C22H26N2O7	3	0.64	-	75595.12	67944.33	52610.6	21901.93	26106.68	33098.62	1.53480371	0.01593416	0.16425584	2.418404007	1.274055274	up	-	-

MW0148753	dTDP-4-keto-2-deoxy-beta-L-xylose	Nucleotides and derivatives	Nucleotides and derivatives	C15H22N2O14P2	3	0.6376	-	39746.33	22332.79	32357.45	79617.14	72520.96	49992.83	1.41827862	0.03639574	0.22648518	0.467204945	-1.09787255	down	C21353	ko01110
MW0158678	Tyr-Abu-OH	Amino acids and derivatives	Amino acids and derivatives	C19H20N2O7	3	0.6372	-	36281.51	32752.33	31082.05	74385.35	73717.68	65165.91	1.61183161	0.00149967	0.08134154	0.46943493	-1.0910029	down	-	-
MW0132939	3-[4-hydroxy-3-(3-methylbut-2-en-1-yl)phenyl]-1-(2,4,6-trihydroxyphenyl)propan-1-one	Others	Ketone compounds	C20H22O5	3	0.6359	-	29073.34	16573.47	34758.87	3111.92	6429.23	4050.46	1.54234336	0.04938112	0.25906006	5.915831899	2.564581058	up	-	-
MW0112429	3,4,5-trihydroxy-6-[(3-phenyloxiran-2-yl)methoxy]oxane-2-carboxylic acid	Organic acids	Organic acids	C15H18O8	3	0.6353	-	17596.31	62715.75	37617.41	5150.91	14616.72	13911.05	1.26563745	0.1585116	0.44441596	3.501606061	1.808016786	up	-	-
MW0013634	2-Phenylethyl 3-O-(4-carboxy-3-hydroxy-3-methylbutanoyl)-beta-D-glucopyranoside	Benzene and substituted derivatives	Benzene and substituted derivatives	C20H28O10	3	0.6346	-	6789.2	18308.01	11708.94	3799.49	6873.13	412.13	1.09618814	0.10674955	0.3691444	3.320431223	1.731370616	up	-	-
MW0113635	6-{[2-(3,4-dihydroxyphenyl)-7-hydroxy-3-{[3,4,5-trihydroxy-6-(hydroxymethyl)oxan-2-yl]oxy}-8aH-chromen-5-yl]oxy}-3,4,5-trihydroxyoxane-2-carboxylic acid	Organic acids	Organic acids	C27H29O17	3	0.6336	-	31453.75	17399.6	27168.06	1247.4	2048.27	15021.98	1.28378246	0.03466382	0.22565754	4.150172648	2.053171354	up	-	-
MW0132951	3-[5,7-dihydroxy-2-(4-hydroxy-3-methoxyphenyl)-4-oxo-3,4-dihydro-2H-1-benzopyran-8-yl]-5,7-dihydroxy-2-phenyl-3,4-dihydro-2H-1-benzopyran-4-one	Benzene and substituted derivatives	Benzene and substituted derivatives	C31H24O10	3	0.6322	-	92251.22	92326.87	87463.86	286341.78	187369.19	142004.02	1.44413448	0.11478438	0.38027413	0.441830968	-1.17843355	down	-	-
MW0158011	trans-Zeatin riboside triphosphate	Nucleotides and derivatives	Nucleotides and derivatives	C15H24N5O14P3	3	0.6294	-	26.09	532.43	1533.03	4776.89	3798.17	975.93	1.05620565	0.14911188	0.43451026	0.21898777	-2.1910778	down	-	-
MW0143534	12-Dehydrotetracycline	Benzene and substituted derivatives	Benzene and substituted derivatives	C22H22N2O8	3	0.6278	-	13572.1	4115.39	6099.57	2393.48	3325.88	3007.91	1.25837965	0.22251716	0.50639868	2.725601477	1.446574634	up	C03206	ko01100,ko01110
MW0112629	3,4,5-Trihydroxy-6-[4-oxo-1-(5,6,7-trimethoxy-4-oxochromen-2-yl)cyclohexa-2,5-dien-1-yl]oxyoxane-2-carboxylic acid	Organic acids	Organic acids	C24H24O13	3	0.6271	-	351605.72	324842.22	374255.86	46970.15	67010.66	93496.11	1.58636508	0.00014132	0.04121132	5.064196056	2.340333259	up	-	-
MW0110081	Trp-Ser-Lys	Amino acids and derivatives	Amino acids and derivatives	C20H29N5O5	3	0.6268	-	432786.59	309567.08	391847.07	177316.79	112151.8	242337.84	1.39095643	0.01840273	0.17394261	2.13273228	1.092702877	up	-	-
MW0150880	His-Asp-Tyr-Lys	Amino acids and derivatives	Amino acids and derivatives	C25H35N7O8	3	0.6258	-	83103.82	88896.68	94502.73	27517.74	35784.18	69068.11	1.33120509	0.06389981	0.29035184	2.013320009	1.009576501	up	-	-
MW0105462	Ala-Glu-Arg	Amino acids and derivatives	Amino acids and derivatives	C14H26N6O6	3	0.6256	-	25828.37	14995.13	9676.66	1935.03	4081.49	6497.34	1.37893177	0.10768637	0.37038694	4.035538195	2.01276109	up	-	-
MW0006824	Difenacoum	Benzene and substituted derivatives	Benzene and substituted derivatives	C31H24O3	3	0.6255	-	20514.61	24205.83	29499.02	415.02	2883.56	1898.4	1.51138852	0.00870551	0.14010122	14.2812672	3.836052092	up	C16807	-
MW0158606	Tyr-Ser-His-Glu	Amino acids and derivatives	Amino acids and derivatives	C23H30N6O9	3	0.6245	-	113540.41	91721.4	103082.51	55219.35	30715.48	38755.7	1.52152631	0.00326637	0.10290142	2.472876809	1.306190371	up	-	-
ZINC8604739	Terrestrin F	Benzene and substituted derivatives	Benzene and substituted derivatives	C36H26O10	3	0.6238	-	1168.72	247.8	781.15	113.76	72.8	386.08	1.17700348	0.16974633	0.45845338	3.837786393	1.940274415	up	-	-
MW0112507	3,4,5-trihydroxy-6-[3-hydroxy-2-methoxy-4-(prop-2-en-1-yl)phenoxy]oxane-2-carboxylic acid	Organic acids	Organic acids	C16H20O9	3	0.6234	-	120630.21	118833.99	270848.06	82233.23	65184.4	87667.69	1.26691672	0.2084234	0.49545476	2.17075341	1.11819585	up	-	-
MW0128609	[2-hydroxy-3-(methoxymethyl)-6-(3,5,7-trihydroxy-3,4-dihydro-2H-1-benzopyran-2-yl)phenyl]oxidanesulfonic acid	Organic acids	Organic acids	C17H18O10S	3	0.6233	-	710.96	1239.64	317.96	843.4	2785.38	2072.46	1.08637796	0.17043169	0.45861823	0.39790642	-1.32949892	down	-	-
MW0000264	CID 10044441	Alkaloids	Alkaloids	C23H30N2O2	3	0.621	-	84.37	222.59	296.78	42.41	59.22	39.13	1.40422945	0.12997635	0.40526903	4.289144643	2.100689969	up	C20438	ko01100,ko01110

MW0136054	6-{[6-(5,7-dihydroxy-4-oxo-3,4-dihydro-2H-1-benzopyran-2-yl)-2-[2-(3,3-dimethyloxiran-2-yl)ethyl]-2-methyl-2H-chromen-7-yl]oxy}-3,4,5-trihydroxyoxane-2-carboxylic acid	Organic acids	Organic acids	C31H34O13	3	0.621	-	2107.72	4324.06	9839.38	15335.06	10831.78	10606.7	1.19509376	0.07807596	0.32039627	0.442469232	-1.17635096	down	-	-
MW0151222	HoPhe-Ser-OH	Others	Lactones	C18H18N2O7	3	0.6201	-	5517.84	4475.79	7296.31	1351.48	3994.03	753.25	1.27105123	0.04657963	0.25471913	2.834992687	1.503345014	up	-	-
MW0107248	His-Asp-Lys	Amino acids and derivatives	Amino acids and derivatives	C16H26N6O6	3	0.6187	-	126.6	364.34	234.55	783.86	841.59	937.21	1.48141567	0.00306703	0.10260237	0.283100372	-1.82061445	down	-	-
MW0109885	Thr-Lys-Tyr	Amino acids and derivatives	Amino acids and derivatives	C19H30N4O6	3	0.6161	-	3257.63	8505.41	19265.32	3101.21	2496.87	3610.64	1.11865727	0.26230864	0.54282633	3.36945417	1.752514903	up	-	-
MW0132453	3,4,5-trihydroxy-6-{3-hydroxy-5-[(E)-2-(4-{[3,4,5-trihydroxy-6-(hydroxymethyl)oxan-2-yl]oxy}phenyl)ethenyl]phenoxy}oxane-2-carboxylic acid	Organic acids	Organic acids	C26H30O14	3	0.6111	-	402513.23	412588.19	433667.2	162623.66	256078.55	205139.5	1.52939612	0.01009029	0.14566058	2.001739544	1.00125427	up	-	-
MW0104543	2-amino-4-({1-[(carboxymethyl)-C-hydroxycarbonimidoyl]-2-[(2-{3,5-dihydroxy-4-[(1E)-3-methylbut-1-en-1-yl]phenyl}-1-(3,4-dihydroxyphenyl)-2-hydroxyethyl)sulfanyl]ethyl}-C-hydroxycarbonimidoyl)butanoic acid	Organic acids	Organic acids	C29H37N3O11S	3	0.611	-	227.23	61.55	293.83	31.2	73.23	90.29	1.05998073	0.19662925	0.48330265	2.992039852	1.581129391	up	-	-
MW0054718	4-(1,3-Butadienyl)-3,5,5-trimethylcyclohex-2-en-1-one	Others	Aldehyde compounds	C13H18O	3	0.6107	-	18375.28	36007.74	21929.38	48393.45	100661.97	35377.8	1.21585253	0.20710816	0.49365814	0.413767108	-1.27310913	down	-	-
MW0144599	Ala-His-Ser	Amino acids and derivatives	Amino acids and derivatives	C12H19N5O5	3	0.6104	-	9150	1863.89	670.5	31600.34	94995.53	36559.4	1.43459115	0.12899357	0.40449157	0.071615155	-3.80359126	down	-	-
MW0104571	2-amino-4-({1-[(carboxymethyl)-C-hydroxycarbonimidoyl]-2-{[1-(2,5-dihydroxyphenyl)-3-oxopropyl]sulfanyl}ethyl}-C-hydroxycarbonimidoyl)butanoic acid	Organic acids	Organic acids	C19H25N3O9S	3	0.6097	-	35774.57	37065.6	51966.02	20334.86	17929.96	13638.83	1.52551624	0.03046731	0.21312989	2.404574437	1.265781588	up	-	-
MW0002559	2-[4,6-dihydroxy-2-methoxy-3-(3-methylbut-2-en-1-yl)phenyl]acetic acid	Benzene and substituted derivatives	Benzene and substituted derivatives	C14H18O5	3	0.6065	-	96.47	22.87	60.14	22.34	13.41	36.12	1.03175257	0.22809798	0.51368342	2.497286768	1.320361498	up	-	-
MEDN1269	LysoPE(0:0/22:6(4Z,7Z,10Z,13Z,16Z,19Z))	GL	LPE	C27H44NO7P	3	0.6053	-	23552.08	15803.99	9993.7	4682.77	4504.79	8978.15	1.35457227	0.10440826	0.36594594	2.716644161	1.441825607	up	-	-
MW0113604	6-{[(1S,16R)-5,11-dihydroxy-8,8,10,12,16-pentamethyl-3-[1-(2-methyl-1,3-thiazol-4-yl)prop-1-en-2-yl]-9-oxo-17-oxa-4-azabicyclo[14.1.0]heptadec-4-en-7-yl]oxy}-3,4,5-trihydroxyoxane-2-carboxylic acid	Benzene and substituted derivatives	Benzene and substituted derivatives	C33H50N2O11S	3	0.603	-	137.71	289.7	110.64	57.39	83.43	11.25	1.19774703	0.1349282	0.41090462	3.53817321	1.823004676	up	-	-
MW0104619	2-amino-4-({1-[(carboxymethyl)-C-hydroxycarbonimidoyl]-2-{[2-hydroxy-1-(7-methoxy-2-oxo-2H-chromen-6-yl)-3-oxobutyl]sulfanyl}ethyl}-C-hydroxycarbonimidoyl)butanoic acid	Organic acids	Organic acids	C24H29N3O11S	3	0.6019	-	47.23	949.69	261.73	1421.62	1679.38	1423.86	1.19818588	0.04667083	0.25471913	0.278163302	-1.845996	down	-	-
MW0156492	S-Adenosylhomocysteine-[illegible]	Nucleotides and derivatives	Nucleotides and derivatives	C14H16D4N6O5S	3	0.6012	-	48878.34	23628.5	44520.65	3692.72	271.23	19068.25	1.1806653	0.03593428	0.22604178	5.081038286	2.345123335	up	-	-

MW0145800	Asn-Ile-Tyr	Amino acids and derivatives	Amino acids and derivatives	C19H28N4O6	3	0.6012	-	703.33	873.21	1691.97	4476.95	5059.18	3935.56	1.53169672	0.00160397	0.08134154	0.242620636	-2.04322583	down	-	-
MW0109906	Thr-Val-Arg	Amino acids and derivatives	Amino acids and derivatives	C15H30N6O5	3	0.6007	-	4671.61	3205	6812.75	14183.02	11972.33	16772.82	1.50066999	0.00691512	0.13456926	0.342184631	-1.54715313	down	-	-
MW0113343	6-(3-carboxy-5-methoxyphenoxy)-3,4,5-trihydroxyoxane-2-carboxylic acid	Organic acids	Organic acids	C14H16O10	3	0.5999	-	886.08	517.29	335.51	755.1	1205.67	1610.03	1.18505415	0.11850727	0.38787393	0.486972107	-1.03808896	down	-	-
MW0113657	6-{[3,4-dihydroxy-5-(hydroxymethyl)-2-{[3,4,5-trihydroxy-6-(hydroxymethyl)oxan-2-yl]oxy}oxolan-2-yl]methoxy}-3,4,5-trihydroxyoxane-2-carboxylic acid	Organic acids	Organic acids	C18H30O17	3	0.5987	-	28426.62	3091.31	9222.52	42503.05	31418.74	38213.07	1.19443947	0.07268761	0.30777134	0.363316546	-1.46070103	down	-	-
MW0058138	PE(16:0/22:5(4Z,7Z,10Z,13Z,16Z))	GL	PE	C43H76NO8P	3	0.5981	-	26965.72	19074.36	38820.5	8782.44	9237.29	9517.55	1.52572613	0.07937035	0.32274836	3.08166166	1.623708475	up	-	-
MW0130137	2-(3,4-dihydroxyphenyl)-4-[3,5,7-trihydroxy-2-(4-hydroxy-3-methoxyphenyl)-3,4-dihydro-2H-1-benzopyran-8-yl]-6-[3,4,5-trihydroxy-6-(hydroxymethyl)oxan-2-yl]-3,4-dihydro-2H-1-	Benzene and substituted derivatives	Benzene and substituted derivatives	C37H38O17	3	0.5969	-	239.65	106.5	283.08	27.25	25.49	18.5	1.56010662	0.07241646	0.30691781	8.8325379	3.142828035	up	-	-
MW0058215	1-Vaccenoyl-2-myristoyl-sn-glycero-3-phosphoethanolamine	GL	PE	C37H72NO8P	3	0.5961	-	11261.66	12029.17	11509.58	90129.15	21307.8	18215.2	1.16485228	0.3103139	0.58451078	0.268413675	-1.89746992	down	-	-
MW0113194	5-[(6-carboxy-3,4,5-trihydroxyoxan-2-yl)oxy]-1-oxo-1H-isochromene-3-carboxylic acid	Organic acids	Organic acids	C16H14O11	3	0.5958	-	2356.74	1839.53	242.88	11367.26	7991.02	6108.47	1.33209486	0.0308137	0.21409456	0.174311602	-2.52025949	down	-	-
MW0146363	Asp-Met	Amino acids and derivatives	Amino acids and derivatives	C9H16N2O5S	3	0.5956	-	2229.01	2404.95	3395.4	3270.31	11082.71	7613.8	1.24675621	0.17323229	0.46272517	0.365522183	-1.45196913	down	-	-
MW0135445	6-[3-(2H-1,3-benzodioxol-5-yl)-1-(4-methoxy-1-benzofuran-5-yl)-3-oxopropoxy]-3,4,5-trihydroxyoxane-2-carboxylic acid	Organic acids	Organic acids	C25H24O12	3	0.5872	-	5137.09	4020.27	4803.63	398.51	536.8	986.56	1.57668163	0.0015981	0.08134154	7.264273858	2.860818592	up	-	-
MW0150527	Gly-Val-Ala-Asp-Val	Amino acids and derivatives	Amino acids and derivatives	C19H33N5O8	3	0.586	-	5325.89	19817.34	7534.58	29966.7	33313.39	33515.44	1.37775749	0.03495906	0.22565754	0.337596271	-1.56662913	down	-	-
MW0107762	Leu-Thr-Ser	Amino acids and derivatives	Amino acids and derivatives	C13H25N3O6	3	0.5848	-	21510.47	16192.56	8374.74	23701.43	58445	56103.63	1.31255175	0.09902913	0.35695795	0.333292948	-1.5851373	down	-	-
MW0134536	5-hydroxy-6-(2-hydroxy-2-methylpropanoyl)-8,8-dimethyl-4-phenyl-2H,8H-pyrano[2,3-f]chromen-2-one	Others	Ketone compounds	C24H22O6	3	0.5847	-	2963.32	9392.36	4848.68	14232.9	12121.97	8832.56	1.2135961	0.07470491	0.31230283	0.488934827	-1.03228592	down	-	-
MW0143477	5-(3,4-Diacetoxybut-1-ynyl)-2,2'-bithiophene	Heterocyclic compounds	Heterocyclic compounds	C16H14O4S2	3	0.5777	-	1103.49	1892.68	9473.38	7099.03	20887.72	14942.6	1.20353994	0.11209886	0.3775421	0.290466779	-1.78355493	down	C04606	-
MW0130026	2-(2,4-dihydroxyphenyl)-5,7-dihydroxy-3-{[3-methyl-3-(4-methylpent-3-en-1-yl)oxiran-2-yl]methyl}-6-(3-methylbut-2-en-1-yl)-4H-chromen-4-one	Others	Ketone compounds	C30H34O7	3	0.575	-	358.7	732.53	266.37	2036.02	1007.66	1853.35	1.42412809	0.04778281	0.25522731	0.277229259	-1.85084856	down	-	-
MW0134487	5-hydroxy-2-(4-hydroxy-3-methoxyphenyl)-6,8-bis[3,4,5-trihydroxy-6-(hydroxymethyl)oxan-2-yl]-7-[(3,4,5-trihydroxy-6-methyloxan-2-yl)oxy]-4H-chromen-4-one	Others	Ketone compounds	C34H42O20	3	0.5747	-	1695.62	671.59	559.36	1932.88	2576.77	1899.96	1.2810324	0.0639158	0.29035184	0.456590963	-1.13102579	down	-	-
MW0164217	beta-D-4-deoxy-Delta(4)-GlcpA-(1->3)-beta-D-GalpNAc(1-)	Organic acids	Organic acids	C14H20NO11-	3	0.5674	-	6709.75	6124.16	2784.92	3776.05	1172.53	1364.06	1.18923057	0.11345909	0.37822354	2.474215225	1.306971002	up	-	-

MW0112625	3,4,5-trihydroxy-6-{[4-hydroxy-5-(4-hydroxy-3-methoxyphenyl)pentanoyl]oxy}oxane-2-carboxylic acid	Organic acids	Organic acids	C18H24O11	3	0.5606	-	9.81	31.39	3.65	60.07	26.29	40.46	1.159926	0.10330757	0.3645986	0.353650844	-1.49960239	down	-	-
MW0130347	CID 131838408	Others	Ketone compounds	C32H24O20	3	0.5592	-	3069.4	433.93	2500.03	6115.8	7160.67	2311.76	1.02013071	0.150246	0.43473176	0.385121338	-1.37661503	down	-	-
MW0149622	Gln-Arg-Asp-Glu	Amino acids and derivatives	Amino acids and derivatives	C20H34N8O10	3	0.5539	-	1608.6	1529.28	1143.34	942.95	474.64	529.89	1.41428308	0.01956106	0.17648958	2.198338365	1.136413461	up	-	-
MW0109043	Paralinone B	Others	Lactones	C39H48O16	3	0.5525	-	32869.82	38068.94	41718.78	13592.84	7221.59	11659.99	1.55674563	0.00159272	0.08134154	3.469116308	1.79456821	up	-	-
MW0148176	Deacetylisoipecoside	Alcohol and [illegible]	Amines	C25H33NO11	3	0.5521	-	104.32	183.2	245.32	276.42	1309.84	1442.15	1.30414243	0.15099246	0.43582171	0.175947114	-2.50678624	down	C07304	ko00950,ko01110
MW0128739	[4-(3,5,7-trihydroxy-3,4-dihydro-2H-1-benzopyran-2-yl)phenyl]oxidanesulfonic acid	Organic acids	Organic acids	C15H14O8S	3	0.5439	-	4630.87	1860.53	3407.21	7880.51	17016.77	4579.96	1.20479334	0.2177449	0.50443785	0.335805184	-1.57430359	down	-	-
MW0149953	Glu-Asp-Thr-Glu	Amino acids and derivatives	Amino acids and derivatives	C18H28N4O12	3	0.5437	-	505.55	162.01	296.33	241.18	107.31	69.68	1.0793094	0.20458424	0.4907574	2.30501949	1.204778949	up	-	-
MW0149212	Flavonol 3-O-beta-D-glucosyl-(1->2)-beta-D-glucosyl-(1->2)-beta-D-glucoside	Flavonoids	Flavonols	C33H40O18	3	0.5392	-	98.02	259.92	168.17	20.67	134.54	5.82	1.13921152	0.12280529	0.39433546	3.267155188	1.708034984	up	-	-
MW0103415	6-Methylthioguanosine monophosphate	Nucleotides and derivatives	Nucleotides and derivatives	C11H16N5O7PS	3	0.5356	-	20648.62	14714.03	22179.25	83579.45	40032.41	45883.47	1.46258758	0.10795957	0.37038694	0.339489589	-1.55856076	down	C16620	-
MW0157934	Tiliacorine	Lignans and Coumarins	Lignans	C36H36N2O5	3	0.5326	-	12369.84	12047.53	7693.28	17345.45	52114.72	13963.93	1.10511684	0.2950268	0.5721144	0.384908558	-1.37741235	down	C09667	-
ZINC9591035	(1aR,2R,8aS)-1,1aalpha,2,5,5a,6,10,10aalpha-Octahydro-5beta,5abeta-dihydroxy-4-hydroxymethyl-1,1,7,9alpha-tetramethyl-6beta-[[(2E,4Z)-1-oxodeca-2,4-dienyl]oxy]-9H-2,8aalpha-methanocyclopenta[a]cyclopropa[e]cyclodecen-11-one	Others	Lactones	C30H42O6	3	0.5315	-	291.31	322.07	354.26	90.25	56.71	138.63	1.51062421	0.0020958	0.08777948	3.388213873	1.760524944	up	-	-
MW0132484	3,4,5-trihydroxy-6-{4-[3-(5-hydroxy-2,2-dimethyl-2H-chromen-6-yl)prop-2-enoyl]phenoxy}oxane-2-carboxylic acid	Benzene and substituted derivatives	Benzene and substituted derivatives	C26H26O10	3	0.5221	-	75722.55	45409.77	78124.72	17480.32	5253.01	3673.02	1.48759194	0.01970923	0.17658189	7.545800158	2.915673892	up	-	-
MW0107362	His-Tyr	Amino acids and derivatives	Amino acids and derivatives	C15H18N4O4	3	0.521	-	4483.01	2582.79	5207.98	8242.62	8655.59	8025.32	1.41912395	0.02745196	0.20424629	0.492457529	-1.02192879	down	-	-
MW0129320	{4-[5,7-dihydroxy-3-(3,4,5-trihydroxybenzoyloxy)-3,4-dihydro-2H-1-benzopyran-2-yl]-2,6-dihydroxyphenyl}oxidanesulfonic acid	Organic acids	Organic acids	C22H18O14S	3	0.516	-	1839.08	3347.16	1543.79	1137.77	53.77	53.77	1.28439986	0.06110313	0.28609163	5.404300937	2.434108013	up	-	-

Table S5 The conjoint analysis of transcriptome and metabolome between OH 88119 and *yfm*

Description	0 Days post anthesis				45 DPA				55 DPA			
	P-value_meta	-LOG10(p-value)	P-value_gene	-LOG10(p-value)	P-value_meta	-LOG10(p-value)	P-value_gene	-LOG10(p-value)	P-value_meta	-LOG10(p-value)	P-value_gene	-LOG10(p-value)
2-Oxocarboxylic acid metabolism	0.778138263	0.108943229	0.84639066	0.072429138	0.766824951	0.115303764	0.993920156	0.002648502	0.4358532	0.360659761	0.995363486	0.002018295
ABC transporters	0.798814463	0.097554081	0.817692884	0.087409782	0.938341554	0.027639051	0.66278284	0.178628744	0.986328378	0.005978471	0.302359979	0.519475693
Amino sugar and nucleotide sugar metabolism	0.45760604	0.339508252	0.935009454	0.029183998	0.9440606	0.025000127	0.002375655	2.624216641	0.947440329	0.023448133	0.000867669	3.061646031
Anthocyanin biosynthesis	0.287828947	0.540865531	0.295542977	0.529379356	0.357795894	0.446364647	0.729777853	0.136809321	0.377222818	0.423402045	0.388052559	0.411109448
Arginine and proline metabolism	0.193434694	0.713465629	0.924149346	0.034257839	0.34510101	0.46205377	0.06580649	1.181731275	0.644136152	0.191022326	0.264228573	0.57802022
Ascorbate and aldarate metabolism	0.330224231	0.481191062	0.25963257	0.585640828	0.724010053	0.140255404	0.186211219	0.729994158	0.943499862	0.025258159	0.147063901	0.832493918
Biosynthesis of amino acids	0.960639961	0.017439351	0.791045203	0.101798699	0.824179044	0.083978432	0.764695689	0.116511358	0.976448236	0.010350775	0.748290829	0.125929578
Biosynthesis of cofactors	0.702836419	0.153145743	0.92667301	0.033073486	0.864329201	0.063320814	0.094719962	1.023558485	0.976928743	0.010137112	0.023697977	1.625288724
Biosynthesis of nucleotide sugars	0.154675937	0.810577244	0.972169239	0.012258125	0.86882633	0.061067026	0.250765497	0.600732218	0.509076934	0.29321658	0.055570876	1.25515276
Biosynthesis of secondary metabolites	0.715945111	0.145120272	0.000136117	3.866087631	0.30610557	0.514128768	0.0694186	1.158524149	0.216124565	0.665295867	0.32942441	0.482244223
Biosynthesis of various alkaloids	0.892750349	0.049269971	0.051660393	1.286842294	0.945741657	0.024227481	0.678569073	0.168405938	0.538840403	0.268539848	0.91180135	0.040099769
Biosynthesis of various plant secondary metabolites	0.631334443	0.199740517	0.124414785	0.905128007	0.930909637	0.031092474	0.42617055	0.370416565	0.333921655	0.476355415	0.337491266	0.471737462
Carbon metabolism	0.946726006	0.023775693	0.962056086	0.016799609	0.44526258	0.351383802	0.347871009	0.458581763	0.98332891	0.007301192	0.005746128	2.240624704
Cysteine and methionine metabolism	0.847277745	0.071974201	0.410659888	0.386517716	0.585069846	0.232792284	0.049963471	1.301347397	0.935714271	0.028856747	0.057147662	1.243001535
Flavonoid biosynthesis	0.892750349	0.049269971	0.076280615	1.117585814	0.493947392	0.306319304	0.536205442	0.270668783	0.284408571	0.54605732	0.260097271	0.584864204
Fructose and mannose metabolism	0.29988767	0.52304139	0.919549986	0.036424658	0.789564879	0.102612178	0.471896017	0.326153688	0.370876216	0.430771017	0.743827002	0.12852806
Glycerophospholipid metabolism	0.217170199	0.663199771	0.655998654	0.183097052	0.9440606	0.025000127	0.252266332	0.598140707	0.678798269	0.168259274	0.113764379	0.943993701
Glyoxylate and dicarboxylate metabolism	0.785397349	0.104910569	0.847882336	0.071664412	0.01798692	1.745043195	0.047104456	1.326938006	0.883263046	0.053909939	0.002524153	2.597884314
Isoflavonoid biosynthesis	0.848175176	0.071514442	0.315824714	0.500553888	0.047009319	1.327816041	0.946534787	0.02386342	0.418214951	0.378600446	0.442112625	0.354467083
Isoquinoline alkaloid biosynthesis	0.946726006	0.023775693	0.607474316	0.216472079	0.978244067	0.009552777	0.969931385	0.013258988	0.2765582	0.55821346	0.951663162	0.021516742
Metabolic pathways	0.88927334	0.050964727	0.20710731	0.683804572	0.825411224	0.08332963	0.226372302	0.645176712	0.957383817	0.018913918	0.215726125	0.666097258
Pantothenate and CoA biosynthesis	0.785397349	0.104910569	0.837099631	0.07722285	0.256572518	0.590789863	0.169280978	0.771391839	0.883263046	0.053909939	0.246558503	0.608080015
Phenylalanine metabolism	0.510279392	0.292191971	0.027347208	1.563087006	0.44526258	0.351383802	0.341963567	0.466020162	0.2765582	0.55821346	0.636340253	0.196310604
Phenylalanine, tyrosine and tryptophan biosynthesis	0.422450955	0.374223704	0.072943047	1.137016099	0.83335869	0.079168032	0.431887646	0.364629218	0.391714837	0.407029979	0.440574979	0.355980171
Phenylpropanoid biosynthesis	0.422450955	0.374223704	0.047213199	1.325936572	0.598995003	0.2225768	0.428817211	0.367727792	0.391714837	0.407029979	0.298299105	0.52534805
Porphyrin metabolism	0.493547212	0.306671297	0.009237304	2.034454763	0.177777879	0.750122279	0.00967564	2.0143203	0.196762935	0.706056707	0.062071732	1.207106136
Starch and sucrose metabolism	0.573146864	0.24173408	0.947893403	0.023240499	0.670704614	0.173468706	0.006276933	2.202252501	0.695135584	0.157930479	0.135709814	0.867388744
Terpenoid backbone biosynthesis	0.42183521	0.374857173	0.509485657	0.292868038	0.002676425	2.572444952	0.019669994	1.70619578	0.597212953	0.223870781	0.017388796	1.759730487
Tropane, piperidine and pyridine alkaloid biosynthesi	0.819461262	0.086471572	0.822102838	0.085073853	0.316809866	0.499201302	0.967483291	0.014356527	0.657977101	0.181789221	0.787992645	0.103477836
Tyrosine metabolism	0.962539824	0.016581293	0.617795701	0.209155118	0.233638355	0.631455861	0.985976496	0.006133438	0.73152422	0.13577129	0.999671585	0.000142652
Vitamin B6 metabolism	0.64036783	0.193570494	0.010180715	1.99222172	0.340755864	0.467556661	0.026866806	1.57078396	0.759987458	0.119193575	0.014580021	1.836241838

Table S6 Heatmap of the transcriptional level of genes involved in the terpenoid and carotenoid biosynthesis pathway.

Enzyme		Gene ID	OH88119-0DPA-1	OH88119-0DPA-2	OH88119-0DPA-3	OH88119-45DPA-1	OH88119-45DPA-2	OH88119-45DPA-3	OH88119-55DPA-1	OH88119-55DPA-2	OH88119-55DPA-3	*yfm* -0DPA-1	*yfm* -0DPA-2	*yfm* -0DPA-3	*yfm* -45DPA-1	*yfm* -45DPA-2	*yfm* -45DPA-3	*yfm* -55DPA-1	*yfm* -55DPA-2	*yfm* -55DPA-3
**Abbreviation**	**Full name**																			
AACT	acetyl-CoA acetyltransferase	Solyc05g017760	16.2219	24.387	24.2401	48.6733	45.8998	45.3494	48.3295	47.4214	48.4243	40.8779	35.4936	28.435	60.7439	60.6113	57.9149	56.5469	59.6489	61.1675
HMGS	hydroxymethylglutaryl-CoA synthase	Solyc12g056450	46.2647	47.2411	44.7021	13.2108	9.2424	10.5985	1.4689	1.617	1.3984	68.7233	57.8038	53.8368	23.5756	19.3112	20.8128	2.8716	1.9953	2.2427
HMGR	hydroxymethylglutaryl-CoA reductase	Solyc02g082260	461.4527	240.9458	263.8416	7.8642	5.9149	7.0484	5.2692	4.7181	5.9798	233.8976	240.8387	252.0566	9.9219	8.0186	10.8836	7.9836	8.5918	9.3903
MVK	mevalonate kinase	Solyc01g098840	4.0478	4.0849	4.0648	4.0786	4.0602	4.0657	4.0222	4.0263	4.0374	4.0478	4.0849	4.0648	4.0786	4.0602	4.0657	4.0222	4.0263	4.0374
PMK	phosphomevalonate kinase	Solyc08g076140	2.1109	4.2093	4.5378	7.613	7.2825	7.065	4.694	4.4931	4.6992	3.8633	4.8169	4.2009	8.9593	7.196	7.8029	3.4804	2.102	3.2935
MPDC	mevalonate diphosphate decarboxylase	Solyc04g009650	4.0935	4.0278	4.0378	4.0364	4.0311	4.0842	4.0705	4.0485	4.0866	4.0935	4.0278	4.0378	4.0364	4.0311	4.0842	4.0705	4.0485	4.0866
IDI	isopentenyl pyrophosphate isomerase	Solyc04g056390	151.5686	203.2859	199.5842	135.1757	142.2986	120.8143	233.5531	228.5509	240.3967	201.4534	202.7369	209.8125	147.8516	172.035	177.5134	339.4993	335.1	352.8046
FPPS	farnesyl pyrophosphate synthase	Solyc10g005840	-0.85546	-0.92454	-0.67024	1.3492525	1.2136165	1.4330005	0.1594036	-0.309584	-0.07411	-0.71414	-0.68341	-1.2961	1.475725	1.27554	1.024166	-0.63654	-0.96818	-0.79838
FTA	farnesyltransferase	Solyc06g073390	-0.03785	-0.27044	-0.1558	0.9039591	1.3939648	1.3237251	-1.100294	-0.877968	-1.058707	0.027858	-0.27773	-1.19353	1.247241	1.241534	1.51367	-0.85703	-0.90082	-0.92178
FCLY	farnesylcysteine lyase	Solyc05g010050	1.16596	1.132693	1.186697	-0.218275	-0.18717	-0.122766	-0.948108	-1.009723	-1.041612	0.940736	1.550971	1.695753	-0.32227	-0.29709	-0.35015	-0.99657	-1.0917	-1.08738
PCME	prenylcysteine methylesterase	Solyc12g088020	-0.08666	0.039151	0.482758	0.9984722	0.6521257	0.8921137	-1.081678	-0.869254	-1.315136	0.596098	0.15758	-1.05103	1.399203	1.336937	1.296245	-1.20315	-1.05283	-1.19094
DXS	1-deoxy-D-xylulose 5-phosphate synthase	Solyc01g067890	35.1536	73.6253	64.7299	124.1058	153.699	103.2028	204.5896	240.3515	203.5049	72.1937	76.3916	70.026	86.2601	85.5062	98.4285	178.6737	188.6861	187.6335
DXR	1-deoxy-D-xylulose 5-phosphate reductoisomerase	Solyc03g114340	24.118	75.2415	75.0682	109.227	99.8059	127.9882	57.838	62.6278	58.3039	93.0959	99.851	85.9008	34.638	46.3157	42.3031	19.9762	19.0014	17.749
MCT	2-C-methyl-D-erythritol 4-phosphate cytidylyltransferase	Solyc01g102820	1.8937	1.5634	1.6783	1.0981	0.8357	0.9237	0.516	0.6143	0.5768	0.8372	0.7648	0.9056	0.3782	0.352	0.4015	0.5372	0.6125	0.5973
CMK	4-(cytidine 5′-diphospho)-2-C-methyl-D-erythritol kinase	Solyc01g009010	22.8219	47.8774	47.4548	36.7995	41.0887	30.2133	55.7739	68.0788	66.2221	51.8074	49.5817	49.5445	39.7065	40.0182	39.265	42.0086	44.2178	44.2103
MECPS	2-C-methyl-D-erythritol 2,4-cyclodiphosphate synthase	Solyc08g081570	33.7978	57.732	54.4768	24.1728	24.2756	16.8937	29.6131	28.4771	27.5523	58.0662	58.9409	57.3904	47.5061	47.1343	49.5967	35.487	29.6867	30.5991
HDS	4-hydroxy-3-methylbut-2-enyl diphosphate synthase	Solyc11g069380	62.2625	177.6437	168.8324	136.4252	127.4872	130.1829	143.3679	160.3565	154.4424	217.9681	206.8822	192.6836	101.9544	108.3193	120.4997	110.2652	102.1427	109.1142
HDR	4-hydroxy-3-methylbut-2-enyl diphosphate reductase	Solyc01g109300	4.0478	4.0849	4.0648	4.0786	4.0602	4.0657	4.0222	4.0263	4.0374	4.0478	4.0849	4.0648	4.0786	4.0602	4.0657	4.0222	4.0263	4.0374
GPPS	geranyl pyrophosphate synthase	Solyc09g008920	-1.05131	-1.15823	-0.99019	0.3783387	-0.131162	0.1286903	0.5512489	0.5403344	0.771397	-1.0419	-1.18433	-1.93825	0.526015	0.12274	0.470943	1.559098	1.330093	1.116469
GGPPS	geranylgeranyl pyrophosphate synthase	Solyc11g011240	1.113425	0.657989	-0.54911	-0.564178	-0.535675	-0.620777	1.1582159	2.0135085	1.9896882	0.401258	-0.77978	0.235738	-0.93899	-1.03305	-0.93777	-0.46584	-0.85124	-0.2934
PSY1	phytoene synthase 1	Solyc03g031860	129.5623	489.3227	422.9595	3830.8153	4089.2092	3427.561	6637.5896	7651.4654	7117.6837	292.9388	334.4974	284.021	913.9315	1463.232	1386.797	5160.849	5528.522	5425.491
PSY2	phytoene synthase 2	Solyc02g081330	16.1611	34.6102	34.0874	67.0819	61.9054	50.739	94.6442	116.1886	96.8014	39.0804	39.7509	34.439	12.7959	15.1024	15.798	38.7861	46.7279	45.9017
PDS	phytoene desaturase	Solyc03g123760	45.8518	196.4794	205.4562	39.3752	33.2517	25.1482	60.2639	73.0756	64.6346	165.4945	182.8711	170.7465	64.1788	70.3006	75.6531	82.3341	82.3149	83.7246
Z-ISO	15-cis-zeta-carotene isomerase	Solyc12g098710	72.4319	313.7583	310.8976	398.1522	852.1316	498.9495	2078.4227	2186.2479	2030.1557	206.9657	197.7183	207.6489	77.4565	149.3395	145.9416	2074.498	2339.084	2365.457
ZDS	zeta-carotene desaturase	Solyc01g097810	33.8592	83.1482	71.1819	87.2438	82.687	74.3089	105.8521	120.4452	112.8461	77.6491	75.5117	68.5568	48.2397	55.691	56.6059	86.2427	81.5125	85.4467
CRTISO	prolycopene isomerase	Solyc05g010180	3.239	8.4912	9.4587	30.7264	28.1342	38.7025	17.485	20.5782	18.9199	18.3277	19.7681	17.0653	26.0078	35.467	33.836	33.3154	37.4928	36.3749
LCYE	lycopene epsilon cyclase	Solyc12g008980	4.6003	10.9144	12.516	0.5004	0.306	0.8917	0.001	0.0452	0.0235	14.6827	11.9626	12.9087	2.0163	1.8207	1.7435	0.0462	0.0698	0.06
LCYB	lycopene beta-cyclase	Solyc04g040190	8.8989	19.8986	18.8241	35.4653	31.4472	41.3548	7.5258	7.4825	9.6376	29.8659	31.2409	32.5092	11.8123	9.0286	8.7843	5.2939	4.3709	4.8891
CHYB	beta-carotene hydroxylase	Solyc06g036260	7.238	10.6694	8.6669	71.9118	87.5859	69.785	218.5451	221.1332	222.2326	26.4321	24.0066	22.195	18.8253	21.2325	19.0297	128.4764	156.3358	148.2112
LUT1	carotene epsilon-monooxygenase	Solyc10g083790	6.8214	18.5867	16.2363	14.8556	11.6857	12.5306	5.948	5.9454	6.5209	17.8568	16.1985	15.8771	13.7374	14.2186	15.1333	7.3066	6.4179	5.9191
VDE	violaxanthin de-epoxidase	Solyc04g050930	14.9089	25.7616	26.2895	19.8118	19.6619	17.1707	14.0319	17.6441	16.4667	31.8013	30.1588	30.7924	56.4015	74.5902	67.9974	102.6206	98.4856	95.9231
ZEP	zeaxanthin epoxidase	Solyc02g090890	23.6926	86.1239	72.7482	81.4112	70.0442	76.5718	23.4714	21.0918	24.4972	90.8332	94.7348	90.4356	26.1597	25.3354	28.3485	11.0204	7.6388	8.3105
NXS	neoxanthin synthase	Solyc02g063170	27.2759	60.7596	54.7888	1.4943	2.2317	3.2339	3.3672	2.2123	2.9477	55.7376	51.4808	53.1711	1.8173	2.9905	3.8743	2.0791	2.0473	3.2066
CPS	copalyl diphosphate synthase	Solyc08g005710	1.823092	1.805494	1.185444	-0.663346	-0.663346	-0.663346	-0.663346	-0.561322	-0.663346	0.657393	0.608386	1.755819	-0.66335	-0.66335	-0.64085	-0.66335	-0.66335	-0.66335
KO	ent-kaurene oxidase	Solyc04g083160	1.355571	1.473967	1.450557	-0.58048	-0.599328	-0.719868	-0.278732	-0.479586	-0.501759	1.493997	1.796294	0.122837	-0.93383	-0.92624	-0.96394	-0.57089	-0.51978	-0.61878
KAO	ent-kaurenoic acid oxidase	Solyc12g006460	0.814368	0.620487	2.432219	-0.602923	-0.60757	-0.60561	-0.601428	-0.602129	-0.602349	1.757176	1.594109	0.051718	-0.60855	-0.60643	-0.60804	-0.60841	-0.60906	-0.60757
GA20ox	gibberellin 20 oxidase	Solyc01g093980	0.1191	0.3336	0.179	0	0.071	0.0555	0	0	0	0.2304	0.1338	0.4333	0.2023	0.0573	0.0549	0	0	0
GA2ox	gibberellin 2 oxidase	Solyc02g080120	-0.05862	0.160449	2.523225	-0.36607	-0.393941	-0.485325	-0.463387	-0.51507	-0.543489	2.094765	1.716779	-0.33496	-0.52245	-0.56387	-0.52214	-0.5753	-0.5753	-0.5753
GA3ox	gibberellin 3 oxidase	Solyc03g116280	0.441863	0.338151	2.530969	-0.570842	-0.593703	-0.522074	-0.524405	-0.593703	-0.593703	1.844511	1.679536	0.125615	-0.5937	-0.5937	-0.5937	-0.5937	-0.5937	-0.5937
